# Molecular insights into DDX3X–androgen receptor mRNA regulation via non-canonical G-quadruplex in castration-resistant prostate cancer

**DOI:** 10.1038/s41388-026-03777-x

**Published:** 2026-05-04

**Authors:** Han Zhang, Teresa T. Liu, Feixuan Wu, Avan N. Colah, Emily A. Ricke, Lingjun Li, Andrea A. Putnam, William A. Ricke

**Affiliations:** 1 Division of Pharmaceutical Sciences, School of Pharmacy, University of Wisconsin-Madison, Madison, WI USA; 2 Pharmaceutical Sciences, Wegmans School of Pharmacy, St. John Fisher University, Rochester, NY USA; 3 Department of Urology, School of Medicine and Public Health, University of Wisconsin-Madison, Madison, WI USA; 4 George M. O’Brien Urology Research Center of Excellence, School of Medicine and Public Health, University of Wisconsin-Madison, Madison, WI USA; 5 Department of Chemistry, University of Wisconsin-Madison, Madison, WI USA; 6 Lachman Institute for Pharmaceutical Development, School of Pharmacy, University of Wisconsin-Madison, Madison, WI USA; 7 Department of Biomolecular Chemistry, School of Medicine and Public Health, University of Wisconsin-Madison, Madison, WI USA

**Keywords:** Prostate cancer, Proteomics

## Abstract

Prostate cancer (PC) is one of the most common malignancies in men, and the emergence of androgen receptor-low/negative castration-resistant PC (ARL/– CRPC) following androgen receptor signaling inhibitor (ARSI) therapy remains a critical clinical challenge. The RNA-binding protein DEAD-box helicase 3 X-linked (DDX3X) has been implicated in the translational regulation of androgen receptor (AR) mRNA; however, the underlying binding mechanisms are not well defined. Here, we show that DDX3X colocalizes with AR mRNA in ARL/– CRPC cells and selectively recognizes non-canonical RNA G-quadruplex (rG4) motifs within the sequence of AR mRNA. RNA immunoprecipitation sequencing (RIP-seq) revealed enrichment of DDX3X–AR mRNA interactions in ARL/– CRPC cells. Fluorescence imaging confirmed the colocalization of DDX3X and AR mRNA within cytoplasmic granules, and biochemical assays confirmed the ability of selected AR mRNA fragments to form rG4 structures bound by DDX3X. Proteomic profiling of DDX3X-Ras GTPase-activating protein-binding protein 1 (G3BP1) complexes identified several RNA-binding proteins, including IGF2BP1, PUM2, and UBAP2, which may act as candidate cofactors. Together, these findings shed light on the interaction between AR mRNA and DDX3X and identify putative protein partners, offering insights into future therapeutic strategies.

## Introduction

Prostate cancer (PC) is among the most commonly diagnosed malignancies in men worldwide and remains a leading cause of cancer-related mortality [1]. As it is an androgen-driven disease, the androgen deprivation therapy (ADT) targeting prostatic androgen receptor (AR) signaling shows initial repression of tumor growth and extends the survival of patients [2, 3]. However, drug resistance develops quickly due to the selective pressure of treatment and results in the development of castration-resistant prostate cancer (CRPC) [4]. This subtype of PC is refractory to ADT and exhibits rapid disease progression if not effectively managed. Typically, the majority of CRPC retains AR activity through various mechanisms and can be treated with the AR signaling inhibitors (ARSIs), such as enzalutamide and abiraterone [5]. Recent studies have reported the emergence of an ARSI-induced subset of CRPC characterized by low or AR-negative (ARL/-) expression, which currently lacks effective treatment options [6, 7]. The molecular pathways that enable AR-independent survival and proliferation in these contexts are incompletely understood, representing a critical barrier to effective therapeutic intervention.

Previous studies have implicated the RNA helicase, DDX3X (DEAD-box helicase 3 X-linked), in PC progression, and more recently described the expression of AR protein as translationally regulated by DDX3X [8, 9]. As an RNA-binding protein, DDX3X is involved in transcription, RNA splicing, and translation [10]. Characterization of patient samples and in vitro models of CRPC defined a negative regulatory relationship between DDX3X and AR protein expression, particularly ARL /- subtypes [8]. Mechanistically, it is proposed that DDX3X-AR mRNA complexes localize to stress granules (SGs) in ARL /- CRPC in response to hypoxic, metabolic, and therapeutic stress [8, 10, 11]. Consequently, cells are rendered incompetent for AR translation, leading to the ARL /- phenotype. The inhibition of DDX3X resolved SGs and restored AR protein expression [8]. While a relationship between AR and DDX3X has been defined, the detailed recognition and binding mechanisms between DDX3X and AR mRNA remain unclear.

It has been shown that DDX3X can bind RNA G-quadruplex (rG4) in several RNA transcripts, including NRAS and BCL2 [12]. The rG4 is a secondary structure that forms in G-rich RNA sequences, known to regulate mRNA translation, stability, and localization [13]. While the functional relevance of rG4 has been demonstrated in various disease states, its role in ARL/- CRPC remains poorly understood [14–18].

In this study, we profiled the transcriptome of ARL/- CRPC, investigated the DDX3X-AR mRNA interaction, and further explored the SG composition in ARL/- CRPC. Our findings demonstrate that DDX3X can bind to specific non-canonical rG4-forming sequences in AR mRNA and identify putative DDX3X binding cofactors in SGs, including IGF2BP1, PUM2, and UBAP2. These results shed light on the molecular pathology of ARL/- CRPC and provide important information for the development of future therapeutics.

## Materials and methods

### Cell culture

The BPH1-derived Cancer Progression (BCaP) cell lines were developed as previously described [19]. BCaP^MT10^, BCaP^NT1^, and HEK 293T cells were cultured in RPMI 1640 + L-glutamine medium (Corning, 10041CV) supplemented with 5% fetal bovine serum (Gibco, 10437-010), 1% penicillin–streptomycin (Gibco, 15140-122), and 0.2% Normocin (Invivogen, ant-nr), at 37 °C, with 5% CO_2_. All cell lines were tested mycoplasma-negative using the MycoStrip Kit (Invivogen, rep-mys) and authenticated by ATCC or the University of Wisconsin–Madison core services between 6 months and 1 year prior to experimental use.

### RNA immunoprecipitation-sequencing (RIP-seq)

A total of 8 × 10⁶ cells were lysed in 1 mL of non-denaturing buffer (20 mM Tris-HCl, pH 7.5; 150 mM NaCl; 1.5 mM MgCl₂; 0.25% NP-40) supplemented with 1× Halt protease and phosphatase inhibitor cocktail (Thermo Scientific, 87786) and divided for DDX3X or IgG control pulldown. Protein concentrations were normalized, and 60 μL of each lysate was reserved for input RNA. The remaining was incubated with Protein G Dynabeads (Thermo Scientific, 10004D) pre-bound to DDX3X (Bethyl, A300-474A) or normal IgG antibodies for 4 h at 4 °C. RNA from input, IgG, and DDX3X RIP samples was extracted using the RNeasy Mini Kit (Qiagen, 74104), and cDNA was synthesized as described above. Libraries were prepared with the KAPA RNA HyperPrep Kit (Roche, KK8541) and sequenced on an Illumina HiSeq 2500 platform at the University of Massachusetts–Boston CPCT Genomics Core. The average library sizes ranged from 226 to 354 bp, corresponding to cDNA inserts of ~100–200 bp plus adapter sequences.

### Bioinformatics

RIP-seq reads were aligned to the Gencode v47 transcriptome based on the GRCh38 genome using Salmon. [20]. A genome index was first generated with the *index* subcommand and the *--gencode* option, providing a decoys list. FASTQ reads were then trimmed in paired-end mode with Trim Galore! to remove Illumina adapter sequences, followed by alignment with Salmon using the *quant* subcommand with options *-l A* and *--validateMappings*. Consistent with the RIP-seq protocol, the data reflected enrichments of bound RNAs. Abundance estimates generated by Salmon were imported into R using *tximport* and analyzed with DESeq2 [21, 22]. Genes were retained if at least three samples contained ≥10 reads. Differential expression analysis was performed with a design formula incorporating RIP fraction and experimental condition, with contrasts specified for individual comparisons. Fold-change shrinkage was applied using *ashr* via the DESeq2 function *lfcShrink*. Gene set enrichment analysis was performed and visualized on the SRplot platform using default settings [23].

### Immunofluorescence (IF)

BCaP^MT10^ and BCaP^NT1^ cells were fixed in methanol for 10 min at room temperature. After two PBS washes, cells were blocked with 1% normal horse serum in PBS for 1 h at room temperature, followed by incubation with primary antibodies against DDX3X (1:250, Bethyl, A300-474A) and G3BP1 (1:250, Proteintech, 66486-1-Ig) in blocking buffer overnight at 4 °C. After three PBS washes, cells were incubated with Alexa Fluor–conjugated secondary antibodies (1:250) for 1 h at room temperature in the dark, washed three times with PBS, and counterstained with 4′,6-diamidino-2-phenylindole (DAPI, 1 μg/mL) for 10 min. Coverslips were mounted, dried overnight at room temperature, and stored at 4 °C until imaging.

### IF-Single-molecule fluorescence in situ hybridization (IF-smFISH)

A set of 48 androgen receptor (AR) mRNA single-molecule fluorescence in situ hybridization (smFISH) probes was designed using the Stellaris Probe Designer (LGC Biosearch Technologies). BCaP^MT10^ and BCaP^NT1^ cells were fixed in 4% paraformaldehyde (PFA) for 10 min and permeabilized with 0.5% Triton X-100 in PBS for 10 min. After blocking with 1% normal horse serum for 1 h, cells were incubated overnight at 4 °C with anti-DDX3X antibody (1:250, Bethyl, A300-474A), followed by Alexa Fluor–conjugated secondary antibodies (1:250, 1 h, room temperature, dark). Cells were washed and post-fixed with 4% PFA for 10 min, then equilibrated with 2× saline-sodium citrate (SSC) and Stellaris wash buffer (10% formamide, 2× SSC). Hybridization was performed overnight at 37 °C with 125 nM AR smFISH probes in Stellaris Hybe buffer (10% formamide, 2× SSC, 200 μg/mL bovine serum albumin, 2 mM ribonucleoside vanadyl complex, 0.2 mg/mL yeast total RNA, 10% dextran sulfate). After washing with Stellaris wash buffer, 2× SSC, and PBS, nuclei were counterstained with 1 μg/mL DAPI, and coverslips were mounted and stored at 4 °C until imaging.

### Spinning disk confocal (SDC) microscopy

IF and IF–smFISH imaging was performed on a custom-built system (Intelligent Imaging Innovations, 3i) based on an inverted Zeiss Axio Observer equipped with a CSU-W1 SoRa spinning disk scan head (Yokogawa), 1×/2.8×/4× relay lenses (Yokogawa), a fast piezo Z-drive (Applied Scientific Instrumentation), and an ORCA-Fusion BT digital CMOS camera (Hamamatsu). Samples were illuminated using solid-state lasers at 405, 561, and 640 nm (Coherent). Images were acquired with SlideBook software (3i) using a 40×/1.3 NA objective (Zeiss) and a 2.8× relay lens (Yokogawa). All spinning-disk confocal imaging was conducted in a climate-controlled room at 20 °C.

### Plasmid transfection and protein purification

DH5α competent *E. coli* containing the pcDNA3.1 HA-DDX3X plasmid was obtained from Addgene (#44975). Plasmid DNA was purified using the NucleoSpin Plasmid Kit (Macherey-Nagel, 740588.10). Transfection was performed in HEK 293T cells following the manufacturer’s instructions for the Lipofectamine 3000 Transfection Reagent (Invitrogen, L3000001).

### Electrophoretic mobility shift assay (EMSA)

RNA oligonucleotides used for EMSA were as follows: (1) rG4_1: GCGGGAGAGCAGGGAGGCCUCGGGGGCU, (2) rG4_2: GAAGUGCAGUUAGGGCUGGGAAGGG, (3) rG4_3: UAUGGACCGUGUGGUGGUGGUGGGGGUGGUGGCGGCGGCGGCGGCGGCGG, and (4) Non-rG4: AAUUUUUCCUUCGGAAUUUAUCAAUAGUGC. Biotinylated rG4_1, rG4_2, and rG4_3 were synthesized by Integrated DNA Technologies (IDT). The non-rG4 RNA was generated by in vitro transcription of a DNA template (TAATACGACTCACTATAGGGAATTTTTCCTTCGGAATTTATCAATAGTGC; ordered from IDT) using the HiScribe T7 High Yield RNA Synthesis Kit (New England Biolabs, E2040S) and subsequently biotinylated with the Pierce RNA 3’ End Biotinylation Kit (Thermo Scientific, 20160). All oligos were dissolved in nuclease-free water, aliquoted, and stored at –80 °C.

To induce rG4 folding, RNA oligos were diluted to the desired concentration in 1× TE buffer (10 mM Tris-HCl, pH 8.0; 1 mM EDTA) containing 150 mM KCl, heated to 90 °C, and slowly cooled to room temperature as previously described [24]. EMSAs were performed using the LightShift Chemiluminescent RNA EMSA Kit (Thermo Scientific, 20158) with 6% polyacrylamide gels.

### RNA G-quadruplex (rG4) formation prediction

rG4 formation prediction in AR mRNA was performed using the G4Hunter application published previously [25]. The sequence used was full-length AR mRNA (NM_000044.6). The window size of the rG4-forming sequences was set to 25, with the threshold set to 1.2.

### Thioflavin T (ThT) fluorescence assay

RNA samples and ThT were mixed to a final concentration of 2 μM in 20 mM Tris-HCl (pH 7.2) containing 40 mM KCl. rG4 formation was induced by heating the samples to 90 °C, followed by slow cooling to room temperature. After incubation at room temperature for 1 h, samples were transferred to a 96-well plate, and fluorescence was measured using a microplate reader (Thermo Fisher Scientific, Varioskan LUX). Fluorescence emission was recorded at 487 nm with excitation at 440 nm.

### Immunoprecipitation (IP)

BCaP^MT10^ and BCaP^NT1^ cells were lysed in 400 μL of non-denaturing IP buffer with protease/phosphatase inhibitors on ice for 10 min. Lysates were centrifuged (10,000 rpm, 10 min, 4 °C), and supernatants were collected. For each IP, 700 μg protein was incubated overnight at 4 °C with antibodies against DDX3X (Bethyl, A300-474A), G3BP1 (Proteintech, 66486-1-Ig), or control IgG, followed by 4 h incubation with 25 μL protein A/G magnetic beads (Thermo Scientific, 88802). Beads were washed three times with IP wash buffer (20 mM Tris-HCl, pH 7.5; 150 mM NaCl; 1.5 mM MgCl₂) and once with nuclease-free water.

For mass spectrometry, proteins were recovered by on-bead digestion as described previously [26, 27]. Briefly, proteins were solubilized in 8 M urea with 50 mM Tris-HCl, reduced with 5 mM dithiothreitol (DTT) at 37 °C for 1 h, alkylated with 15 mM iodoacetamide (IAA) at room temperature in the dark for 30 min, and quenched with 5 mM DTT for 10 min. The urea concentration was diluted to 1 M with 50 mM Tris-HCl, and proteins were digested with trypsin at an enzyme-to-protein ratio of 1:50 at 37 °C for 18 h. Digestion was terminated with 10% trifluoroacetic acid (TFA) to a final concentration of 0.3%. Peptides were desalted using C18 SepPak cartridges (Waters, 186000383) and dried *in vacuo*.

### Western blotting (WB)

WB was performed as previously described [28, 29]. IP and input samples were collected from BCaP^MT10^ and BCaP^NT1^ cells. Total protein was determined using Bio-Rad stain-free imaging technology and Coomassie blue staining [30]. Primary antibody was applied against DDX3X (1:3000, Bethyl, A300-474A).

### Liquid chromatography-tandem mass spectrometry (LC–MS/MS)

Peptides were reconstituted in 0.1% formic acid (FA) and analyzed using a Dionex UltiMate 3000 UPLC (Thermo Fisher Scientific) coupled to a QE-HF mass spectrometer (Thermo Fisher Scientific). The mobile phase consisted of water with 0.1% FA as phase A and acetonitrile (ACN) with 0.1% FA as phase B. Peptide separation was performed using a 17 cm column packed with Ethylene Bridge Hybrid C18 material (1.7 μm, 130 Å, Waters), with an over 80-min gradient from 0 to 30% mobile phase B. The MS scan range covered m/z 300 to 2000 at a resolution of 60,000, with an automatic gain control (AGC) target of 3E6 and a maximum injection time (IT) of 50 ms. Data-dependent MS/MS was performed in top-20 mode with higher-energy collisional dissociation (HCD) at a normalized collision energy (NCE) of 30. MS/MS scans were acquired at a resolution of 15,000 with an AGC target of 1 × 10⁵ and a maximum injection time of 100 ms. Dynamic exclusion was set to 45 s, and the isolation window was 1.4 m/z.

### MS data analysis

Raw files were processed using the Sequest HT algorithm integrated within Proteome Discoverer 2.5 (Thermo Fisher Scientific). Spectra were searched against the UniProt Homo sapiens reviewed database (January, 2023). Trypsin digestion was allowed with up to two missed cleavages. Precursor mass tolerance was set at 20 ppm and fragment mass tolerance at 0.02 Da. Fixed modifications included carbamidomethylation ( + 57.02146 Da) on C residues, and dynamic modifications included oxidation of M ( + 15.99492 Da). Protein identifications were filtered at a 1% false discovery rate (FDR), and only high-confidence identifications were retained for downstream analysis. Gene ontology annotation and Student’s *t*- test of quantitation results were performed using Perseus [31]. Further data processing was conducted using in-house Python and R scripts [32]. Proteins with a fold change >1.5 or <-1.5 and *P*-value < 0.05 were considered differentially expressed.

### Statistical analysis

All statistics (excluding bioinformatics) were performed with Prism GraphPad software 10.5.0 (GraphPad Software, Boston, MA). Statistical significance for the ThT fluorescence assay (*n* = 3) was calculated using one-way analysis of Variance (ANOVA). Comparisons of SG protein abundance (*n* = 3) were conducted using multiple t-tests. The value of *p* < 0.05 was considered statistically significant.

## Results

### ARL/- CRPC and non-tumorigenic cells exhibit distinct transcriptomes

**Fig. 1:**
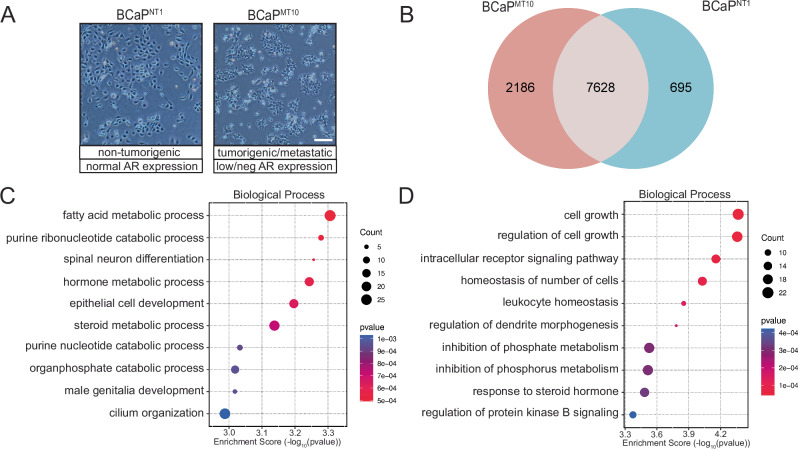
Transcriptome profiling of prostate cancer progression in cell models. **A** Summary of cellular characteristics of BCaP^NT1^ (non-tumorigenic) and BCaP^MT10^ (ARL/– CRPC) cells. AR, androgen receptor. Scale bar: 200 μm. **B** Venn diagram of the number of transcripts identified in BCaP^MT10^ and BCaP^NT1^ cells. **C** Gene ontology (GO) enrichment of transcripts upregulated in BCaP^MT10^ cells, with gene count and p-value indicated. Gene enrichment in biological processes is shown. **D** GO enrichment of transcripts downregulated in BCaP^MT10^ cells, with gene count and p-value indicated. Gene enrichment in biological processes is shown.

To capture transcriptomic alterations accompanying disease progression, we performed RNA sequencing in androgen receptor low/negative castration-resistant prostate cancer (ARL/– CRPC) BCaP^MT10^ and non-tumorigenic BCaP^NT1^ cells, a progression model of prostate cancer (PC) [19] (Fig. 1A). 9814 mRNAs were detected in ARL/– CRPC BCaP^MT10^ cells, whereas 8323 mRNAs were identified in non-tumorigenic BCaP^NT1^ cells (Fig. 1B). Of these, 7628 mRNAs overlapped between the cell lines. Gene Ontology (GO) analysis revealed distinct transcriptomes between non-tumorigenic and ARL/- CRPC cells. Compared to BCaP^NT1^ cells, transcripts upregulated in BCaP^MT10^ cells were significantly enriched in metabolic and developmental processes (Fig. 1C). In contrast, transcripts downregulated in BCaP^MT10^ cells were associated with cell growth regulation and signaling pathways involved in maintaining cellular homeostasis and receptor functions, suggesting a shift from growth (i.e., increase in cell size) to proliferation (i.e., increase in cell number) and the development of AR independence in ARL/– CRPC (Fig. 1D). These transcriptional differences between BCaP^MT10^ and BCaP^NT1^ are consistent with observations from ARL/ − CRPC clinical studies, which describe a reprogrammed transcriptome characterized by enrichment of stress-adaptive and metabolic pathways alongside suppression of androgen-responsive signaling during the transition from AR-high PC [7, 33]. Further analysis using the Kyoto Encyclopedia of Genes and Genomes (KEGG) database identified 39 pathways that were differentially enriched between ARL/- CRPC and non-tumorigenic cells, including the Rap1 signaling, VEGF signaling, Notch signaling, and PI3K-Akt signaling pathways (Supplementary Table 1). Together, these results demonstrate that ARL/– CRPC and non-tumorigenic cells exhibit distinct transcriptome profiles, underscoring their divergent regulatory and adaptive programs.

### AR mRNA is recruited to stress granules in ARL/- CRPC

**Fig. 2:**
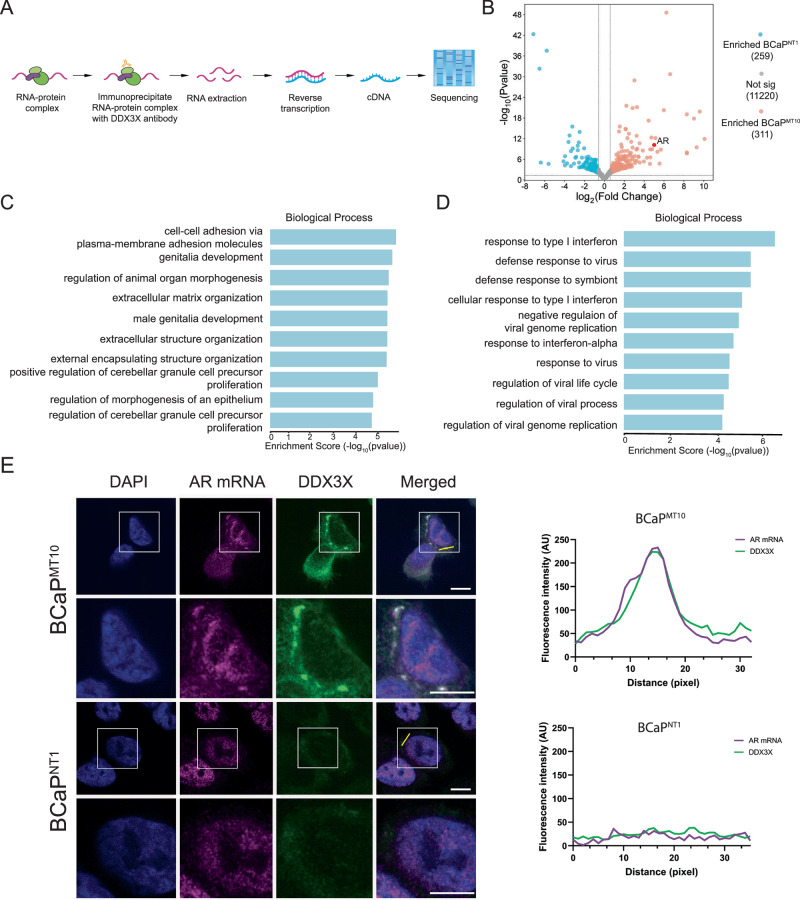
DDX3X–AR mRNA interactions in ARL/– CRPC and non-tumorigenic cells. **A** Schematic of the RIP-seq workflow in BCaP^MT10^ (ARL/– CRPC) and BCaP^NT1^ (non-tumorigenic) cells. **B** Volcano diagram showing differential transcript enrichment between BCaP^MT10^ RIP and BCaP^NT1^ RIP samples. AR mRNA is highlighted in red. **C** GO analysis of genes enriched in BCaP^MT10^ RIP samples, with p-values indicated. Gene enrichment in biological processes is shown. **D** GO analysis of genes enriched in BCaP^NT1^ RIP samples, with p-values indicated. Gene enrichment in biological processes is shown. **E** Left: IF–smFISH imaging showing colocalization of DDX3X (green) with AR mRNA (magenta) in cytoplasmic puncta of BCaP^MT10^ but not BCaP^NT1^ cells. DAPI (blue) marks nuclei. Scale bar: 10 μm. Right: Intensity profiles for AR mRNA and DDX3X channels at representative locations (yellow line) in BCaP^MT10^ and BCaP^NT1^ cells using Fiji software.

Previous clinical studies have indicated that, compared with hormone-naïve and AR-high PC, ARL/- CRPC exhibits increased DEAD-box helicase 3 X-linked (DDX3X) expression [8]. The non-tumorigenic BCaP^NT1^ and ARL/– CRPC BCaP^MT10^ cells recapitulate this characteristic, where decreased amounts of AR protein are coincident with high amounts of DDX3X protein in CRPC. Since DDX3X plays a critical role in translational regulation and has been implicated in cancer drug resistance, a better understanding of DDX3X-mediated mRNA regulation in normal prostate and CRPC would facilitate the development of future therapeutics against CRPC [10, 31, 32]. Yet, the specific mRNAs regulated by DDX3X in ARL/– CRPC that may contribute to PC progression remain unclear. To address this, we profiled DDX3X–mRNA interactions using RNA immunoprecipitation-sequencing (RIP-seq) in ARL/– CRPC BCaP^MT10^ and non-tumorigenic BCaP^NT1^ cells (Fig. 2A). Principal component analysis (PCA) showed clear separation of replicates for the input and RIP of BCaP^MT10^ and BCaP^NT1^ cell models, confirming the robustness of the RIP-seq workflow (Supplementary Fig. 1A). DDX3X-bound transcripts shared between BCaP^MT10^ RIP and BCaP^NT1^ RIP were compared (Fig. 2B and Supplementary Table 2). A total of 311 mRNAs were enriched in BCaP^MT10^ RIP, and 259 mRNAs were enriched in BCaP^NT1^ RIP. GO analysis further indicated enrichment of genes involved in organ development and structural organization in ARL/– CRPC, whereas genes related to immune responses were enriched in non-tumorigenic cells (Fig. 2C, D, and Supplementary Fig. 2).

The current first-line treatment for advanced and metastatic PC targets AR signaling [34]. Absence of prostatic AR protein in CRPC tumor cells contributes to resistance against AR-targeted therapies [4, 35]. Previous work has shown that DDX3X represses AR mRNA translation and implicates the assembly of stress granules (SGs) [8]. However, the mechanisms by which this occurs, whether direct or indirect, are unknown, and evidence of the interaction between DDX3X and AR mRNA is lacking. Here, we found that AR mRNA was significantly enriched in BCaP^MT10^ cells. Moreover, visualization of DDX3X–AR mRNA binding in the Integrative Genomics Viewer (IGV) revealed strong peaks in BCaP^MT10^ RIP samples, confirming enrichment of DDX3X–AR mRNA interactions in ARL/– CRPC and consistent with previous findings [8] (Supplementary Fig. 3A).

To validate the RIP-seq results, we performed immunofluorescence-single molecule fluorescence in situ hybridization (IF-smFISH) to localize DDX3X and AR mRNA. Cytoplasmic puncta, indicative of granule formation, were readily detected in ARL/– CRPC BCaP^MT10^ but not in non-tumorigenic BCaP^NT1^ cells (Fig. 2E and Supplementary Fig. 3B). Intensity profiling confirmed colocalization of DDX3X and AR mRNA within cytoplasmic granules in ARL/– CRPC. Taken together, these results provide evidence that AR mRNA binds to DDX3X and is enriched in SGs in ARL/– CRPC cells.

### DDX3X directly interacts with specific non-canonical rG4s in AR mRNA

**Fig. 3:**
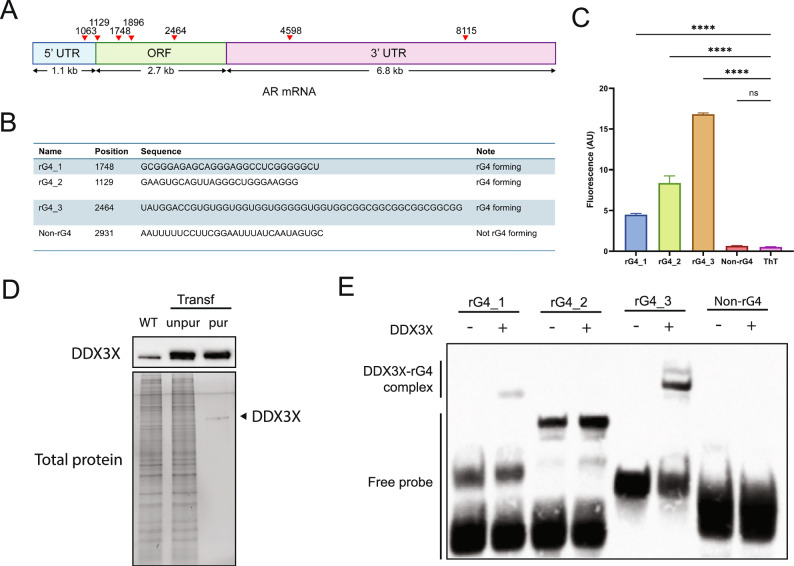
DDX3X directly binds rG4-forming sequences present within AR mRNA. **A** Prediction of rG4-forming sequences in AR mRNA using the G4Hunter web application. Red triangles indicate putative rG4 sites across the transcript. UTR, untranslated region; ORF, open reading frame. **B** Sequences of synthesized RNA oligos, including three predicted rG4 motifs and a non-rG4 control. Their positions within AR mRNA are indicated. **C** ThT fluorescence assay confirms rG4 formation for rG4_1, rG4_2, and rG4_3, but not for the non-rG4 control. Mean ± standard deviation is shown. *N* = 3; ****p < 0.0001; ns, not significant. **D** WB and total protein staining validating purification of HA-tagged DDX3 from transfected HEK 293T cells. WT, wildtype; Transf, transfected; Unpur, unpurified; Pur, purified. **E** EMSA showing direct binding of DDX3X to rG4_1 and rG4_3, but not to rG4_2 or the non-rG4 control.

We next investigated the binding mechanism between DDX3X and AR mRNA. DDX3X has been reported to interact with RNA G-quadruplex (rG4) structures within the 5’ untranslated regions (UTRs) of mRNAs [12, 36]. These rG4 structures are known to promote phase separation and contribute to the formation of SGs, where AR mRNA is enriched with DDX3X [8, 37–39]. Thus, we hypothesized that DDX3X recognizes and binds to rG4 sequences within AR mRNA. To test this, we used the G4Hunter web application to predict rG4-forming sequences and identified seven putative rG4 motifs within AR mRNA [25] (Fig. 3A and Supplementary Fig. 4A). We observed that the sequences are non-canonical rG4 motifs. Compared with the canonical form of rG4 (G_3-5_N_1–7_-G_3-5_N_1–7_-G_3-5_N_1–7_-G_3-5_), which features stacked G-quartets linked by loops and stabilized by monovalent cations like K^+^ and Na^+^, the non-canonical rG4 structure may have incomplete G-tetrads, bulges within G-tracks, varied loop structures, or incorporate non-G nucleotides into the Hoogsteen base pairings [40–43]. Interactions between DDX3X and non-canonical rG4 have been reported in other studies [12, 36].

Next, we assessed whether DDX3X binds to the rG4-forming sequences predicted within the AR mRNA. The top three candidate sequences, selected based on their G4Hunter scores, were synthesized and biotin-labeled [25] (Fig. 3B). Their ability to adopt rG4 structures was confirmed by thioflavin T (ThT) fluorescence assays [44] (Fig. 3C). Additionally, a fragment of AR mRNA (positions 2931–2960 bp) that does not form rG4 served as a negative control. Purified DDX3X protein was obtained using the HA-DDX3X plasmid transfection in HEK 293T cells, followed by HA-tagged protein purification. Western blotting (WB) analysis confirmed DDX3X enrichment in the purified fraction, compared with non-transfected and unpurified lysates (Fig. 3D and Supplementary Fig. 4B). The electrophoretic mobility shift assay (EMSA) showed direct binding of DDX3X to rG4_1 and rG4_3, but not to rG4_2 or the Non-rG4 control, indicating that DDX3X does not bind nonspecifically to all rG4 structures but instead exhibits binding preference (Fig. 3E). To our knowledge, this is the first report demonstrating that regions of AR mRNA can adopt rG4 structures in vitro and that DDX3X directly interacts with specific non-canonical rG4 motifs.

### Putative DDX3X-binding cofactors are identified in ARL/– CRPC

**Fig. 4:**
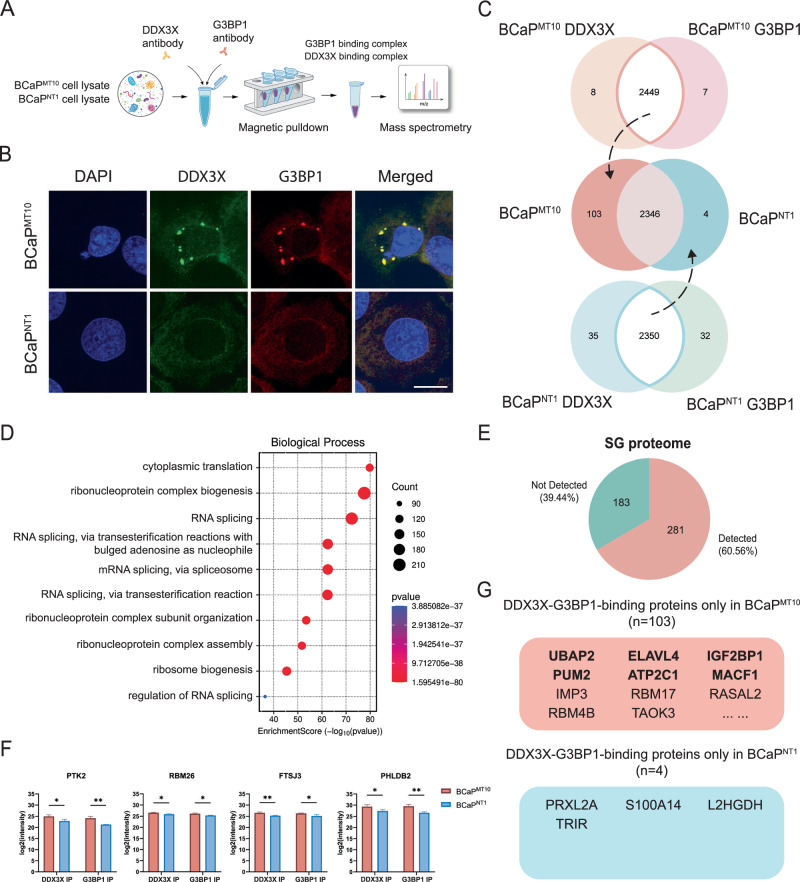
Identification of putative DDX3X-binding cofactors in SGs of ARL/– CRPC. **A** Workflow schematic of IP–MS analysis of DDX3X and G3BP1 complexes. **B** IF showing DDX3X (green) and SG marker G3BP1 (red) colocalization in ARL/– CRPC BCaP^MT10^ but not in non-tumorigenic BCaP^NT1^ cells. DAPI (blue) marks nuclei. Scale bar, 20 μm. **C** Venn diagram of proteins identified in DDX3X- and G3BP1-IP from BCaP^MT10^ and BCaP^NT1^ cells. **D** GO analysis of proteins shared in DDX3X- and G3BP1-IP in BCaP^MT10^, with gene count and p-value indicated. Gene enrichment in biological processes is shown. **E** Numbers and percentages of MSGP proteins identified in DDX3X-G3BP1-IP from BCaP^MT10^. ~60% of known SG proteins in the MSGP database were detected in the DDX3X- and G3BP1-IP overlap. **F** Abundance of selected SG proteins identified in DDX3X- and G3BP1-IP from BCaP^MT10^ and BCaP^NT1^ cells. Mean ± standard deviation is shown. *N* = 3; *, *p* < 0.05; **, *p* < 0.01. **G** Representative lists of BCaP^MT10^- and BCaP^NT1^-specific DDX3X–G3BP1-binding proteins. Known SG proteins are highlighted in bold.

In addition to the direct binding of DDX3X to AR mRNA, DDX3X may interact with transcripts through cofactors [45, 46]. In particular, several SG proteins have been identified as DDX3X-binding partners, including poly(A)-binding protein 1 (PABP1), Ras GTPase-activating protein-binding protein 1 (G3BP1), and eukaryotic translation initiation factor 4 gamma (eIF4G) [11, 47, 48]. Genetic and pharmacological inhibition of DDX3X has been shown to disrupt SG formation and rescue AR mRNA translation in ARL/- CRPC, resulting in increased AR protein expression and enhanced cellular sensitivity to AR signaling inhibitors (ARSIs) [8]. To better understand DDX3X-mediated binding mechanisms in SGs, we performed immunoprecipitation-mass spectrometry (IP-MS) to identify potential DDX3X cofactors in ARL/- CRPC BCaP^MT10^ and non-tumorigenic BCaP^NT1^ cells (Fig. 4A).

Co-staining of DDX3X with the SG markers G3BP1 and PABP1 confirmed SG formation in ARL/– CRPC cells but not in non-tumorigenic cells (Fig. 4B and Supplementary Fig. 5A, B). MS analysis of DDX3X- and G3BP1-IP samples revealed ~99% overlap between DDX3X-binding and G3BP1-binding proteins in both cell lines, further supporting the colocalization of DDX3X and G3BP1 (Fig. 4C). Clustering analysis also suggested overlap among the IP samples (Supplementary Fig. 5C). Moreover, GO analysis indicated that these shared proteins between DDX3X- and G3BP1-IP were primarily involved in RNA processing and protein synthesis in both cell lines, aligning with the established roles of DDX3X and G3BP1 in RNA metabolism and translational control [48] (Fig. 4D). Additionally, ~60% of known SG proteins in the Mammalian Stress Granules Proteome (MSGP) database were detected in the DDX3X- and G3BP1-IP overlap [49] (Fig. 4E and Supplementary 5D). Among those identified components, several SG proteins, including PTK2, RBM26, FTSJ3, and PHLDB2, were significantly enriched in BCaP^MT10^ cells (Fig. 4F).

To identify putative cofactors involved in the DDX3X and AR mRNA interaction, given that AR mRNA is localized to SGs in ARL/- CRPC but not in non-tumorigenic cells, we compared the DDX3X-G3BP1-binding proteins in both cell lines and focused on those identified only in the BCaP^MT10^ (Fig. 4G). Enrichment analysis of BCaP^MT10^-specific proteins showed predominant involvement in xenobiotic and small molecule metabolism (Supplementary Fig. 5E). Notably, several candidates (e.g., IGF2BP1, PUM2, and UBAP2) have been reported to be cytoplasmic and participate in stress response, including translational regulation and SG assembly [50–52]. Co-staining of DDX3X, IGF2BP1, and UBAP2 confirmed their colocalization to SGs in ARL/– CRPC cells but not in non-tumorigenic cells (Supplementary Fig. 6). Together, these results define the DDX3X- and G3BP1-associated protein networks in ARL/– CRPC and non-tumorigenic cells and highlight potential cofactors that may mediate DDX3X–mRNA interactions within SGs.

## Discussion

RNA-protein interactions are important in maintaining cellular homeostasis and function. Elucidating the regulatory mechanisms underlying these interactions is critical for understanding pathogenesis and developing therapeutic targets [53]. In ARL/- CRPC, the absence of AR protein results in resistance to foundational anti-androgen therapies [54]. Previous studies have shown that the RNA-binding protein, DDX3X, regulates AR mRNA translation in ARL/- CRPC, while the mechanisms by which DDX3X recognizes and binds AR mRNA remain unexplored [8]. In this study, we demonstrated a mechanism in which DDX3X directly interacts with non-canonical rG4-forming sequences identified within the AR mRNA in vitro. We further profiled DDX3X-binding partners in ARL/– CRPC cell models and identified putative cofactors that may mediate DDX3X–mRNA interactions in SGs.

The incidence of ARL/- CRPC has increased in recent years, in part due to the widespread use of potent second-generation ARSIs [6, 54]. Existing CRPC models often fail to fully recapitulate PC progression [55]. Our study, by employing the BCaP progression model, profiled the transcriptomes from the non-tumorigenic to the aggressive metastatic ARL/- CRPC stages in a unique prospective [19]. Our data showed that ARL/- CRPC cells exhibit an increased expression of mRNAs linked to fatty acid metabolism and nucleotide catabolism, concomitant with reduced expression of mRNAs involved in cell growth and steroid hormone response. These changes suggest carcinogenesis-associated energy supply reprogramming, a growth-to-proliferation switch, and the establishment of androgen independence [56].

Although DDX3X is essential to normal cellular function and its dysregulation is implicated in various diseases, the set of mRNAs whose translation depends on DDX3X remains incompletely characterized in PC. Our RIP-seq experiments indicate that ARL/– CRPC cells show an increased DDX3X binding to mRNAs involved in tissue development and structural organization. These biological processes are often connected with lineage plasticity and extracellular matrix remodeling, suggesting the key function of DDX3 in tumor survival and metastasis [57, 58]. However, as DDX3X can either facilitate or repress translation, further studies are needed to fully understand the role of DDX3X in cancer progression.

A central finding of this work is the direct association of AR mRNA with DDX3X in SGs of CRPC cells. The rG4 motifs are increasingly appreciated as regulatory elements that control transcript stability and translation, and our data confirms that AR mRNA harbors multiple rG4-forming sequences capable of binding DDX3X [59, 60]. Notably, we noticed that DDX3X engages certain non-canonical rG4s but not others, suggesting a layer of specificity in DDX3X-rG4 recognition. However, RIP-seq does not provide nucleotide-resolution mapping of DDX3X-AR mRNA binding. Future experiments employing advanced techniques, such as crosslinking and immunoprecipitation (CLIP), may improve mapping resolution and identify additional DDX3X-binding sites on transcripts. Another limitation of this study is that we were unable to determine whether the identified DDX3X-binding AR rG4s are required for AR mRNA sequestration to SGs, or whether mutation of these rG4s restores AR protein expression, owing to technical limitations.

At the same time, our model does not rule out the possibility of indirect interactions. In fact, others have reported that DDX3X binds to mRNAs through translation initiation complexes [61, 62]. Our proteomic profiling of DDX3X complexes revealed a network of cofactors that overlaps with core SG proteins, such as G3BP1, PABP1, and eIF4G, as well as additional stress-responsive RNA-binding proteins, including IGF2BP1, PUM2, and UBAP2. Interestingly, over 90% of SG-associated proteins identified in ARL/– CRPC cells were also present in non-tumorigenic cells. A pre-existing SG protein network in unstressed cells likely facilitates rapid SG assembly under stress conditions [63–65]. Such RNA–protein assemblies may provide a mechanism by which tumor cells adapt translational control to evade therapeutic pressure.

Targeting SG-associated RNA translation has emerged as a promising therapeutic strategy. rG4-targeting ligands exploit the enrichment of rG4 structures in oncogenic mRNAs to selectively modulate their translation in cancer cells [66]. In parallel, DDX3X inhibitors such as RK-33 have been shown to sensitize CRPC cells to radiotherapy and ARSIs [8, 9]. Future preclinical and clinical studies of these agents may open novel avenues for PC treatment.

In summary, our study reveals a potential mechanism of DDX3X-AR mRNA recognition and depicts a general picture of DDX3X-mediated SG formation in CRPC. These insights pave the way for strategies to interrupt DDX3X-rG4 in ARL/- CRPC treatment, while further validation and follow-up studies are needed to fully elucidate how DDX3X fine-tunes mRNA fate in PC.

## Supplementary information

Supplementary information

Supplementary Table 1

Supplementary Table 2

## Data Availability

Further information and requests for resources/reagents should be directed to the corresponding author. Plasmid is available via addgene.org. The RNA-seq libraries generated for this paper are deposited at ArrayExpress under accession number E-MTAB-15800 via https://www.ebi.ac.uk/biostudies/ArrayExpress/studies/E-MTAB-15800?key=d20492f5-0e48-4fe2-8574-b729790921c9. The LC-MS/MS raw data have been deposited to the ProteomeXchange Consortium via the MassIVE partner repository with the accession number MSV000099475; the reviewer access password is ‘RickeLab’.
