# Supplementary material for: Molecular insights into DDX3X–androgen receptor mRNA regulation via non-canonical G-quadruplex in castration-resistant prostate cancer
Source: Oncogene. 2026 May 4;45(24):2375–84. doi: 10.1038/s41388-026-03777-x (PMC13249569; doi:10.1038/s41388-026-03777-x)
Supplement: Supplementary file 1 — Supplementary information [file 41388_2026_3777_MOESM1_ESM.pdf]

Supplementary Data

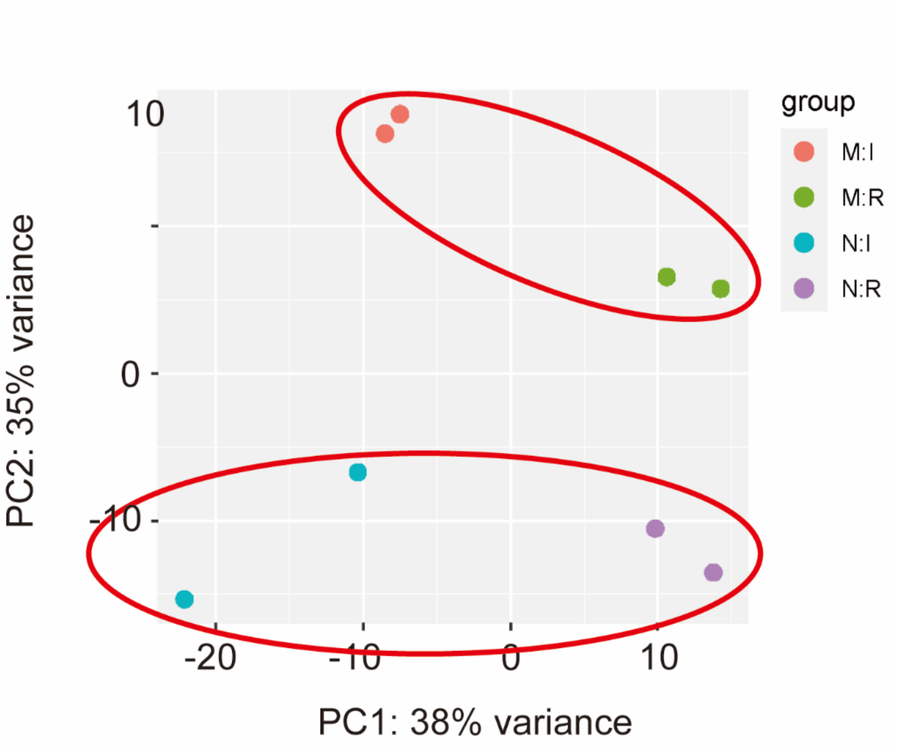

Supplementary Figure 1. Principal component analysis (PCA) shows the separation of samples. M, BCaP^{MT10}; N, BCaP^{NT1}; I, input; R, RNA immunoprecipitation-sequencing.

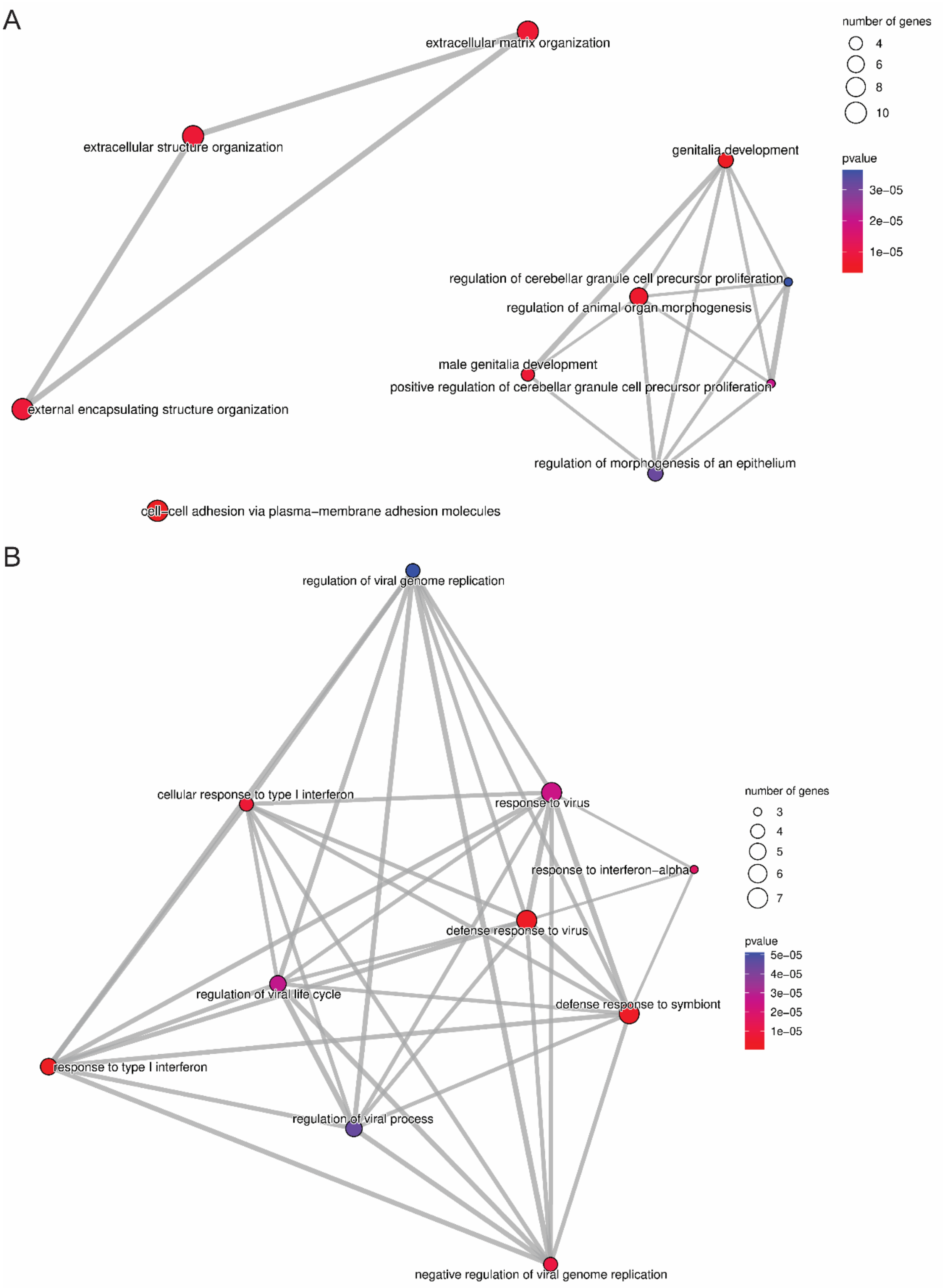

Supplementary Figure 2. (A) Enrichment map of the biological processes enriched in the RNA-immunoprecipitation (RIP) of

BCaP^{MT10} cell model. (B) Enrichment map of the biological processes enriched in the RIP of BCaP^{NT1} cell model.

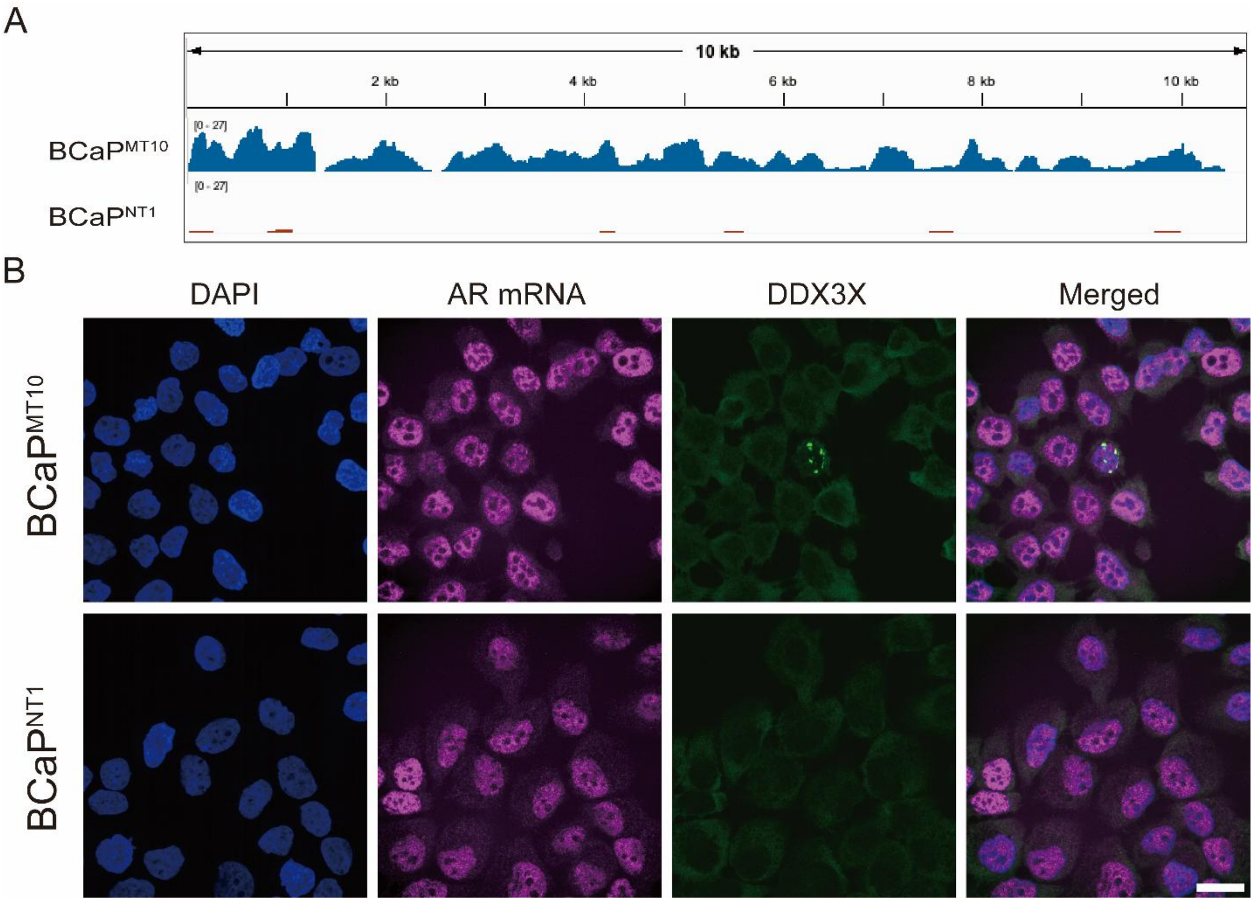

Supplementary Figure 3. (A) Integrative Genomics Viewer (IGV) tracks showing AR mRNA peaks enriched in BCaP^{MT10} compared with BCaP^{NT1}. (B) Immunofluorescence-single-molecule fluorescence *in situ* hybridization (IF–smFISH) imaging showing colocalization of DDX3X (green) with AR mRNA (magenta) in cytoplasmic puncta of BCaP^{MT10} but not BCaP^{NT1} cells. DAPI (blue) marks nuclei. Scale bar: 20 µm.

A

Position	Sequence
1063	UCUGAGCAAGAGAAGGGGAGGCGGGGUAAGGGAAGUAGGUGGAAGAUU
1129	GAAGUGCAGUUAGGGCUGGGAAGGG
1748	GCGGGAGAGCAGGGAGGCCUCGGGGGCU
1896	GAGUCCAGGGGAACAGCUUCGGGGGGAUUGCAUG
2464	UAUGGACCGUGUGGUGGUGGUGGGGGUGGUGGCGGCGGCGGCGGCGGCGG
4589	CAGAGCUGAGUUGGGCAGGGGGGUGGACAGAGAGGAGAGGACA
8115	CCAAAAUUCAUAAGGGCAGGGGGGGAGCAAGCAUUAGUGC

B

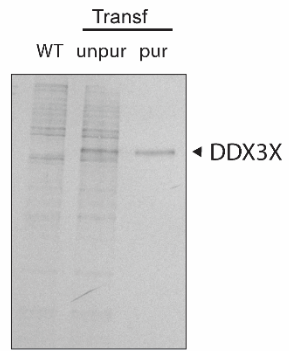

Supplementary Figure 4. (A) Putative RNA G-quadruplex (rG4) sequences predicted within AR mRNA using the G4Hunter web application, with their positions in AR mRNA indicated. (B) Coomassie staining validating purification of HA-tagged DDX3X from transfected HEK 293T cells. WT, wildtype; Transf, transfected; Unpur, unpurified; Pur, purified.

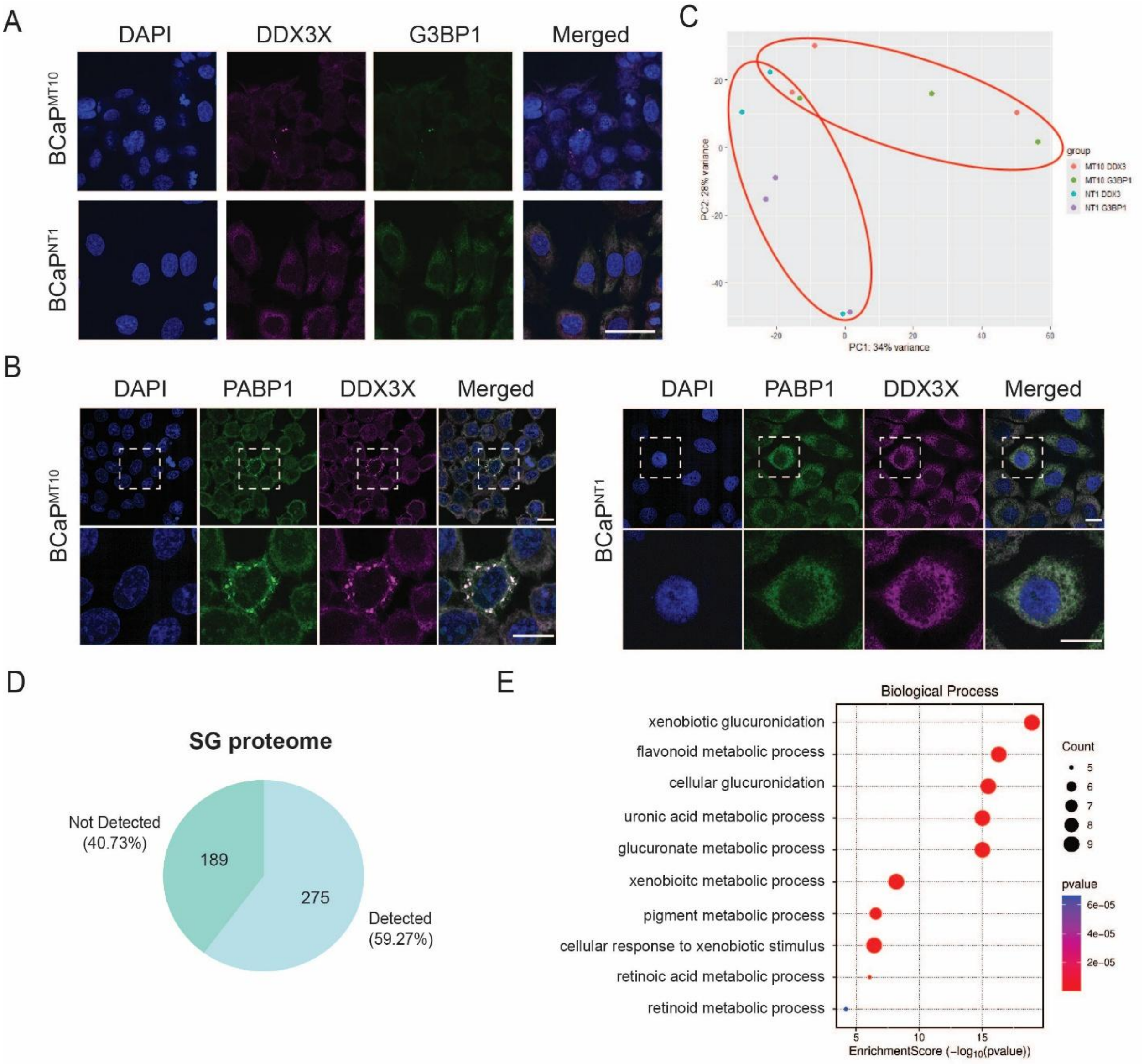

Supplementary Figure 5. (A) Immunofluorescence (IF) showing DDX3X (green) and stress granule (SG) marker G3BP1 (red) colocalization in ARL/– CRPC BCaP^{MT10} but not in non-tumorigenic BCaP^{NT1} cells. DAPI (blue) marks nuclei. Scale bar, 20 µm. (B) IF showing DDX3X (magenta) and SG marker PABP1 (green) colocalization in ARL/– CRPC BCaP^{MT10} (left panel) but not in non-tumorigenic BCaP^{NT1} (right panel) cells. DAPI (blue) marks nuclei. Dashed boxes indicate regions that are shown at higher magnification in the lower panels. Scale bar, 10 µm. (C) PCA shows the clustering of IP samples. MT10, BCaP^{MT10}; NT1, BCaP^{NT1}. (D) Numbers and percentages of Mammalian Stress Granules Proteome (MSGP) proteins identified in DDX3X-G3BP1-IP from

BCaP^{NT1}. SG, stress granule. (E) Gene ontology (GO) analysis of DDX3X-G3BP1-binding proteins specific to BCaP^{MT10}, with gene count and p-value indicated. Gene enrichment in biological processes is shown.

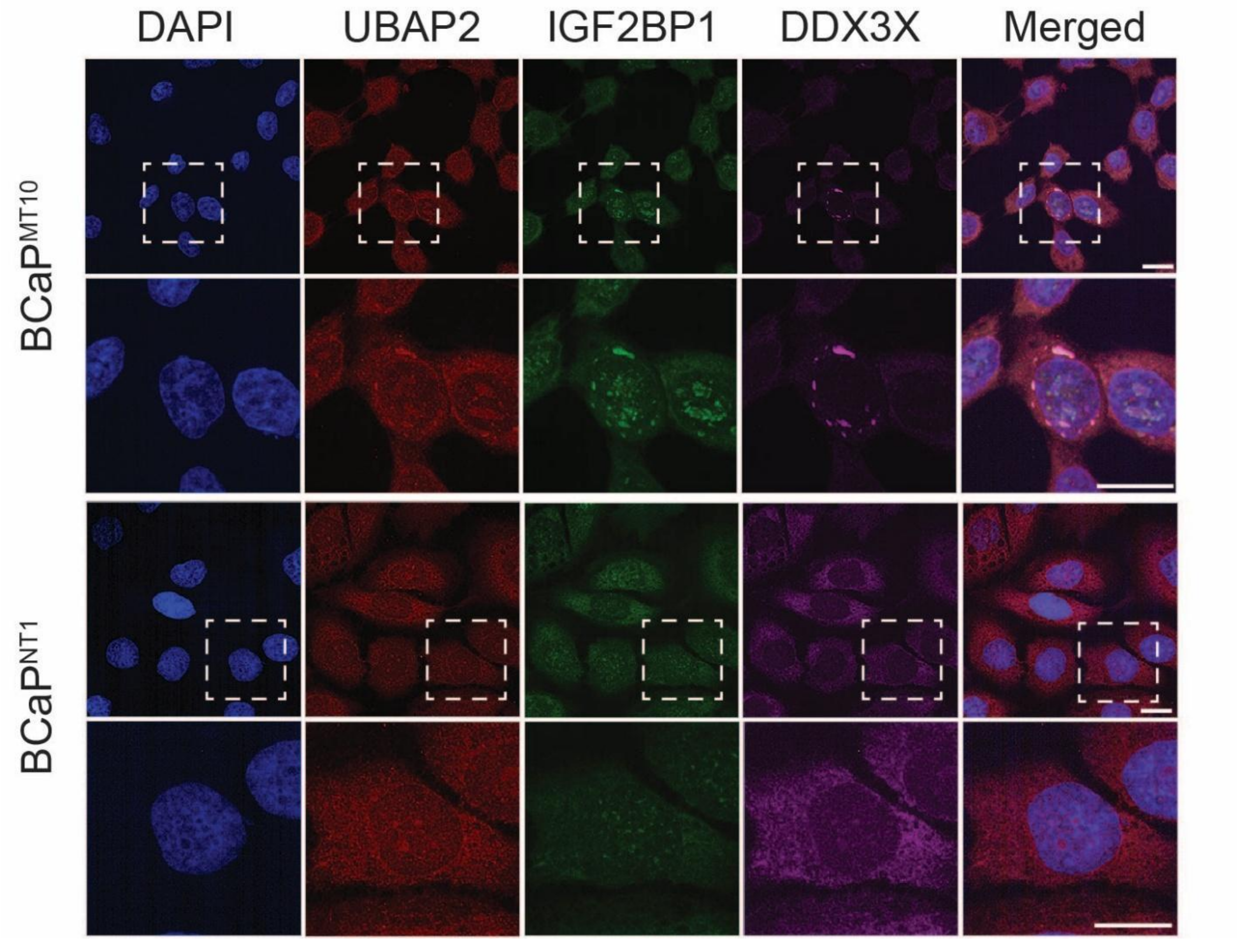

Supplementary Figure 6. IF showing DDX3X (magenta), UBAP2 (red), and IGF2BP1 (green) colocalization in ARL/– CRPC BCaP^{MT10} but not in non-tumorigenic BCaP^{NT1} cells. DAPI (blue) marks nuclei. Dashed boxes indicate regions that are shown at higher magnification in the lower panels. Scale bar, 10 µm.
