# Supplementary material for: Molecular insights into DDX3X–androgen receptor mRNA regulation via non-canonical G-quadruplex in castration-resistant prostate cancer
Source: Oncogene. 2026 May 4;45(24):2375–84. doi: 10.1038/s41388-026-03777-x (PMC13249569; doi:10.1038/s41388-026-03777-x)
Supplement: Supplementary file 2 — Supplementary Table 1 [file 41388_2026_3777_MOESM2_ESM.pdf]

Supplementary Table 1: Differentially enriched pathways between ARL/- CRPC (BCaP^{MT10}) and non-tumorigenic cells (BCaP^{NT1}) identified by the KEGG database.

Column A: Term ID.
Column B: Description, the description of the term.
Column C: GeneRatio, the number of genes enriched in certain term / the number of input genes.
Column D: BgRatio, the number of genes within certain term / the number of background genes.
Column E: pvalue, the pvalue of certain enriched term.
Column F: p.adjust, the adjusted pvalue.
Column G: qvalue, the qvalue of certain enriched term.
Column H: geneID, the gene symbols of certain enriched term overlapped with the input genes
Column I: Count, the gene counts of certain enriched term overlapped with the input genes.
Column J: Enrichment Score, A statistical measure (-log10(p-value)) that quantifies the significance of gene set enrichment in the term.
Column K: Fold Enrichment, The ratio of the proportion of genes in a GO term within a gene set to their proportion in the entire genome.

ID	Description	GeneRatio	BgRatio	pvalue	p.adjust	qvalue	geneID	Count	Enrichment.Score	Fold.Enrichment
hsa05171	Coronavirus disease	50/604	238/9396	3.69618E-14	1.21974E-11	1.1322E-11	MAPK11/MMP1/T	50	13.43224678	3.268128443
hsa04015	Rap1 signaling path	25/604	212/9396	0.002320564	0.200927327	0.186506706	FGF5/ITGB2/MAP	25	2.634406422	1.834468324
hsa04520	Adherens junction	14/604	93/9396	0.00233174	0.200927327	0.186506706	NECTIN3/SORBS	14	2.63231989	2.341807306
hsa00520	Amino sugar and n	8/604	38/9396	0.002435483	0.200927327	0.186506706	PMM1/AMDHD2/	8	2.613414942	3.275008714
hsa05231	Choline metabolism	14/604	99/9396	0.004192035	0.234135806	0.217331801	SLC22A3/MAP2K	14	2.377575059	2.199879591
hsa04120	Ubiquitin mediated	18/604	142/9396	0.004257015	0.234135806	0.217331801	CBL/UBE2B/ANA	18	2.370894854	1.971924261
hsa00100	Steroid biosynthesis	5/604	20/9396	0.007454929	0.266670785	0.247531733	HSD17B7/CYP2R	5	2.127556479	3.889072848
hsa01250	Biosynthesis of nuc	7/604	37/9396	0.008312748	0.266670785	0.247531733	PMM1/UGDH/GA	7	2.080255374	2.943082155
hsa01240	Biosynthesis of cof	18/604	154/9396	0.009824469	0.266670785	0.247531733	DHRS3/ALDH2/N	18	2.007690892	1.818267825
hsa01212	Fatty acid metabolis	9/604	57/9396	0.009915107	0.266670785	0.247531733	ACAA2/HSD17B8	9	2.003702597	2.456256535
hsa04144	Endocytosis	26/604	252/9396	0.011193603	0.266670785	0.247531733	CBL/AP2S1/GRK3	26	1.951030114	1.605014191
hsa01521	EGFR tyrosine kina	11/604	80/9396	0.01291052	0.266670785	0.247531733	MAP2K2/PDGFB/	11	1.88905625	2.138990066
hsa00565	Ether lipid metabol	8/604	50/9396	0.013590481	0.266670785	0.247531733	ENPP6/PLA2G4A/	8	1.86676518	2.489006623
hsa04370	VEGF signaling pat	9/604	60/9396	0.013742954	0.266670785	0.247531733	MAP2K2/MAPK1	9	1.861919914	2.333443709
hsa04514	Cell adhesion mole	18/604	160/9396	0.014248782	0.266670785	0.247531733	CNTN1/CNTNAP2	18	1.846222269	1.750082781
hsa00564	Glycerophospholipi	13/604	103/9396	0.014440315	0.266670785	0.247531733	AGPAT2/PLA2G4	13	1.840423348	1.963415418
hsa04360	Axon guidance	20/604	184/9396	0.014453118	0.266670785	0.247531733	SHH/NTN1/NTNG	20	1.840038462	1.690901238
hsa05219	Bladder cancer	7/604	41/9396	0.014545679	0.266670785	0.247531733	MAP2K2/MMP1/C	7	1.837265996	2.655952189
hsa04216	Ferroptosis	7/604	42/9396	0.01651745	0.269133526	0.249817723	SLC7A11/HMOX1	7	1.782057003	2.592715232
hsa04330	Notch signaling pat	9/604	62/9396	0.016840831	0.269133526	0.249817723	MAML3/ITCH/AD	9	1.773636475	2.258171331
hsa04014	Ras signaling pathw	24/604	238/9396	0.018626594	0.269133526	0.249817723	FGF5/MAP2K2/PD	24	1.729866563	1.568701653
hsa00513	Various types of N-	7/604	43/9396	0.018671031	0.269133526	0.249817723	MGAT1/MAN1B1	7	1.728831707	2.532419529
hsa00051	Fructose and mann	6/604	34/9396	0.019779594	0.269133526	0.249817723	PMM1/AKR1B1/T	6	1.703782626	2.745227892
hsa04510	Focal adhesion	21/604	203/9396	0.020657012	0.269133526	0.249817723	PDGFB/VCL/PDG	21	1.684932505	1.609271523
hsa03410	Base excision repai	7/604	44/9396	0.021014087	0.269133526	0.249817723	POLD2/SMUG1/P	7	1.677489474	2.474864539
hsa04931	Insulin resistance	13/604	109/9396	0.02225415	0.269133526	0.249817723	CREB3L2/SLC27A	13	1.652588982	1.855337505
hsa00561	Glycerolipid metab	9/604	65/9396	0.022402349	0.269133526	0.249817723	ALDH2/DGAT2/A	9	1.649706436	2.153948039
hsa00510	N-Glycan biosynthe	8/604	55/9396	0.023307996	0.269133526	0.249817723	MGAT1/MAN1B1	8	1.632495064	2.262733293
hsa04140	Autophagy - anima	18/604	169/9396	0.023651128	0.269133526	0.249817723	WIPI1/MAP2K2/C	18	1.626148141	1.656883107
hsa04810	Regulation of actin	23/604	232/9396	0.025022319	0.275245509	0.255491047	FGF5/ITGB2/MAP	23	1.601672443	1.542218543

hsa04151	PI3K-Akt signaling	33/604	362/9396	0.026067713	0.277495007	0.257579097	CREB3L2/FGF5/M	33	1.583897074	1.418114961
hsa03015	mRNA surveillance	12/604	103/9396	0.032169618	0.331749183	0.307939433	UPF3B/MSI1/PPP2	12	1.4925541	1.812383463
hsa00480	Glutathione metabo	8/604	59/9396	0.033985836	0.339858364	0.315466616	GSTM3/GPX2/OPI	8	1.468702037	2.109327646
hsa01200	Carbon metabolism	13/604	117/9396	0.037080921	0.357250965	0.331610943	PCCA/SUCLG1/SI	13	1.430849492	1.728476821
hsa05205	Proteoglycans in ca	20/604	204/9396	0.038653211	0.357250965	0.331610943	CBL/MAP2K2/MA	20	1.412814423	1.525126607
hsa05215	Prostate cancer	12/604	106/9396	0.038972833	0.357250965	0.331610943	CREB3L2/MAP2K	12	1.409238029	1.761089591
hsa05218	Melanoma	9/604	73/9396	0.043418425	0.387245411	0.359452678	FGF5/MAP2K2/PD	9	1.362325936	1.917898939
hsa04070	Phosphatidylinosito	11/604	98/9396	0.049294533	0.404457547	0.375429493	CDS1/IMPA1/MTI	11	1.30720124	1.74611434
hsa04350	TGF-beta signaling	12/604	110/9396	0.049534922	0.404457547	0.375429493	RGMB/ACVR1/BI	12	1.305088517	1.69704997
