# Supplementary material for: Molecular insights into DDX3X–androgen receptor mRNA regulation via non-canonical G-quadruplex in castration-resistant prostate cancer
Source: Oncogene. 2026 May 4;45(24):2375–84. doi: 10.1038/s41388-026-03777-x (PMC13249569; doi:10.1038/s41388-026-03777-x)
Supplement: Supplementary file 3 — Supplementary Table 2 [file 41388_2026_3777_MOESM3_ESM.pdf]

Supplementary Table 2: TPM abundance of transcripts in RNA-immunoprecipitation sequencing (RIP-seq). M: BCaP^{MT10}, N: BCaP^{NT1}, I: input, R: RIP.

Gene	M01I	M01R	M02I	M02R	N01I	N01R	N02I	N02R
A1BG	0	9.398729	0	4.633867	0	2.429591	0	3.182203
A1CF	0	0	0	0	0	0	0	0
A2M	0	0	0	0	0	0	0	0
A2ML1	0	0	0	0	4.700588	0.223525	0	0.388943
A2MP1	0	0	0	0	0	0	0	0
A3GALT2	0	0	0	0	0	0	0	0
A4GALT	10.670057	34.493293	24.922808	49.599767	2.34398	65.044358	0	40.997412
AAAS	26.967973	32.788184	29.292613	45.67053	20.366745	28.973764	27.570693	22.752604
AACS	19.491869	19.360723	18.392938	15.814997	27.352312	25.324504	8.229083	19.105502
AACSP1	0	0.152752	0	0.058206	0	0	0	0.281577
AADAC	4.570006	2.592451	20.53711	2.505549	0	0	0	1.213249
AADACL2	0	0	0	0	0	0	0	0
AADACL3	0	0	0	0	0	0	0	0
AADACP1	0	3.054519	0	1.203247	0	0	0	0
AADAT	8.219295	11.245287	5.453991	7.543686	0.866904	3.252508	0	15.179227
AAGAB	81.046472	172.209102	103.382834	131.775331	47.189329	80.063518	94.871216	109.252079
AAK1	43.050037	54.194021	39.208986	63.006705	36.719106	64.335162	109.323205	68.008225
AAMDC	26.200634	24.399831	14.659917	22.560478	0	13.061617	0	18.762519
AAMP	27.692147	50.836565	118.319826	101.420556	25.760155	140.545192	44.239062	82.139114
AANAT	0	0	0	0	0	0	0	0
AAR2	36.693089	28.021766	30.296616	32.03123	20.475603	25.226038	42.787489	27.159707
AARD	0	0	0	0	0	0	0	0
AARS1	0	0	0	0	0	0	0	0
AARS1P1	0	0	0	0	0	0	0	0
AARS2	2.049198	3.136159	6.135218	3.238564	7.5781	4.718423	0	3.618316
AARSD1	33.448824	40.89056	39.391116	38.68178	40.229323	42.50755	0	42.290355
AARSD1P1	0	1.049426	0	1.520911	0	0.461324	0	1.539826
AASDH	15.921714	16.672214	7.537665	17.74558	9.632414	3.947962	4.706821	13.556676
AASDHPPT	157.857233	149.121415	76.406007	57.18068	44.493535	24.921298	94.66649	93.989826

AASS	5.029538	2.924693	2.510937	2.550415	14.909424	2.686183	3.676518	4.307696
AATF	70.475554	143.054258	73.434728	160.536534	78.751366	151.038321	97.938073	118.153035
AATK	0	0.184339	0	1.165149	0	0.380002	0	0.359358
ABAT	6.17301	6.149303	5.136139	7.178006	4.167048	10.156046	0	9.246089
ABCA1	0.924829	1.741478	8.825578	3.313087	8.556173	9.130122	5.492369	6.272469
ABCA10	4.729322	8.985595	15.844266	16.077161	28.169963	4.140901	0	5.737417
ABCA11P	0	0.693584	0	0.141724	0	0	0	0.286442
ABCA12	3.234465	4.713783	4.04571	6.936123	0.122104	0.33014	0	2.00401
ABCA13	3.707206	4.940368	12.367034	4.827077	1.374037	1.43676	4.36981	1.826152
ABCA15P	0	0	0	0	0	0	0	0
ABCA17P	0	0	0	0	0	0	0	0
ABCA2	5.79166	4.690973	15.501727	8.4173	25.036438	7.276436	4.330757	6.38376
ABCA3	14.461777	12.364364	6.623452	16.595217	1.196941	2.145066	0	1.440307
ABCA3P1	0	0	0	0	0	0	0	0
ABCA4	20.546382	6.602743	0	8.068268	0	0.541041	0	0.770928
ABCA5	1.786406	2.171753	9.223584	2.11411	7.502309	1.549892	8.378328	1.947639
ABCA6	0	0	0	0	0	0	0	0
ABCA7	0	10.679149	3.635389	10.461797	0	6.373702	0	3.434106
ABCA8	0	0	0	0	0	0	0	0
ABCA9	0	0	0	0	0	0	0	0
ABCB1	0.726186	0.249139	0	0.017739	17.042586	0.229327	0	0
ABCB10	15.394501	7.394531	7.674239	9.429482	17.618432	7.933693	2.705152	8.210977
ABCB10P1	0	0	0	0	0	0	0	0
ABCB10P3	0	0	0	0	0	0	0	0
ABCB10P4	0	0	0	0	0	0	0	0
ABCB11	7.364268	2.470303	1.516258	2.079056	0	0.244756	0	0.018717
ABCB4	0	0	0	0	0	0	0	0
ABCB5	0	0	0	0	0	0	0	0
ABCB6	0	4.321715	15.425389	4.895645	26.08866	7.263934	32.651575	5.961402
ABCB7	12.146476	24.391716	48.19616	15.314269	55.768946	15.166361	10.572551	9.116072
ABCB8	5.819675	2.876293	8.041379	4.579399	2.294938	4.921422	0	4.69611
ABCB9	3.578181	7.699836	15.668832	8.968991	12.377522	6.484397	0	6.354767
ABCC1	41.826202	33.61861	54.162369	36.751172	60.736499	35.357438	34.69528	28.355676

ABCC10	21.400468	7.927652	2.913518	3.181762	1.209868	4.034571	43.119383	6.141939
ABCC11	1.934414	0.15791	0	0.255688	0	0.279672	0	0.100705
ABCC12	0	0	0	0	0	0	0	0
ABCC13	0	0	0	0	0	0	0	0
ABCC2	0	0.674635	7.894801	1.411374	0.444349	1.13124	0	0.38221
ABCC3	14.969071	2.610085	2.226724	4.059365	0	2.654546	0	3.00267
ABCC4	8.892167	6.822401	8.492168	9.13356	8.096369	5.087706	12.390742	5.5768
ABCC5	47.12862	21.77427	9.87106	17.452806	8.169626	13.619034	29.355707	10.564068
ABCC6	0	0.221495	0	0.760473	0	0.561822	0	1.225027
ABCC6P1	0	0	2.046573	0.442961	0	0	0	0
ABCC6P2	0	0.358768	0	0	0	0	0	0
ABCC8	0	0	0	0	0	0	0	0
ABCC9	0	0.180966	0	0	0.248605	0	0	0
ABCD1	2.712978	3.598261	5.655092	7.393048	1.671451	9.25591	0	10.509656
ABCD1P2	0	0	0	0	0	0	0	0
ABCD1P3	0	0	0	0	0	0	0	0
ABCD1P4	0	0	0	0	0	0	0	0
ABCD1P5	0	0	0	0	0	0	0	0
ABCD2	0	0	0	0	0	0	0	0
ABCD3	86.358421	75.378459	94.374007	29.838202	29.440343	8.467305	36.308545	41.044273
ABCD4	25.648205	19.449514	11.785235	29.089516	19.043341	38.341465	48.077122	41.035375
ABCE1	113.469537	231.646042	130.439631	133.255618	177.241939	102.084882	196.78559	297.056621
ABCF1	34.50906	61.236585	53.50913	105.798547	123.898125	165.231424	122.88184	101.010459
ABCF2	21.770541	13.607858	12.381227	16.608164	3.491873	41.715439	8.499734	28.513406
ABCF2-H2BK	0	22.43775	0	22.365198	47.670492	41.803891	70.561331	39.600496
ABCF2P1	0	0	0	0	0	0	0	0
ABCF2P2	0	0	0	0	0	0	0	0
ABCF3	0	1.815396	0	7.529484	27.872233	9.336195	48.783738	5.098789
ABCG1	1.159056	2.424476	6.184252	1.589088	0.910995	5.784612	0	0
ABCG2	3.917983	6.952392	10.05843	2.572198	0.413895	2.236646	0	3.376209
ABCG4	0	0.371836	0	0.381641	0	0	0	0.104681
ABCG5	0	0.172728	0	0	0	0.134513	0	0
ABCG8	0	0.273926	0	0.323772	0	0	0	0

ABHD1	6.504168	1.602889	0	0.535545	2.95695	0.917272	0	0
ABHD10	26.044759	25.078707	43.528705	26.300659	37.781277	7.975081	30.157662	22.648894
ABHD11	8.187551	3.779697	0	4.551468	28.925304	6.636596	0	5.203835
ABHD12	26.615077	32.606041	28.50943	36.271744	15.103842	39.510394	37.722197	38.022573
ABHD12B	0	0.618067	0	1.354894	2.479897	4.756502	0	6.008067
ABHD13	7.988305	8.234005	5.521387	3.229309	10.064834	2.462575	1.347408	8.110066
ABHD14A	0	8.452932	5.58845	14.809221	0	11.439726	39.414696	13.939921
ABHD14A-A(	0	0	0	0.912623	26.566266	0	0	0.207419
ABHD14B	5.166502	23.734855	23.886105	27.281659	14.164243	16.510154	55.836268	15.095962
ABHD15	6.668954	18.372124	2.563201	17.915018	14.337259	26.53032	6.718531	25.011897
ABHD16A	15.289451	9.15597	26.701695	10.402032	21.776244	10.84248	26.811622	9.700369
ABHD16B	0	0	0	0.291606	0	0.591955	0	0.390451
ABHD17A	10.560402	4.499082	1.392659	0.658163	7.597535	1.030112	0	1.016894
ABHD17AP1	0	0	0	0	0	0	0	0
ABHD17AP3	0	0	0	0	0	0	0	0
ABHD17AP4	0	0	0	0	0	0	0	0
ABHD17AP5	0	0	0	0	0	0	0	0
ABHD17AP6	0	0	0	0	0	0	0	0
ABHD17AP7	0	0	0	0	0	0	0	0
ABHD17AP8	0	0	0	0	0	0	0	0
ABHD17AP9	0	0	0	0	0	0	0	0
ABHD17B	13.966372	10.39052	0	8.897023	0.811064	6.617451	0	18.266825
ABHD17C	40.531925	79.265532	41.425888	67.187946	43.147096	62.722195	57.866658	92.736919
ABHD18	0	3.787574	2.183058	1.452418	0	1.268731	0	4.832928
ABHD2	29.452381	45.735887	36.052359	75.956714	55.499314	71.767563	75.401101	77.578712
ABHD3	66.989765	17.829169	28.809925	11.948667	9.456509	10.241248	52.886269	13.729072
ABHD4	0	21.530045	0	7.073792	39.073728	7.083984	34.040643	6.384343
ABHD5	23.642407	35.768534	24.66656	24.461251	10.984748	21.818409	51.537745	33.789548
ABHD6	4.279808	5.470509	5.103843	2.596016	2.004905	0	0	3.550683
ABHD8	8.480239	22.406458	6.058567	25.337103	9.390566	45.583209	9.446419	29.044273
ABI1	6.420603	6.138913	9.822003	8.671709	9.963016	5.844137	0	9.559582
ABI1P1	0	0	0	0	0	0	0	0
ABI2	49.467001	74.285539	104.831889	92.701227	51.720826	77.957165	106.314755	54.8463

ABI3	0	0	0	0	0	0	0	0
ABI3BP	0	6.723795	0.815658	3.327576	2.143197	2.619498	0	1.324511
ABITRAM	38.541183	58.947037	32.754099	22.647698	13.061472	10.430447	1.33226	39.991224
ABITRAMP1	0	0	0	0	0	0	0	0
ABL1	21.7101	37.141546	15.624636	41.119556	21.7165	57.979213	88.357753	50.639882
ABL2	35.191461	29.415365	14.147389	41.405046	21.016006	43.287544	60.851774	49.548524
ABLIM1	44.782783	103.996302	13.543081	90.351105	82.95893	146.979698	12.537476	112.248665
ABLIM2	0	0	0	0.096338	0	0.524515	0	0.477992
ABLIM3	12.625431	20.844655	15.035693	23.021515	16.8802	19.776868	2.312825	17.711009
ABO	0.510403	1.044269	0	5.220617	0.539461	0.855466	0	0.378408
ABR	34.206712	95.798068	53.688983	73.311935	97.839731	123.32978	344.879557	85.637289
ABRA	0	0	0	0	0	0	0	0
ABRACL	73.736469	112.43864	36.879732	73.066821	67.586513	63.951046	95.02872	127.72872
ABRAXAS1	3.740281	2.35863	10.010824	1.070175	0.984513	0.433056	0	1.820259
ABRAXAS1P1	0	0	0	0	0	0	0	0
ABRAXAS1P2	0	0	0	0	0	0	0	0
ABRAXAS2	23.883483	21.295959	18.33992	16.113188	18.910462	11.951357	44.204195	16.46806
ABT1	10.280291	14.27436	11.256153	14.418664	15.977839	15.227265	3.052169	14.748997
ABT1P1	0	0	0	0	0	0	0	0
ABTB1	3.640909	9.000154	3.249198	8.688964	25.32063	16.914608	11.013314	11.703324
ABTB2	5.340686	6.761757	6.596375	12.580491	4.937715	13.368951	7.154425	11.438535
ABTB3	7.933114	27.27215	8.658042	19.449571	5.793015	21.414478	63.731751	21.199354
ACAA1	11.618744	9.943781	16.542904	10.023087	16.492957	24.175584	0	20.815186
ACAA2	14.974572	42.012983	9.528201	37.937675	0	19.109611	0	24.194131
ACAA2P1	0	1.485649	0	0.19259	0	0.194041	0	0
ACACA	57.00606	86.642265	71.656855	90.78836	64.492624	88.782675	60.107548	91.974427
ACACB	5.216487	5.380398	3.755474	8.355391	6.469425	7.856742	40.595738	4.861547
ACAD10	10.933868	16.701132	8.168738	17.334274	17.082736	5.747482	6.752657	12.615442
ACAD11	46.805456	49.0005	44.948879	46.828369	5.460648	4.230803	87.140015	6.642852
ACAD8	12.09375	9.75284	4.963441	7.217818	4.457098	10.687166	0	5.022807
ACAD9	36.238766	40.627563	36.158716	53.736423	105.150258	50.57657	48.232026	43.874962
ACADL	0	0.460027	0	0.065811	0	0	0	0
ACADM	74.102541	99.184091	73.25651	83.338532	82.677838	24.579993	72.870433	62.537817

ACADS	0	4.847545	5.03497	5.341027	0.864544	4.73923	0	4.178015
ACADSB	8.90754	3.842294	5.948245	4.810722	0	3.822131	4.85389	10.452898
ACADVL	61.053274	66.047934	66.179891	73.055859	110.780334	90.440679	46.716056	53.969936
ACAN	0	0	0	0	0	0	0	0
ACAP1	0	0	0	1.867391	0	1.415242	0	3.643044
ACAP2	53.568148	80.042038	27.404779	32.982616	36.725046	21.856462	107.643425	51.736551
ACAP3	0	6.02843	29.102981	7.964466	29.392294	26.00749	5.831684	25.106054
ACAT1	0	0	88.619621	27.487395	0	52.292022	267.888415	0
ACAT2	0	69.195399	0	16.670077	45.285333	29.682004	69.246309	16.920253
ACBD3	37.232932	57.803477	42.40169	47.680692	26.676357	28.133508	11.04647	23.874925
ACBD4	9.253312	18.000574	6.032414	13.476245	1.193783	13.088657	0	7.203556
ACBD5	1.720303	13.39947	7.18875	10.77367	7.84375	14.122366	1.084935	17.296848
ACBD6	62.796662	152.369654	35.645896	142.82175	62.789031	164.07011	64.882631	171.823766
ACBD7	2.967554	3.547065	1.773859	4.342194	2.08915	1.282989	5.05038	1.630099
ACCS	4.500149	0	3.955873	5.318802	0	0.782908	0	1.757883
ACCSL	0	0	0	0	0	0	0	0
ACCSLP1	0	0.771498	2.739606	0.596053	0.813106	1.959274	0	0.879431
ACD	23.168398	22.424109	23.771139	26.709938	31.624812	31.272865	0	25.195947
ACE	1.423382	0.877694	3.108121	2.005925	0	1.226591	0	0.098837
ACE2	0	0	3.414301	0	0	0	0	1.288467
ACE3P	0	0	0	0	0	0	0	0
ACER2	2.434492	3.526843	3.270011	2.283036	5.781891	1.393082	0	3.183859
ACER2P1	0	0	0	0.117549	0	0	0	0
ACER3	29.676426	36.962262	30.3081	12.497767	81.711416	11.677647	40.03774	17.025443
ACHE	0	0.51873	0	0	0	0	0	0
ACIN1	43.05691	64.752887	28.306337	55.421305	126.112982	110.016909	25.26307	76.567659
ACKR1	0	0	0	0	0	0	0	0
ACKR2	0	0.278697	0	0.937705	0	0	0	0.047045
ACKR3	15.48947	12.323293	47.066704	19.985018	5.898638	15.681486	43.644078	15.691336
ACKR4	0	0	0	0	0	0	0	0
ACKR4P1	0	0	0	0	0	0	0	0.295436
ACLY	159.963524	133.423142	127.100102	120.550543	156.19562	142.741972	106.238765	99.283957
ACMSD	0	0	0	0.071147	0	0	0	0

ACNATP	0	0	0	0	0	0	0	0
ACO1	13.913679	16.083582	16.54583	12.862724	18.067787	16.6519	26.694383	23.84536
ACO2	45.303273	37.198678	55.327749	35.287195	28.283339	42.291372	21.942664	33.718265
ACO2P1	0	0	0	0	0	0	0	0
ACO2P2	0	0	0	0	0	0	0	0
ACOT1	7.469645	5.358777	4.474671	5.474326	0	3.182188	23.823869	1.314683
ACOT11	0	1.568381	6.293813	6.225955	0.927207	0	0.284434	0.083846
ACOT12	0	0	0	0	0	0	0	0
ACOT13	34.574462	41.80589	14.649166	45.519368	28.472258	27.912975	78.748404	48.249368
ACOT2	19.425151	4.347533	23.775197	8.245982	1.212342	5.605367	0	6.42909
ACOT6	0	0	0	0.329113	0	0	0	0
ACOT7	18.093753	38.493064	41.342538	44.221034	91.851396	103.015374	88.44221	73.564384
ACOT8	6.079084	14.301639	18.99383	15.351778	5.181847	19.407167	8.654219	15.104377
ACOT9	38.214833	54.100735	28.243879	39.773893	58.458508	52.156326	35.740966	74.193653
ACOX1	18.607023	17.12926	24.47256	12.696148	18.326528	9.388533	116.398131	17.49886
ACOX2	6.872502	0	0	0.206141	0	0	0	0
ACOX3	5.862106	6.808347	13.705829	3.814992	2.08977	5.809456	0	5.813246
ACOXL	0	1.114153	0	0.514626	0	0.273023	0.381062	2.861287
ACP1	124.341436	146.74519	116.919583	130.894481	70.523801	118.54812	47.317015	133.653321
ACP2	7.81575	6.166191	8.382052	6.853537	1.482749	8.167424	0	7.295365
ACP3	0	0	0	0.416903	0	0.390597	0	0
ACP5	0	0	0	0.381588	0	0	0	0
ACP6	57.33844	86.656342	44.958272	71.595949	13.640402	54.343635	0	48.928312
ACP7	0	0.516044	1.044343	0.603687	0	0	0.783725	0
ACR	0	0	0	0	0	0	0	0.247894
ACRBP	0	0	0	0.261287	0	0	0	0
ACRP1	0	0	0	0	0	0	0	0
ACRV1	0	0	0	0	0	0	0	0.432851
ACSBG1	0	0.044943	6.969659	0.577706	0	0	0	0.943383
ACSBG2	0	0	0	0	0	0	0	0
ACSF2	22.263001	11.55151	8.82449	3.067065	57.060048	5.287242	184.411212	12.730741
ACSF3	25.906074	44.523243	23.92619	55.609511	31.809242	59.604204	24.698367	55.43708
ACSL1	14.320211	18.065226	3.701583	13.706086	26.348319	34.421241	15.637132	30.032381

ACSL3	73.085437	176.228086	80.124235	145.565012	139.700287	117.094608	203.090474	145.403844
ACSL3P1	0	0	0	0	0	0	0	0
ACSL4	65.090676	103.89408	81.243853	87.714701	187.28437	57.820072	70.630742	118.407429
ACSL5	0	0	12.61526	0	1.702912	0	0	0
ACSL6	0	0.42685	0	0.270861	0	0.544733	0	0.406537
ACSM1	0	0.730577	4.416592	1.116916	0	0	0	0.09212
ACSM2A	0	0	0	0	0	0	0	0
ACSM2B	0	0	0	0.252274	0	0	0	0
ACSM3	0	4.316015	6.30917	4.417705	6.224651	2.22864	0	2.10231
ACSM4	0	0	0	0	0	0	0	0
ACSM5	0	0	0	0	0	0	0	0
ACSM5P1	0	0	0	0	0	0	0	0
ACSM6	0	0	0	0.26417	0	0	0	0
ACSS1	6.398204	2.854382	1.700112	3.419817	4.592749	5.438979	0	2.880256
ACSS2	74.795354	60.752334	27.516935	40.284285	21.417874	23.289088	130.741868	22.600515
ACSS3	12.078671	5.637382	0.345525	2.880684	0	0	0	0.31288
ACTA1	0	0	0	0	0	0	0	0
ACTA2	8.113775	5.524961	2.401613	11.287696	0	3.67556	0	1.931083
ACTB	4348.09083	4857.03912	4017.70738	4685.23306	3489.40003	3929.65732	3620.84063	4121.38873
ACTBL2	0	0.154364	0	0.441195	1.593189	0	0	0.291516
ACTBP1	0	0	0	0	0.891352	0	0	0
ACTBP11	0	0	0	0.080479	0	0	0	0
ACTBP12	0	0	0	0	0	0	0	0
ACTBP14	0	0	0	0	0	0	0	0
ACTBP15	0	0	0	0	0	0	0	0
ACTBP16	0	0	0	0	0	0	0	0
ACTBP2	0	0.418087	0	0	0	0.16317	0	0
ACTBP4	0	0	0	0	0	0.344913	0	0
ACTBP6	0	0	0	0	0	0	0	0
ACTBP7	0	0.277846	0	0.322584	0	0.162914	0	0.272742
ACTBP8	0	0	0	0	0	0	0	0
ACTBP9	0	0	0	0	0	0	0	0
ACTC1	0	0	0	0.125646	0	0.242011	0	0

ACTE1P	0	0	0	0	0	0	0	0
ACTG1	2033.78345	1428.89018	2140.36605	1494.96835	2122.84232	1113.92976	1760.31884	1864.42444
ACTG1P1	3.333963	0.414658	0	2.487053	0	0.162083	0	0.678186
ACTG1P10	0	0	0	0	0	0	0	0
ACTG1P12	0	0	0	0	0	0	0	0
ACTG1P13	0	0	0	0	0	0	0	0
ACTG1P14	0	0	0	0.080815	0	0	0	0.136667
ACTG1P15	0	0	0	0	0	0	0	0
ACTG1P16	0	0	0	0	0	0	0	0
ACTG1P17	0	0.139634	0	0	0	0.327506	0	0
ACTG1P18	0	0	0	0	0	0	0	0
ACTG1P19	0	0	0	0.081325	0	0	0	0
ACTG1P2	0	0	0	0	0	0	0	0
ACTG1P20	0	2.086585	0	0.509488	0	1.489495	0	1.864208
ACTG1P21	0	0	0	0	0	0	0	0
ACTG1P22	0	0	0	0	0	0	0	0
ACTG1P23	0	0	0	0	0	0	0	0
ACTG1P24	0	0.556729	0	0	0	0.436225	0	0
ACTG1P25	0	2.107366	3.276533	2.590243	0	0.878018	0	0.237678
ACTG1P3	0	0.701758	0	0.162992	0	0.658402	0	0.137863
ACTG1P4	0	0	0	0	0	0	0	0
ACTG1P9	0	0	0	0.031018	0	0	0	0
ACTG2	0	0	0	0	0	0	0	0
ACTL11P	0	0.178759	0	0.020381	0	0	0	0
ACTL6A	111.83295	181.953942	57.087763	161.763634	227.876219	148.12377	277.80724	153.221956
ACTL6B	0	0	0	0	0	0	0	0
ACTL8	0	0	0	0.102767	0	0	0	0.342915
ACTMAP	4.043019	17.554808	30.965003	21.361767	8.271656	22.005768	5.651299	29.830589
ACTN1	148.006833	242.114512	78.589272	201.488963	200.154776	722.638414	0	457.039604
ACTN2	0	0	0	0	0	0	0	0
ACTN3	0	0.249012	0	0	0	0	0	0
ACTN4	238.256245	265.90584	109.874153	354.835875	549.296859	781.225144	185.82194	535.995956
ACTN4P1	0	0	0	0	0	0	0	0

ACTN4P2	0	0	0	0	0	0	0	0
ACTP1	0	0	0	0	0	0	0	0
ACTR10	82.948468	84.403425	102.51116	48.899078	69.266907	27.779714	80.951315	56.056027
ACTR1A	78.244758	52.822706	50.134204	53.859943	83.858851	39.602544	63.636964	37.88913
ACTR1AP1	0	0	0	0	0	0	0	0
ACTR1B	0	3.140556	0	0.890652	16.341115	5.464794	0	1.238517
ACTR2	42.171965	80.207423	0	89.787836	69.673893	122.877135	0	54.904148
ACTR2P1	0	0	0	0	0	0	0	0
ACTR2P2	0	0	0	0	0	0	0	0
ACTR3	178.933724	205.457178	81.721357	146.917553	245.849439	178.014947	223.022149	259.052482
ACTR3B	14.627112	17.781695	7.447778	13.103619	4.201301	10.220529	0	7.860599
ACTR3BP1	0	0	0	0	0	0	0	0
ACTR3BP2	0	0	0	0	0	0	0	0
ACTR3BP3	0	0	0	0	0	0	0	0
ACTR3BP4	0	0	0	0	0	0	0	0
ACTR3BP5	0	0	0	0	0	0	0	0
ACTR3BP6	0	0	0	0	0	0	0	0
ACTR3BP7	0	0	0	0	0	0	0	0
ACTR3C	0	2.924387	0	6.966624	1.208876	3.677372	0	8.536409
ACTR3P2	0	0	0	0	0	0	0	0
ACTR3P3	0	0	0	0	0	0	0	0
ACTR5	8.00187	4.992974	9.547578	6.038167	15.129834	5.5671	6.795868	6.175333
ACTR6	16.729733	17.110216	40.815353	15.520036	49.935841	10.197779	18.061962	19.713434
ACTR6P1	0	0	0	0	0	0	0	0
ACTR8	2.78489	31.913661	10.589778	23.054671	12.635804	30.534617	54.755862	18.81475
ACTRT1P1	0	0	0	0	0	0	0	0
ACTRT3	6.357268	13.296227	5.663754	7.959155	0	5.637477	0	8.22437
ACVR1	0	18.734876	15.819201	21.627838	0	15.656028	0	18.005778
ACVR1B	16.675391	25.739517	17.578254	26.262879	14.012813	23.26262	12.710276	21.800133
ACVR1C	0	0	2.573405	0.090953	0	0	0	0.397299
ACVR2A	3.163073	8.92172	1.705111	19.360152	4.679359	14.83569	0	19.33833
ACVR2B	2.989322	1.899381	5.382542	3.569081	12.294266	4.470255	1.393111	1.893579
ACVRL1	0	0	0	0	0	0	0	0

ACY1	10.288663	41.853554	25.768377	45.514359	20.51645	58.1616	23.846979	46.261639
ACY3	0	2.220238	0	1.722861	0	0.810222	0	0.968519
ACYP1	16.502268	63.792625	14.217645	70.214524	38.788945	34.661675	0	37.340409
ACYP2	7.955532	8.857582	0	7.173532	1.556871	7.035209	0	3.669668
ADA	16.307499	25.594683	17.090022	24.127469	3.822259	20.051823	0	17.222985
ADA2	0	0	0	0	0	0	0	0
ADAD1	0	0	0	0	0	0	0	0
ADAD1P1	0	0	0	0.043665	0	0	0	0
ADAD1P2	0	0	0	0	0	0	0	0
ADAD2	0	0	0	0	0	0.525351	0	0
ADAL	14.540272	15.965067	13.175965	6.402106	3.248339	5.10394	0	11.605011
ADAM10	179.0471	218.638458	251.63889	280.928821	160.665378	173.135713	165.850916	197.377331
ADAM11	1.178559	0.80313	0	0.419515	0.622059	0.352014	0	0.094832
ADAM12	6.184314	5.737433	5.119283	5.953119	2.470749	4.580709	3.43306	6.036828
ADAM15	36.849775	53.25446	54.270553	46.737379	41.24658	58.25037	25.719144	38.090582
ADAM17	34.564678	49.341253	45.065373	64.283646	19.217733	32.646326	21.885697	26.642638
ADAM18	0	1.376209	0	0.061182	0	0	0	0
ADAM19	0	4.131533	0	6.424711	0	5.054187	8.66282	3.663391
ADAM1A	0	0	0	0	0	0	0	0
ADAM1B	0	0	0	0	0	0	0	0
ADAM2	0	0	0	0	0	0	0	0
ADAM20	0	0	0	0.08834	0	0	0	0
ADAM20P1	0	0	0	0.071567	0	0	0	0
ADAM20P2	0	0	0	0	0	0	0	0
ADAM20P3	0	0	0	0	0	0	0	0
ADAM21P1	0	0	0	0	0	0	0	0
ADAM22	5.939295	3.720934	1.042811	5.153012	3.231188	1.64031	0.171661	7.272244
ADAM23	2.562386	9.287976	0.921496	6.174798	0.135413	3.036356	3.326453	2.511718
ADAM24P	0	0	0	0	0	0	0	0
ADAM28	0	0.738827	2.954541	1.611959	6.061891	0.567845	0	0.27288
ADAM29	0	0	0	0	0	0	0	0
ADAM32	0	0.606151	0	0.126974	0.646606	1.127629	0	0.24596
ADAM33	0	0.307883	0	0	0	0	0	0

ADAM3A	0	0	0	0	0	0	0	0
ADAM3B	0	0	0	0	0	0	0	0
ADAM5	0	0	0	0	0	0	0	0
ADAM6	0	0	0	0	0	0	0	0
ADAM7	0	0	0	0	0	0	0	0
ADAM8	5.124436	8.496915	11.714267	23.588976	45.429055	17.288311	9.251556	10.806258
ADAM9	140.959968	155.18398	160.597805	139.923581	61.448683	44.278864	70.309428	76.335892
ADAMDEC1	0	0	0	0	0	0	0	0
ADAMTS1	20.938745	14.422165	3.747247	7.86648	5.496227	7.303479	8.447509	10.024979
ADAMTS10	0	1.014293	0	0.53482	0	0	0	0
ADAMTS12	23.689713	19.488704	4.733193	15.958676	14.051415	18.934118	10.542113	23.836099
ADAMTS13	0	0.950778	1.376707	0.75534	0	0.304143	0	0.265393
ADAMTS14	0	0	0	0.045449	0	0.031047	0	0
ADAMTS16	0	1.140722	0	0.674939	0	0	0	0
ADAMTS17	0	0	0	0.852671	6.227532	0.305789	0	3.022081
ADAMTS18	0	0	0.501478	0	0	0	0	0
ADAMTS19	0	1.191645	0	0.133888	0	0	0	0
ADAMTS2	0	0.07174	0	0.411547	0	0	0	0
ADAMTS20	0	0	0	0	0	0	0	0
ADAMTS3	0	0.902594	0	0.756971	0	0.37238	0	0
ADAMTS4	0	0	0	0	0	0	0	0
ADAMTS5	0	0	0	0	0	0.016783	0	0
ADAMTS6	30.067599	7.227217	5.182899	4.427136	2.090778	4.817978	0	3.74225
ADAMTS7	0	0.541079	0	0.24452	0.564316	0.562324	0	0.165841
ADAMTS7P1	0	0	0	0	0	0	0	0
ADAMTS7P3	0	0	0	0	0	0	0	0
ADAMTS7P4	0	0	0	0	0	0.045912	0	0
ADAMTS7P5	0	0	0	0	0	0	0	0
ADAMTS8	0	0	0	0	0	0.045687	0	0.122954
ADAMTS9	0	0.098635	0	0.0728	0	0	0	0
ADAMTSL1	0.830835	0.96598	0.989099	0.983648	0	0.312847	6.954505	0.706694
ADAMTSL2	0	0	0	0.065161	0	0	0	0
ADAMTSL3	3.281629	10.086851	6.944022	11.18736	0.173376	1.206053	0	0.583718

ADAMTSL4	3.470787	0.245259	3.551354	3.533356	4.630105	1.300414	0	0
ADAMTSL5	6.859982	3.54855	13.365699	4.438497	0.332756	14.306672	0	4.915748
ADAP1	6.171151	39.790914	37.322198	62.458271	18.374819	50.734947	82.186551	39.071747
ADAP2	0	8.270513	3.833163	2.301719	0	1.048534	0	2.577906
ADAR	35.592945	190.97004	59.471	160.080638	143.277487	260.18695	115.492234	259.179448
ADARB1	4.726864	26.901369	7.503929	19.171282	4.579625	28.725447	6.562709	24.617609
ADARB2	0	0	0	0	0	0.769351	0	0
ADAT1	49.425188	237.890851	36.447383	209.370803	55.620398	271.125114	194.207617	244.791587
ADAT2	3.640614	4.104287	11.657257	2.050687	2.446852	1.225805	1.267808	2.009359
ADAT3	0	2.247479	2.259212	1.509985	0	3.835245	0	2.972597
ADCK1	16.627852	8.639652	12.666179	11.5983	0.960134	8.056929	0	6.743417
ADCK2	31.392256	23.279593	10.620611	19.122009	14.263572	22.033385	15.039044	23.59021
ADCK5	3.548677	0.300216	3.167714	2.497703	0.467744	1.707821	0	3.241105
ADCY1	1.263465	4.181492	6.608915	4.02443	21.02103	17.48264	0.863683	12.195121
ADCY10	0	0	0	0	0	0.098998	0	0.107123
ADCY10P1	0	0.288583	0	0.12597	0	0	0	0.103648
ADCY2	0	0	0	0	0	0	0	0
ADCY3	21.587816	40.170977	19.603078	32.491607	62.69177	62.625929	45.064023	49.43802
ADCY4	0	0.618795	0	0.805342	0	0	0	0.333371
ADCY5	0	0	0	0.102149	0	0	0	0
ADCY6	0	2.272327	5.866097	5.475911	21.576551	4.86571	0	4.04695
ADCY7	10.05758	21.583412	17.607147	17.699147	27.433258	39.931191	19.483674	30.785648
ADCY8	0	0	0	0	0	0	0	0
ADCY9	10.54449	7.072831	2.545884	11.498183	2.457449	21.939282	7.001694	15.362201
ADCYAP1	0	0	0	0	0	0	0	0
ADCYAP1R1	0	0	0	0	0	0	0	0
ADD1	55.070822	93.285969	40.645805	83.902761	45.164708	125.054737	40.432114	91.847601
ADD2	37.614123	74.179443	35.514258	80.71862	30.159157	114.392299	39.148898	80.708753
ADD3	19.463795	46.80423	58.07173	32.07107	54.080593	28.155569	159.707852	21.236656
ADGB	0	0	0	0	0	0	0	0
ADGRA1	0	0.662437	0	0.354378	0	0	0	0
ADGRA2	0	0	0	0.052277	2.116124	0	0	0
ADGRA3	30.155262	30.773448	23.739729	22.375805	34.219875	12.906594	28.9289	17.570274

ADGRA3P1	0	0	0	0	0	0.131348	0	0
ADGRB1	7.521156	0	0	1.578154	0	0	0.263252	0
ADGRB2	8.426836	4.762212	3.014892	4.181782	4.094875	6.029742	0	2.570321
ADGRB3	0	0	0	0.052201	0	0	0	0
ADGRD1	0	2.462546	0	1.03354	0	0	0	0
ADGRD2	0	0	0	0.113642	0	0.115384	0	0
ADGRE1	0	0	0	0	0	0	0	0
ADGRE2	1.00176	2.071	6.653542	0.97244	2.991567	4.378046	0	0.888475
ADGRE3	0	0	0	0	0	0	0	0
ADGRE4P	0	0	0	0	0	0	0	0
ADGRE5	9.882747	13.490731	22.469331	14.910037	21.793409	22.730991	48.753044	21.695904
ADGRF1	15.817441	25.79553	33.859465	47.822945	34.814641	44.204866	33.629608	63.780225
ADGRF3	0	0	0	0.817913	0	1.045121	0	1.513442
ADGRF4	1.090485	2.347944	1.954392	0.644036	5.756659	2.35497	0	0.187354
ADGRF5	0	0	0	0	0	0	0	0
ADGRF5P1	3.312938	1.236461	0	1.165242	0	0	0	0.808603
ADGRF5P2	0	0	0	0	0	0	0	0
ADGRG1	52.45273	66.436568	82.656669	103.995681	55.51382	98.082727	109.989124	71.361755
ADGRG2	0	0.689856	0	0.285789	0	0.139772	0	0.264168
ADGRG3	0	0	0	0	0	0	0	0
ADGRG4	0	0	0	0	0	0	0	0
ADGRG5	0	0	0	0	0	0	0	0
ADGRG6	6.726126	31.465166	28.104584	35.294374	63.5176	44.012234	42.887285	34.091443
ADGRG7	0	0	0	0	0	0	0	0.070366
ADGRL1	3.916151	13.172147	10.466905	17.238587	21.294793	14.439078	30.982813	11.204002
ADGRL2	22.02322	31.596542	14.462206	20.210409	13.219773	12.103143	7.38658	19.742439
ADGRL3	0	1.858696	0.622526	1.675822	0	0.034691	0	0
ADGRL4	0	0	0	0.755278	0	0	0	0
ADGRV1	0	0	0.924132	0.582893	0	0	0	0
ADH1A	0	0	0	0	0	0	0	0
ADH1B	0	0	0	0	0	0	0	0
ADH1C	0	0	0	0	0	0	0	0
ADH4	0	0	0	0	0	0	0	0

ADH5	94.492074	135.414315	73.656569	126.748696	167.980664	116.907968	197.736329	150.485929
ADH5P2	0	0	0	0	0	0	0	0
ADH5P3	0	0	0	0	0	0	0	0
ADH5P4	0	0	0	0	0	0	0	0
ADH5P5	0	0	0	0	0	0	0	0
ADH6	0	0	0	0	0	0	0	0
ADH7	0	0	0	0	0	0	0	0.112012
ADHFE1	0	1.004906	1.707566	3.034034	0	4.764765	0	0.615323
ADI1	29.982366	29.334797	16.922145	18.796764	63.669712	43.229898	63.806614	47.564167
ADI1P1	0	0	0	0	0	0	0	0
ADI1P2	0	0	0	0	0	0	0	0
ADI1P3	0	0	0	0	0	0	0	0
ADIG	0	0	0	0	0	0	0	0
ADIPOQ	0	0	0	0	0	0	0	0
ADIPOR1	140.460578	88.143126	135.80077	92.783061	84.964891	67.072085	124.877009	71.918352
ADIPOR1P1	0	0	0	0	0	0	0	0
ADIPOR2	85.718348	162.329463	75.432238	156.018316	88.081797	177.754097	152.291262	153.379851
ADIRF	0	0	0	26.042584	0	76.939966	0	59.375272
ADISSP	32.329081	75.650487	34.083274	76.817025	56.601664	191.688893	63.424405	121.148301
ADK	0	0	0	0.353105	0	1.641942	0	3.122131
ADM	31.316968	80.743562	93.496678	116.920587	181.69377	196.78816	109.234903	147.468368
ADM2	3.911176	5.945685	9.830778	13.351873	0.206588	1.360841	0	1.267791
ADNP	75.896663	211.929055	69.202795	131.457508	86.302969	111.420423	158.845873	234.937854
ADNP2	35.497281	92.239247	22.206407	77.40256	23.941349	84.41322	78.506299	141.552898
ADORA1	0	0.182653	0	0.337103	0	0.289288	0	0.046748
ADORA2A	0	0	0	0	0	0	0	0
ADORA2B	0	0	0	0	0.798982	0	0	0.681494
ADORA2BP1	0	0	0	0	0	0	0	0
ADORA3	0	0	0	0.3893	0	0	0	0
ADPGK	35.453063	33.761954	38.060071	38.209625	22.933285	36.578442	31.210691	31.797409
ADPRH	2.667963	1.256852	5.947039	1.367419	0	1.842765	12.155134	5.313158
ADPRHL1	3.722611	2.112722	4.219978	2.339526	0	0.291492	0	0.448547
ADPRM	0	0.412149	11.690937	0	0.933262	3.380016	0	0

ADPRS	15.035801	21.341827	13.393364	17.485646	17.541305	42.877329	1.037671	34.886624
ADRA1A	0	0	0	0	0	0	0	0
ADRA1B	0	0.634676	2.427672	1.716924	0	1.915632	0	0
ADRA1D	0	0	0	0	0	0	0	0
ADRA2B	0	0.537559	0	0.459876	0	0.089623	5.020578	0
ADRA2C	0	0	0	0	0	0	0	0
ADRB3	0	0	0	0	0	0	0	0
ADRM1	92.694787	143.23843	102.543623	145.079271	110.482779	211.627983	168.315534	165.95297
ADSL	117.712384	93.108379	52.68351	72.653802	57.765153	71.065451	44.987716	86.959766
ADSS1	0	6.024614	8.742687	0.574471	0	0	0	3.186503
ADSS2	50.507545	54.592403	49.956963	28.019073	48.392807	22.722821	79.978406	50.572765
ADTRP	0	16.850219	0	22.097964	0	6.063154	0	4.523977
AEBP1	0	0.26887	0	0.701051	0	0.724699	0	0.484215
AEBP2	71.877094	91.657123	106.55025	74.331	75.138476	42.452657	14.829139	65.887139
AEN	8.72992	22.009209	10.756551	15.366676	19.390938	18.563455	16.704697	19.717364
AFAP1	7.737565	15.496793	0	11.855744	0	18.238218	3.639981	15.018392
AFAP1L1	7.264911	15.76106	14.985108	17.987484	55.683978	78.543825	35.636885	51.489139
AFAP1L2	61.958237	103.964807	51.145245	107.240322	23.756014	65.188129	63.245245	56.506803
AFDN	18.487942	38.512046	26.266473	57.191692	48.441489	42.22779	66.060067	32.241654
AFF1	11.199522	1.363165	13.347652	21.306787	40.250692	23.039834	0	26.809411
AFF2	0	0	0	0	0	0	0	0
AFF3	0	0	0	0	0	0	0	0
AFF4	61.093483	172.33522	47.315425	210.70487	57.4795	174.690113	113.968952	189.72224
AFF4P1	0	0	0	0	0	0	0	0
AFG1L	0	4.467644	0	3.327106	11.851338	4.650809	1.256494	9.607057
AFG2A	4.768202	3.447671	5.719621	5.445831	3.964966	2.983979	10.61681	4.543176
AFG2B	2.651583	8.008742	9.491777	5.325137	14.591296	2.343088	0	4.653817
AFG3L1P	2.266439	5.192194	3.998417	5.799225	12.088731	17.435206	0	12.682028
AFG3L2	0.990117	10.184073	20.644535	14.746236	22.682289	6.320214	19.994217	41.572224
AFG3L2P1	0	0	0	0	0	0	0	0
AFM	0	0	0	0	0	0	0	0
AFMID	64.990597	39.576232	50.85893	48.667144	9.460253	26.886655	86.971495	26.574023
AFP	0	0	0	0	0	0	0	0

AFTPH	24.5198	24.267307	16.354826	25.239883	1.833954	19.391304	8.200929	28.719395
AGA	5.10187	18.182651	29.168163	17.484566	1.345117	9.034997	0	3.340909
AGAP1	6.100208	10.962976	3.59658	4.240454	15.795175	13.946398	50.592879	26.859769
AGAP10P	0	0	0	0.181867	0	0.204247	0	0
AGAP11	0	0.413042	0	0.361754	0.533613	0.532794	0	0.777102
AGAP12P	0	0	0	0.082056	0	0	0	0
AGAP14P	1.906332	0.115116	0	0.19707	0	0.341761	0	0.868728
AGAP2	19.841784	24.756982	3.809465	38.570999	0.159999	4.178754	1.38762	3.935475
AGAP3	45.317217	18.648963	33.239329	36.758358	46.854988	47.095948	103.031576	38.34074
AGAP4	0	0.408258	0	2.684201	0	1.069282	0	0.820241
AGAP5	0	0	0	0.232692	2.598074	0	0	2.109275
AGAP7P	3.971897	0.355999	0	0.402198	0	0.95647	0	0
AGAP9	5.003948	3.905341	0	2.835888	0	2.640137	0	0.73437
AGBL1	0	0	0	0	0	0	0	0
AGBL2	20.947426	2.754245	0	3.006101	7.203153	2.972201	0	1.445828
AGBL3	0	1.726117	0	0.831359	0	2.039933	0	4.142991
AGBL4	0	0	0	0	0	0	0	0
AGBL5	27.241079	21.798116	24.363573	24.353719	11.491398	21.1322	0	16.116929
AGER	4.480956	0	0	0.256929	0	1.618624	0	1.956246
AGFG1	46.088246	57.693434	57.428769	45.880469	59.430204	40.483563	89.981787	85.634344
AGFG2	2.719885	5.656288	3.259914	3.910828	1.257302	3.110895	0	1.768795
AGGF1	15.531095	22.621979	20.373791	20.336693	23.77813	24.58089	13.365403	26.359247
AGGF1P1	0	1.551747	0	0.906306	0	0.127948	0	1.209013
AGGF1P10	0	0	0	0	0	0	0	0
AGGF1P2	0	1.183879	0	1.140941	0	0.442629	0	1.324521
AGGF1P3	0	0	0	0.073796	0	0	0	0.124393
AGGF1P4	0	0	0	0	0	0	0	0
AGGF1P5	0	0	0	0	0	0	0	0
AGGF1P6	0	0	0	0	0	0	0	0
AGGF1P7	0	0	0	0	0	0	0	0
AGGF1P8	0	0	0	0	0	0	0	0
AGGF1P9	0	0	0	0	0	0	0	0
AGK	66.374403	39.38652	59.467363	42.875758	68.153653	17.762172	24.31745	21.750591

AGKP1	0	0	0	0	0	0	0	0
AGKP2	0	0	0	0	0	0	0	0
AGL	32.942981	28.248883	33.651558	16.964616	55.695744	5.154422	29.932903	18.106763
AGMAT	15.156669	10.860927	5.821144	6.808768	7.429785	3.830492	0	5.354787
AGMO	0	0	0	0	0	0	0	0.110747
AGO1	16.348595	15.572891	15.414047	14.525071	36.76067	27.613051	8.607209	23.989131
AGO2	50.280942	52.025956	61.646233	56.957681	35.119414	48.166998	36.379556	62.006442
AGO3	15.196484	26.710291	7.228055	34.179746	9.06856	31.026687	19.442264	39.310405
AGO4	21.368908	13.29337	9.598377	4.765316	36.330427	4.619699	0	6.790375
AGPAT1	29.583422	26.268214	35.344393	34.831649	39.616662	48.707088	47.453673	31.035587
AGPAT2	43.956784	103.054267	41.177499	115.193817	4.963734	80.435803	0	82.633298
AGPAT3	19.768735	37.474202	24.271673	46.644478	67.320211	102.075234	54.301609	94.112021
AGPAT4	1.221777	1.332971	2.638745	2.973324	1.722111	0.756603	0	2.591877
AGPAT5	18.750436	40.056019	20.742215	20.014978	23.064432	26.174746	43.297214	25.610475
AGPAT5P1	0	0	0	0	0	0	0	0
AGPS	11.825917	11.902326	0	12.499228	18.366957	14.668922	32.002555	10.01331
AGR2	29.157574	61.114701	25.079208	35.26354	0	3.785789	11.574739	3.737542
AGR3	0	0	0	0	0	0	0	0
AGRN	36.627888	26.826377	58.74361	38.970244	66.786416	71.623767	36.699338	29.871073
AGTPBP1	10.433645	6.043226	7.030669	1.102861	8.066946	2.190999	3.622112	5.316899
AGTR1	0	0	0	0	0	0	0	0
AGTR2	0	0	0	0	0	0	0	0
AGTRAP	22.033462	39.444174	43.986023	54.498058	11.750909	44.327019	25.143974	36.852826
AGXT	0	0	0	0	0	0	0	0
AGXT2	0	0	0	0	0	0	0	0
AHCTF1	28.056197	11.339826	1.899459	5.104126	41.686558	14.048905	23.571625	6.315878
AHCTF1P1	0	0	0	0.087231	0	0	0	0.025732
AHCY	169.907023	111.927098	233.21334	139.653654	169.921795	115.473245	142.139544	96.858334
AHCYL1	145.04585	260.183997	142.477888	337.407828	79.161339	199.957557	97.925753	153.95482
AHCYL2	3.254522	1.966948	21.453255	4.002731	27.882233	4.12659	36.910271	2.640692
AHCYP2	0	0	0	0	0	0	0	0
AHCYP3	0	0	0	0	0	0	0	0
AHCYP4	0	0	0	0	0	0	0	0

AHCYP5	0	0	0	0	0	0	0	0
AHCYP7	0	0	0	0	0	0	0	0
AHCYP8	0	0	0	0	0	0	0	0
AHDC1	17.422009	22.754688	4.315334	31.882729	39.748549	143.003476	94.971824	73.564066
AHI1	24.714456	9.110742	3.195524	9.042684	10.033165	13.64232	0	13.426465
AHNAK	62.267185	84.748808	102.124345	177.412277	290.114457	458.150336	231.821068	371.189439
AHNAK2	16.149196	38.254797	22.055398	59.644335	40.400993	107.994601	53.96203	62.804518
AHR	19.766596	38.32543	27.594001	55.892955	84.227883	42.136427	88.319777	91.961621
AHRR	0	0.386578	0	0.284117	0	0.30816	0	0
AHSA1	202.24007	613.95437	219.257119	612.967608	254.445172	419.892312	392.429037	390.944199
AHSA2P	0	4.389558	0	3.156202	1.010163	1.061944	0	0
AHSG	0	0.580182	0	0	0	0	0	0
AHSP	0	0	0	0	0	0	0	0
AICDA	0	0	0	0.058625	0	0	0	0
AIDA	74.524564	67.001944	49.569922	46.328197	51.962202	35.963303	174.091364	67.383001
AIDAP1	0	0	0	0	0	0	0	0
AIDAP2	0	0	0	0.104105	0	0	0	0
AIDAP3	0	0	0	0	0	0	0	0
AIF1	0	0	0	0	0	0	0	0
AIF1L	9.879444	19.745282	9.744061	16.93482	35.854621	42.512616	97.427623	21.516317
AIFM1	34.018145	61.141043	40.376137	60.368278	20.870554	57.191832	25.431755	55.263103
AIFM1P1	0	0	0	0	0	0	0	0
AIFM2	10.683086	44.296781	22.978654	48.515528	18.923118	41.201534	23.420293	35.949807
AIFM3	0	0.712184	0	1.637384	0	1.616798	0	0.633721
AIG1	10.023504	24.771567	16.492384	22.271954	18.821756	22.117518	8.357312	15.219543
AIG1P1	0	0	0	0	0	0	0	0
AIM2	0	0	0	0.062925	0	0.441517	0	0
AIMP1	37.650771	63.699271	48.121656	22.680003	27.959569	11.856415	28.468403	43.658854
AIMP1P1	0	0	0	0	0	0	0	0
AIMP1P2	0	0	0	0	0	0	0	0
AIMP2	74.57247	57.559814	79.833357	52.783427	81.709048	48.198	42.551794	55.341952
AIP	61.87857	39.19691	49.795544	61.171554	14.794004	34.589116	0	24.474872
AIPL1	0	0	0	0	0	0	0	0.33625

AIRE	0	0	0	0	0	0	0	0
AIRIM	28.655412	36.266403	15.577281	37.840354	47.568137	43.529586	20.551203	38.592982
AJAP1	0	0	0	0	0	0.142277	0	0
AJUBA	145.249193	192.93171	102.354379	177.371283	213.602903	248.342493	223.313598	347.859791
AK1	64.636431	43.022313	5.610095	50.970522	15.312776	74.117503	31.521068	70.162037
AK2	85.922495	26.573218	37.722805	65.217302	155.955133	130.743764	155.556751	66.404685
AK2P2	0	0	0	0	0	0	0	0
AK3	21.088949	45.33839	24.481016	49.352051	35.079178	36.690703	14.62186	34.695874
AK3P2	0	0	0	0	0	0	0	0
AK3P3	0	0.522413	0	1.239919	0	0.308127	0	1.356684
AK3P4	0	0	0	0	0	0	0	0
AK3P5	0	0	0	0	0	0	0	0
AK3P6	0	0	0	0	0	0	0	0
AK3P7	0	0	0	0	0	0	0	0
AK4	121.043376	212.552839	93.616067	183.391269	99.159321	178.168453	117.624435	288.494521
AK4P1	0	0	0	4.784869	0	1.056937	0	5.928478
AK4P2	0	0	0	0	0	0	0	0
AK4P4	0	0	0	0	0	0	0	0
AK4P5	0	0	0	0	0	0	0	0
AK4P6	0	0	0	0	0	0	0	0
AK5	1.017936	13.030549	0	4.074291	0	0	0	1.39681
AK6	54.650938	62.834494	13.967306	29.498938	66.040103	8.145141	31.646387	40.575881
AK6P1	0	0	0	0	0	0	0	0
AK6P2	0	0	0	0	0	0	0	0
AK7	1.016951	3.30165	6.381772	5.859107	0	0.701923	0	0.685507
AK8	0	0.887471	0	0	0	0	0	1.304055
AK9	4.121256	1.75637	1.184001	1.390317	4.23824	2.053036	0	8.229647
AKAIN1	0	0	0	0	0	0	0	0
AKAP1	21.000524	62.56179	22.017484	67.538982	44.197387	100.209684	74.618786	60.657056
AKAP10	15.437138	29.726077	28.689743	31.936755	60.760551	41.709677	10.084244	43.590159
AKAP11	19.433631	20.411072	22.738039	14.439027	20.375915	7.504215	6.876703	18.345197
AKAP12	33.023422	36.154935	99.162493	108.304539	21.120236	29.28411	12.161122	19.804649
AKAP13	50.932373	65.229789	45.546344	61.186951	60.606015	35.065206	95.814998	56.318539

AKAP14	0	0	0	0	0	0	0	0
AKAP17A	11.373611	21.691653	14.990844	24.569568	5.518485	47.026396	4.956254	36.318685
AKAP17BP	0	0	0	0	0	0	0	0
AKAP3	0	5.246769	0.90156	8.616434	0	3.373772	0	2.503898
AKAP4	0	0	0	0	0	0	0	0
AKAP5	0	0.396515	1.348789	0.352185	1.717679	0.551785	0	0.987125
AKAP6	11.399317	6.832301	3.470237	7.085229	9.610192	5.661008	0	5.465804
AKAP7	5.182967	5.868382	4.761554	1.34705	0.560946	0.900356	0	7.573062
AKAP8	24.08782	11.581735	17.055387	14.513025	35.479514	24.127787	44.692861	16.62777
AKAP8L	26.621075	27.013071	25.2642	39.063428	75.689278	49.189378	17.391129	27.829725
AKAP8P1	0	0	0	0	0	0	0	0
AKAP9	6.595042	4.222795	0	8.84927	0	3.894636	10.725307	8.704848
AKIP1	12.025678	17.435961	19.91171	26.326717	22.260352	41.664786	42.802839	26.725481
AKIRIN1	40.27391	32.469746	85.605681	22.084199	87.404369	23.723807	33.301908	46.982896
AKIRIN1P1	0	0	0	0	0	0	0	0
AKIRIN1P2	0	0	0	0	0	0	0	0
AKIRIN2	105.599999	132.450974	63.133329	120.840269	8.238231	94.232928	33.364187	144.113608
AKIRIN2P1	0	0	0	0	0	0	0	0
AKNA	4.102801	0.764196	4.083111	0.350067	4.395928	0.38755	5.470113	0.107885
AKNAD1	0	1.742317	0	0.591682	0	0	0	0.630648
AKR1A1	9.160069	62.057992	4.214758	61.722686	80.351242	67.691195	195.569246	87.678805
AKR1B1	235.864148	220.109873	454.103445	419.811999	93.25134	100.743159	30.301747	81.436698
AKR1B10	0	1.94014	0	1.542349	0	3.531535	27.393221	2.612114
AKR1B10P1	0	0	0	0	0	0	0	0
AKR1B10P2	0	0	0	0	0	0	0	0
AKR1B15	0	2.146506	0	2.061271	0	0	0	0
AKR1B1P1	0	0	0	0.097778	0	0	0	0
AKR1B1P2	0	0	0	0	0	0	0	0
AKR1B1P3	0	0	0	0	0	0	0	0
AKR1B1P4	0	0	0	0	0	0	0	0
AKR1B1P5	0	0	0	0	0	0	0	0
AKR1B1P6	0	0	0	0	0	0	0	0
AKR1B1P7	0	0	0	0	0	0	0	0

AKR1B1P8	0	0	0	0	0	0	0	0
AKR1C1	39.33136	59.454527	77.391437	87.045626	7.118577	5.631539	4.989236	7.004054
AKR1C2	68.36682	55.265015	111.944982	105.222915	0.63966	6.798512	0	10.168495
AKR1C3	33.606334	29.114117	64.586524	41.117768	36.801305	3.241869	0	3.087996
AKR1C4	0	0	0	0	0	0	0	0
AKR1C5P	0	0	0	0	0	0	0	0
AKR1C6P	0	0	0	0	0	0	0	0
AKR1C7P	0	0	0	0.423789	0	0	0	0
AKR1C8	0	0	0	0	0	0	0	0
AKR1D1	0	0	0	0	0	0	0	0
AKR1D1P1	0	0	0	0	0	0	0	0
AKR1E2	0	0	0	1.451619	0	0	0	0
AKR7A2	0	0	0	0	0	1.698525	73.187003	2.50513
AKR7A2P1	0	0	0	0	0	0	0	0
AKR7A2P2	0	0	0	0	0	0	0	0
AKR7A3	0	0	0	0.147435	0	0	0	0.248666
AKR7L	0	0	0	0.17033	0	0	0	0.212042
AKT1	0	39.55689	0	26.895916	0.486442	84.258938	0	42.262711
AKT1S1	1.888989	17.584611	1.692467	18.609854	8.593684	80.344241	0	61.584636
AKT2	54.470298	127.708362	37.527076	118.058864	93.637195	290.482314	94.639875	253.660792
AKT3	0	28.242047	9.583394	28.451509	5.396829	20.668008	9.389229	23.111988
AKTIP	21.791502	65.802318	51.400319	70.418494	31.970557	43.457783	1.017599	46.29517
AKTIPP1	0	0	0	0	0	0	0	0
AKTIPP2	0	0	0	0	0	0	0	0
AKTIPP3	0	0	0	0	0	0	0	0
ALAD	3.210366	6.770946	8.631534	8.328649	9.321472	9.484129	8.581767	6.115235
ALAS1	36.396879	47.188888	49.62173	56.608737	30.542219	36.289564	44.444577	33.390567
ALAS2	0	0	0	0	0	0	0	0
ALB	0	0	0	0	0	0	0	0
ALCAM	257.841438	577.313507	335.303596	841.257929	163.831658	380.842088	192.908162	460.510076
ALDH16A1	8.649071	6.689187	1.305261	5.08136	15.207338	15.030019	0.262713	8.689734
ALDH18A1	58.693453	68.923528	64.312636	91.732673	53.050171	88.381925	66.986321	62.540252
ALDH1A1	0	0	0	0.040046	0	0	0	0

ALDH1A2	0	7.558855	0	6.707108	0	7.343344	0	1.897415
ALDH1A3	573.707605	1076.60959	859.543972	2304.02749	215.042037	925.764644	264.912147	339.227616
ALDH1B1	28.811129	40.427212	18.865096	41.661182	23.398262	58.676051	33.922978	58.003483
ALDH1L1	0	3.503935	5.171629	1.094033	0.323084	3.414299	0	1.254869
ALDH1L2	18.342396	8.216209	9.086149	11.534726	0.115378	0.671506	0	3.403176
ALDH2	20.96629	28.700706	15.139906	29.564552	1.237829	16.931541	0	13.251024
ALDH3A1	28.378224	48.19139	61.244435	64.987239	21.995191	61.121895	56.251272	38.874717
ALDH3A2	63.984511	6.299023	70.792482	14.15597	166.692898	53.667801	18.19429	60.827235
ALDH3B1	3.00929	3.438252	0	5.516035	14.727494	6.515656	0	3.244891
ALDH3B2	0	0	0	0	18.466803	0.254288	0	0.142819
ALDH4A1	4.545954	2.140018	4.072618	1.91091	2.999501	6.021051	12.423522	4.27381
ALDH5A1	0	3.751417	2.636184	5.080481	8.292194	5.586491	75.705251	3.378329
ALDH6A1	21.540892	12.859607	8.497482	16.870051	28.749945	7.032926	24.07628	6.657212
ALDH7A1	180.584628	171.982061	76.726757	173.439779	114.011535	132.617389	112.093391	126.384677
ALDH7A1P1	0	0	0	0	0	0	0	0
ALDH7A1P2	0	0	0	0	0	0	0	0
ALDH7A1P3	0	0	0	0	0	0	0	0
ALDH7A1P4	0	0	0	0	0	0	0	0
ALDH8A1	2.173148	0	0	0	0	0	0	0
ALDH9A1	0	2.193726	0	0	0	7.935057	3.598465	0
ALDOA	1.82861	13.786536	3.625216	172.62081	82.772036	427.517388	242.161095	275.985705
ALDOAP1	0	0	0	0	0	0	0	0
ALDOAP2	0	0	0	0	0	0	0	0
ALDOB	0	0	0	0	0	0	0	0
ALDOC	8.124373	0.198168	0	1.314917	22.065476	24.640591	17.08332	16.47644
ALG1	5.068645	1.693298	0	2.563912	16.36428	1.77638	22.731432	2.442538
ALG10	7.505376	2.413485	15.887998	4.921034	9.960647	2.555986	15.311779	4.298556
ALG10B	7.987682	5.789837	2.127889	3.075047	7.109082	2.015617	1.637223	2.708002
ALG11	7.080036	10.770253	8.048717	10.330029	0	5.803248	10.528038	12.036205
ALG12	2.449821	5.830969	1.650963	7.340757	15.004525	12.436897	0.149219	11.742064
ALG13	42.644939	51.073382	33.77204	26.686507	8.502306	35.553423	0	49.005877
ALG14	9.639162	8.114293	19.533146	6.554521	2.084713	0.292924	0	0.576977
ALG1L10P	0	0	0	0	0	0	0	0

ALG1L11P	0	0	0	0	0	0	0	0
ALG1L12P	0	0	0	0	0	0	0	0
ALG1L13P	0	0	0	0	0	0	0	0
ALG1L14P	0	0	0	0	0	0	0	0
ALG1L15P	0	0	0	0	0	0	0	0
ALG1L2	0	1.621266	0	0.237974	0	0	0	0
ALG1L3P	0	0	0	0	0	0	0	0
ALG1L5P	0	0	0	0	0	0	0	0
ALG1L6P	0	0	0	0	0	0	0	0
ALG1L7P	0	0	0	0	0	0	0	0
ALG1L8P	0	0	0	0.131077	0	0	0	0
ALG1L9P	0	0	0	1.208006	0	0	0	0
ALG2	35.360194	37.82046	33.753506	35.452726	32.787493	17.55953	6.329392	24.000057
ALG3	66.265285	17.419462	0	17.943156	29.535138	15.207569	25.805486	16.151033
ALG3P1	0	0	0	0	0	0	0	0
ALG5	36.529998	31.942918	18.885964	20.97711	10.407947	7.390228	0	21.934026
ALG6	11.840478	13.624733	11.694309	5.711914	3.112128	3.729992	22.626201	10.371369
ALG8	47.127163	16.432629	7.273713	12.014928	36.992093	4.197223	88.416019	3.004334
ALG9	2.339022	10.960951	12.367807	12.495999	24.219146	6.121847	0	11.197031
ALK	0	0	0	0	0	0	0	0
ALKAL1	0	0.41009	0	0	0	0	0	0
ALKAL2	0	0	0	0.515068	0	0	0	0
ALKBH1	11.754135	11.341844	29.38395	14.148927	13.917589	8.209854	32.589723	10.305398
ALKBH2	10.69175	112.009489	38.095495	136.868561	36.510759	140.455783	25.622504	145.069605
ALKBH3	16.218413	114.083756	37.803938	96.982173	75.926825	215.82583	116.457974	217.579886
ALKBH4	6.606446	20.108722	0	18.096395	2.061331	21.139587	23.203072	21.428442
ALKBH5	50.847956	132.808727	44.947638	124.141771	74.062713	199.026836	91.574193	239.333633
ALKBH6	35.443678	9.487216	10.491114	14.716272	0	11.689072	0	12.411023
ALKBH7	12.024133	8.237349	7.066294	8.291567	11.57428	7.977759	0	9.81798
ALKBH8	0	59.042159	5.660682	57.491343	0	16.021386	35.97016	15.982764
ALLC	0	0	0	0	0	0	0	0
ALMS1	2.946941	16.087543	15.777394	13.507455	9.95444	7.569607	16.022663	13.965003
ALMS1P1	0	0.110856	0	0.16156	0	0	0	0

ALOX12	0	0	0	0.416676	0	1.091877	0	1.012683
ALOX12B	0	1.398188	2.692882	1.322279	0.356674	0.268684	0	1.17784
ALOX12P1	0	0	0	0	0	0	0	0
ALOX12P2	0	0	0	0	0	0	0	0
ALOX15	0	0	0	0.274091	0	0.069918	0	0.283595
ALOX15B	0	0.327404	0	0.103982	0	1.933618	0	2.043744
ALOX15P1	0	1.737558	0	0.734404	0	0	0	0
ALOX15P2	0	0	0	0	0	0	0	0
ALOX5	1.789449	0.075678	2.555363	1.818307	0.376827	6.255754	46.321673	4.127688
ALOX5AP	0	0	0	0	0	0	0	0
ALOXE3	0	0	0	0	0	0	0	0
ALOXE3P1	0	0	0	0	0	0	0	0
ALPG	0	0	0	0.066461	0	0.070135	0	0
ALPI	0	0	0	0	0	0	0	0
ALPK1	8.315663	17.837272	8.416594	14.913194	32.703533	6.180878	0	5.745231
ALPK2	0	0.196428	0	0.395711	1.245506	0.076513	0	0.124411
ALPK3	0	0	0	0	0	5.544787	0	0
ALPL	5.736394	0.137206	0	0.782733	3.636363	0.7909	0	0.090337
ALPP	0	0	0	0.089602	17.404715	8.688441	8.634209	2.220606
ALS2	12.30707	30.036414	5.239339	25.221541	48.902701	27.778096	3.383277	33.05005
ALS2CL	4.134777	3.241371	2.074133	3.700251	4.673417	9.182921	8.603308	5.070214
ALX1	0	0.458595	0	0.66269	0	0	0	0
ALX3	0	0.421922	0	0.245004	0	0.494826	0	0
ALX4	0	0.04887	0.511037	0.13902	0	0.085494	0	0.068413
ALYREF	0	25.734571	0	3.861718	0	6.178769	0	1.937074
AMACR	6.913659	10.528138	3.013225	6.035996	15.200019	4.41906	0	4.963688
AMBN	0	0	0	0	0	0	0	0
AMBP	0	0	0	0	0	0	0	0
AMBRA1	9.292786	16.620105	7.943204	22.255302	10.040396	31.586541	56.81576	30.279489
AMD1	49.148597	26.077954	52.869012	10.701055	45.852598	15.559407	126.428201	53.881903
AMD1P1	0	0	0	0	0	0	0	0
AMD1P2	0	0	0	0	0	0	0	0
AMD1P3	0	2.154358	3.430787	1.212851	2.042601	0	0	1.269525

AMD1P4	0	0	0	0	0	0	0	0
AMDHD1	0	0.08031	0	0.397109	0	3.768588	0	0.248821
AMDHD2	18.926517	11.970988	0	1.657633	0	2.046252	0	2.227471
AMELX	0	0	0	0	0	0	0	0
AMELY	0	0	0	0	0	0	0	0
AMER1	6.515916	10.94749	7.586788	11.923802	9.421047	17.306077	4.11426	11.960875
AMER2	0	0	0	0	0	0	0	0
AMFR	81.021956	136.016988	88.289693	93.359292	76.645017	142.183161	175.391132	107.991455
AMH	0	0	0	0.101686	0	0	0	0
AMHR2	0	0	0	0	0	0	0	0
AMIGO1	1.274438	4.091941	1.717476	5.742097	7.386456	4.088119	3.57031	2.607694
AMIGO2	1.818947	136.516042	49.14092	115.024565	22.550023	131.825198	1.282442	231.284684
AMMECR1	21.963034	6.685379	13.704954	3.528057	24.337387	6.138099	18.026709	9.876646
AMMECR1L	20.506625	0	10.795757	0	0	0	15.152943	0
AMMECR1LP	0	0	0	0	0	0	0	0
AMN	0	0.580515	0	0.370224	2.24938	0.474065	0	0.060913
AMN1	3.564731	10.414449	4.507606	4.166781	1.838566	4.782537	0	5.697093
AMOT	0	0.222528	0	0	0	0.094356	0	0
AMOTL1	125.783649	233.573546	217.845539	333.683293	107.144083	237.542337	60.734732	179.40004
AMOTL2	72.391795	68.369789	72.255064	54.894694	18.819689	69.080367	8.858788	46.303398
AMPD1	0	0	0	0.121605	0	0	0	0
AMPD2	55.811216	19.66528	38.767527	24.171737	19.812562	16.215509	14.543366	13.015006
AMPD3	16.182698	8.32602	32.19261	14.383398	40.913452	35.726226	66.353095	22.163422
AMPH	0	0	0	0.121833	0	0.401437	0	0
AMT	0	0	0	0	0.271964	0	0	0
AMTN	27.291924	14.476839	29.332143	25.613929	4.011485	3.139495	0	7.517255
AMY1A	0	0	0	0	0	0	0	0
AMY1B	0	0	0	0	0	0	0	0
AMY1C	0	0	0	0	0	0	0	0
AMY2A	0	0	0	0	0	0	0	0
AMY2B	0	0	0.624578	0	0	0	0	0
AMYP1	0	0	0	0	0	0	0	0
AMZ1	0	7.18294	3.18714	5.172371	0	1.125487	7.783546	1.019503

AMZ2	89.37127	163.721194	80.400016	138.406132	131.573893	64.155805	96.94764	124.05281
AMZ2P1	0	2.66633	0	2.590893	0	0.584809	31.531939	2.269857
AMZ2P2	0	0	0	0	0	0	0	0
AMZ2P3	0	0	0	0	0	0	0	0
ANAPC1	39.348164	121.798514	16.265483	120.354811	22.613715	148.418583	31.549553	196.678048
ANAPC10	6.260097	14.090414	20.229884	12.300983	22.760849	4.024645	0	15.572216
ANAPC10P1	0	0	0	0.199587	0	0	0	0
ANAPC11	105.566423	214.344767	108.922895	231.015186	147.49043	249.267191	188.40298	184.888981
ANAPC13	24.186881	98.985935	41.995418	90.73346	6.390117	127.037468	0	96.324834
ANAPC13P1	0	0	0	0	0	0	0	0
ANAPC15	31.059304	125.161213	40.517449	112.148432	59.056159	158.823821	2.539838	87.732297
ANAPC15P1	0	0	0	0	0	0	0	0
ANAPC15P2	0	0	0	0	0	0	0	0
ANAPC16	161.870555	126.849548	72.949257	65.039214	70.524071	50.291176	33.027685	81.987526
ANAPC1P1	0	0	0	0	0	0	0	0
ANAPC1P2	7.697819	20.662671	14.934734	32.408608	0	22.343237	0	28.698606
ANAPC1P3	0	0	0	0	0	0	0	0
ANAPC1P4	4.090467	2.563556	0	1.277783	0	0.277519	0	0.96921
ANAPC1P5	0	0	0	0	0	0	0	0
ANAPC1P6	0	0	0	0	0	0	0	0
ANAPC2	6.376825	6.743303	3.425013	6.96043	31.170144	9.590666	154.693374	7.153641
ANAPC4	5.219735	0	8.17561	5.827108	2.4101	5.61804	0	12.986147
ANAPC5	109.381697	157.287928	37.742466	90.772951	79.073875	99.936303	65.036692	204.298303
ANAPC7	6.834997	29.746385	3.160605	18.128224	30.969704	53.951791	0	38.830129
ANG	0	5.110941	0	10.079923	0	5.411303	0	5.261816
ANGEL1	0	0	0	6.471379	0	14.201011	0	1.161322
ANGEL2	7.363325	31.581374	30.642167	24.102896	4.13906	12.212858	22.814276	32.210832
ANGPT1	0	0.333819	0	0.056947	0	0	0	0
ANGPT2	0	0	0	0	0	0	0	0
ANGPT4	0	0	0	0	0	0	0	0
ANGPTL1	0	0	0	0	0	0	0	0
ANGPTL2	5.27212	13.583313	3.538734	14.524748	8.935691	12.111364	10.555109	22.530475
ANGPTL3	0	0	0	0	0	0	0	0

ANGPTL4	11.714324	6.221012	5.538662	8.668486	0	0	0	0.946507
ANGPTL5	0	0	0	0.177361	0	0	0	0
ANGPTL6	0	0	0	0.334975	0	0.55555	0	0.18277
ANGPTL7	0	0	0	0	0	0	0	0
ANGPTL8	0	0	0	0	0	0	0	0
ANK1	0.39138	2.006197	2.553938	1.293686	1.711653	3.113975	4.212261	2.265166
ANK2	1.879846	1.259677	0.606876	2.752227	0	0.103156	0	0.971125
ANK3	3.708514	18.015329	27.643113	8.504058	7.120784	5.690119	78.916257	3.383609
ANKAR	0	2.046879	12.524282	2.082147	0	2.432487	0	2.134912
ANKDD1A	0	4.19922	0	0.453085	0	0.339222	0	0.868606
ANKDD1B	0	0	0	0	0	0	0	0
ANKEF1	14.621567	12.491827	9.818905	12.681254	9.433861	12.249164	13.430994	19.084236
ANKFN1	1.405608	0.811845	0	1.545228	0	0.488568	0	0.242377
ANKFY1	41.524459	21.510737	11.615495	22.655163	22.7513	43.81253	21.731592	35.709123
ANKH	11.058031	49.364754	5.654895	39.810653	47.071011	64.618872	34.721586	46.705044
ANKHD1	47.414886	37.466734	54.244821	49.67037	84.124396	38.138734	121.539447	48.249614
ANKHD1-EIF	7.401597	8.758957	16.798516	10.044945	21.042048	6.132629	0	5.06456
ANKIB1	55.513687	134.701722	85.502951	103.102561	52.804047	58.233017	14.353095	149.68418
ANKK1	0	0	0	0	0	0	0	0.407144
ANKLE1	2.175701	0	0	1.011504	0	0	0	0
ANKLE2	26.466445	94.101708	43.010919	78.154658	27.793	91.386198	36.987399	56.647078
ANKMY1	0	1.913602	4.310936	3.98787	14.642005	6.602387	5.497929	2.525054
ANKMY2	13.671972	32.002223	12.232397	18.006478	20.198433	18.984896	22.223346	29.620776
ANKRA2	24.248731	47.590218	8.545878	46.293079	8.193512	22.431682	0	30.100017
ANKRD10	24.302335	27.602989	37.033032	25.809178	23.88083	41.655066	32.256719	41.047741
ANKRD11	80.536662	225.182009	111.035022	273.185498	74.950782	227.471482	175.910659	178.722416
ANKRD11P1	0	0	0	0	0	0	0	0
ANKRD11P2	0	0	0	0	0	0	0	0
ANKRD12	32.572944	16.236299	22.580987	17.128271	73.327099	21.299154	9.041382	25.948212
ANKRD13A	0	42.996672	14.273186	54.566687	9.945022	100.591078	51.433421	80.110459
ANKRD13B	9.990645	17.138219	8.951735	22.405508	41.238198	29.483882	33.365431	19.337911
ANKRD13C	7.214555	14.237277	8.796763	12.526581	1.588257	9.903854	2.104616	23.790078
ANKRD13C-I	0	2.185658	0	2.004197	0	0.978166	0	5.102016

ANKRD13D	0	5.962902	4.288623	4.164964	0.634789	6.381218	43.643365	2.019264
ANKRD16	0	1.36079	0	2.191331	19.802052	4.590721	6.247058	3.731623
ANKRD17	36.321444	28.948781	37.041631	28.800722	152.817642	31.596209	37.272099	34.70478
ANKRD18A	5.575942	5.713823	7.829173	4.53084	3.542385	3.772761	38.850647	6.102808
ANKRD18B	1.756248	7.068055	3.015798	3.774833	5.018578	7.676856	65.565588	18.405132
ANKRD18CP	0	0	0	0.054939	0	0	0	0.317363
ANKRD18DP	0	0	0	0.213619	0	0	0	0
ANKRD18EP	0	0	0	0	0	0	0	0
ANKRD18FP	0	0	0	0	0	0	0	0
ANKRD19P	0	0	4.080793	0.294658	0	0	0	0
ANKRD2	0	3.027279	0	2.288712	0	9.446646	12.610633	4.641138
ANKRD20A1	0	0	0	1.24939	0	2.21169	0	2.095796
ANKRD20A1(	0	0	0	0	0	0	0	0
ANKRD20A1:	0	0.971286	2.400809	0.220403	0.471081	0.712766	0	0.796825
ANKRD20A17	0	0	0	0	0	0	0	0
ANKRD20A18	0	0	0	0	0	0	0	0
ANKRD20A19	0	0	0	0	0	0	0	0
ANKRD20A2:	0	0	0	0	0	0	0	0
ANKRD20A5I	0	0	0	0	0	0	0	0.038605
ANKRD20A6I	0	0	0	0	0	0	0	0
ANKRD20A7I	0	0	0	0	0	0	0	0
ANKRD20A8I	0	0	0	0	0	0	0	0.036158
ANKRD20A9I	0	0.14435	0	0	0	0	0	0
ANKRD22	0.85781	0.403957	19.783181	0.670021	4.75684	0.942805	0	1.653835
ANKRD23	0	0.650795	0	2.603358	0	1.356822	0	0.544045
ANKRD24	0	0	0	1.088941	0.355306	1.16637	0	0.759219
ANKRD26	0	0	0	0	0	0	0	0
ANKRD26P1	0	0	0	0	0	0	0	0
ANKRD26P2	0	0	0	0.050053	0	0	0	0
ANKRD26P4	0	0	0	0	0	0	0	0
ANKRD27	52.452529	136.449945	52.950977	136.957176	41.635777	197.190198	101.065074	210.434803
ANKRD28	31.672778	75.114219	38.123552	62.220485	80.132144	25.727239	49.728616	78.233514
ANKRD29	10.500245	24.038696	8.707526	13.010154	7.48422	6.508243	0	5.058719

ANKRD30A	0	0	0	0	0	0	0	0
ANKRD30B	0	0	0	0	0	0	0	0
ANKRD30BL	0	0	0	0	0	0	0	0
ANKRD30BP1	0	0	0	0	0	0	0	0
ANKRD30BP2	0	0	0	0	0	0	0	0
ANKRD30BP3	0	0	0	0	0	0	0	0
ANKRD31	0	0.277847	0	0.289746	0	0.027002	0	0.021597
ANKRD33	0	0	0	0	0	0	0	0
ANKRD33B	8.715908	18.916039	14.179626	22.064612	11.715456	32.799283	8.098507	24.212172
ANKRD33BP1	0	1.361043	0	0.285623	0	0.187376	0	0
ANKRD33BP1	0	0	0	0	0	0	0	0
ANKRD33BP2	0	0	0	0	0	0	0	0
ANKRD33BP5	0	0	0	0	0	0	0	0
ANKRD33BP6	0	0	0	0	0	0	0	0
ANKRD33BP7	0	0	0	0	0	0	0	0
ANKRD33BP8	0	0	0	0	0	0	0	0
ANKRD33BP9	0	0	0	0	0	0	0	0
ANKRD34A	1.841408	2.126693	0	1.752281	0	1.838584	0	2.343102
ANKRD34B	0.888771	8.621537	0	7.455424	0.234678	1.490554	0	1.713427
ANKRD35	0	0.508661	0	0.739456	0	1.025758	0	1.407099
ANKRD36	10.678604	3.094141	4.607063	1.204552	7.940361	1.532678	5.787842	1.83764
ANKRD36B	6.406946	2.738435	6.984874	7.499462	0	4.106156	0	8.757646
ANKRD36BP1	0	0	0	0	0	0	0	0
ANKRD36BP2	0	0	0	0	0	0	0	0
ANKRD36C	3.419645	10.254666	7.409891	7.847611	20.489073	3.785966	0	6.680083
ANKRD36P1	0	0	0	0	0	0	0	0
ANKRD36P2	0	0	0	0	0	0	0	0
ANKRD37	0	6.384363	8.027623	4.944992	17.871977	3.75	0	7.063177
ANKRD39	0	6.586318	3.912312	6.837335	22.173979	13.70691	0	8.445015
ANKRD39P1	0	0	0	0	0	0	0	0
ANKRD40	34.033087	43.021779	34.811972	39.49285	75.484229	68.775583	31.400167	55.687532
ANKRD40CL	0	0	0	0	0	0	0	0
ANKRD42	39.002781	101.180107	16.367407	110.642472	18.093004	48.448197	30.032045	47.360068

ANKRD44	0.448075	0	0	1.374081	0	1.376882	0	2.604647
ANKRD45	0	0	0	0	0	0	0	0
ANKRD46	3.835406	7.493462	4.577717	5.1255	3.4479	6.884736	0	9.368823
ANKRD49	64.95684	38.421829	35.696248	24.313056	12.680438	8.255448	52.182838	20.159848
ANKRD49P1	0	0	0	0.140875	0	0	0	0
ANKRD49P2	0	0	0	0	0	0	0	0
ANKRD49P3	0	0	0	0	0	0	0	0
ANKRD49P4	0	0	0	0	0	0	0	0
ANKRD50	10.976761	28.869702	12.319548	19.570911	12.94667	11.41647	9.18102	29.484189
ANKRD52	18.546499	14.767793	18.690427	13.909995	18.529058	16.247291	72.804496	12.812322
ANKRD53	0	0.966542	0	0.236922	0	0.862031	0	0.257012
ANKRD54	9.903136	52.282614	11.750143	52.343962	30.34256	25.540086	19.619166	24.341904
ANKRD54P1	0	0	0	0	0	0	0	0
ANKRD55	0	0	0	0.114314	0	0	0	0
ANKRD6	5.143497	12.984382	1.53096	10.212582	2.90434	6.683945	0	6.134513
ANKRD62	0	0	0	0	0	0	0	0
ANKRD62P1	0	0	0	0	0	0	0	0
ANKRD65	24.913237	38.62155	15.505028	38.823341	49.298765	60.212125	21.022352	42.887322
ANKRD66	0	0	0	0	0	0	0	0
ANKRD7	0	7.266047	4.912568	1.459331	0	0.266774	0	2.238685
ANKRD9	0.483323	6.122464	3.911477	5.969252	3.831488	12.10236	1.885489	9.995927
ANKS1A	13.289834	14.562223	11.029348	16.15644	11.398152	18.502366	25.631339	13.690341
ANKS1B	8.047882	5.426353	0	3.88801	0	0.161663	0	0
ANKS3	13.219058	12.438688	8.086988	12.39936	1.592238	19.456547	11.05626	9.928904
ANKS4B	0	0	0	0	0	0	0	0
ANKS6	11.071	56.671226	20.01804	50.154851	2.811188	49.249589	3.07178	28.26829
ANKUB1	0	0.469828	0	0.402015	0.450797	0.549815	0	0.502001
ANKZF1	7.994155	6.974213	12.857478	23.169099	37.491984	13.810026	61.889027	13.931613
ANLN	211.533688	153.996241	107.011465	118.191835	280.672731	225.955495	401.979207	392.826521
ANO1	0	4.882729	0	21.412786	55.286728	19.683779	21.385531	10.340326
ANO10	35.651562	11.024395	9.397908	8.166464	10.04269	6.120567	0	12.680817
ANO2	0	0	0	0.702301	0	3.674103	42.236589	3.208164
ANO3	0	0	0	0	0	0	0	0

ANO4	0	0	0	0	0	0	0	0
ANO5	0	0	0	0	0	0	0	0
ANO6	37.274517	39.133278	30.927379	33.804954	30.740675	24.314665	59.247626	37.380787
ANO7	0	0.930051	0	1.453045	0.482638	4.234769	0	1.458898
ANO7L1	0	0	0	0	0	0.30343	0	0
ANO8	2.367268	2.333025	3.541618	4.068901	0.416792	5.542872	9.411083	3.548547
ANO9	2.567351	4.063531	4.208301	3.318736	0.677409	2.843038	0	4.084259
ANOS1	10.720356	6.341212	8.387288	6.227455	4.793176	6.660476	0.126344	6.458517
ANOS2P	0	0	0	0	0	0.318835	0	0
ANP32A	300.871264	435.076865	264.254929	386.623821	346.979134	527.170552	314.628141	557.417733
ANP32AP1	0	0	0	0	0	0	0	0
ANP32B	363.852435	498.800023	416.365273	505.770165	317.423718	454.107464	503.105649	397.355098
ANP32BP1	0	0.22996	0	0	0	0	0	0
ANP32BP2	0	0	0	0	0	0	0	0
ANP32BP3	0	0	0	0.857751	0	0	0	0
ANP32CP	0	0	0	0	0	0	0	0
ANP32E	118.218933	161.788968	47.389979	127.534679	159.651146	164.662697	182.813339	114.362228
ANPEP	0	0	0	0	0	0.045245	0	0
ANTKMT	23.126999	21.799793	0	22.203629	14.937497	16.000461	37.202005	25.805637
ANTXR1	0	17.372336	4.10181	23.225802	18.208004	10.152801	0.532577	24.53473
ANTXR2	17.334663	14.319485	3.099173	11.29214	5.348589	5.850537	11.78236	7.638897
ANTXRL	0	0	0	0	0	0	0	0
ANTXRLP1	0	0	0	0	0	0	0	0
ANXA1	393.538379	320.140641	404.89469	243.092285	420.255688	204.123691	421.716441	261.523689
ANXA10	0	0	0	0	0	0	0	0
ANXA11	89.568997	278.440258	220.631126	324.667253	131.301935	338.814772	166.858033	250.648601
ANXA13	0	0	0	0.059903	0	0.243119	0	0
ANXA2	545.95961	737.939004	158.85202	611.588594	972.16392	951.356283	1156.61263	700.928325
ANXA2P1	0	0	0	0.203354	0	0	0	0
ANXA2P2	0	0	0	0.33125	1.127865	2.616201	0	0.371398
ANXA2P3	0	0	0	0	0	0	0	0
ANXA2R	0	0.387782	0	0.141362	1.621663	0.128684	0	0
ANXA3	65.64421	96.827358	38.151604	67.441711	105.547817	117.593149	125.196716	143.22748

ANXA4	72.380661	129.350359	58.031955	115.586835	81.935983	149.013852	72.415718	144.74133
ANXA5	257.893338	311.657758	171.790531	208.276043	176.974682	149.300021	155.454835	296.734096
ANXA6	27.572344	25.189421	32.880176	23.012109	35.607283	4.670675	0	7.744188
ANXA7	103.005977	102.034121	87.356308	94.118086	98.022182	73.011625	99.148798	90.631249
ANXA8	38.855654	149.487147	52.882551	142.39614	65.379697	161.374932	35.531645	148.48224
ANXA8L1	75.924612	186.109024	106.427965	210.110969	167.473867	272.811019	85.472828	249.435649
ANXA9	4.610111	2.327394	0	2.411875	0	6.416294	0	8.496333
AOAH	0	8.947915	3.928412	12.421333	0	0.39545	0	1.116586
AOC1	0	0	0	0	0	0.068978	0	0.226236
AOC2	0	1.453394	3.543146	4.248064	0	2.361292	0	0.903255
AOC3	1.696232	1.635064	7.315232	0.601889	0	0.16463	0	0.132287
AOC4P	0	0	0	0.067216	0	0	0	0
AOPEP	17.567058	27.717407	6.299326	15.11828	26.801887	148.79409	93.242452	111.095396
AOX1	23.535477	4.219253	1.909741	1.727032	11.080435	7.05147	0	10.649043
AOX2P	0	0	0	0	0	0	0	0
AOX3P-AOX:	0	0	0	0	0	0	0	0
AP1AR	0	0	57.619678	16.38562	36.432544	11.488858	74.438987	0
AP1B1	19.88655	41.071378	35.787313	53.450493	62.175481	119.872439	60.089305	100.93055
AP1B1P1	0	0	0	0	0	0	0	0
AP1B1P2	0	0	0	0	0	0	0	0
AP1G1	3.624984	44.069142	17.386579	47.194073	41.538725	79.451263	0	69.495536
AP1G2	2.202509	11.24941	24.436219	11.321985	66.728211	17.662691	89.640945	7.54536
AP1M1	39.631751	44.398237	58.912756	44.319043	58.752174	90.55019	97.142354	49.255242
AP1M2	17.252879	15.278004	33.89604	14.524309	262.214472	6.789164	140.299915	23.588409
AP1M2P1	0	0	0	0	0	0	0	0
AP1S1	80.590385	53.365911	32.162217	44.542867	60.44014	65.199412	25.438283	48.799703
AP1S2	3.069968	1.071099	0	0.655715	1.214476	0.960567	0	0.781862
AP1S2P1	0	0	0	0	0	0	0	0
AP1S3	18.888627	36.474765	12.646665	18.760195	21.890268	35.381705	20.011014	64.388518
AP2A1	0	0.137166	0	0.326459	40.976593	0.496861	0	2.260625
AP2A2	22.68579	44.684506	49.578876	25.510471	193.205169	59.450317	177.549198	36.841598
AP2B1	148.794637	167.339182	166.128871	148.814468	176.503117	127.846738	82.402017	150.510627
AP2B1P1	0	0	0	0	0	0	0	0

AP2M1	218.714045	405.113689	157.687094	368.695231	183.206786	432.646273	199.861876	397.011842
AP2S1	21.982127	114.153994	64.137532	57.092868	0	81.28314	0	106.848006
AP3B1	0	0	0	0	0	0	26.298691	0
AP3B2	2.38772	5.579824	20.186745	3.36313	3.709171	4.102521	0	6.480396
AP3D1	12.38557	14.59447	0	28.230307	19.404766	39.678731	57.593519	32.294851
AP3M1	50.625766	28.105735	39.165502	31.062578	33.984424	16.645396	0	43.522171
AP3M2	5.712793	41.257585	23.644894	22.465937	33.946384	7.343547	17.332802	12.556867
AP3S1	31.369389	38.838072	66.546993	22.822067	31.121418	24.091309	6.078169	51.846022
AP3S1P1	0	0	0	0	0	0	0	0
AP3S2	28.796321	46.086554	102.306398	47.152422	35.662795	31.687341	92.780672	41.373326
AP4B1	1.759676	6.29879	0	5.08302	0	7.213872	0	5.646441
AP4E1	8.769734	13.244987	8.323751	4.147054	7.7456	6.48478	22.460988	19.629114
AP4M1	16.63231	5.943751	0	0.40169	0	0.976384	50.544613	6.70352
AP4S1	0	0	0	0	0	0	0	0
AP5M1	23.987772	40.045448	27.63541	31.18176	28.201554	25.255215	26.189277	82.843037
AP5S1	3.175885	11.202391	10.678989	7.606206	6.730719	18.351742	0	21.845693
AP5Z1	15.164049	5.11913	37.042085	7.649724	24.028505	6.908984	4.968767	4.726785
APAF1	39.295329	30.870987	16.594545	19.378155	33.87216	31.8271	23.783927	42.220486
APBA1	0	0.137347	0	0.288517	4.23302	0.14792	0	0
APBA2	8.305746	26.10088	6.003541	42.477092	5.555145	31.064927	19.678338	23.046533
APBA3	3.951278	11.202725	5.079234	7.398815	1.668733	17.851723	0	9.344044
APBB1	8.740802	21.830859	31.263675	26.999151	6.488106	17.892679	0	11.162601
APBB1IP	0	0	0	0	0	0	0	0
APBB2	13.311512	55.69739	15.45898	92.135327	18.270448	58.527946	12.980818	41.437232
APBB3	2.479103	1.24652	3.469327	3.391602	28.138051	0.79972	0	4.550123
APC	13.893983	13.203893	13.416205	9.126791	22.810032	6.831354	10.475138	12.129402
APC2	0	0.127647	0	0.24873	0	0.424822	0	0.908802
APCDD1	0	0	0	0	0	0.659987	0	0.452116
APCDD1L	5.096679	0.784483	0	0.776664	27.551711	4.818396	0	3.409852
APCS	0	0	0	0	0	0	0	0
APEH	87.527752	75.384974	71.417485	72.480599	148.049175	76.710611	64.213263	55.634995
APELA	0	0	0	0	0	0	0	0
APEX1	189.106136	206.729071	192.55376	265.550743	201.994637	296.605832	241.888871	263.784662

APEX2	23.726703	78.901623	26.817279	71.801544	22.330148	69.171905	55.735205	55.583047
APH1A	123.375716	144.203366	134.580284	115.111237	74.047461	137.408517	83.328828	133.603479
APH1B	1.594143	4.995923	5.008193	3.403849	1.894508	0.199237	36.526499	4.55031
API5	92.66323	139.753297	115.848098	134.241928	110.444577	198.594012	270.911567	236.023735
API5P1	0	0	0	0	0	0	0	0
API5P2	0	0	0	0	0	0	0	0
APIP	11.867207	43.016269	29.185972	23.721251	26.559322	48.998977	46.609547	80.657436
APLF	0	0.716086	1.053635	0.486186	0	0.681964	9.428088	0.626179
APLN	1.036685	1.239748	0.929269	1.415574	7.662475	7.443067	0	4.417856
APLNR	0	0	0	0	0	0	0	0
APLP1	0	2.798387	7.533062	0.670493	25.133656	8.007618	0	1.68855
APLP2	172.155723	233.35713	158.714668	299.474658	271.207867	417.324728	118.652298	295.604126
APMAP	56.217641	56.369189	69.781829	82.789554	68.774735	53.614252	35.543466	45.30724
APOA1	0	0	0	0	0	0	0	0
APOA2	0	0	0	0	0	0	0	0
APOA4	0	0	0	0	0	0	0	0
APOA5	0	0	0	0	0	0	0	0
APOB	0	0	0	0	0	0	0	0
APOBEC1	0	0	0	0	0	0	0	0
APOBEC3A	0	0	0	0	0	0	0	0
APOBEC3AP1	0	0	0	0	0	0	0	0
APOBEC3B	17.929085	14.910473	19.952761	19.052429	0.754935	6.329803	24.347564	4.292634
APOBEC3C	30.745604	27.003754	25.228237	30.849771	19.605028	20.192677	23.320984	23.809929
APOBEC3D	0	0	0	0.192036	0	0	0	0.223881
APOBEC3F	11.368633	7.257142	5.758137	11.567873	6.563684	10.746079	0	7.171847
APOBEC3G	11.416738	12.344039	2.492659	16.465223	15.635381	12.933707	2.204471	8.625726
APOBEC3H	11.122819	0	0	0.843654	0	0	0	0
APOBEC4	0	0	0	0	0	0	0	0
APOBR	0	0	0	0	0	0	0	0
APOC1	25.789597	29.347044	21.713469	37.996947	0	5.842539	0	2.136139
APOC1P1	0	0	0	0	0	0	0	0
APOC2	0	0	0	0	0	0	0	0
APOC3	0	0	0	0	0	0	0	0

APOC4	0	0	0	0	0	0	0	0
APOC4-APO	0	0	0	0	0	0	0	0
APOD	0	0	0	0.443855	0	0.596601	0	0
APOE	16.041986	34.864506	50.155648	61.286856	32.783014	17.847517	0	11.217299
APOF	0	0	0	0	0	0	0	0
APOH	0	0	0	0	0	0	0	0
APOL1	0	6.105567	14.84438	44.64626	57.764404	10.917789	39.663939	20.212674
APOL2	41.79075	106.042942	44.265728	175.801255	46.552	153.542003	63.330212	143.597463
APOL3	0	0.737853	14.213729	9.741041	14.11856	39.576821	0	16.0037
APOL4	0	0.749548	0	0.025974	0	0.284431	1.035671	0.085679
APOL5	0	0	0	0	0	0	0	0
APOL6	14.672948	13.918747	13.49352	15.162104	9.274567	11.703381	15.101903	11.575695
APOLD1	0	0	0	2.379546	0	0.471065	0	0.504319
APOM	0	5.771984	5.228716	6.824159	0	3.68439	0	4.651101
APONP	0	0	0	0	0	0	0	0
APOO	0	29.371828	47.940083	13.169395	65.045179	13.419978	0	42.886386
APOOL	14.016631	3.007738	13.052752	4.408127	0	2.084277	0	3.632685
APOOP1	0	0	0	0	0	0	0	0
APOOP2	0	0	0	0	0	0	0	0
APOOP3	0	0	0	0	0	0	0	0
APOOP4	0	0	0	0	0	0	0	0
APOOP5	0	0	0	0	0	0	0	0
APP	254.477872	366.682419	415.920813	714.462539	426.707457	945.198999	301.859449	382.710102
APPBP2	28.160579	36.167429	13.477234	23.795351	21.496431	6.228802	27.433708	23.915403
APPL1	24.790598	13.923717	27.525334	9.322323	13.875255	7.211016	81.621042	15.453834
APPL2	39.685618	25.502362	57.08738	22.440745	90.473646	28.032825	48.764464	36.632406
APRT	164.676797	161.587036	112.223916	155.124347	81.763822	150.269122	106.370904	95.382616
APTX	14.47668	2.615359	0	8.04932	50.222036	2.486767	0	10.668556
AQP1	0	0	0	0	0	0	0	0.349184
AQP10	0	0.408031	0	0	0	0	0	0.061119
AQP11	3.094982	0	0	0	0.813899	0	0	0
AQP12A	0	0	0	0	0	0	0	0
AQP12B	0	0	0	0	0	0	0	0

AQP2	0	0	0	0	0	0	0	0
AQP3	8.823971	3.161846	11.902843	4.891418	27.131458	27.210975	21.065346	13.003769
AQP4	0	0	0	0	0	0	0	0
AQP5	0	0	0	0	0	0	0	0
AQP6	0	0	0	0	0	0.031919	0	0
AQP7	0	0	0	0.528672	0	0.172831	0	0
AQP7P1	0	0	0	0	0	0	0	0
AQP7P2	0	0	0	0	0	0	0	0
AQP7P3	0	0	0	0	0	0	0	0
AQP7P4	0	0	0	0	0	0	0	0
AQP7P5	0	0	0	0	0	0	0	0
AQP8	0	0	0	0	0	0.281918	0	0
AQP9	0	0	0	0	0	0	0	0
AQR	28.445188	17.691611	24.079768	13.611801	32.970583	10.856639	16.249064	19.086109
AR	1.803961	4.274044	2.165106	4.944761	0.158951	0.090686	0	0.132461
ARAF	28.012937	36.720096	52.955589	40.359373	81.387872	45.497898	34.136764	32.032362
ARAFP1	0	0	0	0	0	0	0	0
ARAFP2	0	0	0	0	0	0	0	0
ARAFP3	0	0	0	0	0	0	0	0
ARAP1	43.010863	71.944519	47.537029	116.909358	39.079341	77.292457	72.122906	60.756459
ARAP2	59.191699	15.91484	6.962657	5.286736	5.81486	3.950785	0.260846	8.910966
ARAP3	5.315916	16.129062	7.255326	15.244237	6.833777	10.55201	4.017693	6.429022
ARB2A	39.219671	10.6724	12.627771	9.255495	1.479964	3.019834	11.357862	8.658225
ARB2BP	0	9.075471	6.155655	4.101374	0	2.53461	0	4.252147
ARC	0	1.19809	0	0.444245	0	0	0	0.09166
ARCN1	1165.29939	1545.45208	1154.91593	1654.17269	147.960938	163.684757	115.744132	217.008313
AREG	195.045181	539.80149	343.174624	743.004675	166.105956	303.78306	180.246317	403.135445
AREL1	30.715961	64.104731	16.23612	56.045963	79.00169	64.643049	26.193783	87.614316
ARF1	254.986241	295.335551	280.065706	380.850712	344.70575	311.933171	244.338719	266.150259
ARF1P1	0	0	0	0	0	0	0	0
ARF1P2	0	0	0	0	0	0.815211	0	0
ARF1P3	0	0	0	0	0	0	0	0
ARF3	184.69007	236.023462	111.164783	243.661245	76.239547	213.307474	132.437101	153.856144

ARF4	198.613741	196.456819	208.364672	214.951187	121.476306	137.474944	174.567062	141.189317
ARF4P1	0	0	0	0	0	0	0	0
ARF4P2	0	0	0	0	0	0	0	0
ARF4P3	0	0	0	0	0	0	0	0
ARF4P4	0	0	0	0	0	0	0	0
ARF5	116.593617	107.458055	60.646457	133.115021	87.931948	139.944305	65.486226	142.508488
ARF6	74.285812	219.406274	68.190246	168.729249	104.620237	251.829104	89.541126	309.34141
ARFGAP1	78.114865	62.736372	42.220342	81.431001	89.049293	97.549044	35.953685	60.980825
ARFGAP2	32.491485	17.118835	75.128929	21.106447	21.889718	49.371416	90.802526	38.756597
ARFGAP3	18.862003	15.660093	20.240355	15.302538	12.421596	7.789049	41.02098	8.910711
ARFGEF1	51.305064	63.098045	38.44326	65.308047	33.514802	35.419441	70.615274	33.904505
ARFGEF2	29.569258	27.62534	30.455205	26.698455	16.854924	22.031705	17.558708	27.725456
ARFGEF3	3.651212	5.94418	4.835641	7.27222	4.372242	7.81376	4.143255	7.728159
ARFIP1	18.656628	46.660567	19.189079	38.652046	37.995832	18.058146	68.935908	38.236984
ARFIP2	3.34539	73.174562	58.646082	46.120632	4.682869	81.194172	0	44.753217
ARFRP1	11.03309	13.97045	23.271749	19.537488	4.762252	14.502898	0.710065	12.017173
ARG1	0	0	0	0	0	0	0	0
ARG2	14.892096	9.420702	22.551049	13.774206	54.034503	43.500678	119.925747	45.892404
ARGFX	0	0	0	0	0	0	0	0
ARGFXP1	0	0	0	0	0	0	0	0
ARGFXP2	0	0	0	0	0	0	0	0
ARGLU1	15.386609	90.002882	58.483441	66.063204	30.876694	106.987801	10.517732	34.210665
ARHGAP1	44.280299	21.010508	29.042094	24.519042	45.863269	40.459529	83.916977	28.008295
ARHGAP10	9.809474	1.828561	0	4.298237	0	0.469911	2.305405	2.26081
ARHGAP11A	116.391867	225.169422	53.156559	155.597294	164.30229	264.809728	140.322199	220.993286
ARHGAP11B	6.584858	5.840046	0	8.175111	11.191205	8.332612	0	7.531205
ARHGAP12	2.094541	28.565685	3.454205	16.921767	1.062248	0.316633	10.266787	6.14726
ARHGAP15	0	0	0	0	0	0	0	0
ARHGAP16P	0	0	0	0	0	0	0	0
ARHGAP17	16.318522	58.076866	32.352857	50.356143	19.014857	73.729086	79.308789	81.049853
ARHGAP18	13.411238	13.288806	5.351809	10.281957	25.972588	10.099969	14.511472	15.630122
ARHGAP19	10.197734	9.808979	12.937161	6.959623	24.923456	3.347159	4.39116	4.50094
ARHGAP19-S	0	5.079969	0	8.030833	0	3.245914	0	1.975298

ARHGAP20	0	0.1427	0	0.094378	0	0.330266	0	0.671011
ARHGAP21	19.393026	12.559281	15.75563	21.323889	69.163523	31.35979	10.956807	23.9393
ARHGAP22	23.554924	13.349322	8.191455	10.95412	6.111531	10.493925	0	11.872399
ARHGAP23	158.546285	126.299224	89.902006	125.210241	191.560675	299.669931	341.544813	165.546979
ARHGAP23P1	0	0.215945	0	0	0	1.159141	0	0
ARHGAP24	12.447067	4.966666	0	4.620556	0	2.713787	0	2.831678
ARHGAP25	1.812082	1.958379	2.944544	7.146362	2.002429	5.270017	0	4.51995
ARHGAP26	17.509178	24.438451	6.930574	15.169148	79.356921	32.3734	0	7.052531
ARHGAP27	8.080972	20.48913	14.221635	38.01802	91.457964	42.446672	50.417833	31.366135
ARHGAP27P2	0	0	0	0	0	0	0	0
ARHGAP28	0	1.921375	0	1.379888	3.106373	0	0	0.711676
ARHGAP29	643.759311	1083.9797	536.966707	469.752293	96.563108	104.465599	90.123537	269.279867
ARHGAP30	0	0	0	0.04155	0	0	0	0.038402
ARHGAP31	0	0	0	0	0	0	0	0
ARHGAP32	16.972835	21.376468	11.049491	26.63184	19.909239	50.543133	57.883934	42.29218
ARHGAP33	0	0.697743	0	0	0	1.198294	0	1.932927
ARHGAP35	0	0	0	0	0	0.607721	0	2.560601
ARHGAP36	0	0	0	0	0	0	0	0
ARHGAP39	0	0	0	0.288366	0	0	0	0
ARHGAP4	0	0.415807	0	0.351323	0	0.101115	0	0
ARHGAP40	1.920272	0	1.712711	0	0	1.516963	0	0
ARHGAP42	18.148296	20.858607	5.66648	16.385642	2.403237	13.73494	33.803483	26.560137
ARHGAP42P1	0	0	0	0	0	0	0	0
ARHGAP42P2	0	0.54635	0	0	0	0	0	0
ARHGAP42P3	0	0.057673	0	0.099011	0	0	0	0
ARHGAP42P4	0	0	0	0	0	0	0	0
ARHGAP42P5	0	0	0	0	0	0	0	0
ARHGAP44	19.667138	8.269156	6.883841	8.847952	5.500597	4.305986	5.425086	5.753519
ARHGAP45	0	3.013365	2.599589	4.772282	24.820865	6.503288	52.77559	1.94556
ARHGAP5	84.156781	109.788903	102.302824	39.007587	124.328993	34.858558	206.063165	153.126214
ARHGAP6	0	0.35191	0	0.21069	0	0	0	0
ARHGAP8	0	4.008308	21.870506	6.720518	18.478394	12.954625	7.422283	2.625172
ARHGAP9	23.413354	14.501809	0	37.657896	0	2.458985	0	2.097487

ARHGDIA	176.596163	224.814185	307.474656	255.549916	335.285526	417.733842	282.956355	306.064219
ARHGDIB	305.294889	269.675161	149.374813	227.931319	202.271356	172.727631	178.84443	175.093155
ARHGDIG	0	1.267246	0	1.502499	0	2.455049	0	0.786945
ARHGEF1	23.850391	3.568681	0	9.309902	11.902008	17.433882	59.428611	18.238362
ARHGEF10	5.36409	10.657861	6.811265	13.346808	16.762953	24.804335	8.492548	10.223821
ARHGEF10L	5.667022	5.232328	2.549292	7.767429	0.648366	10.045152	0	6.936287
ARHGEF11	36.7233	10.667809	0	25.108894	19.143873	35.801632	22.100371	28.417761
ARHGEF12	230.117155	492.65789	191.873382	452.053198	81.739959	159.272353	50.078554	206.151082
ARHGEF15	0	0	0	0	0	0	0	0
ARHGEF16	5.973198	8.373695	10.855989	12.501161	11.868967	21.524107	6.405202	13.277309
ARHGEF17	7.961054	6.328841	10.923752	5.536734	12.230228	12.94344	25.797742	6.556447
ARHGEF18	0	0	0	0	0	0	0	0
ARHGEF19	4.017255	7.280064	3.601669	13.543433	5.037477	12.278798	4.534771	5.923475
ARHGEF2	33.336974	50.710354	38.816497	42.080454	35.302166	49.645083	2.160739	39.664575
ARHGEF25	3.322699	13.37989	13.089445	18.774104	15.394041	25.821904	0	11.254132
ARHGEF26	4.290219	9.607491	3.866245	8.722665	5.480048	11.139421	4.71758	11.390439
ARHGEF28	15.46469	30.463136	26.073812	24.344206	63.945093	26.711278	27.102537	34.572544
ARHGEF28P1	0	0	0	0	0	0	0	0
ARHGEF3	20.823613	21.397532	3.272963	24.417352	5.110517	20.673707	0	11.43141
ARHGEF33	0	0.29538	0	0.019551	0	0	0	0
ARHGEF34P	5.648232	9.264233	0	17.331071	16.301283	24.888431	0	13.096648
ARHGEF35-A	1.744871	0.900534	0	0.92479	0	0.235271	0	0.070405
ARHGEF37	1.324511	5.104214	3.569404	6.400956	4.023636	3.282593	3.548758	2.816794
ARHGEF38	0	0	0	0	0	0	0	0.203746
ARHGEF39	3.530396	14.926893	10.725155	11.758219	20.12887	15.072669	0	15.426242
ARHGEF4	0	1.024002	3.447858	2.191986	4.794844	4.860599	15.720357	4.719273
ARHGEF40	23.348044	29.04859	13.801157	25.667505	13.663829	67.155217	22.727519	35.65141
ARHGEF5	6.118298	18.067007	7.995495	15.337147	15.746921	28.512254	19.534774	24.761174
ARHGEF6	0.688136	1.535325	1.236116	1.731753	1.999454	0.654423	0	0.773239
ARHGEF7	22.229425	78.104095	45.258499	92.400125	93.308741	87.156899	66.122664	102.970168
ARHGEF9	36.210366	71.370949	42.427492	107.265322	57.55825	42.016113	5.150375	42.278262
ARID1A	21.549689	30.873316	16.873208	33.889093	46.085156	80.00904	144.388035	53.93763
ARID1B	28.20178	17.163578	14.142327	23.961855	28.879334	71.64659	19.59348	28.623421

ARID2	8.990132	4.577387	2.97973	2.577144	7.547008	2.64636	9.052949	4.084225
ARID3A	20.175369	14.218094	7.864081	21.184901	57.849541	31.573238	10.794935	25.757307
ARID3B	26.608572	1.099725	8.364383	1.85252	0	3.917709	0	0
ARID3BP1	0	0	0	0	0	0	0	0
ARID4A	0	7.843289	0	11.957087	22.219521	17.677251	5.524617	9.53749
ARID4B	24.293862	31.938889	52.416833	25.670321	61.43059	21.053439	56.682772	18.536945
ARID5A	4.734058	9.125886	4.553632	14.177027	5.825707	23.390416	10.289784	17.56033
ARID5B	6.900422	11.167731	6.594692	12.900888	23.959413	23.42303	27.700899	17.954281
ARIH1	75.780174	185.322586	107.69554	147.146184	166.614277	107.523786	122.819944	117.960944
ARIH2	45.310749	174.574536	29.243844	186.868887	68.277227	257.485458	0	236.864039
ARIH2P1	0	0	0	0	0	0	0	0
ARK2C	6.231386	6.186191	5.273326	7.60842	7.872485	16.555087	0	6.740106
ARK2N	19.91743	78.253977	37.328279	91.576182	74.04969	133.42952	131.01833	103.520785
ARL1	145.755635	136.269214	122.154266	124.809373	99.223582	76.367357	105.789615	104.371284
ARL10	5.627196	15.542109	7.198244	17.764203	15.460785	15.392346	2.457784	10.948352
ARL11	0	0	0	0	0	0	0	0
ARL13A	0	0	0	0	0	0	0	0
ARL13B	1.746654	18.017862	12.276864	24.076325	21.330233	11.773348	36.064189	12.372607
ARL14EP	8.637808	5.178899	13.714423	6.047658	11.77211	7.34962	51.193114	13.238721
ARL14EPL	0	0	0	0	0	0	0	0
ARL14EPP1	0	0	0	0	0	0.253587	0	0
ARL15	7.016781	20.234716	0	16.996223	14.658401	9.259537	4.750254	27.44569
ARL16	0	8.573354	0	6.066537	20.141569	3.898225	0	7.060507
ARL17A	1.299901	0.938762	0	2.404882	36.257735	0	4.06324	1.953104
ARL17B	2.599802	6.994002	6.601156	7.579335	0	7.425892	0	10.676565
ARL2	25.269304	50.99077	44.499138	50.117	55.629563	138.519001	55.31059	91.028798
ARL2-SNX15	0	0	0	0	0	0	0	0
ARL2BP	93.565896	155.659271	55.158351	85.825688	98.669188	114.226912	96.005007	136.809348
ARL2BPP1	0	0	0	0	0	0	0	0
ARL2BPP10	0	0	0	0	0	0	0	0
ARL2BPP2	0	0	0	0	0	0	0	0
ARL2BPP3	0	0	0	0	0	0	0	0
ARL2BPP4	0	0	0	0	0	0	0	0

ARL2BPP5	0	0	0	0	0	0	0	0
ARL2BPP6	0	0	0	0	0	0	0	0
ARL2BPP7	0	0	0	0	0	0	0	0
ARL2BPP8	0	0	0	0	0	0	0	0
ARL3	13.815202	19.368098	9.29695	12.771989	16.872226	8.244466	7.352592	10.370045
ARL4A	15.145703	111.118404	35.206917	69.975673	18.117846	33.241871	39.473346	69.577582
ARL4AP1	0	0	0	0	0	0	0	0
ARL4AP2	0	0.950828	0	0.358991	0	0	0	0
ARL4AP3	0	0	0	0	0	0	0	0
ARL4AP4	0	0	0	0	0	0	0	0
ARL4AP5	0	0	0	0	0	0	0	0
ARL4C	38.685202	54.793212	16.395208	40.66871	10.868081	33.585945	12.018002	48.357036
ARL4D	15.822957	21.33986	28.171196	35.683741	5.953483	26.138057	0	20.302155
ARL5A	7.981349	41.219068	72.890201	14.296056	11.164846	21.417525	3.514426	58.225326
ARL5AP1	0	0	0	0	0	0	0	0
ARL5AP2	0	0	0	0	0	0	0	0
ARL5AP3	0	0	0	0	0	0	0	0
ARL5AP4	0	0	0	0	0	0	0	0
ARL5AP5	0	0	0	0	0	0	0	0
ARL5B	4.511537	2.116581	8.520882	1.832332	8.3456	1.245286	4.730846	2.694413
ARL5C	0	0	0	0	0	0	0	0
ARL6	28.083276	17.662732	12.162371	10.440341	1.750302	4.608352	0	13.398049
ARL6IP1	174.572464	76.719902	152.032963	52.617344	117.030009	61.90312	137.539171	69.108448
ARL6IP1P1	0	0	0	0	0	0	0	0
ARL6IP1P2	0	0	0	0	0	0	0	0
ARL6IP1P3	0	0	0	0	0	0	0	0
ARL6IP4	37.3415	206.823792	99.514259	171.62377	92.336225	228.663958	36.441618	121.741128
ARL6IP5	38.785097	32.78048	41.871647	35.075448	50.710655	17.23788	21.141885	13.854186
ARL8A	31.413322	40.179642	31.512447	56.456341	29.490937	38.966969	7.897272	30.068458
ARL8B	77.955823	59.554596	60.592071	46.685374	43.338759	36.829541	75.618531	43.354692
ARL8BP1	0	0	0	0	0	0	0	0
ARL8BP2	0	0	0	0	0	0	0	0
ARL9	0	0	0	0.224715	0	1.984742	0	2.175889

ARMC1	38.050658	58.766732	30.081248	58.284781	41.563683	26.477092	45.960515	51.137918
ARMC10	70.63395	40.124947	46.229913	11.766619	28.433525	7.946572	30.216016	25.127809
ARMC10P1	0	0	0	0	0	0	0	0
ARMC12	0	0	0	0.308122	0	0	0	0.26008
ARMC2	0	9.920854	4.23886	4.349993	0	6.230377	0	5.973606
ARMC3	3.987761	0.175167	0	0.118327	0	0	0	0.055301
ARMC5	1.873143	0.471163	0	2.108909	0	6.135654	0	4.406443
ARMC6	44.824421	62.521645	13.8395	60.605461	10.962681	151.762557	75.067446	144.819077
ARMC7	12.816654	24.158527	8.588922	28.451086	27.557659	36.334764	9.315931	34.814068
ARMC8	47.580612	57.715177	12.328067	36.114631	17.047972	35.970896	66.876669	64.843459
ARMC8P1	0	0	0	0	0	0	0	0
ARMC9	0	4.024482	11.298226	4.585381	0	4.975706	0	3.862776
ARMCX2	38.48575	130.920027	50.177116	192.922231	43.24979	125.953307	55.830538	102.67866
ARMCX3	29.395837	125.547049	33.987583	171.920094	25.243773	122.695558	39.35535	87.085575
ARMCX4	0	5.605054	0	2.240056	0	3.382764	83.432389	7.258
ARMCX5	15.287197	20.343758	13.516862	24.791893	5.744307	20.940254	0	34.266621
ARMCX5-GP	0	1.976065	1.057979	1.189207	0.323574	1.548485	0	1.280303
ARMCX6	34.083963	102.038625	22.084979	106.031133	9.316505	112.788332	0	88.548317
ARMCX7P	8.316155	0	0	0	13.17899	0	0	0
ARMH1	0	1.777563	0	1.279267	0	0.275079	0	0
ARMH3	20.125266	19.481201	11.560798	18.771649	16.369776	23.606409	38.292802	14.726262
ARMH4	0	20.318998	0	18.588166	0	22.172627	0	23.424173
ARMS2	0	0	0	0	0	0	0	0
ARMT1	19.936346	18.79951	17.831428	6.172645	17.784072	6.318768	32.442277	28.605401
ARNT	20.813475	28.55804	25.100342	42.215266	21.59028	32.968123	19.671044	25.220975
ARNT2	12.093841	10.37267	4.127723	9.215198	0.262759	1.52131	3.151636	1.754157
ARPC1A	140.968007	113.108851	94.307646	87.590334	94.390734	58.813348	151.529502	80.651947
ARPC1AP1	0	0	0	0	0	0	0	0
ARPC1AP2	0	0	0	0	0	0	0	0
ARPC1AP3	0	0	0	0	0	0	0	0
ARPC1AP4	0	0	0	0.081841	0	0	0	0.138468
ARPC1B	127.934385	139.748424	59.148194	108.469348	33.764385	84.513993	64.634617	85.615758
ARPC1BP1	0	0	0	0	0	0	0	0

ARPC2	398.300791	373.185371	226.104885	275.914516	638.563974	548.18547	475.024974	757.294788
ARPC3	454.763668	319.182087	317.297554	257.465338	623.91246	381.060822	966.439457	355.724302
ARPC3P1	0	0	0	0	0	0	0	0
ARPC3P2	0	0	0	0	0	0	0	0
ARPC3P3	0	0	0	0	0	0	0	0
ARPC3P4	0	0	0	0	0	0	0	0
ARPC3P5	0	0	0	0	0	0	0	0
ARPC4	107.394017	171.859946	78.388125	151.527142	34.101199	91.656048	68.574998	80.519851
ARPC4-TTLL3	14.808111	6.646498	23.628171	7.875498	70.407744	4.860221	0	4.167703
ARPC5	171.825495	259.664396	110.76292	216.423106	182.970864	353.01379	48.221624	276.833399
ARPC5L	54.698397	191.064085	76.506812	127.406194	57.799684	133.182435	83.155164	157.151379
ARPIN	12.974426	12.729705	9.210965	11.961266	8.663492	8.016349	2.180591	4.459997
ARPIN-AP3S2	13.880646	17.382433	13.569734	22.93304	0	20.797394	0	14.64114
ARPP19	24.321608	156.385987	0	90.019972	6.332862	100.192692	0	85.992686
ARPP19P1	0	0	0	0	0	0.823512	0	0
ARPP19P2	0	5.506363	0	0	0	0	0	0.851746
ARPP21	0	0	0	0	0	0	0	0
ARR3	0	0	0	0	0	0	0	0
ARRB1	1.319098	6.289065	2.038867	2.614061	14.217487	7.602053	0	5.510407
ARRB2	15.904802	33.77456	21.151619	46.870871	53.136015	51.220883	19.440463	39.389181
ARRDC1	25.16799	46.481803	15.077561	44.924767	28.902414	52.795344	27.618417	42.767983
ARRDC2	6.800588	17.660605	9.735635	21.037368	6.458819	31.140299	5.939771	25.147563
ARRDC3	16.788373	11.695693	13.954327	10.636769	3.769266	10.515656	63.795559	24.087196
ARRDC4	11.377666	5.952922	2.188234	1.76594	14.594132	4.630806	17.998074	12.662395
ARSA	4.600923	2.34877	5.74388	7.199969	0	1.45527	0	0.532412
ARSB	10.127471	12.837703	6.670976	7.601924	10.879136	13.207811	12.724617	8.424391
ARSD	7.312417	14.565444	16.678965	14.542365	1.006959	12.778031	6.569004	19.726708
ARSDP1	0	0	0	0	0	0	0	0
ARSF	0	0	0	0	0	0	0	0
ARSFP1	0	0	0	0	0	0	0	0
ARSG	0	0	0	0.066045	0	0.141514	0	0
ARSI	7.780171	14.835617	7.714125	25.574673	3.124094	28.688338	4.44491	16.527562
ARSJ	7.132052	59.848152	10.144497	69.722248	19.801044	45.686639	11.125793	52.658601

ARSK	3.485104	14.771216	0	16.916032	9.103777	9.336057	0	8.354457
ARSL	0	0	0	0.494175	0	0	0	0
ARSLP1	0	0	0	0	0	0	0	0
ART1	0	0	0	0	0	0	0	0
ART2BP	0	0	0	0	0	0	0	0
ART2P	0	0	0	0	0	0	0	0
ART3	0	0	0	0	0	0	0	0
ART4	0	0	0	0	0	0	0	0
ART5	0	0	0	0	0	0	0	0
ARTN	18.470502	12.513695	2.498742	15.611793	38.080715	80.184756	16.939961	57.467112
ARV1	21.10419	13.155535	19.807318	9.691513	17.898718	6.210931	19.446685	12.332294
ARVCF	4.079729	4.157467	6.492829	3.526021	25.924976	5.551025	9.187518	3.099559
ARX	0	0	0	0.056691	0	0.115842	0	0
AS3MT	15.297962	7.547487	9.952986	13.741506	0	0	0	3.736645
ASAH1	29.024864	28.085251	35.098027	32.01738	37.438796	15.189601	62.334458	18.610195
ASAH2	0	11.069608	0.457244	10.088298	0.119206	0.098374	41.24407	4.846323
ASAH2B	3.570186	0	2.300426	4.47865	0.235674	12.431611	0	8.355809
ASAP1	102.36621	192.610311	55.207271	218.495537	182.759206	395.928219	147.331246	301.318475
ASAP2	52.698263	115.544965	64.540227	130.739861	109.917204	148.482473	65.978758	114.789464
ASAP3	6.654148	8.66586	3.657381	2.170464	15.821633	3.973169	0	1.963695
ASB1	26.160957	29.754915	2.112944	41.472047	18.395435	58.465966	9.739631	42.806225
ASB10	0	0	0	0	0	0	0	0
ASB11	0	0	0	0	0	0	0	0
ASB12	0	0	0	0	0	0	0	0
ASB13	2.647129	18.599001	8.291459	18.325058	23.908662	17.629999	12.205027	13.719761
ASB14	0	0	0	0	0	0	0	0
ASB15	0	0	0	0	0	0	0	0
ASB16	0	0	0	0	0	0.19937	0	0
ASB18	0	0	0	0	0	0	0	0
ASB2	3.176714	2.173281	11.349129	8.970628	0	9.073162	0.384868	7.338648
ASB3	0	21.057732	21.119262	3.86007	12.855727	3.938019	0	4.94766
ASB4	0	0	0	0	0	0	0	0
ASB5	0	0	0	0	0	0	0	0

ASB6	9.984873	17.921541	9.756756	15.483099	5.073418	30.511706	23.25649	19.318496
ASB7	0	23.705808	0	31.887045	11.101843	38.585158	0	28.879047
ASB8	10.728171	18.101027	7.199988	18.934305	15.920904	17.856691	171.407766	30.018552
ASB9	2.206618	8.682178	1.964928	6.841218	3.6914	8.605374	10.147146	11.605175
ASB9P1	0	0	0	0	0	0	0	0
ASCC1	7.85859	18.954555	0	31.436167	45.115752	30.351023	0	18.202411
ASCC2	36.488469	35.524046	44.700206	48.431907	30.616608	107.07266	64.005878	70.063284
ASCC3	50.012083	89.522871	40.263888	60.7455	47.83564	51.251028	94.02222	127.487879
ASCL1	0	0	0	0	0	0	0	0
ASDURF	0	0	7.68264	4.563228	0	0	78.400578	0
ASF1A	16.807339	34.269001	11.276087	18.702751	18.473933	30.287084	40.29973	40.934473
ASF1B	50.250038	50.361283	31.715389	41.181762	101.113664	96.993469	56.154072	72.052467
ASGR1	0	1.284884	2.483458	4.548192	0	0.410502	0	1.185414
ASGR2	0	0	0	0	0	0	0	0
ASH1L	18.764105	19.249612	27.443786	18.835649	24.221554	16.647107	24.800732	21.621411
ASH2L	11.675637	38.079527	41.791245	27.970324	14.77939	20.244759	53.597992	19.702134
ASH2LP1	0	0	0	0	0	0	0	0
ASH2LP2	0	0	0	0	0	0	0	0
ASH2LP3	0	0.72769	0	0	0	0	0	0
ASH2LP4	0	0	0	0	0	0	0	0
ASIC1	9.449849	4.966626	4.494966	4.445557	0	0.771261	0	4.489813
ASIC2	0	0	0	0	0	2.112917	6.137721	0
ASIC3	0	0.378245	0	0	0	0	0	0
ASIC4	0	0	0	0	0	0	0	0
ASIP	0	0	0	0	0	0	0	0
ASL	0	0	0	0.573693	0	0.874609	0	0
ASLP1	0	0	0	0	0	0	0	0
ASMT	0	0	0	0.093863	0	0	0	0
ASMTL	16.753137	22.850099	19.451911	34.840204	37.98793	46.063481	20.240954	29.396186
ASNS	92.769622	75.608348	77.258613	67.688941	35.373626	13.326622	0	23.946816
ASNSD1	33.288397	62.069727	36.706233	19.076558	23.516885	36.318381	52.492123	82.713889
ASNSP1	0	0	0	0	0	0	0	0
ASNSP3	0	0	0	0.067779	0	0	0	0

ASNSP4	0	0	0	0	0	0	0	0
ASNSP6	0	0	0	0.053223	0	0	0	0
ASPA	0	0	0	0	0	0	0	0
ASPDH	0	0	0	0	0	0	0	0
ASPG	0	0	0	0.295521	0	0	0	0
ASPH	470.733535	961.64087	549.150517	1049.19573	108.166606	316.077616	99.030199	234.674216
ASPHD1	8.294325	30.252381	6.888203	26.1947	3.928387	8.118123	0	6.587765
ASPHD2	2.967554	2.536893	0.88693	2.919306	0.522288	5.477375	0	4.7739
ASPM	9.917928	11.264967	14.081014	5.599431	22.000923	7.84033	13.11087	18.56278
ASPN	0	0	0	0	0	0	0	0
ASPSCR1	2.966802	13.822571	8.890367	9.360781	49.5153	19.91854	42.689529	14.623001
ASRGL1	11.649405	12.940182	11.275835	9.257093	0.831869	11.727635	0	10.444585
ASS1	41.582227	17.222779	0	3.119792	45.628703	0	48.526901	21.83104
ASS1P1	0	0	0	0	0	0	0	0
ASS1P10	0	0	0	0	0	0	0	0
ASS1P11	0	0	0	0	0	0	0	0
ASS1P12	0	0	0	0	0	0	0	0
ASS1P13	0	0	0	0	0	0	0	0
ASS1P14	0	0	0	0	0	0	0	0
ASS1P2	0	0.504819	0	0.073098	0	0	0	0
ASS1P3	0	0	0	0	0	0	0	0
ASS1P4	0	0	0	0	0	0	0	0
ASS1P5	0	0	0	0	0	0.14574	0	0
ASS1P6	0	0	0	0	0	0	0	0
ASS1P7	0	0	0	0	0	0	0	0
ASS1P8	0	0	0	0	0	0	0	0
ASS1P9	0	0	0	0	0	0	0	0
ASTE1	5.433437	9.061591	0	6.440987	24.65068	6.54023	7.909506	5.503496
ASTL	0	0	0	0	0	0	0	0
ASTN1	0	0.095795	0	0.134577	0	0	0	0
ASTN2	4.006526	2.591294	1.785894	3.432788	0	1.338951	0	2.047512
ASXL1	155.897255	359.190794	168.936943	473.243391	23.075013	155.903135	19.809462	89.480493
ASXL2	0	22.571038	0	44.16048	11.684282	65.087185	21.403775	22.970415

ASXL3	0	0	0	0	0	0	0	0
ASZ1	0	0	0	0	0	0	0	0
ATAD1	37.94557	51.218592	28.782889	15.655689	19.623849	7.723028	66.265131	38.44984
ATAD2	48.777078	41.425861	50.965953	35.495381	86.168692	46.004499	178.03928	73.002813
ATAD2B	4.770607	6.665193	8.826501	5.360945	2.308373	5.534649	2.997418	6.765344
ATAD3A	0	0	0	0	104.079824	10.44559	0	19.811853
ATAD3B	3.496208	4.274087	1.029396	0.670997	0.335543	0.634715	0	0.905431
ATAD3C	0	0	0	0.981884	0	0	0	0
ATAD5	10.837238	19.867654	10.169351	9.811621	8.342275	7.905792	16.294667	22.812983
ATAT1	11.794232	5.71647	5.143565	4.81526	9.740955	3.768627	0	5.258685
ATCAY	0.658382	0.1413	1.182869	0.343951	0	0.05769	0	0
ATF1	12.729953	169.715486	35.481187	148.959565	29.613154	88.274586	14.408598	81.702662
ATF1P1	0	0	0	0	0	0	0	0
ATF2	44.498449	46.263113	22.127253	51.144199	69.791243	46.853575	28.199766	67.962927
ATF3	39.47994	62.281	16.227436	47.08847	52.633077	148.704237	0	32.941546
ATF4	3.391566	1.762619	2.856786	0.858107	0	1.349329	0	1.889791
ATF4P2	0	0	0	0.085177	0	0	0	0
ATF4P3	0	0	0	0.088399	0	0	0	0
ATF4P4	0	0.455245	0	0	0	0	0	0.148602
ATF5	12.880485	23.340604	7.717154	20.780829	10.937418	66.149909	8.37042	52.789506
ATF6	19.466033	10.144699	22.178211	12.225222	19.298035	7.295863	9.495087	9.015305
ATF6B	23.147754	10.680388	45.65726	33.571553	42.228888	29.922884	38.143889	20.11175
ATF7	43.31495	17.602608	10.940499	16.467178	37.183479	14.520616	8.236461	10.910365
ATF7-NPFF	0	0.833015	0	3.223938	0	0	0	0
ATF7IP	10.116847	45.868819	31.631743	58.974574	83.545749	39.65066	49.473882	47.403995
ATF7IP2	0	4.365049	8.656665	1.653133	0.48208	0.905601	0	1.114843
ATG10	9.780111	14.011537	8.978435	5.375682	3.608257	3.189037	0	5.201117
ATG101	0	32.942811	8.563014	27.75775	22.613211	36.381382	18.180127	24.737088
ATG12	25.436456	50.397759	77.268037	25.21133	126.589361	16.812497	35.219389	36.519942
ATG12P1	0	0	0	0	0	0	0	0
ATG12P2	0	0	0	0	0	0	0	0
ATG13	38.793003	105.093295	21.450231	113.786234	80.430837	235.030527	109.287346	283.427221
ATG14	1.399183	0	0	0	0	0	6.47388	0.317137

ATG16L1	6.242697	22.954895	15.60883	22.168368	23.583202	39.844398	53.555375	42.300574
ATG16L2	2.920959	12.534226	10.154444	9.68394	1.005263	4.548088	0	3.495661
ATG2A	3.457085	3.124113	5.178338	4.045154	14.119645	15.189585	4.633529	9.546426
ATG2B	8.257257	22.307502	7.653874	13.651049	6.163549	16.224829	4.269203	18.322974
ATG3	0	101.349514	0	98.772567	5.94875	87.952547	0	89.953046
ATG3P1	0	0	0	0	0	0	0	0
ATG4A	7.174086	23.945151	3.201615	12.344381	5.200624	6.930593	50.823724	12.69009
ATG4AP1	0	0	0	0	0	0	0	0
ATG4B	61.916358	45.753256	45.996103	50.281294	78.81405	48.91166	91.291003	49.141332
ATG4C	9.316989	43.321198	20.456655	25.551086	36.052998	6.461754	0	18.974007
ATG4D	6.028948	10.20216	21.831378	8.560107	7.519357	11.166209	0	11.496795
ATG5	28.88574	84.176767	27.242026	38.560442	18.582584	32.473159	96.114549	106.061005
ATG7	53.292939	26.555072	20.832013	45.828332	17.913407	17.907637	111.027121	28.923838
ATG9A	31.549284	25.910895	13.433169	22.424746	27.219378	51.789122	3.570375	75.823481
ATG9B	0	1.125214	0	0.295194	0	0.718603	0	0
ATIC	109.026647	113.44356	94.515239	73.573442	143.566181	78.985464	358.058362	141.503601
ATL1	0	2.771272	0	5.098826	0	0.883863	0	2.271356
ATL2	39.026615	73.952711	87.379519	55.025521	108.065749	34.130444	89.686802	71.005458
ATL3	18.124772	24.12159	19.540263	19.388143	56.583728	32.23789	26.592886	40.945108
ATM	0.594273	10.805724	7.575937	16.142419	0	13.193059	62.723978	19.184827
ATMIN	62.293302	70.989026	57.757173	42.791551	58.739711	51.94131	154.461632	85.46971
ATN1	45.514699	55.610555	39.384652	58.857797	60.32663	84.886885	30.108861	47.498123
ATOH8	0	0	0	0.04223	0	0	0	0
ATOSA	8.074072	14.705158	4.496553	17.373167	129.70012	17.913449	0	24.32632
ATOSB	4.478138	5.798302	3.009186	9.546748	17.987239	2.018634	0.321051	5.492755
ATOSBP1	0	0	0	0	0	0	0	0
ATOX1	121.110975	158.939265	83.875638	155.317337	71.838191	167.535351	75.462709	195.191683
ATP10A	0	0	0	0	0	0	0	0
ATP10B	0	0	0	0	0	0	0	0
ATP10D	7.025109	34.113425	4.202573	37.227048	0	19.108912	5.494495	13.135042
ATP11A	35.723093	41.921867	30.432862	62.536222	16.417371	72.613504	55.486289	44.021311
ATP11B	34.188578	72.576276	95.184832	50.385714	77.548636	30.667594	138.552258	70.15822
ATP11C	39.809891	28.333678	20.327906	20.522917	42.910568	10.467954	16.350264	35.308348

ATP12A	0	0	0	0.021678	0	0	0	0
ATP13A1	7.73925	6.502642	21.294649	4.570298	11.580645	11.4434	13.541619	6.346263
ATP13A2	39.628915	33.943952	30.818442	34.914032	31.12659	57.259961	90.421059	50.290486
ATP13A3	20.056809	90.674544	102.996196	12.783643	105.595682	11.414299	171.00561	80.630683
ATP13A4	0	0	0	0.195734	0	0	0	0.698368
ATP13A5	0	0	0	0	0	0	0	0
ATP1A1	356.202296	363.6465	562.024758	453.598727	233.219994	172.860169	158.520691	154.630756
ATP1A2	0	0	0	0.162472	0	0	0	0
ATP1A3	7.508253	0	5.446978	0.783415	0.769688	1.962283	0	2.497336
ATP1A4	0	0	0	0	0	0	0	0
ATP1B1	31.626643	18.888364	11.937307	5.80045	31.420145	3.188023	22.0768	13.952864
ATP1B1P1	0	0.705844	0	0	0	0	0	0.704827
ATP1B2	0	0.810493	0	1.105452	0	0.24897	0	0.120377
ATP1B3	176.147051	157.774524	193.830996	113.563939	316.83301	112.061063	168.847092	203.478978
ATP1B3P1	0	0	0	0	0	0	0	0
ATP1B4	0	0	0	0	0	0	0	0
ATP23	2.331832	0	0	5.558631	33.480512	3.446769	1.812809	8.448484
ATP2A1	0	0.319686	0	0.469432	0.497063	0.866029	0	0.264779
ATP2A2	246.187885	327.840592	269.866798	327.686832	311.950464	297.390999	265.176265	372.89401
ATP2A3	3.231422	0.949008	0.627473	0.942806	1.02759	2.147798	0	0.365077
ATP2B1	0	3.61276	0	0	67.431639	0	0	16.229312
ATP2B2	0	0	0	0	0	0	0	0
ATP2B3	0	0	0	0.019832	0	0.0406	0	0
ATP2B4	0	0	0	0	0	0.757485	3.741418	0.624751
ATP2C1	67.294999	141.84789	130.497573	109.302561	44.176158	56.398534	317.840813	105.029917
ATP2C2	4.85212	0	8.690035	1.80949	0	1.09442	87.068157	2.389096
ATP4A	0	0	0	0	0	0	0	0
ATP5F1A	323.809649	201.785956	283.498313	175.33364	339.107322	169.866922	357.428242	267.933449
ATP5F1AP1	0	0	0	0	0	0	0	0
ATP5F1AP10	0	2.801659	1.977231	1.319645	0	0	0	0
ATP5F1AP2	0	0	0	0	0	0	0	0
ATP5F1AP3	0	0	0	0	0	0	0	0
ATP5F1AP4	0	0	0	0	0	0	0	0

ATP5F1AP7	0	0	2.552603	1.628739	0	0	0	0
ATP5F1AP8	0	0	0	0	0	0	0	0
ATP5F1B	625.92701	507.268527	580.694897	450.148836	375.367011	281.181149	410.424453	351.080872
ATP5F1BP1	0	0	0	0	0	0	0	0
ATP5F1C	103.071564	51.008225	100.265321	52.121823	57.774897	43.253546	115.144315	50.249861
ATP5F1CP1	0	0	0	0	0	0	0	0.727386
ATP5F1D	23.307321	103.824364	51.399761	101.057503	153.766513	422.127779	188.53162	238.245211
ATP5F1E	70.284458	114.640851	66.300716	68.397355	68.272297	110.178826	24.16353	126.338259
ATP5F1EP1	0	0	0	0	0	0	0	0
ATP5F1EP2	0	0	0	0	0	0	0	0
ATP5IF1	188.669551	308.655904	100.835264	275.429385	132.248578	279.407223	143.653605	267.24533
ATP5MC1	182.360278	149.964972	129.460265	130.094465	188.212836	80.252234	99.787766	94.793109
ATP5MC1P1	0	0	0	0	0	0	0	0
ATP5MC1P2	0	0	0	0.624922	0	0	0	0
ATP5MC1P3	0	0	0	0.313701	0	0	0	0
ATP5MC1P4	0	0	0	0	0	0	0	0
ATP5MC1P5	0	0	0	0	0	0	0	0
ATP5MC1P6	0	0	0	0	0	0	0	0
ATP5MC1P7	0	0	0	0	0	0	0	0
ATP5MC1P8	0	0	0	0	0	0	0	0
ATP5MC2	2.403499	48.392596	67.190661	128.572054	0	40.232123	31.519579	36.82754
ATP5MC2P1	0	0	0	0	0	0	0	0
ATP5MC2P2	0	0	0	0	0	0	0	0
ATP5MC2P3	0	0	0	0	0	0	0	0
ATP5MC2P4	0	0	0	0	0	0	0	0
ATP5MC2P5	0	0	0	0	0	0	0	0
ATP5MC3	132.243347	301.716864	160.758912	218.745281	192.747993	328.566993	205.692998	398.324956
ATP5ME	261.426256	248.85106	289.903817	255.384053	409.17179	417.663097	313.687811	395.678649
ATP5MF	434.668824	301.24771	99.480986	341.454088	324.926856	400.360188	647.163488	297.353479
ATP5MF-PTC	0	4.672582	3.921627	7.798749	7.428729	8.641064	0.376866	6.560491
ATP5MFP1	0	0	0	0	0	0	0	0
ATP5MFP2	0	0	0	0	0	0	0	0
ATP5MFP3	0	0	0	0	0	0	0	0

ATP5MFP4	0	0	0	0	0	0	0	0
ATP5MFP5	0	0	0	0	0	0	0	0
ATP5MFP6	0	0	0	0	0	0	0	0
ATP5MFP7	0	0	0	0	0	0	0	0
ATP5MG	2580.17001	1721.66558	2379.67522	1569.87256	609.643428	181.250275	456.970065	257.899566
ATP5MGL	0	0	0	0	0	0	0	0
ATP5MGP1	0	0	0	0	0	0	0	0
ATP5MGP2	0	0	0	0	0	0	0	0
ATP5MGP3	0	0	0	0	0	0	0	0
ATP5MGP4	0	0	0	0	0	0	0	0
ATP5MGP5	0	0	0	0	0	0	0	0
ATP5MGP6	0	0	0	0	0	0	0	0
ATP5MGP7	0	0	0	0	0	0	0	0
ATP5MGP8	0	0	0	0	0	0	0	0
ATP5MJ	196.939056	77.749424	9.307862	39.705292	153.767412	34.643205	0	21.025249
ATP5MK	583.28964	367.557109	549.747886	159.67709	319.854088	122.850529	87.246216	347.830919
ATP5MKP1	0	0	0	0	0	0	0	0
ATP5PB	746.015347	422.609204	527.528795	327.566187	346.565817	154.655453	482.189243	259.690228
ATP5PBP1	0	0	0	0	0	0	0	0
ATP5PBP2	0	0	0	0	0	0	0	0
ATP5PBP3	0	0	0	0	0	0	0	0
ATP5PBP4	0	0	0	0	0	0	0	0
ATP5PBP5	0	0.882694	0	1.039878	0	0	0	0
ATP5PBP6	0	0	0	0	0	0	0	0
ATP5PBP7	0	0	0	0	0	0	0	0
ATP5PBP8	0	0	0	0.170339	0	0	0	0
ATP5PD	521.999856	480.231211	409.933585	410.672672	424.799454	317.835539	214.315915	372.799633
ATP5PDP1	0	0	0	0	0	0	0	0
ATP5PDP2	0	0	0	0	0	0	0	0
ATP5PDP3	0	0	0	0	0	0	0	0
ATP5PDP4	0	0	0	0	0	0	0	0
ATP5PF	104.029708	74.986102	3.543728	45.309362	20.588452	67.217822	140.427274	124.543263
ATP5PFP1	0	0	0	0	0	0	0	0

ATP5PFP2	0	0	0	0	0	0	0	0
ATP5PFP3	0	0	0	0	0	0	0	0
ATP5PFP4	0	0	0	0	0	0	0	0
ATP5PO	280.807486	143.769072	197.323317	132.884352	275.274072	139.853546	369.437376	230.568189
ATP5POP1	0	0	0	0	0	0	0	0
ATP6AP1	32.018162	26.54069	43.645488	38.183987	46.208221	28.133274	17.548462	16.169537
ATP6AP1L	0	0	0	0.489626	0	1.447152	16.105352	0
ATP6AP2	64.181548	72.31918	39.351694	62.630624	103.418341	60.921579	162.422269	47.921184
ATP6V0A1	8.027554	44.20977	25.981417	22.845088	28.653082	35.99569	44.582532	43.558673
ATP6V0A2	28.087197	10.667024	2.689071	10.297062	12.602134	9.794369	0.729136	8.083053
ATP6V0A4	0	0.106316	0	0.030396	0	0.310412	0	0.619607
ATP6V0B	70.618219	52.31181	48.44034	46.31879	6.180711	63.292364	73.636802	64.088873
ATP6V0C	152.50148	255.038334	186.9377	263.408905	135.713583	247.279752	94.851996	276.089209
ATP6V0CP1	0	0	0	0	0	0	0	0
ATP6V0CP2	0	0	0	0	0	0	0	0
ATP6V0CP3	0	0	0	0	0	0	0	0
ATP6V0CP4	0	0	0	0	0	0	0	0
ATP6V0D1	43.403103	49.512125	44.455691	45.747754	30.831387	40.581581	22.013416	61.340234
ATP6V0D2	0	0	0	0.105208	0	0	0	0
ATP6V0E1	183.14047	181.887018	127.742256	149.212187	119.832396	118.516075	57.172349	138.848275
ATP6V0E1P1	0	0	0	0	0	0	0	0
ATP6V0E1P2	0	0	0	0	0	0	0	0
ATP6V0E1P3	0	0	0	0	0	0	0	0
ATP6V0E1P4	0	0	0	0	0	0	0	0
ATP6V0E2	0	3.878525	22.570104	0.276265	57.63688	0.955958	0	1.426565
ATP6V1A	57.420212	60.588683	79.522296	61.227779	134.520675	54.168084	72.303787	90.065053
ATP6V1B1	0	0	0	0.310742	0	0	0	2.338676
ATP6V1B2	40.100982	48.653969	50.463516	58.305508	56.833545	36.357268	86.909331	39.859693
ATP6V1C1	67.62638	112.644889	94.551673	64.968808	89.758395	26.261642	116.082427	83.20576
ATP6V1C2	3.271672	0.092211	0	0.131641	0	0.827372	0	0.434299
ATP6V1D	185.289159	126.078448	124.526915	122.145368	183.689559	124.728404	156.642059	166.849206
ATP6V1E1	68.55601	83.05066	58.428695	88.299457	104.254884	114.086963	71.717936	146.421139
ATP6V1E1P1	0	0	0	0	0	0	0	0

ATP6V1E1P2	0	0	0	0	0	0	0	0
ATP6V1E1P3	0	0	0	0	0	0	0	0
ATP6V1E2	3.692629	48.629582	24.352678	39.477278	3.681515	13.084634	0	14.396769
ATP6V1F	31.8648	95.3813	121.986521	94.964666	108.261064	80.067981	30.082591	70.330555
ATP6V1FP1	0	0	0	0	0	0	0	0
ATP6V1FP2	0	0	0	0	0	0	0	0
ATP6V1G1	43.415054	71.104618	54.913354	63.52601	30.089746	50.5885	81.623791	49.047334
ATP6V1G1P1	0	0	0	0	0	0	0	0
ATP6V1G1P2	0	0	0	1.255167	0	0	0	0
ATP6V1G1P3	0	0	0	0	0	0	0	0
ATP6V1G1P4	0	0	0	0	0	0	0	0
ATP6V1G1P5	0	0	0	0	0	0	0	0
ATP6V1G1P6	0	0	0	0	0	0	0	0
ATP6V1G1P7	0	0	0	0	0	0	0	0
ATP6V1G2	0	0	0	0.211688	0	0	0	0
ATP6V1G2-D	5.587582	0	0	0	0	0	0	0
ATP6V1G3	0	0	0	0	0	0	0	0
ATP6V1H	59.929988	80.406666	14.816183	35.8427	44.418702	31.887322	15.624071	40.470916
ATP7A	0	5.645102	0	4.486019	2.406382	3.190126	4.799715	7.931958
ATP7B	1.676148	0.587288	2.064006	2.652498	1.355837	1.308963	0	0.823442
ATP7BP1	0	0	0	0	0	0	0	0
ATP8A1	1.963728	11.655462	13.350616	1.18232	5.780366	0.138062	0	0.18885
ATP8A2	0	0.392668	0	0.008133	0	0.544343	0	2.264622
ATP8A2P2	0	0	0	0	0	0	0	0
ATP8A2P3	0	0	0	0	0	0	0	0
ATP8B1	0	38.60175	0	25.079594	11.639083	26.684867	49.034118	20.760129
ATP8B2	0	11.113299	0	9.276425	36.670231	13.542555	20.265851	8.229748
ATP8B3	4.162007	1.85152	2.306227	3.348436	0	0.123376	0	0.548069
ATP8B4	1.263099	0.268934	2.261539	0.203377	0	1.122702	0	0.224836
ATP8B5P	0	0	0	0	0	0	0	0
ATP9A	0	0	0	0	0	0	9.750139	0
ATP9B	43.137945	7.384676	6.806686	8.40364	27.589187	7.986545	36.560571	14.908325
ATPAF1	5.261026	11.428634	24.56384	11.270059	55.458352	19.430609	35.195026	16.832412

ATPAF2	6.905067	35.76318	26.576013	33.159849	20.160346	32.187997	22.489598	44.694998
ATPSCKMT	28.78918	18.998776	3.225231	14.516644	1.428887	12.902487	0	13.593306
ATR	19.514944	16.528676	16.422055	9.352892	18.550822	10.190943	101.51453	20.364796
ATRAID	0	35.159236	0	21.010561	0	26.943043	0	41.646729
ATRIP	17.863155	5.487756	6.063999	4.310176	18.132737	5.241632	4.881093	9.039033
ATRN	26.796629	17.530322	32.92842	21.359551	23.513884	11.05113	17.339207	12.65501
ATRNL1	0	0	0	0.054537	0	0.190415	0	0
ATRX	51.285884	69.724826	43.492467	78.815312	116.516143	87.155286	76.209751	75.208494
ATXN1	3.441265	8.227426	7.580016	16.791806	23.633461	17.878888	0	20.789747
ATXN10	171.117829	78.187601	65.761491	97.796487	99.548643	94.790333	225.714604	107.295201
ATXN1L	22.886346	38.123186	20.220736	35.081026	35.684945	61.572245	25.719247	49.017569
ATXN2	45.397322	88.849473	64.499755	79.441053	249.226123	110.677197	87.573056	108.834732
ATXN2L	78.686906	91.604126	92.990825	113.225083	96.660206	110.069396	73.086414	93.204595
ATXN3	54.944995	53.669228	36.460372	39.413265	49.579517	20.116885	30.401273	42.541342
ATXN7	7.339642	14.504982	26.00156	23.269104	23.383477	17.340143	24.319465	18.513092
ATXN7L1	9.028283	8.669713	0	4.212734	4.546502	1.820979	10.719178	3.706483
ATXN7L2	5.552314	4.624686	4.747429	6.29401	0.779008	5.3528	0	2.888045
ATXN7L3	22.592049	80.049396	19.360101	56.30574	21.32469	76.311254	47.563665	57.317504
ATXN7L3P1	0	0	0	0	0	0	0	0
AUH	2.412686	0	0	0.96231	34.751818	0.430817	0	0
AUNIP	7.825054	2.627241	9.269634	7.156207	0	10.854776	9.484945	14.195645
AUP1	98.930702	93.850546	78.361412	107.852431	53.3379	95.12612	59.036082	82.151421
AURKA	99.572645	111.319551	76.874904	112.067047	90.213013	169.046683	95.965905	206.349387
AURKAIP1	48.937314	157.019695	72.109014	144.183518	72.300212	322.760248	119.437454	248.274145
AURKAP1	0	3.479663	0	0.207817	12.09045	1.707821	0	1.65601
AURKAP2	0	0	0	0	0	0	0	0
AURKB	71.404876	48.724898	42.20632	46.360394	51.743895	86.555363	32.885939	79.409397
AURKBP1	0	0	0	0	0	0	0	0
AURKC	0	1.400825	7.481014	0.543129	0	1.627471	0	1.405525
AUTS2	20.171241	33.305657	28.974407	26.765803	5.677109	38.768306	76.240328	21.512905
AVEN	4.349294	23.792627	7.747231	14.545976	21.166981	15.953336	2.669048	14.246582
AVIL	0	0.634094	2.163903	0.227448	0	2.371925	0	0.849871
AVL9	16.158348	35.353878	13.332532	32.891486	77.452879	25.799241	12.591824	46.83416

AVP	0	0	0	0	0	0	0	0
AVPI1	29.101265	70.263947	21.149848	79.29837	45.259028	151.873235	66.911662	152.934148
AVPR1A	0	0	0	0	0	0	0	0
AVPR1B	0	0	0	0.015907	0	0.032593	0	0
AVPR2	0	0	0	0	0	0.43771	0	0
AWAT1	0	0	0	0	0	0	0	0
AWAT2	0	0	0	0	0	0	0	0
AXDND1	0	0	0	0.667178	0	0.048815	0	0
AXIN1	16.058784	42.755903	17.097093	50.503654	37.837375	133.447332	9.027892	91.591477
AXIN2	0	0.787896	0	0.039704	0	0	0	0.162369
AXL	17.641131	15.689454	7.493285	7.724442	127.000139	181.249513	105.870952	134.456961
AZGP1	0	0	0	0	0	0.405529	0	0.344899
AZGP1P1	0	0	0	0	0	0	0	0
AZGP1P2	0	0	0	0	0	0	0	0
AZI2	18.674634	70.995709	37.549631	61.508613	30.339659	42.682284	84.540383	85.833126
AZIN1	162.949885	230.832358	162.382883	160.029058	186.833723	71.040955	116.467258	176.659924
AZIN2	0	4.033868	4.529442	2.239975	0	1.40652	0	1.398404
AZU1	0	0	0	0	0	0	0	0
AZU1P1	0	0	0	0	0	0	0	0
B2M	1796.85697	1182.39473	2168.27309	1222.79325	2122.14294	682.699347	1073.66916	1510.82555
B3GALNT1	16.854868	72.077617	9.725873	44.441683	24.855269	53.911735	0	76.89158
B3GALNT1P1	0	0	0	0	0	0	0	0
B3GALNT2	31.48511	48.078624	32.133383	32.595611	38.772801	27.117737	24.447839	42.081957
B3GALNT2P1	0	0	0	0	0	0	0	0
B3GALT4	5.113215	1.78219	1.859508	2.652969	5.886147	5.450758	0	7.587459
B3GALT5	0.242711	0.251412	0	0.196201	0	0.085464	0	0.072976
B3GALT6	8.416709	13.886634	9.691098	14.323015	7.297986	29.585181	0	25.356806
B3GALT9	19.200894	35.229737	9.502714	18.938408	14.612874	5.685926	0	28.413383
B3GAT1	0.944146	0.220683	0	0.04058	0.149955	0.141368	0	0.151758
B3GAT2	0	0	0	0	0	0.031433	0	0.10071
B3GAT3	4.969934	22.079787	13.255925	23.302345	11.303927	35.454691	0	17.153934
B3GAT3P1	0	0	0	0	0	0	0	0
B3GLCT	3.127389	4.489647	2.105555	2.214814	8.476761	2.189203	4.948006	2.731061

B3GNT2	16.792415	41.452023	22.814328	30.705137	7.383029	22.756768	14.256278	38.461834
B3GNT2P1	0	0	0	0	0	0	0	0
B3GNT3	0	0	0	1.887091	0	9.756022	0	0
B3GNT4	0	0.83839	0	3.558494	0	2.100389	0.298308	4.808815
B3GNT5	32.175312	63.986935	58.877096	62.597802	55.837256	31.297281	5.082304	61.73106
B3GNT6	0	0	0	0.09995	0	0	0	0
B3GNT7	0.939363	1.245315	0.842496	2.681399	2.480137	4.381244	0	1.585347
B3GNT8	0	1.024138	0	0.678736	0	2.94779	0	1.3551
B3GNT9	12.515633	24.67955	14.566703	24.795028	2.642008	24.278536	11.241918	22.784567
B3GNTL1	0	0.472817	12.475963	1.492169	0	1.076834	0	0.319566
B3GNTL1P1	0	0	0	0	0	0	0	0
B3GNTL1P2	0	0	0	0	0	0	0	0
B4GALNT1	20.260032	70.048376	15.031247	79.189948	12.13072	70.697304	0	49.413441
B4GALNT2	0	0	0	0.624675	0	0	0	0
B4GALNT2P1	0	0	0	0	0	0	0	0
B4GALNT3	1.808962	1.461779	1.614447	2.08957	0.377873	6.662463	0	4.631991
B4GALNT4	38.205163	11.498027	9.514822	20.408611	27.725024	29.362845	11.179861	29.882226
B4GALT2	19.476235	24.252096	2.452149	23.118594	35.605	45.230526	0	23.365687
B4GALT3	54.0517	117.162804	65.928565	121.86721	14.985903	49.91266	0	41.853121
B4GALT4	42.875452	157.406734	73.045139	156.153411	56.130349	79.328904	42.486562	107.057079
B4GALT5	72.92297	60.139677	79.216912	45.673139	56.135259	41.468197	40.591522	45.936522
B4GALT6	7.192162	12.737931	11.42076	5.285936	12.12943	2.317234	5.105276	3.863357
B4GALT7	24.628412	33.122111	16.691887	31.181187	3.290129	44.657593	0	34.083358
B4GAT1	15.557429	28.241322	13.890878	24.145094	6.38016	23.349025	0	14.297349
B9D1	34.47707	27.5961	0.86481	11.984636	45.171771	15.134398	35.978051	27.057311
B9D2	4.743832	12.417207	0	8.849323	0	15.381633	56.85053	10.213352
BAALC	0	0	0	0	0	0.119141	0	0
BAAT	0	0	0	0.018526	0	0	0	0
BABAM1	57.715278	77.977357	38.955881	80.021399	57.559416	172.010984	51.442355	125.913236
BABAM2	7.83917	23.170417	35.649904	14.710052	10.075553	15.88985	0	3.677996
BACE1	2.536058	4.097135	8.027105	5.965108	26.161878	18.989787	4.131136	23.866096
BACE2	44.206078	48.220794	52.509036	56.7455	93.381755	63.086079	49.964702	46.51749
BACH1	23.199908	70.427128	43.79321	86.834119	72.811332	131.867269	102.210845	430.458006

BACH2	0	0.446853	0	0.895273	10.360523	1.406811	0	3.453498
BAD	0	15.131144	0	17.116247	63.671058	42.388376	143.055351	42.993174
BAG1	10.531226	37.643234	11.756467	35.744192	61.166667	200.821973	0	166.752485
BAG1P1	0	0	0	0	0	0	0	0
BAG2	6.823014	12.034712	6.135182	7.410439	3.219542	5.280899	0	9.487768
BAG3	0	5.995356	5.567739	5.777201	27.292217	9.02205	0	2.905297
BAG4	30.837142	24.298693	39.228743	30.843743	29.240838	21.031707	27.454995	28.943394
BAG5	0	0	0	0	15.288736	7.655764	0	4.394922
BAG6	141.969534	95.403739	115.067391	124.541879	277.700343	135.968144	116.017667	115.562997
BAGE2	0	0	0	0	0	0	0	0
BAHCC1	7.116284	5.364426	4.542881	5.379423	0	7.987747	5.254758	11.544473
BAHD1	14.66213	18.595454	13.070047	18.077078	19.203465	31.110193	13.005222	20.948783
BAIAP2	54.578686	33.320051	19.607909	26.274867	9.22488	51.63974	33.229378	30.696169
BAIAP2L1	87.861073	116.274217	70.95107	146.495167	31.79815	77.328911	92.461816	82.341613
BAIAP2L2	0	0	0	0	0	0	0	0
BAIAP3	9.75648	0.218059	0.883795	2.267473	0	0.252181	0	0.115024
BAK1	0	13.927735	0	10.157454	39.333322	27.127521	0	27.18662
BAK1P1	0	0	0	1.089562	0	0	0	0.298011
BAK1P2	0	0	0	0	0	0	0	0
BAMBI	2.090974	0.880845	0	1.165555	0	1.442622	0	1.267824
BANF1	478.544442	616.109177	406.540263	610.511578	351.442083	494.811985	349.263199	434.742513
BANF1P1	0	0	0	0	0	0	0	0
BANF1P2	0	0	0	0	0	0	0	0
BANF1P3	0	6.229151	0	2.350302	0	6.239696	0	1.489121
BANF1P4	0	0	0	0	0	0	0	0
BANF1P5	0	0	0	0	0	0	0	0
BANF2	0	0	0	0	0	0	0	0
BANK1	3.19734	6.696295	0	5.922095	0	0.646651	0	0.526484
BANP	4.324836	16.962847	14.077584	21.804152	11.418371	18.052281	50.370555	26.77711
BAP1	19.374401	57.470919	19.130368	59.690976	25.965684	92.785723	41.994269	90.496409
BARD1	78.940851	27.310881	38.394332	11.779364	23.27018	9.267142	10.879641	20.300423
BARHL1	0	0	0	0	0	0	0	0
BARHL2	0	0	0	0	0	0	0	0

BARX1	47.027232	47.626513	33.798968	41.388205	13.83025	14.382617	0	15.527976
BARX2	0	0	0	0	0	0	0	0
BASP1	152.169725	319.646111	157.565866	346.076376	110.119022	287.142857	117.380233	207.370388
BASP1P1	0	0	0	0	0	0	0	0
BATF	0	0	0	0	7.583727	0.677989	0	0
BATF2	0	1.669185	1.593626	2.481379	0	1.989516	0	1.080834
BATF3	0	2.358361	0	3.11212	0	3.47892	0	2.376858
BAX	50.817062	207.078254	108.080752	159.503844	102.49742	282.975576	0	173.045351
BAZ1A	46.855636	35.04292	53.108699	30.972724	42.948871	39.999733	85.776131	48.975106
BAZ1B	49.410443	49.38491	52.273958	53.316671	51.375827	60.604855	27.830532	57.187305
BAZ2A	74.123464	96.19705	33.071232	117.899834	96.359365	84.871502	28.688994	77.189378
BAZ2B	7.584497	32.865505	5.041447	30.172922	23.89693	36.618641	26.114207	25.877305
BBC3	5.753805	5.711951	0	8.659445	0	12.446968	0	4.049187
BBIP1	6.98722	45.960374	16.766683	33.056994	19.486831	19.237046	19.30888	31.629503
BBIP1P1	0	0	0	0	0	0	0	0
BBLN	24.222922	33.645642	79.158175	43.415593	22.167969	43.930939	5.725982	27.050856
BBLNP1	0	0	0	0	0	0	0	0
BBLNP2	0	0	0	0	0	0	0	0
BBOF1	6.455802	9.222864	12.503325	8.107077	0	3.962177	40.703251	1.514305
BBOX1	0	0	0	0	1.89167	0.109805	0	0.631906
BBS1	33.801983	21.226635	37.930876	18.80622	9.070625	14.713072	6.978691	12.733475
BBS10	5.587882	4.461124	3.341262	2.794882	0.737677	1.115731	2.264984	4.977041
BBS12	4.127734	4.319498	1.850069	3.875091	0.272408	2.31542	0	3.141813
BBS2	0	18.815686	0	3.861401	19.414415	5.642828	48.745959	12.399599
BBS4	0	11.955843	27.081535	13.582683	4.005678	6.092841	19.134574	5.726813
BBS5	3.234526	3.707318	1.932465	3.157642	1.707542	3.439664	0	3.307125
BBS7	17.564623	18.096544	15.099511	13.713222	27.345888	10.797432	0	13.345484
BBS9	33.048707	7.517458	6.423862	3.671957	12.111279	5.287649	0.848564	17.897471
BBX	78.898298	270.770778	83.19228	370.427031	190.424802	306.86969	128.734152	323.101634
BCAM	17.814225	12.008582	2.102532	10.038414	58.218762	33.813399	49.111723	15.470638
BCAN	1.324677	0.37655	6.537708	1.431703	0	0	0	0
BCAP29	40.333691	29.5712	44.394492	11.069817	0	4.893991	27.552828	16.440106
BCAP31	99.174796	175.718927	142.339449	162.979706	129.754076	106.278489	149.826352	147.50109

BCAP31P1	0	0	0	0	0	0	0	0
BCAP31P2	0	0	0	0	0	0	0	0
BCAR1	62.418088	66.512765	62.468038	76.880813	25.205509	246.359467	67.223861	150.057707
BCAR1P1	0	0	0	0	0	0	0	0
BCAR1P2	0	0	0	0	0	0	0	0
BCAR3	174.957881	365.046111	168.521719	383.64421	56.618401	151.089332	118.245743	185.015288
BCAS1	3.333737	1.942662	1.991365	2.13236	0	0	0	0
BCAS2	75.467814	42.628626	55.410107	30.862641	81.293331	17.635493	56.624628	42.319124
BCAS2P1	0	1.947641	0	0.496803	0	0	0	0
BCAS2P2	0	0	0	0	0	0	0	0
BCAS2P3	0	0	0	0	0	0	0	0
BCAS3	29.917338	5.250432	0	5.11728	55.640455	2.669532	0	17.979643
BCAS4	31.204056	17.152237	0	15.251459	16.959047	26.186418	1.15684	14.045272
BCAT1	97.177428	177.89497	93.586931	177.716373	29.78101	27.66749	62.053262	45.658027
BCAT2	23.167442	24.823469	32.2812	35.167217	74.123568	73.801506	108.19114	47.132314
BCCIP	169.879664	188.987729	131.105098	112.475607	104.962098	86.033459	203.515177	107.912245
BCDIN3D	4.144015	4.757119	1.857324	5.490023	6.837006	2.014521	0	2.326228
BCHE	9.932274	3.798771	2.538247	1.722726	0	0	0	0.400094
BCKDHA	8.090179	8.271924	16.428384	12.749662	8.204569	14.383692	10.266532	9.487241
BCKDHB	14.382645	6.181927	11.404795	5.296253	5.48413	2.69595	28.006232	3.509795
BCKDK	36.500847	63.750782	40.34832	68.974611	25.660922	101.668102	79.532416	84.037492
BCL10	64.265215	70.497558	70.136705	43.560762	26.277779	13.9783	44.033186	39.011281
BCL11A	10.602795	32.914146	11.700505	42.783672	4.651432	31.455543	6.704222	24.694305
BCL11B	1.888656	4.393929	0.764267	2.599792	2.613053	14.99612	0.460764	15.231374
BCL2	0.941243	1.680776	0.586552	0.504378	0.918657	0.628432	0	1.777627
BCL2A1	0	0	0	2.640907	1.383895	0.251581	0	1.95453
BCL2L1	71.97138	227.565041	97.649133	280.31349	63.080645	196.764168	87.207611	177.033365
BCL2L10	0	1.397851	0	0.555187	0	1.010912	0	0.120125
BCL2L11	0	10.747412	10.947914	12.803112	0	9.283187	18.488926	18.750934
BCL2L12	37.992012	4.597828	12.936245	10.885739	66.502808	37.457984	0	21.317062
BCL2L12P1	0	0	0	0	0	0.27279	0	0
BCL2L13	38.723776	30.032353	39.448286	40.9313	22.690823	67.653842	97.632054	66.137272
BCL2L14	0	1.710449	3.374582	9.319481	0	0.823341	0	0.448486

BCL2L15	0	0	0	0	0	0	0	0
BCL2L2	0	0.97212	0	1.740191	0	1.461735	0	0
BCL2L2-PABI	10.190098	30.412552	18.15072	31.058535	14.18599	40.433856	46.810169	33.807319
BCL3	9.306527	8.394544	14.946043	9.448574	13.24595	16.503177	70.829343	14.836806
BCL6	17.968846	23.031094	23.902138	24.90174	2.131891	23.79525	5.825778	7.885622
BCL6B	0	0	0	0	0	0	0	0
BCL7A	4.678474	8.385199	1.474065	8.42406	5.611275	19.192144	2.50187	8.995738
BCL7B	21.671709	67.757775	35.500302	48.725855	64.133418	54.403679	36.252196	58.591777
BCL7C	6.114887	24.969489	5.042741	16.596499	1.309373	14.495171	14.608539	12.537956
BCL9	7.212589	42.193657	25.47252	35.096379	40.234996	45.391369	7.29249	33.189573
BCL9L	295.488826	1053.50686	491.207262	1404.61886	67.833414	376.148389	295.735175	229.28442
BCL9P1	0	0	0	0	0	0	0	0
BCLAF1	70.449934	102.693303	46.732781	81.356275	133.301398	94.047961	145.46646	145.113598
BCLAF1P1	0	0	0	0	0	0	0.297962	0.049555
BCLAF1P2	0	0	0	0	0	0	0	0
BCLAF3	0	0	0	0	10.430959	0	0	0.622938
BCO1	0	0.079561	0	1.098573	0	0.656124	0	0.672862
BCO2	0	1.702416	0	1.165197	2.622038	2.032334	0	0.706818
BCOR	12.453338	32.580365	15.394361	25.924052	23.076406	33.700268	8.346538	24.384278
BCORL1	5.102725	27.549968	11.292524	37.127001	1.657887	38.127647	0	24.534065
BCR	39.350952	86.931404	10.414501	65.062612	48.04924	156.739258	68.387765	67.454207
BCRP3	0	0	0	0	0	0	0	0
BCRP4	0	0	0	0	0	0	0	0
BCRP5	0	0	0	0	0	0	0	0
BCRP6	0	0	0	0	0	0	0	0
BCRP7	0	0	0	0	0	0	0	0
BCRP8	0	0	0	1.496838	0	0	0	0
BCRP9	0	0	0	0	0	0	0	0
BCS1L	17.147417	21.254099	44.466095	15.69009	23.091859	23.552778	22.149863	27.78497
BDH1	27.933248	126.667938	30.832674	94.831529	13.739162	190.461895	59.513704	125.643267
BDH2	8.090179	8.11116	9.278742	12.612299	2.255418	3.099244	0.488882	8.218284
BDH2P1	0	0	0	0	0	0	0	0
BDKRB1	14.103861	7.500966	7.50943	15.075238	0	10.081089	0	7.608901

BDKRB2	3.650568	7.258857	1.843237	11.388317	5.311546	16.338929	1.305322	13.735055
BDNF	1.658304	5.40856	0	2.74362	1.158391	7.309128	0	10.174811
BDP1	21.502569	16.017028	28.189984	15.449769	36.263706	14.238953	45.065062	15.350218
BDP1P	0	0	0	0	0	0	0	0
BEAN1	0	0.067617	1.233861	2.729701	0	0	0	0
BECN1	76.696467	148.373474	29.248472	152.378874	85.083001	145.62361	94.616486	147.466091
BECN1P2	0	0	0	0	0	0	0	0
BECN2	0	0	0	0	0	0	0	0
BEGAIN	0	0.720489	0	0.936511	0	1.035219	0	0.428487
BEND2	0	0	0	0	0	0	0	0
BEND3	0	0	0	0	0	0.34641	0	0.534859
BEND3P1	0	0	0	0.130405	0	0.088528	0	0.216786
BEND3P2	0	0	0	0	0	0	0	0
BEND3P3	0	0	0	0	0	0.138738	0	0
BEND4	0	0	0	0.018275	0	0	0	0.014957
BEND5	0	0	0	0.592162	0	0.411644	0	0
BEND6	0	2.482513	0	0.422275	2.074348	1.805893	7.995122	1.261496
BEND7	1.49136	1.937072	0	1.620972	0	1.750861	0	0.159444
BEND7P1	0	0	0	0	0	0	0	0
BEST1	0.77272	1.588024	5.378921	1.926986	3.467294	9.072991	7.897169	1.704489
BEST2	0	0.386318	0	0	0	0	0	0
BEST3	0	0.815737	0	0.351445	0	0.174717	86.051067	5.724843
BEST4	0	0	0	0.032947	0	0.134513	0	0
BET1	21.8648	12.333224	21.606131	16.801904	7.878853	10.824075	0	18.137812
BET1L	0	16.38236	0	18.234836	9.087124	21.230384	70.024195	18.413614
BET1P1	0	0	0	0	0	0	0	0
BEX1	61.86034	161.68437	54.177196	102.930703	24.304999	132.142202	37.961348	137.593679
BEX2	23.708531	35.831668	56.550923	49.733994	9.491139	45.45663	0	18.198364
BEX3	162.301388	340.122811	117.740925	195.943521	169.329556	162.913751	151.880994	100.025006
BEX4	16.759647	39.296893	34.70775	50.095689	44.09803	32.237343	2.016835	23.969091
BEX5	26.55625	33.719373	27.885679	24.401811	38.942518	22.244728	0	23.357694
BFAR	58.886996	123.940093	59.582702	149.974616	149.629229	113.182577	52.505683	153.219521
BFSP1	1.677097	8.097496	1.497878	6.011142	4.421887	5.704902	0	4.382197

BFSP2	0	0	0	0	0	0	0	0
BGLAP	19.480636	0	0	0	0	0	0	0
BGN	1.504935	0.982613	2.760099	1.837782	0	0.07655	0	0.186708
BHLHA15	0	0	0	0	0	1.42469	0	0.248871
BHLHE23	0	0	0	0	0	0	0	0
BHLHE40	8.218457	12.285044	0	3.176327	23.347863	6.717028	0	2.778893
BHLHE41	10.580145	21.370731	4.76672	29.613959	0	4.759522	0	8.123531
BHMT	0	0	0	0	0	0	0	0
BHMT2	0	0	0	0	0	0	0	0.18851
BICC1	0	5.101801	0	4.388814	0	3.531254	32.405017	3.385055
BICD1	80.771338	36.843647	15.841302	30.545469	6.418384	30.778731	9.945352	46.150163
BICD1P1	0	0	0	0	0	0	0	0
BICD2	29.438649	42.185424	30.73625	33.712905	24.589291	46.62269	36.749655	39.984384
BICDL1	0	4.76517	14.827473	4.121574	5.215206	12.910625	0	10.836832
BICDL2	1.936791	5.821822	1.727284	1.688948	5.438607	0.641714	0	0.923573
BICDL3P	0	0	0	0.39411	0	0	0	0
BICRA	0	3.205285	3.569554	4.880962	18.269506	10.02884	0	5.298794
BICRAL	0	6.957422	0	0	0	0	0	0
BID	0	62.529084	25.809015	90.319275	89.733347	174.877666	22.039674	153.249429
BIK	4.107279	5.912829	28.948936	9.544777	28.029482	6.1385	0	4.697504
BIN1	15.00604	40.660154	25.151648	43.379001	18.757341	60.928479	33.298301	43.507556
BIN2	0	0	0	0	0	0	0	0
BIN2P1	0	0	0	0	0	0	0	0
BIN2P2	0	0	0	0	0	0	0	0
BIN3	0	29.580169	35.70622	39.0905	4.004654	16.859063	0	6.23239
BIRC2	33.472864	208.040286	54.267269	239.650828	30.5944	29.875304	20.004857	31.863566
BIRC3	0	0	0	0.633909	0	0	0	0.67182
BIRC5	58.564575	122.769818	47.62532	62.356472	71.665754	110.684074	54.090277	131.570887
BIRC6	18.301615	24.438793	5.512135	32.018603	97.495353	40.548196	45.290829	25.351416
BIRC6-AS2	0	0	0	0	0	0	0	0
BIRC7	0	0	0	0.136036	0	0	0	0
BIRC8	0	0	0	0	0	0	0	0
BIVM	7.868044	30.950559	5.807385	15.435556	4.10991	7.107091	13.003628	17.292058

BIVM-ERCC5	0	0	12.911201	0	0	1.196265	0	0
BLACAT1	2.513348	2.313418	0.451956	2.54504	0.66412	2.164557	0	1.471207
BLCAP	317.068871	468.252886	331.809996	424.336054	162.873304	212.257896	199.281026	293.0551
BLK	0	0	0	0	0	0	0	0
BLM	7.181206	13.693908	33.174601	6.89926	43.278135	9.279977	5.474986	13.346952
BLMH	31.449089	28.479519	21.235264	25.511984	49.752909	51.305594	62.119112	52.346205
BLNK	0	2.098526	0	0	1.862212	0	0	0
BLOC1S1	18.975003	110.001154	93.25573	110.842174	0	42.223883	0	16.779235
BLOC1S2	57.081178	97.20143	60.092654	36.600895	35.827043	45.323918	124.734301	75.654435
BLOC1S2P1	0	0	0	0	0	0	0	0
BLOC1S3	0	0	0	1.276792	1.503571	1.399471	0	0.946581
BLOC1S5	12.733041	18.532588	13.136416	34.626185	39.941235	49.585482	9.128561	12.983387
BLOC1S5-TX	0	3.053815	0	6.191551	0	0	0	4.770686
BLOC1S6	21.633667	50.913807	37.973376	53.034253	64.351974	53.104541	73.393241	82.484635
BLOC1S6P1	0	0	0	0	0	0	0	0
BLTP1	0.955573	13.972328	16.881597	17.995309	0	5.988161	25.474255	12.158448
BLTP2	75.965861	86.220982	59.875268	81.105385	64.963881	128.523445	171.384006	125.950785
BLTP3A	12.764429	18.479293	13.301371	15.962439	15.715162	14.550782	16.229748	16.863488
BLTP3B	24.651185	20.674434	10.730366	9.899036	12.786212	21.742907	30.473519	34.705032
BLVRA	35.399012	38.349613	21.90055	32.988407	64.179557	80.693022	131.809867	67.645683
BLVRB	0	13.949242	0	4.015189	0	7.418614	0	0
BLVRBP1	0	8.863362	0	0	0	0	0	0
BLZF1	17.78303	46.289472	19.281379	36.589805	30.200993	25.179507	4.574765	48.240355
BLZF2P	0	0	0	0	0	0	0	0
BMAL1	27.563951	49.954456	4.390954	27.597777	23.306969	29.287102	72.61746	36.886123
BMAL2	177.475018	117.681009	187.088382	95.561855	132.615951	48.053565	88.528231	71.454896
BMERB1	12.864901	57.182219	18.98624	41.722533	3.733567	20.818385	0	12.38464
BMF	10.024018	3.684467	1.29793	4.363207	1.908835	4.678736	0	3.100151
BMI1	31.62998	12.110637	22.122793	8.790049	28.747832	6.027741	78.749739	15.537673
BMI1P1	0	0	0	0	0	0	0	0
BMP1	10.169332	13.511489	14.685849	11.581567	36.283592	14.39349	0	8.036243
BMP10	0	0	0	0	0	0	0	0
BMP2	1.875372	13.621749	5.887004	16.74615	7.674726	25.788756	0	28.240365

BMP2K	0	6.774417	0	4.424183	10.738937	3.583149	35.344333	2.156163
BMP2KL	0	0	0	0	0	0	0	0
BMP4	6.215197	12.054955	1.846221	8.958554	7.328336	31.72597	12.51585	32.274413
BMP5	0	0	0	0	0	0	0	0.100294
BMP6	4.377206	13.414046	3.927257	13.439421	0.231164	0.743359	0.212977	0.632992
BMP6P1	0	0	0	0	0	0	0	0
BMP7	2.017367	3.519358	8.141456	1.696816	0	0.082102	0	0.244058
BMP8A	1.156166	0.411045	0	0.454524	0	0.097932	0	0.119096
BMP8B	1.357892	3.218627	3.659014	4.824886	0.538032	2.807976	0	2.284908
BMPER	0	0	0	0	0	1.029513	0	0.138035
BMPR1A	0	2.540462	0	2.794891	0	2.773486	0	3.408675
BMPR1AP1	0	0	0	0	0	0	0	0
BMPR1AP2	0	0	0	0	0	0	0	0
BMPR1B	2.107753	1.296132	1.877938	0	0	0.805659	0	0
BMPR2	0	0	0	0.874716	0	0	0	0.898223
BMS1	27.87603	18.408348	24.299424	21.69001	36.286879	23.945647	27.60408	23.447969
BMS1P1	0	0.333885	0	0.336638	0	0	0	0
BMS1P10	0	0	0	1.08421	0	1.422876	0	1.009199
BMS1P11	0	0	0	0.722641	0	0.596814	0	0.235408
BMS1P12	0	0.483186	0	0.977783	0	3.421763	0	1.189945
BMS1P13	0	0	0	0	0	0	0	0
BMS1P15	0	1.548822	0	0	0	0.537956	0	0.369963
BMS1P16	0	0	0	0	0	0	0	0
BMS1P17	0	0	0	0	0	0	0	0
BMS1P19	0	0	0	0	0	0	0	0
BMS1P2	0	1.992842	0	2.559305	10.823967	0	0	0
BMS1P20	29.740126	68.179046	32.705322	57.049622	57.446466	91.327196	37.108064	96.256347
BMS1P21	0	0	0	0	0	0	0	1.453524
BMS1P22	0	0	0	0	0	0	0	0
BMS1P23	0	0	3.223425	0	0	0	37.954494	0.942192
BMS1P3	0	0	0	0	0	0.405627	0	0.688473
BMS1P4	0	1.08808	0	0	0	0	0	0
BMS1P7	0	2.595804	3.206301	3.125387	0	0	7.825523	1.873498

BMS1P8	0	0.138904	0	0.156175	0	0	0	0
BMS1P9	0	0	0	0	0	0	0	0
BMT2	11.654068	8.729097	2.258964	7.231116	1.772633	0.75518	3.675135	8.715572
BMX	0	0	0	0.803225	0	0	0	0
BNC2	0.991987	3.208342	0	0.642801	0	0.887329	0	1.444505
BNIP1	0	1.045488	0	1.332648	0	0.696451	2.775382	0.135491
BNIP2	0	141.909546	55.73206	196.381987	25.67317	156.987972	23.913108	93.861168
BNIP3	0	0	0	0.146144	0	0	0	0
BNIP3L	97.222095	98.112768	147.836543	117.535975	106.084984	94.429128	128.066992	116.676309
BNIP3P1	0	0	0	0	0	0	0	0
BNIP3P10	0	0	0	0	0	0	0	0
BNIP3P11	0	0	0	0	0	0	0	0
BNIP3P13	0	0	0	0	0	0	0	0
BNIP3P14	0	0	0	0	0	0	0	0
BNIP3P15	0	0	0	0	0	0	0	0
BNIP3P16	0	0	0	0.209483	0	0	0	0
BNIP3P17	0	0	0	0	0	0	0	0
BNIP3P18	0	0	0	0	0	0	0	0
BNIP3P19	0	0	0	0	0	0	0	0
BNIP3P2	0	0	0	0	0	0	0	0
BNIP3P20	0	0	0	0	0	0	0	0
BNIP3P22	0	0	0	0	0	0	0	0
BNIP3P23	0	0	0	0	0	0	0	0
BNIP3P24	0	0	0	0	0	0	0	0
BNIP3P25	0	0	0	0	0	0	0	0
BNIP3P26	0	0	0	0	0	0	0	0
BNIP3P27	0	0	0	0	0	0	0	0
BNIP3P28	0	0	0	0	0	0	0	0
BNIP3P29	0	0	0	0	0	0	0	0
BNIP3P3	0	0	0	0	0	0	0	0
BNIP3P30	0	0	0	0	0	0	0	0
BNIP3P31	0	0	0	0	0	0	0	0
BNIP3P32	0	0	0	0	0	0	0	0

BNIP3P33	0	0	0	0	0	0	0	0
BNIP3P34	0	0	0	0	0	0	0	0
BNIP3P35	0	0	0	0	0	0	0	0
BNIP3P36	0	0	0	0	0	0	0	0
BNIP3P37	0	0	0	0	0	0	0	0
BNIP3P38	0	0	0	0	0	0	0	0
BNIP3P39	0	0	0	0	0	0	0	0
BNIP3P4	0	0	0	0	0	0	0	0
BNIP3P40	0	0	0	0	0	0	0	0
BNIP3P41	0	0	0	0	0	0	0	0
BNIP3P42	0	0	0	0	0	0	0	0
BNIP3P43	0	0	0	0	0	0	0	0
BNIP3P44	0	0	0	0	0	0	0	0
BNIP3P45	0	0	0	0	0	0	0	0
BNIP3P46	0	0	0	0	0	0	0	0
BNIP3P47	0	0	0	0	0	0	0	0
BNIP3P5	0	0	0	0	0	0	0	0
BNIP3P6	0	0	0	0	0	0	0	0
BNIP3P7	0	0	0	0	0	0	0	0
BNIP3P8	0	0	0	0	0	0	0	0
BNIP3P9	0	0	0	0	0	0	0	0
BNIP5	0	0	0	0	0	0	0	0
BNIPL	0	0.186582	0	0.297701	0	0	0	0
BOC	0	6.169106	0	2.348814	0.75221	3.583838	0	5.610021
BOD1	0	3.531539	0	31.240411	0	1.932013	132.49179	5.928989
BOD1L1	18.133107	7.267591	21.841662	10.421903	10.270752	9.413706	14.891648	8.003302
BOD1L2	0	0	0	0	0	0	0	0
BOD1P1	0	0	0	0	0	0	0	0
BOD1P2	0	0	0	0	0	0	0	0
BOK	10.324064	26.148661	18.487614	25.10543	33.369284	66.293055	21.299057	50.755267
BOLA1	16.901907	23.75344	19.588523	31.965799	13.268933	28.721917	0	22.813271
BOLA2B	0	29.458739	30.40173	91.006748	48.398603	71.739832	0	94.919438
BOLA2P1	0	0	0	0	0	0	0	0

BOLA2P2	0	0	0	0	0	0	0	0
BOLA2P3	0	0	0	0	0	0	0	0
BOLA3	185.63275	77.249168	65.612567	53.90902	53.88407	47.736875	104.834646	87.785869
BOLA3P1	0	0	0	0	0	0	0	0
BOLA3P2	0	0	0	0	0	0	0	0
BOLA3P3	0	0	0	0	0	0	0	0
BOLA3P4	0	0	0	0	0	0	0	0
BOLL	0	0	0	0	0	0	0	0
BOP1	23.87405	39.694775	26.380647	38.49588	36.652233	37.348869	17.713047	32.706079
BORA	0	10.053111	11.364117	4.437179	29.665145	5.866365	15.849145	11.245883
BORCS5	2.020992	35.010654	14.57903	36.097732	13.449621	44.051341	2.832994	39.434103
BORCS7	12.524941	0	8.452507	0.790563	0	0	0	0.692113
BORCS7-ASN	0	0	0	0	0	2.415205	0	0
BORCS8	3.477439	14.566959	0.804574	10.269723	10.515257	20.998335	0	16.729933
BORCS8-MEI	6.009547	9.977211	0	8.434699	37.047374	7.245166	0	7.252212
BORCS8P1	0	0	0	0	0	0	0	0
BPGM	10.317971	16.786876	23.16422	14.077313	2.718527	10.178082	28.924026	18.098008
BPHL	48.037462	29.898133	14.67171	28.459705	23.20507	17.654508	39.061763	26.180247
BPI	0	0	0	0	0	0	0	0
BPIFA1	0	0	0	0	0	0	0	0
BPIFA2	0	0	0	0	0	0	0	0
BPIFA3	0	0	0	0	0	0	0	0
BPIFA4P	0	0	0	0	0	0	0	0
BPIFB1	0	0	0	0	0	0	0	0
BPIFB2	0	0	0	0	0	0	0	0
BPIFB4	0	0	0	0.397487	0	0	0	0
BPIFB5P	0	0	0	0	0	0	0	0
BPIFB6	0	0	0	0	0	0	0	0
BPIFB9P	0	0	0	0	0	0	0	0
BPIFC	0	0	0	0	0	0	0	0
BPNT1	11.601863	55.626217	12.840313	48.45273	23.258161	43.632276	0	34.812269
BPNT2	36.710852	83.746782	62.999079	43.194038	42.028741	25.515178	40.030025	27.015112
BPNT2P1	0	0	0	0	0	0	0	0

BPTF	31.828286	30.181306	61.126195	31.227043	81.579316	31.456548	78.375913	24.181652
BPTFP1	10.175883	1.73485	0	2.197645	0	0.569562	0	2.828262
BPY2C	0	0	0	0	0	0	0	0
BPY2DP	0	0	0	0	0	0	0	0
BRAF	16.028522	7.45995	21.084634	14.752704	41.442626	5.498632	10.519288	11.822679
BRAFP1	0	0	0	0	0	0	0	0
BRAP	18.188713	38.9292	11.871669	32.813578	59.732683	51.270793	25.899682	58.383282
BRAT1	3.643296	6.660797	11.889025	6.422292	35.642864	28.483054	17.366925	22.84908
BRCA1	4.426139	10.916528	0	22.866333	18.007145	16.238319	4.447341	28.778147
BRCA1P1	0	0	0	0	0	0	0	0
BRCA2	0	0	0	2.541523	31.652079	0	0	1.904612
BRCC3	43.570856	24.002513	7.456043	30.053192	50.878103	16.977224	12.613144	26.664583
BRCC3P1	0	0	0	0.198243	0	0.792132	0	0
BRD1	0	11.543388	1.789647	12.445908	6.117988	22.313545	8.142172	15.184729
BRD10	7.158333	12.825831	8.905057	7.742304	21.317853	8.980697	34.673914	12.134693
BRD2	83.280974	327.311913	108.567419	382.810673	158.87485	417.442086	398.633693	364.443567
BRD3	0	7.717127	0	19.798675	33.895392	50.38104	34.345927	5.559232
BRD3OS	29.995796	39.683015	22.636464	43.936118	13.066735	36.368915	5.029101	39.936533
BRD4	69.341474	106.253416	59.983368	121.386887	62.750255	283.670618	166.916278	206.926007
BRD7	124.074886	159.699021	112.121947	138.148402	93.003817	174.991533	132.723328	121.38084
BRD7P1	0	0	0	0	0	0	0	0
BRD7P2	0	0	0	0.101652	0	0.098588	0	0.077101
BRD7P3	0	0	0	0	0	0	0	0
BRD7P4	0	0	0	0	0	0	0	0
BRD7P5	0	0	0	0	0	0	0	0
BRD7P6	0	0	0	0	0	0	0	0
BRD7P7	0	0	0	0	0	0	0	0
BRD8	23.267382	33.174995	20.619208	37.10915	95.245333	37.648139	102.480513	37.913003
BRD9	21.820255	52.553608	31.860915	56.483544	22.021384	61.255498	29.574086	36.106432
BRD9P1	0	0	0	0	0	0	0	0
BRD9P2	0	0	0	0	0	0	0	0
BRDT	0	0	0	0	0	0	0	0
BRDTP1	0	0	0	0	0	0	0	0

BRF1	7.509251	40.878073	6.682832	37.919838	25.280462	94.368975	82.413209	70.515903
BRF2	0	0	10.051724	0.921721	0	0	0	4.348934
BRI3	2.183236	1.630623	8.641384	1.483624	48.398065	4.977981	0.265245	3.013386
BRI3BP	19.822305	25.877623	22.606559	18.186912	24.912308	17.293571	14.852769	28.442937
BRI3BPP1	0	0	0	0	0	0	0	0
BRI3P1	0	0	0	0	0	0	0	0
BRI3P2	0	0	0	0	0	0	0	0
BRI3P3	0	0	0	0	0	0	0	0
BRICD5	0	0	0	0.753303	0	0	0	0
BRINP1	0	0	0	0.308686	0	0.210377	0	1.214793
BRINP2	0	0.138017	0	0.950732	0	0.067772	0	0.097026
BRINP3	0	0	0	0	0	0	0	0
BRIP1	0	0	0	0.799686	0	0	0	3.835287
BRIX1	36.927515	27.89551	38.702241	2.031308	21.270405	0	194.908812	31.246842
BRIX1P1	0	0	0	0	0	0	0	0
BRK1	117.414578	85.129487	112.597122	83.384696	60.020119	43.797361	110.392955	74.410979
BRK1P1	0	0	0	0	0	0	0	0
BRK1P2	0	0	0	0	0	0	0	0
BRME1	0	4.414951	0	2.395367	0	8.93889	0	2.63968
BRMS1	73.690765	222.114493	56.964812	178.405155	43.748139	195.804491	66.08067	150.204227
BRMS1L	21.425153	43.665684	21.043637	24.600451	24.390348	31.405907	22.330782	39.334071
BROX	74.53802	86.324578	83.75153	57.711736	35.458067	36.631602	110.949076	85.817874
BRPF1	0	0	0	2.483312	1.145975	5.842537	0.19371	2.987497
BRPF3	9.130764	7.48692	6.276676	12.150152	5.948171	28.077417	0.197023	14.733323
BRS3	0	0	0	0	0	0	0	0
BRSK1	6.849041	1.311071	1.473035	2.537329	1.800025	0.48997	0	0.731584
BRSK2	1.614144	2.600318	1.476095	4.717315	0.500908	0.161371	0	0.830072
BRWD1	11.815093	14.385966	16.362091	8.68857	37.73534	17.410029	71.380909	22.696034
BRWD1P1	0	0	0	0	0	0	0	0
BRWD1P2	0	0	0	0	0	0	0	0
BRWD1P3	0	0	0	0	0	0	0	0
BRWD3	16.519593	21.931713	8.6877	33.561134	26.188571	21.721515	38.726467	22.114945
BSCL2	12.768687	6.151204	6.560433	5.090392	12.739329	18.906951	0	15.881613

BSDC1	0	7.310205	19.574618	14.085804	49.272409	18.356012	73.194372	13.690009
BSG	22.89028	1.042802	5.529826	48.976199	85.665781	13.336208	85.817278	35.12969
BSN	0.199649	1.09783	0	0.313079	0	1.215795	0	0.703306
BSND	0	0.085127	0.296192	0.056404	0	0.049617	0	0
BSNDP1	0	0	0	0	0	0	0	0
BSNDP2	0	0	0	0	0	0	0	0
BSNDP3	0	0	0	0	0	0	0	0
BSNDP4	0	0	0	0	0	0	0	0
BSPH1	0	0	0	0	0	0	0	0
BSPRY	0	4.99372	5.25726	10.081638	0	6.56399	0	5.389394
BST1	0	0	0	0.255672	0	0	0	0.444256
BST2	0	0	0	0.092515	26.568364	24.419504	0	10.382092
BTAF1	0	0	0	1.264916	3.111815	0	0	0
BTBD1	17.775918	34.373795	45.824869	25.935879	0	24.772824	0	58.818024
BTBD10	30.759535	29.251652	18.828289	39.263368	1.902328	37.085633	81.121497	41.98344
BTBD10P1	0	0	0	0	0	0	0	0
BTBD10P2	0	0	0	0	0	0.301125	0	0
BTBD16	0	0	0	0	0	0	0	0
BTBD17	0	0.258253	0	0	0	0	0	0
BTBD18	0	0	0	0	0	0	0	0
BTBD19	0	0	1.139839	0.733561	0	0	0	0
BTBD2	0	1.838763	0	10.894271	85.935292	0.603359	14.318422	0.270668
BTBD3	9.800806	55.667582	25.093089	54.052105	57.269402	145.397558	38.666674	165.352054
BTBD6	0	1.563409	0	2.483097	0	14.322864	0	11.873116
BTBD6P1	0	0	0	0	0	0	0	0
BTBD7	107.042126	243.367822	47.811162	273.729153	95.36747	233.396043	51.142502	181.078301
BTBD7P1	0	2.393622	0	1.741223	0	0	0	0
BTBD7P2	0	0	0	0	0	0	0	0
BTBD8	4.37739	6.304112	5.410298	0	1.456831	1.413586	0	4.32586
BTBD9	3.493665	12.098949	3.482889	10.122423	7.828538	11.675103	3.149015	7.678209
BTC	2.552796	0.543436	1.142589	1.132656	0	0.634542	0	0.61654
BTD	4.708129	0	0	0.975456	0	5.899946	0	2.612159
BTF3	1015.84003	760.446241	818.474295	625.789087	716.93169	498.441488	698.643292	776.601547

BTF3L4	173.887796	135.639579	208.179332	96.4621	111.771804	41.238004	54.668505	46.672025
BTF3L4P1	0	0	0	0	0	0	0	0
BTF3L4P2	168.539129	143.419144	175.481199	99.18228	49.922816	18.003159	91.009536	78.459077
BTF3L4P3	0	0	0	0	0	0	0	0
BTF3L4P4	0	0	0	0	0	0	0	0
BTF3P1	0	0	0	0	0	0	0	0.424645
BTF3P10	0	0	0	0.234188	0	0	0	0
BTF3P11	0	0	0	0	0	0	0	0
BTF3P12	0	0	0	0	0	0	0	0
BTF3P13	0	0	0	0	0	0	0	0
BTF3P14	0	0	0	0	0	0	0	0
BTF3P15	0	0	0	0	0	0	0	0
BTF3P16	0	0	0	0	0	0	0	0
BTF3P2	0	0	0	0	0	0	0	0
BTF3P3	0	0	0	0	0	0	0	0
BTF3P4	0	0.385687	0	0	0	0	0	0
BTF3P5	0	0	0	0	0	0	0	0
BTF3P6	0	0	0	0	0	1.811248	0	0
BTF3P7	0	0	0	0.239175	0	0	0	0
BTF3P8	0	0	0	0	0	0	0	0
BTF3P9	0	0	0	0	0	0	0	0
BTG1	27.65232	76.128946	101.252518	84.92516	127.658041	102.734971	50.744894	101.643694
BTG1P1	0	0	0	0	0	0	0	0
BTG2	26.004037	19.516513	21.065554	21.970652	27.120747	24.170318	34.040418	14.376443
BTG3	0	7.489765	0	12.666964	0	24.957916	41.033857	21.73972
BTG3P1	0	0	0	0	0	0	0	0
BTG4	0	0	0	0	0	0	0	0
BTG4P1	0	0	0	0	0	0	0	0
BTK	0	0	0	0.029718	0	0	0	0
BTLA	0	0	0	0	0	0	0	0
BTN1A1	0	0	0	0	0	0	0	0
BTN1A1P1	0	0	0	0	0	0	0	0
BTN2A1	5.921577	19.355657	6.364282	25.036797	8.70196	51.434061	7.598365	33.657033

BTN2A2	4.945379	16.000224	3.320958	15.79761	22.710212	28.082846	0	15.756358
BTN2A3P	0	0.078996	0	0.613603	0	1.39055	0	0.834263
BTN3A1	13.779307	19.563448	2.707887	25.834967	5.901974	31.242603	1.45191	28.357134
BTN3A2	3.614194	13.022277	19.375856	24.208888	50.379768	41.50828	0	29.220181
BTN3A3	10.622567	11.060095	19.884246	9.738913	2.062603	11.681031	4.297386	10.009021
BTNL10P	0	0	0	0	0	0	0	0
BTNL2	0	0	0	0	0	0	0	0
BTNL8	0	0	0	0.211882	0	0	0	0
BTNL9	0	0.205825	0	0.634199	0	0.215745	0	0.154786
BTRC	16.730041	29.587047	12.704431	25.672697	29.821654	18.970211	7.949504	31.8278
BUB1	32.847883	41.299568	38.118425	24.264606	49.307627	58.563893	50.491489	90.67438
BUB1B	26.894155	32.944197	0	84.321734	64.696267	144.068608	1.648792	63.864114
BUB1B-PAK6	0	0	16.664031	0.644619	35.558002	0	0	0.795209
BUB1P1	0	0	0	0	0	0	0	0
BUB3	222.228788	367.655361	193.462526	330.375095	170.532401	321.839739	204.294803	354.280726
BUB3P1	0	0	0	0	0	0	0	0
BUD13	36.095659	87.845521	54.69843	106.923867	24.79673	59.739777	100.237638	40.807179
BUD13P1	0	0	0	0	0	0	0	0
BUD31	61.537338	167.418872	100.980732	176.741925	129.377653	203.952852	35.544613	213.257839
BUD31P1	0	0	0	0	0	0	0	0
BUD31P2	0	0	0	0	0	0	0	0
BVES	0.587347	0.757037	2.111343	1.220364	0.630468	0.382612	0	0.259146
BYSL	30.8127	23.294123	23.799883	23.37891	27.602957	25.621875	82.409246	27.111526
BZW1	159.057058	380.964215	247.401111	273.964544	206.455746	381.128506	830.644116	654.092717
BZW1P1	0	0	0	0	0	0	0	0
BZW1P2	111.116935	119.483023	94.550923	60.886156	170.922991	54.407275	0	261.813621
BZW2	112.766639	201.320691	102.345145	200.757796	156.899922	148.867723	100.191094	201.511829
C10orf105	0	0	0	0.033877	0	0	0	0
C10orf120	0	0	0	0	0	0	0	0
C10orf143	4.779802	0.58371	0	0.34222	0	0.457563	0	0.391834
C10orf53	0	0	0	0	0	0	0	0
C10orf55	1.353111	0	0	0	0	0	0	0
C10orf67	0	1.861807	0	0	2.347246	0	0	0

C10orf71	0	0	0	0	0	0	0	0
C10orf88	18.076713	3.818133	7.241222	9.37252	0.60955	2.901127	5.048998	6.793086
C10orf88B	0	3.059781	0	1.199464	0	1.119062	0	1.517608
C10orf90	0	0	0	0	0	0	0	0
C10orf95	0	0.205632	0	0	0	0.240726	0	0
C11orf16	0	0	0	0	0	1.149213	0	0
C11orf21	0	0	0	0	0	0	0	0
C11orf24	66.612872	143.173738	45.950675	165.361178	42.986682	132.071046	23.855348	121.386729
C11orf40	0	0	0	0	0	0	0	0
C11orf52	0	14.621329	9.085889	9.803224	23.606762	4.08056	0	3.827581
C11orf54	23.17866	32.263354	25.017782	52.678058	0	4.250213	0	23.736922
C11orf58	139.373217	602.6424	163.966595	497.064757	302.974309	651.310345	292.354359	653.564603
C11orf65	3.992813	2.118166	6.819469	1.10331	0	1.566555	0	1.364691
C11orf68	11.458241	35.671113	35.463304	38.680609	32.721889	30.904586	0	18.075549
C11orf71	1.754584	5.208493	1.566399	2.684182	1.387659	6.917303	0	6.414521
C11orf86	0	0.261102	0	0	0	0	0	0
C11orf91	0	0	0	0	0	0	0	0
C11orf98	0	4.242845	0	0	0	1.869496	0	5.474778
C11orf98P1	0	0	0	0	0	0	0	0
C11orf98P2	0	0	0	0	0	0	0	0
C11orf98P3	0	0	0	0	0	0	0	0
C12orf42	0	0	0	0	0	0	0	0
C12orf43	8.953944	25.278184	10.434774	23.853879	19.17166	23.880283	4.65595	21.252053
C12orf50	0	0.483733	0	0.114809	0	0	0	0.034165
C12orf54	10.241224	3.855519	0	4.797412	0	6.377104	0	3.722884
C12orf56	0	0	0	0.305269	0	0.202041	0	0.348753
C12orf57	136.926592	82.153206	90.961273	85.809258	41.24912	87.757103	0	84.339945
C12orf60	0	4.441665	2.348042	4.199941	0.309643	0.116821	0	0.767467
C12orf71BP	0	0	0	0	0	0	0	1.557573
C12orf75	44.532584	26.236928	0	15.240254	136.622443	33.709831	89.719688	51.664433
C12orf76	2.005813	1.86099	0	1.463872	0	0.494702	0	2.222826
C13orf42	0	0	0	0	0	0	0	0
C13orf46	0	0	0	0.65498	0	0	0	0

C14orf119	92.721641	118.282003	52.601614	120.260615	44.661445	88.390104	83.558148	114.377138
C14orf132	0	0.613932	0	0	0	1.673415	0	0
C14orf178	0	0	0	0	0	0	0	0
C14orf180	0	0	0	0	0	0	0	0
C14orf28	0	5.111148	0	2.959867	0	2.652593	0	1.925201
C14orf39	0	0.817281	0	0	0	1.943673	0	0
C14orf93	23.390323	22.583321	23.297622	27.770009	0	26.392837	18.841515	16.562639
C15orf39	17.835275	25.538323	14.012217	33.304969	14.917028	58.653221	119.995271	43.410183
C15orf40	97.524045	124.692659	51.462236	73.911124	65.813563	94.814621	2.636151	41.169766
C15orf48	0	0	0	0.840352	0	3.607233	0	4.877623
C15orf61	1.572275	8.108786	0.705686	5.034998	0	4.98678	0	4.539752
C16orf46	0	8.635589	0	3.042305	10.855032	5.278394	0	7.481654
C16orf54	0	0	0	0	0	0	0	0
C16orf74	162.882444	227.984251	68.150332	245.040673	55.121042	344.479447	119.892249	292.295203
C16orf86	0	0.232818	0	0.634715	0	0.202416	0	0.956152
C16orf87	23.37312	10.008787	9.594026	21.124705	27.904337	19.686192	4.730294	19.56246
C16orf89	0	0	0	0	0	0	0	0
C16orf90	0	0	0	0	0	0	0	0
C16orf92	0	0	0	0	0	0	0	0
C16orf95	0	3.933558	10.546995	3.053738	0	4.859777	0	5.534784
C17orf100	0	1.949725	0	2.956706	1.316252	2.540763	0	0
C17orf107	0	0.847582	0.93631	4.389581	0	1.781915	0	1.617497
C17orf114	0	0	0	0	0	0	0	0.694549
C17orf49	97.538737	88.582996	47.808735	108.616468	79.031743	125.554115	79.52352	108.247078
C17orf50	0	0	0	0	0	0	0	0
C17orf58	17.148713	45.770957	9.73804	41.031051	14.682165	22.765794	17.661704	31.838323
C17orf67	0	4.320301	3.037377	3.924834	0	5.21849	0	3.636031
C17orf75	34.286593	48.63099	51.890865	35.00722	38.769756	19.695201	0	40.161576
C17orf78	0	0	0	0	0	0	0	0
C17orf99	8.187272	7.261235	0	13.084162	0	6.716279	1.458589	11.492611
C18orf21	18.002786	23.665354	32.279862	16.337111	30.686631	17.756584	14.143635	27.33836
C18orf32	0.645347	0.560315	0	1.789756	14.26805	0.092705	0	5.15881
C18orf54	12.158642	26.496692	8.84139	13.255836	12.231546	16.476125	4.710624	34.281421

C19orf12	0	24.059255	7.772985	15.360377	14.457031	31.65966	10.36488	30.63751
C19orf18	0	0.705844	3.792246	0.751386	0	0.829228	0	1.057241
C19orf25	6.329432	20.288804	13.689866	24.124949	35.951501	39.442277	0	28.441629
C19orf33	253.528556	262.515554	210.442138	266.944397	397.337466	347.077052	333.609575	319.88337
C19orf38	0	0	0	0.086414	0	0	0	0
C19orf44	3.89866	8.15754	5.361735	9.733125	0	10.523161	0	11.010984
C19orf47	6.441165	35.709345	7.068074	17.935906	15.683927	51.123205	12.522423	42.70057
C19orf48P	0	0	0	0	0	0	0	0
C19orf53	141.546616	181.992646	62.169099	208.619792	142.454961	550.548414	72.71073	397.910258
C19orf67	0	0	0	0	0	0	0	0
C19orf73	0	2.045581	0	2.546758	5.907751	4.28275	0	1.858956
C19orf81	0	2.843581	0	6.611108	0	1.448933	0	0.451431
C19orf84	0	0	0	0	0	0	0	0
C1D	0	7.036943	7.442689	8.437397	28.329206	7.257654	0	9.080077
C1DP1	0	0	0	0	0	0	0	0
C1DP2	0	0	0	0	0	0	0	0
C1DP3	0	0	0	0	0	0	0	0
C1DP4	0	0	0	0	0	0	0	0
C1DP5	0	0	0	0	0	0	0	0
C1GALT1	34.658065	88.414898	32.541786	45.703163	11.407367	24.030744	35.231532	65.362294
C1GALT1C1	28.907971	22.667116	30.925698	14.036555	14.589034	3.526165	0	10.355465
C1GALT1C1L	2.877424	2.99978	0	2.429787	0	0.702925	0	1.401436
C1GALT1P1	0	0	0	0	0	0	0	0
C1GALT1P2	0	0	0	0	0	0	0	0
C1GALT1P3	0	0	0	0	0	0	0	0
C1orf105	0	0	3.933971	0.91187	30.061678	0	0	0
C1orf115	3.49258	14.147219	7.299675	18.438082	0.307246	9.563915	0	6.510076
C1orf116	29.266076	69.240017	24.503434	92.336137	43.768863	120.469283	26.14301	104.437283
C1orf122	24.005561	39.413707	13.30058	28.649262	32.358909	50.471048	0	43.66001
C1orf127	0	0	0	0	0	0	0	0
C1orf131	42.954564	25.027511	13.486571	24.219692	5.77679	16.480834	74.697266	26.118835
C1orf141	0	0	0	0	0	0	0	0
C1orf146	0	0	0	0	0	0	0	0

C1orf159	0	0.96234	0	1.319299	16.209857	1.448093	17.766861	2.733953
C1orf162	0	0.377987	0	0	0	0	0	0
C1orf167	0	0.212765	0	0	0	0	0	0
C1orf174	32.351855	65.120702	23.053171	54.714806	9.0906	45.62695	25.005125	67.363831
C1orf185	0	0	0	0	0	0	0	0
C1orf198	0	4.428903	0	6.917922	15.339134	19.272843	0	9.620706
C1orf21	0	11.188137	1.403164	8.401424	8.486184	28.792917	41.778359	49.461007
C1orf210	0	0	0	0	0	0	0	1.943565
C1orf216	12.894521	24.261368	9.233514	19.974222	5.783824	24.737633	20.96879	28.114526
C1orf226	0.800309	3.668779	3.591747	6.356783	2.324903	6.481261	8.261734	5.074244
C1orf232	0	0	0	0	0	0	0	0
C1orf35	14.244083	34.149562	15.165788	40.943775	10.682302	63.896322	23.990177	37.107789
C1orf43	96.258461	97.272292	103.60681	129.996851	69.686448	106.15826	187.637037	76.610585
C1orf50	2.066213	12.802122	0	10.224992	41.086686	6.653316	0	10.680158
C1orf52	33.269821	51.16353	33.804303	56.893766	3.891556	31.062963	56.299344	32.76208
C1orf53	30.248034	2.381067	0	1.717426	0	1.406949	0	2.991833
C1orf54	0	1.968314	0	4.712154	0	1.441541	0	2.144502
C1orf56	0	5.971059	0	3.202242	0	0	0	0
C1orf74	6.303318	6.527278	7.548012	7.427654	7.954808	4.556604	19.102024	7.195736
C1orf87	0	0	0	0	0	0	0	0
C1orf94	0	0	0	0	0	0.239932	0	0
C1QA	0	0	0	0	0	0	0	0
C1QB	0	0	0	0	0	0	0	0
C1QBP	241.359045	369.051875	213.246538	304.187472	419.869953	417.0703	243.394169	426.81327
C1QBPP1	0	0	0	0	0	0	0	0
C1QBPP2	0	0	0	0.351554	0	0	0	0
C1QC	0	0	0	0	0	0	0	0
C1QL1	4.692471	8.176472	8.350436	7.38132	0	1.037701	0	1.139581
C1QL1P1	0	0	0	0	0	0	0	0
C1QL2	0	0	0	0	0	0	0	0
C1QL3	0	0	0	0	0	0	0	0
C1QL4	0	3.533145	0	9.970155	0	1.404116	0	0.538288
C1QTNF1	2.583131	19.979705	6.691436	13.951494	0.915833	7.163132	0	5.583277

C1QTNF12	0	0.716916	0	1.15435	0	1.700061	0	1.926473
C1QTNF2	0	0.243342	0	0	0	0.284299	0	0.057679
C1QTNF3	0	0	0	0	0	0	0	0
C1QTNF3-AI	0	0	0	0	0	0	0	0
C1QTNF4	0	0	0	0	0	0	0	0
C1QTNF5	0	3.943149	0	5.451476	0	4.769444	0	1.087984
C1QTNF6	5.307347	14.907933	9.378414	15.25304	53.315033	18.724892	0	17.839024
C1QTNF7	0	0	0	0	0	0	0	0
C1QTNF9	0	0	0	0	0	0	0	0
C1QTNF9B	0	0	0	0	0	0	0	0
C1R	34.060486	18.867163	32.336475	46.679069	27.266622	37.424754	32.485783	17.344277
C1RL	12.608445	20.542967	26.004994	39.733702	4.752697	22.376647	0	10.757104
C1S	9.435322	15.590822	23.952291	42.529967	15.355796	11.218959	47.30234	8.784479
C2	1.841728	0.386755	0	0.599563	0	0.621837	0	0
C20orf141	0	0	0	0.410658	0	0.546379	0	0
C20orf144	1.52238	0.71069	0	0.518239	0	0.150992	0	0.122715
C20orf173	0	0	0	0	0	0	0	0
C20orf204	0	0.537146	0	0.195598	0	0.226647	0	0
C20orf96	12.746408	2.322983	2.265681	5.376968	0	1.002091	0	0.697775
C21orf58	0	8.397039	4.829625	8.907998	6.181247	31.500858	0	23.101182
C21orf91	4.232902	11.733882	1.086754	5.719156	4.313357	11.181576	27.197406	31.844003
C22orf15	0	0	0	0	0	0	0	0
C22orf23	1.978119	0.083453	0	0.149981	0	0.48802	0	0.239739
C22orf39	14.126524	8.035161	6.771657	11.307731	19.213182	15.874666	33.390935	12.671126
C22orf42	0	0	0	0	0	0	0	0
C22orf46P	8.482778	17.717635	6.448078	19.615872	11.771563	21.380048	5.333221	13.345667
C2CD2	2.784202	10.711844	15.198114	12.099533	14.544044	29.750349	6.139227	21.434444
C2CD2L	0	7.497082	14.865615	8.602988	1.945703	2.406823	3.934839	7.551791
C2CD3	12.315534	5.800479	0	5.876734	78.462812	0	27.710203	8.244705
C2CD4A	0.966437	0.123944	0	0.212173	0	0	0	0.077679
C2CD4B	0	0	0	0	0	0	0	0
C2CD4C	1.081134	4.19948	3.875471	4.638505	0.28537	0.64653	0	0.260849
C2CD4D	0	0	0	0.145725	0	0.395269	0	0

C2CD5	21.495209	22.812035	16.638242	20.655953	43.53197	21.075979	25.322857	21.53463
C2CD6	0	0.073634	0	0	0	0	0	0
C2orf15	0	0	0	0.786174	0	0.390175	0	0.817322
C2orf42	4.705786	14.020224	5.607328	16.883551	6.861696	10.648814	0	9.525822
C2orf49	20.011232	19.040991	18.910015	18.460364	20.143592	12.031712	12.549286	19.80414
C2orf50	0	0	0	0	0	0	0	0.64615
C2orf68	12.975394	59.890691	14.028697	53.921128	26.337847	51.240271	6.752695	40.390543
C2orf69	11.682499	7.113465	8.867887	3.552646	19.694528	6.192759	9.618206	12.060257
C2orf69P1	0	0	0	0	0	0	0	0
C2orf69P2	0	0	0	0	0	0	0	0
C2orf69P3	0	0	0	0	0	0	0	0
C2orf69P4	0	0	0	0	0	0	0	0
C2orf72	0	0.182008	0	0.389747	2.074348	0.849942	0	1.433473
C2orf73	0	0	0	0	0	0	0	0
C2orf74	31.727474	156.923188	62.34602	139.848142	23.232459	62.320382	121.879613	62.532868
C2orf76	19.304346	6.529073	5.470155	2.209691	1.642637	2.072552	0	8.181901
C2orf80	0	3.421444	0	0	0	0	0	0
C2orf81	4.411986	17.951123	5.071964	15.506907	5.801732	4.934641	0	2.631783
C2orf88	0	0	0	0.573711	0	0.476639	0	0.335877
C2orf92	0	0.169451	0	1.351664	0	0.17906	0	0.60651
C3	0	0	0	49.56417	81.032121	1.748125	0	0
C3AR1	0	0	0	0	0	0	0	0
C3orf18	2.889829	6.416145	4.196797	4.944262	4.954747	3.087632	0	3.182212
C3orf20	0	0	0	0.354035	0	0	0	0
C3orf22	0	0	0	0	0	0.19473	0	0
C3orf33	8.567574	7.742619	10.407981	5.31824	0	3.552466	0	7.173967
C3orf38	21.197563	32.768356	30.928553	16.420753	6.337313	12.5707	50.045731	38.251826
C3orf49	0	0	0	0	0	0	0	0
C3orf49P1	0	0	0	0	0	0	0	0
C3orf52	0	0.247234	2.397433	1.489068	9.193676	0.046434	9.386387	1.098798
C3orf62	1.00176	5.199104	0	2.935019	1.05783	2.119885	0	2.796992
C3orf70	0	0	0	0.011056	0	0	0	0.036224
C3orf80	0	0	0	0	0	0	0	0

C3orf84	0	0	0	0	0	0	0	0
C3orf85	0	0	0	0	0	0	0	0
C3orf86P	0	0	0	0	0	0	0	0
C3P1	0	0	0	0	0	0	0	0
C4A	0	0.745346	0	0.166585	0	0	0	0
C4B	0	0	0	0.663182	0	0.821456	0	0
C4B_2	0	0	0	0	0	0	0	0.152836
C4BPA	0	0	0	0	0	0	0	0
C4BPAP1	0	0	1.64529	0.089503	0	0	0	0
C4BPAP2	0	0	0	0	0	0	0	0
C4BPB	0	0	0	0	0	0.31546	0	1.009294
C4orf17	0	0	0	0	0	0	0	0
C4orf19	0.911123	0.878418	0	0.222326	3.608554	0.088282	10.803332	0.360278
C4orf3	36.747676	19.38744	24.95636	15.55938	37.78237	17.102911	4.306334	22.788075
C4orf33	60.549508	11.602914	15.470408	8.470153	5.468772	1.464409	0.256687	2.84526
C4orf36	11.817411	18.181624	0	10.379308	0	7.294351	73.37675	15.715872
C4orf46	10.064456	7.924347	8.012371	2.736261	6.275313	3.491857	3.620975	2.510052
C4orf46P1	0	0	0	0	0	0	0	0
C4orf46P2	0	0	0	0	0	0	0	0.609528
C4orf46P3	0	0	0	0	0	0	0	0
C4orf50	0	0	0	0	0	0	0	0
C4orf51	0	0	0	0	0	2.025928	0	0
C5	0	0	0	0	0	0	0	0
C5AR1	1.472592	8.107266	11.638319	3.277068	0	3.034116	0	1.425039
C5AR2	0	0	0	0	0	0	0	0
C5orf15	77.315519	99.239211	46.374828	107.551499	66.350854	107.166622	163.854915	84.784109
C5orf22	31.812812	38.155843	23.361367	25.480567	24.40988	11.198742	17.682835	19.914099
C5orf24	18.013287	78.328003	14.449091	51.786856	21.818692	67.047948	129.681989	61.119816
C5orf34	9.516717	15.721404	7.298601	6.657699	5.021365	3.193102	0	17.227323
C5orf46	0	0	0	0	0	0.748134	0	0
C5orf47	0	0	0	0	0	0	0	0
C5orf52	0	0	0	0.278202	0	0	0	0.725159
C5orf58	0	0	0	0	0	0	0	0

C5orf63	1.128352	0.175584	0	2.44007	18.110693	4.739974	0	0.987786
C5orf67	0	0	0	0	0	0	0	0
C6	0	0	0	0	0	0	0	0
C6orf118	0	0	0	0.140008	0	0	0	0
C6orf132	24.16597	17.587353	20.854797	31.690754	34.084605	66.320988	11.895019	54.090968
C6orf136	16.924633	10.033521	32.384279	9.607892	18.554118	11.91559	28.304923	9.383954
C6orf141	6.754929	4.284524	0	4.521398	1.015237	9.029821	42.25182	6.270067
C6orf15	0	0	0	0	0	0	0	0
C6orf163	0	0	0	0.722538	0	0	0	1.215587
C6orf226	0	1.313477	0	2.95841	0	0	0	3.520833
C6orf47	16.521026	59.496856	12.317219	54.151638	10.169618	104.605322	0.333992	84.836279
C6orf52	0	0	0	1.867039	0	0	0	0
C6orf58	0	0	0	0.298027	0	0	0	0
C6orf62	61.137895	171.433976	77.074431	109.779303	123.109569	139.751738	79.381866	354.223747
C6orf89	8.562387	57.818432	16.910195	71.520228	13.457014	73.780591	3.013704	75.141153
C7	0	0	0	0	0	0	0	0
C7orf25	0	1.822836	0	2.002635	0	4.114436	0	5.790309
C7orf33	0	0	0	0	0	0	0	0
C7orf50	127.265333	327.271371	104.688569	325.878354	62.451434	266.290081	45.074677	223.219314
C7orf57	0	3.350648	0	3.364123	0	3.231096	0	0.407413
C7orf78	0	0	1.466953	0	0	0	0	0
C8B	0	0	0	0	0	0	0	0
C8G	0	0	0	0.494955	0	0.562907	0	1.805726
C8orf33	28.83988	75.811004	32.850938	70.426815	14.682275	73.45338	79.27165	40.959512
C8orf34	1.389498	0.755526	2.251323	0.679791	0	0.223621	0	0
C8orf44	1.929677	2.390881	0	6.149195	0	1.665869	0	7.516375
C8orf44-SGK	0	5.442904	0	1.449658	0	1.806709	0	0
C8orf48	2.549282	14.091915	11.328404	6.408615	2.684279	1.999999	0	3.925098
C8orf58	8.896734	6.481663	0	9.444429	23.517747	13.282162	0	16.144077
C8orf74	0	0	0	0.071172	0	0	0	0
C8orf76	54.692237	73.942888	26.821522	35.101344	49.203282	47.890386	38.418063	94.872744
C8orf82	4.337324	2.719564	0	8.640706	4.151058	9.432025	0	8.621569
C8orf88	0	0	0	0.088289	0	0	0	0

C8orf89	0	0	0	0	0	0	0	0
C8orf90	0	0	0	0	0	0	0	0
C9	0	0	0	0	0	0	0	0
C9orf152	0	0	0	0	0	0	0	0
C9orf153	0	0	0	0.795996	0	0	0	0
C9orf40	8.725485	4.202716	1.300483	5.730636	6.904614	9.033152	11.284422	8.788639
C9orf43	0	0	0	0	0	0	0	0
C9orf50	0	1.251232	0	0	0	0	0	0
C9orf57	0	0	0	0	0	0	0	0
C9orf72	0	7.323845	1.946967	3.301049	5.250904	3.573733	45.950141	5.428488
C9orf78	60.636689	195.914508	70.553661	191.879748	85.420125	188.335366	61.014303	124.921681
C9orf78P1	0	0	0	0	0	0	0	0.557446
C9orf78P2	0	0	0	0	0	0	0	0
C9orf85	12.590092	11.020601	11.151922	10.654942	32.277812	9.434151	19.648694	14.023358
C9orf85P2	0	0	0	0	0	0	0	0
CA1	0	0	0	0	0	0	0	0
CA10	0	0	0	0	0	0.598348	0	0.696569
CA11	4.116408	3.469638	3.66861	10.161209	42.520364	10.07012	0	4.099497
CA12	17.465572	13.594291	16.654054	16.305829	21.4388	17.12367	6.495049	15.093103
CA13	1.703566	6.64511	10.338839	6.386202	0.682051	3.568799	0.621748	6.406388
CA14	0	0	2.081493	0.51343	0	0	0	0.665208
CA15P1	0	0	0	0	0	0	0	0.178704
CA15P2	0	0	0	0	0	0	0	0
CA2	50.306532	66.86334	36.635119	67.8414	20.475808	33.14211	49.301452	20.959926
CA3	0	0	0	0.099398	0	0	0	0
CA4	0	0	0	0	0	0	0	0
CA5A	0	0	0	0	0	0	0	0
CA5AP1	0	0	0	0	0	0	0	0
CA5B	1.894935	1.236057	0.852019	2.003294	1.377033	0.341403	0	0.942498
CA5BP1	0	0	0	0.62158	0	1.218955	0	0.523877
CA6	0	0	0	0	0	0	0	0
CA7	0	0	2.855201	0.232948	0	0	0	0
CA8	0	0	0	0.055528	0	0	0	0

CA9	120.46435	15.451455	32.942	37.029112	0	49.864877	44.148205	46.029433
CAAP1	10.887938	40.951975	26.118071	28.911606	18.606516	45.620409	96.882305	30.097575
CAB39	59.659777	68.998999	52.786784	50.220272	89.121599	76.762273	121.863844	124.253907
CAB39L	1.983973	6.025232	5.111037	5.233003	1.517249	1.109144	0	4.667483
CAB39P1	0	0	0	0	0	0	0	0
CABCOCO1	0	0	0	0	0	0	0	0
CABIN1	16.538873	21.132722	19.003517	30.48306	32.360008	54.094108	6.136947	34.362837
CABLES1	5.511824	5.985105	1.489244	3.57904	0	16.830259	18.385491	6.250851
CABLES2	0	14.479814	0	5.501037	20.736869	6.001936	0	0
CABP1	5.374647	0.217163	0	0.112374	0	0	0	0.922845
CABP2	0	0	0	0	0	0	0	0
CABP4	0	0	0	0	0	0	0	0
CABP5	0	0	0	0	0	0	0	0
CABP7	0	1.19703	0	1.049226	0.264122	0.848356	0	1.24667
CABS1	0	0	0	0	0	0	0	0
CABYR	0	12.621828	4.13814	3.331908	3.019049	2.048222	16.577068	2.682059
CABYRP1	0	0	0	0	0	0	0	0
CACFD1	2.337886	1.271185	5.633674	0.298752	2.988836	1.536285	24.491192	0.126481
CACHD1	4.346304	3.643902	9.484009	0.22446	3.107988	0	2.5477	0
CACNA1A	0	0	0	0.257701	0	0.056161	0	0
CACNA1B	0	0.584818	1.199645	1.841386	0	0.03402	0	0.144916
CACNA1C	0	0	0	0	0	0	0	0
CACNA1D	1.91444	1.544019	0	0.717508	0	0	0	0
CACNA1E	0	0	0	0.011363	0	0	0	0
CACNA1F	0	0	0	0	0	0	0	0
CACNA1G	0	0.858044	0	0.166879	0	0.169257	0	0.174544
CACNA1H	0	0	0	0	0	0	0	0
CACNA1I	0	0.111978	0	0.09353	0	0	0	0
CACNA1S	0	0	0	0	0	0	0	0.021746
CACNA2D1	0	2.782603	0	0.606614	0	0.71182	0	0
CACNA2D2	0	0	0	0.178825	3.054312	0	0	0.66459
CACNA2D3	0	0	0	0	0	0	0	0
CACNA2D4	0	0	0	0	0	0	0	0

CACNB1	2.680676	1.699601	7.894801	3.387455	13.871957	4.438902	0	1.364099
CACNB2	0	0	0	0	0	0	0	0
CACNB3	22.795506	13.502115	13.665457	15.752863	13.945053	28.376472	26.114347	24.270465
CACNB4	0	0	0	0	0	0	0	0.037913
CACNG1	0	0	0	0.068018	0	0.275565	0	0
CACNG2	0	0	0	0	0	0	0	0
CACNG4	6.49043	9.002333	4.158245	7.194081	5.140991	9.164581	2.70605	9.649521
CACNG5	0	0	0	0	0	0	0	0
CACNG6	2.17165	0	3.868343	1.589979	0	0	0	0
CACNG7	7.040566	0	0	0	0	0	0	0
CACTIN	14.257196	13.378572	11.141622	14.314563	8.189382	20.740654	48.176802	16.342753
CACUL1	42.6534	96.053391	56.546524	80.749832	98.073903	63.469644	87.955221	84.253375
CACYBP	155.059104	504.806406	75.986061	337.731404	173.37432	225.591779	171.219111	326.708135
CACYBPP1	0	0	0	0	0	0	0	0
CACYBPP2	0	0	0	0	0	0	0	0
CACYBPP3	0	0	0	0	0	0	0	0
CAD	39.496884	27.447346	39.116311	31.285614	40.108822	40.305921	38.677594	32.565316
CADM1	0	0.966542	0.847268	1.233726	16.19049	3.657787	0	3.126404
CADM2	0	0	0	0.535157	0	0.370644	0	0
CADM3	0	0.227048	0	0.032477	0	0	0	0
CADM4	1.571724	4.466176	0	3.400333	6.631786	15.735903	8.379216	6.906497
CADPS	0	0	0	0.047182	0	0	0	0
CADPS2	2.292653	3.391061	2.744378	3.674479	0	0.191095	0	0.736341
CAGE1	0	0	0	0.148447	0	0	0	0.192342
CALB1	0	0	0	0.586971	12.137462	20.94668	70.344324	23.890545
CALB2	6.05573	6.989977	9.238655	5.849863	4.184747	1.3392	0	1.399679
CALCA	0	0	0	0	0	0	0	0
CALCB	0	0	0	0.175976	0	0	0	0
CALCOCO1	44.83741	13.36092	27.138923	18.421997	12.170243	9.519231	0	2.598594
CALCOCO2	0	66.413239	50.485891	85.563941	86.600968	68.294247	90.311867	63.124358
CALCP	0	0	0	0	0	0	0	0
CALCR	0	0	0	0	0	0	0	0
CALCRL	0	3.412844	0	0.815072	0	0	0	0.654326

CALD1	30.041498	24.515086	19.087611	30.695519	64.661602	85.796123	81.334706	38.091446
CALHM2	3.798884	65.480975	8.910997	74.077296	15.829134	98.090407	0	79.442546
CALHM3	0	0	0	0.156017	0	0.31715	0	0
CALHM4	0	0	0	0	0	0	0	0
CALHM5	0	0	0	0.024108	0	0	0	0
CALHM6	0	0.6925	0	0.207236	0	0.487244	0	0.286267
CALM1	392.787736	616.428588	336.304783	401.576748	236.144062	348.82589	499.791518	741.195583
CALM1P1	0	0	0	0	0	0	0	0
CALM1P2	0	0	0	0	0	0	0	0
CALM2	654.522567	692.092284	533.70452	440.554685	480.247237	242.574593	557.56755	552.146398
CALM2P1	0	0	0	0	0	0	0	0
CALM2P2	0	0	0	0	0	0	0	0
CALM2P3	0	0	0	0	0	0	0	0
CALM2P4	0	0	0	0	0	0	0	0
CALM3	189.034944	277.184656	106.996205	268.060193	201.696471	527.517641	199.023996	384.491482
CALML4	0	12.934774	0	10.694728	0	0	0	0
CALML6	0	0	0	0	0	0	0	0
CALN1	0	0	0	0	0	0	0	0
CALR	0	0	0	0.445745	0	0.874861	0	1.098018
CALR3	0	0	0	0	0	1.718931	0	0
CALR4P	0	1.01797	0	0.154608	0	0	0	0
CALU	53.168008	119.745257	68.518916	181.594214	91.839827	85.946239	142.28972	59.527609
CALY	0	0	0	0.157835	0	0	0	0
CAMK1	12.472832	3.931585	11.038023	6.844809	0	7.527881	0	6.173933
CAMK1D	0	0	5.186362	2.333423	0	1.564073	0	0
CAMK1G	0	0	0	0	0	0	0	0
CAMK2A	0	0	0	0	0	0	0	0
CAMK2B	0	1.151702	0	0.521788	0	0.988516	0	0.221449
CAMK2D	13.075644	32.270931	11.977007	30.717713	11.720096	25.346415	12.682538	29.043665
CAMK2G	34.986901	40.535971	4.457662	37.009027	7.263487	35.091863	36.713021	30.875343
CAMK2N1	88.134698	73.379291	53.936801	34.226881	68.169104	17.958048	49.461511	29.304535
CAMK2N2	7.454901	11.768943	6.627962	10.973258	5.887956	6.950135	0	4.529367
CAMK4	0.536117	2.350359	0.241337	1.131504	0	1.270857	0	2.528361

CAMKK1	0.950983	0.168065	4.98718	0.39759	7.784518	0.886315	5.550152	0.348657
CAMKK2	19.395313	39.959624	18.755033	37.428327	11.76669	41.133608	16.536385	33.364966
CAMKMT	0	5.377859	0	3.042825	0	2.354085	0	3.404089
CAMKV	13.160989	49.142191	18.674663	69.844675	8.375763	17.455459	30.848857	14.938174
CAMLG	33.305147	27.97678	76.510849	27.054951	7.946364	14.837629	26.131879	23.136436
CAMSAP1	23.62238	41.047299	25.863648	35.948275	20.53658	49.252753	90.050301	32.165693
CAMSAP2	60.354202	104.100386	57.658653	91.642484	67.884231	70.541704	40.782482	114.582646
CAMSAP3	20.984777	2.346092	0	4.022774	1.875095	4.778731	0	2.086702
CAMTA1	61.861743	37.628831	45.209766	25.264191	5.076326	28.690794	124.236116	44.384761
CAMTA2	21.354159	11.036078	5.782589	13.318687	37.178517	13.66818	7.50782	12.281774
CAND1	200.347459	204.198893	121.656749	106.323878	218.53784	118.409932	126.577727	260.327046
CAND2	0	0.159162	0.666622	0.403459	0	1.940607	0	0.059606
CANT1	62.486735	170.348676	51.156686	199.631949	41.427366	240.0438	39.319442	178.561642
CANT1P1	0	0	0	0	0	0	0	0
CANX	448.010392	525.967766	632.209763	746.505042	346.234587	407.114621	506.692359	365.434236
CAP1	197.036496	129.753556	205.090112	139.789551	319.485391	185.613049	283.842285	167.398229
CAP1P1	2.573645	0.754244	2.282054	1.987443	2.704182	2.143755	0	2.925867
CAP1P2	0	0	0	0	0	0	0	0
CAP2	4.652797	5.114911	0	2.504825	0	3.332465	0	4.607013
CAP2P1	0	0	0	0	0	0	0	0
CAPG	116.96532	89.691665	99.640881	137.174934	143.905543	130.093255	214.414465	66.486683
CAPN1	37.967519	22.384902	10.372579	19.091711	121.679963	98.025399	178.155078	64.662658
CAPN10	0	4.335916	4.080793	3.414508	5.459196	7.405116	24.348118	6.945804
CAPN11	0	0	0	0	0	0	0	0
CAPN12	4.42437	4.263979	12.537258	9.099489	2.671124	22.597385	0	11.929518
CAPN13	0	0	0	0	0	0	0	0
CAPN14	0	0.187193	0	0.40552	0	0.436717	0	0.280703
CAPN15	22.709669	30.052752	16.082667	22.259688	41.547474	61.32634	15.111631	49.356344
CAPN2	211.666855	247.138053	168.676921	240.975468	126.170511	227.000878	217.501954	148.768667
CAPN3	0	0	0	0	0	0.54265	0	0
CAPN5	4.257362	3.435086	2.705839	4.683771	0	3.555565	0	3.978901
CAPN6	0	0.040254	0	0.091862	2.982376	0	6.180856	0.113477
CAPN7	18.16505	45.017593	21.783974	29.103482	33.262498	27.538082	42.048012	45.264356

CAPN8	0	0	0	0	0	0	0	0
CAPN9	0	0	0	0	0	0	0	0
CAPNS1	301.440068	255.166586	281.494301	258.762329	485.481036	399.398412	548.470002	263.285619
CAPNS1P1	0	0	0	0	0	0	0	0
CAPRIN1	206.788581	346.103515	207.863931	331.347989	664.972884	707.674885	573.689451	755.688881
CAPRIN2	3.469444	25.916629	9.428865	18.625872	41.50194	16.859036	53.076735	10.98528
CAPS	0	0	0	0	0	0.439081	0	0
CAPS2	0	6.750004	11.986919	8.835397	0	1.816315	0	4.43454
CAPSL	0	0	0	0.472541	0	0.625956	0	0.819229
CAPZA1	267.14038	226.665663	223.236905	202.358656	139.17835	141.980881	187.440445	180.15668
CAPZA1P1	0	0	0	0	0	0	0	0
CAPZA1P2	0	0	0	0	0	0	0	0
CAPZA1P3	0	0	0	0	0	0	0	0
CAPZA1P4	0	0	0	0	0	0	0	0
CAPZA1P5	0	0	0	0	0	0	0	0
CAPZA2	190.293004	166.593361	99.873564	80.94241	191.758924	50.695631	65.707034	182.138262
CAPZB	120.464137	131.101603	94.14575	123.544258	176.785118	216.843627	123.119333	189.487571
CAPZBP1	0	0	0	0	0	0	0	0
CARD10	16.85357	26.081865	15.847749	24.230428	49.592705	36.794493	20.509083	20.925468
CARD11	0	2.471096	0	3.307317	16.678285	6.335157	30.666917	1.456906
CARD14	0	2.972853	6.626257	1.05469	0.378883	1.383114	0	0.526666
CARD16	0	18.43962	0	20.585795	40.819766	6.128734	46.642529	0
CARD17P	0	0	0	0	0	0	0	0
CARD18	0	0	0	0	0	0	0	0
CARD19	10.954707	65.731864	24.660829	72.632851	44.178184	98.896168	28.058442	58.910111
CARD6	1.635502	6.921768	4.403591	6.546858	10.149834	10.340548	4.179144	13.8817
CARD8	14.646291	25.58591	1.841436	28.202938	19.616395	36.753507	0	39.566632
CARD9	0	0	0	0	0	0.071844	0	0.439032
CARF	0.627202	5.517084	2.254097	6.231595	1.069278	2.475282	0	5.376011
CARHSP1	59.377561	44.982465	77.565389	79.07116	88.121798	109.940935	16.664965	84.907256
CARM1	17.529064	27.498659	28.489094	27.479561	70.922224	57.322431	59.081317	60.15058
CARM1P1	0	0	0	0	0	0	0	0
CARMIL1	13.674616	23.932418	22.439994	28.666989	23.561244	31.738411	18.205241	31.620177

CARMIL2	4.096602	0.930887	0	1.252399	0	0.589157	0	0.872078
CARMIL2P1	0	0	0	0	0	0	0	0
CARMIL3	0	0.065341	0	0.271141	0	0.143202	0	0.181099
CARNMT1	45.70863	17.131332	6.248767	15.212525	63.566172	17.093436	44.883737	33.493676
CARNS1	0	0.574586	0	0.596523	0	0.293342	0	0.303134
CARS1	82.305716	74.312352	101.564378	104.549133	79.869146	79.98679	95.224454	52.146425
CARS1P1	0	0	0	0	0	0	0	0
CARS1P2	0	0	0	0	0	0	0	0
CARS2	24.873258	53.535718	22.933321	38.7742	49.207265	52.454709	115.011274	54.328734
CARTPT	0	0	0	0	0	0	0	0
CASC3	57.827492	52.397285	40.931069	70.119022	45.661737	75.156539	36.003259	49.242644
CASD1	15.95145	2.699594	8.770212	1.978707	3.553768	3.023255	9.475006	4.470735
CASK	27.102285	23.186027	26.317649	17.560987	8.730644	18.375519	6.975688	28.428747
CASKIN1	0	0	0	0	0	0	0	0
CASKIN2	4.610602	18.163052	6.508486	27.317182	59.971667	50.798448	38.358004	36.896115
CASKP1	0	0	0	0	0	0	0	0
CASP1	21.5541	6.339956	38.5502	9.7056	53.207608	4.332195	0.555238	20.746099
CASP10	0	0.4247	0	1.050676	0	2.278228	0	2.428237
CASP12	0	0	0	0	0	0	0	0
CASP14	0	0.272648	0	0.856293	0	1.169458	0	0.085592
CASP16P	0	0	0	0	0	0	0	0
CASP1P1	0	0	0	0	0	0	0	0
CASP1P2	0	0	0	0	0	0	0	0
CASP2	40.29212	42.199532	54.733097	38.972686	37.225376	37.221197	31.820023	33.734225
CASP3	24.076922	26.157674	13.166623	12.795363	17.352583	13.037533	50.956998	31.941873
CASP3P1	0	0	0	0	0	0	0	0
CASP4	189.84121	215.656722	155.881335	280.658152	114.087159	168.460723	219.484416	168.064212
CASP4LP	0	0	0	0	0	0	0	0
CASP5	0	0	0	0	0	0	0	0
CASP6	10.836965	27.411249	7.520553	21.748995	41.204729	19.612694	72.932505	40.218092
CASP7	0	12.145467	4.234672	27.597034	46.486602	30.253899	61.975373	22.741076
CASP8	0	15.158289	16.151824	14.700831	1.623086	9.869371	15.799089	11.892381
CASP8AP2	5.383238	9.313178	0	10.778232	29.7272	9.68799	45.471871	10.860482

CASP9	0	2.72321	8.298508	5.291654	23.104421	12.520618	0	7.915729
CASQ1	0	0	0	0	0	0	0	0
CASR	0	0	0	0	0	0	0	0
CASS4	0	0	0	0.11763	0	0	0	0
CASTOR1	19.099319	0.837996	0	0	0	0	0	0.336431
CASTOR2	10.079271	0	0	0	0	0	0	0
CASTOR3P	8.427112	4.205724	0	4.548115	0.455726	5.621532	0	6.512966
CASZ1	25.262591	5.656069	2.738559	5.713058	7.88733	16.734688	2.038852	12.663834
CAT	37.391288	26.128574	30.755489	21.487154	55.798036	20.625518	100.094794	28.976586
CATIP	0	0	0	0	0	0	0	0
CATSPER1	0	15.340277	0	9.926826	0	5.246165	0	2.138909
CATSPER2	0	0.653527	9.702802	0.27395	0	1.091318	0	0.484404
CATSPER2P1	0	0.199516	0	0	0	0.502958	0	0
CATSPER2P2	0	0	0	0	0	0	0	0
CATSPER3	8.562846	0	2.389498	0.064988	0	0	0	0.109104
CATSPER4	0	0	0	0	0	0	0	0
CATSPERB	0	0.233616	0	0	0	0.136834	0	0.113526
CATSPERD	0	0	0	0	0	0	0	0
CATSPERE	0	0.659681	0	0.113299	0	0	0	0
CATSPERG	0	1.600929	0	1.254449	0	0.863771	0	4.016661
CATSPERZ	5.284538	15.818553	4.624969	9.51265	9.687267	14.088564	0	10.206991
CAV1	1701.72654	1276.4344	1402.67689	1338.19565	1298.93631	1259.83405	1666.20791	1342.65106
CAV2	368.581376	408.473987	265.677601	338.033957	251.510195	195.409155	433.90138	284.674236
CAV3	0	0	0	0	0	0	0	0
CAVIN1	182.429684	192.069216	125.223703	166.408074	216.749088	302.871078	218.216133	222.735955
CAVIN2	0	0	0	0.026766	8.405198	3.282708	3.201677	0.839044
CAVIN3	0	2.639579	3.307034	1.97163	0	0	0	0
CBARP	2.777771	4.490663	10.045234	5.660866	3.328732	6.155747	0	6.170294
CBFA2T2	22.306326	19.341889	11.365621	17.712845	21.178982	16.716127	0.858019	10.216303
CBFA2T3	0	3.994222	2.939523	3.383363	5.600855	0.839973	0	0.940671
CBFB	161.260299	358.579004	115.482791	362.970021	110.331616	407.969827	107.081792	325.671442
CBL	17.535706	52.55716	28.43602	83.872356	0	17.637732	0	12.283294
CBLB	15.45573	28.050506	43.738309	11.460623	15.176278	13.944347	0	10.923686

CBLC	6.210768	9.038918	10.075409	15.293932	44.714987	31.580976	4.834187	24.041581
CBLIF	0	0	0	0	0	0	0	0
CBLL1	19.889185	12.877923	7.060147	5.533082	5.485201	6.986092	33.80421	10.212854
CBLL1P1	0	0	0	0	0	0	0	0
CBLN1	0	0	1.248495	0.169802	0.368173	0	0	0.562131
CBLN2	0	0.120543	0	0	0	0	0	0
CBLN3	0	0.546318	0	0.139856	0	0.651886	0	1.458704
CBR1	49.229673	88.377045	59.982241	90.817574	84.152586	121.128487	47.319051	110.064685
CBR3	7.740488	25.81665	17.071505	23.769113	0	28.995295	30.555323	30.000149
CBR4	12.582973	11.322936	11.942302	6.298757	0.766621	2.750167	8.708054	9.62099
CBS	14.942545	1.79592	2.988391	8.569855	12.87008	13.786931	76.389498	3.484178
CBX1	118.292221	359.547749	102.903847	366.735895	77.363776	392.553584	372.095135	279.904502
CBX1P1	0	0.952566	0	0	0	0	0	0
CBX1P2	0	0	0	0	0	0	0	0
CBX1P3	0	1.769146	0	0	0	1.258244	0	1.535639
CBX1P4	0	0	0	0	0	0	0	0
CBX1P5	0	0	0	0	0	0	0	0
CBX2	6.38274	6.722934	5.095156	6.851264	5.807026	13.941191	3.453884	8.795315
CBX3	365.738246	507.210941	268.264715	255.459462	207.648799	167.638525	209.803775	394.485432
CBX3P1	0	0.330751	0	0	0	0	0	0
CBX3P10	0	0	0	0	0	0	0	0
CBX3P2	8.948578	3.048427	0	2.099548	0	0	0	0
CBX3P3	0	0	0	0	0	0	0	0
CBX3P4	0	3.800704	0	0	0	0	0	0
CBX3P5	0	0	0	0	0	0	0	0
CBX3P6	0	0	0	0	0	0	0	0
CBX3P7	0	0	0	0	0	0	0	0
CBX3P8	0	0	0	0	0	0	0	0
CBX3P9	0	0	0	0	0	0	0	0
CBX4	26.567663	80.377238	30.579439	94.39905	16.691085	91.972003	16.276562	49.486225
CBX5	134.755339	148.082441	74.462613	125.148406	26.129295	36.346211	59.478354	50.117642
CBX5P1	0	0	0	0	0	0	0	0
CBX6	0	0.599551	0	3.400556	0	1.388825	0	2.245708

CBX7	1.605102	5.610699	4.322113	5.134205	5.510593	4.282686	0	1.950646
CBX8	5.299751	10.267182	2.377379	9.23398	0.466468	5.632302	9.454606	3.11329
CBY1	6.728802	31.470166	0	54.749796	0	15.024552	0	10.763939
CBY1P1	0	0	0	0	0	0	0	0
CBY2	0	0.500506	0	0.230847	0	0	0	0
CBY3	0	0	0	0	0	0	0	0
CC2D1A	14.844002	18.738693	9.153496	20.686255	54.473549	53.950559	9.251084	33.126247
CC2D1B	17.964409	8.687243	9.462119	11.410611	13.295841	3.223928	4.907777	1.774353
CC2D2A	10.474485	12.768839	14.487341	4.99597	0	6.446745	0	7.781365
CC2D2B	0	0.894171	0	0	0	1.065871	0	0
CCAR1	44.236831	42.803671	54.240317	45.326559	110.820915	42.393421	112.514129	63.681905
CCAR2	57.096216	56.594688	66.499171	86.883187	141.06379	140.209751	54.096883	67.788088
CCBE1	16.502955	24.914981	8.658572	13.813024	16.366565	8.83602	13.586266	9.059616
CCDC102A	9.813002	11.559906	6.270021	15.668504	0.739613	12.947853	0	8.752062
CCDC102B	0	0.079468	0	1.1041	0	0.501185	0	0
CCDC103	0	5.516947	3.762127	8.594609	0	3.832816	1.877162	4.235761
CCDC106	16.257113	8.114234	11.513336	6.614314	3.04636	18.263553	0	10.305007
CCDC107	3.185698	10.933826	11.62492	10.712975	13.309268	13.181007	0	12.507526
CCDC110	0	0	0	1.510366	0	0	0	0
CCDC112	10.030557	5.905419	5.972626	3.240133	3.813814	2.343372	0	6.281299
CCDC115	14.684894	20.742286	21.025102	14.329815	19.856071	16.999159	23.780121	15.556299
CCDC116	0	0	0	0.037429	0	0.152659	0	0
CCDC117	8.33758	12.41186	13.564564	12.757564	13.010137	13.478555	6.635297	18.329844
CCDC12	26.47407	40.788409	44.981602	68.774001	38.166613	73.79797	48.883638	23.853423
CCDC120	13.728702	46.123163	18.477801	49.276968	19.599613	57.087272	4.953414	38.882118
CCDC121	16.720387	1.673574	2.175498	1.703551	0	2.701017	0	2.016217
CCDC121P1	0	0	0	0	0	0	0	0
CCDC122	5.042021	20.025083	3.002085	7.537839	3.166853	1.367232	0	1.363818
CCDC124	38.52747	72.049647	76.120534	105.503591	114.343159	175.830943	45.977243	103.585743
CCDC125	3.779052	8.762261	15.078409	8.245352	13.903976	3.157707	9.142276	7.207556
CCDC126	8.271352	32.140537	11.335881	20.620342	0	5.54634	24.636429	11.462187
CCDC127	8.021462	8.371633	0	6.025308	0	0	0	0
CCDC12P1	0	0	0	0	0	0	0	0

CCDC13	0	0	0	0.235798	0	0.396528	0	0
CCDC134	0	0	31.579206	3.077448	24.480412	0	0	0
CCDC136	15.923644	3.964045	0	5.402612	10.320879	3.834163	0	1.562209
CCDC137	34.916511	52.77574	22.095527	56.989035	84.772707	72.870496	74.769203	40.540404
CCDC137P1	0	0	0	0	0	0	0	0
CCDC137P2	0	0	0	0	0	0	0	0
CCDC138	3.612939	7.066002	3.185693	4.644443	18.69419	3.855364	13.310152	18.246742
CCDC14	12.961437	5.275814	7.391955	5.388799	49.912694	6.400823	30.457424	10.948893
CCDC140	0	0	0	0.151236	0	0	0	0
CCDC141	0	0	0	0	0	0	0	0
CCDC142	23.074261	4.172442	11.850371	4.677689	13.474197	3.673476	0	0.565458
CCDC144A	1.853559	0.753401	9.024988	0.575809	0	0.10791	0	0.56259
CCDC144BP	0	0.662308	1.341532	1.047447	0	0.54264	0	0.706925
CCDC144CP	0	0.434853	0	0.081598	0	0	0	0.191121
CCDC144NL	0	0	0	0	0	0	0	0
CCDC146	1.418896	3.317112	14.326839	1.772699	25.98474	0.704449	0	1.448742
CCDC148	0	2.27504	0	0.914661	0	1.043162	0	0.652388
CCDC149	5.1315	16.279371	15.191017	23.742775	0.42767	12.438524	4.57795	11.921192
CCDC15	3.245082	2.19383	2.33826	1.716119	19.956344	3.02275	7.608296	5.716549
CCDC150	3.055972	2.219054	0	1.103761	0.803699	3.552137	0	3.069683
CCDC152	0	0	0	0	0	0	0	0
CCDC154	0	0	0	0	0	0	0	0
CCDC157	0	1.667933	0	0.521582	0	0.930667	0	2.590726
CCDC158	0	0	0	0.088422	0	0	0	0
CCDC159	0	2.55689	12.167988	2.580104	21.049995	3.652148	0	1.93364
CCDC160	0	0	0	0	0	0	0	0
CCDC162P	0	0	0	0	0	0	0	0
CCDC163	2.08682	4.48554	0	3.172838	0	3.865403	0	2.394177
CCDC166	0	0	0	0	0	0	0	0
CCDC167	8.103613	18.700401	0	19.947468	6.332409	32.620465	15.164621	26.704361
CCDC169	4.352297	4.220844	7.878848	12.003566	0	4.42621	0	5.042386
CCDC169-SC	1.687874	0.678239	0	0.609037	0	0	0	0
CCDC17	0	0	0	0.279574	0	0.447867	0	0.385005

CCDC170	0	1.318459	0	0.594027	0	0	0	0
CCDC171	1.48457	3.086801	0.444951	1.172402	0	1.092198	0	1.567318
CCDC172	0	0	0	0	0	0	0	0
CCDC174	14.865471	24.045662	25.54487	26.267246	14.197967	21.316338	8.164931	20.720584
CCDC175	0	0	0	0	0	0	0	0
CCDC177	0	0	0	0.096246	0	0	0	0.063303
CCDC178	0	0.473114	0	0	0	0	0	0
CCDC179	0	0	0	0	0	0	0	0
CCDC18	0	22.802673	29.810861	12.304292	4.629373	1.578062	38.492416	5.536908
CCDC180	0	1.873715	0	0.767155	0	1.640268	0	2.010799
CCDC181	0	0	2.63057	4.218259	7.023383	0.133746	0	0.433957
CCDC183	0	0	0	0.274804	0	0	0	0
CCDC186	18.564683	12.618853	26.880272	10.268526	21.726163	2.766124	52.534154	11.291073
CCDC187	0	0.49721	0.949765	1.590648	0	0	0	0
CCDC188	0	0	0	0	0	0	0	0
CCDC188BP	0	0	0	0	0	0	0	0
CCDC190	0	0	0	0	0	0	0	0
CCDC191	10.956389	7.398627	6.106414	14.736724	3.593985	1.804638	0.430203	1.009073
CCDC192	0	0	0	0	0	0	0	0
CCDC194	0	0	0	0	0	0	0	0
CCDC195	0	0	0	0	0	0	0	0
CCDC196	0	0	0	0	0	0	0	0
CCDC197	0	0	0	0	0	0	0	0
CCDC198	0	2.112816	0	0.755085	0	0	0	0
CCDC200	0	0	5.876294	1.203471	0	0.270403	0	0
CCDC201	0	0.266233	0.797351	0.2169	0	0	0	0
CCDC22	4.446862	4.091078	5.301389	7.426409	1.954828	11.251691	39.583263	8.262028
CCDC24	10.086025	18.772703	7.612352	16.130549	2.849641	16.300695	0	7.283733
CCDC25	92.90466	92.9377	49.56766	87.749652	41.384759	63.180379	14.249914	82.130075
CCDC27	0	0	0	0	0	0	0	0
CCDC28A-A[illegible]	1.260069	0.638915	0	2.120089	0	0	0	0.878927
CCDC28B	0	7.06855	4.624969	0.203497	0	1.94401	3.084249	1.047552
CCDC3	0	1.699267	4.403929	0.808608	0.324467	0.428223	0	0.890683

CCDC30	5.163473	10.629069	1.265169	5.541002	0	1.345569	0	1.221435
CCDC32	13.078004	25.272542	11.317735	34.625728	35.776397	13.505302	0	15.267643
CCDC33	0	4.72095	0	9.987327	0	0	0	0
CCDC34	51.816557	33.717985	11.046604	17.541678	18.318086	40.714653	29.955286	56.894529
CCDC34P1	0	0	0	0	0	0	0	0
CCDC38	0	0	0	0	0	0	0	0
CCDC39	0	0.171628	0	0	0	0	0	0
CCDC40	0	9.887151	9.606027	5.509623	20.663092	2.442869	2.073234	6.11585
CCDC42	0	0	0	0	0	0	0	0
CCDC43	31.022987	31.642911	30.632085	24.1517	43.596651	30.074083	54.311986	31.233932
CCDC47	26.809327	147.869188	77.249022	164.067098	29.622322	87.936829	88.904645	126.991834
CCDC50	49.134061	70.425858	25.392914	49.47783	17.431213	94.795368	66.583315	98.349639
CCDC51	11.04082	35.15223	11.653558	35.338933	15.882078	46.313446	23.993788	39.976126
CCDC57	10.165019	18.650593	15.269535	17.459593	50.28407	15.735451	0.739083	7.029644
CCDC59	33.736974	159.732199	38.98563	156.421026	26.807941	134.866215	222.230956	136.854556
CCDC6	72.779931	38.645496	75.633413	34.521707	41.912776	24.971617	46.142769	35.393024
CCDC60	0	0	0	0.750889	0	0	0	1.101902
CCDC61	0	2.205114	15.911052	4.311937	1.241796	5.918894	0	4.130948
CCDC62	0	0.233556	0	0.036806	0	0.409251	0	0.110601
CCDC63	0	0	0	0.17963	0	0	0	0
CCDC65	0	1.190909	0	1.115069	0	0.267228	0	0.535145
CCDC66	24.292256	6.301667	14.820706	8.671469	4.742785	4.086419	75.798773	17.240212
CCDC68	14.860952	10.562768	7.251308	6.852578	7.467468	2.650145	0	1.487023
CCDC69	11.07711	6.036758	4.50066	7.154244	2.172342	5.674787	78.427577	4.177506
CCDC7	0	1.131568	6.83584	0.175399	0	0	0	0.44367
CCDC71	1.96089	36.32469	17.485373	43.088623	0	42.289506	30.345162	31.44639
CCDC73	0	0	0	0.036681	0	0.112679	0	0
CCDC74A	3.064846	2.098307	4.659041	6.828885	2.455688	0.999948	0	5.569345
CCDC74B	0	3.90718	12.600535	4.663252	16.362045	2.147677	0	1.977022
CCDC74BP1	0	0	0	0	0	0	0	0
CCDC77	25.022647	8.123283	9.530766	13.501589	18.317179	12.779152	0	14.47776
CCDC78	0	2.761703	0	1.267301	0	2.268653	0	0.132481
CCDC8	6.195658	28.867786	9.256373	30.826027	14.174473	53.403606	2.258915	43.495025

CCDC80	35.5325	41.828038	2.739548	56.66756	23.995302	110.54885	34.421914	50.843382
CCDC81	0	1.127491	0	0.29051	0	0.286309	0	0.207715
CCDC82	15.634839	47.398792	27.31354	13.790722	5.457566	9.120177	1.586725	17.534823
CCDC83	0	0	0	0	0	0	0	0
CCDC85A	1.603877	0.927631	2.159415	0.450357	0	0.320708	0	0.354216
CCDC85C	35.882487	66.387038	36.926668	72.020646	131.798901	195.471999	36.558782	173.927553
CCDC86	37.806578	19.245184	30.353217	27.868203	75.230292	67.221098	47.789143	49.157761
CCDC87	0	0.546629	0	0.482758	2.460692	0.232106	5.661518	0.140661
CCDC88A	34.413865	68.040107	57.118816	63.67118	44.992771	47.62753	18.403585	46.183765
CCDC88B	0	6.581255	1.78807	4.464755	0	8.473176	0	4.48175
CCDC88C	11.615866	11.543286	31.466809	14.140531	85.294097	45.30794	9.340426	39.664887
CCDC89	0	1.113402	3.906811	1.381494	0	1.084052	0	0.938764
CCDC9	6.369477	22.617224	4.580542	33.111401	7.212752	46.976885	11.574948	18.370823
CCDC90B	102.067843	138.33248	107.056532	54.769585	11.638817	15.992229	59.86769	54.890648
CCDC91	41.432227	89.889058	14.738387	32.800336	5.057177	18.379231	32.88071	52.947263
CCDC92	7.672169	3.288592	0	4.94095	0.360374	14.792257	0	12.677682
CCDC93	9.480374	20.362772	12.361685	16.580619	26.708326	25.936154	190.91233	19.947322
CCDC97	2.004157	15.539247	7.187398	19.249856	5.55537	36.776837	17.783633	23.997358
CCDC9B	0	18.494714	23.910124	18.20895	21.388827	20.486342	132.99597	31.619267
CCER1	0	0	0	0	0	0	0	0
CCER2	0	0	0	0	0	0	0	0
CCHCR1	14.635536	23.5763	19.455572	31.389836	32.245736	38.598625	1.018944	26.698795
CCK	9.75185	6.740253	9.774462	3.256502	0	0.932569	0	1.39969
CCKBR	0	0	0	0.098574	0	0.100251	0	0
CCL13	0	0	0	0	0	0	0	0
CCL14	0	0	0	0	0	0	0	0
CCL15	0	0	0	0	0	0	0	0
CCL15-CCL1	0	0	0	0	0	0	0	0
CCL16	0	0	0	0	0	0	0	0
CCL17	0	0	0	0	0	0	0	0
CCL18	0	0	0	0	0	0	0	0
CCL19	0	0	0	0	0	0	0	0
CCL2	0	0	0	0	1.479559	5.362466	0	0.698395

CCL20	0	0.506898	0	3.453424	0	0	0	0.794782
CCL21	0	0	0	0	0	0	0	0
CCL22	0	0	0	0.561965	0	0.057419	0	0.13916
CCL23	0	0	0	0	0	0	0	0
CCL24	0	0	0	0	0	0	0	0
CCL25	0	0	0	0	0	0	0	0
CCL26	0	0	0	0	0	0	0	0
CCL27	0	0.185073	0	0.649929	0	0.217516	0	0.556625
CCL28	5.356066	15.363344	5.760188	13.081084	4.241344	7.200078	0	8.009277
CCL3	0	0	0	0	0	0	0	0
CCL3L3	0	0	0	0	0	0	0	0
CCL4	0	0	0	0	0	0	0	0
CCL4L2	0	0	0	0	0	0	0	0
CCL5	0	0	0	0.977223	0	1.743137	0	1.013122
CCL7	0	0	0	0	0	0	0	0
CCL8	0	0	0	0	0	0	0	0
CCM2	13.16597	17.816982	10.369342	12.621635	0	21.355796	181.315065	23.59055
CCM2L	0	0.916963	1.205597	0	0	0.066945	0	0
CCN1	272.393242	702.033749	301.375076	685.014332	133.762848	349.052734	178.76444	266.215929
CCN2	115.578793	104.825636	85.037222	80.744129	70.619343	75.43158	80.173173	38.669325
CCN3	0	0	1.237083	0.2692	31.523217	2.197757	0	3.063034
CCN4	0	1.014293	0	0.076939	0	0	0	0
CCN5	0	1.584879	0	0.25555	0	0	0	0.28684
CCN6	0	0	0	0	0	0.146276	0	0
CCNA1	0	0	0	0.326924	0	0	0	0
CCNA2	40.560724	31.417085	18.709452	6.599526	0	18.81678	49.836667	13.052227
CCNB1	174.849958	324.780304	144.578633	174.523254	205.430922	353.564102	339.755411	510.508485
CCNB1IP1	22.296295	31.32813	58.828504	36.113767	111.922349	12.146536	10.822543	23.252936
CCNB1IP1P1	0	0	0	0	0	0	0	0
CCNB1IP1P2	0	0	0	0	0	0	0	0
CCNB1IP1P3	0	0	0	0	0	0	0	0
CCNB2	115.687461	123.290901	64.475285	83.58603	113.678923	120.083849	47.789143	173.584248
CCNB2P1	0	0	0	0	0	0	0	0

CCNB3	0	0.187325	0	0.426811	0	0.254968	0	0.263004
CCNB3P1	0	0	0	0	0	0	0	0
CCNC	45.639538	35.082796	54.814526	15.179575	55.286339	20.682805	89.617586	28.257854
CCND1	0	4.95893	0	269.35507	0	625.950016	0	118.052014
CCND2	108.141677	74.617409	99.677702	61.519664	273.19577	125.525814	173.102862	163.802924
CCND2P1	0	0	0	0	0	0	0	0
CCND3	0	6.663455	0	4.482688	31.695416	4.026299	19.781189	5.729067
CCND3P1	0	0	0	0	0	0	0	0
CCND3P2	0	0	0	0	0	0	0	0
CCNDBP1	24.368166	27.471842	39.733787	26.634791	29.72609	20.833563	6.454673	22.484198
CCNE1	27.703978	40.252141	43.53337	28.164914	14.428055	38.052233	0	48.418427
CCNE2	12.136269	10.03598	18.247111	12.020242	16.995704	11.803828	50.593691	5.798134
CCNF	20.252671	24.403375	20.977841	23.680561	33.738374	49.710683	13.461995	40.001966
CCNG1	79.228159	35.134412	62.369977	25.234184	55.346606	21.884974	19.24061	47.798673
CCNG1P1	0	0	0	0	0	0	0	0
CCNG2	44.100163	58.039599	50.018739	28.830705	11.765337	20.110869	10.936617	26.285771
CCNG2P1	0	0	0	0	0	0	0	0
CCNH	53.411831	73.102126	98.627497	48.623937	169.685912	48.03637	101.410791	56.134601
CCNHP1	0	0	0	0	0	0	0	0
CCNI	275.261204	118.504598	246.574793	87.535724	130.069024	81.449569	251.356059	88.973348
CCNI2	0	0	0	0	0	0	0	0.862494
CCNJ	5.009173	3.468349	3.746082	2.479427	6.173041	2.950313	0	5.63415
CCNJL	5.370668	1.628407	7.465996	5.37736	2.455892	6.90561	0	1.976928
CCNJP1	0	0	0	0	0	0	0	0
CCNJP2	0	0	0	0	0	0	0	0
CCNK	83.497082	148.580834	82.650111	135.171753	176.875198	94.199631	88.369855	124.57008
CCNL1	48.364406	43.031067	51.818852	35.328696	7.958809	22.813065	15.577489	16.046196
CCNL2	17.302717	11.054083	15.766776	7.279211	22.852565	11.362987	25.776959	8.821312
CCNL2P1	0	0	0	0.192395	0	0	0	0
CCNO	4.087051	12.145561	7.285497	7.214788	1.615313	5.440974	0	3.798835
CCNP	0	0	0	0.826926	0	0.361325	0	0
CCNQ	43.28332	55.493515	13.068182	30.986748	13.579216	40.748478	42.115617	60.344112
CCNQP1	0	0	0	0	0	0	0	0

CCNQP2	0	0	0	0	0	0	0	0
CCNQP3	0	0	0	0	0	0	0	0
CCNT1	19.466245	12.38859	15.449815	16.409713	10.691298	7.171914	27.3604	13.822068
CCNT2	6.32517	6.317477	5.575458	5.399339	5.807449	7.098953	0	13.260953
CCNT2P1	0	0	0	0	0	0	0	0
CCNY	28.532101	60.653579	12.174373	44.88063	14.921361	34.545645	8.016899	64.337767
CCNYL1	22.733596	39.842503	20.039517	25.742404	11.632889	28.828108	152.44667	53.641019
CCNYL2	0	0	0	0	0	0	0	0
CCNYL3	0	0	0	0	0	0	0	0
CCNYL4	0	0	0	0	0	0	0	0
CCNYL5	0	0	0	0	0	0	0	0
CCNYL6	0	0	0	0	0	0	0	0
CCNYL7	0	0	0	0	0	0	0	0
CCP110	42.632487	78.444374	33.29252	71.160597	20.952455	78.839627	0	77.220376
CCPG1	5.173312	20.487169	36.793798	23.298213	27.251594	13.070281	21.434276	17.647954
CCR1	0	0	0	0.062335	0	0	0	0
CCR10	1.792502	1.638281	1.599906	0.658949	0	1.503265	0	0.578761
CCR12P	0	0	0	0	0	0	0	0
CCR2	0	0	0	0	0	0	0	0
CCR3	0	0	0	0.5057	0	0.858509	0	0.560115
CCR5	0	0	0	0	0	0	0	0
CCR6	0	0	0	0.148334	0	0.100568	0	0
CCR7	0	0.582357	0	0.385105	0	0	0	0
CCR8	0	0	0	0	0	0	0	0
CCR9	0	0	0	0	0	0	0	0
CCRL2	0	1.226062	0	1.309857	0	0.33195	0	0.084015
CCS	20.877872	30.150715	26.221947	33.235996	9.385158	35.884291	69.468723	33.042731
CCSAP	6.425448	4.783323	5.626319	3.532366	17.899183	7.563386	9.46588	7.433395
CCSER1	5.042292	5.075845	1.128524	3.85216	0	0.564251	6.426155	1.217694
CCSER2	14.747594	52.374012	41.752328	37.551175	55.0275	35.915653	52.185331	68.179138
CCSER2P1	0	0	0	0	0	0	0	0
CCT2	304.578833	208.222745	272.092626	124.66275	438.819062	191.947333	477.264444	379.722114
CCT3	321.215555	258.388368	236.899666	279.829564	269.654763	314.324154	342.557933	301.625639

CCT3P1	0	0	0	0	0	0	0	0
CCT4	264.530315	290.489178	367.550766	242.066039	295.328918	144.193486	240.497627	257.103294
CCT4P1	0	0	0	0	0	0	0	0
CCT4P2	0	0	0	0	0	0	0	0
CCT5	610.950394	582.192094	509.508781	537.73411	488.546172	385.63678	500.111476	439.712025
CCT5P1	0	0	0	0	0	0	0	0
CCT5P2	0	0	0	0	0	0	0	0
CCT6A	158.883661	142.555246	108.117326	94.367024	308.437954	129.899956	363.592669	274.254254
CCT6B	4.331364	2.87655	3.857841	1.076668	0.570495	0.420852	10.686885	3.467689
CCT6P1	0	0	0	0.068277	0	0	0	0
CCT6P2	0	0	0	0	0	0	0	0
CCT6P3	0	0	0	0	0	0.147825	0	0
CCT6P4	0	0	0	0	0	0	0	0
CCT7	312.654311	248.246808	289.21394	260.606203	277.670692	265.195806	500.411111	260.538067
CCT7P1	0	0	0	0	0	0	0	0
CCT7P2	0	0	0	0	0	0	0	0
CCT8	216.997403	193.951868	196.94535	120.676211	236.228044	91.856118	267.819281	233.863906
CCT8L1P	0	0	0	0	0	0	0	0
CCT8L2	0	0	0	0	0	0	0	0
CCT8P1	0	0	0	0	0	0	0	0.086511
CCZ1	0	4.518748	36.453701	0.278667	0	0	0	0
CCZ1B	77.552154	124.046634	67.743366	94.806908	161.734929	58.319731	142.451333	61.866886
CCZ1P1	0	4.875461	0	1.189848	18.372205	0	0	2.519614
CD101	1.016951	0.304116	0.911682	1.88724	0	1.976239	0	1.202324
CD109	23.921422	17.150424	40.906138	11.302419	44.145575	10.125333	25.476548	20.436923
CD14	0	0	0	0	0.707521	0	0	0.218018
CD151	133.105253	166.324596	167.138557	219.131637	81.30164	188.192973	92.50298	125.816629
CD160	0	0	0	0	0	0	0	0
CD163	0	0	0	0	0	0.461916	0	0.074357
CD163L1	0	7.344844	12.014371	4.219798	0	1.952258	0	2.224716
CD164	73.310044	73.37691	127.439449	68.081474	60.420109	51.643811	68.107349	63.412327
CD164L2	0	0	0	0.359138	0	0	0	0
CD177	0	0	0	0.152372	0	0.854394	0	1.200265

CD177P1	0	0	0	0	0	0	0	0
CD180	0	0	0	0	0	0	0	0
CD19	0	0	0	0.340213	0	0	0	0
CD1A	0	0	0	0	0	0	0	0
CD1B	0	0	0	0	0	0	0	0
CD1C	0	0	0	0	0	0	0	0
CD1E	0	0	0	0	0	0	0	0
CD2	0	0	0	0	0	0	0	0
CD200	0	0	0	0.113809	0	0	0	0
CD200LP	0	0	0	0	0	0	0	0
CD200R1	0	0.352111	0	0	0	0	0	0
CD200R1L	0	0	0	0	0	0	0	0
CD207	0	0	0	0.045462	0	0	0	0
CD209	0	0.032947	0	0.018773	0	0	0	0
CD22	0	0	0	0.059712	0	0	0	0
CD226	0	0.033623	0	0.436622	0.686615	0	0	0.130174
CD24	408.804546	229.068077	228.462555	162.843882	300.853221	109.795354	268.347053	204.721737
CD244	0	0	0	0	0	0	0	0
CD248	0	0	0	0	0	0	0	0
CD24P1	0	0	0	0	0	0	0	0
CD24P2	0	0	0	0	0	0	0	0
CD24P4	0	0	0	0	0	0	0	0
CD24P5	0	0	0	0	0	0	0	0
CD27	0	0	0	0.175776	0	0.177264	0	0
CD274	17.920333	3.685835	1.638841	3.020579	31.176527	5.498703	0	8.992711
CD276	67.831399	46.563724	115.545143	48.751393	92.342484	61.280593	86.36443	43.497837
CD28	0	0	0	0	0	0	0	0
CD2AP	26.525387	41.274423	22.210511	35.260868	49.053813	40.293174	35.105948	65.216541
CD2BP2	65.475571	90.744647	36.603698	98.276609	80.113759	108.160008	32.130833	89.774877
CD300A	0	0	0	0	0	0	0	0
CD300E	0	0	0	0	0	0	0	0
CD300H	0	0	0	0	0	0	0	0
CD300LB	0	0	0	0	0	0	0	0

CD300LD	0	0	0	0	0	0	0	0
CD300LF	0	0	0	0	0	0	0	0
CD300LG	0	0	0	0	0	0	0	0
CD302	0.870846	1.152204	3.53565	1.017089	0.229953	0	0	0.623426
CD320	13.958392	24.537465	31.363637	26.423735	8.528251	30.254603	0	20.926683
CD33	0	0	0	0	0	0	0	0
CD34	0	0	0	0	0	0.416737	0	0
CD36	0.714013	1.288722	0	0.453121	0	0	0	0
CD37	0	0	0	0	0	0	0	0
CD38	0	1.206293	0	0.104162	0	0.174331	0	0.093018
CD3D	0	0	0	0	0	0	0	0
CD3E	0	0.237891	0	0.344024	0	0	0	0
CD3G	0	0	0	0	0	0	0	0.257424
CD4	0	0	0	0	0	0	0	0
CD40	7.065001	0	15.784247	5.963244	4.61449	7.436384	10.795504	0.667488
CD40LG	0	0	0	0	0	0	0	0
CD44	494.367816	439.024983	699.624383	728.204123	693.208817	674.534794	579.978198	458.553042
CD46	115.106707	53.023479	230.084403	56.582438	58.520375	15.908406	52.275935	45.448384
CD46P1	0	0	0	0	0	0	0	0
CD47	47.959582	117.31338	63.539086	77.994483	35.237014	10.062911	235.575941	48.566461
CD48	0	0	0	0	0	0	0	0
CD5	0	0	0	0.026106	0	0	0	0
CD52	0	0	0	0	0	0	0	0
CD53	0	0	0	0	0	0	0	0
CD55	36.796497	32.919053	27.771758	39.201317	69.272102	26.203168	49.280634	46.231164
CD58	56.446236	29.187588	43.399614	8.572269	11.972122	2.564578	32.639943	9.806426
CD59	257.545621	229.049138	250.357896	312.428648	348.871421	301.489494	126.170801	360.33158
CD5L	0	0	0	0.189624	0	0	0	0
CD6	0	0.142145	0	0.075146	2.07857	0.768325	0	0.289069
CD63	293.112043	632.118198	361.308675	756.153923	245.992635	330.065036	298.965523	292.322278
CD68	75.123147	26.115552	71.59641	71.120169	90.094926	28.755251	0	13.068051
CD69	2.11199	0.266837	0	0.307077	0	0	0	0.16535
CD7	0	0	0	0	0	0.279932	0	0

CD70	0	1.596406	0	2.810477	0	4.180008	0	0.708788
CD72	0	0	0	0.405092	0	0.476703	0	0
CD74	0	0	0	0	10.451937	5.635964	0	1.376823
CD79A	0	0	0	0	0	0	0	0
CD79B	0	0	0	0	0	0	0	0.131395
CD80	0	0	0	0	0	0	0	0
CD81	81.241774	168.733986	52.21939	155.706771	64.703235	195.511885	96.690142	161.020509
CD82	19.976838	32.130558	45.93168	61.897417	14.665378	77.221373	27.114656	54.414392
CD83	0	0	0	17.303237	0	0	1.425557	19.543957
CD83P1	0	0	0	0	0	0	0	0
CD84	0	0	0	0	0	0	0	0
CD84P1	0	0	0	0	0	0	0	0
CD86	0	0	0	0.031319	0	0	0	0.104578
CD8A	5.272558	0.281747	0	0.946851	0	0	0	0
CD8B	0	2.415471	0	2.587165	0	0	0	0
CD8B2	0	0	0	0.183691	0	0	0	0
CD9	258.662211	201.453883	270.944726	252.809789	121.528164	103.876867	100.859552	86.915564
CD93	0	0	0	0	0	0	0	0
CD96	0	0	0	0	0	0	0	0
CD99	64.807014	155.906227	85.5511	189.413415	190.223184	299.689958	197.216755	244.783422
CD99L2	6.82904	14.775336	3.416574	11.607483	5.65719	13.115579	0.231604	7.831632
CD99P1	0	0.61848	1.083769	0.312832	0	0.849638	0	0.785124
CDA	20.158541	16.498871	8.833183	28.211016	201.173368	131.679232	214.714251	142.111756
CDADC1	0.984242	10.608447	5.555681	9.825502	4.157462	6.829109	0	8.693481
CDAN1	5.087327	6.807693	3.163911	4.439233	3.582402	7.545787	0	4.959448
CDC123	77.592816	57.524506	48.029195	47.247434	37.188245	33.91404	63.34396	52.027584
CDC14A	14.930134	19.490835	6.990894	8.670511	27.035813	2.309178	4.738973	7.091543
CDC14B	0	9.976787	0	9.528032	12.487884	10.633783	62.629807	10.832854
CDC16	33.716016	54.051671	40.620033	31.212793	26.657621	23.828585	16.371189	47.859167
CDC20	120.074683	67.255963	55.517688	57.715743	194.796578	188.212488	180.758041	180.937369
CDC20B	0	0.388163	0	0.039799	0	0	0	0.280458
CDC20P1	5.207153	5.296812	2.178667	1.656177	33.548255	7.926341	14.483539	7.235231
CDC23	45.655879	13.485161	18.197565	11.267659	50.006627	5.80575	20.516534	19.406562

CDC25A	10.6225	14.423221	7.393836	13.607825	56.963963	20.347508	13.000398	21.799179
CDC25B	42.211108	33.103795	11.956006	34.462916	40.766427	58.973325	46.112061	57.201165
CDC25C	28.789541	10.895237	12.724716	13.343852	33.387963	10.82866	0	20.303585
CDC26	91.896401	145.419594	44.316485	103.86372	55.310059	57.01517	75.316107	122.658897
CDC26P1	161.794319	218.884245	0	219.055042	0	159.02416	127.037467	34.225989
CDC27	65.978059	82.662579	50.317245	68.111808	150.611435	44.860108	150.729	94.112935
CDC27P1	0	0	0	0	0	0	0	0
CDC27P2	0	0	0	0	0	0	0	0
CDC27P3	0	0	0	0	0	0	0	0
CDC34	79.043303	96.967677	52.193875	93.190119	74.092629	160.128746	32.084693	119.247323
CDC37	70.316414	191.352174	81.074463	240.116298	106.844179	346.737335	64.256372	212.195018
CDC37L1	5.977613	13.87415	13.043462	11.76352	2.509252	18.734255	15.823313	24.270245
CDC37P1	0	0	0	0	0	0	0	0
CDC37P2	0	0	0	0	0	0	0	0
CDC40	20.921591	11.703805	12.312027	11.574221	16.757643	5.389788	0	11.75929
CDC42	28.087246	39.830655	0	14.946332	52.350242	9.955065	1.954209	21.01975
CDC42BPA	18.234796	36.783227	10.635134	21.525976	11.555235	17.619813	25.294287	27.514421
CDC42BPB	21.295613	43.331844	25.533774	41.839539	32.513839	68.012472	14.503957	48.84419
CDC42BPG	1.581928	3.741067	5.214476	5.18449	4.179849	11.343182	9.51166	5.854004
CDC42EP1	40.11321	105.267509	32.974024	109.95372	22.848044	84.237934	14.383796	63.348146
CDC42EP2	12.399429	9.86525	12.649709	18.965258	49.383795	33.575238	0	28.423295
CDC42EP3	15.907437	246.373783	25.220122	175.990439	31.001585	284.227212	56.336221	328.901472
CDC42EP3P1	0	0	0	0	0	0	0	0
CDC42EP4	25.956115	71.080232	18.414764	68.587048	42.249176	196.409267	51.509355	155.966593
CDC42EP5	9.305595	67.284106	78.25475	60.819369	0	81.20475	0	61.657224
CDC42P1	0	0	0	0	0	0	0	0
CDC42P2	0	0	0	0	0	0	0	0
CDC42P3	0	0	0	0	0	0	0	0
CDC42P4	0	0	0	0	0	0	0	0
CDC42P5	0	0	0	0	0	0	0	0
CDC42P6	0	0	0	0	0	0	0	0
CDC42P7	0	0	0	0	0	0	0	0
CDC42SE1	101.486279	169.54608	58.759606	191.99949	63.684919	188.268343	129.498383	146.147262

CDC42SE2	31.45093	75.491274	27.245112	43.1854	26.25713	27.287749	17.471662	65.969407
CDC45	0	10.632126	0	15.014304	45.037012	38.567067	18.72602	10.731349
CDC5L	22.994201	19.872069	18.712045	17.727186	30.660264	19.534027	14.71383	24.078423
CDC6	100.654623	127.75495	55.751223	103.798794	123.08533	123.060723	32.334726	116.017092
CDC7	74.459098	43.400458	28.892365	32.206452	14.546948	9.369269	6.823416	18.695968
CDC73	52.290959	176.708147	47.0164	130.882326	54.798145	104.652746	55.90826	168.991091
CDCA2	23.64818	26.609307	11.983219	18.30654	53.913961	21.456196	48.739351	24.356285
CDCA3	30.725659	25.295339	17.129743	29.093912	60.220371	56.954767	40.936009	37.050621
CDCA3P1	0	0	0	0	0	0	0	0
CDCA4	13.179797	35.760623	15.640414	38.463648	30.924005	56.048426	67.221648	41.349508
CDCA4P1	0	0	0	0	0	0	0	0
CDCA4P2	0	0	0	0	0	0	0	0
CDCA4P3	0	0	0	0	0	0	0	0
CDCA4P4	0	0	0	0	0	0	0	0
CDCA5	23.384359	72.227124	30.143044	48.095424	84.534026	173.556415	88.12894	142.782654
CDCA7	35.857654	54.21591	40.176698	45.797293	113.532568	50.683835	45.802561	67.460925
CDCA7L	148.143262	374.569573	135.425012	435.098249	119.337156	300.511975	140.080718	347.73269
CDCA7P1	0	0.41976	0	0	0	0	0	0
CDCA7P2	0	0	0	0	0.900013	0	0	0
CDCA8	20.173314	43.734624	24.485092	36.466746	64.329536	88.322823	76.527364	90.994549
CDCP1	65.444988	88.598786	98.930354	120.389857	40.635301	64.794697	40.941199	56.338634
CDCP2	0	0	0	0	0	0	0	0
CDH1	74.032587	64.886522	136.400005	135.206452	58.939596	66.89667	84.264633	52.109934
CDH10	1.179882	0.53823	0	0.574005	0	0.241629	0	0.04747
CDH11	17.300932	27.572885	20.467759	76.298644	35.050048	111.423529	58.005264	106.447317
CDH12	0	0	0	0	0	0	0	0
CDH12P2	0	0	0	0	0	0	0	0
CDH12P3	0	0	0	0	0	0	0	0
CDH12P4	0	0	0	0	0	0	0	0
CDH13	58.214015	126.441446	109.367382	202.441402	113.018255	163.969416	79.46501	183.724333
CDH15	0	0	0	0.143085	0	0	0	0
CDH16	0	0	0	0	0	0	0	0
CDH17	0	0	0	0	0	0	0	0

CDH18	0	1.310043	0	0.364187	0	0	0	0
CDH19	0	0.355551	0	0.549712	0	0	0	0
CDH2	16.448902	30.788049	20.748585	34.058257	11.945545	21.172384	6.254321	8.639437
CDH20	0	0	0	0	0	0	0	0
CDH22	0	0	0	0	0	0	0	0
CDH23	6.891699	2.881846	3.751026	1.960795	0	0.118105	0	1.249277
CDH24	4.895452	5.850152	4.383552	3.019414	9.899955	5.628456	0	5.720576
CDH26	0	2.214931	0	0	0	0	0	0
CDH3	203.041941	185.297806	221.869568	309.948777	248.920121	264.774884	185.864487	117.154283
CDH4	4.8553	12.31986	4.364104	8.696597	0.320676	3.382069	0	1.614634
CDH5	0	0	0	0	0	0	0	0
CDH6	40.154547	100.021243	35.988354	76.763017	0.09969	0.111971	0	0.0532
CDH7	0	0.649808	0	1.248872	0	1.996042	0	1.466548
CDH8	10.359179	55.520118	12.214584	61.754913	0.656708	12.396443	0	20.076224
CDH9	0	0	0	0	0	0	0	0
CDHR1	0	1.682509	0	0.288	0	3.04947	0	0.566304
CDHR17P	0	0	0	0	0	0	0	0
CDHR18P	0	0	0	0	0	0	0	0
CDHR2	0	0.070652	0	0.080546	0	0	0	1.341414
CDHR3	0	4.432889	0	3.892836	0	3.194563	0	3.594854
CDHR4	0	1.091323	0	0.181554	0	0	0	0
CDHR5	0	0	0	0	0	0	0	0
CDIN1	26.088256	50.48947	17.000963	43.767426	39.85521	29.11551	0	26.964837
CDIP1	33.006519	24.340606	22.025048	20.912127	23.774671	52.375644	89.889039	33.935019
CDIPT	26.592191	44.617799	38.752977	53.479124	19.676119	56.735395	57.848754	55.414153
CDIPTOSP	0	2.96411	0	0	0	0	0	0
CDK1	15.318798	31.901276	42.979805	5.789812	0	53.661076	68.122513	15.893324
CDK10	17.915502	30.456901	30.006621	40.068405	57.315918	50.871071	3.346017	44.230333
CDK11A	2.463976	6.517145	23.217318	14.614845	8.398537	18.052896	34.613061	11.039055
CDK11B	0	19.682838	27.257662	27.7407	0	37.859174	47.181428	23.072273
CDK12	22.572735	63.082002	28.907713	66.701628	42.591137	79.181182	44.801321	60.3053
CDK13	12.4056	33.685516	17.410847	34.947716	44.030356	46.928883	103.329113	77.7567
CDK14	8.847726	20.832441	7.381432	24.918513	29.595927	45.567406	8.671127	82.929169

CDK15	0	0	0	0	0	0	0	0.036437
CDK16	83.975721	199.1009	100.988249	185.716872	171.04585	251.456292	142.174312	194.011239
CDK17	47.455009	124.729538	40.454628	100.112779	18.581665	147.396417	140.181468	198.944415
CDK18	7.46046	17.842768	12.417489	19.371173	7.877099	27.485107	3.88507	13.626273
CDK19	8.375767	5.798576	1.459622	7.724837	2.717174	6.73404	3.166193	12.674987
CDK2	105.317003	162.109136	138.548333	183.234769	70.080109	103.605315	79.874415	91.680659
CDK20	2.767988	1.129402	3.354666	7.377188	0.964159	1.643452	8.641834	0.738688
CDK2AP1	46.059568	55.435166	27.40679	21.546003	78.185613	42.417229	42.316583	59.998878
CDK2AP1P1	0	0	0	0	0	0	0	0
CDK2AP2	38.59018	55.122803	25.34569	32.284809	2.570952	36.246036	0	22.783291
CDK2AP2P1	0	0	0	0	0	0.871346	0	0
CDK2AP2P2	0	0	0	0	0	0	0	1.438685
CDK2AP2P3	0	0	0	0	0	0	0	0
CDK3	0	0	0	0	0	0	0	0
CDK4	311.931539	401.014889	264.433351	337.346793	420.973785	701.006195	412.560587	626.896329
CDK4P1	0	0	0	0	0	0	0	0
CDK5	6.717666	13.920628	0	16.160776	17.502241	24.813757	0	27.23541
CDK5P1	0	0	0	0	0	0	0	0
CDK5R1	4.186509	11.080815	3.005581	8.073799	1.989981	16.894864	0	10.760831
CDK5RAP1	59.261166	23.318976	34.145267	28.951908	60.690933	30.171272	66.956396	19.408346
CDK5RAP2	19.677193	26.247775	47.657248	33.258906	7.789397	33.141643	56.627896	19.581495
CDK5RAP3	47.745007	28.631099	37.476386	49.933365	28.689673	38.663767	203.273797	41.057956
CDK5RAP3P1	0	0	0	0	0	0	0	0
CDK6	37.308787	57.77766	34.516328	44.098874	49.67486	53.336618	152.849904	133.186258
CDK7	88.213986	86.647562	54.117487	85.769881	27.169674	39.17309	56.892573	48.588497
CDK7P1	0	0	0	0	0	0	0	0
CDK8	11.065642	25.297982	11.898171	17.806272	8.762052	15.23254	25.66365	24.150036
CDK8P1	0	0	0	0	0	0	0	0
CDK8P2	0	0	0	0	0	0	0	0
CDK9	0	47.388016	0	46.428677	0	56.171852	32.826436	43.506645
CDKAL1	16.128493	12.014765	7.708894	12.227156	37.705395	7.409282	14.109552	11.234113
CDKL1	2.625853	54.396202	0	45.100536	0	43.133392	0	86.102673
CDKL2	0.985165	0.799991	1.766692	1.817918	0	0.19659	0	2.976529

CDKL3	3.107186	4.599497	0	1.388925	0	0.362974	0	1.392603
CDKL5	0	1.525091	0	1.920062	2.968168	4.525942	0	5.775584
CDKN1A	0	2.32189	0	4.180123	0	6.08811	58.902054	7.247497
CDKN1B	16.980891	45.862375	18.985982	43.435258	32.77957	76.114169	11.669298	74.097228
CDKN1C	0	2.072725	8.298815	3.726872	13.507786	0.875888	0	0.214369
CDKN2A	164.262155	199.096624	133.127179	179.657324	233.877336	297.04404	290.9606	163.755278
CDKN2AIP	26.638724	19.183904	3.43744	18.426663	0.675488	12.903868	13.44614	19.507156
CDKN2AIPNL	27.161404	26.506667	47.538403	19.827291	20.637515	22.198751	24.664949	29.446672
CDKN2AIPNL	0	0	0	0	0	0	0	0
CDKN2AIPNL	0	0	0	0	0	0	0	0
CDKN2AIPNL	0	0	0	0	0	0	0	0
CDKN2B	6.871834	32.029923	9.372413	21.248946	20.571501	71.948853	44.821419	72.687314
CDKN2C	13.489096	50.410647	11.901482	48.210976	5.269925	24.183531	9.68169	25.853514
CDKN2D	3.492828	4.802035	6.175719	2.210735	18.356986	9.418711	0	5.435174
CDKN3	118.45157	116.876107	81.434123	69.830416	86.25508	86.026233	72.553358	158.488171
CDNF	0	4.739954	4.642388	1.67287	0	0	0	0.182322
CDO1	0	2.431298	0	0.386666	0	0.336624	0	0
CDON	0	0.126649	0	0.598876	1.24788	0.657256	3.833614	1.250769
CDPF1	5.021399	5.252081	0	9.817711	0	8.481925	0	9.587344
CDPF1P1	0	0	0	0	0	0	0	0
CDR2	32.514823	88.750713	38.067513	86.229157	32.33898	73.186391	23.096271	62.551303
CDR2L	7.521628	14.273474	6.74597	13.146496	8.44	27.687452	11.203797	21.232523
CDRT15	0	0	0	0	0	0	0	0
CDRT15L2	0	0	0	0	0	0	0	0
CDRT15P1	0	0	0	0	0	0	0	0
CDRT15P10	0	0	0	0	0	0	0	0
CDRT15P11	0	0	0	0	0	0	0	0
CDRT15P12	0	0	0	0	0	0	0	0
CDRT15P13	0	0	0	0	0	0	0	0
CDRT15P14	0	0	0	0	0	1.554054	0	0
CDRT15P2	0	0	0	0	0	0	0	0
CDRT15P3	0	0	0	0	0	0	0	0
CDRT15P4	0	0	0	0	0	0	0	0

CDRT15P5	0	0	0	0	0	0	0	0
CDRT15P6	0	0	0	0	0	0	0	0
CDRT15P7	0	0	0	0	0	0	0	0
CDRT15P8	0	0	0	0	0	0	0	0
CDRT15P9	0	0	0	0.673902	0	0.440296	0	0
CDRT4	0	0	0	0	0	0	0	0
CDS1	25.213066	12.81156	15.090436	9.329642	3.026818	5.569963	3.533816	12.238694
CDS2	32.884061	52.293011	28.203896	39.133169	25.063938	39.826538	110.23795	48.293605
CDSN	0	0	0	0	0	0.400391	0	0.482875
CDT1	36.851382	34.033039	78.491523	44.468182	26.82389	34.819299	38.865601	21.153998
CDV3	253.062168	388.638755	236.663353	343.540818	302.961744	334.77855	232.113193	332.726914
CDV3P1	0	0	0	0	0	0	0	0
CDX2	0	0	0	3.575744	0	0	0	0
CDY1	0	0	0	0	0	0	0	0
CDY10P	0	0	0	0	0	0	0	0
CDY11P	0	0	0	0	0	0	0	0
CDY12P	0	0	0	0	0	0	0	0
CDY15P	0	0	0	0	0	0	0	0
CDY17P	0	0	0	0	0	0	0	0
CDY18P	0	0	0	0	0	0	0	0
CDY19P	0	0	0	0	0	0	0	0
CDY1B	0	0	0	0	0	0	0	0
CDY20P	0	0	0	0	0	0	0	0
CDY22P	0	0	0	0	0	0	0	0
CDY23P	0	0	0	0	0	0	0	0
CDY2B	0	0	0	0	0	0	0	0
CDY3P	0	0	0	0	0	0	0	0
CDY4P	0	0	0	0	0	0	0	0
CDY5P	0	0	0	0	0	0	0	0
CDY6P	0	0	0	0	0	0	0	0
CDY7P	0	0	0	0	0	0	0	0
CDY8P	0	0	0	0	0	0	0	0
CDYL	84.729812	112.081872	63.664907	142.834659	75.578806	188.473621	42.957676	174.277315

CDYL2	0.382359	2.920585	0	0.187174	2.02112	11.095782	28.384219	5.714696
CDYLP1	0	0	0	0	0	0	0	0
CEACAM1	5.991215	0.766126	16.153114	5.754798	6.964961	6.115258	9.826981	2.068724
CEACAM16	0	0	0	0	0	0	0	0
CEACAM19	4.891057	0.674744	18.843819	1.565293	0	1.046471	0	1.166318
CEACAM20	0	0	0	0	0	0	0	0
CEACAM21	0	0	0	0	0	0	0	0
CEACAM22P	0	0	0	0.054245	0	0	0	0
CEACAM3	0	0	0	0	0	0	0	0
CEACAM4	0	0	0	0	0	0	0	0
CEACAM5	0	0	0	0.287028	0	0	0	0
CEACAM6	0	0	0	0	0	0	0	0
CEACAM7	0	0	0	0	0	0	0	0
CEACAM8	0	0	0	0	0	0	0	0
CEACAMP1	0	0	0	0.088898	0	0	0	0
CEACAMP10	0	0	0	0	0	0	0	0
CEACAMP11	0	0	0	0	0	0	0	0
CEACAMP2	0	0	0	0	0	0	0	0
CEACAMP3	0	0	0	0	0	0	0	0
CEACAMP4	0	0	0	0	0	0	0	0
CEACAMP5	0	0	0	0.388655	0	0	0	0
CEACAMP6	0	0	0	0	0	0	0	0
CEACAMP7	0	0	0	0	0	0	0	0.84649
CEACAMP8	0	0	0	0	0	0	0	0
CEACAMP9	0	0	0	0.444136	0	0	0	0
CEBPA	5.215416	8.785207	1.166985	8.475531	0	10.565002	0	4.514224
CEBPG	42.17215	57.187876	47.51512	56.763977	20.886373	37.805445	10.810186	49.063539
CEBPZ	79.735338	63.674094	61.65995	40.60621	43.152003	26.747245	222.115414	58.285237
CEBPZOS	40.740377	39.509458	46.805323	43.872331	30.747593	13.894017	11.991437	21.579039
CECR2	0	0.057444	0	0.04559	0	0	0	0
CECR7	0	0	0	0	0	0	0	0
CELA2A	0	0	0	0	0	0	0	0
CELA2B	0	0	0	0	0	0	0	0

CELA3A	0	0	0	0	0	0	0	0
CELA3B	0	0	0	0	0	0	0	0
CELF1	69.028413	111.042004	43.061901	106.032347	89.782605	177.014494	207.003365	242.542924
CELF2	0	0	0	0.129519	0	0	0	0
CELF3	0	0	0	0	0	0	0	0
CELF4	0	0	0	0	0	0	0	0
CELF5	0	0.284886	0	0.216202	0	0.219316	0	0
CELF6	0	0	0	0	0	0	0	0.164602
CELP	0	0	0	0	0	0	0	0
CELSR1	20.071474	2.228173	0	0	4.784773	0	90.119557	4.667757
CELSR1P1	0	1.958877	0	1.520137	0	1.170313	0	1.621814
CELSR2	17.346151	37.480267	22.484171	43.563903	9.60755	49.1731	7.601223	16.698049
CELSR3	3.219168	4.36356	5.313473	6.20946	0.425496	4.417832	0	0.676783
CEMIP	0	0.597205	0	0.394221	2.602063	4.618361	4.80247	1.937432
CEMIP2	19.804628	9.568009	2.37378	15.997912	76.865621	36.219328	18.17382	34.13519
CEMP1	0	0	0	0	0	0.389071	0	0
CENATAC	67.654517	86.254718	55.767485	69.268596	3.098301	18.119348	11.659196	17.926685
CENATACP1	0	0	0	0	0	0	0	0
CEND1	0	0	0	0	0	0.218254	0	0.089694
CEND1P1	0	0	0	0	0	0	0	0
CENPA	23.533586	59.229387	22.568042	36.789958	3.896607	49.912254	111.946661	55.957946
CENPB	50.078814	119.487311	30.252609	87.320102	49.792263	246.074585	85.991896	182.238146
CENPBD1P	7.178171	14.801542	14.415385	16.155658	15.137672	11.764602	0	0
CENPBD2P	0	0	0	5.316417	0	14.924275	0	14.511479
CENPC	12.213675	7.190635	9.559334	4.449652	9.222871	3.036894	8.779302	5.593911
CENPCP1	0	0	0	0	0	0	0	0
CENPE	0	2.20171	0	0	0	0	0	2.500929
CENPF	23.702553	35.389847	32.843759	46.935133	50.063499	83.76207	54.259215	79.564947
CENPH	45.988316	39.005164	81.311307	31.835192	74.814937	17.152231	22.698061	43.253463
CENPI	2.421285	19.552013	12.236292	14.290502	76.714662	17.573098	63.49545	23.337825
CENPIP1	0	0	0	0	0	0	0	0
CENPJ	5.791167	1.893198	0	1.072815	0	2.986758	18.966965	0.688686
CENPK	38.645693	99.563309	39.079801	41.317625	8.757499	32.761117	16.747307	81.645444

CENPL	25.58984	50.384847	13.99399	39.957172	57.139581	35.184452	32.901194	53.968599
CENPM	4.968143	11.605263	14.717826	12.2438	0	14.388939	0	11.889926
CENPN	138.672205	178.683025	54.470499	106.214947	241.602595	148.62473	171.518207	264.713196
CENPNP1	0	0	0	0	0	0	0	0
CENPNP2	0	0	0	0	0	0	0	0
CENPO	19.040889	58.793155	10.151831	64.591946	48.615797	107.165606	2.588936	106.2267
CENPP	4.664128	16.874034	6.785265	18.452729	1.951768	5.338786	0	5.544618
CENPPP1	0	0	0	0	0	0	0	0
CENPQ	16.190748	13.56761	14.432257	7.065833	8.532341	5.788977	0	10.549576
CENPS	34.845725	97.226178	42.076111	67.761754	39.934573	58.432495	37.104939	99.670621
CENPS-CORT	0	5.583843	0	3.394323	0	2.170683	35.10038	3.667194
CENPT	7.183055	10.863006	12.573078	15.473729	16.279089	31.372703	102.572021	15.616215
CENPU	23.192053	58.292756	37.579166	28.089801	10.487275	14.037976	64.603701	38.529547
CENPUP1	0	0	0	0	0	0	0	0
CENPUP2	0	0	0	0	0	0	0	0
CENPV	52.880843	31.403325	26.143212	27.544552	21.840099	19.140039	0	23.138278
CENPVL1	6.582985	7.338042	1.95412	4.135317	0	0	0	0
CENPW	0	13.911182	0	8.250623	0	39.329048	74.355521	31.45584
CENPX	65.85391	98.010194	109.448664	100.775364	92.739013	106.165386	2.873667	87.319301
CEP104	7.188397	24.301993	8.165069	17.181658	59.096455	33.630316	5.657849	30.499543
CEP112	0	10.701445	1.636762	2.104868	0	0.915685	0.443831	2.160166
CEP120	0	0.630433	0	1.783058	0	2.983261	0	0.470247
CEP126	3.928011	13.929494	13.026305	5.624175	0	1.060857	0	0.426803
CEP128	37.348028	20.892257	11.248155	32.732883	20.574761	25.274979	44.613313	23.464357
CEP131	10.634069	9.253768	24.652171	9.934774	14.554035	12.796524	0	7.807479
CEP135	2.584075	10.287396	0	0.933327	0	4.363304	57.582975	3.938988
CEP15	59.680738	16.350994	19.680032	2.945769	0	0.05105	0	7.682212
CEP152	13.642826	8.749608	20.100224	20.289981	49.742606	12.321714	50.358224	11.26247
CEP162	5.167749	2.571127	11.729078	1.953805	6.690415	2.086281	0	2.907378
CEP164	26.703594	28.999337	17.701028	54.319987	27.5946	63.525979	56.290912	43.988268
CEP164P1	0	0	0	0	0	0	0	0
CEP170	51.408432	122.643047	36.005414	117.294294	72.612043	65.06336	52.413346	82.391269
CEP170B	12.608924	17.163853	13.082268	16.324958	17.675166	44.41435	21.756318	23.783112

CEP170P1	0	1.279176	0	1.068789	0	0.915172	0	0.39802
CEP19	3.188921	0	0	1.254407	0	4.378581	0.765812	0
CEP192	8.917068	8.565566	13.88041	6.963875	17.257803	4.57151	7.504992	9.840933
CEP192P1	0	0	0	0	0	0	0	0.067143
CEP19P1	0	0	0	0	0	0	0	0
CEP20	62.742224	54.625262	37.923221	32.441208	21.805063	17.463907	12.341148	21.12569
CEP250	59.592036	137.354649	73.470679	204.377163	42.014336	175.185341	53.646052	102.352612
CEP290	0	7.424961	13.00203	5.604472	6.797415	4.202892	12.674656	7.003242
CEP295	40.251678	35.036008	39.596882	24.697726	120.532465	7.033366	16.29924	12.731314
CEP295NL	0	0	0	0	0	0	0	0
CEP350	15.988763	15.107125	15.669119	14.913785	13.776305	11.366754	5.816662	18.586005
CEP41	2.506812	7.47451	10.871383	4.726792	0.450772	1.986417	0	4.617395
CEP43	3.245714	1.576015	3.25336	1.266196	0.195884	1.44931	0	2.143264
CEP44	2.487067	16.040701	9.628567	21.961606	6.401594	13.977132	0	20.322592
CEP55	40.641354	9.327113	0	0.320117	0	0.6291	38.166906	40.094742
CEP57	121.672258	131.729276	205.663152	110.312288	87.7287	45.358886	96.33723	74.221795
CEP57L1	20.957832	41.167792	7.521101	26.996604	22.208393	21.702614	0	28.623523
CEP57L1P1	0	0	0	0	0	0	0	0
CEP63	0	0	0	3.817647	0	7.20995	0	5.485284
CEP68	5.048129	8.499125	8.066354	10.602615	10.686112	9.753505	10.653461	8.708252
CEP70	36.355895	41.437989	33.477915	22.461021	23.562326	23.09708	6.673818	36.976343
CEP72	11.554018	8.995398	10.332939	13.712069	4.571523	18.780275	27.398246	15.420254
CEP76	17.013391	30.354312	15.770324	6.041009	11.589392	5.088702	30.115718	10.727461
CEP78	29.190912	18.031737	15.053705	12.27024	24.613931	14.525966	10.742979	31.357535
CEP83	14.301547	13.599615	13.755936	12.692772	15.416611	13.301811	72.242307	21.056858
CEP85	0	1.238965	10.053599	5.521878	0	6.453491	3.814479	7.239825
CEP85L	5.536021	4.867619	16.959039	8.634459	1.315402	7.543035	0	6.837095
CEP89	13.281072	31.737328	19.674932	39.319714	50.598152	56.383515	18.567125	37.751353
CEP95	21.365529	35.261973	17.609083	31.693368	15.208502	27.481996	83.751723	25.777884
CEP97	11.364562	7.348722	8.069581	6.060707	9.677598	5.385305	0	7.744501
CEPT1	24.117989	35.374486	22.44051	41.244978	5.843489	15.711515	16.643269	18.377068
CERCAM	11.633069	21.302084	47.500397	27.37504	49.036709	22.759857	54.870589	15.713896
CERK	17.764074	19.644742	15.950302	22.463599	13.880088	22.826925	8.469667	25.498098

CERKL	0	0.133436	0	0.132766	0	0	0	0
CERS1	0	3.911873	4.786247	3.350501	6.125348	2.142355	15.141646	0.437839
CERS2	0	96.637076	181.497672	89.946118	68.311073	48.710906	89.950879	74.062032
CERS3	0	0	0	0	0	0	0	0.874921
CERS4	11.973438	17.829943	6.79584	9.115305	33.57247	7.435651	1.415716	12.010173
CERS5	1.856181	1.288563	0	4.560404	7.234127	1.028115	0	3.038142
CERS6	20.800516	4.831544	25.507038	0	0	3.618561	32.044915	2.915309
CERT1	32.61405	21.044887	29.418002	28.167703	41.392571	16.991464	57.160518	31.127601
CES1	0	0	0	0	0	0	0	0
CES1P1	0	0	0	0	0	0	0	0
CES1P2	0	0	0	0	0	0.14534	0	0
CES2	4.912359	37.27696	2.693511	54.108622	35.591118	83.120281	90.456706	31.192521
CES3	0	0.314632	0	0.148213	0	0.472254	0	0.169286
CES4A	0	0	0	0.269012	0	0	0	0
CES5A	0	0	0	0	0	0	0	0
CES5AP1	0	0	0	0	0	0	0	0
CETN2	0	0	0	0	0	0	0	0
CETN3	96.505382	114.027373	77.860395	43.16478	21.69218	15.918391	6.935649	55.841817
CETN4P	0	0	0	0	0	0	0	0
CETP	0	0	0	0	0	0	0	0
CFAP100	0	0	0	0	0	0	0	0
CFAP107	0	0	0	0	0	0	0	0
CFAP119	3.617085	7.678982	0	6.514536	1.054402	8.998533	0	6.085203
CFAP141	0	0	0	0	0	0	0	0
CFAP144	0	2.662391	0	0.381113	0	0.728242	0	2.990315
CFAP144P2	0	0	0	0	0	0	0	0
CFAP157	0	0.053179	0	0	0	0.096479	0	0
CFAP161	0	1.09507	0	0.567148	0	1.11957	0	0.33803
CFAP20	40.670134	121.482139	99.022406	153.661865	73.29503	197.625584	52.513338	142.584213
CFAP206	0	0.528627	0	0.178016	0	0.176281	0	0
CFAP20DC	1.27795	2.08731	3.28513	0.912958	6.131458	2.022444	0	5.645725
CFAP210	0	0.27141	0	0.857436	0	2.51261	0	1.993001
CFAP221	0	0	0	0	0	0	0	0

CFAP251	0.973902	6.306396	7.099534	3.823822	9.580079	6.002424	6.867178	7.332565
CFAP263	21.242268	14.49436	16.140584	17.306438	3.245336	5.821194	39.343649	5.767194
CFAP276	0	0	0	0	0	0	0	0
CFAP298	18.610204	73.765119	42.009175	55.853535	27.742187	62.005217	65.018644	85.6862
CFAP298-TCI	19.395553	31.202971	14.401561	42.845	0	50.727635	20.494588	43.32254
CFAP299	0	0.994309	0	0.291689	0	0	0	0
CFAP300	106.547963	51.484358	146.022259	47.098551	0.827328	0.854592	0	1.36717
CFAP36	53.819821	94.307371	53.255582	76.560052	13.617904	54.284325	69.647975	70.913399
CFAP410	6.519443	4.270028	17.450049	4.846556	1.749222	10.491261	0.176491	5.755995
CFAP418	0	3.584353	2.685935	1.875295	3.954318	1.344857	0	1.966778
CFAP43	0	0.247782	0	0	0	0	0	0
CFAP44	6.5981	3.810532	1.384757	2.08285	0.408595	0.227498	0	0.569017
CFAP45	0	6.368084	3.386646	6.603321	0	4.312408	0	2.299707
CFAP46	0	1.560668	0	0.970031	0.515529	0.795846	0	0.59744
CFAP47	0	0.041029	0.290738	0.070232	0	0	0	0
CFAP52	0	0	0	0.177059	0	0	0	0
CFAP53	0	2.75812	3.427488	0.885647	0	0.2846	0	0.388004
CFAP53P1	0	0	0	0	0	0	0	0
CFAP54	3.534748	1.624391	1.577703	0.112631	0	1.194013	0	0.850932
CFAP57	26.918227	0	0	2.20651	0	0	0	0
CFAP58	0	1.146475	1.839294	0.825541	0	1.434885	0	0.041242
CFAP61	0	1.015682	0	0.058981	0	0	0	0
CFAP65	0	0	1.490025	0.34893	0	0	0	0
CFAP68	50.595997	37.83971	131.440313	39.028484	61.36811	12.948889	202.734457	19.54852
CFAP69	0	2.096169	0	1.336033	0	0.402453	0	3.577728
CFAP69P1	0	0	0	0	0	0	0	0
CFAP70	0.96947	0	0.630845	0	0	0	0	0
CFAP73	0	0	0	0	0	0.279195	0	0
CFAP74	0	3.482862	10.253065	7.491719	0	0.621571	0	0.083839
CFAP77	0	0	0	0	0	0.190415	0	0
CFAP90	3.768445	0.310742	0	0.041508	0	0.547039	0	0.308014
CFAP91	0	0	2.598587	0.4554	0	0	0	2.682493
CFAP92	11.201277	13.223949	0	4.180806	0	1.090002	0	0.864092

CFAP95	0	0	0	0	0	0	0	0
CFAP96	0	1.908933	4.2643	2.14014	0	0.929842	0	1.262432
CFAP97	27.22374	42.348966	19.410617	16.307782	63.22814	13.32696	9.195942	24.191189
CFAP97D1	0	0	0	0	0	0	0	0
CFAP97D2	0	0	0	0.197959	0	0	0	0
CFAP97P1	0	0	0	0	0	0	0	0
CFAP99	0	0	0	0.423396	0	0	0	0
CFB	1.814168	5.468905	16.133546	7.429919	25.204987	17.472069	0	8.327247
CFC1	0	0	0	0	0	0	0	0
CFC1B	0	0	0	0	0	0	0	0
CFD	0	3.735944	10.979593	5.076236	7.332254	3.472992	0	2.769447
CFDP1	125.925418	304.109927	86.105521	378.316934	145.888151	257.11122	149.715034	181.692381
CFH	8.240954	4.86188	7.617453	4.305734	0	1.231406	0	2.652642
CFHR1	0	0	0	0	0	0	0	1.030747
CFHR2	0	0	0	0	0	0	0	0
CFHR3	0	0	0	0	0	0	0	0
CFHR4	0	0	0	0	0	0	0	0
CFI	0	0	0	0	0	0	0	0
CFL1	1611.31497	2189.43219	1448.45644	2284.6751	1045.80399	1500.26048	945.311465	1248.81172
CFL1P1	0	0	0	0	0	0	0	0
CFL1P2	0	0	0	0	0	0	0	0
CFL1P3	0	0	0	0	0	0	0	0
CFL1P4	0	0	0	0	0	0	0	0
CFL1P5	0	1.891225	0	1.14633	0	0	0	0
CFL1P6	0	0	0	0	0	0	0	0
CFL1P7	0	0	0	0	0	0	0	0
CFL1P8	0	0	0	0	0	0	0	0
CFL2	103.63041	56.498229	55.804429	11.608714	46.503564	17.818682	14.183096	45.508682
CFLAR	15.489834	14.672425	15.455217	23.373086	27.871076	46.11427	4.46732	55.010753
CFP	0	0.906722	0	0.079819	0	0.081701	0	0.640218
CFTR	0	0	0	0	0	0	0	0
CFTRP1	0	0	0	0	0	0	0	0
CFTRP2	0	0	0	0	0	0	0	0

CFTRP3	0	0	0	0	0	0	0	0
CGA	0	0	0	0	0	0	0	0
CGAS	30.293032	15.256494	17.789898	12.149008	11.856894	5.328764	10.120677	6.307489
CGB1	0	0	0	0	0	0	0	0
CGB2	0	1.374627	0	0.149265	0	0	0	0
CGB3	0	0	0	0.531833	0	0	0	0
CGB5	0	3.715381	0	5.43709	0	1.412039	0	0.679274
CGB7	0	0.964836	0	1.138308	0	1.184098	0	1.007649
CGGBP1	32.957792	74.905624	26.914861	82.176751	20.533346	65.972024	23.317942	78.630848
CGN	22.408	4.856641	23.348795	8.249062	13.878328	9.438131	0	3.523214
CGNL1	0	1.058767	6.902107	0.618731	0	0.621921	0	0
CGREF1	0	11.03286	13.106855	15.668137	1.164093	9.599199	0	5.365111
CGRRF1	16.808155	39.08033	20.868975	18.406084	1.010928	16.857721	33.748591	19.000992
CHAC1	28.302298	24.775228	27.279179	59.275332	10.778396	7.618936	0	1.909372
CHAC2	8.402162	8.651166	12.428148	8.044588	13.264492	9.449682	16.177016	13.52172
CHAD	0	0	0	0	0	0	0	0
CHADL	0	0.285711	0	0.163483	0	0.454271	0	0.540903
CHAF1A	19.808559	69.035384	26.935262	94.22787	24.055368	116.571356	57.801994	102.078074
CHAF1B	25.405296	19.902246	8.59153	25.664508	15.552006	43.632506	26.342392	43.261855
CHAMP1	31.20457	50.818497	37.122145	33.727452	29.696669	42.088633	46.449409	74.725749
CHAT	0	0	0	0	0	0	0	0
CHCHD1	56.755149	116.333985	43.007391	87.479029	69.437128	75.97007	83.334409	94.232799
CHCHD10	168.180847	184.09759	187.34225	213.670881	207.833753	238.614555	263.432819	227.93711
CHCHD2	246.510116	235.835657	234.024339	206.429098	379.548797	341.006675	390.61763	348.566888
CHCHD2P1	0	0	0	0	0	0	0	0
CHCHD2P11	0	0	0	0	0	0	0	0
CHCHD2P2	0	0	0	0	0	0	0	0
CHCHD2P3	0	0	0	0	0	0	0	0
CHCHD2P4	0	0	0	0	0	0	0	0
CHCHD2P5	0	0	0	0	0	0	0	0
CHCHD2P6	0	0.904472	0	1.933968	0	0.536244	0	0
CHCHD2P7	0	1.315679	0	0	0	0	0	0
CHCHD2P8	0	0	0	0	0	0	0	0

CHCHD2P9	0	0.434424	0	0	0	0	0	0
CHCHD3	42.606285	137.40125	58.364737	106.93715	54.489128	153.322188	36.109875	165.239424
CHCHD3P1	0	0	0	0	0	0	0	0
CHCHD3P2	0	0	0	0	0	0	0	0
CHCHD3P3	0	0	0	0	0	0	0	0
CHCHD4	27.750322	55.65366	13.454648	42.553529	23.244195	23.398265	21.891171	27.711998
CHCHD4P2	0	0	0	0	0	0	0	0
CHCHD4P3	0	0	0	0	0	0	0	1.717213
CHCHD4P4	0	0	0	0	0	0	0	0
CHCHD4P5	0	0	0	0	0	0	0	0
CHCHD5	27.308648	5.428395	0	0.133355	0.275159	6.23904	0	2.724371
CHCHD6	0	0.857815	0	8.176555	46.702846	12.675304	0	4.278043
CHCHD7	48.869841	158.05112	44.518715	75.511526	35.73792	35.353485	126.61529	54.409402
CHCT1	0	0	0	0	0	0	0	0
CHD1	5.48688	7.985009	0	0	81.953403	0	0	0
CHD1L	43.052888	110.503707	57.782181	77.156484	29.444208	49.269339	34.951583	63.752148
CHD2	23.307318	42.278325	30.005341	124.832349	141.05513	128.218406	296.181749	71.143787
CHD3	40.248587	6.995539	21.553885	12.990806	17.637507	45.247819	477.401148	28.237788
CHD4	44.443877	73.377543	60.097805	103.188882	110.7419	218.034168	130.242859	80.811172
CHD5	0.978215	1.137851	2.641229	4.188943	0	0.562558	0	0.230153
CHD6	77.373723	27.035127	15.604027	32.070542	24.918153	62.606899	83.075656	24.162096
CHD7	15.405515	37.922019	17.889239	58.381513	54.384972	42.946554	134.955342	30.846386
CHD8	4.545816	39.00917	24.93631	51.427958	52.103606	90.510082	67.583543	63.651488
CHD9	23.742531	43.721721	27.616918	23.494911	20.412904	24.672772	105.814161	46.087905
CHD9NB	0	0.422437	0	0	0	0	0	0
CHDH	2.937737	11.758531	6.622658	10.714805	0.221822	3.033979	0.204724	2.909674
CHEK1	42.100414	38.771381	28.246388	32.615142	55.240603	76.245094	176.502007	106.093122
CHEK2	0	12.412306	24.357442	12.518472	74.378187	6.084397	0	11.336296
CHEK2P1	0	0	0	0	0	0	0	0
CHEK2P2	0	0	0	0	0	0	0	0
CHEK2P3	0	0	0	0	0	0	0	0
CHEK2P4	0	0	0	0	0	0	0	0
CHEK2P5	0	0	0	0	0	0	0	0

CHEK2P6	0	0	0	0	0	0	0	0
CHEK2P7	0	0	0	0	0	0	0	0
CHERP	54.15889	26.311227	23.990527	28.880137	30.374323	61.21632	77.736084	48.450988
CHFR	5.11595	9.531717	47.082183	3.870755	27.759123	15.221065	57.099427	6.597766
CHGA	0	0.444217	0	0.84978	0	0	0	0.181013
CHGB	3.333963	2.705571	1.237622	2.592181	3.284547	1.458289	0	1.281471
CHI3L1	0	0	0	0	0	0	0	0
CHI3L2	0	0.134634	0	0	0	0	0	0
CHIA	0	0	0	0	0	0	0	0
CHIAP1	0	0	0	0	0	0	0	0
CHIAP2	0	0	0	0	0	0	0	0
CHIAP3	0	0	0	0	0	0	0	0
CHIC1	19.230789	2.180062	1.714316	3.308705	0	2.553945	44.266863	2.849732
CHIC2	0	22.518436	24.100625	20.57924	40.333494	18.73956	17.156154	25.644823
CHID1	71.474146	116.860989	54.337281	145.883435	74.649233	139.036545	78.538073	147.552205
CHIT1	0	0	0	0	0	0	0	0
CHKA	29.895071	67.976056	34.572417	66.866931	54.878129	32.19072	66.228198	33.947073
CHKB	0	5.423714	0	2.750676	0.953545	11.155002	0	6.079793
CHKB-CPT1B	0	0	7.461455	0.457089	0	0	0	0.388893
CHL1	0	0	7.520553	0	0	0	0	0
CHM	14.463285	10.096291	14.070938	11.793616	4.451304	6.718815	18.5626	9.959526
CHML	0	25.700863	0	24.135063	51.529255	37.500746	0	43.862226
CHMP1A	55.491833	126.627891	60.069583	126.607656	42.939713	161.527551	44.57626	98.902138
CHMP1AP1	0	0	0	0	0	0	0	0
CHMP1B	0	0	0	0	0	0	0	0
CHMP1B2P	0	0	0	0	0	0	0	0.965505
CHMP2A	47.607257	48.186738	0	47.996219	47.856845	59.832621	0	78.217026
CHMP2B	41.044301	77.139169	41.919539	55.091511	14.6913	41.700027	39.779332	55.261538
CHMP3	101.375547	193.030615	113.020625	193.586946	40.134361	134.826774	164.822192	133.689271
CHMP4A	16.327144	95.033227	0	55.872708	41.365837	46.451875	16.966492	61.521628
CHMP4AP1	0	0	0	0	0	0	0	0
CHMP4B	182.218358	460.533155	189.946294	463.713177	132.866745	355.842791	121.793771	269.495258
CHMP4BP1	0	1.83371	0	2.193658	0	4.335904	0	2.917856

CHMP4C	34.005526	34.63024	28.649928	39.6968	18.422902	17.448567	19.650621	24.640291
CHMP5	36.513633	41.544783	47.493443	52.298671	61.92105	53.915906	103.744449	44.966777
CHMP5P1	0	0	0	0	0	1.259623	0	0
CHMP6	2.129109	50.280739	5.690213	47.694242	22.956496	69.838768	0	61.644587
CHMP7	14.12043	47.919254	14.749045	40.027237	43.231431	61.598169	26.795456	38.425896
CHN1	5.644057	7.134151	0	4.040446	0	6.436529	1.972951	4.156862
CHN2	0	3.862893	0	3.364784	23.236614	8.664256	0	1.390696
CHODL	0	0	0	0	0	0	0	0
CHORDC1	158.468802	134.555426	127.573969	89.9495	47.597774	38.2663	157.390117	68.265403
CHORDC1P1	0	0	0	0.094988	0	0	0	0
CHORDC1P3	0	0	0	0	0	0	0	0
CHORDC1P4	0	0	0	0	0	0	0	0
CHORDC1P5	0	0	0	0	0	0	0	0
CHORDC2P	0	0	0	0	0	0	0	0
CHP1	80.433223	73.764676	120.479563	78.548429	79.511765	96.192311	192.343469	50.24612
CHP1P1	0	0	0	0	0	0	0	0
CHP1P2	0	0	0	0	0	0	0	0.858958
CHP1P3	0	0	0	0	0	0	0	0
CHP2	0	0	0	0.12874	0	0.437042	0	0.142643
CHPF	11.081203	11.565394	4.916712	26.879537	1.1526	42.902942	0	23.825171
CHPF2	0	1.057301	0	0.27151	2.63686	1.047688	0	0.476359
CHPT1	20.055601	15.639003	8.042047	2.987587	20.624805	8.008951	14.453097	13.877598
CHRAC1	69.183037	61.293992	40.506384	45.46217	37.447885	28.407565	17.640405	40.79916
CHRD	0	0	0	0	0	0	0	0
CHRDL1	0	0	0	0	0	0	0	0
CHRDL2	0	0	0	0	0	0	0	0
CHRFAM7A	0	0	0	1.127289	0	0.192264	0	0
CHRM1	0	0	0	0.062335	0	0	0	0.271639
CHRM2	0	0	0	0	0	0	0	0
CHRM3	0	0.327136	0	1.207873	0	0.294291	0	1.573666
CHRM4	2.374516	1.295615	6.337307	1.723634	10.629529	0.583269	0	0.288372
CHRM5	0	0.107235	0	0	0	0	0	0
CHRNA1	0	0	0	0	0	0	0	0

CHRNA10	0	0.309427	2.172172	0	0	0	0	0.288883
CHRNA2	0	0	0	0	0	0	0	0
CHRNA3	0	0	0	0	0	0	0	0
CHRNA4	0	0.466943	0	0.699319	0	0	0	0
CHRNA5	58.151491	29.215483	30.231254	16.450852	19.222638	6.723834	65.185715	21.214206
CHRNA6	0	0	0	0.271813	0	0	0	0
CHRNA7	0	0.089132	0	0.522366	0	0	0	0.390016
CHRNA9	0	0	0	0	0	0	0	0.121649
CHRNB1	11.809083	16.300828	8.308774	18.286769	4.952023	17.107584	0	8.338746
CHRNB2	0	0	0	0	0	0.283665	0	0.235911
CHRNB3	0	0	0	0	0	0	0	0
CHRNB4	0	0.872349	0	1.447452	0	0.164592	0	0.402213
CHRND	0	0	0	0	0	0	0	0
CHRNE	0	0.134369	0	0.229878	0	0.544966	0	0.076517
CHRNG	0	0	1.478939	0	0.217589	0	0	0
CHST1	0	0.036255	0	0.062008	0	0.549996	0	0.340087
CHST10	16.54529	17.81204	4.228651	26.475513	5.093097	41.487799	0	47.625382
CHST11	35.940064	49.84795	27.105163	69.883131	12.538721	47.956041	18.907453	47.530418
CHST12	0	7.888682	0.89863	10.764335	5.584228	14.774658	2.485315	10.298291
CHST14	14.131069	19.077166	8.273219	16.983639	9.778725	20.839252	29.930431	14.122733
CHST15	13.468699	37.124887	6.732752	31.518418	17.28023	48.058487	14.936612	83.406988
CHST2	0	1.978742	0.714736	1.108243	0	0.875764	0	1.470748
CHST3	13.540866	17.942915	7.978179	13.698941	18.878271	45.780265	8.994617	31.631786
CHST4	0	0.311755	0	0	0	0	0	0.556108
CHST5	0	0	0	0	0	0	0	0
CHST6	3.47053	1.026003	0.346409	2.782637	1.221109	1.144436	0	1.364675
CHST8	0	3.659081	2.889168	1.437379	2.13179	0.913401	0	0.31118
CHST9	0	0	0	0	0	0	0	0
CHSY1	7.741901	28.36744	19.932522	27.535609	23.096439	48.55362	87.068476	62.312987
CHSY3	0	1.359129	2.309756	1.214748	0	0	0	0
CHTF18	37.236293	4.85705	5.034857	7.378049	32.567267	12.436527	40.981892	11.16437
CHTF8	57.626037	137.301428	39.786068	141.268996	105.720889	178.739954	13.258338	171.152041
CHTF8P1	0	0	0	0	0	0	0	0

CHTOP	51.56281	122.850346	42.103188	111.566029	72.441026	119.701511	52.457862	114.764686
CHUK	25.832534	29.010539	14.895583	16.5646	16.564324	8.631752	40.289816	25.663225
CHURC1	0	1.208475	0	0	61.518045	0	36.716881	1.265431
CHURC1-FN	0	0	0	0	0	2.604265	0	6.823039
CIAO1	9.351383	28.357634	28.170776	18.695867	12.982628	15.293431	14.195775	24.564446
CIAO2A	69.668335	34.202176	28.459109	27.792098	9.953188	13.227556	0	48.943536
CIAO2AP1	0	0	0	0	0	0	0	0
CIAO2AP2	0	1.624903	0	0	0	0	0	0
CIAO2B	58.53751	123.31476	101.791662	151.406939	83.795561	130.446146	113.517988	97.270923
CIAO3	25.350395	11.348672	11.352723	16.89553	14.285903	24.730966	0.650706	24.081763
CIAPIN1	47.657893	51.290559	32.167769	56.864861	51.669633	64.803886	105.25715	72.954815
CIAPIN1P	0	0	0	0	0	0	0	0
CIART	9.499486	4.134818	14.022436	5.838642	0.832596	3.19912	0	2.702951
CIB1	14.180721	58.676634	0	45.381828	61.24669	18.773966	58.294169	19.247415
CIB2	0	5.21317	7.39722	5.341139	10.889023	5.94934	0	2.86483
CIB3	0	0	0	0	0	0	0	0
CIB4	0	0	0	0	0	0	0	0
CIBAR1	43.254522	58.687563	3.758633	40.079516	62.979509	29.892733	53.760396	40.446974
CIBAR1P1	0	0	0	0	0	0.256723	0	0
CIBAR1P2	0	0	0	0	0	0	0	0
CIBAR2	0	0	0	0.404275	0	0	0	0
CIC	16.161762	21.973701	9.292568	19.75373	33.822679	104.153894	37.508395	57.855323
CICP1	0	0	0	0	0	0	0	0
CICP10	0	0	0	0	0	0	0	0
CICP11	0	0	0	0	0	0	0	0
CICP12	0	0	0	0	0	0	0	0
CICP13	0	0	0	0	0	0	0	0
CICP14	0	0	0	0.030123	0	0.218714	0	0
CICP15	0	0	0	0	0	0	0	0
CICP16	0	0.102177	0	0	0	0	0	0
CICP17	0	0	0	0	0	0	0	0
CICP19	0	0	0	0	0	0	0	0
CICP2	0	0	0	0	0	0	0	0

CICP20	0	0	0	0	0	0	0	0
CICP21	0	0	0	0	0	0	0	0
CICP22	0	0	0	0	0	0	0	0
CICP23	0	0	0	0	0	0	0	0
CICP24	0	0	0	0	0	0	0	0
CICP25	0	0	0	0	0	0	0	0
CICP26	0	0	0	0	0	0	0	0
CICP27	0	0	0	0.021202	0	0.11995	0	0
CICP28	0	0	0	0	0	0	0	0
CICP3	0	0	0	0.033768	0	0	0	0
CICP4	0	0	0	0	0	0	0	0
CICP5	0	0	0	0	0	0	0	0
CICP6	0	0	0	0	0	0.349965	0	0
CICP7	0	0	0	0	0	0	0	0
CICP8	0	0	0	0	0	0	0	0
CICP9	0	0	0	0	0	0	0	0
CIDEA	0	0	0	0.372893	0	0	0	0
CIDEB	0	0	0	0	0	0	0	0
CIDEC	0	0	0	0	0	0	0	0
CIDECP1	0	0	9.399879	0.580394	0	0	26.779359	5.965529
CIDECP2	0	0	0	0.915759	0	0	0	0
CIITA	0	1.050412	0	1.242372	12.266093	6.733847	0	3.363286
CILK1	0	0.982462	0	0	0	0.296124	0	4.391633
CILP	0	0	0	0	0	0	0	0
CILP2	0.784963	1.96518	1.409271	1.84014	0	0.470968	0	0.189103
CIMAP1A	0	0	0	0	0	0	0	0
CIMAP1B	0	0.345427	3.65211	2.586729	0	1.938266	0	1.220826
CIMAP1C	0	0	0	1.1197	0	0.161589	0	0.676004
CIMAP1D	0	1.394013	0	0.062976	0	0	0	0.422517
CIMAP2	0	0	0	0	0	0	0	0
CIMAP3	1.494233	1.078429	6.67969	3.091834	5.612118	0.22237	0	2.060935
CIMIP2A	0	0.25946	0	0.099462	0	0.101143	0	0
CIMIP2B	6.335252	0.505959	0	1.505817	0	1.205242	0	1.047083

CIMIP2C	22.111952	15.526417	5.143565	12.106217	0	3.616132	0	2.93724
CIMIP4	0	0	0	0	0	0	0	0
CINP	34.191367	76.717771	36.304767	70.63389	27.949249	59.826989	84.057019	77.417655
CIP2A	9.303457	27.009379	20.781805	10.502529	16.351529	15.33666	31.838092	18.045056
CIPC	15.982691	32.390664	12.299227	40.957537	66.526539	51.142358	9.263743	42.315395
CIR1	25.442278	18.2852	35.679988	19.169027	27.783885	22.351046	0.439778	22.292298
CIR1P1	0	0	0	0	0	0	0	0
CIR1P2	0	0	0	0	0	0	0	0
CIR1P3	0	0	0	0	0	0	0	0
CIRBP	115.933935	58.417791	53.506521	81.182509	122.176872	104.080691	120.610939	78.442732
CIROP	0	0	0	0	0	0	0	0
CISD1	59.834901	73.336088	74.342944	43.050054	24.982251	31.717407	16.393945	63.711003
CISD1P1	0	0	0	0	0	0	0	0
CISD2	20.381858	29.221277	39.895385	10.303549	23.035385	11.1611	44.971923	34.05259
CISD3	32.509643	42.440876	31.975261	32.316316	3.516989	58.927559	63.445866	45.086571
CISH	0	0	0	0.021192	0	0	0	0.605488
CIST1	0	0	0	0.266566	0	0	0	0
CIT	21.931132	13.790021	19.17831	13.347323	131.021318	61.849705	80.216418	45.87964
CITED1	0	0	0	0.186421	0	0	0	0.244444
CITED2	15.806128	25.914682	23.08128	23.233449	48.853845	32.594541	25.300827	32.474793
CIZ1	62.260743	94.257927	112.663091	79.497586	124.076388	140.031214	142.055909	66.574454
CKAP2	56.904837	52.801876	33.048969	26.919092	45.978045	31.960125	126.967533	41.509181
CKAP2L	40.653413	37.543426	16.214077	25.457744	41.450253	56.757574	40.765438	49.07097
CKAP2LP1	0	0	0	0	0	0	0	0
CKAP2P1	0	0	0	0	0	0	0	0
CKAP4	87.988999	106.026091	109.628722	138.275864	178.950632	265.360373	64.667862	137.590479
CKAP5	18.495007	65.399086	57.37144	85.776335	139.313665	161.856114	191.699555	149.614015
CKB	71.379887	89.69538	74.767466	104.862491	70.462329	105.920004	74.743354	61.927963
CKBP1	0	0	0	0	0	0	0	0
CKLF	144.669285	92.356715	67.994062	77.748522	27.960796	47.21345	91.755677	34.822021
CKLF-CMTM:	7.804328	15.067266	0	18.353561	0	6.12755	0	2.86009
CKM	0	0	0	0	0	0	0	0
CKMT1A	0	12.980549	0	20.477898	14.46766	40.47153	0	25.840239

CKMT1B	51.073076	53.330268	42.277018	76.895066	53.220307	71.913458	26.27185	46.951114
CKMT2	0	0	0	0	0	0	0	0
CKS1B	243.496934	206.706499	162.137641	132.548093	237.773645	137.343262	350.451324	163.411569
CKS1BP1	0	0	0	1.73258	0	1.556618	0	10.508802
CKS1BP2	0	0	0	0	0	0	0	0
CKS1BP3	0	0	0	50.570677	0	89.681089	0	0
CKS1BP4	0	0	0	0	0	0	0	0
CKS1BP5	0	0	0	0	0	0	0	0
CKS1BP6	0	0	0	0	0	0	0	0
CKS1BP7	0	0	0	0	0	0	0	0
CKS2	138.388528	109.864893	75.671274	75.406264	239.427881	157.969122	138.57933	235.049545
CLASP1	13.462497	28.929584	13.21317	29.972959	109.958776	49.978415	42.76455	98.855767
CLASP2	42.084214	31.123422	11.870485	18.468229	108.545221	26.530853	23.025124	24.531583
CLASRP	16.950862	22.915037	13.881383	36.43077	210.06283	66.898816	30.329242	28.987487
CLBA1	8.627161	35.319386	13.349019	33.802499	0.48069	25.905518	14.586323	25.722536
CLC	0	0	0	0	0	0	0	0
CLCA1	0	0	0	0	0	0	0	0
CLCA2	0.840203	1.906829	0	1.600113	2.662488	1.469419	0	0.777252
CLCA3P	2.426994	1.871102	3.260009	1.465943	0	0	0	0
CLCA4	0	0	0.933236	0.304632	0	0	0	0
CLCC1	5.372022	5.077541	0	4.025827	0	8.160818	15.279259	4.959362
CLCF1	2.071732	10.737379	9.231103	11.866389	2.729207	9.0822	0.500634	7.201251
CLCN1	0	0	0	0	0	0	0	0
CLCN2	6.969715	4.250953	13.523164	3.552039	3.537335	6.609447	0	3.436933
CLCN3	0	17.930172	9.92686	21.982547	20.431351	6.914688	0	17.186582
CLCN3P1	0	0	0	0	0	0	0	0
CLCN4	0	0	0	0	0	0	0	0
CLCN5	2.279437	3.726186	0.586153	6.874892	1.699381	8.329268	1.589328	8.60711
CLCN6	5.247985	4.529791	5.2404	4.724208	4.424849	5.078315	20.610735	3.571905
CLCN7	14.239257	17.201985	12.066822	30.56272	56.836635	27.022082	14.104686	32.892485
CLCNKA	0	0.233824	0	0.06649	0	1.411259	0	0
CLCNKB	0	0.343359	9.481331	0.275613	0	0	0	0
CLCP1	0	0	0	0	0	0	0	0

CLCP2	0	0	0	0	0	0	0	1.118962
CLDN1	118.83533	71.21651	152.483098	111.310089	180.869877	158.190418	174.077434	132.356061
CLDN10	0	0.587155	0	0.504092	0	0	0	0
CLDN11	0	0.958485	0	0.944648	5.740123	0.508761	0	0.683639
CLDN12	35.106274	27.040684	39.483887	43.373382	28.366162	32.240756	34.777046	40.825
CLDN14	0	0	0	0	0	0	0	0
CLDN15	0	2.372022	0	1.156863	0	2.364619	0	0.652652
CLDN16	7.981058	7.751971	6.885401	10.535686	0	2.426041	0	0.783958
CLDN18	0	0	0	0	0	0	0	0
CLDN19	0	0	0	0	0	0	0	0
CLDN2	0	0	0	0	0	0	0	0
CLDN20	0	0	0	0	0	0	0	0
CLDN24	0	0	0	0	0	0	0	0
CLDN3	2.890619	2.169378	0	3.207836	0	0.141209	0	0.11733
CLDN34	0	0	0	0.186368	0	0	0	0.475473
CLDN4	0	1.522328	0	2.88699	0	1.583445	0	0.908936
CLDN5	0	0	0	0	0	0	0	0
CLDN6	0	0.891722	0	0.128785	0	0.099935	0	0
CLDN7	68.755719	199.554154	94.313469	304.184026	83.103942	333.209308	43.050305	274.936039
CLDN7P1	0	0	0	0	0	0	0	0
CLDN9	11.26173	11.648275	0	12.979722	0	4.986013	0	4.283911
CLDND1	92.245335	88.52927	119.635455	107.069753	96.403136	51.669964	54.825271	84.017311
CLDND2	0	1.930648	0	0.49383	0	0.589865	0	2.276103
CLEC10A	0	0	0	0	0	0	0	0
CLEC11A	0	1.697484	0	0.703048	0	0.411552	0	0.750729
CLEC12A	0	0	0	0	0	0	0	0
CLEC12B	0	0	0	0.225695	0	0	0	0
CLEC16A	26.993291	16.349704	15.926507	17.202231	28.221575	35.827383	12.332844	22.4579
CLEC17A	2.964012	0.617324	0	0.071476	0	0	0	0
CLEC18A	0	0	0	0	0	0	0	0
CLEC18B	0	0	0	0	0	0	0	0
CLEC18C	0	0	0	0	0	0	0	0.031272
CLEC19A	0	0	0	0.069791	0	0	0	0

CLEC1A	0	0	0	0	0	0	0	0
CLEC1B	0	0	0	0	0	0	0	0
CLEC20A	0	0	0	0	0	0	0	0
CLEC2A	0	0	0	0	0	0	0	0
CLEC2B	0	0	0	1.198156	0	0	0	0.717046
CLEC2D	0.743075	1.937843	0	1.384526	0	3.378272	0	0.346048
CLEC2L	0	0	0	0	0	0	0	0
CLEC3A	0	0	0	0	0	0	0	0
CLEC3B	0	0	0	0	0	0	0	0
CLEC4A	0	0.195126	0	0	0	0	0	0.786112
CLEC4C	0	0	0	0	0	0	0	0
CLEC4D	0	0	0	0	0	0	0	0
CLEC4E	0	0	0	0	0	0	0	0
CLEC4F	0	0	0	0.03349	0	0	0	0
CLEC4G	0	0	0	0	0	0	0	0
CLEC4GP1	0	0	0	0	0	0	0	0
CLEC4M	0	0	0	0	0	0	0	0
CLEC4OP	0	0	0	0	0	0	0	0
CLEC5A	0	0	0	0.023172	0	0	0	0
CLEC7A	0	0	0	0.986687	1.102572	0.158055	0	0.691401
CLEC9A	0	0	0	0	0	0	0	0
CLGN	0	0.475461	0	0.665967	0	0.061697	0	0
CLHC1	1.978119	3.983745	0	6.228489	0	3.609979	47.903966	3.672434
CLIC1	259.28693	346.90858	227.17745	365.376329	322.997846	497.811455	369.847234	440.906796
CLIC1P1	0	0	0	0	0	0	0	0
CLIC2	0	0	0	0.251675	0	0	0	0
CLIC3	5.163473	0.46227	0	0	1.352468	0	0	0.473463
CLIC4	105.208696	52.723699	67.457403	64.148703	35.984759	31.475694	35.445259	34.160457
CLIC4P1	0	0	0	0.706165	0	0	0	0
CLIC4P2	0	0	0	0	0	0	0	0
CLIC4P3	0	0	0	0	0	0	0	0
CLIC5	0	0	0	0	0	0	0	0.830313
CLIC6	0	0	0	0	0	0	0	0

CLINT1	51.864938	101.455019	47.486367	104.658582	32.258116	89.461442	22.898812	77.262098
CLIP1	29.256329	36.598747	40.412998	33.809055	36.623596	36.643463	85.696921	22.179823
CLIP2	10.783235	12.341935	6.998337	14.946724	0	25.530773	0.145969	14.7098
CLIP3	1.662063	0	0	0	0	0.082296	0	0
CLIP4	66.916646	63.632539	33.805676	33.635611	58.708679	19.831201	13.050823	31.209897
CLK1	44.666127	13.181955	18.593316	14.698032	14.63981	12.763121	58.333405	13.376709
CLK2	26.752071	30.264276	19.835336	42.884165	17.639395	38.131121	41.659005	26.465471
CLK2P1	2.838554	5.87184	2.518671	5.099605	0	0	0	0
CLK3	33.481542	26.454763	101.702847	35.618907	45.746902	29.220033	58.449217	12.844196
CLK3P2	0	0	0	0	0	0	0	0
CLK4	5.33804	0.565447	0	3.949861	0	5.186826	6.890947	1.765817
CLLU1-AS1	0	0	0	0	0	0	0	0
CLMN	10.719326	51.298453	17.782154	57.425859	6.630898	24.235491	44.506264	18.371622
CLMP	6.500989	27.185534	8.17629	22.531192	0	1.820808	0	0.339093
CLN3	3.209118	16.062221	10.177646	21.281671	16.677242	14.150354	25.781726	10.474211
CLN5	1.945522	0.750695	5.205495	0.826414	17.194156	0.525034	0.237173	0.815267
CLN6	25.722065	11.928792	28.750732	10.55478	61.370489	24.676038	28.288263	17.923422
CLN8	1.819984	16.047095	2.864422	18.152357	1.923799	16.282405	0	12.192146
CLNK	0	0	0	0	0	0	0	0
CLNS1A	321.299479	400.82335	367.904344	337.058469	483.981336	211.292134	251.068836	255.485418
CLNS1AP1	0	0	0	0	0	0	0	0
CLOCK	27.502241	26.531599	6.727878	8.96929	28.689754	15.778341	60.469745	35.891235
CLP1	17.385181	9.136504	15.48391	10.209698	24.088415	14.953262	16.794973	20.828249
CLPB	24.488991	29.193471	11.872565	34.41818	24.2825	24.416156	68.243589	20.381067
CLPP	20.039798	13.720971	0	23.002557	31.082185	24.621569	0	14.925787
CLPS	0	0	0	0	0	0	0	0
CLPSL1	0	0	0	0	0	0	0	0
CLPSL2	0	2.867512	0	1.474855	0	0	0	0
CLPTM1	23.435497	29.104173	41.933065	27.972592	78.554009	46.243616	197.851963	31.858775
CLPTM1L	134.093413	187.078116	93.663681	124.522084	57.835008	107.670248	96.566885	177.681803
CLPTM1LP1	0	0	0	0	0	0	0	0
CLPX	48.053696	129.046084	64.047323	96.361625	129.749689	79.525375	31.388336	136.950125
CLRN1	0	0	0	0	0	0	0	0

CLRN2	0	0	0	0	0	0	0	0
CLRN3	0	0	0	0	0	0	0	0
CLSPN	24.862128	23.134589	15.625638	15.248669	49.077751	30.175297	47.470078	33.4727
CLSTN1	69.002674	74.6507	92.805562	109.18358	98.216075	148.818363	77.250558	84.183541
CLSTN2	0	0	0	0.011017	0	0.022625	0	0.072014
CLSTN3	0	5.364582	0	11.25406	100.987908	7.696096	46.851945	9.485297
CLTA	130.390585	132.895323	79.384821	152.503888	117.602724	219.581845	213.66819	228.287022
CLTB	0	3.763816	0	2.654943	0	0	0	0.328608
CLTC	125.552523	117.091419	95.267268	126.890089	137.731497	106.327979	108.857979	140.034691
CLTCL1	0.674154	0.633385	2.422191	0.967948	11.771068	2.760993	3.612274	3.827885
CLTRN	2.697647	3.972475	7.186622	2.376164	16.657561	1.056483	0	3.565416
CLU	41.466979	74.06276	48.602558	87.799886	67.447562	45.199489	94.189885	35.026435
CLUAP1	67.186361	35.490024	80.332621	35.056032	11.890091	26.210164	45.673216	26.007082
CLUH	0	8.140255	13.611921	20.966312	31.285268	19.792324	80.894303	10.960434
CLUHP1	0	0	0	0	0	0	0	0
CLUHP10	0	0	0	0	0	0	0	0
CLUHP11	0	0	0	0	0	0	0	0
CLUHP2	0	0	0	0	0	0	0	0
CLUHP3	2.815732	0	0	0	0	0	0	0.298695
CLUHP4	0	0	0	0	0	0	0	0
CLUHP5	0	0	0	0	0	0	0	0
CLUHP6	0	0	0	0	0	0	0	0
CLUHP8	0	0	0	0	0	0	0	0
CLUL1	0	0	0	0	0	0	0	0
CLVS1	0	0	0	0.14217	0	0	0	0.047027
CLVS2	0	0.135525	0	0.027979	0	0	0	0
CLXN	4.639708	24.726634	11.776764	12.725046	0	0.40643	0	4.449207
CLYBL	0	0.702014	2.956175	3.621348	0	0	0	0
CMA1	0	0	0	0	0	0	0	0
CMAHP	0	1.577077	0	0.542351	0	0.71456	0	3.517547
CMAS	62.105539	56.58281	30.53288	35.017696	79.62858	23.650834	121.691653	45.742694
CMBL	34.717345	31.965763	41.057079	20.463295	20.655771	22.035192	32.234049	20.122803
CMC1	40.197682	47.743899	35.355952	21.653015	2.707313	11.946618	0	41.040869

CMC2	87.962018	171.245152	77.058494	115.358594	173.4917	123.127105	73.580752	212.055542
CMC4	49.869884	60.49566	19.8623	33.974103	3.266188	35.474038	0	55.957144
CMIP	1.959643	17.898641	0	25.756502	3.006463	56.364175	3.090925	24.135263
CMKLR1	0	0	0	0	0	0.029773	0	0.074571
CMKLR2	0	1.009305	0	0	0	0	0	0
CMPK1	0	56.011006	64.013073	37.604124	12.780896	39.19668	95.257086	50.251633
CMPK1P1	0	0	0	0	0	0	0	0
CMPK1P2	0	0	0	0	0	0	0	0
CMPK2	0	2.896419	0	0.459449	5.584255	14.804068	0	8.124087
CMSS1	54.400725	200.937738	89.611345	134.395186	94.848344	121.404967	183.696756	169.302183
CMTM1	0	8.095141	3.485484	18.396048	0	10.074755	0	5.899458
CMTM2	0	0	0	0.209313	0	0	0	0
CMTM3	60.461296	36.506788	56.75499	57.30754	22.048209	51.440506	51.321221	39.90678
CMTM4	33.627327	25.925756	25.27066	20.880035	37.979696	26.502549	86.735649	37.024925
CMTM5	0	0	0	0	0	0	0	0
CMTM6	48.438566	29.074878	45.235831	20.41519	39.96019	13.444745	58.586628	25.719148
CMTM7	9.49185	20.872838	14.166822	17.224809	48.779027	20.64134	13.518914	15.807228
CMTM8	6.397259	5.180686	3.422524	5.777282	0	2.80319	0.928149	3.641117
CMTR1	10.641804	18.185474	11.020361	15.540922	26.575066	24.087806	15.99128	18.780062
CMTR2	1.316879	19.810795	0	9.626815	23.938556	15.575638	27.962378	21.802219
CMYA5	0.248098	0.928292	0.446784	0.774208	0	0.274217	0	0.735679
CNBD1	0	0	0	0	0	0	0	0
CNBD2	13.171285	3.143635	5.723209	2.96445	17.203999	5.935759	0	5.471575
CNBP	417.685541	343.44553	209.366669	260.703301	257.516425	288.478669	117.917272	323.889362
CNDP1	0	0	0	0	0	0	0	0
CNDP2	61.610524	58.048878	61.788951	56.183591	107.316814	87.64857	204.072692	84.898272
CNEP1R1	10.457648	23.722717	15.866907	12.127549	7.506144	2.638175	9.999687	9.102115
CNEP1R1P1	0	0	0	0	0	0	0	0
CNFN	17.998936	3.484299	0	5.671386	0	7.43204	35.627089	6.41083
CNGA1	0	1.35112	0	0.551082	1.5925	0	0	1.005383
CNGA3	0	0	0	0	0	0	0	0
CNGA4	0	0	0	0	0	0	0	0
CNGB1	0	2.269803	0	9.466505	0	0	0	0.18161

CNGB3	0	0	0	0	0	0	0	0
CNIH1	291.05877	143.702796	270.103448	139.125933	212.344128	56.940294	94.129441	141.001858
CNIH2	9.99795	2.079481	7.055783	3.211139	7.448847	2.874576	0.789344	0.475901
CNIH3	0	10.321828	0	5.328118	0	1.953634	0	1.123196
CNIH4	58.749683	57.02671	67.398772	30.175628	21.069487	17.492153	14.077672	33.712546
CNKSR1	9.496236	4.030814	8.496851	7.061467	15.747556	13.906453	9.874249	8.222182
CNKSR2	0	0	0	0.00942	0	0	0	0
CNKSR3	1.713567	7.564885	1.302867	5.454152	5.889375	12.777077	12.447466	11.807567
CNMD	0	0	0	0	0	0	0	0
CNN1	0	0	0	0.289857	0	1.279946	0	0.097012
CNN2	271.112281	220.389801	289.984363	232.89622	270.654266	275.940282	390.067896	279.682641
CNN2P1	0	0	0	0	0	0	0	0
CNN2P10	0	0	0	0	0	0	0	0
CNN2P11	0	0	0	0	0	0	0	0
CNN2P12	0	0	0	0	0	0	0	0
CNN2P2	0	0	0	0	0	0	0	0
CNN2P3	0	0	0	0	0	0	0	0
CNN2P4	0	0	0	0	0	0	0	0
CNN2P6	0	0	0	0	0	0	0	0
CNN2P7	0	0	0	0	0	0	0	0
CNN2P8	0	0	0	0	0	0	0	0.1767
CNN2P9	0	1.067044	0	0.62372	0	0.20894	0	0.177697
CNN3	324.21485	560.949921	169.869694	295.155618	167.296798	119.475361	101.637292	174.194931
CNN3P1	0	0	0	0	0	0	0	0
CNNM1	5.140522	6.430725	5.646786	5.921061	9.507005	7.846293	12.388141	6.577836
CNNM2	2.574132	3.231161	3.85351	5.40531	0.85069	4.487001	0	3.652254
CNNM3	27.35075	3.671247	9.004557	3.347691	12.710424	5.044809	11.596083	4.424745
CNNM4	4.797055	5.8825	11.694782	13.352791	5.430559	14.807299	10.180711	9.543645
CNOT1	314.509245	446.287048	142.818298	759.939574	474.927219	943.625388	436.923493	686.931922
CNOT10	10.969638	16.486358	0	7.473412	5.757327	2.001298	41.564935	8.615637
CNOT11	59.907168	88.674386	32.66197	78.593803	22.827134	100.305845	129.009186	84.893392
CNOT2	79.65114	105.730748	38.066925	132.458441	226.55887	107.497124	104.486962	112.353737
CNOT3	21.536163	92.143936	38.27356	96.783395	79.487199	202.518189	50.491521	127.516332

CNOT4	14.942313	9.097852	18.640947	9.960664	7.831825	8.380943	8.691466	16.242093
CNOT4P1	0	0	0	0	0	0	0	0
CNOT6	13.545785	80.976038	21.20405	48.206347	42.064866	35.273769	41.341003	50.405278
CNOT6L	17.271764	25.321854	16.622801	21.093637	14.85298	13.449596	62.167199	23.077732
CNOT6LP1	0	0	0	0.063675	0	0	0	0
CNOT7	81.09661	167.679503	149.125592	83.839662	36.664779	46.51854	165.875259	118.240423
CNOT7P1	0	0	0	0	0	0	0	0
CNOT7P2	0	0	0	0	0	0	0	0
CNOT8	25.679462	116.942412	21.316451	141.219626	2.634413	123.120692	0	205.56338
CNOT9	10.311361	12.371787	0	15.911374	4.386182	31.032955	37.100371	37.052031
CNP	33.164355	61.763665	33.165649	57.088673	91.564628	148.028615	112.838296	140.968058
CNPPD1	20.607618	13.957513	16.867816	16.974482	5.885823	20.473481	22.828215	16.954205
CNPY1	0	0	0	0	0	0	0	0
CNPY2	170.468917	262.426974	115.645548	263.680456	49.127581	113.531081	91.364463	121.382296
CNPY4	3.846628	9.434555	11.83549	9.612366	1.010163	8.667255	0	10.965204
CNR2	0	0	0	0.030323	0	0	0	0
CNRIP1	0	0.84959	0	0.47061	0	0	0	1.747083
CNST	19.929998	42.111925	8.59257	50.157885	5.69956	52.048405	67.589364	52.906958
CNTD1	0	0	0	0.135321	0	0	0	0.11373
CNTF	1.834226	0.69505	0	0.447751	0.483478	0.09082	0	1.705954
CNTFR	0	0.071967	0	0.354523	0	0	0	0
CNTLN	9.194613	6.695456	9.873087	5.108569	14.238336	7.04029	36.331888	6.916827
CNTN1	10.657646	30.621399	33.251432	22.958577	0	1.271815	0	2.220656
CNTN2	0	0	0	0	0	0	0	0.186972
CNTN3	0	0	0	0	0	0	0	0
CNTN4	0	0	0	0.17925	0	0	0	0
CNTN5	0	0	1.95412	1.403445	0	0	0	0.253593
CNTN6	0	0	0	0	0	0	0	0
CNTNAP1	7.737893	2.352469	5.291408	3.812347	2.022162	7.309289	36.191113	4.392594
CNTNAP2	9.093574	9.980005	5.289468	6.908916	0	0.635379	0	0.810954
CNTNAP3	5.420836	0	0	0.648901	0	1.227899	0	1.512851
CNTNAP3B	0	3.565055	1.169245	1.622765	8.912556	1.472197	0	13.811335
CNTNAP3C	0	2.858325	0	10.304513	3.20686	5.234261	0	4.311617

CNTNAP3P1	0	0	0	0	0	0	0	0
CNTNAP3P2	0	0.550548	0.770128	0.30709	0	0.397439	0	0.466037
CNTNAP3P5	0	0	0	0.641839	0	0	0	0
CNTNAP3P7	0	0	0	0	0	0	0	0
CNTNAP4	0	0	0	0	0	0	0	0
CNTNAP5	0	0	0	0	0	0	0	0
CNTRL	0	3.743656	7.085611	2.167144	0	2.377066	0	1.612374
CNTROB	3.073854	4.686428	0	16.765931	78.508592	28.198585	30.305957	13.056802
COA1	11.02168	35.773287	29.648931	33.162426	12.722616	65.433532	3.351912	74.721303
COA1P1	0	0	0	0	0	0	0	0
COA3	68.698996	43.821913	46.249692	59.916264	56.739709	50.316299	75.266929	47.994574
COA4	176.853592	274.713837	126.314727	244.960153	158.338114	173.164433	174.384667	170.193897
COA5	7.947388	47.477297	8.857119	18.645519	46.639368	14.283569	45.262115	34.595123
COA6	42.967545	156.564526	30.462719	69.090909	4.81428	77.009287	0	112.305006
COA7	85.335315	139.740582	64.687305	127.79953	21.003627	40.998423	21.746463	47.766997
COA8	13.744265	41.258717	57.937873	29.972954	11.644581	17.303275	49.733005	47.98044
COASY	38.448297	67.409996	36.855821	79.198665	135.695748	94.178343	75.37739	90.256421
COBL	1.900588	2.756032	1.106858	1.763458	6.814931	3.803831	4.458079	2.526962
COBLL1	9.065349	6.905444	0	11.657896	35.227277	15.558084	7.812609	8.05548
COBLP1	0	0	0	0	0	0	0	0
COCH	0	0	0	0.514293	0.427232	1.304934	0	0.474952
COG1	18.931224	6.969823	14.966699	11.287359	22.905428	15.275459	86.558965	11.235755
COG2	19.609875	65.650955	34.823756	52.030415	25.594362	46.653478	65.497993	35.195476
COG3	24.002866	12.889656	10.359787	12.134381	12.379085	3.893867	34.037093	12.655056
COG4	32.279739	22.793657	31.050408	35.475162	34.910362	25.588418	11.305056	18.712398
COG5	0	28.22141	0	41.981956	54.105625	17.921684	0	34.359029
COG6	14.478799	22.304608	14.175364	33.39481	4.332232	14.306929	3.520615	13.976979
COG7	13.74691	10.35812	9.236183	13.611673	19.954661	9.013582	16.974563	7.741678
COG8	9.7222	11.882819	12.15999	16.773591	19.905493	30.039714	15.509913	23.455633
COIL	16.721832	12.555089	21.876291	14.188821	15.272725	6.203687	27.786355	10.770113
COILP1	0	0	0	0	0	0	0	0
COL10A1	0	0	0	0	0	0	0	0
COL11A1	0	0	0	0	0	0.092778	0	0

COL11A2	0	0.259418	0	0	0	0.539736	0	0
COL11A2P1	0	0	0	0	0	0	0	0
COL12A1	33.585722	32.353324	55.610263	33.014651	51.114899	17.171746	44.271091	22.370802
COL13A1	0	0.821756	0	1.156309	4.029053	2.117342	0	0
COL14A1	0	0	0	0.145377	0	0	0	0
COL15A1	0	0.026858	0	0.16816	0	0	0	0
COL16A1	4.50384	5.703936	16.028401	6.544215	5.090665	23.424585	9.313559	12.190088
COL17A1	105.505605	156.646961	188.632662	388.546983	332.516804	634.638038	650.852715	375.021947
COL18A1	1.206623	1.399409	2.168532	1.686398	30.359241	7.125406	6.908948	2.937185
COL19A1	0	0	0	0.009017	0	0	0	0
COL1A1	0	0	9.209325	1.444874	0	10.812254	32.034216	5.117483
COL1A2	0	0	0	0	0	0.712933	0	0
COL20A1	0	0	1.616284	0	0	0	0	0
COL21A1	1.495652	0	0	0	0	0	0	0
COL22A1	0	0	0	0	0	0	0	0
COL23A1	0	0	0	0	0	0	0	0
COL24A1	0	0.322166	0.968274	0.546654	0	0	0	0
COL25A1	0	0	0	0	0	0	0	0
COL26A1	0	0.144759	0	0.110269	0	0.05634	0	0
COL27A1	6.746003	4.68831	11.828197	8.452183	7.752212	8.265138	2.116148	3.852412
COL28A1	0	0.253771	0	0	0	0	0	0
COL2A1	0	1.032487	0	1.426046	0	0.074052	0	0
COL3A1	0	0	0	0	0	0	0	0
COL4A1	36.415637	23.685051	20.391103	30.922944	37.480902	40.105158	1.039825	23.131846
COL4A2	20.742291	0.41618	36.027042	2.187493	0.168809	0.758762	25.39168	11.94782
COL4A3	0	0	0	0	0	0	0	0
COL4A4	0	0	0	0	0	3.367456	0	1.237227
COL4A5	8.077496	6.075373	15.886666	18.551891	20.14069	24.442798	39.07971	11.418241
COL4A6	18.727563	10.583702	0	11.698751	6.390264	7.750046	0	7.174685
COL5A1	0	0	1.707566	0.306298	0	0.114126	0	0
COL5A2	0.507118	1.244884	0	1.562777	0.626676	1.732489	0	3.430461
COL5A3	0	0.067504	0.940868	0.10238	0.829592	0.209922	0	0.146875
COL6A1	0	0	0	0	1.05487	0	0	0

COL6A2	0	0	0	0	0	0	0	0
COL6A3	0	0.243995	0	0	0	0.048303	0	0.077227
COL6A4P1	0	0	0	0	0	0	0	0
COL6A4P2	0	0	0	0	0	0	0	0
COL6A5	0	0	0	0	0	0	0	0
COL6A6	0	0	0	0	0	0	0	0
COL7A1	5.546506	5.539784	15.917648	8.11658	11.89375	6.020375	2.114667	2.427844
COL8A1	10.05045	15.538086	5.113829	10.513273	1.561921	4.763151	0	5.4924
COL8A2	0.739302	0.542611	0.66382	1.46269	0.195265	1.886141	0	0.979334
COL9A1	0	0	0	0	0	0	0	0
COL9A2	8.632846	0.604243	9.800873	2.718442	1.246908	0.587992	0	0
COL9A3	4.81632	3.67509	2.438085	2.561606	0	0.270743	0	0.635563
COLEC10	0	0	0	0.07816	0	0.213055	0	1.07048
COLEC11	0	0	0	0.172443	0	0	0	0
COLEC12	0	0	0	0.013909	0	0	0	0.022817
COLGALT1	45.503526	80.498305	65.013852	138.013519	128.050546	180.083586	89.79657	148.949921
COLGALT2	0	0	0	0	0	0	0	0.13933
COLQ	0	0	2.158972	0.177152	0	0	0	0
COMMD1	0	13.833082	0	0.129981	29.532568	11.057214	41.57516	0.223366
COMMD10	5.045513	26.412591	20.181218	9.718241	14.360855	4.585688	4.864705	21.883055
COMMD2	17.536721	35.460106	22.462258	31.984348	98.051424	45.03296	103.123587	56.962127
COMMD3	38.857252	16.185348	5.774211	9.55772	5.845644	3.82351	0	11.823365
COMMD3-BI	20.311515	8.325045	0	2.835295	0	2.571673	0	10.225208
COMMD4	110.21959	51.646115	18.65752	50.590399	43.319415	53.176576	25.620117	28.376245
COMMD4P1	0	0	0	0	0	0	0	0
COMMD4P2	0	0	0	0	0	0	0	0
COMMD5	16.864264	17.910145	12.343492	15.833963	6.175775	19.579509	2.258883	18.455476
COMMD5P1	0	0	0	0	0	0	0	0
COMMD6	166.357553	84.052475	143.243198	36.473594	47.625548	16.038202	175.455861	44.436955
COMMD7	30.536349	50.718398	21.56908	48.122985	26.964221	13.761535	14.400663	29.568775
COMMD8	28.01517	8.275161	6.581914	3.281932	16.240726	8.241528	14.27863	8.694291
COMMD9	24.66691	23.623442	14.103292	24.417578	37.298196	51.051752	55.057083	46.159211
COMP	0	0.126933	0	0.574802	0	0.345039	0	0

COMT	60.242439	91.71053	51.211974	104.736019	36.005057	145.12402	45.432008	129.518526
COMTD1	0	14.381631	0	4.449884	14.202605	4.520065	0	6.555504
COP1	41.0027	60.794976	39.342924	40.516954	127.807221	26.50795	91.663451	51.896733
COP1P1	0	0	0	0	0	0	0	0
COPA	130.918349	109.42863	238.002458	159.808005	107.4859	64.665157	102.704592	70.626134
COPB1	10.061996	35.155879	22.630047	33.437577	19.948048	35.600266	18.758569	53.53229
COPB2	0	90.119883	84.16827	106.608967	100.987653	88.813295	47.074927	101.692258
COPE	59.884652	79.762429	52.990675	95.343252	91.608953	147.926527	115.916184	104.277949
COPG1	153.255957	170.663798	288.780121	202.207885	205.768567	109.166746	165.11387	89.861148
COPG2	21.366172	21.663977	22.978654	17.150536	14.663537	8.679086	15.987192	12.207627
COPRS	64.472958	187.322295	56.643676	126.938974	42.564332	156.091961	76.80983	167.999384
COPRSP1	0	0	0	0	0	0	0	0
COPS2	146.023558	84.24758	142.020238	38.593992	111.294369	20.26719	80.200723	63.02317
COPS3	103.500816	62.511969	55.936218	43.642164	97.011226	70.317777	216.692428	126.155606
COPS3P1	0	0	0	0	0	0	0	0
COPS4	15.035801	24.993182	24.873389	10.669333	48.246855	8.084122	43.042007	13.438543
COPS5	68.751977	78.928789	62.244145	73.285594	27.496789	67.493733	29.99898	80.689519
COPS5P1	0	0	0	0	0	0	0	0
COPS5P2	0	0	0	0	0	0	0	0
COPS6	85.935528	107.314009	68.848591	113.114671	73.074071	106.100987	36.841302	102.360158
COPS7A	52.036464	72.586502	59.351581	65.234084	15.8087	46.260414	23.085569	51.37458
COPS7B	13.71298	29.360472	0	37.361813	28.481368	62.375377	29.021475	42.286375
COPS8	36.740258	65.364875	44.134049	48.795584	44.276178	60.589674	85.765993	66.925272
COPS8P1	0	0	0	0	0	0	0	0
COPS8P2	0	0	0	0	0	0	0	0
COPS8P3	0	0	0	0	0	0	0	0
COPS9	159.115052	206.400697	100.388368	135.661696	139.469693	223.1779	308.708499	299.597391
COPZ1	120.174544	150.491341	134.709506	137.419677	46.739137	53.941035	122.63013	48.41713
COPZ2	12.946015	9.211736	25.384642	12.183937	3.75771	8.308483	0	12.777357
COQ10A	2.65896	7.411107	0	5.41277	20.097028	4.194207	0	0.215602
COQ10B	0	0	15.845597	0.091791	0	0.177637	0	0
COQ10BP2	0	0	0	0	0	0	0	0
COQ2	0	5.245877	7.50943	1.745024	16.544366	0.198372	25.10696	2.59361

COQ3	31.753292	25.426403	14.707965	13.992901	19.569298	12.28803	0	25.489896
COQ4	21.150865	58.167621	33.369266	57.340385	58.536254	47.846516	14.643039	49.027609
COQ5	21.451446	35.428118	31.80473	28.900163	48.961159	41.937633	187.187876	44.289719
COQ6	9.680382	26.71862	17.084437	22.33006	17.948046	29.691612	10.108073	40.184111
COQ7	2.564218	24.08365	54.551843	39.222947	62.010574	33.758794	205.175883	31.517059
COQ8A	4.926056	11.022579	7.914683	15.96642	20.676774	26.096378	7.923557	16.332673
COQ8B	32.298662	5.079119	1.548017	12.328877	6.00247	19.196849	6.481999	10.545081
COQ9	89.301337	101.597032	67.681944	111.348849	49.366658	110.626275	75.079204	86.966414
CORIN	0	0	0	0	0	0	0	0
CORO1A	0	0.832192	27.057299	0.656756	23.529952	9.245202	29.603724	3.34676
CORO1B	58.716177	68.790645	50.166298	78.411176	44.292691	92.007605	30.144999	49.027838
CORO1C	123.290928	203.648899	133.489962	231.54129	69.830875	472.611095	298.450219	574.275435
CORO1CP1	0	0	0	0	0	0	0	0
CORO2A	13.152012	15.562633	16.653644	20.433361	22.901006	27.46679	10.342224	24.789646
CORO6	6.766709	4.761487	0	4.590038	2.970377	1.257925	0	2.384655
CORO7	2.947205	11.809356	4.297842	12.807072	37.641647	18.316409	15.203584	1.318192
CORO7-PAM	0	0	0	0.219599	0	0	0	2.29843
COTL1	566.747157	1067.8672	431.894432	1004.14899	626.486012	1347.83904	702.460445	1484.02683
COTL1P1	0	0	0	0	0	0	0	0
COTL1P2	0	0	0	0	0	0	0	0
COX10	11.611912	16.043353	23.931228	15.097613	39.103297	20.375793	9.550998	18.776303
COX11	0	1.025193	0	1.363525	0	0.308124	0	0.165616
COX11P1	0	0	0	0	0	0	0	0
COX14	23.823632	80.243371	41.691812	88.514749	67.379597	79.402531	0	87.896966
COX15	11.077262	8.481791	10.981719	6.340154	38.324667	4.559259	0	6.127172
COX16	131.68104	163.489634	65.437474	62.309678	144.575254	70.145899	423.70741	127.925236
COX16P1	0	0	0	0	0	0	0	0
COX17	0	11.992896	19.49462	8.88158	29.029881	14.572542	0	28.024919
COX17P1	0	0	0	0	0	0	0	0
COX18	1.419281	9.312231	9.105718	3.274776	10.002293	1.61798	12.601749	3.094859
COX19	38.305739	40.226673	27.49387	39.199869	10.194102	9.69329	37.95647	14.138544
COX20	64.716106	62.250869	20.547057	26.501576	19.293034	20.153522	72.110264	62.629193
COX20P1	0	0	0	0	0	0	0	0

COX20P2	0	0	0	0	0	0	0	0
COX4I1	41.99593	31.439322	41.416165	65.738355	44.900668	33.717258	32.21239	62.279735
COX4I1P1	0	0	0	0	0	0	0	0
COX4I1P2	0	0	0	0	0	0	0	0
COX4I2	0	0	0	0	0	0	0	0
COX5A	459.327865	340.85236	282.79611	325.773812	392.461402	300.989742	303.037666	384.463195
COX5AP1	0	0	0	0	0	0	0	0
COX5AP2	0	0	0	0	0	0	0	0
COX5B	83.855707	0	0	0.496441	76.9556	0.943428	72.336601	5.46674
COX5BP1	0	0	0	0	0	0	0	0
COX5BP2	0	0	0	0	0	0	0	0
COX5BP3	0	0	0	0	0	0	0	0
COX5BP4	0	3.939657	0	0	0	0	0	0
COX5BP6	0	0	0	0	0	0	0	0
COX5BP7	0	0	0	0	0	0	0	0
COX5BP8	0	0	0	0	0	0	0	0
COX6A1	533.927286	172.772366	412.638424	214.851742	629.478709	257.959708	677.805689	228.377627
COX6A1P1	0	0	0	0	0	0	0	0
COX6A1P2	0	0	0	0	0	0	0	0
COX6A1P3	0	0	0	0	0	0	0	0
COX6A1P4	0	0	0	0	0	0	0	0
COX6A1P5	0	0	0	0	0	0	0	0
COX6A1P6	0	0	0	0	0	0	0	0
COX6A1P7	0	0	0	0	0	0	0	0
COX6A2	0	0	0	0	0	0	0	0
COX6B1	303.870778	295.077319	298.790628	345.763805	499.500115	527.136283	708.29097	444.632129
COX6B1P1	0	0	0	0	0	0	0	0
COX6B1P2	0	0	0	0	0	0	0	0
COX6B1P3	0	0	0	0	0	0	0	0
COX6B1P4	0	0	0	0	0	0	0	0
COX6B1P5	0	0	0	0	0	0	0	0
COX6B1P6	0	0	0	0	0	0	0	0
COX6B1P7	0	0	0	0	0	0	0	0

COX6B2	2.599746	5.112138	2.029471	9.270881	0	8.903434	0	2.880713
COX6C	266.116964	222.513519	243.523395	182.392983	295.554041	89.098385	283.707108	153.59307
COX6CP1	0	0	0	0	0	0	0	0
COX6CP10	0	0	0	0	0	0	0	0
COX6CP11	0	0	0	0	0	0	0	0
COX6CP12	0	0	0	0	0	0	0	0
COX6CP13	0	0	0	0	0	0	0	0
COX6CP14	0	0	0	0	0	0	0	0
COX6CP15	0	0	0	0	0	0	0	0
COX6CP16	0	0	0	0	0	0	0	0
COX6CP17	0	0	0	0	0	0	0	0
COX6CP18	0	0	0	0	0	0	0	0
COX6CP2	0	0	0	0	0	0	0	0
COX6CP3	0	0	0	0	0	0	0	0
COX6CP4	0	0	0	0	0	0	0	0
COX6CP5	0	0	0	0	0	0	0	0
COX6CP6	0	0	0	0	0	0	0	0
COX6CP7	0	0	0	0	0	0	0	0
COX6CP8	0	0	0	0	0	0	0	0
COX7A1	0	0	0	0	0	0	0	0
COX7A2	105.664073	120.900731	152.316244	117.506291	134.002717	80.823018	0	113.548034
COX7A2L	110.01278	79.557317	159.45031	69.47739	103.842335	40.95481	130.60326	67.048869
COX7A2P1	0	0	0	0	0	0	0	0
COX7A2P2	0	0	0	0	0	0	0	0
COX7B	341.745093	398.010106	302.606044	331.569217	382.610314	274.009842	490.74528	303.369647
COX7B2	0	0	0	0	0	0	0	0
COX7BP2	0	0	0	0	0	0	0	0
COX7BP3	0	0	0	0	0	0	0	0
COX7BP4	0	0	0	0	0	0	0	0
COX7BP6	0	0	0	0.833969	0	0	0	0
COX7C	1045.87798	722.328629	1066.80516	482.959138	587.289689	360.731696	268.792723	818.512446
COX7CP1	0	2.116536	0	0	0	0	0	0
COX7CP2	0	0	0	0	0	0	0	0

COX7CP3	0	0	0	0	0	0	0	0
COX7CP4	0	0	0	0	0	0	0	0
COX8A	202.224882	153.544504	215.745558	155.013909	367.085378	262.738177	217.797416	284.360799
COX8CP1	0	0	0	0	0	0	0	0
CP	0	0.406023	0	0	0	0	0	0
CPA1	0	0	0	0	0	0	0	0
CPA2	0	0	0	0	10.715986	0	0	0.560316
CPA3	0	0	0	0	0	0	0	0
CPA4	35.069896	24.519312	48.918594	32.909292	86.649281	32.979759	96.812041	47.752382
CPA5	0	0.628738	0	0	0	2.69002	0	0
CPA6	11.198226	1.301593	6.418407	0	0	0.52561	0	0.753476
CPAMD8	0	0	0	0.078988	0	0.439081	0	0.131073
CPB1	0	0	0	0	0	0	0	0
CPB2	0	0	0	0	0	0	0	0
CPD	21.053281	27.812142	49.446441	32.687174	127.713497	30.726803	186.600373	28.17086
CPDP1	0	0	0	0	0	0	0	0
CPE	3.942549	4.082497	3.528483	8.680617	0	2.575417	0	1.735883
CPEB1	0	1.173018	0	0.132264	0	3.971272	0	1.05657
CPEB2	0	0.087818	0	0.051982	0	0.551003	0	0.999711
CPEB3	0	0.435431	0.373202	0.641303	0	0.544071	0	0.496129
CPEB4	0.966645	4.470804	2.631233	6.108308	0.721743	6.247565	9.580711	11.979196
CPED1	0.615565	0.105748	0	0.844442	0	0	0	0.190087
CPHL1P	0	0	0	0	0	0	0	0
CPHXL	0	0	0	0	0	0	0	0
CPHXL2	0	0	0	0	0	0	0	0
CPLANE1	23.735196	14.425189	7.614173	3.535632	37.26991	3.712035	39.356429	8.614894
CPLANE2	4.533851	12.047729	2.017943	4.884771	4.930183	17.95299	91.420391	7.547111
CPLX1	0	2.682331	0	1.270949	0	1.621534	20.888783	1.670139
CPLX2	0	0	0	0	0	0	0	0
CPLX3	0	0	0	0	0	0	0	0
CPLX4	0	0	0	0	0	0	0	0
CPM	3.424214	3.827393	15.209327	11.187356	16.090357	8.977512	0	0.313777
CPN1	0	0	0	0	0	0	0	0

CPN2	0	0	0	0	0	0	0	0
CPNE1	41.843083	115.987345	34.118458	125.541722	164.476644	119.365732	108.486558	90.847212
CPNE2	22.17175	25.585427	21.146389	26.006154	7.537687	22.287052	32.649151	22.948821
CPNE3	41.140987	88.681503	65.033825	55.20044	43.554057	32.001427	133.469964	72.155082
CPNE4	0	0.884968	1.585625	0.833931	0	0	0	0
CPNE5	0	0.127933	0	0.06966	0	0.265654	0	0.107626
CPNE6	0	0	0	0	0	0	0	0
CPNE7	1.39565	10.23566	23.69442	11.170059	63.051403	14.281953	0	10.189952
CPNE8	16.391644	8.508205	1.785894	4.960905	5.238457	7.204995	96.664865	13.395537
CPNE9	0	0	0	0	0	0	0	0
CPOX	21.2176	16.53331	18.996339	15.198371	2.305364	24.114726	111.09298	14.516978
CPP	0	0	0	0	0	0	0	0
CPPED1	0	0.543176	7.056014	1.404714	0.139749	1.470669	13.917011	3.039671
CPQ	1.802754	0.331551	0	0.743981	0	0.623864	0	2.439048
CPS1	4.957597	0	0	0	0	0.248497	0	0.523947
CPSF1	29.295651	18.434551	24.475581	22.960309	33.191324	36.18765	82.398593	26.459837
CPSF1P1	0	0	0	0	0	0	0	0
CPSF1P2	0	0	0	0	0	0	0	0
CPSF2	54.07884	154.457988	96.807506	113.033908	48.157476	100.83163	71.433053	140.495608
CPSF3	69.860774	56.372906	68.121686	39.515438	41.95551	26.91494	98.951064	53.935193
CPSF4	15.380917	52.01966	24.006889	32.027119	31.073487	36.76699	12.480687	37.70164
CPSF4L	0	0	0	0.481354	0	0.318621	0	0
CPSF6	71.48105	105.09171	45.845583	89.045035	119.439192	81.20668	154.138097	90.974059
CPSF6P1	0	0	0	0	0	0	0	0
CPSF7	39.766118	26.249937	35.92925	42.823069	0	94.084485	196.922956	79.093602
CPT1A	64.231117	74.651064	104.367189	99.10722	36.407999	49.48887	27.352897	37.780441
CPT1B	0	1.675961	8.874527	0.118896	0.408796	0.94929	0	0
CPT1C	1.782365	7.034415	0	5.926561	0	3.737403	0	2.494283
CPT2	52.250798	44.683336	48.239226	44.374439	12.849884	14.162147	1.789394	18.142654
CPTP	11.197497	31.750204	15.722599	35.094382	16.201622	55.305482	15.503124	43.270165
CPVL	19.593997	14.421621	19.869579	14.041324	23.534365	8.335526	131.262876	7.841294
CPXCR1	0	0	0	0.058586	0	0	0	0
CPXM1	0	0	0	0.314768	0	0.927704	0	0.405425

CPXM2	0	0.040112	0	0.032947	0	0	0	0
CPZ	11.146584	16.242505	18.967106	17.575497	4.682752	3.002362	0	1.73344
CR1	0	0	0	0	0	0	0	0
CR1L	0	0	0	0	0	0	0	0
CR2	0	0.073073	0	0.364712	0.225353	0.444501	0	0.358892
CRABP1	0	0.915244	11.921787	3.111322	0	0	0	0
CRABP2	83.74298	141.657617	52.734975	151.138351	80.613955	139.010328	123.586881	71.059489
CRACD	1.341422	7.412847	0	2.122271	0	0.692097	0	0.565194
CRACDL	0	0.447296	0	0.247035	1.35358	9.900578	0	8.987198
CRACR2A	11.351758	10.159384	1.468336	8.173134	7.096135	14.055783	12.108674	18.524923
CRACR2B	6.391646	9.069891	1.420753	12.215103	7.296599	24.155865	25.382622	13.159342
CRADD	0	2.029553	0	2.615449	10.092957	10.073463	61.849404	13.12531
CRADDP1	0	0.600494	0	0	0	0	0	0
CRAMP1	19.360832	17.813679	0	8.339653	17.619379	19.662477	2.360569	13.118308
CRAT	1.523116	5.983866	3.276294	8.094741	4.912915	16.165041	5.907121	12.181962
CRB1	0	0	0	0.102739	0	0	0	0
CRB2	0	0.224496	0	0.253478	0	0	0	0
CRB3	25.943244	31.544904	27.790735	47.018698	50.340639	43.226539	18.214494	34.513171
CRB3P1	0	0	0	0	0	0	0	0
CRBN	43.236323	39.172973	19.500807	24.634429	52.795058	14.299355	0	9.082158
CRCP	32.428757	50.419117	29.41882	60.488133	25.245733	45.302927	57.983564	47.685037
CRCT1	0	0	0	0	0	0.55788	0	0
CREB1	32.476945	134.649931	54.921117	129.870227	70.697616	165.159879	142.184634	188.132188
CREB3	24.016553	46.856408	23.49878	60.664749	12.015242	79.149268	43.446719	71.565795
CREB3L1	0	0	0	0.419141	0	0	0	0
CREB3L2	25.18981	18.13633	6.285909	29.070308	0	20.522104	0	13.130418
CREB3L3	0	0	1.173248	0.031914	0	0	0	0
CREB3L4	23.755116	20.925251	17.949175	25.702293	21.691217	23.709394	0	28.743848
CREB5	1.508936	1.377848	5.280734	2.418984	0	0.531167	0	1.490537
CREBBP	24.088273	65.46739	32.281234	59.860054	65.843215	81.104323	150.109128	88.418602
CREBL2	14.940596	15.965974	13.404538	10.073877	14.852172	17.287668	18.64347	15.522319
CREBRF	2.075174	8.069545	9.509073	8.654414	10.144505	7.732185	38.668088	14.044719
CREBZF	6.30772	11.454197	2.697491	18.670676	2.11144	31.066128	0	17.256923

CREG1	31.777097	11.35203	34.670964	8.229414	29.319798	7.334244	22.221705	10.540996
CREG2	7.459132	37.45946	11.433199	23.114762	15.698814	43.608215	4.150985	44.401437
CRELD1	0	3.513215	0	4.953479	0	0	0	0
CRELD2	0	9.908849	4.304696	5.462944	46.505496	15.299514	0	9.37448
CREM	23.564439	17.78968	0	10.090163	28.077401	7.067173	0	11.807036
CRH	0	0	0	0	0	0	0	0
CRHBP	0	0	0	0	0	0	0	0
CRHR1	0	0	0	0	0	0	0	0
CRHR2	0	0	0	0	0	0	0	0
CRIM1	49.43294	87.706306	59.213318	83.198672	112.189902	91.070588	57.001964	59.592614
CRIP1	0	4.182858	0	2.498835	0	0	0	0.786132
CRIP1P1	0	0	0	0	0	0	0	0
CRIP1P2	0	0	0	0	0	0	0	0
CRIP1P3	0	0	0	0	0	0	0	0
CRIP1P4	0	0	0	0	0	0	0	0
CRIP2	7.807596	14.875306	11.672703	17.084883	17.088976	15.331949	0	3.377867
CRIP3	0	0	0	0.754117	0	0	0	0
CRIPT	0	0	0	0	0	0	0	0
CRIPTO	0	0	0	0	0	0	0	0
CRIPTOP1	0	0	0	0	0	0	0	0
CRIPTOP2	0	1.297895	0	0	0	0	0	0
CRIPTOP4	0	0	0	0	0	0	0	0
CRIPTOP5	0	0	0	0	0	0	0	0
CRIPTOP6	0	0	0	0	0	0	0	0
CRIPTOP7	0	0.653599	0	0	0	0	0	0
CRISP1	0	0	0	0	0	0	0	0
CRISP2	0	0	0	0	0	0	0	0
CRISP3	0	0	0	0.109394	0	0	0	0
CRISPLD1	7.081408	15.831567	6.512559	16.554313	2.683497	6.142963	0	4.558566
CRISPLD2	13.071681	6.281471	9.650738	10.690486	14.793288	6.029314	0	5.779417
CRK	39.667546	113.1583	37.932044	94.939125	71.112674	161.602329	50.067032	155.04966
CRKL	26.274309	64.275906	35.138906	74.703858	80.010178	105.342842	48.234917	117.279323
CRLF1	0	6.058493	19.553774	5.046354	0	0.910348	0	0.654237

CRLF2	0	0	0	0	0	0	0	0
CRLF3	11.719853	23.453842	20.994874	20.156766	13.609125	14.651751	7.541034	23.666682
CRLF3P1	0	0	0	0	0	0	0	0
CRLF3P2	0	0	0	0	0	0	0	0
CRLF3P3	0	0	0	0	0	0	0	0
CRLS1	46.010841	22.353504	34.187248	4.91083	52.59741	6.173078	0	28.945047
CRMP1	3.664475	0	0	0	0	0.515782	0.339435	0.933933
CRNKL1	0	2.057003	11.40278	2.661223	21.609409	9.179279	0	5.891337
CRNN	0	0	0	0	0	0	0	0
CROCC	2.225776	1.461875	0.87524	3.083064	12.607483	9.36529	0	4.345983
CROCC2	0	0	0	0	0	0	0	0
CROCCP1	0	0	0	0	0	0	0	0
CROCCP2	0	0	0	0.323674	0	0	0	0.47176
CROCCP4	0	0	0	0	0	0	0	0
CROCCP5	0	0	0	0.232607	0	0	0	0
CROT	1.741978	4.662645	5.087127	2.566232	11.644817	1.55969	10.54319	9.312672
CRP	0	0	0	0	0	0	0	0
CRPP1	0	0	0	0	0	0	0	0
CRPPA	0	1.043285	0	0.101658	0.29964	0.19895	0	0.181941
CRTAC1	0	0.334933	18.70723	6.503883	0	0	0	0
CRTAM	0	0.381779	0	0.355448	0	0	0	0
CRTAP	106.503069	38.416864	74.238123	48.581042	56.86861	33.647378	57.713614	24.550911
CRTC1	1.9098	2.37924	2.101073	6.30235	2.736802	12.779933	5.194763	7.345636
CRTC1P1	0	0	0	0	0.491169	0.092065	0	0
CRTC2	15.454458	38.696199	20.803076	27.483112	11.701905	29.511515	126.017525	21.431682
CRTC3	11.278409	14.701972	49.466752	17.79215	40.859006	21.119559	8.862357	20.374981
CRX	0	0	0	0	0	0	0	0
CRY1	8.994613	28.011328	10.073284	17.999375	14.869571	18.099328	0.273143	24.794303
CRY2	4.705088	0.661829	2.111813	1.914913	5.177543	5.759935	3.626598	1.700222
CRYAA	0	0	0	0.079404	0	0	0	0
CRYAB	17.63726	0.978183	0	6.810903	1.610959	2.443007	0	3.667664
CRYBA1	0	0	0	0	0	0	0	0
CRYBA2	4.38742	5.549106	0	3.326782	0	1.987444	0	0.427471

CRYBA4	0	0	0	0	0	0	0	0
CRYBB1	0	0	0	0	0	0	0	0
CRYBB2	0	0	0	0	0	0.698347	0	0
CRYBB3	0	0	0	0.224179	0	0	0	0
CRYBG1	0	6.644352	0	0	0	24.194302	0	0
CRYBG2	0	0.083506	2.686751	1.139923	0	0.091987	15.993274	1.241711
CRYBG3	13.386689	14.686044	15.799152	11.393982	11.402167	6.445674	7.406006	12.525105
CRYGB	0	0	0	0	0	0	0	0
CRYGC	0	0	0	0	0	0	0	0
CRYGD	0	0	0	0	0	0	0	0
CRYGEP	0	0	0	0	0	0	0	0
CRYGFP	0	0	0	0	0	0	0	0
CRYGGP	0	0	0	0	0	0	0	0
CRYGN	0	0	0	0	0	0	0	0
CRYGS	0	0.639908	0	0.056407	1.251114	0	0	0.738698
CRYL1	8.241061	7.28706	17.258664	14.04497	7.228584	9.991107	14.64473	6.187933
CRYM	0	0	0	0.327256	0	0	0	0
CRYZ	48.788837	30.880427	30.991386	22.608683	9.431276	20.216393	18.473844	22.686799
CRYZL1	9.710204	11.925677	55.415878	10.756936	51.756028	5.473128	62.801412	18.48995
CRYZL2P	0	1.858278	0	2.033483	8.631268	0	0	0
CRYZP1	0	0	0	0	0	0	0	0
CRYZP2	0	0	0	0	0	0	0	0
CS	206.248883	128.015909	175.42703	165.95866	79.271701	98.109937	264.473899	69.729209
CSAD	7.8431	2.303964	0	1.854833	0	1.667608	0	5.124664
CSAG1	0	0	0	0	0	0.989563	0	0
CSAG2	0	0.421587	0	0.319467	0	0	0	0
CSAG3	4.772564	0.421587	0	0.319467	0	0.360738	0	0
CSAG4	0	0	0	0	0	0	0	0
CSDC2	0	0	0	0.228876	0	0.467236	0	0.270451
CSDE1	347.128899	607.186551	369.705207	575.29209	301.790393	449.731125	356.523155	727.72708
CSE1L	182.136561	98.047084	137.144092	43.741725	176.504179	31.52112	191.498253	111.485773
CSF1	2.481581	15.987191	22.463087	30.74843	7.952302	34.538327	46.273832	27.85921
CSF1R	0	0	0	0.379404	0	1.198867	0	0.326962

CSF2	10.429457	10.555969	36.524273	28.552772	38.241346	29.564049	26.005005	29.133914
CSF2RA	0	0	0	0	0	1.314646	0	0.639312
CSF2RB	0	0	0	0	0	0	0	0
CSF2RBP1	0	0	0	0	0	0	0	0
CSF3	0	0	0	0.29867	0	0	0	0
CSF3R	0	0	0	0	0	0	0	0
CSGALNACT1	0	0.32927	0	1.705837	0	0.391558	0	0.068647
CSGALNACT2	12.321198	14.614362	18.950341	13.286374	8.365991	8.175945	3.639722	14.707116
CSGALNACT2	0	0	0	0	0	0	0	0
CSGALNACT2	0	0	0	0	0	0	0	0
CSH1	0	0	0	0	0	0	0	0
CSH2	0	0	0	0	0	0	0	0
CSHL1	0	0	0	0	0	0	0	0
CSK	52.318688	50.331348	24.67941	49.383498	62.120117	54.348118	144.123946	54.975537
CSKMT	15.918417	19.033662	66.256813	5.313118	22.557913	15.529555	0	3.827495
CSMD1	0	0	0	0	0	0	0	0
CSMD2	0	0	0	0.132434	0	0	0	0
CSMD3	0	0.218235	0	0.167646	0	0	0	0.035763
CSN1S1	0	0	0	0	0	0	0	0
CSN1S2AP	0	0	0	0	0	0	0	0
CSNK1A1	110.624941	187.850469	172.898144	293.267475	110.771126	412.829096	121.058945	137.664459
CSNK1A1L	0	0	0	0	0	0	0	0
CSNK1A1P1	0	0	0	0	0	0	0	0
CSNK1A1P3	0	0	0	0	0	0	0	0
CSNK1D	0.848505	51.316091	3.688503	52.988971	17.012585	32.33495	142.959359	48.403783
CSNK1E	97.242311	136.152712	91.616096	170.634386	169.804449	159.944336	66.511417	109.428229
CSNK1G1	15.028162	49.287835	21.764959	55.208181	19.736094	38.498447	52.789316	54.860201
CSNK1G2	30.224375	116.843674	36.444883	100.680821	89.804543	141.111872	20.272266	126.861241
CSNK1G2P1	0	0	0	0	0	0	0	0
CSNK1G3	38.657844	90.103111	60.169273	91.983049	43.865546	55.534303	11.971201	92.627677
CSNK2A1	164.328052	308.607466	167.829546	326.035115	198.903764	226.380121	122.425375	309.945357
CSNK2A2	66.389233	125.038396	92.961462	140.588762	78.557354	118.826953	179.4433	160.096643
CSNK2B	164.923744	204.502026	148.260817	202.314825	166.252257	242.741453	118.798087	271.510813

CSNKA2IP	0	0	0	0	0	0	0	0
CSP1	0	0	0	0	0	0	0	0
CSP2	0	0	0	0	0	0	0	0
CSPG4	0.388821	0.720213	1.749101	0.808905	1.541439	3.280535	1.422788	1.230812
CSPG4BP	0	0	0	0	0	0	0	0
CSPG4P10	1.029017	0	0	0.531371	0	0.739586	0	0
CSPG4P11	0.993611	1.938705	0.599039	1.165956	0	1.187306	0	0.95561
CSPG4P12	0	0	0	0	0	0	0	0
CSPG4P13	0	0.029291	0	0	0	0	0	0
CSPG4P3Y	0	0	0	0	0	0	0	0
CSPG4P4Y	0	0	0	0	0	0	0	0
CSPG4P5	0	0	0	0.364267	1.40807	0.310276	0	0
CSPG5	0	1.450812	1.490025	1.286144	0	0.409571	0	0.201858
CSPP1	5.355278	1.835541	0	3.221484	25.819941	0.424811	8.951788	0.450153
CSRNP1	9.517997	15.352928	9.478644	11.957586	12.841277	14.290933	0.257018	20.240228
CSRNP2	0	5.288566	0	10.388445	40.98425	15.486726	0	5.066054
CSRNP3	10.046755	0.173579	0	2.736869	0	0.861053	0	0.342538
CSRP1	61.636457	64.364118	32.639858	62.621145	52.284697	146.358908	272.717524	119.46785
CSRP1-AS1	0	8.442302	1.314215	7.386432	0	2.921144	0	3.366289
CSRP2	109.209445	74.790038	68.904008	79.354188	44.517843	57.072096	57.098938	79.228649
CSRP2P2	0	0	0	0	0	0	0	0
CSRP3	0	0	0	0	0	0	0	0
CST1	0	0	0	0	0	0	0	0
CST11	0	0	0	0	0	0	0	0
CST12P	0	0	0	0	0	0	0	0
CST13P	0	0	0	0	0	0	0	0
CST2	0	0	0	0	0	0	0	0
CST2P1	0	0	0	0	0	0	0	0
CST3	80.838613	140.469052	140.942599	203.671303	169.710021	257.638708	105.687107	176.090178
CST5	0	0	0	0	0	0	0	0
CST6	0	17.265902	0	18.83768	26.12611	29.361851	0	21.180675
CST7	0	0	0	0	0	0	0	0
CST8	0	0	0	0	0	0	0	0

CST9L	0	0	0	0	0	0	0	0
CST9LP1	0	0	0	0	0	0	0	0
CST9LP2	0	0	0	0	0	0	0	0
CSTA	112.478347	100.353774	171.939885	138.957352	151.58748	69.592861	75.953664	91.680673
CSTB	0	0	2.463259	0	0	0	0	0.347021
CSTBP1	0	0	0	0	0	0	0	0
CSTF1	34.002598	54.301331	58.702113	44.127829	84.421618	41.828125	47.757019	54.863373
CSTF2	10.542391	0	0	15.680755	13.410184	10.213388	16.236954	5.082259
CSTF3	20.493355	22.419765	24.70063	28.86625	14.240725	35.093894	61.433234	50.57959
CSTL1	0	0	0	0	0	0	0	0
CSTP1	0	0	0	0	0	0	0	0
CSTP2	0	0	0	0	0	0	0	0
CSTPP1	18.819849	19.898749	15.306701	19.132059	53.960088	23.92831	47.055324	21.242098
CT45A1	0	0	0	0	0	0	0	0
CT45A10	0	0	0	0	0	0	0	0
CT45A11P	0	0	0	0	0	0	0	0
CT45A2	0	0	0	0	0	0	0	0
CT45A3	0	0	0	0	0	0	0	0
CT45A5	0	0	0	0.137862	0	0	0	0
CT45A6	0	0	0	0	0	0	0	0
CT45A7	0	0	0	0	0	0	0	0
CT45A8	0	0	0	0	0	0	0	0
CT47A10	0	0	0	0	0	0	0	0
CT47A11	0	0	0	0	0	0	0	0
CT47A12	0	0.133357	0	0	0	0	0	0
CT47A6	0	0	0	0	0	0	0	0
CT55	0	0	0	0	0	0	0	0
CT62	0	1.337704	0	3.597872	0	0.730194	0	1.958881
CT75	0	0	0	0	0	0	0	0
CT83	0	0	0	0	0	0	0	0
CTAG1A	0	0	0	0	0	0	0	0
CTAG1B	0	0	0	0	0	0	0	0
CTAG2	0	0	0	0	0	0	0	0

CTAGE1	0	0	0	0	0	0	0	0
CTAGE10P	0	0	0	0	0	0	0	0
CTAGE11P	0	0	0	0	0	0	0	0
CTAGE12P	0	0	0	0	0	0	0	0
CTAGE13P	0	0	0	0	0	0	0	0
CTAGE14P	0	0	0	0	0	0	0	0
CTAGE15	0	0	0	0	0	0	0	0
CTAGE16P	0	0	0	0	0	0	0	0
CTAGE3P	0	0	0	0	0	0	0	0
CTAGE6	0	0	0	0	0	0	0	0
CTAGE7P	0	0	0	0	0	0	0	0
CTBP1	0	74.923561	0.704411	70.793077	37.199123	165.219635	62.985805	70.535451
CTBP2	34.354388	99.093474	29.476425	77.889646	71.096023	126.131478	53.780966	148.686328
CTBP2P1	0	0	0	0	0	0	0	0
CTBP2P10	0	0.261602	0	1.15127	0	3.51266	0	4.829518
CTBP2P2	0	0	0	0	0	0	0	0
CTBP2P3	0	0	0	0	0	0	0	0
CTBP2P4	0	0	0	1.76815	0	0.227634	0	0.754452
CTBP2P5	0	0	0	0	0	0	0	0
CTBP2P6	0	0	0	0	0	0	0	0
CTBP2P7	0	0	0	0	0	0	0	0
CTBP2P8	0	0.232228	0	0.200043	0	0.270546	0	0.558866
CTBS	25.747951	19.019199	28.265187	7.821104	8.340318	2.39484	0	5.813883
CTC1	5.198614	2.615135	3.069597	5.265263	12.393675	4.712275	0	4.236726
CTCF	40.616248	91.372046	28.84219	100.422218	66.796503	109.578834	82.94379	107.372667
CTCFL	0	0	0	0	0	0	0	0
CTDNEP1	73.746281	109.543831	70.495767	105.270729	224.21073	128.236716	345.513098	132.720352
CTDNEP1P1	0	0	0	3.312096	0	0	0	0.707577
CTDNEP1P2	0	0	0	0	0	0	0	0
CTDP1	6.211939	26.062587	16.014247	9.375977	9.427327	19.885544	2.065412	21.169004
CTDSP1	0	4.703129	9.746484	4.008483	2.926798	12.037202	0	9.316938
CTDSP2	87.679447	231.520178	102.746476	238.207304	148.027562	310.545009	119.012825	293.212348
CTDSPL	12.521016	13.984875	6.987051	7.548019	24.685385	14.726124	35.624862	18.420772

CTDSPL2	89.589352	157.895074	74.538529	157.182773	74.641556	177.038406	186.651364	174.320431
CTDSPL2P1	0	0	0	0	0	0	0	0
CTDSPL2P2	0	0	0	0	0	0	0	0
CTF1	0	1.017527	1.958159	0	0	0	0	0.889347
CTF2P	0	0	0	0	0	0	0	0
CTH	13.824994	0	0	7.891945	0.818695	2.21118	8.227691	1.24702
CTHRC1	18.643117	25.232187	34.388389	25.145025	0.81709	0	0	0
CTHRC1P1	0	0	0	0	0	0	0	0
CTIF	3.953054	19.960229	13.8657	20.446236	3.942241	29.124167	18.245672	5.002771
CTLA4	0	0	0	0	0	0	0	0
CTNNA1	176.448192	172.573378	140.979204	299.567852	252.041449	330.78509	608.021905	245.977016
CTNNA1P1	0	0.263839	0	0.060341	0	0.061625	0	0
CTNNA2	0	0	0	0.029435	0	0	0	0
CTNNA3	0	0	0	0.582603	0	0	0	0
CTNNAL1	188.369954	296.593915	142.824086	110.257892	151.006729	80.15081	141.441344	237.074828
CTNNB1	233.59547	296.744305	209.236131	315.731558	229.644243	332.209316	248.878865	329.355874
CTNNBIP1	52.586898	55.329684	26.232376	39.828377	42.948545	75.994824	0.740782	64.077456
CTNNBL1	59.10801	107.572029	59.333467	108.871772	68.408137	98.227707	57.138868	110.102482
CTNNBL1P1	0	0	0	0	0	0	0	0
CTNND1	65.607815	120.621434	68.829422	157.602408	144.557764	246.248176	214.380368	215.498508
CTNND2	0	0	0	0	0	0.112204	0	0
CTNS	0	4.244325	16.559151	8.200239	0	9.461691	25.780104	14.054101
CTPS1	65.30952	70.998556	33.515695	65.289652	95.594943	115.630359	98.088451	93.904078
CTPS2	7.525719	9.97487	5.252823	7.632189	18.335125	17.168581	5.503905	25.235274
CTR9	23.565045	31.952229	18.426689	16.977897	44.853128	39.632678	49.038888	41.52708
CTRB1	0	0	0	0	0	0	0	0
CTRB2	0	0	0	0	0	0	0	0.376065
CTRC	0	0	0	0	0	0	0	0
CTRL	0	0	0	0.79209	0	0.992794	0	2.637902
CTSA	0	0	46.382957	0.104651	0	1.95335	37.224194	1.028613
CTSB	36.957419	42.506911	42.941643	55.495996	79.555876	38.967603	236.838594	54.368918
CTSC	67.246562	29.118774	36.502922	32.61168	64.035396	18.560861	35.268834	19.199154
CTSD	83.989848	71.287949	134.243348	108.874631	223.375803	176.652911	124.405764	103.146822

CTSE	0	0	0	0	0	0.153916	0	0
CTSF	1.694224	0.360667	0	0.678195	0	2.431894	0	1.054674
CTSG	0	0	0	0	0	0	0	0
CTSH	0	3.571037	0	3.063488	0	1.236582	0	1.838258
CTSK	2.180665	0	0	0.686696	0	0	0	0.44094
CTSL	0	11.853394	15.646024	20.100824	0	20.925702	0	14.243143
CTSL3P	0	0	0	0	0	0	0	0
CTSLP1	0	0	0	0	0	0	0	0
CTSLP2	0	0	0	0	0	0	0	0
CTSLP3	0	0	0	0	0	0	0	0
CTSLP4	0	0	0	0	0	0	0	0
CTSLP6	0	0	0	0	0	0	0	0
CTSLP8	0	0	0	0	0	0	0	0
CTSO	0	1.581557	3.114718	0.819016	0	0.288553	0	0.652777
CTSS	2.519938	0.916369	2.259829	0.667198	5.121422	0	0	0.320598
CTSV	0	3.071285	0	4.153946	0	8.311559	0	9.608892
CTSW	0	0	0	0	0	0	0	0
CTSZ	148.224981	225.515353	178.639581	256.98323	181.930094	223.307366	39.791506	152.377544
CTTN	254.543455	406.602288	243.349076	506.701694	196.111253	372.716987	146.05333	289.572195
CTTNBP2	0	0	0	0	0	0	0	0
CTTNBP2NL	19.420796	36.694207	25.436267	51.279704	13.34111	44.337574	44.373613	44.653721
CTU1	0	1.169185	2.899484	2.247713	8.557796	2.974127	0	1.243258
CTU2	21.273131	15.428604	11.395637	19.771864	9.959684	15.319982	19.532909	16.063278
CTXN1	2.992149	17.31827	5.305335	21.787196	0	15.622798	0	7.898477
CTXN2	0	0.438063	1.119633	0.031319	0	0.053086	0	0
CTXN3	0	0	0	0	0	0	0	0
CTXND1	0	0	0	0	0	0	0	0
CTXND2	0	0	0	0	0	0	0	0
CUBN	0	0	0	0	0	0	0	0
CUBNP1	0	0	0	0	0	0	0	0
CUBNP2	0	0	0	0	0	0	0	0
CUBNP3	0	0	0	0	0	0	0	0
CUEDC1	20.351827	34.770204	23.171003	26.691914	2.071403	41.811285	38.281633	25.857755

CUEDC2	48.152551	89.630243	36.499136	114.094535	40.676224	88.745472	25.343226	51.393459
CUL1	39.724741	91.023004	29.518866	59.109583	57.284978	73.630954	25.436027	138.854524
CUL1P1	0	0	0	0	0	0	0	0
CUL2	0	2.279401	2.961733	1.395472	9.2886	2.080608	0	2.282982
CUL3	48.002345	100.789781	47.248725	38.276132	71.416282	43.504571	95.749287	133.979236
CUL4A	62.556559	121.470907	79.687284	115.790371	81.640136	124.164713	38.596493	166.544053
CUL4AP1	0	0	0	0	0	0	0	0
CUL4B	0	0	0	0	0	0	0	0
CUL5	27.833937	93.6166	47.091588	57.17268	72.832378	44.336625	25.572493	87.845641
CUL9	2.101181	3.866254	1.890083	3.529673	21.760765	6.481154	26.572189	3.608668
CUPIN1P	0	0	0	0	0	0	0	0
CUTA	72.91118	101.167897	7.988693	95.416851	107.470006	110.199997	1.174181	98.374628
CUTALP	1.772149	13.788016	7.593048	8.972209	3.977687	0.962268	1.501204	4.868676
CUTC	13.723141	38.591462	17.056313	35.924909	38.368111	37.57424	29.072975	34.726639
CUX1	18.721381	32.200358	12.312586	35.684325	47.303718	35.659459	23.721644	34.714888
CUX2	0.483471	0.603207	0.869483	0.307492	0	0.121273	0	0.182586
CUX2P1	0	0	0	0	0	0	0	0
CUZD1	0	0.293066	0	0.197268	0	0.784647	0	0.556147
CWC15	195.140127	285.395992	161.758426	226.116271	65.735883	107.89801	103.315761	123.642729
CWC22	17.490344	23.711912	11.00137	19.872646	24.178026	20.421803	39.468141	32.506793
CWC25	10.29655	16.879322	9.795535	15.189469	11.295944	17.856075	10.255365	11.718319
CWC27	40.20753	46.379166	23.940814	36.995177	22.866998	19.324958	0	37.706594
CWF19L1	18.556542	25.067464	28.123118	29.352206	48.331358	16.477935	27.4801	29.459454
CWF19L2	0	0	0	0	0	0	7.232746	0
CWH43	1.615218	2.845276	0	0.983988	0	0.080018	0	0.945437
CX3CL1	0	0	0	0.049568	1.609725	2.802977	0	1.715862
CX3CR1	0	0	0	0	0	0	0	0
CXADR	11.070886	18.922704	29.939844	20.953789	24.343709	22.703832	49.711212	22.598078
CXADRP1	0	0	0	0	0	0	0	0
CXADRP2	0	0	0	0	0	0	0	0
CXADRP3	0	0	0	0	0	0	0	0
CXCL1	0	0	0	0.304894	0	1.014167	0	0
CXCL10	0	0	0	0	0	0	0	0.12924

CXCL11	0	0	0	0	0	0	0	0.222138
CXCL12	0	0	0	0	0	0	0	0
CXCL13	0	0	0	0	0	0	0	0
CXCL14	0	0	0	0	0	0	0	0
CXCL16	0	19.405777	0	32.595829	9.338056	38.207013	0	19.655731
CXCL17	0	0	0	0	0	0	0	0
CXCL1P1	0	0	0	0	0	0	0	0
CXCL2	0	5.888708	23.948315	9.95906	36.467432	9.577511	28.08903	11.051655
CXCL3	7.07186	3.816722	6.250419	6.343515	11.149222	5.932389	0	2.578341
CXCL5	0	0.17845	0	0.0681	0.738303	3.544011	6.109833	3.325471
CXCL6	2.3289	5.379316	6.21716	4.730115	1.839919	6.033692	0	4.250218
CXCL8	97.255213	55.577289	151.238966	107.667179	35.867193	30.762602	19.835952	36.874715
CXCR2	0	0.10067	0	0.43151	0	0.293887	0	0.950111
CXCR2P1	0	0	0	0	0	0	0	0
CXCR3	0	0	0	0	0	0	0	0
CXCR4	0	0	0	0.117173	0	0	0	0
CXCR6	0	0	0	0	0	0	0	0
CXorf38	4.657579	5.829172	10.452915	7.007536	8.1781	4.663203	10.770417	5.789877
CXorf49	0	0	0	0	0	0.827929	0	2.269357
CXorf58	0	0.368263	0	0	0	0	0	0
CXorf65	0	0	0	0	0	0	0	0
CXXC1	9.020996	16.029325	42.376839	11.233805	14.099989	14.120687	49.681173	9.348245
CXXC1P1	0	0	0	0	0	0	0	0
CXXC4	0	0.340933	0	0.31461	0	0	0	0
CXXC5	18.89471	34.12345	4.66794	36.714512	7.780768	41.994206	0	36.000006
CYB561	45.923178	50.832308	44.224244	57.226821	17.87716	29.421859	7.808521	22.143494
CYB561A3	14.731705	18.244498	13.885783	22.168392	33.067707	35.404307	7.754468	39.038394
CYB561D1	15.818095	13.991598	7.463737	13.449146	0.359798	12.863551	0	9.561103
CYB561D2	6.021027	9.662357	2.716043	10.812954	4.83618	11.268283	0	10.224165
CYB5A	58.420261	80.753632	60.856369	58.694332	58.17866	95.347358	27.549348	70.035175
CYB5AP2	0	0	0	0	0	0	0	0
CYB5AP3	0	0	0	0	0	0	0	0
CYB5AP4	0	0	0	0	0	0	0	0

CYB5AP5	0	0	0	0	0	0	0	0
CYB5B	0	0.463098	0	0	0	0	0	0.081402
CYB5D1	17.164303	27.64829	15.922929	25.763738	26.886618	29.302807	0	38.27468
CYB5D2	0	18.133578	0	21.113318	0.591127	17.197941	0	16.81146
CYB5R1	10.941174	10.13419	17.516293	14.166268	7.493361	17.254887	1.105145	8.876485
CYB5R2	0	1.943028	0	4.966692	0	2.205671	0	4.59122
CYB5R3	0	5.216168	0	6.890889	0	3.452239	21.800589	5.175385
CYB5R4	9.117252	21.776481	23.757555	4.007477	22.584592	6.107032	57.228365	22.576069
CYB5RL	9.59056	28.69692	8.190067	26.130715	9.222212	15.409505	4.394629	13.550978
CYBA	149.781004	94.89288	205.102726	172.054498	138.065341	89.481099	83.455507	59.663551
CYBB	0	0	0	0	0	0	0	0
CYBC1	20.379437	53.407219	3.804145	51.37115	34.263097	75.63263	54.823277	56.679116
CYBRD1	18.811487	16.412836	11.872837	14.349419	32.101786	13.017604	37.550552	16.632191
CYC1	115.764737	86.076922	91.465014	81.055842	104.717249	87.299702	50.358513	63.919266
CYCS	321.512578	378.754009	186.390442	308.067809	272.574465	339.053422	300.600044	252.450296
CYCSP1	0	0	0	0	0	0	0	0
CYCSP10	0	0	0	0	0	0	0	0
CYCSP11	0	0	0	0	0	0	0	0
CYCSP12	0	0	0	0	0	0	0	0
CYCSP17	0	0	0	0	0	0	0	0
CYCSP19	0	0	0	0	0	0	0	0
CYCSP2	0	0	0	0	0	0	0	0
CYCSP20	0	0	0	0	0	0	0	0
CYCSP22	0	0	0	0	0	0	0	0
CYCSP23	0	0	0	0	0	0	0	0
CYCSP24	0	0.760311	0	1.478733	0	7.436833	0	4.803257
CYCSP25	0	0	0	0	0	0	0	0
CYCSP26	0	0	0	0	0	0	0	0
CYCSP27	0	0	0	0	0	0	0	0
CYCSP28	0	0	0	0	0	0	0	0
CYCSP29	0	0	0	0	0	0	0	0
CYCSP30	0	0	0	0	0	0	0	0
CYCSP32	0	0	0	0	0	0	0	0

CYCSP33	0	0	0	0	0	0	0	0
CYCSP34	0	0	0	0.529936	0	0	0	0
CYCSP35	0	0	0	0	0	0	0	0
CYCSP38	0	0	0	0	0	0	0	0
CYCSP39	0	0	0	0	0	0	0	0
CYCSP4	0	0	0	0	0	0	0	0
CYCSP40	0	0	0	0	0	0	0	0
CYCSP41	0	0	0	0	0	0	0	0
CYCSP42	0	0	0	0	0	0	0	0
CYCSP43	0	0	0	0	0	0	0	0
CYCSP44	0	0	0	0	0	0	0	0
CYCSP45	0	0	0	0	0	0	0	0
CYCSP46	0	0	0	0	0	0	0	0
CYCSP48	0	0	0	0	0	0	0	0
CYCSP49	0	0	0	0	0	0	0	0
CYCSP5	0	0	0	0	0	0	0	0
CYCSP51	0	0	0	0	0	0	0	0
CYCSP52	0	0	0	0	0	0	0	0
CYCSP53	0	0	0	0	0	0	0	0
CYCSP55	0	0	0	0	0	0	0	0
CYCSP56	0	0	0	0	0	0	0	0
CYCSP6	0	0	0	0	0	0	0	0
CYCSP7	0	0	0	0	0	0	0	0
CYCSP8	0	0	0	0	0	0	0	0
CYCTP	0	0	0	0	0	0	0	0
CYFIP1	95.592287	114.377861	83.110499	145.745811	39.824258	136.171786	66.033088	100.280151
CYFIP2	1.914094	4.195857	6.583003	0.796661	0.252674	3.468319	3.850387	4.227138
CYGB	0	6.936498	3.164188	2.194678	0	1.94442	0	1.380031
CYLC1	0	0	0	0	0	0	0	0
CYLC2	0	0	0	0	0	0	0	0
CYLD	8.632446	30.057868	17.068659	21.004701	10.593161	32.470242	13.949678	54.505581
CYMP	0	0	0	0	0	0	0	0
CYP11A1	4.013148	1.529484	0	1.494424	0	0.085805	0	0

CYP11B1	0	0	0	0	0	0	0	0
CYP11B2	0	0	0	0	0	0	0	0
CYP17A1	0	0	0	0.057669	0	0	0	0
CYP19A1	0	0	0	0	0	0	0	0
CYP1A1	0	0.555524	0	1.46742	25.61379	0.542006	0	0.813615
CYP1A2	0	0	0	0	0	0	0	0
CYP1B1	11.974587	8.004286	7.312766	9.040182	4.465686	18.40273	0	5.340872
CYP1D1P	0	0	0	0	0	0	0	0
CYP20A1	11.036449	8.196695	0	6.426983	18.027121	5.049837	13.561771	6.7538
CYP21A1P	0	0	0	0	0	0	0	0
CYP21A2	0	0	0	0	0	0	0	0
CYP24A1	98.500086	133.963817	103.011278	134.123316	20.884433	22.535696	0.605698	22.040137
CYP26A1	2.477155	0.389695	0	0.237692	0	0.15164	0	0.881271
CYP26B1	5.743627	32.229963	13.991676	22.61502	13.345682	21.166868	12.579665	20.25345
CYP26C1	0	0	0	0.076502	0	0	0	0
CYP27A1	4.381322	2.894104	0	4.521063	0	3.786668	0	2.020893
CYP27B1	8.256907	5.324788	12.88105	10.589621	5.445637	7.309103	0	3.6725
CYP27C1	4.411593	0.151555	0	0.669879	2.327239	0.668894	0.18195	0.654424
CYP2A6	0	0	0	0	0	0	0	0
CYP2A7	0	0	0	0	0	0	0	0
CYP2A7P1	0	0	0	0	0	0	0	0
CYP2A7P2	0	0	0	0	0	0	0	0
CYP2AB1P	0	0	0	0	0	0	0	0
CYP2AC1P	0	0	0	0	0	0	0	0
CYP2B6	2.53697	0.850066	0	0.425746	0	0	0	0.61672
CYP2B7P	0	0	0	0	0	0	0	0
CYP2C18	0	0	0	0	0	0	0	0
CYP2C19	0	0	0	0	0	0	0	0
CYP2C23P	0	0	0	0	0	0	0	0
CYP2C56P	0	0	0	0	0	0	0	0
CYP2C58P	0	0	0	0	0	0	0	0
CYP2C59P	0	0	0	0	0	0	0	0
CYP2C60P	0	0	0	0	0	0	0	0

CYP2C61P	0	0	0	0	0	0	0	0
CYP2C8	0	0	0	0	0	0	0	0
CYP2C9	0	0	0	0	0	0.102645	0	0
CYP2D6	0	0.19355	0	0	0	0	0	0
CYP2D7	0	0	0	0.28735	0	0	0	0.037836
CYP2D8P	0	0	0	0	0	0	0	0.097689
CYP2E1	5.746851	3.906432	8.614455	3.866942	0	2.253925	0	0.877357
CYP2F1	0	0	0	0	0	0	0	0
CYP2F2P	0	0	0	0	0	0	0	0
CYP2G1P	0	0	0	0	0	0	0	0
CYP2G2P	0	0	0	0	0	0	0	0
CYP2J2	6.374411	2.354737	7.571791	3.744716	0	4.225208	12.319345	5.165948
CYP2R1	20.982862	37.385451	30.707396	36.697036	1.193783	2.187283	0	2.46354
CYP2S1	28.554854	9.060258	18.587806	14.637137	32.517416	8.065824	17.32577	6.8977
CYP2T1P	0	0	0	0.11962	0	0.242747	0	0
CYP2T3P	0	0	0	0	0	0	0	0
CYP2U1	4.812751	3.021303	0.617521	2.116614	3.394227	2.340118	6.249721	2.20732
CYP2W1	0	0.125187	0	0.32265	0	0.511942	0	0.593915
CYP39A1	0	0.064153	0	0.375213	0	0.374811	0	1.068672
CYP3A137P	0	0	0	0	0	0	0	0
CYP3A4	0	0	0	0	0	0	0	0
CYP3A43	0	0	0	0	0	0	0	0
CYP3A5	0.73482	0.162242	0	0.131297	0	0.065001	0	0
CYP3A51P	0	0	0	0	0	0	0	4.415948
CYP3A52P	0	0	0	0	0	0	0	0
CYP3A54P	0	0	0	0	0	0	0	0
CYP3A7	0	0	0	0	0	0	0	0.067071
CYP3A7-CYP	0	0	0	0	0.582861	0.217806	0	0
CYP46A1	0	0	0	0.035997	0	0	0	0.019666
CYP46A4P	0	0	0	0	0	0	0	0
CYP4A11	0	0	0	0	0	0	0	0
CYP4A22	0	0	0	0	0	0	0	0
CYP4A26P	0	0	0	0	0	0	0	0

CYP4A27P	0	0	0	0	0	0	0	0
CYP4A43P	0	0	0	0	0	0	0	0
CYP4A44P	0	0	0	0	0	0	0	0
CYP4B1	0	0	0	0	0	0.509874	0	0
CYP4F10P	0	0	0	0	0	0	0	0
CYP4F11	3.510734	3.383317	10.109114	5.404205	8.029838	5.729985	0	3.092601
CYP4F12	0	0	0	0	0	0.723186	0	0.338996
CYP4F2	0	0.425299	0	0.081316	0	0	0	0.404046
CYP4F22	0	0	0	0.033916	0	0	0	0
CYP4F23P	0	0	0	0	0	0	0	0
CYP4F24P	0	0	0	0	0	0	0	0
CYP4F25P	0	0	0	0	0	0	0	0
CYP4F27P	0	0	0	0	0	0	0	0
CYP4F29P	0	0	0	0	0	0	0	0
CYP4F3	5.050099	0.529231	7.217924	3.123248	3.077883	0.973626	0	2.2043
CYP4F30P	0	0	0	0	0	0	0	0
CYP4F32P	0	0	0	0	0	0	0	0
CYP4F33P	24.202378	0	0	0	0	0	0	0
CYP4F34P	0	0	0	0	0	0	0	0
CYP4F35P	0	0	0	0.414152	0	0.058282	0	0
CYP4F36P	0	0	0	0	0	0	0	0
CYP4F44P	0	0	0	0	0	0	0	0
CYP4F59P	0	0	0	0	0	0	0	0
CYP4F60P	0	0	0	0	0	0	0	0
CYP4F62P	0	0	0	0	0	0	0	0
CYP4F8	0	0	0	0	0	0	0	0
CYP4F9P	0	0	0	0	0	0	0	0
CYP4V2	4.575453	1.329906	1.888168	1.096421	0.372221	0.931738	0	1.329095
CYP4X1	0	0	1.359584	0.147927	0	0	0	0
CYP4Z1	0	0	0	0	0	0	0	0
CYP4Z2P	0	0	0	0	0	0	0	0
CYP51A1	145.323908	110.41026	104.578987	58.341269	89.320663	33.903324	105.178988	72.702681
CYP51A1P1	0	0	0	0	0	0	0	0

CYP51A1P2	0	0	0	0	0	0	0	0
CYP51A1P3	0	0	0	0	0	0	0	0
CYP7A1	0	0	0	0	0	0	0	0
CYP7B1	0.433054	1.374289	0.389518	0.127157	1.878544	0.173901	27.395999	2.176259
CYP8B1	0	0	0	0	0	0	0	0
CYREN	0	87.270999	34.671582	124.349438	44.491914	174.639382	47.413779	160.259468
CYRIA	0	0	0	0.25335	0.185572	0.140637	0	0.212505
CYRIB	116.857058	162.522528	81.99337	130.663185	140.61286	251.865677	127.345545	407.861917
CYS1	0	0	0	0	0	0	0	0
CYSLTR1	1.105788	0.047185	0	0	0	0	0	0.150863
CYSLTR2	0	0	0	0	0	0	0	0
CYSTM1	85.860825	47.456251	77.921896	60.155158	32.776276	32.064473	54.71998	28.04041
CYTH1	19.997063	9.229219	0	6.650117	4.719118	9.04395	57.712854	4.775999
CYTH1P1	0	0	0	0	0	0	0	0
CYTH2	18.969459	21.297243	29.064862	24.780169	16.419955	37.452636	1.93316	33.812941
CYTH3	91.660238	183.972767	81.821955	202.80923	98.736714	164.326666	53.217013	81.950347
CYTH4	0	0.425842	6.076953	1.918171	0	0.778132	0	0
CYTIP	0	0	0	0	0	0	0	0
CYTL1	0	0	0	0	0	0	0	0
CYYR1	0	3.07384	2.933605	3.433145	0	0.271875	0	0
CZIB	51.964886	53.487765	62.995302	60.886626	15.333322	19.538696	37.796689	23.94635
D2HGDH	10.89881	16.973732	8.34922	11.432059	5.179528	6.179584	53.115043	8.581719
DAAM1	28.738273	40.216694	12.70862	17.997991	81.472623	42.492335	86.2828	128.098924
DAAM2	0	0	0	0	0	0.499899	0	0
DAB1	0	0	0	0.031929	0	0	0	0
DAB2	20.746161	47.17787	11.56035	43.887034	40.250271	12.133158	4.240929	9.431791
DAB2IP	0	7.618952	12.852385	13.465125	113.017277	41.423924	56.009789	18.043686
DACH1	0	0	0	0	0	0	0	0
DACH2	0	0	0	0	0	0	0	0
DACT1	3.894152	0	0	1.199159	0.404432	1.115572	0	0
DACT2	0	0	0	0	0	0	0	0
DACT3	0	0	1.204065	0.16376	0	0.334301	0	0.322099
DAD1	157.440267	162.395928	221.374442	128.983986	162.941445	74.418481	425.565966	170.026928

DAG1	39.386713	0	0	0	0	6.596711	0	0
DAGLA	0	0.785856	0	0.824502	0.741504	3.066692	0	1.980973
DAGLB	9.172511	10.135949	13.820225	13.66561	10.229828	11.422863	0	9.685049
DALRD3	13.727911	15.112439	13.838272	14.384929	35.66326	9.346587	10.699881	9.316518
DAND5	0	0	0	0	0	0	0	0
DAO	0	0	0	0	0	0	0	0
DAOA	0	0	0	0	0	0	0	0
DAP	61.984967	20.255254	4.935622	2.079593	24.544008	26.084745	53.676214	29.196993
DAP3	149.981328	141.926616	141.362828	163.2583	285.792201	102.314286	78.116136	103.782691
DAP3P1	0	0	0	0	0	0	0	0
DAP3P2	0	0	0	0	0	0.878161	0	0.40457
DAPK1	1.51886	2.102034	0	0.932577	32.602626	153.980857	30.120919	118.949685
DAPK2	0	0	7.600812	0.220945	0	0.394493	0	2.55871
DAPK3	31.168301	55.699014	27.843357	65.217134	14.843424	107.963054	30.200209	87.88715
DAPL1	0	1.355744	0	0	0	0	0	0
DAPP1	16.038062	10.406642	19.498609	13.149033	12.093734	4.278599	3.61753	6.313154
DARS1	62.705763	184.506147	103.799747	162.621544	58.500785	160.326397	273.261954	203.368022
DARS2	90.126802	170.169165	78.440174	148.780911	27.715285	75.640793	28.331378	158.240834
DAW1	4.20426	4.015603	4.92049	3.538301	0	0.207163	0.507893	0.679819
DAXX	58.033712	83.375686	36.718311	88.320773	63.032522	148.199799	101.097439	94.224137
DAZ1	0	0	0	0	0	0	0	0
DAZ2	0	0	0	0	0	0	0	0
DAZ3	0	0	0	0	0	0	0	0
DAZ4	0	0	0	0.092779	0	0	0	0
DAZAP1	40.041936	106.041361	46.693899	91.116848	111.526166	166.210671	85.878833	114.758027
DAZAP2	141.795042	100.680442	150.063278	118.604395	115.120768	69.089124	20.703648	60.434184
DAZAP2P1	0	0	0	0	0	0	0	0
DAZL	0	0	0	0	0	0	0	0
DBF4	64.872192	69.927804	45.495149	35.010366	55.313405	44.556624	11.331089	90.395618
DBF4B	15.48737	24.503932	13.826673	23.278303	3.536095	41.549476	0	31.63125
DBF4P1	0	0.303012	0	0.094342	0	0	0	0.776174
DBF4P2	0	0	0	0	0	0	0	0
DBF4P3	0	0	0	0	0	0	0	0

DBH	0	0	0	0	0	0	0	0
DBI	15.928459	21.983122	0	9.998784	143.523736	43.784288	0	7.098504
DBIL5P	0	0	0	0	0	0	0	0
DBIL5P2	0	0	0	0	0	0	0	0
DBIP1	0	0	0	0	0	0	0	0
DBIP2	0	0	0	0	0	0	0	0
DBN1	22.801897	32.369107	29.129926	50.790704	67.943834	100.833116	98.875948	45.797493
DBNDD1	2.033445	1.296342	0	0.852801	0	0.234227	0	0.803372
DBNDD2	29.581674	42.824748	65.413612	42.169899	33.336205	34.652064	0	35.526494
DBNL	60.551556	48.899391	58.736544	65.578255	165.470884	150.145888	294.29678	81.668926
DBP	2.414534	6.137548	19.669981	8.326227	13.861545	12.340314	0	15.161837
DBR1	0	0	0	0	0	2.78479	0	0
DBT	20.462624	17.701162	12.027536	14.518054	10.988259	4.134566	7.899375	17.248694
DBTP1	0	1.872952	0	0	0	0	0	2.138656
DBX2	0	0	0	0	0	0	0	0
DCAF1	12.200539	12.678085	9.297299	12.558477	0.564968	9.639734	2.399704	12.88764
DCAF10	16.734029	19.713176	26.969047	6.518784	16.847109	11.595468	73.583843	18.438201
DCAF11	17.6791	90.720135	15.617329	108.446703	77.895429	166.718904	56.396446	117.132374
DCAF12	36.990238	29.930725	31.519651	28.108895	75.680463	41.960441	49.255683	39.869946
DCAF12L1	0	0	0	0	0	0	0	0
DCAF12L2	0	0	0	0	0	0	0	0
DCAF13	47.638963	115.70031	69.79013	95.065347	39.81067	44.524638	69.153197	67.644719
DCAF13P1	0	0	0	0	0	0	0	0
DCAF13P2	0	0	0	0	0	0	0	0
DCAF13P3	0	0.114457	0	0	0	0	0	0
DCAF15	13.698896	0	11.592025	0	0	2.939742	12.404643	0.662367
DCAF16	27.449622	117.221769	36.203844	124.60979	41.348298	108.806569	17.940466	120.231827
DCAF17	21.115649	16.741999	19.517791	11.177186	16.847292	9.941885	1.694491	35.992435
DCAF4	8.689403	15.496992	23.810392	25.253004	40.370003	24.784031	36.038318	17.572695
DCAF4L1	0	0	0	0.017013	0.183934	0	0	0
DCAF4L2	0	0	0	0	0	0	0	0
DCAF5	27.525005	62.708044	25.23469	56.065624	26.636069	81.113219	6.813261	67.624655
DCAF6	27.987415	56.68088	58.781314	52.496798	40.891656	35.752194	75.741449	48.575834

DCAF7	63.650664	39.377344	60.45602	38.028267	72.12479	23.702928	44.965081	25.448267
DCAF8	85.720024	65.892934	17.51817	75.975188	13.462913	83.510811	51.16634	58.053651
DCAF8L2	0	0	0	0	0	0	0	0
DCAKD	0	0	0	0.379857	0	0	0	0
DCANP1	0	0	0	0.026036	0	0	0	0
DCBLD1	0	18.568511	2.864615	10.176945	0	14.303985	0	10.11683
DCBLD2	424.816861	412.112579	233.082065	533.578652	205.751802	206.109806	254.147608	251.694546
DCC	0	0	0	0	0	0	0	0
DCD	0	0	0	0	0	0	0	0
DCDC1	2.070372	0.61068	2.017943	0.24856	0	0	0	0.08368
DCDC2	0	0.179051	0	0.186936	0	0.069626	0	2.504239
DCDC2B	0	0	0	0	0	0	0	0
DCDC2C	0	0	0	0	0	0	0	0
DCHS1	0	0.180743	0	0.095295	0	0.195632	0	0.011989
DCHS2	0	0.553109	0	0.341785	0	0.129365	0	0.07007
DCK	28.084679	19.281838	18.87748	8.512871	8.951318	8.968158	19.756936	23.343643
DCLK1	0	0.122118	0	0.034776	0	0	0	0
DCLK2	0	0	0	0	0	0	0	0.240283
DCLK3	0	0	0	0	0	0	0	0
DCLRE1A	18.64409	51.597341	16.039601	33.744774	9.901079	14.837449	0.189094	35.343763
DCLRE1B	14.120797	52.638666	19.727286	48.907145	14.448373	42.164147	9.448648	44.41106
DCLRE1C	7.97995	4.984027	4.178702	6.278894	0	3.19029	16.768736	3.012697
DCLRE1CP1	0	0	0	0	0	0	0	0
DCN	0	0	0	0	0.661485	0	0	0
DCP1A	32.684252	10.927135	15.294849	16.813509	10.849806	10.487207	45.084114	7.75792
DCP1B	9.617944	6.782786	6.087766	9.955136	0	1.424973	0	6.037339
DCP2	28.04765	23.636358	18.568828	14.752804	12.384437	18.380219	18.118532	29.731403
DCPS	0.601125	14.67048	10.089104	15.115658	37.538145	28.63555	31.9356	27.394766
DCST1	0	0	0	0	0	0	0	0
DCST2	0	0.140309	1.261239	0.137228	0	0	0	0.056796
DCSTAMP	0	0	0	0	0	0	0	0
DCT	0	0	0	0	0	0	0	0
DCTD	113.089223	112.134243	61.846387	106.258865	69.892633	101.151206	161.470283	113.645578

DCTN1	50.412802	90.190577	64.97101	105.459192	39.220566	123.045709	108.682918	93.271562
DCTN2	209.68974	195.987399	181.066004	187.063157	153.907116	113.53357	542.767124	105.229666
DCTN3	48.910574	29.287195	77.220858	26.903162	41.019686	36.362302	25.343226	39.824586
DCTN4	0	9.080894	6.346972	14.732992	25.002701	16.229203	88.636163	1.049777
DCTN5	62.805692	119.210616	40.999548	121.062884	97.183555	108.071483	71.301063	130.562504
DCTN6	47.076126	24.591146	41.588078	25.179582	73.405201	19.060565	58.88851	26.212202
DCTPP1	0	0	0	1.233232	0	0	41.431918	1.481558
DCUN1D1	84.733992	61.476705	89.873963	60.792291	88.906965	44.362722	61.314326	57.852388
DCUN1D2	13.695425	30.822222	7.582915	27.717442	5.024848	17.803915	0	28.47082
DCUN1D3	7.413339	18.089517	5.712339	21.654744	8.394774	19.812534	14.607013	26.963635
DCUN1D4	37.177427	33.949913	9.142132	27.389065	11.187522	12.89506	38.130461	26.412115
DCUN1D5	693.751545	1677.50772	701.721213	1147.45158	115.673595	273.254607	122.550647	331.182597
DCX	0	0	0	0	0	0	0	0
DCXR	26.576141	27.35754	36.992516	33.937575	78.851682	51.836273	40.770363	27.093672
DDA1	78.582754	83.701727	37.076407	103.248219	45.38022	212.614433	68.663266	175.154064
DDAH1	66.305359	79.749628	49.710373	44.783688	18.174321	29.399061	0	24.745887
DDAH2	4.190286	7.047555	5.532543	8.295342	4.105573	13.177815	1.380235	14.260781
DDB1	0	23.771928	32.607748	53.179558	53.665879	89.048405	90.664093	86.509337
DDB2	47.635197	4.303415	41.153618	9.327621	59.83478	24.090178	0	12.133952
DDC	0	0	0	0.426124	0	0	0	0
DDHD1	4.537151	7.584483	16.297939	4.963967	0	5.775534	32.884478	4.618146
DDHD2	17.30907	141.38245	53.699401	130.247611	56.360696	100.091223	3.353332	82.107964
DDI2	20.790035	18.705822	15.967654	16.575731	40.75675	27.94462	38.419443	22.025688
DDIAS	22.772687	61.82712	49.315813	48.666737	20.649763	37.661768	12.627775	89.990175
DDIT3	7.161506	11.990033	31.771574	32.330459	1.200661	7.631615	0	0.805067
DDIT4	191.895491	247.721024	363.721875	531.885527	230.996444	291.509596	184.563398	305.483047
DDIT4L	0	0.387975	1.165549	0	0	0	0	0
DDN	1.735936	3.60386	0.778779	3.622609	0.916776	5.593094	0	3.521334
DDO	0	0	0	0	0	0	0	0
DDOST	0	0	0	0	29.172736	0	0	0
DDR1	59.360652	123.430316	107.082117	163.738527	139.746769	323.910197	234.243522	197.770567
DDR2	0	0.288208	0	0.32727	0	0.055787	0	0.090088
DDRGK1	36.604521	36.546107	27.486159	43.63402	22.225368	49.928573	44.721498	26.847772

DDT	90.498182	47.281023	18.281706	59.094184	102.985214	130.723248	102.171596	133.26209
DDTL	0	2.153492	0	2.130302	9.165037	2.348971	0	1.684198
DDTP1	0	0	0	0	0	0	0	0
DDX1	135.646718	135.982433	83.359694	94.460577	108.079018	59.286629	95.520637	109.205192
DDX10	30.133248	25.507442	28.198077	24.039387	32.091941	31.59253	164.164325	28.153781
DDX10P1	0	0	0	0	0	0	0	0
DDX10P2	0	0	0	0	0	0	0	0
DDX11	50.400034	58.546378	45.494662	68.528122	100.540077	60.621201	74.638199	48.905789
DDX11L1	0	0	0	0	0	0	0	0
DDX11L10	0	0	0	0	0	0	0	0
DDX11L16	0	0	0	0	0	0	0	0
DDX11L17	0	0	0	0	0	0	0	0
DDX11L2	0	0	0	0.158263	0	3.013758	0	0.466573
DDX11L5	0	0	0	0	0	0	0	0
DDX11L8	0	0	0	0	0	0	0	0
DDX11L9	0	0	0	0	0	0	0	0
DDX12B	1.092102	1.830136	5.910338	2.907467	0	1.489024	0	1.626783
DDX12P	0	1.121029	0	0.856045	0.699801	0.511037	6.393593	0.402616
DDX17	3.716009	33.604333	41.678602	47.471069	62.835571	57.162076	43.357261	14.248477
DDX18	43.269171	77.290765	48.434624	43.285173	80.504264	54.297024	140.552828	123.606591
DDX18P1	0	0	0	0	0	0	0	0
DDX18P3	0	0	0	0	0	0	0	0
DDX18P4	0	0	0	0	0	0	0	0
DDX18P5	0	0	0	0	0	0	0	0
DDX18P6	0	0	0	0	0	0	0	0
DDX19A	61.223012	83.685587	75.752068	73.556071	39.059449	66.082243	166.785843	45.959298
DDX19B	29.068504	41.783368	39.871787	57.864269	12.461351	26.895693	0.645879	15.795331
DDX20	31.368077	16.771738	25.653504	10.174938	5.602639	5.124553	21.990011	8.451776
DDX20P1	0	0	0	0	0	0	0	0
DDX21	142.809488	90.500096	168.058154	85.85174	130.433815	71.803903	172.853907	95.940641
DDX23	36.547459	67.685326	24.830466	89.857747	154.461581	91.156043	139.288805	86.300243
DDX24	73.835766	198.25311	82.923836	230.00738	231.601318	309.145386	115.473586	217.015882
DDX25	0	0.970255	0	0	0	0	0	0.479845

DDX27	0	1.122659	0.868209	2.093867	0	3.182922	75.764583	1.474661
DDX31	0	2.240549	0	0	0.616004	6.777602	4.042103	0
DDX39A	4.448261	23.295018	23.372309	17.533487	31.840786	66.577005	69.689217	68.042856
DDX39AP1	0	0	0	0	0	0	0	0
DDX39B	140.646043	268.58284	138.297962	226.803524	357.693425	230.086893	290.099888	308.84794
DDX39BP1	0	0	0	0	0	0	0	0
DDX39BP2	0	0	0	0	0	0	0	0
DDX3P1	0	0	0	0	0	0	0	0
DDX3P2	0	0	0	0	0	0	0	0
DDX3P3	0	0	0	0	0	0	0	0
DDX3X	133.661395	212.926771	106.739185	299.214587	243.472629	265.275322	230.045152	282.80494
DDX3Y	50.760964	32.331722	26.986213	44.88962	85.500577	49.190715	74.808877	68.960499
DDX4	0	0	0	0.253091	0	0	0	0
DDX41	44.338933	86.081682	240.221111	34.277836	29.939312	104.77778	36.273004	28.135601
DDX42	57.595936	165.059247	55.739527	179.65421	51.031197	145.647137	50.675705	153.377042
DDX43	0	0	0	0	0	0	0	0
DDX43P1	0	0	0	0	0	0	0	0
DDX43P2	0	0	0	0	0	0	0	0
DDX43P3	0	0	0	0	0	0	0	0
DDX46	90.406119	79.280277	50.495218	89.939907	59.061269	71.804704	112.474784	81.08932
DDX47	86.835462	89.583079	80.153351	93.160447	87.421654	94.50566	224.866391	126.162367
DDX49	19.055378	14.132556	35.091864	9.248353	79.265465	38.292537	80.932859	22.428627
DDX5	193.446561	293.199086	209.66606	271.870859	185.753701	219.145268	357.015317	337.774126
DDX50	69.365997	97.682424	77.596978	71.602344	37.929787	43.448972	132.376419	59.028917
DDX50P1	0	0.121712	0	0	0	0	0	0
DDX50P2	0	0	0	0	0	0	0	0
DDX51	9.609511	5.969387	9.499188	2.880866	3.187938	4.433856	2.472695	2.267996
DDX52	46.590512	18.286391	18.473797	22.566	56.095791	16.695411	34.577338	22.892428
DDX54	27.800373	59.106262	47.620992	56.747002	184.542968	150.607764	167.106402	68.177652
DDX55	15.298189	24.361974	15.976975	30.666603	22.066933	29.558974	15.162956	30.060702
DDX55P1	0	0	0	0	0	0	0	0
DDX56	18.880642	18.010541	27.402975	14.546601	100.42519	45.249109	59.482727	43.490619
DDX59	10.786912	20.007388	26.771757	17.795549	0.812808	13.763804	25.82865	14.834339

DDX5P1	0	0	0	0	0	0	0	0
DDX6	481.108853	614.280101	461.388108	565.004266	121.327482	136.727477	112.898552	269.428916
DDX60	5.945628	6.24452	7.33749	3.512002	48.686129	8.1568	69.090142	16.08279
DDX60L	0	10.628514	0	22.623506	50.298259	46.371939	4.307038	60.911279
DDX6P1	0	0	0	0	0	0	0	0
DDX6P2	0	0	0	0	0	0	0	0
DEAF1	0	11.590411	0	5.369083	2.137724	14.349317	1.466919	15.512363
DECR1	73.16753	46.025792	78.198137	42.634332	70.819984	16.34823	90.71314	16.743042
DECR2	4.610111	7.776224	10.257222	4.987425	1.533982	9.23073	10.863031	6.559618
DEDD	32.097109	53.730552	50.752599	46.342597	21.211619	31.836036	17.331109	30.674343
DEDD2	14.129337	59.060115	9.935002	83.54623	21.684688	167.777548	0	122.936438
DEF6	1.492111	7.773891	10.672455	12.294816	4.722594	20.207405	0	15.870987
DEF8	58.126576	62.041096	24.870981	51.843026	36.540604	120.949247	16.711163	96.200051
DEFA10P	0	0	0	0	0	0	0	0
DEFA11P	0	0	0	0	0	0	0	0
DEFA3	0	0	0	0	0	0	0	0
DEFA5	0	0	0	0	0	0	0	0
DEFA6	0	0	0	0	0	0	0	0
DEFA7P	0	0	0	0	0	0	0	0
DEFA8P	0	0	0	0	0	0	0	0
DEFA9P	0	0	0	0	0	0	0	0
DEFB1	0	0	0	7.277672	0	0	0	0
DEFB106B	0	0	0	0	0	0	0	0
DEFB107A	0	0	0	0	0	0	0	0
DEFB108A	0	0	0	0	0	0	0	0
DEFB108B	0	0	0	0	0	0	0	0
DEFB108C	0	0	0	0	0	0	0	0
DEFB108D	0	0	0	0	0	0	0	0
DEFB108E	0	0	0	0	0	0	0	0
DEFB108F	0	0	0	0	0	0	0	0
DEFB109F	0	0	0	0	0	0	0	0
DEFB110	0	0	0	0	0	0	0	0
DEFB113	0	0	0	0	0	0	0	0

DEFB115	0	0	0	0	0	0	0	0
DEFB116	0	0	0	0	0	0	0	0
DEFB117	0	0	0	0	0	0	0	0
DEFB119	0	0	0	0	0	0	0	0
DEFB121	0	0	0	0	0	0	0	0
DEFB122	0	0	0	0	0	0	0	0
DEFB123	0	0	0	0	0	0	0	0
DEFB124	0	0	0	0	0	0	0	0
DEFB125	0	0	0	0	0	0	0	0
DEFB126	0	0	0	0	0	0	0	0
DEFB129	0	0	0	0	0	0	0	0
DEFB130A	0	0	0	0	0	0	0	0
DEFB130C	0	0	0	0	0	0	0	0
DEFB130D	0	0	0	0	0	0	0	0
DEFB131B	0	0	0	0	0	0	0	0
DEFB131D	0	0	0	0	0	0	0	0
DEFB131E	0	0	0	0	0	0	0	0
DEFB132	0	0	0	0	0	0	0	0
DEFB133	0	0	0	0	0	0	0	0
DEFB136	0	0	0	0	0	0	0	0
DEFB4B	0	0	0	1.315469	0	7.637191	0	3.437401
DEGS1	42.203228	41.788829	60.349456	26.998887	50.705765	17.753314	136.458798	39.937384
DEGS2	0.86345	0.39081	0	0.400428	0	0.603874	0	0.208095
DEK	315.60223	454.127722	240.788213	266.355152	337.558965	444.374183	528.072911	714.300725
DELE1	42.138842	24.879096	52.131911	27.851413	64.621255	16.296028	21.1853	9.174221
DELEC1	0	2.690519	0	1.390002	0.594075	0.44382	11.978949	0.938608
DENND10	56.812145	69.67081	29.505295	58.35653	40.626554	77.387793	78.543601	103.947875
DENND10P1	0	0	13.708617	0	0	1.821927	0	0
DENND11	19.055934	17.487299	16.740622	16.034058	7.02675	10.859043	2.377661	10.931818
DENND1A	25.19391	13.525576	19.045482	16.10898	30.568091	17.041193	0	13.644088
DENND1B	3.921807	13.261885	3.812825	8.975868	13.580118	8.112407	5.733078	13.630151
DENND1C	0	0	0	0.308352	0	0.141517	0	0.056719
DENND2A	1.000488	0.171004	0	0.122003	0	0	0	0.184586

DENND2B	44.21805	52.174845	106.042343	48.127147	105.652456	269.74468	387.666795	199.248375
DENND2C	2.856363	6.674082	4.039756	7.183541	0	3.599351	9.049153	3.482898
DENND2D	25.787341	56.470557	22.271681	47.314098	22.245236	33.723191	0	35.463404
DENND3	0.807046	24.154452	29.033933	14.073309	24.405463	14.595073	73.731891	5.053829
DENND4A	9.535312	19.163658	4.451132	11.64556	32.550157	3.578871	45.570463	11.393028
DENND4B	20.874892	13.753294	11.240783	11.539735	15.607925	22.486679	111.613494	18.80156
DENND4C	16.902307	7.455679	13.21818	2.323851	10.274401	4.33346	9.393149	3.986107
DENND5A	11.145935	18.000938	16.097829	21.915223	11.766841	52.313985	51.909725	46.528049
DENND5B	1.534761	4.560636	5.625325	2.660017	1.790486	0.435026	0	4.890592
DENND6A	14.832987	49.137439	58.036849	12.365661	21.860797	9.721234	3.003513	36.705709
DENND6B	1.390724	3.291883	1.804341	2.656851	0	2.309444	0.163082	0.337442
DENR	84.53566	104.341763	61.218049	74.475478	73.703064	54.444065	104.975351	79.936113
DENRP1	0	0	0	0	0	0	0	0
DENRP2	0	0	0	0	0	0	0	0
DENRP3	0	0	0	0	0	0	0	0
DENRP4	0	0	0	0	0	0	0	0
DEPDC1	59.485493	79.257745	39.008815	15.843925	73.324708	15.450354	25.452921	44.554015
DEPDC1B	68.336784	101.243654	43.509782	61.923367	47.552075	109.033404	43.646277	166.984426
DEPDC1P1	0	0	0	0	0	0	0	0
DEPDC1P2	0	0.360725	0	0	0	0	0	0
DEPDC4	0	0.91863	0	1.280591	0.853043	0.473694	0	0.453791
DEPDC5	11.194078	24.409392	18.137428	20.386651	5.262189	9.639233	0	8.826048
DEPDC7	4.047687	2.219706	1.804032	0.539798	11.731969	6.188217	34.732691	11.612711
DEPP1	0	0	0	0	0	0	0	0
DEPTOR	0	0	0	0.546825	5.469831	0.459709	0	0.425599
DERA	85.580663	245.60624	93.866865	172.273542	126.877538	68.08295	73.804439	113.920889
DERL1	38.959379	56.293865	55.471019	57.11705	58.702089	41.482231	116.528442	63.05303
DERL2	32.349312	41.466234	72.112673	34.098222	41.917864	23.503049	86.257179	38.773398
DERL3	0	0	0	0	0	0	0	0
DERPC	0	54.98891	0	33.77071	53.995347	36.400357	0.910964	51.572158
DES	0	1.208887	0	0	0	0	0	0
DESI1	20.011586	33.825834	20.444337	22.105094	8.331155	25.58002	23.453402	25.679971
DESI2	83.750198	81.66249	51.684693	49.791676	33.415455	45.422449	30.213892	85.534501

DET1	0	2.637431	0	6.759293	0	0.95464	0	1.245106
DEUP1	0	0	0	0	0	0	0	0.086553
DEXI	9.100427	46.928521	31.087056	47.829661	31.416873	85.016745	36.925107	67.02639
DFFA	86.278964	111.014803	86.407352	105.472122	38.912527	69.54617	42.680248	55.917628
DFFB	0	1.008147	3.323547	1.280078	17.419827	1.542607	0	2.875987
DFFBP1	0	0.977862	0	0.665109	0	0.57403	0	0
DGAT1	2.735102	7.331449	10.558784	8.06191	1.930831	6.823849	0	8.254306
DGAT2	16.63251	13.413798	2.633501	12.945586	0	2.892509	0	4.296146
DGAT2L7P	0	0	0	0	0	0	0	0
DGCR2	32.116547	35.046531	11.308709	40.933742	9.392532	94.289184	31.042765	85.486396
DGCR5	0.593765	2.134	1.60075	1.659108	1.254944	3.116271	0	2.488333
DGCR6	0	0	7.916695	1.823044	0	4.726794	0	2.473035
DGCR6L	4.194736	1.341781	18.469014	0	0	1.517538	0	0
DGCR8	14.584208	27.238246	2.0919	30.102673	20.223368	44.192989	0	43.819056
DGKA	45.099984	66.923822	35.400444	69.608679	22.191003	57.140375	87.840704	39.637202
DGKB	0	0	0	0.075268	0	0	0	0
DGKD	36.979433	2.781033	26.759924	5.668795	41.103318	0	48.689484	9.490319
DGKE	1.496577	4.391859	3.366436	2.547534	8.621635	1.962474	1.916872	3.214564
DGKG	14.744318	16.673159	3.025419	19.431989	1.930907	18.574668	0	17.773416
DGKH	10.735341	26.223055	29.287685	18.740359	33.830416	6.004073	13.38531	12.357968
DGKI	0	1.364373	0.222692	0.535708	0	0.01244	0	1.398772
DGKK	0	0	0	0	0	0	0	0
DGKQ	0.754341	2.262775	0.677266	1.681157	0	4.792993	0	3.721872
DGKZ	27.898998	41.368299	18.752967	31.558666	89.472086	67.552355	6.190239	59.666493
DGKZP1	0	0	0	0.19479	0	0.299493	0	0.06585
DGLUCY	4.016229	40.019363	25.658516	47.388758	11.302345	33.262956	7.241654	32.310198
DGUOK	126.014464	80.819693	87.734377	107.522691	86.381883	62.183445	19.638714	70.739662
DHCR24	372.47524	502.892098	285.088205	506.784796	121.697205	173.676712	87.722627	112.920316
DHCR7	0	2.191295	37.001143	87.101467	211.56981	70.515524	55.376459	7.144056
DHDDS	23.282161	30.503292	26.438529	30.860117	31.92265	37.838702	44.963154	27.825671
DHDH	6.994287	5.195086	0	4.05149	0	2.783784	0	4.005601
DHFR	68.813279	55.809315	29.226781	39.164252	76.250308	32.132611	71.364922	72.763036
DHFR2	0	1.768828	0	0.206514	0	2.316017	12.715803	1.531948

DHFRP1	61.535177	125.976952	76.595767	43.545774	0	27.022158	30.182397	74.921358
DHFRP2	0	0	0	0	0	0	0	0
DHFRP3	0	0	0	0	0	0	0	0
DHFRP5	0	0	0	0	0	0	0	0
DHFRP6	0	0	0	0	0	0	0	0
DHH	0.700524	0	0	0.102688	0	0.070117	0	0.056229
DHODH	8.609364	6.295547	5.048769	15.55871	6.361374	16.194123	0	7.272491
DHPS	34.444846	42.702813	63.941435	68.932908	86.19632	115.662776	135.249724	70.824108
DHRS1	18.258148	9.389262	18.388125	7.714469	0	7.890215	0	3.90271
DHRS11	13.519749	16.897006	13.80039	14.118847	3.545806	9.158938	0	4.400184
DHRS12	8.338116	5.762362	7.180469	8.716152	0.846976	6.4569	0	5.322946
DHRS13	7.22341	17.331196	14.505308	20.013814	10.186393	24.944325	23.412274	18.646229
DHRS2	9.691878	2.67088	0	0.24351	0	0.25492	0	0
DHRS3	34.372254	75.579898	59.794734	193.567125	0	35.287925	0	23.73458
DHRS4	36.631947	22.709534	9.175286	22.560876	15.119727	28.201988	0	23.340531
DHRS4L1	0	0.467435	0	0	0	0	0	0
DHRS4L2	4.429303	16.111989	10.406695	13.475371	0	16.678803	0	9.846402
DHRS7	107.332359	206.900383	221.891161	213.880504	88.715686	65.93885	58.455284	94.098703
DHRS7B	35.119009	6.46576	17.423265	10.677209	1.699451	7.520211	0	5.480517
DHRS7C	0	0	0	0	0	0	0	0
DHRS9	58.872499	96.300868	98.983072	96.125787	3.559832	14.987313	0	12.692823
DHRSX	0	5.672283	0	4.722102	11.57951	19.055472	5.946947	11.926492
DHTKD1	12.002815	18.297292	25.540906	22.330409	109.110166	17.171641	37.973427	11.591572
DHX15	136.848894	86.321725	109.911444	62.209486	143.229129	52.526239	129.892318	90.2432
DHX16	25.431476	40.348753	31.947795	54.427093	34.241338	78.82315	23.068768	45.998321
DHX29	20.900496	13.157764	18.32213	8.03349	39.885289	8.308623	18.592309	20.994362
DHX30	22.800187	21.188963	21.420302	20.374247	54.296189	29.569643	91.915688	26.086536
DHX32	13.492808	61.688909	0	65.451007	50.309715	64.760663	0	100.612002
DHX33	25.915711	21.348956	21.172412	27.522061	23.181053	45.883382	22.67646	43.679793
DHX34	2.276097	9.136292	2.043484	11.033257	10.620207	22.959234	38.178792	20.791727
DHX35	19.130724	17.584272	13.657702	14.49282	12.333284	7.388475	11.6584	10.96381
DHX36	104.261246	52.090932	39.701351	32.688947	160.493587	18.887457	123.705434	35.27547
DHX37	11.526037	10.480412	12.937944	11.392002	10.274773	14.886213	7.717886	8.386571

DHX38	28.173585	41.452099	52.874352	65.665236	58.63711	72.777521	67.898239	56.192805
DHX40	54.26735	61.501075	64.360301	38.10115	50.180669	18.918284	54.789949	38.124051
DHX40P1	0	0	0	0	0	0	0	0
DHX57	27.738613	15.939864	16.86193	14.654327	20.011871	10.525291	51.418585	12.510201
DHX58	1.467792	3.33211	5.034385	2.620453	2.840728	5.069468	0	2.829601
DHX8	35.936407	22.931166	20.525636	30.958338	36.834147	36.33793	29.438941	21.794188
DHX9	98.318981	89.193615	106.048031	91.365444	99.296441	100.057039	98.479248	135.076213
DHX9P1	0	0	0	0	0	0	0	0
DIABLO	52.327509	68.605969	34.702741	52.007848	75.914578	32.657732	30.290585	35.300221
DIAPH1	84.466633	149.952797	109.10468	157.024925	136.550138	186.472898	244.784644	164.134959
DIAPH2	1.58473	6.016497	1.942579	5.289347	2.961581	8.041356	2.154089	6.723721
DIAPH3	9.356226	5.014986	0	4.997154	15.463432	2.963731	27.386581	9.001193
DICER1	18.367205	54.417672	11.907603	37.417763	64.851814	42.53961	44.461728	76.926119
DIDO1	23.778662	77.607752	34.876389	84.338116	0	87.512397	0	70.441861
DIMT1	18.199283	40.988899	35.389765	9.428763	3.545106	7.137485	11.565193	20.198059
DIMT1P1	0	0	0	0	0	0	0	0
DIO1	0	0	0	0	0	0	0	0
DIO2	0	0.52847	0	1.893801	0	35.347561	0	16.323193
DIP2A	12.477021	12.149994	11.9286	15.995731	27.419	28.150855	5.281775	24.458567
DIP2B	28.863199	28.924153	39.202867	26.277176	16.423918	16.843958	33.026647	17.129238
DIP2C	0	2.228056	0	3.7783	0	0.472824	0	0.685845
DIP2C-AS1	0	0	0	0	0	0	0	0
DIPK1A	56.275011	65.103595	41.964494	37.699168	18.910463	7.012129	2.203969	11.661297
DIPK1B	5.731664	11.431247	7.725306	13.846687	0	1.582749	0	2.491866
DIPK1C	0	0	0	0	0	0	0	0
DIPK2A	35.213924	18.142548	0	5.383827	5.39439	0	12.645381	9.885971
DIPK2B	0	0	0	0	0	0	0	0
DIRAS1	3.946829	4.849533	3.538883	8.375115	0.260491	0.246118	0	0.118975
DIRAS2	0	0	0	0	0	0	0	0
DIRAS3	0	0	0	0.176293	0	0	0	0
DIRC1	0	0	0	0	0	0	0	0
DIS3	38.263158	32.653712	30.117284	22.987304	41.418903	17.125293	58.582843	28.980243
DIS3L	23.843846	35.406627	9.435848	31.111934	99.695682	31.781263	16.481726	40.42843

DIS3L2	2.489732	24.019235	19.74067	11.459168	0	17.679808	23.8933	10.443596
DIS3L2P1	0	0	0	0	0	0	0	0
DISC1	0	0.244206	0	0.231999	0	0.815795	0	0.43606
DISP1	0.688437	0.206768	1.854981	1.152439	0	0.219014	0	1.243362
DISP2	2.282471	11.410785	22.930648	6.879058	2.296494	15.056749	3.033821	2.800506
DISP3	0	0	1.114672	0.627515	0	0.236079	0	0.20233
DIXDC1	1.358185	3.911115	7.657746	5.909657	6.278393	8.788749	1.984709	13.184069
DKC1	126.882888	161.413174	108.579758	140.741449	120.581536	133.263603	87.571262	152.087125
DKK1	234.857172	204.809369	219.3668	137.579879	180.275818	81.108711	203.843926	189.631129
DKK2	0	0	0	0	0	0	0	0
DKK3	53.029346	69.073471	73.363851	58.541956	89.032327	164.036337	54.018823	65.289724
DKK4	0	0	0	0	0	0	0	0
DKKL1	0	0	0	0	0	1.027458	0	0
DKKL1P1	0	0	0	0	0	0	0	0
DLAT	12.620882	30.225392	46.214919	29.476971	61.152926	25.512279	0	25.0504
DLC1	36.179728	73.782892	21.938998	107.714167	48.918748	70.960291	9.645953	56.001379
DLD	44.573352	61.315388	49.873905	32.999278	61.888435	22.708933	0	60.141405
DLEC1	0	0	0	0	0	0.05937	0	0.047994
DLEC1P1	0	0	0	0	0	0	0	0
DLEU7	0	0	0	0	0	0	0	0
DLG1	145.408095	152.212286	116.641282	111.998536	108.925193	83.851733	91.190997	111.512059
DLG2	0	12.186764	2.346103	6.014799	0	2.199354	1.272403	2.827268
DLG3	0	67.858446	47.602593	99.504777	41.160949	54.507213	30.506545	45.451114
DLG4	1.842806	3.737164	7.75952	2.734728	22.787325	2.600334	0	0
DLG5	42.899515	58.435668	25.090777	46.71584	42.832863	146.584208	30.189596	93.207021
DLGAP1	0	0.293349	0	2.096848	0	0	0.483666	0
DLGAP2	0	0.03154	0	0.017917	0	0	0	0
DLGAP3	0	0	0	0.112982	0	0.924639	0	0.744229
DLGAP4	82.701196	224.873614	75.3384	356.30983	73.734787	325.763168	56.300147	207.358608
DLGAP5	78.904408	57.866428	35.586134	33.07087	73.042554	52.741097	228.321956	96.665973
DLGAP5P1	0	0	0	0	0	0	0	0
DLGAP5P2	0	0	0	0	0	0	0	0
DLK1	0	0	0	0	0	0	0	0

DLK2	11.550619	25.813268	4.553632	24.8027	24.679252	41.543447	10.38715	26.131758
DLL1	13.917539	38.683521	17.25473	67.183299	11.836974	66.083141	31.436356	46.375932
DLL3	0	0	0	0.332193	5.41762	0.152778	0	0.330626
DLL4	0	0.249321	0	0.507796	0	0.145508	0	0.352318
DLST	89.574715	73.45478	80.515108	107.612313	112.218551	100.44188	98.474196	74.988054
DLSTP1	0	0	0	0	0	0.132209	0	0.109382
DLX1	0	9.785274	8.243083	8.661759	0.854026	18.764372	0	8.844185
DLX2	0	1.534526	2.483897	1.824247	3.296091	4.895305	0	7.030584
DLX3	9.844392	6.609352	5.832531	6.531048	2.750991	1.814107	0	0.996913
DLX4	0	3.561294	1.534266	6.223765	0	4.042819	20.07831	1.941663
DLX5	0	0.855394	6.341065	0.123439	0	0.751181	0	0.310385
DLX6	2.978583	1.264625	0	0.713988	2.356847	0.147764	0	0.060014
DMAC1	137.128241	113.804125	35.332523	62.198299	59.045723	42.948444	97.111588	57.424638
DMAC1P1	0	0	0	0	0	0	0	0
DMAC2	12.678773	22.863604	22.65781	29.33741	69.140064	31.151311	86.085453	21.910975
DMAC2L	0	6.441707	0	0.35208	0	0	0	3.43361
DMAP1	25.862192	10.938473	12.299788	10.083423	108.20862	20.478234	36.210385	18.900184
DMBT1	0	0.055491	0	0	0	1.336213	0	0
DMBT1L1	0	0	0	0	0	0	0	0
DMC1	0	3.21725	0	0.405784	0	0	0	0
DMD	4.238946	5.154383	6.979252	6.585967	2.036985	1.685933	0	1.340495
DMGDH	0	0	14.693338	1.405616	0	0.907041	0	1.79523
DMKN	197.592925	371.080049	193.348775	459.448605	250.140161	450.133401	458.338254	436.079739
DMPK	0	2.225991	8.525982	1.626386	2.87306	4.468564	0	1.565924
DMRT1	0	0.329286	0	0	0.409197	0	0	0
DMRT2	5.944795	8.603909	0	4.96411	0.658351	0.738118	0	1.400418
DMRT3	0	0.87062	0	0.564678	2.598074	0.818975	0	0.164889
DMRTA1	1.7503	1.679537	0.52433	1.012716	0.154142	0.906341	2.132572	1.661489
DMRTA2	0	0	0	0	0	0	0	0
DMRTB1	0	0	0	0	0	0	0	0
DMRTC1	0	0	0	0.227619	0	0	0	0
DMRTC1B	0	0	0	0	0	0	0	0.448942
DMRTC2	0	0	0	0	0	0	0	0

DMTF1	10.30535	24.026278	16.922486	50.71979	122.807501	42.058692	74.581717	39.112529
DMTN	6.145921	9.296834	9.613666	11.903939	7.175391	23.132835	14.698619	17.878796
DMWD	11.154072	18.578162	25.766724	16.513172	12.354378	30.86372	48.924376	22.609495
DMXL1	26.007887	22.734953	15.243484	8.468911	21.10817	12.061368	81.494487	21.589239
DMXL2	8.021461	12.117621	21.128963	8.788294	6.931846	2.354932	0.207562	10.501951
DNA2	45.562571	12.685652	9.00839	9.189934	5.708091	9.544543	37.943379	15.157077
DNAAF1	0	2.095664	0	1.076613	0	1.126281	0	3.616131
DNAAF10	14.095178	18.974422	15.233356	15.868256	19.718298	12.244299	30.023571	23.452495
DNAAF11	3.990553	9.500098	23.229258	11.202815	0	3.52347	0	3.846591
DNAAF11P1	0	0	0	0	0	0	0	0
DNAAF2	9.63565	19.714693	3.06795	14.161169	0	18.136637	19.749267	24.061087
DNAAF3	8.898788	0	6.652773	2.146072	5.938641	2.989898	0	0.736291
DNAAF4	11.39198	10.89147	8.112091	9.848287	0.600068	6.396416	0	9.277733
DNAAF5	60.531581	42.386932	60.406392	55.199919	24.486492	40.707481	32.736682	35.282245
DNAAF6	0	0	0	0	0	0	0	0
DNAAF8	0	1.035089	0	2.081661	0	0.877057	0	1.004133
DNAAF9	0	0	0	0	0	0.169627	12.421241	0
DNAH1	7.961054	0.609655	0	0	0	0.285648	0	0
DNAH10	0	0	0.933236	0	0	0	0	0
DNAH11	7.521318	5.193164	8.064074	4.467293	0	1.360907	0	2.959694
DNAH12	0	0	0	0.111051	0	0	0	0
DNAH14	56.160811	42.426979	55.442484	15.223444	0.080574	15.291795	11.533908	33.891357
DNAH17	1.896014	0.273927	3.989075	0.168429	5.708211	0	0	0
DNAH2	0	0.163406	5.44918	0.289129	6.518604	2.111062	1.675325	2.216744
DNAH3	0	3.947437	0.232416	0.718378	0	0.142811	0	0.186056
DNAH5	10.959203	8.164704	10.016593	11.522585	0	3.161359	0	6.166637
DNAH6	0	0	0	0	0	0	0	0
DNAH7	0	0.369452	0	0.472973	6.160166	0.266623	0	0.349718
DNAH8	0	0	0	0	0	0	0	0
DNAH9	0	0	0	0	0	0	0	0.085592
DNAI1	0	0.456244	0	0.098131	0	0	0	0
DNAI2	0	0.707784	0	0	0	0.172353	0	0
DNAI3	1.165485	1.490808	1.04396	0.574814	1.185721	0.17408	0	0.361385

DNAI4	2.358525	4.966158	0	1.018481	15.742728	0.231777	0	0
DNAI7	3.9637	1.869598	9.388877	0.960577	0	1.915355	0	1.757269
DNAJA1	85.610327	139.462612	63.350788	105.945266	169.355177	178.918289	261.967275	236.708408
DNAJA1P1	0	0	0	0	0	0	0	0
DNAJA1P2	0	0	0	0	0	0	0	0
DNAJA1P3	0	1.03746	0	0.225494	0	0	0	0
DNAJA1P4	0	0	0	0	0	0	0	0
DNAJA1P5	0	0	0	0	0	0	0	0
DNAJA1P6	0	0	0	0	0	0	0	0
DNAJA2	137.932178	131.206883	128.822223	118.610533	88.992127	92.150902	151.511948	120.017336
DNAJA3	66.629954	53.900455	61.559712	69.480527	54.148944	68.838484	58.637684	43.863074
DNAJA4	0	1.389537	1.016698	0.304217	1.743554	4.357289	0	2.566095
DNAJB1	102.594103	129.016616	94.443816	141.189627	149.018667	241.575792	150.46689	172.749018
DNAJB11	0	2.25077	0	0.842643	0	0.374593	0	0.657009
DNAJB12	10.827624	2.138915	5.155578	12.469041	5.117967	10.707256	0	3.26294
DNAJB12P1	0	0	0	0	0	0	0	0
DNAJB13	0	0.603147	0	0	0	0	0	0
DNAJB14	7.629968	60.576225	13.775697	44.390186	30.256645	33.748485	7.60935	38.940051
DNAJB1P1	0	0	0	0	0	0	0	0
DNAJB1P2	0	0	0	0	0	0	0	0
DNAJB2	31.405758	18.30698	13.780925	21.451876	26.190546	34.867458	141.909145	29.566041
DNAJB3	0	0	0	0	0	0	0	0
DNAJB4	8.113393	26.674209	10.391826	23.453559	31.749144	17.081813	15.741134	26.569524
DNAJB5	2.855791	3.640394	5.10843	5.068291	22.883788	4.768516	0	4.026498
DNAJB5P1	0	0	0	0	0	0	0	0
DNAJB6	104.626634	220.336668	66.707592	262.048193	165.464725	253.436519	92.597299	271.470646
DNAJB6P1	0	1.092598	0	0.78333	0	2.000061	0	1.714673
DNAJB6P2	0	0	0	0	0	0	0	0
DNAJB6P3	0	0.874213	0	0.128687	0	0.25728	0	0
DNAJB6P4	0	0	0	0	0	0	0	0
DNAJB6P5	0	0	0	0	0	0	0	0
DNAJB6P6	0	0	0	0	0	0	0	0
DNAJB6P7	0	0	0	0	0	0	0	0.736445

DNAJB6P8	0	0	0	0	0	0	0	0
DNAJB8	0	0	0	0	0	0	0	0
DNAJB9	5.665384	9.498581	12.668606	13.883231	10.461318	9.50661	0	7.138281
DNAJC1	9.868301	9.271635	5.876911	9.311493	4.219684	3.828268	7.171171	5.240065
DNAJC10	0.651639	52.804781	43.337791	20.118303	2.145327	9.008607	1.22882	42.168321
DNAJC11	19.847159	27.036574	41.096059	54.906824	48.134981	75.887599	78.123546	52.422074
DNAJC12	12.153191	7.192174	5.385764	8.590238	0	4.342675	0	7.264396
DNAJC13	33.308044	46.462924	28.089711	24.495708	35.90694	11.981492	55.473242	34.12291
DNAJC14	0	0	0	8.946113	0	12.762948	6.204416	0
DNAJC15	8.514109	0.186513	0	0.074205	0	0.043493	0	0
DNAJC16	6.637526	7.869096	32.085558	13.616086	58.017174	8.394425	16.283395	9.951787
DNAJC17	27.875196	59.037833	14.800624	57.812995	32.889746	39.573433	66.367627	26.827211
DNAJC17P1	0	0	0	0	0	0	0	0
DNAJC18	26.690024	15.940882	4.605913	16.481147	18.478177	10.766958	2.475681	21.238827
DNAJC19	88.526378	106.80191	46.556915	70.965136	66.289862	39.650998	120.640529	94.765988
DNAJC19P1	0	0	0	0	0	0	0	0
DNAJC19P2	0	0	0	0	0	0	0	0
DNAJC19P3	0	0	0	0	0	0	0	0
DNAJC19P4	0	0	0	0	0	0	0	0
DNAJC19P5	0	5.322175	0	0.985822	5.500455	0.929604	0	0
DNAJC19P6	0	0	0	0	0	0	0	0
DNAJC19P7	0	0	0	0	0	0	0	0
DNAJC19P8	0	0	0	0	0	0	0	0
DNAJC19P9	0	0	0	0	0	0	0	0
DNAJC2	31.754472	37.07139	28.299817	31.46162	25.542098	29.848214	50.907157	47.656148
DNAJC21	36.087553	48.57378	36.596279	21.194532	48.344941	11.938817	36.438806	42.412953
DNAJC22	10.315958	27.410764	9.610504	22.953095	0.272319	4.117025	0	1.515617
DNAJC24	12.443049	9.403558	17.397056	8.858429	29.554468	22.151413	79.374357	22.221745
DNAJC25	1.503499	10.127224	3.655559	6.935593	2.775813	6.03426	11.663369	8.622039
DNAJC25-GN	0	5.751355	20.51527	2.972416	18.206712	1.898683	0	4.149908
DNAJC27	2.712862	2.033846	0	2.221099	1.25406	0.599267	0	1.358222
DNAJC28	0	1.926044	0	0.780076	0	0	0	0
DNAJC2P1	0	0	0	0	0	0	0	0

DNAJC3	40.227106	35.01828	35.10421	29.1077	21.648489	4.969776	51.562435	15.974063
DNAJC4	11.415903	18.047607	9.576889	12.636479	22.900739	22.238543	29.729622	9.158792
DNAJC5	35.204089	80.67453	49.594127	81.534806	48.442175	113.558881	41.832097	102.444244
DNAJC5B	0	0	0	0	0	0	0	0
DNAJC5G	0	0	0	0.130423	0	0.521305	0	0
DNAJC6	4.535654	4.411585	5.095706	6.211458	3.295448	4.155387	4.145081	6.556722
DNAJC7	71.88213	83.186779	76.031576	70.405957	30.945632	60.096221	110.139002	63.855207
DNAJC8	25.181117	63.898641	64.050712	57.474729	21.835703	72.288434	104.692712	86.697628
DNAJC8P1	0	0	0	0	0	0	0	0
DNAJC8P2	0	0.46385	0	0.410669	0	0.54853	0	2.37637
DNAJC8P3	0	0	0	0	0	0	0	0
DNAJC8P4	0	3.221351	0	0	0	0	0	0
DNAJC9	0	0	0	0.129549	0	0	75.881162	0
DNAJC9P1	0	0	0	0	0	0	0	0.117744
DNAL1	15.834433	43.665716	4.822105	37.776681	64.347678	12.237791	20.10099	23.350401
DNAL4	19.698625	26.318366	8.68869	22.701088	3.764642	16.781569	0.589031	9.580536
DNALI1	0	0	0	0	2.04135	1.173127	0	0.103815
DNASE1	0.753296	5.894494	0.677783	7.406912	2.30899	15.426542	0	6.921273
DNASE1L1	14.131644	26.352499	28.570065	33.845844	4.043098	26.834521	21.454428	22.010744
DNASE1L2	3.035372	0	0	0.272311	0	0	0	0
DNASE1L3	0	0	0	0	0	0	0	0
DNASE2	14.537302	10.801942	27.259787	21.532605	35.051204	19.570914	43.046726	15.816954
DNASE2B	0	0	0	0	0	0	0	0
DND1	0	0.563416	0	0.50327	1.176414	0.879051	0	0.090337
DND1P1	0	1.933956	0	1.271175	0	0.698156	0	0.733434
DNER	0	2.351893	0	1.574951	0	1.605809	0	2.569168
DNHD1	4.33756	0.282277	6.078932	2.055356	2.23763	1.607352	0	0.348868
DNLZ	0	2.097553	0	1.418051	1.999641	4.547764	0	6.259962
DNM1	30.876641	29.136927	33.404388	37.853807	16.330883	37.902881	18.691497	22.694194
DNM1L	53.415313	46.48717	32.903205	24.250201	12.948535	28.230572	98.838152	51.607123
DNM1P17	0	0	0	0	0	0	0	0
DNM1P24	0	0	0	0	0	0	0	0
DNM1P28	0	0	0	1.000064	0	0.487402	0	0

DNM1P30	0	0	0	0	0	0	0	0
DNM1P31	0	0	0	0	0	0	0	0
DNM1P32	0	0	0	0	0	0	0	0
DNM1P33	0	0	0	0	0	0	0	0
DNM1P34	0	0	0	0	0	0	0	0
DNM1P38	0	0	0	0	0	0	0	0
DNM1P46	0	0	0	0	0	0	0	0
DNM1P47	0	0	0	0	0.467238	0	0	0.080881
DNM1P48	0	0	0	0	0	0	0	0
DNM1P49	0	0	0	0	0	0	0	0
DNM1P5	0	0	0	0	0	0	0	0
DNM1P50	0	0	0	0	0	0	0	0
DNM1P51	0	0	0	0.309196	0	0	0	0
DNM2	48.376525	97.553676	46.54408	93.199473	36.464539	115.331069	71.687503	85.404497
DNM3	1.272757	1.663555	0	1.521129	1.881039	1.025291	0	0.639307
DNMBP	28.978923	27.350138	20.632565	31.486912	21.350962	27.852927	7.192644	24.876588
DNMT1	115.469103	118.091016	160.015262	130.669691	241.080892	258.786699	174.790818	116.965078
DNMT3A	15.767034	11.264677	14.167891	11.62207	50.997553	19.575768	39.838467	13.180877
DNMT3AP1	0	0	0	0	0	0	0	0
DNMT3B	11.418885	7.941687	5.12429	7.703165	1.723168	9.403519	1.324957	5.90281
DNMT3L	0	0	0	0	0	0	0	0
DNPEP	47.921118	43.029696	21.054955	51.413226	65.496695	84.828498	110.376286	84.666003
DNPH1	0	0	0	1.690454	15.358048	0	0	6.049201
DNTT	0	0	0	0	0	0	0	0
DNTTIP1	29.173736	54.269583	31.049545	50.637358	107.442222	49.885968	93.055776	42.882793
DNTTIP2	135.498623	98.055781	143.554499	69.848514	66.146592	38.905791	82.801985	59.411752
DOC2A	0	12.423345	4.615254	15.159224	1.54915	5.858948	0	2.094586
DOC2B	2.68095	2.611081	2.415748	3.02892	0	0.134737	0	0
DOC2GP	0	0	0	0.232481	0	0	0	0
DOCK1	31.44635	21.609496	16.922379	25.551059	16.700916	23.172778	17.663638	25.159531
DOCK10	6.571932	7.664993	0	6.643029	12.924545	0.092517	0	0
DOCK11	0	0.231343	0	0	0	0	0	0
DOCK11P1	0	0	0	0	0	0	0	0

DOCK2	0	0.181471	7.384429	0	1.577307	0	0	2.72709
DOCK3	0	0.305752	0	0.434196	0	0.035644	0	0.028423
DOCK4	0	2.050344	1.774742	1.745487	0	0.770366	0	4.175124
DOCK5	41.974085	57.067858	42.855061	79.297107	25.592091	57.949128	270.167335	45.705867
DOCK6	13.036072	7.652424	9.193911	8.527856	182.626616	10.505952	14.010056	9.60956
DOCK7	37.92656	56.392265	38.255851	51.643602	34.128348	49.734414	35.518541	28.442771
DOCK8	2.601892	2.22941	7.128135	0.941137	13.129663	1.409562	0	1.172182
DOCK8-AS1	0	0.825766	1.084596	0.646025	0	0.180811	0	0
DOCK9	42.691058	42.080029	107.891929	50.466951	15.485106	29.694817	115.820611	40.060137
DOK1	5.418482	11.460742	2.405679	9.904391	0	9.156078	0	7.270214
DOK2	0	0	0	0	0	0	0	0
DOK3	0	1.359114	0	0.526714	0	1.111387	0	1.882022
DOK4	5.378261	0	11.701885	14.744691	1.247375	13.954056	4.984054	4.276061
DOK5	0	0	0	0	0	0	0	0
DOK6	0	0	0	0.008696	0	0	0	0
DOK7	9.094746	3.541078	2.710093	5.064864	0.399778	9.720726	0	5.419017
DOLPP1	23.813355	22.560885	19.307563	20.691343	26.620313	15.752753	34.903399	13.689452
DONSON	24.00079	16.918236	21.927379	9.43281	29.52087	17.054263	34.830594	22.123251
DONSONP1	0	0	0	0	0	0	0	0
DOP1A	18.810535	0.731367	2.250439	1.492804	14.726685	0.077297	10.194736	5.0492
DOP1B	3.201859	7.705054	2.267798	6.753942	3.109236	10.986022	0.109109	5.427904
DOT1L	5.486479	13.591945	8.351911	16.619112	1.784551	21.298123	76.378124	11.168245
DPAGT1	109.900137	69.43311	125.549465	105.809094	38.055073	16.026245	51.289084	22.295949
DPCD	49.603247	35.109735	65.235231	49.530718	28.904706	26.539814	85.286346	30.020908
DPEP1	0	0	0	0	0	0	0	0
DPEP2	0	0	0	0	0	0	0	0
DPEP2NB	0	0	0	0	0	0	0	0
DPEP3	0	0	0	0	0	0	0	0
DPF1	7.664459	6.758409	2.659376	1.622958	0	11.705786	0	2.856095
DPF2	64.53165	20.648868	34.416373	36.365301	60.236649	47.645163	136.25678	48.27471
DPF3	0	1.370355	6.684147	0.683815	0	0	0	0.070906
DPH1	0	0	0	0.267876	55.542239	0	41.464677	0
DPH2	13.214763	14.880275	9.88376	18.257142	21.114472	37.549776	8.22616	36.020188

DPH3	29.466482	21.723337	16.041188	11.798624	8.411046	3.774812	23.6566	8.592434
DPH3P1	0	0	0	0	0	0	0	0
DPH3P2	0	0	0	0	0	0	0	0
DPH5	72.420239	126.575859	78.583312	93.648565	32.607013	25.038234	31.743586	41.312739
DPH6	40.502427	76.576795	21.755204	34.124582	2.402237	13.817651	2.564735	16.022245
DPH7	26.354209	17.017815	5.22632	18.824991	34.93581	30.313902	31.383225	21.598916
DPM1	0	3.362082	23.098865	34.339231	28.137763	36.230116	24.982365	24.581296
DPM2	23.451886	31.789268	5.845595	40.818539	5.300033	29.42018	32.446345	21.886593
DPM3	0	0	0	4.116425	0	2.854741	0	2.539879
DPP10	0	0	0	0	0	0	0	0
DPP3	63.253632	56.521416	41.888464	35.960588	96.902491	42.480153	211.263234	28.479375
DPP3P1	0	0	0	0	0	0	0	0
DPP3P2	0	0	0	0	0	0	0	0
DPP4	7.901805	2.523208	0	1.724201	0	0.506196	0	1.463078
DPP6	0	0	0	0.069571	0	0	0	1.062127
DPP7	62.087617	27.126929	23.759828	25.921942	63.453574	32.34202	0	10.874418
DPP8	31.316273	49.825618	24.447327	61.825059	55.534302	45.550117	99.212639	86.349949
DPP9	0	2.252614	0	5.304687	14.069592	23.519348	20.701395	17.89326
DPPA2	0	0	0	0	0	0	0	0
DPPA2P1	0	0	0	0	0	0	0	0
DPPA2P2	0	0	0	0	0	0	0	0
DPPA2P3	0	0	0	0	0	0	0	0
DPPA2P4	0	0	0	0	0	0	0	0
DPPA3	0	0	0	0	0	0	0	0
DPPA3P1	0	0	0	0.253915	0	0	0	0
DPPA3P10	0	0	0	0	0	0	0	0
DPPA3P11	0	0	0	0	0	0	0	0
DPPA3P12	0	0	0	0	0	0	0	0
DPPA3P2	0	0	0	0	0	0	0	0
DPPA3P3	0	0	0	0	0	0	0	0
DPPA3P5	0	0	0	0	0	0	0	0
DPPA3P6	0	0	0	0	0	0	0	0
DPPA3P7	0	0	0	0	0	0	0	0

DPPA3P8	0	0	0	0	0	0	0	0
DPPA3P9	0	0	0	0	0	0	0	0
DPPA4	0	0	0	0	0	0	0	0
DPPA4P2	0	0	0	0	0	0	0	0
DPPA4P3	0	0	0	0	0	0	0	0
DPPA5	0	0	0	0	0	0	0	0
DPPA5P1	0	0	0	0	0	0	0	0
DPPA5P2	0	0	0	0	0	0	0	0
DPPA5P4	0	0	0	0	0	0	0	0
DPRXP1	0	0	0	0	0	0	0	0
DPRXP2	0	0	0	0	0	0	0	0
DPRXP3	0	0	0	0	0	0	0	0.337133
DPRXP4	0	0	0	0	0	0	0	0.341669
DPRXP5	0	0	0	0	0	0	0	0
DPRXP6	0	0	0	0	0	0	0	0
DPRXP7	0	0	0	0	0	0	0	0
DPT	0	0	0	0	0	0	0	0
DPY19L1	36.81513	8.375226	17.519826	0	57.822116	3.776305	137.380252	10.894738
DPY19L1P1	3.922922	0.055654	0	0.25656	0.345012	0.260006	0	0.24605
DPY19L1P2	0	0	0	0.107962	0	0	0	1.807502
DPY19L2	0	0	0	0.949024	0	1.335656	0	0.204035
DPY19L2P2	0	0.937939	0	0.257666	0	0	0	0
DPY19L2P3	0	0	0	0	0	0.073	0	0
DPY19L2P4	0	0	0	0	0	0	0	0
DPY19L2P5	0	0	0	0	0	0	0	0
DPY19L3	8.622417	6.910593	22.086331	10.5977	0.580198	6.874177	2.024534	22.986975
DPY19L4	13.707805	51.288124	79.13406	36.824202	25.55803	21.788957	30.113857	33.846257
DPY19L4P1	0	0	0	0	0	0	0	0
DPY19L4P2	0	0	0	0	0	0	0	0
DPY30	156.192374	175.597188	121.36705	138.631349	145.33741	91.609326	109.175247	137.64974
DPYD	2.597157	3.138252	0	1.04513	0	0.170409	0	2.234266
DPYS	0	0	0	0	0	0	0	0
DPYSL2	49.211158	38.234607	47.120496	48.251097	20.156842	20.940213	30.746362	26.143773

DPYSL3	41.563515	45.468647	40.018756	31.364317	12.863181	18.974233	13.784212	18.884621
DPYSL4	2.315214	2.867649	6.181103	4.248839	0	0.71874	0	0.255745
DPYSL5	0.788497	1.040592	0	0.825432	0	1.182493	0	0.142241
DQX1	0	0	0	0.373051	0	0	0	0.065097
DR1	142.127636	246.300343	73.338233	167.870914	51.542097	105.760194	57.175959	169.96254
DRAM1	13.391735	28.957722	45.452	17.344084	179.675394	78.48687	131.501185	137.694362
DRAM2	18.053069	15.069814	0	22.552566	0	4.549469	36.39905	16.844028
DRAP1	536.94729	957.184592	566.92992	1172.7114	365.029475	827.186541	461.610202	548.225559
DRAXIN	0	0	0	0.139609	0.579941	0.02203	0	0
DRAXINP1	0	0	0	0	0	0	0	0
DRC1	0	0	0	0	0	0	0	0
DRC12	0	3.572719	5.642381	5.064765	0	2.792249	0	1.813076
DRC3	0	0.782384	0	1.149127	0.148143	0.065628	0	0
DRC7	0	1.385945	0	1.04459	0	0	0	0
DRD1	0	1.298325	0	0.480027	0	0.122836	0	0.263193
DRD2	0	0.119122	0	0	0	0.417215	0	0
DRD3	0	0	0	0	0	0	0	0
DRD4	0	0	0	0.063913	0	0.129585	0	0.321741
DRD5P1	0	0	0	0	0	0	0	0
DRD5P2	0	0	0	0	0	0	0	0
DRG1	17.586176	12.021214	62.552729	17.951792	22.349713	19.489185	57.589178	7.786777
DRG1P1	0	0	0	0	0	0	0	0
DRG1P2	0	0	0	0	0	0	0	0
DRG2	11.018182	25.14182	12.210263	20.422246	25.292556	28.764433	53.253111	26.321957
DRICH1	0	0	0	0	0	0.276044	0	0
DROSHA	27.566417	131.573093	56.467613	180.272471	205.0049	180.231111	289.535236	108.475649
DRP2	0	0	0	0.080801	0	0	0	0
DSC1	0	0.165935	0	0	0	0	0	0
DSC2	0	0.832941	0	0.834343	6.887605	0.518296	0	0.39352
DSC3	16.943764	8.793267	21.939284	16.521742	46.832453	16.401098	55.560902	18.445126
DSCAM	0	0.182584	0	0.059377	0	0.056627	0	0.02436
DSCAML1	0	0.044023	0	0.162732	0	0.053433	0	0.020521
DSCC1	15.64702	15.666011	9.788684	4.298162	17.330887	9.143545	6.446175	25.420023

DSE	31.367103	80.166529	43.339294	84.609447	52.245127	115.16949	34.338604	110.342561
DSEL	0	0	0	0.160727	0	0.343895	0	2.749226
DSG1	0	0	0	0	0	0	0	0
DSG2	57.168709	80.124858	85.805973	72.898975	99.251088	52.410515	60.742831	82.169941
DSG3	13.436373	10.014893	16.27525	12.240863	23.922906	10.743741	16.655573	14.199614
DSG4	0	0	0	0	0	0	0	0
DSN1	20.367219	109.754054	44.16363	85.332578	7.457891	32.46655	78.524387	55.330869
DSP	38.990995	24.375242	58.733902	43.915253	112.047435	81.030652	86.509963	56.707127
DSPP	0	0	0	0	0	0	0	0
DST	17.602671	39.892377	37.10897	28.963207	71.839743	22.26432	64.679422	47.848019
DSTN	311.491535	447.191428	200.991801	172.945063	298.851553	284.986781	372.757909	900.796272
DSTNP1	0	0	0	0	0	0	0	0
DSTNP2	0	0	0	0	0	0	0	0
DSTNP3	0	0	0	0	0	0	0	0.656071
DSTNP4	0	0	0	0	0	0	0	0
DSTNP5	0	0	0	0	0	0	0	0
DSTYK	8.343543	13.717075	3.752713	12.31341	8.820087	14.700099	5.291187	19.201116
DTD1	0	0.419471	2.314543	0.102161	0.719343	0.586255	0	1.045498
DTD2	36.492994	22.158683	44.977924	13.076473	38.624127	11.231041	91.150025	21.197941
DTHD1	0	0	0	0	0	0	0	0
DTL	63.1729	48.32231	31.478924	47.925785	44.723285	59.664899	18.031062	52.282947
DTNA	5.610421	1.393606	2.117156	1.297619	0	0	0	1.728946
DTNB	19.98513	9.648994	9.89236	18.349164	13.286566	12.71626	0	20.982106
DTNBP1	0	0	12.722603	7.352448	34.498543	18.044909	1.137776	4.266905
DTWD1	19.86219	41.763189	32.927505	10.458177	4.076982	10.101293	0	31.509073
DTWD1P1	0	0	0	0	0	0	0	0
DTWD1P2	0	0	0	0	0	0	0	0
DTWD2	2.254445	1.418855	2.030346	0.979384	5.509675	0.706244	5.505301	2.410902
DTX1	0	0.268473	0	0.107966	0	0	0	0
DTX2	13.410423	32.105518	18.565187	15.823866	21.0111	19.609018	25.421466	28.516703
DTX2P1	2.174647	1.921099	1.93736	2.454105	6.172839	4.958168	0	4.202762
DTX3	27.398949	20.398285	12.953559	26.293397	16.468	55.982508	29.754494	32.909255
DTX3L	34.993957	34.342316	39.061843	44.579193	131.643957	83.868665	76.516394	65.441538

DTX4	11.374151	11.092414	21.826661	21.649997	38.589287	24.111653	70.251512	11.196361
DTYMK	54.506473	89.763693	32.200839	66.901498	92.921565	150.740498	87.048476	197.902799
DUOX1	2.091347	5.521926	2.599422	7.05327	18.310185	6.341153	10.750241	8.253698
DUOX2	1.534687	0.152525	0	0.158983	0.135171	0	0	0
DUOXA1	8.298224	21.426978	11.742932	33.666343	14.645232	40.705455	14.520418	28.436255
DUOXA2	0	0.689981	2.242441	0	0	0.128434	0	0.408813
DUS1L	5.119379	82.02144	73.021783	32.054193	51.383457	101.567825	70.021382	74.105305
DUS2	26.915902	20.763922	31.701428	27.371511	19.132224	29.539209	13.455925	39.091449
DUS3L	13.293699	13.747578	4.553632	16.413947	5.816936	22.574894	255.255192	16.791614
DUS4L	8.764802	25.103952	9.408342	16.019017	27.239406	15.336902	0	24.410061
DUSP1	31.012808	37.849866	33.845199	57.195201	35.88463	82.351234	7.508909	51.717196
DUSP10	5.241435	9.17213	8.209346	11.760232	6.223101	13.548	42.325729	17.145248
DUSP11	0	3.903743	1.611533	0.983506	0.460357	0.323696	0	0.758112
DUSP12	0	12.55799	0	15.429602	66.553711	10.119927	0.709412	14.628023
DUSP12P1	0	0	0	0	0	0	0	0
DUSP13A	0	0	0	0	0	0	0	0
DUSP13B	0	0	0	0	0	0	0	0
DUSP14	22.538744	110.635705	33.158185	96.427895	45.245147	153.091656	20.616086	210.157029
DUSP15	0	1.327133	3.118352	0.934586	0	0	0	0
DUSP16	13.021036	24.749765	0	23.387087	12.682626	21.906075	78.854638	32.929511
DUSP18	0	4.714641	5.750232	5.210212	0	5.331664	0	3.241607
DUSP19	0	0	5.974493	0	0	0	0	0.594062
DUSP2	16.794638	11.598248	13.093658	14.937181	6.637012	6.103357	0	5.600959
DUSP22	13.988153	26.54573	31.139536	20.59701	38.068882	35.100335	85.310624	17.991125
DUSP23	0	16.562976	0	34.123409	19.368932	14.818891	0	5.206348
DUSP26	0	0.46463	0	0.243334	0	0	0	0
DUSP28	0	2.391076	0	1.58761	0	1.154786	0	2.66343
DUSP3	52.051043	40.254098	50.439614	37.341085	27.170658	42.529191	41.165599	52.225085
DUSP4	11.740379	27.039051	12.133462	22.60688	4.652636	17.591819	8.296457	40.471765
DUSP5	68.717547	99.862192	54.101976	79.052373	66.432498	69.918366	63.684014	98.104463
DUSP5P1	0	0.684168	0	2.143898	0	1.764924	0	1.878705
DUSP5P2	0	0	0	0.178206	0	0	0	0
DUSP6	123.529164	280.421604	120.911942	417.943498	99.507324	193.284397	83.490779	290.285755

DUSP7	25.791649	43.198519	32.908821	33.040157	15.974808	40.613446	32.075835	46.665488
DUSP8	0.737918	0.56953	6.314665	1.671511	0	8.191001	0	0.770146
DUSP8P1	0	0	0	0.132435	0	0	0	0
DUSP8P5	1.932043	0.489345	1.723095	0.70301	0	0.476895	0	1.716729
DUSP9	5.602446	5.671193	5.353819	8.133	1.184563	6.212737	0	7.683111
DUT	303.160791	259.05808	209.866976	157.027741	292.283474	153.138418	361.371139	223.400453
DUTP1	0	0	0	0	0	0	0	0
DUTP2	0	0	0	0	0	0	0	0
DUTP3	0	0	0	0	0	0	0	0
DUTP4	0	0	0	0	0	0	0	0
DUTP5	0	0	0	0	0	0	0	0
DUTP6	11.238645	0	0	0	0	0	0	0
DUTP7	0	0	0	0	0	0	0	0
DUTP8	0	0	0	0	0	0	0	0
DUX4	0	0	0	0	0	0	0	0
DUX4L10	0	0	0	0	0	0	0	0
DUX4L11	0	0	0	0	0	0	0	0
DUX4L12	0	0	0	0	0	0	0	0
DUX4L13	0	0	0	0	0	0	0	0
DUX4L16	0	0	0	0	0	0	0	0
DUX4L17	0	0	0	0	0	0	0	0
DUX4L18	0	0	0	0	0	0	0	0
DUX4L19	0	0	0	0	0	0	0	0
DUX4L21	0	0	0	0	0	0	0	0
DUX4L24	0	0	0	0	0	0	0	0
DUX4L25	0	0	0	0	0	0	0	0
DUX4L26	0	0	0	0.141944	0	0.287266	0	0.238885
DUX4L27	0	1.463048	0	0.776192	0	0.571435	0	0.356301
DUX4L28	0	0	0	0	0	0	0	0
DUX4L29	0	0	0	0	0	0	0	0
DUX4L31	0	0	0	0	0	0	0	0
DUX4L32	0	0	0	0	0	0	0	0
DUX4L33	0	0	0	0	0	0	0	0

DUX4L34	0	0	0	0.142122	0	0	0	0
DUX4L35	0	0	0	0	0	0	0	0
DUX4L37	0	0	0	0	0	0	0	0
DUX4L4	0	0	0	0	0	0	0	0
DUX4L45	0	0	0	0	0	0	0	0
DUX4L46	0	0	0	0	0	0	0	0
DUX4L47	0	0	0	0	0	0	0	0
DUX4L50	0	8.329443	15.514888	5.62475	0	10.936217	0	9.292172
DUX4L51	0	0	3.012578	0	0	0	0	0
DUX4L52	0	0	0	0	0	0	0	0
DUX4L6	0	0	0	0	0	0	0	0
DUX4L7	0	0	0	0	0	0	0	0
DUX4L8	0	0	0	0	0	0	0	0
DUX4L9	0	0	0	0	0	0	0	0
DUXAP1	0	0	0	0.258966	0	0	0	0
DUXAP10	0	0	0	0	0	0	0	0
DUXAP11	0	0	0	0	0	0	0	0
DUXAP2	0	0	0	0	0	0	0	0
DUXAP3	0	0	0	0	0	0	0	0
DUXAP4	0	0	0	0	0	0	0	0
DUXAP5	0	0	0	0	0	0	0	0
DUXAP6	0	0	0	0	0	0	0	0
DUXAP7	0	0	0	0	0	0	0	0
DUXAP8	0	0	0	0	0	0	0	0
DUXAP9	0	0	0	0	0	0	0	0
DVL1	19.538701	29.16564	35.00601	31.982883	39.708105	60.510529	0.914135	44.288292
DVL2	20.223453	26.839875	18.444393	36.093619	11.7591	51.764361	73.587272	30.102072
DVL3	31.609885	37.465426	33.970842	44.45319	31.765363	67.615411	66.749729	44.717393
DXO	41.341729	5.590303	3.894849	8.25897	0.684852	11.961543	60.478123	8.400768
DYDC1	0	0	0	0.163501	0	0	0	0
DYDC2	0	0	0	0	0	0	0	0.259011
DYM	32.843324	77.175555	106.54117	58.639321	32.58746	47.179626	45.147341	102.581286
DYNAP	0	0	0	0	0	0	0	0

DYNAPP1	0	0	0	0	0	0	0	0
DYNC1H1	53.057863	80.537464	64.669529	81.140735	161.921534	80.774163	132.301707	58.045521
DYNC1I1	0	1.003011	0	0.648722	0	0.347656	0	0.518098
DYNC1I2	105.937505	109.810689	58.966315	143.186602	74.553255	139.701304	223.195095	171.393761
DYNC1I2P1	0	0	0	0	0	0	0	0
DYNC1LI1	21.913176	26.173613	35.784463	16.28877	15.034398	19.682263	59.770016	23.557809
DYNC1LI2	84.668078	109.49088	168.15427	114.266857	80.161202	133.55709	264.384267	142.425671
DYNC2H1	15.284722	14.981903	29.686976	15.318994	8.955683	1.719318	8.787121	7.132429
DYNC2I1	16.315806	10.822202	7.059308	19.003481	20.34036	14.8189	0.888047	11.440191
DYNC2I2	0	1.266162	0	0.175607	0	1.831144	0	2.160222
DYNC2LI1	24.404148	43.074084	39.172358	40.88436	12.969574	12.628227	22.851912	16.732353
DYNLL1	342.030499	477.899136	295.776238	410.530694	371.326807	530.78059	378.791071	709.44645
DYNLL1P1	0	0	0	0	0	0	0	3.995374
DYNLL1P2	0	0	0	0	0	0	0	0
DYNLL1P3	0	0	0	0	0	0	0	0
DYNLL1P4	0	0	0	0	0	0	0	0
DYNLL1P5	0	0	0	0	0	0	0	0
DYNLL1P6	0	0	0	0	0	0	0	0
DYNLL1P7	0	0	0	0	0	0	0	0
DYNLL2	53.774378	129.282261	42.793375	88.638268	38.731366	86.682205	40.031443	105.334616
DYNLRB1	7.385033	9.404729	0	4.273169	0	5.431005	0	3.025283
DYNLRB2	0	0	0	0	0	0	0	0.428895
DYNLT1	80.75037	64.927668	70.481385	68.397816	61.579709	61.354857	5.46224	79.751733
DYNLT2	0	0.845765	1.486129	0.202116	0	0.329524	0	0.872389
DYNLT2B	21.406785	43.482625	30.913553	23.691721	54.004143	25.66491	63.743635	54.675155
DYNLT3	35.455638	16.497413	5.725948	11.629991	22.813308	11.867809	6.598784	20.663969
DYNLT3P1	0	0	0	0	0	0	0	0
DYNLT3P2	0	0	0	0	0	0	0	0
DYNLT5	0	0	0	0	0	0	0	0
DYRK1A	28.576387	97.267783	29.550347	74.228554	101.220184	146.466501	71.104841	209.707334
DYRK1B	1.94276	10.26189	2.490427	2.550125	3.595972	19.883842	33.826117	11.036815
DYRK2	24.391668	42.906121	14.620527	49.559716	20.598061	159.004934	62.683247	110.307514
DYRK3	0	14.126788	11.2984	19.66785	48.12869	16.564968	45.037165	27.515675

DYRK4	23.471877	40.492197	26.092744	45.729205	53.684888	23.360326	2.523508	23.187098
DYSF	2.478174	2.827799	0	2.21904	2.619335	1.80597	46.652478	1.013993
DYTN	0	0	0	0	0	0	0	0
DZANK1	0	3.726171	1.807707	3.053105	0.842837	2.142568	6.895232	2.269858
DZIP1	24.49539	2.716279	0	23.008438	8.468963	32.380813	55.283103	17.691424
DZIP1L	3.810831	6.243896	1.708801	5.125508	22.746549	1.992376	0	3.93105
DZIP3	12.926867	19.075813	12.721432	8.011774	15.511413	2.545495	8.248581	5.611682
E2F1	15.090665	19.708078	24.768525	16.153578	42.142223	18.388684	17.095682	14.475893
E2F2	5.030627	7.560544	2.259922	7.100645	5.149747	9.543182	0.153194	5.777191
E2F3	9.743444	12.98512	9.046459	10.832814	16.919037	19.746188	20.094822	26.440214
E2F3P1	0	0	0	0.290469	0	0.293904	0	0
E2F3P2	0	0	0	0.123046	0	0.124804	0	0.721861
E2F4	125.85572	70.80681	78.93026	71.918991	54.251401	80.58506	83.244669	67.203084
E2F4P1	0	0	0	0	0	0	0	0
E2F5	13.766043	14.686163	10.257222	8.571693	6.201751	8.260971	0	10.771282
E2F6	33.608364	71.066905	17.8487	48.911155	16.272027	27.787166	0	50.892144
E2F6P1	0	0	0	0	0	0	0	0
E2F6P2	0	0	0	0	0	0	0	0
E2F6P3	0	0	0	0	0	0	0	0
E2F6P4	0	0	0	0	0	0	0	0
E2F7	14.219961	26.564314	10.735629	28.059582	31.012358	32.610755	31.752516	36.84732
E2F8	22.767158	31.201596	11.238584	21.394762	24.456478	11.931942	26.423317	14.807826
E4F1	7.998486	7.678939	8.350624	10.939704	13.218305	9.533055	7.034626	12.215655
EAF1	20.700448	28.017295	29.208071	34.842895	24.856394	12.474851	8.999761	17.920839
EAF2	13.715099	5.099585	12.850745	3.138918	15.358769	2.666359	37.838066	4.57897
EAPP	42.235986	78.243006	29.9849	63.780246	47.414118	62.116805	40.688808	69.906043
EARS2	58.813068	13.827666	0	7.144236	31.719739	14.47173	61.006962	10.746036
EBAG9	2.641175	48.434489	23.025615	52.78496	69.180894	18.216757	0	14.29701
EBAG9P1	0	0	0	0	0	0	0	0
EBF1	0	0	0	0	0	0	0	0
EBF2	0	0.057747	0	0.046385	0	0	0	0.074659
EBF4	0	0	0	0	0	0.475574	0	0.783821
EBI3	0	1.342757	2.863832	3.195325	0	0.472287	0	0

EBLN2	0	0	0	0.155183	0	0.210315	0	0
EBNA1BP2	0	13.92859	0	12.394711	48.362108	14.830486	159.3811	10.293064
EBP	140.621424	156.371793	108.729692	125.128337	125.848545	156.028108	97.503008	147.442303
EBPL	83.529195	24.765695	37.135808	18.378419	29.003277	7.288595	41.058336	18.900816
ECD	39.445534	44.06769	49.414195	48.201408	40.161908	48.651239	84.960444	61.440947
ECE1	21.299504	24.225591	42.083798	44.12968	50.867203	66.821591	53.340199	36.332865
ECE2	0	0	0	0.068314	0	0	0	0
ECEL1	0	0	0	0.228608	0	0.073135	0	0
ECEL1P1	0	0	0	0	0	0	0	0
ECEL1P2	0	0	0	0	0	0	0	0
ECEL1P3	0	0	0	0	0	0	0	0
ECH1	55.819299	83.237339	63.193691	115.933238	68.278913	93.31113	41.642503	60.472541
ECHDC1	58.149479	51.316475	64.696169	46.610852	52.752132	68.687162	247.204684	69.859791
ECHDC2	49.061017	49.177337	31.414119	56.424611	39.714748	18.745855	0	19.670298
ECHDC3	0	0	0	0.675413	0	0.685052	0	0.703535
ECHS1	123.955565	141.477847	158.546201	128.488721	198.692534	119.89401	143.544587	162.862569
ECI1	66.869821	60.377142	90.199686	67.572378	20.427206	55.853579	0	36.604058
ECI2	41.606789	60.883864	16.382153	42.912229	31.337179	65.306569	123.584481	53.927944
ECM1	0	0.340594	2.223146	0.369998	0	4.45154	0	2.048098
ECM1P1	0	0	0	0	0	0	0	0
ECM1P2	0	0	0	0	0	0	0	0
ECM2	0	0	0	0	0	0.317987	0	0
ECMXP	0	0	0	0	0	0	0	0
ECPAS	64.737122	93.225909	66.319359	76.006509	74.266289	62.444387	112.936685	87.69415
ECRG4	0	0	0	0	0	0	0	0
ECSCR	0	0	0	0	0	0	0	0
ECSIT	21.538427	27.057376	21.282542	27.548852	27.637043	44.189631	51.765474	44.804271
ECT2	37.985835	167.971154	61.869954	71.643097	92.917236	76.207001	96.488605	187.805062
ECT2L	0	0	0	0.132737	0	0	0	0
EDA	0	0.435262	0.634761	0.418565	0	0.669786	0	0.141839
EDA2R	0	0	0	0	1.017483	0	0	0
EDAR	0	0.313312	0.729411	0.615287	1.502337	0.73118	0	0.358985
EDARADD	19.261358	0	6.018502	0.23566	0	0	0.352265	0

EDC3	47.217233	61.942111	31.806337	69.732042	24.056811	62.159576	25.508631	54.041813
EDC4	20.630574	48.444008	17.911998	43.589276	129.105656	54.89928	18.947269	40.974504
EDDM13	0	0	0	0.54534	0	0.280416	0	0.738934
EDDM3CP	0	0	0	0	0	0	0	0
EDDM3DP	0	0	0	0	0	0	0	0
EDEM1	67.51043	112.697586	108.175308	124.362056	31.461941	54.168107	43.450666	56.484739
EDEM2	24.097359	47.608752	33.078131	62.012495	10.56406	17.485964	14.537357	16.836546
EDEM3	16.106947	27.229125	11.253825	24.64295	9.760832	13.46283	8.960671	18.587028
EDF1	261.141297	238.563255	241.296089	311.837753	274.575267	365.768459	181.645209	264.19515
EDIL3	4.657579	5.430408	21.061045	14.501056	3.895256	0.23852	0	1.346437
EDN1	1.70246	3.387811	1.520313	4.631511	16.158787	17.272973	19.788092	12.293564
EDN2	0	0	0	0	0.775296	1.007252	0	1.196691
EDN3	0	0	0	0	0	0	0	0
EDNRA	7.418412	10.485563	15.741758	9.605746	1.143455	3.889277	19.570702	8.884448
EDNRB	0	0	0	0	0	0	0	0
EDRF1	9.530448	7.758686	9.874269	4.378292	6.787879	3.179071	17.989111	6.971363
EEA1	9.494632	17.359407	10.27355	12.517145	24.554897	25.339276	34.634711	27.543741
EED	89.617374	91.569447	62.499552	71.900702	56.567057	22.465667	71.733897	41.940965
EEDP1	0	0	0	0	0	0	0	0
EEF1A1	593.303923	249.640567	700.092914	355.420146	568.953524	228.36954	423.970024	236.939462
EEF1A1P1	0	0	0	0	0	0	0	0.233661
EEF1A1P10	0	0	2.321194	0.126261	0	0	0	0
EEF1A1P11	0	0.79368	0	0.322306	0	0.391326	0	0.106991
EEF1A1P12	0	0.345327	0	0.396242	0	0	0.654615	0.220901
EEF1A1P13	2.65421	0.463561	0	0.587182	0.702099	0.659565	0	1.419952
EEF1A1P14	0	0	0	0.127885	0	0	0	0
EEF1A1P15	0	0	0	0	0	0	0	0
EEF1A1P16	0	0	0	0	0	0	0	0
EEF1A1P17	0	0	0	0	0	0	0	0
EEF1A1P18	0	0	0	0	0	0	0	0
EEF1A1P19	0	0	0	0.064603	1.407924	0.264177	0	0
EEF1A1P2	0	0	0	0	0	0	0	0
EEF1A1P20	0	0	0	0	0	0	0	0

EEF1A1P21	0	0	0	0	0	0	0	0
EEF1A1P22	0	0.110302	0	0.191055	0	0.12913	0	0
EEF1A1P23	0	0	0	0	0	0.350189	0	1.251941
EEF1A1P24	0	0	0	0	0	0	0	0
EEF1A1P25	0	0	0	0	0	0.429344	0	0
EEF1A1P26	0	0	0	0	0	0	0	0
EEF1A1P27	0	0	0	0	0	0	0	0
EEF1A1P28	0	0.108202	0	0	0	0	0	0
EEF1A1P29	0	0	0	0	0	0	0	0
EEF1A1P3	0	0	0	0.253558	0	0	0	0.319026
EEF1A1P30	0	0	0	0	0	0	0	0
EEF1A1P31	0	0	0	0	0	0	0	0
EEF1A1P32	0	0	0	0	0	0	0	0
EEF1A1P33	0	0	0	0	0	0.261945	0	0
EEF1A1P34	0	0	0	0.070179	0	0	0	0
EEF1A1P35	2.622477	0	0	0.06381	0	0.128546	0	0
EEF1A1P36	0	0	0	0	0	0	0	0
EEF1A1P37	0	0	0	0	0	0	0	0
EEF1A1P38	0	0	0	0.063192	0	0.256308	0	0
EEF1A1P39	0	0	0	0	0	0	0	0
EEF1A1P4	0	0.544845	0	0	0	0	0	0
EEF1A1P40	0	0	0	0	0	0	0	0
EEF1A1P41	0	0	0	0	0	0	0	0
EEF1A1P42	0	0	0	0	0	0	0	0
EEF1A1P44	0	0	0	0	0	0	0	0
EEF1A1P45	0	0	0	0	0	0	0	0
EEF1A1P46	0	0	0	0	0	0	0	0
EEF1A1P47	0	0	0	0	0	0	0	0
EEF1A1P48	0	0	0	0	0	0	0	0
EEF1A1P49	0	0	0	0	0	0	0	0
EEF1A1P5	0	8.495217	39.740382	5.350387	11.479827	4.197235	1.338758	3.119264
EEF1A1P50	0	0	0	0	0	0	0	0
EEF1A1P6	0	2.81649	4.004152	4.061236	2.443287	1.872133	0	4.618844

EEF1A1P7	0	0	0	0.125947	0.685827	0.127777	0.627396	0.105563
EEF1A1P8	0	0	0	0.126474	0	0	0	0
EEF1A1P9	0	0	0	0.383228	0.694848	0.654252	0	0.214078
EEF1A2	279.691785	229.873886	353.434582	386.34716	414.583124	598.422189	450.341102	393.619688
EEF1AKMT1	4.558588	12.440949	4.006056	8.818416	1.195495	0	0	7.264783
EEF1AKMT2	41.362875	53.813663	26.275112	33.592772	20.887196	24.530503	29.767767	51.629031
EEF1AKMT3	15.108749	13.481178	5.635908	14.421988	9.302307	18.007186	0	18.570136
EEF1AKMT4-	0	0	0	0.051266	0	0	0	0
EEF1B2	689.558907	501.746324	739.673132	425.846922	754.71823	517.91229	577.889787	667.541805
EEF1B2P1	0	0	0	0	0	0	0	0
EEF1B2P2	0	0	0	0	0	0	0	0
EEF1B2P3	0	1.447138	0	0	0	0.356273	0	0.641414
EEF1B2P4	0	0	0	0	0	0	0	0
EEF1B2P5	0	0	0	0	0	0	0	0
EEF1B2P6	0	0	0	0.157652	1.736849	0.311484	0	0
EEF1B2P7	0	0	0	0	0	0	0	0
EEF1B2P8	0	0	0	0	0	0	0	0
EEF1D	463.78706	452.219119	557.865159	604.700549	483.19506	435.873487	397.822853	326.310047
EEF1DP1	0	0	0	0	0	0	0	0.181016
EEF1DP2	0	1.556559	4.195063	0.456294	0	0	0	1.567337
EEF1DP3	0	0	0	0	0	0	0	0.432851
EEF1DP4	0	0	0	0	0	0	0	0
EEF1DP5	0	0	0	0	0	0	0	0
EEF1DP6	0	0	0	0	0	0	0	0
EEF1DP7	0	0	0	0	0	0	0	0.400768
EEF1DP8	0	0	0	0	0	0	0	0
EEF1E1	36.505995	36.534438	38.626434	36.990858	84.654309	22.901005	38.863987	37.142522
EEF1E1-BLO(	0	0	0	0	0	0	0	0
EEF1E1P1	0	2.357137	0	0.871837	0	0	0	2.156096
EEF1G	1614.5191	695.75954	1911.90378	936.289147	1956.90172	1044.31405	1527.93471	847.096615
EEF1GP1	0	0	0	0	0	0.276284	0.67999	0
EEF1GP2	0	0	0	0	0	0	0	0
EEF1GP3	0	0	0	0	0	0	0	0

EEF1GP4	0	0	0	0	0	0	0	0
EEF1GP5	0	0	0	0	0	0.136834	0	0
EEF1GP6	0	0	0	0	0	0	0	0
EEF1GP7	0	0	0	0.143756	0	0	0	0
EEF1GP8	0	0	0	0	0	0	0	0
EEF2	1545.37698	813.041806	2082.55735	1119.90407	1215.15681	845.11049	1406.39918	536.412526
EEF2K	0	8.570533	0	18.305005	3.436336	17.077951	26.531265	5.136355
EEF2KMT	11.561463	12.204235	9.983302	9.582137	3.42876	17.970205	0	18.465703
EEFSEC	9.355701	13.438396	12.542252	16.767761	20.085793	31.514825	61.902426	19.98715
EEIG1	7.10953	15.271294	10.215494	20.178335	8.212616	36.215908	30.305525	46.867602
EEIG2	7.003791	9.020401	5.76976	6.136836	5.242752	3.0922	2.417807	4.057837
EEPD1	21.886812	13.530633	0	19.802872	0	15.319199	0	13.534477
EFCAB10	0	0	0	0	0	0.269944	0	0
EFCAB11	47.779024	57.99369	18.762266	28.708027	78.932921	23.112615	44.029274	30.017078
EFCAB12	0	0.204984	0	0.274217	0	0.159714	0	0.32498
EFCAB13	0	2.191861	0.861131	3.014137	0	0	0	0.098683
EFCAB14	32.992119	32.490237	29.254614	30.202636	56.674099	27.879962	23.088797	41.293862
EFCAB14P1	0	0	0	0	0	0	0	0
EFCAB15P	0	0	0	0	0	0.169515	0	0
EFCAB2	43.832085	37.772193	16.369715	17.996344	26.749288	11.834995	10.660163	32.644813
EFCAB3	0	0	0	0	0	0	0	0
EFCAB3P1	0	1.765476	9.93624	2.706139	0	1.051872	0	1.494527
EFCAB5	0	0.110805	0	1.233071	2.270724	1.441792	0	3.757596
EFCAB6	0	1.592992	0.667405	2.613205	0	1.244064	0	0.711544
EFCAB7	18.964715	20.439122	8.473686	11.33328	61.027226	5.858465	3.412748	6.173468
EFCAB8	0	0	0	0	0	0	0	0
EFCC1	0	0.374652	0	0.153062	0	0.175202	0	0.071483
EFEMP1	244.128853	411.275909	250.622607	301.115693	154.565524	118.462713	69.661799	105.632214
EFEMP2	31.412621	17.132309	38.208809	14.708454	8.66655	2.006688	0	3.914609
EFHB	0	0.301233	0	0.688457	0	0	0	0
EFHC1	49.040815	12.297513	21.285975	8.798064	7.402613	5.056256	0	4.964141
EFHC2	2.051108	2.584654	0	1.425473	0	0.204557	0	0.301863
EFHD1	0	0.943181	0	0.205594	0	0.686344	0	0

EFHD2	119.69022	241.758612	156.185652	271.130994	53.489415	144.977615	70.35752	121.104665
EFL1	18.748145	36.221708	26.970883	41.701255	8.392803	28.846744	0	23.433431
EFL1P1	0	0	0	0	0	0	0	0
EFL1P2	0	0	0	0	0	0	0	0
EFNA1	29.943823	70.155025	49.199153	85.708597	15.77191	62.94614	48.831274	55.255503
EFNA2	0	0.717552	0	0.479235	0	0.20957	0	0.113336
EFNA3	0	15.830999	15.489073	18.413816	21.823186	15.654275	5.050683	8.648954
EFNA4	2.944628	12.988857	15.66741	14.888326	0	8.498834	0	7.292894
EFNA5	19.946855	29.84545	10.371753	67.946695	88.173981	23.333333	0	34.836205
EFNB1	45.469386	145.390139	48.916985	184.827105	44.545953	268.900849	44.213462	229.605016
EFNB2	22.715365	47.246095	14.712378	32.717153	12.989069	29.750261	14.976588	43.798219
EFNB3	3.118261	0.754663	1.863412	2.63605	1.920663	3.523947	7.073815	2.758036
EFR3A	53.343241	70.223324	42.885462	49.222887	41.112398	90.243954	36.281814	151.56719
EFR3B	2.829149	0.330203	0	0.337974	0.237689	0.786734	0	0.207589
EFS	12.883856	20.181842	11.367878	22.751152	9.093295	33.971886	7.12589	24.424054
EFTUD2	145.817967	115.646823	114.620585	135.2801	174.937303	84.80546	314.79966	133.025634
EGF	0	1.020487	0	0.155673	0	0.956851	0	0.960775
EGFEM1P	0	0	0	0	0	0	0	0
EGFL6	0	0	0	0.034832	0	0.071075	0	0
EGFL7	14.347827	12.446255	28.003184	21.438525	0	9.244128	23.471763	4.309116
EGFL8	0	2.165112	0	0.041113	0	1.260343	0	0
EGFLAM	0	0.41009	0	0.199767	0	0	0	0
EGFR	96.641722	95.652061	128.060303	158.463222	222.185831	341.211899	236.421679	357.947358
EGLN1	39.116459	72.98722	21.714255	40.354762	26.5947	38.909479	40.2567	91.51717
EGLN1P1	0	0	0	0	0	0	0	0
EGLN2	24.885298	27.650985	0	26.36283	35.127866	72.588364	88.87449	50.743288
EGLN3	11.231492	15.425193	2.44536	22.93185	17.316441	2.120747	38.120778	39.785325
EGLN3P1	0	0	0	0	0	0	0	0
EGR1	420.43805	602.181089	499.281135	699.84614	304.523347	456.07494	202.016244	240.265262
EGR2	18.040709	13.100526	16.163084	21.282512	3.235102	2.808065	0	0
EGR3	8.006496	8.048251	2.614367	4.793824	1.345737	3.451479	0	2.421049
EGR4	0	0.489521	0	0.700753	0	0.14298	0	0.348147
EHBP1	13.340881	102.137192	90.73699	134.775719	70.302476	97.959238	120.144908	89.680836

EHBP1L1	17.066894	37.475867	25.556217	61.845327	115.050786	253.942045	141.684832	126.807683
EHD1	43.927215	23.828379	19.721704	31.486358	97.84538	120.743819	74.789154	95.71648
EHD2	32.255567	26.65229	31.480317	33.456148	122.731682	99.132662	86.844611	68.326378
EHD3	6.111831	12.11495	9.149499	10.885033	6.099011	8.91437	4.796381	10.764332
EHD4	28.896415	40.565867	23.701465	35.339615	37.775338	42.707656	19.403394	29.928461
EHF	10.632968	32.714655	7.726283	99.636217	96.598434	151.738387	0	89.928702
EHHADH	30.653117	38.668111	16.417348	42.788979	10.583665	19.330806	8.055281	28.334533
EHMT1	40.418163	79.264788	59.243096	73.193436	44.512791	109.346034	155.457244	82.20744
EHMT2	19.217072	14.527454	15.708699	13.905174	27.013717	22.212494	55.970935	14.564045
EI24	121.337526	107.524981	126.173967	83.718453	136.674137	197.113656	366.467135	272.207566
EI24P1	0	0	0	0	0	0	0	0
EI24P2	0	0	0	0	0	0	0	0
EI24P3	0	0	0	0	0	0	0	0
EI24P4	0	0	0	0	0	0	0	0
EI24P6	0	0	0	0	0	0	0	0
EID1	32.232168	158.206115	0	142.819682	58.939155	133.782484	78.549434	116.440531
EID2B	0	0	0	0.774201	0	1.593443	0	0
EID3	0	1.132684	0	0.653361	0	0.482182	0	1.094074
EIF1	1269.74699	2188.32684	1403.909	1994.31903	730.332627	1021.74088	1108.12016	1489.10122
EIF1AD	70.0112	190.872384	44.258387	211.560639	70.823205	182.634957	46.617745	194.825789
EIF1AX	19.07672	87.399322	39.09289	38.357568	76.29408	114.102341	62.762118	263.21
EIF1AXP1	66.937273	150.05602	31.057737	94.611319	0	135.277066	0	237.368368
EIF1AXP2	0	0	0	0	0	0	0	0
EIF1AY	16.368	66.951918	23.73345	39.248558	69.567073	64.604459	41.165766	81.615575
EIF1B	51.002047	66.215421	31.140219	45.340501	32.171189	28.617415	0	61.205003
EIF1P1	0	0	0	0	0	0	0	0
EIF1P2	0	0	0	0	0	0	0	0
EIF1P3	0	0	0	0	0	0	0	0
EIF1P4	0	0	0	0	0	0	0	0
EIF1P5	0	0	0	0	0	0	0	0
EIF1P6	0	0	0	0	0	0	0	0
EIF1P7	0	0	0	0	0	0	0	0
EIF2A	126.358944	99.09074	242.47288	96.573946	149.310028	61.2043	217.514883	101.119482

EIF2AK1	281.245548	532.885158	292.239554	542.181155	137.110227	233.065286	119.683274	291.233639
EIF2AK1P1	0	0	0	0	0	0	0	0
EIF2AK2	94.864976	107.873271	58.75588	37.831807	113.831892	41.087885	57.470262	123.06745
EIF2AK3	5.463444	6.548849	0	6.611844	2.799953	0.950973	0	8.160962
EIF2AK4	50.516307	106.626328	44.795985	68.56464	32.643753	62.710858	95.589237	70.963661
EIF2AP4	0	0	0	0	0	0	0	0
EIF2B1	0	37.585457	19.964549	28.0863	0	31.614603	29.697105	14.081391
EIF2B2	38.248643	38.817625	20.44507	37.112454	34.72672	44.096721	43.528521	42.01367
EIF2B3	0	6.796862	0	2.44599	9.647188	1.480346	30.614112	6.365739
EIF2B4	8.851259	9.526807	26.366477	18.269481	29.271046	19.964507	70.780518	17.290662
EIF2B5	56.455718	56.230032	24.905518	44.654307	90.757408	52.966307	41.16314	36.646181
EIF2D	20.697834	38.491657	32.341794	37.859403	22.898143	34.664351	16.704551	28.137037
EIF2S1	478.83158	610.680152	268.227449	516.79548	252.505503	547.982105	847.727415	648.937395
EIF2S2	257.075486	529.450291	311.505921	468.452825	226.438539	337.513041	171.836148	344.507035
EIF2S2P1	0	0	0	0	0	0	0	0
EIF2S2P2	0	0	0	0	0	0	0	0
EIF2S2P3	0	0	0	0.185787	1.016972	0	0	0.158268
EIF2S2P4	5.924218	1.364113	0	3.912513	0	0.796693	0	0
EIF2S2P5	0	0	0	0	0	0	0	0
EIF2S2P6	0	0	0	0	0	0	0	0
EIF2S2P7	0	0	0	0	0	0	0	0
EIF2S3	135.777559	60.018608	182.810168	59.39998	210.998862	58.360612	199.016585	89.434098
EIF2S3B	0	0	0	0	0	0	0	0
EIF3A	37.541761	14.90834	34.067357	10.599012	63.13824	4.975388	22.695719	7.086554
EIF3B	493.614905	692.137443	436.524809	780.839347	386.400114	482.355769	443.556787	399.93319
EIF3C	210.351363	654.342418	347.703243	900.103119	716.037369	1279.14035	597.989049	561.964652
EIF3CL	0	0	0	0	0	2.1314	0	0
EIF3D	172.795281	261.129236	220.44633	332.265798	200.034366	262.267501	159.874283	204.798483
EIF3E	0	0	0	0	0	0	0	0
EIF3EP1	0	6.159174	0	2.821995	0	0.408393	0	4.064953
EIF3EP2	0	0	0	0	0	0	0	0
EIF3EP4	0	0	0	0	0	0	0	0
EIF3F	20.816056	5.852626	15.009177	6.022403	9.880706	4.335409	19.537012	9.212752

EIF3FP1	5.516294	0	0	0	0	0	0	0
EIF3FP3	54.930332	16.438175	43.091596	25.369463	84.040478	23.642555	6.773723	23.904682
EIF3G	63.79717	143.925741	106.115121	177.891518	139.660553	193.16887	131.745959	125.401964
EIF3H	132.449453	59.198916	174.026714	80.480416	54.184388	33.524699	8.502354	28.678315
EIF3I	0	0	0	2.738849	0	0	0	0
EIF3IP1	0	0	0	0	0	0	0	0
EIF3J	241.992379	258.3913	128.679449	187.313017	249.663312	175.642676	152.204569	369.936962
EIF3JP1	0	0	0	0	0	0	0	0
EIF3JP2	0	0	0	0	0	0	0	0
EIF3JP3	0	0	0	0	0	0	0	0
EIF3K	83.451337	14.531575	72.327526	21.667568	147.266758	122.73003	212.800635	35.469671
EIF3KP1	0	0	0	0.315027	0	0	0	0
EIF3KP2	0	0	0	0	0	0	0	0
EIF3KP3	0	0	0	0	0	0	0	0
EIF3L	190.622511	281.379555	503.943463	265.997936	157.512805	117.995586	118.721701	167.491711
EIF3LP1	0	0	0	0	0	0	0	0
EIF3LP2	0	0	0	0	0	0	0	0
EIF3LP3	0	0	0	0	0	0	0	0
EIF3M	246.924624	248.936171	410.377987	219.500319	464.754442	207.732614	688.735036	459.91722
EIF3MP1	0	0	0	0	0	0	0	0
EIF4A1	710.71761	522.671158	719.959745	709.793135	1004.77445	697.923045	775.212417	752.882857
EIF4A1P1	0	0	0	0	0	0	0	0
EIF4A1P10	0	0	2.736092	1.35624	0	1.225661	0	0
EIF4A1P11	0	0	0	0	0	0	0	0
EIF4A1P12	0	0	0	0	0	0	0	0
EIF4A1P13	0	0	0	0	0	0	0	0
EIF4A1P2	0	0	0	0.076028	0	0	0	0
EIF4A1P3	0	0	0	0	0	0	0	0
EIF4A1P4	0	0.128239	0	0.295479	0	0.149504	0	0
EIF4A1P5	0	0	0	0	0	0	0	0
EIF4A1P6	0	0	0	0	0	0	0	0
EIF4A1P7	0	0	0	0	0	0	0	0
EIF4A1P8	0	0	0	0	0	0	0	0

EIF4A1P9	0	0	0	0	0	0	0	0
EIF4A2	625.857996	204.456507	907.612878	188.710115	410.600923	67.812184	346.779366	110.751108
EIF4A2P1	0	2.66616	0	1.464135	0	0.173776	0	0.584028
EIF4A2P2	0	0	0	0.085472	0	0	0	0
EIF4A2P3	0	0	0	0	0	0	0	0
EIF4A2P4	0	0	0	0	0	0	0	0
EIF4A2P5	0	0	0	0	0	0	0	0
EIF4A3	125.629133	191.872765	114.853451	191.154428	198.299095	207.307603	138.647651	226.07724
EIF4A3P1	0	0	0	0	0	0	0	0
EIF4B	320.091986	361.900681	461.585323	570.944408	165.242712	167.420625	66.061257	90.453303
EIF4BP1	0	0	0	0	0	0	0	0
EIF4BP2	0	0	0	0	0	0	0	0
EIF4BP3	0	0	0	0.047506	0	0	0	0
EIF4BP4	0	0	0	0	0	0	0	0
EIF4BP5	0	0	0	0	0	0	0	0
EIF4BP6	6.5831	4.524634	1.960679	8.782375	7.413234	0.670889	0	1.449554
EIF4BP7	0	2.484177	1.872984	3.353985	0	1.24748	0	0
EIF4BP8	0	0	0	0	0	0	0	0
EIF4BP9	0	0	0	0	0	0	0	0
EIF4E	104.392485	92.125438	74.612409	75.66779	63.742612	49.704892	137.3932	59.088667
EIF4E1B	0	0	0	0	0	0.412891	0	0
EIF4E2	95.961482	63.365994	39.504921	48.508332	78.163388	113.072159	122.78427	88.884046
EIF4E2P1	0	0	0	0	0	0	0	0
EIF4E2P2	0	0	0	0	0	0	0	0
EIF4E3	4.552112	5.212471	14.863858	5.765577	40.338649	5.019121	0	5.069677
EIF4EBP1	102.871838	154.189785	154.675193	208.085798	130.95161	103.728305	125.201688	69.813834
EIF4EBP1P1	0	0	0	0	0	0	0	0
EIF4EBP2	25.448787	12.21909	33.365936	8.485796	32.487099	7.751336	30.086775	14.130193
EIF4EBP2P1	0	0	0	0	0	0	0	0
EIF4EBP2P2	0	0	0	0	0	0	0	0
EIF4EBP2P3	0	0	0	0	0	0	0	0
EIF4EBP3	0	1.446806	0	0.962503	0	10.501734	0	0
EIF4ENIF1	10.932441	13.339967	65.782623	31.449341	5.399448	11.618081	208.819845	10.849571

EIF4EP1	0	0	0	0	0	0	0	0
EIF4EP2	88.878339	0	0	7.275009	0	0	0	0
EIF4EP3	0	0	0	0	0	0	0	0
EIF4EP4	0	0	0	0	0	0	0	0
EIF4EP5	0	0	0	0	0	0	0	0
EIF4G1	175.121174	341.127648	175.593951	388.161108	314.189393	568.792764	266.989089	454.547154
EIF4G2	26.450542	240.94049	17.665144	0.771096	5.747232	0.224763	0	8.376684
EIF4G3	18.419686	49.31926	35.203351	71.487876	24.147168	85.102448	86.669357	53.661803
EIF4H	155.468723	125.715006	174.957222	124.35059	179.137387	126.053547	212.628271	114.799964
EIF4HP1	0	1.036175	0	0.264706	0	0	0	0
EIF4HP2	0	0	0	0	0	0	0	0
EIF5	182.347082	773.488789	168.07997	597.224964	176.723808	747.187992	243.108243	912.807733
EIF5A	599.680117	1166.07477	489.434246	974.376089	743.724109	1539.01625	858.097725	1431.1071
EIF5A2	51.37137	41.164158	6.352907	16.682429	36.537447	12.165326	0.287097	43.15932
EIF5A2P1	0	0	0	0	0	0	73.004565	0.479845
EIF5AP2	0	0	0	0	0	0	0.875027	0
EIF5AP3	0	0	0	0	0	0	0	2.3816
EIF5AP4	0	0	0	0.633053	0	0.704908	0	1.936278
EIF5B	5.006639	3.232709	13.364732	6.433049	14.890655	10.768191	14.533208	4.316596
EIF5P1	0	0	0	0	0	0	0	0
EIF5P2	0	0	0	0	0	0	0	0
EIF6	158.750653	166.408498	149.258963	193.068378	162.5369	202.305303	99.817137	188.864508
EIPR1	51.49318	5.677484	0	9.620937	29.022746	5.025109	90.166079	5.404804
ELAC1	4.664007	22.005169	2.121898	13.202337	14.526699	12.069131	0	21.331453
ELAC2	62.545821	59.048979	23.353889	61.79158	50.219526	83.005774	100.550351	97.465609
ELANE	0	0	0	0	0	0	0	0
ELAPOR1	6.240553	2.054644	0.979622	1.227112	0.143972	0.63958	5.890899	0.469443
ELAPOR2	9.427344	19.67784	29.385761	19.828555	9.101379	7.110842	0	2.745465
ELAVL1	73.40316	138.166977	43.210298	114.85778	57.291167	145.460802	28.280338	172.026191
ELAVL2	9.616498	16.833071	9.480438	22.114481	18.230524	1.87447	3.828296	8.410138
ELAVL3	0	0.631024	0	1.030803	0	0	0	0
ELAVL4	0	0	0	0	0	0	0	0
ELF1	24.353919	106.078577	42.554355	117.420192	23.37224	93.928765	34.058456	124.443311

ELF2	21.864013	34.065312	12.397118	33.90528	11.958046	22.818562	45.764315	31.455143
ELF2P1	0	0	0	0.350356	0	0.530018	0	0.297178
ELF2P2	0	0	0	0.216771	0	0	0	0
ELF2P3	0	0	0	0	0	0	0	0
ELF2P4	0	0	0	0	0	0	0	0
ELF3	33.884489	55.934433	117.076404	149.497694	31.97322	47.354429	0	28.89514
ELF4	36.477622	9.349422	0	27.356601	73.862309	29.047554	0	21.903969
ELF5	0	0	0	0	0	0	0	0
ELFN2	0	10.548886	0	1.093058	0	0.920587	0	2.15995
ELK1	7.788183	59.558578	11.526083	76.242434	2.910867	75.844566	36.962802	66.168913
ELK1P1	0	0	0	0	0	0	0	0
ELK2AP	0	0	0	0	0	0	0	0
ELK2BP	0	0	0	0	0	0	0	0
ELK3	54.920026	69.821621	64.79436	84.478575	70.808029	117.663389	133.81266	96.289704
ELK4	0	59.981512	35.450353	54.601829	69.224285	71.109016	0	62.683203
ELL	6.659494	7.756297	6.723451	10.718502	7.549239	15.864179	15.994691	9.563211
ELL2	147.918579	157.385863	142.11885	129.105787	60.53398	60.230108	136.436793	82.9088
ELL2P1	0	0	0	0.045631	0	0	0	0
ELL2P2	0	0	0	0	0	0	0	0
ELL2P3	0	0	0	0	0	0	0	0
ELL2P4	0	0	0	0	0	0	0	0
ELL3	5.964301	6.318951	0	5.004379	1.060235	4.376915	0	4.789455
ELMO1	0	0.553719	0	0.042559	0	0	0	0.558268
ELMO2	16.73398	21.471263	10.00614	28.527607	35.116364	34.236919	66.753889	27.067665
ELMO2P1	0	0	0	0	0	0	0	0
ELMO3	27.050084	5.60297	7.876477	3.805195	19.553201	21.914457	0	7.880299
ELMOD1	3.057693	11.238126	2.052485	12.372984	0	0.660913	0	0.748852
ELMOD2	15.719704	28.610865	6.713464	9.51203	21.395287	4.80073	27.097256	11.570811
ELMOD3	3.073854	19.529037	6.861574	20.681595	0.408996	12.077429	16.988984	16.481852
ELN	0	0	1.120074	0.26868	0	0	0	0.151056
ELOA	0	0	0	0	0	0	0	2.864482
ELOAP1	0	0	0	0	0	0	0	0
ELOB	64.129025	249.277737	270.831317	207.533085	0	253.827427	230.31773	175.365672

ELOBP1	0	0	0	0	0	0	0	0
ELOBP2	0	0	0	0	0	0	0	0
ELOBP3	0	0	0	0	0	0	0	0
ELOBP4	0	0	0	0	0	0	0	0
ELOC	94.253139	150.551737	59.667698	100.934831	82.109549	47.628193	177.558599	73.547634
ELOCP11	0	0	0	0	0	0	0	0
ELOCP13	0	0	0	0	0	0	0	0
ELOCP15	0	0	0	0	0	0	0	0
ELOCP17	0	0	0	0	0	0	0	0
ELOCP18	0	0	0	0	0	0	0	0
ELOCP19	0	0	0	0.974377	0	0	0	0
ELOCP2	0	0	0	0	0	0	0	0
ELOCP20	0	0	0	0	0	0	0	0
ELOCP21	0	0	0	0	0	0	0	0
ELOCP22	0	0	0	0	0	0	0	0
ELOCP23	0	0	0	0	0	0	0	0
ELOCP24	0	0	0	0	0	0	0	0
ELOCP27	0	0	0	0	0	0	0	0
ELOCP28	0	0	0	0	0	0	0	0
ELOCP29	0	0	0	0	0	0	0	0
ELOCP3	0	0	0	0	0	0	0	0
ELOCP30	0	0	0	0	0	0	0	0
ELOCP31	0	0	0	0	0	0	0	0
ELOCP32	0	0	0	0	0	0	0	0
ELOCP33	0	0	0	0	0	0	0	0
ELOCP34	0	0	0	0	0	0	0	0
ELOCP35	0	0	0	0	0	0	0	0
ELOCP36	0	0	0	0	0	0	0	0
ELOCP4	0	0	0	0	0	0	0	0
ELOCP5	0	0	0	0	0	0	0	0
ELOCP6	0	0	0	0	0	0	0	0
ELOCP7	0	0	0	0	0	0	0	0
ELOCP9	0	0	0	0	0	0	0	0

ELOF1	0	5.613267	18.91145	4.956079	0	15.536646	0	7.64986
ELOVL1	43.734332	60.665617	67.751601	59.800769	177.95504	80.67469	89.533913	79.239968
ELOVL2	0	0	0	0	0	0	0	0
ELOVL3	0	0.650722	0	1.127149	0	0	0	0
ELOVL4	4.45598	5.370923	6.986912	2.117782	0.588051	2.386276	0	4.121948
ELOVL5	75.097226	56.705631	82.907	49.513036	78.317608	35.457794	48.621305	50.35665
ELOVL6	7.045478	27.087936	3.990384	10.110656	42.393521	7.497487	0.643196	12.653181
ELOVL7	2.595327	2.884358	19.208227	4.196354	9.366057	4.878254	25.976007	14.109139
ELP1	48.437366	53.078292	23.241415	51.216546	29.07061	43.68389	129.702088	48.658925
ELP2	82.390447	52.484562	42.006932	36.572082	89.670147	33.259172	40.462805	57.452658
ELP3	46.478615	25.034903	61.285551	29.522153	25.111667	12.823377	71.892949	18.965162
ELP4	11.833791	7.593037	29.92808	6.89053	29.049386	10.288492	16.547112	15.953645
ELP5	0	3.862933	0	3.187341	0	3.328709	0	6.761679
ELP6	42.980448	45.709095	15.173003	38.318054	19.878068	35.416642	0	47.803532
ELSPBP1	0	0	0	0	0	0	0	0
EMB	92.218066	77.576324	81.90852	19.718906	91.047868	12.65387	57.56734	51.239
EMBP1	0	0	0	0	0	0	0	0
EMC1	18.064308	11.070562	36.66022	18.176407	36.169465	14.938074	20.209294	10.892563
EMC10	24.107895	21.454072	28.698653	22.560425	31.870524	40.894143	63.226762	32.160403
EMC2	75.765553	69.168303	64.31867	65.967544	61.861746	14.078187	3.149453	38.511352
EMC3	0	0	0	1.756172	0	2.893819	0	0
EMC3-AS1	11.705435	0	0	0.418967	0	0	0	0.375995
EMC4	166.493797	199.231504	114.47751	155.54345	18.82805	143.433494	161.098115	147.614071
EMC6	33.345995	71.263653	28.586262	70.975703	114.967293	106.508646	1.571515	139.006435
EMC7	103.236854	70.343301	56.627385	46.199066	43.026407	24.297853	64.838482	37.822209
EMC8	64.645171	124.288353	42.62163	97.10338	20.511672	182.586064	73.196995	159.441371
EMC9	9.63264	51.685593	27.012027	51.589528	75.74752	105.391917	15.923698	110.420698
EMCN	0	0	0	0	0	0	0	0
EMD	0	46.175472	53.964628	66.861507	0	63.54303	0	54.315911
EME1	4.476334	5.174729	1.334057	2.689426	3.541946	2.079944	0	7.971869
EME2	1.488169	2.793599	0	4.211439	19.321657	7.894606	3.486701	2.964359
EMG1	59.007246	28.714267	27.408631	39.766616	43.695077	31.27038	58.103645	27.301587
EMID1	0	1.85104	5.822729	1.933327	0	2.046676	0	0.581117

EMILIN1	2.526801	0.361132	0	0.329128	0	1.246995	0	1.454761
EMILIN2	1.094348	13.212631	2.061862	10.708322	18.140827	21.711421	11.182299	21.043926
EMILIN3	0	0.224385	0	0.106625	0	0.349144	0	0.315879
EML1	9.181964	11.026297	12.872479	8.213016	0	13.211821	0	27.346587
EML2	15.954533	18.01094	18.981204	9.323806	7.23077	3.919087	8.898487	3.144923
EML3	8.132631	16.096233	19.404798	7.850303	24.44292	13.482997	43.624069	11.02617
EML4	59.933324	80.311513	45.955849	63.475687	59.364455	75.373013	69.50832	75.255531
EML5	3.302535	3.84505	3.958052	2.337218	2.776485	0.9994	0	3.398788
EML6	0	0	13.314292	0.506018	0	0.159965	0	1.081035
EMP1	82.307276	91.415822	61.501923	135.564245	233.523501	142.260978	179.239022	141.166557
EMP2	40.414413	59.713903	38.613631	67.277984	42.176149	33.886597	17.971012	37.102455
EMP2P1	0	0	0	0	0	0	0	0
EMP3	27.07763	36.559246	21.600229	45.553645	36.379455	32.419831	73.368735	41.179498
EMSY	28.025872	42.482888	21.492169	48.444178	25.133694	35.438274	23.582102	30.126676
EMX1	0	1.611789	0	1.617519	0	0	0	0
EMX2	4.501475	1.703138	0	0.897023	3.011557	0	0	0
EN1	0	0	0	0	0	0	0	0
EN2	0	0.713142	0.880063	1.388505	0	0.293795	0	0.039447
ENAH	102.609176	263.294482	92.055794	263.453052	147.42383	249.708902	85.925597	166.360775
ENAHP1	0	0	0	0	0	0	0	0
ENAM	0	0	0	0	0	0	0	0
ENC1	2.580589	22.115008	21.811961	15.361533	15.950986	26.312789	29.333048	31.039143
ENDOD1	22.577349	39.249102	18.375232	22.595708	7.268051	15.111683	9.621283	17.968151
ENDOG	13.113913	15.913802	2.901845	17.835323	10.342351	27.271421	13.377747	19.334514
ENDOU	0	0	0	0	0	0	0	0
ENDOV	10.933341	57.752753	30.48808	48.69145	6.018832	39.38473	4.447923	30.832564
ENG	0	0.381354	0	0	0	1.836752	0	0
ENGASE	0.755345	0.856062	0	0.854797	1.333552	0.373452	0	0.397149
ENHO	0	1.385717	0	0.627337	0	1.987572	0	1.82612
ENKD1	79.118098	21.275752	22.340133	47.67158	20.666673	20.870665	0.708759	22.227147
ENKUR	1.013355	1.071854	0	0.250646	0	0.050538	0	0.50108
ENO1	1235.93688	1141.10949	1167.6618	1212.55345	2570.41095	2373.91022	2689.88123	2257.70433
ENO1P1	0	0	0	4.127507	0	0	0	0

ENO1P2	0	0	0	0	0	0	0	0
ENO1P3	0	0	0	0	0	0	0	0.492229
ENO1P4	1.968237	0.599183	0	1.272725	0	0	0	0
ENO2	31.364033	26.475222	43.280837	41.697788	53.351343	38.447609	9.272386	28.801524
ENO3	0	2.444263	0	2.19317	0	2.467354	0	0.818324
ENO4	0	0	0	0	0	0	0	0
ENOPH1	49.783121	66.616923	39.181112	50.953807	49.702633	56.691193	22.910061	65.472255
ENOPH1P1	0	0	0	0	0	0	0	0
ENOSF1	15.28295	20.086279	7.078663	15.203066	8.625766	18.891914	71.643862	25.766683
ENOX1	0	0.48049	0	1.069286	0	0.224407	0	0.543636
ENOX2	9.619166	52.283333	23.681646	40.769121	2.330556	37.40102	14.369703	36.822207
ENPEP	5.322807	2.60033	0.424482	0.473449	0	0	0	0
ENPP1	0	2.311702	0.966233	0.339102	0	0	0	0.06772
ENPP2	0	0.263573	0	0	0	0	0	0
ENPP3	0	0	0	0	0	0	0	0.377302
ENPP4	2.119948	1.242809	3.173096	1.05308	6.906137	0.318264	4.818161	1.786764
ENPP5	2.582568	1.184202	0.77243	1.082869	0	0.066358	0	1.236647
ENPP6	1.720303	2.945792	5.402602	3.485326	0	0.601589	0	0.483691
ENPP7	0	0	0	0	0	0	0	0
ENPP7P10	0	0	0	0	0	0	0	0
ENPP7P11	0	0	0	0	0	0	0	0
ENPP7P13	0	0	0	0	0	0	0	0
ENPP7P14	0	0	0	0	0	0	0	0
ENPP7P15	0	0	0	0	0	0	0	0
ENPP7P2	0	0	0	0	0	0	0	0
ENPP7P3	0	0	0	0.841242	0	0	0	0
ENPP7P4	0	0	0	0	0	0	0	0
ENPP7P5	0	0	0	0	0	0	0	0
ENPP7P6	0	0	0	0	0	0	0	0
ENPP7P7	0	0	0	0.33016	0	0.166671	0	0
ENPP7P8	0	0	0	0	0	0	0	0
ENPP7P9	0	0	0	0	0	0	0	0
ENSA	133.489991	215.348855	100.612491	245.568654	24.13248	373.701596	0	202.631495

ENSAP1	0	0	0	0	0	0	0	0
ENSAP2	0	0	0	0	0	0	0	0
ENSAP3	0	0	0	0	0	0	0	0
ENTHD1	0	0	0	0	0	0	0	0
ENTPD1	0.25606	0.198894	0.23055	0.09408	0.067691	0.154545	0.157967	0.082026
ENTPD2	4.788715	1.004697	8.519396	3.817102	0	2.639192	0	0.096937
ENTPD3	0	2.395652	0	6.941408	0	2.327297	0	1.126661
ENTPD4	11.778968	16.583678	16.38031	24.691938	65.828384	20.731445	3.554168	18.996801
ENTPD5	0	11.107773	14.08676	9.180797	8.709124	8.165081	11.465054	10.150449
ENTPD6	17.862336	63.350969	37.318682	65.275352	61.739457	62.960709	65.934507	72.45214
ENTPD7	8.290217	6.169336	15.596448	6.565251	21.677578	4.258626	4.688584	6.74899
ENTPD8	0	0	0	0	0	0	0	0
ENTR1	44.264757	193.920586	91.577294	168.245151	36.636275	219.676058	150.646391	209.411646
ENTR1P1	0	0	0	0	0	0	0	0
ENTR1P2	0	0	0	0	0	0	0	0
ENTREP1	0	0	0	0.041002	0	0.384417	0	0
ENTREP2	0	0.330616	0	0.20505	0	0	0	0
ENTREP3	18.871367	24.352998	23.076454	29.107679	20.953927	43.523371	21.864667	29.514499
ENY2	191.538816	193.4335	130.965096	191.849948	156.30787	161.289015	98.039268	184.819268
EOGT	7.39996	26.305497	14.392756	32.86488	22.76257	24.771414	33.827392	27.162657
EOLA1	13.209639	30.414719	13.411269	35.560586	35.303423	28.826426	51.01474	29.549221
EOLA2	41.061065	82.748422	23.714732	89.602732	2.1367	83.29366	11.727443	82.531628
EOMES	0	1.475432	0	0.06383	0	0	0	0.335177
EP300	38.430323	55.340747	21.777377	50.076148	38.963761	58.827675	75.877482	71.637088
EP400	5.592183	5.889391	1.63453	7.215786	18.120289	6.346472	26.559818	5.034302
EP400P1	0	0	0	0.224199	0	0	0	0.071864
EPAS1	71.009616	177.916902	62.08894	243.822617	78.942654	307.28643	57.456532	157.314682
EPB41	27.244564	21.290425	17.982174	28.875189	27.47723	36.783972	3.668453	36.764981
EPB41L1	36.687918	123.776941	84.948125	168.174191	127.289165	208.937566	40.893089	112.356288
EPB41L2	51.794584	40.272723	25.764904	62.258065	72.880044	82.251791	63.015498	41.784383
EPB41L3	0	0	0	0.400567	0	1.642836	0	2.798345
EPB41L4A	9.984563	5.275415	7.144491	0	0	2.519323	3.592762	0.451142
EPB41L4B	4.94758	5.4388	7.400473	4.276816	7.621567	4.712616	0	7.551515

EPB41L5	14.236413	14.221293	14.701842	12.846735	6.819163	9.616206	4.271243	17.889633
EPB42	0	0	0	0	0	0	0	0
EPC1	6.164703	7.159571	3.796118	3.494717	0.22342	2.869549	0	7.081598
EPC2	9.372148	19.559004	11.468005	15.350399	14.174233	14.089984	5.597233	19.571954
EPCAM	118.287246	195.948525	84.87014	106.971687	64.051044	59.069005	220.20912	85.279936
EPCIP	0	0	0	0	0	0	0	0
EPDR1	18.927302	23.518863	13.306729	27.328121	16.050329	60.618965	26.547167	59.963859
EPG5	14.610961	15.214396	4.16111	11.393274	8.349338	11.013563	15.263941	15.047471
EPGN	0	0	0	0	33.76523	1.319418	105.122218	0
EPHA1	16.719136	6.926731	4.519267	5.230387	27.306276	5.847279	14.334464	5.317239
EPHA10	0	0	0	1.052381	0	0.328183	0	0.191077
EPHA2	42.725087	58.79753	60.143426	80.495056	61.058469	142.703121	103.697482	107.044908
EPHA3	1.33421	2.143712	0.512419	1.939317	0	0	0	0
EPHA4	1.020309	1.239293	0	0.374322	0	1.939968	0	2.15905
EPHA5	0	0	0	0	0	0	0	0
EPHA6	0	0.997024	0	2.243031	0	0	0	0.143513
EPHA7	0	0.042127	0	0.071861	0	0	0	0
EPHA8	0	0	0	0.094479	0	0	0	0
EPHB1	0	0.742162	0	0.120041	0	0.414597	0	1.001536
EPHB2	5.664915	20.831898	17.714083	33.01404	13.275367	55.729758	20.410447	46.372168
EPHB3	0	0.566938	0	1.564176	0.616557	7.436576	0	3.436802
EPHB4	25.649997	31.176108	24.315394	27.360584	27.448612	47.560557	21.139007	30.118649
EPHB6	15.07351	32.811308	0	24.155541	2.784018	5.487449	0	12.633835
EPHX1	0	1.921323	0	1.475285	29.602349	0.618693	0	4.167828
EPHX2	8.876689	7.584102	7.253313	2.684527	0	4.640384	0	1.501466
EPHX3	0	0	0	0	0	0	0	0
EPHX4	10.066854	12.547042	6.711438	4.867634	15.900874	0.740682	0	3.058657
EPM2A	7.851042	10.519358	2.554898	4.039055	11.630946	8.198875	9.93014	15.606723
EPM2AIP1	0	2.333355	13.985961	1.656394	0	2.294152	0	3.492422
EPN1	42.887653	59.052307	82.673372	61.088865	85.508087	175.144494	42.828211	139.377365
EPN2	23.524883	128.78084	18.32213	157.501223	56.912318	178.502254	51.269684	165.707464
EPN3	11.05011	25.079006	1.530714	19.696129	5.265816	40.640479	0	28.085281
EPO	2.13199	0	0	0.309949	0	0	0	0

EPOP	8.915366	51.14959	11.997287	44.754854	9.887107	77.438521	10.193033	66.192792
EPOR	0	6.96845	3.21975	10.276783	0	12.036889	0	4.211777
EPPIN	0	0	0	0	0	0	0	0
EPPIN-WFDC	0	0	0	0	0	0	0	0
EPPK1	1.232318	0	1.10987	0.746234	5.212855	10.466141	2.808724	0
EPRS1	125.831776	87.153825	94.405123	89.036974	78.838832	37.774525	162.845377	57.231225
EPS15	91.784392	164.95013	88.718983	151.672851	39.039697	43.719531	13.156971	82.085118
EPS15L1	9.570381	11.039483	14.9073	19.152142	43.043652	28.980526	19.612097	21.174261
EPS15P1	0	0	0	0	0	0	0	0
EPS8	34.766583	108.145597	27.294674	80.975321	30.710513	30.783961	55.633666	41.81439
EPS8L1	3.129404	2.520679	25.689773	4.300423	16.311919	4.484289	70.368406	2.765412
EPS8L2	37.992661	34.774797	24.805163	44.679234	111.824528	175.450694	169.020808	99.277095
EPS8L3	0	0	0	0	0	0	0	0
EPSTI1	0	8.025297	7.180469	5.641912	0	6.406817	0	8.371526
EPX	0	0	0	0	0	0	0	0
EPYC	0	0	0	0	0	0	0	0
EQTN	0	1.121126	0	0	0	0	0	0
ERAL1	47.543357	68.453124	49.194289	71.029684	40.268355	68.618399	54.012495	57.978396
ERAP1	13.535188	19.951386	69.451482	28.209945	37.260709	10.893814	168.594601	9.314513
ERAP2	0	15.599572	6.284589	34.914544	19.152505	10.041754	28.00323	19.525751
ERAS	0	0	0	0	0	0	0	0
ERBB2	15.591505	26.022107	21.585103	25.938896	100.66826	47.840267	29.926322	17.039994
ERBB3	0	6.700401	52.685098	18.595436	7.561772	25.578196	20.92453	4.103978
ERBB4	0	0.011416	0	0.116439	0	0	0	0
ERBIN	81.40493	149.076983	76.989946	135.487375	42.981793	113.760062	8.999094	112.091299
ERC1	22.140973	20.328765	53.022508	34.658051	63.348149	43.41119	20.373729	39.807751
ERC2	0	4.625808	0	5.882192	0	3.249034	0	1.374414
ERCC1	101.848131	74.71538	36.413428	77.644727	87.751006	129.409115	100.480804	109.477022
ERCC2	4.83277	9.82819	0	8.932757	18.751239	34.13888	19.970487	19.878607
ERCC3	2.765256	11.030133	0	20.768053	51.549696	18.889857	84.569533	14.957413
ERCC4	6.061624	10.211377	6.012684	6.178511	57.689428	9.621818	0	7.742604
ERCC5	24.934812	5.47277	20.317905	16.748397	24.276639	4.2057	0	11.807536
ERCC6	33.03873	16.621472	39.117834	15.224794	5.403674	29.30349	23.678731	21.302924

ERCC6L	15.625316	13.023788	9.117316	8.562645	14.443297	8.098114	20.918376	17.990683
ERCC6L2	8.848004	17.129441	11.80454	8.955779	15.126814	6.996633	0.570262	14.709702
ERCC8	0	8.136358	0	10.601528	4.790944	14.090032	0	5.884898
EREG	23.886179	10.885189	31.937769	23.27293	9.393612	3.473012	15.587889	8.616996
ERF	15.199824	66.050174	98.231886	66.100735	44.785909	200.685043	77.899807	133.926542
ERFE	0	0.390301	0	2.976285	2.019713	3.393073	0	0.427681
ERG	0	1.593432	0	0.41206	0	2.209206	0	2.376558
ERGIC1	81.13393	76.772634	78.906184	75.475125	68.353006	45.599531	34.730566	47.320702
ERGIC2	136.254016	169.689731	102.832219	98.691726	36.350547	43.935641	265.093734	129.019358
ERGIC3	20.553563	33.573254	19.302805	25.66127	105.311246	0.579436	102.752876	19.599494
ERH	467.007624	313.817155	340.772803	210.823379	350.643447	133.579218	222.485944	247.032895
ERHP1	0	0	0	0	0	0	0	0
ERHP2	0	0	0	0	0	0	0	0
ERI1	0	16.904219	17.417311	8.525444	20.451351	8.467847	0	20.333038
ERI2	28.119477	47.263065	27.978024	38.459981	48.493703	36.926549	10.16308	65.196122
ERI3	0	7.502365	0	10.172054	30.244004	30.473153	61.007581	17.604866
ERICH1	34.384222	14.031407	12.187275	16.424642	8.498751	13.81235	70.984925	8.217697
ERICH2	0	0.296986	6.522711	0.709442	0	0	0	0.941283
ERICH3	0	0	0	0.081271	0	0	0	0
ERICH5	4.777827	8.019868	4.250143	12.974162	3.774245	5.328557	0.576257	5.319263
ERICH6	0	0.169181	0	0	0	0	0	0.405218
ERICH6B	0	0	0	0	0	0	0	0
ERLEC1	78.56857	113.465528	67.71284	76.316671	27.931822	35.185035	31.013028	56.219505
ERLEC1P1	0	0	0	0	0	0	0	0
ERLIN1	66.683277	72.38016	74.660931	82.643194	79.719952	57.655894	26.450538	71.904924
ERLIN2	0	15.29342	0	29.793702	7.64705	13.650342	32.258357	17.745343
ERMAP	6.284185	3.859841	5.632708	5.021346	2.696436	3.720716	3.054668	4.909629
ERMARD	3.803469	8.46987	8.481774	3.80263	30.065202	1.751523	10.579812	12.326051
ERMN	0	0	0	0	0	0	0	0
ERMP1	12.745163	8.406323	39.811133	15.771469	21.393912	7.798826	16.118193	7.430819
ERN1	3.845834	9.360637	8.824735	18.469214	2.052426	3.957915	0.0997	2.285764
ERN2	0	0	0	0	0	0.052629	0	0
ERO1A	155.444968	275.181783	210.071604	281.744463	178.869076	210.453417	163.295587	322.576403

ERO1B	3.17287	0.937697	0	2.676182	0	2.764429	0	0
ERP27	11.559091	17.084182	10.304801	7.938176	4.261789	6.315433	0.557901	9.425203
ERP29	72.261913	63.131209	95.033	97.843244	127.475276	95.025177	22.738599	61.36945
ERP29P1	0	0	0	0	0	0	0	0
ERP44	28.626729	23.74586	34.895412	19.063146	23.765448	8.702191	6.307928	11.245659
ERRFI1	44.500668	169.288522	48.271454	198.463005	36.543206	130.315008	79.779262	253.33904
ERV3-1	1.042859	4.535867	28.783403	1.487503	0	2.983248	12.36504	0.586984
ERVFRD-1	0	0	0	0	0	0	0	0
ERVFRD-3	0	0	0	0	0	0	0	0
ERVK3-1	36.582077	34.898788	0	35.500246	32.19268	83.37185	32.110321	43.550665
ERVMER34-1	12.31033	24.322895	9.890073	23.289623	12.993843	16.970857	0	16.444032
ERVW-1	0	0.127534	0	0	0	0	0	0
ESAM	0	1.941139	1.708593	0.650621	3.02911	0.378338	0	0.309451
ESCO1	7.322598	21.930487	11.835417	9.590622	12.184694	8.448773	7.666377	13.829542
ESCO2	58.904694	38.279289	0	11.721341	0	18.536982	0	52.427935
ESD	103.384385	117.173371	142.787511	79.43683	66.478348	40.549864	79.113689	76.687761
ESDP1	0	0	0	0	0	0	0	0
ESF1	36.379706	9.010104	27.019481	9.144763	30.181841	10.416372	23.747807	15.819452
ESM1	0	3.657259	0	2.181797	0	1.954574	11.178271	1.48349
ESPL1	0	2.228727	1.369489	0.698621	8.870626	2.553098	3.934905	0.386737
ESPN	6.599403	5.34837	15.830775	5.614881	0.633342	23.558834	16.143381	14.249666
ESPNL	0	0.123831	0	0.561139	0	0	0	0
ESR1	0	0.220197	0	0.04995	0	0.484801	0	2.230716
ESR2	0	0.310637	0	0.459496	0	0.512743	0	0.071913
ESRP1	49.325706	86.347729	107.213576	83.115276	29.733298	47.816402	44.130128	58.386713
ESRP2	24.77699	13.639153	30.56289	18.731595	18.974132	30.262418	23.852536	12.349887
ESRRA	0	29.727412	0	25.737149	22.063768	65.467003	0	52.487991
ESRRAP1	0	0	0	0	0	0	0	0
ESRRAP2	0	0	0	0	0	0	0	0
ESRRB	0	0	0	0	0	0	0	0
ESRRG	2.461678	0.215973	0	1.644466	0	0	0	0
ESS2	12.02601	12.326041	4.745731	14.502519	12.259088	16.420159	41.292683	14.013091
ESX1	0	0.907568	0	0.755895	0	0	0	0

ESYT1	5.341405	11.476744	16.94621	14.252603	55.959838	13.232499	33.681938	4.118731
ESYT2	16.027142	26.781751	20.254952	7.145829	57.582423	4.397615	0.880752	6.096306
ESYT3	0	0.58059	0	1.042098	2.324838	0.892878	0	0.753825
ETAA1	1.274695	0.628171	1.270134	1.256502	0	0.154361	4.424777	0.409579
ETDA	0	0	0	0	0	0	0	0
ETDB	0	0	0	0	0	0	0	0
ETDC	0	0	0	0	0	0	0	0
ETF1	190.225148	250.491006	143.407937	183.773246	140.085658	231.489903	211.993796	303.558761
ETF1P1	0	0	0	0	0	0	0	0
ETF1P2	0	0	0	0	0	0	0	0
ETF1P3	0	0	0	0	0	0	0	0
ETFA	142.644094	208.205143	191.921294	210.743459	45.482374	133.412336	95.213072	165.127275
ETFB	0	0.152862	0	0	0	0.111645	0	0.041987
ETFBKMT	0	1.747501	3.152946	1.437145	0	0.463817	0	0.612286
ETFDH	4.008044	18.390305	3.533147	22.379956	8.331846	8.795089	26.447633	16.22304
ETFRF1	17.748285	3.09647	0	4.352105	0	2.038027	0	5.574213
ETFRF1P1	0	0	0	0	0	0	0	0
ETHE1	12.840534	20.220124	37.671134	17.620537	11.581852	32.417607	22.476215	39.26692
ETNK1	16.546939	13.516408	0	9.291625	25.694175	0	44.41131	8.8674
ETNK2	11.004404	14.633783	14.767863	13.219969	6.53192	6.018417	0	3.711653
ETNPPL	0	0	0	0	0	0	0	0
ETS1	64.528374	79.798744	73.26388	55.15724	72.447762	22.413909	43.170259	76.616982
ETS2	84.654469	119.248356	72.775096	167.199969	123.692429	243.527991	122.818423	258.440387
ETS2P1	0	0	0	0	0	0	0	0
ETV1	0	16.074584	2.643465	19.807757	0.445259	3.84199	0	4.210029
ETV2	0	1.385345	0	0.823368	0	2.30464	0	1.413733
ETV3	22.379885	17.252419	19.441942	18.444656	13.882366	13.207952	23.195339	14.366604
ETV3L	0	0	0	0	0	0	0	0
ETV4	32.106912	48.351514	32.751853	54.168901	8.115129	33.780094	1.025726	24.954508
ETV5	36.096092	84.572343	36.284314	90.114208	13.451771	25.598031	95.525695	30.972779
ETV6	0	2.223114	0	0.527342	17.320156	3.66842	32.803956	0.238685
ETV7	0	5.433097	2.240673	6.46898	0.752889	9.091476	0	5.579594
EVA1A	79.324046	159.446786	42.035271	135.036838	1.593697	122.44159	62.52438	126.385846

EVA1B	0	2.243716	0	2.156972	0.951369	4.568562	0	5.352967
EVA1C	26.226898	10.143265	10.250041	7.476175	29.264304	6.898903	20.008497	8.364354
EVA1CP1	0	0	0	0	0	0	0	0
EVA1CP2	0	2.457787	0	2.753011	0	0.832251	0	0
EVA1CP3	0	0	0	0	0	0	0	0
EVA1CP4	0	0	0	0	0	0	0	0
EVA1CP5	0	0	0	0	0	0	0	0
EVA1CP6	0	0	0	0	0	0	0	0
EVC	9.592026	8.294595	8.170285	12.203179	6.936685	20.883354	2.953807	10.896728
EVC2	0.809144	1.834563	2.178728	3.373871	1.923159	2.703668	4.529202	1.172858
EVI2A	0	0	0	0	0	0	0	0
EVI2B	0	0	0	0.054003	0	0.10973	0	0.090208
EVI5	58.634939	81.371575	18.169433	53.943829	22.31353	26.87175	10.90881	44.772015
EVI5L	1.717023	9.067556	15.496271	6.957122	1.360206	17.122998	0	9.000108
EVL	27.758515	40.06172	32.166491	54.96592	29.832772	96.290319	48.383892	62.815749
EVPL	0	0	5.161104	0.217626	0	0.770357	54.677569	0
EVPLL	0	1.467983	0	0.21315	0	0.434192	11.899544	0.425067
EVX1	0	0.251212	0	0.258425	0	0.106925	0	0.142249
EWSR1	152.038026	88.943901	155.477248	125.349038	130.09172	168.065841	254.949071	100.688365
EXD1	0	0	0	0	0	0	0	0
EXD2	20.546651	29.685638	16.469935	37.287163	25.564966	24.926458	45.157636	33.019606
EXD3	8.5475	10.193674	7.573246	10.425622	4.146708	15.849082	0	11.408703
EXO1	33.888623	113.086354	17.311793	77.019455	41.937867	100.028467	59.070384	121.477759
EXO5	7.379852	8.159019	0	9.060537	0.486298	9.438336	0	11.274814
EXOC1	16.457057	27.638416	38.236303	31.478343	11.402893	12.067767	13.250709	26.224163
EXOC1L	0	0	0	0	0	0	0	0
EXOC2	14.017143	22.751424	10.598837	20.286113	16.757484	19.242802	16.524822	26.732324
EXOC3	18.923733	48.16426	17.330857	53.095262	24.889262	62.584192	42.262584	55.428261
EXOC3L1	0	1.025471	0	0.12047	0	0.241276	0	0.639781
EXOC3L4	0	0	0	0.716125	0	0.922594	0	0
EXOC4	32.624572	31.790114	46.475269	39.722931	51.187831	32.735743	59.174961	23.961663
EXOC5	94.955956	142.757891	57.725008	119.210389	181.256055	96.202953	91.922105	190.20965
EXOC5P1	0	0	0	0	0	0	0	0

EXOC6	25.701862	6.819141	30.735923	6.032597	7.308454	1.432398	6.133669	5.581473
EXOC6B	0.755417	21.204289	15.821737	14.269147	13.784864	10.123953	54.240293	31.304964
EXOC7	46.032014	63.729422	52.179006	70.173691	31.231586	91.676552	4.957632	62.622594
EXOC7P1	0	0	0	0	0	0	0	0
EXOC8	8.9755	9.881378	8.063614	8.99512	12.193976	8.219198	8.433432	8.719806
EXOG	4.358923	6.241878	9.895825	2.763498	3.739246	3.641876	0.707458	4.809127
EXOGP1	0	0	0	0	0	0	0	0
EXOSC1	43.082914	5.567943	33.623999	4.210649	29.839858	8.54631	0	0.06632
EXOSC10	32.576291	29.892891	37.816552	33.634789	42.346459	52.999549	117.285473	47.06751
EXOSC2	49.006148	93.118367	55.570388	95.77275	105.444809	63.819503	41.563698	70.736137
EXOSC3	19.344141	91.982958	66.586291	60.868115	20.391655	64.965901	181.444663	87.817942
EXOSC3P1	0	0.380342	0	0.926672	0	0	0	0
EXOSC3P2	0	0	0	0	0	0	0	0
EXOSC4	28.722366	25.228243	42.012639	31.229215	41.411065	29.850509	0	19.725568
EXOSC5	0	5.896395	23.900107	18.476379	11.572376	24.96155	0	16.693792
EXOSC6	17.090887	11.904576	28.435572	12.702755	11.705437	13.727358	12.953286	14.34854
EXOSC7	22.03118	64.880381	61.197016	69.937624	41.78238	60.013711	39.574144	55.14888
EXOSC8	29.449248	35.970694	19.886515	30.532541	19.168377	18.323597	75.403027	18.058927
EXOSC8P1	0	0	0	0	0	0	0	0
EXOSC9	2.284806	1.613587	0	3.424159	15.3385	14.710912	11.011035	11.21864
EXPH5	4.048776	6.564864	8.029442	5.938087	10.452143	5.132504	3.876497	4.61278
EXT1	68.481466	249.531096	87.600884	234.346885	37.942261	187.617824	0	145.68641
EXT2	30.780266	28.053886	36.672133	58.562588	13.003445	81.335272	35.752838	34.139786
EXTL1	0	0	0	0	0	0	0	0
EXTL2	29.83207	29.987236	8.549525	23.825812	8.502475	8.919703	10.721941	16.786295
EXTL2P1	0	0	0	0	0	0	0	0
EXTL3	0	40.754837	22.737468	25.117959	0	10.805283	0	55.509428
EYA1	0	0.199006	0	0.346475	0	0	0	0
EYA2	46.64496	52.674651	38.800548	57.03966	31.461383	48.730968	24.31745	50.435985
EYA3	14.091968	11.578563	14.855122	9.569844	8.325771	7.582642	35.681033	9.061461
EYA4	0	0	0	0	0	9.184301	0	15.693873
EYS	1.492818	0.426708	0.275411	0.833868	0	0.82658	0	0.060157
EZH1	14.767901	9.296278	6.997999	12.148783	20.318588	14.196402	62.910525	9.8653

EZH2	14.40197	35.954555	17.576949	26.342718	29.808167	23.900885	15.41038	39.856001
EZH2P1	0	0	0	0	0	0	0	0
EZHIP	0	0	0	0.089348	0	0	0	0
EZR	204.385853	259.462939	216.420182	260.27204	236.346795	288.081108	0	268.478806
EZRP1	0	1.603348	0	0	0	5.311843	0	9.249985
F10	0	0	0	0	0	0	0	0
F11	0	0	0	0	0	0	0	0
F11R	53.564911	63.043957	88.428616	109.782807	118.19786	86.750502	54.78301	50.276678
F12	0	4.587242	6.486771	6.34671	0	12.262097	0	8.589408
F13A1	0	0	0	0	0	0	0	0
F13B	0	0	0	0	0	0	0	0
F2	0	0	0	0	0	0	0	0
F2R	0	4.10334	1.596045	2.970109	13.387972	6.181902	15.363396	11.499683
F2RL1	46.474749	69.001607	30.578098	46.493989	79.560812	28.587903	6.290056	52.489623
F2RL2	0	0.167643	0	0.19134	0	0	0	0
F2RL3	0	0	0	0	0	0	0	0
F3	1382.72934	1384.32578	1507.67524	1778.21479	1784.33628	1176.55827	1917.29197	1083.91906
F5	2.758649	2.534263	6.555401	1.978462	0	0.023072	0	0
F7	0	0	0	0.417989	0	0	0	0
F8	5.174744	8.944732	10.421561	5.048477	2.730715	0.450224	0	2.072215
F9	0	0	0	0	0	0	0	0
FA2H	22.627941	17.196308	45.318768	20.265322	1.757568	7.578968	0	2.935229
FAAH	0	2.441729	0	1.318353	0	1.511046	0	0.29788
FAAH2	0	0	12.852385	0	0	0	0	0
FAAHP1	0	0	0	0	0	0	0	0
FAAP100	8.310612	19.159714	13.252094	15.471364	7.710573	27.428652	0	23.708237
FAAP20	6.233622	20.51424	49.490158	17.563609	16.396989	44.840648	27.457159	28.591034
FAAP24	0	13.27291	4.59506	10.840435	0	13.525312	0	7.932834
FABP1	0	0	0	0	0	0	0	0
FABP12	0	0	0	0	0	0	0	0
FABP12P1	0	0	0	0	0	0	0	0
FABP3	35.794311	7.564638	0	0	0	0	0	0
FABP3P2	0	0	0	0	0	0	0	0

FABP4	65.736056	57.866324	64.44096	43.512841	3.707905	0	0	0
FABP5	349.082452	508.882457	312.82527	348.188831	125.417759	93.31119	0	119.133024
FABP5P1	0	0	0	0	0	0	0	0
FABP5P10	0	0	0	0	0	0	0	0
FABP5P11	0	0	0	0	0	0	0	0
FABP5P12	0	0	0	0	0	0	0	0
FABP5P13	0	0	0	0	0	0	0	0
FABP5P14	0	0	0	0	0	0	0	0
FABP5P15	0	0	0	0	0	0	0	0
FABP5P2	0	0	0	0	0	0	0	0
FABP5P3	0	0.674299	0	0.064933	0	0.238741	0	0
FABP5P4	0	0	0	0	0	0	0	0
FABP5P5	0	0	0	0	0	0	0	0
FABP5P6	0	0	0	0	0	0	0	0
FABP5P7	285.980972	553.162513	115.001452	532.747035	0	129.355157	165.546397	165.853681
FABP5P8	0	0	0	0	0	0	0	0
FABP5P9	0	24.18737	23.369549	25.518059	0	1.229466	0	1.803615
FABP6	0	0	0	0	0	0	0	0
FABP7	0	0	0	0	0	0	0	0
FABP7P1	0	0	0	0	0	0	0	0
FABP7P2	0	0	0	0	0	0	0	0
FADS1	8.062294	67.775079	0	28.038609	149.271705	55.163362	98.7436	64.629436
FADS2	28.396054	11.270115	10.156076	14.758199	32.066738	28.129672	14.595545	21.814361
FADS2B	0	0	0	0.120741	0	0	0	0
FADS3	9.106056	26.890338	43.989873	26.194575	44.267265	84.91588	34.246388	68.420124
FADS6	0	0	0	0	0	0	0	0
FAF1	195.976381	296.839381	201.236966	190.879908	16.011168	44.157203	147.047402	114.064568
FAF2	51.293934	38.428408	50.006467	36.478167	44.1277	16.895139	117.011727	24.069212
FAF2P1	0	0	0	0	0	0	0	0
FAH	19.586343	15.931816	23.948809	25.417587	38.982981	17.47371	0	15.108519
FAHD2A	5.95398	56.513023	13.747501	56.560304	21.09376	53.367695	0.642032	31.931167
FAHD2B	0	13.803708	43.450779	25.504212	3.239258	10.76975	0	7.987262
FAHD2CP	4.746908	5.221857	0	4.948315	0	2.181313	0	1.481184

FAHD2P1	0	0	0	0	0	0	0	0
FAIM	34.284159	51.64753	11.949641	27.60156	54.334774	20.304831	0	66.448601
FAIM2	0	0	0	0	0	0	0	0
FAM106A	0	0	0	0.030068	0	0	0	0
FAM106B	0	0	0	0	0	0	0	0
FAM106C	0	0	0	0	0	0	0	0
FAM107A	6.039927	0.862385	0	0.218667	0	0	0	0.135325
FAM107B	16.099798	66.001487	22.136643	61.828742	10.939478	12.822249	0.358212	17.453575
FAM110A	4.407065	31.356856	13.735502	34.764209	8.12596	54.08016	0	33.504246
FAM110B	0	0.165609	0	0.68994	0	0.144977	0	0.311389
FAM110C	14.495984	29.771628	13.772721	23.573229	1.801361	9.842793	65.364631	16.902395
FAM110D	0	0.308147	1.079242	0.146786	0	0	0	0
FAM111A	42.498459	45.607189	37.664322	21.741051	19.303932	12.41665	53.253473	65.041142
FAM111B	39.845112	17.214157	19.568846	7.747708	43.761385	16.583178	74.747604	47.302904
FAM114A1	51.57526	116.193265	63.431625	173.154845	11.506409	35.878164	0	22.895876
FAM114A2	2.959196	25.656806	23.791312	34.993291	3.345368	41.295585	50.089696	34.65557
FAM117A	15.131558	1.468768	1.313707	4.436503	26.70795	4.388056	0	4.746327
FAM117B	2.717939	2.243331	4.397688	2.372868	19.593275	5.521712	15.775934	4.185335
FAM118A	17.635842	24.956845	16.036293	23.946816	13.563502	23.259088	13.208903	28.299413
FAM118B	6.601368	28.733358	9.886589	16.104524	45.831775	29.490791	25.255369	41.90084
FAM120A	122.558	295.193213	172.841277	289.784803	226.806663	373.662742	74.063237	285.481861
FAM120A2P	0	0	0	0	0	0	0	0
FAM120AOS	44.356607	77.23266	36.733594	69.847303	18.654665	71.238786	56.873602	90.960238
FAM120B	12.366425	23.995541	7.804923	20.811487	9.463867	19.299748	28.83835	19.084886
FAM120C	2.402271	2.575045	12.002281	3.964203	0.866341	3.628941	5.426407	3.231782
FAM124A	0	0.347733	0	0.079762	0	0.526897	0	0
FAM124B	0	0	0	0	0	0	0	0
FAM131A	7.59414	39.381847	17.641682	39.820453	8.106998	39.818108	8.200627	26.645443
FAM131B	2.59818	0	0.710804	0.042277	0	0.043263	0	0
FAM131C	3.53593	5.342949	0	4.960121	1.081065	4.171413	0	1.907238
FAM131C2P	0	1.506204	0	1.75933	0	3.648152	0	1.248301
FAM133A	1.059332	0.497535	0.949448	0.608274	0	0	0	0.085187
FAM133B	20.155783	17.807206	0	4.415388	21.908971	16.581787	8.491476	13.015068

FAM133CP	0	0	0	0	0	0	0	0
FAM133DP	0	0	0	0	0	0	0	0
FAM133EP	0	0	0	0	0	0	0	0
FAM133FP	0	0	0	0	0	0	0	0
FAM133GP	0	0	0	0	0	0	0	0
FAM135A	10.683599	13.277993	7.981221	8.382952	45.764375	11.603532	6.205863	10.593138
FAM135B	0	0	0	0	0	0	0	0
FAM136A	0	0.518287	2.931594	2.255291	0	6.825379	0	0.789701
FAM136BP	0	1.479549	0	0.305248	3.425708	0	0	0
FAM136CP	0	0	0	0	0	0	0	0
FAM136DP	0	0	0	0	0	0	0	0
FAM136EP	0	0	0	0	0	0	0	0
FAM136FP	0	0	0	0	0	0	0	0
FAM136GP	0	0	0	0	0	0	0	0
FAM13A	8.365894	25.717852	7.375805	19.823193	13.543326	10.976699	0	13.711746
FAM13B	8.3552	16.928811	11.262341	12.669249	24.751851	4.377513	13.253804	15.49276
FAM13C	0	0	0	0.858956	0	0.163913	0	1.55158
FAM149A	0	1.859304	0	1.115091	2.710462	0.34234	0	0.667862
FAM149B1	26.123178	16.758703	33.881136	19.065732	25.67	13.365648	9.01122	9.701347
FAM149B1P1	0	0	0	0	0	0	0	0
FAM151A	0	0	0	0.060465	0	0	0	0
FAM151AP1	0	0.403209	0	0.278002	0	0.282912	0	0
FAM151B	0	0.80812	0	0.812825	0	0.597194	0	2.57363
FAM153A	0	0.888428	0	0.768519	0	0	0	0
FAM153CP	0	0	0	0	0	0	0	0
FAM153DP	0	0	0	0	0	0	0	0
FAM156A	12.131805	1.217033	4.88945	5.213395	0	1.438765	0	0.960218
FAM156B	4.823325	47.030534	1.418839	42.763074	3.618933	37.942151	18.319613	40.615285
FAM157A	0	2.847193	0	0	0	0	0	0
FAM157B	0	0	0	0	0	0	0	0
FAM161A	3.713186	9.074766	2.360922	8.429909	5.882328	4.886115	11.12782	5.727913
FAM161B	1.849512	2.353097	4.147323	2.92417	4.150784	3.424173	8.321736	3.004179
FAM162A	56.426817	111.263379	57.541559	114.333849	107.034365	144.049337	101.99751	178.919507

FAM162B	0	0	0	0	0	0	0	0
FAM167A	2.434932	1.703635	2.185429	3.133693	0.857375	6.236704	0	4.644162
FAM167B	4.1288	0	3.637131	1.582446	0	0	0	2.361321
FAM168A	23.930708	23.454032	24.349167	25.881064	30.348452	24.638519	26.586574	21.864398
FAM168B	33.611724	50.063211	41.530356	43.156779	55.918053	53.807541	95.436538	73.359199
FAM169A	8.963286	15.746164	9.042572	11.880862	10.402179	13.485401	11.111463	31.101958
FAM169BP	0	0	0	0	0	0	0	0
FAM170A	0	0	0	0	0	0	0	0
FAM171A1	3.1532	11.654549	6.368564	10.289687	0	1.182381	0	0.411471
FAM171A2	4.03886	1.483406	0	0.159152	0	1.069572	0	0
FAM171B	3.409099	5.616003	1.873632	3.931437	0.45035	3.217954	0.138467	4.534773
FAM174A	2.856562	11.319546	10.137573	9.580908	1.471723	5.444299	20.618026	4.753008
FAM174B	0	8.932452	1.165549	2.966266	0	1.179038	0	2.497228
FAM174C	13.008336	33.037809	8.068981	27.598399	2.187896	90.15136	23.010403	70.077207
FAM177A1	53.616231	134.118329	57.507609	127.909079	137.387382	136.023608	60.078102	178.593844
FAM177A1P1	0	0	0	0	0	0	0	0
FAM177B	0	0	0	0	0	0	0	0
FAM178B	0	0	0	0	0	0	0	0
FAM180A	0	0	0	0.225956	0	0.114718	0	0
FAM181A	0	1.987864	1.91205	0.526464	0	0	0	0
FAM181B	2.527347	5.591314	2.267977	4.627134	2.002195	1.894255	0	0.947386
FAM184A	0	0.263948	0	0.226025	0	0.061793	0	0.124212
FAM185A	12.89536	7.772701	2.219674	3.307313	13.146248	6.47813	0	5.622928
FAM185BP	0	0	0	0	0	0.370244	0	0.162041
FAM186A	0	0	0	0	0	0	0	0
FAM186B	1.141841	2.240226	0	1.252136	0	0.341186	0	0.740886
FAM187B2P	0	0.955266	0	0.141132	0	0.562817	0	1.718373
FAM192BP	0	0.455263	0	0	0	0	0	0
FAM193A	7.578896	11.579518	6.072298	6.278073	28.234631	8.955312	7.573505	4.508068
FAM193B	24.72675	10.380566	26.773798	8.670278	0	9.038324	24.035216	8.3283
FAM197Y1	0	0	0	0	0	0	0	0
FAM197Y3	0	0	0	0	0	0	0	0
FAM197Y5	0	0	0	0	0	0	0	0

FAM197Y8	0	0	0	0	0	0	0	0
FAM197Y9	0	0	0	0	0	0	0	0
FAM199X	33.042757	37.175036	22.863452	18.705033	19.558236	16.388766	47.616822	29.554769
FAM200A	11.038124	7.934867	11.109571	8.839639	1.416745	6.15501	0	7.179097
FAM200B	27.705753	39.332575	0	11.010669	7.260166	17.380106	51.018674	24.5289
FAM200C	6.005062	17.423422	7.528963	9.333037	6.506429	2.988591	0	6.744269
FAM201B	0	0	0	0	0	0	0	0
FAM204A	85.504167	106.983915	52.325056	90.865828	89.288116	93.452666	41.700329	140.964261
FAM204BP	0	0	0	0	0	0	0	0.251346
FAM204CP	0	0	0	0	0	0	0	0
FAM204DP	0	0	0	0	0	0	0	0
FAM207BP	0	0	0	0	0	0	0	0
FAM207CP	0	0	0	0	0	0	0	0
FAM209A	0	0	0	0	0	0	0	0
FAM209B	0	0.494056	0	0.438605	0	0.582399	0	0
FAM20A	0	0	0	0	0	0	0	0
FAM20B	54.817286	81.981412	46.836196	83.8973	37.571523	53.507709	21.562788	51.612356
FAM20BP1	0	0	0	0	0	0	0	0
FAM20C	23.685397	88.923063	44.808834	96.285853	9.453391	61.837413	7.38689	30.916344
FAM210A	50.377627	65.78931	48.451844	47.483594	45.220874	70.761045	121.867261	112.751843
FAM210B	31.481147	19.834942	29.212523	12.056536	14.540598	7.112386	26.767972	15.445758
FAM210CP	0	0	0	0	0	0	0	0
FAM216A	59.095626	27.696428	27.85678	22.727488	18.493891	22.995132	36.253702	34.252468
FAM216B	0	0	0	0	0	0	0	0
FAM217A	0	0	0	0	0	0	0	0
FAM217B	13.501698	70.239511	15.017893	55.28055	27.574189	39.44734	10.493475	55.222282
FAM218BP	0	0	0	0	0	0	0	0
FAM219A	12.197177	17.336615	8.414985	27.092332	14.367698	34.066822	17.852906	21.362212
FAM219B	14.085002	13.615816	12.037198	9.08163	85.010745	13.421457	0	9.055626
FAM21FP	0	0	0	0	0	0	0	0.786194
FAM220A	0	10.83768	0	0	0	0	0	0
FAM220BP	0	0	0	0.119006	0	0	0	0
FAM220CP	0	0	0	0	0	0	0	0

FAM221A	8.949189	6.770377	17.627782	4.681928	0	1.278063	0	2.077289
FAM221B	0	0	0	0	0	0	0	0
FAM222A	1.237315	2.582623	0	0	3.5915	0.513679	0	3.487922
FAM222B	0	0	0	0	0	0.387384	0	0
FAM227A	0	0.255913	0	1.945906	0	0	0	0.101893
FAM227B	8.316155	0.319174	7.29381	1.593078	0	1.754924	0	0.805159
FAM228A	0	0	0	0.220178	0	0	0	0
FAM228B	2.747504	7.759185	2.458117	11.045457	0.724822	3.853349	6.665393	7.848562
FAM229A	0	0.052143	0	0.18608	0.322958	1.720594	0	0.21719
FAM229B	7.598345	18.554807	26.271632	22.01961	3.962376	9.560758	0	16.4517
FAM234A	23.13009	105.720799	68.562751	132.728009	136.402154	129.211196	95.741908	110.856568
FAM234B	9.746686	6.310328	8.753706	8.093764	8.275217	5.19713	19.443438	3.934424
FAM236A	0	0	0	0	0	0	0	0
FAM236B	0	0	0	0	0	0	0	0
FAM236C	0	0	0	0	0	0	0	0
FAM237A	0	0	0	0	0	0	0	0
FAM238A	0	0	0	0	0	0	0	0
FAM238B	0	0	0	0	0	0	0	0
FAM238C	0	0	0	0	0	0	0	0
FAM240B	0	0	0	0	0	0	0	0
FAM240C	0	0	0	0.225937	0	1.268274	0	0
FAM241A	3.639486	4.892301	4.584845	2.690533	2.789609	1.224959	0.443987	2.580373
FAM241B	24.807169	24.616993	18.792587	25.93547	24.210614	26.760656	0	22.371942
FAM245B	0	0	0	0	0	0	0	0
FAM246A	0	0	0	0	0	0	0	0
FAM246B	0	0	0	0	0	0	0	0
FAM246C	0	0	0	0.049763	0	0	0	0
FAM247A	0	0	0	0	0	0	0	0
FAM24B	17.367212	10.93843	5.277212	15.684577	0	4.386091	0	2.295866
FAM25A	17.467537	3.125539	0	4.750934	0	14.723237	0	6.820088
FAM25BP	0	0	0	1.660649	0	0	0	0
FAM25EP	0	0	0	0	0	0	0	0
FAM25G	0	0	0	0	0	0	0	0

FAM27B	48.577364	21.364276	0	13.176109	23.293163	0	0	10.73148
FAM30B	0	0	0	0	0	0	0	0
FAM30C	0	0	0	0	0	0	0	0
FAM32A	75.822719	61.19179	61.336496	60.362777	49.294977	104.698305	33.265437	72.121396
FAM32BP	0	0	0	0	0	0	0	0
FAM32CP	0	0	0	0	0	0	0	0
FAM32DP	0	0	0	0	0	0	0	0
FAM32EP	0	0	0	0	0	0	0	0
FAM3A	22.469201	28.261101	9.155893	22.358325	28.825013	25.373671	9.666262	21.444583
FAM3B	0	0	0	0.331938	0	0.061293	0	0
FAM3C	71.672726	99.386737	69.238074	195.520177	36.752557	47.370734	47.487501	41.609915
FAM3C2P	84.575787	347.422658	105.985404	413.601108	87.965373	65.173936	62.929273	101.151173
FAM3D	0	0	0	0	0	0	0	0
FAM43B	0	0.650912	0	0.372583	0.3672	0.829534	0	0.616694
FAM47B	0	0	0	0	0	0	0	0
FAM47DP	0	0	0	0.024744	0	0.050602	0	0.040788
FAM47E	0	0	0	0.17534	0	1.242206	0	1.117855
FAM47E-STB	12.652405	13.126629	11.119494	9.645388	3.915781	2.713836	0	5.680496
FAM50A	87.067704	248.478489	87.343768	339.825066	85.574934	331.405039	85.885027	169.183381
FAM50B	6.479238	7.92225	5.771095	5.327341	0	6.610966	0	4.360122
FAM53A	0	1.657831	0	1.168959	0	0.410205	0	0.759173
FAM53B	9.610606	19.626615	15.77708	16.962244	6.721366	26.892567	27.063281	24.364715
FAM53C	15.12523	30.790653	22.151257	37.812889	16.398365	65.825339	17.244023	55.993984
FAM72A	47.306683	29.759981	21.938444	4.8472	0	17.484975	29.614117	13.929878
FAM72B	41.926543	74.750627	20.681033	51.039474	83.228899	32.31661	10.433111	33.630441
FAM72C	0	31.834811	0	39.611096	0	25.568001	0	12.869118
FAM72D	24.33638	34.933794	14.295295	26.37783	15.093456	19.89026	0	28.553902
FAM74A3	0	0	0	0	0	0	0	0
FAM74A4	0	0	0	0	0	0	0	0
FAM74A6	0	0	0	0	0	0	0	0
FAM76A	0	12.050826	0	7.003321	0	10.693329	0	13.582491
FAM76AP1	0	0	0	0	0	0	0	0
FAM76B	31.199264	89.213281	53.193289	73.566223	2.442564	18.799604	3.467825	28.113012

FAM78A	5.180445	0.576863	0.813556	0.657731	0.222347	0.967663	0	1.497619
FAM78B	0	0	0	0	0	0	0	0
FAM81A	21.498163	24.07834	10.604148	21.999812	8.505794	8.6341	1.895578	9.330463
FAM81B	0	0	0	0	0	0	0	0.233229
FAM83A	108.010386	132.882281	170.363904	222.37158	18.951466	34.153564	18.427589	28.833331
FAM83B	17.682983	20.200444	22.910942	17.523381	20.750719	10.484542	22.947318	25.402799
FAM83C	0	0	0	0	0	0.052629	0	0
FAM83D	33.157031	42.998042	23.206926	27.882139	52.096324	43.750608	44.049417	52.461583
FAM83E	0	1.624903	0	2.547234	5.532064	4.316295	0	1.612766
FAM83F	2.66464	12.046167	5.825957	8.774508	2.872131	13.038559	9.240618	13.149757
FAM83G	11.876656	17.160053	14.602411	21.557686	22.788448	56.234583	19.035741	41.659338
FAM83H	38.759994	53.257463	45.239102	52.039373	31.026837	79.528763	47.367625	52.164341
FAM85B	0	0	0	0	0	0	0	0
FAM86B1	15.223802	20.775484	8.165317	25.92663	23.431675	18.060876	0	13.744477
FAM86C2P	0	1.92765	0	1.520232	0	0.20356	0	0.926146
FAM86DP	0	6.990375	6.646175	6.111017	0	3.086852	0	5.756159
FAM86EP	0	0.163519	0	1.664437	0	1.434689	0	0.972068
FAM86FP	0	0	0	0	0	0	0	0
FAM86GP	0	0	0	0	0	0	0	0
FAM86HP	0	0.362753	0	0.522615	0	0.78425	0	0
FAM86JP	0	1.311387	0	2.356021	15.432774	3.49491	0	2.575854
FAM86KP	0	0	0	0	0	0	0	0
FAM86LP	0	0	0	0	0	0	0	0
FAM86MP	0	0	0	0	0	0	0	0
FAM89A	9.555654	15.075424	4.250143	7.687139	6.290409	4.928385	0	5.609405
FAM89B	5.905809	2.144714	5.236905	13.662844	6.502233	33.925793	0	24.043723
FAM8A1	6.938929	2.054145	5.608873	1.663807	5.498617	1.284981	8.618132	2.896142
FAM8A2P	0	0	0	0	0	0	0	0
FAM8A3P	0	0	0	0	0	0	0	0
FAM8A4P	0	0	0	0	0	0	0	0
FAM8A5P	0	0	0	0	0	0	0	0
FAM8A6P	0	0	0	0	0	0	0	0
FAM90A1	0	0.248097	6.659867	0.63937	0	0.072468	0	0.058837

FAM90A10	0	0	0	0	0	0	0	0
FAM90A13	0	0	0	0	0	0	0	0
FAM90A14	0	0	0	0	0	0	0	0
FAM90A15	0	0	0	0	0	0	0	0
FAM90A16	0	0	0	0	0	0	0	0
FAM90A18	0	0	0	0	0	0	0	0
FAM90A20	0	0	0	0	0	0	0	0
FAM90A21P	0	0	0	0	0	0	0	0
FAM90A22	0	0	0	0	0	0	0	0
FAM90A23	0	0	0	0	0	0	0	0
FAM90A24	0	0	0	0	0	0	0	0
FAM90A25P	0	0.489988	0	0.282927	0	0.127506	0	0
FAM90A26	0	0	0	0.033237	0	0	0	0
FAM90A2P	0	0.489988	0	0.282927	0	0.127506	0	0
FAM90A3	0	0	0	0	0	0	0	0
FAM90A4P	0	0	0	0	0	0	0	0
FAM90A5	0	0	0	0	0	0	0	0
FAM90A6P	0	0	0	0	0	0	0	0
FAM90A7	0	0	0	0	0	0	0	0
FAM90A8	0	0	0	0	0	0	0	0
FAM90A9	0	0	0	0	0	0	0	0
FAM91A1	66.228047	147.197828	83.455055	73.573367	60.571627	58.606689	101.17301	212.890408
FAM91A2P	0	0	1.198986	0	0	0	0	0
FAM91A3P	0	0.882132	0	0.099229	0	0	0	0.905801
FAM95B1	0	0	0	0.034161	0	0.019446	0	0.028058
FAM98A	34.374946	29.372566	32.072521	31.94287	18.726431	21.487515	17.672917	22.975918
FAM98B	22.490288	12.341696	16.827385	6.115608	28.116223	5.213272	41.708669	14.295055
FAM98C	0	2.199209	12.867708	0.21198	0	1.48032	0	1.558299
FAM9A	0	0	0	0	0	0	0	0
FAM9B	0	0	0	0	0	0	0	0.145838
FAM9C	0	0	0	0	0	0	0	0
FAM9CP1	0	0	0	0	0	0	0	0
FAN1	24.386105	24.71664	25.630457	19.136774	10.767172	11.593473	41.843455	22.053665

FANCA	19.35597	25.529111	8.639055	21.84837	68.913071	31.301587	44.278678	30.810927
FANCB	0	4.414168	0	0.465095	0	0.962271	0	1.338215
FANCC	18.206282	27.448096	9.145144	22.135912	8.876003	15.83795	30.718933	29.148376
FANCD2	13.379326	7.52991	3.52066	6.685439	17.971353	13.28433	25.005556	10.880469
FANCD2OS	0	0	0	0	0	0	0	0
FANCD2P2	0	0	0	0	0	0	0	0
FANCE	0	0	9.535566	0.828335	0	2.856782	13.89784	0
FANCG	16.378796	6.854832	30.513757	8.145438	9.648692	9.199797	0	1.970585
FANCI	7.821003	69.758367	41.513114	84.856227	51.884074	69.235377	94.04268	121.324018
FANCM	4.165964	3.238917	5.209263	0.980865	0.416669	0.109468	4.664141	5.176768
FANK1	9.949911	4.374954	0	1.352572	0	1.3635	0	4.3319
FAP	0	0	2.336849	1.233369	0.701567	0.612585	2.384818	2.218934
FAR1	46.223319	144.740154	62.58381	153.113836	116.695297	150.831667	63.956489	176.50397
FAR1P1	0	0	0	0	0	0	0	0
FAR2	0	0.497672	4.458111	0.562969	0	0.582051	0	0
FAR2P1	0	0	0	0	0	0.566829	0	0
FAR2P2	0	0	0	0	0	0	0	0
FAR2P3	0	0	0	0	0	0	0	0
FAR2P4	0	0	0	0	0	0	0	0
FARP1	49.546893	42.924234	15.956774	68.761753	38.343557	13.181826	0	31.187413
FARP2	28.367642	23.144908	19.148205	19.478815	36.373366	54.932593	12.409626	61.469878
FARS2	0	1.491317	0	1.663941	5.375605	4.599205	0	3.404989
FARSA	44.546201	35.700694	35.032678	49.744709	55.615266	85.182415	141.569801	63.790235
FARSB	0	0	0	0	0	0	0	0
FARSBP1	0	0	0	0	0	0	0	0
FAS	0	0	0	3.206285	0	0	0	0.257221
FASLG	0	0	0	0	0	0	0	0
FASN	153.885462	96.54898	92.813504	92.85487	124.146254	142.807663	190.706615	84.584169
FASTK	7.828947	10.865311	12.446513	11.406988	16.300527	22.893996	25.883258	18.418048
FASTKD1	9.930378	23.092437	32.871856	13.497543	48.127175	16.688423	17.612111	29.101197
FASTKD2	27.422848	28.535659	14.285995	8.520835	32.679153	8.880951	65.846087	50.059938
FASTKD3	8.414887	21.738863	30.835662	14.025176	29.731451	6.712702	63.88662	17.805015
FASTKD5	26.084343	35.074823	25.485662	25.940139	16.583548	26.262755	26.490613	54.176541

FAT1	71.832726	176.525079	133.951031	293.232998	66.984403	159.051804	34.741888	194.539384
FAT1P1	0	0	0	0	0	0	0	0
FAT2	0	0	1.704936	0.028994	0	0.81553	5.442213	0.202511
FAT3	1.505193	1.517362	1.054609	2.028962	0	1.907776	0	0.087019
FAT4	0	5.103387	0	0	0	0	0	0
FATE1	0	0	0	0	0	0	0	0
FAU	428.574001	486.095097	391.512374	548.067128	1022.76503	939.728786	1113.23141	847.131993
FAUP1	0	0	0	0.311432	0	0	0	0
FAUP2	0	0	0	0	0	0	0	0
FAXC	4.52631	3.453981	0	1.795077	5.143278	6.880811	0	5.879241
FAXDC2	1.140601	3.891781	2.04363	1.285992	3.311365	4.447985	0	2.8713
FBF1	12.754912	14.494557	0	19.87216	2.034045	17.008315	0	13.206678
FBH1	11.18994	10.491315	7.232976	12.688686	3.102304	23.756255	32.14951	15.846145
FBL	320.10827	324.454323	313.32457	396.68656	545.991911	503.563932	310.88996	381.691273
FBLIM1	46.061428	54.078684	30.703029	86.925299	34.289061	86.118277	19.092225	58.18385
FBLIM1P1	0	0	0	0	0	0	0	0
FBLIM1P2	0	0	0	0	0	0	0	0
FBLN1	46.623227	35.18241	59.02512	45.848999	14.126292	41.822106	12.736243	17.144087
FBLN2	0	0	0	0.058549	0	0.039958	0	1.868624
FBLN5	0	3.375059	1.662614	1.247483	4.333136	3.32215	0	0
FBLN7	0	2.265599	2.659376	1.40651	0	1.47699	0	0.738785
FBLP1	0	0	0	0	0	0	0	0
FBN1	0.80296	0.4472	0	0.629318	0	2.124576	0	3.254486
FBN2	7.041214	14.498268	16.229338	15.514935	4.575624	8.350376	3.079201	7.599401
FBN3	0	0.015259	0.318605	0	0	0	0	0
FBP1	0	0.559889	0	0	0	0	0	0
FBP2	0	0	0	0	0	0	0	0
FBP2P1	0	0	0	0	0	0	0	0
FBRS	12.408893	1.749928	15.981468	6.880197	16.482966	6.456717	0	3.459358
FBRSL1	5.815961	39.579082	34.889831	41.052448	51.379711	80.96893	2.304705	61.546419
FBRSL1P1	0	0	0	0.708283	0	0	0	0
FBXL12	10.15181	8.475009	5.082997	10.888327	0	4.896671	73.107521	6.810959
FBXL12P1	0	0	0	0	0	0	0	0

FBXL13	0	0.161435	0	0.134077	0	0.447277	0	0.926172
FBXL14	9.41519	12.12938	6.017776	11.921582	1.419408	16.240221	6.200722	13.647844
FBXL15	5.320215	1.174664	0	2.826394	0	4.336485	0	2.521924
FBXL16	0	14.432506	0	27.470493	5.313734	8.694157	0	5.168194
FBXL17	0	4.696848	15.732197	5.030055	0	0.826285	0	11.31275
FBXL18	21.328453	18.450137	19.602453	18.778972	18.946508	22.346598	14.479737	17.559808
FBXL19	8.060191	7.295851	7.300569	7.907549	10.537852	20.23207	0	10.947728
FBXL2	4.319492	1.042976	2.007963	3.77117	5.209323	0.929261	2.227918	1.416161
FBXL20	15.535421	19.141513	18.733118	25.449309	3.28389	12.60208	9.399249	15.925025
FBXL21P	0	0	0	0	0	0.12833	0	0
FBXL22	0	0.394331	0	1.385111	0	0.854033	0	1.378362
FBXL3	8.644269	26.458169	9.46011	16.218762	11.595767	17.625792	12.919339	28.476636
FBXL4	5.426495	17.750935	10.236303	16.405677	5.869829	7.03471	13.697023	19.872835
FBXL5	32.742687	22.337344	10.818128	9.448603	29.027969	12.015491	35.191502	27.80101
FBXL6	6.126605	4.730576	0	3.237664	6.416915	6.49075	0	7.19643
FBXL7	0	0	0	0	0	0	0	0.143879
FBXL8	12.420381	6.1931	7.520553	4.717479	2.182252	3.42488	0	3.708056
FBXL9P	0	1.290422	5.949126	0.934638	0	0.372197	0	0.527283
FBXO10	0	2.262272	2.506021	3.711245	0.87002	6.681817	3.227664	9.051096
FBXO11	34.927966	32.140597	0	44.955393	35.088026	30.876837	22.332949	37.765074
FBXO15	0	0.276439	0	0.254744	0	0	0	0.345386
FBXO16	0	5.999344	1.581482	8.835311	5.943563	6.571403	0	3.728019
FBXO17	29.757765	30.947722	4.15427	23.15451	7.354713	15.422714	5.603746	13.784443
FBXO2	2.856562	14.712114	12.671966	14.417513	6.272006	13.349522	0	11.558404
FBXO21	0	6.225266	18.957853	13.389857	7.449203	26.672783	0	15.035005
FBXO22	124.590841	54.584286	52.415613	31.406828	24.5645	33.932486	28.92289	68.423909
FBXO24	0	0.641862	0	0.812003	0	0.181399	0	0.332878
FBXO25	0.617882	4.857027	1.390396	0.296183	0.996149	2.374819	1.055598	14.22189
FBXO27	20.703343	20.61982	39.316033	24.744646	12.375447	29.050589	67.264278	19.327135
FBXO28	40.276022	30.42499	21.066839	28.225297	30.968051	21.180669	4.394041	27.339788
FBXO3	20.322844	14.486789	19.265483	6.327781	56.885027	7.671734	121.631109	21.519748
FBXO30	4.620884	5.239267	2.878736	3.367484	5.73091	1.857356	2.081487	4.727813
FBXO31	8.741123	24.215429	25.766623	27.373001	14.7809	57.885928	0.302049	33.155072

FBXO32	8.648088	35.961024	24.346257	90.263416	32.839527	104.850775	1.522877	93.204519
FBXO33	0	0	0	2.217045	0	1.941488	0	1.79465
FBXO34	29.844161	67.808504	24.524555	96.804801	44.280073	83.802802	31.611725	106.209028
FBXO36	8.587216	6.230699	3.220604	2.709031	0.968078	1.066802	7.553828	1.640723
FBXO36P1	0	0	0	0	0	0	0	0
FBXO38	8.436399	6.850653	9.733648	11.830891	31.707958	8.062227	34.538806	23.027951
FBXO39	0	1.21163	0	0.771265	0	0.310215	0	0
FBXO4	35.670962	24.843336	5.724597	15.808097	10.919369	9.162816	17.227782	14.234648
FBXO40	0	0	0	0	0	0	0	0
FBXO41	5.608102	9.242382	6.341804	10.498768	5.804362	17.767401	3.760579	14.516127
FBXO42	7.30171	27.329091	13.314146	23.073793	15.105106	34.023955	4.449772	37.764517
FBXO43	0	3.026641	1.143048	0.758442	0	0.487707	0	0.524774
FBXO44	2.032981	3.673758	3.086529	3.579069	2.67835	9.19811	0	7.038235
FBXO45	29.811814	36.987986	31.650718	19.805384	32.956136	17.549026	42.916729	37.242242
FBXO46	8.975404	9.703001	21.006022	19.877964	5.626174	38.044199	0.272563	25.32867
FBXO48	0.57148	2.08743	0.513623	1.718597	0	0.859249	1.810485	1.675273
FBXO5	15.470383	29.795917	29.483811	15.261259	19.105322	9.593457	6.937764	25.67384
FBXO6	2.50074	12.37411	4.446293	10.916014	4.608454	19.355662	0	21.324819
FBXO7	57.820411	56.849674	59.956943	37.928811	55.181806	34.35601	76.480489	53.91998
FBXO8	10.564543	30.706539	11.730517	25.136485	6.256184	10.828525	0	14.832424
FBXO9	41.507285	63.216102	45.360127	60.767669	40.969772	88.607969	99.071154	81.324975
FBXW10	0	0	0	0	0	0.05258	0	0
FBXW10B	0	1.836934	3.859188	0	0	0	0	0.324718
FBXW11	58.420521	91.099672	67.322264	59.555394	46.850554	43.471438	24.910292	76.093744
FBXW11P1	0	0.819442	0	0.295394	0	0.839363	0	1.088064
FBXW12	0	0	0	0	0	0	0	0
FBXW2	20.94936	31.965342	15.212101	18.702142	15.985965	13.689808	18.153699	26.913524
FBXW4	7.129787	6.014265	5.101553	12.457824	7.146797	16.346643	0.691666	15.768899
FBXW4P1	0	0.520562	0	0.298278	0	0.98849	0	0.679897
FBXW5	23.322183	26.628979	11.645727	25.075188	37.272209	56.852295	15.094999	49.46512
FBXW7	116.563256	78.500966	74.665011	93.423318	7.156001	63.738733	52.701663	57.229665
FBXW8	7.060163	5.778863	20.546223	4.57084	4.998332	3.281518	6.017327	3.597158
FBXW9	0	3.711865	7.280831	3.762619	4.842813	8.861157	0	5.034287

FCAMR	0	0	0	0.042583	0	0.134975	0	0
FCAR	0	0	0	0	0	0	0	0.102038
FCER1A	0	0	0	0	0	0	0	0
FCER1G	0	0	0	0.3895	0	0	0	0
FCER2	0	0	0	0.121605	0	0	0	0
FCF1	136.185183	121.5277	149.834385	117.432533	175.621432	57.883225	229.873549	107.721999
FCF1P1	0	0	0	0	0	0	0	0
FCF1P10	0	0	0	0	0	0	0	0
FCF1P2	0	2.803442	0	0	0	0	0	2.771351
FCF1P3	0	0	0	0	0	0	0	0
FCF1P4	0	0	0	0	0	0	0	0
FCF1P5	0	0	0	0	0	0	0	0
FCF1P6	0	0	0	0	0	0	0	0
FCF1P7	0	0	0	0	0	0	0	0
FCF1P8	0	0	0	0	0	0	0	0
FCF1P9	0	0	0	0	0	0	0	0
FCGBP	0	0	0	0.568185	0.743236	6.635131	1.343237	0
FCGR1A	0	0	0	0	0	0	0	0
FCGR1BP	0	0	0	0	0	0	0	0
FCGR1CP	0	0	0	0	0	0	0	0
FCGR2A	0	0	0	0	0	0	0	0
FCGR2B	0	0	0	0	0	0	0	0
FCGR2C	0	0	0	0	0	0	0	0
FCGR3A	0	0	0	0	0	0	0	0
FCGR3B	0	0	0	0	0	0	0	0
FCGRT	2.200456	21.077083	16.242476	17.175714	18.551738	39.899776	23.08606	37.420126
FCHO1	0	1.713953	0	0.721718	0	0.856456	0	0
FCHO2	18.857043	8.642247	11.447167	11.979325	31.213026	11.340024	32.910936	12.708083
FCHSD1	28.115824	6.449152	12.842583	8.41264	10.698448	3.438625	2.422176	1.328827
FCHSD2	35.916164	99.929335	36.516884	67.774359	43.140362	34.289945	17.168645	51.511829
FCMR	0	3.24606	0	1.979444	1.149352	5.718888	0	6.633163
FCN1	0	0	0	0	0	0	0	0
FCN2	0	0	0	0	0	0	0	0

FCN3	0	0	0	0	0	0	0	0
FCRL1	0	0	0	0.281237	0	0	0	0
FCRL2	0	0	0	0	0	0	0	0
FCRL3	0	0	0	0	0	0	0	0
FCRL4	0	0	0	0	0	0	0	0
FCRL4P1	0	0	0	0	0	0	0	0
FCRL5	0	0	0	0	0	0	0	0
FCRL6	0	0	0	0	0	0	0	0
FCRLA	0	1.279897	0	0.122035	0	0	0	0
FCRLB	0	4.625571	0	4.253278	0	2.159096	0	1.62023
FCSK	2.196166	8.993666	6.969659	8.127246	0.337061	3.142676	8.992049	1.215665
FDFT1	64.020385	49.634789	40.593659	29.825143	20.288765	26.459468	23.870372	27.436348
FDPS	336.376658	271.514217	201.23563	239.480562	269.630407	201.029427	161.97922	183.575336
FDPSP1	0	0	0	0	0	0	0	0
FDPSP2	0	0	0	0	0	0	0	0
FDPSP3	0	0.626476	0	0	0	0	0	0
FDPSP4	0	0	0	0	0	0.352953	0	0
FDPSP5	0	0	0	0	0	0	0	0
FDPSP6	0	0	0	0	0	0	0	0
FDPSP7	0	0	0	0	0	0	0	0
FDPSP8	0	0	0	0	0	0	0	0
FDX1	13.841099	14.797155	7.633834	8.643922	10.960577	9.499207	5.433619	15.925442
FDX1P1	0	0	0	0	0	0	0	0
FDX1P2	0	0	0	0	0	0	0	0
FDX2	19.024185	43.609786	41.861634	39.021112	28.685487	66.828871	49.718381	48.471835
FDXACB1	0	8.75829	0	5.017796	5.137479	5.789722	10.130711	12.27103
FDXR	18.645592	6.528623	11.974545	8.494398	3.275049	21.833839	23.555134	16.879457
FECH	21.453269	35.140495	12.124048	19.626984	43.69534	32.116064	116.478868	36.183348
FECHP1	0	0	0	0	0	0	0	0
FEM1AP1	0	0	0	0	0	0	0	0
FEM1AP2	0	0	0	0	0	0	0	0
FEM1AP3	0	0	0	0	0	0	0	0
FEM1AP4	0	0	0	0	0	0	0	0

FEM1B	52.449704	154.344733	35.590319	121.259985	65.157915	121.475013	79.822483	212.385597
FEM1C	9.146993	13.685623	8.220932	11.978622	7.401036	7.87929	4.458189	19.475263
FEN1	72.244747	128.524887	55.293262	104.347436	173.688402	238.272843	168.540936	250.380918
FEN1P1	0	0	0	0	0	0	0	0
FER	25.260706	22.259969	11.00617	16.602629	7.927258	11.846931	6.098285	24.966712
FER1L4	0	0.519274	0	1.402837	0	0.434171	0	0
FER1L5	0	0	0	0	0	0.069248	0	0
FER1L6	0	0.092381	0	0.026273	0	0	0	0
FERD3L	0	0	0	0	0	0	0	0
FERMT1	0	31.048476	26.207619	34.04049	32.920391	31.485346	106.109085	38.585688
FERMT2	25.252882	21.092028	14.572884	23.520427	43.541166	17.879079	34.430895	31.218166
FERMT3	0	0	0	0	0	0	0	0
FERP1	0	0	0	0.123155	0	0	0	0
FERRY3	33.225698	11.963085	10.29388	9.761837	18.429844	10.183565	48.583793	32.276885
FES	0	0.306302	0	0.582147	0	3.420566	0	3.233336
FETUB	0	0	0	0.17678	0	0	0	0
FEV	0	0	0	0	0	0	0	0.730476
FEZ1	40.042888	10.416643	8.566796	14.434635	37.703418	57.757007	166.059681	57.554981
FEZ2	52.82458	93.476522	82.671919	56.81675	45.005569	37.033105	80.900036	68.482991
FEZF1	0	0.667701	0	0.340652	0	0	0	0.056389
FEZF2	0	0	0	0	0	0	0.439778	0
FFAR2	0	0	0	0	0	0	0	0
FFAR3	0	0	0	0	0	0	0	0
FFAR4	0	0	0	0	0	0	0	0
FGA	0	0	0	0	0	0	0	0
FGB	0	0.533951	0	0.077416	0	0	0	0
FGD1	9.85123	16.075268	12.926577	17.772268	12.608865	19.080161	7.010107	14.967482
FGD2	0	1.555498	2.318737	5.767522	0	1.652606	0	3.132048
FGD3	5.484683	7.058056	0	5.787655	0	13.045518	3.464884	10.310408
FGD4	10.360717	0	0	0.047406	0	0.171035	43.935914	2.156388
FGD5	0	0.749716	0	1.167892	0	0	0	0
FGD5P1	0	0	0	0.2356	0	0	0	0
FGD6	58.424433	71.010305	30.210373	90.788157	50.360439	61.8277	59.521079	92.00324

FGF1	0	4.658406	0	0.218587	0	1.59995	28.302383	0.423277
FGF10	0	0	0	0.080061	0	0	0	0
FGF11	10.531028	33.084927	14.137334	44.439023	19.102174	37.409015	42.698228	33.814331
FGF12	0	0	0	0	0	0	0	0.43056
FGF13	3.684741	0	0	0.099048	0	0	0	0
FGF14	0	0	0	0	0	0	0	0
FGF17	0	0.336099	0	0	0	0.135666	0	0
FGF18	0	0	0	0.084375	0	0	0	0
FGF19	0	3.006845	1.71685	4.342866	0	0.095037	0	0
FGF2	2.436414	1.956059	3.943437	2.434359	1.545142	1.946948	12.205596	9.165537
FGF20	0	0	0	0	0	0	0	0
FGF21	0	0.726504	0	0	0	0	0	0
FGF22	0	0	0	0	0	0	0	0
FGF23	0	0	0	0	0	0	0	0
FGF3	0	0	0	0	0	0	0	0
FGF4	0	0	0	0	0	0	0	0
FGF5	64.655109	5.309395	27.502293	18.158219	0	4.08459	0	6.161699
FGF6	0	0	0	0	0	0	0	0
FGF7	0	0	0	0	0	0	0	0
FGF7P1	0	0	0	0	0	0	0	0
FGF7P2	0	0	0	0	0	0	0	0
FGF7P3	0	0	0	11.184701	0	0	0	5.377073
FGF7P4	0	0	0	0	0	0	0	0
FGF7P5	0	8.152581	0	5.252649	0	0	0	15.566925
FGF7P6	0	0	0	0	0	0	0	0
FGF7P8	0	0	0	0	0	0	0	1.804241
FGF8	0	0.287604	0	0	0	0	0	0
FGF9	0	0	0	0	0	0	0	0
FGFBP2	0	0	0	0	0	0	0	0
FGFR1	13.962784	35.879998	13.827814	38.306884	39.75792	42.25719	29.403242	29.395778
FGFR1OP2	66.18083	103.920453	23.022471	86.401269	10.987622	42.500084	68.648188	60.28021
FGFR1OP2P1	0	0	0	0	0	0	0	0
FGFR2	15.608984	23.420682	8.876632	14.520194	16.700816	17.152854	10.592488	14.158819

FGFR3	0	4.034244	0	5.896749	85.909729	25.362065	28.46797	5.431019
FGFR3P1	0	0	0	0	0	0	0	0
FGFR3P3	0	0	0	0	0	0	0	0
FGFR3P4	0	0	0	0	0	0	0	0
FGFR3P5	0	0	0	0	0	0	0	0
FGFR3P6	0	0	0	0	0	0	0	0
FGFR4	8.463846	9.879462	8.737681	17.042215	24.818706	8.666065	0	9.799018
FGFRL1	10.807631	17.731243	20.339288	23.585507	8.843433	26.155584	0	19.317302
FGG	0	0	0	0	0	0	0	0
FGGY	25.706539	7.006084	18.290965	21.381942	21.10848	12.032532	0	13.694016
FGL1	0	0.648397	0	0	0	0	0	0.142643
FGL2	0	0	0	0.018901	0	0.077405	0	0
FGR	0	0.25392	0	0.318514	0	0	0	0.418161
FH	0	0	0	4.852769	0	0	36.287644	2.114832
FHAD1	8.041809	0.42109	0	1.353087	0	0.317375	0	2.047609
FHIP1A	8.343323	39.831494	6.509689	47.838599	6.543079	115.572382	60.338231	162.941918
FHIP1B	0	6.878056	0	7.031295	13.823999	5.790046	34.068616	6.705229
FHIP2A	2.255252	5.003973	7.601309	4.546461	30.78377	4.696209	8.381742	11.769381
FHIP2B	5.835838	7.012541	10.925005	9.472615	6.948353	13.512565	0	7.039045
FHIT	0	1.187946	0	0.413764	0	0.204105	0	0.16736
FHL1	4.459445	5.393059	8.473686	6.846427	10.419151	17.994777	36.021386	2.474115
FHL2	70.616531	77.362786	88.539691	84.102302	92.357932	107.743232	76.598809	133.13647
FHL3	7.362034	8.234934	13.348432	11.503035	17.481974	32.394356	15.977113	29.31992
FHL5	0	0	0	0	0	0	0	0
FHOD1	54.976533	10.133273	14.025715	6.698083	25.456957	24.001758	35.823619	11.930326
FHOD3	35.550117	69.556212	29.457914	68.815868	39.641689	73.609653	27.394462	56.948482
FHP1	0	0	0	0	0	0	0	0
FHP2	0	0	5.512583	0	0	0	0	0
FIBCD1	2.793774	24.354196	24.949451	19.49801	0	7.624232	0	5.379709
FIBP	115.386784	87.516033	17.62789	85.429033	153.066362	82.480572	31.081533	63.055922
FICD	2.052444	2.10412	3.679778	3.48076	3.01903	4.827062	0	3.300401
FIGLA	0	0	0	0	0	0	0	0
FIGLAP1	0	0	0	0	0	0	0	0

FIGN	2.358884	11.273024	1.516358	8.251937	0.623496	15.052426	1.97354	23.443678
FIGNL1	10.096515	27.235413	0	28.87294	49.777171	34.80545	52.059012	58.730513
FILIP1	0	0.037676	0	0.064455	0	0	0	0
FILIP1L	4.721296	14.507828	10.571626	13.109181	20.055766	20.349337	30.958861	21.500317
FIP1L1	14.980918	82.890294	35.867481	80.86564	58.434761	55.421137	152.380786	60.306253
FIRRM	27.153843	7.130008	23.062796	6.185926	5.775083	4.695414	4.63242	10.021535
FIS1	16.990235	10.489367	0	24.977748	35.626124	8.734241	59.646988	12.828117
FITM1	0	0	0	0.36539	0	0.243867	0	0
FITM2	5.673547	4.959517	4.458262	3.638357	2.622528	2.164826	6.735073	2.134785
FIZ1	25.038383	11.331114	9.751002	15.076158	5.743972	28.810825	5.9056	19.046885
FJX1	30.667918	0	0	0.546137	0	0	0	2.67366
FKBP10	0	15.616896	0	19.915186	0	8.139427	0	2.647897
FKBP11	19.826696	77.600175	81.160485	82.386784	34.301824	20.176156	9.05353	9.29467
FKBP14	0	20.445126	24.974213	23.831136	27.85583	19.3848	0	19.777636
FKBP15	11.002978	15.957494	10.905147	13.111294	20.807685	11.82157	2.508875	15.976505
FKBP1A	238.304456	192.448142	247.781905	158.751904	248.219865	104.197908	193.511869	180.655033
FKBP1AP1	0	0	0	0	0	0	0	0
FKBP1AP2	0	0	0	0	0	0	0	0
FKBP1AP3	0	0	0	0	0	0	0	0
FKBP1AP4	0	0	0	0	0	0	0	0
FKBP1B	0	6.968377	4.098303	7.915771	14.023327	4.39968	0	8.322787
FKBP1BP1	0	0	0	0	0	0	0	0
FKBP2	81.458303	134.935389	102.970053	148.186557	120.309926	142.802232	0	104.524917
FKBP3	163.898141	141.403582	135.408252	74.775137	168.028406	68.126092	146.877868	101.396747
FKBP4	53.552783	113.915397	48.843951	111.983473	46.89903	145.827088	51.891071	115.825326
FKBP4P1	0	0	0	0	0	0	0	0
FKBP4P2	0	0	0	0	0	0	0	0
FKBP4P6	0	0	0	0	0	0	0	0
FKBP4P7	0	0	0	0	0	0	0	0
FKBP4P8	0	0	0	0	0	0	0	0
FKBP5	0	0	0	0	0	0	0	0.038799
FKBP6	0	0	4.640052	0.229947	0	0	0	0
FKBP6P1	0	0	0	0	0	0	0	0.233026

FKBP6P2	0	0	0	0	0	0	0	0.233026
FKBP7	0	3.848268	0	5.963517	0	0	0	1.556474
FKBP8	47.956394	107.778371	71.465809	86.560071	64.495948	176.174152	34.007317	117.549192
FKBP9	31.864149	71.784685	65.359296	93.745978	185.952814	140.125354	90.912027	48.410396
FKBP9P1	5.404031	2.195713	1.919957	5.431167	0	5.301746	0	4.954544
FKBPL	8.162805	19.327943	12.079519	27.20589	17.18469	36.62078	0	27.475968
FKRP	7.009701	4.527066	25.273282	7.284921	46.562754	26.513121	0	15.51594
FKTN	18.361631	29.791735	15.018516	11.31928	4.756177	7.74791	15.067965	9.070824
FLACC1	0	0.426194	0	0.052931	0	0	0	0.432851
FLAD1	21.945302	53.172773	17.946812	45.802257	29.563682	58.291053	0.380873	47.980043
FLCN	8.510013	16.465274	12.668242	21.650241	13.246443	39.866602	20.628473	40.560001
FLG2	0	0	0	0.017261	0	0	0	0
FLI1	9.62783	34.768176	24.299362	38.802291	10.855032	2.066971	0	6.706626
FLII	55.141101	90.58718	62.993706	92.304164	243.353425	302.989098	531.903245	161.319638
FLNA	300.432115	287.63204	342.04515	335.784045	402.280822	786.735519	722.202718	366.119341
FLNB	43.205245	37.734396	87.096811	73.492226	120.023724	152.401478	104.628414	70.480735
FLNC	0	2.762503	1.885555	1.478678	0	2.138855	39.931769	0
FLOT1	118.740215	88.609804	88.298169	101.003067	75.047014	126.304194	37.045143	80.279848
FLOT2	58.139327	115.782104	26.939657	107.846932	75.630578	188.959599	85.833012	140.624151
FLRT1	0	0	0	0.224781	0	0.041829	0	0.067237
FLRT2	28.627435	72.304459	24.300687	65.202394	12.644239	43.86172	23.022848	59.77242
FLRT3	2.059014	2.446154	1.77209	6.28085	0.906471	3.097238	0	4.602353
FLT1	0	0	0	0	0	0	0	0
FLT1P1	0	0	0	0	0	0	0	0
FLT3	0	0	0	0.066871	0	0	0	0
FLT3LG	11.145429	7.649296	12.854207	10.00956	4.3196	14.357519	45.278733	13.568894
FLT4	2.231699	2.058977	6.774217	2.537527	0	2.581004	0	0.625054
FLVCR1	2.747794	8.817421	32.227425	3.079595	0.435603	3.357789	6.542614	3.754672
FLVCR2	3.768445	3.422485	0	0.825135	0	1.404649	0	1.509438
FLYWCH1	5.94622	59.956104	23.597947	41.371981	39.364747	56.518774	108.441524	37.176031
FLYWCH1P1	0	0	0	0	0	0	0	0
FLYWCH2	57.00926	70.288716	30.379826	61.209571	11.28962	98.909161	0	61.228984
FMC1	3.795673	0.970685	0	3.370214	7.975	9.00217	0	11.515304

FMC1-LUC7L	0	7.887951	0	8.98956	0	35.119597	0	10.789787
FMN1	13.278446	9.707492	6.853608	9.72933	5.006824	10.332188	2.781685	12.474564
FMN2	6.110063	2.409443	1.290532	2.642783	1.333551	0.379691	0	0.263047
FMN2P1	0	0	0	0	0	0	0	0
FMNL1	2.475733	1.009341	0	1.768295	2.997939	0.910302	18.246015	1.966869
FMNL2	15.625435	25.786815	18.723892	27.522717	24.004928	28.683125	21.295027	39.334849
FMNL3	0	3.979378	0.912429	1.170659	0	0.954828	0	0.393916
FMO1	0	0	0	0	0	0	0	0
FMO10P	0	0	0	0	0	0	0	0
FMO11P	0	0	0	0	0	0	0	0
FMO2	0	0	0	0	0	0	0	0
FMO3	0	0	0	0	0	0	0	0
FMO4	1.487183	1.326014	0	3.036204	0	1.532736	0	1.018777
FMO5	0	0.286398	0	0.680639	0	0	0	0.128077
FMO7P	0	0	0	0	0	0	0	0
FMO8P	0	0	0	0	0	0	0	0
FMO9P	0	0	0	0	0	0	0	0
FMOD	6.287777	5.212821	10.232866	8.659653	3.014687	1.194579	2.13696	0.45947
FMR1	28.183023	51.242009	24.965531	21.516747	26.088773	25.565455	0	41.800186
FMR1NB	0	0	0	0	0	0	0	0
FN1	105.531164	96.796052	110.861638	118.620473	33.425245	22.802324	147.55645	16.415807
FN3K	7.818584	17.688571	9.262082	20.789276	2.743918	14.872658	16.953501	8.344706
FN3KRP	26.933032	42.186606	46.923042	38.409337	23.658613	42.437539	46.526869	27.944105
FNBP1	16.410402	16.136379	0	13.226367	53.925416	25.983972	5.46224	23.876114
FNBP1L	28.113086	14.077462	20.711975	14.851728	1.988174	10.155409	40.054629	8.792678
FNBP1P1	0	2.210294	3.108809	3.086432	5.507852	2.411637	7.586945	4.56615
FNBP4	27.656898	19.289926	9.907335	20.148248	18.004049	21.479443	101.738836	29.351903
FNDC1	0	0.621081	0	0.193821	0	0	0	0.172835
FNDC10	6.497496	6.402585	5.80489	6.276339	0	6.597979	0.393516	3.275133
FNDC11	0	2.008366	7.487683	2.97919	0	0.793264	0	0.045764
FNDC3A	11.448613	20.691214	13.466488	15.57576	40.820049	13.08616	12.361348	18.373321
FNDC3B	107.350376	108.187961	192.840765	242.478455	188.761565	162.892883	156.42621	113.729924
FNDC3CP	0	0	0	0	0	0	0	0

FNDC4	4.413236	0.92817	1.964928	3.473825	0	0.977448	0	0.713996
FNDC5	0	0	0	0.663804	0	0.674199	0	0
FNDC7	0	0	0	0	0	0	0	0
FNDC8	0	0	0	0	0	0	0	0
FNDC9	0	0	0	0	0	0	0	0
FNIP1	18.94192	19.54915	20.232083	19.412048	44.934356	16.724445	50.146384	23.64366
FNIP2	3.218893	6.082001	2.894848	5.89951	13.894828	12.00782	3.47612	17.747018
FNTA	79.997925	80.478268	67.533992	59.824658	71.515893	32.465579	86.419246	38.032605
FNTAP1	0	0	0	0	0	0	0	0
FNTAP2	0	0	0	0	0	0	0	0
FNTB	7.482639	15.819585	6.838703	13.041462	118.263513	9.902074	24.053026	13.367634
FOCAD	14.393879	4.618353	11.501736	7.10815	27.372288	10.909804	5.652112	6.048543
FOLH1	0	0.308097	0	0.167844	0	0.388931	26.866896	0
FOLH1B	0	2.308799	7.215584	2.071991	0	0	0	0
FOLR1	0	0	0	0	0	1.219988	0	1.170769
FOLR1P1	0	0	0	0	0	0	0	0
FOLR2	0	0	0	0	0	0	0	0
FOLR3	0	1.3832	0	1.010184	0	4.14995	0	0.239631
FOS	64.010353	67.083379	79.175281	131.149251	175.094768	267.370585	93.718556	97.492499
FOSB	7.973534	16.34522	6.883954	19.905792	45.624623	50.767584	31.500196	16.161892
FOSL1	98.718114	66.881032	51.432181	114.252325	67.197435	59.32081	0	52.346141
FOSL1P1	0	0	0	0	0	0	0	0
FOSL2	38.618433	85.280074	49.05076	125.082171	58.406563	191.603715	47.273896	148.607016
FOXA1	0	13.815053	0	26.797469	14.444675	38.28528	0	9.129949
FOXA2	12.793103	0	0	0	0	0.7057	0	0.629861
FOXA3	0	1.83871	1.617203	2.727237	0	0.537452	0	0.365714
FOXB1	1.172857	0.350022	0	0.385657	0	0.642278	0	0.754967
FOXB2	0	0	0	0	0	0	0	0
FOXC1	4.977531	6.501686	8.934073	9.094064	7.448559	13.639653	0	11.99394
FOXC2	5.801681	11.577712	7.275648	9.07561	2.143614	7.048373	17.191846	13.070037
FOXD1	23.405026	45.506807	26.219766	35.766021	8.571177	25.316636	7.554125	35.425013
FOXD4L5	1.077438	0	0	0.078797	0	0	0	0.043325
FOXE3	0	0	0	0	0	0	0	0

FOXF1	0	1.413909	0	1.152387	0	0.047151	0	0.113887
FOXF2	8.340663	13.710483	6.217894	12.104176	2.933725	12.771989	7.418558	12.037501
FOXH1	0	0	0	0	0	0	0	0
FOXI1	0	0	0	0	0	0	0	0
FOXJ1	0	0.167431	0	0.255417	0	0	0	0.105598
FOXJ2	14.755166	29.438856	10.607938	32.329787	10.135393	32.36025	1.869615	24.647783
FOXJ3	28.92298	24.860768	38.121132	30.693963	21.62531	26.728243	42.852057	33.772394
FOXK1	75.046563	75.330465	81.139194	89.329892	56.968209	168.925496	29.697139	94.554627
FOXK2	38.69798	116.575665	88.069893	101.050097	70.41844	148.114448	73.474084	102.256288
FOXL1	11.287542	7.82943	0	8.637344	0	9.276165	0	2.259204
FOXL2	13.85264	33.404251	20.681678	28.69899	7.616594	29.027967	5.047161	21.60688
FOXL2NB	4.0302	7.250864	2.170409	8.59063	0.851464	6.441157	0	2.692157
FOXL3	0	0	0	0	0	0	0	0
FOXM1	7.574673	60.436714	18.056402	63.36368	79.778884	130.218823	67.655937	97.518254
FOXN1	0	0	0	0	0	1.400744	0	1.168431
FOXN2	9.599694	8.086003	19.371649	9.375235	28.072229	5.512201	0	12.318089
FOXN3	9.464307	15.094997	12.932041	12.360623	38.139288	37.91295	16.083075	18.707659
FOXN3P1	0	0	0	0	0	0	0	0
FOXN3P2	0	0	0	0	0	0	0	0
FOXN4	0	0	0	0	0	0	0	0.090467
FOXO1	7.323018	5.462638	4.556723	6.969399	5.804384	5.099611	7.962427	8.626818
FOXO1B	0	0	0	0	0	0.1153	0	0
FOXO3	38.783089	35.898318	30.952487	43.544496	52.589939	41.67224	31.257063	39.655714
FOXO3B	0	0	0	9.273013	0	9.565839	0	4.682817
FOXO4	0	0	0	0	0	2.197154	0	1.817184
FOXO6	0	0.786305	0	0.263037	0	1.72243	0	2.567684
FOXP1	0	30.780441	6.362272	35.465952	21.336386	43.039349	56.528146	36.996513
FOXP2	0	0	0	0.148732	0	0	0	0
FOXP3	0	0.752339	0	0.281111	0	0.438919	0	0
FOXP4	2.896784	4.110352	4.091224	4.958931	5.333486	10.64226	17.776735	4.450797
FOXQ1	17.811866	46.380339	19.36174	42.319253	11.74963	31.918379	20.07382	22.225738
FOXR1	0	0.598981	0	0	0	0	0	0
FOXRED1	5.998352	0.60787	0	0.886389	10.537353	1.413587	28.963918	1.636142

FOXRED2	0	4.041446	0	10.076015	2.137724	14.091264	0	10.503945
FPGS	6.716864	43.074676	29.26008	43.256691	19.84612	60.276971	56.838891	34.67945
FPGT	14.142084	5.224407	0	0.668164	35.457475	1.57367	0	1.754517
FPGT-TNNI3I	0	0.301638	4.669765	7.464188	0	5.828312	0	11.66499
FPR1	0	0	0	0	0	0	0	0
FPR2	0	0	0	0	0	0	0	0
FPR3	0	0	0	0	0	0	0	0
FRA10AC1	10.345502	11.410263	7.724608	10.79252	6.825507	7.034111	32.00652	9.70488
FRAS1	2.812906	2.45321	3.004635	2.346159	4.355895	2.86899	0.932269	3.156143
FRAT1	7.009701	0	0	0	0	0	0	0
FREM1	0	0	0	0	0	0	0	0
FREM2	0.197758	0	0	0.08722	0	0.009953	0	0.047498
FREM3	0	0	0	0	0	0	0	0
FREY1	0	0	0	0	0	0.519601	0	0
FRG1	56.119699	127.672229	51.780222	91.645273	61.885443	100.897317	0	134.420151
FRG1BP	11.759542	40.120677	21.95371	35.598211	8.781157	30.05215	1.294468	67.515382
FRG1DP	0	6.689864	0	2.978465	0	0	0	12.279175
FRG1EP	0	0	4.704998	0.469958	0	1.65614	0	0.830241
FRG1FP	0	0	0	0	0	0	0	0.483938
FRG1GP	0	5.943516	0	4.641198	13.977827	0.672971	0	5.512615
FRG1HP	0	0	0	3.306887	0	3.953262	0	1.631089
FRG1JP	0	0.922895	0	1.935589	0	1.469508	0	0.372017
FRG1KP	0	0	0	0	0	0	0	0
FRG2	0	0.069689	0	0	0	0	0	0
FRG2B	0	0	0	0	0	0	0	0
FRG2C	0	0.07059	0	0	0	0	0	0
FRG2DP	0	0	0	0	0	0	0	0
FRG2EP	0	0	0	0	0	0	0	0
FRG2FP	0	0	0	0	0	0	0	0
FRG2GP	0	0	0	0	0	0	0	0
FRG2HP	0	0	0	0	0	0	0	0
FRG2IP	0	0	0	0	0	0	0	0
FRG2JP	0	0	0	0	0	0	0	0

FRG2KP	0	0	0	0	0	0	0	0
FRG2LP	0	0	0	0	0	0	0	0
FRG2MP	0	0	0	0	0	0	0	0
FRK	2.391566	1.992255	2.584218	2.384422	5.563791	3.970188	14.189111	6.195388
FRMD1	0	0	0	0	0	0	0	0
FRMD3	0	0.893816	0	1.799074	0	0	0	0.077738
FRMD4A	5.306817	54.869066	11.145071	31.961491	8.037453	23.73021	0	16.528014
FRMD4B	14.572076	72.499897	14.821457	62.597182	55.950143	48.954907	8.440156	55.33797
FRMD5	12.173599	12.874824	7.086357	7.711615	20.20067	10.859817	1.264491	15.567851
FRMD6	102.959817	234.611891	87.644319	260.527396	79.102105	291.164101	108.840051	256.526959
FRMD7	0	0.139377	0	0	0	0	0	0
FRMD8	9.464143	18.294794	11.305644	18.342029	46.074168	84.713485	0	71.166307
FRMD8P1	0	0	0	0	0	0	0	0
FRMPD1	0	0.08398	0	0.031873	0	0.359189	0	0.627968
FRMPD2	0	0.033678	0	0.191919	0	0.235775	0	0.534733
FRMPD2B	0	0	0	0	0	0	0	0
FRMPD3	0.452124	0.895146	0.813254	1.02875	0	0.09076	0	0.036245
FRMPD4	0	0.357874	0	0.352886	0	0.166947	0	0.40226
FRRS1	0	0	0	0	0	0	0	0
FRRS1L	0	0	0	0	0	0.047474	0	0.038227
FRS2	9.242758	11.786803	10.238679	6.025848	12.166528	10.133328	11.426441	27.678547
FRS3	0	2.027183	1.473799	1.693617	0	1.7265	0	0.829969
FRY	0	0.106363	0	1.107929	0	0	0	0
FRYL	14.86617	77.59646	42.474283	107.895867	21.346458	72.573469	69.218294	78.827646
FRZB	0	0	0	0	0	0.063878	0	0
FSBP	6.658089	1.426569	4.351175	1.155045	0.205452	0.234316	0	1.006208
FSCN1	389.083143	413.243395	394.221574	414.244254	335.853438	434.267215	255.469725	350.760322
FSCN1P1	0	0	0	0	0	0	0	0
FSCN2	2.305055	1.549347	0	0.465869	0	0.679966	0	0.186557
FSCN3	0	0	0	0.17335	0	0	0	0
FSD1	4.346295	21.284811	26.94299	20.337708	8.561739	26.03082	68.930035	20.247538
FSD1L	2.063594	2.105171	5.517158	2.071916	2.058585	1.944568	0	1.429209
FSD2	0	0.494798	0	0	0	0	0	0

FSHB	0	0	0	0	0	0	0	0
FSHR	0	0	0	0	0	0	0	0
FSIP1	0	3.414986	3.455817	5.251829	0	2.396083	0	1.358775
FSIP2	1.239749	0.141762	0	0.089834	0	0	0	0
FSIP2LP	0	0	0	0	0	0	0	0
FST	256.975132	428.627241	203.360581	531.747652	141.659442	265.619909	71.885569	263.565952
FSTL1	18.343894	36.791782	80.443193	77.827942	113.160271	157.734502	104.540508	137.508899
FSTL3	31.018303	55.984646	36.14613	75.923188	49.876439	80.080529	29.215297	47.521893
FSTL4	1.814101	10.574131	1.630131	9.013654	1.118278	10.268832	1.400288	7.739411
FSTL5	1.151855	0	0	0.112271	0	0	0	0
FTCD	0	1.120133	0	0.42755	0	0	0	0
FTCDNL1	0	0	0	2.199134	0	0.65552	0	0.660606
FTH1	713.001252	537.01709	827.163916	681.16392	5156.79205	3258.3751	3747.46344	2402.28648
FTH1P1	0	0.34932	0	0	0	0	0	0
FTH1P10	0	0	0	0	0	0.466967	2.078406	0.466493
FTH1P11	0	0.360767	0	0.20912	0	0.589905	0	0.364925
FTH1P12	0	0.333983	0	0	0	0	0	0
FTH1P13	0	0	0	0	0	0	0	0
FTH1P14	0	0	0	0	0	0	0	0
FTH1P15	0	0	0	0	0	0	0	0
FTH1P16	0	0	0	0.599924	2.227543	0.393687	0	1.072479
FTH1P19	0	0	0	0	0	0	0	0
FTH1P2	0	8.090738	0	1.870484	3.734198	1.833723	0	1.91801
FTH1P20	0	7.565972	15.245628	5.034699	0	3.545636	0	2.477433
FTH1P21	0	0	0	0	0	0	0	0
FTH1P22	0	0	0	0	0	0	0	0
FTH1P23	0	2.067231	7.494928	0.201981	2.243283	0.397145	0	2.170773
FTH1P24	0	0	0	0	0	0	0	0
FTH1P25	0	0	0	0	0	0	0	0
FTH1P26	0	0	0	0	0	0	0	0
FTH1P27	0	0	0	0	0	0	0	0
FTH1P28	0	0	0	0	24.843841	0	0	0
FTH1P29	0	0	0	0	0	0	0	0

FTH1P3	0	0.941516	0	0.184899	0	0	0	0.328914
FTH1P4	0	0	0	0	0	0	0	0.356023
FTH1P5	0	0	0	0	0	0.406421	2.072101	0
FTH1P6	0	0	0	0	0	0	0	0
FTH1P7	0	0.359234	0	0	0	0	0	0.377818
FTH1P8	0	0	0	0.224485	0	0	0	0.402484
FTH1P9	0	0	0	0	0	0	0	0
FTHL17	0	0	0	0	0	0	0	0
FTHL18P	0	0	0	0	0	0	0	0
FTL	1765.2688	531.555186	1869.3499	589.335466	1459.7046	472.455048	1103.55232	397.280822
FTLP1	0	0	0	0	0	0	0	0
FTLP10	0	0	0	0	0	0	0	0
FTLP12	0	0	0	1.086209	0	0	0	0
FTLP13	0	0	0	0	0	0	0	0
FTLP14	0	0	0	0.968377	0	0.472958	0	0
FTLP15	0	0	0	0	0	0	0	0
FTLP16	0	0	0	0	0	0	0	0
FTLP17	0	0	0	0	0	0	0	0
FTLP18	0	0	0	0	0	0	0	0
FTLP19	0	0	0	0	0	0	0	0
FTLP2	0	0	0	0	0	0	0	0
FTLP20	0	0	0	0	0	0	0	0
FTLP3	0	0	0	0	0	0	0	0.775185
FTLP4	0	0	0	0	0	0	0	0
FTLP5	0	0	0	0	0	0	0	0
FTLP6	0	0	0	0	0	0	0	0
FTLP8	0	0	0	0	0	0	0	0
FTMT	0	0	0	0	0	0	0	0
FTO	88.516991	82.998828	99.107482	104.878048	68.494613	61.415731	34.46725	70.827623
FTOP1	0	0	0	0.111123	0	0	0	0
FTSJ1	58.783186	177.862646	61.662641	176.480308	105.347104	260.125229	146.467431	253.447686
FTSJ3	29.952492	54.5084	32.74089	43.845601	55.097811	47.750815	92.116943	30.403242
FUBP1	66.990108	57.823919	69.671139	45.643445	26.666329	43.972655	173.426275	32.967042

FUBP3	73.365736	135.578938	89.834934	136.33812	43.709686	104.784631	78.29811	101.645734
FUCA1P1	0	0	0	0	0	0	0	0
FUCA2	0	0	0	1.273524	0	10.009658	110.134144	0
FUNDC1	18.106202	24.381713	9.597249	18.269531	25.68695	17.374744	15.615806	32.459952
FUNDC2	22.99905	71.093405	39.119429	66.960644	56.096279	62.813876	68.297375	75.066168
FUNDC2P1	0	0	0	0	0	0	0	0
FUNDC2P2	0	0	0	0	0	0	0	0
FUNDC2P3	0	0	0	0	0	0	0	0
FUNDC2P4	0	0	0	0	0	0	0	0.559481
FUOM	0	0.912775	0	3.408746	0	0	0	0
FURIN	29.101595	52.521203	23.299192	50.804872	41.544192	119.320905	7.466262	82.946011
FUS	32.44154	102.374432	106.861832	91.465541	227.363582	107.10576	135.288902	109.525549
FUT1	0	0	0	0	0	0	0	0
FUT10	5.874224	18.494551	13.958079	15.122567	7.421574	16.217591	0	17.364892
FUT11	23.214831	47.406782	31.861907	57.667701	33.636443	53.408766	71.91538	79.127967
FUT2	0	1.148278	0	1.276687	11.803702	0	0	0.379279
FUT3	5.143497	2.675195	4.592879	1.877106	0.452019	3.168726	0	8.636604
FUT5	0	0	0	0.130787	0	0	0	0
FUT6	0	0	0	0	0	0	0	0
FUT8	91.061202	243.860287	124.307422	310.793887	77.700546	136.436968	107.733518	255.621263
FUT9	0.99987	0.77671	0.90029	0.446977	0.528653	0.176025	0	0.310278
FUZ	11.69675	22.899885	7.384429	14.044841	68.059059	22.498865	19.434661	27.957516
FXN	36.338587	16.751959	6.729313	11.762435	1.825912	17.492315	6.249188	24.104765
FXNP1	0	0	0	0	0	0	0	0
FXNP2	0	1.34574	0	0	0	0	0	0
FXR1	184.95785	139.959702	223.501757	142.375027	164.233503	78.56594	469.254878	117.562328
FXR2	0	8.018956	0	2.067133	0	9.79964	10.349853	0
FXYD1	0	0	0	0	0	0.436671	0	0
FXYD2	0	0.160675	3.443257	1.555711	0	0	0	1.60307
FXYD3	208.852215	190.178232	196.996984	237.78192	212.414199	207.224995	126.538978	108.86786
FXYD4	0	0	0	0	0	0	0	0
FXYD5	7.111801	2.045994	0	17.792892	0	29.377201	134.062405	12.861126
FXYD6	14.764143	29.494257	43.221141	52.127008	6.66798	5.196003	0	1.278973

FXYD6-FXYD	0	0	0	0	0	0	0	0
FXYD6P1	0	0	0	0	0	0	0	0
FXYD6P2	0	0	0	0	0	0	0	0
FXYD6P3	0	0	0	0	0	0	0	0
FXYD7	0	0	0	0	0	0	0	0
FYB1	22.364517	16.123631	9.128736	39.249784	1.50631	2.870403	0	0.677237
FYB2	0	0	0	0	0	0	0	0
FYCO1	9.080785	15.873104	13.276928	19.366324	24.015153	20.220416	26.174082	14.320483
FYN	20.992534	102.967284	46.642771	112.305863	37.729584	185.613438	3.028205	116.365685
FYTTD1	72.51662	124.425298	97.894041	103.466712	93.266545	112.182984	168.100608	121.00857
FYTTD1P1	0	0	0	0.102303	0	0	0	0
FZD10	0	0	0	0	0	0	0	0
FZD3	7.068579	7.384314	1.674965	3.874666	6.017714	5.263587	2.497866	14.659791
FZD4	3.079609	4.257281	3.553217	3.169797	3.237758	2.042545	4.497538	3.033959
FZD5	5.977072	7.796068	6.615833	5.815578	7.897445	13.49712	7.287578	16.721503
FZD6	42.485813	57.842469	43.916503	48.604558	45.583461	25.027769	81.791174	41.659704
FZR1	0	39.639245	19.500642	36.643218	23.974275	92.485571	72.952062	55.468323
G0S2	132.008391	206.095812	116.012109	175.968504	21.4881	37.566864	30.378167	54.604833
G2E3	50.863828	95.95142	45.759105	51.421266	119.460426	70.426303	148.59792	96.783205
G2E3P1	0	0	0	0	0	0	0	0
G3BP1	145.597225	96.166598	121.234324	81.188803	152.964834	89.279302	23.521567	122.367836
G3BP1P1	0	0.10867	0	0.188163	0	0.5088	0	0.420971
G3BP2	144.933312	174.851723	89.636678	138.649245	109.745277	102.343188	117.85177	181.327545
G6PC1	0	0	0	0	0	0	0	0
G6PC2	0	0	0	0	0	0	0	0
G6PC3	0	0.20367	0	0.356413	0	0	0	0
G6PD	0	25.21849	0	26.901664	97.71092	32.268592	33.26641	21.783762
GAA	19.668004	16.705486	61.246188	30.309649	3.709171	7.335869	11.685273	5.395662
GAB1	0	6.473269	5.858292	12.463786	3.121685	4.666665	66.571122	17.920277
GAB2	0.537104	1.593298	2.414106	3.805412	0.709568	1.786699	2.487407	5.450633
GAB3	0.777029	0.45973	0.697545	0.356643	0	0.19427	1.960132	0.035969
GAB4	0	0	0	0	0	0	0	0
GABARAP	151.533086	197.986875	168.728885	216.516391	209.601386	223.756092	229.019911	202.988848

GABARAPL1	38.949409	75.627943	44.681479	135.71017	103.328016	118.291298	51.77774	53.799756
GABARAPL2	107.669546	195.863252	105.46995	200.854784	73.285414	164.786723	29.555482	197.597736
GABARAPL3	0	0	0	0	0	0	0	0
GABBR1	1.94756	0.993149	2.9154	1.613942	5.357738	2.269451	0	1.403924
GABBR2	0.592983	0.076424	9.567568	0.101474	0	0.614163	0	0.047571
GABPA	0	4.958707	9.401631	5.293656	0	0.835568	13.086997	1.390528
GABPAP	0	0	0	0	0	2.080406	6.385669	0
GABPB1	18.620973	30.45391	19.321617	34.094529	57.036683	30.895703	26.755027	43.530633
GABPB2	8.014402	22.569614	4.933367	23.100975	21.431629	23.699257	23.650446	23.268363
GABRA1	0	0	0	0	0	0	0	0
GABRA2	0	0	0	0.013693	0	0.065161	0	0
GABRA3	0	0.232146	0	0.357779	0	0.812794	6.379082	1.198954
GABRA4	0	0	0	0	0	0	0	0
GABRA5	0	0	0	0	0	0	0	0
GABRA6	0	0	0	0	0	0	0	0
GABRB1	0	0	0	0.175751	0	0	0	0
GABRB2	0	0	2.401613	0.265544	0	0	0	0.071483
GABRB3	0	0.485082	0	0	0	0	0	0
GABRD	0	0.264903	0	0.328378	0	0	0	0
GABRE	0	2.962311	0	1.405016	1.320263	0.397137	0	0.717078
GABRG1	0	0	0	0	0	0	0	0
GABRG2	0	0.191495	0	0.638537	0	0	0	0.541238
GABRG3	0	0	0	0	0	0	0	0
GABRP	0	0	0	0	0	0	0	0
GABRQ	0.50155	1.488529	0.901904	1.557981	0	1.081731	0	0.382062
GABRR1	0	0	0	0	0	0	0	0
GABRR2	0	0	0	0.050676	0	0	0	0
GABRR3	0	0	0	0	0	0	0	0
GAD1	11.18498	7.472941	0	4.622637	41.942779	9.627428	0	9.279944
GAD2	0	0	0	0	0	0	0	0
GAD3P	0	0	0	0	0	0	0	0
GADD45A	186.253459	370.181161	183.441661	364.178064	164.498729	199.363234	57.815237	168.299088
GADD45B	23.890335	55.820637	35.366719	100.961065	21.657432	53.580614	74.467972	38.777138

GADD45G	0	0.163367	0	0.343495	0	0.519624	0	0.436504
GADD45GIP1	29.821503	33.377371	26.996006	41.807267	35.193779	105.188502	33.164806	65.167376
GADL1	0	0	0	0	0	0	0	0
GAGE1	0	0	0	0	0	0	0	0
GAGE12B	0	0	0	0	0	0	0	0
GAGE12F	0	0	0	0	0	0	0	0
GAGE2E	0	0	0	0	0	0	0	0
GAK	23.658044	31.561118	8.601531	30.399408	33.022411	30.163404	19.451662	19.293346
GAL	55.741887	79.042338	34.093368	57.343459	11.670904	32.859408	0	62.417789
GAL3ST1	0	0	0	0.157821	0	0.226178	0	0.754215
GAL3ST2	0	0.383221	0	0.222022	0	0.748599	0	0
GAL3ST3	0	0	0	0	0	0.748174	0	0.977214
GAL3ST4	0	2.096908	2.294953	2.123121	2.319683	2.747351	0	0.944922
GALC	19.453595	12.094156	16.449021	13.812892	9.690924	5.415646	0	4.764874
GALE	60.114153	88.309529	76.991736	77.923894	53.146829	79.536395	39.320754	69.027251
GALK1	0	6.835428	7.95211	8.677082	0.657831	6.006926	0	4.058047
GALK2	66.295873	54.896336	53.066582	64.76547	15.812154	29.591499	26.94912	41.014925
GALM	4.845939	0	0	0.675354	0	1.416346	0	0.694485
GALNS	77.879598	22.677031	15.655939	34.95895	73.744912	28.144112	10.818757	15.700515
GALNT1	139.753354	291.309786	86.053221	175.474544	51.770896	149.616581	137.598703	229.321718
GALNT10	44.038918	102.009842	25.51414	58.049546	15.732696	43.647767	5.374282	53.009572
GALNT11	7.40065	28.991659	14.357265	29.082369	60.466715	45.947544	29.743382	38.090439
GALNT12	14.6579	20.998836	16.406688	22.253758	16.439032	10.474565	0	5.097599
GALNT13	0	1.065486	0	0.014079	0	0	0	0
GALNT14	25.724154	48.669802	18.686388	60.697519	59.03407	67.593788	86.369198	42.023583
GALNT15	0	0	0	0.570517	0	0	0	0
GALNT16	0	0.034409	0	1.005942	0	0	0	0
GALNT17	0	0	0	0	0	0	0	0
GALNT18	40.768343	110.065216	27.966001	103.949885	33.700379	107.397416	12.528787	97.699758
GALNT2	103.344697	164.164544	107.477465	189.129201	73.8348	155.857192	37.107036	79.507304
GALNT3	66.059897	52.358295	61.880059	38.257897	68.80223	25.039875	81.08366	41.711409
GALNT4	3.635874	2.636336	3.267133	2.602626	0.219279	3.632317	4.825313	5.682236
GALNT5	1.861309	4.146907	2.792266	5.71991	6.806029	7.476357	6.208304	7.380632

GALNT6	39.357589	138.086652	18.722354	188.347032	10.608483	113.645447	16.300869	80.792198
GALNT7	14.73086	16.17617	21.61595	23.454342	38.057219	18.927857	99.049229	20.264108
GALNT8	0	0	0	0	0	0	0	0
GALNT9	0	0	0	0.423397	0	0	0	0
GALNTL5	0	0	0	0	0	0	0	0
GALNTL6	0	0	0	0	0	0	0	0
GALP	0	0	0	0	0	0	0	0
GALR1	0	0	0	0	0	0	0	0
GALR2	0	1.440811	0	2.048618	0	0.259593	0	0
GALT	0	6.377218	0	5.933109	34.825255	2.058048	26.084301	4.590691
GAMT	0	0.230497	11.921787	3.909389	0	7.639749	0	0.350643
GAMTP2	0	0	0	0	0	0	0	0
GAN	0	2.467938	7.393063	9.07578	10.160938	17.307391	28.644901	10.84389
GANAB	88.548555	65.885782	170.494792	133.740648	231.010195	167.216403	116.704114	101.303477
GANC	21.585681	29.385942	15.031041	34.995462	25.609238	33.932972	0	38.932255
GAP43	0	0	0	0	0	0	0	0
GAPDH	6499.35418	2434.87087	6413.44195	2447.04774	5440.7951	2381.40605	4445.7517	2372.66464
GAPDHP1	0	0	0	0.27656	1.008043	0.185717	0.918207	0.470108
GAPDHP14	0	0	0	0.200367	0	2.418348	0	2.393327
GAPDHP15	0	0	0	0	0	0	0	0
GAPDHP16	0	0	0	0	0	0	0	0
GAPDHP17	0	0	0	0	0	0	0	0
GAPDHP19	0	0	0	0	0	0	0	0
GAPDHP2	0	0	0	0	0	0	0	0
GAPDHP20	0	0	0	0	0	0	0	0
GAPDHP21	0	0	0	0	0	0	0	0
GAPDHP22	0	3.001662	0	3.100153	0	0.598688	0	1.736691
GAPDHP23	0	0	0	0	0	0	0	0
GAPDHP24	0	0	0	0	0	0	0	0
GAPDHP25	0	0	0	0	0	0	0	0
GAPDHP26	0	0	0	0	0	0	0	0
GAPDHP27	0	0	0	0	0	0	0	0
GAPDHP28	0	0	0	0	0	0	0	0

GAPDHP29	0	0.80909	0	0	0	0	0	0
GAPDHP30	0	0	0	0	0	0	0	0
GAPDHP31	0	0	0	0	0	0	0	0
GAPDHP32	0	0	0	0.278544	0	0.187066	0	0
GAPDHP33	0	0	0	0.182411	0	0	0	0
GAPDHP34	0	0	0	0	0	0	0	0
GAPDHP35	0	0.327126	0	0.095367	0	0.57611	0	0
GAPDHP36	0	0	0	0	0	0	0	0
GAPDHP37	0	0	0	0	0	0	0	0
GAPDHP38	0	0	0	0	0	0	0	0
GAPDHP39	0	0	0	0	0	0	0	0
GAPDHP40	0	0	0	0	0	0	0	0
GAPDHP41	0	0	0	0	0	0	0	0
GAPDHP42	0	0	0	0	0	0	0	0
GAPDHP43	0	0	3.397973	0.092404	4.045553	0.18619	0	0
GAPDHP44	0	0.946229	0	0	0	0	0	0
GAPDHP45	0	0	0	0	0	0	0	0
GAPDHP46	0	0	0	0	0	0	0	0
GAPDHP47	0	0	0	0	0	0	0	0
GAPDHP48	0	0	0	0	0	0	0	0
GAPDHP49	0	0	0	0	0	0	0	0
GAPDHP50	0	0	0	0	0	0	0	0
GAPDHP51	0	0	0	0	0	0	0	0
GAPDHP52	0	0	0	0	0	0	0	0
GAPDHP53	0	0	0	0	0	0	0	0
GAPDHP54	0	0	0	0	0	0	0	0
GAPDHP55	0	0	0	0	0	0	0	0
GAPDHP56	0	0	0	0	0	0	0	0
GAPDHP57	0	0	0	0	0	0	0	0
GAPDHP58	0	0	0	0	0	0	0	0
GAPDHP59	0	0	0	0	0	0	0	0
GAPDHP60	0	0	0	0	0	0	0	0
GAPDHP61	0	0	0	0	0	0	0	0

GAPDHP62	0	0	0	0.441186	0	0	0	3.06872
GAPDHP63	0	0	0	0	0	0	0	0
GAPDHP64	0	0	0	0	0	0	0	0
GAPDHP65	0	0	0	0	0	0	0	0
GAPDHP66	0	0	0	0	0	0	0	0
GAPDHP67	0	0	0	0	0	0	0	0
GAPDHP68	0	0	0	0	0	0	0	0
GAPDHP69	0	0	0	0	0	0	0	0.623768
GAPDHP70	0	0	0	0	0	0	0	0
GAPDHP71	0	0	0	0	0	0	0	0
GAPDHP72	0	0	0	0	0	0	0	0
GAPDHP73	0	0	0	0	0	0	0	0
GAPDHP74	0	0	0	0	0	0	0	0
GAPDHP75	0	0	0	0	0	0	0	0
GAPDHP76	0	0	0	0	0	0	0	0
GAPDHP77	0	0	0	0	0	0	0	0
GAPDHS	0	0	0	0	0	0	0	0
GAPT	0	0	0	0	0	0	0	0
GAPVD1	16.832644	61.912201	62.016175	66.893027	29.987502	51.381066	54.213479	82.979478
GAR1	61.963155	28.618383	32.346784	24.212239	91.084336	21.236232	0	33.618055
GAREM1	33.238054	27.176256	4.96621	23.533077	2.310504	11.094818	0.112215	11.931788
GAREM2	3.959324	6.331389	3.554019	3.986092	0	13.798626	0	8.24777
GARIN1A	0	0	0.509023	0	18.80086	0	0	0.068142
GARIN1B	0	0	0	0	0	0	0	0
GARIN2	0	0	0	0	0	0	0	0
GARIN3	0	0	0	0	0	0	0	0
GARIN3P1	0	0	0	0	0	0	0	0
GARIN5A	0	1.007775	8.377759	0	6.368195	0.307774	0	0
GARIN5B	0	0	0	0	0	0	0	0
GARIN6	0	0	0	0	0	0	0	0
GARNL3	0	7.904698	0	5.47315	0	1.985563	0	0.299774
GARRE1	12.567897	1.062417	0	9.48802	0	15.569714	0.540158	14.585193
GARS1	187.197186	228.42671	205.361363	219.825639	131.761299	118.119314	172.357152	179.764993

GARS1P1	0	0	0	0	0	0	0	0
GART	194.525541	109.474444	91.945053	159.084239	256.928404	185.376483	317.679034	155.437982
GAS1	0	2.091033	0	0.90834	0	4.29709	0	3.124093
GAS2	0	0.724575	1.417918	2.154387	11.621706	1.702851	0	2.719102
GAS2L1	12.553281	14.348928	3.471446	10.328127	4.046287	39.895987	10.781865	31.507897
GAS2L1P1	0	0	0	0	0	0	0	0
GAS2L1P2	0	0	0	0	0	0.225631	0	0.109009
GAS2L3	10.553199	16.739061	14.051797	12.451135	75.068263	29.159897	33.837422	48.510998
GAS6	13.48422	24.097299	8.493224	22.845932	51.515339	78.385193	4.971131	57.646111
GAS7	0	1.201858	0.36263	1.051623	0	3.118205	30.527438	1.79492
GAS8	16.686416	21.859199	21.300947	33.896898	33.427726	48.946749	0	32.173334
GASK1A	0	0	0	0	0	0	0	0
GASK1B	6.505699	22.815664	11.463689	6.099183	0	4.329612	0	1.470458
GAST	0	0	0	0.25726	0	1.503554	0	1.413408
GATA1	0	0	0	0	0	0	0	0
GATA2	0	15.654421	0	19.358071	19.690982	33.765966	0	17.257805
GATA3	2.555901	16.310005	8.767632	29.676625	2.473003	8.312706	29.764242	5.865061
GATA4	0	0	0	0.045918	0	0	0	0
GATA5	0	2.25782	2.315902	2.047361	0	0.064318	0	0
GATA6	0	0	0	4.453072	0	6.155463	0	2.23956
GATAD1	20.833804	18.136518	12.902702	18.84348	13.093016	11.795951	1.749287	13.420224
GATAD2A	119.821898	195.699943	36.722893	189.901036	102.147036	426.465927	187.094231	371.045218
GATAD2B	31.421921	62.473813	43.005389	70.928904	90.254806	76.161689	11.881881	49.935032
GATB	16.767651	17.228448	19.743474	18.530433	30.745584	10.300132	88.49942	15.451501
GATC	34.983535	27.722523	33.585872	18.884141	32.459778	21.794442	22.201044	27.101948
GATD1	19.608176	16.771545	16.250505	26.10543	7.572863	21.492964	89.20686	17.948389
GATD3	0	2.411383	12.842493	5.432509	39.687835	16.999102	0	2.963802
GATM	0	1.308758	0	0.235049	0	0.497406	0	0.972484
GBA1	27.704614	30.088218	27.67959	44.773252	18.617618	19.99534	2.906177	13.775772
GBA1LP	0	0	0	0	0	0	0	0
GBA2	28.663826	13.317434	9.073794	16.612417	35.379829	25.132849	20.040959	25.678929
GBA3	0	0	0	0	0	0	0	0
GBE1	20.575496	19.947825	28.235228	13.403549	16.186402	8.384183	18.384816	20.546417

GBF1	0	0	0	0	0	0	0	0
GBGT1	0	0.626334	0	1.383211	0	2.002897	0	0.217911
GBP1	7.005862	13.971026	6.275284	21.474965	43.449849	39.606027	10.209484	35.836427
GBP1P1	0	1.400799	0	0.088527	0	0.441217	0	0.495292
GBP2	2.421836	2.412328	0.862438	7.684741	8.551942	6.159003	36.79526	4.16483
GBP3	46.620475	80.778208	18.805966	77.211043	16.568514	58.613261	58.668372	45.961365
GBP4	0.52908	0.682462	1.42689	0.918659	2.795912	1.379519	44.675708	2.771779
GBP5	0	0	0	0	0	0	0	0.126042
GBP7	0	0	0	0	0	0	0	0
GBX1	0	0	0	0.19773	0	0	0	0
GBX2	0	8.708871	1.439462	5.399941	0	3.056666	0	1.5591
GC	0	0	0	0	0	0	0	0
GCA	39.186496	27.244529	49.837449	26.77292	44.279917	12.691633	0	20.001415
GCAT	0	0.885255	0	1.370494	0	0.583293	0	0.660818
GCATP1	0	0.243841	0	0.070563	0	0.571435	0	0
GCC1	6.392736	32.371291	7.889879	31.626305	13.928229	35.034363	6.22357	36.546785
GCC2	7.895607	7.32745	4.843016	3.937866	28.501593	0	39.746736	7.102809
GCDH	2.03824	5.984488	6.74116	8.509889	19.418735	19.176733	0.719481	15.424037
GCFC2	20.64283	28.76166	23.472292	20.664619	40.330194	15.558322	48.320161	25.582283
GCG	0	0	0	0	0	0	0	0
GCGR	0	0.662989	0	0.303996	0	0	0	0
GCH1	10.439183	12.417981	9.350985	7.138354	4.285654	9.330515	0	12.099266
GCHFR	5.799722	26.929105	10.733744	15.672441	0	28.659512	43.358927	22.463584
GCK	0	0	0	0	0	0	0	0
GCKR	0	0	0	0.381615	0	0	0	0
GCLC	119.019395	225.246516	136.685678	325.384328	128.777301	199.130726	25.773194	159.151786
GCLM	189.560076	296.475835	109.542285	107.286708	18.516885	9.619424	88.748561	65.217923
GCM1	0	0	0	0	0	0	0	0
GCM1P1	0	0	0	0	0	0	0	0
GCN1	35.065648	39.438216	49.062371	37.771081	116.79714	77.406603	67.691843	55.445139
GCNA	0	0	0	0	0	0	0	0
GCNAP1	0	0	0	0	0	0	0	0
GCNT1	9.955219	72.040654	9.098248	39.464513	55.14688	67.690021	8.126525	121.209489

GCNT1P1	0	0	0	0	0	0	0	0.11508
GCNT1P2	0	0	0	0	0	0	0	0
GCNT1P3	0	0	0	0.377178	0	0	0	0
GCNT1P4	0	0	0	0	0	0	0	0
GCNT1P5	0	0	0	0	0	0	0	0
GCNT2	3.630095	24.390551	16.750544	30.42753	8.053992	33.521	6.985153	31.855417
GCNT2P1	0	0	0	0	0	0	0	0.3433
GCNT3	0	3.088772	15.805688	8.32673	2.182252	3.77857	2.107151	1.750405
GCNT4	2.554664	2.751835	1.582332	1.412008	0	0.49957	0	0.523527
GCNT7	0	0	0	0	0	0	0	0
GCOM1	0	15.993495	17.035348	21.395025	6.009887	19.784609	0	12.699336
GCSAM	0	0	0	0	0	0.050748	0	0.90713
GCSAML	0	0	0	0	0	0.122868	0	0
GCSH	94.438496	54.604531	70.867578	3.247223	120.336707	0.764516	34.677085	32.592217
GCSHP1	0	0	0	0	0	0	0	0
GCSHP2	0	0	0	0	0	0	0	0
GCSHP3	0	0	0	0	0	0	0	0
GCSHP4	0	0	0	0	0	0	0	0
GCSHP5	127.713742	70.226393	132.567922	23.898386	54.236748	10.922513	191.471275	58.227467
GCSHP6	0	0	0	0	0	0	0	0
GDA	28.928364	17.917635	17.355748	29.513947	0	3.942752	14.854764	4.499041
GDAP1L1	0	0	0	0.207243	0	0	0	0
GDAP2	15.558766	37.974502	19.016691	42.873772	72.115156	25.842559	22.498293	43.815515
GDE1	96.385271	115.576241	104.345526	96.655482	62.337378	72.897807	70.908095	124.392954
GDF1	0	0	0	0	0	0	0	0
GDF10	0	0	0	0	0	0	0	0
GDF11	0	1.526988	0	2.487858	0	4.974441	0	6.761395
GDF15	9.303216	8.510527	30.223069	11.736159	0	2.720578	0	1.260098
GDF3	0	0	0	0	0	0	0	0
GDF5	0	0	0	0.245709	0	0	0	0
GDF5-AS1	0	0	0	0	0	0	0	0
GDF6	0	0.726275	0	0.348801	0	0	0	0.035887
GDF7	0	0.014956	0	0.008495	0	0	0	0

GDF9	0	0.382384	0	0	0	0.063802	0	0
GDI1	91.753261	90.624198	102.136558	117.923749	119.951346	80.640647	50.153702	68.578599
GDI2	108.872588	68.505318	108.007905	48.124065	111.295655	25.777299	104.133025	77.282653
GDI2P1	0	0	0	0	0	0	0	0
GDI2P2	0	0	0	0	0	0	0	0
GDNF	0	0.312272	0	0.089058	0	0.227782	0	0.03666
GDPD1	0	0.220169	1.848265	2.99595	4.082116	0.693454	0	2.90687
GDPD2	0	0	0	0	0	0	0	0.405739
GDPD3	0	0.437242	0	0.508261	0	0	0	0
GDPD4	0	0	0	0	0	0	0	0
GDPD5	6.224206	19.21462	6.50914	23.191673	0.368336	14.167693	0	16.379024
GDPGP1	3.496934	2.253881	3.705893	2.538121	3.270023	0.577696	0	0.662154
GEM	1.642146	2.402083	2.811986	1.606239	0	0.714036	0	0.914418
GEMIN2	0	0	18.249582	0	0	0	51.526563	0
GEMIN2P1	0	0	0	0	0	0	0	0
GEMIN2P2	0	0	0	0	0	0	0	0
GEMIN4	20.361359	61.655339	52.956405	80.037306	43.343678	172.77137	29.299806	153.270252
GEMIN5	19.320754	20.203112	18.989148	18.142003	31.104337	16.818567	48.326666	19.376991
GEMIN6	19.536668	54.488162	21.89878	53.517387	6.408379	34.414037	0	34.938201
GEMIN7	14.231497	27.038022	23.855558	15.345302	14.151922	38.562794	29.365483	51.269556
GEMIN7P1	0	0	0	0	0	0	0	0
GEMIN8	13.815634	4.995296	1.874223	11.874303	2.21675	7.288004	0	9.620887
GEMIN8P1	0	0	0	0	0	0	0	0
GEMIN8P2	0	0	0	0	0	0	0	0
GEMIN8P3	0	0	0	0	0	0	0	0
GEMIN8P4	0	10.924972	5.043318	11.518785	0	4.104887	0	9.999041
GEN1	9.126234	32.128122	17.549004	16.916452	45.123424	17.890209	46.823023	31.531641
GET1	36.925167	36.570259	49.238877	25.226786	14.013501	13.796065	16.421195	15.687169
GET1-SH3BG	0	0	0	0	0	0	0	0
GET1P1	0	0	0	0	0	0	0	0
GET3	0	0	0	0	0	1.984215	0	0
GET4	31.41354	88.014211	50.213321	80.903682	56.672882	97.324797	11.213125	94.985797
GFAP	0	0.141516	0	0.121663	0	0	0	0

GFER	5.026852	9.180564	15.506456	12.004297	1.797099	20.503181	63.5168	10.68002
GFI1	0.721201	3.141996	1.304903	8.945203	0	0	0	0.45818
GFI1B	0	0	0	0.529274	0	0	0	0.044677
GFM1	23.163176	61.562832	48.089526	54.949878	26.482012	37.058154	53.439702	55.15652
GFM2	31.150542	53.83179	36.517018	36.85936	37.731791	21.721474	30.468644	54.615946
GFOD1	9.337045	20.699818	6.863452	12.482619	12.934051	33.285634	16.718738	35.558847
GFOD2	40.973509	80.750374	30.060389	100.576948	37.003246	117.108627	21.325028	85.440485
GFOD3P	6.6004	9.476763	5.51496	11.20648	6.450157	28.763395	15.716878	21.179852
GFPT1	29.745897	41.684319	45.775513	32.928566	21.200195	22.587445	18.386219	16.326537
GFPT2	10.069285	12.793262	5.944937	14.4574	1.738303	0.385637	20.388117	0.48919
GFRA1	0	0	0	0.020487	0	0	0	0
GFRA2	0	0	0	0	0	0	0	0
GFRA3	0	0	0	0.046251	1.507253	0.432054	0	0.422905
GFRA4	0	0	0	0	0	0	0	0
GFRAL	0	0	0	0	0	0	0	0
GFUS	30.402702	42.678618	29.080245	38.065828	28.770959	52.510846	45.128206	49.232494
GFY	0	0	0	0.045196	0	0	0	0
GGA1	33.496847	24.139121	6.472994	28.551159	38.277357	22.493467	66.651626	18.551665
GGA2	23.955046	85.43368	55.288377	92.456365	85.36457	179.196515	136.590752	114.358373
GGA3	51.355929	28.557814	57.760707	33.29705	19.585642	42.582772	40.048664	20.933978
GGACT	27.045544	19.235244	41.484661	17.473219	0	3.112315	0	3.120641
GGCT	138.083228	209.254875	132.659353	107.5634	248.332749	97.881059	192.600888	216.413779
GGCTP1	0	0	0	0.306105	0	0	0	2.328328
GGCTP2	0	0	0	0	0	0	0	0
GGCTP3	0	0	0	0	0	0	0	0
GGCX	7.297845	5.820551	10.141323	8.127992	3.001907	4.108263	3.649564	5.696294
GGH	65.146999	47.984383	50.384124	22.469966	31.649988	8.301704	0	30.05324
GGN	0	0	0	0.413237	0.531232	0	0	1.059013
GGNBP1	0	0	0	0	0	0	0	0
GGNBP2	27.820786	59.040527	43.944221	49.835036	61.115364	38.768659	58.346043	61.171762
GGPS1	41.579985	64.511922	23.046754	50.551126	17.876873	18.668215	16.047751	52.535592
GGT1	0	0.559009	0	2.708568	0	7.820997	0	2.79374
GGT3P	0	0	0	0	0	0	0	0

GGT4P	0	0	0	0	0	0	0	0
GGT5	0	0	0	0	0	0	0	0
GGT6	0	1.426166	0	1.825811	2.830383	2.005926	0	0.292816
GGT7	5.136794	3.170866	2.299042	2.251338	0	0.255411	0	0.206807
GGT8P	0	0	0	0	0	0	0	1.134678
GGTA1	0	0	0	0	0	0	0	0
GGTA2P	0	0.83269	0	0.241686	0	0	0	0
GGTLC1	0	0	0	0	0	0	0	0
GGTLC2	0	0	0	0	0	0	0	0
GGTLC3	0	0	0	0	0	0	0	0
GGTLC4P	0	0	0	0	0	0	0	0
GGTLC5P	0	0	0	0	0	0	0	0
GH1	0	0	0	0	0	0	0	0
GH2	0	0	0	0	0	0	0	0
GHDC	8.338116	3.008626	3.802277	2.329594	3.73786	3.942456	0	1.131281
GHITM	192.121294	99.981296	133.358506	68.791488	118.753219	51.456055	117.524506	76.85984
GHR	0	0.459002	0.685433	0.130669	0	1.003154	0	0.753576
GHRHR	0	0	0	0	0	0	0	0
GHRL	0	0	0	0	0	0.674695	0	0
GHSR	0	0	0	0	0	0	0	0
GID4	0	3.856289	0	6.499453	17.749915	1.309305	0	4.993073
GID8	53.468643	99.28297	56.795582	78.189102	35.205152	78.990428	22.733589	119.68088
GIGYF1	30.587437	8.71926	10.468246	11.139327	6.501288	23.984559	5.676898	13.495266
GIGYF2	28.823829	35.033784	11.047969	36.712266	126.161829	38.839162	294.833905	46.633446
GIMAP1	0	0	0	0	0	0	0	0
GIMAP1-GIM	0	0	0	0	0	0	0	0
GIMAP2	5.00545	3.250976	0	4.675625	0.658871	3.479683	0	3.548342
GIMAP3P	0	0	0	0	0	0	0	0
GIMAP4	0	0	0	0	0	0	0	0
GIMAP5	0	0	0	0	0	0	0	0
GIMAP6	0	0	0	0	0.25554	0	0	0
GIMAP7	0	0	0	0	0	0	0	0
GIMAP8	0	0	0	0	0	0.078767	0	0

GIMD1	0	0	0	0	0	0	0	0
GIN1	11.301313	7.083417	4.200031	2.885729	1.059511	1.064562	0	3.022532
GINM1	41.204104	29.915838	39.975151	14.178659	31.640636	10.193212	16.476501	21.071721
GINS1	26.203998	32.813368	26.203417	23.079896	36.450381	25.751749	25.970591	39.113991
GINS2	24.49959	70.508553	23.083785	42.704141	42.187896	67.656156	25.350328	54.650543
GINS3	0	7.004409	0	7.946766	0.434038	10.273981	17.015407	9.500138
GINS4	29.551392	31.483117	32.874984	37.311732	29.077366	35.546	10.048802	41.41723
GIP	0	0	0	0.140364	0	0	0	0
GIPC1	84.170206	152.529816	80.673132	134.491611	124.645709	392.645535	130.202307	271.691402
GIPC2	0	0	0	0	0	0	0	0
GIPC3	0	2.269754	0	0.895113	0	0	0	0
GIPR	0	0.933792	0.903853	0.936715	0	0.452539	0	0.364729
GIT1	0	12.59652	45.642877	17.433968	20.375477	3.546222	100.869362	26.1435
GIT2	20.40937	22.657087	14.214844	25.360559	31.846288	37.194793	43.450654	44.712314
GJA1	25.523463	16.736102	24.354532	23.63071	7.633332	0.704225	0	1.049185
GJA10	0	0	0	0	0	0	0	0
GJA1P1	0	0	0	0	0	0	0	0
GJA3	0	1.130057	1.125932	1.347682	0.165527	1.475036	3.66355	0.879807
GJA4	0	0	0	0.371791	0	0	0	0.177369
GJA5	0	0	0	0	0	0	0	0
GJA6P	0	0	0	0	0	0	0	0
GJA9	0	0	0	0	0	0	0	0
GJB1	0	0	0	0	0	0	0	0
GJB2	17.956339	29.7935	26.756493	47.253936	28.416054	28.113097	18.500989	55.109544
GJB3	76.190302	129.206952	72.168652	155.480195	55.592605	171.445961	75.111473	175.96106
GJB4	0	0.628738	0	1.511619	6.583506	24.294033	0	34.745808
GJB5	10.762955	29.356159	9.557993	29.504491	27.616753	42.812925	26.5668	43.205017
GJB6	5.028614	17.812494	9.300993	14.905356	16.425289	21.899116	0	15.622774
GJB7	0	0	0	0	0	0	0	0
GJC1	46.016398	140.853904	49.380143	129.739501	54.090102	113.746906	19.160352	133.371845
GJC2	0	0.4047	0	0.270652	0	0.394137	0	0.320681
GJD2	0	0	0	0	0	0	0	0
GK	6.708386	8.937822	28.420979	13.186315	12.737918	9.006024	0	19.945258

GK4P	0	0	0	0	0	0	0	0
GK5	32.755509	20.582677	6.185222	7.258645	23.177579	16.970695	54.119804	35.812273
GKAP1	1.948766	3.898578	0	1.777582	0	0.707169	0	2.738784
GKN1	0	0	0	0	0	0	0	0.330125
GKN2	0	0	0	0	0	0	0	0
GKN3P	0	0	0	0	0	0	0	0
GLA	0	1.810263	0	0	0	0.590054	0	0.778499
GLB1	41.2818	26.900811	47.515881	39.665761	47.762912	36.363354	34.638944	16.647166
GLB1L	18.548909	6.87228	20.50079	9.739446	0	6.334407	2.709946	9.036723
GLB1L2	0	0	0	1.794982	0	0	23.79718	0.107029
GLB1L3	0	0.039855	0	0.02472	9.898066	0.418644	0	0.373336
GLCCI1	20.970326	15.593993	12.918099	9.896523	21.642865	14.395209	12.360204	9.796741
GLCE	15.178681	31.01697	10.061399	27.752633	23.065841	25.555912	13.68352	37.224251
GLDC	9.987937	1.609466	0	0.262746	4.254469	5.745761	8.683669	3.297287
GLDCP1	0	0	0	0	0	0	0	0
GLDN	0	0	0	0	0	0	0	0
GLE1	0	0	0	9.754475	9.071363	23.038591	0	3.42755
GLG1	11.651107	13.169028	24.088351	37.900538	83.869042	59.809047	97.488596	37.474782
GLI1	0	2.290901	0.937855	2.421437	0.276199	0.166309	0	2.213008
GLI2	0	0.662976	1.199594	0.123337	1.058306	0	0	0
GLI3	3.72656	4.815567	3.449377	2.663561	13.415419	19.842348	6.079359	15.249039
GLI4	18.066729	11.356728	7.535979	15.93718	5.635098	12.121234	0	8.099419
GLIPR1	55.12293	28.453114	37.946009	27.01537	21.051686	13.4032	3.275541	22.212178
GLIPR1L1	0	0	0	0	0	0	0	0
GLIPR1L2	0	0.154966	0	0.761219	0	0	0	0.164843
GLIPR2	4.313581	2.257254	1.921098	2.46356	9.0906	2.481598	0	3.271871
GLIS1	0	0	0	0	0	0	0	0
GLIS2	3.677532	2.410172	9.245967	4.920048	1.165585	5.676706	4.028022	4.216429
GLIS3	6.331737	8.664751	2.185744	8.548058	1.027713	4.760798	0	1.524905
GLMN	20.022472	12.50519	10.809887	3.504106	11.41522	1.454751	0	7.056502
GLMP	21.343704	9.801021	47.934055	10.812468	48.803431	6.889252	0	4.017933
GLO1	307.900232	164.093272	207.349731	94.365021	162.804533	52.005553	142.02147	98.698816
GLOD4	84.710895	79.766546	31.128945	67.19086	66.956437	94.514813	80.875141	56.269085

GLOD5	0	0	0	0.403517	0	0	0	0
GLP1R	0	0	0	0	0	0	0	0
GLP2R	3.142198	0.930469	0.70516	1.467682	0	0.248071	0	0
GLRA1	0	0	0	0	0	0	0	0
GLRA2	0	0	0	0	0	0	0	0
GLRA3	0	0	0	0	0	0	0	0.325039
GLRB	8.062294	8.251901	1.785894	1.920859	2.70694	2.225992	24.4888	5.02194
GLRX	10.826884	9.496665	14.833046	10.907912	26.418933	16.757769	41.231531	9.374692
GLRX2	3.216937	1.197185	1.145023	0.644888	3.569644	0.195328	0	1.101977
GLRX3	134.426194	87.901892	187.111191	64.573619	170.5805	55.353542	66.877756	122.837294
GLRX3P2	0	0	0	0	0	0	0	0
GLRX5	72.521075	137.305886	69.732794	87.373894	33.336455	102.007289	80.574579	113.892615
GLRX5P1	0	0	0	0	0	0	0	0
GLRX5P2	0	0	0	0	0	0	0	0
GLRX5P3	0	0	0	0	0	0	0	0
GLRXP1	0	0	0	0	0	0	0	0
GLRXP2	0	0	0	0	0	0	0	0
GLRXP3	0	0	0	0	0	0	0	0
GLS	111.370669	92.920817	119.701755	49.842485	93.375384	99.456436	267.311731	253.32634
GLS2	40.816438	21.759517	26.289771	16.325579	4.500061	2.991281	0.628419	2.798033
GLT1D1	0	0	0	0	0	0.247278	0	0
GLT8D1	41.13473	124.380957	0	99.278259	51.975116	68.227718	12.012902	76.04566
GLT8D2	0	3.517024	0	6.240842	0.595348	7.181656	0	10.072936
GLTP	49.6167	47.350993	63.399549	40.308011	82.643764	88.281551	73.922844	73.665674
GLTPD2	0	0	0	0.073515	0	0.148739	0	0.247808
GLTPP1	0	0	0	0	0	0	0	0
GLUD1	111.254385	81.340216	116.969088	65.862021	102.696527	43.682458	93.428871	55.583328
GLUD1P2	0	0	0	0	0	0	0	0
GLUD1P3	0	0	0	0	0	0	0	0
GLUD1P4	0	0	0	0	0	0	0	0
GLUD1P9	0	0	0	0	0	0	0	0
GLUL	139.961546	126.193726	186.969812	146.802719	225.288829	111.523648	114.401393	102.722422
GLULP3	0	0	0	0.088088	0	0	0	0

GLULP4	0	0	0	0	0	0	0	0
GLULP5	0	0	0	0	0	0	0	0
GLULP6	0	0	0	0	0	0	0	0
GLYAT	0	0	0	0	0	0	0	0
GLYATL1	6.798518	0	0	0.48355	0	0.496498	0	0.379574
GLYATL1B	0	0	0	0	0	0	0	0
GLYATL1P1	0	0	0	0	0	0	0	0
GLYATL1P2	0	0	0	0	0	0	0	0
GLYATL1P4	0	0	0	0	0	0	0	0
GLYATL2	0	0	0	1.156869	0	0.563026	0	1.477723
GLYATL3	0	0	0	0.387689	0	0	0	0
GLYCAM1	0	0	0	0	0	0	0	0
GLYCTK	0	0.560963	3.693803	2.067288	0	1.69958	0	0.315879
GLYR1	45.253177	51.359002	48.53019	71.265664	119.295568	61.9999	170.447506	38.424274
GLYR1P1	0	0	0	0.051971	0	0	0	0.260195
GM2A	0	0	0	1.887735	0	10.435129	21.384647	1.491107
GM2AP1	0	0	0	0	0	0	0	0
GM2AP2	0	0	0	0	0	0	0	0
GMCL1	10.302007	17.070928	9.247387	24.519232	41.82292	31.326138	69.172411	29.901668
GMCL1P2	0	0	0	0	0	0	0	0
GMDS	35.337011	29.382466	20.071117	35.96251	58.466942	27.957332	44.969591	41.863106
GMEB1	0	8.884228	9.299748	14.168423	12.662001	12.874349	9.883826	13.799753
GMEB2	10.002735	25.026757	12.433745	24.899944	5.283573	37.900755	6.412935	27.833175
GMFB	43.325538	111.457815	60.505658	74.236006	65.612371	67.683363	37.94773	194.519108
GMFBP1	0	0	0	0	0	0	0	0
GMFG	0	0	0	0.661443	0	0	0	0
GMIP	3.76981	6.636064	5.916768	13.462715	6.657047	27.93115	3.437894	15.554796
GML	0	0	0	0	0	0	0	0
GMNC	0	0	0	0	0	0	0	0
GMNN	64.09176	187.398052	51.67956	123.225897	50.33278	110.950438	37.182079	208.877871
GMPPA	0	19.423582	5.26257	23.293514	40.319464	11.818228	0	8.808965
GMPPB	3.005851	5.843328	1.168863	5.521906	5.135496	4.071108	0	5.668614
GMPR	0	0	0	0	0	0	0	0

GMPR2	20.418917	38.862185	107.526239	45.688071	59.869362	82.844212	253.113575	64.595679
GMPS	302.79803	479.442505	249.75792	267.311758	259.464363	170.479532	230.595183	548.004205
GMPSP1	0	0	0	0	0	0	0	0
GNA11	14.161067	67.068915	25.423616	49.510236	24.616018	72.365571	74.077796	48.149419
GNA12	23.345464	64.342731	20.960274	44.056317	53.498779	155.7649	19.433552	137.448034
GNA13	64.359706	59.362948	56.832378	36.514111	21.969409	26.759909	47.123679	55.033053
GNA13P1	0	0	0	0	0	0	0	0
GNA14	0	0.173828	0	0.066833	0	0.067686	0	0.164602
GNA15	0	0	0	0	0	0	0	0
GNAI1	9.225319	36.099826	4.224331	29.115547	21.849453	20.253425	34.349512	43.108297
GNAI2	118.592689	199.009457	109.086455	188.212938	150.762058	219.650463	87.431661	174.546021
GNAI2P1	0	0	0	0	0	0	0	0
GNAI2P2	0	0	0	0	0	0	0	0
GNAL	7.30171	18.478433	5.626579	20.791169	0	1.255387	0.127136	1.51797
GNAO1	10.594916	2.376874	1.160885	2.92016	8.923258	0.730488	0	0
GNAQ	0	23.862196	0	34.149182	19.02739	49.640072	8.943967	56.928836
GNAQP1	0	0	0	0.087838	0	0	0	0
GNAS	753.969187	1153.836	749.570717	939.858609	854.922602	1244.5706	724.835411	1589.29636
GNAT1	0	0	0	0	0	0	0	0
GNAT2	0	1.259585	0	0	0	0	46.014634	0
GNAZ	0	0.721059	0	0.874901	4.736173	3.245706	0	1.739859
GNB1	151.850854	511.507368	182.375042	623.401498	358.858157	891.63484	132.017084	858.635906
GNB1L	12.991271	5.754794	11.260444	7.353401	2.191977	24.453161	0	12.288016
GNB2	87.005005	240.741218	110.010789	229.32289	82.728692	351.117817	31.710721	268.662246
GNB3	0	0	0	0.577555	0	0	0	0.094442
GNB4	10.281305	10.963179	5.083889	2.294668	8.285763	1.058811	8.888425	6.110211
GNB5	21.86928	36.931313	28.845276	30.013089	4.977954	33.887165	60.164903	30.994003
GNE	4.53029	2.043196	0	1.692243	0	13.390579	0	6.169118
GNG10	61.960483	28.227731	26.743796	10.660853	52.350339	11.517197	37.96233	31.329104
GNG10P1	0	0	0	0	0	0	0	0
GNG12	124.987841	66.71415	108.189	43.810178	87.371431	22.424161	83.2692	45.968536
GNG13	0	0.80909	0	0.09432	0	0.189972	0	0
GNG14	0	0	0	0	0	0	0	0

GNG2	0	0	0	0.039834	0	0	0	0.448292
GNG3	0	0	0	0	0	0	0	0
GNG4	15.602132	43.553353	29.960391	25.627397	2.119751	14.821337	6.189269	10.991048
GNG5	239.900808	303.794597	221.86317	183.802814	179.769748	145.462777	287.385617	222.260168
GNG5P1	0	0	0	0	0	0	0	0
GNG5P3	0	0	0	0	0	0	0	0
GNG5P5	0	0	0	0	0	0	0	0
GNG7	0	0.067374	0	0.7337	0	0.196528	0	0.189387
GNGT1	0	0	0	0	0	0.088577	0	0.289214
GNGT2	0	0	0	0	0	0.451529	0	0
GNL1	73.195773	135.010401	69.548647	99.569996	173.920356	148.830874	90.578797	92.202256
GNL2	61.106591	76.52949	79.426413	111.011889	115.759517	129.15427	276.65631	120.514262
GNL2P1	0	0	0	0	0	0	0	0
GNL3	111.706846	126.342626	103.383387	142.630026	77.716349	68.459885	105.983529	96.931458
GNL3L	51.499392	75.629411	45.061151	95.639839	36.555033	103.615578	56.912686	89.919669
GNL3LP1	4.319492	2.494895	0	4.038753	0	3.489286	0	2.58776
GNLY	0	0	0	0	0	0	0	0
GNMT	0	0.292119	0	0.339585	0	0.685366	0	0.719205
GNPAT	29.500507	38.299607	36.74009	25.499805	15.567752	17.833408	38.153649	34.004588
GNPATP	0	0	0	0	0	0	0	0
GNPDA1	61.993685	102.431351	107.900163	91.379844	63.841954	105.216419	24.204525	87.837089
GNPDA2	18.533393	12.105185	17.502029	9.1194	14.476245	3.163455	26.144075	5.810682
GNPNAT1	88.994299	125.532721	74.576369	76.869948	63.45586	28.300271	206.32901	97.888633
GNPTAB	50.140301	59.080075	21.490586	38.810948	60.287199	120.324451	50.64065	151.923135
GNPTG	12.31637	11.044023	0	16.135792	0.943052	3.353755	0	16.684859
GNRH1	0	0	0	0	0	0	0	0
GNRH2	0	0	0	0	0	0	0	0
GNRHR	0	0	0	0	0	0	0	0
GNRHR2	7.221779	10.58455	3.26524	4.696095	0.971321	4.588892	0	3.709126
GNRHR2P1	0	0	0	0	0	0	0	0
GNS	43.651947	27.851651	56.085285	37.839727	112.010495	38.559118	32.386567	30.855757
GOLGA1	0	9.760771	0	21.514515	17.878469	27.176473	17.362785	16.11417
GOLGA2	0	0	0	0	0	0	0	0

GOLGA2P1	0	0	0	0	0	0	0	0
GOLGA2P10	0	7.597981	0	2.395584	0	4.562734	0	3.663918
GOLGA2P11	0	0	0	0	0	0	0	0
GOLGA2P2Y	0	0	0	0	0	0	0	0
GOLGA2P3Y	0	0	0	0	0	0	0	0
GOLGA2P4	0	0	0	0	0	0	0	0
GOLGA2P5	0	0	0	0.215134	0	0.392703	0	0
GOLGA2P6	0	0	0	0	0	0	0	0
GOLGA2P7	0	0	0	0	0	0	0	0
GOLGA2P8	0	0	0	0	0	0	0	0
GOLGA2P9	0	0	0	0	0	0	0	0
GOLGA3	0	4.920698	0	18.819246	0	25.354737	0	8.124475
GOLGA4	25.050186	36.42565	0	46.404158	12.31171	49.300041	0	40.320863
GOLGA5	67.823833	147.128932	75.414417	135.505279	64.384642	81.256567	41.466171	106.903901
GOLGA5P1	0	0	0	0	0	0	0	0
GOLGA6A	0	0	0	0.025681	0	0	0	0
GOLGA6B	0	0	0	0	0	0	0	0
GOLGA6EP	0	0	0	0	0	0	0	0
GOLGA6GP	0	0	0	0	0	0	0	0
GOLGA6L1	0	0	0	0	0	0	0	0
GOLGA6L10	0	0.055163	0	0	0	0	0	0
GOLGA6L11F	0	0	0	0.068258	0	0	0	0
GOLGA6L16F	0	0	0	0	0	0	0	0
GOLGA6L17F	0	0.559217	0	0	0	0	0	0
GOLGA6L2	0	0	0	0	0	0	0	0
GOLGA6L22	0	0	0	0	0	0	0	0
GOLGA6L23F	0	0	0	0	0	0	0	0
GOLGA6L24	0	0	0	0	0	0	0	0
GOLGA6L25	0	0	0	0	0	0	0	0
GOLGA6L3P	0	0	0	0.903546	0	0.556303	0	0
GOLGA6L4	0	0	0	0.988402	0	0	0	0.535759
GOLGA6L5P	0	0	0	0.310736	0	1.276515	5.563071	0.798908
GOLGA6L6	0	0	0	0	0	0	0	0

GOLGA6L7	0	0	0	0	0	0	0	0
GOLGA6L9	2.477395	0.366009	0.569313	0.313532	0	0.902364	0	1.039038
GOLGA7B	0	0	0.851253	2.049424	0	0.071242	0	0
GOLGA8A	3.9126	12.024148	2.864195	3.94838	6.88767	3.771613	33.204947	4.527471
GOLGA8B	27.310487	9.127719	3.994206	2.969316	3.131327	4.966652	15.48997	11.388368
GOLGA8CP	0	0	0	0	0	0	0	0
GOLGA8DP	0	0	0	0	0	0	0	0
GOLGA8EP	0	0	0	0	0	0	0	0
GOLGA8F	0	0	0	0	0	0.213589	0	0
GOLGA8G	0	0.075594	0	0	0	0	0	0
GOLGA8IP	0	0	0	0.090116	0	0	0	0
GOLGA8J	0	0.327701	0	0	0	0	0	0.087713
GOLGA8K	2.621367	0	0	0	0	0	0	0
GOLGA8M	0	0	0	0.387551	0	0.382151	0	0.078422
GOLGA8N	0	0	0	1.205644	0	0.090513	4.030477	0.252379
GOLGA8O	0	0	0	0.737579	0	0	0	0
GOLGA8Q	0	0.271922	0	0	0	0	0	0.12064
GOLGA8R	0	0	0	0	0	0	0	0
GOLGA8S	0	0	0	0	0	0	0	0
GOLGA8T	0	0	0.558431	0.073509	0	0	0	0
GOLGA8UP	4.011625	0.253771	0	0.048639	0	0.494702	0	0.729392
GOLGA8VP	0	0	0	0	0	0	0	0
GOLIM4	0	25.477532	25.150993	37.488103	31.222451	39.521402	74.054455	29.215454
GOLM1	33.03456	101.979968	25.654071	112.961785	43.091235	107.799036	20.774257	52.493682
GOLM2	77.310412	66.521209	104.086495	54.034796	149.319875	33.687392	193.020967	50.563599
GOLM2P1	0	0	0	0	0	0	0	0
GOLPH3	49.280604	89.366389	55.429752	73.976028	71.018704	59.717281	67.067477	81.596946
GOLPH3L	24.654661	33.62509	25.938368	49.367682	20.731109	25.325753	29.592093	20.999571
GOLT1A	10.64043	1.505777	9.310382	8.087825	0	1.012734	0	2.618789
GOLT1B	58.135187	40.184753	46.161488	48.014631	112.09269	38.369213	17.283197	33.252102
GON4L	5.007609	16.022034	16.211847	17.459555	39.615732	20.342973	34.094638	25.981076
GON7	0	8.743241	17.774024	6.686637	14.080956	9.974182	0	14.439706
GOPC	15.736805	12.189756	20.637374	9.376073	13.792382	10.472605	21.062093	17.250323

GORAB	0	7.061112	12.991741	6.735286	0.415313	15.253363	0	1.519899
GORASP1	0	0	7.395464	0.149738	0	0	0	0
GORASP2	71.802512	104.359503	100.394764	122.022731	95.548044	117.155879	154.487412	96.652355
GOSR1	15.53028	47.770795	45.270558	33.477931	49.664317	30.54051	31.427569	30.907102
GOSR2	18.472664	52.156094	22.45897	42.865885	16.884682	37.668029	43.714828	58.113013
GOT1	52.696193	39.261643	54.884447	50.361531	63.591245	37.924173	62.081861	22.398246
GOT1L1	0	0	0	0	0	0	0	0
GOT2	272.433103	141.291107	238.289079	163.355	259.390661	166.35012	232.608047	145.695727
GOT2P1	0	0	0	0	0	0	0	0
GOT2P2	0	0	0	0	0	0	0	0
GOT2P3	0	0	0	0	0	0	0	0
GOT2P5	0	0	0	0	0	0	0	0
GOT2P6	0	0	0	0	0	0	0	0
GOT2P7	0	0	0	0	0	0	0	0
GP1BA	0	0	0	0	0	0	0	0
GP2	0	0	0	0	0	0	0	0
GP5	0	0	0	0	0	0	0	0
GP6	0	0	0	0	0	0.223633	0	0
GPA33	1.333081	0	0	0.064899	0	0	0	0
GPAA1	5.57527	27.074595	36.120404	23.115552	7.091599	12.149554	0	15.404197
GPAA1P1	0	0	0	0	0	0	0	0
GPAA1P2	0	0	0	0	0	0	0	0
GPALPP1	5.601079	1.484799	6.884212	1.975595	11.656349	0.782995	4.513054	2.038492
GPAM	6.111964	9.506892	4.579272	5.631325	6.190783	3.57624	108.930868	10.951181
GPANK1	11.142298	40.322949	29.496399	52.208041	25.07897	57.526058	0	41.586171
GPAT2	0	0	0	0.029092	0	0	0	0
GPAT2P1	0	0	0	0	0	0	0	0
GPAT2P2	0	0	0	0	0	0	0	0
GPAT3	15.650032	6.80825	5.666335	4.504339	8.495223	0.972258	0	2.494781
GPAT4	37.244699	193.532329	13.440136	217.821665	22.676324	167.859277	95.623852	230.369522
GPATCH1	0	0	0	0	5.361632	0	0	0
GPATCH11	0	2.186956	0	6.162975	0	3.008988	0	10.887895
GPATCH11P1	0	0	0	0	0	0	0	0

GPATCH2	32.269123	22.664253	24.966966	31.217719	8.500196	13.440993	10.170938	13.963523
GPATCH2L	17.817326	25.326326	19.695581	35.858953	30.137157	32.37299	66.126351	23.551256
GPATCH3	8.117687	8.463666	1.450482	9.942329	2.996767	17.692941	0	14.075756
GPATCH4	0	1.034222	19.791742	3.358396	15.840078	0.471705	0	0
GPATCH8	34.726239	42.442683	52.851684	56.291329	78.205546	50.31737	9.132243	45.1012
GPBAR1	0	0	0	0	0	0.277158	0	0
GPBP1	56.882286	194.534321	64.954461	156.604982	67.087145	123.094377	143.431493	239.478469
GPBP1L1	59.347961	138.580617	60.243246	138.598179	61.286572	287.106376	32.994624	281.519219
GPC1	61.516022	119.992606	99.267841	120.047043	84.40283	203.850261	53.133894	116.216752
GPC2	0	0	0	0.194861	0	0	0	0
GPC3	2.25557	0.652131	2.7798	0.572613	0	0.22191	0	0
GPC4	13.860771	13.513576	8.893724	12.792851	0	0.264378	0.160769	0.556168
GPC5	0	0	0	0.055671	0	0	0	0
GPC6	2.726041	1.557663	1.634459	1.636313	4.922634	1.785792	0	2.604221
GPCPD1	0	5.491507	3.691585	2.419106	4.862688	7.777126	11.299451	12.944551
GPCPD1P1	0	0	0	0	0	0	0	0
GPD1	0	0	0	0	0	0	0	0
GPD1L	0	0	5.510343	5.352691	19.917837	19.931718	5.763122	12.410255
GPD2	35.280399	74.824604	28.195555	78.064933	116.380526	51.11233	33.596325	74.285935
GPER1	0	1.177036	0	0.896286	0	2.711603	0	2.637169
GPHA2	0	0	0	0	0	0	0	0
GPHB5	0	0	0	0	0	0	0	0
GPHN	12.483883	64.195782	38.002964	69.289075	88.248824	31.346116	36.985407	53.601941
GPI	304.161687	296.731423	321.290872	373.762311	779.357077	720.487155	530.835598	571.76809
GPIHBP1	0	0	0	0	0	0	0	0
GPKOW	12.826642	18.900302	38.087266	22.800341	33.790362	21.585798	14.975654	16.422505
GPLD1	0	0.856429	0	0.357403	0	1.156766	0	0.067642
GPM6A	0	1.833561	0	0.270423	0	0	0	0
GPM6B	0	0	0	0.329124	0	0.594371	0	0
GPM6BP1	0	0	0	0	0	0	0	0
GPM6BP3	0	0	0	0	0	0	0	0
GPN1	61.636397	3.368385	3.137424	7.776444	73.503013	17.442743	16.286259	9.07415
GPN2	8.440056	38.757795	20.247519	32.630595	30.764854	36.492491	33.52595	37.370769

GPN3	120.061974	95.150512	36.745221	54.192158	102.962328	72.892607	42.536386	98.68812
GPN3P1	0	0	0	0	0	0	0	0
GPNMB	0	0.409573	0	0.179413	0	1.490624	0	0.190301
GPR107	20.22133	14.321748	51.633286	18.731787	20.994128	14.531961	32.088542	11.156759
GPR108	0	15.682442	0	4.111585	68.838246	20.067421	0	5.470193
GPR119	0	0	0	0	0	0	0	0
GPR12	0	0	0	0	0	0	0	0
GPR132	0	0	0	0.08865	0.320475	0.099588	0	0.040126
GPR135	0	1.963033	1.196966	0.293066	0.352899	1.195923	7.789605	0.457257
GPR137	0	17.440065	18.448574	12.388583	12.500591	40.103829	14.680889	40.786575
GPR137B	13.634412	17.770448	15.219416	13.145541	0.900013	7.88628	1.209188	7.602994
GPR137C	1.201012	0	0	0.491522	0	0	0	0.75437
GPR139	0	0	0	0	0	0	0	0
GPR141	0	0	0	0	0	0	0	0
GPR141BP	0	0	0	0	0	0	0	0
GPR142	0	0	0	0	0	0	0	0.509364
GPR143	0	0.918907	0	0.104503	0	0	0	0
GPR143YP	0	0	0	0	0	0	0	0
GPR146	1.854734	3.57435	0	5.733934	2.933187	7.84388	0	22.856025
GPR148	0	0	0	0	0	0	0	0
GPR149	0	0	0	0	0	0	0	0
GPR15	0	0	0	0	0	0	0	0
GPR150	0	0	0	0.12268	0	0	0	0
GPR152	0	0	0	0.122365	0	0	0	0
GPR153	8.636739	13.962778	11.981778	13.518099	10.990042	21.202994	10.128845	12.263214
GPR155	0.841325	7.013228	0	0.219926	0	0	0	0.377468
GPR156	0	0.519166	1.425172	1.895782	0	0.687343	0	0.466631
GPR157	2.514812	2.672664	5.648683	4.825603	0.664351	17.892899	11.334114	5.892165
GPR158	0	0	0	0.016729	0	0	0	0
GPR15LG	0	0	0	0.4812	0	0.469725	0	0
GPR160	1.772343	6.241122	4.746282	4.61793	5.606633	2.002477	0	7.191551
GPR160P1	0	0	0	0	0	0	0	0
GPR160P2	0	0	0	0	0	0	0	0

GPR161	12.449231	21.768336	4.215484	20.176428	2.196746	34.948407	1.066988	18.064754
GPR162	0	0.406023	0	0.872919	0	0.506367	0	0.178752
GPR166P	0	0	0	0	0	0	0	0
GPR17	0	0	0	0	0	0	0	0
GPR171	0	0	0	0	0	0	0	0
GPR173	7.961054	3.503095	0	1.300046	0	3.033953	0	2.585264
GPR176	0	0	0	1.269902	0	0.849678	0	0.339736
GPR179	0	0.06094	0	0.639427	0	0.01015	0	0.024301
GPR18	0	0	0	0	0	0	0	0
GPR180	4.347304	2.919281	4.237823	1.081918	4.59607	0.545977	2.474918	1.889518
GPR182	0	0	0	0	0	0	0	0
GPR183	0	0	0	0	0	0	0	0
GPR19	6.03255	14.620286	7.206979	18.220196	0.53259	1.295902	0	6.708138
GPR199P	0	0	0	0	0	0	0	0
GPR22	0	0	0	0	0	0	0	0
GPR3	1.613564	1.504723	0	1.372475	0	0.87931	0	0.455442
GPR32	0	0	0	0.210154	0	0	0	0
GPR32P1	0	0	0	0	0	0	0	0
GPR34	0	0	0	0	0	0	0	0
GPR35	0	0	0	0	0	0	0	0
GPR37L1	0	0.192302	0	0.0972	0	0.024916	0	0
GPR39	2.634958	8.353305	4.716355	8.05026	9.674117	11.917974	15.985955	10.608643
GPR4	0	0.093787	0.984644	0.321411	0	0	0	0
GPR42	0	0	0	0	0	0	0	0
GPR53P	0	0	0	0	0	0	0	0
GPR55	0	0	0	0.313404	0	0.043204	0	0
GPR6	0	0	0	0	0	0	0	0
GPR61	0	0.902726	0	0	0	0.35567	0	0
GPR62	0	0.069019	0	0.158299	0	0	0	0
GPR63	0	0.702846	0.489895	0.426461	0.287995	0.21858	0	0.240408
GPR65	0	0	0	0	0	0	0	0
GPR68	1.178118	3.96769	5.276	7.118516	0	6.861737	0	6.363052
GPR78	0	1.897591	2.635906	1.643936	0	0	5.853925	0

GPR79	0	0	0	0	0	0	0	0
GPR82	0	0	0	0	0	0	0	0
GPR83	0	0.07722	0	0	0	0	0	0
GPR84	0	0.199617	0	0	0	0	0	0
GPR85	1.287715	4.098687	6.819469	1.261261	0	0	0	1.286936
GPR87	0	0	0	0	0	0	79.800622	1.237322
GPR88	0	0	0	0	0	0	0	0
GPR89A	16.190001	18.7974	3.566479	15.179559	12.380745	6.835824	21.902072	28.14417
GPR89B	1.128432	0.275816	0	2.758212	0	0	0	5.90941
GPR89P	0	0	0	0	0	0.236079	0	0
GPRASP1	0	0.805687	0.503611	1.314225	2.072525	0.786382	0	0.574816
GPRASP3	3.326233	19.513084	5.957973	16.358492	34.572161	22.834937	6.27289	20.417456
GPRASP3P1	0	0	0	0	0	0	0	0
GPRC5A	0	19.530235	0	34.399269	52.725585	103.495248	80.517798	94.682369
GPRC5B	2.541682	12.885293	7.421465	14.127056	0.438698	5.974141	13.701382	6.293028
GPRC5C	3.497754	20.701881	28.85742	31.268027	19.80296	21.44191	0	17.195463
GPRC5D	0	0	0	0	0	0.842404	0	0
GPRC6A	0	0	0	0	0	0	0	0
GPRIN1	10.894515	40.085832	10.478608	31.98369	4.110378	62.893738	0.189419	39.741933
GPRIN3	0	0	0	0.089182	0	0	0	0
GPS1	31.218069	15.748014	25.336697	26.243419	50.967313	61.674823	0	35.489667
GPS2	37.715333	65.385424	50.630651	76.830782	86.954287	108.440476	56.885459	74.738451
GPS2P1	0	1.139154	0	1.432725	0	2.697478	0	2.098715
GPS2P2	0	0	0	0.32092	0	0	0	0.366234
GPSM1	3.764182	13.715885	13.50396	18.681552	3.975316	13.232465	10.253947	6.814734
GPSM2	51.402583	143.672354	36.187549	116.639116	22.587245	55.545259	33.286415	92.781701
GPSM3	5.110964	0.89168	0	1.70978	0	5.283437	0	5.256
GPT	0	0	0	0	0	0	0	0
GPT2	46.558714	39.689078	41.012131	77.849897	29.081176	55.885616	47.662857	40.720814
GPX1	106.270317	117.301876	127.874119	128.821588	98.236691	112.320977	16.87437	123.920544
GPX1P1	0	0	0	0.386129	0	0.782024	0	0.321765
GPX1P2	0	0	0	0	0	0	0	0
GPX2	169.893196	140.531806	163.420011	174.887919	2.240025	15.105789	25.47361	24.183592

GPX3	0	33.992627	0	26.865306	0	57.060358	0	45.919347
GPX4	407.098688	812.829506	469.346854	769.811972	478.764509	776.908943	254.292025	694.607283
GPX5	0	0	0	0	0	0	0	0
GPX6	0	0	0	0	0	0	0	0
GPX7	36.180563	17.44568	13.363118	19.448206	18.304673	4.702042	0	6.980191
GPX8	40.559675	104.928488	49.039101	71.201257	32.082236	22.1222	99.509841	41.618762
GRAMD1A	6.736635	5.568655	19.402372	13.279209	10.574768	21.760708	27.133328	10.494459
GRAMD1B	0.859917	1.452164	0.931768	1.985478	16.010527	3.341555	0	3.237104
GRAMD1C	2.872885	14.128304	0	11.86351	5.240193	2.159508	0.465727	2.079818
GRAMD2A	7.501017	6.707527	1.047809	3.141014	4.373273	7.687186	0	5.055932
GRAMD2B	23.363676	35.011941	22.506122	31.130559	43.073477	47.17236	65.251189	39.830741
GRAMD4	4.577536	11.531469	55.497531	25.649828	10.425553	29.843432	0	25.51767
GRAMD4P1	0	0	0	0	0	0	0	0
GRAMD4P2	0	0	0	0	0	0	0	0
GRAMD4P3	0	0	0	0	0	0	0	0
GRAMD4P4	0	0	0	0	0	0	0	0
GRAMD4P5	0	0	0	0	0	0	0	0
GRAMD4P6	0	0	0	0	0	0	0	0
GRAMD4P7	0	0	0	0	0	0	0	0
GRAMD4P8	0	0	0	0	0	0	0	0
GRAP	0	0	0	0	0	0	0	0
GRAP2	0	2.23961	0	0	0	0	0	0
GRAPL	0	0	0	0	0	0	0	0
GRB10	6.274042	29.478968	23.65213	33.590497	5.108908	4.951927	2.724691	5.274848
GRB14	13.929182	0.741166	3.472708	1.889518	0	0	6.071299	3.063472
GRB2	58.867567	162.050045	98.408314	203.138245	71.744909	186.134988	63.1724	165.979202
GRB7	11.555226	10.017694	21.144374	22.489153	15.669446	20.676628	8.396753	9.778302
GREB1	0	0.211825	0.553968	0.557583	0	0.324773	0	0.049466
GREB1L	15.711299	3.122604	3.289853	3.096799	10.177772	3.331688	3.860547	7.666011
GREM1	2.557975	0.845117	1.14489	1.2189	10.489603	15.924217	80.811653	21.792153
GREM2	0	0	0	0	0	0.039471	0	0
GREP1	0	0	0	0	0	0.581488	0	0.349877
GRHL1	5.039334	5.87367	34.924952	9.265493	19.191759	14.58691	0	6.861014

GRHL2	28.09549	122.590222	24.682508	102.283236	47.425964	156.525136	106.757576	146.22386
GRHL3	0	1.142069	1.077197	0	0.858865	0	0	0
GRHPR	47.290777	42.259106	51.294425	56.434886	105.147841	89.515414	51.17186	65.460729
GRIA1	0	2.000069	1.682566	1.977061	0	0.331068	0	0.24606
GRIA2	0	0	1.079652	0.08066	0	0	0	0
GRIA3	0	0	0	0	0	0	0	0
GRIA4	0	0	0	0.088779	0	0	0	0
GRID1	1.030247	0	0	0.580626	0	0.359594	0	0.124253
GRID2	0	0	0	0	0	0	0	0
GRID2IP	0	0.754908	0	0.639836	1.921295	0.362878	0	0.292616
GRIFIN	0	0	0	0	0	0	0	0
GRIK1	0	0	0	0	0	0	0	0
GRIK2	0	1.084303	0	0.212913	0	0	0	0
GRIK3	0	0	0	0	0	0	0	0
GRIK4	0	0	0	0.25294	0	0.113041	0	0
GRIK5	0	0.278612	0	0	0	0.712967	0	0.198885
GRIN1	0	2.72062	2.19924	2.704604	0.490482	0.324567	0	1.373512
GRIN2A	0	0	0	0.010825	0	0.135435	0	0
GRIN2B	0	0	0	0	0	0	0	0
GRIN2C	0	0.293776	0	0.112992	0	0.024967	0	0
GRIN3A	0	0	0	0	0	0	0	0
GRIN3B	0	0.78251	3.649068	1.067073	0.268633	0.253738	0	0.163628
GRINA	92.238885	140.249632	83.304796	155.642365	86.742272	77.953775	27.870968	64.702008
GRIP1	0	3.22511	23.068395	7.013603	0.297488	45.264865	0	38.247351
GRIP2	2.668571	1.989833	2.24683	2.857652	0	0.488658	1.731087	0.097868
GRIPAP1	21.032104	33.801458	29.755808	38.178569	24.479416	36.888438	16.704315	25.205067
GRK1	0	0	0	0.114473	0	0.175424	0	0
GRK2	53.867841	78.349432	50.917546	69.542851	33.018588	72.423369	148.293643	52.180842
GRK3	12.392873	3.999321	1.300332	3.417063	0.243768	2.872744	0	4.339414
GRK4	1.61439	4.539963	1.574207	6.058361	3.405638	4.814071	0	5.493606
GRK5	0	0.596044	1.981017	0.793878	0	0.172292	0	0.14571
GRK6	36.711627	50.898157	57.319044	49.367975	25.077089	78.890626	17.898828	65.54114
GRK6P1	0	0	0	0	0	0	0	0

GRK7	0	0	0	0	0	0	0	0
GRM1	0	0.03776	0	0.081136	0	0.048135	0	0.096155
GRM2	0.49509	0.624192	0	0.03633	0	0.074503	0	0.375524
GRM3	0	0	0	0	0	0	0	0
GRM4	1.127948	0	7.021211	0	19.093315	1.812376	0	0.553116
GRM5	1.221146	5.447689	1.098544	4.300743	0	0	0	0
GRM5P1	0	0	0	0	0	0	0	0
GRM6	0	0	0	0	0	0	0	0
GRM7	0	0	6.86879	0.179831	0	0	0	0
GRM8	0	0.077801	0	0	0	0.144679	0	0
GRN	52.563411	34.592898	105.891307	57.910329	132.801085	62.464422	181.974397	39.485472
GRP	0	0	0	0	0	0	0	0
GRPEL1	52.431508	154.138869	71.422241	197.928702	81.486919	172.698012	88.795196	104.106156
GRPEL2	27.11239	23.702899	40.554761	23.138711	19.092974	13.628083	0	14.712341
GRPEL2P1	0	0	0	0	0	0	0	0
GRPEL2P2	0	0	0	0.154475	0	0	0	0
GRPEL2P3	0	0	0	0	0	0	0	0
GRSF1	70.665207	91.931227	21.330585	60.394169	45.340242	44.890419	33.247757	82.015483
GRTP1	7.087763	9.949908	3.979155	9.986139	9.53661	9.328879	0.683004	5.680779
GRWD1	0	12.844569	0	16.262057	22.868217	48.527649	20.520443	28.690757
GRXCR2	0	0	0	0	0	0	0	0
GSAP	3.011308	5.042529	8.429571	6.504932	2.15423	3.742306	0	2.725446
GSDMA	0	0	0	1.59387	0	2.013456	0	0
GSDMB	7.283607	0.181431	4.506215	1.124931	4.003857	1.398495	0	0.715263
GSDMC	4.148511	29.502132	9.952986	30.158121	5.136278	18.026688	0	22.797395
GSDMD	13.772558	32.504656	25.445633	22.217757	19.056268	42.078909	19.273846	41.217521
GSDME	54.408148	95.290677	56.932133	99.961678	42.720874	62.854979	25.394467	46.416543
GSE1	56.073132	48.510332	58.601564	68.141263	36.208621	64.337801	79.887388	37.861191
GSG1	0	0.242801	1.277682	0	0	0	0	0.057548
GSG1L	0	0	0	0.076725	0	0	0	0
GSK3A	31.371909	39.741615	26.634809	36.744526	65.174508	71.097458	30.885329	54.227111
GSK3B	0	0	0	0	0	0	0	0
GSKIP	12.888716	10.530783	11.73547	18.695936	11.053108	18.254318	43.853064	40.311082

GSN	11.469003	12.429332	21.42804	19.687614	14.122304	19.55492	16.929865	10.921672
GSPT1	199.393359	238.279771	196.083464	233.211825	214.63723	195.623327	208.96489	241.702683
GSR	83.562389	142.935804	87.893767	122.300959	98.640265	81.568218	82.642606	59.12655
GSS	107.246116	113.387245	77.138872	121.144682	30.95879	47.537786	33.504993	43.124368
GSTA1	0	0	0	0	0	0	0	0
GSTA11P	0	0	0	0	0	0	0	0
GSTA12P	0	0	0	0	0	0	0	0
GSTA2	0	0	0	0	0	0	0	0
GSTA3	0	0	0	0	0	0	0	0
GSTA4	0	2.236529	4.098303	1.497153	0	0.582425	0	0.605823
GSTA6P	0	0	0	0	0	0	0	0
GSTA7P	0	0	0	0	0	0	0	0
GSTA8P	0	0	0	0	0	0	0	0
GSTA9P	0	0	0	0	0	0	0	0
GSTCD	21.46451	25.438299	0	20.559489	4.882205	22.439751	25.241279	29.626263
GSTK1	24.853473	59.272122	54.870694	63.363734	61.671877	44.558898	0	34.389731
GSTM1	0	0	23.068708	1.212906	17.696932	0	0	12.565814
GSTM2	0.761816	0.872175	0	0.66778	0	0.857573	0	1.25448
GSTM2P1	0	0	0	0	0	0	0	0
GSTM3	47.285499	13.816982	73.667276	17.409327	0	4.175131	0	0.195747
GSTM3P1	0	0	0	0	0	0	0	0
GSTM3P2	0	0	0	0	0	0	0	0
GSTM4	4.278776	9.278071	4.627271	19.685221	0	4.48774	0	3.395379
GSTM5	0	0	0	0.055432	0	0	0	0
GSTM5P1	0	0	0	0	0	0	0	0
GSTO1	330.502169	251.307669	210.915704	245.928069	203.377079	231.051341	145.241844	233.200678
GSTO2	80.848003	56.829217	76.246444	38.491334	54.342837	31.836664	1.84731	20.420985
GSTO3P	0	0.237124	5.143565	0	0	0	0	0
GSTP1	23.470643	31.157113	18.727641	45.911728	72.823184	21.579487	38.340484	12.41517
GSTP1P1	0	0	0	0	0	0.914399	0	0
GSTT1	0	0.162715	8.869273	2.671164	19.305164	3.392292	0	0.140056
GSTT2	0	0	0	0	0	0	0	0
GSTT2B	0	0	0	0	0	4.905029	0	0

GSTT3P	0	0	0	0	0	0	0	0
GSTT4	0	0	0	0	0	0	0	0
GSTZ1	24.939077	28.051778	33.852888	32.576974	1.350278	14.66761	30.129659	16.688689
GSX2	11.278016	0.525218	0	1.68244	0	0	0	0
GTDC1	16.78759	26.451365	20.242938	20.777202	18.414421	16.020618	10.182925	40.463269
GTF2A1	48.570376	137.311268	55.848453	133.358951	30.767992	71.838885	47.007416	120.00394
GTF2A1L	0	0	0	0	0	0	0	0
GTF2A2	200.41283	421.943637	209.129186	271.395932	130.547315	274.62211	138.637884	436.562994
GTF2B	37.857046	54.658402	29.741224	40.464179	54.280764	18.002313	37.339315	28.797549
GTF2E1	37.620219	20.575971	13.035208	31.647956	41.584051	16.800777	68.958514	16.805724
GTF2E2	30.241147	108.646306	26.957813	72.75605	33.467638	53.297376	79.189381	73.257434
GTF2F1	64.485444	202.334164	47.652159	266.769504	103.384963	260.868898	101.721254	215.093886
GTF2F2	44.710496	59.469504	30.316931	39.820312	22.363124	31.290284	0	52.469145
GTF2F2P1	0	0	0	0	0	0	0	0
GTF2F2P2	0	0	0	0	0	0	0	0
GTF2H1	42.249373	49.444381	32.643984	33.204425	109.325784	35.635815	40.42617	78.573757
GTF2H2	77.181469	66.359361	30.730825	38.435893	12.356656	26.411704	32.544466	60.134817
GTF2H2B	31.609806	18.607268	13.629515	4.351254	6.251843	0	27.51513	4.645224
GTF2H2C	2.501584	31.437797	13.606325	21.29901	23.605453	14.631124	61.184279	19.037169
GTF2H2C_2	15.814652	10.453693	0	7.881307	0	0	0	4.256293
GTF2H3	27.073336	54.834249	27.868787	39.451879	33.651441	18.55638	63.372082	39.80974
GTF2H4	20.006332	28.546213	5.656239	19.292084	26.797152	26.995177	0	24.070409
GTF2H5	17.159889	31.850611	20.501757	16.807149	8.042427	12.730048	0	27.168115
GTF2I	65.359096	159.348099	43.802956	189.561475	102.168931	293.160167	232.090731	278.930364
GTF2IP1	0	0	39.114937	20.453406	11.117768	0	56.671509	21.971633
GTF2IP11	0	0	0	0	0	0	0	0
GTF2IP12	0	0	0	0	0	0	0	0
GTF2IP13	0	0	0	0	4.133325	0	0	0
GTF2IP14	0	0	0	0	0	0	0	0.651753
GTF2IP2	0	0	0	0	0	0	0	0
GTF2IP20	0	0	0	0	0	0	0	0
GTF2IP22	0	0	0	0	0	0	0	0
GTF2IP23	0	1.925992	0	1.22395	0	0	0	0

GTF2IP3	0	0	0	0	0	0	0	0
GTF2IP4	33.547203	55.207581	5.580962	37.84927	35.280568	25.172215	0	48.820579
GTF2IP5	0	0	0	0	0	0	0	0
GTF2IP6	0	0	0	0	0	0	0	0
GTF2IP7	22.874058	5.522551	0	0	0	0	0	10.707782
GTF2IP8	0	0	0	0	0	0	0	0
GTF2IP9	0	2.472703	0	0	0	0	0	0
GTF2IRD1	13.592636	5.225331	13.930043	11.623678	53.03831	13.314264	1.170614	10.778431
GTF2IRD1P1	0	0	0	0	0	0	0	0
GTF2IRD2	0	1.412637	13.226691	0.505634	0.195669	16.458631	0	9.028692
GTF2IRD2B	3.818911	17.607433	23.963148	11.574323	31.471628	11.710451	0	26.365626
GTF2IRD2P1	0	0	0	0	0	0	0	0
GTF3A	0	6.277323	11.832501	5.082585	2.954406	7.289703	42.986209	12.270694
GTF3AP1	0	0	0	0	0	0	0	0
GTF3AP2	0	0	0	0	0	0	0	0
GTF3AP5	0	0	0	0	0	0	0	0
GTF3AP6	0	0	0	0	0	0	0	0
GTF3C1	56.769355	25.011965	60.942028	29.571865	36.577692	38.090717	183.534228	26.621294
GTF3C2	5.810618	6.950412	0	6.141625	32.902039	1.775235	33.26112	6.002443
GTF3C3	31.22105	32.390285	22.900955	23.091669	11.307692	35.22932	97.468324	31.299565
GTF3C4	25.429731	50.704079	22.207954	42.568101	27.971545	43.871155	21.987976	60.244838
GTF3C5	14.562898	32.44364	21.019473	22.499469	22.450988	23.352862	0	13.624635
GTF3C6	41.717826	93.186687	9.131068	58.694593	77.848455	102.563088	28.481672	177.690282
GTF3C6P1	0	0	0	0	0	0	0	0
GTF3C6P2	0	1.162777	0	0	0	0	0	0
GTF3C6P3	0	0	0	0	0	0	0	0
GTPBP1	20.59036	14.86887	10.884379	15.610496	29.105865	16.856489	7.154425	10.330624
GTPBP10	55.168345	23.577057	35.275788	19.663818	61.804636	16.09813	42.667139	18.684975
GTPBP2	14.390387	26.209676	24.234812	36.921077	17.530245	46.851332	82.243978	28.109683
GTPBP3	13.648292	21.178478	13.167387	28.49912	29.101789	44.256064	34.546454	46.287491
GTPBP4	36.964592	40.404715	17.025733	40.538124	20.382975	41.31386	44.260798	34.487884
GTPBP6	0	0.736971	8.251857	0.252209	0.481654	3.557272	0	1.084338
GTPBP8	3.748607	1.375744	27.483962	0.291	0	5.029373	34.697847	2.644471

GTSE1	19.140865	24.024632	10.087585	17.550657	21.693411	45.089975	30.940111	33.720588
GTSF1	0	0	0	0	0	0	0	0
GTSF1L	0	0	0	0	0	0	0	0
GUCA1ANB-	0	0	0	0	0	0	0	0
GUCA1B	1.510705	1.346573	0	0.736483	0	0.599414	0	0.243533
GUCA1C	0	0	0	0	0	0	0	0
GUCA2A	0	0	0	0	0	0	0	0
GUCA2B	0	0	0	0	0	0	0	0
GUCD1	5.875495	52.50506	0	28.326489	17.54327	51.642413	15.417218	26.445761
GUCY1A1	2.661659	4.525815	2.30557	3.387318	0	0	0	0
GUCY1A2	0	0	0	0.060859	0	0	0	0
GUCY1B1	4.43435	2.721944	1.360235	2.256256	0	0.088184	0	0
GUCY1B2	0	0	0	0.374259	0	0	0	0
GUCY2C	0	0	0	0	0	0	0	0
GUCY2D	0	0	0	0.206141	0	1.216588	0	1.847334
GUCY2EP	0	0	0	0	0	0	0	0
GUCY2GP	0	0	0	0	0	0	0	0
GUF1	20.824037	14.075818	15.877221	7.912117	12.848382	2.87599	19.201185	16.518747
GUK1	0	23.403012	104.242657	20.411288	80.834033	36.100135	69.393767	43.775939
GULOP	0	0	0	0	0	0	0	0
GULP1	11.828596	34.563124	6.803163	16.087516	1.428897	5.690905	0	5.013055
GUSB	14.075331	9.346561	33.544696	17.72129	30.107828	14.49564	0	8.311444
GUSBP10	0	0	0	0	0	0	0	0
GUSBP11	0	1.270436	1.762201	1.781145	0	2.452495	0.318547	1.49133
GUSBP12	0	0	0	0	0	0	0	0
GUSBP13	23.133012	5.898271	0	3.842274	0	8.921185	0	2.81727
GUSBP14	37.057534	0	0	6.144518	12.795014	9.150974	0	0
GUSBP15	0	8.145013	20.275627	11.130043	0	0	0	0
GUSBP16	0	0	0	0	0	0	0	0
GUSBP17	0	0	0	0	0	0	0	0
GUSBP18	0	0	0	0	0	0	0	0
GUSBP19	0	0	0	0.403213	0	0	0	0
GUSBP2	0	0	0	0	0	0	0	0

GUSBP3	0	0.536602	55.678004	0	0	0.478414	0	1.534152
GUSBP4	0	0.299462	0	1.002894	0	2.013692	0	0
GUSBP5	0	0	0	0	0	2.27137	0	0
GUSBP6	0	0	0	0	0	0	0	0
GUSBP7	0	0	0	0	0	0	0	0
GUSBP8	0	0	0	0	0	0	0	0
GUSBP9	0	4.152632	0	4.706647	0	3.695304	0	1.423487
GVINP1	0	0	0	0.250269	0	0.066962	0	0.409976
GVINP2	0	0	0	0	0	0	0	0
GVQW3	1.833931	25.512086	12.385055	10.782062	0.096943	0.479872	0	5.429924
GXYLT1	3.881487	7.136397	9.402307	2.013229	4.934004	1.51981	11.28514	4.91761
GXYLT1P1	0	0	0	0	0	0	0	0
GXYLT1P2	0	0	0	0	0	0	0	0
GXYLT1P3	0	0	0	0	0	0	0	0
GXYLT1P4	0	0	0	0.15061	0	0	0	0
GXYLT1P6	0	0	0	0	0	0	0	0
GXYLT1P7	0	0	0	0	0	0	0	0
GXYLT2	0	0	0	1.377681	2.253097	0	40.605981	0
GYG1	31.40113	30.905463	26.37043	21.804525	95.093423	29.1971	65.408907	34.687066
GYG1P1	0	0	0	0	0	0	0	0
GYG1P2	0	0	0	0	0	0	0	0
GYG1P3	0	0	0	0	0	0	0	0
GYG2	0	0	0	0.057556	0	0.699743	0	1.007512
GYPA	0	0	0	0	0	0	0	0
GYPB	0	0	0	0	0	0	0	0
GYPC	0	0.518	4.796521	1.326183	0	0.202814	0	0
GYPE	0	0	0	0	0	0	0	0
GYS1	13.965579	14.95151	14.155532	13.866322	37.856238	26.351432	111.827685	25.306229
GZF1	5.534087	13.048413	7.455609	7.923993	66.823971	13.801397	77.868488	8.788789
GZMA	0	0	0	0	0	0	0	0
GZMAP1	0	0	0	0	0	0	0	0
GZMB	0	0	0	0.219555	0	0.861527	0	0
GZMH	0	0	0	0	0	0	0	0

GZMK	0	0	0	0	0	0	0	0
GZMM	0	0	0	0.099667	0	0	0	0.174019
H1-10	260.216591	612.146923	284.122894	762.274538	304.612898	953.228226	172.924881	537.606804
H1-12P	0	1.577463	0	1.512394	0	0.37319	0	0
H1-7	0	0	0	0	0	0	0	0
H1-8	0	0	0	0	0	0	0	0
H1-8P1	0	0	0	0	0	0	0	0
H1-8P2	0	0	0	0	0	0	0	0
H1-9P	0	0	0	0	0	0	0	0
H2AB3	0	0	0	2.35974	0	0.35888	0	0.643468
H2AC10P	0	0	0	0.414168	0	0	0	0
H2AC25	22.152916	19.744675	23.377333	13.879087	24.405582	21.924211	0	16.133339
H2AC2P	0	0	0	0	0	0	0	0
H2AC3P	0	0	0	0	0	0	0	0
H2AC5P	0	1.187313	0	0	4.19439	3.577224	0	0.712014
H2AC6	0	0	0	0	0	0	0	0
H2AC9P	0	0	0	0	0	0	0	0
H2ACP1	0	0	0	0	0	0	0	0
H2ACP2	0	0	0	0	0	0	0	0
H2AJ	0	0.755056	0	2.316344	0.289646	0.056242	0	0.50724
H2AL1MP	0	0	0	0	0	0	0	0
H2AQ1P	0	0	0	0	0	0	0	0
H2AX	0	326.440705	276.408813	345.715249	81.276971	123.56922	157.662477	63.629588
H2AZ1	421.932151	401.194326	319.021948	295.665947	323.370811	322.903347	311.416381	414.350247
H2AZ2	8.123984	64.012349	44.348097	32.810072	194.846462	56.332521	129.515939	96.898694
H2AZ2P1	0	0	0	0	0	0	0	0
H2AZP1	0	0	0	0	0	0	0	0
H2AZP2	0	0	0	0	0	0	0	0
H2AZP3	0	0	0	0	0	0	0	0
H2AZP4	0	0	0	0	0	0	0	0
H2AZP5	0	0	0	0.352709	0	0.676608	0	0
H2AZP6	0	0	0	0	0	0	0	0
H2AZP7	0	0	0	0	0	0	0	0

H2BC11	0	0	0	0	0	0.680547	0	0.591723
H2BC15	38.845486	106.204806	57.472206	119.173921	119.601154	134.45514	4.907029	112.343232
H2BC16P	0	0	0	0	0	0	0	0
H2BC18	162.602504	315.654364	186.865906	300.796033	157.247969	345.142367	163.873351	220.190384
H2BC19P	0	2.205418	0	8.613629	16.333724	0	0	11.264453
H2BC20P	0	0	0	0	0	4.797231	0	0
H2BC4	0	0	0	0	0	0	0	0
H2BC5	6.742512	1.132382	0	3.1529	0	0.366211	0	8.915903
H2BC6	0	0.12655	1.282923	0.10873	0	0	0	0.117374
H2BL1P	0	0	0	0	0	0	0	0
H2BP1	0	0	0	0.388303	0	1.597111	0	0
H2BP2	0	0	0	0	0	0	0	4.577976
H2BP3	0	0	0	0	0	0	0	0
H2BP5	0	0	0	0	0	0	0	0
H2BP6	0	0	0	0	0	0	0	0
H2BP7	0	0	0	0	0	0	0	0
H2BP8	0	0	0	0	0	0	0	0
H2BP9	0	0	0	0.287432	0	0	0	0
H2BW2	0	0	0	0.215296	0	0	0	0
H3-3A	0	224.016713	163.927841	88.140214	0	328.262742	506.910368	9.318959
H3-3B	568.892429	796.072419	470.842387	710.946278	380.328459	1136.75776	594.198351	1056.02903
H3-5	3.423735	0	0	0	0	0	0	0
H3-7	0	0	0	0.04167	0	0	0	0
H3C12	0	7.296306	0	0.849013	9.465757	6.461231	0	5.159464
H3C5P	0	0	0	0	0	0	0	0
H3C6	3.789297	8.605876	0	3.641526	1.118558	4.522519	6.132323	10.259322
H3C9P	0	3.950054	0	0.610482	0	0	0	0
H3P1	0	0	0	0	0	0	0	0
H3P10	0	0	0	0	0	0	0	0
H3P11	0	0	0	0	0	0	0	0
H3P12	0	0	0	0	0	0	0	0
H3P13	0	0	0	0	0	0	0	0.248382
H3P14	0	0	0	0.320357	0	0	0	0

H3P15	0	0	0	0	0	0	0	0
H3P16	0	0	0	0	0	2.322646	0	0.873101
H3P17	0	0	0	0	0	0	0	0
H3P18	0	0	0	0	0	0	0	0
H3P19	0	0	0	0	0	0	0	0
H3P2	0	0	0	0	0	0	0	0
H3P20	0	0	0	0	0	0	0	0
H3P21	0	0	0	0	0	0	0	0
H3P22	0	0	0	0	0	0	0	0
H3P23	0	0	0	0	0	0	0	0
H3P24	0	0	0	0	0	0	0	0
H3P25	0	0	0	0	0	0	0	0
H3P26	0	0	0	0	0	0	0	0
H3P27	0	0	0	0	0	0	0	0
H3P28	0	0	0	0	0	0	0	0
H3P29	0	0	0	0	0	0	0	0
H3P3	0	0	0	0	0	0	0	0
H3P30	0	0	0	0	0	0	0	0
H3P31	0	0	0	0	0	0	0	0
H3P32	0	0	0	0	0	0	0	0
H3P33	0	0	0	0	0	0	0	0
H3P34	0	0	0	0	0	0	0	0
H3P35	0	0	0	0	0	0	0	0
H3P36	0	0	0	0	0	1.329248	0	0
H3P37	0	0	0	0	0	0	0	0
H3P38	0	0	0	0	0	0	0	0
H3P39	0	0	0	0	0	0	0	0
H3P4	0	0	0	0	0	0	0	0
H3P40	0	0	0	0	0	0	0	0
H3P41	0	0	0	0	0	0	0	0
H3P42	0	0	0	0	0	0	0	0
H3P43	0	0	0	0	0	0	0	0
H3P44	0	0	0	0.291689	0	0	0	0

H3P45	0	0	0	0	0	0	0	0
H3P46	0	0	0	0	0	0	0	0
H3P47	0	0	0	0	0	0	0	0
H3P5	0	0	0	0	0	0	0	0
H3P6	0	261.973332	0	248.634043	0	165.101234	168.963963	321.202492
H3P7	0	0	0	0	0	0	0	0
H3P8	0	0	0	0	0	0	0	0
H3P9	0	0	0	0	0	0	0	0
H4C10P	0	0	0	0	0	0	0	0
H4C14	7.819903	4.8012	11.37605	14.589373	25.595432	2.892342	20.660326	3.604815
H4C15	0	7.680051	0	0	0	0	0	0
H4C16	0	0	0	0.596693	0	0.460475	0	2.092957
H4C8	14.47907	29.162114	12.93952	19.159577	38.037019	20.245268	7.844072	22.332602
H4P1	0	0	0	0	0	0	0	0
H6PD	8.797085	2.035008	0	0.944073	19.497058	7.154674	2.119219	2.089568
HAAO	6.740504	7.241716	4.780398	5.712889	0	3.157077	0	1.23191
HABP2	0	0	0	0	0	0	0	0
HABP4	6.387162	14.575684	11.435083	15.614736	10.448892	12.76775	0	7.918725
HACD1	4.611689	2.800581	5.152883	2.354172	0	1.830853	1.923838	3.655345
HACD2	25.590409	11.888855	26.558791	6.349326	43.928385	7.795633	26.859482	18.078635
HACD3	89.03779	99.343247	120.67582	90.739557	124.766926	68.475769	67.285658	101.514885
HACD4	0	0	0	0.047659	0	0	0	0
HACE1	9.584535	21.417699	17.661052	14.049517	25.045198	10.59941	6.823565	19.199391
HACL1	14.86411	8.575273	15.871567	9.953866	18.558395	5.012386	0	6.021569
HADH	22.978652	2.472647	39.241912	5.561648	18.742986	12.202006	13.449063	0.607792
HADHA	103.59642	142.72266	89.025935	148.810701	94.359689	91.035042	66.201397	92.832775
HADHAP1	0	0	0	0	0	0	0	0
HADHAP2	0	0	0	0	0	0	0	0
HADHB	89.959351	61.521667	95.276585	60.442485	52.31111	36.789997	74.16425	50.179723
HADHBP1	0	0	0	0	0	0	0	0
HAGH	8.809196	47.595869	20.724279	46.614306	11.900644	57.209547	23.174819	38.511918
HAGHL	0	16.504743	11.066652	8.474215	4.28477	10.835563	0	4.062579
HAL	0	0	0	0.290754	0	0	0	0

HAMP	0	0	0	0	0	0	0	0
HAND1	2.077194	2.363032	0	1.661428	0	0	0	0
HAND2	0	3.143106	0	0.720524	0	0.284938	0	0
HAO1	0	0	0	0	0	0	0	0
HAO2	0	0	0	0	0	0	0	0
HAP1	2.558115	2.176447	3.060605	1.67586	1.125851	1.40635	0	0.616481
HAPLN1	0	0	0	0	0	0	0	0
HAPLN2	0	2.856145	0	2.901196	0	0	0	0
HAPLN3	0	0	4.472142	0.096945	0.608827	2.015884	0.557901	0.192861
HAPLN4	0	0.032492	0	0.222142	0	0.644455	0	0
HAPSTR1	60.602438	65.141791	62.315633	49.005249	47.430439	29.473119	49.711636	60.341641
HARBI1	5.302122	7.082948	3.155405	12.861873	2.329569	11.343116	42.39757	19.141924
HARS1	50.263719	65.292192	52.477783	72.046416	76.726297	71.901048	103.3378	62.900547
HARS2	11.038124	47.396667	29.747757	33.457537	20.383639	26.730392	37.329596	27.136127
HAS1	0	0	0	0	0	0	0	0
HAS2	4.662172	4.628763	4.185271	4.421234	1.026077	0.505103	0	3.119726
HAS3	70.424912	126.377348	105.050516	163.44495	42.998238	157.574907	47.286328	117.896492
HASPIN	8.442473	10.125125	4.320249	8.791497	18.141553	18.306108	3.807256	15.869254
HAT1	82.579611	51.246302	61.935639	20.048067	104.186659	14.38519	118.089653	73.80098
HAUS1	78.805641	81.66278	28.219981	53.987545	106.029777	76.355863	162.839778	89.732264
HAUS1P1	0	0	0	0	0	0	0	0
HAUS1P2	0	0	0	0	0	0	0	0
HAUS1P3	0	0	0	0	0	0	0	0
HAUS2	40.154845	32.361156	31.679188	23.814839	28.259053	12.023956	28.351422	23.994203
HAUS3	9.156608	3.402755	0	2.075935	1.35492	10.141177	8.475934	3.166323
HAUS4	67.962616	60.072971	41.627308	35.829974	47.931361	38.277848	13.335431	49.06612
HAUS4P1	0	0	0	0	0	0	0	0
HAUS5	3.806322	8.992834	10.49321	12.538069	13.642102	28.429112	17.892585	16.056996
HAUS6	25.996403	36.674863	18.00128	14.261165	37.746992	9.574645	41.390135	42.418677
HAUS6P1	0	0	0	0.061727	0	0	0	0.372693
HAUS6P2	0	0	0	0	0	0	0	0
HAUS6P3	0	0	0	0	0	0	0	0
HAUS7	0	19.092548	20.768542	9.227366	34.534194	13.773636	0	1.160394

HAUS8	7.325954	18.153849	31.624576	16.809816	75.467312	27.215831	24.343836	28.127172
HAUS8P1	0	0	0	0	0	0	0	0
HAVCR1	0	0	0	0	0	0	0	0
HAVCR2	0	0	0	0	0	0	0	0
HAX1	0	15.511411	0	3.531152	0	14.117517	20.29994	5.746836
HAX1P1	0	0	0	0	0	0	0	0
HBA1	0	0	0	0	0	0.446467	0	0
HBA2	0	2.512337	0	0.988856	0	0	0	0.605006
HBAP1	0	0	0	0	0	0	0	0
HBB	0	0	0	0	0	0	0	0
HBBP1	0	0	0	0	0	0	0	0
HBD	0	0	0	0	0	0	0	0
HBE1	0	0	0	0	0	0	0	0
HBEGF	28.991319	57.143088	70.0773	134.058806	41.25532	47.770236	22.306893	72.592093
HBG1	0	0	0	0	0	0	0	0
HBG2	0	0	0	0.520217	0	0	0	0
HBM	0	1.891225	0	0	0	0	0	0
HBP1	7.503421	20.29006	26.372652	18.389045	13.529573	5.369957	60.04039	9.108442
HBQ1	0	1.058758	0	1.490522	0	1.254936	0	0.765517
HBS1L	71.320512	291.923933	171.105154	211.74102	38.327299	134.456244	278.354947	231.578559
HBZ	0	0	0	0.607125	0	0	0	0
HBZP1	0	0	0	0	0	0	0	0
HCCS	42.768342	100.563064	27.140918	65.514473	98.575799	133.856567	90.240062	132.209829
HCFC1	53.898979	135.201487	42.090867	132.528019	44.729917	170.379923	47.229496	142.428342
HCFC1R1	43.501423	44.59666	41.048618	45.44484	75.04838	24.404981	0	21.279682
HCFC2	8.032458	8.736964	8.385823	5.44192	4.744064	4.961389	9.420375	7.390945
HCFC2P1	0	0	0	0	0	0	0	0
HCG19P	0	0	0	0	0	0	0	0
HCG22	0	0	0	0	0	0	0	0
HCG4	11.610732	13.864497	6.828602	11.327468	0	15.995067	0	12.473746
HCG4B	0	0	0	0.18727	0	0.188619	0	0
HCG4P8	0	0	0	0	0	0	0	0
HCG9P5	0	0	0	0	0	0	0	0

HCK	0	0	0	0.147111	0	0	0	0
HCLS1	0	0	0	0.042326	4.221707	0.742308	0	0
HCN1	0	0	0	0	0	0	0	0
HCN2	0	0.499555	2.619909	0.878967	1.028466	0.388733	0	0.430553
HCN3	2.675366	1.057032	0.922932	2.341182	0	1.149872	0	1.044384
HCN4	0	0	0	0.06866	0	0	0	0
HCRT	0	0	0	0	0	0	0	0
HCRTR1	0	1.592507	0	0.644106	0	0	0	0
HCRTR2	0	0	0	0	0	0	0	0
HCST	0	0	0	0.550734	0	0	0	1.015629
HDAC1	122.205934	136.9895	85.885253	128.075006	173.150586	177.202681	77.36612	155.906595
HDAC10	0	5.433265	10.651569	9.782237	4.495114	14.264229	0	6.561606
HDAC11	5.085407	2.75261	9.836442	7.078164	0.315622	5.830222	0.296357	3.957961
HDAC1P1	0	0.732965	0	0.120835	0	0.245181	0	0.101218
HDAC1P2	0	0.434505	0	0.062721	0	0	0	0.631131
HDAC2	130.877133	113.28596	166.184693	93.4456	112.602988	79.508885	52.934892	151.900049
HDAC3	115.452961	71.004665	72.474305	69.690846	65.066615	65.19463	129.375705	79.377701
HDAC4	11.673774	3.927492	0	1.87353	3.600508	11.53949	31.622317	2.427284
HDAC5	8.356423	7.226863	12.48062	10.602416	15.370447	13.335445	22.516865	9.044918
HDAC6	3.74408	5.466234	0	6.201659	4.213152	7.927972	1.515969	7.103151
HDAC7	15.429184	18.481416	49.315608	21.470007	105.351598	47.470889	75.207731	29.963201
HDAC8	50.886631	27.189223	35.991463	31.918528	57.684328	21.285542	65.706942	22.607792
HDAC9	42.390538	72.294847	38.847514	139.575829	47.877248	60.189387	24.841535	45.685038
HDC	0	0	0	0	0	0	0	0
HDDC2	5.384086	8.628029	16.819022	8.498774	39.535791	9.137757	57.259683	14.988066
HDDC3	21.903597	52.938444	9.673469	39.174408	35.994408	49.99551	0	27.680826
HDGF	212.831945	596.073704	93.061392	647.08372	90.060946	1193.70444	21.60147	813.925167
HDGFL2	28.744736	40.837712	31.106746	44.387663	22.691092	63.271301	37.290302	38.66939
HDGFL3	71.140959	207.993152	87.286232	157.109368	169.531738	129.284386	137.61432	162.382147
HDGFL3P1	0	0	0	0	0	0	0	0
HDGFP1	0	29.119626	0	32.971666	0	45.282007	0	19.795255
HDHD2	46.79734	23.751999	5.629542	33.880121	22.296641	10.764697	32.127674	35.347669
HDHD3	3.819601	15.806326	0	17.595347	0	21.911882	0	18.393199

HDHD5	24.380048	34.531353	16.298363	35.175119	35.953329	69.376804	12.947816	78.122751
HDLBP	223.698345	473.723709	280.701882	669.370965	414.340419	857.731886	1191.99446	638.312253
HDX	4.10836	4.620491	11.996705	5.397632	19.179635	2.968031	1.242319	8.699527
HEATR1	38.224289	34.201576	46.224169	39.037536	70.994168	32.508969	74.764117	36.796359
HEATR3	12.660184	32.402758	21.198082	13.474826	51.790561	11.514337	26.608574	18.227665
HEATR4	0	0.943381	0	0.199767	0	0.622371	0	1.425338
HEATR5A	12.397174	48.938825	9.808715	41.231804	31.452328	35.301688	13.956076	45.695363
HEATR5B	5.600628	4.371713	8.394362	3.813591	7.07569	2.014004	0	4.134622
HEATR6	10.737022	0	0	2.261492	3.936598	0	29.563401	1.090737
HEATR9	0	0	0	0	0	0	0	0
HEBP1	42.008489	77.943581	24.107063	87.524974	74.344241	116.420505	55.084256	95.176905
HEBP2	38.045846	55.658161	46.103223	23.28338	56.939754	27.685448	46.494849	43.802737
HEBP2P1	0	0	0	0	0	0	0	0
HECA	6.924296	8.901212	11.408972	7.23736	6.555361	14.748051	4.077833	12.960333
HECTD1	22.754019	28.112314	35.127131	23.566981	61.269234	41.81985	0	51.82397
HECTD2	1.511576	0	0	0	0.199614	0.882229	0.735968	0.406304
HECTD3	8.310612	6.280051	6.626047	6.147631	6.80899	17.612605	14.583896	8.573042
HECTD4	6.273365	8.190048	8.739485	9.865164	30.730414	22.010492	75.790706	17.209933
HECW1	0	3.348688	0	0.465227	0	0	0	0
HECW2	0	0.020835	0	0.108322	0.166619	0	0	0.076874
HEG1	9.443859	12.583704	13.155119	11.226649	20.416933	30.855423	27.344946	21.899595
HELB	0	2.989422	4.892433	2.296782	0	3.138301	6.162262	6.611493
HELLS	76.043838	58.074604	0	22.487946	0	14.566713	0	48.377435
HELQ	5.032793	5.760029	22.104272	5.923968	0.265722	5.682697	2.597608	6.642479
HELT	0	0	0	0	0	0	0	0
HELZ	21.66299	45.030145	41.192418	43.35684	34.160392	38.117793	67.747882	67.560757
HELZ2	4.953305	8.718337	18.620562	17.909841	99.958406	105.892218	122.100028	49.502784
HEMGN	0	0	0	0	0	0	0	0
HEMK1	21.560299	9.097046	12.170048	12.164875	9.212856	6.696645	29.417483	7.524171
HENMT1	0	0.179529	0	0.215025	0	0.525092	0	1.465363
HEPACAM	0	0	0	0	0	1.77835	0	0
HEPACAM2	0	0	0	0.136146	0	0	0	0
HEPH	0	0	0	0	0	0	0	0

HERC1	35.67136	37.137847	33.125389	32.692957	75.60963	23.24562	122.655616	34.229611
HERC2	29.087263	40.288017	25.483006	55.096249	74.173758	84.929035	148.395463	90.252513
HERC2P1	0	0	0	0	0	0	0	0
HERC2P10	2.853206	0	0	0	0.376317	0.361374	0	0.177364
HERC2P11	0	0	0	0	0	0	0	0
HERC2P2	0	4.279636	0	2.140795	0	0	0	3.104379
HERC2P3	0	0	0	3.381051	3.873367	2.953024	0	1.248821
HERC2P4	0	0	0	0	0	0	0	0
HERC2P5	0	0.037529	0	0	0	0.043239	0	0.034805
HERC2P6	0	0	0	0	0	0	0	0
HERC2P7	0	0	0	0	0	0	0	0
HERC2P8	0	0	0	0	0	0	0	0
HERC2P9	4.95615	4.698595	7.647018	4.480326	0.233503	8.754296	1.041045	4.975601
HERC3	0	14.214881	13.544375	16.457476	9.328628	18.937754	13.287142	21.86104
HERC4	12.189444	13.97995	41.312517	26.514661	10.755229	23.76841	37.774156	40.027762
HERC5	1.894535	1.458191	2.736184	1.663901	13.15485	6.240531	11.313029	7.384712
HERC6	0	1.665969	11.010885	7.685114	71.842556	31.453419	61.00546	16.916569
HERPUD1	71.461426	184.042814	160.079914	346.362938	90.137263	162.620141	108.767021	112.747225
HERPUD2	38.801911	65.360497	25.091902	60.06839	66.268776	74.148815	6.803819	87.809427
HES1	147.287117	168.662845	154.482252	167.815477	86.353632	81.562013	61.129898	46.994759
HES2	2.318511	6.23191	4.856592	9.01316	4.694529	43.619759	9.782467	28.035385
HES4	0	20.359958	5.131733	11.635173	0	8.781338	0	3.857184
HES5	2.808206	0	0	0.406676	12.560887	0.82384	16.895393	1.79379
HES6	0	4.491674	8.187742	3.728556	4.513259	2.606565	0	4.128679
HES7	2.08682	2.197858	0	3.782738	0.549801	3.861387	0	2.674744
HESX1	0	0	0	0.960217	0	0	0	0
HEXA	68.2635	30.733105	78.125063	58.835462	38.442647	42.843312	26.382916	28.144703
HEXB	118.277504	75.876126	107.026547	69.985018	35.707232	45.577613	91.047438	56.024003
HEXD	9.174846	8.465409	2.681962	11.033445	9.094806	6.851988	0	13.561764
HEXIM2	0	3.955172	5.625628	4.902093	8.026124	7.22727	0.762779	7.018102
HEY1	0	2.090506	1.399072	2.123562	0	0.520072	0	0.120256
HEY2	0	1.812827	0	0.691263	0	0.705782	0	0.278704
HEYL	0	0.450836	0	0.889467	0	0.080932	0	0.065018

HFE	0	5.065584	5.044223	2.913127	0	1.503498	0	2.223372
HFM1	0	0	0	0	0	0	0	0
HGD	0	0	0	0	0	0	0	0
HGF	1.251066	0	0	0.078998	2.26461	0	0	0
HGFAC	0	0	0	0	0	0	0	0
HGH1	1.96089	7.220873	3.497075	7.510738	13.047279	10.759995	9.957006	8.504916
HGS	73.317795	60.505482	88.56777	74.31696	31.92218	70.238307	18.193591	42.343751
HGSNAT	7.499558	5.41284	16.715452	6.476116	29.401439	6.143021	23.478254	7.556691
HHAT	2.845223	4.560515	1.732762	3.235785	2.408483	4.885464	15.422647	8.553276
HHATL	0	0	0	0	0	0.187508	0	0
HHEX	6.652455	7.93478	3.948967	6.349239	1.322502	2.845865	14.456288	3.983767
HHIP	0	4.314095	0	0.007913	0	0	0	0.325487
HHIPL1	0	0.373253	0	0.592439	0	0.149509	0	0.479063
HHIPL2	0	0	0	0	0	0	0	0.053133
HHLA1	0	0	0	0.073541	0.398821	1.274957	0	0.731306
HHLA2	0	0	0	0	0	0	0	0
HIBADH	64.683643	140.447387	104.561201	134.136982	55.894452	55.693105	11.277709	76.899033
HIBCH	9.804281	26.767703	84.411377	12.874313	70.490692	12.951027	38.050261	35.985169
HIC1	0	0.665202	0.456089	0.830364	0	1.296534	0	1.197657
HIC2	1.422484	2.436014	2.131964	1.676734	2.380654	6.33486	0	5.121259
HID1	18.729891	2.649806	2.727925	4.367071	0	6.550825	0	2.783861
HIF1A	266.643829	323.474219	160.986739	267.575895	184.709874	294.547867	414.289666	489.592997
HIF1AN	71.16594	63.24522	38.102356	67.727	80.287151	35.220238	35.880572	59.445386
HIF1AP1	0	8.986373	0	0	0	0	0	0
HIF3A	0	0	0	0	0	0	0	0
HIGD1A	0	23.838299	28.26378	8.979558	62.672365	13.528295	64.647005	11.495451
HIGD1AP1	0	0	0	0	0	0	0	0
HIGD1AP10	0	0	0	0	0	0	0	0
HIGD1AP11	0	0	0	0	0	0	0	0
HIGD1AP12	0	0	0	0	0	0	0	0
HIGD1AP13	0	0	0	0	0	0	0	0
HIGD1AP14	0	0	0	0	0	0	0	0
HIGD1AP15	0	0	0	0	0	0	0	0

HIGD1AP16	0	5.371608	0	0.730795	0	0	0	0
HIGD1AP17	0	0	0	0	0	0	0	0
HIGD1AP18	0	0	0	0	0	0	0	0
HIGD1AP2	0	0	0	0	0	0	0	0
HIGD1AP3	0	0	0	0	0	0	0	0
HIGD1AP4	0	0	0	0	0	0	0	0
HIGD1AP5	0	0	0	0	0	0	0	0
HIGD1AP6	0	0	0	0	0	0	0	0
HIGD1AP8	0	0	0	0	0	0	0	0
HIGD1AP9	0	0	0	0	0	0	0	0
HIGD1B	0	0	0	0	0	0	0	0
HIGD1C	0	0	0	0	0	0	0	0
HIGD2A	66.975417	133.752935	157.050443	112.648017	103.204113	107.337391	64.695985	125.305534
HIGD2AP1	0	0	0	0	0	0	0	0
HIGD2AP2	0	0	0	0	0	0	0	0
HIKESHI	119.798723	141.023995	52.658424	83.809239	45.607311	39.422667	29.050142	69.073395
HIKESHIP1	0	1.376784	0	0.282279	0	0	0	0
HIKESHIP2	0	4.805353	0	3.100002	0	0	0	6.007169
HIKESHIP3	0	0	0	0	0	0	0	0
HILPDA	29.372455	60.437359	26.087086	83.674926	41.462215	51.516892	17.420502	45.645225
HINFP	44.832946	21.10604	39.612096	54.162986	11.413072	16.292739	35.13067	8.86549
HINT1	0	5.986889	0	23.526251	151.464443	46.924379	25.211422	5.308135
HINT1P1	0	0	0	0	0	0	0	0
HINT1P2	0	0	0	0	0	0	0	0
HINT2	20.308222	45.736901	43.578039	59.145791	100.337	64.653906	47.827896	62.303047
HINT2P1	0	0	0	0	0	0	0	0
HINT3	17.057027	8.788747	12.593871	7.145269	9.00584	6.910409	13.171672	8.22752
HIP1	18.655271	24.102333	27.055478	22.659769	13.732042	23.218167	9.425449	17.55758
HIP1R	19.74433	12.411394	15.049914	16.661504	27.973469	20.358301	13.133677	18.321474
HIPK1	24.520842	32.900056	37.975269	37.994567	36.205675	49.246231	34.543756	41.483304
HIPK2	15.251155	14.806741	14.972996	17.986313	13.535151	21.916358	16.773177	17.217064
HIPK3	22.861305	24.531006	22.237391	21.389039	49.951673	70.055617	87.74064	123.643549
HIRA	11.40413	19.396796	9.504299	19.554859	15.039836	46.088948	58.727805	33.222822

HIRAP1	0	0	0	0	0	0	0	0
HIRIP3	0	19.460198	0	21.255452	0	6.815282	20.617739	26.676616
HIVEP1	0	8.343853	0	11.933564	0	10.960018	10.71973	13.459665
HIVEP2	8.961287	18.62568	10.436326	22.127819	23.11946	53.675188	16.993657	46.322191
HIVEP3	4.667374	6.456941	3.301892	5.928602	1.454228	10.273013	0	4.380322
HJURP	23.320989	29.282522	26.263574	31.540284	51.213234	67.942707	57.239895	47.395784
HJV	0	0	0	0.157976	0	0.140461	0	0
HK1	0	0	0	1.319501	0	0.372317	0	0.094095
HK2	0	8.251335	0	29.526696	0	37.088533	89.778782	0.154255
HK2P1	0	0	0	0	0	0	0	0.050381
HK3	0	0	0	0	0	0	0	0
HKDC1	2.72549	20.737228	14.057358	18.521424	3.358293	11.91291	2.872709	12.8373
HLA-A	115.670825	95.426664	197.267168	186.569531	239.479681	215.200392	274.557176	120.556549
HLA-B	126.385212	89.556848	229.893411	245.457416	357.492514	298.07721	146.190713	139.150125
HLA-C	127.394339	124.286276	219.652514	179.546212	315.301852	307.237681	137.501077	148.044296
HLA-DMA	9.397894	3.984743	9.295226	0.833406	32.868462	5.58237	0	2.481494
HLA-DMB	0	0.226257	0	0.196117	0	3.525318	0	0.76905
HLA-DOA	0	0.123235	1.710344	1.117387	2.518348	1.047251	0	0.306604
HLA-DOB	5.50728	2.075998	0	1.199007	0	0.700026	0	0.095904
HLA-DPA1	0	8.399985	1.946091	16.362957	0	4.587873	0	9.701202
HLA-DPA2	0	0	0	0	0	0	0	0
HLA-DPA3	0	0	0	0	0	0	0	0
HLA-DPB1	23.816375	38.62459	21.218754	51.627443	0	45.507184	74.803328	31.168698
HLA-DPB2	0	0	0	0	0	0	0	0
HLA-DQA1	0.357468	0.581306	0	0.580203	1.228263	0.347726	0	0.466989
HLA-DQA2	0	0	0	0	0	0	0	0
HLA-DQB1	0	0.153395	0	0.342528	3.919561	6.14619	213.406269	3.002979
HLA-DQB2	0	0	0	0	0	0	0	0
HLA-DQB3	0	0	0	0	0	0	0	0
HLA-DRA	0	0	0	0	17.252695	4.687352	16.898232	2.132759
HLA-DRB1	0	0	0	0.076201	0	0.634646	1.519542	0.234836
HLA-DRB5	0	0.123131	0	0.570228	9.329576	1.731419	0	0.600055
HLA-DRB6	0	0	0	0	0	0	0	0

HLA-DRB7	0	0	0	0	0	0	0	0
HLA-DRB8	0	0	0	0	0	0	0	0
HLA-DRB9	0	0	0	0	0	0	0	0
HLA-E	71.107644	31.213112	102.763309	62.773799	64.180291	62.683577	52.858247	29.435366
HLA-F	57.670336	30.931175	55.058147	40.307122	29.413633	54.207903	46.885539	21.73775
HLA-G	0	0	0	0	0	0.162579	0	0
HLA-H	0	2.170399	0	2.209378	0	4.845353	0	5.451018
HLA-J	0	0.7218	0	0.730848	0	0	0	0
HLA-K	0	0	0	0.529455	0	3.546678	0	1.643181
HLA-L	0	0	0	0.46258	5.00232	1.06442	0	0.047009
HLA-N	0	0	0	0	0	0	0	0
HLA-P	0	0	0	0	0	0	0	0
HLA-S	0	0	0	0	0	0	0	0
HLA-T	0	0	0	0	0	0	0	0
HLA-U	0	4.014125	0	6.099767	0	2.84881	0	2.837602
HLA-V	5.694062	7.023774	2.543588	6.877251	21.244555	15.987336	0	11.388475
HLA-W	0	0	0	0	0	0	0	0
HLA-Z	0	0	0	0	0	0	0	0
HLCS	7.573657	17.345066	6.808074	18.414591	7.960026	15.6213	0.659285	19.988169
HLF	0	0	0	0.142426	0	0	0	0
HLFP1	0	0	0	0	0	0	0	0
HLTF	45.702284	33.914651	33.173433	30.818155	19.853568	6.940568	12.892762	16.231732
HLX	0	0.48609	0	0.330227	1.214476	0.080175	0	0.064771
HM13	155.273724	149.450004	153.920351	147.874885	134.83566	88.385219	57.785004	64.281382
HMBOX1	15.439723	12.373447	14.276808	20.343215	125.744671	13.506858	32.424075	19.234656
HMBS	125.654075	212.311413	160.358481	213.66113	51.467574	73.840674	77.063951	59.897071
HMCES	108.109268	164.119682	85.172511	201.432313	108.583564	191.019867	109.612257	137.544177
HMCN1	0.519996	1.150562	5.12502	0.229375	0	0.544419	0	1.448465
HMCN2	0	0	0	0.463378	0	0	0	0.727186
HMG20A	20.861014	39.852594	36.249095	68.19476	49.930271	53.301745	83.967739	49.425114
HMG20B	37.316583	82.218385	53.21767	86.909026	78.915428	84.859534	7.474158	63.132186
HMGA1	632.90016	886.395835	860.488469	1105.10636	321.592066	439.009041	340.718217	357.311116
HMGA1P1	0	0.471169	0	0.086248	0	0.348354	0	0

HMGA1P2	0	2.145106	0	0	0	0	0	0
HMGA1P3	0	0	0	4.238703	0	0	0	0
HMGA1P5	0	0	0	0	0	0	0	0
HMGA1P6	0	0	0	0	0	0	0	0
HMGA1P7	0	0	0	0	0	0	0	0
HMGA1P8	0	8.892331	0	19.153133	0	12.662445	0	5.587572
HMGA2	3.162974	81.268729	23.177953	137.10477	24.959899	222.622097	164.546456	178.949991
HMGB1	465.178769	540.531396	320.518507	442.672092	542.451794	508.254856	409.86717	551.227364
HMGB1P10	0	0.851073	0	0	0	1.328264	0	0.780904
HMGB1P11	0	0	0	0	0	0	0	0
HMGB1P12	0	0	0	0	0	0	0	0
HMGB1P13	0	0	0	0	0	0	0	0
HMGB1P14	0	0	0	0	0	0	0	0
HMGB1P15	0	0	0	0	0	0	0	0
HMGB1P16	0	0	0	0	0	0	0	0
HMGB1P17	0	0	0	0	0	0	0	0
HMGB1P18	0	0	0	0	0	0	0	0
HMGB1P19	0	0	0	0.187674	0	0	0	1.00078
HMGB1P20	0	0	0	0	0	0	0	0
HMGB1P21	0	0	0	0	0	0	0	0
HMGB1P22	0	0	0	0	0	0	0	0
HMGB1P23	0	0	0	0	0	0	0	0
HMGB1P24	0	0	0	0	0	0	0	0
HMGB1P25	0	0	0	0	0	0	0	0
HMGB1P26	0	0	0	0	0	0	0	0
HMGB1P27	0	0	0	0	0	0	0	0
HMGB1P28	0	0	0	0	0	0	0	0
HMGB1P29	0	0	0	0	0	0	0	0
HMGB1P3	14.567213	0	0	0	19.002213	0	25.662839	2.209683
HMGB1P30	0	0	0	0	0	0	0	0
HMGB1P31	0	2.381416	0	0.881605	0	0.568906	0	0
HMGB1P32	0	0	0	0	0	0	0	0
HMGB1P33	0	0	0	0	0	0	0	0

HMGB1P35	0	0	0	0	0	0	0	0
HMGB1P36	0	0	0	0	0	0	0	0
HMGB1P37	0	1.488125	0	0	0	0	0	0
HMGB1P38	0	0	0	0	0	0	0	0
HMGB1P39	0	0	0	0	0	0	0	0
HMGB1P4	0	0	0	0	0	0	0	0
HMGB1P40	0	0	0	0	0	0	0	0
HMGB1P41	0	1.367743	0	3.082953	0	0	0	1.553522
HMGB1P42	0	0	0	0	0	0	0	0
HMGB1P44	0	0	0	0	0	0	0	0
HMGB1P45	0	0	0	0	0	0	0	0
HMGB1P46	0	0	0	0	0	0	0	0
HMGB1P47	0	0	0	0	0	0	0	0
HMGB1P48	0	0	0	0	0	0	0	0
HMGB1P49	0	0	0	0.191383	0	0	0	0.340751
HMGB1P5	185.332164	99.316539	146.03859	42.420822	162.722533	21.329077	0	153.164993
HMGB1P50	0	0	0	0.174956	0	0	0	0
HMGB1P51	0	0	0	0	0	0	0	0
HMGB1P6	0	204.60862	22.932618	221.058242	0	262.722269	246.685756	252.870322
HMGB1P7	0	0	0	0	0	0	0	0
HMGB1P8	0	1.332281	0	0	0	0.397161	0	0
HMGB1P9	0	0	0	0	0	0	0	0
HMGB2	182.794393	260.82783	115.009907	247.284515	213.255712	357.180717	219.233298	239.319485
HMGB2P1	0	0	0	1.154267	0	0.981421	0	0
HMGB3	145.152391	208.558975	72.258705	169.912872	287.146657	353.043904	313.264901	292.653559
HMGB3P1	0	0	0	0	0	0	0	0
HMGB3P10	0	0	0	0	0	0	0	0
HMGB3P11	0	0	0	0	0	0	0	0
HMGB3P12	0	0	0	0	0	0	0	0
HMGB3P13	0	0	0	0	0	0	0	0
HMGB3P14	0	0	0	0	0	0	0	0
HMGB3P15	0	0	0	0	0	0	0	0
HMGB3P16	0	0	0	0	0	0	0	0

HMGB3P17	0	0	0	0.159789	0	0	0	0
HMGB3P18	0	0	0	0	0	0	0	0
HMGB3P19	0	0	0	0	0	0	0	0
HMGB3P2	0	0	0	0	0	0	0	0
HMGB3P20	0	0	0	0	0	0	0	0
HMGB3P21	0	0	0	0	0	0	0	0
HMGB3P22	0	0	0	0	0	0	0	0
HMGB3P23	0	0	0	0	0	0	0	0
HMGB3P24	0	1.138787	0	0	0	0	0	0
HMGB3P26	0	0	0	0	0	0	0	0
HMGB3P27	0	0	0	0	0	0	0	0
HMGB3P3	0	0	0	0	0	0	0	0
HMGB3P30	0	0	0	0	0	0	0	0
HMGB3P31	0	0	0	0	0	0	0	0
HMGB3P32	0	0	0	0.195241	0	0	0	0
HMGB3P4	0	0	0	0	0	0	0	0
HMGB3P5	0	0	0	0	0	0	0	0
HMGB3P6	0	0	0	0.179436	0	0	0	0
HMGB3P7	0	0	0	0	0	0	0	0
HMGB3P8	0	0	0	0	0	0	0	0
HMGB3P9	0	0	0	0	0	1.096427	0	0
HMGB4	0	0	0	0	0	0	0	0
HMGCL	31.152316	25.364814	18.213216	31.408059	31.135998	19.601128	37.901598	17.393834
HMGCLL1	0	0	0	0.169091	0	0	0	0
HMGCR	60.844463	35.443988	76.394056	34.587855	48.955608	24.751809	55.973408	18.76388
HMGCS1	206.057332	182.088542	111.621984	89.238987	118.910675	33.379327	81.024033	76.490227
HMGCS2	0	0	0	0	0	0	0	0
HMGN1	375.933164	629.236277	296.246708	451.151462	406.180471	740.737667	499.588987	600.470672
HMGN1P1	25.230641	0	0	0	0	0	0	0
HMGN1P10	0	0	0	0	0	0	0	0
HMGN1P11	0	0	0	0	0	0	0	0
HMGN1P12	0	0	0	0	0	0	0	0
HMGN1P13	0	0	0	0	0	0	0	0

HMGN1P14	0	0	0	0	0	0	0	0
HMGN1P15	0	0	0	0	0	0	0	0
HMGN1P16	0	0	0	0	0	0	0	0
HMGN1P17	0	0	0	0	0	0	0	0
HMGN1P18	0	0	0	0	0	0	0	0
HMGN1P19	0	0	0	0	0	0	0	0
HMGN1P2	0	0	0	0	0	0	0	0
HMGN1P20	0	0	0	0	0	0	0	0
HMGN1P24	0	0	0	0	0	0	0	0
HMGN1P26	0	0	0	0	0	0	0	0
HMGN1P28	0	0	0	0.382925	0	0	0	0
HMGN1P3	0	0	0	4.461244	0	1.86253	0	11.658939
HMGN1P30	0	2.020304	0	0	0	0	0	0
HMGN1P31	0	0	0	0	0	0	0	0
HMGN1P32	0	0	0	0	0	0	0	0
HMGN1P33	0	0	0	0	0	0	0	0
HMGN1P34	0	0	0	0	0	0	0	0
HMGN1P35	0	0	0	0	0	0	0	0
HMGN1P36	0	0	0	0	0	0	0	0
HMGN1P37	0	0	0	0	0	0	0	0
HMGN1P38	0	0	0	0	0	1.022543	0	0
HMGN1P4	0	0	0	0	0	0	0	1.178213
HMGN1P5	0	0	0	0	0	0	0	0
HMGN1P6	0	0	0	0	0	0	0	0
HMGN1P7	0	0	0	0.501683	0	0	0	0
HMGN1P8	0	1.775866	0	0	0	0	0	2.314262
HMGN1P9	0	0	0	0	0	0	0	0
HMGN2	83.31955	414.656018	96.434554	408.096976	262.982376	1066.70768	181.183953	550.166419
HMGN2P10	0	0	0	0	0	0	0	0
HMGN2P11	0	0	0	0	0	0	0	0
HMGN2P13	0	0	0	0	0	0	0	0
HMGN2P15	0	3.051156	0	6.796766	0	1.259402	0	0
HMGN2P16	0	0	0	0	0	0	0	0

HMGN2P17	0	0	0	0	0	0	0	0.231977
HMGN2P18	0	0	0	0	0	0	0	0
HMGN2P19	0	0	0	0	0	0	0	0
HMGN2P2	0	0	0	0	0	0	0	0
HMGN2P20	0	0	0	0	0	0	0	0
HMGN2P21	0	0	0	0	0	0	0	0
HMGN2P22	0	0	0	0	0	3.042288	0	0
HMGN2P23	0	0	0	0	0	0	0	0
HMGN2P24	0	0	0	0	0	0	0	0
HMGN2P25	0	0	0	0	0	0	0	0
HMGN2P26	0	0	0	0	0	0	0	0
HMGN2P27	0	0	0	0	0	0	0	0
HMGN2P28	0	0	0	0	0	0	0	0
HMGN2P3	0	0	0	0	0	0	0	1.497842
HMGN2P30	0	0	0	0	0	0	0	0
HMGN2P31	0	0	0	0	0	0	0	0
HMGN2P32	0	0	0	0.671777	0	0	0	0
HMGN2P34	0	0	0	0	0	0	0	0
HMGN2P35	0	0	0	0	0	0	0	0
HMGN2P36	0	0	0	0	38.861425	0	0	0
HMGN2P38	0	0	0	0	0	0	0	0
HMGN2P39	0	0	0	0	0	0	0	0
HMGN2P4	0	1.132649	0	0	0	2.7192	0	1.341269
HMGN2P40	0	0	0	0	0	0	0	0
HMGN2P41	0	0	0	0	0	1.201859	0	0
HMGN2P46	0	0	0	0	0	0	0	0
HMGN2P47	0	0	0	0	0	0	0	0
HMGN2P48	0	0	0	0	0	0	0	0
HMGN2P6	0	0	0	0	0	0	0	0
HMGN2P7	0	0	0	0	0	0	0	0
HMGN2P8	0	0	0	0	0	0	0	0
HMGN2P9	0	0	0	0	0	0	0	0
HMGN3	42.966358	39.968545	64.060621	37.005992	21.509228	45.097486	7.609361	30.942812

HMGN3P1	0	0	0	0	0	0	0	0
HMGN4	78.825243	138.646281	51.168193	111.95713	33.057381	88.400041	63.738045	117.794494
HMGN5	51.598777	12.288749	38.24536	11.592365	33.969566	7.388898	4.011429	13.491659
HMGXB3	21.118938	51.617808	31.339652	45.953046	15.313814	56.228917	47.371559	55.842457
HMGXB4	22.563349	65.076234	34.079399	76.647503	26.149988	61.142456	60.772679	62.8905
HMMR	70.185602	49.537938	57.051773	36.380333	33.919583	26.816484	34.417251	42.170926
HMOX1	11.500047	13.334737	16.376094	13.830289	2.422921	4.184175	0	2.699082
HMOX2	98.210649	207.027649	66.840197	216.63145	97.096032	259.538686	96.685928	188.980944
HMSD	0	0	0	0	0	0	0	0
HMX1	1.818354	4.535439	1.622744	2.868993	0	0	0	0
HNF1A	0	0	0	0	0	0	0	0
HNF1B	4.511791	13.721548	9.094967	13.455352	5.425138	6.514581	0	2.608879
HNF4A	0	0	0	0.382342	0	0.376297	0	0.127026
HNF4G	0	1.167838	0	6.698809	0	0.357486	0	0
HNF4GP1	0	0	0	0	0	0	0	0
HNMT	0	5.717749	18.673098	2.705503	22.590102	4.530737	0	8.454879
HNRNPA1	1714.80596	1320.0262	1996.37747	1664.20735	1405.18171	997.249275	1403.41955	835.332245
HNRNPA1P1	0	0	0	0.099284	0	0	0	0
HNRNPA1P1[illegible]	5.832405	2.588552	0	8.367334	0	1.141554	0	2.977496
HNRNPA1P1[illegible]	0	0	0	0	0	0	0	0
HNRNPA1P1[illegible]	0	0	0	0.097195	0	0	0	0
HNRNPA1P1[illegible]	0	0	0	0	0	0	0	0
HNRNPA1P1[illegible]	0	0	0	0.100444	0	0	0	0
HNRNPA1P1[illegible]	0	0	0	0	0	0	0	0
HNRNPA1P1[illegible]	0	5.592668	0	6.40121	10.725091	5.122898	0	9.178394
HNRNPA1P1[illegible]	0	0	0	0	0	0	0	0
HNRNPA1P1[illegible]	0	0	0	0	0	0	0	0
HNRNPA1P1[illegible]	0	0	0	0	0	0	0	0
HNRNPA1P2	0	0	0	0	0	0	0	0
HNRNPA1P2[illegible]	0	0	0	0	0	0	0	0
HNRNPA1P2[illegible]	0	0	0	0	0	0	0	0
HNRNPA1P2[illegible]	0	0	0	0.099029	0	0	0	0.337809
HNRNPA1P2[illegible]	0	0	0	0	0	0	0	0

HNRNPA1P2	0	0	0	0	0	0	0	0
HNRNPA1P2	0	0	0	0	0	0	0	0
HNRNPA1P2	0	0	0	0.250849	0	0	0	0
HNRNPA1P2	0	0.839076	0	0.293705	0	0	0	0
HNRNPA1P2	0	0	0	0	0	0	0	0
HNRNPA1P2	0	0	0	0	0	0	0	0
HNRNPA1P3	0	0	0	0	0	0	0	0
HNRNPA1P3	0	0	0	0	0	0	0	0
HNRNPA1P3	0	0	0	0	0	0	0	0
HNRNPA1P3	0	0	0	0	0	0	0	0
HNRNPA1P3	291.834053	558.306524	201.686406	369.646561	410.587362	644.916376	481.18181	687.290095
HNRNPA1P3	0	0	0	0	0	0	0	0
HNRNPA1P3	0	0	0	0.195105	0	0	0	0
HNRNPA1P3	0	0	0	0	0	0	0	0
HNRNPA1P3	0	0	0	0	0	0	0	0
HNRNPA1P3	0	0	3.591183	0	0	0	0	0
HNRNPA1P3	0	0	0	0.49052	0	0.197129	0	0.166149
HNRNPA1P4	0	0.719139	0	0.68108	0	0.819274	0	0.234858
HNRNPA1P4	0	0.480387	0	0	0	0	0	0
HNRNPA1P4	0	0	0	0	0	0	0	0
HNRNPA1P4	0	0	0	0	0	0	0	0
HNRNPA1P4	0	0	0	0	0	0.208666	0	0
HNRNPA1P4	0	0	0	0	0	0	0	0
HNRNPA1P4	0	0	0	0	0	0	0	0
HNRNPA1P4	0	0	0	0	0	0	0	0
HNRNPA1P4	0	0	0	0.100968	0	0	0	0
HNRNPA1P4	0	0	0	0	0	0	0	0
HNRNPA1P5	0	0	0	0	0	0	0	0
HNRNPA1P5	0	0	0	0	0	0	0	0
HNRNPA1P5	0	0	0	0	0	0	0	0
HNRNPA1P5	0	0.16947	0	0	0	0	0	0
HNRNPA1P5	0	0	0	0	0	0	0	0
HNRNPA1P5	0	0.042829	0	0.016199	0	0	0	0

HNRNPA1P5[illegible]	0	0	0	0.097905	0	0	0	0.333844
HNRNPA1P5[illegible]	0	0	0	0	0	0	0	0
HNRNPA1P5[illegible]	0	0	0	0	0	0	0	0
HNRNPA1P5[illegible]	0	0	0	0	0	0	0	0
HNRNPA1P5[illegible]	0	0	0	0	0	0	0	0
HNRNPA1P6	0	0	0	0	0	0.164091	0	0.274866
HNRNPA1P6[illegible]	0	0	0	0	0	0	0	0
HNRNPA1P6[illegible]	0	0	0	0	0	0	0	0
HNRNPA1P6[illegible]	0	0	0	0	0	0	0	0
HNRNPA1P6[illegible]	0	0	0	0	0	0	0	0
HNRNPA1P6[illegible]	0	0	0	0	0	0	0	0
HNRNPA1P6[illegible]	0	1.007948	0	0	0	0	0	0
HNRNPA1P6[illegible]	0	0.344462	0	0	0	0	0	0
HNRNPA1P6[illegible]	0	0	0	0	0	0	0	0
HNRNPA1P6[illegible]	0	0	0	0	0	0	0	0
HNRNPA1P6[illegible]	0	0	0	0	0	0	0	0
HNRNPA1P7	0	0	0	0.167296	0	0.843675	0	0.302792
HNRNPA1P7[illegible]	0	0.14436	0	0	0	0	0	0
HNRNPA1P7[illegible]	0	0	0	0	0	0	0	0
HNRNPA1P7[illegible]	0	0	0	0	0	0	0	0
HNRNPA1P7[illegible]	0	0	0	0.101365	0	0	0	0
HNRNPA1P7[illegible]	0	0	0	0	0	0	0	0
HNRNPA1P7[illegible]	0	0	0	0	0	0	0	0
HNRNPA1P8	0	0	0	0.099172	0	0	0	0.169367
HNRNPA1P9	0	0	0	0	0	0	0	0
HNRNPA2B1	168.555124	99.532896	47.24761	76.463177	188.334532	82.35781	344.228181	133.853214
HNRNPA3	16.7403	108.989824	18.270998	52.425999	26.647513	71.839984	130.371618	63.402759
HNRNPA3P1	0	0	0	0	0	0	0	0
HNRNPA3P1[illegible]	0	0	0	0	0	0	0	0
HNRNPA3P1[illegible]	0	0	0	0	0	0	0	0
HNRNPA3P1[illegible]	0	0	3.204426	0	0	0	0	0
HNRNPA3P1[illegible]	0	0	0	0	0	0	0	0
HNRNPA3P1[illegible]	0	0	0	0	0	0	0	0

HNRNPA3P1[illegible]	0	0	0	0	0	0	0	0
HNRNPA3P1[illegible]	0	0	0	0	0	0	0	0
HNRNPA3P1[illegible]	0	0	0	0	0	0	0	0.173302
HNRNPA3P2	6.585642	0	0	0.311226	0	0	0	0
HNRNPA3P3	0	0	0	0	0	0	0	0.138918
HNRNPA3P4	0	0	0	0	0	0	0	0
HNRNPA3P5	0	0	0	0	0	0	0	0
HNRNPA3P6	0	0	0	0	2.399972	0	0	0
HNRNPA3P7	0	0	0	0	0	0	0	0
HNRNPA3P8	0	0	0	0	0	0	0	0
HNRNPA3P9	0	0	0	0	0	0	0	0
HNRNPAB	214.571362	590.650225	185.041144	491.425607	344.094608	455.813871	261.594193	535.717213
HNRNPABP1	0	0	0	0	0	0	0	0
HNRNPC	143.838626	406.590973	155.279951	388.378292	289.224126	630.774649	221.350471	523.529319
HNRNPCL2	0	0	0	0	0	0	0	0
HNRNPCL4	0	0	0	0	0	0	0	0
HNRNPCP1	0	0	9.46529	1.221352	0	1.870352	0	0
HNRNPCP10	0	0	0	0	0	0	0	0
HNRNPCP2	0	0	0	0.83764	0	0	0	0
HNRNPCP3	0	0	0	0.108947	0	0	0	0
HNRNPCP4	0	0	0	0	0	0	0	0
HNRNPCP6	0	0.187306	0	0	0	0	0	0
HNRNPCP7	0	0	0	0	0	0	0	0
HNRNPCP8	0	0	0	0	0	0	0	0
HNRNPCP9	0	0	0	0	0	0	0	0
HNRNPD	462.959571	782.015677	376.397426	638.525657	493.293414	579.747701	710.624801	786.938659
HNRNPDL	147.599109	179.165347	131.677172	163.143565	220.124774	224.138647	284.016787	335.592965
HNRNPDLP1	0	0	0	0	0	0	0	0
HNRNPDLP2	0	0	0	0	0	0	0	0
HNRNPDLP3	0	0	0	0	0	0	0	0
HNRNPDLP4	0	0	0	0	0	0	0	0
HNRNPDP1	0	0	0	0	0	0	0	0
HNRNPDP2	0	0	0	0	0	0	0	0

HNRNPF	236.512373	445.723887	193.538313	504.61668	338.041108	890.284181	219.005559	923.623509
HNRNPFP1	0	0	0	0	0	0	0	0
HNRNPH1	42.13062	99.452756	167.735177	161.637825	389.904786	202.974568	321.286574	148.3327
HNRNPH1P1	0	0	0	0	0	0	0	0
HNRNPH1P2	0	0	0	0	0	0	0	0
HNRNPH1P3	0	0	0	0	0	0	0	0
HNRNPH2	94.003014	94.810591	68.445877	104.695195	93.664785	68.987166	141.079667	85.141492
HNRNPH3	249.288622	376.470011	220.22723	446.898717	574.796348	389.316825	161.780833	371.714355
HNRNPH3P1	0	0	0	0	0	0	0	0
HNRNPK	427.641843	425.175514	425.384241	363.268943	532.135274	313.92105	599.497707	614.633286
HNRNPKP1	0	0	0	0	0	0	0	0
HNRNPKP2	2.612694	0	0	0.063193	0	0	0	0
HNRNPKP3	0	0	0	0	0	0	0	0
HNRNPKP4	0	0.110021	0	0.126825	0	0	0	0.212996
HNRNPKP5	0	0	0	0	0	0	0	0
HNRNPL	254.750346	276.825656	180.764287	272.552256	307.681478	305.043841	402.622913	284.778273
HNRNPLL	31.014248	55.193705	28.955437	34.505445	14.033993	28.33895	13.711378	47.509747
HNRNPLP1	0	0	0	0.117696	0	0.361207	0	0
HNRNPLP2	0	0.418583	4.304864	1.12573	0	0.117346	0	0
HNRNPM	247.071981	177.34422	140.645129	237.415467	280.511074	357.169081	489.793709	211.887055
HNRNPMP1	0	0	0	0	0	0	0	0
HNRNPMP2	0	0	0	0	0	0	0	0
HNRNPR	123.933156	176.852402	75.479671	155.156171	133.640164	175.659171	189.301143	240.359241
HNRNPRP1	0	0	0	0	0	0	0	0
HNRNPRP2	0	0	0	0	0	0	0	0
HNRNPU	435.210937	505.450288	323.368433	408.905404	507.719591	566.697397	594.217939	592.615147
HNRNPUL1	73.547963	119.243184	74.138933	144.650432	202.427895	469.641093	211.364934	282.332941
HNRNPUL2	0	0	0	0	0	0	0	0
HNRNPUL2-I	22.329267	44.020482	0	49.206088	28.156514	133.525225	12.840261	92.741995
HNRNPUP1	0	0	0	0	0	0	0	0
HOATZ	0	1.697044	0	0	0	0	0	0
HOGA1	0	0.792379	0	2.217678	0	2.455569	0	1.291857
HOMER1	35.564005	108.173089	19.293831	94.345576	37.591035	48.95292	21.307899	95.429108

HOMER2	34.797392	67.026861	42.863168	104.162578	35.26888	96.191689	26.079463	60.533309
HOMER2P1	0	0	0	0	0	0	0	0
HOMER2P2	0	0	0	0	0	0	0	0
HOMER3	18.258272	58.263451	21.063634	54.081714	61.845786	116.047724	124.567231	103.991152
HOMEZ	8.294325	18.304708	0	16.916228	0	21.952359	0.955768	10.354032
HOOK1	31.188605	38.175711	0	49.543628	0	22.185769	0	12.147377
HOOK2	4.461926	25.536998	6.770778	14.797683	10.346065	40.467544	0	38.184559
HOOK3	21.550251	33.055053	32.626295	28.7292	33.777148	14.038445	103.823734	22.778691
HOPX	0	0	0	0.160455	0	1.782917	0	1.923839
HORMAD1	0	0	0	0	0	0	0	0
HORMAD2	0	0	0	0	0	0	0	0
HOXA1	0	12.78805	0	5.571018	0.906989	4.534214	21.657233	3.036431
HOXA10	13.370394	30.94022	14.165835	34.001183	41.50656	48.096722	23.440822	31.932604
HOXA11	0	0	8.649916	1.336002	0	0	16.027742	8.880207
HOXA13	7.17625	7.916986	9.377089	6.834588	15.682647	5.527314	10.964939	8.873729
HOXA2	0	1.767394	0	1.626973	0	1.757451	0	0.678447
HOXA3	0	13.640882	6.42298	14.627746	32.45443	17.85535	0	6.945093
HOXA4	3.947804	16.901951	3.481215	22.170757	0	11.000799	0	8.555495
HOXA5	4.24103	5.268394	5.667519	11.626324	5.115909	6.163513	0	14.676219
HOXA6	0	8.428009	8.765088	9.69763	0	17.423833	14.264047	10.488949
HOXA7	12.040791	24.12268	4.607772	24.862864	11.790858	27.212038	22.906689	20.965604
HOXA9	77.494666	135.892056	81.285676	161.640765	48.164643	189.536211	52.626756	157.113746
HOXB1	0	0	0	0	0	0	0	0
HOXB2	0	0	0	0	0	0	0	2.254391
HOXB3	6.265657	14.92019	10.617837	12.578061	6.344887	12.473642	20.807267	8.78243
HOXB4	6.933425	3.301184	0	3.830713	0	0.857582	0	0.699366
HOXB6	11.010827	6.915447	0	7.536054	0.556407	7.32364	0	4.866681
HOXB7	0	25.305201	0	32.625894	12.720656	35.272741	0	15.413421
HOXB8	0	3.625049	0	0.691048	0	0	0	0
HOXB9	2.62399	22.333239	9.393738	40.308606	0.692317	7.630128	0	4.965167
HOXC10	12.576576	8.127064	12.558875	10.736306	0	1.765722	0	0.354802
HOXC11	0	11.596734	1.517067	9.700629	0.447891	4.375113	0	2.909246
HOXC12	3.067679	3.975026	0.916681	3.341385	0	0	0	0

HOXC13	2.900466	8.008572	2.593844	6.66744	3.442802	5.038299	0	2.044979
HOXC4	2.067655	1.742572	0	1.052475	0	0.694515	0	0
HOXC6	6.443915	9.654264	5.740013	9.264385	0	2.664914	0	1.669682
HOXC8	5.645091	11.576132	8.836513	9.511476	0	3.853978	0	2.444417
HOXC9	15.842974	10.801715	6.83584	10.977257	0.643114	3.117635	0	4.329239
HOXD1	3.702931	0.39119	0	0.494269	0	0	0	0
HOXD10	39.78667	88.095105	32.758113	117.417424	0	141.899716	30.188052	104.195314
HOXD11	27.988164	43.328253	13.427839	56.785682	22.358806	60.541715	0	87.030823
HOXD12	0	0	0	0	0	0	0	0
HOXD13	7.059524	4.680494	2.52584	4.259737	0	1.261858	0	1.819922
HOXD3	0	0.298835	0	1.533513	0	1.577781	0	0.354673
HOXD4	0	0	0	0.318596	0	0.64374	0	0
HOXD8	7.922353	18.044263	5.91113	14.884892	10.491491	15.815243	0	12.361477
HOXD9	1.867924	5.922021	3.333028	5.704696	3.938633	9.234225	0	5.88477
HP	0	0	0	0	0	0	0	0
HP1BP3	81.2373	260.309196	45.398425	284.696325	154.650768	590.158496	223.986506	421.926021
HPCA	0	0	0	0.439787	0	0.503165	0	1.404819
HPCAL1	42.058041	72.219103	44.600635	66.699531	10.614112	33.207754	23.637178	31.321772
HPCAL4	2.223642	0.520397	2.662119	0.714831	0	0.107136	5.413766	0.830703
HPD	0	0	0	0	0	0	0	0
HPF1	33.583136	30.867586	21.645604	17.586841	39.796275	7.592813	0.733728	17.859736
HPGD	3.19215	4.714384	14.838125	2.639002	0	0	0	0
HPGDS	0	0	0	0	0	0	0	0
HPN	0	2.394112	0	2.311113	0	0	0	0
HPR	0	0	0	0	0	0	0	0
HPRT1	217.125688	192.763562	108.783102	91.334415	153.321097	72.937761	134.741035	168.416262
HPRT1P1	0	0	0	0	0	0	0	0
HPRT1P2	0	0	0	0	0	0	0	0
HPRT1P3	0	0	0	0	0	0	0	0
HPS1	16.615593	37.988018	22.266623	31.99428	31.157402	37.712072	36.681998	37.378544
HPS3	9.845972	45.124991	13.801515	35.815955	24.396958	28.049229	4.90474	50.664205
HPS4	20.483367	46.239227	0.906844	46.881144	27.343337	86.088391	6.662699	94.193426
HPS5	35.938548	14.180113	0	11.131331	21.976899	16.147068	17.182391	40.819382

HPSE	9.694503	5.07375	4.481917	3.892836	10.77011	5.662084	12.600813	5.385351
HPSE2	0	0	0	0	0	0	0	0
HPX	0	0	0	0	0	0	0	0
HR	3.659797	6.680626	4.38474	8.566651	1.614646	15.935557	3.120911	11.613141
HRAS	80.854511	109.760356	53.21029	96.879186	79.040695	220.068865	73.142466	198.489134
HRC	0	0	0	0	0	0	0	0
HRCT1	0	1.032083	0	0.502218	0	0.404082	0	0.51425
HRG	0	0	0	0	0	0	0	0
HRGP1	0	0	0	0	0	0	0	0
HRGP2	0	0	0	0	0	0	0	0
HRH1	6.265727	3.326836	0	9.521715	1.857637	5.499857	0	3.836735
HRH2	0	0	0	0	0	0.197671	0	0.10676
HRH3	0	0	0	0	0	0	0	0
HRH4	0	0	0	0	0	0	0	0
HRK	0	1.963984	1.010804	3.354483	9.161204	6.916676	0	3.823943
HROB	4.960946	4.286445	0	2.062063	2.904735	3.606445	0	3.839251
HS1BP3	20.740139	35.489664	27.189429	45.923839	0.378023	35.501936	2.085293	21.082088
HS2ST1	24.44702	74.780413	28.018192	57.13361	53.067202	23.705206	209.753525	43.779881
HS3ST1	6.325205	7.957072	4.876037	13.866067	3.343021	16.428653	4.957964	11.04776
HS3ST2	0	1.934182	0	2.433343	0.399203	24.230817	19.446222	33.876346
HS3ST3A1	0.787512	3.33076	4.598307	11.752551	0.20798	7.563623	0	8.035136
HS3ST3B1	1.823547	2.529815	3.199959	2.650334	6.619871	13.84441	10.515149	14.33446
HS3ST4	0	0	0	0	0	0	0	0
HS3ST5	0	0	0	0	0	0	0	0
HS6ST1	7.022689	21.795676	10.507016	23.665998	18.959156	104.94488	23.741587	72.673177
HS6ST1P1	0	0.1498	0	0	0	0	0	0.14838
HS6ST2	31.094432	10.21627	15.210417	12.757851	11.131882	4.82412	27.938414	4.244792
HSBP1	43.444787	83.338948	101.16857	86.885846	57.611663	76.295488	60.900864	59.798712
HSBP1L1	12.470679	30.398665	9.56495	28.14426	32.59632	34.015364	0	38.720509
HSBP1P1	0	0	0	0	0	0	0	0
HSBP1P2	0	0	0	0	0	0	0	0
HSCB	0	13.989225	7.144491	11.562621	0	16.539038	0	26.40581
HSD11B1	0	0	0	0	0	0	0	0

HSD11B1L	0	1.159238	5.428367	3.501396	0	4.483347	0	2.169851
HSD11B2	3.681307	9.647722	12.507941	10.190047	2.910995	10.555099	11.579621	8.14305
HSD17B1	2.914676	2.691052	0	2.839467	0	2.502875	0	0.697399
HSD17B10	0	0	0	3.453741	0	3.979362	2.14473	12.779544
HSD17B11	58.355918	18.484642	21.099276	18.130927	6.699504	15.309608	112.735917	12.769534
HSD17B12	49.863392	55.459661	86.216078	32.439868	192.465792	38.030745	156.42682	51.968987
HSD17B13	0	0.256611	0	0	1.903062	0	0	0
HSD17B14	0	0	0	1.38765	0	0	0	1.593763
HSD17B1P1	0	0	0	0	0	0	0	0
HSD17B2	10.317024	2.745613	15.684714	12.065047	4.645138	8.582824	0	6.038507
HSD17B3	0	0	0	0.307614	0	0.650268	0	0
HSD17B4	46.283944	91.490128	0	129.089456	75.658747	94.015054	6.656272	83.294164
HSD17B6	0	4.000711	13.350932	2.647921	0	0	0	0.576304
HSD17B7	15.254377	5.40028	9.46754	4.471816	0	1.047539	0	3.32326
HSD17B7P1	0	0	0	0	0	0	0	0
HSD17B7P2	0	0.201655	0	0	0	0.174367	0	0
HSD17B8	43.699508	21.402021	35.026666	26.158895	4.172023	16.822012	0	12.366191
HSD3B1	0	0	0	0	0	0	0	0
HSD3B2	0	0	0	0	0	0	0	0
HSD3B7	3.171919	2.000475	2.834421	3.661645	0.836459	0.707343	0	1.595773
HSD3BP1	0	0	0	0	0	0	0	0
HSD3BP2	0	0	0	0	0	0	0	0
HSD3BP3	0	0	0	0	0	0	0	0
HSD3BP4	0	0	0	0	0	0	0	0
HSD3BP5	0	0	0	0.079979	0	0	0	0
HSDL1	13.154083	22.716457	12.24189	24.686353	18.016774	31.984353	12.88232	25.335195
HSDL2	60.421437	42.218512	34.954406	26.80611	16.464473	9.72028	46.081858	27.297471
HSF1	56.605398	54.214314	61.601818	74.689392	56.094915	69.442144	179.240038	42.463158
HSF2	0	7.202028	0	3.237834	0	2.270595	0	2.942957
HSF2BP	0	3.570291	0	3.88043	0.484039	3.263637	0	6.589636
HSF4	0	2.510404	7.108823	1.669133	0	2.690789	0	0.791394
HSFX1	0	0.824235	0	1.161242	0	1.226415	0	1.072252
HSFX2	0	0	0	0	0	0	0	0

HSFY1	0	0	0	0	0	0	0	0
HSFY1P1	0	0	0	0	0	0	0	0
HSFY2	0	0	0	0	0	0	0	0
HSFY3P	0	0	0	0	0	0	0	0
HSFY4P	0	0	0	0	0	0	0	0
HSFY5P	0	0	0	0	0	0	0	0
HSFY6P	0	0	0	0	0	0	0	0
HSFY8P	0	0	0	0	0	0	0	0
HSH2D	0	0.599289	0	2.210974	0.937065	7.769806	0	6.378119
HSP90AA1	0	151.403698	0	70.765161	0	81.599686	0	93.978052
HSP90AA2P	0	0.475162	0	0.115604	0	0.156628	0	0
HSP90AA3P	0	0	0	0.038715	0.419748	0	0	0
HSP90AA4P	0	0	0	0	0	0	0	0
HSP90AA5P	0	0	0	0	0	0	0	0
HSP90AA6P	0	0	0	0	0	0	0	0
HSP90AB1	1026.10065	877.38088	816.81511	1021.88935	959.710345	947.407603	1128.53172	768.582157
HSP90AB2P	0	0	0	0	0	0	0	0
HSP90AB3P	0	0.137253	0	0.077546	0	0.07902	0	0
HSP90AB4P	0	0	0	0.054885	0.597054	0	0	0
HSP90AB5P	0	0	0	0	0	0	0	0
HSP90AB6P	0	0	0	0	0	0	0	0
HSP90AB7P	0	0	0	0	0	0	0	0
HSP90B1	686.421426	617.733353	900.070885	764.875034	805.772576	717.143776	650.649801	588.813781
HSP90B2P	0	0.060876	0	0.209115	0	0.142224	0	0.461686
HSP90B3P	0	0	0	0	0	0	0	0
HSPA12A	1.338289	7.438761	7.473094	4.881154	0.353517	0.703497	0	1.63099
HSPA12B	0	0	0	0	0	0	0	0
HSPA13	12.569543	14.590717	28.575686	11.94291	19.779526	3.266081	8.360065	5.485689
HSPA14	17.594389	16.022974	6.278661	4.912704	8.399732	4.509178	0	15.110525
HSPA1A	78.461408	107.608212	67.94496	104.29955	79.689395	125.966216	89.42879	100.30886
HSPA1B	116.445986	115.322023	88.842234	152.460893	160.881679	187.658759	93.167394	106.974073
HSPA1L	2.670681	4.480314	2.389905	3.633025	0	1.859534	0	1.32405
HSPA2	0.952167	0.434771	0	0.350568	0	0.161492	0	0.218793

HSPA4	210.61545	204.634147	113.580554	144.654916	97.568322	140.618233	426.119743	223.848216
HSPA4L	25.930559	28.693787	23.068723	14.243633	23.477179	12.131963	16.284404	27.482234
HSPA5	289.269255	466.038419	469.17155	729.326302	172.150067	204.852413	167.859472	214.654615
HSPA5P1	0	0	0	0.043149	0	0	0	0
HSPA6	0	1.408154	0	2.990052	0	0.576327	0	0.52634
HSPA7	3.615848	0.54752	0	0.101785	0	0	0	0
HSPA8	1456.39571	1519.44071	1246.97516	1235.35603	1100.09206	1041.7803	1302.92695	1455.24397
HSPA8P1	0	0.080707	0	0	0	0	0	0
HSPA8P11	0	0	0	0.044622	0	0	0	0
HSPA8P13	0	0	0	0	0	0	0	0
HSPA8P14	0	0	0	0.094306	0	0.095955	0	0
HSPA8P15	0	0	0	0.124542	0	0	0	0
HSPA8P16	0	0	0	0	0	0	0	0
HSPA8P17	0	0	0	0	0	0	0	0
HSPA8P18	0	0	0	0	0	0	0	0
HSPA8P19	0	0	0	0	0	0	0	0
HSPA8P20	0	0	0	0	0	0	0	0
HSPA8P3	0	0	0	0	0	0	0	0
HSPA8P4	0	0	1.619969	0	0	0	0	0
HSPA8P5	0	0	0	0.040615	0	0	0	0.06743
HSPA8P6	0	0	0	0	0	0	0	0
HSPA8P7	0	0.377922	0	0	0	0	0	0
HSPA8P8	0	0	0	0	0	0	0	0
HSPA8P9	0	0	0	0	0	0	0	0
HSPA9	320.163335	329.327023	235.912255	398.696686	313.68538	251.700774	609.440968	253.16796
HSPA9P1	0	0.144897	0	0	0	0	0	0
HSPB1	206.037331	174.814344	150.457829	229.187882	228.504475	288.256181	188.167237	189.509454
HSPB1P1	0	0	0	0.761724	8.11731	1.490393	0	0
HSPB1P2	0	0	0	0	0	0	0	0
HSPB2	0	1.361989	4.195063	1.825176	0	0	0	0
HSPB2-C11o	0	0	0	1.271834	0	1.873936	0	0
HSPB3	0	0	0	0	0	0	0	0
HSPB6	5.975371	0	0	0.298588	0	0.848321	0	0.200027

HSPB7	0	0	0	0	0	0	0	0
HSPB8	0	0	0	0	0	0	0	2.906804
HSPBAP1	3.374184	10.473114	6.321338	9.392775	7.292978	6.582105	39.26389	9.0453
HSPBAP1P1	0	0	0	0	0	0	0	0
HSPBP1	13.029902	18.623872	11.605029	16.235868	29.001708	40.085753	55.595483	39.639814
HSPD1	452.427268	579.074464	514.451914	428.411942	644.765661	353.179114	519.360121	638.38461
HSPD1P1	0	0	0	0	0	0	0	0.082972
HSPD1P10	0	0	0	0.051179	0	0	0	0
HSPD1P11	0	0	0	0.050314	0	0	0	0.251708
HSPD1P12	0	0	0	0	0	0	0	0
HSPD1P13	0	0	0	0	0	0	0	0
HSPD1P14	0	0	0	0	0	0	0	0
HSPD1P15	0	0	0	0	0	0	0	0
HSPD1P16	0	0	0	0	0	0	0	0
HSPD1P18	0	0	0	0	0	0	0	0
HSPD1P19	0	0	0	0	0	0	0	0
HSPD1P2	0	0.096696	0	0	0	0	0	0
HSPD1P21	0	0	0	0	0	0	0	0
HSPD1P3	0	0	0	0	0	0	0	0
HSPD1P4	0	0	0	0.100689	0	0	0	0
HSPD1P5	0	0	0	0	0	0	0	0
HSPD1P6	0	0	0	0	0	0.208692	0	0
HSPD1P7	0	0	0	0	0	0	0	0
HSPD1P8	0	0	0	0	0	0	0	0
HSPD1P9	0	0.639285	0	0	0	0	0	0
HSPE1	437.054255	573.083938	275.396936	421.860191	403.547244	301.218215	381.776104	535.367859
HSPE1-MOB4	25.884699	12.474381	0	2.842886	0	4.207178	0	5.572962
HSPE1P1	0	0	0	0	0	0	0	0
HSPE1P10	0	0	0	0	0	0	0	0
HSPE1P11	0	0	0	0	0	0	0	0
HSPE1P12	0	0	0	0	0	0	0	0
HSPE1P13	0	0	0	0	0	0	0	0
HSPE1P14	0	0	0	0	0	0	0	0

HSPE1P16	0	0	0	0	0	0	0	0
HSPE1P18	0	0	0	0	0	0	0	0
HSPE1P19	0	0	0	0	0	0	0	0
HSPE1P2	0	0	0	0	0	0	0	0
HSPE1P20	0	0	0	0	0	0	0	0
HSPE1P21	0	0	0	0	0	0	0	0
HSPE1P22	0	0	0	0	0	0	0	0
HSPE1P23	0	0	0	0	0	0	0	0
HSPE1P24	0	0	0	0	0	0	0	0
HSPE1P25	0	0	0	0	0	0	0	0
HSPE1P26	0	0	0	0	0	0	0	0
HSPE1P27	0	0	0	0	0	0	0	0
HSPE1P28	0	0	0	0	0	0	0	0
HSPE1P3	0	0	0	0	0	0	0	0
HSPE1P4	0	0	0	0	0	0	0	0
HSPE1P5	0	0	0	0	0	0	0	0
HSPE1P6	0	0	0	0	0	0	0	0
HSPE1P7	0	0	0	0	0	0	0	0
HSPE1P8	0	0	0	0	0	0	0	0
HSPE1P9	0	0	0	0	0	0	0	0
HSPG2	7.883306	8.682059	11.628643	13.531475	19.975364	12.483647	42.889063	8.078865
HSPH1	111.886945	120.484101	63.451903	63.373537	53.913329	44.661473	74.326144	118.173258
HTATIP2	56.049516	74.444016	56.183685	54.432431	50.047059	52.558526	54.548906	68.990801
HTATSF1	52.73317	72.332041	53.954926	60.105168	30.810996	72.93479	40.842803	79.763265
HTATSF1P1	0	0	0	0	0	0	0	0
HTATSF1P2	0	1.951616	0	1.65956	0	5.595917	0	7.592782
HTD2	0	36.14345	5.581049	20.545466	11.124749	27.556142	42.877844	55.501638
HTN1	0	0	0	0	0	0	0	0
HTN3	0	0	0	0	0	0	0	0
HTR1A	0	0	0	0	0	0	0	0
HTR1DP1	0	0	0	0	0	0	0	0
HTR1E	0	0	0	0	0	0	0	0
HTR2A	0	0	0	0	0	0	0	0

HTR2B	0	0	0	0	0	0	0	0
HTR2C	0	0	0	0	0	0	0	0
HTR3A	0	0	0	0	0	0	0	0
HTR3B	0	0	0	0	0	0	0	0
HTR3C2P	0	0	0	0	0	0	0	0
HTR3D	0	0	0	0	0	0	0	0
HTR3E	0	0	0	0	0	0	0	0
HTR4	0	0.562852	0	0	0	0	0	0
HTR5A	0	0	0	0	0	0	0	0
HTR5BP	0	0	0	0	0	0	0	0
HTR6	0	0.223833	0	0.042545	0	0.348283	0	0.07002
HTR7	4.432481	11.125809	2.582611	9.080504	5.849565	11.571617	8.34613	18.736031
HTR7P1	4.473585	8.918707	3.347244	9.728667	3.150813	14.945212	13.169819	19.903776
HTRA1	3.058938	8.850245	16.494056	6.616633	11.21044	12.147975	28.269494	12.187659
HTRA2	7.756514	0	0	0.869466	0	0	0	1.360186
HTRA3	1.713567	4.315722	2.727605	3.978141	0	2.92078	0	2.067221
HTRA4	0	0.216586	0	0.207096	0	0	0	0
HTT	0	6.975665	0	7.230696	32.40085	0	187.748822	11.860115
HUNK	0	0.072418	0	0.113179	1.111181	0.759905	0	0.623465
HUS1	26.776345	24.251732	30.858199	24.378614	12.925628	21.170172	69.844424	39.263429
HUWE1	113.676408	241.31525	213.13443	286.988457	166.710746	319.255906	162.632008	318.42329
HVCN1	2.27675	6.653295	2.708279	1.793778	2.998153	7.651116	0	4.785447
HYAL1	0	1.146975	0	0.082883	0	0.347159	0	0
HYAL2	9.686341	46.449086	6.623335	31.714472	12.717937	41.476994	6.29178	31.638386
HYAL3	8.703946	5.769673	3.582131	7.918214	7.469126	6.311446	0	8.729237
HYAL4	0	0.465034	0	2.634456	0	0.108828	0	1.945699
HYAL6P	0	0	0	0	0	0	0	0
HYCC1	24.188425	41.172801	19.721978	37.837026	10.812351	10.085609	17.256739	45.685979
HYCC2	1.372079	4.060778	3.410223	1.341968	7.143967	4.340932	43.195752	13.085824
HYDIN	22.272048	6.068383	12.415158	6.340867	0	0.871929	0	0.981742
HYDIN2	1.250938	0	0	0	0	0	0	0
HYDINP1	0	0	0	0	0	0	0	0
HYI	17.859972	14.516153	26.164015	8.911904	11.656704	24.000767	0	6.049564

HYKK	0	6.914087	8.34088	3.594394	0	2.964951	8.034081	4.776474
HYLS1	5.839078	11.091195	5.207169	3.790375	51.072835	7.869991	14.590709	14.347893
HYOU1	409.644679	561.077829	653.158079	1115.40556	84.866127	126.113652	94.431192	74.617199
HYPK	0	0	0	0	0	0	0	0
IAH1	42.089234	31.406934	34.036532	27.844813	58.765382	10.527375	65.247286	22.264487
IAPP	0	0	0	0	0	0	0	0
IARS1	27.805635	45.244259	50.626647	34.411436	6.4693	18.044426	0	47.982402
IARS2	107.347227	110.349507	107.283258	75.072684	65.900092	61.719335	64.364931	71.855504
IARS2P1	0	0	0	0	0	0	0	0
IBA57	9.362169	0.923305	4.068882	1.160395	3.922865	1.407714	42.627559	1.602778
IBSP	0	0	0	0	0	0	0	0
IBTK	45.299253	41.198541	27.61425	31.161502	32.914774	10.876763	12.11867	32.505827
ICA1	19.98783	68.206913	55.928019	61.670177	23.376417	5.960252	0	7.709059
ICA1L	9.481068	6.99905	1.702727	0.930843	0.166886	0.918607	0	6.544776
ICAM1	11.324055	15.136777	50.167812	28.445162	16.438107	13.318825	10.90872	10.878785
ICAM2	19.877626	38.087333	40.247544	49.632586	7.002309	24.672691	0	16.629319
ICAM3	2.031671	7.401042	1.222188	10.711838	24.285705	8.229743	0	6.159848
ICAM4	0	0	0	0	0	0	0	0
ICAM5	1.304934	1.916009	2.335888	3.416	1.866372	2.807852	0	1.050695
ICE1	49.779775	61.298148	30.747344	38.024449	20.539132	21.507727	87.680428	52.973958
ICE2	38.050193	103.930929	69.540156	77.134848	57.229389	53.789521	13.025	121.116587
ICE2P1	0	0	0	0	0	0	0	0
ICE2P2	0	0	0	0	0	0	0	0
ICMT	49.459876	100.123683	49.303001	71.444982	80.279355	166.680085	82.198292	163.771112
ICOS	0	0	0	0	0	0	0	0
ID1	112.835857	92.435771	246.756746	222.37342	72.723117	119.469811	72.603569	135.570605
ID2	8.47749	18.356142	15.045361	16.928358	0	9.738045	82.215713	5.052847
ID2B	0	0	0	0	0	0	0	0
ID3	44.738701	20.427501	39.855505	26.032511	70.164441	45.005706	88.430983	33.934282
ID4	1.708646	1.609331	1.533192	1.501449	0.451193	1.536559	0	1.029426
IDE	15.086266	29.308551	2.773404	23.451318	16.680004	13.584864	22.841604	50.307188
IDH1	0	19.069719	0	21.600699	0	40.878776	80.837189	28.244475
IDH1P1	0	0	0	0	0	0	0	0

IDH2	105.483706	53.346308	91.019904	58.981614	52.297723	41.081072	47.292585	27.876344
IDH3A	83.073937	64.620831	42.10758	68.942477	112.333636	61.83148	77.830105	71.045546
IDH3B	58.678054	52.342786	45.402532	54.339534	82.167013	48.151474	10.638622	35.442262
IDH3G	52.012065	30.864498	43.746575	32.911325	48.264113	29.974624	90.349577	22.944357
IDI1	0	13.86164	0	2.253459	0	1.940434	0	6.87867
IDI1P1	0	0	0	0	0	0	0	0
IDI1P2	0	0	0	0	0	0	0	0
IDI1P3	0	0	0	0	0	0	0	0
IDI2	0	0	0	0	0	0	0	0
IDNK	8.378322	11.504913	0	4.199315	29.098045	2.824983	0	4.024965
IDO1	1.892599	1.358867	3.376587	1.847224	0	1.124478	0	1.222792
IDO2	0	0	0	0	0	0	0	0
IDS	73.333275	31.288679	26.832321	30.011937	9.910497	17.246951	58.4571	19.847219
IDSP1	0	0	0	0	0	0	0	0
IDUA	0	2.156984	0	4.413568	0.854026	6.471182	0	5.913698
IER2	35.436762	73.760151	71.621902	76.696292	127.30277	124.294902	43.919216	91.421139
IER3	252.653782	187.339089	276.722136	286.21447	347.568486	223.26374	202.967726	198.443409
IER3IP1	26.150429	16.944636	12.942801	23.094336	9.761966	24.234255	8.773689	24.722185
IFFO1	0	2.068372	0	1.383586	0	0.131237	0	0.053155
IFFO2	11.56598	19.777455	17.487099	18.183234	33.338498	60.102961	69.04552	48.243572
IFI16	83.471945	59.332091	73.895761	54.458196	106.365676	71.822775	99.014615	74.542385
IFI27	0	0	0	0.263644	8.584038	0.323616	0	2.847955
IFI27L1	24.373119	39.268043	17.701759	34.981429	0	19.843879	0	19.38779
IFI27L2	151.960857	107.945733	104.034178	132.545651	84.533368	85.814356	0	44.906343
IFI30	0	20.636333	38.013988	21.711929	59.673394	42.69855	73.099257	34.288967
IFI35	15.032097	14.518929	10.66042	14.423743	29.257341	37.517373	10.326647	23.081647
IFI44	56.795387	48.225601	41.232909	33.083163	117.617061	33.731656	86.400483	44.619175
IFI44L	1.903192	2.028112	7.944841	4.484162	9.2752	7.872934	0	17.107696
IFI6	19.56423	27.168184	57.149269	45.059018	334.767895	228.725587	204.138289	122.48207
IFIH1	6.596763	13.723338	8.647071	9.04426	15.365685	37.183056	15.069249	41.136798
IFIT1	7.653309	4.340684	2.748359	1.82669	24.862034	25.12297	10.805696	32.90401
IFIT1B	0	0	0	0	0	0.174338	0	0
IFIT1P1	0	0.208778	0	0	0	0.122213	0	0

IFIT2	6.87467	3.885544	4.820621	1.493632	1.422791	27.835276	39.637537	23.504703
IFIT3	8.571255	12.564947	2.555363	7.751767	114.214297	106.694135	104.286606	110.282218
IFIT5	10.655641	14.42936	7.356416	9.145252	25.325655	20.442291	10.970876	34.924173
IFIT6P	0	0	0	0	0	0	0	0
IFITM1	178.495048	209.122087	159.373476	231.887176	609.107554	1002.34468	391.359909	573.083173
IFITM10	0	0.100871	0	0.059984	0	0.314996	0	0.344302
IFITM2	22.8252	0.458063	0	3.774292	0	3.971434	0	0
IFITM3	551.418442	467.945117	512.000769	596.31393	1295.67632	1297.89942	466.89932	840.943667
IFITM3P1	0	0	0	0.348962	0	0	0	0.659069
IFITM3P3	0	0	0	0	0	0	0	0
IFITM3P4	0	0	0	0	0	0	0	0
IFITM3P5	0	0	0	0	0	0	0	0
IFITM3P6	0	3.10193	0	4.498793	0	1.238897	0	1.204609
IFITM3P7	0	0	0	0	0	0	0	0
IFITM3P8	0	0	0	0	0	0	0	0
IFITM3P9	0	0	0	0	0	1.412245	0	0
IFITM4P	0	2.927881	0	0.601198	0	0.583413	0	0
IFITM5	0	0	0	0	0	0	0	0
IFITM8P	0	0	0	0	0	0	0	0
IFITM9P	0	0	0	0	0	0	0	0
IFNA10	0	0	0	0	0	0	0	0
IFNA11P	0	0	0	0	0	0	0	0
IFNA12P	0	0	0	0	0	0	0	0
IFNA13	0	0	0	0	0	0	0	0
IFNA16	0	0	0	0	0	0	0	0
IFNA17	0	0	0	0	0	0	0	0
IFNA20P	0	0	0	0	0	0	0	0
IFNA21	0	0	0	0	0	0	0	0
IFNA22P	0	0	0	0	0	0	0	0
IFNA6	0	0	0	0	0	0	0	0
IFNA7	0	0	0	0	0	0	0	0
IFNAR1	6.082377	8.226497	11.913053	7.568869	11.770078	7.030053	15.883867	11.102969
IFNAR2	30.418529	56.962535	23.756214	33.617338	52.58923	44.636993	41.125176	57.852686

IFNB1	0	0	0	0	0	0	0	0
IFNE	2.44071	0	0	0	9.639273	0	0	0
IFNG	0	0	0	0	0	0	0	0
IFNGR1	46.668839	18.014174	0	11.195422	54.854363	4.582122	18.127727	14.371141
IFNGR2	104.630064	93.908697	92.523582	102.347781	115.453878	53.591709	97.795078	75.926278
IFNL1	0	0	0	0	0	0	0	0
IFNL3P1	0	0	0	0.229944	0	0	0	0
IFNL4	0	0	0	0	0	0	0	0
IFNL4P1	0	0	0	0	0	0	0	0
IFNLR1	8.63072	11.219127	11.443238	9.455546	23.214758	18.541833	12.099219	12.643522
IFNNP1	0	0	0	0	0	0	0	0
IFNWP15	0	0	0	0	0	0	0	0
IFNWP18	0	0	0	0	0	0	0	0
IFNWP19	0	0	0	0	0	0	0	0
IFNWP2	0	0	0	0	0	0	0	0
IFNWP4	0	0	0	0	0	0	0	0
IFNWP5	0	0	0	0	0	0	0	0
IFNWP9	0	0	0	0	0	0	0	0
IFRD1	24.812931	34.100271	41.926735	41.178756	15.263879	12.068227	14.864509	18.920363
IFRD2	0	15.600484	22.713722	24.586883	16.324942	22.440737	39.381907	11.894898
IFT122	8.208498	6.668741	12.744181	10.852867	0	8.103464	3.849341	3.929532
IFT122P1	0	0	0	0	0	0	0	0
IFT122P2	0	0	0	0	0	0	0	0
IFT140	7.929906	10.640514	14.024152	22.222083	4.594471	15.217751	17.555416	9.527537
IFT172	3.024081	6.918732	4.920758	7.908858	1.278277	6.977214	0	7.11916
IFT20	44.523454	16.948082	40.807215	26.182804	16.416034	12.301916	0	18.262086
IFT22	31.617525	61.538882	10.776804	44.634827	33.447254	31.460095	38.756112	48.403168
IFT25	0	4.574515	0	1.403685	0	0.357517	11.669816	0.734909
IFT27	14.175525	28.576713	8.999577	46.906808	3.575751	25.26888	0	27.058039
IFT43	0	8.075016	0	9.41289	24.984896	8.426832	0	8.805619
IFT46	60.591231	75.600509	44.088886	155.098557	19.398241	34.585671	58.249809	12.983608
IFT52	55.672127	60.168311	29.865844	41.820804	45.26529	16.640391	100.047356	25.004896
IFT56	11.488332	27.81883	9.867347	18.920771	7.888822	7.319986	16.724881	22.801369

IFT57	134.727283	319.512673	79.400504	188.719789	105.958996	139.759045	171.09741	225.174674
IFT57P1	0	0	0	0	0	0	0	0
IFT70A	2.70213	1.274204	1.528438	0.790198	5.079034	0.846513	0.32495	2.735673
IFT74	0	1.607915	0	12.771237	74.323626	23.967141	13.413783	21.954106
IFT80	49.774039	55.300207	40.028107	63.649899	11.496272	19.901574	110.174371	30.269695
IFT81	8.575534	12.988275	5.584963	10.58247	16.41287	6.002555	11.582287	16.88427
IFT88	0	2.210919	0	2.188423	0	0.599323	0	4.666041
IFTAP	0	36.852925	0	31.927351	0	24.546695	0	39.371823
IGBP1	77.830715	66.748563	116.201834	82.750928	33.805431	43.890381	28.968999	47.299154
IGBP1P1	0	0	0	0	0	0	0	0
IGBP1P3	0	0	0	0	0	0	0	0
IGBP1P4	0	0	0	0	0	0	0	0
IGBP1P5	0	0	0	0	0	0	0	0
IGDCC3	0	0.773017	0	0.047794	0	0	0	0
IGDCC4	0	0.376158	0	0.344032	0	0.922629	0	0
IGF1	0	0	0	0	0	0	0	0
IGF1R	48.507381	115.318759	48.90603	108.791662	138.8809	256.029133	202.321004	213.91994
IGF2	17.036146	4.43352	14.529576	4.234179	2.108409	0.43569	0	0.278454
IGF2BP1	3.65977	18.444433	13.261257	8.251871	0	0.396899	0	0.029326
IGF2BP2	7.441031	105.716129	14.960707	134.029865	94.628711	182.361604	166.958958	83.248684
IGF2BP3	55.501885	108.391076	65.320982	100.962653	167.013619	55.454875	56.334297	61.997974
IGF2R	0	7.530164	15.131983	10.784676	24.631608	9.459967	25.840013	3.794989
IGFALS	0	0	0	0	0	0	0	0
IGFBP1	2.369162	1.790007	2.107736	3.095639	0	0.390665	0	1.419784
IGFBP2	4.758155	0.581224	0	1.02214	13.721071	10.216255	0	6.294019
IGFBP3	50.326866	50.870499	225.807102	190.610467	276.931937	167.428881	0	87.75706
IGFBP4	70.167759	94.054577	85.008599	115.19756	63.738376	138.518445	110.720347	117.937887
IGFBP5	0	0.111915	0	0.05092	0	0.182717	0	0.125226
IGFBP6	100.710672	183.960684	145.775727	245.240907	63.585534	83.055384	37.514644	35.593677
IGFBP7	63.243204	48.859776	85.618092	46.977187	30.194414	40.121799	81.130931	19.10274
IGFBPL1	3.727458	1.195642	0	0.886703	0	0	0	0.187218
IGFL1	0	0	0	0.125962	22.179092	8.86708	0	7.178954
IGFL1P1	0	0	0	0	0	0	0	0

IGFL1P2	0	0	0	0	0	0	0	0
IGFL2	0	0	0	0	0	0	0	0
IGFL3	0	0	0	0	0	0	0	0
IGFL4	0	0	0	0	0	0	0	0
IGFLR1	0	7.847687	5.034385	7.179988	57.865386	16.047935	0	16.512831
IGFN1	0	0	0	0	0	0	0	0
IGHA1	0	0	0	0	0	0	0	0
IGHA2	0	0	0	0	0	0	0	0
IGHD	0	0	0	0	0	0	0	0
IGHD1-1	0	0	0	0	0	0	0	0
IGHD1-14	0	0	0	0	0	0	0	0
IGHD1-20	0	0	0	0	0	0	0	0
IGHD1-26	0	0	0	0	0	0	0	0
IGHD1-7	0	0	0	0	0	0	0	0
IGHD1OR15-	0	0	0	0	0	0	0	0
IGHD2-15	0	0	0	0	0	0	0	0
IGHD2-2	0	0	0	0	0	0	0	0
IGHD2-21	0	0	0	0	0	0	0	0
IGHD2-8	0	0	0	0	0	0	0	0
IGHD2OR15-	0	0	0	0	0	0	0	0
IGHD3-10	0	0	0	0	0	0	0	0
IGHD3-16	0	0	0	0	0	0	0	0
IGHD3-22	0	0	0	0	0	0	0	0
IGHD3-3	0	0	0	0	0	0	0	0
IGHD3OR15-	0	0	0	0	0	0	0	0
IGHD4-11	0	0	0	0	0	0	0	0
IGHD4-17	0	0	0	0	0	0	0	0
IGHD4-23	0	0	0	0	0	0	0	0
IGHD4OR15-	0	0	0	0	0	0	0	0
IGHD5-12	0	0	0	0	0	0	0	0
IGHD5-18	0	0	0	0	0	0	0	0
IGHD5-24	0	0	0	0	0	0	0	0
IGHD5OR15-	0	0	0	0	0	0	0	0

IGHD6-13	0	0	0	0	0	0	0	0
IGHD6-19	0	0	0	0	0	0	0	0
IGHD6-25	0	0	0	0	0	0	0	0
IGHD6-6	0	0	0	0	0	0	0	0
IGHD7-27	0	0	0	0	0	0	0	0
IGHE	0	0	0	0	0	0	0	0
IGHEP1	0	0	0	0	0	0	0	0
IGHEP2	0	0	0	0	0	0.43051	0	0
IGHG1	0	0	0	0	0	0	0	0
IGHG2	0	0	0	0	0	0	0	0
IGHG3	0	0	0	0	0	0	0	0
IGHG4	0	0	0	0	0	0	0	0
IGHGP	0	0	0	0	0	0	0	0
IGHJ1	0	0	0	0	0	0	0	0
IGHJ1P	0	0	0	0	0	0	0	0
IGHJ2	0	0	0	0	0	0	0	0
IGHJ2P	0	0	0	0	0	0	0	0
IGHJ3	0	0	0	0	0	0	0	0
IGHJ3P	0	0	0	0	0	0	0	0
IGHJ4	0	0	0	0	0	0	0	0
IGHJ5	0	0	0	0	0	0	0	0
IGHJ6	0	0	0	0	0	0	0	0
IGHM	0	0	0	0	0	0	0	0
IGHMBP2	0	2.059241	0	7.694582	17.290134	3.405212	1.437559	0
IGHV1-12	0	0	0	0	0	0	0	0
IGHV1-14	0	0	0	0	0	0	0	0
IGHV1-17	0	0	0	0	0	0	0	0
IGHV1-18	0	0	0	0	0	0	0	0
IGHV1-2	0	0	0	0	0	0	0	0
IGHV1-24	0	0	0	0	0	0	0	0
IGHV1-3	0	0	0	0	0	0	0	0
IGHV1-45	0	0	0	0	0	0	0	0
IGHV1-46	0	0	0	0	0	0	0	0

IGHV1-58	0	0	0	0	0	0	0	0
IGHV1-67	0	0	0	0	0	0	0	0
IGHV1-68	0	0	0	0	0	0	0	0
IGHV1-69	0	0	0	0	0	0	0	0
IGHV1-69-2	0	0	0	0	0	0	0	0
IGHV1-69D	0	0	0	0	0	0	0	0
IGHV1OR15-	0	0	0	0	0	0	0	0
IGHV1OR15-	0	0	0	0	0	0	0	0
IGHV1OR15-	0	0	0	0	0	0	0	0
IGHV1OR15-	0	0	0	0	0	0	0	0
IGHV1OR16-	0	0	0	0	0	0	0	0
IGHV1OR16-	0	0	0	0	0	0	0	0
IGHV1OR16-	0	0	0	0	0	0	0	0
IGHV1OR16-	0	0	0	0	0	0	0	0
IGHV1OR21-	0	0	0	0	0	0	0	0
IGHV2-26	0	0	0	0	0	0	0	0
IGHV2-5	0	0	0	0	0	0	0	0
IGHV2-70	0	0	0	0	0	0	0	0
IGHV2-70D	0	0	0	0	0	0	0	0
IGHV2OR16-	0	0	0	0	0	0	0	0
IGHV3-11	0	0	0	0	0	0	0	0
IGHV3-13	0	0	0	0	0	0	0	0
IGHV3-15	0	0	0	0	0	0	0	0
IGHV3-16	0	0	0	0	0	0	0	0
IGHV3-19	0	0	0	0	0	0	0	0
IGHV3-20	0	0	0	0	0	0	0	0
IGHV3-21	0	0	0	0	0	0	0	0
IGHV3-22	0	0	0	0	0	0	0	0
IGHV3-23	0	0	0	0	0	0	0	0
IGHV3-25	0	0	0	0	0	0	0	0
IGHV3-29	0	0	0	0	0	0	0	0
IGHV3-30	0	0	0	0	0	0	0	0
IGHV3-30-2	0	0	0	0	0	0	0	0

IGHV3-32	0	0	0	0	0	0	0	0
IGHV3-33	0	0	0	0	0	0	0	0
IGHV3-33-2	0	0	0	0	0	0	0	0
IGHV3-35	0	0	0	0	0	0	0	0
IGHV3-36	0	0	0	0	0	0	0	0
IGHV3-37	0	0	0	0	0	0	0	0
IGHV3-38	0	0	0	0	0	0	0	0
IGHV3-41	0	0	0	0	0	0	0	0
IGHV3-42	0	0	0	0	0	0	0	0
IGHV3-43	0	0	0	0	0	0	0	0
IGHV3-47	0	0	0	0	0	0	0	0
IGHV3-48	0	0	0	0	0	0	0	0
IGHV3-49	0	0	0	0	0	0	0	0
IGHV3-50	0	0	0	0	0	0	0	0
IGHV3-52	0	0	0	0	0	0	0	0
IGHV3-53	0	0	0	0	0	0	0	0
IGHV3-54	0	0	0	0	0	0	0	0
IGHV3-57	0	0	0	0	0	0	0	0
IGHV3-6	0	0	0	0	0	0	0	0
IGHV3-60	0	0	0	0	0	0	0	0
IGHV3-62	0	0	0	0	0	0	0	0
IGHV3-63	0	0	0	0	0	0	0	0
IGHV3-64	0	0	0	0	0	0	0	0
IGHV3-65	0	0	0	0	0	0	0	0
IGHV3-66	0	0	0	0	0	0	0	0
IGHV3-69-1	0	0	0	0	0	0	0	0
IGHV3-7	0	0	0	0	0	0	0	0
IGHV3-71	0	0	0	0	0	0	0	0
IGHV3-72	0	0	0	0	0	0	0	0
IGHV3-73	0	0	0	0	0	0	0	0
IGHV3-74	0	0	0	0.182374	0	0	0	0
IGHV3-75	0	0	0	0	0	0	0	0
IGHV3-76	0	0	0	0	0	0	0	0

IGHV3-79	0	0	0	0	0	0	0	0
IGHV3OR15-	0	0	0	0	0	0	0	0
IGHV3OR16-	0	0	0	0	0	0	0	0
IGHV3OR16-	0	0	0	0	0	0	0	0
IGHV3OR16-	0	0	0	0	0	0	0	0
IGHV3OR16-	0	0	0	0	0	0	0	0
IGHV3OR16-	0	0	0	0	0	0	0	0
IGHV3OR16-	0	0	0	0	0	0	0	0
IGHV3OR16-	0	0	0	0	0	0	0	0
IGHV3OR16-	0	0	0	0	0	0	0	0
IGHV3OR16-	0	0	0	0	0	0	0	0
IGHV3OR16-	0	0	0	0	0	0	0	0
IGHV3OR16-	0	0	0	0	0	0	0	0
IGHV4-28	0	0	0	0	0	0	0	0
IGHV4-31	0	0	0	0	0	0	0	0
IGHV4-34	0	0	0	0	0	0	0	0
IGHV4-39	0	0	0	0	0	0	0	0
IGHV4-4	0	0	0	0	0	0	0	0
IGHV4-55	0	0	0	0	0	0	0	0
IGHV4-59	0	0	0	0	0	0	0	0
IGHV4-61	0	0	0	0	0	0	0	0
IGHV4-80	0	0	0	0	0	0	0	0
IGHV4OR15-	0	0	0	0	0	0	0	0
IGHV5-10-1	0	0	0	0	0	0	0	0
IGHV5-51	0	0	0	0	0	0	0	0
IGHV5-78	0	0	0	0	0	0	0	0
IGHV6-1	0	0	0	0	0	0	0	0
IGHV7-27	0	0	0	0	0	0	0	0
IGHV7-34-1	0	0	0	0	0	0	0	0
IGHV7-40	0	0	0	0	0	0	0	0
IGHV7-56	0	0	0	0	0	0	0	0
IGHV7-81	0	0	0	0	0	0	0	0
IGHVII-1-1	0	0	0	0	0	0	0	0

IGHVII-15-1	0	0	0	0	0	0	0	0
IGHVII-22-1	0	0	0	0	0	0	0	0
IGHVII-26-2	0	0	0	0	0	0	0	0
IGHVII-28-1	0	0	0	0	0	0	0	0
IGHVII-30-1	0	0	0	0	0	0	0	0
IGHVII-30-21	0	0	0	0	0	0	0	0
IGHVII-33-1	0	0	0	0	0	0	0	0
IGHVII-40-1	0	0	0	0	0	0	0	0
IGHVII-43-1	0	0	0	0	0	0	0	0
IGHVII-44-2	0	0	0	0	0	0	0	0
IGHVII-46-1	0	0	0	0	0	0	0	0
IGHVII-49-1	0	0	0	0	0	0	0	0
IGHVII-51-2	0	0	0	0	0	0	0	0
IGHVII-53-1	0	0	0	0	0	0	0	0
IGHVII-60-1	0	0	0	0	0	0	0	0
IGHVII-62-1	0	0	0	0	0	0	0	0
IGHVII-65-1	0	0	0	0	0	0	0	0
IGHVII-67-1	0	0	0	0	0	0	0	0
IGHVII-74-1	0	0	0	0	0	0	0	0
IGHVII-78-1	0	0	0	0	0	0	0	0
IGHVIII-11-1	0	0	0	0	0	0	0	0
IGHVIII-13-1	0	0	0	0	0	0	0	0
IGHVIII-16-1	0	0	0	0	0	0	0	0
IGHVIII-2-1	0	0	0	0	0	0	0	0
IGHVIII-22-2	0	0	0	0	0	0	0	0
IGHVIII-25-1	0	0	0	0	0	0	0	0
IGHVIII-26-1	0	0	0	0	0	0	0	0
IGHVIII-38-1	0	0	0	0	0	0	0	0
IGHVIII-44	0	0	0	0	0	0	0	0
IGHVIII-47-1	0	0	0	0	0	0	0	0
IGHVIII-5-1	0	0	0	0	0	0	0	0
IGHVIII-5-2	0	0	0	0	0	0	0	0
IGHVIII-67-2	0	0	0	0	0	0	0	0

IGHVIII-67-3	0	0	0	0	0	0	0	0
IGHVIII-67-4	0	0	0	0	0	0	0	0
IGHVIII-76-1	0	0	0	0	0	0	0	0
IGHVIII-82	0	0	0	0	0	0	0	0
IGHVIV-44-1	0	0	0	0	0	0	0	0
IGKC	0	0	0	0	0	0	0	0
IGKJ1	0	0	0	0	0	0	0	0
IGKJ2	0	0	0	0	0	0	0	0
IGKJ3	0	0	0	0	0	0	0	0
IGKJ4	0	0	0	0	0	0	0	0
IGKJ5	0	0	0	0	0	0	0	0
IGKV1-12	0	0	0	0	0	0	0	0
IGKV1-13	0	0	0	0	0	0	0	0
IGKV1-16	0	0	0	0	0	0	0	0
IGKV1-17	0	0	0	0	0	0	0	0
IGKV1-22	0	0	0	0	0	0	0	0
IGKV1-27	0	0	0	0	0	0	0	0
IGKV1-32	0	0	0	0	0	0	0	0
IGKV1-33	0	0	0	0	0	0	0	0
IGKV1-35	0	0	0	0	0	0	0	0
IGKV1-37	0	0	0	0	0	0	0	0
IGKV1-39	0	0	0	0	0	0	0	0
IGKV1-5	0	0	0	0	0	0	0	0
IGKV1-6	0	0	0	0	0	0	0	0
IGKV1-8	0	0	0	0	0	0	0	0
IGKV1-9	0	0	0	0	0	0	0	0
IGKV1D-12	0	0	0	0	0	0	0	0
IGKV1D-13	0	0	0	0	0	0	0	0
IGKV1D-16	0	0	0	0	0	0	0	0
IGKV1D-17	0	0	0	0	0	0	0	0
IGKV1D-22	0	0	0	0	0	0	0	0
IGKV1D-27	0	0	0	0	0	0	0	0
IGKV1D-32	0	0	0	0	0	0	0	0

IGKV1D-33	0	0	0	0	0	0	0	0
IGKV1D-35	0	0	0	0	0	0	0	0
IGKV1D-37	0	0	0	0	0	0	0	0
IGKV1D-39	0	0	0	0	0	0	0	0
IGKV1D-42	0	0	0	0	0	0	0	0
IGKV1D-43	0	0	0	0	0	0	0	0
IGKV1D-8	0	0	0	0	0	0	0	0
IGKV1OR-2	0	0	0	0	0	0	0	0
IGKV1OR-3	0	0	0	0	0	0	0	0
IGKV1OR1-1	0	0	0	0	0	0	0	0
IGKV1OR10-[illegible]	0	0	0	0	0	0	0	2.662647
IGKV1OR2-1	0	0	0	0	0	0	0	0
IGKV1OR2-1[illegible]	0	0	0	0.40394	0	0.769466	0	1.548368
IGKV1OR2-1[illegible]	0	0	0	0	0	0	0	0
IGKV1OR2-2	0	0	0	0	0	0	0	0
IGKV1OR2-3	0	0	0	0	0	0	0	0
IGKV1OR2-6	0	0	0	0	0	0	0	0
IGKV1OR2-9	0	0	0	0	0	0	0	0
IGKV1OR22-[illegible]	0	0	0	0	0	0	0	0
IGKV1OR22-[illegible]	0	0	0	0	0	0	0	0
IGKV1OR9-1	0	0	0	0	0	0	0	0
IGKV1OR9-2	0	0	0	0	0	0	0	0
IGKV2-10	0	0	0	0	0	0	0	0
IGKV2-14	0	0	0	0	0	0	0	0
IGKV2-18	0	0	0	0	0	0	0	0
IGKV2-19	0	0	0	0	0	0	0	0
IGKV2-23	0	0	0	0	0	0	0	0
IGKV2-24	0	0	0	0	0	0	0	0
IGKV2-26	0	0	0	0	0	0	0	0
IGKV2-28	0	0	0	0	0	0	0	0
IGKV2-29	0	0	0	0	0	0	0	0
IGKV2-30	0	0	0	0	0	0	0	0
IGKV2-36	0	0	0	0	0	0	0	0

IGKV2-38	0	0	0	0	0	0	0	0
IGKV2-4	0	0	0	0	0	0	0	0
IGKV2-40	0	0	0	0	0	0	0	0
IGKV2D-10	0	0	0	0	0	0	0	0
IGKV2D-14	0	0	0	0	0	0	0	0
IGKV2D-18	0	0	0	0	0	0	0	0
IGKV2D-19	0	0	0	0	0	0	0	0
IGKV2D-23	0	0	0	0	0	0	0	0
IGKV2D-24	0	0	0	0	0	0	0	0
IGKV2D-26	0	0	0	0	0	0	0	0
IGKV2D-28	0	0	0	0	0	0	0	0
IGKV2D-29	0	0	0	0	0	0	0	0
IGKV2D-30	0	0	0	0	0	0	0	0
IGKV2D-36	0	0	0	0	0	0	0	0
IGKV2D-38	0	0	0	0	0	0	0	0
IGKV2OR2-1	0	0	0	0	0	0	0	0
IGKV2OR2-1	0	0	0	0	0	0	0	0
IGKV2OR2-2	0	0	0	0	0	0	0	0
IGKV2OR2-7	0	0	0	0	0	0	0	0
IGKV2OR2-8	0	0	0	0	0	0	0	0
IGKV2OR22-	0	0	0	0	0	0	0	0
IGKV2OR22-	0	0	0	0	0	0	0	0
IGKV3-11	0	0	0	0	0	0	0	0
IGKV3-15	0	0	0	0	0	0	0	0
IGKV3-20	0	0	0	0	0	0	0	0
IGKV3-25	0	0	0	0	0	0	0	0
IGKV3-31	0	0	0	0	0	0	0	0
IGKV3-34	0	0	0	0	0	0	0	0
IGKV3-7	0	0	0	0	0	0	0	0
IGKV3D-11	0	0	0	0	0	0	0	0
IGKV3D-15	0	0	0	0	0	0	0	0
IGKV3D-20	0	0	0	0	0	0	0	0
IGKV3D-25	0	0	0	0	0	0	0	0

IGKV3D-31	0	0	0	0	0	0	0	0
IGKV3D-34	0	0	0	0	0	0	0	0
IGKV3D-7	0	0	0	0	0	0	0	0
IGKV3OR2-2	0	0	0	0	0	0	0	0
IGKV3OR2-5	0	0	0	0	0	0	0	0
IGKV3OR22-	0	0	0	0	0	0	0	0
IGKV4-1	0	0	0	0	0	0	0	0
IGKV5-2	0	0	0	0	0	0	0	0
IGKV6-21	0	0	0	0	0	0	0	0
IGKV6D-21	0	0	0	0	0	0	0	0
IGKV6D-41	0	0	0	0	0	0	0	0
IGKV7-3	0	0	0	0	0	0	0	0
IGLC1	0	0	0	0	0	0	0	0
IGLC2	0	0	0	0	0	0	0	0
IGLC3	0	0	0	0	0	0	0	0
IGLC4	0	0	0	0	0	0	0	0
IGLC5	0	0	0	0	0	0	0	0
IGLC6	0	0	0	0	0	0	0	0
IGLC7	0	0	0	0	0	0	0	0
IGLCOR22-1	0	0	0	0	0	0	0	0
IGLCOR22-2	0	0	0	0	0	0	0	0
IGLJ1	0	0	0	0	0	0	0	0
IGLJ2	0	0	0	0	0	0	0	0
IGLJ3	0	0	0	0	0	0	0	0
IGLJ4	0	0	0	0	0	0	0	0
IGLJ5	0	0	0	0	0	0	0	0
IGLJ6	0	0	0	0	0	0	0	0
IGLJ7	0	0	0	0	0	0	0	0
IGLJCOR18	0	0	0	0	0	0	0	0
IGLL1	0	0	0	0	0	0	0	0
IGLL3P	0	0	0	0	0	0	0	0
IGLL4P	0	0	0	0	0	0	0	0
IGLL5	0	0	0	0	0	0	0	0

IGLV1-36	0	0	0	0	0	0	0	0
IGLV1-40	0	0	0	0	0	0	0	0
IGLV1-41	0	0	0	0	0	0	0	0.596974
IGLV1-44	0	0	0	0	0	0	0	0
IGLV1-47	0	0	0	0	0	0	0	0
IGLV1-50	0	0	0	0	0	0	0	0
IGLV1-51	0	1.642249	0	2.052727	0	3.943937	0	0
IGLV1-62	0	0	0	0	0	0	0	0
IGLV10-54	0	0	0	0	0	0	0	0
IGLV10-67	0	0	0	0	0	0	0	0
IGLV11-55	0	0	0	0	0	0	0	0
IGLV2-11	0	0	0	0	0	0	0	0
IGLV2-14	0	0	0	0	0	0	0	0
IGLV2-18	0	0	0	0	0	0	0	0
IGLV2-23	0	0	0	0	0	0	0	0
IGLV2-28	0	0	0	0	0	0	0	0
IGLV2-33	0	0	0	0	0	0	0	0
IGLV2-34	0	0	0	0	0	0	0	0
IGLV2-5	0	0	0	0	0	0	0	0
IGLV2-8	0	0	0	0	0	0	0	0
IGLV3-1	0	0	0	0	0	0	0	0
IGLV3-10	0	0	0	0	0	0	0	0
IGLV3-12	0	0	0	0	0	0	0	0
IGLV3-13	0	0	0	0	0	0	0	0
IGLV3-15	0	0	0	0	0	0	0	0
IGLV3-16	0	0	0	0	0	0	0	0
IGLV3-17	0	0	0	0	0	0	0	0
IGLV3-19	0	0	0	0	0	0	0	0
IGLV3-2	0	0	0	0	0	0	0	0
IGLV3-21	0	0	0	0	0	0	0	0
IGLV3-22	0	0	0	0	0	0	0	0
IGLV3-24	0	0	0	0	0	0	0	0
IGLV3-25	0	0	0	0	0	0	0	0

IGLV3-26	0	0	0	0	0	0	0	0
IGLV3-27	0	0	0	0	0	0	0	0
IGLV3-29	0	0	0	0	0	0	0	0
IGLV3-30	0	0	0	0	0	0	0	0
IGLV3-31	0	0	0	0	0	0	0	0
IGLV3-32	0	0	0	0	0	0	0	0
IGLV3-4	0	0	0	0	0	0	0	0
IGLV3-6	0	0	0	0	0	0	0	0
IGLV3-7	0	0	0	0	0	0	0	0
IGLV3-9	0	0	0	0	0	0	0	0
IGLV4-3	0	0	0	0	0	0	0	0
IGLV4-60	0	0	0	0	0	0	0	0
IGLV4-69	0	0	0	0	0	0	0	0
IGLV5-37	0	0	0	0	0	0	0	0
IGLV5-45	0	0	0	0	0	0	0	0
IGLV5-48	0	0	0	0	0	0	0	0
IGLV5-52	0	14.810479	0	8.307482	0	20.63491	0	31.592431
IGLV6-57	0	0	0	0	0	0	0	0
IGLV7-35	0	0	0	0	0	0	0	0
IGLV7-43	0	0	0	0	0	0	0	0
IGLV7-46	0	0	0	0	0	0	0	0
IGLV8-61	0	0	0	0	0	0	0	0
IGLV8OR8-1	0	0	0	0	0	0	0	0
IGLV9-49	0	0	0	0	0	0	0	0
IGLVI-20	0	0	0	0	0	0	0	0
IGLVI-38	0	0	0	0	0	0	0	0
IGLVI-42	0	0	0	0	0	0	0	0
IGLVI-56	0	0	0	0	0	0	0	0
IGLVI-63	0	0	0	0	0	0	0	0
IGLVI-68	0	0	0	0	0	0	0	0
IGLVI-70	0	0	0	0	0	0	0	0
IGLVIV-53	0	0	0	0	0	0	0	0
IGLVIV-59	0	0	0	0	0	0	0	0

IGLVIV-64	0	0	0	0	0	0	0	0
IGLVIV-65	0	0	0	0	0	0	0	0
IGLVIV-66-1	0	0	0	0	0	0	0	0
IGLVIVOR22-	0	0	0	0	0	0	0	0
IGLVIVOR22-	0	0	0	0	0	0	0	0
IGLVV-58	0	0	0	0	0	0	0	0
IGLVV-66	0	0	0	0	0	0	0	0
IGLVVI-22-1	0	0	0	0	0	0	0	0
IGLVVI-25-1	0	0	0	0	0	0	0	0
IGLVVII-41-1	0	0	0	0	0	0	0	0
IGSF1	0	0	0	0	0	0	0	0
IGSF10	1.621032	0	0	0.535826	0	0	0	0
IGSF11	0	2.342122	0	1.218566	0	0.296631	0	0
IGSF21	0	0	0	0	0	0	0	0
IGSF22	0	0.367099	0	0.205105	0.221874	5.6284	0	0.168723
IGSF23	0	0	0	0	0	0.175116	0	0
IGSF3	0	3.120869	0	13.171271	0	17.312647	0	8.025665
IGSF3P1	0	0	0	0	0	0.150418	0	0.241955
IGSF3P2	0	0	0	0.041927	0	0	0	0
IGSF5	0	0.593124	0	0	0	0	0	0
IGSF6	0	0	0	0	0	0	0	0
IGSF8	15.107055	13.34707	22.451461	22.220116	15.320771	16.296593	8.751439	8.43948
IGSF9	8.133196	2.135866	1.710156	5.289057	6.895152	2.953764	0	4.493332
IGSF9B	0	1.121365	3.848736	0.503578	0.456474	0	0	0
IHH	0	0	0	0	0	0.205153	0	0
IHO1	0	0	0	0	0	0	0	0
IK	64.086043	95.026788	104.496504	108.830467	132.242787	135.295687	173.986524	82.161098
IKBIP	25.532875	28.564468	25.964846	14.08978	36.140622	18.888552	58.696456	31.670326
IKBKB	8.395319	20.32006	23.562202	27.819426	37.169858	15.342677	6.618352	9.057166
IKBKE	1.368384	21.751119	22.335138	21.843998	0.360999	35.209734	7.770583	37.79271
IKBKG	4.287178	10.574958	13.438407	15.404077	2.493294	12.062733	1.782077	2.327502
IKBKGP1	0	12.891476	0	9.893808	17.454462	13.546646	0	5.682311
IKZF1	0	0	0	0.014838	0	0	0	0.024351

IKZF2	6.795074	3.748792	12.016963	11.202119	15.4609	16.613952	2.518799	12.505484
IKZF3	0.328211	0	0	0.04018	0	0.065977	0	0
IKZF4	3.909463	1.154858	4.84901	2.856671	5.71497	2.81097	40.686475	0.827058
IKZF5	0	13.563657	0	14.788446	8.022619	7.276856	0	14.806753
IL10	0	0	0	0	0	0	0	0
IL10RA	8.316155	0.807831	0	1.888285	0	0.879186	0	2.807437
IL10RB	5.97227	18.752916	15.948985	8.41361	24.014836	4.027001	37.758308	6.272728
IL11	5.745418	4.978516	6.423058	4.803485	14.824828	1.386185	0	2.140994
IL11RA	0	1.094978	0	0.842498	0	2.643307	0	0
IL12A	0	1.571845	0	2.204157	1.216428	3.191738	0	1.519496
IL12RB1	0	0	0	0	0	0	0	0
IL12RB2	0	0.405655	0	0.939228	0	0.789474	0	1.09078
IL13	0	0	0	0	0	0	0	0
IL13RA1	38.030945	49.163247	36.426203	27.289103	28.726817	15.919249	28.971221	18.693738
IL13RA2	2.730363	9.883994	9.696585	5.820593	0	0.133614	0	0.664398
IL15	0	1.799611	2.324992	0.405086	0	0.861243	0	1.317512
IL15RA	11.40022	8.751738	22.19576	18.541614	12.590795	13.117406	0	6.661673
IL16	0	0	0	0.008188	0	0.228168	0	1.04939
IL17A	0	0	0	0	0	0	0	0
IL17B	0	0	0	0	0	0	0	0
IL17C	0	0	0	0	0	0	0	0
IL17D	0	0.715573	0	0.298344	0	0.929409	0	0.192013
IL17F	0	0	0	0	0	0	0	0
IL17RA	0	7.560564	0	4.819234	0	3.788776	0	7.990632
IL17RB	3.440226	3.082602	0	1.009794	0	0.536003	0	0
IL17RC	26.273181	11.362034	10.925375	10.868696	0	12.323702	62.296928	10.05667
IL17RD	2.203948	0.622069	2.715417	2.28366	0.194038	2.366467	0	0.738897
IL17RE	32.243105	7.416629	8.078144	7.037703	0	4.183499	0	2.186568
IL18	126.464095	61.418652	140.696352	27.272019	180.662957	60.772622	111.216045	123.004977
IL18BP	2.426808	2.211108	9.756594	5.548994	19.691061	7.172815	15.036029	1.413542
IL18R1	0	5.263676	5.384752	5.058017	0	5.34198	0	2.533143
IL18RAP	0	0	0	0	0	0	0	0.149141
IL19	0	0	0.841997	0	0	0	0	0

IL1B	6.056458	3.77996	2.707676	2.327462	1.48305	4.227718	0	2.066271
IL1F10	0	0	0	0	0	0	0	0.154983
IL1R1	0.630464	3.492627	2.265778	1.155689	1.238227	1.073617	7.755653	1.487116
IL1R2	6.68334	2.982755	9.07277	2.086891	0	2.276851	0	5.542609
IL1RAP	37.905067	53.029165	45.737039	84.153158	28.448989	72.202705	18.337004	59.048878
IL1RAPL2	0	0	0	0	0	0	0	0.725867
IL1RL1	12.341859	10.439864	4.498023	3.581957	0	0.562255	0	0.255449
IL1RL2	1.323565	8.868334	2.368988	2.384263	0.636728	4.166927	9.956733	2.286933
IL1RN	0	9.944613	3.594375	6.380256	21.249294	25.537917	10.194876	30.467554
IL2	0	0	0	0	0	0	0	0
IL20	0	0	0	0	0	0	0	0
IL20RA	0.985473	6.695875	0	3.703594	5.607597	8.5103	2.156373	1.641728
IL20RB	0	4.659033	0	5.656212	29.793773	8.522708	0	7.771236
IL21	0	0	0	0	0	0	0	0
IL21R	0	0.832707	0	0.249619	0	0.033907	0	0
IL22	0	0	0	0	0	0	0	0
IL22RA1	2.393816	2.040494	1.071915	2.33263	6.633082	4.884761	0	3.32297
IL22RA2	0	0	0	0	0	0	0	0
IL23A	0	5.579433	6.522986	9.20913	0	2.671556	0	1.957032
IL23R	0	0	0	0	0	0	0	0
IL24	0	0	0	0.168853	0	0.542666	0	0.562561
IL25	0	0	0	0	0	0	0	0
IL26	0	0	0	0	0	0	0	0
IL27	0	0	0	0	0	0	0	0
IL27RA	12.533007	12.780886	7.144998	16.949532	5.412734	21.767502	21.311472	13.111672
IL2RA	0	0	0	0	0	0	0	0
IL2RB	0	0.210377	0	0.439684	0	0.163655	0	1.090009
IL2RG	0	0	0	1.001176	0	0	0	0
IL31RA	4.313998	13.149883	9.326429	10.622287	0	0.096433	0	0.882437
IL32	10.41223	10.991605	27.106176	16.162122	20.960772	52.813457	60.877498	27.884232
IL33	0	0.1249	0	0.231718	0	0	0	0.059252
IL34	0	0.476417	0	0.092515	0	0.389937	0	0
IL36B	0	0	0	0	0	0	0	0

IL36G	0	1.697797	0	1.700544	0	1.047053	0	1.378338
IL36RN	0	2.211356	0	1.296893	0	2.648382	0	2.367293
IL37	0	1.524682	0	0	0	0	0	0
IL3RA	0	0	0	0.058989	0	0	0	0
IL4	0	0	0	0	0	0	0	0
IL4I1	0	0	0	1.810171	0	4.155906	0	1.047418
IL4R	43.86478	128.597899	39.528327	144.574512	38.696038	121.20727	41.005787	71.792316
IL5	0	0	0	0	0	0	0	1.261927
IL5RA	0	0	0	0	0	0	0	0
IL6	25.111978	31.201875	5.91235	17.56322	47.245689	7.908007	0	7.188088
IL6R	5.195388	7.600313	19.71314	4.862334	6.71535	19.615244	57.769203	19.52795
IL6RP1	0	0	0	0	0	0	0	0
IL6ST	81.163572	101.329647	105.089528	75.523122	82.740259	32.118969	49.04944	61.068994
IL6STP1	0	0	0	0.090633	0	0	0	0
IL7	7.882152	3.784493	0	5.891231	0	1.504563	0	1.595228
IL7R	22.374001	6.02483	9.648554	5.77992	15.703103	10.867105	0.348828	16.663792
IL9R	0	0	0	0	0	0	0	0
IL9RP1	0	0	0	0	0	0	0	0
IL9RP2	0	0	0	0	0	0	0	0
IL9RP3	0	0	0	0.307484	0	0.268405	0	0.170992
IL9RP4	0	0	0	0	0	0	0	0
IL9RP5	0	0	0	0	0	0	0	0
IL9RP6	0	0	0	0	0	0	0	0
ILDR1	0	0.379498	0	1.954455	0.335974	2.774296	0	1.946261
ILDR2	0	0.18158	0	0.035159	0	0	0	0.267465
ILF2	222.672281	177.711954	188.752469	162.990188	138.582977	139.483049	205.189038	167.366379
ILF2P1	0	0	0	0	0	0	0	0
ILF2P2	0	0	0	0	0	0	0	0
ILF3	54.614919	109.787819	55.60673	117.808759	139.166913	141.456075	202.711525	182.103944
ILK	43.866635	33.344484	7.72364	37.829917	125.38254	29.123835	151.644438	19.94586
ILKAP	12.395517	30.342318	15.429263	20.837861	28.512254	43.61548	0	40.923262
ILRUN	69.041603	157.805261	58.031201	151.904218	109.11966	231.149641	66.373949	170.508194
ILRUNP1	0	0	0	0	0	0	0	0

ILVBL	10.400468	21.690667	17.804416	17.928155	22.7293	49.786785	13.328275	31.864372
IMMP1L	27.923259	19.25773	23.184037	13.872672	10.506056	21.177694	25.396245	88.375222
IMMP1LP1	0	0	0	0	0	0	0	0
IMMP1LP2	0	0	0	0	0	0	0	0
IMMP1LP3	0	0	0	0	0	0	0	0
IMMP2L	10.916769	24.676933	4.257912	14.489853	0	7.420732	0	3.185914
IMMT	107.370588	147.974127	99.465661	184.089101	62.642999	77.985234	12.686802	77.47981
IMMTP1	0	0	0	0	0	0	0	0
IMP3	0	5.247103	0	2.812321	6.080234	10.837582	0	2.483346
IMP3P1	0	0	0	0	0	0	0	0
IMP3P2	0	0	0	0	0	0	0	0
IMP4	67.017523	94.196094	16.692407	106.03613	115.664811	206.489444	47.026208	179.211822
IMPA1	37.929602	28.861328	15.330936	18.123533	4.702061	7.008275	0	11.064138
IMPA1P1	0	0	0	0	0	0	0	0
IMPA2	9.963433	23.996245	6.643455	21.631308	68.903807	41.575727	29.66707	36.781806
IMPACT	39.442795	53.073156	36.863269	38.173836	41.685285	18.964291	100.903169	47.127608
IMPDH1	28.460113	47.916422	0	41.396477	20.579398	63.215062	49.118112	49.889726
IMPDH1P10	0	0	0	0.336965	0.611058	2.509343	0	0.939039
IMPDH1P11	0	0	0	0	0	0	0	0
IMPDH1P2	0	0	0	0	0	0	0	0
IMPDH1P3	0	0	0	0	0	0	0	0
IMPDH1P4	0	0	0	0	0	0	0	0
IMPDH1P5	0	1.073555	2.067863	0.281212	0	2.293488	15.140044	1.598748
IMPDH1P6	0	0	0	0	0	0.232795	0	0.383621
IMPDH1P7	0	0	0	0	0	0	0	0
IMPDH1P8	0	0.197308	0	1.023532	0	1.847482	0	1.047335
IMPDH1P9	0	0	0	0	0	0	0	0
IMPDH2	246.390591	243.00619	197.073778	266.700971	200.661232	219.819861	163.433232	221.656818
IMPG1	0	0	0	0	0	0	0	0
IMPG2	0.77174	0.083113	0	0	0.611899	0.038759	0	0
INA	55.488245	35.289355	46.977998	23.403165	18.98789	6.865918	3.746554	7.972654
INAFM2	0	4.339255	0	0	44.666581	0	0	0
INAVA	24.909132	25.419071	17.913459	26.089349	28.249919	63.058178	9.32594	35.746206

INAVAP1	0	0	0	0.228982	0	0.922149	0	0.423767
INCA1	0	0	0	0.382886	0	0.740924	0	0
INCENP	0	9.955021	6.772432	27.300766	72.363151	49.749592	0	54.55956
INF2	18.901205	14.078689	11.075238	19.739753	32.605469	27.601801	64.65517	27.165031
ING1	0	9.364868	0	6.836796	1.018613	5.367404	0	11.102508
ING2	11.086656	47.134673	19.67817	25.752195	3.438872	35.556077	5.042662	32.122058
ING3	4.989603	6.415714	10.433417	7.236278	6.38649	3.327318	0	6.962598
ING4	17.168353	11.120105	7.210934	15.220722	1.393121	4.903629	0	5.580781
ING5	4.219987	29.586582	4.567624	36.740123	24.275461	44.075933	8.215704	25.035891
INGX	0	0	0	0.113571	0	0	0	1.365055
INHA	0	1.128477	0	0.065207	0	0	0	0.109483
INHBA	20.06188	26.856325	21.414379	50.293293	35.407202	69.491516	49.048532	57.418752
INHBB	0	0.267221	0	0.279705	0	0.727891	0	0.335419
INHBC	0	0.579735	0	1.706817	0	0.364422	0	0.167933
INHBE	0	0.647542	0	1.516231	0	1.134623	0	0
INHCAP	0	0.286038	0	0	0	0	0	0
INIP	12.029076	31.506864	30.263604	32.778161	5.082824	32.15104	66.468524	39.11617
INKA2	4.918587	18.251182	3.438751	11.981829	0.433189	13.917501	0	6.553643
INMT	0	0.056457	0	0	0	0	0	0
INMT-MIND\	0	0	2.09639	0	0	0	0	0
INO80	44.963799	27.392238	32.644612	47.478911	12.176408	30.870147	0	31.620772
INO80B	36.865071	38.596926	27.045635	57.063673	47.122269	36.346537	22.011661	19.663136
INO80B-WBI	0	5.933596	0	0	0	5.174039	0	5.042536
INO80C	0	38.892956	0.967961	13.07635	0	5.645074	5.382978	5.425699
INO80D	8.137308	9.53879	6.920782	18.667376	27.348691	22.018959	10.100155	18.771462
INO80E	43.051605	84.221049	55.852517	90.539014	43.897069	104.79813	127.754058	78.78131
INPP1	7.265033	68.985279	29.176068	55.782353	37.330655	67.135574	79.887381	70.118969
INPP4A	0	0.108229	0	1.250156	0	2.424741	0	0.715436
INPP4B	32.669887	110.173661	28.992782	139.882784	22.933327	111.969781	11.089346	122.117407
INPP5A	12.310507	27.343422	43.646348	16.030793	31.90104	25.492167	48.596492	37.886363
INPP5B	2.54093	3.191293	6.080286	4.527829	19.11853	8.24835	6.035568	7.003995
INPP5D	2.745272	3.372721	6.959232	2.303897	1.691313	5.896345	2.893452	1.849561
INPP5E	10.722867	8.083093	9.393853	7.861053	12.848283	14.735395	0	12.510123

INPP5F	0	9.710533	0	14.156388	15.298041	9.466599	29.862471	18.114169
INPP5J	0	0	2.032374	0.39643	0	0.59622	0	0.09239
INPP5K	4.998314	21.061552	10.068761	27.267095	48.199799	38.154078	0	35.681739
INPPL1	26.691629	35.14667	89.199097	43.196961	65.985308	69.610717	11.596834	48.036638
INS	0	0	0	0	0	0	0	0
INS-IGF2	0	0	0	0	0	0	0	0
INSC	0	0	0	0.201254	0	0	0	0
INSIG1	53.218733	60.258204	43.330504	32.345194	95.842842	86.877346	33.15934	94.089169
INSIG2	23.707377	21.826367	37.30288	13.058117	29.027115	1.352995	0	37.085949
INSL3	0	0	0	0.395944	0	0	0	0
INSL4	0	0	0	0	0	0	0	0
INSL6	0	0	0	0	0	0	0	0
INSM1	0	0.100931	0	0.11537	0	0	0	0
INSM2	0	0	0	0	0	0	0	0
INSR	1.437536	4.385337	3.557289	8.533204	4.554342	2.60759	0	1.598506
INSRR	0	0	0	0	0	0	0	0
INSYN1	0	0	0	0	0	0.25198	0	0
INSYN2A	0	0	0	0.018543	0	0	0	0.128213
INSYN2B	24.979133	22.941739	5.633558	32.850993	0.150551	3.541356	0.138867	6.19341
INTS1	48.688278	55.56072	69.813231	64.474066	24.597843	57.509081	22.727217	35.703298
INTS10	31.068201	34.44868	68.588164	24.810993	38.313809	19.785629	88.844381	46.829958
INTS11	62.774522	23.210825	42.979655	13.131005	76.146401	34.397028	94.620781	31.873544
INTS12	0	32.01987	18.307602	28.910303	2.498821	21.666412	0	22.985665
INTS13	83.835114	64.267215	50.074667	43.57881	48.663246	33.449476	56.739297	57.178705
INTS14	16.272097	60.587193	0	62.849404	27.137149	80.933272	34.938569	91.706344
INTS15	22.125501	63.617703	18.359653	57.986844	15.003578	58.331365	18.435852	51.004198
INTS2	5.651253	3.685641	0	1.42919	0.285534	0.792001	28.221648	2.959043
INTS3	16.847283	52.737753	29.336727	47.322686	31.794714	69.998047	18.508538	61.176968
INTS4	19.15166	15.174682	27.077031	21.256019	20.122802	8.912692	0.796868	14.785985
INTS4P1	0	0	0	0	0	0	0	0
INTS4P2	0	0	0	0	0	0	0	0
INTS5	7.114066	40.378258	8.199875	33.829946	15.815841	124.629102	8.89372	131.159908
INTS6	20.034669	59.742074	18.640397	45.209784	49.632199	19.548011	10.192617	60.443944

INTS6L	0.873258	0	3.134011	2.028187	0	3.140569	5.500631	2.348126
INTS6P1	0	0	0	0	0	0	0	0
INTS7	20.352342	31.554106	28.477243	21.433968	9.490551	16.333241	5.707695	38.393314
INTS8	81.278899	23.389221	29.565114	25.034052	19.337284	14.759801	47.563699	19.168812
INTS9	7.995104	7.677456	9.539567	14.709258	12.388805	7.40554	2.790455	9.7128
INTU	3.420019	6.504458	3.761827	2.833421	2.670223	0.560732	0.76811	1.97484
INVS	13.453348	26.020507	23.231608	27.575093	5.299377	27.37245	24.62536	27.504337
IP6K1	11.027449	37.538301	11.22203	30.804987	80.220471	61.530577	38.184008	50.172236
IP6K2	56.800193	77.819075	37.292758	93.90443	28.795768	73.850713	74.412336	64.879315
IP6K3	0	0	0	0	0	0	0	0
IPCEF1	2.044456	0.166758	0	0.715528	0	0	0	0
IPMK	9.067414	5.571273	4.794816	2.551834	3.94604	3.07549	7.540613	7.20777
IPMKP1	0	0	0	0	0	0	0	0
IPO11	0	16.185496	0	29.2272	45.016948	24.056046	178.345984	16.20844
IPO13	5.112081	12.03772	7.645337	10.501388	16.883419	38.445371	0	33.60973
IPO4	30.010909	16.656622	17.870434	16.744864	38.46275	35.739003	33.420098	32.534874
IPO5	136.955486	150.788921	106.833459	116.141793	78.698493	46.708645	147.692201	108.671148
IPO5P1	0	0	0	0	0	2.844263	147.013228	0
IPO7	105.387207	90.774453	118.017088	45.118435	142.910858	40.976281	78.265337	143.0928
IPO7P1	0	0	0	0	0	0	0	0
IPO7P2	0	0	0	0	0	0	0	0
IPO8	25.093061	55.770839	25.927259	42.437248	26.847383	39.53553	57.386734	44.06899
IPO8P1	0	0	0	0	0	0	0	0
IPO9	26.09687	51.963514	49.55407	65.208984	49.084045	50.984535	114.33379	41.357106
IPP	0	15.160521	0	2.995807	44.157292	1.263178	0	4.92851
IPPK	10.032123	10.331676	10.112159	9.686864	4.192571	10.623161	6.604346	12.825021
IPPKP1	0	0	0	0	0	0	0	0
IQANK1	62.467415	182.564271	55.09736	179.989313	33.317771	186.466441	60.541934	191.879415
IQCA1	16.11272	16.091889	31.690271	16.585006	36.604863	12.454626	12.330568	14.566464
IQCA1L	0	0	0	0	0	0	0	0
IQCB1	21.070856	44.387474	38.826127	51.167574	22.270282	39.695676	11.867242	44.16439
IQCB2P	0	0	0	0	0	0	0	0
IQCC	0	4.755701	0	1.473081	0	0.766229	0	0

IQCD	0	10.233262	2.3617	13.208711	1.675932	10.152656	0	6.209527
IQCE	5.667767	12.822967	6.020079	15.609219	32.498293	32.300907	39.196093	20.414452
IQCF1	0	0	0	0	0	0	0	0
IQCF2	0	0	0	0	0	0	0	0
IQCF3	0	0	0	0	0	0	0	0
IQCF4P	0	0	0	0	0	0	0	0
IQCF5	0	0	0	0	0	0	0	0
IQCF6	0	0	0	0	0	0	0	0
IQCG	21.426647	48.577142	22.081885	67.16208	22.752486	20.758425	33.803474	19.905966
IQCH	27.336612	25.271301	19.584159	19.866524	4.056008	2.994356	0	2.737339
IQCJ	0	0	0	0	0	0	0	0
IQCJ-SCHIP1	11.143875	16.895182	0	27.012895	27.177348	33.83549	0	32.954462
IQCK	7.281818	20.562694	3.249198	15.192618	6.718036	8.614362	9.691716	14.156899
IQCM	0	0	0	0.527418	0	0.093748	0	0.076657
IQCN	0.867013	0	0.530793	0.063485	0	0.293792	18.8614	0.023692
IQGAP1	66.886813	61.915227	99.447536	105.646135	240.047486	174.224201	198.148149	133.126817
IQGAP2	0	0	0	0.273448	0	0	0	1.007846
IQGAP3	12.496318	6.880417	9.72083	7.199424	17.337166	8.864381	7.513332	5.197982
IQSEC1	11.168942	19.544727	9.315098	21.178431	7.943664	24.981522	6.691089	17.383467
IQSEC2	1.637541	5.581747	10.664763	5.065369	1.52511	6.533455	0	1.499025
IQSEC3	0	0	0	0	0	0	0	0
IQSEC3P1	0	0	0	0	0	0	0	0
IQSEC3P3	0	0	0	0	0	0	0	0
IQUB	0	0	0	0.026952	0	0	0	0.222359
IRAG1	0	0.336782	7.541651	0	0	0.168332	0	1.398187
IRAG2	0	0	0	0.079109	0	0	0	0.366234
IRAK1	85.353009	115.91849	125.738557	93.969577	115.535692	237.731491	139.894442	172.459412
IRAK1BP1	2.619189	1.481444	1.575606	2.242582	4.007179	0.882773	6.265949	0.867296
IRAK2	11.647243	9.542792	28.721915	19.721981	5.893685	6.198889	7.552048	5.773177
IRAK3	0	0	0	0	0.497619	0	0	0
IRAK4	15.558336	2.488545	8.435278	1.518971	0	0.970695	57.194221	1.766272
IREB2	25.443522	31.934725	25.942553	18.493933	47.241908	31.157544	17.14333	64.339348
IRF1	24.3452	29.662685	14.772361	31.094853	30.200717	64.359635	11.077014	38.765053

IRF2	19.762307	15.775197	16.307208	42.558725	59.608319	33.233682	20.266889	32.159469
IRF2BP1	4.023064	31.765329	8.399986	31.988598	16.6285	93.490457	36.443586	54.248392
IRF2BP2	34.515283	135.955566	91.175528	171.352664	84.999165	175.763044	24.780609	99.654584
IRF2BPL	22.16108	76.950395	22.734349	72.990471	21.111327	94.98495	10.402509	71.184695
IRF3	95.815138	81.374224	59.525544	107.500305	99.537242	193.346698	159.547618	151.183971
IRF4	0	0	0	0.019286	0	0	0	0
IRF5	8.125345	3.932134	8.464708	7.594772	0	3.978657	41.712288	3.697449
IRF5P1	0	0	0	0	0	0	0	0
IRF6	205.504072	168.483564	146.724612	250.642446	132.376396	222.644664	112.760503	164.496219
IRF7	8.977309	6.365326	9.67254	9.163288	55.705512	71.903838	17.292863	50.604245
IRF8	0	0	0	1.950704	0	0.199257	0	0
IRF9	0	0	0	0	0	0	0	0
IRGC	0	0	0	0	0	0	0	0
IRGM	0	0.754408	0	0	0	0	0	0
IRGQ	0	0	0	0	0	0	0	0
IRS1	0	4.451774	0	3.050317	0	8.438574	0	20.048213
IRS2	7.90713	6.879791	10.314916	10.672756	6.68727	9.033455	8.101231	8.003333
IRS3P	0	0	0	0	0	0	0	0
IRX1	0	0	0	0	0	0	0	0
IRX1P1	0	0	0	0	0	0	0	0
IRX2	26.912978	53.088375	16.58382	42.238741	5.746738	44.458446	12.167773	36.736504
IRX2-DT	11.430558	50.658121	35.417289	36.093614	4.531734	30.913387	0	18.566693
IRX3	11.619329	27.58547	20.015401	21.473176	91.596138	20.301751	7.520371	14.234897
IRX4	0	4.909623	0	1.986952	0	0.892811	0	0.148104
IRX5	5.03665	2.02357	10.276266	5.306977	1.327975	5.187183	0	1.599019
IRX6	0	0	0	0	0	0	0	0
ISCA1	22.975863	24.146655	20.510134	17.427198	28.886657	11.103215	53.238278	43.995856
ISCA1P1	0	16.688333	0	0	0	11.421852	0	17.296405
ISCA1P2	0	0	0	0	0	0	0	0
ISCA1P3	0	0	0	0	0	0	0	0
ISCA1P4	0	0	0	0	0	0	0	0
ISCA1P6	0	0	0	0	0	0	0	0
ISCA1P7	0	0	0	0	0	0	0	0

ISCA2	67.21643	58.376903	16.164538	29.573656	32.176826	34.751723	34.85942	49.908176
ISCA2P1	0	0	0	0	0	0	0	0
ISCU	13.692195	0.390418	0	1.919671	4.993043	17.436102	16.507268	13.487061
ISCUP1	0	0	0	0	0	0	0	0
ISG15	20.84442	33.602509	18.076273	20.636046	152.357717	318.68746	58.853335	256.067487
ISG20	0	6.350589	0	9.092658	0	8.970033	0	5.656452
ISG20L2	0	43.855531	22.782683	77.707552	31.781751	72.99906	0	74.835249
ISG20L2P1	0	0	0	0	0	0	0	0
ISG20L2P2	0	0	0	0	0	0	0	0.15181
ISL1	0	4.01459	2.550776	2.239962	0	2.84018	0	3.345979
ISL2	0	1.873129	2.219674	2.934245	0	1.039194	0	0.738385
ISLR	0	0	0	0	0	0	0	0
ISLR2	0	1.103946	0	0.076616	0	0	0	0
ISM1	1.084482	0.138867	0	0.290799	0	0	0	0
ISM2	0	1.11069	1.024393	0.473711	0	0.099935	12.245368	0.137992
ISOC1	28.680026	28.123441	28.798311	19.408714	19.845671	12.053214	16.485777	22.137599
ISOC2	14.14372	28.049281	24.988387	33.627193	31.38035	70.393949	0.847508	44.267907
IST1	196.012333	293.240593	169.175927	359.11666	312.852981	318.275864	228.825967	319.23321
ISX	0	0	0	0	0	0	0	0
ISY1	41.175755	192.058891	63.068772	171.182169	0	147.234923	72.105597	126.164465
ISY1-RAB43	0	16.386428	6.677514	18.416193	16.883691	29.989495	15.062669	23.25862
ISYNA1	35.910959	19.049375	12.483638	41.336547	63.560467	61.758031	27.262135	37.18374
ITCH	23.190045	73.973812	59.882029	51.294142	12.051978	22.988898	3.626249	35.841529
ITFG1	96.040193	40.559586	70.924829	20.566814	22.755097	5.696514	8.012352	21.197859
ITFG2	30.033971	16.388113	1.854294	8.264244	3.858785	12.218054	110.844641	12.978704
ITFG2-AS1	0	0	0	0.379952	0	0	0	0
ITGA1	10.006562	19.792674	29.083302	26.532576	6.192283	4.466215	0.100494	9.206078
ITGA10	0	0	0	0.01692	0	0.034661	0	0.31475
ITGA11	0	1.040741	0	0.158558	0	0	0	0
ITGA2	84.44265	49.801662	121.421585	75.579732	22.988164	17.633702	35.642972	30.73054
ITGA2B	11.238645	1.211211	0	1.498985	0.341619	0.622233	0	0.104276
ITGA3	111.430297	196.915289	153.324481	290.789956	171.621079	367.289897	423.555558	247.155521
ITGA4	0	0.082223	0	0.362187	0	0.253387	0	0.404363

ITGA5	12.401884	9.467529	16.700095	15.634322	63.808261	20.54245	104.322399	15.625345
ITGA6	59.538662	98.25497	10.19025	152.667915	235.112383	384.605096	244.584882	203.401996
ITGA7	10.143456	0.327582	0	0.990163	0	0	0	0.745146
ITGA8	0	0	0	0	0	0	0	0
ITGA9	0	0	0	0.17417	0	0	0	0
ITGAD	0	0	0	0	0	0	0	0
ITGAE	32.254684	46.991519	5.610482	24.794709	0	40.051877	0	78.431115
ITGAEP1	0	0	0	0	0	0	0	0
ITGAL	0	0	0	0.245926	0	0	0	0
ITGAM	0	0.119263	0.624166	0.050938	0	0.68813	0	0
ITGAV	39.445993	51.540179	97.911066	76.412032	44.28875	25.761716	22.418441	43.355844
ITGAX	0	0	0	0.676508	0	0	0	0
ITGB1	165.543625	242.55229	246.433661	286.945166	114.132446	84.587686	166.262882	145.042953
ITGB1BP1	126.72023	289.678512	149.418232	274.643331	120.433728	231.289445	227.056972	368.081824
ITGB1BP2	0	0	0	0	0	0	0	0
ITGB1P1	142.809115	54.257923	0	70.002551	100.712874	9.134024	118.100605	13.618307
ITGB2	14.736551	30.70258	7.241222	25.413825	0	2.744413	0	1.206588
ITGB3	0	0	0	0	0	0.638118	0	0
ITGB3BP	58.456951	30.865621	25.916395	12.671721	57.915119	4.151007	0	4.845869
ITGB4	128.819362	91.652867	211.471122	157.346835	168.016953	180.757796	163.679216	94.049576
ITGB5	45.477346	105.551073	79.708355	130.132236	120.886299	185.661249	69.815865	100.687782
ITGB6	28.103516	45.046387	43.452989	59.923652	32.367567	24.929709	29.430282	39.325608
ITGB7	0	0	0	0.222068	0	1.641955	0	0
ITGB8	26.239954	39.25076	14.662297	31.218715	25.94972	64.595319	44.652715	78.529525
ITGBL1	3.091781	5.265944	3.086862	5.214358	0	2.553316	0	2.022552
ITIH1	0	0	0	0	0	0	0	0
ITIH2	0	0	0	0	0	0	0	0
ITIH3	0	0	0	0	0	0	0	0
ITIH4	0	0	0	0	0	0	0	0
ITIH5	0	0	0	0	0	0	0	0
ITIH6	0	0	0	0	0	0	0	0
ITK	0	0	0	0	0	0	0	0
ITLN1	0	0	0	0	0	0	0	0

ITLN2	0	0	0	0	0	0.864151	0	0
ITM2A	9.262123	0	0	0	0	0	0	0
ITM2B	159.559266	300.604643	271.199289	214.354915	178.099251	200.83668	173.428095	326.755612
ITM2BP1	0	0	0	0	0	0	0	0
ITM2C	40.814959	42.267388	45.560656	65.052915	39.776777	58.515904	40.185746	50.480087
ITPA	33.240388	58.798019	39.137916	63.147099	24.254776	56.045329	19.151772	57.291537
ITPK1	78.215092	79.701092	58.893281	105.842353	66.277928	209.650262	28.992967	150.490381
ITPK1P1	0	0	0	0	0	0.144547	0	0
ITPKA	1.920272	1.621459	0	1.980796	0	4.485943	0	2.093932
ITPKB	1.639141	6.510156	0	3.461816	1.112186	3.501887	0.25857	2.808913
ITPKC	3.949302	17.722291	8.852719	24.913414	11.208115	39.649471	13.442509	25.870087
ITPR1	3.543107	2.101122	1.596363	6.51741	2.258068	1.190058	23.745757	7.111809
ITPR2	2.545912	18.748605	25.671993	15.555199	41.134219	20.93296	11.622874	33.746169
ITPR3	27.992366	36.609715	50.226246	55.488827	52.633446	76.752925	35.792862	43.46606
ITPRID1	0	0	0	0	0	0	0	0
ITPRID2	93.764888	245.913285	60.934055	165.683091	140.85181	249.592796	170.115624	270.820236
ITPRIP	10.669606	18.492122	12.815092	24.073911	19.347684	26.184374	9.897021	18.506203
ITPRIPL1	17.629829	21.019292	3.854139	17.474594	0	4.755286	0	4.856262
ITPRIPL2	0	5.837681	0	5.876187	0	14.933644	0	3.264631
ITSN1	13.16768	20.471978	59.79402	16.757472	22.256231	27.672149	91.696382	26.128277
ITSN2	21.678143	24.128785	17.004825	28.043757	17.881999	15.067952	43.714245	23.347481
IVD	13.948689	17.055617	21.78135	17.881732	13.419208	42.272679	32.067256	34.624898
IVL	0	2.036585	1.430766	1.050773	0	1.110744	0	0.839322
IVNS1ABP	23.096517	33.75296	19.317989	43.991671	61.149766	20.774956	52.834823	64.035759
IWS1	69.596813	77.386108	26.321145	72.363418	38.770309	110.549229	63.625333	103.752806
IYD	0	0	0	0	0	0	0	0
IZUMO1	0	0.262913	0	0.037666	0	0	0	0.433732
IZUMO1R	0	0	0	0	0	0	0	0
IZUMO2	0	0	0	0	0	0	0	0
IZUMO3	0	0	0	0	0	0	0	0
IZUMO4	0	0.370078	0	0.170735	0	0.268423	0	1.03762
JADE1	16.081183	14.893391	11.18374	20.780865	9.85004	24.268186	0	21.127244
JADE2	18.14799	36.116613	25.693934	36.081893	48.51008	88.243435	15.903271	61.993793

JADE3	11.91059	29.205549	7.499877	25.92965	22.494302	35.555938	14.404617	25.223817
JAG1	24.639929	34.244485	40.851439	54.405646	41.412587	35.893603	98.846004	57.316325
JAG2	5.075224	25.295989	40.090211	32.045087	28.207259	48.903362	53.513778	29.8188
JAGN1	21.68662	41.865362	37.154465	34.146879	15.55398	20.165917	9.427909	33.200805
JAK1	80.269011	113.514398	153.457469	169.053388	55.907169	63.599455	60.537744	91.724216
JAK2	0	2.802303	0	1.699782	0	1.723271	0	2.466034
JAK3	1.200555	0	0	0	2.21775	0	0	0
JAKMIP1	0	0	0	0.046306	0	0.065242	0	0
JAKMIP1-DT	0	0	0	0	0	0	0	0
JAKMIP2	0	0	0	0	0	0	0	0
JAKMIP3	1.126738	0.211254	2.361942	0.983941	0	0.434008	0	2.336826
JAM2	0.783013	0	0	0.038241	0	0.296452	0	0.242818
JAM3	1.982725	2.310408	2.666614	1.441293	7.328094	8.02601	50.463547	6.217456
JAML	2.421955	0	0	0.305826	0	0	0	0
JARID2	7.572017	24.255831	12.171853	30.871728	9.84867	57.03281	13.597554	43.348788
JAZF1	2.108743	8.787483	7.225442	14.120454	2.432864	3.356992	0	3.299449
JCAD	13.793446	25.295129	15.205921	28.858061	2.825499	3.967783	0.084143	3.38451
JCADP1	0	0	0	0	0	0	0	0
JCHAIN	0	0	0	0	0	0	0	0
JCHAINP1	0	0	0	0	0	0	0	0
JDP2	32.754829	37.137815	18.159279	48.585707	60.58674	94.272843	5.524921	47.420589
JHY	9.296155	35.144022	16.202052	31.400039	0.466696	6.876452	12.85586	6.402954
JKAMP	48.476163	60.920917	67.415079	28.627332	23.225522	12.105334	90.566147	50.771821
JKAMPP1	0	0	0	0	0	0	0	0
JMJD1C	1.870352	49.000797	28.488655	65.333612	36.3407	52.753325	25.065444	61.783741
JMJD4	0	14.684872	0	3.688161	0	4.133699	0	5.499715
JMJD4P1	0	0	0	0	0	0	0	0
JMJD6	0.699771	7.848955	25.579808	9.784038	2.003977	13.009249	3.971307	6.518839
JMJD7	8.729209	12.841178	40.637902	13.511073	0	18.753164	15.495667	18.257135
JMJD7-PLA2(	0.472627	0.227756	1.102176	0.532126	0.23682	1.230306	0	0.94856
JMJD8	63.720362	37.69385	39.494554	37.446072	47.087525	39.111643	40.888637	33.66235
JMY	4.95121	6.066127	7.319698	9.32776	8.693202	9.88124	4.83233	12.553174
JOSD1	34.572698	140.17142	51.874954	218.32027	59.434605	198.622134	129.038881	168.161201

JOSD2	0	6.79684	8.930231	12.460898	1.500865	10.599825	0	9.533972
JPH1	8.592334	30.750002	6.423646	27.159525	13.936152	30.00744	24.447264	26.680372
JPH2	0	4.990688	1.617203	1.034054	5.489239	7.720451	0	12.734509
JPH3	11.261215	27.019405	10.110798	29.234643	0.650389	13.782942	3.424476	8.88222
JPH4	0	0	0	0.219827	0	0	0	0
JPT1	708.046614	716.202628	436.107982	634.25817	539.10135	515.21082	531.008957	519.907426
JPT1P1	0	0	0	0	0	0	0	0
JPT2	159.128907	215.422997	184.373288	278.077746	171.922224	348.601168	98.076168	246.848359
JRK	3.469758	4.289574	3.566216	12.784321	0.476132	11.526378	0.703248	8.143374
JRKL	14.925966	31.880604	26.754352	28.375711	25.046374	9.405629	30.572827	23.546513
JSRP1	0	2.493582	4.195063	1.949558	0	1.372688	0	0.587751
JTB	106.677112	225.425226	120.132032	174.662345	71.96871	170.781909	53.652976	232.730021
JTBP1	0	0	0	0	0	0	0	0
JUNB	97.63645	184.211784	175.879294	267.432256	226.036665	481.769098	201.882029	363.539556
JUND	0	12.502024	0	0	0	81.292227	0	7.847583
JUP	153.916744	139.224053	169.403748	182.122576	209.373211	228.438663	112.847298	174.76368
KALRN	0	1.359207	0	1.994012	1.318331	1.520024	0	0.059933
KANK1	0	10.576967	0	17.578132	17.991127	31.423437	19.404401	42.234946
KANK1P1	0	0	0	0	0	0	0	0
KANK2	18.388199	38.064955	17.741827	64.67033	0	113.493307	33.055516	42.296064
KANK3	0	2.295923	0	1.526359	0	0.361485	0	0.292304
KANK4	0	0	0	0	0	0.059424	0	0
KANSL1	53.561505	45.143696	121.344254	50.376237	106.705604	40.330501	201.955859	48.982088
KANSL1L	2.767545	4.270738	1.310678	2.515329	1.99793	3.217037	15.254859	4.662263
KANSL2	5.378781	15.605644	0	14.823959	24.418745	21.627969	0	21.475925
KANSL3	20.508213	40.996333	21.116453	50.077275	20.417563	66.09174	51.788996	52.452503
KANTR	3.031148	14.037929	9.055384	16.157386	1.601126	15.356753	3.874007	18.162003
KARS1	44.417734	100.895566	40.870499	128.255987	56.865592	133.458023	84.988441	138.723414
KARS1P1	0	0	0	0	0	0	0	0
KARS1P2	0	0	0	0	0	0	0	0
KARS1P3	0	0	0	0	0	0	0	0
KASH5	0	0	0	0	0	0	0	0
KAT14	0	0.427388	0	0	0	1.831715	0	3.78127

KAT2A	15.05326	21.439729	18.308623	17.209945	10.967922	26.524208	76.474587	21.940681
KAT2B	2.237322	6.574768	7.003934	6.023964	2.757613	4.360271	0	6.552248
KAT5	46.100122	32.796928	40.312712	43.452024	13.403495	40.333687	56.910512	23.797169
KAT6A	31.440124	84.85606	28.92079	85.042184	25.26337	49.597461	61.909616	57.864744
KAT6B	7.924044	12.284786	10.432808	19.922224	42.715344	17.107106	30.963532	23.29566
KAT7	20.274849	19.331723	29.620981	21.253007	46.658694	25.985348	66.216183	32.299093
KAT7P1	0	0.498555	0	0	0	0	0	0
KAT8	26.797125	23.242864	32.536374	24.673059	27.249401	21.003536	39.439259	16.061823
KATNA1	30.062065	33.341853	26.204562	29.551244	15.496643	19.929822	7.973537	39.609367
KATNAL1	6.522596	12.67875	5.720465	14.948686	7.97667	11.423591	44.291569	12.630798
KATNAL2	0	0.754617	0	0.601909	0	1.947054	0	2.619788
KATNB1	41.122244	55.220315	19.701225	69.672745	20.760396	80.80697	42.194944	65.657879
KATNBL1	42.649027	86.822853	20.067573	35.204927	45.2379	27.04209	17.362332	64.994432
KATNBL1P1	0	0	0	0	0	0	0	0
KATNBL1P2	0	0	0	0	0	0	0	0
KATNBL1P3	0	0	0	0	0	0	0	0
KATNBL1P4	0	0	0	0	0	0	0	0
KATNBL1P5	0	0	0	0	0	0	0	0
KATNBL1P6	0	0	0	0	0	0	0	0
KATNIP	0	9.202719	6.739886	25.06804	0	20.014048	0	16.592788
KAZALD1	6.770971	9.517448	7.180426	14.087351	0.907854	5.524161	0	9.188536
KAZN	1.822246	3.107623	8.572195	7.658281	10.491588	26.024981	29.271783	14.688136
KBTBD11	1.448857	2.327144	2.605659	3.273075	0	0.751092	0	0.92933
KBTBD12	0	0	0	0	0	0	0	0
KBTBD2	23.010879	71.301087	26.419495	45.902393	36.138982	54.215493	53.047208	146.403202
KBTBD3	29.29494	12.754312	0.879791	14.0646	0.259034	8.486823	0	4.946811
KBTBD4	3.070389	5.032436	2.974365	7.63621	17.451575	18.809952	8.791815	17.571049
KBTBD8	2.102975	1.804318	12.877915	1.915179	2.036759	0.841962	0	15.328098
KCMF1	100.6897	99.666013	60.811187	49.680979	20.250186	23.841242	2.941944	65.542926
KCNA1	0	0	0	0.019775	0	0	0	0
KCNA10	0	0.300381	0	0.043105	0	0	0	0
KCNA2	0	0.283856	0	0.326989	0	0	0	1.643949
KCNA3	0	0	0	0	0	0	0	0

KCNA4	0	0	0	0	0	0	0	0
KCNAB1	0	1.979719	0	1.823828	0	2.198867	0	0.280875
KCNAB2	10.237999	16.803398	19.293029	31.301093	29.83865	42.336298	10.01737	22.760918
KCNAB3	0	0	0	0	0	0	0	0
KCNB1	0	0.751199	0	0.078323	0	0	0	0
KCNB2	0	0.151374	0	0.021581	0	0.176657	0	0.177615
KCNC1	0	0	0	0.059478	0	0.020339	0	0
KCNC2	0	0	0	0	0	0	0	0
KCNC3	0	0.307869	0.759133	0.996337	0	0.815353	9.931554	1.201472
KCNC4	6.653166	11.363847	2.905638	13.766321	4.229491	15.014609	1.534225	8.280771
KCND1	13.649534	2.445426	5.618729	4.106971	0.441555	3.200682	0	1.701707
KCND2	0	0.431864	0.501744	1.608774	0	0	0	1.141442
KCND3	0	0	0	0.106931	0	0.065807	0	0
KCNE1	0	0	1.905899	0.123662	0	0.251974	0	0
KCNE3	0	0	0	0.493473	0	1.343012	0	0
KCNE4	0	0	0	0	0	0	0	0
KCNE5	0	0	0	0	0	0.479998	0	0.396861
KCNF1	0	0.253866	0	0.072711	0	0	0	0.240972
KCNG1	1.781561	8.939825	8.930231	7.551168	19.763454	26.071184	48.768965	22.763736
KCNG2	0	0	0	0	0	0	0	0
KCNG3	0	0	0	0.0443	0	0.090645	0	0
KCNG4	0	0	0	0	0	0	0	0
KCNH1	0	0	0	0	0	0	0	0
KCNH2	0	0	0	0.051719	0	0	0	0
KCNH3	2.55328	5.621132	2.291136	3.954752	0	1.395018	0	1.56771
KCNH4	0.890529	0.114323	0	0.341484	2.116275	0.044473	0	0.107325
KCNH5	0	0	0	0	0	0	0	0
KCNH6	0	0	0	0	0	0	0	0
KCNH7	0	0	0	0	0	0	0	0
KCNH8	0	0	0	0.047225	0	0	0	0
KCNIP1	0	0	0	0	0	0	0	0
KCNIP2	0	0	0	0.080384	6.867486	0.081914	0	0
KCNIP3	0	0.205509	0	0.592163	0	3.151252	0	0.194045

KCNIP4	0	0	0	0	0	0	0	0
KCNJ1	0	0	0	0	0	0	0	0
KCNJ10	0	0	0	0	0	0.169141	0	0
KCNJ11	0	0.616312	0	0	0	0.954926	0	0.58286
KCNJ12	1.241068	1.359083	0	2.396741	0	0.590579	0	0.472988
KCNJ13	0	0	0	0	0	0	0	0
KCNJ14	3.312252	0	0	0.889484	0	2.855384	0	1.048724
KCNJ15	8.844967	4.176965	2.201965	4.282075	29.571637	60.395229	48.946387	69.922358
KCNJ16	0	0	0	0	0	0	0	0
KCNJ18	0	0	0	0	2.891343	0	0	0
KCNJ2	0	0.815498	5.611568	1.26586	0	0	0	0.084863
KCNJ3	0	0	0	0	0	0	0	0
KCNJ4	0	0	0	0	0	0.331759	0	0.067575
KCNJ5	0	0.092116	0	0.288175	0	0	0	0.128875
KCNJ6	0	0.221743	0	0.151917	0	0	0	0.036718
KCNJ8	0	0	0	0.272149	0	0	0	0
KCNJ9	0	0	0	0.076616	0	0.039218	0	0
KCNK1	0	22.964253	20.549603	21.278768	59.991183	29.407928	42.787784	30.322423
KCNK10	0	0	0	0	0	0	0	0
KCNK12	0	0	0	0	0	0	0	0
KCNK13	0	3.369612	0	1.856757	0	0	0	0
KCNK15	0	0.460221	0	0.427952	0	0.134399	0	0
KCNK16	0	0	0	0	0	0	0	0
KCNK17	0	0.197451	0	0.113809	0	0	0	0
KCNK18	0	0	0	0	0	0	0	0
KCNK2	0	0.932611	5.893958	0.838665	0	0	0	0
KCNK3	0	0.130861	0.471683	0.102652	0	0.052619	0	0.042076
KCNK4	0	0	0	0	0	0	0	0
KCNK5	0.875684	3.972847	3.142674	5.44992	5.087025	11.197189	0.213036	8.618132
KCNK6	14.230233	17.484285	16.882421	17.791343	18.046513	13.879358	12.345771	11.723938
KCNK7	0	0	0	0.069174	0	0.280179	0	0
KCNK9	0	0	0	0	0	0	0	0
KCNMA1	4.195314	8.319911	5.991743	3.529747	8.493617	3.743432	0	8.955254

KCNMB1	0	0.029667	0	0.016894	0	0	0	0
KCNMB2	0	0	0	0	0	0	0	0
KCNMB3	4.84391	0.637619	0	0.654786	0.444073	0.878008	0	0.23707
KCNMB3P1	0	0	0	0	0	0	0	0
KCNMB4	0.69527	2.028357	0.62444	2.225263	0.367301	2.1151	5.248832	0.474351
KCNN1	0	0	0	0	0	0	0	0
KCNN2	0	0.103754	0	0	0	0	0	0
KCNN3	0	0.180003	0.220857	0.043395	0	0.037014	0	0
KCNN4	7.040566	1.498047	3.393915	3.555973	18.317488	5.796688	0	3.418753
KCNQ1	0	4.402988	0	0	0	0	0	1.037617
KCNQ2	0	1.608585	0	0.926183	0	0.183313	0	0.369467
KCNQ3	0	0.19988	0	0.114561	0	0.155725	0	0
KCNQ4	0	0	0	0.048786	0	0.038072	0	0
KCNQ5	5.584672	4.447552	0	5.326475	3.225628	2.965751	2.365163	3.354611
KCNRG	0	3.739013	0	1.363328	0	1.33147	0	1.955349
KCNS1	0	0	0	0	0	0.072433	0	0.261489
KCNS2	0	0	0	0	0	0	0	0
KCNS3	4.382965	7.942726	3.919382	7.754112	8.867206	2.900034	0	5.229371
KCNT1	0	0	0	0	0	0	0	0
KCNT2	0	0	0.862176	0.313147	0	0	0	0
KCNU1	0	0	0	0	0	0.044862	0	0
KCNV1	3.537208	0.752504	1.029216	0.893899	0	0	0	0.056335
KCNV2	0	0	0	0	0	0	0	0
KCP	0	0.556053	1.364807	0.590068	0	1.133387	0	0.516534
KCTD1	29.949954	32.851418	6.801643	20.42152	23.76908	24.049685	49.917798	19.914282
KCTD10	49.685792	30.857306	37.427741	32.746911	184.963876	41.92913	88.720628	65.462008
KCTD10P1	0	0	0	0	0	0	0	0
KCTD11	0	0	0	2.749976	0	0.665419	0	0
KCTD13	8.529464	9.29238	28.363671	14.429514	8.876819	8.242257	0	8.513624
KCTD14	13.285789	6.301384	18.854132	4.206078	6.690277	3.023563	24.683671	4.559909
KCTD15	1.331505	11.433699	2.39212	14.569442	14.890135	44.611575	37.786412	42.170081
KCTD16	0.23691	0.214744	0.213331	0.365625	0	0.107263	0	0.094855
KCTD17	7.862583	6.359832	0	2.233364	27.071405	3.922167	0	0.497515

KCTD18	33.497317	29.121351	4.755816	37.526804	18.155188	47.488846	63.116771	37.621409
KCTD19	0	0	0	0	0	0	0	0
KCTD2	12.984679	20.197412	11.646494	23.850577	21.549528	27.860446	35.927974	16.996752
KCTD20	16.970676	51.972937	31.478943	36.679901	15.883411	57.679733	30.60871	52.352506
KCTD21	11.799855	10.522611	14.107226	16.474463	2.336388	3.188668	0	6.732055
KCTD3	44.377297	81.793298	30.239577	41.476081	55.508798	24.282133	24.998518	36.668349
KCTD5	35.01529	22.082674	49.99917	21.145664	21.799241	24.263169	46.632064	24.209231
KCTD5P1	0	0	0	0	0	0	0	0
KCTD6	12.86931	7.242012	4.091077	6.175532	12.105811	5.726823	13.312511	5.082066
KCTD7	3.675816	3.083862	0.660119	2.09556	2.570607	2.588674	0	1.570267
KCTD8	0	0.834424	0	1.177412	0	0.194925	0	0
KCTD9	18.81222	40.131321	22.194016	31.369882	39.358844	22.006266	75.933023	38.722158
KCTD9P1	0	0	0	0	0	0	0	0
KCTD9P2	0	0	0	0	0	0	0	0
KCTD9P3	0	0	0	0	0	0	0	0
KCTD9P4	0	0	0	0.077475	0	0	0	0
KCTD9P5	0	0	0	0	0	0	0	0
KCTD9P6	0	0	0	0	0	0	0	0
KDELR1	149.938257	81.357588	172.445802	129.936543	151.874005	163.439244	118.018601	146.883224
KDELR2	504.025717	567.006436	582.22046	414.853226	121.134141	147.454004	106.756598	205.529776
KDELR3	33.389128	14.149433	18.59508	14.339869	1.099603	4.428337	0	6.746114
KDF1	0	18.638912	10.093783	16.84867	0	36.220674	0	27.41309
KDM1A	59.744207	127.639715	43.341937	102.569503	93.894574	129.543288	44.627842	175.935584
KDM1B	0	22.895311	3.250687	21.000557	37.462232	32.17371	0	40.910808
KDM2A	79.756693	103.846168	101.075301	117.071889	85.524489	115.612956	51.46384	114.682836
KDM2B	7.396035	19.499994	3.612308	17.676987	14.882135	19.138105	54.397124	20.927143
KDM3A	18.468115	38.034622	22.964226	39.856063	62.50353	18.07799	141.134642	24.003088
KDM3AP1	0	0	0	0	0	0	0	0
KDM3B	31.806623	43.512281	26.588625	47.435203	47.755403	59.785908	158.239215	42.422449
KDM4A	36.492823	33.263491	15.130033	35.951892	28.502257	48.258993	39.954961	40.199482
KDM4B	6.770232	2.671294	9.639052	11.293912	0	9.739622	0	17.587954
KDM4C	0	1.186011	20.226764	7.087208	4.467883	2.924793	7.24138	2.107287
KDM4D	8.640855	28.127371	8.842112	24.690818	2.104193	5.048288	0	6.505507

KDM4E	0	0	0	0	0	0	0	0
KDM5A	24.808861	27.450972	6.672575	43.42304	8.700726	45.272436	123.051061	44.935001
KDM5B	47.851539	33.066842	33.811128	68.829085	110.990303	45.024779	149.294037	56.3605
KDM5C	9.865036	81.32729	4.91771	120.869584	25.683249	184.56954	146.603952	97.892703
KDM5D	1.395466	9.063414	0.722624	5.859516	6.695825	19.973769	23.471416	7.567752
KDM5DP1	0	0	0	0	0	0	0	0
KDM6A	0	0	0	0	6.828108	0	27.231671	0
KDM6B	10.620069	8.911626	48.380057	20.414008	15.562312	55.67685	3.060176	34.253978
KDM7A	1.744024	2.089635	3.452783	4.62	1.960814	4.284024	0	3.966671
KDM8	0	2.604637	1.908199	0.492718	0	4.929824	0	0
KDR	1.700623	0.025499	0	0	0	0	0	0
KDSR	76.181676	116.754262	46.889536	96.208632	87.440883	137.073095	183.079931	221.437054
KEAP1	62.643627	35.857999	46.41813	36.543693	66.365275	39.14697	41.356893	38.822576
KEL	0	0.116033	0	0.204491	0	0	0	0
KGD4	109.01998	30.867601	46.980433	11.299779	10.995425	6.617401	0	21.49326
KHDC1	0	7.201288	2.247762	7.878482	0.444547	7.659176	0	9.31328
KHDC1L	6.360325	3.808807	0	3.310304	8.311169	5.988478	0	4.994369
KHDC1P1	0	0	0	0	0	0	0	0
KHDC3L	0	0	0	0	0	0	0	0
KHDC4	23.78841	24.573162	18.042846	23.405222	24.71143	27.97874	4.686614	10.062197
KHDRBS1	129.596522	99.196304	137.282943	111.314718	144.351168	106.362784	192.443339	94.821478
KHDRBS2	0	0	0	0	0	0	0	0
KHDRBS3	0	2.917343	12.167988	3.106799	0	2.397653	0	3.829376
KHK	0	10.209022	0	4.469467	0	8.545083	0	8.083292
KHNYN	16.122906	49.346633	19.931037	52.969051	43.085953	120.900593	16.682692	69.605721
KHSRP	282.440837	304.987912	290.053087	285.5699	325.654123	336.743918	469.694311	224.958709
KHSRPP1	0	0	0	0	0	0	0	0
KIAA0040	28.887719	92.280003	21.668186	124.927501	59.628614	289.949482	83.396501	281.579916
KIAA0232	6.646683	9.753288	7.100154	5.588026	13.520723	8.28024	7.092782	13.989734
KIAA0319	0	0.105334	0	0.203638	0	0	0	0
KIAA0319L	13.037585	34.996416	11.285542	27.182403	6.921062	24.437855	86.665329	15.784028
KIAA0408	0	0.482576	11.903256	0.581913	0	0	0	1.801776
KIAA0513	0	0	0	0	14.049936	0	0	0

KIAA0586	28.692303	28.768816	30.918354	21.773673	25.788175	13.805521	14.21113	33.605927
KIAA0753	33.916908	20.67111	10.765311	24.692846	25.485887	26.816401	2.985631	16.209254
KIAA0825	1.51652	0.255876	0	0.133996	0.799939	0.07521	0	0
KIAA0895LP1	0	0	0	0	0	0	0	0
KIAA0930	32.69816	57.178544	34.062001	50.869683	79.611325	91.732495	136.45317	61.127426
KIAA1143	21.89115	22.37641	37.59374	27.0603	5.580696	22.248684	71.760996	27.578278
KIAA1143P1	0	22.197384	0	6.064637	96.189227	11.118072	0	5.484622
KIAA1143P2	0	0	0	0	0	0	0	0
KIAA1191	115.741335	82.645579	67.244965	102.769476	73.764752	90.814428	84.427468	69.226583
KIAA1191P1	0	0	0	0	0	0	0	0
KIAA1191P2	0	1.235228	0	0	0	0	0	0
KIAA1191P3	0	0	0	0	0	0	0	0
KIAA1210	0	0	0	0.010096	0	0	0	0
KIAA1217	11.686548	11.296317	4.321268	19.804633	25.132994	28.451672	11.822829	15.359936
KIAA1328	2.262045	3.41838	9.908175	4.292453	3.190629	3.230154	0	4.452596
KIAA1328P1	0	0	0	0	0	0	0	0
KIAA1549	0	0.334051	0	0.162611	1.348853	0.545373	0	0.685935
KIAA1549L	2.205234	1.676802	1.761543	1.507517	3.652657	1.498258	0	0.492236
KIAA1586	6.048854	2.163781	0	3.451443	6.065711	3.510368	18.713725	8.684268
KIAA1614	0.645731	0.556806	0.290589	0.51337	0.266231	1.172939	0	0.103462
KIAA1671	4.962204	7.800683	3.91206	11.90493	7.436979	55.871801	15.479443	35.444107
KIAA1755	0	1.038855	0	1.4292	0	0.643899	0	1.062952
KIAA1958	3.226384	10.474772	7.869726	12.57736	4.157721	18.851371	0.102964	16.948852
KIAA2012	0	0	0	0	0	0	0	0
KIAA2013	22.723991	27.234556	16.066609	21.245813	8.848763	53.938107	0	44.954233
KICS2	13.408425	18.679205	11.122321	11.841957	4.061051	15.019486	0	16.843689
KIDINS220	12.492826	8.708014	25.832772	19.7786	16.782294	16.593897	41.34797	20.897795
KIF11	43.700838	43.473873	35.156834	20.673768	51.001145	23.846538	36.383382	49.33204
KIF12	0	0	0	0.580879	0	0	0	0
KIF13A	25.780667	34.042611	17.401696	25.233809	35.956016	78.534386	50.966722	61.972153
KIF13B	10.340685	3.712886	15.448291	7.716837	4.506754	6.224987	17.043127	3.814958
KIF14	23.060597	25.385796	21.139895	13.400623	20.743484	9.214822	20.557944	30.160796
KIF15	13.774754	11.768724	12.371923	9.496158	18.367389	13.179323	32.639272	17.37616

KIF16B	11.135109	5.673478	11.792282	11.405505	16.023608	16.08683	0	7.466578
KIF17	1.860429	0.278495	0	0.610852	0	0.562852	0	0.299016
KIF18A	11.697673	9.083568	13.11163	4.351334	30.882562	12.742693	27.221457	20.484044
KIF18B	12.503352	7.580816	6.706347	7.207686	14.653256	10.332926	19.667867	9.55456
KIF18BP1	0	0	0	0	0	0	0	0
KIF19	0	0	0	0	0	0.182696	0	0
KIF19BP	0	0	0	0	0	0	0	0
KIF1A	5.930403	0.819227	1.260271	3.023898	0	1.638235	0	0.15871
KIF1B	18.682085	21.503951	11.2799	25.180515	15.059803	33.115175	53.812915	37.309778
KIF1C	83.781823	112.624005	27.499028	117.070515	73.210325	265.534994	52.499452	181.406254
KIF20A	43.304769	45.73347	29.10572	32.690335	118.646664	63.650884	41.184186	52.984014
KIF20B	27.800331	7.171052	12.496928	3.827725	34.552044	4.260157	41.764681	9.647895
KIF21A	12.010322	8.817696	13.928861	15.417146	22.370456	8.861867	103.311171	16.949019
KIF21B	0.605513	0.421106	1.08821	0.662424	0	0.394012	0	0.308597
KIF22	0	0.417663	0	0	0	0	0	1.36761
KIF23	63.788263	87.834311	46.277139	49.932882	142.520205	74.530052	73.463567	105.827995
KIF24	3.410329	0	0	0.449563	0	0	2.216273	0
KIF25	0	0	0	0	0	0	0	0
KIF26A	0.850436	2.533382	1.736045	1.936306	0	1.791838	0	0.708512
KIF26B	0	2.622928	0.237883	1.567183	0.069847	0.973858	0	0.574078
KIF27	1.495085	2.819878	4.027127	2.651018	0.942212	1.611374	2.365911	3.51355
KIF28P	0	0	0	0	0	0	0	0
KIF2A	20.984831	46.28304	27.39531	38.036591	22.936576	21.289633	0	31.530635
KIF2C	46.381748	36.983791	43.886662	39.56224	50.932789	72.797132	92.641804	72.740915
KIF3A	4.522581	24.316188	8.13947	19.350921	66.839984	16.573368	0	5.347178
KIF3AP1	0	0	0	0.346781	0	0	0	0
KIF3B	145.047459	188.358195	141.855582	180.296766	43.097929	61.748656	41.442568	65.947565
KIF3C	7.943396	11.493631	9.333772	21.844831	11.028398	43.176784	4.090457	28.421339
KIF4A	35.984461	28.964149	26.923816	28.11222	40.194319	48.733597	33.216892	46.517813
KIF4CP	0	0	0	0	0	0	0	0
KIF5A	0.942734	1.026762	1.012605	1.393054	0	1.216339	0	0.497019
KIF5B	87.44243	40.483972	37.891726	31.078938	20.517567	28.392792	117.595195	64.444578
KIF5C	1.353519	0.97765	0.607879	1.090386	0.17094	4.115021	0	4.553247

KIF6	15.803609	3.01764	0	1.187276	0	0.437107	0	0
KIF7	1.438125	4.194217	6.457214	3.847303	2.848902	3.382011	0	2.30891
KIF9	14.019401	13.770725	6.076953	9.627049	0	3.722905	0	10.185575
KIFAP3	15.928069	12.956817	16.307997	6.838743	12.010967	4.986229	40.62717	10.943809
KIFBP	29.263075	19.245954	24.75244	16.220665	36.130016	18.257147	0	24.914605
KIFC1	30.71756	36.528798	22.987934	38.055278	51.643822	77.95708	31.166942	50.706579
KIFC2	3.086705	1.933746	1.844666	2.139475	0.543218	2.770434	0.250095	1.571806
KIFC3	30.514132	54.589352	48.368479	60.463289	41.327463	112.595909	68.08446	73.110597
KIN	1.02064	0.395056	10.76007	2.674356	0.269687	4.069189	0	4.919778
KIR2DL1	0	0.757759	0	0	0	0	0	0
KIR2DL3	0	0	0	0	0	0	0	0
KIR2DL4	0	0	0	0	0	0	0	0
KIR2DL5A	0	0	0	0	0	0	0	0
KIR2DL5B	0	0	0	0	0	0	0	0
KIR2DP1	0	0	0	0	0	0	0	0
KIR2DS1	0	0	0	0	0	0	0	0
KIR2DS2	0	0	0	0	0	0	0	0
KIR2DS3	0	0	0	0	0	0	0	0
KIR2DS4	0	0	0	0	0	0	0	0
KIR2DS5	0	0	0	0	0	0	0	0
KIR3DL1	0	0	0	0	0	0	0	0
KIR3DL2	0	0	0	0	0	0	0	0
KIR3DL3	0	0	0	0	0	0	0	0
KIR3DP1	0	0	0	0	0	0	0	0
KIR3DS1	0	0	0	0	0	0	0	0
KIR3DX1	0	0	0	0	0	0	0	0
KIRREL1	22.562206	12.405036	24.977079	14.991245	12.60758	20.731988	0	15.444521
KIRREL2	0	0	4.724777	0.468676	0	0	0	0.284964
KIRREL3	0	0	0	0	0	0.084979	0	1.468232
KISS1	0	0.2384	0	1.282704	0	0	0	0
KISS1R	9.710741	1.841314	3.89752	1.696123	1.060235	3.662558	2.253734	2.232454
KIT	0	0	0	0	0	0	0	0
KITLG	59.373509	111.374771	51.180474	90.562074	23.452247	36.678353	17.230917	56.560475

KIZ	33.0148	62.789539	39.336387	60.445983	20.211051	19.344124	55.593602	25.034757
KL	0	0	0	0	0	0	0	0
KLB	0	0	0	0	0	0	0	0
KLC1	45.660232	146.136581	53.827382	128.577788	102.852216	155.048574	179.260986	153.216674
KLC2	25.83187	30.412468	1.371463	39.400146	35.235013	54.788628	59.262173	22.619195
KLC3	5.915792	8.07022	3.556147	8.309794	2.627695	18.366365	0	10.67452
KLC4	0	6.456804	13.576371	9.466671	0	9.47975	22.303039	5.628098
KLF10	61.160093	49.209804	58.921343	46.878955	30.759798	22.926679	68.111894	38.194378
KLF11	2.554659	7.234504	4.584735	10.913219	0.224866	17.345659	0	12.756379
KLF12	0	3.115041	7.354221	6.085684	0	2.273162	0	2.967194
KLF13	44.001975	28.946087	12.350538	30.826767	9.059552	43.21785	12.887444	30.991468
KLF15	0	0	0	0.260472	0.705798	1.528786	0	0.969465
KLF16	16.238729	20.080546	19.740969	24.428964	17.142615	34.030431	10.705759	23.330712
KLF17	0	0	0	0	0	0	0	0
KLF17P1	0	0	0	0	0	0	0	0
KLF18	0	0.271383	0	0	0	0	0	0
KLF2	0	0	0	0	0	5.865025	0	2.37833
KLF2P1	0	0	0	0	0	0	0	0
KLF2P2	0	0	0	0	0	0	0	0
KLF2P3	0	0	0	0	0	0	0	0
KLF2P4	0	0	0	0	0	0	0	0
KLF3	27.806335	19.12363	22.512966	23.116081	19.39315	19.258659	28.108467	24.297483
KLF3P1	0	0.155018	0	0	0	0	0	0
KLF3P2	0	0	0	0	0	0	0	0
KLF4	18.651376	78.678186	28.12639	98.382503	26.342191	85.724352	11.621977	97.121229
KLF4P1	0	0	0	0	0	0	0	0
KLF5	84.132555	123.68124	135.895543	209.852862	148.025405	200.902314	162.595468	211.843293
KLF6	17.882733	17.327398	7.066011	21.79115	20.016115	59.250719	91.602577	67.717019
KLF7	35.444745	58.731438	25.996533	112.329873	21.721196	114.083203	49.896832	110.479104
KLF7P1	0	0.150571	0	0.175178	0	0	0	0.148589
KLF8	2.718712	5.403496	1.909487	4.501539	9.675344	6.25213	0	3.71346
KLF8P1	0	0	0	0	0	0	0	0
KLHDC1	0	2.493582	5.804863	0.398905	0	0	0	0.391517

KLHDC10	12.172785	14.08241	15.048554	18.11916	45.92817	7.561542	28.023194	10.893595
KLHDC2	62.938493	57.645218	42.682088	45.925991	19.447097	31.947326	64.941789	65.962832
KLHDC3	33.662526	59.72862	45.048709	66.993955	5.947968	85.168184	44.790571	77.29087
KLHDC4	38.796693	37.347605	88.090473	49.06645	33.327495	57.510117	5.555463	34.186891
KLHDC7B	0	0	0	0.103733	0	0	0	0
KLHDC8A	0	0	0	0	0	0	0	0
KLHDC8B	0	1.573827	0	2.40602	3.927866	3.050908	0	1.920253
KLHDC9	0	7.437457	6.935756	4.75467	0	1.250606	0	3.705961
KLHL1	0	0	0	0	0	0	0	0
KLHL10	0	0	0	0	0	0	0	0
KLHL11	13.099607	9.417638	16.888151	7.286732	16.385967	5.391688	14.37647	11.060513
KLHL12	61.879639	70.613268	43.981954	59.290362	46.353004	39.395171	24.688755	58.267148
KLHL12P1	0	0	0	0	0	0	0	0
KLHL13	17.28036	23.318131	21.687877	10.871606	5.701538	21.339666	5.586541	19.5157
KLHL14	0	0	0	0.179943	0	0	0	0
KLHL15	4.141567	3.317128	3.257984	2.836132	5.881996	3.920217	9.969754	10.744261
KLHL17	0	0.450164	7.200564	0.973802	0	1.971811	0	1.523432
KLHL18	7.546532	29.459735	8.290776	13.556705	31.112845	32.025478	11.836926	33.00617
KLHL2	19.266765	69.554833	16.230441	58.493769	7.811973	34.066318	0.481852	35.8813
KLHL20	10.732825	13.503641	16.935568	30.354567	15.964623	26.647149	0	28.362556
KLHL21	20.508038	19.129622	17.926085	16.783192	20.520589	56.738364	48.897755	33.890974
KLHL22	4.03336	13.389234	12.501048	9.266241	5.431415	19.163988	39.807131	14.555772
KLHL23	25.078838	23.774145	15.243304	32.6701	28.817994	31.601836	20.705754	42.726183
KLHL24	8.676874	30.214196	25.395201	43.966042	30.761576	17.041606	47.457006	56.170721
KLHL25	0.909283	7.585599	4.893945	5.480574	4.321535	6.775783	0	5.151012
KLHL25P1	0	0	0	0	0	0	0	0
KLHL26	3.025329	2.301986	0.679046	3.23893	0.998785	6.234732	13.625168	3.522118
KLHL28	8.033493	9.195643	4.098303	8.518755	4.074479	5.187951	0	15.122861
KLHL29	8.582711	22.24099	6.610254	18.796701	2.267406	11.365603	0.448091	7.834172
KLHL2P1	13.741655	0	0	2.586564	0	0.582391	0	0.935635
KLHL3	2.856139	9.085782	3.852564	11.687789	0.652476	4.636101	12.072784	4.030193
KLHL30	1.75283	0.337595	0	0.727259	0	0.131325	0	0.281644
KLHL31	0	0	0	0.224532	0	0	0	0.14783

KLHL32	0	0	0	0.246086	0	0	0	0
KLHL33	0	0	0	0.045542	0	0	0	0.048439
KLHL34	0	0	0	0	0	0	0	0
KLHL35	0	7.629096	2.129849	5.522031	1.260936	4.46891	0	2.520362
KLHL36	22.222644	55.999104	26.687752	50.869139	18.991409	116.983721	25.474552	86.055706
KLHL38	0	0	0	0	3.696323	0.346774	0	0
KLHL4	1.393799	5.360551	1.246852	1.865365	0	0.138436	0	0.224552
KLHL41	0	0.058867	0	0	0	0	0	0
KLHL42	8.996403	15.809513	7.190131	11.376288	5.650576	19.246615	0	12.375436
KLHL5	71.914807	59.183019	56.904564	57.214956	44.295744	35.548471	36.411138	32.398368
KLHL5P1	0	0	0	0	0	0	0	0
KLHL6	0	0	0	0	0	0	0	0
KLHL7	68.279891	118.024352	41.619693	78.699574	28.854476	111.155164	202.354014	170.470016
KLHL8	12.424931	20.637813	13.28996	37.358766	9.085871	26.118636	25.34093	24.195567
KLK1	21.988199	21.069992	42.400924	26.17449	0	4.20117	0	2.312525
KLK10	9.004249	11.911963	15.07008	15.965559	13.070821	11.220365	57.631517	13.433836
KLK11	0	10.798587	10.015545	13.069773	1.670196	8.224761	0	1.678397
KLK12	0	0	0	0	0	0	0	0
KLK13	15.32567	8.426208	3.435775	5.239577	0	0	0	0
KLK14	0	0	0	0	0	0	0	0
KLK15	0	0	0	0	0	0	0	0
KLK2	0	0.117918	0	0	1.343748	0.062943	4.345981	0.944594
KLK3	0	0	0	0	0	0	0	0
KLK4	0	0.248377	0	0	0	0	0	0
KLK5	82.578857	70.671832	39.345836	47.518428	7.421647	1.514276	0	1.439662
KLK6	4.870106	4.740013	0	3.566323	0	1.223057	0	0.75212
KLK7	5.470842	5.431952	0	1.942634	0	0.526507	0	0.646284
KLK8	0	0	0	0	0	0	0	0
KLK9	0	0	0	0.312844	0	0.126896	0	1.230027
KLKB1	0	0	0	0	0	0	0	0
KLKP1	0	0	0	0	0	0	0	0
KLLN	0	0	0	0	0	0	0	0
KLRA1P	0	0	0	0	0	0	0	0

KLRC1	0	0	0	0.267367	0	0	0	0
KLRC2	0	0	0	0	0	0	0	0
KLRC3	0	0	0	0	0	0	0	0
KLRC4-KLRK1	0	0	0	0	0	0	0	0
KLRD1	0	0	0	0	0	0.052699	0	0
KLRF1	0	0	0	0	0	0	0	0
KLRF2	0	0	0	0	0	0	0	0
KLRG1	0	1.428849	0	0.370198	0	0	0	0
KLRG2	0	0	2.892983	0	0	0	0	0
KLRK1	0	0.095147	0	0	0	0	0	0
KMO	5.715595	0.107808	0	0.868945	0.177558	0.689749	36.910271	0.615323
KMT2A	92.351028	72.71418	91.30833	116.436578	31.718071	22.444975	6.66672	17.32903
KMT2B	0	0	0	0.075229	6.508789	0	0	0
KMT2C	6.908025	5.535066	4.860319	6.792357	0	7.106432	0.000127	6.408278
KMT2CP1	0	0	0	0	0	0	0	0
KMT2CP2	0	0	0	0	0	0	0	0
KMT2CP3	0	0	0	0	0	0	0	0
KMT2CP4	0	0	0	0	0	0	0	0
KMT2CP5	0	0	0	0	0	0	0	0
KMT2D	0	0	0	0	0	0	0	0
KMT2E	18.724245	12.47658	0	17.335967	16.25258	20.147309	0	19.268094
KMT5A	28.855735	57.154751	38.760304	60.694199	24.063395	106.982322	24.503426	72.735752
KMT5AP1	0	0	0	0	0	0.895144	0	0
KMT5AP2	0	0	0	0	0	0	0	0
KMT5AP3	0	0	0	0	0	0	0	0
KMT5B	43.502714	132.996116	72.228471	157.695118	34.990009	99.088166	114.941032	86.518549
KMT5C	0	8.233838	3.037377	10.291715	12.520241	14.129557	0	5.108557
KNCN	0	0	0	0	0	0	0	0
KNDC1	0	0.128324	0	0.547247	0.121819	0.097582	0	0
KNG1	0	0	0	0	0	0	0	0
KNL1	2.775763	11.495985	7.341447	7.794288	28.47029	10.448978	19.312142	15.145155
KNOP1	42.117425	70.566975	48.383667	86.61842	19.652774	87.391538	99.993959	57.477767
KNOP1P1	0	0	0	0	0	0	0	0

KNOP1P2	0	0	0	0	0	0	0	0
KNOP1P3	0	0	0	0	0	0	0	0
KNOP1P4	0	0	0	0.1215	0	0	0	0
KNOP1P5	0	0	0	0	0	0	0	0
KNSTRN	81.089502	130.775578	64.135789	108.991223	114.181852	156.508661	116.638074	160.156312
KNTC1	21.220508	17.137081	24.736268	16.020065	13.860714	5.731514	33.405403	17.272764
KPLCE	0	0.338063	0	0	0	0	0	0.168216
KPNA1	91.795398	140.993661	63.615709	109.435755	56.353412	146.745504	102.918921	200.226573
KPNA2	256.597806	205.997574	142.778211	111.053875	345.411241	168.121684	299.852527	292.416057
KPNA2P1	0	0	0	0	0	0	0	0
KPNA2P2	0	0	0	0	0	0	0	0
KPNA2P3	0	0.833625	4.164676	0.232334	0	0.467794	0	0.744296
KPNA3	27.317219	36.340544	28.726258	12.177259	19.994487	10.108448	37.814397	27.247633
KPNA4	47.090027	58.9719	46.255281	38.33	60.466304	55.180368	24.867566	66.781428
KPNA4P1	0	0	0	0.060845	0.662536	0	0	0
KPNA5	10.745385	6.81258	5.728833	3.294007	25.952598	0.943913	0	2.471308
KPNA6	57.424374	58.602007	35.183056	69.002413	42.626972	80.877023	50.378625	99.883959
KPNB1	295.538766	468.108217	271.15726	531.607971	396.168527	580.835506	570.763649	620.801726
KPNB1P1	0	0	0	0	0	0	0	0
KPTN	30.761902	10.258139	11.160018	7.699691	4.037678	16.619682	0	19.684087
KRASP1	0	0	0	0	0	0	0	0
KRBA1	13.115739	5.192621	7.923411	2.613984	4.6082	5.325518	0	2.2531
KRBA2	0	0.816935	0	0.941353	0	1.507017	0	1.254194
KRBOX1	0	0	0	0	0	0	0	0
KRBOX4	11.922502	16.087216	3.932035	13.073283	0.779646	18.854083	0	23.949109
KRBOX5	3.330919	34.302971	36.337492	18.859749	0	10.759707	4.892101	20.568811
KRBOX5P1	0	0	0	0	0	0	0	0
KRCC1	1.941564	6.39204	6.925972	7.53531	2.046675	4.504431	0	6.587409
KREMEN1	24.449352	0.736414	0	9.598011	0	8.280186	0	4.388174
KREMEN2	0	9.606	10.446236	11.032121	0	17.650651	12.746964	12.535687
KRI1	12.811237	25.840388	21.019794	35.805789	38.667162	38.599123	26.251025	30.271063
KRIT1	1.965645	22.724423	0	3.417611	0.498807	1.621752	1.859104	5.936347
KRR1	55.942653	70.216778	94.85847	46.531139	78.356829	25.882603	108.855152	40.107875

KRR1P1	0	0	0	0	0	0	0	0
KRT1	0	0	0	0	0	0	0	0.223954
KRT10	3.921688	20.748763	7.472266	19.968045	3.139705	23.180903	0	19.012553
KRT10-AS1	0	0	18.232847	11.943547	23.179991	0	30.90869	0
KRT12	0	0	0	0	0	0	0	0
KRT125P	0	0	0	0	0	0	0	0
KRT126P	0	0	0	0	0	0	0	0
KRT127P	0	0	0	0	0	0	0	0
KRT128P	0	0	0	0	0	0	0	0
KRT13	0	2.07666	0	0.249619	95.536818	53.112123	32.849772	22.742405
KRT14	17.361241	5.752741	20.700529	8.690192	438.033172	546.40943	412.665819	322.951747
KRT15	30.93366	46.076458	25.379037	61.001971	14.571347	41.275191	17.856367	21.546473
KRT16	0	0.186684	3.808727	1.198875	15.636154	28.896781	23.813574	23.022962
KRT16P1	0	0	0	0	0	0	0	0
KRT16P2	0	0	0	0	0	0.124804	0	0
KRT16P3	0	0	0	0	0	0	0	0.319036
KRT16P4	0	0	0	0	0	0	0	0
KRT16P5	0	0	0	0	0	0.425015	0	0
KRT16P6	0	0	0	0	0	0	0	0
KRT17	284.660158	250.27449	250.763763	311.297313	2385.29565	2738.16169	1487.21873	1487.75645
KRT17P1	0	0	0	0	0	0	0	0
KRT17P2	0	0	0	0	0	0	0	0
KRT17P3	0	0	0	0	0.77012	0.146734	0	0
KRT17P4	0	0	0	0	0	0	0	0
KRT17P5	0	0	0	0	0	0	0	0
KRT17P6	0	0	0	0	0	0.137366	0	0
KRT17P7	0	0	0	0	0	0	0	0
KRT17P8	0	0	0	0	0	0	0	0
KRT18	740.937285	659.779499	542.258008	493.432489	511.661124	512.594121	409.00933	362.069037
KRT18P1	0	0	0	0	0	0	0	0
KRT18P10	0	0	0	0	0.751532	0	0	0
KRT18P11	0	0	0	0	0	0	0	0
KRT18P12	0	0	0	0	0	0	0	0

KRT18P13	0	0	0	0	0	0	0	0
KRT18P14	0	0	0	0	0	0	0	0
KRT18P15	0	0	0	0	0	0	0	0
KRT18P16	0	0	0	0	0	0	0	0
KRT18P17	0	0	0	0	0	0.282245	0	0.352827
KRT18P18	0	0	0	0	0	0	0	0
KRT18P19	0	0	0	0	0	0	0	0
KRT18P2	0	0	0	0	0	0	0	0
KRT18P20	0	0	0	0	0	0	0	0
KRT18P21	0	0.120734	0	0	0	0	0	0
KRT18P22	0	0	0	0	0	0	0	0
KRT18P23	0	0	0	0	0	0	0	0
KRT18P24	0	0	0	0	0	0	0	0
KRT18P25	0	0	0	0	0	0	0	0
KRT18P26	0	0	0	0	0	0	0	0
KRT18P27	0	0	0	0	0	0	0	0
KRT18P28	0	0	0	0.066294	0	0	0	0
KRT18P29	0	0.121285	0	0	0	0	0	0
KRT18P3	0	0	0	0.068866	0	0.83685	0	0.115821
KRT18P31	0	0.474541	0	0.068622	0	0	0	0
KRT18P32	0	0	0	0	0	0	0	0
KRT18P33	0	0	0	0	0	0	0	0
KRT18P34	0	0	0	0	0	0	0	0
KRT18P35	0	0	0	0	0	0	0	0
KRT18P36	0	0	0	0	0	0	0	0
KRT18P37	0	0	0	0	0	0	0	0
KRT18P38	0	0	0	0	0	0	0	0
KRT18P39	0	0	0	0	0	0	0	0
KRT18P4	0	0	0	0.137845	0	0.279116	0	0
KRT18P40	0	0	0	0	0	0	0	0
KRT18P41	0	0	0	0	0	0	0	0
KRT18P42	0	0	0	0	0	0	0	0
KRT18P43	0	0	0	0	0	0	0	0

KRT18P44	0	0	0	0	0	0	0	0
KRT18P45	0	0	0	0	0	0	0	0
KRT18P46	0	0	0	0	0	0	0	0
KRT18P47	0	0	0	0	0	0	0	0
KRT18P48	0	0	0	0	0	0	1.122859	0
KRT18P49	0	0.197668	0	0	0	0	0	0
KRT18P5	0	0	0	0.412095	0	0	0	0.346506
KRT18P50	0	0	0	0	0	0	0	0
KRT18P51	0	0	0	0	0	0	0	0
KRT18P52	0	0	0	0.113571	0	0	0	0
KRT18P53	0	0	0	0	0	0	0	0
KRT18P54	0	0	0	0	0	0	0	0
KRT18P56	0	0	0	0	0	0	0	0
KRT18P57	0	0	0	0	0	0	0	0
KRT18P58	0	0	0	0	0	0	0	0
KRT18P59	0	0	0	0	0	0	0	0
KRT18P6	0	0	0	0	0	0	0	0
KRT18P60	0	0	0	0	0	0	0	0
KRT18P61	0	0	0	0	0	0	0	0
KRT18P62	0	0	0	0	0	0	0	0
KRT18P63	0	0	0	0	0	0.191343	0	0
KRT18P64	0	0	0	0	0	0	0	0
KRT18P65	0	0	0	0	0	0	0	0
KRT18P66	0	0	0	0	0	0	0	0
KRT18P67	0	0	0	0	0	0	0	0
KRT18P68	0	0	0	0.092626	0	0	0	0
KRT18P7	0	0	0	0	0	0	0	0
KRT18P8	0	0	0	0	0	0	0	0
KRT18P9	0	0	0	0	0	0	0	0
KRT19	399.73934	310.204227	382.996978	339.561001	608.466971	437.239821	423.617228	275.160056
KRT19P1	0	2.485128	2.785248	0.833354	6.614523	2.762197	0	0.383473
KRT19P2	0	0	0	0	0	0	0	0
KRT19P3	0	0	0	0	0	0	0	0

KRT19P4	0	0	0	0	0	0	0	0
KRT19P6	0	0	0	0	0	0	0	0
KRT2	0	0	0	0	4.402524	0.069068	0	0
KRT20	0	0	1.808624	0	0	0	0	0
KRT222	0	0.189622	0	0.489712	0	0.761958	0	0
KRT223P	0	0	0	0	0	0	0	0
KRT224P	0	0	0	0	0	0	0	0
KRT23	0	3.401973	2.192279	9.208703	0	0.345831	0	0.714948
KRT24	0	0	0	0	0	0	0	0
KRT27	0	0	0	0	0	0	0	0
KRT3	0	0	0	0	0	0.146471	0	0
KRT31	0	2.29396	0	1.953204	0	0	0	0
KRT33A	0	0	0	0.206782	0	0.55839	0	0
KRT33B	0	0.279401	0	0.796768	0	0.217955	0	0.089567
KRT34	2.035607	0	0	0	0	0	0	1.06938
KRT36	0	0	0	0	0	0	0	0
KRT37	0	0	0	0	0	0	0	0
KRT39	0	0	0	0	0	0	0	0
KRT4	0	0	0	0.078268	14.050755	2.552857	0	1.688155
KRT40	0	0	0	0	0	0	0	0
KRT42P	0	0	0	0	0	0	0	0
KRT43P	0	0	0	0	0	0	0	0
KRT5	613.980318	349.433804	252.336278	250.479059	1905.11738	1779.86557	1435.37915	1128.57674
KRT6A	28.16665	17.74091	18.572183	14.966368	92.74592	67.173592	422.106461	62.246164
KRT6B	0	0.815768	0	0.376849	0	0	0	0.250586
KRT6C	0	0	0	0	0	0	0	0
KRT7	585.61067	625.005528	579.602796	736.039017	0	11.012291	3.326013	6.48395
KRT72	0	0	0	0	0	0	0	0
KRT73	0	0	0	0	0	0	0	0
KRT74	0	0	0	0	0	0	0	0
KRT75	0	0.134567	0	0.269977	0	0.31453	0	1.087547
KRT76	0	0	0	0	0	0	0	0
KRT77	0	0	0	0	0	0	0	0

KRT78	0	0	0	0	0	0	0	0
KRT79	0	0	0	0	0	0	0	0
KRT8	636.932726	532.324698	265.414458	369.562874	582.60077	613.212955	684.517989	433.105689
KRT80	55.216689	116.628024	98.244146	178.873435	38.111648	97.584385	30.797299	68.9659
KRT81	0	2.701395	8.058504	3.28376	0	0.714188	0	0.218667
KRT83	0	0	0	0.316558	0	0	0	0.075238
KRT84	0	0	0	0	0	0	0	0.06661
KRT85	0	0	0	0	0	0	0	0
KRT86	1.652477	1.233649	0	2.091563	0	0.245489	0	0.133291
KRT87P	0	0	0	0	0	0	0	0
KRT89P	0	0	0	0	0	0	0	0
KRT8P1	0	0	0	0	0	0	0	0
KRT8P10	0	0	0	0	0	0	0	0
KRT8P11	0	0	0	0	0	0	0	0
KRT8P12	3.447076	15.551793	13.847993	16.164642	3.418645	21.963448	13.143219	20.051986
KRT8P13	0	0.15716	3.36578	0	0	0	0	0
KRT8P14	0	0	0	0	0	0	0	0
KRT8P15	0	0	0	0.360813	0	0	0	0
KRT8P17	0	0	0	0	0	0	0	0
KRT8P18	0	0	0	0	0	0	0	0
KRT8P19	0	0	0	0	0	0	0	0
KRT8P2	0	0	0	0	0	0	0	0
KRT8P20	0	0	0	0	0	0	0	0
KRT8P21	0	0	0	0	0	0	0	0
KRT8P22	0	0	0	0	0	0	0	0
KRT8P23	0	0	0	0	0	0	0	0
KRT8P24	0	0	0	0	0	0	0	0
KRT8P25	0	0	0	0	0	0	0	0
KRT8P26	0	0	0	0	0	0	0	0
KRT8P27	0	0	0	0	0	0	0	0
KRT8P28	0	0	0	0	0	0	0	0
KRT8P29	0	0	0	0	0	0	0	0
KRT8P3	0	0	0	0	0	0	0	0.204577

KRT8P30	0	0	0	0	0	0	0	0
KRT8P31	0	0	0	0	0	0	0	0
KRT8P32	0	0	0	0	0	0	0	0
KRT8P33	0	0.107408	0	0.928519	0	1.133651	0	1.351381
KRT8P34	0	0	0	0	0	0	0	0
KRT8P35	0	0	0	0	0	0	0	0
KRT8P36	0	0.315511	0	0.364026	0	0	0	0.609759
KRT8P37	0	0	0	0	0	0	0	0
KRT8P38	0	0	0	0	0	0	0	0
KRT8P39	0	0.105682	0	0.304947	0	0.371206	0	0.30661
KRT8P4	0	0	0	0	0	0	0	0
KRT8P40	0	0	0	0	0	0	0	0
KRT8P41	0	0	0	0	0	0	0	0
KRT8P42	0	0	0	0	0	0	0	0
KRT8P43	0	0	0	0	0	0	0	0
KRT8P44	0	0	0	0	0	0	0	0
KRT8P45	0	0	0	0.243518	0	0	0	0
KRT8P46	0	0.850593	4.509791	2.146481	7.346286	2.130435	0	0.205573
KRT8P47	0	0	0	0	0	0	0	0
KRT8P48	0	0	0	0	0	0	0	0.247152
KRT8P49	0	0	0	0	0	0	0	0
KRT8P5	0	0	0	0	0	0.121542	0	0
KRT8P50	0	0	0	0	0	0	0	0
KRT8P51	0	0	0	0	0	0	0	0
KRT8P52	0	0	0	0.136758	0	0	0	0.229951
KRT8P6	0	0	0	0	0	0	0	0
KRT8P7	0	0	0	0	0	0	0	0
KRT8P8	0	0	0	0.06032	0	0.122401	0	0
KRT8P9	0	0	0	0	0	0.122213	0	0
KRT9	0	0.908911	0	0.760674	4.436765	6.044976	0	2.970307
KRTAP1-1	0	0	0	0	0	0	0	0
KRTAP1-3	0	0	0	0	0	0	0	0
KRTAP1-4	0	0	0	0	0	0	0	0

KRTAP1-5	0	0	0	0	0	0	0	0
KRTAP10-1	0	0	0	0	0	0	0	0
KRTAP10-10	0	0	0	0	0	0	0	0
KRTAP10-11	0	0	0	0	0	0	0	0
KRTAP10-12	0	0	0	0	0	0	0	0
KRTAP10-13I	0	0	0	0	0	0	0	0
KRTAP10-2	0	0	0	0	0	0	0	0
KRTAP10-4	0	0	0	0	0	0	0	0
KRTAP10-5	0	0	0	0	0	0	0	0
KRTAP10-6	0	0	0	0	0	0	0	0
KRTAP10-7	0	0	0	0	0	0	0	0
KRTAP10-8	0	0	0	0	0	0	0	0
KRTAP10-9	0	0	0	0	0	0	0	0
KRTAP12-1	0	0	0	0	0	0	0	0
KRTAP12-2	0	0	0	0	0	0	0	0
KRTAP12-3	0	0	0	0	0	0	0	0
KRTAP12-4	0	0	0	0	0	0	0	0
KRTAP12-5P	0	0	0	0	0	0	0	0
KRTAP13-5P	0	0	0	0	0	0	0	0
KRTAP13-6P	0	0	0	0	0	0	0	0
KRTAP16-1	0	0	0	0	0	0	0	0
KRTAP17-1	0	0	0	0	0	0	0	0
KRTAP19-10I	0	0	0	0	0	0	0	0
KRTAP19-11I	0	0	0	0	0	0	0	0
KRTAP19-2	0	0	0	0	0	0	0	0
KRTAP19-6	0	0	0	0	0	0	0	0
KRTAP19-7	0	0	0	0	0	0	0	0
KRTAP19-8	0	0	0	0	0	0	0	0
KRTAP19-9P	0	0	0	0	0	0	0	0
KRTAP2-1	0	0	0	0	0	0	0	0
KRTAP2-2	0	0	0	0	0	0	0	0
KRTAP2-3	0	0.930412	0	0.217864	2.390991	8.529785	1.086464	11.19938
KRTAP2-4	0	0	0	0	0	0	0	0

KRTAP2-5P	0	0	0	0	0	0	0	0
KRTAP20-1	0	0	0	0	0	0	0	0
KRTAP20-3	0	0	0	0	0	0	0	0
KRTAP20-4	0	0	0	0	0	0	0	0
KRTAP21-1	0	0	0	0	0	0	0	0
KRTAP21-3	0	0	0	0	0	0	0	0
KRTAP21-4P	0	0	0	0	0	0	0	0
KRTAP22-1	0	0	0	0	0	0	0	0
KRTAP22-2	0	0	0	0	0	0	0	0
KRTAP25-1	0	0	0	0	0	0	0	0
KRTAP27-1	0	0	0	0	0	0	0	0
KRTAP29-1	0	0	0	0.179656	0	0	0	0
KRTAP3-1	0	0	0	0.18025	0	0	0	0
KRTAP3-2	0	0	0	0	0	0	0	0
KRTAP3-3	0	0	0	0	0	0	0	0
KRTAP3-4P	0	0	0	0	0	0	0	0
KRTAP4-11	0	0	0	0	0	0	0	0
KRTAP4-12	0	0	0	0	0	0	0	0
KRTAP4-16	0	0	0	0	0	0	0	0
KRTAP4-17P	0	0	0	0	0	0	0	0
KRTAP4-8	0	0	0	0	0	0	0	0.133483
KRTAP4-9	0	0	0	0	0	0	0	0
KRTAP5-1	0	0	0	0	0	0	0	0
KRTAP5-11	0	0	0	0	0	0	0	0
KRTAP5-13P	0	0	0	0	0	0	0	0
KRTAP5-14P	0	0	0	0	0	0	0	0
KRTAP5-2	0	0	0	0	0	0	0	0
KRTAP5-3	0	0	0	0	0	0	0	0
KRTAP5-4	0	0	0	0	0	0	0	0
KRTAP5-5	0	0	0	0	0	0	0	0
KRTAP5-6	0	0.650491	0	0	0	0	0	0
KRTAP6-1	0	0	0	0	0	0	0	0
KRTAP6-2	0	0	0	0	0	0	0	0

KRTAP6-3	0	0	0	0	0	0	0	0
KRTAP7-1	0	0	0	0	0	0	0	0
KRTAP8-2P	0	0	0	0	0	0	0	0
KRTAP8-3P	0	0	0	0	0	0	0	0
KRTAP9-1	0	0	0	0	0	0	0	0
KRTAP9-10P	0	0	0	0	0	0	0	0
KRTAP9-11P	0	0	0	0	0	0	0	0
KRTAP9-12P	0	0	0	0	0	0	0	0
KRTAP9-2	0	0	0	0	0	0	0	0
KRTAP9-3	0	0	0	0	0	0	0	0
KRTAP9-4	0	0	0	0	0	0	0	0
KRTAP9-6	0	0	0	0	0	0	0	0
KRTAP9-7	0	0	0	0	0	0	0	0
KRTAP9-9	0	0	0	0	0	0	0	0
KRTCAP2	0	3.947446	0	6.308114	0	0.420658	145.606944	5.25089
KRTCAP2P1	0	0	0	0	0	0	0	0
KRTCAP3	0	0	0	0	7.627852	12.795831	70.274242	9.436248
KRTDAP	0	0	0	0	0	1.712802	0	0
KSR1	2.328131	1.600512	1.046151	4.644223	32.530478	4.898872	0	4.19177
KSR1P1	0	0	0	0	0	0	0	0
KSR2	0	0	0	0.075402	0	0	0	0
KTN1	345.22152	230.343117	297.564056	177.658463	192.466773	136.49118	216.833657	144.965446
KXD1	39.092631	89.351983	51.048677	101.321784	37.625951	118.300029	55.591703	108.371194
KY	0	0	0	0.803143	0	0.143079	0	0.375635
KYAT1	0	7.457951	6.278093	5.34207	16.420852	7.900845	0	3.632734
KYAT3	26.197508	24.260688	31.719564	14.084826	17.467096	5.920786	34.521488	13.137264
KYNU	0.210587	0.475733	0	1.707864	0	0.097905	0	0.732672
KYNUP1	0	0	0	0	0	0	0	0
KYNUP2	0	0	0	0	0	0	0	0
KYNUP3	0	0	0	0	0	0	0	0
L1CAM	15.914346	6.606222	11.160609	12.168268	63.322938	46.329913	22.799052	28.521247
L1TD1	0	0	0	0	0	0	0	0
L2HGDH	43.608461	75.42276	36.858709	73.068238	43.460312	44.203658	17.963395	76.443968

L3HYPDH	23.337598	26.192167	26.347398	16.791839	8.187416	15.046168	38.939694	13.047738
L3MBTL1	8.316155	1.38875	1.44385	1.162325	0	1.264764	5.97525	0.653016
L3MBTL2	13.633856	14.501439	7.520213	15.804393	5.813652	20.538292	0	15.649134
L3MBTL3	8.94053	23.514671	2.842645	15.671163	4.469599	7.636112	30.916938	6.423689
L3MBTL4	0	2.30941	2.522255	0.055003	1.011388	0.140369	0	1.44839
LACC1	8.501208	8.54221	27.197013	6.184658	0	1.377937	10.175628	4.350563
LACRT	0	0	0	0	0	0	0	0
LACTB	19.137445	12.976351	42.615638	6.225479	9.138376	7.466671	124.209271	14.751884
LACTB2	66.715302	54.691508	32.727601	32.868159	29.207708	11.53486	21.300679	23.91173
LAD1	126.487546	183.508206	105.942293	193.37848	267.87946	321.957348	97.290679	207.586197
LAG3	0	0	0	0	0	0.065188	0	0
LAGE3P1	0	0	0	0	0	0	0	0
LAIR1	0	0	0	0	0	0	0	0
LAIR2	0	0	0	0	0	0	0	0
LALBA	0	0	0	0	0	0	0	0
LAMA1	1.666212	2.041321	4.49867	2.839632	5.19793	2.03	1.707713	1.734616
LAMA3	23.885783	27.505587	47.93234	29.578987	115.958533	28.262044	217.730934	35.509153
LAMA4	43.436737	55.589518	21.511687	80.100869	40.732069	36.523047	38.423861	11.792419
LAMA5	41.411333	14.006364	39.46766	19.697826	105.437414	37.739416	21.136663	28.152509
LAMB1	31.713134	30.19734	58.354236	38.71168	47.115616	28.918057	38.5109	23.655961
LAMB2	5.74082	7.595641	13.885373	13.354625	26.126282	13.874062	46.519775	3.070348
LAMB2P1	0	0	0	0.946526	0	0.385214	0	0.078814
LAMB3	123.431179	118.928856	274.461214	234.034818	106.696734	94.779054	54.867948	72.867145
LAMB4	0	0	7.308909	0	0	0	0	0
LAMC1	109.305456	126.936866	202.715354	179.965726	219.862159	193.312067	108.722242	123.461676
LAMC2	34.265689	96.541133	86.384942	278.14679	51.577665	130.610975	18.377805	78.554959
LAMC3	0.433471	0.623104	1.169676	0.301784	0	0.065275	0	0
LAMP1	91.763112	111.868888	146.072356	141.781775	95.906989	102.493022	75.988869	88.387313
LAMP2	55.006389	23.787947	87.746559	17.21225	24.190041	4.011822	51.327695	13.283565
LAMP3	3.138963	7.57456	12.192119	14.118125	13.100849	13.500748	16.265052	3.976045
LAMP5	0	0	0	0	0	0	0	0.469653
LAMTOR1	104.437649	197.234846	97.060058	148.414751	157.191508	247.912613	48.850358	241.45879
LAMTOR2	28.41544	15.150433	24.838895	9.06974	0	24.816936	79.185101	11.250858

LAMTOR3	20.272228	26.605285	18.173185	10.681431	9.90193	6.711682	7.309445	24.412169
LAMTOR3P1	0	0	0	0	0	0	0	0
LAMTOR3P2	0	0	0	0	0	0	0	0
LAMTOR4	0	37.263782	49.446225	60.592871	59.661544	35.733394	0	32.41831
LAMTOR5	4.397728	13.624872	0	6.996939	36.069542	4.793433	0	9.830054
LAMTOR5P1	0	0	0	0	0	0	0	0
LANCL1	20.432072	9.521743	24.244222	19.79482	14.329528	14.651777	10.127261	13.110221
LANCL2	8.522194	5.621582	13.939318	3.668792	20.612252	13.296702	0	19.831425
LANCL3	2.452102	0	0	0.017861	0	0.039564	0.093523	0.083369
LAP3	21.381319	37.055605	24.924635	25.91277	104.577542	50.746272	112.911043	43.828824
LAP3P1	0	0	0	0	0	0	0	0
LAP3P2	0	0	0	0	0	0	0	0
LAPTM4A	72.856479	82.440137	90.043888	57.238449	117.260301	58.530999	97.670281	90.644195
LAPTM4B	123.658189	83.524992	98.92476	54.843781	43.110244	15.54525	18.123122	43.489588
LAPTM4BP1	0	0	0	0	0	0	0	0
LAPTM4BP2	0	0	0	0	0	0	0	0
LAPTM5	1.581186	0.067052	4.238949	1.422054	0	0.705251	0	1.505905
LARGE1	0	0	0	0	0	0	0	0
LARGE2	38.862912	5.787116	12.392409	10.63386	44.28337	17.142787	61.701142	11.003485
LARP1	161.421225	252.963849	130.220319	320.07555	238.248178	585.731252	151.35455	289.779565
LARP1B	20.827062	23.594641	21.708321	35.312038	32.235901	15.407173	19.020026	16.641944
LARP1BP1	0	0	0	0	0	0	0	0
LARP1BP2	0	0	0	0	0	0	0	0
LARP1BP3	0	0	0	0	0	0	0	0
LARP1P1	25.57053	2.60221	0	0	0	0	0	0
LARP4	57.775465	54.580566	34.398329	31.297133	73.949733	30.388696	85.970909	52.02113
LARP4B	0	19.052733	15.364473	20.113617	0	19.955371	0	6.326857
LARP4P	0	0	0	0	0	0	0	0.254988
LARP6	54.210572	38.875447	49.045199	43.887751	38.071397	26.74319	0	24.598226
LARP7	38.710826	29.0838	22.668799	16.397349	13.030032	12.921759	30.784859	30.26985
LARP7P1	0	0	0	0	0	0	0	0
LARP7P2	0	0	0	0	0	0	0	0
LARP7P3	0	0	0	0	0	0	0	0

LARP7P4	0	0	0	0	0	0	0	0
LARS1	85.822401	66.540315	102.588762	73.91603	44.489807	30.03526	107.101733	64.09387
LARS2	19.013676	31.374742	6.157816	20.270423	27.643409	20.956	6.177861	25.38121
LAS1L	22.898193	43.637532	29.439616	56.996455	78.39628	50.737001	45.237606	46.085347
LASP1	127.601926	167.344386	126.756477	204.110596	72.299657	246.287173	124.515075	188.740743
LAT	0	0	0	0.067891	0	0.575029	1.242319	0.403901
LAT2	0	5.956946	4.699623	3.65376	0	0.92766	0	1.736703
LATS1	27.224142	26.482074	7.898614	29.161474	32.418208	32.069509	13.824463	41.870359
LATS2	9.404564	16.843915	8.979878	22.759036	4.348123	20.88251	60.73975	20.564272
LAX1	0	0.158856	0	0.575259	0	0	0	0
LAYN	7.629955	13.773478	3.697245	21.61904	49.168022	18.975369	19.445752	18.298054
LBH	61.577379	42.451652	13.365284	35.62696	40.570035	18.867404	13.381193	8.912294
LBHD1	0	2.880195	3.760143	16.609055	18.039982	12.886082	0	7.312468
LBHD2	0	0.231135	0	1.09123	0	0	0	0
LBP	0	0.405365	0	0.046585	0	0	0	0
LBR	33.602754	76.222747	57.423214	66.236525	81.291125	43.301007	110.627283	57.008784
LBX2	0	0.14653	0	1.095933	0	0.292283	0	0
LCA5	5.756441	9.26825	6.461618	7.963431	0.380113	3.132286	0	4.368452
LCA5L	0	1.744403	2.487805	2.09346	0	0.082296	0	1.291041
LCAT	0	2.593888	2.645261	1.234337	0	0.873638	19.322682	1.212991
LCE1C	0	0	0	0	0	0	0	0
LCE1E	0	0	0	0.188589	0	0	0	0
LCE2A	0	0	0	0	0	0	0	0
LCE3A	0	0	0	0	0	0	0	0
LCE3B	0	0	0	0	0	0	0	0
LCE3C	0	0	0	0	0	0	0	0
LCE3D	0	0	0	0	0	0.999933	0	0
LCE5A	0	0	0	0	0	0.223319	0	0
LCEP1	0	0	0	0	0	0	0	0
LCEP2	0	0	0	0	0	0	0	0
LCEP3	0	0	0	0	0	0	0	0
LCEP4	0	0	0	0.468109	0	0	0	0
LCK	1.652477	2.532136	7.416523	0.315038	2.228581	1.472044	0	0

LCLAT1	48.602802	16.23244	28.363596	33.056111	2.875093	18.064615	18.473995	27.250804
LCMT1	51.512225	53.568902	47.927392	68.298681	24.719988	61.243234	65.026745	52.646887
LCMT2	14.978107	12.573996	16.163694	14.066455	18.175014	11.409754	5.587096	11.913344
LCN1	0	0	0	0	0	0	0	0
LCN10	0	5.367382	1.214874	8.410925	0	0	0	2.093666
LCN12	0	0	0	0	0	0.821518	0	0
LCN15	0	0	0	0.128473	0	0	0	0
LCN1P1	0	0	0	0	0	0	0	0
LCN1P2	0	0	0	0	0	0	0	0
LCN2	0	3.620964	124.012627	192.628146	189.67399	160.470299	159.492631	62.984826
LCN6	0	0	0	0	0	0	0	0
LCN8	0	0	0	0	0	0	0	0
LCN9	0	0	0	0	0	0	0	0
LCNL1	0	0	0	0	0	0	0	0
LCOR	15.333517	34.892041	11.779767	43.03369	22.801799	26.402656	26.242352	56.081085
LCORL	4.422572	4.746673	9.700795	6.344856	2.635395	3.310062	0.479804	2.723462
LCORLP1	0	0	0	0	0	0	0	0
LCP1	0	0.116935	0	0.400187	0.481141	0.591385	0	1.131033
LCP2	0	0	0	0	0	0	0	0
LCT	0	0.040541	0	0	0.250322	0	0	0
LCTL	0	0	0	0	0	0	0	0
LDAF1	31.372205	56.05896	55.250733	84.786122	25.94731	36.825717	25.965227	37.196636
LDAH	10.074768	13.752173	11.382104	8.832321	10.00023	7.00396	10.273377	9.821522
LDB1	19.27693	92.786153	43.114813	108.210502	36.966586	127.739321	0	117.921195
LDB2	0	0	0	0	0	0	0	0
LDB3	0	0	0	0	0	0	0	0
LDC1P	0	0	0	0	0	0	0	0
LDHA	1800.02026	1620.08879	1757.04139	1697.28811	3308.91302	2203.91537	3127.96597	3067.55804
LDHAL6A	0	0	0	0	0	0	0	0
LDHAL6CP	0	0	0	0	0	0	0	0
LDHAL6DP	0	0	0	0	0	0	0	0
LDHAL6EP	0	0	0	0	0	0	0	0
LDHAL6FP	0	0	0	0	0	0	0	0

LDHAP1	0	0	0	0	0	0	0	0
LDHAP2	0	0	0	0	0	0	0	0
LDHAP3	44.353748	29.823812	43.859019	24.035904	59.552301	27.100851	63.914525	46.331834
LDHAP4	29.239672	35.624373	17.407112	25.676329	27.826388	50.842633	20.257706	49.859118
LDHAP5	0	5.977324	12.446593	4.061727	24.387206	3.690212	1.688476	5.36994
LDHAP7	4.063397	9.117069	10.665743	7.2739	36.732494	11.704916	4.957968	15.532151
LDHB	1008.11655	722.827882	899.54701	591.223473	1105.56411	532.251457	1101.77509	831.374385
LDHBP1	0	0	0	0	0	0	0	0
LDHBP2	0	0	0	0	0	0	0	0
LDHBP3	0	0	0	0	0	0	0	0
LDHC	0	0	0	0	0	0	0	0
LDHD	0	0	0	1.179052	0	0	0	0
LDLR	0	8.61821	0	3.514627	19.55988	1.650577	6.394673	10.620656
LDLRAD1	0	0	0	0.158158	0	0	0	0
LDLRAD2	0	0.683746	0	0.099796	0	0	0	0
LDLRAD3	13.891311	7.953064	10.9059	8.917616	11.34759	7.202769	2.95716	12.494438
LDLRAD4	0	0	0	0	0	0.161416	0	0
LDLRAP1	0	0	12.875189	1.366045	0	1.503866	0	0.493656
LDOC1	33.617774	44.922949	26.051663	41.297149	20.799082	16.304928	33.582915	38.892928
LEAP2	0	0	0	0	0	0	0	0
LECT2	0	0	0	0	0	0	0	0
LEF1	0	8.043305	3.094584	2.840926	2.110803	1.60244	0	0.663408
LEFTY1	0	0	0	0	0	0	0	0
LEFTY2	0	0	0	0	0	0	0	0
LEFTY3P	0	0	0	0.540003	0	0	0	0
LEKR1	0	9.339399	0	2.545519	0	0.266176	0	0.651854
LELP1	0	0	0	0	0	0	0	0
LEMD1	0	0	0	0.176151	0	0	0	0
LEMD2	0	22.574781	64.543095	28.271711	61.059804	26.416001	56.449524	19.131658
LEMD3	13.029076	10.59251	40.450191	11.45586	36.046192	25.553463	50.962177	15.826384
LENEP	0	0.80677	0	0	0	0	0	0
LENG1	4.487335	20.72632	7.889784	24.639448	8.238524	25.018856	0	17.173008
LENG8	6.337136	4.508896	11.979075	5.644392	28.76867	20.029062	24.441987	5.698319

LENG9	0	0	0	0	0	0	0	0
LEO1	42.950571	25.211248	24.16488	33.331036	38.593301	27.195482	29.293868	28.925396
LEP	0	0	0	0	0	0	0	0
LEPR	2.186265	1.959556	4.307155	1.07107	4.124948	0.544835	0	0.70851
LEPROT	32.143288	36.710819	39.234128	19.626417	12.609863	13.359913	9.510919	23.137668
LEPROTL1	36.784684	45.140709	24.933777	35.748883	18.079497	16.740339	30.560971	27.944074
LERFS	0	0	0	0.511065	0	0	0	0
LETM1	23.696965	48.243887	16.925584	37.965907	21.028514	79.977893	25.0194	62.652745
LETM1P2	0	0	0	0.038415	0	0	0	0
LETM2	28.399364	66.080067	29.041511	61.810231	9.885694	34.961223	0	51.802683
LETMD1	109.599766	38.216149	98.126118	37.486629	38.189144	8.697849	46.930992	11.980334
LEUTX	0	0	0	0	0	0	0	0
LFNG	5.299151	2.844716	4.730482	2.626576	15.991512	6.783376	26.50981	7.230229
LGALS1	520.35723	478.816492	422.814157	502.154233	185.080637	304.758442	82.853295	256.768015
LGALS12	0	0	0	0	0	0	0	0
LGALS13	0	0	0	0	0	0	0	0
LGALS14	0	0	0	0	0	0	0	0
LGALS16	0	0	0	0	0	0	0	0
LGALS2	0	0	0	0	0	0	0	0
LGALS3	248.27929	110.996674	193.038675	85.089384	147.424477	46.730497	124.700592	80.101322
LGALS3BP	110.820804	64.397728	110.255291	120.885155	166.815143	130.595454	81.795511	65.569685
LGALS4	0	0	0	0	0	0	0	0
LGALS7	11.085162	1.462221	9.419545	1.903001	0	4.996132	0	1.40829
LGALS7B	0	0	0	0	0	0	0	0
LGALS8	58.403801	59.616216	113.474656	77.588135	89.962829	68.385598	67.481977	103.457055
LGALS9	0	0.142343	0	0.057412	10.243551	11.154122	0	5.886116
LGALS9B	0	0	0	0	0	0	0	0
LGALS9C	0	0	0	0	0	0.234167	0	0
LGALS9DP	0	0	0	0	0	0	0	0
LGALSL	6.007943	21.281828	27.817522	29.199773	3.473206	22.414759	55.799379	22.038085
LGI1	0	0	0	0	0	0	0	0
LGI2	0	0.17185	0	0.134368	0	0.050099	0	0
LGI3	0	0	0	0	0	0.926176	0	0

LGI4	0	0.164538	0	0.161328	0	0.309932	0	0.363052
LGMN	30.822566	51.413445	61.001072	93.54079	27.206579	92.159356	60.259949	56.265267
LGMNP1	0	0	0	0	0	0	0	0
LGR4	21.774411	12.262879	16.210424	16.401673	27.118134	25.938526	23.439279	26.996884
LGR5	0	0	0	0	0	0	0	0.153447
LGR6	2.736769	0.758376	0.884996	1.049774	2.19359	0.271901	0	1.962744
LGSN	0	0	0	0	0	0	0	0
LHB	0	0	0	0	0	0	0	0
LHCGR	0	0	0	0	0	0	0	0
LHFPL1	0	0	0	0	0	0	0	0
LHFPL2	26.303026	41.741431	15.738959	45.730601	29.816758	32.12875	0	43.971818
LHFPL4	0	0	0	0	0	0	0	0
LHFPL5	4.779802	0.564725	0	0.080067	0	0.317423	0	0.06585
LHFPL6	30.736386	62.969623	18.789139	53.246906	9.81125	47.611661	18.419987	46.631348
LHFPL7	0	0	0	0	0	0	0	0
LHPP	34.770362	21.430845	19.464936	29.118575	30.552023	19.563795	12.740383	7.488719
LHX1	10.095649	86.952323	12.305305	87.029909	0	0.864148	0	1.653064
LHX2	0	1.997776	0	5.224096	0	0.568879	13.307315	0.934222
LHX3	0	0.138773	0	0.174162	0	0	0	0
LHX4	0.55684	0.119668	0	0.081693	0.588492	0.11165	0	0.022332
LHX5	2.466883	15.710732	5.522025	15.441346	0	2.147727	0	1.240958
LHX6	0	5.666436	0	9.816195	25.667045	7.481487	0	6.313579
LHX8	0	0	0	0	0	0	0	0
LHX9	0	0.444286	0	0.484917	0	0.215591	0	0.194487
LIAS	17.952896	14.392884	26.493607	13.807913	0.849566	9.306262	83.875709	8.058293
LIAT1	5.705203	5.03757	8.481774	4.429454	1.002419	4.038308	0	2.457504
LIF	5.128717	17.49095	10.738135	29.397747	1.805751	7.345284	4.367503	15.137784
LIFR	23.858876	8.425539	1.432009	3.309859	3.040734	0.175925	1.397819	1.120791
LIG1	19.288392	24.622271	42.27673	40.359224	45.918755	118.62664	13.187826	36.10676
LIG3	11.146373	56.181338	21.669064	69.734329	44.569745	60.143109	104.295599	69.247227
LIG4	0	0	10.93275	0.771927	0	0	0	3.58297
LILRA1	0	0	0	0	0	0	0	0
LILRA2	0	0	0	0	0	0	0	0

LILRA2P1	0	0	0	0	0	0	0	0
LILRA3	0	0	0	0	0	0	0	0
LILRA4	0	0	0	0	0	0	0	0
LILRA5	0	0	0	0	0	0	0	0
LILRA6	0	0	0	0	0	0	0	0
LILRB1	0	0	0	0	0	0	0	0
LILRB2	0	0	0	0	0	0	0	0
LILRB3	0	0	0	0	0	0.422758	0	0
LILRB4	0	0	0	0	0	0	0	0
LILRB5	0	0	0	0	0	0.98991	0	0
LILRP1	0	0	0	0	0	0	0	0
LILRP2	0	0	0	0	0	0	0	0
LIM2	0	0	0	0	0	0	0	0
LIMA1	130.331903	309.690747	132.450005	322.159586	141.108543	362.099945	149.314425	263.349482
LIMCH1	10.499283	5.95349	6.080035	13.281613	15.153941	23.72015	8.012514	20.049627
LIMD1	16.51271	25.837498	5.544834	29.447829	13.618103	52.114959	5.467107	35.529008
LIMD2	16.68868	8.55318	12.684551	14.729168	25.201175	13.978798	12.931223	10.088418
LIME1	0	0.067177	0	2.039827	0	2.633175	0	1.358904
LIMK1	20.871128	36.307304	29.195174	32.033332	26.511001	36.846753	15.449945	28.323681
LIMK2	31.317108	21.592683	12.984448	23.680451	25.994451	71.798274	20.470081	62.272156
LIMK2P1	0	0	0	0	0	0	0	0
LIMS1	0	27.116583	33.622441	29.999125	41.145237	26.460176	61.951753	54.257487
LIMS2	0	0.263573	2.925743	0.716103	0	0.601551	13.79372	2.013432
LIMS3	10.533781	1.331805	0	0	0	0.752365	0	0.65034
LIMS4	0	0.607815	0	1.396419	0	0	0	0
LIN28A	0	0	0	0	0	0	0	0
LIN28AP1	0	0	0	0	0	0	0	0
LIN28AP2	0	0	0	0	0	0	0	0
LIN28AP3	0	0	0	0	0	0	0	0
LIN28B	1.197909	0.875774	0	1.070749	0	0.255719	0	0.359023
LIN37	16.676232	0.668046	0	6.019072	9.907163	8.909846	0	5.971793
LIN52	0	0.449468	3.288018	0.576871	0	0	0	12.467833
LIN54	6.901205	18.612096	12.290948	14.735151	19.93169	15.821016	9.042607	19.489169

LIN7A	0	0.796717	2.651566	1.453306	0	0	0	0
LIN7B	9.316393	2.16841	0	2.446356	16.472418	5.526216	0	3.944882
LIN7C	25.034722	6.591066	21.65354	8.345618	59.968081	6.953474	36.913223	16.400677
LIN9	8.095531	3.205866	4.143874	2.449159	0	0.46929	10.56674	4.010993
LINC00115	0	0	0	0.853593	0.802925	0.812173	0	1.36567
LINC00265-2	0	0	0	0	0	0	0	0
LINC00265-3	0	0	0	0	0	0	0	0
LINC00301	0	0	0	0	0	0	0	0
LINC00328-2	0	0	0	0	0	0	0	0
LINC00359	0	0	0	0	0	0	0	0
LINC00367	0	0	0	0	0	0	0	0
LINC00431	0	0	0	0	0	0	0	0
LINC00475	0	0	0	0	0	0	0	0
LINC00623	0	1.535603	0	0	0	0.401026	0	0.692749
LINC00674	0	44.536124	0	28.379298	0	40.756414	0	0
LINC00680	0	1.305087	2.314017	1.85826	0	1.452296	0	1.885695
LINC00869	0	0	0	0	0	0	0	0
LINC00933	0	0	0	0	0	0	0	0
LINC00993	0	0	0	0	0	0	0	0
LINC01002	0	0	13.612428	1.004156	0	0	0	0
LINC01085	0	0	0	0	0	0	0	0
LINC01145	0	0	0	0	0	0	0	0
LINC01347	0	0	0	0	0	0	0	0
LINC01399	0	0	0	0.819719	0	0	0	0
LINC01410	0	0	0	0	0	0	0	0
LINC01556	0	0	0	1.025662	0	0	0	0
LINC01667	0	0	0	0	0	0	0	0
LINC01881	0	0	0	0	0	0	0	0
LINC02113	0	0	0	0	0	0	0	0
LINC02142	0	0	0	0	0	0	0	0
LINC02203	0	0.374323	0	0	0	0	0	0
LINC02210	0	0	0	0	0	0	0	0.357576
LINC02210-C	0	0	0	0.038979	0	0	0	0

LINC02218	0	0	0	0	0	0	0	0
LINC02693	3.866165	5.040516	5.772612	4.091579	6.407042	10.103649	0	10.382426
LINC02694	0	0	0	0	0	0	0	0
LINC02750	0	0	0	0	0	0	0	0
LINC02842	0	0	0	0	0	0	0	0
LINC02898	0	0	0	0	0	0	0	0
LINC02914	0	0	0	0	0	0	0	0
LINC02929	0	0	0	0.122256	0	0	0	0
LINC03003	0	0	0	0	0	0	0	0
LINC03006	0	0	0	0.094254	0	0	0	0.143048
LINC03009	0	4.526944	0	8.239484	6.028102	5.887107	0	5.028817
LINC03028	0	0	0	0	0	0	0	0
LINC03040	0	0	0	0	0	0	0	0.376626
LINC03041	0	0	0	0	0	0	0	0
LINC03042	0	0	0	0	0	0	0	0
LINC03043	0	0	0	0	0	0	0	0
LINC03124	0	0	0	0	0	0	0	0
LINGO1	0	0.161487	0	0.338614	0	1.131625	0	0.957899
LINGO2	0	1.731262	8.52754	2.138049	0	0	0	0
LINGO3	0	0	0	0.484235	0	0	0	0
LINS1	5.531584	6.627677	5.853422	6.809597	13.032647	5.793742	0	6.198866
LIPA	92.679589	40.427485	29.26957	28.730385	53.551003	21.079869	19.511025	31.998655
LIPC	0	0.294897	0	0	0	0	0	0
LIPE	0	2.959421	0.78894	1.034169	1.487478	1.346462	0	1.866639
LIPF	0	0	0	0	0	0	0	0
LIPG	33.372334	33.605012	23.820855	36.186108	35.926436	33.451963	70.711071	34.898056
LIPH	12.02533	7.673655	23.391803	14.30072	4.448427	10.630779	1.972951	4.344682
LIPI	0	0	0	0	0	0	0	0
LIPJ	0	0.508336	0	0	0	0	0	0
LIPM	0	0	0	0	0	0	0	0
LIPT1	7.882152	3.441132	3.950375	3.975235	24.647924	2.359205	0	3.189242
LIPT1P1	0	0	0	0	0	0	0	0
LIPT2	0	11.250951	2.622566	8.386168	4.615556	4.544198	0	5.823956

LITAF	0	40.622729	0	120.949463	51.750627	89.098659	117.787559	22.932111
LITAFD	0	0	0	0	0	0	0	0
LIX1	0	0	0	0.042958	0	0	0	0
LIX1L	14.901217	10.529988	17.08783	8.751043	10.712167	5.41968	7.453724	6.720418
LKAAEAR1	0	0	0	0.110016	0	0	0	0.565785
LLCFC1	0	0	0	0	0	0	0	0
LLGL1	7.824294	14.393006	12.642695	14.038626	3.009382	19.906484	0	20.754579
LLGL2	21.900505	5.141367	36.382125	15.188235	46.393117	32.848214	15.944533	20.549638
LLPH	66.346591	82.115945	39.35445	67.57476	97.594115	72.555252	116.611889	84.428004
LLPHP1	0	0	0	0	0	0	0	0
LLPHP2	0	0	0	0	0	0	0	0
LLPHP3	0	0	0	0	0	0	0	0
LMAN1	76.753971	49.382044	148.253698	92.32922	86.711666	36.518922	54.618774	18.328675
LMAN1L	0	0	0	0	0	0	0	0
LMAN2	72.768163	43.123753	82.584061	69.595515	101.427977	32.767876	21.627269	22.68544
LMAN2L	0	3.700107	33.637226	0.695661	12.165292	2.250701	4.987042	3.787598
LMBR1	19.555607	75.173302	40.578793	37.579047	18.818813	45.274591	31.124933	55.096808
LMBR1L	24.634777	15.094494	14.096326	12.466579	0	5.943602	0	12.876648
LMBRD1	27.904614	8.753803	9.309722	6.120436	33.976167	3.787775	0	6.830586
LMBRD2	30.831627	10.060372	7.864479	5.66356	2.205317	0.75823	2.52014	6.21738
LMCD1	26.486485	18.680812	1.050236	16.241486	0.629992	16.161405	0	9.805226
LMF1	4.441219	9.719803	10.936574	6.360565	0	3.69403	0	0.63037
LMF2	7.849099	9.59588	11.708311	9.268299	19.361889	8.973871	21.787195	5.920254
LMLN	12.318234	3.872789	34.820419	3.975486	2.671424	2.576218	5.285131	6.851711
LMLN-AS1	0	0	0	0	0	0	0	0
LMNA	129.42297	170.611222	149.037723	167.509674	376.28967	318.704874	225.283838	220.384271
LMNB1	114.030658	259.842774	65.32569	208.395051	182.991238	298.840445	245.770868	356.545991
LMNB2	131.124775	179.315748	93.750136	147.489865	85.678618	283.773319	127.177223	205.100307
LMNTD1	7.901805	0	3.66152	0	0	0	0	0
LMNTD2	0	0.783785	0	1.400232	1.307152	2.167201	0	0.666456
LMO1	0	1.065864	0	0.63918	2.90719	0.766877	0	0
LMO2	0	0	0	0.203238	0	0	0	0.423744
LMO3	0	0.120703	0	0	0	0	0	0

LMO4	29.716796	48.159676	13.116636	58.975296	3.85759	19.949004	0	33.263758
LMO7	48.471524	53.182838	13.676678	45.270175	71.741571	28.206418	69.277354	53.389301
LMOD1	0	0	0	0	0	0	0	0
LMOD2	0	0	0	0	0	0	0	0
LMOD3	0	0	0	0	0	0	0	0
LMTK2	8.247023	16.367426	10.002324	23.424847	15.258253	25.741529	4.899261	29.360488
LMTK3	4.638838	4.9129	4.785725	3.766848	2.038978	2.475936	0	1.399873
LMX1A	0	0	0	0	0	0	0	0
LNP1	3.868828	21.60091	6.69353	23.394936	0.539483	4.54011	3.958778	5.440259
LNPEP	26.126877	18.006253	28.835568	15.61477	36.345082	12.298663	17.204979	18.513886
LNPK	23.829409	13.839782	36.356843	7.492798	24.76674	23.305178	8.04428	23.804996
LNX1	0	0	0	0.722009	0	0.74892	0	2.25528
LNX2	7.060163	12.586453	5.706475	12.850151	2.187902	9.915305	8.252334	12.519924
LONP1	18.689222	21.522922	15.408312	34.241739	28.052471	51.010902	55.39071	37.097227
LONP2	43.440028	41.106078	29.58474	43.71325	23.927868	46.200246	51.622617	30.648046
LONRF1	8.259796	10.296256	8.498396	6.990182	7.431427	3.266752	22.835576	5.420489
LONRF2	0	0	0	0.065019	0	0	0	0
LONRF2P1	0	0	0	0	0	0	0	0
LONRF2P2	0	0	0	0	0	0	0	0
LONRF2P3	0	0	0	0	0	0	0	0
LONRF2P4	0	0	0	0	0	0	0	0
LONRF2P5	0	0	0	0	0	0	0	0
LONRF3	0	0	0	1.35126	0	0	0	0.222324
LORICRIN	0	0	0	0	0	0	0	0
LOX	10.980903	4.739413	20.469448	2.930352	6.746444	0.905	10.98566	4.528076
LOXHD1	0	0	0	0	0	0	0	0
LOXL1	19.744833	5.879028	14.75557	6.455878	38.36799	9.799792	86.041094	6.296548
LOXL2	0	1.334443	10.022525	4.462212	0	2.727216	5.871679	0.624227
LOXL3	1.730501	1.822818	7.600812	3.317	2.301688	5.186598	0	7.01554
LOXL4	1.846803	2.804299	7.454303	6.488814	10.239883	6.269827	6.288354	3.190766
LPAL2	0	1.326407	0	1.850386	0	0.797967	0	3.097313
LPAR1	44.371175	77.89506	25.467634	90.963595	19.866645	51.410677	16.205717	90.441375
LPAR2	1.967008	15.690165	8.769664	23.944996	0.51835	35.210321	0	25.582696

LPAR3	39.88028	59.4703	42.42666	45.565073	21.232774	14.648972	41.512622	29.001176
LPAR4	0	0	0	0	0	0	0	0
LPAR5	3.783232	10.794116	3.386918	9.964768	4.65858	14.739027	0.306129	18.58274
LPAR6	0	0.785379	0	0	0	0.794464	0	0
LPCAT1	68.514976	105.89371	134.089324	196.74914	53.533532	132.28337	87.629953	76.874225
LPCAT2	176.974644	74.125745	62.41195	61.04118	51.78903	33.586987	11.976957	43.738783
LPCAT2BP	0	0	0	0	0	0	0	0
LPCAT3	35.587485	35.061136	20.600714	34.464415	46.175065	23.855006	60.064366	22.506906
LPCAT4	37.30664	38.199653	56.38768	46.379352	72.183291	22.176288	32.173571	24.029057
LPGAT1	56.764865	58.368781	27.645961	48.501504	26.904243	54.643121	17.727293	60.194561
LPIN1	13.940198	21.504974	17.227789	17.528553	7.568883	31.161403	36.8672	18.7568
LPIN2	0	0	0	0	0	0	0	0
LPIN3	0	0	3.918141	1.056707	7.800351	1.211803	0	0.258995
LPL	0	0.039866	1.360886	1.199134	0	0	0	0
LPO	0	0	0.886653	0	0	0	0	0
LPP	0	20.757425	0	47.854761	78.632185	28.161345	0	13.802033
LPXN	9.480942	2.128261	0	1.335801	11.657397	1.830589	0	0
LRAT	1.336304	2.379272	4.129825	1.343922	0.336653	1.30323	0	0.776579
LRATD1	26.879057	76.373942	18.190328	57.986861	8.833057	40.428845	18.519101	35.164201
LRATD2	35.538804	33.378475	18.673343	34.108492	38.177449	78.673266	94.057584	106.619016
LRBA	22.548277	17.136339	19.947547	2.796906	10.429917	15.676277	8.560775	12.619977
LRCH1	32.02186	21.946953	13.133448	17.928588	4.72382	15.826513	9.15721	14.618404
LRCH2	0	0	0	0.016441	0	0	0	0
LRCH3	16.108646	28.836387	23.747888	12.35327	22.843244	15.288977	35.313734	8.242323
LRCH4	16.248746	7.168517	3.170067	6.695598	0	14.130029	0	6.827037
LRCOL1	0	0	0	0	0	0	0	0
LRFN3	23.600764	9.433623	0	16.863984	21.284032	40.780205	10.061105	29.314259
LRFN4	23.47462	100.462535	28.385017	108.300809	69.446137	247.469113	26.226027	196.302667
LRFN5	0	0	0	0	0	0	0	0
LRG1	0	0.387817	1.165072	0.41199	0.343449	0.647104	0	0.052404
LRGUK	0	0	0	0.55496	0	0.047976	0	0
LRIF1	42.16332	65.734166	19.865823	41.941974	24.456096	30.856077	15.598736	48.955276
LRIG1	0	4.252992	2.151242	3.093365	25.269366	0.699663	0	0

LRIG2	2.769135	2.113994	7.362208	3.381658	4.099279	2.423184	4.996189	2.502497
LRIG3	0	7.910985	0	3.778685	48.832922	3.738173	17.040788	7.489482
LRIT3	0	0	0	0	0	0	0	0
LRMDA	0	10.580169	0	2.291616	0	0.628336	0	0.036405
LRP1	0	3.374925	0	8.265777	29.432906	3.724456	48.487346	2.163018
LRP10	20.203539	45.825977	37.182293	55.299589	37.773258	73.105413	11.800386	39.262432
LRP11	0	12.368467	89.808895	30.231313	63.341283	44.962637	6.180796	39.720926
LRP12	7.258072	23.717933	0	25.168722	35.788083	5.92226	37.098964	14.337186
LRP1B	0	0.258905	0	0	0	0	0	0
LRP2	0	0.074754	0	0.009982	0	0.065482	0	0.040772
LRP2BP	0	0.13606	0	0.346681	0	0	0	0
LRP3	1.627059	5.715066	19.450293	4.40111	10.58092	17.688796	46.687984	9.636936
LRP4	3.163249	6.260717	3.201582	4.943603	7.6129	8.515973	4.147578	4.547311
LRP5	50.248168	37.488503	50.473917	64.027805	16.008214	42.562005	24.450221	22.22359
LRP5L	0	0	0	0	0	0.855985	0	0
LRP6	15.808709	32.878709	30.074586	32.501987	16.029499	33.719991	42.716799	22.310016
LRP8	62.265687	51.972465	106.694223	77.762499	137.22708	34.003086	3.567535	23.990936
LRPAP1	60.506009	95.608539	88.227721	133.091247	66.770002	112.889982	40.704537	75.205637
LRPPRC	54.636103	45.298576	80.651984	28.47088	48.970259	11.470311	48.462814	39.101131
LRR1	38.982457	27.0839	37.205736	14.845432	20.769579	27.986127	29.411113	55.57007
LRRC1	16.949319	35.540971	19.938401	25.687672	12.303561	17.870016	4.891496	25.137775
LRRC10B	0	1.28245	0	0.661242	0	0.299707	0	0
LRRC14	18.074084	14.381979	20.530504	15.532227	5.484181	14.098061	0	13.122129
LRRC14B	0	0.112402	0	0	0	0	0	0
LRRC15	0	0	0	0	0	0	0	0
LRRC17	0	3.435401	0	1.042686	0	0.486987	0	2.090708
LRRC19	0	0	0	0	0	0	0	0
LRRC2	0.669713	0.449583	0.601574	0.305043	5.160182	1.027137	9.004109	3.760124
LRRC20	23.849061	32.834843	16.260958	21.108318	10.083385	17.08972	6.475067	12.068138
LRRC23	14.318341	10.59336	2.280223	12.01742	0.675419	5.770764	15.946246	6.234472
LRRC24	0	0.262559	1.85103	0.251732	0	0.614285	0	0.839586
LRRC25	0	0	0	0.087925	0	0	0	0
LRRC26	0	8.69449	0	5.111407	0	1.798332	0	0

LRRC27	1.993129	12.263824	25.525606	14.358441	1.723057	9.5705	0	6.630737
LRRC28	29.288243	15.186755	12.133731	14.738789	0.587663	22.749527	2.574895	27.664926
LRRC2P1	0	0	0	0	0	0	0	0
LRRC3	1.113484	2.656158	6.005026	3.827105	0.735486	2.651295	0	1.183364
LRRC31	0	0	0	0.105929	0	0	0	0
LRRC32	0	0	0	0.267823	0	0	0	0
LRRC34	4.321254	5.10674	0	1.311698	0	0	0	2.036033
LRRC34P1	0	0	0	0	0	0	0	0
LRRC34P2	0	0	0	0	0	0	0	0
LRRC36	0	0	0	0.236029	0	0	0	0
LRRC37A	2.010882	0.814669	0	0.734639	0	0.032889	0	0.359384
LRRC37A11P	0	0	0	0.022643	0	0	0	0
LRRC37A12P	0	0	0	0	0	0	0	0
LRRC37A13P	0	0	0	0	0	0	0	0
LRRC37A14P	0	0	0	0	0	0	0	0
LRRC37A15P	2.690739	0	2.389498	0.667855	0	1.517584	0	1.186188
LRRC37A16P	0	0.03828	0	0.043441	0	0	0	0.035755
LRRC37A17P	0	0.612108	0	0.819804	1.200636	0.542347	0	1.383356
LRRC37A2	0	2.924213	5.885551	2.537664	0	2.152353	0	1.337357
LRRC37A3	9.536725	6.936767	0	1.065413	0	2.475995	0	3.26042
LRRC37A4P	3.879268	4.968078	2.901622	5.737821	6.878704	9.334306	5.482951	5.873957
LRRC37A5P	0	0	0	0.247356	0	0	0	0
LRRC37A6P	0	0	0	0	0	0	0	0
LRRC37A7P	2.604041	0	0	0	0	0	0	0
LRRC37A9P	1.214059	0.521468	0.545463	0.652891	1.12258	0.212879	0	0.535714
LRRC37B	1.389498	4.682243	11.219217	5.245719	6.516808	9.136702	45.661393	4.351775
LRRC37BP1	0	3.673716	0	12.173078	20.980914	1.120642	0	6.382871
LRRC38	0	0	0	0	0	0.157421	0	0.192115
LRRC39	0	0	0	0	0	0	0	0
LRRC3B	0	0	0	0	0	0	0	0
LRRC4	0.785745	0.06734	0	0.153497	0	1.529163	0	1.041105
LRRC40	11.852061	7.174036	22.291625	3.782593	13.762309	1.946852	7.483681	5.674549
LRRC41	14.391523	14.626173	14.866636	14.249044	36.923247	23.586571	16.952838	23.487439

LRRC42	123.146747	166.157816	118.302985	149.287591	27.328032	68.484803	47.213477	74.691184
LRRC43	0	0	0	0	0	0.358071	0	0.91889
LRRC45	7.521326	8.454481	8.978284	8.638131	6.615504	21.770128	28.570917	10.093839
LRRC46	0	1.68162	0	2.324797	0	0.91535	0	0.962406
LRRC47	19.128628	25.996382	23.970487	27.303866	19.803973	29.923097	93.788093	45.49809
LRRC49	36.870715	11.081344	28.057957	13.004342	5.364209	3.022251	0	2.087735
LRRC4B	0	0.290751	1.01779	1.052059	0	0	0	0.091394
LRRC4C	0	0.069767	0	0.109376	0	0	0	0.131057
LRRC51	19.620763	43.219761	0	38.784795	0	23.641786	157.419829	26.18557
LRRC55	0	0	0	0	0	0	0	0
LRRC56	0	4.571098	1.093358	5.879688	0	4.428271	0	2.845405
LRRC57	12.800713	34.084139	24.111955	45.703347	14.529623	41.203818	9.796067	56.026383
LRRC57P1	0	0	0	0	0	0	0	0
LRRC58	25.26638	14.057846	23.46281	7.981155	39.418727	7.386742	49.304436	21.685704
LRRC59	90.583301	115.154071	79.57478	97.518259	96.251085	137.013063	130.882618	155.551551
LRRC61	5.796128	23.373769	6.89238	19.697546	5.600855	27.033764	0	28.836437
LRRC63	0	0	0	0	0	0	0	0
LRRC66	0	0	0	0.048909	0	0	0	0.08061
LRRC69	0	0	0	0.045596	0	0	0	0
LRRC7	0.82676	2.288951	0.896357	0.919609	2.02742	1.051904	5.929725	1.771022
LRRC70	0	0	0	0	0	0	0	0
LRRC71	0	0.464897	0	0.266964	0	0.181192	0	0.074002
LRRC72	0	0	0	0	0	0	0	0
LRRC74A	0	0	0	0	0	0	0	0
LRRC74B	0	0	0	0	0	0	0	0
LRRC75A	1.06614	0	0	0.682838	1.12568	0.159417	0.259092	0.162673
LRRC75B	0	1.563606	0	2.559359	9.479875	5.404081	0	1.682919
LRRC77P	0	0	0	0	0	0	0	0
LRRC78P	0	0	0	0	0	0	0	0
LRRC8A	46.755263	26.453474	7.041192	21.575442	0	34.254649	37.758929	27.068299
LRRC8B	0	8.115562	0	3.652156	0	2.517094	0	4.447982
LRRC8C	20.551161	38.67271	21.099327	27.332563	9.776275	9.318811	14.283857	19.269577
LRRC8D	59.82042	122.420159	101.044634	126.592661	54.427003	105.379631	16.966979	111.303889

LRRC8E	3.701196	9.057729	9.959387	10.7138	14.658413	7.656559	8.101554	8.575674
LRRC9	0	0	0	0	0	0	0	0
LRRCC1	14.236397	18.345455	27.744913	9.766036	12.381938	3.252164	26.392506	8.767668
LRRD1	0	0	0	0	0	0	0	0
LRRFIP1	52.324842	79.705621	47.031907	120.143157	90.080169	129.150507	110.909776	109.790684
LRRFIP1P1	0	2.422838	0	3.582317	0	2.378199	0	2.032607
LRRFIP2	26.104814	97.216913	94.118968	63.742461	43.762887	85.938707	99.655174	105.901131
LRRFIP2P1	0	0	0	0	0	0	0	0
LRRIQ1	0	1.99312	14.733419	0.811544	0	0	0	0.149141
LRRIQ3	0.918825	2.28908	4.466394	2.737643	0	0	0	0.090597
LRRK1	6.78953	7.375051	5.651028	8.422449	10.57132	28.842384	5.62335	26.491745
LRRK2	0	0.360719	0.455871	0.051127	0	0	0	0.34645
LRRN1	0	0	0	0	0	0	0	0
LRRN2	0	2.801246	0.933236	4.246068	0	0.934332	0	0.539929
LRRN3	0	0	0	0	0	0	0	0
LRRN4CL	0	0	0	0	0	0	0	0
LRRTM1	0	0	0	0	0	0	0	0
LRRTM2	0	0	0	0	0	0	0	0
LRRTM3	0	0	0	0	0	0	0	0
LRRTM4	0	0	0	0	0	0	0	0
LRSAM1	1.182538	6.196379	9.873806	11.5774	6.516346	12.956157	4.882235	10.273677
LRTM1	0	0	0	0	0	0	0	0
LRTM2	0	0	0	0.088475	0	0.060247	0	0.048717
LRTOMT	0	1.701701	0.942208	15.377174	0	1.492528	0	0.564542
LRWD1	7.973931	14.387678	20.526523	17.265951	66.666777	26.051095	20.35979	20.873169
LSAMP	0	0	0	0	0	0	0	0
LSG1	68.093605	68.009947	53.107978	61.714707	107.195045	67.051683	24.967834	56.447449
LSM1	64.184268	94.5965	23.030041	72.992207	27.514133	60.755678	68.703842	85.258797
LSM10	13.979027	48.821522	11.273381	41.697069	3.665328	63.303344	54.353182	75.078044
LSM11	3.950171	2.017847	3.9958	1.678822	12.003681	1.114585	5.056109	2.217135
LSM12	0	38.596808	0	20.833456	17.525795	29.457206	0	16.276796
LSM12P1	0	62.031385	42.976481	101.446901	0	60.366848	0	78.877422
LSM14A	148.198509	132.284531	109.801552	145.630672	153.175926	191.876029	75.570337	143.702865

LSM14B	63.526993	98.960597	43.626282	102.700733	78.311256	117.461962	61.705785	106.422365
LSM1P1	0	0	0	0	0	0	0	0
LSM1P2	0	0	0	0	0	0	0	0
LSM2	55.935364	111.625826	41.823745	105.362654	95.408958	141.149568	0	119.657384
LSM2P1	0	0	0	0	0	0	0	0
LSM2P2	0	0	0	0	0	0	0	0
LSM3	28.785728	25.388208	17.946329	23.289631	20.701692	15.547639	14.479364	21.584246
LSM3P2	0	0	0	0	0	0	0	0
LSM3P3	0	0	0	0	0	0	0	0
LSM3P4	0	0	0	0	0	0	0	0
LSM3P5	0	0	0	0	0	0	0	0
LSM4	124.158188	308.483158	102.348827	217.924752	177.736159	372.164844	236.283609	363.857539
LSM5	177.456157	119.770344	99.489839	47.746	173.356904	47.542576	213.118687	152.260349
LSM6	67.912184	93.178003	40.22019	73.813072	81.300687	82.514948	71.044064	88.792705
LSM6P2	0	0	0	0	0	0	0	0
LSM7	91.876248	69.757688	121.909857	95.893424	63.509931	85.390018	0	67.959138
LSM8	16.274735	80.269543	16.149988	56.159773	7.361873	27.676865	69.339415	34.642859
LSMEM1	0	0	0	0.226776	0	0	0	0.150927
LSMEM2	0	0	0	0	0	0	0	0
LSP1	0	1.274641	0	2.025105	0	0	0	0
LSP1P1	0	0	0	0	0	0	0	0
LSP1P2	0	0	0	0	0	0	0	0
LSP1P3	0	0	0	0	0	0	0	0
LSR	48.18604	96.506912	71.367094	140.354775	55.120939	135.54866	125.487049	96.600728
LSS	11.989117	9.42362	10.764428	10.420136	34.908359	43.356924	143.510742	40.216587
LST1	0	1.886583	0	0.629463	0	0	0	0
LTA	0	0	0	0	0	0	0	0.42826
LTA4H	118.580639	50.910085	119.475361	63.112851	189.052438	52.789084	151.678075	76.917277
LTB	0	0	5.238353	0.885032	27.177453	27.681129	28.418747	15.087589
LTB4R	1.251066	2.065929	0	0.600099	5.822491	2.056264	2.735238	0.676923
LTB4R2	0	0	0	0.328305	0	0.588671	0	0.4358
LTBP1	6.573173	7.509454	6.352433	4.258343	0	1.210212	26.751183	0.22
LTBP2	15.561906	10.805979	7.856112	12.202519	4.026133	9.083634	3.671734	5.319899

LTBP3	17.145628	17.667586	22.174774	22.409441	5.23173	8.28496	0	4.118692
LTBP4	13.552884	38.074644	12.711866	44.417394	102.535538	82.779765	48.873843	33.996592
LTBR	96.962741	138.427492	204.32347	226.45266	74.265163	260.845161	78.694793	138.61243
LTC4S	0	2.69224	0	0	0	0.382151	0	1.106233
LTF	0	0	0	0	0	0	0	0
LTK	0	0	0	0.083596	0	0	0	0
LTN1	15.142638	8.568354	19.296809	5.054726	27.134281	5.325175	22.678503	14.769569
LTO1	25.632851	21.671655	39.190278	18.180375	11.161075	6.005794	18.693038	10.762092
LTO1P1	0	0	0	0	0	0	0	0
LTV1	39.649349	40.192264	16.842912	24.842062	36.823811	28.815212	9.591127	39.568377
LTV1P1	0	0	0	0	0	0	0	0
LUC7L	7.665192	3.096123	0	8.432402	36.406063	11.837521	3.732864	7.55054
LUC7L2	47.600072	82.141467	30.790386	80.234993	46.660486	89.020849	76.298666	132.843162
LUC7L3	45.124747	55.947277	33.852488	39.8455	72.256299	61.433454	110.811768	81.498303
LUM	0	0	0	0	17.610659	1.213693	0	0.255021
LURAP1	0	1.758534	0	1.561311	0.49881	0.934724	0	0.840669
LURAP1L	1.261077	10.198915	4.515891	13.861045	0	6.216293	0	11.758071
LUZP1	0	8.112274	0	23.505664	20.889288	35.221074	16.59092	23.491762
LUZP2	3.285049	0.648636	0.590206	1.103395	0.173557	0.164484	0	0.276621
LUZP4	0	0	0	0	0	0	0	0
LUZP4P1	0	0	0	0	0	0	0	0
LVRN	0	0	0	0.040657	0	0	0	0
LXN	0	8.957547	4.870481	6.916617	13.065674	21.705977	38.306389	26.329289
LY6D	27.870943	38.038679	73.057218	94.09797	10.212041	15.07958	0	9.635319
LY6E	161.775123	299.199401	215.152009	322.84464	126.35736	212.174838	83.027335	169.357263
LY6G5B	0	0	0	0.070826	0	0	0	0.142713
LY6G5C	8.874142	3.265785	0	2.492914	1.266292	3.030848	0	3.998957
LY6G6C	0	0	0	0.212477	0	1.707423	0	0.545389
LY6G6D	0	0	0	0	0	0	0	0
LY6G6E	0	0	0	0	0	0	0	0
LY6G6F	0	0	0	0	0	0	0	0
LY6G6F-LY6(	0	0	0	0	0	0	0	0
LY6H	0	0	0	0	0	0	0	0

LY6K	46.644803	77.224336	11.310436	61.681098	4.346644	85.837394	0.307595	69.51931
LY6L	0	0	0	0	0	0	0	0
LY75	0	0	0	0	0	0	0	0
LY75-CD302	0	0	0	0	0	0	0	0
LY86	0	0	0	0	0	0	0	0
LY9	0	0	0	0	0	0	0	0.025812
LY96	0	0	0	0	0	0	0	0
LYAR	75.900309	131.511429	54.554484	141.854571	53.2322	138.565295	62.724962	125.865793
LYARP1	0	0	0	0	0	0	0	0
LYG1	0	0	0	0	0	0	0	0
LYG2	0	0	0	0	0	0	0	0
LYL1	0	0	0	0.104941	0	0	0	0
LYN	28.185669	147.374535	67.334247	176.303026	39.65579	111.41408	6.009415	79.985287
LYNX1	0	1.614617	1.300306	1.427052	0	0.615821	0	0.261549
LYNX1-SLUR	0	0	0	0.295679	0	0	0	0
LYPD1	0	0.625209	0	0	0	0.739444	0	1.449837
LYPD3	4.322454	4.275176	5.775003	4.869273	12.762679	6.248784	12.283177	4.368669
LYPD4	0	0	0	0	0	0	0	0
LYPD5	0	5.367469	4.199898	7.005091	0	8.760328	0	7.254541
LYPD6	6.49999	4.503667	4.43451	3.372648	0	1.105596	0	0.485038
LYPD6B	0	18.655546	6.083189	11.049478	9.943077	9.369745	0	8.261885
LYPD8	0	0	0	0	0	0	0	0
LYPD9P	0	0	0	0	0	0	0	0
LYPLA1	294.321796	292.037365	283.0482	162.794676	104.027049	51.383556	125.09499	128.754454
LYPLA1P1	0	0	0	0	0	0	0	0
LYPLA1P2	0	0	0	0	0	0	0	0
LYPLA1P3	0	0	0	0	0	0	0	0
LYPLA2	34.746406	55.035325	12.342954	47.784143	21.16627	82.766184	76.474102	56.924168
LYPLA2P1	0	0	0	0	0	0	0	0
LYPLA2P2	0	0	0	0	0	0	0	0
LYPLA2P3	0	0	0	0	0	0	0	0
LYPLAL1	74.390579	19.132279	21.129996	8.309542	5.465572	5.824451	6.991695	11.661329
LYRM1	29.028171	30.738684	21.407003	21.648149	23.61478	18.109076	21.180012	21.938009

LYRM2	36.066376	59.305675	45.462408	38.036605	18.129183	28.87039	46.214572	71.617008
LYRM4	16.323058	36.083268	16.810036	29.264275	30.54093	43.809494	0	47.410572
LYRM7	11.489325	28.112009	8.920524	15.396933	15.251542	8.218839	9.03861	11.263234
LYRM9	0	2.447708	0	1.79623	0	0.31034	0	0
LYSET	29.640308	25.049206	22.150596	26.893569	0	12.205389	0	46.027751
LYSETP1	0	0	0	0	0	0	0	0
LYSMD1	0	12.70442	6.614843	17.562902	2.283085	16.709735	0	11.024092
LYSMD2	14.966416	10.519485	9.90957	10.977968	22.751053	8.207872	0	10.647285
LYSMD3	12.365393	19.349689	13.875978	22.011269	41.716502	16.047351	34.675992	20.893665
LYSMD4	2.804962	6.352375	2.267855	5.214294	2.959765	16.86036	8.50461	12.031004
LYST	10.05514	10.29017	2.218619	6.154821	0	7.72173	1.734786	5.3009
LYVE1	0	0	0	0	0	0	0	0
LYZ	0	0.655165	0	0	0	0	0	0
LYZL1	0	0	0	0	0	0	0	0
LYZL2	0	0	0	0	0	0	0	0
LYZL4	0	0	0	0	0	0	0	0
LYZL6	0	0	0	0	0	0	0	0
LZIC	24.418127	48.914765	10.016862	36.441562	35.749772	37.613806	7.82867	72.693444
LZTFL1	27.654444	43.311032	20.466144	26.589566	44.079981	13.485449	16.91019	31.158651
LZTR1	3.076263	7.784656	3.989187	6.763364	51.52159	13.566746	5.307746	11.167034
LZTS1	0	1.888587	3.924429	0.851507	0	0.486295	0	0.335529
LZTS2	5.838218	19.906513	11.695108	21.698784	2.376788	33.544722	6.806323	17.891054
LZTS3	8.955759	1.85596	5.115068	0.752682	3.440113	0.353319	9.90685	0.19316
M1AP	0	0	0	0	0	0	0	0
M6PR	122.072686	160.219663	119.270086	262.613756	106.862296	196.281846	133.893516	155.223733
M6PRP1	0	0	0	0	0	0	0	0
MAB21L3	0	0	0	0	0	0	0	0
MAB21L4	0	0	0	0	0	0	0	0.143374
MACC1	0	19.605184	4.578489	19.428104	24.001017	20.656269	13.156842	20.517636
MACF1	13.173455	32.975007	47.708728	50.708432	52.036493	109.642879	60.523953	120.128324
MACIR	11.464092	19.850075	13.174084	16.825936	9.076884	14.645171	34.498363	18.64029
MACO1	11.65916	14.080761	17.753838	15.576057	7.37894	13.838011	48.766627	13.529009
MACROD1	17.618392	12.696973	3.874973	30.592652	29.021712	41.961747	0	32.290803

MACROD2	0	2.145102	0	3.254226	0	2.957089	0	1.980248
MACROH2A1	170.259123	528.401293	170.921057	498.418014	206.279298	771.592872	79.859092	603.327118
MACROH2A2	50.477107	107.071654	20.916527	120.220768	44.669696	143.488653	13.96136	111.581184
MAD1L1	55.02579	81.438223	27.159362	72.218071	35.514502	35.560492	8.013977	25.828599
MAD2L1	34.28815	68.854939	20.390924	35.126261	79.598279	39.127236	95.574482	82.132205
MAD2L1BP	0	0	0	0.765471	0	0	0	0
MAD2L1P1	0	0	0	0.341468	0	0	0	0
MAD2L2	58.006491	72.146218	38.432255	58.658983	51.837554	75.375264	60.695955	67.840582
MADCAM1	0	0.690417	0	0.958497	0	0	0	0
MADD	10.314688	23.180353	16.576919	28.798863	25.251631	70.993517	26.996156	51.958295
MAEA	31.544592	29.22117	16.913753	30.922814	40.708107	53.067284	54.2718	40.820233
MAEL	0	0	0	0	0	0	0	0
MAF	0.508345	0.459272	0	0.361125	0.805932	3.104188	0	2.614351
MAF1	43.222287	95.836672	32.902293	108.543398	43.281067	122.757841	56.500337	88.755436
MAF1P1	0	0	0	0	0	0	0	0
MAFA	0	0	0	0.742882	0	0	0	0
MAFB	0	0.378243	0	0.143906	0.778796	0.147169	0	0.079043
MAFF	14.389774	24.240005	24.451831	43.274027	7.36193	26.208159	0	27.095837
MAFG	20.033239	23.609263	23.222441	31.485355	15.023618	23.055506	9.787679	21.000388
MAFIP	3.677838	4.850235	5.797494	1.771358	0	0.918395	0	3.802781
MAFK	38.457509	128.105916	81.620627	179.322409	12.235428	80.799569	14.384765	67.755682
MAG	0	0	0	0	0	0	0	0
MAGEA1	0	0	0	0	0	0	0	0
MAGEA10	0	0	0	0	0	0	0	0
MAGEA11	0	0	0	0	0	0	0	0
MAGEA12	0	0	0	0	0	0	0	0
MAGEA13P	0	0	0	0	0	0	0	0
MAGEA2	0	0	0	0	0	0	0	0
MAGEA2B	0	0	0	0	0	0	0	0
MAGEA3	0	0	0	0	0	0	0	0
MAGEA4	0	0	0	0	0	0	0	0
MAGEA5P	0	0	0	0	0	0	0	0
MAGEA6	0	0	0	0	0	0	0	0

MAGEA7P	0	0	0	0	0	0	0	0
MAGEA8	0	0	0	0	0	0	0	0
MAGEA9	0	0	0	0	0	0	0	0
MAGEA9B	0	0	0	0	0	0	0	0
MAGEB10	0	0	0	0	0	0	0	0
MAGEB16	0	0	0	0.051486	0	0	0	0
MAGEB17	0	0	0	0	0	0	0	0
MAGEB2	0	0	0	0	0	0	0	0
MAGEB3	0	0	0	0	0	0	0	0
MAGEB5	0	0	0	0	0	0	0	0
MAGEB6	0	0	0	0	0	0	0	0
MAGEC1	0	0	0	0	0	0	0	0
MAGEC2	0	0	0	0.08456	0	0	0	0
MAGEC3	0	0	0	0.091098	0	0	0	0
MAGED1	110.181318	46.387265	14.398969	56.931584	1.149575	5.863902	38.223677	14.899761
MAGED2	32.121202	93.545202	69.451442	120.81575	52.028025	45.504807	21.545453	45.189772
MAGED4	23.174744	0	1.26135	2.318918	41.135874	5.554803	0	4.962953
MAGED4B	16.985974	3.247043	38.383814	11.833415	47.604554	0.286155	7.984659	0.90491
MAGEL2	0	0	0	0	0	0	0	0
MAGI1	4.447688	12.999844	7.144536	12.119709	0.275707	8.398001	0	4.229223
MAGI2	1.118026	0.141976	0	0.078418	0	0.235292	0	0.229211
MAGI3	4.983777	12.900211	3.584863	17.631527	11.323671	12.100443	7.533214	18.421184
MAGIX	1.018265	1.174514	3.188317	1.183522	0.268807	1.075656	0	2.361851
MAGOH	368.338028	318.569341	223.929928	284.541419	139.354295	73.445939	44.002419	110.307496
MAGOH2P	0	0	0	0	0	0	0	0
MAGOH3P	0	0	0	0	0	0	0	0
MAGOHB	8.222607	34.644777	18.629829	25.80647	2.204488	15.885784	0	25.703854
MAGT1	0	18.21742	0	30.845438	9.885205	25.282068	0	11.564387
MAIP1	0	0	0	1.363779	0	16.099491	0	13.178054
MAJIN	0	0	0	0	0	0	0	0
MAK	0	0.279366	0	0.573584	0	0	0	0.723355
MAK16	28.930563	51.161463	28.459275	62.325167	31.418535	28.646877	36.087486	41.896237
MAL	0	0	0	0	0	0	0	0

MAL2	46.520417	32.927558	47.004715	20.62697	108.286927	42.146219	160.41741	104.264164
MALL	0	15.112488	0	14.074714	0	13.383783	49.537466	20.457054
MALLP1	71.06689	13.121354	0	11.672654	0	0	0	0
MALLP2	0	0	0	0	0	0	0	0
MALRD1	0	0	0	0	0	0	0	0
MALSU1	37.144139	132.246661	57.101925	74.051328	92.776304	66.243339	32.223771	104.833276
MALT1	22.268899	19.882069	26.271822	14.010846	29.894184	20.878822	47.423925	22.710032
MAMDC2	0	0	0	0.097082	0	0.248192	0	0.43998
MAMDC4	0	0.137501	0	0.019591	0	0.045002	0	0
MAML1	18.126315	54.294361	15.273424	36.216633	25.292312	88.750704	27.471074	54.439846
MAML2	19.08229	36.648858	19.204895	51.716911	10.334514	20.876306	9.541891	33.829932
MAML3	2.839415	8.002524	4.681192	10.628635	0.125053	1.804537	0	1.100293
MAMLD1	7.728251	6.184948	2.701338	1.481745	0.307586	0.960655	0	0
MAMSTR	1.882426	0	3.721451	0.502444	0	0.926199	0	1.140146
MAN1A1	4.56387	3.610397	4.685646	3.059158	1.20561	0.228528	0	0.549358
MAN1A2	67.511371	160.826173	74.761169	106.505596	25.734291	39.169888	125.256924	70.063289
MAN1A2P1	0	0	0	0	0	0	0	0
MAN1B1	31.227084	21.356484	21.635405	27.330245	3.996452	24.039817	3.949135	19.45941
MAN1C1	4.443058	0.411895	0	1.071634	0	0	24.370212	0.390185
MAN2A1	19.21977	1.971685	35.511156	1.081465	7.218833	0.294114	3.403952	1.481343
MAN2A2	22.376343	10.742001	26.3729	11.160932	35.200542	20.027396	35.69917	13.022837
MAN2B1	15.366094	10.904081	0	11.180926	0	19.355221	9.987516	9.022975
MAN2B2	2.944512	4.327966	1.698107	2.578224	11.460813	3.193175	0	0.377318
MAN2C1	6.145326	13.606219	31.396105	11.52599	25.91676	19.978878	3.925196	7.168514
MANBA	0	8.648146	19.893692	3.596251	4.024191	0.736198	10.273027	13.727354
MANBAL	55.811116	81.599507	52.000957	100.586633	18.953796	41.59269	0	37.986014
MANEA	3.586313	3.845902	4.217335	3.287026	1.951381	1.41129	48.169752	5.54945
MANEAL	35.254451	49.900953	0	49.447387	24.660202	84.179537	58.529919	57.340626
MANEALP1	0	0	0	0	0	0	0	0
MANF	0	103.861741	58.915078	38.842269	110.378424	35.245944	82.318326	4.341948
MANSC1	12.151481	35.674744	15.217493	36.60419	11.021265	5.461222	4.673151	7.906516
MAOA	0	4.704388	0	0	0	9.699037	0	6.722495
MAOB	0	0	0	0.289375	0	0	0	0

MAP1A	0.625236	2.211584	1.406915	1.890605	0	3.599562	2.212576	2.755229
MAP1B	17.673001	7.15674	15.647528	13.076685	29.372822	5.085034	6.364123	6.97145
MAP1LC3A	0	7.816287	7.119397	20.378329	14.857286	8.174932	0	3.46326
MAP1LC3B	0	57.164342	0	96.440992	30.439191	131.782796	15.053116	62.29722
MAP1LC3B2	0	0	0	0	0	0.272254	0	0
MAP1LC3BP1	0	0	0	0	0	0	0	0
MAP1LC3P	0	0	0	0	0	0	0	0
MAP1S	22.106483	11.043398	10.012441	17.47825	49.389217	30.279735	78.616667	20.975775
MAP2	12.245741	10.223052	2.269897	4.349552	1.908887	2.369288	0	1.806782
MAP2K1	36.046403	122.481211	39.245428	82.063717	83.452043	127.577306	51.075465	103.905971
MAP2K1P1	0	0	0	0	0	0	0	0
MAP2K2	96.948564	20.716112	77.110641	44.659027	0	35.682657	0	25.710837
MAP2K3	0	64.132122	31.304486	70.724816	0	103.587076	34.205415	45.729283
MAP2K4	11.399733	21.284961	10.227828	11.654098	25.547741	17.078931	61.905791	23.284135
MAP2K4P1	0	0	0	0	0	0	0	0
MAP2K5	91.771824	41.18842	104.94482	48.070514	45.943104	29.427386	58.089617	38.300731
MAP2K6	0.476794	3.144582	0.214668	0.215636	0.630241	1.319893	0	0.939097
MAP2K7	2.360291	20.535381	0	3.027255	15.488444	10.242126	10.249992	2.490066
MAP3K1	5.519713	5.226034	6.205052	5.120305	12.884544	4.777984	7.627257	6.318035
MAP3K10	24.400219	2.821089	2.376054	5.060653	37.445475	6.557415	16.521783	5.882627
MAP3K11	59.238079	117.486051	32.042767	101.164885	27.922413	142.03028	31.436746	87.068402
MAP3K12	0	2.761672	9.855687	3.330757	0	1.663283	0.141104	1.913019
MAP3K13	21.049376	65.746435	49.055215	47.527007	21.934391	38.894768	48.472559	73.25687
MAP3K14	9.622207	10.121406	8.639747	8.466564	6.513581	6.474379	18.117978	6.762367
MAP3K15	1.66884	0	0	0.617659	0	0	0	0.281874
MAP3K19	0	0	0	0	0	0	0	0
MAP3K2	27.179635	47.51419	21.388506	30.170024	34.305322	41.125255	31.120092	101.95802
MAP3K20	37.925369	77.668201	42.202755	63.372836	29.428003	54.653315	124.381153	71.526041
MAP3K21	15.566781	19.218587	15.419129	15.0715	11.457401	14.992787	22.881784	15.522072
MAP3K3	4.080708	18.028706	19.03118	16.311073	10.422623	32.551066	44.978835	22.365368
MAP3K4	16.649521	51.037394	8.242321	24.910645	133.621342	22.987242	10.516614	21.486704
MAP3K5	4.436316	4.789297	5.693951	3.996448	6.194615	4.31676	11.270554	7.043108
MAP3K6	2.292097	3.017751	4.243018	3.624195	36.988946	18.604414	29.214061	10.102229

MAP3K7	22.385932	29.909668	21.935258	22.375164	17.817416	18.50137	24.862267	25.154211
MAP3K7CL	7.729112	3.561714	8.490383	3.047047	3.804657	5.708355	0	4.665673
MAP3K8	11.010069	2.234145	11.759923	2.760972	3.986119	1.827677	0	7.460535
MAP3K9	0	5.654751	1.760144	8.641622	0.669027	17.473766	0	9.487826
MAP4	68.538904	66.614661	39.603996	100.835636	80.972027	94.367625	33.907917	71.66183
MAP4K1	0	2.942359	10.180687	1.120992	0	1.738784	0	0.442177
MAP4K2	14.070554	6.614571	10.737819	10.267066	18.452787	29.256758	25.644824	25.764896
MAP4K3	25.070544	23.930393	18.339146	28.643702	47.184335	18.343678	3.895824	26.298562
MAP4K4	56.192861	102.674067	55.038016	108.039985	31.154571	115.163903	93.089054	98.492107
MAP4K5	0	6.734601	0	10.653607	0	0	0	0
MAP6	0	0.539357	2.047902	0.625642	0	0.989347	0	0.602813
MAP6D1	0	3.357842	0	1.203434	0	0.744907	0	1.491773
MAP7	16.317451	16.986773	16.450733	23.827208	32.99516	36.205638	12.406204	20.532201
MAP7D1	44.495891	98.607779	34.394454	102.750846	108.922614	292.490052	81.137945	185.621404
MAP7D2	0.841325	2.416554	1.37471	2.256838	0	1.001628	0	0.135159
MAP7D3	15.004231	23.167505	22.488726	25.755273	20.733536	41.312292	9.860994	30.49106
MAP9	20.502181	12.191293	14.981105	7.228985	6.118689	2.507438	5.435195	8.329234
MAPK1	49.019312	79.425562	102.190312	64.031016	166.395731	96.314872	142.755474	169.359717
MAPK10	0	3.860022	6.144451	3.628271	0	2.794329	5.903754	4.85651
MAPK11	7.08173	8.213644	5.067442	8.052571	0	6.534164	0	5.955234
MAPK12	19.869876	15.708535	14.278383	16.449951	5.562699	34.16888	15.492437	37.23367
MAPK13	17.475369	41.257888	19.870155	36.095859	82.611198	30.365733	9.647835	23.297781
MAPK14	26.059339	25.094182	33.670516	27.767252	0	23.067788	45.070665	33.076539
MAPK15	0	1.613908	3.97246	2.535183	0	0.662516	0	0.226255
MAPK1IP1L	51.743591	107.178671	46.580875	149.232978	161.247302	153.165985	15.057402	116.457109
MAPK3	68.917116	25.801501	66.258707	32.034544	37.148559	33.066411	28.043466	23.813611
MAPK4	0	0	0	0	0	0.053068	0	0.375903
MAPK6	88.137479	120.925162	41.824408	90.843935	69.439474	69.193233	25.367252	180.823793
MAPK6P1	0	0	0	0	0	0	0	0
MAPK6P2	0	0	0	0	0	0	0	0
MAPK6P3	0	0	0	0	0	0	0	0
MAPK6P4	0	0	0	0	0	0	0	0
MAPK6P5	0	0	0	0	0	0	0	0

MAPK6P6	0	0	0	0	0	0	0	0
MAPK7	7.440333	15.30191	18.544004	17.99135	43.929209	38.591306	5.117072	29.445063
MAPK8	76.89933	111.159899	76.933522	68.574198	15.658983	40.793958	67.419645	110.091766
MAPK8IP1	3.298846	7.114469	4.926634	6.328581	4.643809	10.459299	7.748116	3.935774
MAPK8IP1P1	0	0	0	0.12141	0	0	0	0
MAPK8IP1P2	0	0	0	0	0	0	0	0
MAPK8IP2	0.571376	0.885121	0	0.852159	0	0.343638	0	0.114581
MAPK8IP3	6.01079	4.085753	4.143938	4.703631	0.587309	5.033802	5.93241	2.173422
MAPK9	49.921173	73.261307	12.532833	53.775186	29.288278	49.804007	21.059802	83.795697
MAPKAP1	74.781447	153.115225	66.659468	115.861032	85.511449	114.453764	170.628825	150.21651
MAPKAPK2	0	5.0359	3.75824	6.284903	4.482654	7.659961	0	6.995281
MAPKAPK3	21.771276	30.838389	28.001941	43.46156	29.436181	44.991342	68.204777	38.273995
MAPKAPK5	15.426211	20.485154	47.790438	19.876002	4.847699	54.970625	48.268534	30.43478
MAPKAPK5P1	0	0	0	0	0	0	0	0
MAPKBP1	13.919678	31.162086	13.367057	34.459895	55.101356	46.116954	11.461683	28.909461
MAPRE1	327.332808	304.191379	317.84185	230.363269	124.472894	92.180784	126.383703	153.265673
MAPRE1P1	0	0	0	0	0	0	0	0
MAPRE1P2	0	0	0	0	0	0	0	0
MAPRE1P3	0	0	0	0	0	0	0	0
MAPRE2	30.321668	27.606423	20.744413	11.7342	21.753337	19.732412	44.366787	18.033314
MAPRE3	0	6.993198	17.378456	14.487081	2.562947	7.573958	23.062096	9.531453
MAPT	1.708554	3.051071	2.593645	9.719139	1.290646	2.312141	0	1.979989
MARCHF1	14.257554	21.302261	1.639219	15.138833	0.362727	1.932089	0	3.590664
MARCHF10	1.096937	0.69158	0	0.166594	0	0	0	0.040344
MARCHF11	0	0	0	0	0	0	0	0
MARCHF2	10.407836	4.516187	7.98288	3.273067	0	5.671375	0	1.570883
MARCHF3	0	5.306816	0	3.888353	0	0.586065	0	3.712925
MARCHF4	0	1.347187	0	0.310077	4.057697	9.328765	2.928046	11.764994
MARCHF5	23.81819	58.826781	47.531093	53.338956	26.459559	34.88414	39.72019	46.780048
MARCHF6	73.068685	38.55727	98.969646	39.511758	98.239337	11.554368	50.546898	39.102479
MARCHF7	50.278341	52.809499	86.63824	35.319931	69.651733	30.336505	70.414682	99.669811
MARCHF8	43.830459	47.018404	24.104701	45.829962	70.438585	46.431928	0.158716	39.356644
MARCHF9	13.369413	39.77569	8.075801	30.606773	60.207507	86.180308	10.675263	67.175443

MARCKS	75.111858	68.298426	92.891855	81.684957	124.086432	118.821448	97.579115	145.382907
MARCKSL1	43.956784	110.686835	63.825124	114.682547	82.25172	180.72172	104.961304	176.027195
MARCKSL1P1	0	0	0	0	0	0	0	0
MARCKSL1P2	0	0.981377	0	0	0	0	0	0.649474
MARCO	0	0	0	0	0	0	0	0
MARCOL	0	0	0	0	0	0	0	0
MARF1	14.883156	47.043352	14.358747	35.713069	17.330049	41.883002	47.068395	45.998591
MARK1	3.912974	3.76773	0.922704	3.109852	0.629129	4.521621	0	3.505117
MARK2	66.497611	61.301105	21.404528	51.521851	43.025218	91.172257	59.847923	99.716768
MARK2P10	0	0	0	0	0	0	0	0
MARK2P11	0	0	0	0	0	0	0	0
MARK2P12	0	0	0	0	0	0	0	0
MARK2P13	0	0	0	0	0	0	0	0
MARK2P14	0	0	0	0	0	0	0	0
MARK2P15	0	0	0	0	0	0	0	0
MARK2P16	0	0	0	0.099271	0	0	0	0
MARK2P17	0	0	0	0	0	0	0	0
MARK2P18	0	0	0	0	0	0	0	0.122698
MARK2P19	0	3.119686	0	0.919163	0	0	0	0
MARK2P21	0	0	0	0	0	0	0	0
MARK2P3	0	0	0	0	0	0.932082	0	0
MARK2P4	0	0	0	0	0	0	0	0
MARK2P5	0	0	0	0	0	0	0	0
MARK2P6	0	0	0	0	0	0	0	0
MARK2P8	0	0	0	0	0	0	0	0
MARK2P9	0	0	0	0	0	0	0	0
MARK3	34.954862	77.037506	28.018012	69.534553	17.111196	62.636797	58.727356	81.707017
MARK3P1	0	0	0	0	0	0	0	0
MARK3P3	0	0	0	0	0	0	0	0
MARK4	20.62828	37.268681	33.382765	35.180497	23.421885	68.856522	34.943856	77.645683
MARS1	185.247092	127.793859	127.983549	144.35219	65.732879	38.726409	138.202891	41.674951
MARVELD1	20.80899	59.770876	22.382963	55.554143	40.232855	66.683697	41.380175	52.988558
MARVELD2	21.048237	42.048897	12.825905	50.721085	34.179202	99.51769	26.386182	81.191342

MARVELD3	20.616699	27.936884	5.320211	25.399631	18.655256	57.174278	13.920315	29.360965
MAS1	0	0	0	0	0	0	0	0
MAS1L	0	0	0	0	0	0	0	0
MAS1LP1	0	0	0	0	0	0	0	0
MASP1	0	0	0	0	0	0	0	0
MASP2	0	0	0	0	0	0.077862	0	0
MAST1	0.67502	2.729395	4.244261	3.120673	0	1.560802	0	0.391051
MAST2	21.885163	27.675602	10.712506	25.416366	58.994537	45.684785	59.206983	35.660752
MAST3	3.862401	3.870478	2.479826	5.303277	5.102481	11.159182	10.423408	5.891938
MAST4	19.28702	27.18004	18.242201	77.727082	18.080359	32.448762	12.465006	23.525658
MASTL	12.174569	4.551818	3.527253	6.275159	19.718659	6.385588	40.319227	4.81523
MAT1A	0	0.252551	10.474226	0.264237	0	0.049132	0	0.039584
MAT2A	105.599621	76.97842	98.616199	83.632768	102.34701	86.189876	89.812885	106.82629
MAT2AP1	0	0	0	0	0	0	0	0
MAT2B	56.66083	72.509297	41.111907	50.374817	63.900079	44.390875	32.882264	75.984753
MATCAP1	0	17.318079	5.295138	10.316391	1.658928	10.3298	0	9.746791
MATCAP2	6.214697	17.510219	12.056568	8.078638	5.043184	18.703777	19.282345	18.441332
MATK	17.17695	8.775129	10.625081	9.482202	0	0.268123	0	0.654662
MATN1	0	0.341446	0	0.168785	0	0.801043	0	0.508717
MATN2	13.396988	7.512185	4.431396	4.929885	20.153641	21.307412	97.941494	18.266837
MATN3	0	1.751607	0	1.3104	2.452644	1.992048	0	1.440147
MATN4	0	0	0	0	0	0	0	0
MATR3	167.821596	150.565343	140.682022	132.44276	285.564382	107.430653	313.291215	194.286485
MATR3P1	0	0	0	0	0	0	0	0
MATR3P2	0	0	0	0	0	0	0	0
MATR3P3	0	0	0	0	0	0	0	0
MAU2	9.713443	8.253382	20.365221	7.206195	14.968351	8.921502	4.446509	10.236563
MAVS	33.027766	29.62552	28.058714	29.272068	40.840283	42.219164	25.686299	36.272008
MAX	71.796477	57.509143	54.827284	89.985201	54.756276	131.503442	5.567768	114.505298
MAZ	38.170651	82.915863	6.798378	80.430566	115.615054	198.30772	185.5685	177.719114
MB	0	0	0	0.264264	0.963463	1.201281	0	0.14947
MB21D2	21.893016	20.555396	13.732681	11.98047	11.557213	12.708489	3.989664	17.65258
MBD1	37.233447	72.114427	27.781712	91.864385	57.703028	139.367156	83.2825	103.793847

MBD2	31.645977	160.098231	70.67037	133.459961	242.851347	178.649435	69.075358	150.286063
MBD3	17.543322	25.948091	8.988219	19.128454	28.020989	119.393781	55.703914	96.021053
MBD3L1	0	0	0	0	0	0	0	0
MBD3L2	0	0	0	0	0	0	0	0
MBD3L5	0	0	0	0	0	0	0	0
MBD4	14.434202	33.875842	21.046411	31.271275	20.424482	29.670747	0	26.307883
MBD5	14.928979	20.264935	2.804794	19.230817	3.431856	9.404333	6.606707	10.766933
MBD6	14.903155	19.094987	22.531585	18.14771	6.007442	13.143155	9.736965	9.198167
MBIP	22.22463	13.103525	30.319125	9.517334	13.115224	5.26872	33.047174	7.400364
MBL1P	0	0	0	0	0	0	0	0
MBL2	0	0	0	0	0	0	0	0
MBL3P	0	0	0	0	0	0	0	0
MBLAC1	0	2.391258	0	0.580294	0.795265	3.987239	0	1.591592
MBLAC2	2.310015	3.681777	5.996612	2.384842	3.457172	1.560385	7.49752	2.64489
MBNL1	68.662623	340.700774	51.711756	299.163157	173.816681	409.919186	94.809014	681.643545
MBNL2	43.348335	144.522443	49.900002	142.354387	44.488005	51.361103	137.962822	91.784836
MBNL3	3.675132	5.712837	2.314828	5.825863	2.428373	10.582708	1.448188	18.878692
MBOAT2	65.378202	57.101559	39.966796	30.761765	48.61752	15.150052	42.975012	46.277013
MBOAT7	15.848605	41.369402	41.258211	46.125643	67.16851	92.331514	20.184496	74.109598
MBP	26.376626	78.966334	26.789502	82.149899	25.790462	189.924268	19.62159	102.123398
MBTD1	9.70896	5.150246	12.375863	7.989935	7.874725	4.915445	18.432346	7.786834
MBTPS1	57.419849	141.906261	60.337723	147.541985	120.373018	177.663342	79.599397	190.943323
MBTPS2	11.092127	5.199909	7.303724	3.166982	15.038946	2.981633	0	8.258445
MC1R	5.289816	0.608381	0	0.85968	0	1.083006	0	0.961901
MC2R	0	0	0	0	0	0	0	0
MC3R	0	0	0	0	0	0	0	0
MC4R	0	0	0	0.199695	0	0.101531	0	0
MC5R	0	0	0	0	0	0	0	0
MCAM	118.631004	126.547266	151.488196	178.971756	12.706053	18.045699	51.086561	9.01323
MCAT	0	7.675758	0	7.511303	0	9.278008	0	9.730052
MCC	14.392308	73.029927	0	127.587114	14.78066	123.005615	24.902195	61.279601
MCCC1	31.930293	53.420382	130.534746	36.602105	22.89999	34.37122	122.214672	15.533931
MCCC2	56.801584	43.601425	90.527573	82.541498	77.519139	69.988944	33.647426	67.508237

MCCD1	0	0	0	0	0	0	0	0
MCCD1P1	0	0	0	0	0	0	0	0
MCCD1P2	0	0	0	0	0	0	0	0
MCEE	9.95332	35.614918	4.316101	24.510518	2.579595	13.212269	0	13.82426
MCEMP1	0	1.24762	0	0	0	0	0	0
MCF2	0	0.503115	0	0.061369	0	0	0	0
MCF2L	0	1.579497	0.875185	2.399178	0	3.06911	0	0.836107
MCF2L2	6.599403	0.961281	12.755959	1.957939	0	1.442843	0.268948	0.057443
MCF2L2P1	0	0	0	0	0	0	0	0
MCFD2	159.109398	32.791679	16.799192	35.214157	40.456478	27.6443	114.134926	54.532864
MCFD2P1	0	0	0	0	0	0	0	0
MCHR1	0	0	0	0.378098	0	0	0	0.118099
MCHR2	0	0	0	0	0	0	0	0
MCIDAS	0	1.190177	0	0.384408	14.427009	0	0	1.768606
MCL1	175.750429	129.330843	160.043054	125.703035	132.847974	147.837028	181.466584	115.555016
MCM10	7.236348	11.355088	0	13.67605	5.1629	21.254426	144.146111	21.874827
MCM2	126.062006	70.831905	148.688622	103.04246	123.270839	137.643514	64.546861	107.947927
MCM3	0	24.416955	8.846016	17.026733	20.505422	18.2785	0	9.793074
MCM3AP	24.993404	87.204788	25.788209	75.466087	37.310302	131.92191	34.611477	152.729306
MCM4	200.250515	192.620426	248.896353	170.717496	424.402176	248.718619	220.07922	225.244363
MCM5	48.981998	53.004267	69.792148	51.730546	213.443442	57.202031	105.658547	42.105674
MCM6	75.941811	103.539631	55.456714	80.768981	124.958364	86.708232	60.363723	101.949572
MCM7	0	0	0	0.856821	0	0.559705	8.710354	0
MCM8	22.115547	58.742945	25.527264	42.104293	26.729325	45.025312	6.054589	72.617763
MCM9	0	12.710026	15.837595	9.011472	4.327685	11.481053	9.817673	18.468256
MCMBP	22.731282	19.045069	5.796092	11.366675	11.192135	3.575774	0	22.534035
MCMDC2	3.058054	1.312474	0	0.164706	0	0.094472	0	0.511067
MCOLN1	6.842663	3.660307	16.767664	1.839965	28.856575	2.217394	0	4.519626
MCOLN2	1.101922	12.092655	25.813884	15.412296	6.689449	2.580334	15.844328	11.446231
MCOLN3	9.600783	3.633876	4.438316	2.400639	2.216907	1.090924	4.426634	2.634142
MCPH1	12.201066	7.248388	8.734621	7.485595	4.293649	2.96059	9.820494	3.913718
MCRIP1	18.766646	25.238282	0	10.670798	22.048046	43.272981	0	17.342381
MCRIP2	36.516871	48.275195	12.224339	39.492373	7.05321	63.979455	0	57.498345

MCRIP2P1	0	0	0	0	0	0	0	0.403296
MCRIP2P2	0	0	0	0	0	0	0	0
MCRS1	26.211338	34.40885	19.396287	39.325712	39.727004	40.948738	26.52374	32.217387
MCTP1	9.406887	10.61961	14.102532	9.291008	19.203952	6.448851	0	10.145154
MCTP2	3.894112	15.733725	17.519383	12.623245	44.120385	26.513292	13.7123	30.418652
MCTS1	117.096089	106.759196	61.619742	31.327186	117.61653	16.591473	40.757552	48.287941
MCU	0	6.953631	13.012512	10.271661	0	7.739928	51.44152	5.370442
MCUB	13.86211	23.345361	28.616402	14.941812	5.280678	13.717416	1.549324	32.606271
MCUR1	0	20.683002	18.528206	6.707775	46.4793	5.248612	9.747103	27.838325
MCUR1P1	0	0	0	0	0	0.485918	0	2.273002
MCUR1P2	0	0	0	0	0	0	0	0
MDC1	30.278994	28.797622	15.458799	37.599121	50.871543	42.823516	23.90352	26.332067
MDFI	24.453193	59.392795	23.727335	71.584935	17.310657	109.708887	8.820736	76.851998
MDFIC	0	13.593956	10.814281	5.622894	0	11.829482	0	30.058962
MDGA1	11.286554	5.458362	0.543242	3.477115	0.239293	6.790421	0	2.674138
MDGA2	0	0.171075	0	0.107215	0	0	0	0
MDH1	193.465017	166.606716	148.376078	117.933679	141.641794	122.469364	139.155602	193.992584
MDH1B	5.853338	2.693274	0	1.83946	0	1.104838	0.484274	1.207848
MDH1P1	0	0	0	0	0	0	0	0
MDH1P2	0	0	0	0	0	0	0	0
MDH2	118.730809	214.366565	150.732699	206.707258	236.034323	231.05482	111.39817	182.688103
MDK	0	13.463209	8.18698	33.92858	5.19914	23.01197	0	11.376979
MDM1	35.057754	14.113348	0	13.124302	24.467136	8.834213	47.594157	12.518302
MDM2	31.060622	102.549717	76.000609	62.628496	124.190892	103.596013	341.300051	201.275856
MDM4	8.625476	29.744621	7.796488	16.320779	17.664912	4.746575	19.315746	14.8974
MDM4P1	0	0	0	0	0	0	0	0
MDN1	1.833309	9.463299	10.084131	6.019698	4.735681	3.482263	26.505425	12.392627
MDP1	0	17.449567	0	25.668434	16.812009	20.429668	0	23.954944
ME1	31.975023	36.812425	18.813395	20.178224	27.170169	13.707521	29.636273	27.538099
ME2	64.621037	55.329985	45.371725	30.292886	84.457486	21.159261	112.954259	61.420171
ME2P1	0	0	0	0	0	0	0	0
ME3	0	8.206206	12.983859	8.157544	24.702709	5.554239	0	12.749442
MEA1	37.22992	146.360822	77.684636	132.786795	40.845251	169.571054	67.358241	96.923165

MEAF6	30.537437	30.152965	37.296497	37.691487	47.103563	30.98058	23.276375	28.304498
MEAF6P1	0	0	0	0	0	0	0	0
MEAK7	77.750362	108.409898	33.769517	152.439425	29.943351	103.671054	20.98506	87.619436
MECOM	17.079262	44.1665	29.81571	52.068053	92.382219	9.898171	3.122528	21.649356
MECP2	25.195341	48.030388	17.837928	49.659298	4.899244	58.485064	62.234453	32.350563
MECR	8.124373	6.198024	14.007868	7.627427	20.96335	10.038143	22.802596	15.445164
MED1	24.570661	29.459046	34.583852	30.451328	25.137124	29.588842	40.452458	26.89054
MED10	30.921469	72.852996	45.575146	71.120587	9.932307	35.078244	10.711321	38.076299
MED11	33.817588	28.073333	8.47772	32.650504	3.51317	38.249094	0	49.801166
MED12	0	5.435797	10.104325	0.766278	0	4.622465	13.224872	2.143039
MED12L	2.027819	3.35941	1.612009	4.916297	4.187199	7.381402	3.213301	17.922271
MED13	51.581153	97.503468	38.647473	110.67256	58.269171	92.314588	88.57026	133.190265
MED13L	23.28566	45.645478	38.683643	56.431221	61.738222	120.231168	94.780569	124.12841
MED13P1	0	0	0	0	0	0	0	0
MED14	10.086338	71.166464	23.497161	53.72208	46.82271	52.151576	133.961676	95.748287
MED14P1	0	0	0	0	0	0	0	0
MED15	14.250562	26.838163	16.059956	34.309691	62.059569	63.165546	41.735409	45.683746
MED15P1	0	0	0	0	0	0	0	0
MED15P3	0	0	0	0	0	0	0	0
MED15P4	0	0	0	0	0	0	0	0
MED15P5	0	0	0	0	0	0	0	0
MED15P6	0	0	0	0	0	0	0	0
MED15P7	0	0	0	0	0	0	0	0
MED15P8	0	0	0	0	0	0	0	0
MED15P9	0	0	0	0	0	0	0	0
MED16	23.745368	31.791551	92.067592	28.645899	37.834085	58.601841	113.643124	46.244329
MED17	95.91855	68.771632	71.60723	44.814788	26.811832	20.422927	50.943375	28.952901
MED18	17.095658	17.169754	6.83437	16.426243	6.05822	19.366582	63.292319	28.213392
MED19	0	0	0	0.291419	0	0	0	0
MED20	20.606279	17.092054	9.832469	14.697337	30.004229	20.660525	5.998854	24.868981
MED21	42.0411	31.665109	21.570439	24.895774	17.400535	12.640743	0	37.217426
MED22	22.248575	44.040569	16.783865	42.290543	9.038543	32.517054	6.021579	25.521647
MED23	7.939063	9.917569	8.631673	8.035216	6.333166	6.556376	13.983161	8.673433

MED24	21.806009	24.900053	77.247044	37.537739	57.708779	37.253281	69.496336	32.211957
MED25	16.134669	15.755627	4.819714	13.647265	24.348652	37.390401	18.475626	20.929726
MED26	0	0.457027	0	0.878618	5.632041	15.0253	0	0.785459
MED27	50.933406	30.491054	18.645692	29.411878	36.538172	27.705563	91.297606	22.210891
MED28	31.493718	14.515369	31.757563	14.122423	11.573898	8.848955	2.018	6.472512
MED28P1	0	0.635385	0	0	0	0	0	0
MED28P2	0	0	0	0	0	0	0	0
MED28P3	0	0	0	0	0	0	0	0
MED28P4	0	0	0	0	0	0	0	0
MED28P5	0	0	0	0	0	0	0	0
MED28P6	0	0	0	0	0	0	0	0
MED28P7	0	0	0	0.21177	0	0	0	1.141442
MED28P8	0	0	0	0	0	0	0	0
MED29	17.146247	12.518041	13.376841	13.486834	31.502082	23.311327	43.299365	22.823139
MED30	11.799076	20.874524	38.153164	22.47416	39.241441	12.34818	0	16.411033
MED31	6.541995	13.592779	13.288722	19.108998	4.020985	9.708702	39.659518	29.354755
MED4	0	43.88173	31.529192	36.066128	53.293179	15.349052	0	36.250376
MED6	0	33.534986	46.382623	28.394629	46.767136	22.783743	55.730253	56.248937
MED6P1	0	0	0	0	0	0	0	0
MED7	19.813467	15.889004	10.739082	10.400247	18.128064	10.800491	15.160657	10.35904
MED8	10.598302	51.966163	25.229236	54.088902	40.475766	76.435017	33.345374	57.572299
MED9	6.224816	12.014637	6.954278	16.034657	18.320551	18.926446	18.857269	15.348628
MEDAG	0	0	0	0	0	0	0	0
MEF2A	15.206049	31.966738	0	25.07024	11.633566	32.5309	32.451441	55.898466
MEF2AP1	0	0	0	0	0	0	0	0
MEF2B	0	2.60474	0	2.355474	0.850201	1.465456	0	1.425265
MEF2C	0	1.752533	1.365463	2.365693	0	0	0	0
MEF2D	19.11227	72.600808	19.492044	101.362995	13.298758	103.287678	4.255723	89.613941
MEFV	3.697709	0	0	0.027676	0	0	0	0.045681
MEGF10	0	0	0	0.074606	0	0	0	0
MEGF11	0	0.298705	0	0.02845	0	0	0	0
MEGF6	0	1.78278	2.34321	0.969039	1.95063	1.915562	0	0.942771
MEGF8	1.753936	3.752296	3.922491	2.746251	0.98887	9.135862	22.443042	5.708785

MEGF9	5.668586	5.628991	3.243113	4.288917	4.901939	9.381204	10.42674	11.095648
MEI1	0	0.77939	0	0.035203	0	0.795611	0	0
MEI4	0	0	0	0	0	0	0	0
MEIG1	0	0	0	0.702887	0	0	0	0
MEIKIN	0	0	0	0	0	0	0	0
MEIOB	0	0	0	0	0	0	0	0
MEIOC	1.509259	1.381375	7.327588	2.190702	1.500406	0.14612	0	1.440284
MEIS1	53.376456	25.458042	0.84475	20.840473	6.463969	22.470098	61.633488	14.516774
MEIS2	18.35611	54.604237	31.631769	92.184375	30.494013	28.197228	0	12.112462
MEIS3	0	2.143228	2.750211	2.641457	16.734054	3.955923	46.864298	4.819858
MEIS3P1	0	3.453936	0	5.253487	0	21.69013	0	10.249528
MEIS3P2	0	0.195782	0	0.691419	0	4.486241	0	2.208678
MELK	46.025122	71.617871	33.071048	48.611093	109.306413	63.170749	200.647851	100.864458
MELTF	17.819478	12.595666	28.449373	29.739222	5.059635	12.252437	6.898057	5.242764
MEMO1	0	15.723668	39.207054	16.615823	2.170796	17.108139	66.095777	23.639908
MEMO1P1	0	0	0	1.051108	0	0	0	0
MEMO1P2	0	0	0	0	0	0	0	0
MEMO1P3	0	0	0	0	0	0	0	0
MEMO1P4	0	0	0	0	0	0	0	0
MEMO1P5	0	0	0	0	0	0	0	0
MEN1	0	8.403935	17.411833	6.260752	31.922305	22.054531	64.525116	13.449692
MEOX1	0	0.384662	0	0.246559	0	0.468336	0	0.356596
MEOX2	0	0	0	0	0	0	0	0
MEP1A	0	0	0	0.028345	0	0	0	0.234
MEP1AP1	0	0	0	0	0	0	0	0
MEP1AP4	0	0	0	0	0	0	0	0
MEP1B	0	0	0	0	0	0	0	0
MEPCE	19.114234	40.197992	27.881506	34.4504	14.419764	49.028337	7.961996	48.353442
MEPE	0	0	0	0	0	0	0	0
MERTK	8.703946	0	2.025425	0.250223	3.580079	0.683679	0	0
MESD	43.848449	49.148399	33.474272	51.762599	22.441214	43.39964	10.641573	34.347611
MESDP1	0	0	5.599447	0	0	0	0	0
MESP1	0	0	0	4.675978	0	9.76769	0	0

MESP2	0	0.546496	0	0.605264	0	0.213134	0	0.584529
MEST	85.821672	114.422879	92.348097	89.801428	67.253255	80.910448	325.896949	141.295553
MESTP1	0	0	0	0	0	0	0	0
MESTP2	0	0	0	0	0	0	0	0
MESTP3	0	0	0	0	0	0	0	0
MESTP4	0	0	0	0	0	0	0	0
MET	0	27.574596	17.923232	125.548876	40.994288	208.962268	62.239721	175.818317
METAP1	56.680451	52.348313	58.636699	46.07917	134.557693	34.12476	78.204039	56.955499
METAP1D	60.931282	9.971083	0	7.068086	0.871017	6.301319	5.345381	10.934801
METAP2	124.232484	230.614961	164.225888	262.572015	403.852917	629.605473	334.872251	449.062533
METAP2P1	0	0	0	0	0	0	0	0
METRN	14.441721	17.956863	18.745704	14.936667	1.917636	12.211517	28.542081	2.287875
METRNL	58.641675	61.887665	21.110252	53.226036	40.255069	39.066775	42.626438	25.732483
METTL1	13.818187	15.624349	29.558454	23.974828	43.647582	40.458944	77.383268	33.243162
METTL13	14.436016	77.359125	17.288556	58.287213	5.487457	69.1155	6.744889	58.590128
METTL14	49.487805	37.513006	19.223001	30.450513	57.414437	16.560515	2.863262	32.190838
METTL15	7.832071	3.586461	4.218402	4.071277	23.384377	5.584805	9.722512	11.562843
METTL15P1	0	2.533937	0	1.942697	0	0	0	4.099222
METTL15P3	0	0	0	0	0	0	0	0
METTL16	9.076206	20.794274	12.639594	20.499808	36.71703	32.920373	91.937373	26.448868
METTL17	45.492796	24.193312	0	24.444386	83.967364	24.548905	57.280105	22.28107
METTL18	15.221822	27.75015	22.548953	22.805268	12.659601	14.903813	41.667205	25.936891
METTL1P1	0	0	0	0	0	0	0	0
METTL21A	18.168824	30.553888	15.158064	18.67945	6.562399	23.128036	0	36.748411
METTL21AP1	0	0	0	0	0	0.979406	0	0
METTL22	18.097651	10.953492	10.974116	13.123938	11.902283	19.024465	65.154122	17.632341
METTL23	42.513128	78.037227	18.051419	52.055945	57.825082	29.613939	2.029837	42.056326
METTL24	0	0	0	0	0	0	0	0
METTL25	8.187272	3.151562	5.686418	1.40563	0	0.387741	0	2.260514
METTL25B	60.342392	77.942174	42.158032	109.440671	21.556242	76.026542	0	42.310295
METTL26	48.663565	82.6674	72.612201	78.959116	42.648256	99.668443	81.42166	86.518429
METTL27	4.268585	7.488714	3.757153	7.828951	1.120013	2.670617	0	3.315501
METTL2A	26.236257	17.176883	23.973177	18.277352	29.007997	7.760155	0	13.503195

METTL2B	17.703114	21.212093	4.880444	12.028155	3.308356	6.510965	15.749831	10.187818
METTL3	26.318762	20.935286	25.062376	22.503844	44.405097	23.486749	26.34062	19.780829
METTL4	1.844641	31.840651	6.618332	29.690731	5.844524	28.789571	96.448802	33.500642
METTL5	93.824743	109.040172	87.516446	50.668679	104.188107	90.513351	123.678789	106.072107
METTL5P1	0	0	0	0	0	0	0	0
METTL5P2	0	0	0	0	0	0	0	0
METTL5P3	0	0	0	0	0	0	0	0
METTL5P4	0	0	0	0	0	0	0	0
METTL6	3.453478	8.470122	0	13.292876	9.52271	3.317498	32.578261	16.707281
METTL8	4.348816	13.853491	0	11.126197	25.625833	23.937524	192.461789	48.929582
METTL8P1	0	0	0	0	0	0	0	0
METTL9	59.89956	107.159536	95.969777	78.541422	23.535985	52.209786	51.479106	104.042519
MEX3A	3.443453	8.816937	4.865514	8.801528	2.209771	9.837442	1.199339	3.549873
MEX3B	0	1.75648	0	1.837671	0	1.366832	0	0.550541
MEX3C	2.390074	10.516707	0	10.222703	5.615242	18.047607	0	12.957319
MEX3D	0	0	0	3.262803	2.10755	10.916925	40.75912	9.160308
MFAP1	37.292984	55.986685	42.387379	65.270636	49.609237	73.247037	42.280994	52.902747
MFAP1P1	0	0	0	0	0	0	0	0
MFAP2	0	0	0	14.616108	1.052207	0.083465	0	7.156402
MFAP3	25.070004	17.771491	16.24159	24.397023	34.359811	14.79338	53.733792	32.074536
MFAP3L	0	0.04801	7.924821	1.075889	0.147566	0.306346	0	0.282727
MFAP4	0	0	0	0	0	0	0	0
MFAP5	0	0	0	0	15.758083	33.474147	28.272708	33.764332
MFF	57.237417	182.948775	57.373324	131.533196	68.335745	125.090916	98.906965	225.915716
MFFP1	0	0	0	0	0	0	0	0
MFFP2	0	0	0	0	0	0	0	0
MFFP3	0	0	0	0	0	0	0	0
MFGE8	12.590472	11.810396	8.957616	13.591153	33.81786	11.330755	32.164083	9.002272
MFHAS1	9.12812	13.679753	17.388811	16.001578	10.317859	50.811945	5.468623	43.749783
MFN1	61.607396	76.491563	45.15932	68.349375	80.703239	31.891618	22.106859	73.881513
MFN1P1	0	0	0	0	0	0	0	0
MFN2	0	29.471084	0	37.368903	37.418496	66.796992	63.727909	46.860707
MFNG	4.028535	0	0	0.301646	0	2.132285	0	0.958595

MFRP	0	0	0	0.025676	0	0	0	0
MFSD1	51.873329	8.916537	16.186607	7.315901	71.352509	4.739165	25.118522	12.280053
MFSD10	20.303325	21.95918	30.486424	23.307773	13.840691	20.974778	31.610392	9.704442
MFSD11	21.724921	10.736366	43.1723	16.231003	30.293679	5.397114	19.575163	6.034355
MFSD12	17.633784	39.282065	22.617418	43.410629	58.880038	23.192571	42.275474	21.782719
MFSD13A	5.906043	20.166438	18.309715	21.018945	22.154605	7.066301	0	13.764314
MFSD14A	47.064144	36.378658	42.767466	24.61531	54.619213	12.045795	11.82786	17.839672
MFSD14CP	38.858601	49.517116	7.076882	38.448752	21.126762	32.640121	2.252285	25.648179
MFSD1P1	0	0	0	0	0	0	0	0
MFSD2A	5.464519	0	6.446803	0.686707	33.324089	5.663018	0	6.2403
MFSD2B	0	1.404359	2.928091	1.100082	0.867912	1.125514	0	0.537803
MFSD3	10.091026	6.09623	10.249823	4.598105	1.819836	4.41663	0	2.330236
MFSD4A	1.780052	0.114259	1.696355	0.337661	0	0.682152	0	0.035755
MFSD4BP1	0	0	0	0	0	0	0	0
MFSD5	5.557001	3.420068	0	7.850129	0	7.58679	0	0.685184
MFSD6	9.973589	26.049344	15.994047	28.030228	34.941485	36.095936	20.935499	46.373274
MFSD8	9.402755	6.594996	9.273963	2.364639	8.326918	2.272149	37.11802	3.965435
MFSD9	24.175092	15.348501	2.217169	11.054023	55.701765	17.534587	5.421411	18.294603
MGA	4.594419	3.547651	7.702913	4.801438	0	5.960046	10.061369	9.015794
MGAM	0	0.213802	0.844801	0.370425	0	0	0	0.042145
MGAM2	0	0	0	0	0	0	0	0
MGARP	0	1.513059	0	0.083145	0	0	0	0
MGAT1	33.937111	96.813065	86.111144	95.439137	7.753212	111.73143	0.439527	114.296157
MGAT2P1	0	0	0	0	0	0	0	0
MGAT2P2	0	0	0	0	0	0	0	0
MGAT3	0	0.051968	0	0.251385	0	1.928675	0	0.218396
MGAT4A	0	0	21.645918	0.170062	0.236074	0.077179	0	1.033338
MGAT4B	86.134273	233.791205	109.399353	331.028723	97.536187	319.839007	114.502592	234.434937
MGAT4C	0	0	0	0	0	0	0	0
MGAT4D	0	0	0	0	0	0	0	0
MGAT4EP	0	0	0	0.318925	0	0	0	0
MGAT4FP	0	0	0	0	0	0	0	0
MGAT5	19.086997	54.057021	15.112958	54.890368	14.144737	56.185756	32.945104	59.920588

MGAT5B	7.686259	3.382915	3.289138	3.507554	5.436746	5.843482	4.757735	2.36383
MGLL	0	0	0	0.611574	32.77579	0.56549	107.477306	0
MGME1	41.566019	67.734424	33.656804	68.695495	49.092365	62.204342	86.965035	82.534552
MGMT	8.744028	30.147668	25.851086	33.987362	2.408148	29.490984	0	27.589786
MGP	0	0	0	0.146202	0	1.602543	0	0
MGRN1	33.673191	31.894934	15.854752	34.558182	25.649744	63.843317	68.732673	43.766984
MGST1	491.229018	396.40239	517.451097	403.214711	437.731729	181.851174	228.875836	204.91476
MGST2	15.759583	28.065714	34.627316	25.559449	35.572534	17.420091	0	32.396676
MGST3	83.301261	44.344987	66.279498	53.585158	95.763478	35.830067	201.109898	49.380542
MIA	0	0	0	0	0	0	0	0
MIA-RAB4B	0	0	0	0	0	0	0	0
MIA2	38.269896	34.161667	29.132129	25.33525	5.161335	10.907471	0.326492	18.179414
MIA3	66.484633	25.202416	44.987917	53.833465	35.227646	33.385283	37.144424	23.189858
MIB1	12.081902	28.90793	20.827482	29.731229	21.095137	18.976086	4.67359	27.213134
MIB2	31.752641	12.661355	19.063715	13.48175	4.970109	9.540376	9.672005	8.612811
MICA	36.55962	25.869703	25.583348	28.312633	56.265269	30.94804	36.903731	23.327937
MICAL1	5.327099	3.200922	3.819482	1.319819	0.28123	3.003502	4.142682	2.737515
MICAL2	72.097201	121.810047	86.229587	146.461749	125.493298	273.902845	214.28354	227.936028
MICAL3	11.120743	48.271604	19.512027	45.440426	66.187408	88.852789	150.239108	74.125803
MICALL1	34.651264	43.829078	29.948176	37.196245	31.461348	98.925003	33.698489	66.284088
MICALL2	11.01152	15.187133	5.819157	13.67581	39.202783	21.808119	0	14.58589
MICB	30.120945	31.505035	39.474004	30.847198	3.149883	14.986928	21.67084	14.073986
MICC	0	0	0	0	0	0	0	0
MICD	0	0	0	0	0	0	0	0
MICE	3.728327	0.259365	0	0.836749	0	0.94551	5.4418	3.98951
MICG	0	0	0	0	0	0	0	0
MICOS10	177.365051	101.20159	68.890434	53.956282	121.201564	82.267218	63.951557	97.369153
MICOS10-NE	0	0	0	0	0	0	1.849615	5.031211
MICOS10P2	0	0	0	0	0	0	0	0
MICOS10P3	0	0	0	0	0	0	0	0
MICOS10P4	0	0	0	0	0	0	0	0
MICOS13	0	30.358573	34.829175	24.603533	0	17.152216	0	20.642951
MICU1	95.39792	68.350978	33.672393	71.935832	28.339241	43.606447	41.416069	58.967227

MICU2	18.525535	16.808044	18.856482	10.156547	29.297513	11.539331	0	20.36399
MICU3	3.312252	0.414239	0	0.593989	2.766032	0	0	1.309337
MID1	9.668302	27.934026	8.534479	37.65657	18.813787	56.844664	21.756965	64.362402
MID1IP1	0	49.949317	14.653399	48.354291	0	100.942202	0	67.639216
MID2	4.573768	16.325207	10.902751	14.174944	0.850979	11.511909	29.923563	13.632528
MIDEAS	22.374197	46.410225	21.484833	41.783297	18.403969	78.580827	86.380116	80.924256
MIDN	0	0	0	0	0	2.543142	0	1.942995
MIEF1	17.751277	47.206594	19.736412	56.184167	25.416426	63.016077	23.937439	70.060366
MIEF2	6.981342	11.31499	3.747132	12.30315	11.418413	23.424198	0.338689	16.289445
MIEN1	65.83103	69.4751	25.857379	86.470502	11.590253	78.732948	52.769286	62.289163
MIER1	38.353011	32.387006	43.976286	35.275347	16.881686	19.552302	62.631416	23.199162
MIER2	32.638201	25.81449	54.804564	40.768247	101.155525	63.265085	0	34.079206
MIER3	13.074994	39.278947	12.73799	24.200602	5.31117	15.081527	0	26.030274
MIF	384.306233	495.650172	442.452097	508.098974	536.031429	759.283921	667.124224	679.231887
MIF4GD	2.402441	17.034829	5.331505	13.311302	0	10.388807	0	7.063838
MIGA1	2.774404	19.22787	11.988669	8.59058	3.223356	4.585371	0	10.168234
MIGA2	5.174865	4.185172	7.012686	6.348381	13.905055	10.570748	1.919064	7.854504
MIIP	5.293412	4.486204	4.632504	6.761052	4.290037	8.333216	0	6.946618
MILR1	0	1.746793	4.764377	1.825343	0	1.669203	0	0.630648
MINAR1	0	0	0	0.704456	0.611703	0.348089	0	0
MINDY1	0	7.967918	7.801873	7.282828	11.702807	7.201949	0	7.989979
MINDY2	22.466961	22.443471	12.446052	21.055284	9.280745	22.58243	14.918889	23.11608
MINDY3	1.484382	3.193339	8.684262	3.155009	8.770101	2.008953	0	5.094199
MINDY4	0	1.579977	0	1.716368	0	1.353134	0	1.741455
MINDY4B	0	0	0	0	0	0	0	0
MINK1	27.965492	43.641505	26.727025	43.670723	90.928283	106.968714	92.884054	76.782649
MINPP1	10.861253	8.088825	9.866604	6.049398	11.048315	2.533495	55.594232	3.997426
MIOS	49.41969	135.493016	59.096935	76.30047	8.85859	38.542302	81.873073	104.551238
MIOX	0	0.775971	0	0	0	0	0	0
MIOXP1	0	0	0	0	0	0	0	0
MIP	0	0	0	0	0	0	0	0
MIPEP	17.638411	12.798277	7.697233	10.857148	19.297468	5.055347	5.565802	8.354554
MIPEPP1	0	0	0	0	0	0	0	0

MIPEPP2	0	0	0	0	0	0	0	0
MIPEPP3	0	2.717137	0	2.113626	0	1.271388	0	1.529365
MIPOL1	30.914639	15.502678	8.907753	17.879758	10.762995	11.127775	36.055272	14.220753
MIR1915HG	0	0.51509	0.782209	0.424792	0	0.250956	0	0.269873
MIR3171HG	0	0	0	0	0	0	0	0
MIR9-1HG	0	1.314286	4.317018	0.994682	0	0.637614	0	0.072299
MIS12	11.165542	30.304865	11.551241	35.403261	48.384906	11.738505	0	20.382501
MIS18A	18.50812	20.893476	26.765375	17.359401	26.198696	22.974064	29.03185	42.575474
MIS18BP1	25.827333	55.645065	18.683471	43.135758	106.700823	37.522014	33.755264	64.115837
MISFA	0	3.878683	0	3.39832	0	3.685272	0	4.698505
MISP	43.170829	52.13228	34.488678	52.119172	66.000711	105.224048	86.704691	75.319585
MISP3	0	1.961163	0	1.687333	0	1.451929	0	0.940381
MITD1	29.207669	29.678544	25.722894	21.294236	54.358181	15.514868	2.429333	18.633282
MITF	0	32.577712	0	21.732364	19.672159	21.106481	0	9.487795
MIX23	76.376716	123.514261	41.000162	74.752684	47.640916	49.632485	135.257768	137.486633
MIX23P1	0	0	0	0	0	0	0	0
MIX23P2	0	0	0	0	0	0	0	0
MIX23P3	0	0	0	0	0	0	0	0
MIX23P5	0	0	0	0	0	0	0	0
MIXL1	0	0	0	0	0	0	0	0
MKI67	83.750622	167.971168	81.182552	102.170485	150.031522	155.810212	216.103481	207.302733
MKI67P1	0	0	0	0	0	0	0	0
MKKS	24.828445	72.886202	18.238794	49.052764	28.759018	48.604369	22.470966	74.882772
MKLN1	27.467991	20.045699	24.719693	12.550297	46.220647	8.091664	59.786923	15.154276
MKNK1	13.043936	54.669889	37.107078	38.435422	50.229068	89.406893	35.91903	98.240722
MKNK2	23.770668	11.819445	28.032031	18.673176	40.560353	25.432266	88.403939	28.212078
MKNK2P1	0	0	0	0	0	0	0	0
MKRN1	52.393705	38.414268	64.670264	60.101321	22.685865	53.169842	45.362272	33.935526
MKRN2	20.677213	22.14641	19.60503	22.491902	12.817721	12.876802	11.518071	16.459532
MKRN2OS	0	0	0	0.292903	2.30506	0	0	0
MKRN3	0	0	0	0	0	0	0	0
MKRN4P	0	0	0	0	0	0	0	0
MKRN5P	0	0	0	0	0	0	0	0

MKRN6P	0	0	0	0	0	0	0	0
MKRN7P	0	0	0	0.192376	0	0	0	0
MKRN8P	0	0	0	0	0	0	0	0
MKRN9P	0	0	0	0	0	0	0	0
MKS1	6.167893	11.100003	0	13.130737	11.272953	10.14872	49.772223	7.704696
MKX	0	0.314884	0	0.067356	0	0	0	0
MLANA	0	0	0	0	0	0	0	0
MLC1	0.922321	0	0	0.472592	0	0	0	0.609395
MLEC	70.996906	117.519597	64.891853	99.990138	90.019833	91.379871	116.039122	72.807992
MLECP1	0	0	0	0	0	0	0	0
MLF1	80.384984	39.933183	80.799072	44.825271	14.42166	6.650956	13.359755	12.850219
MLF2	230.028353	172.613767	118.54761	173.311502	133.975756	174.166393	107.34085	101.081246
MLH1	23.869625	17.149752	0	9.219282	6.309561	1.285273	0	12.97236
MLH3	38.870269	16.585772	11.393845	15.809745	57.802856	8.184592	19.870248	7.524215
MLIP	0	0	0	0	0	0	0	0
MLKL	26.318207	58.117378	14.755084	43.99428	47.419976	94.865374	42.287407	86.830071
MLLT1	23.920059	40.726391	24.734099	44.231359	24.314586	69.584072	10.942453	48.273366
MLLT10	10.263886	32.069445	5.27371	23.463355	2.854765	17.333865	59.963518	25.875805
MLLT10P1	0	0	0	0	0	0	0	0
MLLT3	1.445756	21.671252	9.205439	12.785838	4.009114	11.644179	64.401994	16.267558
MLLT6	26.428147	32.939873	30.148123	32.737119	43.251121	59.159633	36.271022	27.189364
MLN	0	0	0	0	0	0	0	0
MLNR	0	0	0	0.072009	0	0	0	0
MLPH	0	3.152643	6.83584	3.466053	12.160099	2.199694	0	0.495885
MLST8	30.991169	41.330372	35.400136	47.684354	24.827321	53.893163	40.636337	46.607018
MLX	29.228939	8.212574	0	16.062804	11.286054	12.271405	93.179785	2.666331
MLXIP	31.182269	18.827516	14.795727	24.973673	80.361743	55.43231	7.777032	28.747785
MLXIPL	8.037198	4.664441	12.37385	1.328937	0	0.325471	0	0.084786
MLXP1	0	0	0	0	0	0	0	0
MLYCD	2.256348	5.692033	9.250946	4.673589	3.493716	4.315511	49.868113	2.899046
MMAA	0	0.544422	4.813409	2.726951	0	1.925619	5.846485	1.754436
MMAB	51.464034	141.885818	42.176277	106.194654	48.643784	118.253709	37.691268	89.45111
MMACHC	4.825047	4.950021	1.7471	5.220391	15.560089	9.33117	0	13.82644

MMACHCP1	0	0	0	0	0	0	0	0
MMADHC	93.900645	67.446382	119.224531	31.60231	97.202617	34.678034	92.377666	108.006946
MMADHCP1	0	0	0	0	0	0	0	0
MMADHCP2	0	0	0	0	0	0	0	0.367301
MMD	31.712283	15.483859	17.737843	19.887393	16.036494	1.86988	0	10.098758
MMD2	0	0	0	0	0	0	0	0
MME	21.937542	20.511236	0	25.678873	19.417957	4.987335	0	5.867771
MMEL1	0	0.227288	8.952491	1.794368	0	0	0	0
MMP1	10.580513	10.892003	18.890485	7.921407	0.464875	0.78495	0	0.142321
MMP10	2.00072	1.478687	0	0.731348	0	0	0	0.660251
MMP11	1.51725	5.949193	3.777126	1.217483	0	1.655411	0	0.698445
MMP13	0	0.371384	1.115239	1.069909	0	0.189991	12.407588	2.236194
MMP14	40.446099	154.633561	88.780283	389.811183	218.56935	541.895674	84.139223	321.385122
MMP15	10.564976	21.307934	37.19997	42.872549	7.082446	17.144646	0	9.786454
MMP16	1.109116	3.983898	0.49925	4.831763	0.073298	0.460515	0	0.382314
MMP17	1.488588	5.838901	0	9.350892	0	8.054087	0	4.809413
MMP19	0	0	0	0.534968	0	0	0	0
MMP2	69.377996	145.796204	79.726192	113.753804	94.604298	125.521923	96.184931	84.868895
MMP20	0	0	0	0	0	0	0	0
MMP21	0	0	0	0	0	0	0	0
MMP23A	0	0	0	0	0	0	0	0.925349
MMP23B	0	2.378136	6.16929	5.795681	8.045465	3.381433	0	0
MMP24	0.751822	0.032231	0	0.036725	0	0.225625	0	0.090544
MMP24OS	28.309564	23.423203	30.738224	26.307202	28.334734	27.993944	69.614971	25.356446
MMP25	1.806061	4.134074	9.090568	7.007501	0.476874	1.488009	0.219655	0.906991
MMP28	14.004697	8.052165	18.284298	25.06387	4.379396	7.383564	0	3.487938
MMP3	0	0	0	0	0	0	0	0.155393
MMP7	195.437101	115.197677	841.812973	589.475096	0.935357	0.327506	0.80839	0.579997
MMP8	0	0	0	0.062925	0	0	0	0
MMP9	0	0.435358	0	0.213712	0	0.290666	0	0.236009
MMRN1	0	0	0	0	0	0	0	0
MMRN2	0	15.027689	6.373305	14.547984	2.533046	22.69078	39.181867	13.912221
MMS19	22.201211	31.622213	17.052121	31.551848	23.343068	35.848386	42.659579	30.589549

MMS22L	33.064573	37.406349	25.651271	24.818377	36.33147	24.610717	121.091671	68.041497
MMUT	20.854199	18.559655	18.711214	15.12137	13.537338	11.462472	22.196952	24.85116
MN1	1.23923	1.832957	1.486366	0.960333	0.109168	2.177646	0	2.019571
MNAT1	78.676297	167.37495	105.673847	105.364997	146.057644	75.549634	67.438703	179.545337
MND1	0	13.693137	0	7.267718	0	3.727714	0	8.015147
MND1P1	0	0	0	0	0	0	0	0
MNDA	0	0	0	0	0	0	0	0
MNS1	15.363635	11.63578	6.097562	6.867907	0.900134	3.632738	0	7.988543
MNT	3.323849	18.848675	11.229982	17.962946	17.52698	30.206117	30.296048	20.056304
MNX1	0	4.308815	1.95412	4.3219	2.076563	21.322932	0	2.049339
MOAP1	22.68192	35.002183	17.486618	40.591098	16.080008	31.522249	9.283334	32.738063
MOB1A	217.297774	233.810259	163.921432	123.532471	63.959365	128.325013	121.550057	243.347316
MOB1AP1	0	0	0	0	0	0	0	0
MOB1AP2	0	0	0	0	0	0	0	0
MOB1B	30.786273	21.893485	0	15.679319	28.88894	9.809735	30.826022	13.487055
MOB2	8.074635	37.879213	31.287093	28.493372	1.564777	62.099206	24.885643	26.536121
MOB3A	13.775093	27.66688	14.998233	45.937636	38.258468	46.185312	0	47.248103
MOB3B	12.000913	20.970577	7.193543	15.655295	16.25189	34.496222	5.607726	41.26371
MOB3C	0	0.248152	0	0.747703	0	0.46466	0	0.879059
MOB4	20.048345	7.934996	42.752606	4.099097	13.197482	4.399812	19.768723	13.009112
MOB4P1	0	0	0	0	0	0	0	0
MOB4P2	0	0	0	0	0	0	0	0
MOBP	0	0	0	0	0	0.400427	0	0
MOCOS	12.547669	2.914666	2.361943	2.929959	0.971887	3.082826	0	3.097268
MOCS1	0	0.29369	0	0	3.117271	1.634497	0.965593	1.349667
MOCS1P1	0	0	0	0	0	0	0	0
MOCS2	0.966067	0.520497	1.855277	0.477296	39.722868	0.375233	82.19754	1.215745
MOG	0	0	0	0	0	0	0	0
MOGAT2	0	0	0	0	0	0	0	0
MOGAT3	0	0	0	0	0	0	0	0
MOGS	0	4.877709	19.904694	16.702633	18.299479	14.721852	36.050041	3.941382
MOK	34.805654	22.862668	7.403593	13.306787	12.663641	8.847406	0	11.692216
MON1A	0	0.617509	1.31837	0.478714	0	4.300039	0	1.3759

MON1B	28.301176	52.602601	14.552819	89.700851	19.722342	152.258331	0	92.229953
MON2	13.88229	15.20448	12.644244	13.282819	20.465599	27.685317	30.778751	28.303179
MORC1	0	0	0	0	0	0	0	0
MORC2	46.384965	77.348502	9.315915	73.998168	48.595192	80.346362	17.370276	92.648071
MORC3	15.602112	28.343188	18.207674	16.442239	23.617845	17.150342	19.433942	40.969
MORC4	42.571326	40.810863	29.732117	33.931841	7.829191	18.882719	13.61073	22.980908
MORF4	0	0.659593	0	0	0	0.193273	0	0
MORF4L1	199.492055	760.203665	332.256944	852.70568	347.873639	791.969889	585.148435	816.802463
MORF4L1P1	0	0.226718	0	0.261231	0	0.252228	0	0.206946
MORF4L1P3	0	0	0	0	0	0	0	0
MORF4L1P4	0	0	0	0	0	0	0	0
MORF4L1P5	0	0	0	0	0	0	0	0
MORF4L1P6	0	0	0	0	0	0	0	0
MORF4L1P7	0	0	0	0	0	0	0	0
MORF4L2	379.148871	817.982678	350.269579	1006.33922	316.053711	795.636215	508.745388	844.284638
MORF4L2P1	0	0	0	0	0	0	0	0
MORN1	24.736029	10.321461	5.420185	11.974586	33.426805	10.951513	0	12.431101
MORN2	14.248667	3.838237	23.553528	4.715247	2.270724	5.17691	0	6.981206
MORN3	0.870846	2.648918	0.781349	2.462421	0	0.217498	0	1.11325
MORN4	14.66426	31.767166	24.914377	34.888095	7.329565	14.635674	0	17.288492
MORN5	0	0	0	0.64588	0	0	0	0
MOS	0	0	0	0	0	0	0	0
MOSMO	14.59396	36.487318	42.802088	21.795908	3.691019	15.355216	49.000791	20.766897
MOSPD1	14.562632	57.102646	15.627244	44.814223	44.878908	13.978964	39.556919	41.194016
MOSPD2	12.201318	11.622986	2.122326	8.386514	30.561625	14.391064	28.570658	15.812682
MOSPD3	6.768153	6.727839	18.554579	7.681107	5.714733	9.641359	30.403461	10.585313
MOV10	16.747811	8.805807	10.650637	15.306409	38.330831	38.382384	0	25.466148
MOV10L1	0	0.337932	0	0.20398	0	0	0	0.502864
MOXD1	1.139364	0.892088	0	0.355964	0	0	0	0
MOXD2P	0	0	0	0	0	0	0	0
MPC1	13.125719	13.137113	11.546208	10.326362	35.883652	10.159921	15.656809	18.098175
MPC2	12.649486	23.739392	21.194747	16.326243	10.841337	31.255082	0	30.794797
MPDU1	44.682791	15.699923	17.508635	17.614753	47.960662	21.74202	32.343556	25.986584

MPDZ	8.996407	14.702074	0	4.368827	30.583135	8.745782	0	11.450267
MPG	0	29.765278	0	22.774165	33.665697	42.200569	0.882447	23.370194
MPHOSPH10	35.766673	41.762236	27.161214	24.171706	23.083698	19.045107	12.726071	27.563418
MPHOSPH10	0	1.014329	0	0.795481	0	0.594401	0	0.123782
MPHOSPH10	0	0	0	0	0	0	0	0
MPHOSPH10	0	1.538646	0	0.148584	0	0	0	0
MPHOSPH10	0	0	0	0	0	0	0	0
MPHOSPH10	0	0	0	0	0	0	0	0
MPHOSPH10	0	0	0	0	0	0	0	0
MPHOSPH10	0	0	0	0	0	0	0	0
MPHOSPH10	0	0	0	0	0	0	0	0
MPHOSPH6	54.971501	202.956315	81.815277	136.17526	96.614263	214.960412	113.114393	196.916207
MPHOSPH6P	0	0	0	0	0	0	0	0
MPHOSPH8	30.283457	10.297626	53.094608	17.375723	26.179141	14.978686	29.294455	15.100519
MPHOSPH9	31.700663	91.01239	22.363798	48.458707	32.923327	47.926632	106.508	63.421527
MPI	49.658687	33.263032	78.894214	58.755429	29.874066	36.99416	39.743604	38.916657
MPIG6B	0	0	0	0.037448	0	0	0	0
MPL	0	0.702225	0	1.720012	0	0.274289	0	0
MPND	4.881419	0.673182	0	5.340815	1.315662	10.385085	9.03576	8.912666
MPO	0	0	0	0	0	0	0	0
MPP1	26.001204	23.158801	29.512988	11.776425	51.061768	13.92244	49.073197	10.397485
MPP2	3.917983	8.229976	7.737565	9.859464	4.917484	6.505943	9.585062	3.397898
MPP3	9.725822	3.745426	21.959522	3.955541	7.122663	6.329902	0	4.087964
MPP4	0	0	0	0	0	0	0	0
MPP7	2.676011	10.362828	3.605631	7.146236	3.180974	1.891088	7.658386	2.606246
MPPE1	13.063795	21.684666	13.513581	30.055611	0	21.221225	81.628284	43.048071
MPPE1P1	0	0	0	0	0	0	0	0
MPPE1P2	0	0	0	0	0	0.885327	0	0
MPPED1	0	0	0	0	0	0	0	0
MPPED2	0	0	0	0.230872	0	1.028199	0	0.690747
MPRIP	169.422783	169.800367	98.106667	169.38907	268.578138	367.35796	182.877002	276.440349
MPRIPP1	0	0	0	0	0	0.176437	0	0.146193
MPST	0	11.100458	0	5.501357	0	11.239437	0	1.823317

MPV17	73.534637	64.497979	84.949315	66.204342	35.688372	22.573948	0	20.576096
MPV17L	13.421076	11.73165	12.058888	9.406366	14.170007	2.962993	0	1.390471
MPV17L2	0	0	0	0	0	0	0	0
MPV17L2P1	0	0	0	0	0	0	0	0
MPZ	0	1.404876	3.989187	0	0	0.217814	0	0
MPZL1	84.523682	147.59878	94.887901	124.024664	134.427787	95.81688	57.431126	120.366984
MPZL2	189.85542	238.398366	319.911607	521.376465	76.412622	94.15488	58.35406	82.386686
MPZL3	64.707904	29.807949	74.239833	48.409323	14.239892	5.024943	38.601036	9.244711
MR1	0	26.31977	5.675325	43.325404	5.589038	22.055936	20.005148	21.582454
MRAP	0	0	0	0.280268	0	0	0	0
MRAP2	0	0.889154	0	0.823485	0	0.159874	0	0.195189
MRAS	0	7.755789	0.781778	1.618799	0.23008	2.678754	0	2.04858
MRC1	0	0	0	0	0	0	0	0
MRC2	13.038424	5.750436	10.747228	11.722105	6.601417	5.808181	0	5.725672
MRE11	111.558936	82.481076	67.561015	67.821591	33.420096	26.83971	89.690588	50.112527
MRE11P1	0	0	0	0	0	0	0	0
MREG	0	23.991905	0	12.982994	33.440424	12.7155	41.013722	18.006236
MREGP1	0	0	0	0	0	0	0	0
MRFAP1	4.078309	0.532637	0	19.436193	0	115.91471	0	1.815903
MRFAP1L1	55.181285	80.531154	36.220109	72.034286	29.9759	45.435807	67.115485	67.283834
MRFAP1L2	13.754658	45.758144	36.136894	44.635784	9.929458	15.98211	0	17.274067
MRFAP1P1	0	0	0	0	0	0	0	0
MRGBP	40.497556	88.780644	30.76791	84.770332	22.020885	87.044763	13.11031	86.230711
MRGPRD	0	0	0	0	0	0	0	0
MRGPRF	1.617705	0	0	0.143223	5.972065	1.442497	0	0.978472
MRGPRG	0	0	0	0	0	0	0	0
MRGPRX1	0	0	0	0	0	0	0	0
MRGPRX10P	0	0	0	0	0	0	0	0
MRGPRX11P	0	0	0	0	0	0	0	0
MRGPRX12P	0	0	0	0	0	0	0	0
MRGPRX13P	0	0	0	0	0	0	0	0
MRGPRX3	0	0.091143	0	0	0	0	0	0
MRGPRX4	0	0	0	0	0	0	0	0

MRGPRX5P	0	0	0	0	0	0	0	0
MRGPRX6P	0	0	0	0	0	0	0	0
MRGPRX7P	0	0	0	0	0	0	0	0
MRGPRX8P	0	0	0	0	0	0	0	0
MRGPRX9P	0	0	0	0	0	0	0	0
MRI1	8.86808	4.045272	14.151889	5.52477	32.231246	9.497557	8.618371	7.796478
MRLN	0	0	0	0	0	0	0	0
MRM1	2.86435	5.913901	7.623575	6.558075	4.014502	5.023881	0	5.180339
MRM2	104.912933	196.026194	114.270931	117.124853	139.892981	116.239507	10.832019	93.417368
MRM3	12.237352	12.73442	10.907275	19.20945	60.183755	21.606627	23.505171	11.621413
MRM3P1	0	0	0	0	0	0	0	0
MRM3P2	0	0	0	0	0	0	0	0
MRNIP	25.233565	18.902446	20.225143	33.982025	4.044944	17.329036	45.211232	15.624454
MRO	0	0	0	0	0	0.031684	0	0.02538
MROH1	11.044663	8.825754	8.779385	9.542693	1.999986	10.962951	0.135999	8.975215
MROH2A	0	0	0	0	0	0	0	0
MROH2B	0	0.22725	0	0.177767	0	0	0	0
MROH3P	0	0	0	0	0	0	0	0
MROH4P	0	0	0	0	0	0	0	0
MROH5	0	0	0	0	0	0	0	0
MROH6	26.375116	9.832084	8.218512	9.500357	4.219673	16.473463	9.494547	16.887464
MROH7	0	0	0	0	0	0	0	0
MROH7-TTC	0	0.101869	0	0.094691	0	0	0	0
MROH8	0	1.541457	7.766077	0.494211	0	0.491904	0	0
MROH9	0	0	0	0	0	0	0	0
MRPL1	19.075627	21.658964	16.893603	7.35098	12.539031	4.848899	71.008769	29.029263
MRPL10	46.310516	45.684602	76.780691	50.977515	16.097395	25.522973	0	29.239106
MRPL11	283.121626	401.794502	236.776477	349.390147	117.985058	203.606713	103.335315	214.382834
MRPL12	126.015979	229.718064	114.572196	199.56864	90.258408	303.579581	86.814862	291.05117
MRPL13	0	7.206324	25.674779	1.446735	0	1.043562	185.795046	2.823386
MRPL14	40.75415	147.782714	32.32065	143.338202	85.580524	250.79445	105.1785	232.943625
MRPL15	0	0	0	17.880533	24.280851	10.462724	0	0
MRPL15P1	0	0	0	0.108473	0	0	0	0

MRPL16	91.116447	88.074348	94.888825	88.411319	149.068746	92.404718	72.072965	115.683964
MRPL17	0	74.238964	71.939744	103.378933	208.433291	85.766948	243.941764	75.60576
MRPL18	104.346437	144.438733	105.994411	129.607666	93.734489	132.163168	104.081957	156.387589
MRPL19	71.986923	41.508828	99.45476	30.863573	27.197077	21.534652	24.898772	44.202333
MRPL2	32.671812	70.646716	45.996908	97.252494	77.474421	89.813853	0	68.00222
MRPL20	156.314337	264.828485	93.19567	228.482627	165.966561	331.957387	146.486811	379.859695
MRPL20P1	0	0	0	0	0	0	0	0
MRPL21	250.918506	225.699654	196.560571	207.881056	138.129874	178.78097	45.023172	172.204233
MRPL22	110.681572	80.977089	86.39108	69.839053	160.307545	44.86546	18.695	55.667683
MRPL22P1	0	0	0	0	0	0	0	0
MRPL23	84.862892	87.240508	63.337385	75.241393	120.073953	102.516959	0	100.595175
MRPL24	127.979461	252.148581	108.641122	247.742107	98.100642	176.11506	83.799032	148.647182
MRPL27	43.907857	48.19989	55.310981	52.292092	65.579752	89.452902	88.860639	77.65783
MRPL28	69.871062	0	55.422551	27.299655	2.123788	34.959001	23.586261	18.820046
MRPL2P1	0	0	0	0	0	0	0	0
MRPL3	268.154306	232.367194	207.521016	138.087883	133.659335	108.94724	96.652453	202.728449
MRPL30	54.118271	37.960279	56.243194	39.528886	51.900072	51.004149	122.35027	71.496795
MRPL30P1	0	0.846386	0	0	0	0	0	0
MRPL32	55.998895	31.958913	32.786425	33.639433	110.121248	47.069698	61.450205	56.500084
MRPL32P1	0	0	0	0	0	0	0	0
MRPL32P2	0	0	0	0	0	0	0	0
MRPL33	123.285532	193.091861	74.539692	148.75645	71.814378	145.965745	11.236066	208.851164
MRPL34	52.101266	42.011813	0	16.508935	32.376583	26.157673	75.750645	15.401967
MRPL35	52.724444	57.448661	63.874714	46.16194	37.324359	19.566553	161.355724	29.907923
MRPL35P1	0	0	0	0	0	0	0	0
MRPL35P2	0	0	0	0	0	0	0	0
MRPL35P3	0	0	13.99279	0	0	0	0	0
MRPL35P4	0	0	0	0	0	0	0	0
MRPL36	92.716803	69.484027	83.267759	62.016054	111.872486	70.714747	109.69953	70.785669
MRPL36P1	0	0	0	0	0	0	0	0
MRPL37	229.993482	250.316015	230.050437	250.079949	166.056673	119.957534	138.646557	148.580386
MRPL37P1	0	0	0	0	0	0	0	0
MRPL38	0	4.408554	0	10.466858	0	0.75545	125.453719	0

MRPL39	0	0	0	0	0	0	0	0
MRPL3P1	0	0	0	0	0	0	0	0
MRPL4	38.830547	61.600384	20.933344	58.895705	18.730238	68.873291	21.308893	47.179728
MRPL40	32.730279	42.994508	18.024242	40.227749	48.551276	66.270814	23.824617	78.037831
MRPL40P1	0	0	0	0	0	0.348654	0	0
MRPL41	55.312633	91.383002	81.833626	94.726702	90.598299	142.13106	37.004655	115.902109
MRPL42	93.091167	101.008026	97.873397	61.330408	113.542304	56.323445	159.013739	125.523813
MRPL42P1	0	0.482902	0	0	0	0	0	0
MRPL42P2	0	0	0	0	0	0	0	0
MRPL42P3	0	0	0	0	0	0	0	0
MRPL42P4	0	0	0	0	0	0	0	0
MRPL42P6	0	0	0	0	0	0	0	0
MRPL43	54.261583	133.485329	66.468073	150.737563	80.566868	107.677914	64.924263	95.751671
MRPL44	29.312522	17.375084	29.849778	17.658494	49.094218	17.220748	28.329982	28.014212
MRPL45	67.130911	44.66274	75.560219	43.740061	40.71866	37.965565	41.051987	44.311113
MRPL45P1	0	0	0	0	0	0	0	0
MRPL45P2	0	5.086943	9.881693	2.386201	57.069911	2.602404	0	0
MRPL46	65.923701	52.604867	31.106722	58.793442	72.99391	55.732174	18.7891	51.863258
MRPL47	0	61.70612	39.957707	64.386224	60.345726	43.146185	78.589066	47.780279
MRPL48	31.102523	102.970402	33.977212	119.808081	51.692817	42.5818	0	75.648323
MRPL48P1	0	0	0	0	0	0	0	0
MRPL49	0	8.178314	0	1.024048	0	21.48129	58.893839	0.665069
MRPL49P1	0	0	0	0	0	0	0	0
MRPL49P2	0	0	0	0	0	0	0	0
MRPL50	30.995456	11.050748	14.343058	8.418666	14.781485	7.031954	8.99372	11.172688
MRPL50P1	0	0	0	0	0	0	0	0
MRPL50P2	0	0	0	0	0	0	0	0
MRPL50P4	0	0	0	0	0	0	0	0
MRPL51	374.108802	572.913654	362.790019	479.708154	454.187374	288.236623	317.052038	423.680058
MRPL51P2	0	0	0	0	0	0	0	0
MRPL52	85.156752	65.516884	100.147374	81.013249	103.921589	116.01514	0	104.618815
MRPL53	50.237356	60.026644	77.206179	57.235369	68.994562	51.397809	95.445769	75.22625
MRPL53P1	0	0	0	0	0	0	0	0

MRPL54	22.2606	28.083162	44.937738	27.40632	9.676881	40.092366	45.285467	28.065326
MRPL55	30.981148	93.958099	29.288569	63.229761	10.098825	71.659286	62.411725	48.304629
MRPL57	19.993737	22.519795	16.496514	14.676833	14.196458	16.242631	25.348148	17.915981
MRPL57P1	0	0	0	0	0	0	0	0
MRPL57P3	0	0	0	0	0	0	0	0
MRPL57P6	0	0	0	0	0	0	0	0
MRPL57P7	0	0	0	0	0	0	0	0
MRPL57P8	0	0	0	0	0	0	0	0
MRPL58	62.115347	55.964539	55.716969	58.158146	17.456895	59.847713	40.611498	53.832331
MRPL9	81.194413	89.118498	69.816482	73.049483	87.634347	80.967095	99.714423	82.908955
MRPL9P1	0	0	0	0	0	0	0	0
MRPS10	57.56509	40.34975	64.646017	22.400064	69.388376	19.548604	39.165195	39.665301
MRPS10P1	0	0	0	0	0	0	542.664517	0
MRPS10P2	0	0	0	0	0	0	0	0
MRPS11	34.703386	50.570174	31.098723	57.838505	95.372403	56.664729	86.409449	47.950474
MRPS11P1	0	0	0	0	0	0	0	0
MRPS12	8.456999	31.885205	13.081962	24.647996	50.86861	55.901213	24.230393	43.273958
MRPS14	14.68749	22.780414	34.807422	20.563747	20.655332	14.392503	5.139324	19.049171
MRPS15	86.028649	232.450093	64.969676	181.015107	203.227147	317.735668	103.755166	370.434438
MRPS15P1	0	0	0	0	0	0	0	0
MRPS16	151.21063	194.186152	69.479759	192.394247	104.747164	173.90511	113.069214	226.250153
MRPS16P1	0	0	0	0	0	0	0	0
MRPS16P2	0	0	0	0	0	0	0	0
MRPS16P3	0	0	0	0	0	0	0	0
MRPS17	21.637092	34.883301	40.185439	24.361076	48.079687	26.074031	33.768049	52.687241
MRPS17P1	0	0	0	0	0	0	0	0
MRPS17P3	0	0	0	0	0	0	0	0
MRPS17P5	0	0	0	0	0	0	0	0
MRPS17P9	0	0	0	0	0	0	0	0
MRPS18A	0	6.508307	0	1.984874	0	6.296655	32.603915	1.576302
MRPS18AP1	0	0	0	0	0	0	0	0
MRPS18B	59.695676	37.801943	66.877656	51.920922	117.504996	40.231749	43.349787	38.44202
MRPS18BP1	0	0	0	0	0	0	0	0

MRPS18BP2	0	0	0	0	0	0	0	0
MRPS18C	155.523154	43.202512	26.504826	26.457099	83.085246	20.739634	0	24.97162
MRPS18CP4	0	0	0	0	0	0	0	0
MRPS18CP6	0	0	0	0	0	0	0	0
MRPS18CP7	0	0	0	0	0	0	0	0
MRPS2	71.724759	80.124192	58.908348	82.610065	95.742462	98.236573	23.037443	88.039801
MRPS21	18.675607	5.554734	11.974941	8.618981	27.248024	9.805443	0	11.142205
MRPS21P1	0	0	0	0	0	0	0	0
MRPS21P2	0	0	0	0	0	0	0	0
MRPS21P3	0	0	0	0	0	0	0	0
MRPS21P5	0	0	0	0	0	0	0	0
MRPS21P6	0	0	0	0	0	0	0	0
MRPS21P7	0	0	0	0	0	0	0	0
MRPS21P8	0	0	0	0	0	0	0	0
MRPS21P9	0	0	0	0	0	0	0	0
MRPS22	5.20639	0.53601	3.544091	1.636396	2.283821	0	102.333827	0.928515
MRPS22P1	0	0	0	0	0	0	0	0
MRPS23	71.665911	65.231386	40.029293	57.801525	105.590183	39.782493	13.013728	54.970795
MRPS24	0	117.484799	90.661364	110.968516	345.913446	252.837626	339.692151	315.184749
MRPS25	8.51632	59.076178	37.430236	44.645836	19.189334	44.359235	0	26.664459
MRPS26	30.292427	108.632764	63.49862	117.134589	52.708314	127.86431	36.252689	73.809799
MRPS27	0	0.438414	0	24.888221	29.683089	18.220076	0	15.17468
MRPS28	63.227917	61.092601	62.804878	85.725652	40.645026	60.781751	76.968209	46.364807
MRPS30	38.997828	30.554442	68.6421	18.677983	29.785802	15.127642	37.458393	22.632008
MRPS31	0	4.780966	0	0	0	0	0	0
MRPS31P1	0	0	0	0	0	0	0	0
MRPS31P2	0	0	0	0	0	0	0	0
MRPS31P4	0	19.024609	0	11.380113	1.114026	6.279177	23.31242	17.235596
MRPS31P5	0	0	0	0	0	0.79001	0	3.628489
MRPS33	84.443941	104.076793	77.654464	65.281704	141.737856	46.745924	181.502167	70.107393
MRPS33P2	0	0	0	0	0	0	0	0
MRPS33P3	0	0	0	0	0	0	0	0
MRPS33P4	0	0	0	0	0	0	0	0

MRPS34	95.275157	115.947182	44.728735	139.726388	57.252217	146.391382	0	126.518973
MRPS35	70.524639	72.315875	61.50933	49.189418	44.426948	34.837605	40.289607	66.718849
MRPS35P2	0	0	0	0	0	0	0	0
MRPS35P3	0	0	0	0	0	0	0	0
MRPS36P1	0	0	0	0	0	0	0	0
MRPS36P2	0	0	0	0	0	0	0	0
MRPS36P3	0	0	0	0	0	0	0	0
MRPS36P4	0	0	0	0	0	0	0	0
MRPS36P5	0	0	0	0	0	0	0	0
MRPS5	12.350028	17.336933	4.179762	22.439841	9.409344	22.704894	40.951348	23.5724
MRPS5P3	0	0	0	0	0	0	0	0
MRPS5P4	0	0	0	0	0	0	0	0
MRPS6	93.23053	75.938015	55.696494	49.416444	67.831163	76.79716	135.291053	128.333814
MRPS6P2	0	0	0	0	0	0	0	0
MRPS6P4	0	0	0	0	0	0	0	0
MRPS7	77.267239	197.829634	82.995794	160.712322	87.154163	168.778971	85.998599	168.59097
MRPS9	38.425697	32.393468	47.807969	29.410752	9.440575	30.895729	38.892666	35.786105
MRRF	116.773911	92.325972	93.006947	74.713915	38.848211	64.572973	76.40431	88.602753
MRRFP1	0	0	0	0	0	0	0	0
MRS2	16.930833	24.529537	12.749592	11.256193	6.641924	10.225667	46.992343	22.628929
MRS2P1	0	0	0	0	0	0	0	0
MRS2P2	0	0	0	0	0	0	0	0
MRTFA	0	3.083985	11.254871	9.335326	29.046366	6.556658	0	9.253946
MRTFB	2.560958	26.419328	19.00678	44.840457	20.172177	48.117048	166.002666	61.619829
MRTO4	50.690499	69.834489	37.725308	72.432942	43.119852	103.625657	73.244521	108.283402
MS4A1	0	0	0	0	0	0	0	0
MS4A10	0	0	0	0	0	0	0	0
MS4A12	0	0	0	0	0	0	0	0
MS4A13	0	0	0	0	0	0	0	0
MS4A14	0	0	0	0	0	0	0	0
MS4A15	0	0	0	0	0	0	0	0
MS4A19P	0	0	0	0	0	0	0	0
MS4A2	0	0	0	0	0	0	0	0

MS4A3	0	0	0	0	0	0	0	0
MS4A4A	0	0	0	0	0	0	0	0
MS4A4E	0	2.481056	1.236007	2.070892	0	0.792741	0	0.800171
MS4A5	0	0	0	0	0	0	0	0
MS4A6A	0	0	0	0	0	0	0	0
MS4A6E	0	0	0	0	0	0	0	0
MS4A7	0	0	0	0	0	0	0	0
MS4A8	0	0	0	0	0	0	0	0
MSANTD1	0	0	0	0.165058	0	0	0	0
MSANTD2	2.171953	2.158441	0	0.89178	14.229661	1.763718	22.241821	3.364358
MSANTD2P1	0	0	0	0	0	0	0	0
MSANTD3	27.837087	58.36882	68.012187	54.772144	36.731739	55.709561	40.732884	53.396756
MSANTD3-TI	1.671759	2.620384	0	1.429949	0	0.579369	0	1.366057
MSANTD3P1	0	0	0	0	0	0	0	0
MSANTD4	33.146142	88.821929	56.095276	87.035475	32.515208	42.722463	4.283828	45.408279
MSANTD7	0	0	0	0.874575	25.419272	5.553839	0	2.36532
MSC	0	31.561713	0	52.190885	2.977214	0	50.370555	4.132557
MSH2	0	8.263933	1.36056	3.377685	0	1.489493	0	1.675812
MSH3	15.306267	20.797055	28.900098	12.209473	15.293084	9.84027	7.92122	14.971674
MSH4	0	0	0	0	0	0	0	0
MSH5	26.379752	18.618343	46.726675	5.665524	0	8.029153	0	3.394061
MSH5-SAPCI	0	0.221732	4.386088	0.238945	0	0	0	0.16042
MSH6	0	0	0	12.867612	0	26.9341	0	44.066097
MSI1	14.763705	16.675345	10.174255	20.199082	2.098141	8.60996	0	6.236752
MSI2	12.763019	84.01508	26.522448	73.387494	126.19122	52.943723	19.879937	46.498138
MSL1	29.76131	51.672243	62.763861	59.006628	7.47535	65.212133	104.850037	54.313209
MSL2	30.568055	87.537403	26.878466	55.704636	20.806572	35.086966	43.223924	105.812335
MSL3	25.447689	42.133078	4.674548	32.879661	6.215905	43.69212	13.670916	58.9578
MSL3P2	0	0	0	0	0	0	0	0
MSL3P3	0	0	0	0	0	0	0	0
MSLN	3.563917	4.146093	3.149452	4.247591	0	1.710587	6.344852	0
MSLNL	0	0	0	0	0	0	0	0
MSMB	0	0	0	0.4812	0	0	0	0

MSMO1	169.362644	89.276959	103.262909	26.165112	71.129479	11.279309	35.604551	34.242815
MSMP	0	0	0	0	0	1.299323	0	0.796542
MSN	193.636729	156.967936	164.784175	170.893834	250.382882	242.198697	273.419288	211.624223
MSNP1	0	0	0	0	0	0	0	0.08231
MSR1	0	0	0	0.057369	0	0	0	0
MSRA	6.424716	5.960604	4.221733	2.978639	0.650136	6.638551	0	3.553872
MSRB1	0	6.328316	0	1.926556	0	5.334927	0	0
MSRB1P1	0	0	0	0	0	0	0	0
MSRB2	4.076479	3.522794	7.266868	4.682721	4.296403	1.708568	0	3.047598
MSRB3	6.170593	17.851994	7.255471	8.920341	23.0841	9.636005	99.522299	10.642872
MSS51	0	0.073796	0	0.114994	0	0.116739	0	0.192394
MST1	0	0.980677	0	0.899831	0	2.298282	37.767461	4.020543
MST1L	0	0.324045	0	0	0	0	0	0.343919
MST1P2	0	0	0	0.21868	0	0	0	0
MST1R	3.140632	3.981256	16.745164	7.920491	1.453269	10.807934	0	8.904679
MSTO1	30.267086	17.692656	4.895392	25.010953	25.366568	6.43657	40.987152	7.245436
MSTO2P	0	1.2649	0	1.504004	0	0	0	0
MSX1	3.589085	6.450542	3.203419	4.095236	0.946104	4.081373	0.868651	4.273266
MSX2	7.835179	9.649469	4.201352	8.749125	10.859951	4.149208	22.614761	3.522188
MSX2P1	0	0	0	0.844651	0	0.254399	0	0.659454
MT-ATP6	8636.75265	1237.8524	5926.47798	759.007946	9351.30186	1005.43495	9178.70056	1546.24355
MT-ATP8	17355.4372	2042.11544	10538.5856	914.447551	15056.1085	717.199242	14691.6565	1954.36532
MT-CO1	10896.0321	1652.92099	7734.34763	1164.23282	10157.2771	1397.53149	8914.93624	1989.87966
MT-CO2	10409.5415	1509.5281	6164.4529	933.508776	10551.6597	1579.34718	9443.67884	1803.23727
MT-CO3	9202.1026	1158.53812	5639.5352	644.27523	9318.17393	1041.23249	10167.5873	1637.7436
MT-CYB	5359.662	849.629412	3513.48649	653.13689	5141.57393	893.007028	5095.40582	900.568752
MT-ND1	8796.63423	1750.32534	8323.69707	1857.944	8662.237	1634.65048	8172.36828	1695.40107
MT-ND2	7895.43298	1291.10531	5582.20562	936.119488	7473.49884	1181.98994	6554.33634	1454.19582
MT-ND3	5417.12475	585.801745	2973.40889	313.791769	3469.94259	354.842679	2539.9934	433.638806
MT-ND4	9542.29034	1482.83625	7370.52034	1132.67658	9275.73759	1453.26593	9622.20112	1760.52159
MT-ND4L	7994.48067	1041.92586	6429.26169	420.822651	6309.7622	474.360914	6030.04827	1022.31648
MT-ND5	6242.41043	1062.29339	4674.22586	783.559854	5163.77579	868.061315	4359.36788	941.922805
MT-ND6	1427.32863	310.138703	1309.9558	312.276312	1416.54299	210.578522	1653.55009	177.6693

MT1A	0	0	0	0	0	0	0	0
MT1B	0	1.077934	0	0	0	0	0	0
MT1CP	0	0	0	0	0	0	0	0
MT1DP	0	0	0	0	0	0	0	0
MT1E	18.167191	157.952974	132.736716	130.593346	120.846748	41.989885	114.902767	82.485035
MT1F	13.366992	38.825922	10.863437	66.844049	0	10.474737	0	22.133068
MT1G	0	0	0	0	5.860535	0	0	0.294069
MT1H	0	0	0	0	0	0	0	0
MT1IP	0	0	0	0	0	0	0	0
MT1JP	0	0	0	0	0	0	0	0
MT1M	0	6.345159	0	6.586977	0	6.340688	0	6.188799
MT1P1	0	0	0	1.552752	0	0	0	0
MT1P3	0	9.708025	0	0	0	5.930982	0	3.089307
MT1X	98.332173	106.579934	106.007344	154.510185	31.366343	112.2106	0	180.437129
MT1XP1	0	0	0	0	0	0	0	0
MT2A	1315.54099	1162.59286	1180.25689	1557.26449	1424.94721	2091.60186	1611.27633	2522.73647
MT2P1	0	0	0	0	0	0	0	0
MT3	0	0	0	0.664304	0	0.581295	0	1.118962
MTA1	52.030561	44.164871	88.182404	64.459433	19.384708	59.154723	58.918611	40.030276
MTA2	81.532496	164.369848	60.734594	154.30531	121.144613	346.968775	356.0354	372.837207
MTA3	24.108183	22.56706	65.587555	16.59947	54.121521	14.01258	159.273068	11.594541
MTA3P1	0	0	0	0	0	0	0	0
MTAP	65.815521	64.971664	58.497884	52.441249	161.921839	76.462717	213.256171	97.185029
MTAPP1	0	0	0	0	0	0	0	0
MTAPP2	0	0	0	0	0	0	0	0
MTARC1	35.478717	19.80145	6.709866	18.425133	1.079933	4.876733	4.548567	8.356493
MTARC2	6.424655	23.172417	32.723276	14.906737	19.645521	9.570076	1.301024	17.767393
MTARC2P1	0	0	0	0	0	0	0	0
MTATP6P1	1119.53556	157.219219	859.511528	118.243585	1092.48049	162.348067	1508.78267	166.289792
MTATP6P10	0	0	0	0	0	0	0	0
MTATP6P11	0	0	0	0	0	0	0	0.267882
MTATP6P13	0	0	0	0	0	0	0	0
MTATP6P14	0	0	0	0	0	0	0	0

MTATP6P15	0	0	0	0	0	0	0	0
MTATP6P16	0	0	0	0	0	0	0	0
MTATP6P17	0	0	0	0	0	0	0	0
MTATP6P18	0	0	0	0	0	0	0	0
MTATP6P19	0	0	0	0	0	0	0	0
MTATP6P2	0	0	0	0	0	0	0	0
MTATP6P20	0	0	0	0	0	0	0	0
MTATP6P21	0	0	0	0	0	0	0	0
MTATP6P22	0	0	0	0	0	0	0	0
MTATP6P23	0	0	0	0	0	0	0	0
MTATP6P24	0	0	0	0	0	0	0	0
MTATP6P25	0	0	0	0	0	0	0	0
MTATP6P26	0	0	0	0	0	0	0	0
MTATP6P27	0	0	0	0	0	0	0	0
MTATP6P29	0	0	0	0	0	0	0	0
MTATP6P3	0	0	0	0	0	0	0	0
MTATP6P30	0	0	0	0	0	0	0	0
MTATP6P31	0	0	0	0	0	0	0	0
MTATP6P4	0	0	0	0	0	0	0	0
MTATP6P5	0	0	0	0	0	0	0	0
MTATP6P7	0	0	0	0	0	0	0	0
MTATP6P8	0	0	0	0	0	0	0	0
MTATP6P9	0	0	0	0	0	0	0	0
MTATP8P1	0	0	0	0	0	0	0	0
MTATP8P2	0	0	0	0	0	0	0	0
MTBP	10.931323	9.337256	25.723554	5.086296	55.640644	6.397697	5.121469	4.659758
MTCH1	7.83393	59.764077	52.101952	53.954281	18.349581	56.252739	96.555833	61.770107
MTCH1P1	0	0	0	0	0	0.264995	0	0
MTCH1P2	0	0	0	0	0	0	0	0
MTCH2	102.606633	48.084306	48.536708	33.719706	136.1634	50.805826	60.60437	104.041543
MTCH2P1	0	0	0	0	0	0	0	0
MTCH2P2	0	0	0	0	0	0	0	0
MTCH2P3	0	0	0	0	0	0	0	0

MTCH2P4	0	0	0	0	0	0	0	0
MTCL1	69.702284	35.124642	15.679362	38.663538	4.727666	51.010999	8.726311	31.378058
MTCL1P1	0	0	0	0	0	0	0	0
MTCL2	5.390083	7.415987	8.898945	8.98637	17.040547	34.851946	13.270946	19.414671
MTCL3	0	0	0	0	0	0	0	0
MTCO1P1	0	0	0	0	0	0	0	0
MTCO1P10	0	0	0	0	0	0	0	0
MTCO1P11	0	0	0	0	0	0	0	0
MTCO1P12	46.843901	2.555059	20.875536	1.575981	12.815565	2.494495	25.966848	2.711923
MTCO1P14	0	0	0	0	0	0	0	0
MTCO1P15	0	0	0	0	0	0	0	0
MTCO1P17	0	0	0	0	0	0	0	0
MTCO1P18	0	0	0	0	0	0	0	0
MTCO1P19	0	0	0	0	0	0	0	0
MTCO1P2	0	0	0	0	0	0	0	0
MTCO1P20	0	0	0	0	0	0	0	0
MTCO1P21	0	0	0	0	0	0	0	0
MTCO1P22	0	0	0	0.064716	0	0	0	0
MTCO1P23	0	0	0	0	0	0	0	0
MTCO1P24	0	0	0	0	0	0	0	0
MTCO1P25	0	0	0	0	0	0	0	0
MTCO1P27	0	0	0	0	0	0	0	0
MTCO1P28	0	0	0	0	0	0	0	0
MTCO1P29	0	0	0	0	0	0	0	0
MTCO1P3	0	0	0	0	0	0	0	0
MTCO1P30	0	0	0	0	0	0	0	0
MTCO1P31	0	0	0	0	0	0	0	0
MTCO1P35	0	0	0	0	0	0	0	0
MTCO1P38	0	0	0	0	0	0	0	0
MTCO1P39	0	0	0	0	0	0	0	0
MTCO1P4	0	0	0	0	0	0	0	0
MTCO1P40	0	0.282257	0	0	0	0	0	0
MTCO1P42	0	0.233507	0	0.137862	0	0	1.375308	0

MTCO1P43	0	0	0	0	0	0	0	0
MTCO1P44	0	0	0	0	0	0	0	0
MTCO1P45	0	0	0	0	0	0	0	0
MTCO1P46	0	0	0	0	0	0	0	0
MTCO1P47	0	0	0	0	0	0	0	0
MTCO1P48	0	0	0	0	0	0	0	0
MTCO1P49	0	0	0	0	0	0	0	0
MTCO1P5	0	0	0	0	0	0	0	0
MTCO1P51	0	0	0	0	0	0	0	0
MTCO1P52	0	0	0	0	0	0	0	0
MTCO1P53	0	0	0	0	0	0	0	0.25235
MTCO1P54	0	0	0	0	0	0	0	0
MTCO1P55	0	0	0	0	0	0	0	0
MTCO1P56	0	0	0	0	0	0	0	0
MTCO1P57	0	0	0	0	0	0	0	0
MTCO1P58	0	0	0	0	0	0	0	0
MTCO1P59	0	0	0	0	0	0	0	0
MTCO1P6	0	0	0	0	0	0	0	0
MTCO1P7	0	0	0	0	0	0	0	0
MTCO1P8	0	0	0	0	0	0	0	0
MTCO1P9	0	0	0	0	0	0	0	0
MTCO2P1	0	0	0	0	0	0	0	0
MTCO2P10	0	0	0	0	0	0	0	0
MTCO2P11	0	0	0	0	0	0	0	0
MTCO2P12	0	0	0	0	0	0.29975	0	0
MTCO2P15	0	0	0	0	0	0	0	0
MTCO2P16	0	0	0	0	0	0	0	0
MTCO2P17	0	0	0	0	0	0	0	0
MTCO2P18	0	0	0	0	0	0	0	0
MTCO2P19	0	0	0	0	0	0	0	0
MTCO2P2	0	0	0	0	0	0	0	0
MTCO2P20	0	0	0	0	0	0	0	0
MTCO2P21	0	0	0	0	0	0	0	0

MTCO2P22	0	0	0	0	0	0	0	0
MTCO2P23	0	0	0	0	0	0	0	0
MTCO2P24	0	0	0	0	0	0	0	0
MTCO2P25	0	0	0	0	0	0	0	0
MTCO2P27	0	0	0	0	0	0	0	0
MTCO2P29	0	0	0	0	0	0	0	0
MTCO2P3	0	0	0	0	0	0	0	0
MTCO2P30	0	0	0	0	0	0	0	0
MTCO2P31	0	0	0	0	0	0	0	0
MTCO2P32	0	0	0	0	0	0	0	0
MTCO2P33	0	0	0	0	0	0	0	0
MTCO2P34	0	0	0	0	0	0	0	0
MTCO2P4	0	0	0	0	0	0	0	0
MTCO2P5	0	0	0	0	0	0	0	0
MTCO2P6	0	0	0	0	0	0	0	0
MTCO2P7	0	0	0	0	0	0	0	0
MTCO2P8	0	0	0	0	0	0	0	0
MTCO2P9	0	0	0	0	0	0	0	0
MTCO3P1	0	0	0	0	0	0	0	0
MTCO3P10	0	0	0	0	0	0	0	0
MTCO3P11	0	0	0	0	0	0	0	0
MTCO3P12	0	0.352682	0	0	0	0	0	0.369634
MTCO3P13	0	0	0	0	0	0	0	0
MTCO3P15	0	0	0	0	0	0	0	0
MTCO3P16	0	0	0	0	0	0	0	0
MTCO3P17	0	0	0	0	0	0	0	0
MTCO3P18	0	0	0	0	0	0	0	0
MTCO3P19	0	0	0	0	0	0	0	0
MTCO3P2	0	0	0	0	0	0	0	0
MTCO3P20	0	0	0	0	0	0	0	0
MTCO3P21	0	0	0	0	0	0	0	0
MTCO3P22	0	0	0	0	0	0	0	0
MTCO3P23	0	0	0	0	0	0	0	0

MTCO3P24	0	0	0	0	0	0	0	0
MTCO3P27	0	0	0	0	0	0	0	0
MTCO3P28	0	0	0	0	0	0	0	0
MTCO3P29	0	0	0	0	0	0	0	0
MTCO3P30	0	0	0	0	0	0	0	0
MTCO3P31	0	0	0	0	0	0	0	0
MTCO3P35	0	0	0	0	0	0	0	0
MTCO3P38	0	0	0	0	0	0	0	0
MTCO3P39	0	0	0	0	0	0	0	0
MTCO3P4	0	0	0	0	0	0	0	0
MTCO3P40	0	0	0	0	0	0	0	0
MTCO3P41	0	0	0	0	0	0	0	0
MTCO3P42	0	0	0	0	0	0	0	0
MTCO3P43	0	0	0	0	0	0	0	0
MTCO3P44	0	0	0	0	0	0	0	0
MTCO3P45	0	0	0	0	0	0	0	0
MTCO3P46	0	0	0	0	0	0	0	0
MTCO3P47	0	0	0	0	0	0	0	0
MTCO3P5	0	0	0	0	0	0	0	0
MTCO3P7	0	0	0	0	0	0	0	0
MTCO3P8	0	0	0	0	0	0	0	0
MTCO3P9	0	0	0	0	0	0	0	0
MTCP1	0	10.081396	4.96404	12.633142	5.421058	7.787988	0	8.353778
MTCYBP1	0	0	0	0	0	0	0	0
MTCYBP10	0	0	0	0	0	0	0	0
MTCYBP11	0	0	0	0	0	0	0	0
MTCYBP12	0	0	0	0	0	0	0	0
MTCYBP13	0	0	0	0	0	0	0	0
MTCYBP14	0	0	0	0	0	0	0	0
MTCYBP15	0	0	0	0	0	0	0	0
MTCYBP16	0	0	0	0	0	0	0	0
MTCYBP17	0	0	0	0	0	0	0	0
MTCYBP18	0	0	0	0	0	0	0	0

MTCYBP19	0	0	0	0	0	0	0	0
MTCYBP2	0	0	0	0	0	0	0	0
MTCYBP20	0	0	0	0	0	0	0	0
MTCYBP21	0	0	0	0	0	0	0	0
MTCYBP22	0	0	0	0	0	0	0	0
MTCYBP23	0	0	0	0	0	0	0	0
MTCYBP24	0	0	0	0	0	0	0	0
MTCYBP27	0	0	0	0	0	0	0	0
MTCYBP28	0	0	0	0	0	0	0	0
MTCYBP29	0	0	0	0	0	0	0	0
MTCYBP3	0	0	0	0	0	0	0	0
MTCYBP31	0	0	0	0	0	0	0	0
MTCYBP32	3.500587	0	0	0	0	0	0	0
MTCYBP33	0	0	0	0	0	0	0	0
MTCYBP34	0	0	0	0	0	0	0	0
MTCYBP35	0	0	0	0	0	0	0	0
MTCYBP36	0	0	0	0	0	0	0	0
MTCYBP37	0	0	0	0	0	0	0	0
MTCYBP38	0	0	0	0	0	0	0	0
MTCYBP39	0	0	0	0	0	0	0	0
MTCYBP4	0	0	0	0	0	0	0	0
MTCYBP40	0	0	0	0	0	0	0	0
MTCYBP41	0	0	0	0	0	0	0	0
MTCYBP42	0	0	0	0	0	0	0	0
MTCYBP43	0	0	0	0	0	0	0	0
MTCYBP44	0	0	0	0	0	0	0	0
MTCYBP45	0	0	0	0	0	0	0	0
MTCYBP5	0	0	0	0	0	0	0	0
MTCYBP6	0	0	0	0	0	0	0	0
MTCYBP7	0	0	0	0	0	0	0	0
MTCYBP8	0	0	0	0	0	0	0	0
MTCYBP9	0	0	0	0	0	0	0	0
MTDH	165.34187	605.777043	132.129003	489.810995	84.771445	330.581077	241.622211	379.014253

MTDHP1	0	0	0	0	0	0	0	0
MTDHP2	0	0	0	0	0	0	0	0
MTDHP3	0	0	0	0	0	0	0	0
MTDHP4	0	0	0	0	0	0	0	0
MTDHP5	0	0	0	0	0	0	0	0
MTERF1	31.734198	2.792635	16.585778	3.47195	5.177072	0.323526	43.627977	14.255499
MTERF1P1	0	0	0	0	0	0	0	0
MTERF2	9.978264	11.798744	0	9.053726	26.805705	33.768417	0	28.245632
MTERF3	0	5.691743	0	5.047731	17.875724	5.411258	0	0.809169
MTERF4	33.477698	31.406059	16.424532	37.050591	5.20892	49.938001	5.129411	35.871884
MTF1	8.94352	13.342492	21.39697	14.07395	7.628078	29.095394	6.544631	35.235105
MTF2	81.812831	181.963057	56.263692	140.023215	40.758366	65.433538	38.333212	104.485344
MTFMT	20.232091	35.155517	16.521135	24.61516	33.826301	22.921238	22.727376	31.361244
MTFP1	13.279667	24.235744	10.705073	25.711533	4.67475	42.914305	0	35.668063
MTFR1	29.380946	96.263399	32.018233	83.157965	21.218699	58.29488	29.875671	61.390635
MTFR1L	20.149651	28.874511	15.025057	28.553731	37.558938	46.846371	9.506612	38.714423
MTFR1P1	0	0	0	0	0	0	0	0
MTFR2	13.678231	14.408369	6.276658	11.861841	24.769142	15.308884	22.693911	14.157475
MTFR2P1	0	0	0	0	0	0	0	0
MTFR2P2	0	0	0	0	0	0	0	0
MTG1	7.505052	27.111958	7.015505	34.115208	0	38.418388	106.006741	43.82248
MTG2	26.208105	40.118766	13.382289	66.641109	40.177393	76.682245	26.045263	52.116356
MTHFD1	232.278815	208.091581	194.601427	232.907283	236.950952	188.388151	237.763429	160.139035
MTHFD1L	43.891794	91.27318	38.795258	71.825427	79.08219	51.726681	134.900602	37.14297
MTHFD1P1	0	0.210448	0	0	0	0	0	0
MTHFD2	238.502185	205.119179	281.359637	133.50507	85.471601	68.031236	125.744678	90.823308
MTHFD2L	3.003944	2.69993	18.71671	0.941185	3.912321	1.081355	17.596451	3.452883
MTHFD2P1	0	0	0	0	0	0	0	0
MTHFD2P2	0	0	0	0	0	0	0	0
MTHFD2P3	0	0	0	0	0	0	0	0
MTHFD2P4	0	0	0	0	0	0	0	0
MTHFD2P5	0	0	0	0	0	0	0	0
MTHFD2P6	0	0	0	0	0	0	0	0

MTHFD2P7	0	0	0	0	0	0	0	0
MTHFR	4.291463	13.433674	13.720602	13.121846	3.278088	15.751027	3.361053	17.858078
MTHFS	0	24.996687	0	17.604104	0	9.82731	111.313875	15.433062
MTHFSD	26.935824	15.94636	15.396975	34.350978	57.69433	40.899168	35.301345	29.212662
MTIF2	0	18.744821	3.901754	14.982526	24.808349	17.925447	91.302355	22.715323
MTIF2P1	0	1.266003	0	0	0	0	0	0.093486
MTIF3	0	35.442587	0	21.914562	9.375789	10.885193	1.065434	18.52246
MTL3P	0	0	0	0	0	0	0	0
MTLN	0	0	0	0.044995	0	0	0	0
MTM1	0	5.489875	8.125274	1.214556	8.683667	2.10428	0	3.428103
MTMR1	49.964845	83.931938	34.041043	81.819565	30.413157	49.018788	26.785669	37.344969
MTMR10	30.909009	15.834262	12.384041	9.505886	19.283652	17.295635	45.501538	17.267741
MTMR11	3.613775	4.58734	5.267003	5.326306	9.776601	3.48529	0	4.166948
MTMR12	23.157335	35.788703	18.371548	31.03701	24.943596	40.275618	86.265288	45.285603
MTMR12P1	0	0	0	0	0	0	0	0
MTMR14	15.174569	30.965815	0	28.42429	11.708567	11.540183	1.268908	13.101254
MTMR2	61.747134	50.47166	54.046178	27.452906	11.904816	11.503933	13.664816	42.786427
MTMR3	38.741546	9.501086	1.570372	16.205729	23.69022	47.517683	10.679847	37.270714
MTMR4	28.42397	53.473624	33.062573	63.254595	39.573105	63.545348	29.339566	73.132208
MTMR7	0	0.082408	0.574808	1.347905	0	0	0	0.643654
MTMR8	0	0.921273	5.900586	2.203252	0.335839	1.202577	0	1.383957
MTMR9	2.741851	2.854852	1.232935	2.061774	2.415199	2.07532	0	4.507383
MTMR9LP	0	0.862296	0	0.644126	0	0.348819	0	0.106721
MTMR9P1	0	0	0	0	0	0	0	0
MTNAP1	15.444052	35.212083	13.069275	21.057466	9.446831	23.633771	7.530513	30.205122
MTND1P1	0	0	0	0	0	0	0	0
MTND1P10	0	0	0	0	0	0	0	0
MTND1P11	0	0	0	0	0	0	0	0
MTND1P12	0	0	0	0	0	0	0	0
MTND1P14	0	0	0	0	0	0	0	0
MTND1P15	0	0	0	0	0	0	0	0
MTND1P16	0	0	0	0	0	0	0	0
MTND1P17	0	0	0	0	0	0	0	0

MTND1P18	0	0	0	0	0	0	0	0
MTND1P19	0	0	0	0	0	0	0	0
MTND1P2	0	0	0	0	0	0	0	0
MTND1P20	0	0	0	0	0	0	0	0
MTND1P21	0	0	0	0	0	0	0	0
MTND1P22	0	0	0	0	0	0	0	0
MTND1P23	0	0	75.346651	1.905458	0	7.924899	0	14.582181
MTND1P24	0	0	0	0	0	0	0	0
MTND1P26	0	0	0	0	0	0	0	0
MTND1P27	0	0	0	0	0	0	0	0
MTND1P28	0	0	0	0	0	0	0	0
MTND1P29	0	0	0	0	0	0	0	0
MTND1P3	0	0	0	0	0	0	0	0
MTND1P30	0	0	0	0	0	0	0	0
MTND1P31	0	0	0	0	0	0	0	0
MTND1P32	0	0	0	0	0	0	0	0.170721
MTND1P33	0	0	0	0	0	0	0	0
MTND1P34	0	0	0	0	0	0	0	0
MTND1P35	0	0	0	0	0	0	0	0
MTND1P36	0	0	0	0	0	0	0	0
MTND1P37	0	0	0	0	0	0	0	0
MTND1P4	0	0	0	0	0	0	0	0
MTND1P5	0	0	0	0	0	0	0	0
MTND1P6	0	0	0	0	0	0	0	0
MTND1P7	0	0	0	0	0	0	0	0
MTND1P8	0	0	0	0	0	0	0	0
MTND1P9	0	0	0	0	0	0	0	0
MTND2P11	0	0	0	0	0	0	0	0
MTND2P12	0	0	0	0	0	0	0	0
MTND2P13	0	0	0	0	0	0	0	0
MTND2P14	0	0	0	0	0	0	0	0
MTND2P15	0	0	0	0	0	0	0	0
MTND2P16	0	0	0	0	0	0	0	0

MTND2P17	0	0	0	0	0	0	0	0
MTND2P18	0	0	0	0	0	0	0	0
MTND2P19	0	0	0	0	0	0	0	0
MTND2P2	0	0	0	0	0	0	0	0
MTND2P20	0	0	0	0	0	0	0	0
MTND2P21	0	0	0	0	0	0	0	0
MTND2P22	0	0	0	0	0	0	0	0
MTND2P23	0	0	0	0	0	0	0	0
MTND2P24	0	0	0	0	0	0	0	0
MTND2P25	0	0	0	0	0	0	0	0
MTND2P26	0	0	0	0	0	0	0	0
MTND2P28	273.588351	61.245254	218.81761	23.023023	199.815551	26.260653	262.370701	53.463288
MTND2P29	0	0	0	0	0	0	0	0
MTND2P3	0	0	0	0	0	0	0	0
MTND2P30	0	0	0	0	0	0	0	0
MTND2P31	0	0	0	0	0	0	0	0
MTND2P32	0	0	0	0	0	0	0	0
MTND2P33	0	0	0	0	0	0	0	0
MTND2P38	0	0	0	0	0	0	0	0
MTND2P39	0	0	0	0	0	0	0	0
MTND2P4	0	0	0	0	0	0	0	0
MTND2P40	0	0	0	0	0	0	0	0
MTND2P41	0	0	0	0	0	0	0	0
MTND2P5	0	0	0	0	0	0	0	0
MTND2P6	0	0	0	0	0	0	0	0
MTND2P7	0	0	0	0	0	0	0	0
MTND2P8	0	0	0	0	0	0	0	0
MTND2P9	0	0	0	0	0	0	0	0
MTND3P1	0	0	0	0	0	0	0	0
MTND3P10	0	0	0	0	0	0	0	0
MTND3P12	0	0	0	0	0	0	0	0
MTND3P13	0	0	0	0	0	0	0	0
MTND3P15	0	0	0	0	0	0	0	0

MTND3P16	0	0	0	0	0	0	0	0
MTND3P17	0	0	0	0	0	0	0	0
MTND3P18	0	0	0	0	0	0	0	0
MTND3P19	0	0	0	0	0	0	0	0
MTND3P2	0	0	0	0	0	0	0	0
MTND3P20	0	0	0	0	0	0	0	0
MTND3P21	0	0	0	0	0	0	0	0
MTND3P22	0	0	0	0	0	0	0	0
MTND3P23	0	0	0	0	0	0	0	0
MTND3P24	0	0	0	0	0	0	0	0
MTND3P25	0	0	0	0	0	0	0	0
MTND3P3	0	0	0	0	0	0	0	0
MTND3P4	0	0	0	0	0	0	0	0
MTND3P5	0	0	0	0	0	0	0	0
MTND3P6	0	0	0	0	0	0	0	0
MTND3P7	0	0	0	0	0	0	0	0
MTND3P8	0	0	0	0	0	0	0	0
MTND3P9	0	0	0	0	0	0	0	0
MTND4LP1	0	0	0	0	0	0	0	0
MTND4LP10	0	0	0	0	0	0	0	0
MTND4LP11	0	0	0	0	0	0	0	0
MTND4LP12	0	0	0	0	0	0	0	0
MTND4LP13	0	0	0	0	0	0	0	0
MTND4LP14	0	0	0	0	0	0	0	0
MTND4LP16	0	0	0	0	0	0	0	0
MTND4LP17	0	0	0	0	0	0	0	0
MTND4LP18	0	0	0	0	0	0	0	0
MTND4LP19	0	0	0	0	0	0	0	0
MTND4LP2	0	0	0	0	0	0	0	0
MTND4LP20	0	0	0	0	0	0	0	0
MTND4LP21	0	0	0	0	0	0	0	0
MTND4LP22	0	0	0	0	0	0	0	0
MTND4LP24	0	0	0	0	0	0	0	0

MTND4LP25	0	0	0	0	0	0	0	0
MTND4LP26	0	0	0	0	0	0	0	0
MTND4LP3	0	0	0	0	0	0	0	0
MTND4LP30	0	0	0	0	0	0	0	0
MTND4LP31	0	0	0	0	0	0	0	0
MTND4LP32	0	0	0	0	0	0	0	0
MTND4LP5	0	0	0	0	0	0	0	0
MTND4LP7	0	0	0	0	0	0	0	0
MTND4LP9	0	0	0	0	0	0	0	0
MTND4P1	0	0	0	0	0	0	0	0
MTND4P10	0	0	0	0	0	0	0	0
MTND4P11	0	0	0	0	0	0	0	0
MTND4P12	29.052494	5.200626	28.161811	3.832747	4.180005	7.38338	48.391471	6.107477
MTND4P13	0	0	0	0	0	0	0	0
MTND4P14	0	0	0	0	0	0	0	0
MTND4P15	0	0	0	0	0	0	0	0
MTND4P16	0	0	0	0	0	0	0	0
MTND4P17	0	0	0	0	0	0	0	0
MTND4P18	0	0	0	0	0	0	0	0
MTND4P19	0	0	0	0	0	0	0	0
MTND4P2	0	0	0	0	0	0	0	0
MTND4P20	0	0	0	0	0	0	0	0
MTND4P21	0	0	0	0	0	0	0	0
MTND4P22	0	0	0	0	0	0	0	0
MTND4P23	0	0	0	0	0	0	0	0
MTND4P24	0	0	0	0	0	0	0	0
MTND4P25	0	0	0	0	0	0	0	0
MTND4P26	0	0	0	0	0	0	0	0
MTND4P27	0	0	0	0	0	0	0	0
MTND4P28	0	0	0	0	0	0	0	0
MTND4P29	0	0	0	0	0	0	0	0
MTND4P3	0	0	0	0	0	0	0	0
MTND4P30	0	0	0	0	0	0	0	0

MTND4P31	0	0	0	0	0	0	0	0
MTND4P32	0	0	2.652668	0	0	0	0	0
MTND4P33	0	0	0	0	0	0	0	0
MTND4P34	0	0	0	0	0	0	0	0
MTND4P35	0	0	0	0	0	0	0	0
MTND4P4	0	0	0	0	0	0	0	0
MTND4P5	0	0	0	0	0	0	0	0
MTND4P6	0	0	0	0	0	0	0	0
MTND4P7	0	0	0	0	0	0	0	0
MTND4P8	0	0	0	0	0	0	0	0
MTND4P9	0	0	0	0	0	0	0	0
MTND5P1	0	0	0	0	0	0	0	0
MTND5P10	0	0	0	0	0	0	0	0
MTND5P11	0	0.245446	0	0.234802	1.020284	0.191118	0.468095	0.078208
MTND5P12	0	0	0	0	0	0	0	0
MTND5P13	0	0	0	0	0	0	0	0
MTND5P14	0	0	0	0	0	0.142093	0	0
MTND5P15	0	0	15.027157	0	0	0	0	0
MTND5P16	0	0	0	0	0	0	0	0
MTND5P17	0	0	0	0	0	0	0	0
MTND5P18	0	0	0	0	0	0	0	0
MTND5P19	0	0	0	0	0	0	0	0
MTND5P2	0	0	0	0	0	0	0	0
MTND5P20	0	0	0	0	0	0	0	0
MTND5P21	0	0	0	0	0	0	0	0
MTND5P22	0	0	0	0	0	0	0	0
MTND5P23	0	0	0	0	0	0	0	0
MTND5P24	0	0	0	0	0	0	0	0
MTND5P25	0	0	0	0	0	0	0	0
MTND5P26	0	0	0	0	0	0	0	0
MTND5P27	0	0	0	0	0	0	0	0
MTND5P28	0	0	0	0	0	0	0	0
MTND5P29	0	0	0	0	0	0	0	0

MTND5P3	0	0	0	0	0	0	0	0
MTND5P30	0	0	0	0	0	0	0	0
MTND5P31	0	0	0	0	0	0	0	0
MTND5P32	0	0	0	0	0	0	0	0
MTND5P33	0	0	0	0	0	0	0	0
MTND5P34	0	0	0	0	0	0	0	0
MTND5P35	0	0	0	0	0	0	0	0
MTND5P4	0	0	0	0	0	0	0	0
MTND5P40	0	0	0	0	0	0	0	0
MTND5P41	0	0	0	0	0	0	0	0
MTND5P42	0	0	0	0	0	0	0	0
MTND5P5	0	0	0	0	0	0	0	0
MTND5P6	0	0	0	0	0	0	0	0
MTND5P7	0	0	0	0	0	0	0	0
MTND5P8	0	0	0	0	0	0	0	0
MTND5P9	0	0	0	0	0	0	0	0
MTND6P1	0	0	0	0	0	0	0	0
MTND6P10	0	0	0	0	0	0	0	0
MTND6P11	0	0	0	0	0	0	0	0
MTND6P12	0	0	0	0	0	0	0	0
MTND6P13	0	0	0	0	0	0	0	0
MTND6P14	0	0	0	0	0	0	0	0
MTND6P15	0	0	0	0	0	0	0	0
MTND6P16	0	0	0	0	0	0	0	0
MTND6P17	0	0	0	0	0	0	0	0
MTND6P18	0	0	0	0	0	0	0	0
MTND6P19	0	0	0	0	0	0	0	0
MTND6P2	0	0	0	0	0	0	0	0
MTND6P20	0	0	0	0	0	0	0	0
MTND6P21	0	0	0	0	0	0	0	0
MTND6P22	0	0	0	0	0	0	0	0
MTND6P24	0	0	0	0	0	0	0	0
MTND6P25	0	0	0	0	0	0	0	0

MTND6P29	0	0	0	0	0	0	0	0
MTND6P3	0	0	0	0	0	0	0	0
MTND6P32	0	0	0	0	0	0	0	0
MTND6P33	0	0	0	0	0	0	0	0
MTND6P35	0	0	0	0	0	0	0	0
MTND6P4	0	0	0	0.435278	0	0	0	0
MTND6P5	0	0	0	0	0	0	0	0
MTND6P6	0	0	0	0	0	0	0	0
MTND6P7	0	0	0	0	0	0	0	0
MTND6P8	0	0	0	0	0	0	0	0
MTND6P9	0	0	0	0	0	0	0	0
MTNR1A	0	0.237891	0	0.206414	0	0.696763	0	0
MTNR1B	0	0	0	0	0	0	0	0
MTO1	9.503089	23.415887	10.209398	23.486595	17.059986	14.356155	2.687346	25.52308
MTOR	0	0	20.81306	0	4.087762	0	15.296944	2.400787
MTPAP	0	8.98719	5.171233	3.508956	3.129791	3.381766	35.550442	7.269849
MTPN	90.220558	75.036826	87.233324	71.64844	125.045511	64.902523	82.680793	81.367713
MTR	35.065666	45.714454	27.478482	49.505072	18.274588	46.260232	29.208378	70.989271
MTRES1	29.334898	27.005925	14.494425	23.507915	22.551657	14.488499	20.889285	17.393615
MTRES1P1	0	0	0	0	0	0	0	0
MTRES1P2	0	0	0	0	0	0	0	0
MTREX	98.9139	70.32631	47.924584	93.730896	153.950839	57.377582	201.307581	74.133793
MTRF1	6.302611	19.992755	20.670643	10.811654	9.277032	9.289958	25.62792	10.971767
MTRF1L	19.740248	12.742975	20.457086	8.262753	8.383712	6.105609	0.758406	6.495406
MTRF1LP1	0	0	0	0	0	0	0	0
MTRF1LP2	0	0	0	0	0	0	0	0
MTRFR	27.416792	15.497426	0	18.493258	2.178018	3.185733	16.443101	8.25711
MTRR	23.850391	34.728142	76.116713	54.874887	23.806613	35.360957	35.223682	30.713562
MTSS1	8.111391	43.770691	0	85.027475	23.703513	52.083168	13.902622	59.10928
MTSS2	49.716307	39.044045	14.761283	35.166422	129.761772	121.12083	57.827591	89.396321
MTTP	0	0	0	0	0	0	0	0
MTURN	27.10567	16.055808	6.603107	14.443172	13.98397	10.295843	34.133285	23.616909
MTUS1	43.47407	51.421606	25.96164	64.638843	57.616832	57.154402	111.170188	82.963952

MTUS2	0	0.957521	0	0.191383	0	0.377616	0	0.261157
MTX1	41.337769	29.756716	42.113866	31.453094	45.024188	27.755423	21.795509	27.149851
MTX1LP	0	0	0	0	0	0	0	0
MTX2	51.742434	71.837849	31.433469	53.075019	43.972316	62.740942	113.164731	93.797469
MTX2P1	0	0	0	0	0	0	0	0
MTX3	6.36798	3.898446	4.563903	2.528653	5.244535	1.043215	0	3.004888
MUC1	10.765051	1.757301	0	2.861102	37.078009	12.634975	24.28815	15.706662
MUC12	0	4.089219	0	2.55801	0	0.448984	0	0
MUC13	0	0	0	0	0	0	0	0
MUC15	0	0	0	0	0	0	0	0.045532
MUC16	0	0	0.06511	0.017713	0	0.040049	0	0.00289
MUC17	0	0	0	0	0	0	0	0
MUC19	0	0	0	0	0	0	0	0
MUC2	0	0	0	0	0	0	0	0
MUC20	11.27021	4.098993	6.519631	9.267107	5.505203	11.429462	0	3.245842
MUC20P1	0	0.363785	0	0.432023	0	0.99686	0	0.196412
MUC21	0	0	0	0	0	0	0	0
MUC22	0	0	0	0	0	0.02708	0	0
MUC3A	0.284872	1.577555	0.25645	4.739852	0	1.02353	0	0.565194
MUC4	0.143863	0.78103	0.648066	1.889872	3.06062	0.349497	0.421725	0.148592
MUC5AC	0	0.055202	0	0.076054	0	0	0	0
MUC5B	0	0	0	0	0	0	0	0
MUC6	0	0	0	0	0	0	0	0
MUC7	0	0	0	0	0	0	0	0.289355
MUCL1	0	0	0	0	0	0	0	0
MUCL3	0	0	0	0.101486	0	0.103179	0	0
MUL1	22.469694	49.126015	21.355762	40.518069	38.898703	55.31883	36.107365	51.991774
MUPP	0	0	0	0	0	0	0	0
MUS81	14.542526	28.956697	57.487887	31.154306	24.070817	28.678796	83.26706	24.084313
MUSK	0	0	0	0.068951	0	0	0	0
MUSTN1	0	0	0	0	0	0	0	0
MUTYH	18.487541	10.257476	6.191093	12.757673	32.608157	13.586076	0	10.716585
MVB12A	0	20.754849	10.483468	20.898331	22.546476	37.211434	64.444557	33.628602

MVB12B	2.539427	3.843112	0	3.927024	11.518981	7.607467	0	9.520414
MVD	36.249748	46.096799	40.443382	31.402752	11.550294	32.62278	20.321687	25.782963
MVK	29.109212	25.65309	19.485663	14.408746	26.255024	40.022297	0	23.935086
MVP	12.056063	15.663292	20.513388	29.737657	28.905971	32.407281	25.750713	18.400861
MX1	15.229752	15.435164	0	18.739537	77.389312	156.0897	41.10955	130.258739
MX2	0	4.620604	0	2.277619	24.429962	24.562997	52.466383	27.351665
MXD1	9.971766	6.327136	2.639669	12.715918	2.160379	14.563142	3.292419	5.726718
MXD3	0	3.000491	10.289567	1.714518	0	4.834578	0	0.449312
MXD4	4.273931	21.258527	20.292999	15.301353	6.094409	18.488006	3.535597	13.880754
MXI1	19.075607	42.320812	38.954614	89.231956	46.031263	73.3161	22.557428	71.265776
MXRA5	7.863142	12.600583	5.60254	12.178022	6.06202	5.219484	2.158727	3.530334
MXRA5Y	0	0	0	0	0	0	0	0
MXRA7	7.015342	30.095435	69.861327	38.241657	6.38541	41.417389	8.83456	5.318318
MXRA7P1	0	0	0	0	0	0	0	0
MXRA8	0	0.26516	0	0	0	0.155428	0	0
MYADM	23.059744	13.212673	34.36343	22.351531	15.361223	11.115215	45.891818	6.22755
MYADML	0	0	0	0	0	0	0	0
MYADML2	0	0	0	0	0	0	0	0
MYB	4.779429	5.214613	0	6.110287	4.645464	5.747021	12.630639	2.997289
MYBBP1A	24.202638	36.211234	61.663794	26.523664	47.808905	63.96267	65.533624	38.21869
MYBL1	11.231699	10.452523	5.076615	5.239938	0	12.781763	10.463445	23.340664
MYBL2	39.236601	57.673906	20.443319	54.150103	101.767056	126.892639	74.527303	94.000643
MYBPC1	0	0	0	0	0	0	0	0
MYBPC2	0	0	0	0.233276	0	0	0	0
MYBPC3	0	0	0	0	0	0	0	0
MYBPH	0	0	0	0	0	0	0	0
MYBPHL	0	0	0	0	0	0	0	0
MYC	181.619619	367.230531	208.115302	725.916547	206.334184	1145.59336	389.137905	980.956206
MYCBP	17.448124	89.557284	76.643063	28.614532	6.443128	15.737156	10.042003	67.872074
MYCBP2	16.081918	20.763768	2.018521	13.062003	50.864189	33.67959	14.763791	16.066
MYCBPAP	0	0	0	0	0	0	0	0
MYCL	0	0.528159	0	0.245843	2.991294	8.342539	0	4.902109
MYCLP1	0	0	0	0	0	0	0	0

MYCLP2	0	0	0	0	0	0	0	0
MYCN	0	0	0	0	9.352792	0	0	0
MYCT1	0	0	0	0	0	0	0	0
MYD88	0	0	0	1.326374	39.86905	3.759975	0	0.970427
MYDGF	0	0	0	0	0	0	0	0
MYEF2	28.253954	35.477712	9.604992	32.759652	11.08183	17.485992	76.97289	23.082837
MYEOV	26.075933	40.108115	12.343968	39.666415	0	0	0	0
MYF5	0	0	0	0	0	0	0	0
MYF6	0	0	0	0	0	0	0	0
MYG1	68.089606	43.75942	54.844994	41.814868	7.201961	30.006031	62.465852	30.493398
MYG1P1	0	0	0	0	0	0	0	0
MYH1	0	0	0	0	0	0	0	0
MYH10	24.866879	20.093872	15.581278	19.58807	78.122671	31.827597	23.338497	40.181836
MYH11	0	0	0	0	0	0.040363	0	0
MYH13	0	0	0	0	0	0	0	0
MYH14	4.295887	2.433984	0	1.000879	23.961886	4.794436	5.211853	3.320426
MYH15	0	1.50616	0	1.871901	1.375978	0.7017	0	1.161077
MYH16	1.672886	1.969509	0	2.164971	0	0.35972	0	0.060182
MYH2	0	0	0	0	0	0	0	0
MYH3	1.077881	0.301206	0	0.408557	0.142398	0.351269	0	0.021612
MYH4	0	0	0	0	0	0	0	0
MYH6	0	0	1.402524	0	0	0	0	0
MYH7	0	0	0	0.013191	0	0.027044	0	0
MYH7B	0	0	0	0	0	0	0	0
MYH8	0	0	0	0	0	0	0	0
MYH9	31.499226	67.170586	0	105.346731	44.91086	93.521028	78.96375	142.884213
MYL1	0	0	0	0	0	0	0	0
MYL10	0	0	0	0	0	0	0	0
MYL11	0	1.185368	0	0.353912	0	0	0	0
MYL12A	132.024	96.305965	129.442368	45.686971	112.198067	115.486577	17.184639	69.997521
MYL12AP1	0	0	0	0.220975	0	0	0	0
MYL12B	194.054784	99.405805	90.667437	7.644435	114.379474	2.421987	258.298902	32.041946
MYL12BP1	0	0	0	0	0	0	0	0

MYL12BP2	0	0.368085	0	0	0	0	0	0
MYL12BP3	0	0	0	0	0	0	0	0
MYL2	0	0	8.805815	0.718342	0	0	0	0
MYL3	0	1.189427	0	0.439661	0	0.29812	0	0.08141
MYL4	0	0	0	0	0	0	0	0
MYL5	7.862583	12.701194	0	5.471648	0	12.824097	0	15.110898
MYL6	697.765191	834.765045	549.960726	861.551044	749.773623	844.233617	621.004785	782.831723
MYL6B	100.479997	62.016936	94.274092	98.818492	50.251449	39.432826	0	40.214727
MYL6BP1	0	0	0	0	0	0	0	0
MYL6P1	0	0	0	0	0	0	0	0
MYL6P2	0	0	0	0	0	0	0	0
MYL6P3	0	0	0	0	0	0	0	0
MYL6P4	0	0	0	0	0	0	0	0.492541
MYL6P5	0	0	0	0	0	0	0	0
MYL7	0	0	0	0	0	0	0	0
MYL9	45.0061	26.210081	43.380983	50.363372	12.937925	32.076566	38.608379	18.690172
MYLIP	6.54744	9.454386	0	9.551278	0	11.758283	0	10.312613
MYLK	8.543475	1.394644	0	0.576884	0	0.217521	1.309548	0.183551
MYLK2	1.136898	0	0	0.156958	0	0.215883	0	0
MYLK3	0	0.154016	7.162453	0.672208	0	0	0	0
MYLK4	0.586582	0.151212	0	0	0	0	3.859278	0
MYLKP1	0	0	0	0	0	0	0	0
MYLKP2	0	0	0	0	0	0	0	0
MYMK	0	0	0	0	0	0	0	0
MYNN	16.749174	23.883418	5.962511	13.395004	9.858733	10.985617	0.625426	17.050226
MYO10	79.006231	202.391457	77.609454	216.341165	57.764337	120.59637	94.102163	128.535074
MYO15A	0	2.374579	0	0.797367	0	0	0	0.039897
MYO15B	0	0.049028	0	0.792739	0	0.537169	0	0
MYO16	1.0745	0.121812	0.424292	0.24239	0	0.267754	0	0.037827
MYO18A	34.431677	11.849725	0	24.384697	74.773322	85.831992	113.755353	38.043828
MYO18B	0	0.087748	0	0.074852	0	0	0	0
MYO19	52.514891	125.645103	32.590247	145.337374	69.817157	209.152455	98.972929	201.048143
MYO1A	0	0	0	0.044593	0	0.053877	0	0

MYO1B	142.548055	377.325653	189.388017	375.92337	209.42735	275.637036	508.654804	536.856221
MYO1C	56.042916	99.928118	96.572863	91.712247	95.016136	233.982462	136.260919	136.391191
MYO1D	207.9253	64.709654	35.629682	63.856485	79.420064	41.282645	192.187809	60.085172
MYO1E	103.874556	407.349248	136.749756	465.299524	57.174932	536.882754	370.346561	479.949027
MYO1F	0	0	0	1.471581	0	0	0	0
MYO1G	0	0	0	0	0	0.115127	0	0.157604
MYO1H	0	0	0	0.150904	0	0	0	0
MYO3A	14.600801	1.308074	1.631995	0.482073	0	0	0	0
MYO3B	0	0.591445	0	0.049731	0.265299	0	0	0.080791
MYO5A	0	0	0	0	42.340874	0	0	0.629915
MYO5B	32.930339	18.585153	27.969948	21.081245	10.252555	41.657261	6.021875	57.660536
MYO5BP1	0	0	0	0	0	0	0	0
MYO5BP2	0	0	0	0	0	0	0	0
MYO5BP3	0	0	0	0	0	0	0	0
MYO5C	7.423535	10.632439	16.741494	13.016667	15.266785	28.910646	14.24637	25.328609
MYO6	29.537928	23.489127	17.92147	25.789023	0.437104	25.306961	0	16.580458
MYO7A	5.688907	6.016291	5.920503	3.970958	0.329859	1.067528	7.28344	0.770611
MYO7B	0	0	0	0	0	0.372019	0	0.618639
MYO9A	7.154437	35.694699	4.224698	28.292408	16.970998	29.251647	14.226846	46.836244
MYO9B	25.908727	51.67983	16.590114	44.914957	22.998725	114.675282	244.875708	77.208721
MYOC	0	0	0	0	0	0	0	0
MYOCD	1.196908	0.112317	0	0.759077	0	0.289607	0	0.240795
MYOCOS	0	0	0	0	0	0	0	0
MYOD1	0	0	0	0.047211	0	0	0	0
MYOF	246.125988	306.937481	272.39194	299.648251	264.657325	228.433708	179.370915	256.109015
MYOG	0	0	0	0	0	0	0	0
MYOM1	0	0.432662	0	0.421724	0	0	0	0
MYOM2	0	2.56517	6.333988	3.665125	0	1.643698	0	1.4644
MYOM3	0	0.32095	0.576674	0.29022	0	0.424955	0	0
MYORG	7.548472	9.242474	4.524595	8.480612	3.457278	11.888236	4.66197	10.75386
MYOT	0	0	0	0	0	0	0	0
MYOZ3	0	0.216743	0	0.098977	0.267856	1.810608	0	0.73418
MYPOP	3.681307	4.045184	3.284874	3.886629	0.970332	9.368776	0	5.12718

MYRF	0	1.607522	0	2.954057	9.847769	3.76529	1.519318	2.122443
MYRFL	0	0	0	0.203784	0	0.135782	0	0
MYRIP	0	0.215232	0	0	0	0.670839	0	0.05086
MYSM1	17.07714	10.518194	16.070668	10.648537	5.359653	2.512277	6.911818	7.060112
MYT1	0	1.584684	1.587931	3.277865	0	0.088541	0	0.135585
MYT1L	0	0	0	0	0	0	0	0
MYZAP	7.685713	16.339785	2.793259	8.811884	0.445105	4.739043	7.577778	11.510123
MZB1	0	0	0	0.164195	0	0	0	0
MZF1	2.622898	2.061807	0	4.895271	1.510724	10.509497	23.817342	7.559456
MZF1-AS1	12.20735	28.104355	12.419317	26.173285	3.40014	45.38822	10.290505	43.326172
MZT1	12.315857	12.737283	17.888529	9.060273	9.744196	6.182706	31.342741	16.878209
MZT1P1	0	0	0	0	0	0	0	0
MZT1P2	0	0	0	0	0	0	0	0
MZT2A	66.945692	55.117486	42.63729	68.372589	61.927215	162.370864	85.420407	113.650356
MZT2B	0	142.156847	69.304241	106.882081	110.270097	171.525565	205.565379	157.86242
N4BP1	50.769981	141.138822	50.498573	94.917454	33.408555	152.587527	259.312293	201.072104
N4BP2	2.974651	4.642912	7.765281	2.957413	5.65672	2.536771	5.403939	7.147198
N4BP2L1	0	0.871465	2.901845	5.148079	3.394186	3.462361	0	5.005443
N4BP2L2	15.414844	28.852629	55.47208	28.7417	12.288301	10.224481	57.029197	21.302883
N4BP3	2.716534	6.446266	6.348941	7.625989	3.445222	5.502124	6.621172	4.052441
N6AMT1	0	3.840123	3.078908	3.171748	0.928585	2.46133	15.64046	4.045294
NAA10	159.459999	197.656049	135.326193	173.874815	156.244506	206.782602	108.059464	187.496765
NAA11	0	0	0	0	0	0	0	0
NAA15	38.410056	86.020385	53.344084	45.513384	65.602377	26.655701	55.669903	81.399777
NAA16	7.313246	11.119419	4.829476	5.992469	35.91485	5.674545	8.809824	14.380287
NAA20	98.03087	36.224428	13.659805	22.74773	0	20.180126	179.197636	56.259907
NAA20P1	0	0	0	0	0	0	0	0
NAA25	26.503841	21.253281	42.653857	25.659103	37.305417	22.491489	26.258519	40.440632
NAA30	16.754663	18.300995	23.82215	6.458597	35.925375	5.627929	3.938932	18.051936
NAA35	0	10.086912	0	2.168955	31.510236	8.261276	0	12.680094
NAA38	9.102883	37.805116	34.219118	40.737524	56.335023	83.372956	0	78.058163
NAA40	11.824089	30.154166	7.343026	49.388353	28.373588	79.768516	19.689589	70.60783
NAA40P1	0	0	0	0	0	0	0	0

NAA50	277.913056	564.47084	248.123917	424.419678	207.653678	522.902681	197.805871	607.713715
NAA50P1	0	0	0	0	0	0	0	0
NAA50P2	0	1.019237	0	0	0	0.199507	0	0
NAA60	25.763509	110.541285	39.899203	122.80025	72.868782	154.860384	25.182566	148.822903
NAA80	9.654674	3.780971	2.849476	8.709416	0	3.818229	0	4.28747
NAAA	5.77482	21.845282	50.709843	17.758496	42.226003	10.316992	11.549641	4.918926
NAALAD2	0	0	0	0	0	0	0	0
NAALADL1	0	0	0	0	0	0	0	0
NAALADL2	0	0.42762	0	3.36128	0	0.301696	0	0
NAB1	82.869507	123.794377	24.216306	129.991023	59.198234	138.174465	47.897595	174.96196
NAB1P1	0	0	0	0	0	0	0	0
NAB2	19.49035	40.019003	32.999067	47.299144	27.265291	32.101903	0.662813	22.996683
NABP1	53.392474	45.526625	15.302934	63.751614	6.732827	45.802737	11.379155	39.631747
NABP2	89.796163	99.347702	75.447484	103.296093	66.958569	67.8204	24.973853	70.813713
NACA	627.198638	940.94182	472.144432	774.507123	233.367165	601.80713	877.583765	723.652431
NACA3P	0	12.155856	5.888425	7.692223	0	3.503432	0	2.249969
NACA4P	0	0	0	0.162538	0	0.645538	0	0
NACAD	0.677487	1.511777	2.43412	1.738166	9.305872	12.480467	3.795066	7.693869
NACAP10	0	0	0	0	0	0	0	0
NACAP2	0	0	0	0	0	0	0	0
NACAP5	0	0	0	0	0	0	0	0
NACAP6	0	0	0	0	0	0	0	0
NACAP7	0	0	0	0	0	0	0	0
NACAP8	0	0	0	0	0	0	0	0
NACC1	18.880844	78.751961	26.083683	51.738665	50.636741	214.995511	53.574987	196.024599
NACC2	6.667345	5.147168	1.713025	4.641713	3.020307	7.084493	0	6.694079
NADK	48.974259	35.802483	37.872928	36.875315	37.356499	121.043922	26.166985	84.564645
NADK2	44.522114	37.222744	19.852678	24.155306	3.91455	19.360268	0	34.409405
NADSYN1	15.016505	13.077874	67.930995	7.873319	26.3853	6.72351	6.501663	16.69179
NAE1	147.015469	184.798423	130.678058	64.826048	148.125445	43.038501	118.77701	134.536687
NAF1	26.398522	12.474276	24.400817	13.590033	11.781111	10.408023	15.00213	10.258495
NAGA	0	0	0	10.783452	0	5.699344	0	2.841113
NAGK	51.373634	25.926512	11.885613	44.147102	34.144298	46.199913	119.838159	23.032113

NAGLU	8.253303	2.865965	0	3.148664	13.784378	9.312217	42.451993	3.916935
NAGPA	4.593393	6.764343	11.159613	8.856304	2.904066	9.806262	0	13.318171
NAGS	0	5.271644	1.588283	4.199924	0.46906	1.833222	12.920525	4.015304
NAIF1	4.90284	17.989386	3.516987	18.010078	6.682455	17.931423	27.063591	17.965415
NAIP	10.000395	1.221147	0	0.921767	0	1.737436	0	0.354676
NAIPP1	0	0	0	0	0	0	0	0
NAIPP2	0	0	0	0.554377	0	0.31782	0	0.445926
NAIPP4	0	0	0	0	0	0	0	0
NALCN	6.463907	2.871164	5.538721	3.36304	0.950464	0.669573	0	1.07149
NAMPT	70.206056	62.721434	118.447576	34.219202	32.924041	5.931777	95.181119	23.071689
NAMPTP1	0	0.077133	0	0.039715	0	0	0	0
NAMPTP2	0	0	0	0	0	0	0	0
NAMPTP3	0	0	0	0	0	0	0	0
NANOG	0	0	0	0	0	0	0	0
NANOGNB	0	0	0	0	0	0	0	0
NANOGNBP1	0	0	0	0	0	0	0	0
NANOGNBP2	0	0	0	0	0	0	0	0
NANOGNBP3	0	0	0	0.193292	0	0	0	0
NANOGP1	0	0	0	0	0	0	0	0
NANOGP10	0	0	0	0	0	0	0	0
NANOGP2	0	0	0	0	0	0	0	0
NANOGP3	0	0	0	0	0	0	0	0
NANOGP4	0	0	0	0	0	0	0	0
NANOGP5	0	0	0	0	0	0	0	0
NANOGP6	0	0	0	0	0	0	0	0
NANOGP7	0	0	0	0	0	0	0	0
NANOGP9	0	0	0	0	0	0	0	0
NANOS1	6.577844	11.938152	11.548792	4.648255	1.823772	6.617231	0	10.386548
NANOS3	12.404917	0	0	1.097619	0	3.319149	0	2.112941
NANP	15.67522	22.133655	8.594835	12.709829	7.358487	13.745897	22.245773	25.459345
NANS	75.995889	135.328115	81.330127	126.821536	88.10655	111.039916	76.81262	89.114137
NAP1L1	606.530164	779.568066	658.612328	576.010668	340.864188	293.981375	476.557124	694.049714
NAP1L1P1	3.584206	0	0	0.258415	0	0	0	1.022988

NAP1L1P2	0	0	0	0	0	0	0	0
NAP1L1P3	0	0	0	0	0	0	0	0
NAP1L3	6.64573	0	2.378903	0	0.350673	0	0	0
NAP1L4	146.09192	239.677512	147.118885	214.061059	182.316283	301.865918	337.566734	283.64669
NAP1L4P1	0	1.075264	2.866721	0.233887	0	0.31517	0	0.26334
NAP1L4P2	0	0	0	0	0	0	0	0
NAP1L4P3	0	0	0	0	0	0	0	0
NAP1L5P1	0	0	0	0	0	0	0	0
NAP1L6P	0	0	0	0	0	0	0	0
NAPA	23.451886	44.605412	20.892001	42.489415	69.586779	102.068754	119.767631	83.000728
NAPB	1.765594	1.185997	2.376054	1.809563	0.69931	1.615217	0	1.793496
NAPEPLD	14.416012	10.906271	2.189357	7.451834	3.469386	3.6002	1.861074	7.360086
NAPG	41.510063	26.456513	20.699349	20.835955	13.89774	12.775962	0	11.636107
NAPGP1	0	0	0	0	0	0	0	0
NAPGP2	0	0	0	0	0	0	0	0
NAPRT	48.228532	14.114882	11.48776	10.506512	91.779027	29.427829	5.502056	21.250214
NAPSA	0	0.645882	0	0.04952	0	0	0	0
NAPSB	0	0	0	0	0	0	0	0
NARF	36.889422	61.355444	29.0929	67.477914	78.595018	48.880923	193.089001	103.451447
NARS1	130.088605	219.237767	133.081213	271.486958	272.497227	221.569016	213.297781	227.65756
NARS1P2	0	0	0	0	0	0	0	0
NARS2	32.226174	33.863551	37.548727	31.808837	14.243211	15.95253	11.004323	26.237531
NASP	218.766591	181.884364	158.4445	232.694521	402.67761	330.743869	330.520779	280.172634
NASPP1	0	0.135137	0	0.155204	0	0.239349	0	0
NAT1	4.993555	8.360353	6.659021	5.363299	0	1.251772	2.414586	4.951318
NAT10	44.043258	30.448574	37.323076	38.530251	50.106462	63.07504	109.093852	65.305489
NAT14	0	20.440621	38.240591	25.55908	29.439928	33.413574	0	22.080274
NAT16	0	0	0	0.194427	0	0	0	0
NAT2	0	0	0	0.280727	0	0	0	0
NAT8B	0	0	0	0	0	0	0	0
NAT9	30.048664	13.823464	18.450662	19.973279	0.991097	16.795983	7.732167	6.485685
NATD1	0.66393	1.795246	0	3.082555	1.403059	4.221568	0.161713	2.477606
NATP	0	0	0	0	0	0	0	0

NAV1	22.608888	52.312265	27.008803	55.516033	16.638969	42.173196	18.936464	32.478991
NAV2	11.425086	21.718998	21.68861	28.513253	6.281779	48.739806	55.254598	33.542271
NAV3	9.459698	39.674925	19.451866	38.591887	6.595369	4.793082	0	8.864862
NAXD	14.370093	23.312183	15.230043	15.276948	11.371644	18.577753	0	24.906028
NAXE	132.548215	150.690151	103.386848	134.300581	161.810694	175.705508	96.197278	153.221489
NBAS	15.105712	14.888536	12.787039	13.595213	23.683945	5.084083	11.15995	7.782314
NBDY	32.260034	37.15689	25.47168	46.225854	26.701241	33.760301	49.727501	20.344139
NBEAL1	3.944239	10.459801	8.970213	11.070774	4.459018	7.705259	14.7365	18.095842
NBEAL2	4.117052	8.085315	0	1.916351	21.329745	9.505814	8.106178	11.746647
NBEAP1	0	0	0	0	0	0	0	0.323489
NBEAP2	0	0.329892	0	0	0.526769	0	0.959337	4.097804
NBEAP3	0	0	0	0	0	0	0	0
NBEAP4	0	0	0	0	0	0	0	0
NBEAP5	0	0	0	0	0	0	0	0
NBEAP6	0	0	0	0	0	0	0	0
NBL1	90.130787	82.243513	27.535188	79.643925	52.59387	142.915804	68.986581	97.482983
NBN	24.696024	11.772344	25.437427	1.05899	52.738601	3.43121	28.904021	7.03555
NBPF1	19.493067	26.482472	6.711972	30.505979	7.344525	35.784675	0	39.214483
NBPF10	0	4.679173	7.89853	5.540371	8.541438	5.608745	0	3.888056
NBPF11	3.402479	3.171081	4.420449	6.23152	5.619913	4.3284	11.254747	4.66788
NBPF12	0	0.830512	3.133022	1.618133	2.675058	1.292802	0	1.276179
NBPF13P	0	0	0	0	0	0	0	0
NBPF14	38.009121	15.73802	25.344354	21.695871	21.726988	22.539027	6.216292	19.583823
NBPF15	10.97333	6.738691	0	15.034336	0	12.922066	0	12.399357
NBPF17P	0	0	0	0	0	0	0	0
NBPF18P	0	1.599598	0	0.959283	0	0	0	0
NBPF19	21.317092	14.555396	11.337241	15.988881	13.799614	8.341129	0	5.360226
NBPF20	0.934229	0	0	0	0	0.198431	1.035338	0.372309
NBPF21P	0	0	0	0	0	0	0	0
NBPF22P	0	0	0	0	0	0	0	0
NBPF25P	0	0	0	0	0	0	0	0
NBPF26	0	1.481109	2.85069	1.607314	0	0	0	0
NBPF2P	0	7.982203	0	1.963628	0	1.26946	0	2.551094

NBPF3	37.082715	40.352873	0	43.770091	27.231074	68.590541	5.822406	56.413496
NBPF4	0	0	0	0	0	0	0	0
NBPF5P	0	0	0	0	0	0	0	0
NBPF6	0	0	0	1.040377	0	0	0	0
NBPF7P	0	0	0	0	0	0	0	0
NBPF8	0	11.180986	11.164714	11.588122	2.626248	7.183908	0	7.433843
NBPF9	5.382561	22.043383	2.534172	27.837127	7.429098	30.5039	0	24.472261
NBR1	65.686277	161.092042	102.756234	215.631394	59.233603	152.420189	50.987886	136.064058
NCALD	0	0.204652	0	0.042451	0	0.136248	0	0.115417
NCAM1	0	0.160431	0	1.177876	0	1.162279	0	0.632172
NCAM2	0.698042	0.628878	0.62692	2.28888	0	0	0	0.016075
NCAN	0	0	0	0.140344	0	0	0	0.05832
NCAPD2	157.501813	267.297439	138.957836	300.853763	113.539642	214.691019	279.722823	171.035144
NCAPD2P1	0	0	0	0	0	0	0	0
NCAPD3	0	1.678821	0	1.332305	0	2.131949	0	0
NCAPG	19.32651	22.201457	13.498811	8.341605	28.899496	20.343768	94.94947	33.365207
NCAPG2	10.865776	32.060825	22.814681	14.858452	31.371132	26.305206	0	18.950514
NCAPGP1	0	0	0	0	0	0	0	0
NCAPGP2	0	0	0	0	0	0	0	0
NCAPH	57.694818	56.352145	78.261605	49.534096	101.33137	79.045172	53.308269	62.304619
NCAPH2	26.59816	43.310224	28.399645	41.640912	89.305793	59.469453	33.535052	45.260296
NCBP1	38.74743	58.315311	70.573261	55.657364	59.277793	30.955816	134.362917	62.479689
NCBP2	132.174551	280.255775	146.34831	229.233521	103.884599	187.682535	107.910169	234.81262
NCBP2AS2	78.060488	97.801721	32.275922	88.218435	61.410597	72.234496	59.085537	63.742974
NCBP2L	0	0	0	0	0	0	0	0
NCBP3	15.841696	27.80081	15.501603	20.942836	21.670367	47.459225	132.112126	21.322484
NCCRP1	1.759481	0.44664	0	0.854476	0	0.348103	0	0
NCDN	4.850031	17.793351	13.047425	16.117419	15.273391	31.566147	29.953844	26.905543
NCEH1	31.909808	15.441056	20.21791	7.964242	16.312056	4.49492	28.826619	13.906603
NCF1	0	0	0	0.047175	0	0	0	0
NCF1B	0	0.2979	0	0.090921	0	0.170707	0	0.287276
NCF1C	0	0	0	0.061336	0	0.190361	0	0
NCF2	12.706856	25.819189	18.928699	17.844411	34.568434	57.566951	6.927626	42.251401

NCF4	0	0.732178	0	0.141255	0	0	0	0.475516
NCK1	52.498682	190.029139	37.123115	195.5428	48.761587	130.367336	147.407212	199.581954
NCK2	13.966849	31.041227	12.503132	34.725649	60.937209	91.185986	20.966979	93.770839
NCKAP1	101.086347	102.734133	119.184809	39.717859	102.593182	34.315385	146.736823	126.574555
NCKAP1L	0	0	0	0	0	0	0	0
NCKAP1P1	0	0	0	0	0	0	0	0
NCKAP5	0	0	0	0.343158	0	0	0	0.706981
NCKAP5L	4.695976	11.738617	7.538659	15.701391	6.379529	24.897339	8.521767	13.941207
NCKIPSD	27.553578	21.185848	7.209732	15.007896	12.093762	26.326965	18.137814	19.634122
NCL	394.415053	299.277753	394.889255	248.372819	596.903964	457.876602	646.174701	435.838006
NCLN	35.256049	27.311915	40.084367	26.418642	22.850759	33.777256	35.760195	27.125182
NCLP1	0	0	0	0	0	0.175202	0	0
NCLP2	0	0	0	0	0	0	0	0
NCMAP	0	0.533342	0.745092	0.344565	0	0.124471	0	0.100029
NCOA1	5.676482	8.446067	2.512742	9.830771	6.393059	18.234554	34.820596	18.099583
NCOA2	8.026623	21.279545	5.502308	20.623499	125.069171	38.022221	21.659779	31.152407
NCOA3	28.069338	41.754417	32.259052	45.352046	27.418822	31.673818	29.375823	43.483963
NCOA4	0	43.718734	0	78.934783	32.394993	48.907121	0	47.17909
NCOA4P1	0	0	0	0	0	0	0	0
NCOA4P2	0	0	0	0	0	0	0	0
NCOA4P3	0	0	0	0	0	0	0	0
NCOA4P4	0	0	0	0	0	0	0	0
NCOA5	16.586964	50.773467	29.730196	54.348188	37.765053	58.727381	35.566801	80.974921
NCOA5LP	0	0	0	0	0	0	0	0
NCOA6	34.72004	72.213785	39.853369	74.117301	21.971944	51.461389	33.421027	34.765676
NCOA7	33.837971	43.786269	83.005627	67.76211	60.317642	57.771221	66.407794	59.02625
NCOR1	46.602673	63.915538	57.962949	94.559464	39.408433	138.786136	89.055389	118.33269
NCOR1P1	0	0	0	0	0	0	0	2.265891
NCOR1P2	0	0	0	0	0	0	0	0
NCOR1P3	0	0	0	0	0	0	0	0
NCOR1P4	0	0	0	0	0	0	0	0
NCOR2	69.677385	47.120857	72.422389	61.52738	112.49182	165.044962	129.716492	92.569141
NCR1	0	0	0	0	0	0	0	0

NCR2	0	0	0	0	0	0	0	0
NCR3	0	0	0	0	0	0	0	0
NCR3LG1	0	2.188558	0	1.473606	0	1.711711	0	1.539391
NCS1	36.073517	105.952715	36.958847	102.996703	33.012106	127.902037	14.646618	96.326759
NCSTN	28.157979	21.862416	43.015033	22.775297	41.930081	14.259803	34.484008	16.009036
NCSTNP1	0	0	0	0	0	0	0	0
NDC1	94.52065	67.604908	88.32353	38.628615	25.156305	11.598964	33.556108	32.170468
NDC80	30.831329	24.578836	38.464709	11.534291	32.091734	22.325565	45.702927	42.844005
NDE1	30.576911	59.694104	26.73866	59.311862	146.474732	105.117183	205.265824	95.277029
NDE1P2	0	0	0	0	0	0	0	0
NDEL1	53.557826	62.690933	0	123.310584	19.354992	96.852786	29.25682	89.069839
NDFIP1	43.694282	39.208929	43.359938	26.961547	19.882084	20.220019	23.062161	31.714243
NDFIP1P1	0	0	0	0	0	0.421638	0	0
NDFIP2	85.295825	30.945259	58.932328	2.103909	31.388037	0	23.627139	21.136797
NDNF	0	0	0	0	0	0	0	0
NDOR1	5.804482	3.776326	0	4.711152	0	10.772497	13.266159	8.589399
NDP	0	0.605647	0	0.149289	0	0	0	0
NDRG1	103.266625	107.345789	147.663222	399.507667	203.849697	886.516938	124.059453	440.589195
NDRG2	0	10.133561	7.024401	16.444839	4.14124	35.019005	71.152249	24.61949
NDRG3	41.066951	38.899167	38.496791	35.337253	41.659294	10.98249	21.426636	19.474166
NDRG4	11.472437	7.509479	40.844682	13.113722	71.887234	20.816065	61.791462	14.391689
NDST1	63.941948	145.581734	74.073736	124.201405	82.04152	242.183782	54.711737	200.28134
NDST2	0	15.470523	6.197542	2.708548	37.29534	4.750099	4.345619	4.051101
NDST3	0	0	0	0	0	0	0	0
NDST4	0	0	0	0	0	0	0	0
NDUFA1	484.71173	356.927118	463.164946	392.010973	414.582678	285.213298	589.450233	280.859366
NDUFA10	14.295307	28.744766	65.436293	40.681129	43.267898	40.698532	0	47.522499
NDUFA11	3.586433	10.053561	0	8.867228	0	10.514861	0	6.71973
NDUFA12	277.752761	199.264582	200.548678	169.203861	293.735952	218.599111	73.625312	317.444899
NDUFA12P1	0	0	0	0	0	0	0	0
NDUFA13	288.808172	134.698819	247.469274	216.854109	254.105813	244.84184	186.251824	186.263122
NDUFA2	0	18.843765	0	13.502579	101.728648	19.978337	0	6.312429
NDUFA3	135.171824	208.981031	63.519723	295.940521	191.786201	319.113225	236.956751	267.437702

NDUFA3P1	0	0	0	0	0	0	0	0
NDUFA3P2	0	0	0	0	0	0	0	0
NDUFA3P3	0	0	0	0	0	0	0	0
NDUFA3P4	0	0	0	0	0	0	0	0
NDUFA3P6	0	0	0	0	0	0	0	0
NDUFA4	115.767307	136.40506	135.550498	55.927524	201.787706	40.011945	148.595051	41.844061
NDUFA4L2	0	0	0	0.188182	0	0	0	0
NDUFA4P1	0	1.220651	0	0	0	0	0	0
NDUFA4P2	0	0	0	0	0	0	0	0
NDUFA5	24.030967	15.118213	36.323838	19.022222	48.076347	6.005401	0	18.040689
NDUFA5P1	0	0	0	0	0	0	0	0
NDUFA5P10	0	0	0	0	0	0	0	0
NDUFA5P11	0	0	0	0	0	0	0	0
NDUFA5P12	0	0	0	0	0	0	0	0
NDUFA5P2	0	0	0	0	0	0	0	0
NDUFA5P3	0	0	0	0	0	0	0	0
NDUFA5P4	0	0	0	0	0	0	0	0
NDUFA5P5	0	0	0	0	0	0	0	0
NDUFA5P6	0	0	0	0	0	0	0	0
NDUFA5P7	0	0	0	0	0	0	0	0
NDUFA5P8	0	0	0	0	0	0	0	0
NDUFA5P9	0	0	0	0	0	0	0	0
NDUFA6	49.226901	130.103932	27.973088	103.053672	77.338905	57.239798	157.38616	93.066489
NDUFA7	0	13.904696	43.965527	7.165916	20.780851	20.452796	87.84117	13.746343
NDUFA8	91.444487	205.825791	106.827178	190.992494	98.488583	166.272476	127.973079	165.441727
NDUFA8P1	0	0	0	0	0	0	0	0
NDUFA9	196.521099	101.061037	126.558905	77.964254	50.199702	52.735535	85.505431	90.139308
NDUFA9P1	0	0	0	0	0	0.483294	0	0
NDUFAB1	150.527025	94.376304	176.357567	66.423383	82.073907	50.885157	234.566388	100.601157
NDUFAB1P1	0	0.855361	0	0	0	0	0	0
NDUFAF1	0	45.535975	10.577902	23.72966	24.046815	39.005665	0.869851	61.742031
NDUFAF2	140.784502	238.618031	112.068834	179.537379	112.102752	155.471209	119.907669	177.372799
NDUFAF2P1	0	1.488667	0	0	0	0.459877	0	0

NDUFAF2P2	0	0	0	0	19.988262	0	0	0
NDUFAF3	41.598887	46.136109	46.29044	48.083433	34.76934	40.372387	18.574937	26.539993
NDUFAF4	46.323503	22.891728	48.278349	21.620812	28.147116	26.391231	5.845062	35.955282
NDUFAF4P1	0	0	0	0.218319	0	0.428446	0	0
NDUFAF4P2	0	0	0	0	0	0	0	0
NDUFAF4P3	0	0	0	0	0	0	0	0
NDUFAF4P4	0	0	0	0	0	0	0	0
NDUFAF5	24.762106	32.333944	34.54045	24.663337	5.292121	23.363613	0	45.896681
NDUFAF6	33.401998	23.129409	21.36601	15.295247	2.17013	15.951024	0	30.881577
NDUFAF7	6.302611	18.719908	12.672745	25.367445	4.015862	9.607555	48.685741	17.786468
NDUFAF8	66.009073	306.194598	135.411116	207.874358	178.659061	181.396248	86.848913	256.554331
NDUFB1	0	13.316689	0	18.455368	14.228753	22.369825	0	27.688185
NDUFB10	168.12985	362.436939	120.663689	335.927948	231.253914	559.047151	432.430105	504.094304
NDUFB10P1	0	0	0	0	0	0	0	0
NDUFB10P2	0	0	0	0	0	0	0	0
NDUFB11P1	0	0	0	0	0	0	0	0
NDUFB1P1	0	0	0	0	0	0	0	0
NDUFB1P2	0	0	0	0	0	0	0	0
NDUFB2	144.596653	249.942022	85.053263	150.487364	173.222515	239.300711	197.484623	280.578152
NDUFB2P1	0	0	0	0	0	0	0	0
NDUFB3	276.231401	83.8182	77.900841	66.431076	233.947594	58.008203	0	102.417864
NDUFB3P5	0	0	0	0	0	0	0	0
NDUFB4	235.917628	200.890552	385.514441	219.220002	350.428457	176.515931	123.840369	203.660833
NDUFB4P1	0	0	0	0	0	0	0	0
NDUFB4P10	0	0	0	0	0	0	0	0
NDUFB4P11	0	0	0	0	0	0	0	0
NDUFB4P12	0	0	0	0	0	0	0	0
NDUFB4P2	0	0	0	0	0	0	0	0
NDUFB4P3	0	0	0	0	0	0	0	0
NDUFB4P4	0	0	0	0	0	0	0	0
NDUFB4P5	0	0	0	0	0	0	0	0
NDUFB4P6	0	0	0	0	0	0	0	0
NDUFB4P7	0	0	0	0	0	0	0	0

NDUFB4P8	0	0	0	0	0	0	0	0
NDUFB4P9	0	0	0	0	0	0	0	0
NDUFB5	181.365491	61.382033	95.383505	44.635862	139.866039	37.793228	137.382752	54.516086
NDUFB5P1	0	0	0	0	0	0	0	0
NDUFB5P2	0	0	0	0	0	0	0	0
NDUFB6	34.87632	49.117001	34.816166	23.906535	42.361361	56.46877	12.249642	48.166421
NDUFB7	80.5372	103.306109	46.095842	81.616221	148.931568	227.56052	101.695333	187.818969
NDUFB8	368.461921	306.789791	194.670034	353.49867	208.384469	321.933835	251.189638	256.277273
NDUFB8P1	0	0	0	0	0	0	0	0
NDUFB8P2	0	0	0	0	0	0	0	0
NDUFB9	167.690723	453.121108	202.330542	393.725637	357.29455	611.082196	428.885192	646.176504
NDUFB9P1	0	0	0	0	0	0	0	0
NDUFB9P2	0	0	0	0	0	0	0	0
NDUFB9P3	0	0	0	0	0	0	0	0
NDUFC1	60.152276	66.379163	48.342965	42.008606	66.282812	34.826656	1.146237	43.791664
NDUFC2	387.088747	238.623274	349.484958	181.105755	64.78978	139.554454	251.490711	129.633808
NDUFC2-KCT	51.206943	2.724402	0	8.502977	47.243234	2.703604	0	10.754387
NDUFS1	99.667737	68.342124	93.534334	59.12434	69.634941	62.533576	152.519975	73.095486
NDUFS2	71.234007	29.201008	71.188162	36.691634	74.677357	31.914004	45.101139	22.763942
NDUFS3	13.310432	36.380912	70.074799	49.809818	59.353443	70.174624	158.039709	75.523897
NDUFS4	190.736055	77.575532	65.639845	79.003269	105.776456	50.2955	107.169728	65.374317
NDUFS5	338.054729	354.900844	358.334868	351.857248	523.838426	418.691192	390.886511	401.246205
NDUFS5P1	0	0	0	0	0	0	0	0
NDUFS5P2	0	0	0	0	0	0	0	0
NDUFS5P3	0	0	0	0	0	0	0	0
NDUFS5P4	0	0	0	0	0	0	0	0
NDUFS5P5	0	0	0	0	0	0	0	0
NDUFS5P6	0	0	0	0	0	0	0	0
NDUFS6	0	36.293617	0	8.006984	0	29.392404	0	0
NDUFS6P1	0	0	0	0	0	0	0	0
NDUFS7	0	9.741125	7.689343	10.015616	8.814777	15.696975	75.822552	19.688215
NDUFS8	164.32739	313.978076	154.154479	302.371481	82.540712	275.027321	149.721007	197.938262
NDUFV1	96.826298	110.531886	165.272417	199.642483	98.078817	88.018028	146.853404	66.209354

NDUFV2	149.77388	123.477907	90.423038	108.798146	181.621425	77.489995	114.707148	134.493001
NDUFV2P1	0	1.290279	0	0.568226	0	0.910432	0	1.141549
NDUFV3	27.81687	53.317977	44.644788	46.877542	35.855402	53.004728	88.342419	53.979536
NEB	0	0.029346	0	0	0	0	0	0
NEBL	0	0.714198	0	0.501195	0	0.500326	0	0.774527
NECAB1	0	0	0	0	0	0	0	0
NECAB2	0	0	0	2.314225	0	0	0	0
NECAB3	1.725764	15.633195	7.999531	16.785355	18.756046	18.459573	56.984014	9.37701
NECAP1	22.030964	19.441651	16.970049	24.655562	12.150755	9.284128	8.061353	16.684654
NECAP1P1	0	0	0	0	0	0	0	0
NECAP1P2	0	0	0	0	0	0	0	0
NECAP2	40.750128	36.965347	24.54825	39.214961	41.458854	57.95726	59.493083	50.470946
NECTIN1	441.851678	1013.43616	713.639632	1497.34543	79.13573	372.63487	77.722615	240.741583
NECTIN2	10.060443	35.92748	15.056141	43.384415	46.120959	65.841947	67.555071	46.567596
NECTIN2P1	0	0	0	0	0	0	0	0.569026
NECTIN3	14.855623	22.582361	21.546443	11.24467	0	1.167096	0	1.297798
NECTIN4	1.9252	6.565289	8.284904	7.045091	7.62414	14.656299	0	9.174609
NEDD1	75.433028	97.891785	69.100113	49.180258	70.97107	78.98612	139.345484	242.280163
NEDD4	55.097581	67.855367	31.973188	36.580289	18.580882	31.865964	14.822316	72.483445
NEDD4L	48.063406	169.900461	42.598313	181.850549	143.036293	358.864254	365.067496	220.354632
NEDD8	354.822437	505.576961	327.695026	531.975434	317.570546	719.509276	321.579441	526.49283
NEDD8-MDF	9.064205	3.340013	19.492908	3.357765	0	10.745224	0	1.62397
NEDD8P1	0	0	0	0	0	0	0	0
NEDD9	12.350858	22.072951	16.309786	34.804861	3.626128	20.669501	10.790675	17.541687
NEFHP1	0	0	0	0	0	0	0	0
NEFHP2	0	0	0	0	0	0	0	0
NEFL	0	0	0	3.330847	0	5.709319	0	0
NEFLP1	0	0	0	0	0	0	0	0
NEFM	20.296064	13.807788	6.038233	6.134377	2.512845	1.072923	0	1.086937
NEFMP1	0	0	0	0	0	0	0	0
NEGR1	0.584407	0.13132	0.525202	0.554529	1.520012	1.343225	0	2.346391
NEIL1	4.172524	1.457953	4.574544	2.983638	0	2.778417	0	3.904601
NEIL2	5.160339	13.908085	3.071848	8.897589	2.210947	6.017796	0	6.582515

NEIL3	8.399532	7.126809	9.053665	3.016298	3.433349	1.866299	28.388312	6.117921
NEK1	4.610289	7.321544	0	6.828686	4.31076	5.602168	0.311613	10.634079
NEK10	1.284199	0.332194	0	1.08914	0	0.638257	0	1.172754
NEK11	16.693662	42.070337	14.062837	33.891793	18.943589	15.614531	31.239205	22.897539
NEK2	49.93623	66.684594	35.008572	44.778701	48.664632	34.634823	11.369219	44.635516
NEK2P1	0	0	0	0	0	0	0	0
NEK2P2	0	0	0	0	0	0	0	0
NEK2P4	0	0	0	0	0	0	0	0
NEK3	0	17.671476	5.007773	2.295162	1.500865	0.321202	0	0.457567
NEK4	28.876091	51.534462	22.37149	43.160169	49.576336	41.807585	27.176123	37.919153
NEK4P1	0	0	0	0	0	0	0	0
NEK4P2	0	0	0	0	0	0	0	0
NEK4P3	0	0	0	0	0	0	0	0
NEK5	0	0.035009	0	0.227653	0	0	0	0.047431
NEK6	52.505925	53.117065	38.05158	60.557501	0	62.387438	9.746305	51.721757
NEK7	21.169873	29.562991	37.217622	15.394194	37.592607	40.361879	109.134698	87.952064
NEK8	0.92994	11.260287	1.668179	6.536088	43.48827	9.888488	12.914325	12.381025
NEK9	36.601474	48.800832	42.849371	56.644064	43.241342	55.711054	58.019416	49.328078
NELFA	14.545154	12.55964	36.292009	19.205211	28.006505	20.471731	0	12.968392
NELFCD	1.064516	4.165281	5.1466	4.633674	58.399053	27.146121	26.259778	24.298683
NELFE	69.908011	120.472724	70.900616	133.120193	76.890301	214.083863	87.293352	105.654429
NELL1	0	0	0	0.069197	0	0	0	0
NELL2	13.747066	0	2.046573	5.347716	0	0	0	0
NEMF	53.242894	34.87115	38.62227	25.766846	31.706359	18.532626	80.177783	36.246644
NEMP1	18.23195	19.079733	21.079231	11.099365	33.647348	2.814746	24.584061	5.147642
NEMP2	23.008478	13.986655	6.624452	9.161407	28.158583	6.8161	14.626262	14.393389
NENF	64.553313	101.155933	76.974768	103.444203	50.810154	121.251448	55.463017	84.486591
NENFP1	0	0	0	0	0	0	0	0
NENFP2	0	0	0	0	0	0	0	0
NENFP3	0	0	0	0	0	0	0	0
NEO1	16.742102	37.798872	49.241209	33.429828	21.049718	3.03638	15.600597	2.705135
NEPNP	0	0	0	0	0	0	0	0
NEPRO	55.012289	79.254094	32.774019	52.196731	45.91767	37.906875	40.448222	72.369295

NES	1.756151	1.911466	3.156462	2.819291	0.463969	1.028131	0	0.493074
NET1	86.258805	259.258567	93.441559	407.227205	82.762336	112.433905	111.784375	131.917953
NETO1	23.067042	51.194343	20.501968	46.049061	39.363434	37.442269	50.823785	33.724619
NETO2	117.874961	193.951034	82.796117	102.411931	67.846839	95.682676	83.387102	184.715767
NEU1	13.546514	6.402367	13.610016	9.205508	19.326533	15.25186	0	14.748549
NEU2	0	0	0	0	0	0	0	0.400873
NEU3	12.617853	28.369138	11.835009	24.392105	9.452168	38.061126	8.824871	21.803536
NEU4	0	0	0	0	0	0	0	0
NEURL1	21.262528	0	0	0.980437	1.849857	3.96256	0	3.988004
NEURL1B	0	0	0	0.292921	0	0	0	0.767346
NEURL2	0	2.690607	0	0	0	1.394048	0	0
NEURL3	0	0.103675	2.199064	4.012771	0	1.1197	0	1.025259
NEURL4	5.371664	8.185068	7.820468	7.507451	26.831011	26.398007	1.410534	13.501348
NEUROD2	0	0.856026	0	0.404849	0	0	0	0
NEUROD4	0	0	0	0	0	0	0	0
NEUROD6	0	0	0	0	0	0	0	0
NEUROG1	0	0	0	0.050943	0	0	0	0
NEUROG2	0	0.253512	0	0.871306	0	0.074054	0	0
NEXMIF	0	0.102797	0	0	0	0	0	0
NEXN	23.370894	7.566281	0	4.533393	14.59488	2.465383	0	2.503516
NF1	45.012262	63.1492	20.86364	55.906249	60.369477	35.510086	189.280418	38.140126
NF1P1	0	0	0	0	0	0	0	0
NF1P10	0	0	0	0	0	0	0	0
NF1P11	0	0	0	0	0	0	0	0
NF1P12	0	0	0	0	0	0	0	0
NF1P2	0	0	0	0	0	0	0	0
NF1P3	0	0	0	0	0	0	0	0
NF1P4	0	0	0	0	0	0	0	0
NF1P5	0	0	0	0	0	0	0	0
NF1P6	0	0	0	0	0	0	0	0
NF1P7	0	0	0	0	0	0	0	0
NF1P8	0	0	0	0	0	0	0	0
NF1P9	0	0	0	0	0	0	0	0

NF2	39.553773	40.277743	23.857569	35.965464	34.740765	84.829852	132.22944	66.416093
NFAM1	0	1.082367	0	0	0	0	0	0
NFASC	19.867803	5.708038	7.825701	10.837247	6.258281	7.192058	1.871075	5.877004
NFAT5	27.533654	28.287207	29.618934	26.591887	60.317574	37.280142	44.171079	51.55047
NFATC1	1.642146	13.0943	14.775871	6.803107	3.410187	9.37474	0	4.646969
NFATC2	1.738186	1.868456	2.572296	2.322935	5.056509	2.87843	3.709292	2.380919
NFATC2IP	0	2.562487	5.196249	0.54293	58.131723	10.22564	1.877162	4.51498
NFATC3	26.720479	36.717826	16.141887	28.826541	21.008126	31.583773	43.478938	58.970482
NFATC4	7.490338	8.341444	14.077977	12.293437	9.029332	11.07654	12.671726	4.036158
NFE2	2.353242	0.89586	0	0.777991	0	0.231271	0	0.259659
NFE2L1	61.686941	435.59617	186.024589	981.323265	145.421586	1009.37777	385.646423	627.346614
NFE2L2	69.490868	97.804905	67.405061	67.804605	105.791938	78.364669	83.582884	133.141752
NFE2L3	26.588165	53.991976	82.193777	49.489585	18.253525	17.170658	15.468884	12.461154
NFE2L3P1	0	0	0	0	0	0	0	0
NFIA	7.732017	15.540897	9.621799	27.022493	33.697979	44.037168	36.636294	37.42898
NFIB	17.548258	44.808148	13.154686	49.703825	28.874577	31.618009	2.590351	40.83937
NFIC	22.498363	49.967099	32.355829	66.459037	47.172648	91.667483	44.147163	52.996951
NFIL3	13.474127	54.280337	16.546423	43.764135	7.993316	30.848632	0	46.075927
NFIX	11.823489	13.877055	13.779338	15.996989	75.02162	126.152001	189.961676	105.771004
NFKB1	33.278655	130.992075	27.433179	119.594376	56.592901	126.276937	90.538546	111.95065
NFKB2	31.966667	36.455824	38.92387	73.61916	86.980082	54.486402	66.380661	39.424355
NFKBIA	0	7.620626	0	2.665463	15.003296	59.72559	21.937201	1.232957
NFKBIB	16.770576	10.461896	14.37492	18.177151	57.180692	37.7425	13.111044	21.605539
NFKBID	2.33927	4.140479	0	0.628446	4.279859	0.904747	0	0.853673
NFKBIE	15.432175	7.323681	11.593307	12.581971	15.772189	0	0	11.24705
NFKBIL1	8.494662	11.496767	13.803156	20.34022	12.843375	33.445719	13.724499	19.569298
NFKBIZ	24.186499	49.046105	50.542321	68.686651	41.361937	45.14058	104.1594	37.17121
NFRKB	9.818316	16.912872	6.921488	19.218661	25.132795	57.574956	20.076704	53.772944
NFS1	65.323366	65.141486	54.287684	70.672503	31.967513	36.087833	78.616634	41.087374
NFU1	0	50.302292	25.524773	11.014295	0	23.007095	90.294682	32.291958
NFU1P1	0	0	0	0	0	0	0	0
NFU1P2	0	0	0	0	0	0	0	0
NFX1	26.418956	35.253459	26.478651	36.281055	46.58206	37.135367	24.856812	33.709963

NFXL1	7.124232	12.454174	11.983797	10.381617	20.497572	5.905467	24.059658	12.004013
NFYA	0	17.95698	0	29.853812	14.823741	47.991181	11.345635	29.988999
NFYAP1	0	0	0	0	0	0	0	0
NFYB	39.348817	96.011181	23.96955	24.844287	42.545834	25.12165	10.102153	89.974381
NFYBP1	0	0	0	0	0	0	0	0
NFYC	2.900288	31.486259	20.952729	36.333695	63.927974	47.553086	1.507066	24.368683
NFYCP1	0	0	0	0	0	0	0	0
NFYCP2	0	0	0	0	0	0	0	0
NGDN	40.916424	48.399471	33.173523	51.423206	44.539951	41.172993	63.790485	54.511035
NGEF	0	8.058571	8.396362	8.256992	4.187248	8.143637	39.202657	6.700965
NGFR	9.77527	18.311898	14.024559	21.892243	5.677578	28.238126	0	11.471979
NGLY1	19.623953	35.092151	20.719139	18.930142	24.204504	14.196984	26.48792	35.173401
NGRN	45.427643	47.895541	54.655675	55.053534	37.353266	39.835858	48.034119	44.844565
NGRNP1	0	0	0	0	0	0	0	0
NGRNP2	0	0	0	0	0	0	0	0
NGRNP3	0	0	0	0	0	0	0	0
NGRNP4	0	0	0	0	0	0.348654	0	0
NHEJ1	0	0	0	0	32.061219	6.169842	0	0
NHERF1	90.503378	52.595085	60.654957	59.924663	48.679492	90.558142	111.161429	51.169403
NHERF2	20.550128	57.26906	22.985711	66.560363	14.850103	119.031675	0	74.257816
NHERF4	0	0	0	0	0	0	0	0
NHLH1	0	0	0	0.096983	0	0	0	0
NHLH2	0	0.975879	0	0.970551	0	0.134513	0	0
NHLRC2	22.530103	13.845714	13.05455	11.704518	8.970217	12.133496	7.362532	15.673725
NHLRC3	5.112946	4.096439	7.312219	5.60043	1.349112	2.680068	6.150561	3.903496
NHLRC4	0	0.282945	0	0.162286	0	0.744128	0	0.202074
NHP2	0	0	0	46.40579	0	61.191362	0	31.204181
NHP2P1	0	0	0	0	0	0	0	0
NHP2P2	0	0	0	0	0	0	0	0
NHS	1.975048	4.013006	3.90913	8.707477	9.129769	24.009384	6.552399	13.433449
NHSL1	4.012394	20.46828	6.583202	30.2922	7.323144	17.30881	0	17.953546
NHSL2	1.137309	0.076726	0	0.101349	0.805754	0.247727	1.178178	1.476843
NHSL3	48.956605	54.585057	54.991211	59.048519	68.140516	142.261064	57.559548	93.186463

NIBAN1	62.531236	69.235523	83.902715	80.73619	37.416508	44.213021	34.295292	29.208794
NIBAN2	82.30943	147.423818	64.532582	154.496739	126.385793	291.748769	137.986429	174.323675
NIBAN3	0	0	0	0	0	0	0	0
NICN1	0	0	0	0	6.862775	0.971608	0	0
NICN2P	0	0	0	0	0	0	0	0
NICOL1	0	0	0	0.906383	0	1.253398	0	1.121696
NID1	28.100168	13.769854	42.294957	20.917177	58.213533	17.489453	17.120752	9.698722
NID2	30.237298	10.712969	52.552391	12.831047	49.60137	22.632518	27.847756	11.824795
NIF3L1	21.879694	26.879053	16.901445	34.994136	14.673159	37.759321	59.541162	47.253413
NIFK	107.959813	74.590858	82.999325	72.156114	209.467762	63.600983	104.095397	95.941241
NIFKP1	0	0	0	0	0	0	0	0
NIFKP2	0	0	0	0	0	0	0	0
NIFKP3	0	0	0	0	0	0	0	0
NIFKP4	0	0	0	0	0	0	0	0
NIFKP6	0	0	0	0	0	0	0	0
NIFKP7	0	0	0	0	0	0	0	0
NIFKP8	0	0	0	0	0	0	0	0
NIFKP9	0	0	0	0	0	0	0	0
NIM1K	0	0.73444	0	1.307173	0	0.440631	0	0.812677
NIN	35.506164	25.679146	13.298819	43.682977	74.151067	127.040741	86.888873	99.205691
NINJ1	42.19471	27.580112	15.916005	36.200439	3.148026	47.656652	8.63214	30.863827
NINJ2	0	0	0	0	0	0	0	0
NINL	9.425264	7.182146	3.143087	6.438397	3.945998	12.62334	0	6.608161
NIP7	58.949307	84.901603	84.308582	84.333923	92.583905	80.578859	99.116723	108.791145
NIP7P1	0	0	0	0	0	0	0	0
NIP7P2	0	0	0	0	0	0	0	0
NIP7P3	0	0	0	0	0	0	0	0
NIPA1	12.866273	23.318086	23.71579	26.259247	23.220524	20.574771	18.430553	24.576555
NIPA2	49.989442	97.377183	15.028967	71.068615	27.149985	83.415055	65.681313	144.755706
NIPA2P1	0	0	0	0	0	0	0	0
NIPA2P2	0	0	0	0	0	0	0	0
NIPA2P3	0	0	0	0	0	0	0	0
NIPA2P4	0	0	0	0	0	0	0	0

NIPA2P5	0	0	0	0	0	0	0	0
NIPAL1	6.158056	33.02555	6.086532	23.392544	13.958193	14.045158	3.45075	23.753643
NIPAL1P1	0	0	0	0	0	0	0	0
NIPAL2	28.694252	2.744973	8.721323	1.803654	0.394691	2.35363	0	1.049811
NIPAL3	23.703767	48.841573	28.875971	52.243879	46.499819	79.007071	12.526655	84.500155
NIPAL4	0	0	0	0.087578	0	0	0	0
NIPBL	32.045798	60.444895	30.877498	69.506032	41.056674	38.21614	48.007703	45.351734
NIPSNAP1	0	0	0	1.430127	40.993939	0.541954	0	0
NIPSNAP2	60.969231	57.180159	105.498935	50.788239	287.377678	40.206814	237.379854	85.776385
NIPSNAP3A	10.850776	4.657593	1.932856	4.573708	5.71669	4.487847	0	7.459445
NIPSNAP3B	0	0.255128	0	0.545836	0.155261	0	0	0.414949
NISCH	5.590643	8.95747	3.153205	8.574512	6.567823	16.426626	10.812133	12.07591
NIT1	17.408708	30.977897	16.424982	47.184064	51.497364	26.463377	0.884472	41.538528
NIT2	108.233431	96.381759	103.635425	70.385426	96.586729	53.466224	100.519449	88.39542
NKAIN1	0	0.392687	0	0.944492	0	0.114631	0	0
NKAIN1P1	0	0	0	0	0	0	0	0
NKAIN1P2	0	0	0	0	0	0	0	0
NKAIN2	0	0	0	0	0	0	0	0
NKAIN3	0	0	0	0	0	0	0	0
NKAIN4	0	1.675992	4.529156	1.367043	0	0	0	0.969725
NKAP	42.141076	58.21889	39.53448	52.614	21.995182	44.93016	15.114107	47.697827
NKAPD1	57.962616	46.413747	51.425049	86.128026	27.117006	85.628043	118.506837	88.5259
NKAPP1	0	0	0	1.259648	0	2.655163	0	0
NKD1	0	0.072692	0	0.009164	0.049441	0.160014	0	0.074852
NKD2	1.794543	3.474362	0	3.3068	0	3.107821	0	0
NKG7	0	0	0	0	0	0	0	0
NKIRAS1	15.842048	20.044843	0	6.378822	12.265801	2.837276	0	9.814934
NKIRAS2	20.83328	27.815904	38.825032	44.191272	34.656824	43.664857	26.225534	30.371332
NKPD1	0	0	0	0	0	0	0	0
NKRF	0	0	0	0	0	0	0	0
NKTR	11.203627	12.567686	14.011434	6.929234	48.293777	12.595707	17.983152	19.125863
NKX1-2	38.522064	31.421811	27.997078	28.033696	25.559438	16.869887	1.518306	15.654823
NKX2-1	0	3.029845	0	1.710273	0	0	0	0

NKX2-3	0	0	0	0	0	0	0	0
NKX2-4	0	0	0	0	0	0	0	0
NKX2-5	0	1.122604	0	1.031877	0	0.338295	0	0.545484
NKX3-1	3.055783	12.003018	6.392025	9.132089	4.033398	6.448233	0.247603	5.573525
NKX6-1	6.271261	0	0	0.122824	0	0.216759	0	0
NKX6-2	0	0	0	0.059414	0	0	0	0
NKX6-3	0	0	0	0	0	0	0	0
NLE1	27.289159	24.437321	19.750106	26.090482	7.980417	21.866643	8.186956	15.12963
NLGN1	0	4.537955	0	0.231557	0	0	0	0
NLGN2	3.285734	22.911987	12.061096	14.329059	9.894791	23.105889	8.606526	16.220457
NLGN3	0	0	0	0.309463	0	0	0	0
NLGN4X	0	0.097068	0	0.601951	2.423538	2.685833	6.512327	3.20657
NLGN4Y	1.897463	6.632704	17.58098	13.455707	19.747134	6.865352	0.308165	15.114567
NLK	14.53078	8.806666	39.662657	6.083203	8.214727	4.557374	20.064465	10.477149
NLN	67.895066	160.301943	47.230019	106.109824	56.955686	68.351367	64.922971	129.122057
NLRC3	0	0	0	0.118999	0	0.025377	0	0.06086
NLRC4	0	0	0	0.048419	0	0	0	0
NLRC5	16.58865	4.545613	0	11.591903	91.978405	47.250273	86.182041	30.123481
NLRP1	0	0	0	1.569412	0	0.860161	0	0.628185
NLRP10	2.909838	1.566268	2.792236	1.610341	8.009314	7.708132	3.596312	8.741786
NLRP11	0	0	0	0.08002	0	0	0	0
NLRP12	0	0	0	0	0	0	0	0
NLRP13	0	0	0	0	0	0	0	0.037814
NLRP2	0	0	0	0	0	0	0	0
NLRP2B	0	0	0	0	0	0	0	0
NLRP3	0	0	0	0	0	0	0	0
NLRP3P1	1.968237	1.245694	1.755016	1.145649	0.518672	0.582762	0	2.544315
NLRP4	0	0	0	0	0	0	0	0
NLRP5	0	0	0	0	0	0	0	0
NLRP6	0	0	0	0	0	0	0	0
NLRP7	0	0	0	0	0	0.10596	0	0.29088
NLRP7P1	0	0	0	0	0	0	0	0
NLRP8	0	0	0	0	0	0	0	0

NLRP9	0	0	0	0	0	0	0	0
NLRP9P1	0	0	0	0	0	0	0	0
NLRX1	42.850521	54.642142	59.207996	60.952782	0	31.754623	28.123021	20.696979
NMB	20.050021	26.65353	40.634038	18.22864	8.72784	29.066866	33.069253	29.704572
NMBR	0	0	0	0	0	0	0	0
NMD3	64.211605	32.725717	49.742921	46.500996	89.450322	24.764527	94.017463	58.453047
NMD3P1	0	0	0	0	0	0	0	0
NMD3P2	0	0	0	0	0	0	0	0
NME1	50.530165	101.822666	112.129063	64.935571	82.256798	69.825268	0	84.190067
NME1-NME2	0	27.80428	59.511764	3.873737	0	28.771795	0	11.166972
NME1P1	0	0	0	0	0	0	0	0
NME2	434.368313	299.057092	409.356249	211.090977	575.51383	421.533997	751.311349	414.477033
NME2P1	0	0	0	0	0	0	0	0
NME2P2	0	0	0	0	0	0	0	0
NME2P3	0	0	0	0	0	0	0	0
NME3	11.089542	27.887226	15.068453	12.075331	0	19.15222	53.738853	9.400202
NME4	81.175478	72.766061	78.435222	75.885765	85.562789	109.925818	47.699404	81.935591
NME5	0	0.831152	0	0.222448	0	0	0	0
NME6	29.210604	26.423519	14.596456	36.186139	45.213127	36.763761	16.388902	37.108277
NME7	69.282993	52.848848	25.192845	19.97099	16.826989	13.341151	7.89348	40.201846
NME8	0	0	0	0	0	0	0	0
NME9	0	0.24974	0	0.23437	0	0	0	0
NMI	0	1.914444	0	1.588083	0	1.943614	0	0.805201
NMNAT1	5.32662	7.441534	9.657003	8.141078	11.015498	7.619815	0	5.044823
NMNAT1P1	0	0	0	0	0	0	0	0
NMNAT1P2	0	0	0	0	0	0	0	0
NMNAT1P3	0	0	0	0	0	0	0	0
NMNAT1P4	0	0	0	0	0	0	0	0
NMNAT1P5	0	0	0	0	0	0	0	0
NMNAT2	5.395719	4.275125	9.158611	8.156755	0.475164	0.54068	0	0.673362
NMNAT3	5.068431	3.144224	3.348737	4.509894	5.437054	7.616406	0	4.565512
NMRAL1	0	76.456568	52.784045	78.540875	54.43044	82.387808	133.062639	68.378964
NMRAL2P	0	0	0	0	0	1.697034	0	0

NMRK1	23.647818	20.989371	17.375454	20.704802	4.452591	13.81067	0	28.000033
NMRK2	0	0	0	0.294071	0	0	0	0
NMS	0	0	0	0	0	0	0	0
NMT1	36.796078	78.276536	32.681956	72.834386	107.641548	103.923966	156.647107	79.078098
NMT2	11.237709	21.829447	12.782404	12.013029	0	7.081077	7.735456	7.157727
NMTRQ-TTG	0	0	0	0	0	0	0	0
NMTRS-TGA	0	0	0	0	0	0	0	0
NMU	16.518087	2.023577	4.812764	2.210726	0	4.998558	0	5.11238
NMUR1	0	0	0	0	0	0	0	0
NMUR2	0	0	0	0	0	0	0	0
NNAT	0	0	0	0	0	0.187066	0	0
NNMT	0	2.608239	0	1.233266	2.491169	3.82629	0	3.307899
NNT	58.895459	59.413675	33.46667	52.45073	33.230939	35.296484	49.381954	45.652591
NOA1	24.789113	17.023644	24.925618	15.367825	25.3329	10.522846	15.395149	16.143538
NOB1	96.672467	136.425599	183.774662	155.19439	108.387631	195.300362	125.761671	167.559397
NOBOX	0	0	0	0	0	0	0	0
NOC2L	38.960509	39.20922	38.385681	53.419608	116.360816	97.818172	37.47072	72.220444
NOC2LP1	0	0	0	0	0	0.076614	0	0
NOC2LP2	0	0	0	0	0	0	0	0
NOC3L	42.393249	21.079039	26.784334	11.343794	32.381544	7.731703	14.056771	19.559842
NOC4L	6.448309	8.052611	7.658506	9.88004	27.182326	8.786168	27.668316	8.337863
NOCT	8.861715	19.267699	3.164188	20.677027	23.530007	26.025373	31.789393	34.291281
NOD1	7.848833	19.365672	15.591567	20.28489	30.869825	34.516588	19.805132	33.637318
NOD2	0.7326	0.219904	0	0.518952	0	0.146603	0	0.147034
NODAL	0	0	0	0	0	0	0	0
NOL10	32.333422	53.606774	30.703029	37.691334	41.935868	34.506402	27.493324	48.608167
NOL11	77.794894	59.162045	57.615862	56.730037	177.427416	45.071653	68.939419	75.753439
NOL12	17.525286	22.742173	19.269775	23.735077	21.99426	32.109224	0	18.810989
NOL3	15.4341	7.178836	10.816867	19.123915	2.008079	26.433877	9.752362	6.672196
NOL4	0	1.335898	0	0.94717	0	0	0	0
NOL4L	4.493733	11.710981	0	19.440769	7.240516	3.197243	52.20473	3.361748
NOL6	14.802284	21.638021	18.345662	13.470695	25.450583	32.168208	36.882828	24.868033
NOL7	46.246859	116.456462	33.713784	58.620171	54.828102	68.082492	122.332496	121.114263

NOL8	34.749682	21.903142	19.133743	25.82637	34.882354	47.139629	43.561227	49.431864
NOL8P1	0	0	0	0	0	0	0	0
NOL9	31.750203	15.590465	13.635505	7.907931	22.620663	10.062497	25.761382	22.633632
NOLC1	85.699323	137.048133	51.688338	206.609189	152.632373	211.268109	190.265952	123.081465
NOLC1P1	0	0	0	0	0	0	0	0
NOM1	9.084324	6.139391	7.685968	5.880966	3.671006	6.950883	7.815137	7.455626
NOMO1	49.806982	50.050608	74.213846	54.924301	132.573287	31.056742	45.67785	20.298519
NOMO2	0	0	0	3.298086	101.686553	0.336131	0	0.971987
NOMO3	0	9.345828	3.613495	17.079706	25.964452	23.689133	55.861562	7.878714
NONO	352.846528	362.813562	409.233445	553.797438	358.413825	428.427623	606.63791	220.366729
NONOP2	0	1.127503	0	0.145098	0	0.146816	0	0
NOP14	40.533165	48.145546	43.917341	56.362428	55.329403	59.165368	89.732041	41.011122
NOP16	36.74604	42.302819	37.693874	59.943819	18.424607	64.292221	85.259412	39.642768
NOP2	32.4642	39.408291	8.039672	38.385601	14.825978	84.46097	20.996427	44.930332
NOP53	131.3317	88.802408	152.890832	113.440829	130.239866	116.161324	71.029778	59.81933
NOP56	217.336876	215.67491	170.8811	226.998768	225.381146	323.971461	215.723241	242.499956
NOP56P1	0	0	0	0	0	0	0	0
NOP56P2	0	0	0	0	0	0	0	0
NOP56P3	0	0	0	0	0	0	0	0
NOP58	106.201965	162.811385	69.948211	113.740884	116.356878	156.462094	179.860816	318.961499
NOP9	9.141668	9.339936	16.821249	7.87694	15.913288	14.435399	5.782695	9.078308
NOPCHAP1	61.933709	37.493635	30.918849	32.265515	65.350577	31.367241	2.507297	50.616155
NOS1	8.530719	20.508586	7.440377	13.741185	6.131102	8.103436	3.345551	10.207285
NOS1AP	1.304503	4.589084	1.757842	5.339662	1.609078	6.442153	0	5.332961
NOS2	0	0	0	0	0	0	0	0
NOS2P1	0	0	0	0	0	0	0	0
NOS2P2	0	0	0	0	0	0	0	0
NOS2P3	0	0	0	0	0	0	0	0
NOS2P4	0	0	0	0	0	0	0	0
NOS3	0.75362	2.722239	0	1.387021	0	0.63883	0	1.537967
NOSIP	0	4.591905	32.179206	10.596359	0	18.406781	0	5.677454
NOSTRIN	0	0	0	0	0	0	0	0
NOTCH1	5.709794	8.86075	8.161337	9.284074	10.110384	14.211074	0.737637	8.042398

NOTCH2	49.705125	39.87221	25.341677	37.608994	27.466442	61.658296	20.739511	36.231241
NOTCH2NLA	41.946599	9.861608	40.011984	11.28774	8.375536	4.533039	0.604346	11.274321
NOTCH2NLC	6.955473	3.882889	5.493902	2.621368	5.937177	2.417185	2.317638	2.766908
NOTCH2P1	0	0	0	0	0	0	0	0
NOTCH3	0.370974	1.993956	6.008343	3.549073	3.039515	6.092973	1.810155	1.863439
NOTCH4	0	1.007287	0	0.698565	0	0.758935	0	0.440016
NOTUM	0	0.262282	0	0.789069	0	0	0	0
NOVA1	25.973897	19.54065	5.556722	16.014667	0	2.467579	0	4.0467
NOVA2	0	0.205221	0.714413	0.832133	0	1.163269	0	0.127282
NOX1	0	0	0	0	0	0	0	0
NOX4	4.983855	2.704892	0	0.781477	0	0	9.583101	1.127616
NOX4P1	0	0	0	0	0	0	0	0
NOX5	0	0.115612	0	0.319542	0	0.039274	0	0.743526
NOXA1	2.461678	0	2.188902	1.145144	9.073594	0.362461	16.026339	0.199395
NOXO1	0	0	0	0	0	0	0	0
NOXRED1	0	0	0	0	0	0	0	0
NPAP1L	0	0	0	0	0	0	0	0
NPAP1P2	0	0	0	0	0	0	0	0
NPAP1P3	0	0	0	0.108843	0	0	0	0
NPAP1P4	0	0	0	0	0	0	0	0
NPAP1P6	0	0	0	0	0	0	0	0
NPAP1P7	0	0	0	0	0	0	0	0
NPAP1P9	0	0	0	0	0	0	0	0
NPAS1	0	3.452073	2.417959	0.905819	0	0.799669	0	0.523995
NPAS2	3.28635	19.426534	1.474719	13.99517	1.862212	1.19079	0	4.965126
NPAS3	0	0	0	0	0	0	0	0.472853
NPAS4	1.388274	0	0	0.348426	0	0	0	0
NPAT	14.884189	12.876387	22.937719	9.108064	58.873527	7.78296	72.481413	13.98671
NPB	0	0.949462	0	0.984271	0	0.554818	0	0.670707
NPBWR2	0	0	0	0	0	0	0	0
NPC1	21.997669	28.215955	61.196524	25.553481	40.864605	14.369319	7.380227	17.344265
NPC1L1	0	0	0	0	0	0	0	0
NPC2	2.780161	114.486331	94.119521	98.217851	151.125876	118.787086	352.713991	42.007667

NPDC1	26.825285	36.009758	24.680523	45.618082	12.852364	34.733982	0	23.20747
NPEPL1	19.744012	12.936728	5.612865	15.26755	16.713766	10.727331	28.374423	12.377243
NPEPPS	61.412552	63.859101	64.652658	59.698694	29.123755	56.976062	115.535441	25.929585
NPEPPSP1	0	0	0	0.269769	0	0.617251	0	0
NPEPPSP2	0	0	0	0	0	5.247551	0	0
NPFFR2	0	0	0	0	0	0	0	0
NPHP1	0.866298	2.368111	0	6.665957	0.647673	2.898435	1.872602	6.908678
NPHP3	9.312958	9.873535	10.349814	5.131287	36.101675	1.40645	85.186562	5.54498
NPHP3-ACAI	0	0	0	0	0	0	0	0.43174
NPHP4	5.94531	9.51272	4.798945	10.588247	9.982589	14.103551	7.330677	10.42036
NPHS1	0	0	0	0.092737	0	0	0	0
NPHS2	0	0	0	0	0	0	0	0
NPIPA1	1.314111	12.006371	15.311135	12.163997	14.085706	15.406409	70.152497	9.962217
NPIPA3	4.767441	0	0	0.105751	0	0.481598	0	0
NPIPA5	0	0	0	0.52797	0	0	0	0
NPIPA6	23.233573	12.935594	29.153086	18.138505	61.42212	33.695661	83.152124	25.338957
NPIPA7	15.667214	5.935057	9.095547	6.56003	10.49054	15.136217	0	10.13115
NPIPA8	0	11.10701	0	5.631036	0	8.159552	0	2.969271
NPIPA9	0	0.47344	0	0	38.652779	1.335439	6.424898	0.409618
NPIPB10P	0	0	0	0	0	0	0	0.109306
NPIPB11	0	1.489117	19.817162	1.623775	0.252066	1.633006	0	2.898452
NPIPB12	17.837694	6.421451	27.919517	2.50731	0	10.968149	0	5.760191
NPIPB13	0	0	0	0	45.426423	0	0	0
NPIPB14P	0	0.968611	0	2.861312	1.131989	0.637169	0	0
NPIPB15	0	0	0	0.254086	0	0	0	0
NPIPB2	0	0.290518	0	0.893901	0	0	0	0
NPIPB3	25.961261	2.099234	0	9.823828	34.251977	12.113059	21.766552	15.212257
NPIPB4	21.587399	9.936537	13.21367	4.215059	25.651867	1.168014	48.734336	4.408796
NPIPB5	48.211479	25.119642	32.985397	19.210996	23.808555	11.274242	107.710598	20.173852
NPIPB6	0	0	0	0	0	0	0	0
NPIPB7	0	0	0	0	0	0	0	0
NPIPB8	0	0	0	0.378964	0	0	0	0
NPIPB9	0	0	0	0	0	0.322798	0	0

NPIPP1	0	0	0	0	0	0	0	1.184785
NPL	5.613901	8.169094	0	8.163148	0	3.546031	0	5.483284
NPLOC4	63.077826	194.14755	57.308115	161.389166	238.697163	204.42497	53.493197	184.076425
NPLP1	0	0	0	0	0	0	0	0
NPM1	2617.16697	3416.75188	3155.09965	3369.53144	1898.57365	2557.27613	2646.59435	3486.67791
NPM1P1	0	0	0	0	0	0	0	0
NPM1P10	0	0	0	0	0	0	0	0
NPM1P11	0	0	0	0	0	0	0	0
NPM1P12	0	0	0	0	0	0	0	0
NPM1P13	0	0	0	0	0	0	0	0
NPM1P14	0	0	0	0	0	0	0	0
NPM1P17	0	0	0	0	0	0	0	0
NPM1P18	0	0	0	0	0	0	0	0
NPM1P19	0	0	0	0	0	0	0	0
NPM1P2	0	0	0	0	0	0	0	0
NPM1P20	0	0	0	0	0	0	0	0
NPM1P21	0	0	0	0	0	0	0	0
NPM1P22	0	0	0	0	0	0	0	0
NPM1P23	0	0	0	0	0	0	0	0
NPM1P24	0	0	0	0	0	0	0	0
NPM1P25	0	0	0	0	0	0.225217	1.119596	0.19264
NPM1P26	0	0	0	0.134975	0	0.269487	0	0
NPM1P27	0	2.014891	0	0.321818	0	0.695333	0	0.507655
NPM1P28	0	0	0	0	0	0	0	0
NPM1P29	0	0	0	0	0	0	0	0
NPM1P3	0	0	0	0	0	0	0	0
NPM1P30	0	0	0	0	0	0	0	0
NPM1P31	0	0	0	0	0	0	0	0
NPM1P32	0	0	0	0	0	0	0	0
NPM1P33	0	0	0	0	0	0	0	0
NPM1P34	0	0	0	0.106244	0	0	0	0
NPM1P35	0	0	0	0.110039	0	0.220839	0	0
NPM1P36	0	0	0	0	0	0	0	0

NPM1P37	0	0	0	0	0	0.438777	0	0
NPM1P38	0	0	0	0	0	0	0	0
NPM1P39	0	0.596186	0	0.112776	0	0	0	0.190974
NPM1P4	0	0	0	0	0	0	0	0
NPM1P40	0	0	0	0	0	0	0	0
NPM1P41	0	0	0	0	0	0	0	0
NPM1P42	0	0	0	0	0	0	0	0
NPM1P43	0	0	0	0	0	0	0	0
NPM1P45	0	0	0	0	0	0	0	0
NPM1P46	0	0	0	0	0	0	0	0
NPM1P47	0	0	0	0	0	0	0	0
NPM1P48	0	0	0	0	0	0	0	0
NPM1P49	0	0	0	0	0	0	0	0
NPM1P5	0	0.788279	0	0	0	0.230617	0	0
NPM1P50	0	0.376936	0	0	0	0	0	0
NPM1P51	0	0	0	0	0	0	0	0
NPM1P52	0	0	0	0	0	0	0	0
NPM1P6	0	0.190828	0	1.12033	0	0	0	0.378332
NPM1P7	3.88699	1.064158	0	0.386214	0	0	0	0.813472
NPM1P8	0	0	0	0	0	0	0	0
NPM1P9	0	2.141842	0	0.11399	0	1.83054	0	0.978992
NPM2	10.015936	16.102591	7.108823	26.580643	45.122609	58.25913	0	27.220937
NPM3	0	8.707113	0	24.682611	90.77128	10.838461	0	16.050621
NPNT	3.088244	0	0	4.369066	41.188051	4.510034	53.322058	3.218348
NPPA	0	0	0	0	0	0	0	0
NPPB	0	0	0	0	0	0.283921	0	0
NPPC	0	0	0	0	0	0	0	0
NPR1	0	1.788931	0	1.348416	0	0.354451	0	0.08368
NPR2	0	0.236576	0	0.463441	3.510839	0	0	1.374094
NPR3	17.111392	16.783881	17.817114	10.994199	0.134409	0.051024	0	0.425479
NPRL2	4.002954	3.661623	7.349398	25.117515	8.245506	9.78169	0	3.031641
NPRL3	33.026898	36.986702	5.666271	35.748848	31.234283	39.432698	0	42.908044
NPSR1	0	0	0	0	0	0	0	0

NPTN	138.048823	133.36302	109.20337	155.276236	247.676854	145.52659	179.203976	130.147685
NPTX1	7.796783	6.272197	6.467354	4.331322	0	0.661081	0	0.457099
NPTX2	1.24612	3.821439	1.115677	2.558412	0	0.061986	0	0.100304
NPVF	0	0	0	0	0	0	0	0
NPW	0	1.519862	12.067846	3.794887	22.170781	0	0	0
NPY	0	0	0	0	0	0	0	0
NPY1R	0	0.155903	0	0.148877	0	0	0	0.098385
NPY2R	0	0	0	0	0	0	0	0
NPY4R	0	0.123443	0	0.023649	0	0.769961	0	0.708881
NPY4R2	0	0.123443	0	0.023649	0	0	0	0
NPY5R	0	0	0	0	0	0	0	0
NPY6R	0	0	0	0	0	0	0	0
NQO1	398.488456	412.846264	291.349496	341.319554	284.960896	243.297274	124.149638	238.891948
NQO2	0	19.181173	0	20.238074	9.904858	24.294927	14.618467	26.110834
NR0B1	0	0	0	0	0	0	0	0
NR0B2	0	0	0	0	0	0	0	0
NR1D1	6.44199	15.192302	5.766263	17.38574	2.379632	15.184729	2.814417	16.685654
NR1D2	10.012519	10.134721	11.782613	8.290995	7.024072	7.875854	10.114618	10.598654
NR1H2	0	0.95445	0	6.413139	69.406051	5.348747	18.964964	11.171937
NR1H3	6.517609	8.870697	3.694841	12.163152	31.312898	10.284833	19.695794	7.820344
NR1H4	0	0	0	0	0	0	0	0
NR1H5P	0	0	0	0	0	0	0	0
NR1I2	0	0.053158	0	0.646436	0	0.124165	0	0
NR1I3	0	0.210063	0	0.060607	0	0	0	0
NR2C1	17.553032	24.549871	23.203041	32.475042	128.11007	52.435314	5.345576	71.569738
NR2C2	42.741042	46.875189	51.962383	111.60144	69.249306	86.190582	8.955984	53.4169
NR2C2AP	4.008044	20.926206	0	23.576128	55.450328	43.365571	1.050905	14.294352
NR2E1	0	1.614332	0	0	0	0	0	0.156755
NR2E3	0	0	0	0	0	0	0	0
NR2F1	6.85281	16.324377	3.091399	5.444034	0	2.553767	0	4.665778
NR2F2	30.660373	59.751675	31.69182	46.465167	58.865636	109.914485	45.198912	113.424316
NR2F6	0	0	0	0	0	0	0	0
NR3C1	27.896551	79.11367	24.064498	62.685632	58.169225	126.315537	65.207361	122.90004

NR3C2	0	0.096608	0	0.251695	0	0	0	0.045549
NR4A1	13.425114	14.497771	1.525209	17.269282	0	13.724524	41.688085	5.237049
NR4A2	0	3.767091	9.728241	3.27236	0.399395	3.118403	0	2.629476
NR4A3	1.744487	0.574657	1.770251	0.89915	0	0.262263	0	0.34985
NR5A1	0	0	4.303038	0.027518	0	0	0	0
NR5A2	1.372975	1.351919	2.800288	2.687116	0	0	0	0.237332
NR6A1	2.812089	1.458737	0.411931	2.242512	2.54185	3.520343	0	2.653218
NRADDP	0	0.74513	0	0	0	0	0	0
NRAP	0	0	0	0.044092	0	0	0	0
NRARP	42.326815	55.696568	24.110684	38.670892	19.290476	31.313992	11.518963	26.712124
NRAS	70.002504	73.451339	49.185081	53.775627	51.245034	28.807081	65.941998	62.385554
NRBF2	23.212969	50.20539	22.427428	53.016718	17.332907	34.562761	12.163079	41.641842
NRBF2P1	0	0	0	0	0	0	0	0
NRBF2P2	0	0	0	0.111123	0	0	0	0
NRBF2P3	0	0	0	0	0	0	0	0
NRBF2P4	0	0	0	0	0	0	0	0
NRBF2P5	0	0	0	0	0	0	0	0
NRBF2P6	5.214728	0	0	0.94356	0	3.135186	0	0
NRBP1	68.498669	118.70215	65.029554	92.712718	77.280826	152.335733	104.823406	124.122432
NRBP2	3.592567	3.873175	1.597391	7.869211	2.891114	2.991406	0	1.645187
NRCAM	0	1.198156	0	2.446113	0	0	0	0
NRDC	17.898936	17.409015	7.403151	75.776192	33.935095	97.174976	82.730973	4.761059
NRDE2	5.880074	11.49284	3.218674	18.056467	11.991288	26.633537	33.465428	14.651365
NREP	0	10.590689	28.169516	12.962402	7.422464	18.450814	0	9.564597
NRF1	7.034316	14.676877	10.173286	9.24986	4.102564	13.221015	0	10.324323
NRG1	65.232902	326.154268	128.117582	238.633417	78.108178	319.304669	212.487761	515.410853
NRG2	2.103533	3.256511	2.396759	6.138598	0	3.897296	6.707582	1.103476
NRG3	0	0	0	0	0	0	0	0
NRG4	12.136811	30.064557	2.631228	45.799904	0	2.086076	0	0.710457
NRGN	20.502571	15.235249	5.442489	12.251459	2.69422	9.974721	0	6.43288
NRIP1	28.323061	52.775416	64.436305	84.951411	16.03092	27.019465	15.107356	73.937185
NRIP2	0	0	0	0	0	0	0.297387	0
NRIP3	17.621392	9.058021	11.85714	9.705686	4.652961	4.180509	0	13.509412

NRK	0.399871	0	0	0.015354	0	0	0	0
NRL	5.214728	1.833561	0	0.936007	6.828812	0	0	2.800022
NRM	12.415047	13.881247	23.041989	13.461952	23.812517	9.775086	55.345468	7.656083
NRN1	0	0.771935	2.044067	1.036928	0	0	0	0
NRN1L	0	0	0	0	0	0	0	0
NRP1	7.509569	16.620022	10.469232	25.502944	5.898463	6.655934	2.499458	7.823133
NRP2	43.73283	20.084292	9.069726	46.530748	45.962145	13.559402	6.405647	8.548168
NRROS	0	0.877835	0	1.543213	0	0.792327	0.322394	1.016375
NRSN1	0	0	0	0	0	0	0	0
NRSN2	0	17.039439	19.219185	17.615843	0	14.554457	0	9.545755
NRXN1	0	0.834155	0	0.06593	0	0	0	0
NRXN2	1.880968	1.045689	8.203307	2.087559	0	3.299233	0	3.304484
NRXN3	1.562144	3.374887	2.481342	3.788421	0	0	0	0
NSA2	197.688153	284.009496	247.714813	211.180494	237.43783	192.159206	154.993072	242.581259
NSA2P1	0	0	0	0.123351	0	0	0	0
NSA2P2	0	0.215656	0	0	0	0	0	0
NSA2P3	0	0	0	0	0	0	0	0
NSA2P4	0	0	0	0	0	0	0	0
NSA2P5	0	0	0	0	0	0	0	0
NSA2P6	0	0	0	0	0	0	0	0
NSA2P7	0	0	0	0	0	0	0	0
NSD1	8.703412	4.642645	0	7.969301	0	17.336617	13.132553	13.631621
NSD2	97.723375	184.753438	81.29832	241.473935	102.277886	273.322305	138.241151	199.153044
NSD3	53.192635	194.70511	77.397773	187.294387	83.38231	118.265148	51.505605	142.396546
NSDHL	54.478936	62.963935	39.750029	61.21789	34.878865	42.256609	1.052553	45.062019
NSF	71.348867	17.320153	19.561132	20.817159	9.208982	15.9963	21.866476	13.281892
NSFL1C	59.142789	74.483937	54.362729	109.840279	67.321734	102.61828	116.740337	67.112487
NSFP1	7.771063	15.022775	0	11.835854	37.364122	6.848462	17.524196	15.754727
NSG1	0	1.884932	2.721772	1.889893	4.416616	2.326753	0	2.966748
NSG2	0	0	0	0	0	0	0	0
NSL1	22.831557	27.113373	31.657038	12.717305	14.702957	10.974129	32.532975	12.979596
NSMAF	60.026033	37.909361	26.683039	55.00481	28.029077	36.226333	209.589144	37.633083
NSMCE1	99.140311	142.223525	88.598608	152.373879	66.555892	85.191186	117.91427	87.529923

NSMCE2	25.104975	32.811365	25.018199	39.133931	26.405712	30.134846	148.89104	32.731949
NSMCE3	16.839501	28.015531	30.007358	24.777528	8.872897	23.751119	0	23.25408
NSMCE4A	46.345928	73.471833	27.831595	37.17569	65.27547	33.317408	22.010755	44.463539
NSMF	31.641652	37.071018	17.724687	23.095204	23.157928	36.900408	35.417686	27.626431
NSRP1	38.001301	43.699113	39.437382	59.255842	28.301619	33.21319	38.628415	23.877302
NSRP1P1	0	0.177785	1.860725	0	0	0.308735	0	0
NSUN2	111.519396	166.150219	68.829695	138.132146	63.439849	110.837638	99.825789	129.987407
NSUN3	9.585885	4.099457	12.926981	18.551767	4.132686	9.085375	2.582931	13.393722
NSUN4	7.953286	6.120067	10.827888	14.470451	10.994823	15.883938	11.988103	10.768644
NSUN5	19.935444	1.002563	2.204553	2.094062	14.784222	0	0	1.994896
NSUN5P1	0	0	0	0.716601	0	0.671953	0	0
NSUN5P2	0	1.624763	0	1.019214	0	0.503731	0	0
NSUN6	18.867136	5.393335	4.262259	4.233648	0.419281	1.339403	6.5493	3.6539
NSUN7	5.810403	14.191046	6.540057	14.785742	0	5.964469	6.28766	6.818263
NT5C	32.617731	37.165433	25.206143	44.800997	13.458232	32.848154	47.884535	27.994067
NT5C1B	0	0	0	0	0	0	0	0
NT5C1B-RDH	0	0	0	0	0	0	0	0
NT5C2	69.989491	82.184185	25.046675	76.679212	8.968459	60.644223	0	101.83025
NT5C3A	0	17.30448	28.309136	10.168397	2.706897	6.661408	45.254903	13.289501
NT5C3AP1	0	0	0	0	0	0	0	0
NT5C3AP2	0	0	0	0	0	0	0	0
NT5C3B	19.665521	25.638867	50.528555	43.030797	56.075565	52.927472	106.292672	46.96574
NT5CP1	0	0	0	0	0	0	0	0
NT5CP2	0	0	0	0	0	0	0	0.696454
NT5DC1	42.148353	28.495433	27.356034	31.191226	42.684178	33.656224	1.141088	31.518746
NT5DC1P1	0	0	0	0	0	0	0	0
NT5DC1P2	0	0	0	0	0	0	0	0
NT5DC2	29.780886	38.586357	41.516785	29.057521	78.008335	32.002673	186.84975	27.6769
NT5DC3	10.714203	5.169188	6.42423	4.730764	10.499715	15.852749	8.818427	11.212349
NT5DC4	0	0	0	0.033871	0	0.196414	0	3.678769
NT5E	39.184645	28.021553	53.554628	35.402744	129.92508	26.176284	34.942368	35.447839
NT5ELP	0	0	0	0	0	0	0	0
NT5M	0	1.077685	0	2.100262	0	0.252381	0	0.99935

NTAN1	18.337954	38.15676	35.319768	14.446534	68.361813	18.693871	29.866381	26.389428
NTAN1P1	0	0	0	0	0	0	0	0
NTAN1P2	0	0.170937	0	0.099796	0	0	0	0
NTAN1P3	0	0	0	0	0	0	0	0
NTAQ1	9.643361	25.595645	38.864793	32.932984	46.276473	73.342943	40.433866	55.164779
NTF3	5.446572	3.637618	0	2.315727	0	14.123002	41.732175	11.634036
NTF6A	0	0	0	0	0	0	0	0
NTF6B	8.360194	0	0	0	0	0	0	0
NTF6G	0	0	0	0	0	0	0	0
NTHL1	10.193358	15.200908	9.863714	14.931849	18.20408	19.123563	0	14.242697
NTM	6.558266	0	0	0.30998	0	1.920668	0	0.499346
NTMT1	11.633294	59.164063	77.247759	80.378967	15.26015	99.915142	6.602457	81.821505
NTMT2	0	0	0	0	0	0	0	0
NTN1	1.629639	2.218444	2.441481	2.058929	0	0.76254	0	0.348537
NTN4	43.535051	97.468867	48.503854	135.134245	69.570862	177.75201	47.919513	108.791117
NTN5	0	0	0	0	0	0.575029	0	0
NTNG1	11.753539	32.641101	24.961941	25.587384	0	1.11957	1.160041	0.791504
NTNG2	0	0.150592	0	0.102915	0	0.105408	0	0
NTPCR	75.784746	59.093533	40.654243	47.273454	63.918733	40.78064	39.728506	40.502749
NTRK1	0	0	0	0	0	0	0	0
NTRK3	0	0	0	0.017164	0	0	0	0
NTS	0	0	0	0	0	0	0	0
NTSR1	5.696821	8.243376	2.149073	7.025901	1.503537	0.638358	0	0.576827
NTSR2	0	0	0	0	0	0	0	0
NUAK1	0	6.11039	13.641412	10.956495	0	74.046804	82.357368	34.815594
NUB1	72.117412	55.379037	5.33391	77.158498	146.705914	70.851805	130.32378	68.655753
NUBP1	18.350771	41.107835	25.091825	56.009869	61.680773	42.909607	18.191398	36.434342
NUBP2	13.522959	20.619542	22.49941	25.001099	9.252742	36.196226	55.517184	30.630779
NUBPL	23.172766	39.929903	16.289446	32.691612	36.578969	17.811952	1.868069	18.41305
NUCB1	17.410102	29.576614	39.325265	51.777531	47.265325	74.584592	70.820387	32.880571
NUCB2	6.160739	47.854246	30.314087	46.853879	57.032687	14.018343	0.429424	35.13238
NUCKS1	167.855984	390.98597	166.254545	174.886932	172.187641	168.861709	371.67344	250.366839
NUCKS1P1	0	0	0	0	0	0	0	0

NUDC	127.406757	196.576482	106.595196	213.042051	223.611299	304.997514	193.291559	222.079405
NUDCD1	38.280801	39.184554	34.436389	15.510212	28.952528	6.137358	38.731159	26.255801
NUDCD2	36.243732	130.747817	25.442963	86.788077	21.30794	67.055437	67.862538	75.929454
NUDCD3	25.12032	81.335717	34.784796	113.62654	113.73116	227.8378	49.267007	140.527882
NUDCP1	0	0	0	0	0	0.291838	0	0.509798
NUDCP2	0	0	0	0	0	0	0	0
NUDT1	0	56.496643	4.329181	52.673238	4.657452	17.443704	0	25.810941
NUDT10	12.670003	7.675476	5.440759	6.356687	0.477113	0.595205	0	1.587728
NUDT11	18.647115	17.229065	21.336563	11.152422	0.756733	3.157653	0	3.020286
NUDT12	14.158212	13.236589	14.294906	9.243043	5.036696	2.550848	10.972807	8.929646
NUDT13	22.50976	2.757011	3.404903	2.689003	0	0.692249	0	0.308315
NUDT14	8.800323	19.706997	12.384123	17.792977	21.07176	19.787722	3.358789	19.262419
NUDT15	21.559062	28.871924	30.466821	15.71679	26.521025	22.916755	16.957801	40.098897
NUDT16	15.870794	3.95631	0	1.369553	0	2.232939	3.201267	1.447035
NUDT16L1	4.268585	3.952469	0	0.95917	1.120013	1.56473	43.814609	0.621265
NUDT16L2P	0	0	0	0	0	0	0	0
NUDT17	0	2.355826	4.017382	1.537222	0	1.008941	0	1.187911
NUDT18	7.112831	0	4.2186	2.549482	3.121649	3.435109	0	1.105314
NUDT19P2	0	0	0	0	0	0	0	0
NUDT19P3	0	0	0	0	0	0	0	0
NUDT19P4	0	0	0	0	0	0	0	0
NUDT19P5	0	0	0	0	0	0	0	0
NUDT19P6	0	0	0	0	0	0	0	0
NUDT2	4.913931	11.411908	6.828602	17.124905	0	16.909791	0	7.17771
NUDT21	243.707345	295.78324	168.15056	261.34896	239.273304	230.302005	168.191384	310.754686
NUDT22	32.263844	36.16922	31.178176	34.325207	52.28814	44.582277	0	32.900047
NUDT3	19.133462	19.74523	16.992789	17.553777	21.659923	11.56818	13.06212	15.917351
NUDT4	42.038784	86.463528	46.118727	59.21538	69.175371	72.28302	32.953088	84.117862
NUDT4P2	29.775564	94.954052	16.374225	71.920977	100.128171	137.621185	279.695362	159.893305
NUDT5	61.187118	0.720152	4.157835	4.289555	62.133745	1.462822	0	1.757536
NUDT5P1	0	0	0	0	0	0	0	0
NUDT6	3.576047	8.992309	11.089684	6.754401	0.68711	3.59992	0	8.921333
NUDT7	0	2.00198	5.079234	3.35624	0	1.174083	23.422039	0.984419

NUDT8	27.235179	11.472727	9.808191	14.22245	0	8.086498	0	7.857151
NUDT9	4.157496	38.493538	19.982863	36.075734	26.884394	28.129198	0	24.473611
NUDT9P1	0	1.356941	0	1.733509	0	3.9949	0	2.542019
NUF2	29.754649	31.810635	15.258086	15.065309	40.650912	19.842405	9.194349	42.53747
NUFIP1	7.649407	4.864889	10.289924	4.221981	6.311122	4.771661	1.395074	4.073514
NUFIP1P1	0	0	0	0	0	0	0	0
NUFIP2	41.560554	62.158964	35.819856	93.205307	42.936126	107.319423	42.230888	87.404565
NUGGC	0	0	0	0	0	0	0	0
NUMA1	101.576062	78.450513	56.647605	107.263531	49.183513	172.318343	85.458437	148.752657
NUMB	47.234387	221.331441	109.295727	284.257731	80.672606	264.763186	140.558057	214.529345
NUMBL	11.198928	57.805673	17.577788	50.307386	7.884832	65.102775	37.08494	36.639909
NUP107	85.923329	56.397394	92.096582	60.387599	105.552663	62.059802	122.5986	87.737098
NUP133	26.97504	17.839797	20.251523	15.179483	33.10413	15.0437	49.568311	23.321304
NUP153	18.110754	13.066467	7.608242	17.484157	13.015007	29.779269	55.967064	25.266658
NUP155	64.322862	53.000461	59.002676	37.490959	72.241006	15.780988	48.357679	45.208859
NUP160	0	7.484291	0	5.621912	12.888931	22.995969	36.447992	15.557789
NUP188	67.263701	59.639662	33.960459	102.154746	169.632287	69.455511	50.645837	95.262908
NUP205	38.878278	27.501213	44.739536	28.096952	49.994195	22.022318	79.12915	39.312912
NUP210	15.260589	12.828443	33.101884	21.198187	6.216844	12.361533	6.082	5.603311
NUP210L	0	0	0	0	0	0	0	0
NUP210P1	0	0	0	0	0	0	0	0
NUP210P2	0	0	0	0	0	0	0	0
NUP210P3	0	0	0	0	0	0	0	0
NUP214	62.079902	25.310394	23.335833	26.718579	103.539332	20.619949	149.387983	24.016339
NUP35	11.576072	12.50063	20.603676	16.209249	19.683817	11.20549	61.294786	15.156191
NUP35P1	0	0	0	0	0	0	0	0
NUP35P2	0	0	0	0	0	0	0	0
NUP37	106.543488	93.485001	62.713567	56.368129	153.379309	66.613993	71.07635	178.125147
NUP42	50.594195	33.942567	31.869385	36.816422	57.917561	25.05361	16.414719	45.511437
NUP43	19.875688	29.020057	25.587537	30.192764	69.595524	21.957988	26.060377	26.974734
NUP50	44.962549	146.929629	32.494579	138.148633	130.135869	252.745307	89.942396	241.719389
NUP50P1	0	0	0	0.188247	0	0	0	0
NUP50P2	0	0.121796	0	0	0	0	0	0

NUP50P4	0	0	0	0	0	0	0	0
NUP54	45.781968	41.435901	82.95159	56.307486	38.312423	36.223323	48.382287	39.24032
NUP58	29.583079	68.239923	22.0348	33.253904	16.559321	9.368651	22.39027	36.949259
NUP58P1	0	0	0	0	0	0	0	0
NUP62	20.018819	109.569042	24.542658	82.099381	32.944723	134.816836	38.738272	103.67279
NUP62CL	30.872698	28.735361	8.424695	35.167937	4.756841	7.46099	6.853586	13.293865
NUP85	56.454304	65.51711	39.003762	48.870743	20.038821	51.298447	28.226168	56.607412
NUP88	48.083544	41.61967	27.039467	37.801349	86.224909	54.435808	136.156764	72.374204
NUP93	181.942904	141.739135	129.584739	131.967321	146.599815	150.692006	241.789895	122.403587
NUP98	31.355527	28.592234	46.102993	33.759322	50.179272	44.891264	66.854519	35.882365
NUPR1	8.586014	0	0	0.200802	0	36.07334	0	5.807565
NUPR2	0	0	0	0	0	0	0	0
NUPR2P1	0	0	0	0.641672	0	0	0	0
NUS1	34.168103	20.578129	30.075552	12.661974	24.729746	4.974258	17.80783	19.448009
NUS1P1	0	10.581207	0	4.765867	0	4.241574	0	0
NUS1P2	0	0	0	0	0	0	0	0
NUS1P3	0	0	0	0	0	0	0	0
NUS1P4	0	0	0	0	0	0	0	0
NUSAP1	94.560689	174.173201	122.045046	171.131992	69.881817	152.687555	84.624738	149.484573
NUTF2	8.756891	47.577535	50.52534	32.907683	0	21.755059	109.532475	29.979639
NUTF2P2	0	0	0	0	0	0	0	0
NUTF2P3	0	0	0	0	0	0	0	0
NUTF2P4	0	0	0	0	0	0	0	0
NUTF2P5	0	0	0	0	0	0	0	0
NUTF2P6	0	0	0	0	0	0	0	0
NUTF2P7	0	0	0	0	0	0	0	0
NUTF2P8	0	0	0	0	0	0	0	0
NUTM1	0	0	0	0	0	0	0	0
NUTM2A	0	0	0	2.254491	3.974279	0.202139	0.123326	0.715287
NUTM2D	0	1.948338	0	6.468547	3.837671	3.581257	0	0.239107
NUTM2E	0	0	0	0	0	0.416613	0	0
NUTM2G	0	0	0	0	0	0.058996	0	0
NUTM2HP	0	0	0	0	0	0	0	0

NVL	13.23009	21.026259	14.798666	17.882139	11.795475	11.815619	86.865672	12.305335
NWD1	0	0.788019	0	0.63907	0	1.318822	3.084348	1.228437
NWD2	0	0.071283	0	0.020255	0	0.020777	0	0.049753
NXF1	35.139587	20.333761	14.716071	22.749645	19.791389	26.960466	88.984614	38.847967
NXF2	0	0	0	0	0	0	0	0
NXF2B	0	0	0	0	0	0	0	0
NXF3	0	0	0	0	0	0	0	0
NXF4	0	0	0	0	0	0	0	0
NXN	37.452253	55.509881	63.679628	69.863872	94.476285	101.951578	118.454427	69.874748
NXNL2	0	0	0	0	0	0.121466	0	0
NXNP1	0	0	0	0	0	0	0	0
NXPE1	0	0	0	0	0	0	0	0
NXPE2P1	0	0	0	0	0	0	0	0
NXPE3	22.960169	36.953592	19.154007	22.92223	5.284025	23.340687	9.620084	44.686505
NXPE4	0	0	0	0	0	0	0	0
NXPH1	0	0	0	0.176983	0	0	0	0
NXPH2	0	0	0	0	0	0	0	0
NXPH3	0	0.132145	0	0.241947	0	0.6797	0	0.529598
NXPH4	5.831875	35.221465	20.803264	33.569336	7.172167	10.17314	26.308588	5.339309
NXT1	68.176946	148.429212	34.875859	139.132942	5.656568	204.949429	58.466476	206.215398
NXT1P1	0	0	0	0	0	0	0	0
NXT2	0	9.173284	0	8.605601	0	3.765574	0	10.077904
NYAP1	0	0.207146	0.831406	0.18093	0	0.302383	0	0.293439
NYAP2	0	0	0	0	0	0	0	0
NYNRIN	2.114999	3.187308	5.707445	3.954084	25.5197	13.548919	0.10317	6.730098
NYX	0	0	0	0	0	0	0	0
OACYLP	0	0	0	0	0	0.077182	0	0
OAF	110.266434	288.938248	228.403938	456.028303	39.197003	107.613719	52.935835	76.720668
OARD1	16.571675	47.330308	28.071675	43.660309	16.89418	38.745046	27.955085	41.15022
OAS1	3.82313	3.45465	3.656574	8.656751	6.350249	42.665216	33.194766	29.677856
OAS2	3.338207	1.285285	3.63272	2.06905	26.511586	13.602801	0	19.58139
OAS3	35.819535	33.46538	39.211297	30.078747	291.866615	227.769792	218.487382	163.309787
OASL2P	0	0	0	0	0	0	0	0

OAT	56.386507	38.112016	0	10.135459	6.470456	6.795065	39.91787	30.53283
OAZ1	657.881254	731.322239	601.802346	664.346499	491.688216	566.112857	199.982585	487.681026
OAZ1P1	0	0	0	0	0	0	0	0
OAZ2	100.958356	124.659722	64.688898	130.815776	18.631022	104.897286	119.903412	122.156471
OAZ3	0	1.204457	0	1.405626	0	3.286179	0	2.127629
OBI1	22.761785	8.800096	17.86186	6.571021	15.274253	2.745317	7.841537	9.184888
OBP2A	0	0	0	0	0	0	0	0
OBP2B	0	0	0	0	0	0	0	0
OBSCN	9.012481	10.315918	4.531595	9.479507	6.291719	7.958032	41.157807	1.410296
OBSL1	17.233038	17.410928	18.164207	24.300093	27.525696	43.47982	18.83041	35.278342
OC90	0	0	0	0	0	0	0	0
OCA2	0	0	0	0	0	0	0	0
OCEL1	5.446572	60.9728	37.184015	79.371933	2.866577	48.864514	28.310803	29.523988
OCIAD1	87.581787	46.522314	9.967062	54.048486	77.174065	51.217528	96.13172	32.227694
OCIAD2	93.995978	302.176761	161.382251	262.307326	59.610323	169.504394	103.873671	114.539173
OCLN	9.459919	32.015856	29.240183	25.479095	23.199103	12.083308	32.076814	20.157546
OCLNP1	0	0	0	0	0	0	0	0
OCM	0	0	0	0	0	0	0	0
OCM2	0	0	0	0	0	0	0	0
OCRL	59.05267	48.970963	39.438383	47.593721	28.929523	28.118499	20.847756	38.802048
ODAD1	0	0	0	0.075051	0	0	0	0
ODAD2	0	2.720164	0	0.445232	0	0	0	1.268118
ODAD2P1	0	0	0	0	0	0	0	0
ODAD3	0	0.395497	0	1.85355	0	0.563527	9.059238	0.289214
ODAD4	0	0.136279	0	0.842914	0	0	0	1.40829
ODAM	0	0	0	0	0	0	0	0
ODAPH	0	0	0	0.195734	0	0	0	0
ODC1	120.256283	131.209937	95.728802	74.801238	107.931431	61.092228	169.534528	190.307476
ODCP	0	0	0	0	0	0	0	0
ODF1	0	0	0	0	0	0	0	0
ODF2	11.229295	5.329504	0	8.063694	11.692645	17.529721	0	13.63828
ODF2L	39.254399	21.285096	16.037578	8.540816	3.501431	6.606459	0	13.177436
ODF4	0	0	0	0	0	0	0	0

ODR4	12.134842	13.861122	22.260784	8.378749	2.492073	3.247371	19.757704	9.952715
OFCC1	0	0	0	0	0	0	0	0
OFD1	7.811052	9.527237	5.840186	8.96945	10.692882	16.806283	13.53988	24.786458
OFD1P10Y	0	0	0	0	0	0	0	0
OFD1P11Y	0	0	0	0	0	0	0	0
OFD1P12Y	0	0	0	0	0	0	0	0
OFD1P13Y	0	0	0	0	0	0	0	0
OFD1P15Y	0	0	0	0	0	0	0	0
OFD1P16Y	0	0	0	0	0	0	0	0
OFD1P17	0	0	0	0	0	0	0	0
OFD1P18Y	0	0	0	0	0	0	0	0
OFD1P1Y	0	0	0	0	0	0	0	0
OFD1P3Y	0	0	0	0	0	0	0	0
OFD1P4Y	0	0	0	0	0	0	0	0
OFD1P6Y	0	0	0	0	0	0	0	0
OFD1P7Y	0	0	0	0	0	0	0	0
OFD1P8Y	0	0	0	0	0	0	0	0
OFD1P9Y	0	0	0	0	0	0	0	0
OGA	66.846306	141.476874	82.199325	109.674271	81.173978	36.74515	52.204108	65.723143
OGDH	27.514804	25.324037	16.364048	32.843911	77.196805	75.335178	71.559014	66.024992
OGDHL	0	1.642087	2.650038	2.168146	3.3811	1.130391	0	0.673134
OGFOD1	142.988428	71.335379	44.458606	77.04344	67.713854	84.685596	209.362185	65.893217
OGFOD1P1	0	0	0	0.054193	0	0	0	0
OGFOD2	0	17.391278	8.502236	13.060467	7.054662	12.594791	0	10.936705
OGFOD3	20.162128	52.140993	19.025816	54.030779	9.727517	45.852651	7.111921	31.855832
OGFR	16.417797	30.245459	32.923699	38.03752	28.008834	81.184008	32.743274	36.99445
OGFRL1	6.979219	5.174866	4.706389	4.050757	10.327391	4.984793	6.675096	10.743126
OGG1	9.659341	15.096848	1.181394	12.213982	6.2232	11.751322	32.187702	5.715186
OGN	0	0	0	0	0	0	0	0
OGT	63.613162	39.386482	32.061088	77.785975	52.957417	71.644893	74.147477	76.180566
OIP5	15.490121	9.404273	8.234695	7.838306	26.070237	11.777974	22.330782	23.666182
OIT3	0	0	0	0	0	0	0	0
OLA1	163.629426	104.120912	138.304957	74.122775	198.294102	69.350132	321.250624	151.961801

OLA1P1	0	0	0	0	0	0	0	0
OLA1P2	0	0	0	0	0	0	0	0
OLA1P3	0	0	0	0	0	0	0	0
OLAH	0	0	0	0	0	0	0	0
OLFM1	15.88112	19.448827	13.438286	26.679927	0	29.539882	0	30.231052
OLFM2	2.127672	3.53066	0	0	0	1.495378	0	1.078355
OLFM3	0	0	0	0	0	0	0	0
OLFM4	0	0	0	0	0	0	0	0
OLFM5P	0	0	0	0.235416	0	0.477834	0	0.098528
OLFML1	0	0	0	0	0	0	0	0
OLFML2A	16.788225	15.90913	18.203088	23.256522	31.426481	41.197505	15.682559	15.206837
OLFML2B	0	0	0	0.245483	0	0.06267	0	0
OLFML3	0	0	0	0	0	0.198747	0	0.08141
OLIG1	0	0.289946	0	0.168497	0	0.700857	0	0.547157
OLIG2	0	0.116878	0	0.133785	0	0	0	0
OLR1	30.515139	26.589029	38.466787	45.822073	110.505057	99.406942	128.239214	49.311107
OMA1	43.179205	68.476008	30.851432	19.192414	54.561523	3.805521	41.308133	19.695631
OMG	0	0	0	0	0	0	0	0
OMP	0	0	0	0	0	0	0	0
ONECUT1	0	0	0	0.294318	0	0	0	0
ONECUT2	1.357809	1.732676	1.222971	0.413505	0.461557	0.493901	0.047372	0.170822
OOEP	0	0	0	0	0	0	0	0
OOEPP1	0	0	0	0	0	0	0	0
OOEPP2	0	0	0	0	0	0	0	0
OOSP1	0	0	0	0	0	0	0	0
OOSP1P1	0	0	0	0	0	0	0	0
OOSP1P2	0	0	0	0	0	0	0	0
OOSP2	0	0	0	0	0	0	0	0
OOSP4B	0	0	0	0	0	0	0	0
OPA1	95.485816	218.171905	148.277172	184.508791	106.338417	133.625261	197.626547	181.368671
OPA3	0	0	0	0.990663	0	0	0	4.056257
OPALIN	0	0	0	0	0	0	0	0
OPCML	0	0	0	0	0	0	0	0

OPHN1	5.974918	10.391186	9.59721	16.8228	33.449899	12.170258	37.808394	7.339381
OPLAH	0.821588	2.076175	2.949437	1.785181	0	1.026543	0	0.593889
OPN1LW	0	0	0	0	0	0	0	0
OPN1MW	0	0	0	0	0	0	0	0
OPN1MW2	0	0	0	0	0	0	0	0
OPN3	14.969837	25.183531	17.635656	10.827933	32.05469	7.60139	0	11.407066
OPN4	0	0	0	0	0	0	0	0
OPN5	0	0	0	0	0	0	0	0
OPRD1	0	0.029749	0	0.033794	0	0	0	0.041475
OPRK1	0	0	0	0	0	0	0	0
OPRL1	0	4.388568	0	2.507613	0.82815	5.032829	0	4.779366
OPRM1	0	0.018195	0	0	0	0	0	0
OPRPN	0	0	0	0	0	0	0	0
OPTN	31.435118	45.488127	19.574341	51.960029	54.706519	80.673095	0.3353	54.702652
OR10A2	0	0	0	0	0	0	0	0
OR10A3	0	0	0	0	0	0	0	0
OR10A4	0	0	0	0	0	0	0	0
OR10A6	0	0	0	0	0	0	0	0
OR10A7	0	0	0	0	0	0	0	0
OR10AA1P	0	0	0	0	0	0	0	0
OR10AB1P	0	0	0	0	0	0	0	0
OR10AC1	0	0	0	0	0	0	0	0
OR10AE1P	0	0	0	0	0	0	0	0
OR10AE3P	0	0	0	0	0	0	0	0
OR10AF1P	0	0	0	0	0	0	0	0
OR10AG1	0	0	0	0	0	0	0	0
OR10AH1P	0	0	0	0	0	0	0	0
OR10AK1P	0	0	0	0	0	0	0	0
OR10B1P	0	0	0	0	0	0	0	0
OR10C1	0	0	0	0	0	0	0	0
OR10D1P	0	0	0	0	0	0	0	0
OR10D3	0	0	0	0	0	0	0	0
OR10D5P	0	0	0	0	0	0	0	0

OR10G1P	0	0	0	0	0	0	0	0
OR10G2	0	0	0	0	0	0	0	0
OR10G3	0	0	0	0	0	0	0	0
OR10G4	0	0	0	0	0	0	0	0
OR10G5P	0	0	0	0	0	0	0	0
OR10G6	0	0	0	0	0	0	0	0
OR10G7	0	0	0	0	0	0	0	0
OR10G8	0	0	0	0	0	0	0	0
OR10G9	0	0	0	0	0	0	0	0
OR10H1	0	0	0	0	0	0	0	0
OR10H3	0	0	0	0	0	0	0	0
OR10H4	0	0	0	0	0	0	0	0
OR10H5	0	0	0	0	0	0	0	0
OR10J1	0	0	0	0	0	0	0	0
OR10J2P	0	0	0	0	0	0	0	0
OR10J3	0	0	0	0	0	0	0	0
OR10J4	0	0	0	0	0	0	0	0
OR10J5	0	0	0	0	0	0	0	0
OR10J6P	0	0	0	0	0	0	0	0
OR10J7P	0	0	0	0	0	0	0	0
OR10J8P	0	0	0	0	0	0	0	0
OR10J9P	0	0	0	0	0	0	0	0
OR10K1	0	0	0	0	0	0	0	0
OR10K2	0	0	0	0	0	0	0	0
OR10N1P	0	0	0	0	0	0	0	0
OR10Q1	0	0	0	0	0	0	0	0
OR10Q2P	0	0	0	0	0	0	0	0
OR10R1P	0	0	0	0	0	0	0	0
OR10R2	0	0	0	0	0	0	0	0
OR10R3P	0	0	0	0	0	0	0	0
OR10S1	0	0	0	0	0	0	0	0.136815
OR10T1P	0	0	0	0	0	0	0	0
OR10T2	0	0	0	0	0	0	0	0

OR10U1P	0	0	0	0	0	0	0	0
OR10V1	0	0	0	0	0	0	0	0
OR10V2P	0	0	0	0	0	0	0	0
OR10V3P	0	0	0	0	0	0	0	0
OR10W1	0	0	0	0	0	0	0	0
OR10X1	0	0	0	0	0	0	0	0
OR10Y1P	0	0	0	0	0	0	0	0
OR10Z1	0	0	0	0	0	0.077242	0	0
OR11A1	0	0	0	0	0	0	0	0
OR11G1P	0	0	0	0	0	0	0	0
OR11G2	0	0	0	0	0	0	4.470047	0
OR11H1	0	0	0	0	0	0	0	0
OR11H13P	0	0	0	0	0	0	0	0
OR11H2	0	0	0	0	0	0	0	0
OR11H3P	0	0	0	0	0	0	0	0
OR11H4	0	0	0	0	0	0	0	0
OR11H5P	0	0	0	0	0	0	0	0
OR11H6	0	0	0	0	0	0	0	0
OR11H7	0	0	0	0	0	0	0	0
OR11I1P	0	0	0	0	0	0	0	0
OR11J2P	0	0	0	0	0	0	0	0
OR11J5P	0	0	0	0	0	0	0	0
OR11J6P	0	0	0	0	0	0	0	0
OR11J7P	0	0	0	0	0	0	0	0
OR11K1BP	0	0	0	0	0	0	0	0
OR11K1P	0	0	0	0	0	0	0	0
OR11K2P	0	0	0	0	0	0	0	0
OR11L1	0	0	0	0	0	0	0	0
OR11M1P	0	0	0	0	0	0	0	0
OR11N1P	0	0	0	0	0	0	0	0
OR11P1P	0	0	0	0	0	0	0	0
OR11Q1P	0	0	0	0	0	0	0	0
OR12D1	0	0	0	0	0	0	0	0

OR12D2	0	0	0	0	0	0	0	0
OR12D3	0	0	0	0	0	0	0	0
OR13A1	0	0	0	0.076421	0	0	0	0
OR13C1P	0	0	0	0	0	0	0	0
OR13C2	0	0	0	0	0	0	0	0
OR13C3	0	0	0	0	0	0	0	0
OR13C4	0	0	0	0	0	0	0	0
OR13C5	0	0	0	0	0	0	0	0
OR13C6P	0	0	0	0	0	0	0	0
OR13C7	0	0	0	0	0	0	0	0
OR13C8	0	0	0	0	0	0	0	0
OR13C9	0	0	0	0	0	0	0	0
OR13D1	0	0	0	0	0	0	0	0
OR13D2P	0	0	0	0	0	0	0	0
OR13D3P	0	0	0	0	0	0	0	0
OR13E1P	0	0	0	0	0	0	0	0
OR13F1	0	0	0	0	0	0	0	0
OR13G1	0	0	0	0	0	0	0	0
OR13I1P	0	0	0	0	0	0	0	0
OR13K1P	0	0	0	0	0	0	0	0
OR13Z1P	0	0	0	0	0	0	0	0
OR13Z2P	0	0	0	0	0	0	0	0
OR13Z3P	0	0	0	0	0	0	0	0
OR14A16	0	0	0	0	0	0	0	0
OR14A2	0	0	0	0	0	0	0	0
OR14C36	0	0	0	0	0	0	0	0
OR14I1	0	0	0	0	0	0	0	0
OR14J1	0	0	0	0	0	0	0	0
OR14K1	0	0	0	0	0	0	0	0
OR14L1	0	0	0	0	0	0	0	0
OR1A1	0	0	0	0	0	0	0	0
OR1A2	0	0	0	0	0	0	0	0
OR1AA1P	0	0	0	0	0	0	0	0

OR1AB1P	0	0	0	0	0	0	0	0
OR1AC1P	0	0	0	0	0	0	0	0
OR1C1	0	0	0	0	0	0	0	0
OR1D2	0	0	0	0	0	0	0	0
OR1D3P	0	0	0	0	0	0	0	0
OR1D4	0	0	0	0	0	0	0	0
OR1D5	0	0	0	0	0	0	0	0
OR1E1	0	0	0	0	0	0	0	0
OR1E2	0	0	0	0	0	0	0	0
OR1E3	0	0	0	0	0	0	0	0
OR1F2P	0	0	0	0.302115	0	0.202555	0	0.343767
OR1H1P	0	0	0	0	0	0	0	0
OR1I1	0	0	0	0	0	0	0	0
OR1J1	0	0	0	0	0	0	0	0
OR1J2	0	0	0	0	0	0	0	0
OR1J4	0	0	0	0	0	0	0	0
OR1K1	0	0	0	0	0	0	0	0
OR1L1	0	0	0	0	0	0	0	0
OR1L4	0	0	0	0	0	0	0	0
OR1L6	0	0	0	0	0	0	0	0
OR1L8	0	0	0	0	0	0	0	0
OR1M1	0	0	0	0	0	0	0	0
OR1M4P	0	0	0	0	0	0	0	0
OR1N1	0	0	0	0	0	0	0	0
OR1N2	0	0	0	0	0	0	0	0
OR1P1	0	0	0	0	0	0	0	0
OR1Q1	0	0	0	0	0	0	0	0
OR1Q1BP	0	0	0	0	0	0	0	0
OR1R1P	0	0	0	0	0	0	0	0
OR1S1	0	0	0	0	0	0	0	0
OR1X1P	0	0	0	0	0	0	0	0
OR1X5P	0	0	0	0	0	0	0	0
OR2A1	0	0	0	0	0	0	0	0

OR2A1-AS1	0	1.447609	5.156147	1.838212	0	0.283932	0	0
OR2A12	0	0	0	0	0	0	0	0
OR2A13P	0	0	0	0	0	0	0	0
OR2A14	0	0	0	0	0	0	0	0
OR2A15P	0	0	0	0	0	0	0	0
OR2A25	0	0	0	0	0	0	0	0
OR2A3P	0	0	0	0	0	0	0	0
OR2A4	10.009953	0	0	0	0	0.296268	0	0
OR2A41P	0	0	0	0	0	0	0	0
OR2A42	0	0	0	0	0	0	0	0
OR2A5	0	0	0	0	0	0	0	0
OR2A7	0	5.476832	12.312258	6.83134	0	0	0	0.745928
OR2A9P	0	0	0	0	0	0	0	0
OR2AD1P	0	0	0	0	0	0	0	0
OR2AF1P	0	0	0	0	0	0	0	0
OR2AG1	0	0	0	0	0	0	0	0
OR2AG2	0	0.08524	0	0.095321	0	0.195177	0	0
OR2AH1P	0	0	0	0	0	0	0	0
OR2AI1P	0	0	0	0	0	0	0	0
OR2AJ1	0	0	0	0	0	0	0	0
OR2AK2	0	0	0	0	0	0	0	0
OR2AL1P	0	0	0	0	0	0	0	0
OR2AM1P	0	0	0	0	0	0	0	0
OR2AO1P	0	0	0	0	0	0	0	0
OR2AP1	0	0	0	0	0	0	0	0.186972
OR2AQ1P	0	0	0	0	0	0	0	0
OR2AS1P	0	0	0	0	0	0	0	0
OR2AS2P	0	0	0	0	0	0	0	0
OR2AT1P	0	0	0	0	0	0	0	0
OR2AT2P	0	0	0	0	0	0	0	0
OR2AT4	0	0	0	0	0	0	0	0
OR2B11	0	0	0	0	0	0	0	0
OR2B2	0	0	0	0	0	0	0	0

OR2B3	0	0	0	0	0	0	0	0
OR2B4P	0	0	0	0	0	0	0	0
OR2B6	0	0	0	0	0	0	0	0
OR2B7P	0	0	0	0	0	0	0	0
OR2B8P	0	0	0	0	0	0	0	0
OR2BH1P	0	0	0	0	0	0	0	0
OR2C3	0	0	0	0	0	0	0	0
OR2E1P	0	0	0	0	0	0	0	0
OR2F1	0	0	0	0	0	0	0	0
OR2G1P	0	0	0	0	0	0	0	0
OR2G2	0	0	0	0	0	0	0	0
OR2G3	0	0	0	0	0	0	0	0
OR2G6	0	0	0	0	0	0	0	0
OR2H1	0	0	0	0	0	0	0	0
OR2H2	0	0	0	0	0	0	0	0
OR2H4P	0	0	0	0	0	0	0	0
OR2H5P	0	0	0	0	0	0	0	0
OR2I1P	0	0	0	0	0	0	0	0
OR2J1	0	0	0	0	0	0	0	0
OR2J2	0	0.482935	0	0	0	0	0	0
OR2J3	0	0	0	0	0	0	0	0
OR2J4P	0	0	0	0	0	0	0	0
OR2K2	0	0	0	0	0	0	0	0
OR2L13	0	0	0	0	0	0	0	0
OR2L1P	0	0	0	0	0	0	0	0
OR2L2	0.779913	0.24422	0	0.088506	0	0.139304	11.200508	0.371888
OR2L3	0	0	0	0	0	0	0	0
OR2L5	0	0	0	0	0	0	0	0
OR2L6P	0	0	0	0	0	0	0	0
OR2L8	0	0	0	0	0	0	0	0
OR2L9P	0	0	0	0	0	0	0	0
OR2M1P	0	0	0	0	0	0	0	0
OR2M2	0	0	0	0	0	0	0	0

OR2M3	0	0	0	0.031728	0	0	0	0
OR2M4	0	0	0	0	0	0	0	0
OR2M5	0	0	0	0	0	0	0	0
OR2M7	0	0	0	0	0	0	0	0
OR2N1P	0	0	0	0	0	0	0	0
OR2P1P	0	0	0	0	0	0	0	0
OR2Q1P	0	0	0	0	0	0	0	0
OR2R1P	0	0	0	0	0	0	0	0
OR2S1P	0	0	0	0	0	0	0	0
OR2S2	0	0	0	0	0	0	0	0
OR2T1	0	0	0	0	0	0	0	0
OR2T10	0	0	0	0	0	0	0	0
OR2T11	0	0	0	0	0	0	0	0
OR2T12	0	0	0	0	0	0	0	0
OR2T2	0	0	0	0	0	0	0	0
OR2T27	0	0	0	0	0	0	0	0
OR2T29	0	0	0	0	0	0	0	0
OR2T3	0	0	0	0	0	0	0	0
OR2T32P	0	0	0	0	0	0	0	0
OR2T33	0	0	0	0	0	0	0	0
OR2T34	0	0	0	0	0	0	0	0
OR2T35	0	0	0	0	0	0	0	0
OR2T4	0	0	0	0	0	0	0	0
OR2T5	0	0	0	0	0	0	0	0
OR2T6	0	0	0	0	0	0	0	0
OR2T7	0	0	0	0	0	0	0	0
OR2U1P	0	0	0	0	0	0	0	0
OR2U2P	0	0	0	0	0	0	0	0
OR2V1	0	0	0	0	0	0	0	0
OR2V2	0	0	0	0	0	0	0	0
OR2W1	0	0	0	0	0	0	0	0
OR2W2P	0	0	0	0	0	0	0	0
OR2W3	0	0	0	0	0	0	0	0

OR2W4P	0	0	0	0	0	0	0	0
OR2W5P	0	0	0	0	0	0	0	0
OR2W6P	0	0	0	0	0	0	0	0
OR2X1P	0	0	0	0	0	0	0	0
OR2Z1	0	0	0	0	0	0	0	0
OR3A1	0	0	0	0	0	0	0	0
OR3A2	0	0	0	0	0	0	0	0
OR3A3	0	0	0	0	0	0	0	0
OR3A4P	0	0	0	0	0	0	0	0
OR3B1P	0	0	0	0	0	0	0	0
OR3D1P	0	0	0	0	0	0	0	0
OR4A10P	0	0	0	0	0	0	0	0
OR4A11P	0	0	0	0	0	0	0	0
OR4A12P	0	0	0	0	0	0	0	0
OR4A13P	0	0	0	0	0	0	0	0
OR4A15	0	0	0	0	0	0	0	0
OR4A17P	0	0	0	0	0	0	0	0
OR4A18P	0	0	0	0	0	0	0	0
OR4A19P	0	0	0	0	0	0	0	0
OR4A1P	0	0	0	0	0	0	0	0
OR4A21P	0	0	0	0	0	0	0	0
OR4A2P	0	0	0	0	0	0	0	0
OR4A3P	0	0	0	0	0	0	0	0
OR4A40P	0	0	0	0	0	0	0	0
OR4A41P	0	0	0	0	0	0	0	0
OR4A42P	0	0	0	0	0	0	0	0
OR4A43P	0	0	0	0	0	0	0	0
OR4A44P	0	0	0	0	0	0	0	0
OR4A45P	0	0	0	0	0	0	0	0
OR4A46P	0	0	0	0	0	0	0	0
OR4A48P	0	0	0	0	0	0	0	0
OR4A49P	0	0	0	0	0	0	0	0
OR4A5	0	0	0	0	0	0	0	0

OR4A50P	0	0	0	0	0	0	0	0
OR4A6P	0	0	0	0	0	0	0	0
OR4A7P	0	0	0	0	0	0	0	0
OR4A8	0	0	0	0	0	0	0	0
OR4A9P	0	0	0	0	0	0	0	0
OR4B1	0	0	0	0	0	0	0	0
OR4B2P	0	0	0	0	0	0	0	0
OR4C10P	0	0	0	0	0	0	0	0
OR4C11	0	0	0	0	0	0	0	0
OR4C12	0	0	0	0	0	0	0	0
OR4C13	0	0	0	0	0	0	0	0
OR4C14P	0	0	0	0	0	0	0	0
OR4C15	0	0	0	0	0	0	0	0
OR4C16	0	0	0	0	0	0	0	0
OR4C1P	0	0	0	0	0	0	0	0
OR4C2P	0	0	0	0	0	0	0	0
OR4C3	0	0	0	0	0	0	0	0
OR4C45	0	0	0	0	0	0	0	0
OR4C46	0	0	0	0	0	0	0	0
OR4C48P	0	0	0	0	0	0	0	0
OR4C49P	0	0	0	0	0	0	0	0
OR4C4P	0	0	0	0	0	0	0	0
OR4C5	0	0	0	0	0	0	0	0
OR4C50P	0	0	0	0	0	0	0	0
OR4C6	0	0	0	0	0	0	0	0
OR4C7P	0	0	0	0	0	0	0	0
OR4C9P	0	0	0	0	0	0	0	0
OR4D1	0	0.399444	0	0.168291	0	0	0	0
OR4D10	0	0	0	0	0	0	0	0
OR4D11	0	0	0	0	0	0	0	0
OR4D2	0	0	0	0	0	0	0	0
OR4D7P	0	0	0	0	0	0	0	0
OR4D8P	0	0	0	0	0	0	0	0

OR4D9	0	0	0	0.01311	0	0	0	0
OR4E2	0	0	0	0	0	0	0	0
OR4F13P	0	0	0	0	0	0	0	0
OR4F14P	0	0	0	0	0	0	0	0
OR4F15	0	0	0	0	0	0	0	0
OR4F17	0	0.841873	0	0	0	1.48598	0	0.640483
OR4F1P	0	0	0	0	0	0	0	0
OR4F28P	0	0	0	0	0	0	0	0
OR4F2P	0	0	0	0	0	0	0	0
OR4F4	0	0	0	0	0	0	0	0
OR4F5	0	0	0	0	0	0	0	0
OR4F6	0	0	0	0	0	0	0	0
OR4F7P	0	0	0	0	0	0	0	0
OR4F8BP	0	0	0	0	0	0	0	0
OR4F8P	0	0	0	0	0	0	0	0
OR4G11P	0	0	0	0	0	0	0	0
OR4G1P	0	0	0	0	0	0	0	0
OR4G2P	0	0	0	0	0	0	0	0
OR4G3P	0	0	0	0	0	0	0	0
OR4G4P	0	0	0	0	0	0	0	0
OR4G6P	0	0	0	0	0	0	0	0
OR4H12P	0	0	0	0	0	0	0	0
OR4H6BP	0	0	0	0	0	0	0	0
OR4H6P	0	0	0	0	0	0	0	0
OR4K1	0	0	0	0	0	0	0	0
OR4K11P	0	0	0	0	0	0	0	0
OR4K12P	0	0	0	0	0	0	0	0
OR4K13	0	0	0	0	0	0	0	0
OR4K14	0	0	0	0	0	0	0	0
OR4K16P	0	0	0	0	0	0	0	0
OR4K17	0	0	0	0	0	0	0	0
OR4K2	0	0	0	0	0	0	0	0
OR4K3	0	0	0	0	0	0	0	0

OR4K4P	0	0	0	0	0	0	0	0
OR4K6P	0	0	0	0	0	0	0	0
OR4K7P	0	0	0	0	0	0	0	0
OR4K8P	0	0	0	0	0	0	0	0
OR4L1	0	0	0	0	0	0	0	0
OR4M1	0	0	0	0	0	0	0	0
OR4M2	0	0	0	0	0	0	0	0
OR4M2-OT1	0	0	0	0	0	0	0	0
OR4M2B	0	0	0	0	0	0	0	0
OR4N1P	0	0	0	0	0	0	0	0
OR4N2	0	0	0	0	0	0	0	0
OR4N3BP	0	0	0	0	0	0	0	0
OR4N3P	0	0	0	0	0	0	0	0
OR4N4C	0	0	0	0	0	0	0	0
OR4N5	0	0	0	0	0	0	0	0
OR4P1P	0	0	0	0	0	0	0	0
OR4P4	0	0	0	0	0	0	0	0
OR4Q1P	0	0	0	0	0	0	0	0
OR4Q2	0	0	0	0	0	0	0	0
OR4Q3	0	0	0	0	0	0	0	0
OR4R1P	0	0	0	0	0	0	0	0
OR4R2P	0	0	0	0	0	0	0	0
OR4R3P	0	0	0	0	0	0	0	0
OR4S1	0	0	0	0	0	0	0	0
OR4S2	0	0	0	0	0	0	0	0
OR4T1P	0	0	0	0	0	0	0	0
OR4U1P	0	0	0	0	0	0	0	0
OR4V1P	0	0	0	0	0	0	0	0
OR4X1	0	0	0	0	0	0	0	0
OR4X7P	0	0	0	0	0	0	0	0
OR51A10P	0	0	0	0	0	0	0	0
OR51A1P	0	0	0	0	0	0	0	0
OR51A2	0	0	0	0	0	0	0	0

OR51A3P	0	0	0	0	0	0	0	0
OR51A4	0	0	0	0	0	0	0	0
OR51A5P	0	0	0	0	0	0	0	0
OR51A6P	0	0	0	0	0	0	0	0
OR51A7	0	0	0	0	0	0	0	0
OR51A8P	0	0	0	0	0	0	0	0
OR51A9P	0	0	0	0	0	0	0	0
OR51AB1P	0	0	0	0	0	0	0	0
OR51B2	0	0	0	0	0	0	0	0
OR51B3P	0	0	0	0	0	0	0	0
OR51B4	0	0	0	0	0	0	0	0
OR51B5	0	0	0	0	0	0	0	0
OR51B6	0	0	0	0	0	0	0	0
OR51B8P	0	0	0	0	0	0	0	0
OR51C4P	0	0	0	0	0	0	0	0
OR51D1	0	0	0	0	0	0	0	0
OR51E1	0	0	0	0	0	0	0	0
OR51E2	0	0	0	0	0	0	0	0
OR51F2	0	0	0	0	0	0	0	0
OR51F3P	0	0	0	0	0	0	0	0
OR51F4P	0	0	0	0	0	0	0	0
OR51F5P	0	0	0	0	0	0	0	0
OR51G1	0	0	0	0	0	0	0	0
OR51G2	0	0	0	0	0	0	0	0
OR51H2P	0	0	0	0	0	0	0	0
OR51I1	0	0	0	0	0	0	0	0
OR51I2	0	0	0	0	0	0	0	0
OR51J1	0	0	0	0	0	0	0	0
OR51K1P	0	0	0	0	0	0	0	0
OR51L1	0	0	0	0	0	0	0	0
OR51M1	0	0	0	0	0	0	0	0
OR51N1P	0	0	0	0	0	0	0	0
OR51P1P	0	0	0	0	0	0	0	0

OR51Q1	0	0	0	0	0	0	0	0
OR51R1P	0	0	0	0	0	0	0	0
OR51T1	0	0	0	0	0	0	0	0
OR51V1	0	0	0	0	0	0	0	0
OR52A4P	0	0	0	0	0	0	0	0
OR52A5	0	0	0	0	0	0	0	0
OR52B1P	0	0	0	0	0	0	0	0
OR52B3P	0	0	0	0	0	0	0	0
OR52B4	0	0	0	0	0	0	0	0
OR52B5P	0	0	0	0	0	0	0	0
OR52B6	0	0	0	0	0	0	0	0
OR52D1	0	0	0	0	0	0	0	0
OR52E1	0	0	0	0	0	0	0	0
OR52E2	0	0	0	0	0	0	0	0
OR52E3P	0	0	0	0	0	0	0	0
OR52E4	0	0	0	0	0	0	0	0
OR52E5	0	0	0	0	0	0	0	0
OR52E6	0	0	0	0	0	0	0	0
OR52E7P	0	0	0	0	0	0	0	0
OR52H2P	0	0	0	0	0	0	0	0
OR52I2	0	0	0	0	0	0	0	0
OR52J1P	0	0	0	0	0	0	0	0
OR52J2P	0	0	0	0	0	0	0	0
OR52J3	0	0	0	0	0	0	0	0
OR52K1	0	0	0	0	0	0	0	0
OR52K2	0	0	0	0	0	0	0	0
OR52K3P	0	0	0	0	0	0	0	0
OR52L1	0	0	0	0	0	0	0	0
OR52M1	0	0	0	0	0	0	0	0
OR52M2P	0	0	0	0	0	0	0	0
OR52N1	0	0	0	0	0	0	0	0
OR52N3P	0	0	0	0	0	0	0	0
OR52N5	0	0	0	0	0	0	0	0

OR52P1	0	0	0	0	0	0	0	0
OR52P2P	0	0	0	0	0	0	0	0
OR52Q1P	0	0	0	0	0	0	0	0
OR52R1	0	0	0	0	0	0	0	0
OR52S1P	0	0	0	0	0	0	0	0
OR52T1P	0	0	0	0	0	0	0	0
OR52U1P	0	0	0	0	0	0	0	0
OR52V1P	0	0	0	0.105513	0	0	0	0
OR52X1P	0	0	0	0	0	0	0	0
OR52Y1P	0	0	0	0	0	0	0	0
OR52Z1P	0	0	0	0	0	0	0	0
OR55B1P	0	0	0	0	0	0	0	0
OR56A1	0	0	0	0	0	0	0	0
OR56A3	0	0	0	0	0	0	0	0
OR56A4	0	0	0	0	0	0	0	0
OR56A5	0	0	0	0	0	0	0	0
OR56A7P	0	0	0	0	0	0	0	0
OR56B3P	0	0	0	0	0	0	0	0
OR56B4	0	0	0	0	0	0	0	0
OR5A1	0	0	0	0	0	0	0	0
OR5A2	0	0	0	0	0	0	0	0
OR5AC1	0	0	0	0	0	0	0	0
OR5AC2	0	0	0	0	0	0	0	0
OR5AC4P	0	0	0	0	0	0	0	0
OR5AH1P	0	0	0	0	0	0	0	0
OR5AK1P	0	0	0	0	0	0	0	0
OR5AK4P	0	0	0	0	0	0	0	0
OR5AL1	0	0	0	0	0	0	0	0
OR5AL2P	0	0	0	0	0	0	0	0
OR5AM1P	0	0	0	0	0	0	0	0
OR5AN1	0	0	0	0	0	0	0	0
OR5AN2P	0	0	0	0	0	0	0	0
OR5AO1P	0	0	0	0	0	0	0	0

OR5AP1P	0	0	0	0	0	0	0	0
OR5AQ1P	0	0	0	0	0	0	0	0
OR5AS1	0	0	0	0.010048	0	0	0	0
OR5AU1	0	0	0	0	0	0	0	0
OR5AW1P	0	0	0	0	0	0	0	0
OR5AZ1P	0	0	0	0	0	0	0	0
OR5B10P	0	0	0	0	0	0	0	0
OR5B12	0	0	0	0	0	0	0	0
OR5B15P	0	0	0	0	0	0	0	0
OR5B17	0	0	0	0	0	0	0	0
OR5B19P	0	0	0	0	0	0	0	0
OR5B1P	0	0	0	0	0	0	0	0
OR5B2	0	0	0	0	0	0	0	0
OR5B3	0	0	0	0	0	0	0	0
OR5BA1P	0	0	0	0	0	0	0	0
OR5BB1P	0	0	0	0	0	0	0	0
OR5BC1P	0	0	0	0	0	0	0	0
OR5BD1P	0	0	0	0	0	0	0	0
OR5BE1P	0	0	0	0	0	0	0	0
OR5BH1P	0	0	0	0	0	0	0	0
OR5BJ1P	0	0	0	0	0	0	0	0
OR5BK1P	0	0	0	0	0	0	0	0
OR5BL1P	0	0	0	0	0	0	0	0
OR5BM1P	0	0	0	0	0	0	0	0
OR5BN1P	0	0	0	0	0	0	0	0
OR5BN2P	0	0	0	0	0	0	0	0
OR5BP1P	0	0	0	0	0	0	0	0
OR5BQ1P	0	0	0	0	0	0	0	0
OR5BR1P	0	0	0	0	0	0	0	0
OR5BS1P	0	0	0	0	0	0	0	0
OR5BT1P	0	0	0	0	0	0	0	0
OR5C1	0	0	0	0	0	0	0	0
OR5D13	0	0	0	0	0	0	0	0

OR5D14	0	0	0	0	0	0	0	0
OR5D15P	0	0	0	0	0	0	0	0
OR5D16	0	0	0	0	0	0	0	0
OR5D17P	0	0	0	0	0	0	0	0
OR5D2P	0	0	0	0	0	0	0	0
OR5D3P	0	0	0	0	0	0	0	0
OR5E1P	0	0.173766	0	0	0	0	0	0
OR5F1	0	0	0	0	0	0	0	0
OR5F2P	0	0	0	0	0	0	0	0
OR5G1P	0	0	0	0	0	0	0	0
OR5G3	0	0	0	0	0	0	0	0
OR5G4P	0	0	0	0	0	0	0	0
OR5G5P	0	0	0	0	0	0	0	0
OR5H1	0	0	0	0	0	0	0	0
OR5H14	0	0	0	0	0	0	0	0
OR5H15	0	0	0	0	0	0	0	0
OR5H3P	0	0	0	0	0	0	0	0
OR5H4P	0	0	0	0	0	0	0	0
OR5H5P	0	0	0	0	0	0	0	0
OR5H6	0	0	0	0	0	0	0	0
OR5H7P	0	0	0	0	0	0	0	0
OR5H8	0	0	0	0	0	0	0	0
OR5I1	0	0	0	0	0	0	0	0
OR5J1P	0	0	0	0	0	0	0	0
OR5J2	0	0	0	0	0	0	0	0
OR5J7P	0	0	0	0	0	0	0	0
OR5K1	0	0	0	0	0	0	0	0
OR5K3	0	0	0	0	0	0	0	0
OR5K4	0	0	0	0	0	0	0	0
OR5L1	0	0	0	0	0	0	0	0
OR5L2	0	0	0	0	0	0	0	0
OR5M1	0	0	0	0	0	0	0	0
OR5M12P	0	0	0	0	0	0	0	0

OR5M13P	0	0	0	0	0	0	0	0
OR5M14P	0	0	0	0	0	0	0	0
OR5M2P	0	0	0	0	0	0	0	0
OR5M3	0	0	0	0	0	0	0	0
OR5M4P	0	0	0	0	0	0	0	0
OR5M5P	0	0	0	0	0	0	0	0
OR5M6P	0	0	0	0	0	0	0	0
OR5M7P	0	0	0	0	0	0	0	0
OR5M9	0	0	0	0	0	0	0	0
OR5P1P	0	0	0	0	0	0	0	0
OR5P3	0	0	0	0	0	0	0	0
OR5P4P	0	0	0	0	0	0	0	0
OR5S1P	0	0	0	0	0	0	0	0
OR5T1	0	0	0	0	0	0	0	0
OR5T2	0	0	0	0	0	0	0	0
OR5T3	0	0	0	0	0	0	0	0
OR5V1	0	0	0	0	0	0	0	0
OR5W1P	0	0	0	0	0	0	0	0
OR5W2	0	0	0	0	0	0	0	0
OR6A2	0	0	0	0	0	0	0	0
OR6B1	0	0	0	0	0	0	0	0
OR6C1	0	0	0	0	0	0	0	0
OR6C2	0	0	0	0	0	0	0	0
OR6C3	0	0	0	0	0	0	0	0
OR6C4	0	0	0	0	0	0	0	0
OR6C5P	0	0	0	0	0	0	0	0
OR6C6	0	0	0	0	0	0	0	0
OR6C64P	0	0	0	0	0	0	0	0
OR6C66P	0	0	0	0	0	0	0	0
OR6C68	0	0	0	0	0	0	0	0
OR6C69P	0	0	0	0	0	0	0	0
OR6C70	0	0	0	0	0	0	0	0
OR6C71P	0	0	0	0	0	0	0	0

OR6C72P	0	0	0	0	0	0	0	0
OR6C73P	0	0	0	0	0	0	0	0
OR6C75	0	0	0	0	0	0	0	0
OR6C76	0	0	0	0	0	0	0	0
OR6C7P	0	0	0	0	0	0	0	0
OR6D1P	0	0	0	0	0	0	0	0
OR6E1P	0	0	0	0	0	0	0	0.174259
OR6F1	0	0	0	0	0	0	0	0
OR6J1	0	0	0	0	0	0	0	0
OR6K1P	0	0	0	0	0	0	0	0
OR6K2	0	0	0	0	0	0	0	0
OR6K3	0	0	0	0	0	0	0	0
OR6K4P	0	0	0	0	0	0	0	0
OR6K5P	0	0	0	0	0	0	0	0
OR6K6	0	0	0	0	0	0	0	0
OR6L1P	0	0	0	0	0	0	0	0
OR6L2P	0	0	0	0	0	0	0	0
OR6M2P	0	0	0	0	0	0	0	0
OR6M3P	0	0	0	0	0	0	0	0
OR6N1	0	0	0	0	0	0	0	0
OR6N2	0	0	0	0	0	0	0	0
OR6P1	0	0	0	0	0	0	0	0
OR6R1P	0	0	0	0	0	0	0	0
OR6R2P	0	0	0	0	0	0	0	0
OR6S1	0	0	0	0	0	0	0	0
OR6U2P	0	0	0	0	0	0	0	0
OR6V1	0	0	0	0	0	0	0	0
OR6W1P	0	0	0	0	0	0	0	0
OR6Y1	0	0	0	0	0	0	0.217916	0.03599
OR7A10	0	0	0	0	0	0	0	0
OR7A11P	0	0	0	0	0	0	0	0
OR7A15P	0	0	0	0	0	0	0	0
OR7A17	0	0	0	0	0	0	0	0

OR7A18P	0	0	0	0	0	0	0	0
OR7A19P	0	0	0	0	0	0	0	0
OR7A1P	0	0	0	0	0	0	0	0
OR7A2P	0	0	0	0	0	0	0	0
OR7A3P	0	0	0	0	0	0	0	0
OR7A5	0	0	0	0	0	0	0	0
OR7A8P	0	0	0	0	0	0	0	0
OR7C1	0	0	0	0	0	0	0	0
OR7C2	0	0	0	0	0	0	0	0
OR7D1P	0	0	0	0	0	0	0	0
OR7D2	0	0	0	0	0	0	0	0
OR7D4	0	0	0	0	0	0	0	0
OR7E100P	0	0	0	0.272972	0	0	0	0
OR7E101P	0	0	0	0	0	0	0	0
OR7E102P	0	0	0	0	0	0.183398	0	0
OR7E103P	0	0	0	0	0	0	0	0
OR7E104P	0	0	0	0	0	0	0	0
OR7E105P	0	0	0	0	0	0	0	0
OR7E106P	0	0	0	0	0	0	0	0
OR7E108P	0	0	0	0.093635	0	0	0	0
OR7E109P	0	0	0	0	0	0	0	0
OR7E10P	0	0	0	0	0	0	0	0
OR7E110P	0	0	6.952673	0	0	0	0	0
OR7E111FP	0	0	0	0	0	0	0	0
OR7E111P	0	0	0	0	0	0	0	0
OR7E115P	0	0	0	0	0	0	0	0
OR7E116P	0	0	0	0	0	0	0	0
OR7E117P	0	0	0	0	0	0	0	0
OR7E11P	0	0	0	0	0	0	0	0
OR7E121P	0	0	0	0	0	0	0	0
OR7E122P	0	0	0	0	0	0	0	0
OR7E125P	0	0	0	0	0	0	0	0
OR7E126P	0	0	0	0	0	0	0	0

OR7E128P	0	0	0	0	0	0	0	0
OR7E129P	0	0	0	0	0	0	0	0
OR7E12P	0	0	0	0	0	0	0	0
OR7E130P	0	0	0	0	0	0	0	0
OR7E136P	0	0	0	0	0	0	0	0
OR7E13P	0	0	0	0	0	0	0	0
OR7E140P	0	0	0	0	0	0	0	0
OR7E145P	0	0	0	0	0	0	0	0
OR7E148P	0	0	0	0	0	0	0	0
OR7E149P	0	0	0	0	0	0	0	0
OR7E14P	7.721526	0	3.406117	0	0	0	0	0
OR7E154P	0	0	0	0	0	0	0	0
OR7E155P	0	0	0	0	0	0	0	0
OR7E156P	0	0	0	0	0	0	0	0
OR7E157P	0	0	0	0	0	0	0	0
OR7E158P	0	0	0	0	0	0	0	0
OR7E159P	0	0	0	1.100763	0	0	0	0
OR7E15P	0	0	0	0	0	0	0	0
OR7E161P	0	0	0	0	0	0	0	0
OR7E162P	0	0	0	0	0	0	0	0
OR7E163P	0	0	0	0	0	0	0	0
OR7E16P	0	0	0	0	0	0	0	0
OR7E18P	0	0	0	0	0	0	0	0
OR7E19P	0	0	0	0	0	0	0	0
OR7E1P	0	0	0	0	0	0	0	0
OR7E21P	0	0	0	0	0	0	0	0
OR7E22P	0	0	0	0	0	0	0	0
OR7E23P	0	0	0	0	0	0	0	0
OR7E24	0	0	0	0	0	0	0	0
OR7E25P	0	0	0	0	0	0	0	0
OR7E26P	0	0	0	0	0	0	0	0
OR7E28P	0	0	0	0	0	0	0	0
OR7E29P	0	0	0	0	0	0	0	0

OR7E2P	0	0	0	0	0	0	0	0
OR7E31P	0	0	0	0	0	0	0	0
OR7E33P	0	0	0	0	0	0	0	0
OR7E35P	0	0	0	0	0	0	0	0
OR7E36P	0	0	0	0	0	0	0	0
OR7E37P	0	0	0	0	0	0	0	0
OR7E38P	11.799076	3.074542	6.936939	2.546651	0	0.94986	0	4.815281
OR7E39P	0	0	0	0	0	0	0	0
OR7E41P	0	0	0	0	0	0	0	0
OR7E43P	0	0	0	0	0	0	0	0
OR7E46P	0	0	0	0	0	0	0	0
OR7E47P	0	0	0	0	0	0	0	0
OR7E4P	0	0.939713	0	0.364821	0	0	0	0
OR7E53P	0	0	0	0	0	0	0	0
OR7E55P	0	0	0	0	0	0	0	0
OR7E59P	0	0	0	0	0	0	0	0
OR7E5P	0	0	0	0	0	0	0	0
OR7E62P	0	6.499614	9.093789	0.742432	22.128258	0	0	0.451431
OR7E66P	0	0	0	0	0	0	0	0
OR7E7P	0	0.783994	0	0.091313	0	0	0	0.310348
OR7E83P	0	0	0	0	0	0	0	0
OR7E84P	0	0	0	0	0	0	0	0
OR7E85BP	0	0	0	0	0	0	0	0
OR7E85P	0	0	0	0	0	0	0	0
OR7E86P	0	0	0	0	0	0	0	0
OR7E87P	0	0	0	0	0	0	0	0
OR7E89P	0	0	0	0	0	0	0	0
OR7E8P	0	0	0	0	0	0	0	0
OR7E90P	0	0	0	0	0	0	0	0
OR7E91P	4.618708	0	0	0	0	0	24.209047	0
OR7E93P	0	0	0	0	0	0	0	0
OR7E94P	0	0	0	0	0	0	0	0
OR7E96P	0	0	0	0	0	0	0	0

OR7E97P	0	0	0	0	0	0	0	0
OR7E99P	0	0	0	0	0	0	0	0
OR7G1	0	0	0	0	0	0	0	0
OR7G15P	0	0	0	0	0	0	0	0
OR7G2	0	0	0	0	0	0	0	0
OR7G3	0	0	0	0	0	0	0	0
OR7H1P	0	0	0	0	0	0	0	0
OR7H2P	0	0	0	0	0	0	0	0
OR7K1P	0	0	0	0	0	0	0	0
OR7L1P	0	0	0	0	0	0	0	0
OR7M1P	0	0	0	1.050257	0	1.651565	0	0
OR8A1	0	0	0	0	0	0	0	0
OR8A2P	0	0	0	0	0	0	0	0
OR8A3P	0	0	0	0	0	0	0	0
OR8B10P	0	0	0	0	0	0	0	0
OR8B12	0	0	0	0	0	0	0	0
OR8B1P	0	0	0	0	0	0	0	0
OR8B3	0	0	0	0	0	0	0	0
OR8B5P	0	0	0	0	0	0	0	0
OR8B6P	0	0	0	0	0	0	0	0
OR8B7P	0	0	0	0	0	0	0	0
OR8B8	0	0	0	0	0	0	0	0
OR8B9P	0	0	0	0.101765	0	0	0	0
OR8C1P	0	0	0	0	0	0	0	0
OR8D1	0	0	0	0	0	0	0	0
OR8D4	0	0	0	0	0	0	0	0
OR8F1P	0	0	0	0	0	0	0	0
OR8G1	0	0	0	0	0	0	0	0
OR8G3P	0	0	0	0	0	0	0	0
OR8G5	0	0	0	0	0	0	0	0
OR8G7P	0	0	0	0	0	0	0	0
OR8H1	0	0	0	0	0	0	0	0
OR8H2	0	0	0	0	0	0	0	0

OR8H3	0	0	0	0	0	0	0	0
OR8I1P	0	0	0	0	0	0	0	0
OR8I4P	0	0	0	0	0	0	0	0
OR8J1	0	0	0	0	0	0	0	0
OR8J2	0	0	0	0	0	0	0	0
OR8J3	0	0	0	0	0	0	0	0
OR8K1	0	0	0	0	0	0	0	0
OR8K2P	0	0	0	0	0	0	0	0
OR8K3	0	0	0	0	0	0	0	0
OR8K4P	0	0	0	0	0	0	0	0
OR8K5	0	0	0	0	0	0	0	0
OR8L1P	0	0	0	0	0	0	0	0
OR8Q1P	0	0	0	0	0	0	0	0
OR8R1P	0	0	0	0	0	0	0	0
OR8S1	0	0	0	0	0	0	0	0
OR8S21P	0	0	0	0	0	0	0	0
OR8T1P	0	0	0	0	0	0	0	0
OR8U1	0	0	0	0	0	0	0	0
OR8U3	0	0	0	0	0	0	0	0
OR8U8	0	0	0	0	0	0	0	0
OR8U9	0	0	0	0	0	0	0	0
OR8V1P	0	0	0	0	0	0	0	0
OR8X1P	0	0	0	0	0	0	0	0
OR9A1P	0	0	0	0	0	0	0	0
OR9A2	0	0	0	0	0	0	0	0
OR9A3P	0	0	0	0	0	0	0	0
OR9A4	0	0	0	0	0	0	0	0
OR9G1	0	0	0	0	0	0	0	0
OR9G2P	0	0	0	0	0	0	0	0
OR9G3P	0	0	0	0	0	0	0	0
OR9G4	0	0	0	0	0	0	0	0
OR9G9	0	0	0	0	0	0	0	0
OR9H1P	0	0	0	0	0	0	0	0

OR9I1	0	0	0	0	0	0	0	0
OR9I2P	0	0	0	0	0	0	0	0
OR9I3P	0	0	0	0	0	0	0	0
OR9K1P	0	0	0	0	0	0	0	0
OR9K2	0	0	0	0	0	0	0	0
OR9L1P	0	0	0	0	0	0	0	0
OR9M1P	0	0	0	0	0	0	0	0
OR9N1P	0	0	0	0	0	0	0	0
OR9P1P	0	0	0	0	0	0	0	0
OR9Q1	0	0	0	0	0	0	0	0
OR9Q2	0	0	0	0	0	0	0	0
OR9R1P	0	0	0	0	0	0	0	0
OR9S24P	0	0	0	0	0	0	0	0
ORAI1	9.676429	40.913018	19.714176	33.03599	9.537521	28.014007	0.390722	28.47627
ORAI2	0	4.087876	0	5.08986	0	9.983252	0	8.18646
ORAI3	7.800277	6.534795	8.365603	7.773227	6.859794	10.676523	0	7.359052
ORC1	53.651829	69.990985	40.389529	68.228578	26.907479	20.904602	27.640296	25.12316
ORC1P1	0	0	0	0	0	0	0	0
ORC2	15.358829	36.488685	24.741903	35.614844	46.793757	29.677439	76.576648	55.352777
ORC3	44.923389	4.749935	19.47127	2.436181	20.144129	4.066757	11.219568	16.38598
ORC4	0	52.156413	85.064015	30.350628	0	37.714534	41.601051	74.604935
ORC5	21.732485	10.490083	6.465135	13.536798	13.844211	6.407094	14.87584	16.53951
ORC6	40.375578	70.194066	49.497977	43.381008	43.439195	49.130361	52.364066	52.418904
ORM1	0	0	0	0	0	0	0	0
ORMDL1	69.027366	35.164439	36.974141	20.873281	20.820277	12.919137	99.900741	34.113363
ORMDL1P1	0	1.829669	0	0.562541	0	0	0	0
ORMDL2	98.064381	88.544368	67.437059	66.038072	11.8988	31.552017	11.226899	37.760285
ORMDL3	29.478963	44.812951	23.434954	65.083247	17.293116	35.174597	36.555457	37.635511
OS9	34.640275	35.744936	65.690497	47.746831	36.066333	25.654518	45.750552	13.382817
OSBP	32.70657	36.906737	48.749198	46.662359	67.77165	37.962657	42.705454	39.567968
OSBP2	2.759531	3.696682	8.490529	3.669077	0.97151	8.549648	0	11.673402
OSBPL10	29.498807	24.09641	0	14.083452	17.633497	64.954316	2.409187	44.652029
OSBPL11	12.138221	24.163364	9.618079	16.576365	17.72791	21.794202	22.428337	39.488131

OSBPL1A	49.474156	96.430388	78.675808	102.959262	51.765314	95.017252	102.96111	102.476379
OSBPL2	66.554097	116.806037	39.382897	152.541743	30.258284	145.544027	24.935673	111.467365
OSBPL3	20.269915	61.001403	26.762837	59.289166	22.329243	79.990597	126.10329	113.86945
OSBPL5	3.104641	2.593091	17.662751	3.835989	14.903422	10.791579	0	7.962682
OSBPL6	3.358604	7.643546	1.227079	6.257573	0	0.911387	0	2.530242
OSBPL7	4.469559	3.764117	1.624787	1.993486	0	5.896718	0	0.873225
OSBPL8	54.053749	115.878057	47.094509	95.518424	49.061461	76.361775	61.811065	128.594524
OSBPL9	134.340676	174.236373	130.865622	164.832961	143.102496	56.41489	58.175711	78.241386
OSBPL9P1	0	0	0	0	0	0	0	0
OSBPL9P2	0	0	0	0	0	0	0	0
OSBPL9P3	0	0	0	0	0	0	0	0
OSBPL9P4	0	0	0	0	0	0	0	0
OSBPL9P5	0	0	0	0	0	0	0	0
OSCAR	0	0.449902	0	0.186228	3.034624	0.99162	0	0.15501
OSCP1	5.264626	7.639342	7.015146	6.046677	25.359272	5.367117	0	6.491037
OSER1	26.405473	41.342182	39.485437	40.075757	47.991183	35.087662	22.603534	41.695272
OSGEP	12.309498	20.188312	21.633569	18.498584	51.065868	26.333681	20.825313	28.179041
OSGEPL1	5.234028	8.520233	5.284627	8.307315	19.247085	3.136003	11.30001	7.784909
OSGIN1	7.252496	11.36643	14.486749	16.902581	3.823472	14.041836	0	12.133914
OSGIN2	37.556198	35.775421	12.633438	11.811303	7.444565	6.704062	32.578252	20.54277
OSM	0	0	0	0	3.952639	3.796655	0	2.346396
OSMR	52.631537	75.023813	96.942389	109.413416	31.018293	31.692101	41.691234	47.889644
OSR1	1.829967	0.386755	1.633	2.487367	0.482359	0.724963	0	0.812586
OSR2	4.7084	2.560298	5.429328	5.177649	8.641084	16.555773	13.097212	9.454453
OST4	579.893946	584.162052	388.237261	507.012691	296.177008	403.67412	637.673283	545.237793
OSTC	75.353466	57.336672	120.376106	38.906779	55.622914	16.129983	24.074431	36.500284
OSTCP1	0	0	0	0	0	0	0	0
OSTCP2	0	0	0	0	0	0	0	0
OSTCP3	0	0	0	0	0	0	0	0
OSTCP4	0	0	0	0	0	0	0	0
OSTCP5	0	0	0	0	0	0	0	0
OSTCP6	0	0	0	0	0	0.514099	0	0
OSTCP8	0	0	0	0	0	0	0	0

OSTF1P1	0	0	0	0	0	0	0	0
OSTM1	23.006129	3.017865	20.353272	0.633604	4.582614	0.25667	0.297901	2.8928
OSTN	0	0	0	0	0	0	0	0
OTC	0	0	0	0	0	0	0	0
OTOA	0	0.216598	0	0.092904	0	0	0	0
OTOAP1	0	0	0	0	0	0	0	0
OTOF	0	0	0	0	0	0	0	0
OTOG	0	0	0	0	0	0	0	0
OTOGL	0	0	0	0	0	0	0	0
OTOL1	0	0	0	0	0	0	0	0
OTOP2	0	0	0	0	0	0	0	0.108167
OTOR	0	0	0	0	0	0	0	0
OTOS	0	0.317692	7.003934	0.380885	0	0	0	0.282884
OTP	0	0	0	0	0	0	0	0
OTUB1	0	17.948147	0	18.46631	69.624454	33.6584	78.787586	28.130353
OTUB2	5.688907	7.22187	7.663342	13.80491	1.786403	2.545901	0	3.984098
OTUD3	5.476094	6.239546	2.238069	1.595156	1.315454	1.148659	0	1.975926
OTUD4	0	33.179691	18.006829	36.005853	13.205604	24.705578	41.898657	68.233045
OTUD4P1	0	0	0	0.027831	0	0	0	0
OTUD5	17.928184	42.796937	4.132343	61.202134	21.082246	50.470567	9.392374	31.550339
OTUD6B	5.785421	1.420612	0	3.827625	2.407614	1.009238	36.814223	2.568851
OTUD7A	0	0.401709	0	0.02605	0	0.247427	0	0.206216
OTUD7B	11.542398	29.505632	12.982868	38.581479	10.582223	33.082405	12.603261	26.946031
OTULIN	44.770704	27.570429	9.328181	25.579367	7.615518	32.424265	0	18.772392
OTULINL	13.655294	12.475267	2.894006	3.981395	1.457777	0.461373	0	3.263758
OTX1	4.767716	4.300952	3.202459	4.450358	3.298808	3.55094	14.183327	2.353142
OTX2	0	0	0	0	0	0	0	0
OTX2P1	0	0	0	0	0	0	0	0
OTX2P2	0	0	0	0	0	0	0	0
OVCA2	31.865646	27.683901	25.421891	20.935666	88.525019	41.441212	0	39.965182
OVCH1	0	0	0	0	0	0	0	0
OVCH2	0	0	0	0.049413	0	0.301512	0	0.082368
OVGP1	0	3.144797	0	0	0	0	0	0

OVOL1	11.239258	11.986938	7.0488	13.320532	2.966413	8.509465	0	7.368496
OVOL2	15.634556	10.679333	0	9.759934	14.368608	12.91501	0	17.869727
OVOL3	0	0	0	0	0	0	0	0
OXA1L	75.489467	38.902636	89.258832	42.816037	51.499736	36.306492	98.633146	41.908781
OXCT1	32.927554	40.279722	37.679681	26.321837	27.286286	22.931044	15.765569	46.621341
OXCT2	0	3.145778	8.558398	1.800053	0	5.30622	0	3.685027
OXCT2P1	4.664693	5.306245	0	5.776665	4.299469	7.229609	0	5.423486
OXGR1	1.499922	0.382043	0	0.049169	0	0	0	0.060445
OXLD1	13.970456	60.973521	22.646152	61.665722	19.951606	33.888428	0	25.471258
OXNAD1	15.528437	38.483394	10.990416	38.408939	12.538557	17.05118	7.191827	28.491415
OXR1	26.363764	25.638188	35.070134	30.750038	14.458485	9.16702	0.606652	16.747659
OXSM	16.684458	24.193445	21.203154	17.947267	45.414021	10.42529	28.954952	27.397789
OXSR1	37.095326	57.094414	38.537322	56.33886	44.762855	49.687768	57.002385	57.01686
OXT	0	0	0	0	0	0	0	0
OXTR	0	2.799694	0	0.798338	0	1.955395	0	0
P2RX1	0	1.247766	0	0.321912	0	0	0	0
P2RX2	0	0	0	0	0	0	0	0
P2RX3	0	0	0	0	0	0	0	0
P2RX4	11.03309	18.291834	20.788636	7.615198	6.393586	0.93173	0	2.219298
P2RX5	6.483681	17.80731	12.124364	22.250664	12.355787	16.013767	85.854024	11.314316
P2RX5-TAX1	0	0.153493	0	0.19292	0	0.368034	0	0.151396
P2RX6	1.9098	0.115104	1.703472	1.364578	1.006655	0.256307	0	0.709021
P2RX6P	0	0	0	0	0	0	0	0
P2RX7	0	0	0	0.156272	5.141936	0.413639	0	0.256541
P2RY1	3.601982	9.449102	6.476724	5.878428	1.631581	2.735413	0	3.818707
P2RY10	0	0	0	0	0	0	0	0
P2RY10BP	0	0	0	0	0	0	0	0
P2RY11	0	3.483357	0	4.331429	0.517706	9.843084	0	11.875934
P2RY12	0	0	0	0	0	0	0	0
P2RY13	0	0	0	0	0	0	0	0
P2RY14	0	0	0	0	0	0	0	0
P2RY2	14.000689	21.323692	10.863073	14.485867	0	7.47682	0	9.71544
P2RY6	0	0	0	0	0	0	0	1.237802

P2RY8	0	0	0	0	0	0.038599	0	0
P3H1	0	0.804023	0	1.165291	24.543758	8.418878	37.648091	0.872237
P3H2	136.01048	341.917114	184.991118	488.046218	118.610658	114.4516	80.19324	84.477097
P3H3	7.727229	5.929736	27.255168	6.444054	4.272319	9.240493	1.781924	6.008546
P3H4	37.464998	34.629065	26.398012	45.133152	16.269185	57.773226	38.181315	35.509972
P3R3URF-PIK	0	3.629125	0	0	3.202038	0	0	4.272116
P4HA1	0	21.563406	46.474249	30.268641	45.453171	6.095442	0	23.933387
P4HA2	34.485585	27.777218	41.067788	38.011913	15.390471	21.946002	88.319196	32.893316
P4HA3	0	0.645321	0	0.830613	0	0.301841	0	0.184612
P4HB	184.090716	297.73749	251.378928	388.99863	236.23782	302.773067	0	206.009456
P4HTM	3.619588	8.716063	15.932884	12.01134	1.56969	9.073582	0	8.236921
PA2G4	250.44407	425.084382	171.570695	358.565585	127.438783	264.802956	180.198085	295.019306
PA2G4P1	0	0.388151	0	0	0	0	0	0
PA2G4P2	0	0	0	0	0	0	0	0.12601
PA2G4P3	0	0	0	0	0	0	0	0
PA2G4P4	0	0	0	0.306335	0	0.308659	0	1.031917
PA2G4P5	0	0	0	0	0	0	0	0
PA2G4P6	0	0	0	0	0	0	0	0
PAAF1	15.220302	27.857041	42.596982	28.220018	25.819964	18.170392	108.149718	20.068123
PABIR2	29.612782	52.554078	33.457037	56.427245	10.230943	31.746498	66.214844	50.597442
PABIR3	10.678978	5.535592	7.881741	14.185804	0.892306	9.703371	0	3.741151
PABPC1	1707.46051	929.215518	1669.74152	1228.03631	1206.94185	551.370486	1524.85213	846.704231
PABPC1L	1.644717	0.191566	0	1.298767	3.117653	0.626613	0	0.685274
PABPC1L2A	0	0	0	0	0	0	0	0
PABPC1P1	0	0	0	0	0	0	0	0
PABPC1P10	0	0	0	0	0	0	0	0
PABPC1P11	0	0	0	0	0	0	0	0
PABPC1P12	0	0	0	0	0	0	0	0
PABPC1P13	0	0	0	0	0	0	0	0
PABPC1P2	0	0	0	0	0	0	0	0
PABPC1P3	0	0	0	0	0	0	0	0
PABPC1P4	0	0	1.662614	0.135668	0	0	0	0
PABPC1P5	0	0	0	0	0	0	0	0

PABPC1P6	0	0	0	0	0	0	0	0
PABPC1P7	0	0	0	0	0	0	0	0
PABPC1P8	0	0	0	0	0	0	0	0
PABPC1P9	0	0	0	0	0	0	0	0
PABPC4	114.000105	121.100744	111.695298	129.956496	260.945501	208.35841	128.64853	362.75177
PABPC4L	4.595238	3.099609	0	2.69509	0	0.525913	3.198028	0.948412
PABPC5	0	0.534441	0	1.071531	0	0	0	0
PABPN1	51.074505	44.759698	4.013091	51.809897	7.321899	22.681035	150.97979	15.388229
PABPN1L	0	0	0	0.112252	0	0	0	0
PABPN1P1	0	0	0	0	0	0	0	0
PABPN1P2	0	0	0	0	0	0	0	0
PACC1	11.436498	8.642223	13.094434	16.211197	0	21.905506	31.561841	10.436915
PACRG	0	1.426441	0	1.323344	0	0	0	0
PACRGL	24.192543	60.371359	8.395975	56.320407	2.276869	32.764945	76.802033	51.415557
PACS1	70.541858	69.704731	113.753315	70.733506	85.240751	54.18165	33.061522	67.179507
PACS2	5.164869	13.365736	8.023028	13.049942	4.74017	24.294583	27.002433	14.266619
PACSIN1	0	0.400681	0	1.88721	0	0.593858	0	0.722446
PACSIN2	39.860212	54.203665	23.949567	62.506704	26.490833	70.459057	16.129988	52.739759
PACSIN3	18.657124	50.728907	33.969913	47.257149	42.782103	133.477006	85.7603	88.838143
PADI1	0	2.699204	0	1.308951	2.428951	4.562859	0	3.911115
PADI2	0	0.194073	3.943488	1.087254	3.499611	2.613248	0	1.151025
PADI3	1.048758	1.343557	0.940027	0.639267	0	0.261415	0	0.421659
PADI4	0	0.367099	0	0.036788	0	0	0	0
PADI6	0	0	0	0	0	0	0	0
PAEP	0	0	0	0.093522	0	0	0	0
PAEPP1	0	0	0	0	0	0	0	0
PAF1	25.785506	43.055905	11.176069	50.487366	73.231243	103.123978	93.23454	67.641769
PAFAH1B1	89.315192	281.315973	143.178466	257.055916	121.00678	333.945634	37.95936	502.580618
PAFAH1B1P1	0	0	0	0	0	0	0	0
PAFAH1B1P2	0	0	0	0	0	0	0	0
PAFAH1B2	71.861107	205.535378	68.04995	175.626354	115.955142	243.20863	168.368132	323.876426
PAFAH1B2P1	0	0	0	0	0	0	0	0
PAFAH1B2P2	0	0	0	0	0	0	0	0

PAFAH1B3	30.380216	15.273121	7.794645	22.804631	8.463126	29.915712	51.294707	27.604064
PAFAH2	5.724898	4.641118	6.027987	4.850559	2.519109	5.230587	35.73091	5.627872
PAG1	7.16323	17.793741	5.910701	22.845306	9.151949	17.801525	0	16.688895
PAGE1	0	0	0	0	0	0	0	0
PAGE2	0	0	0	0	0	0	0	0
PAGE2B	0	0	0	0	0	0	0	0
PAGE3	0	0	0	0	0	0	0	0
PAGE4	0	0	0	0	0	0	0	0
PAGE4P1	0	0	0	0	0	0	0	0
PAGE5	0	0	0	0	0	0	0	0
PAGR1	17.935917	34.899924	11.495837	33.015673	28.868563	34.921069	2.808191	35.889632
PAH	0	0	0	0	0	0	0	0
PAICS	224.884122	246.635408	219.454443	212.498072	177.162184	108.945752	171.040202	202.56706
PAICSP1	0	0	0	0	0	0	0	0
PAICSP2	0	0	0	0	0	0	0	0
PAICSP3	0	0	0	0	0	0	0	0
PAICSP4	0	0	0	0	0	0	0	0
PAICSP5	0	0	0	0	0	0	0	0
PAICSP6	0	0	0	0	0	0	0	0
PAICSP7	0	0	0	0	0	0	0	0
PAIP1	176.03869	160.883653	100.134469	74.223634	110.573975	46.43535	83.566863	141.545043
PAIP1P1	0	0	0	0	0	0	0	0.125633
PAIP1P2	0	0	0	0	0	0	0	0
PAIP2	128.812066	394.984224	168.384386	294.218684	77.726694	174.047917	62.113835	269.84295
PAIP2B	2.57663	7.69	5.096321	8.159048	0.27233	2.765259	0	3.367953
PAK1	30.452174	158.565823	45.789322	100.07095	55.863373	29.062475	63.282325	18.848921
PAK1IP1	14.119453	48.509307	14.656325	37.189894	37.181331	40.356817	67.564127	65.248916
PAK2	64.04015	129.429693	58.997856	72.559929	72.578256	106.997983	104.884606	166.712876
PAK3	0	3.057932	0.389809	1.10061	0	0.665671	0	3.985435
PAK4	20.968509	34.829972	38.78569	46.993758	55.016642	84.053772	20.363749	57.720147
PAK5	0	0	0	0	0	0	0	0
PAK6	0	5.622523	4.998722	7.373949	4.371907	18.025083	6.166771	18.238296
PALB2	27.306722	0	0	0	13.4342	0	0	0

PALD1	0	0.213801	0.639474	0.313122	0	0.285057	0	0.05716
PALLD	42.814386	39.692343	36.740635	45.387566	43.177125	87.494196	35.191168	70.496193
PALM	23.128962	8.488092	18.062538	8.681026	17.179539	13.665802	13.536992	15.592181
PALM2AKAP2	120.242868	213.608786	82.848182	200.67132	157.48928	324.372897	110.850753	257.287962
PALM3	0	0.708677	0	1.254975	2.402122	0.301126	0	0.244715
PALMD	0	13.53099	0	8.697096	0	2.471626	0	2.48042
PALS1	0	26.679109	26.689139	10.251438	0	24.033217	14.16948	18.096442
PALS2	83.444657	101.768067	66.278407	54.654812	53.999773	49.741653	126.409362	78.920627
PAM	26.97796	61.114742	35.267563	96.076322	11.650426	57.276829	42.713537	43.660723
PAM16	40.573824	61.620796	48.991447	66.197286	38.600299	54.32103	19.681341	55.034422
PAMR1	0	0	0	0	0	0.239718	0	0.339186
PAN2	0	4.098643	5.659757	7.029285	5.946795	8.012079	92.569013	5.472233
PAN3	14.140611	19.790434	5.717872	21.470552	4.551323	13.307903	2.421128	21.026175
PANK1	1.978336	0	4.079886	4.368981	0.713959	1.173393	0	3.329935
PANK2	27.055859	60.576357	46.675213	42.767007	41.099794	32.62952	23.502573	43.081752
PANK3	27.800951	25.612535	49.842288	20.271113	23.449562	10.36305	21.04095	49.165863
PANK4	18.309944	5.074801	0	4.346001	42.062885	9.021038	0	6.410518
PANX1	98.040319	187.133941	87.809824	152.729192	40.254432	69.927545	54.569172	113.833018
PANX2	0	0	0	0.166675	2.692768	0.15059	0	1.533252
PAOX	0	0	0	0.140983	0	0.624215	0	0
PAPLN	17.710324	3.853087	4.142568	1.579752	1.065418	0	0	1.244824
PAPOLA	73.29168	334.65703	152.812768	323.695978	163.426724	318.86021	289.012062	409.54572
PAPOLB	0	0	0	0	0	0	0	0
PAPOLG	8.64311	25.575587	5.144107	17.11033	19.306348	12.150376	51.346022	14.277935
PAPPA	4.090621	6.563319	8.41677	8.128726	0.077237	3.042961	0	0.631909
PAPPA2	0.995413	1.841995	0.298619	0.251835	0	0	0	0.013291
PAPSS1	27.718187	43.661573	50.208172	41.800363	43.876025	16.777157	85.990825	24.384909
PAPSS2	2.689826	4.98741	5.630424	11.641756	10.890242	9.565614	0	4.90832
PAQR3	20.244933	18.304498	21.997161	7.118332	5.745163	2.144527	6.73384	18.685978
PAQR4	13.891032	58.966713	0	57.563579	11.660654	79.377067	17.738359	34.542125
PAQR5	2.649022	6.835446	16.232063	2.808725	8.747034	3.526572	4.273945	3.908954
PAQR6	0	0	0	0	0	0.109578	0	0
PAQR8	1.413933	1.819553	1.904708	1.394176	0	0.636811	0	0.851223

PAQR9	0	0	0	0	0	0	0	0
PARD3	20.030681	51.102956	28.345261	49.961682	18.452632	38.969424	28.32068	42.868029
PARD3B	0	0.268235	0	0.213805	0	0.055432	0	0.091202
PARD6A	0	6.414365	2.826802	0.171666	17.625339	2.820725	18.397759	0.881144
PARD6B	7.888094	6.520364	10.303451	10.752376	38.350765	4.772227	10.651307	15.319372
PARD6BP1	0	0	0	0	0	0	0	0
PARD6G	24.388794	14.578055	4.645942	10.196251	8.203622	21.901492	12.597576	22.56284
PARG	10.233221	12.544748	12.798615	16.24079	33.765403	11.932487	21.976981	19.839437
PARGP1	4.285069	5.002443	0	3.044524	0	1.718766	0	2.43875
PARK7	308.01371	430.838047	201.109496	378.399437	323.562335	420.053418	326.517509	467.820761
PARK7P1	0	0	0	0	0	0	0	0
PARK7P2	0	0	0	0	0	0	0	0
PARL	43.444212	30.115531	0	39.996387	2.085165	40.322704	193.009642	25.292285
PARLP1	0	0	0	0	0	0	0	0
PARLP2	0	0.684855	0	0.158971	0	0.481829	0	0.537345
PARM1	0.652927	0.476473	1.173104	0.558465	0	0	0	0
PARN	38.506855	66.77504	34.237171	54.730537	172.997765	47.84462	127.566492	69.158838
PARP1	175.542167	556.370174	149.096507	547.089105	152.238269	544.583662	209.682774	391.293555
PARP10	9.593773	14.65802	2.97974	14.434131	13.201209	29.703341	29.050034	12.448324
PARP11	2.297666	12.107657	12.413931	18.159503	7.079712	10.159035	18.457592	17.85681
PARP12	6.592906	5.238463	10.620611	7.656891	19.318518	19.810765	71.523079	29.579363
PARP14	43.397103	22.513576	26.048494	27.842737	58.782919	67.488823	46.574632	51.247421
PARP15	0	0	0	0.036183	0	0	0	0
PARP16	0	4.815894	0	3.975938	0	3.448922	0	3.635949
PARP1P1	0	0.047348	0	0	0	0.110562	0	0
PARP1P2	0	0	0	0	0	0	0	0
PARP2	8.861433	7.671775	0	5.188862	0	0	64.662414	0
PARP3	0	0.391619	0	4.615614	0	1.938823	0	0
PARP4	29.267343	23.350388	35.927022	24.763081	14.899804	13.316794	8.040459	20.81797
PARP4P1	0	0	0	0	0	0	0	0
PARP4P2	0	0	0	0	0	0	0	0
PARP4P3	0	0	0	0	0	0	0	0
PARP6	13.34219	13.033345	24.539989	19.552693	57.155938	22.396838	8.950825	18.570808

PARP8	3.93835	52.732809	29.49652	44.434362	43.188243	30.712241	22.18085	47.75758
PARP9	14.069434	36.199204	10.966847	22.752217	57.176454	76.657203	65.217504	87.403849
PARPBP	14.636921	7.166988	11.03974	2.350828	27.818302	6.844162	13.609106	10.166107
PARS2	18.861706	31.43334	20.760225	30.928991	20.283586	8.064929	12.665994	6.663878
PARVA	0	7.841628	0	4.709764	10.753072	19.556265	0	15.470759
PARVB	19.983643	25.308306	0	24.199158	2.465924	16.681256	0	9.578739
PARVG	0	0	0	0.023453	0	0	0	0
PASD1	0	0	0	0	0	0	0	0
PASK	1.2665	9.156283	0	4.885584	36.622387	16.646382	26.208832	11.602641
PATE1	0	0	0	0	0	0	0	0
PATE2	0	0	0	0	0	0	0	0
PATE4	0	0	0	0	0	0	0	0
PATJ	0	28.067582	38.375195	49.383555	44.943982	42.152729	166.985273	24.337074
PATL1	47.933371	54.250525	54.498135	53.743168	79.527965	51.615929	35.776498	56.98563
PATL2	0	0	0	0.697271	0	1.246079	0	0
PATZ1	8.211974	30.134345	16.189669	36.732231	4.781574	39.316922	13.023789	33.258216
PAWR	64.893916	112.04313	39.800398	109.3052	93.792542	136.862656	197.229568	115.402611
PAWRP1	0	0	0	0	0	0	0	0
PAX1	0	0.607381	0	1.04868	0	0	0	0
PAX2	0	0.710716	0	0.128519	0	0.104428	0	0.084219
PAX3	0	0	0	0	0	0	0	0
PAX4	0	0	0	0	0	0	0	0
PAX5	0	0.911877	0	1.134556	0.100508	0.305601	0	0.137134
PAX6	9.735067	55.443303	12.902072	47.96244	8.322988	44.437812	0	37.639958
PAX7	0	0.237511	0	0.294051	0	0	0	0
PAX8	0	0.508169	0	0.56993	2.171399	4.098235	52.421797	2.372811
PAX9	21.375156	12.013007	0	21.861574	3.734164	13.04671	0	5.37404
PAXBP1	11.013799	19.673087	6.10607	17.658197	4.360263	23.849292	1.238657	31.385494
PAXBP1P1	0	0	0	0	0	0	0	0
PAXIP1	1.373278	2.970565	8.174577	7.028397	0	5.320326	0	7.009049
PAXX	59.904868	50.225822	29.475409	41.445778	41.866858	36.757235	1.199775	28.344557
PBDC1	0	0	0	0.393836	32.195083	1.915504	0	13.365087
PBK	22.889865	42.502614	26.421192	17.303931	24.130586	24.269283	70.615761	31.425978

PBLD	4.09593	5.021948	7.20484	2.779201	13.423757	2.874562	13.876043	5.185467
PBOV1	0	0	0	0	0	0	0	0
PBRM1	40.638728	52.355017	29.611763	43.871998	41.21951	37.724396	39.914931	54.88778
PBX1	25.425129	21.831119	12.754626	19.427694	4.593908	31.414325	62.488976	22.26961
PBX2	40.368127	39.498941	25.272056	35.864869	32.251114	45.520304	49.268282	30.971911
PBX2P1	0	0	0	0.075276	0	0	0	0
PBX3	13.512606	8.884638	26.103221	8.578374	30.750935	6.322938	119.841561	11.111939
PBX4	9.090233	0.152933	0	0.930332	0.673238	1.338819	0	2.540642
PBXIP1	14.538626	5.164581	20.641944	16.523289	1.686243	12.230948	25.033953	5.539568
PC	3.41359	7.569953	6.891927	7.265386	23.763044	8.407739	42.630455	3.303024
PCARE	0	0	0	0.010832	0	0	0	0
PCAT6	0	0	0	0	0	0	0	0
PCBD1	114.914013	117.318115	114.322001	105.593354	47.880099	54.882883	79.380712	54.647666
PCBD2	0	1.033239	0.562046	3.102644	16.027738	0.20428	0	0.789021
PCBP2	586.686548	547.904658	524.091398	643.090629	163.999214	412.394804	271.572888	411.624502
PCBP2P1	0	0	0	0	0	0	0	0
PCBP2P2	0	0	0	0	0	0.188935	17.316231	0
PCBP2P3	0	0	0	0	0	0	0	0
PCBP2P4	0	0	0	0	0	0	0	0
PCBP3	0	2.378389	0	0.96905	0	0.696667	0	0.200273
PCBP4	1.908644	7.399251	12.642675	5.594884	29.982393	14.181252	0	10.084622
PCCA	19.748974	8.545416	39.256052	9.416141	4.183871	2.177756	7.988108	2.265397
PCCB	65.143268	52.100592	61.108034	62.812696	43.1998	44.533795	48.757736	47.895886
PCDH1	15.964115	59.423692	45.18627	89.869996	35.954842	79.068245	27.475567	32.985067
PCDH10	1.435832	5.205499	3.421876	11.595187	0	0.143688	0	0.028815
PCDH11X	0	0	0	0	0	0	0	0.027598
PCDH11Y	0	0	0	0	0	0	0	0
PCDH12	0	0	0	0	0	1.124844	0	0
PCDH15	0	0.416216	0	0.092715	0	0	0	0.066971
PCDH17	0	0	0	0.052429	0	1.112707	0	0.11804
PCDH18	0	1.612568	0	0.388625	35.468823	4.097024	0	0.925619
PCDH19	0	0.184538	0	0.056433	0	0.215118	0	0.079139
PCDH7	13.551421	61.583446	10.465484	76.741259	19.036604	148.298378	57.647357	155.118516

PCDH8	0	0	0	0	0	0	0	0
PCDH8P1	0	0	0	0	0	0	0	0
PCDH9	1.289559	1.012995	0	0.753671	0	0.052308	0	0.12935
PCDHA1	0	0.105617	0	0	0	0	0	0
PCDHA10	0	0	0	0.019024	0	0	0	0
PCDHA11	0	0	0	0.484436	0	0	0	0.078331
PCDHA12	0	0	0	0.022305	0	0	0	0.15833
PCDHA13	1.404982	0	0	0.39207	0	0	0	0
PCDHA14	0	0	0	0	0	0	0	0
PCDHA2	0	0	0	0.219947	0	0	0	0
PCDHA3	0	0	0	0.07895	0	0	0	0.110884
PCDHA4	0	0	0	0.029762	0	0	0	0
PCDHA5	0	0	0	0	0	0	0	0
PCDHA6	0	0	0	0	0	0	0	0
PCDHA7	0	0	0	0	0	0	0	0.033178
PCDHA8	0	0	0	0	0	0	0	0
PCDHA9	0	0	0	0	0	0	0	0
PCDHAC1	0	0	0.726614	0.237191	0	0.40466	0	0
PCDHAC2	10.81558	3.579975	0	0.922928	0.294233	2.677999	0	2.420183
PCDHB11	0	0	0	0.042771	0	0	0	0
PCDHB12	0	0	0.770754	0.083866	0	0	0	0
PCDHB13	0	0	0	0	0	0	0	0.181499
PCDHB14	0	0	0	0.259472	1.523145	0.143303	5.951275	0
PCDHB15	0	0	0	0	0	0	0	0
PCDHB16	0	0	0	0	0	0	0	0
PCDHB17P	0	0	0	0	0	0	0	0
PCDHB18P	0	0	0	0	0	0	0	0
PCDHB19P	0	0	0	0	0	0	0	0
PCDHB2	0	3.616762	0	3.422874	0	0	0	0
PCDHB3	0	0	0.891101	0.158298	0	0	0	0
PCDHB4	0	0.715892	0	0	0	0	0	0
PCDHB5	0	0	0	0.623187	0	0	0	0.529702
PCDHB6	0	0	0	0.063305	0	0	0	0

PCDHB9	0	0	0	2.348189	0	0	0	0
PCDHGA1	0	0	0	0	0	0.578594	0	0.879552
PCDHGA10	0	0.667098	0	0.238827	0	0.36357	0	1.150801
PCDHGA11	0	0.410492	0	0	0	0.37125	0	1.140121
PCDHGA12	0	0	0	0.049471	0.253026	0.363062	0	0
PCDHGA2	0	0	0	0	0	0.045072	0	0.132478
PCDHGA3	0	0	0	0	0	0	0	0
PCDHGA4	0	0	0	0.218888	0	0.750263	0	0.326057
PCDHGA5	0	0	0	0	0	0.206122	0	0
PCDHGA6	0	0.098823	0	0	0	0.606831	0	0.231265
PCDHGA7	1.023888	1.19323	0.918805	1.491253	5.797715	1.583896	0	0.860213
PCDHGA8	0	0	0	0.036733	0	0.125544	0	0.08028
PCDHGA9	0	0	0	0	0	0	0	0
PCDHGB1	1.308726	0	0	0.371839	2.071789	0.752239	0	1.001081
PCDHGB2	0	0	0	0	0	0	0	0
PCDHGB3	0	0	0	0.363422	0	0.542239	0	0.670384
PCDHGB4	0	0	0	0	0	0	0	0
PCDHGB5	0	0	0	0	3.679406	1.94985	0.307718	0.434527
PCDHGB6	0	0	0	0.101088	0	0	0	0.114733
PCDHGB7	0	0	0	0.035216	0	0.602932	0	0.390041
PCDHGB8P	0	0	0	0	0	0	0	0
PCDHGB9P	0	0.063319	0	0	0	0.147971	0	0.0601
PCDHGC3	7.269677	24.675076	9.07259	26.614876	7.430592	21.338597	41.830891	14.970352
PCDHGC4	0	0	0	0	0	0	0	1.977663
PCDHGC5	0	0	0	0	0	0	0	0
PCED1A	7.022246	18.63762	37.307888	26.2305	25.786303	24.312732	20.141911	16.919353
PCED1B	5.92915	12.973295	1.675355	12.759241	10.698551	11.993738	7.72305	7.06805
PCED1B-AS1	19.20491	7.46392	15.797443	7.809852	0	0	0	0
PCED1CP	0	0	0	0	0	0	0	0
PCF11	63.544046	50.832508	47.56122	40.73486	57.659768	27.169729	70.6721	45.65957
PCGF1	41.049523	30.323118	38.302767	40.741784	16.427651	33.414855	0	23.960753
PCGF2	53.028897	36.322728	54.967257	39.745019	39.664461	34.449502	39.303056	27.996318
PCGF3	1.442943	28.915188	5.942753	51.075915	16.071353	63.415377	0	31.244391

PCGF5	11.351356	21.387319	20.642626	21.793934	29.502778	31.164604	35.10468	34.545497
PCGF6	14.275067	8.873996	8.236305	4.614603	29.161575	3.350464	5.885045	9.59448
PCGF7P	0	0	0	0	0	0	0	0
PCID2	15.348208	30.761846	13.788173	18.651421	3.545484	17.649441	35.149272	48.917378
PCIF1	23.90309	40.935719	20.273188	42.031949	7.150825	44.788131	14.63689	35.062519
PCK1	0	0	0	0	0	0	0	0
PCK2	8.181396	9.850973	11.669672	12.917759	53.892754	8.982787	21.206079	2.536732
PCLAF	15.53024	7.374031	13.19839	3.777541	39.454157	8.681853	0.455416	33.15725
PCLO	3.964253	4.795785	2.510465	2.035021	3.733597	1.524501	18.496908	2.008929
PCM1	43.159476	59.710684	33.594232	90.404249	60.119762	56.976159	127.792483	73.75913
PCMT1	80.297597	11.508677	23.027752	8.811665	45.92567	6.727002	96.6269	7.830106
PCMTD1	31.055403	23.699859	14.655929	22.298006	18.77028	7.053731	25.429275	16.921807
PCMTD1P1	0	0	0	0	0	0	0	0
PCMTD1P2	0	0	0	0	0	0	0	0
PCMTD1P3	0	0	0	0	0	0	0	0
PCMTD1P7	0	0	0	0	0	0	0	0
PCMTD2	55.269414	85.949212	43.691533	99.563224	22.664183	68.524481	23.385605	78.775853
PCNA	262.564704	403.826979	235.473975	250.222602	384.597303	289.783662	488.629994	564.889445
PCNAP1	0	0	0	0	0	0	0	0
PCNAP3	0	0	0	0	0	0	0	0
PCNAP4	0	0	0	0	0	0	0	0
PCNP	157.657699	137.622889	141.519998	154.682568	28.185728	92.661706	71.168193	120.770183
PCNPP1	0	0.369079	0	0.893379	0	2.189362	0	2.419774
PCNPP2	0	0	0	0	0	0	0	0
PCNPP3	0	2.894855	0	9.334131	0	1.475711	0	1.84433
PCNPP4	0	0	0	0	0	0	0	0
PCNPP5	0	0	0	0	0	0	0	0
PCNT	4.571387	7.874412	19.492213	12.151006	27.756361	44.323855	66.073874	27.594013
PCNX1	12.438567	26.542144	19.110524	30.520642	66.17608	57.514257	14.518228	39.039302
PCNX2	9.823687	7.154132	8.700031	6.314793	3.971258	3.795929	88.628474	3.354264
PCNX3	35.678569	6.306784	7.766873	2.079433	5.621351	0.559527	18.406193	0.280188
PCNX4	27.770238	86.204722	41.548319	49.906931	43.924614	61.530103	52.200156	131.976583
PCOLCE	0	4.551814	3.577622	8.370745	10.656541	3.443002	0	1.677693

PCOLCE2	20.299953	19.885916	18.486985	11.626499	47.524293	11.802825	0	19.027542
PCP2	0	0	0	0	0	0.514099	0	0
PCP4	0	0	0	0	0	0	0	0
PCP4L1	0	1.187329	0	1.495598	0	0.379173	0	0.417963
PCSK1	0	0.71777	0.571801	0.946668	0	0.357838	0	0.295123
PCSK1N	11.238153	12.050331	16.535168	10.881803	0	4.170051	0	4.429166
PCSK2	0	0	0	0	0	0	0	0
PCSK4	0	0	0	0.062461	0	0.143886	0	1.118095
PCSK5	1.184315	4.494451	2.121427	5.012326	0	0.54566	0	0.283792
PCSK6	5.484559	7.132444	9.148465	9.968161	34.843158	36.46607	7.493214	19.043307
PCSK7	9.275863	10.192343	0.309548	1.060826	0.454582	2.057855	4.448425	3.696444
PCSK9	83.056229	105.115734	94.059387	107.831807	45.06467	51.958281	26.639536	26.193563
PCTP	7.53871	20.247078	7.912237	7.90042	1.986918	0	0	1.90036
PCYOX1	26.900984	19.027232	23.073279	25.586765	70.675588	22.216875	47.57961	17.747582
PCYOX1L	4.419841	7.233633	6.586805	7.065804	16.612863	2.494708	0	2.406794
PCYT1A	29.389261	38.697224	35.405907	44.422231	108.319069	42.414304	161.534808	46.856632
PCYT1B	14.082325	19.839972	28.556808	15.762299	16.06876	4.87284	38.844128	5.075197
PCYT2	9.283896	34.149776	8.337023	19.044771	14.130493	42.500126	71.506291	27.414149
PDAP1	134.188531	380.767878	131.193228	408.393302	340.548747	578.011497	121.89783	351.660614
PDC	3.182481	0	0	0	0	0	0	0
PDCD1	0	0	0	0	0	0	0	0
PDCD10	166.763481	256.353916	141.891269	180.89028	94.389915	127.906038	41.728183	329.764512
PDCD11	29.549123	43.730927	28.827967	54.159905	40.731803	44.622047	40.537283	27.695398
PDCD2	56.425594	83.541944	79.159864	55.638424	107.806953	65.110374	49.817905	100.186527
PDCD2L	287.745002	29.363978	19.886839	17.067886	28.354587	30.256335	137.262272	50.788135
PDCD4	134.927311	211.267877	76.876923	205.883004	187.382521	174.98332	176.196884	175.735673
PDCD5	218.063353	300.419654	123.515983	185.796986	311.326765	254.476407	426.317586	364.422473
PDCD5P1	0	0	0	0	0	0	0	0
PDCD5P2	0	0	0	0	0	0	0	0
PDCD6	99.67071	112.787693	110.170882	103.653556	67.488587	88.523303	292.33909	109.578488
PDCD6IP	60.342758	124.401816	99.001793	83.919461	90.799047	80.810932	112.08217	100.038971
PDCD6IPP1	0	0	0	0	0	0	0	0
PDCD6IPP2	0	0	0	0	0	0	0	0

PDCD7	27.466301	15.422157	18.181415	13.149605	17.963241	10.580492	5.220005	12.636845
PDCL	0	2.941587	0	8.40245	0	16.860594	0	2.801011
PDCL2	0	0	0	0	0	0	0	0
PDCL2P1	0	0	0	0	0	0	0	0
PDCL2P2	0	0	0	0	0	0	0	0
PDCL3	22.134322	30.208151	19.546527	30.419142	48.499285	58.181825	47.706747	71.263965
PDCL3P1	0	0	0	0	0	0	0	0
PDCL3P2	0	0	0	0	0	0	0	0
PDCL3P3	0	0	0	0	0	0	0	0
PDCL3P4	0	0	0	0.299183	0	0	0	0.746214
PDCL3P5	0	0	0	0	0	0	0	0
PDCL3P6	0	1.208887	0	0.142959	0	0.569876	0	0
PDCL3P7	0	0	0	0	0	0	0	0
PDE10A	0	0	0	0.022971	0	0	0	0
PDE11A	0	0	7.021211	0.071377	0	0.06945	0	0
PDE12	10.976783	5.466967	7.966462	6.208112	13.729503	4.406088	12.869086	8.413238
PDE1A	0	0	0	0	0	0	0	0
PDE1B	0	0	0	0.200082	0	0	0	0
PDE1C	0	0.422893	0	0.350287	0	0	0	0
PDE2A	3.818666	5.902546	7.851406	5.464177	0	4.137768	0	5.297148
PDE3A	0	0	0	0	0	0	0	0
PDE3B	0	0.038735	0	0.022093	0	0.081575	0	0.109122
PDE4A	9.362063	10.847672	10.211635	14.402607	6.359252	8.367127	0	5.684558
PDE4B	0	1.090636	0	0.141805	0	1.809213	0	0.772891
PDE4C	0	1.463941	0	0	0	0.33211	0	0.198039
PDE4D	0	3.60939	4.114807	4.637834	4.77586	3.538379	0	2.302036
PDE4DIP	40.414486	77.490138	24.260485	68.581074	24.841549	65.627936	31.979312	80.61727
PDE4DIPP1	0	0	0	0.619513	0	0.534625	0	0
PDE4DIPP10	0	0	0	0	0	0	0	0
PDE4DIPP2	1.018669	1.408538	0	1.351872	0.734275	1.72394	8.039491	0.902551
PDE4DIPP3	0	0	0	0	0	0	0	0
PDE4DIPP4	0	0	0	0	0	0	0	0
PDE4DIPP5	0	0.399235	0	0	0	0	0	0

PDE4DIPP6	0	0	0	0.253095	0	0.340481	0	0
PDE4DIPP7	0	0	0	0	0	0	0	0
PDE4DIPP8	0	0	0	0	0	0	0	0
PDE4DIPP9	0	0	0	0	0	0	0	0
PDE5A	15.301207	6.786877	16.507148	8.984212	35.156931	7.534649	3.976931	4.894669
PDE6A	0	0	0	0	0	0	0	0
PDE6B	0	19.429998	14.96411	8.026497	0	15.955724	0	8.905048
PDE6C	0	0	0	0	0	0	0	0
PDE6D	32.956179	52.148243	24.34546	43.547344	51.980458	68.352708	17.401228	61.609867
PDE6G	0	0	0	0	0	0	0	0.48341
PDE6H	0	0	0	0.275232	0	0	0	0
PDE7A	22.194263	35.657896	26.701362	19.448048	0	2.102454	0	9.206006
PDE7B	0	0	0	0	0	0	0	0
PDE8A	13.090805	20.694301	16.246129	38.269246	33.746247	45.243334	13.064206	65.886368
PDE8B	0	0	0	0	0	0	0	0
PDE9A	3.709468	6.911225	18.450343	5.170741	10.661641	7.822136	31.716657	5.323665
PDF	8.763838	16.043563	23.092666	13.758662	0	27.066599	0	25.734044
PDGFA	0	41.267999	3.813036	47.78944	37.412246	99.917001	61.635462	41.175309
PDGFB	10.863926	9.040271	22.021371	10.085299	0	14.311638	0	8.886774
PDGFC	23.94847	96.496962	12.087941	68.525582	4.82604	17.028008	45.965789	29.51664
PDGFD	1.72501	2.769152	2.193864	3.422816	0	0.746985	0	1.27607
PDGFRA	1.526511	2.216016	0.91497	1.656814	0	0	0	0
PDGFRB	0	5.190431	2.054119	5.489519	0	0.779892	2.088643	1.351845
PDGFRL	0	0.360394	0	0.161907	0	0	0	0
PDGFRL2P	0	0	0	0	0	0	0	0
PDHA1	137.268623	104.522887	33.136756	78.856873	50.177066	143.581029	184.531889	139.108094
PDHA1P1	0	0	0	0	0	0	0	0
PDHB	46.266003	42.636515	23.821072	43.092018	48.544752	25.03738	28.16404	43.870743
PDHX	0	22.326509	0	25.418021	0	32.240032	0	39.092047
PDIA2	0	0	0	0.054846	0	0	0	0
PDIA3	428.095655	461.232501	560.578655	488.059056	362.891767	225.859535	239.829795	226.140961
PDIA3P1	0	6.869445	3.086178	5.693823	2.228852	1.463684	0	4.516365
PDIA3P2	0	0	0	0	0	0	0	0

PDIA4	157.012026	288.519737	205.393352	398.855391	100.682086	257.988535	93.315714	151.390957
PDIA5	65.442884	53.906093	70.798899	131.834897	44.527312	50.274548	96.205114	29.767493
PDIA6	0	0.305518	0	0.746714	0	0	0	4.212955
PDIK1L	4.010712	6.41305	8.639847	11.038432	2.118382	13.50609	0	9.951776
PDILT	0	0	0	0	0	0	0	0
PDK1	8.088122	7.01444	23.94924	13.166946	37.822525	13.288751	19.9439	24.190607
PDK1P2	0	0	0	0	0	0	0	0
PDK2	5.729751	10.640747	5.124549	16.264278	0.755704	18.662979	3.191699	12.932495
PDK3	4.02277	3.04923	3.395718	2.438623	4.120906	4.114584	16.504002	5.74258
PDK4	0	0	0	0.292557	0	0	0	0.67606
PDLIM1	439.66343	467.615548	536.020449	596.756691	397.777457	470.614963	358.730038	482.040838
PDLIM1P1	0	0	0	0	0	0	0	0
PDLIM1P2	0	0	0	0	0	0	0	0
PDLIM1P3	0	0	0	0	0	0	0	0
PDLIM1P4	0	0	0	0.367858	0	0	0	0.312658
PDLIM2	9.710741	20.521901	0	13.11021	42.090376	27.559442	0	29.43297
PDLIM3	6.819404	4.103652	7.003934	1.640236	0	0.209767	0	0.45385
PDLIM4	15.330384	22.981405	4.076802	32.320669	39.282506	129.210353	51.254875	82.395384
PDLIM5	84.789727	68.539575	78.346705	92.677016	124.253326	53.248405	47.538411	58.630055
PDLIM7	67.841509	75.646491	44.108724	72.310427	83.901592	96.079227	28.459024	84.337964
PDP1	83.742862	133.71403	83.219962	217.424392	72.47996	142.250827	91.749106	175.407015
PDP2	20.741947	32.089479	0	32.194081	29.944569	31.976333	12.319706	30.93446
PDPK1	16.659303	32.611178	15.387982	38.723908	32.363783	36.164351	29.89921	41.779804
PDPK2P	0	0	0	0	0	0	16.535631	11.217425
PDPN	12.568622	29.43222	0	27.602809	11.901176	92.080578	17.312929	60.159922
PDPR	0	13.211612	0	14.760374	30.598959	16.272779	8.972801	12.352701
PDPR2P	3.116019	7.263622	6.39715	5.079726	1.030243	8.487641	0	9.626414
PDRG1	37.324142	80.891169	34.903212	93.534497	8.45545	40.869617	7.313456	40.884711
PDS5A	45.59133	107.582211	47.676486	55.726228	40.763134	60.949085	44.03301	117.926603
PDS5B	21.937807	37.425977	18.957714	50.700092	98.909963	30.809222	76.583324	40.836209
PDSS1	0	4.068608	2.003716	2.179928	20.860906	2.435909	13.583649	7.376787
PDSS1P1	0	0	0	0	0	0	0	0
PDSS1P2	0	0	0	0	0	0	0	0

PDSS2	11.282442	7.145515	5.059477	8.91392	8.62233	10.132568	12.347041	9.483884
PDX1	0	0.609922	1.166028	0.951526	0	0.323816	0	0.367131
PDXDC1	99.864196	150.609024	88.06426	182.731214	148.099516	158.702366	44.19618	143.273952
PDXK	197.100168	514.487577	137.208269	520.287146	302.822431	815.17544	304.386935	808.821151
PDXP	13.630963	3.661452	0	3.426255	0	13.209313	17.547917	5.841926
PDYN	0	0	0	0	0	0	0	0
PDZD11	0	10.752105	6.276588	6.131055	2.936679	19.549218	0	14.218777
PDZD2	20.183237	13.422762	10.511003	9.482463	9.654527	55.714446	12.027939	34.187392
PDZD4	0.974505	0.597669	0	2.232018	0.257275	0	0	1.021664
PDZD7	7.981058	3.541252	4.568271	3.466659	2.247907	0.928318	0	0.687916
PDZD8	61.752192	133.670783	55.300327	99.156265	81.136586	91.246141	28.782348	146.52097
PDZD9	0	0	0	0.171297	0	0	0	0
PDZK1	0	0.699729	0	0.781254	8.01333	4.729941	0	2.398301
PDZK1IP1	0	0	12.67876	14.93974	20.199339	65.675159	35.340567	43.521474
PDZK1P1	0	0	0	0.16524	0	0	0	0.61172
PDZPH1P	0	0	0	0	0	0	0	0
PDZRN3	0	0	0	0.030065	0	0	0	0
PDZRN4	0	0	0	0	0	0	0	0
PEA15	0	143.660665	89.043078	151.5824	4.083111	320.544956	152.39852	132.864959
PEAK1	12.219286	36.641459	22.361419	39.983135	29.285779	47.524012	6.723805	65.768574
PEAK3	0	0	0	0	0	0	0	0
PEAR1	0	2.284098	7.098689	1.844192	0.388438	1.912367	0	0
PEBP1	118.759662	137.267676	96.577377	105.603484	186.250314	203.812417	165.66141	185.95292
PEBP1P1	0	0	0	0	0	0	0	0
PEBP1P2	0	0	0	0	0	0	0	0
PEBP1P3	0	0	0	0	0	0	0	0
PEBP4	0	0.898565	0	0.210167	0	0	0	0
PECAM1	0	0	0	0.112579	0	0	0	0
PECR	5.015577	2.768482	5.011296	4.764189	0.903916	4.262332	0	2.415657
PEDS1	44.546422	118.297944	75.654613	132.480552	30.446263	113.702747	28.855297	86.394632
PEDS1-UBE2'	0	0	0	0	0	0.617039	0	1.790767
PEF1	28.645926	8.620259	7.848858	11.103286	47.785445	20.449905	94.180073	17.61014
PEG10	10.661481	35.843304	2.642811	17.872247	17.588414	95.269335	50.20296	60.848188

PEG3	0	0	0	0	0	0	0	0
PELI1	4.461469	19.40911	5.603465	21.760527	12.722773	40.294244	4.558182	38.232604
PELI2	1.108394	2.288656	0	2.76263	0.146425	0.33337	0	0.177802
PELI3	13.756271	26.246256	11.196327	18.47376	3.180706	18.355965	142.328115	12.114235
PELO	21.756723	57.09425	18.437738	64.677109	9.144998	34.997953	47.384136	39.362258
PELP1	69.540519	15.776904	34.455683	20.643167	34.388747	42.670911	111.735249	24.666708
PEMT	0	25.641205	15.584889	19.529422	18.391399	30.853249	0	27.725447
PENK	0	0	0	0	0	0	0	0
PEPD	48.804388	27.440979	16.320622	22.006247	21.427206	26.578531	19.663449	28.107797
PER1	12.433851	8.195702	8.957616	2.995864	0	8.426064	67.567077	4.755531
PER2	0	3.369087	1.372013	6.433557	4.747105	17.383211	4.712234	11.63944
PER3	0	2.408966	1.412237	2.336349	3.597284	5.126065	2.93576	4.998229
PER3P1	0	0	0	0	0	0	0	0
PERM1	0	0	0	0.080829	0	0	0	0
PERP	65.953282	59.831368	92.330174	53.944189	97.873579	56.848418	70.519693	78.814165
PERPP1	0	0	0	0	0	0	0	0
PERPP2	0	0	0	0	0	0	0	0
PERPP3	0	0	0	0	0	0	0	0
PES1	32.156045	86.834973	51.993747	72.320864	52.990792	92.128416	72.849593	63.005114
PES1P1	0	0	0	0	0	0	0	0
PES1P2	0	0	0	0	0	0	0	0
PET100	64.640487	25.664732	0	27.097855	173.101236	46.405647	261.361854	7.977144
PET100P1	0	0	0	0	0	0	0	0
PET117	18.867965	72.391467	18.296732	45.760317	10.278591	55.210617	24.058	67.941901
PEX1	32.060254	8.617052	7.276184	3.511338	6.25144	3.350836	0	7.324904
PEX10	0	4.97716	10.8559	1.011933	19.556591	0	0	0.349207
PEX11A	6.242943	11.604237	7.825098	7.972186	3.953615	5.483397	3.637423	11.801332
PEX11B	19.748955	26.8636	25.403555	20.249986	12.138231	13.285848	0	14.979293
PEX11G	0	0.793872	4.437263	1.852749	0	1.367783	0	0.415732
PEX12	9.066967	5.670974	7.903424	11.099094	7.176944	8.867089	0	6.39164
PEX12P1	0	0	0	0	0	0	0	0
PEX13	22.90546	26.831787	9.899492	21.831443	8.232093	19.24314	42.684453	32.397582
PEX14	12.691868	16.283097	19.41749	25.080312	33.068496	21.241315	0	13.235175

PEX16	14.863605	7.978588	6.49171	12.237069	15.362878	15.482472	0	14.319737
PEX19	32.309266	27.767545	21.508573	41.985985	15.847021	20.600095	49.213711	18.534535
PEX2	31.440874	40.687156	62.594752	59.14924	7.741255	44.308825	0	50.277168
PEX26	2.826296	3.97331	6.841119	2.80902	4.063979	3.517014	3.415692	4.261621
PEX3	11.053395	12.156456	18.694973	6.886796	48.05328	10.215165	2.981922	27.273064
PEX5	22.598954	47.389746	32.390488	37.778499	32.436439	39.68163	12.734157	30.307099
PEX5L	0	2.292866	0	0.380351	0.199381	0	0	0.163772
PEX6	2.509104	1.901045	1.73382	2.688345	5.870228	3.328409	0	2.525357
PEX7	12.405275	7.279386	13.235347	2.442233	0	1.655107	14.954391	9.368482
PF4V1	0	0.680727	0	0.669041	0	0	0	0
PFAS	34.830061	7.920549	4.369572	10.040575	33.446715	29.718914	188.228448	18.329533
PFDN1	12.705274	8.062018	6.801626	2.412124	57.396465	4.274532	127.790735	10.525205
PFDN1P1	0	0	0	0	0	0	0	0
PFDN1P2	0	0	0	0	0	0	0	0
PFDN2	136.770209	228.532485	177.333516	307.12613	112.927702	195.600242	231.65017	162.660388
PFDN4	120.672065	95.278272	79.925839	29.788021	72.946909	32.096875	74.542814	85.380547
PFDN5	468.061067	348.146326	423.994096	423.084555	131.366694	186.459724	5.234011	178.329209
PFDN6	109.787127	174.589774	62.706964	212.368922	59.236943	161.448824	163.181659	157.740541
PFKFB1	0	0.509297	2.869615	0.765916	0	0	0	1.844563
PFKFB2	14.641747	36.474021	26.965957	26.234279	34.018849	34.626398	14.838106	24.055072
PFKFB3	19.831496	34.868929	52.872968	36.305113	36.921945	38.208633	85.086471	41.675728
PFKFB4	5.704177	6.42271	13.011269	8.952661	6.526019	11.89519	4.623739	17.10009
PFKL	46.294151	58.109127	27.991558	61.615337	93.271743	171.03636	54.979051	123.836648
PFKM	71.591306	32.45012	43.165963	69.266912	44.459626	45.041398	46.270464	34.079733
PFKP	70.964283	78.033778	75.083284	111.132058	231.846792	106.674343	44.477443	77.606871
PFN1	0	0	0	0.792534	0	7.813952	0	1.995908
PFN1P1	0	0	0	0	0	0	0	0
PFN1P10	0	0	0	0	0	0	0	0
PFN1P11	0	0	0	0	0	0	0	0
PFN1P12	0	0	0	0	0	0	0	0
PFN1P2	0	0	0	0	0	0	0	0
PFN1P3	0	0	0	0	0	0	0	0
PFN1P4	0	0	0	0	0	0	0	0

PFN1P6	0	0	0	0.327989	0	0	0	0
PFN1P8	0	0	0	0	0	0	0	0.366287
PFN1P9	0	0	0	0	0	0	0	0
PFN2	202.348748	421.918929	204.276828	349.207988	142.484385	181.856042	28.914248	196.261958
PFN3	0	0	0	0	0	0	0	0
PFN4	0	3.401636	11.469495	2.651674	0	0	0	2.45789
PFN5P	0	0	0	0	0	0	0	0
PGA3	0	0	0	0	0	0	0	0
PGA4	0	0	0	0	0	0	0	0
PGA5	0	0	0	0	0	0	0	0
PGAM1	424.5685	594.193064	350.830281	518.019989	512.217376	638.250165	600.468475	875.193382
PGAM1P1	0	0	0	0	0	0	0	0
PGAM1P10	0	0	0	0	0	0	0	0
PGAM1P11	0	0	0	0	0	0	0	0.262315
PGAM1P12	0	0	0	0	0	0	0	0
PGAM1P13	0	0	0	0	0	0	0	0
PGAM1P2	0	0	0	0	0	0	0	0
PGAM1P3	0	0	0	0	0	0	0	0
PGAM1P4	0	0	0	0	0	0	0	0
PGAM1P5	0	0	0	0	0	0.258115	0	0.446636
PGAM1P6	0	0	0	0.132889	0	0	0	0
PGAM1P7	0	0	0	1.08169	0	1.889608	0	0
PGAM1P8	0	0	0	0.865588	0	0	0	0
PGAM1P9	0	0	0	0	0	0	0	0
PGAM2	0	0	0	0	0	0.230774	0	0.197761
PGAM3P	0	0	0	0	0	0	0	0
PGAM4P1	0	0	0	0	0	0	0	0
PGAM4P2	0	0	0	0	0	0	0	0
PGAM5	37.188875	58.179175	50.494281	50.596169	39.572591	52.908913	52.348599	49.722992
PGAM5P1	0	0	0	0	0	0	0	0
PGAP1	15.719247	3.680689	3.121907	1.885696	18.384571	2.053339	21.336244	4.790289
PGAP2	24.156965	45.005048	24.226327	48.998606	23.667503	44.192286	72.336365	54.691054
PGAP3	1.649882	2.606631	8.403534	3.771303	3.045247	4.558448	0	4.832275

PGAP4	17.946871	36.932173	23.815903	31.756329	12.976484	37.281147	15.194616	35.127694
PGAP6	48.771955	6.713683	15.400259	16.466656	41.369889	29.739092	18.241751	17.624021
PGBD1	2.161526	11.164074	3.87415	7.305679	0.570544	17.234949	11.818067	17.514299
PGBD2	7.464917	8.862524	0	9.687466	6.764633	11.390786	0	7.65468
PGBD4	1.963703	3.632448	0.882853	2.605834	1.037789	1.797742	3.710443	2.971904
PGBD4P1	0	0	0	0	0	0	0	0
PGBD4P2	0	0	0	0	0	0	0	0
PGBD4P3	0	0	0	0	0	0	0	0
PGBD4P4	0	0	0	0	0	0	0	0
PGBD4P5	0	0	0	0	0	0	0	0
PGBD4P6	0	0	0	0	0	0	0	0
PGBD4P8	0	0	0	0	0	0	0	0
PGBD5	0	0.063346	0	0.152582	2.407052	0.457796	0	1.098182
PGBP	0	0	0	0	0	0	0	0
PGC	0	0	0	0	0	0	0	0
PGD	96.777921	126.825435	70.294843	168.467036	70.192553	101.502529	306.018561	71.939143
PGDP1	0	0	0	0	0	0	0	0.101688
PGF	4.176406	10.109988	0	8.208829	13.162517	9.07391	10.595919	6.588785
PGGHG	0	2.850219	2.943733	3.141926	18.715124	1.904525	0	1.160376
PGGT1B	4.374704	19.99487	14.881149	26.195729	10.366622	6.806395	95.432498	25.867673
PGGT1BP1	0	0	0	0.180284	0	0	0	0
PGGT1BP2	0	0	0	0	0	0	0	0
PGK1	594.363548	425.429298	496.061751	507.142285	569.891792	534.717342	610.56099	589.561798
PGK1P1	0	0	0	0.07141	0	0	0	0
PGK1P2	0	0	0	0.073725	0	0	0	0
PGLS	19.11649	38.046028	16.868853	34.716171	40.775276	60.116761	33.341638	54.886405
PGLYRP1	0	0	0	0	0	0	0	0
PGLYRP2	0	0	0	0	0	0	0	0
PGLYRP3	0	0	0	0	0	0	0	0
PGLYRP4	0	0	0	0	0	0.830085	0	0
PGM1	106.850253	90.392371	89.002987	79.538638	53.561782	40.506132	48.268023	49.774665
PGM2	59.023986	32.125424	33.639227	48.275599	39.568519	30.801227	64.491394	34.722582
PGM2L1	4.180595	4.551801	4.787294	2.924427	7.131748	10.202137	9.828147	8.299217

PGM3	13.578579	20.469777	6.854374	24.929365	21.758464	14.78604	2.71937	27.161387
PGM5	0	0.929646	0	0.023169	0.967025	0.095885	0	0.605018
PGM5P2	0	0	0	1.145673	0	1.58313	0	3.00569
PGM5P4	0	0	0	0	0	0	0	0
PGP	0	1.151594	0	0.627013	0	0	0	0.49527
PGPEP1	12.816722	7.648594	4.93174	10.3103	6.641797	16.218077	1.225792	14.480413
PGPEP1L	0	0	0	0	0	0	0	0
PGR	0	0	0	0	0	0	0	0
PGRMC1	102.837111	48.894781	94.547778	33.014601	111.23318	24.152163	73.765439	30.561435
PGRMC2	46.072832	44.051826	46.202949	26.873621	18.129036	4.697119	23.631922	32.575647
PGS1	15.345282	9.725131	7.059296	11.804971	32.301686	9.439556	0	11.740528
PHACTR1	0	0	0	0	0	0.034295	0	0
PHACTR2	20.725531	21.49384	23.703517	21.214059	58.315585	46.881973	7.73465	37.222784
PHACTR2P1	0	0	0	0	0	0	0	0
PHACTR3	4.498263	26.961193	0	19.932295	3.554321	6.74941	11.945188	12.374509
PHACTR4	29.611821	20.814373	8.696692	21.87965	29.266903	41.737728	55.018812	31.31034
PHAF1	9.559501	19.765206	46.362915	10.529371	32.219186	11.067653	0	15.583454
PHAX	72.548935	101.731569	42.790122	94.431241	51.908201	75.493427	90.954814	119.496029
PHB1	0	34.927044	62.709884	0.194962	39.655521	0	148.062348	23.271782
PHB1P1	0	0	0	0	0	0	0	0
PHB1P10	0	0	0	0	0	0.25	0	0
PHB1P11	0	0	0	0	0	0	0	0
PHB1P12	0	0	0	0	0	0	0	0
PHB1P13	0	0	0	0	0	0	0	0
PHB1P14	0	0	0	0	0	0	0	0
PHB1P15	0	0	0	0	0	0	0	0
PHB1P16	0	0	0	0	0	0	0	0
PHB1P17	0	0	0	0	0	0	0	0
PHB1P18	0	0	0	0.119354	0	0	0	0
PHB1P19	0	0	0	0	0	0	0	0.227344
PHB1P2	0	0	0	0	0	0	0	0
PHB1P20	0	0	0	0	0	0	0	0
PHB1P21	0	0	0	0	0	0	0	0

PHB1P3	0	0	0	0	0	0	0	0
PHB1P4	0	0	0	0	0	0	0	0
PHB1P5	0	0	0	0	0	0	0	0
PHB1P6	0	0	0	0	0	0	0	0
PHB1P7	0	0	0	0	0	0	0	0
PHB1P8	0	0	0	0	0	0	0	0
PHB1P9	0	0	0	0.117185	0	0	0	0
PHB2	39.975689	19.444855	51.223517	12.99768	70.922565	12.62117	318.705103	4.979579
PHB2P1	0	0	0	0	0	0	0	0
PHC1	15.577824	21.19013	54.2681	25.347278	30.07426	21.050076	7.686171	13.184079
PHC1P1	6.016054	3.375025	0	4.671415	0	4.700814	0	2.263267
PHC2	30.634949	96.425965	44.101992	112.314176	54.821953	229.214399	61.364214	227.203402
PHC3	33.226384	28.355753	44.310955	45.696276	25.58138	33.080935	93.069509	31.585783
PHETA1	16.529054	11.786012	1.321035	12.022281	5.065921	22.912778	0	20.504695
PHETA2	4.395188	3.671115	15.861082	3.331259	0	0.945097	0	0.5903
PHEX	1.032678	2.264874	0.92842	2.172053	0	0.362517	0	0.973042
PHF1	19.942872	24.718838	21.907104	13.187678	2.444382	33.018329	0	20.990372
PHF10	8.591886	34.669939	11.436338	25.482542	28.45069	24.635174	8.121557	29.075632
PHF10P1	0	0	0	0	0	0	0	0
PHF10P2	0	0	0	0.628787	0	0.754744	0	0.21717
PHF11	0	6.558934	7.091127	7.031341	26.970452	4.657217	47.389441	11.878807
PHF12	27.645075	48.461933	34.46311	52.628572	33.660255	77.339483	23.613941	42.473969
PHF13	19.194613	26.887835	20.497326	28.213768	17.741732	33.176477	22.453952	29.12184
PHF14	14.308308	7.284707	23.98753	14.129514	0.591982	4.58781	55.317544	4.847812
PHF19	71.351371	92.899922	44.271525	79.288238	93.300207	94.037589	157.83877	88.8752
PHF2	15.129116	29.919037	19.032748	28.172082	12.310491	49.364825	21.82244	31.315443
PHF20	37.134835	110.285156	85.361249	136.154737	20.324563	73.897613	105.347039	79.205246
PHF20L1	23.737927	35.791591	33.068858	29.853629	63.566742	43.749218	44.604833	71.578452
PHF21A	53.948818	19.269476	12.497784	30.523962	13.961233	29.040185	80.75391	28.889796
PHF21B	0	1.411678	0	1.549559	0	0	0	0.816168
PHF23	31.617716	81.901096	28.641377	73.304778	65.114206	168.089649	30.38626	103.050595
PHF24	0	0	0	0	0	0.346123	0	0
PHF3	30.973553	36.476151	47.559099	30.601842	24.734885	20.240872	42.746051	50.250266

PHF5A	61.631897	72.136527	44.837631	86.913472	66.671883	51.038055	19.107497	66.189659
PHF5AP1	0	0	0	0	0	0	0	0
PHF5AP3	0	0	0	0	0	0	0	0
PHF5AP4	0	0	0	0	0	0	0	0
PHF5AP5	0	0	0	0	0	0	0	0
PHF5AP7	0	0	0	0	0	0	0	0
PHF6	9.146891	23.318163	25.701689	29.208	0	20.722806	35.231478	32.587843
PHF7	24.064386	38.315068	0	29.434625	7.936722	37.092985	31.936432	49.931528
PHF8	9.102883	24.387416	17.464639	23.295019	4.844733	45.461113	58.421284	9.698225
PHGDH	167.452275	150.517941	129.340392	200.697855	119.471805	107.701679	80.788568	33.029286
PHGR1	0	0	0	0	0	0	0	0
PHIP	13.958043	6.902458	10.391606	5.964985	14.830288	6.330908	13.563947	10.071848
PHKA1	15.925858	29.068877	10.175204	26.129722	19.39478	24.131996	7.601694	24.151329
PHKA1P1	0	0	19.333815	9.197019	0	21.797993	0	5.720171
PHKA2	2.404819	2.021297	2.197629	2.761505	0	4.231308	0	7.609779
PHKB	63.240255	77.088899	43.873343	47.853842	41.807308	35.999499	74.427112	64.327638
PHKBP1	0	0	0	0	0	0	0	0
PHKBP2	0	0	0	0	0	0	0	0
PHKG1	0	0	0	0	0	0	0	0
PHKG1P1	0	0	0	0	0	0	0	0
PHKG1P2	0	0	0	0	0	0	0	0
PHKG1P3	0	0	0	0	0	0	0	0
PHKG1P4	0	0	0	0	0	0	0	0
PHKG2	6.991865	21.450276	6.994149	21.161208	20.254005	45.22241	0.562379	41.298614
PHLDA1	212.11815	171.512734	261.304762	162.438303	96.351594	55.921393	122.323743	97.659052
PHLDA2	89.883741	75.56636	52.739587	76.2495	35.936966	54.992476	14.303046	52.37034
PHLDA3	33.06034	64.549941	6.819469	51.259355	0	75.180762	44.31931	45.74556
PHLDB1	268.249131	392.486433	247.413343	386.922818	62.723622	100.763116	33.000602	71.605416
PHLDB2	112.334141	162.776445	82.003593	208.035583	140.01101	247.38271	183.953056	213.08667
PHLDB3	10.846164	6.681061	0	5.642921	4.113461	8.240885	6.274083	8.97515
PHLPP1	2.577051	5.932011	3.243642	4.778482	71.655228	8.33831	2.010302	12.752556
PHLPP2	15.334924	17.477152	7.63504	12.596004	30.699888	21.240792	10.138533	21.950572
PHOSPHO1	0	0	0	0.110016	0	0	0	0.090858

PHOSPHO2	17.404323	7.194082	3.828	3.736318	1.141483	4.249479	0	7.938454
PHOX2A	0	0	0	0	0	0	0	0
PHOX2B	0	0	0	0	0	0	0	0
PHPT1	0	38.778943	0	14.853109	40.786181	58.414287	0	29.01809
PHRF1	12.948177	20.930657	11.107242	25.748611	23.516869	51.886548	12.453288	32.793676
PHTF1	18.538387	130.012067	36.581388	86.958732	17.128114	106.127231	0.543779	121.879405
PHTF2	9.403591	77.539994	32.882177	59.80105	53.909002	36.671697	7.045125	62.128895
PHYH	13.032711	9.531107	20.410924	5.680352	1.83452	2.781087	0	6.115666
PHYHD1	0	0	0	0	0	0	0	0
PHYHIP	1.128756	0.963015	2.022542	1.281741	0	0.562209	0	0.090802
PHYHIPL	10.580513	5.40613	9.752131	3.235881	0.929749	0.800447	10.671676	4.324301
PHYKPL	31.615331	7.353568	23.65729	13.661633	0.329338	9.750943	0	13.993383
PI15	0	0.249709	0.865248	0.284707	0	0	0	0.207776
PI16	0	0	0	1.53342	0	0.18307	0	0
PI3	0	0	0	0.801133	11.142906	4.733634	61.950869	2.148189
PI4K2B	32.898227	22.428877	32.155786	12.044136	21.473833	6.661056	11.429181	25.889125
PI4KA	27.494648	1.560351	0	1.317198	0	1.830625	88.867813	0.659251
PI4KAP1	0	0	0	0	0	0	0	0
PI4KAP2	0	0	4.498093	1.119178	0	2.054376	0	2.024529
PI4KB	48.907843	123.069765	32.128901	135.039358	7.179503	126.695258	67.666458	99.27635
PIANP	1.47328	0.938469	0	2.007641	0	1.023412	0	0.333593
PIAS1	13.516647	16.745839	7.814204	22.221456	19.87408	16.89504	43.664429	13.932078
PIAS2	39.936129	34.501773	27.188488	35.872425	59.693356	28.149472	15.714956	78.413454
PIAS3	32.465511	41.362833	21.810992	40.128046	19.714454	46.36625	35.648102	38.485488
PIAS4	6.554247	7.59338	31.230878	16.762482	8.649922	22.596966	0	20.656095
PIBF1	13.058711	18.482686	19.503681	11.746553	8.329899	6.269454	0	24.932923
PICALM	359.510971	367.85918	379.385575	367.922603	233.815804	153.842484	331.550554	214.493701
PICK1	6.726281	21.030386	12.014685	21.700342	34.982069	20.735663	7.737581	16.328915
PID1	1.430488	0.364688	0	0	0.377339	3.102388	1.144534	5.793863
PIDD1	5.831737	5.459286	8.02211	4.391821	1.421577	10.388962	7.082101	1.892324
PIERCE1	15.827089	17.698822	11.154972	12.005042	1.675598	7.269233	0	5.176959
PIEZO1	81.759764	37.696449	51.927835	53.538638	36.593069	46.316427	38.240861	19.737407
PIEZO1P1	0	0	0	0	0	0	0	0

PIEZO1P2	0	0	0	0	0	0	0	0
PIEZO2	0	0.033626	0.35116	0.326259	0	0	0	0.501694
PIF1	31.083561	1.901136	3.381545	1.7296	6.563896	4.357489	0	2.332091
PIGA	257.197659	5.544213	7.411165	15.153495	70.390876	15.56597	30.732854	13.511014
PIGAP1	2.481055	0	0	0.299978	0	0.121745	0	0.803937
PIGB	9.67801	11.850038	16.678133	11.303092	19.365257	4.251968	0	9.408949
PIGBOS1	12.435265	34.533924	27.927844	33.820049	1.337316	25.101026	0	37.20361
PIGC	24.771551	77.368518	22.024723	72.003118	25.726259	52.758497	30.904558	61.66818
PIGCP1	0	0.724575	0	0.211894	0	1.702851	0	0
PIGCP2	0	0	0	0	0	0	0	0
PIGF	17.96175	14.221413	0	7.68922	11.325944	3.629162	6.561822	1.98584
PIGFP1	0	0	0	0	0	0	0	0
PIGFP2	0	0	0	0	0	0	0	0
PIGFP3	0	0	0	0	0	0	0	0
PIGG	18.869178	13.139189	18.925463	16.365719	31.273649	19.692049	23.995685	10.490795
PIGH	33.760894	28.850905	20.238685	22.613833	23.393319	18.941389	0	22.895119
PIGHP1	0	0	0	0	0	0	0	0
PIGK	14.270562	13.882895	45.696144	13.81491	6.654965	1.534467	4.454093	12.496009
PIGL	40.65663	6.671231	13.643412	3.963923	1.261652	3.391231	56.075632	4.489725
PIGN	22.552653	37.728761	9.442687	20.433067	37.526175	19.913226	20.244349	35.41574
PIGO	0	0.20035	2.432374	0.777507	0	6.760812	0	2.216469
PIGP	40.480917	15.416913	18.555648	11.685065	32.016575	6.979952	1.597568	19.733468
PIGPP2	0	0	0	0	0	0	0	0
PIGPP3	0	0	0	0	0	0	0	0
PIGPP4	0	0	0	0	0	0	0	0
PIGQ	11.838706	7.306371	25.723304	6.668162	14.325779	4.573781	16.090353	12.990755
PIGQP1	0	0	0	0	0	0	0	0
PIGR	0	0	0	0	0	0	0	0
PIGS	17.088296	23.339536	31.265955	25.602861	21.127329	31.735372	39.129311	27.672606
PIGT	58.036611	56.17846	56.128401	61.339775	89.616487	55.005543	64.522729	36.016217
PIGU	0	0	48.033133	2.535831	50.866186	0	0	4.274988
PIGUP1	0	0	0	0	0	0	0	0
PIGV	0	3.450218	0	6.69918	0	4.303927	0	0

PIGW	29.869499	40.803124	15.012475	21.595452	10.427365	10.775435	29.748025	43.85016
PIGX	25.90736	7.573926	0	5.679198	25.818841	6.012404	24.0129	14.966921
PIGZ	2.517119	2.614979	1.126735	4.789847	4.240626	2.673436	0	2.198109
PIH1D1	37.332965	50.284512	30.310978	68.787046	155.404901	115.686391	47.99137	83.181583
PIH1D2	20.681178	0	0	4.135392	0	1.411213	0	4.488513
PIK3AP1	1.36554	1.096204	0.951992	0.755237	0.560758	2.044754	7.485975	3.274325
PIK3C2A	65.774962	86.927182	41.755578	99.339066	51.575415	81.729321	166.927748	125.042552
PIK3C2B	8.590265	23.841994	9.102051	15.123738	6.994849	14.462399	1.990349	16.122953
PIK3C2G	5.763019	2.95386	0	0.31981	1.414908	0	0.175406	2.126183
PIK3C3	0.891006	6.81439	1.221447	20.295472	34.472046	15.663145	80.406899	20.847138
PIK3CA	27.209941	26.067631	22.317728	13.590458	20.12602	10.862309	45.523006	24.919562
PIK3CB	0	33.575908	0	23.347738	29.692427	19.003169	10.13694	22.363069
PIK3CD	0	3.651446	0	4.049026	0	3.299467	0	1.23686
PIK3CDP1	0	0	0	0	0	0	0	0
PIK3CG	0	0	0	0	0	0	0	0
PIK3IP1	1.438977	2.017464	0	1.552969	4.175298	2.755388	8.375167	0.870684
PIK3R1	26.192535	16.21784	10.851773	2.977026	10.480357	2.126766	2.94243	9.247025
PIK3R2	0	28.480443	27.568391	25.157788	40.122405	71.226896	30.098964	54.515891
PIK3R3	2.607387	4.697794	2.616823	5.369404	6.078185	16.836144	1.746593	23.186983
PIK3R4	18.915288	25.955129	56.207455	24.223258	57.728008	15.187023	74.986294	28.212077
PIK3R5	0	0	0	0	0	0	0	0
PIK3R6	0	0	0	0	0	0	0	0
PIKFYVE	15.122953	15.535487	10.793742	14.77602	12.422828	21.861877	61.882921	22.227529
PILRA	6.31019	0.78362	0	0.460808	0	2.156889	0	1.617618
PILRB	3.291151	4.394659	2.939975	4.547674	8.049765	9.301551	4.056541	5.145727
PIM1	35.029862	53.323465	57.123773	92.764304	20.178218	50.754707	28.245577	36.404609
PIM2	18.331019	18.635707	13.396179	15.992223	15.378717	16.171823	0	12.97828
PIM3	19.716026	68.146208	26.418783	72.781902	40.723301	134.852098	23.028212	98.41127
PIMREG	9.777981	8.592275	8.163413	8.613929	18.664703	25.829404	0	21.121766
PIMREGP1	0	0	0	0	0	0	0	0
PIMREGP2	0	0	0	0	0	0	0	0
PIMREGP3	0	0	0	0	0	0	0	0
PIMREGP4	0	0	0	0	0	0	0	0

PIN1	28.26802	0.845369	0	4.331136	68.269994	10.279835	26.399576	2.385076
PIN1P1	0	0	0	0	0	0	0	0
PIN4	0	21.490551	8.470978	25.229978	93.89513	14.78458	14.6974	31.83407
PIN4P1	0	0	0	0.332387	0	1.917842	0	0
PINK1	15.292004	14.461785	20.830461	15.049624	23.537173	52.314905	31.551034	45.131565
PINLYP	8.427112	2.473653	0	4.148153	0	0.830311	0	1.235172
PINX1	3.101605	22.976615	17.981272	22.673793	14.47484	18.240453	0	17.817235
PIP	0	0	0	0	0	0	0	0
PIP4K2A	5.200646	20.531068	1.555428	11.172876	7.339559	7.256141	0	8.406517
PIP4K2B	18.172036	68.915619	29.576556	65.912102	18.604432	105.994639	73.622517	96.471212
PIP4K2C	89.572066	50.167859	45.31613	64.873426	76.461252	93.127696	93.544323	97.327602
PIP4P1	39.597157	35.609096	20.427857	32.904776	15.923237	34.620841	64.12785	26.045124
PIP4P2	4.81632	6.226397	12.67876	7.456374	1.262459	6.297369	0	8.419678
PIP5K1A	70.943477	146.628221	52.351578	178.160552	85.072764	247.001983	101.94593	193.775648
PIP5K1B	0	0	0	0	0	0	0	0
PIP5K1C	0	2.124844	0	4.619641	15.872574	13.224173	0	7.85944
PIP5K1P1	0	0	0	0	0	0	0	0
PIP5K1P2	0	0	0	0	0	0	0	0
PIP5KL1	7.071731	7.330672	5.171629	9.184999	0	1.162152	0	2.931929
PIPOX	0	1.176707	0	1.513431	10.890419	0.393357	0	0
PIPSL	1.261715	1.226184	1.11204	1.136116	5.021951	0.253334	0	2.450635
PIR	37.578042	58.147667	51.282361	48.287542	29.16381	29.986715	13.686847	26.921999
PIRT	0	0	0	0	0	0	0	0
PISD	13.635562	22.860474	15.513727	22.926767	26.556908	24.215648	0	22.644434
PITHD1	56.030565	48.715275	47.886627	49.594325	15.937818	41.568918	52.000449	64.871983
PITPNA	65.664875	161.305303	7.003934	213.879471	20.217198	240.813278	0	244.313406
PITPNB	33.044241	48.682113	0	71.42489	71.326048	63.204346	0	42.768703
PITPNC1	12.405844	7.623221	1.810929	9.305292	2.529754	13.325007	0	12.141031
PITPNM1	39.969291	63.121957	46.917502	43.592553	27.913197	54.221287	123.681785	27.421414
PITPNM2	0	3.572195	4.492009	1.462646	0	1.514041	0	2.691009
PITPNM3	0.905028	1.597731	0.546196	0.693471	1.478242	2.475334	4.319505	2.236057
PITRM1	36.779963	33.531788	27.014284	33.426945	15.735863	21.956189	7.406581	26.917648
PITX1	32.218429	40.044404	14.397542	47.600459	63.332489	85.772833	16.691838	70.060814

PITX2	10.490109	20.984237	0	19.648725	0	6.143564	0.838289	4.904861
PITX3	0	0	0	0	2.003532	0	0	0
PIWIL1	0	0.279967	0	1.859405	0	0	0	0
PIWIL2	2.969597	1.989226	0	0.788997	0	0.73619	0	1.713748
PIWIL3	0	0	0	0	0	0	0	0
PIWIL4	0	1.187731	0	0.077599	0	0.053157	0	0.039647
PJA1	30.157266	79.735343	67.224752	87.643028	25.239769	50.933099	0	33.806155
PJA2	48.570247	42.376915	45.445138	57.79557	41.034794	29.210822	58.326246	37.991447
PJVK	0	3.381074	0	0.491523	0	0.44382	0	1.33564
PKD1	24.117644	2.526928	17.607305	13.320527	43.936307	20.662298	42.580863	7.774504
PKD1L1	0	0	0	0	0	0	0	0
PKD1L2	2.416385	0.304096	0	0.921328	0	0	0	0
PKD1P1	4.383533	0.275954	2.005738	0.221052	2.716536	2.007868	0	1.914874
PKD1P2	0	0	0	0	0	0.218599	0	0
PKD1P3	0	3.483571	0	2.59759	0	6.48527	0	1.2546
PKD1P4	0	1.456849	0	1.360712	0	3.337278	0	1.940394
PKD1P5	0	0	0	0.260604	0	0	1.193428	0.021212
PKD1P6	1.857853	0	0	0.59079	1.036793	1.808269	0	0.542302
PKD2	11.565807	14.354678	14.488978	10.246308	5.82146	8.648835	24.843038	7.991657
PKD2L1	0	0	0	0.109842	0	0	0	0
PKD2L2	0	0	0	0.232607	0	0	0	0
PKDCC	0	0	0	0.033259	0	0	0	0
PKHD1	0.55271	0.023844	0	0.067683	0	0	0	0
PKHD1L1	0	0	0.28703	0.011713	0	0	0	0
PKIA	46.496618	64.115219	22.481929	40.373084	0.440582	6.541464	0	5.762866
PKIB	6.699589	12.029454	7.27173	4.447938	17.59472	1.870076	0	1.279884
PKIG	9.784548	42.785465	12.120732	46.270791	14.576315	37.270093	31.31715	32.441935
PKLR	0	0.093948	0	0	0	0	0	0
PKM	1469.21046	1321.54178	1036.29864	1333.27446	2249.67142	2333.4899	2360.99836	1750.9536
PKMP1	0	0	0	0	0	0	0	0
PKMP2	0	0	0	0	0	0	0	0
PKMP3	0	0	0	0	0	0	0	0
PKMP4	0	0	0	0	0	0	0	0

PKMP5	0	0	0	0	0	0	0	0
PKMYT1	51.284101	63.441863	10.929345	46.959917	78.342839	120.036776	16.443251	67.97378
PKN1	18.164169	29.861637	1.864243	31.817225	18.141417	39.176171	66.322066	34.349093
PKN2	49.527984	70.751978	64.688053	41.085335	101.447779	29.685719	111.248616	61.951898
PKN3	2.946287	5.898348	6.164262	3.353641	27.740122	11.651686	8.834873	6.394488
PKNOX1	20.536814	22.252993	15.19327	25.03897	0	16.079362	22.211802	22.020205
PKNOX2	0	0.090239	0	0	0	0	0	0
PKP1	15.636188	1.258064	0	0	24.668731	18.286097	0	8.483215
PKP2	0	5.099011	0	0	0	0	114.526519	0
PKP3	65.829932	92.848817	68.847434	75.280416	78.333534	205.862161	205.873558	160.890378
PKP4	1.475708	40.237794	21.617409	84.676239	81.939675	154.59511	104.333694	94.134661
PKP4P1	0	0	0	0	0	0	0	0
PLA1A	0	0.232711	0	0.179708	0	0.121559	0	0.100332
PLA2G10	0	0	0	1.203446	0	0.996332	0	0
PLA2G10BP	0	0	0	0	4.479309	0	0	0
PLA2G10CP	0	0	0	0	0	0	0	0
PLA2G10DP	0	0	0	0	0	0	0	0
PLA2G10EP	0	0	0	0	0	0	0	0
PLA2G10FP	0	0	0	0	0	0	0	0
PLA2G10GP	0	0	0	0	0	0	0	0
PLA2G10HP	0	0	0	0	0	0	0	0
PLA2G10IP	0	0	0	0	0	0	0	0
PLA2G10JP	0	0	0	0	0	0	0	0
PLA2G10KP	0	0	0	0	0	0	0	0
PLA2G10P1	0	0	0	0	0	0	0	0
PLA2G12A	40.723695	120.389992	57.479513	121.253509	17.073738	97.945298	18.008448	109.883155
PLA2G12AP1	0	1.809612	0	0.210664	0	0	0	0
PLA2G12AP2	0	0	0	0	0	0	0	0
PLA2G15	7.53331	6.7373	17.984975	21.062576	20.807454	14.311397	0	11.916929
PLA2G1B	59.158805	66.272898	88.384296	67.229828	3.344942	0	2.96971	0
PLA2G2A	0	0	0	0	0	0	0	0
PLA2G2C	0	0	0	0	0	0	0	0
PLA2G2D	0	0	0	0	0	0	0	0

PLA2G2E	0	0	0	0	0	0	0	0
PLA2G2F	0	0	0	0	0	0	0	0
PLA2G3	0	0	0	0	0	0	0	0.157539
PLA2G4A	22.218152	13.337681	20.948449	13.751297	1.234445	3.434851	2.840149	7.291175
PLA2G4B	0	0.413902	0	0	0	0.119917	0	0
PLA2G4C	0	0.582146	10.363114	1.122171	0	2.493537	0	6.353871
PLA2G4D	1.006237	0.257961	6.722755	0.528133	0	0	0	1.269252
PLA2G4E	0	0.208662	0	0.151584	0	0	0	0.074638
PLA2G4F	0	0.952398	0	1.535954	2.00347	4.700362	0	2.088064
PLA2G5	0	0	0	0	0	0	0	0
PLA2G6	0	1.392731	0	4.321894	0.359132	1.769175	0	2.316171
PLA2G7	0	0.255036	0	0	0	0	0	0
PLA2R1	1.900132	2.288664	0.569053	1.971143	4.877648	1.908375	0	3.555301
PLAA	32.226567	23.586247	28.469161	17.215116	48.028662	19.732193	137.965485	36.822461
PLAAT1	0	1.779371	0	0.086222	0	0	0	1.68189
PLAAT2	5.68474	0	0	0.134975	1.487478	5.389733	0	8.661526
PLAAT3	9.852198	29.191881	5.145748	50.009071	9.450964	125.237455	5.124325	120.298933
PLAAT4	0	0.267344	0	3.912699	43.088049	29.534614	95.066187	27.099807
PLAAT5	0	0	0	0	0	0	0	0
PLAC1	6.73921	18.741553	0	13.198469	0	1.697927	0	0.411338
PLAC8	0	0	0	0.316008	0	0	0	0
PLAC8L1	0	0	1.620893	0.132264	0	0.089778	0	0.293252
PLAC9	0	0	0	0	0	0	0	0
PLAC9P1	0	0	0	0	0	0	0	0
PLAG1	3.980057	7.171736	5.522966	11.508095	5.083175	7.937758	2.156972	9.638267
PLAGL1	11.17572	12.505314	1.625251	15.781999	3.278536	22.958028	0	28.722033
PLAGL2	55.869114	89.844281	61.599769	98.76164	27.695299	27.290489	18.387005	31.879428
PLAT	0	25.093769	46.92013	22.595478	0.356521	4.679865	0	6.219488
PLAU	114.824815	178.066441	133.989139	185.084278	161.212786	182.748954	84.590199	202.470382
PLAUR	24.126694	25.774146	32.794077	42.178094	33.663353	41.542662	28.056657	34.813498
PLB1	0	0.891283	0	1.23779	0	3.657367	0	5.398297
PLBD1	0	1.200865	1.583857	0.99086	24.384816	2.705384	21.869947	1.652271
PLBD2	28.757319	15.189099	34.392996	18.437046	41.95988	22.688425	28.514667	16.301525

PLCB1	3.016166	10.760789	1.73707	9.463399	4.409059	14.342168	0	16.58313
PLCB2	4.32542	7.799898	0	9.696291	0	8.530898	0	2.548628
PLCB3	0	0	3.114375	1.048137	86.982359	1.19319	0	4.296344
PLCB4	0	1.082796	8.354164	0.093487	0	0	0	2.50919
PLCD1	0	0	21.049159	2.910525	0	2.881515	0	0
PLCD3	4.768922	58.981864	35.617875	47.630026	128.675367	179.761754	19.836577	116.45968
PLCD4	0	0.993929	0	0.613624	0	0.77928	0	0.930209
PLCE1P1	0	0	0	0	0	0	0	0
PLCG1	41.3103	30.55867	43.789093	31.361596	104.77276	76.336829	48.027681	47.818963
PLCG2	21.219245	0.220169	3.009119	1.225676	2.274621	0.966994	2.357077	1.735784
PLCH1	0.530237	1.073314	1.430001	0	0	0	4.129157	0.085047
PLCH2	11.752247	9.71704	7.300796	16.415462	6.328715	18.882958	3.197307	9.963435
PLCL1	0	0	0	0.222078	0	0	0	0
PLCL2	1.411905	0.950739	0	1.364532	0	0.670849	0	0.986402
PLCXD1	4.940925	16.021383	8.879068	12.339377	9.848844	25.434875	41.025721	15.200117
PLCXD2	60.623412	60.120426	15.788293	91.897161	3.313817	107.293238	74.158416	84.732147
PLCXD3	0	0	0	0	0	0	0	0
PLCZ1	0	0	0	0	0	0	0	0
PLD1	10.944237	11.437851	33.142636	17.523437	37.491523	6.946791	0	8.808008
PLD2	7.736241	12.845421	9.692704	9.77418	13.481366	12.753544	163.027481	8.666588
PLD3	56.807639	99.283099	159.371984	175.54885	66.716313	109.763298	117.4668	77.037136
PLD4	0	0	0	0.038093	0	0	0	0
PLD5	0	0.337984	0	0.12313	0	0	0	0
PLD5P1	0.879384	0.712164	0	0	0	0.947533	5.694246	1.246804
PLD6	5.265514	2.464723	7.068667	2.976373	12.850553	5.367148	12.139253	4.911344
PLEC	40.149998	12.488077	30.808949	25.347796	35.637264	51.175093	20.399205	21.504714
PLEK	0	0	0	0	0	0	0	0
PLEK2	78.241143	353.221086	146.266017	372.578379	98.019785	335.199657	130.399842	348.290561
PLEKHA1	21.312352	73.370425	41.018775	50.976317	85.162963	57.59929	26.735617	136.769345
PLEKHA2	91.373101	125.272096	67.967308	140.529541	33.199401	74.539451	69.947605	61.407462
PLEKHA3	10.486603	39.411761	28.759206	30.212072	3.525595	32.217158	0.112282	57.558745
PLEKHA3P1	0	0	0	0	0	0	0	0
PLEKHA4	7.987826	14.262423	10.255826	21.110865	17.737744	32.840607	3.462323	17.873261

PLEKHA5	30.402142	23.381098	24.427075	24.721431	27.306852	18.479048	22.45708	18.073061
PLEKHA6	10.680299	26.885031	10.020949	26.428577	1.018026	8.353718	2.816281	3.44668
PLEKHA7	13.749525	31.480103	7.239992	44.185445	34.2806	28.667086	0	18.901767
PLEKHA8	9.887259	20.912368	13.146311	16.465858	11.22296	29.519872	22.997501	31.163722
PLEKHA8P1	0	0	9.328861	4.056681	6.931025	0	6.249675	4.672458
PLEKHB1	3.515038	4.295892	1.568993	17.292105	6.023216	4.111352	0	4.096118
PLEKHB2	32.608856	109.975655	44.360604	75.774721	40.25242	106.739089	29.585062	132.958475
PLEKHB2P1	0	0	0	0	0	0	0	0
PLEKHD1	0	0.057472	0	0.032723	0	0	0	0.026868
PLEKHF1	4.15987	6.578825	7.413779	11.938685	16.987824	49.845452	8.409477	37.240935
PLEKHF2	17.398638	60.054822	17.662972	37.268779	13.469198	18.704814	20.959929	50.407911
PLEKHG1	2.782187	11.638861	7.729423	11.829812	0	0.581696	0	1.83305
PLEKHG2	27.58988	20.127194	2.884481	27.596292	15.113737	60.461235	6.194153	29.646148
PLEKHG3	33.203014	55.22255	33.606464	63.577805	44.97661	87.31567	39.55034	60.214698
PLEKHG4	0	15.838288	0	4.308262	0.3898	6.867627	0	3.153761
PLEKHG4B	4.2969	3.027719	1.752917	2.114454	9.003972	6.250947	14.811551	4.083726
PLEKHG5	0	3.380961	0	4.130703	0	4.780622	0	5.837967
PLEKHG6	5.842547	36.448813	10.46651	29.253316	0	12.591355	31.962357	8.494424
PLEKHG7	0	0	0	0.137192	0	0	0	0
PLEKHH1	0	0	4.746114	2.757441	3.848518	0	0	2.016824
PLEKHH2	6.185094	4.534909	2.913502	4.134078	14.66931	1.243407	0	3.023341
PLEKHH3	5.309614	20.39417	18.5741	24.2584	70.106063	33.790821	6.228983	15.750332
PLEKHJ1	1.330395	3.337305	44.887192	10.816785	24.484427	23.591514	24.504988	4.431717
PLEKHM1	6.825872	16.809272	13.34308	22.525179	10.866726	68.447061	42.521699	56.499239
PLEKHM1P1	0	0	0	0.074911	0.44731	0	4.109544	0
PLEKHM2	23.925824	37.575286	22.267037	40.056437	48.377894	130.034909	24.367983	89.341424
PLEKHM3	26.934522	13.206261	6.803197	11.019484	5.299132	8.962436	3.724371	11.070888
PLEKHN1	3.13563	0	10.673287	1.316937	0	0.228865	0	0.591896
PLEKHO1	0	9.693432	0.794898	8.595068	9.323691	11.079527	0	7.111242
PLEKHO2	2.793116	9.046487	5.84549	10.177678	2.212378	13.19709	0	7.006365
PLEKHS1	0	0	0	0	0	0	0	0.077601
PLG	0	0.262715	0	0	0	0	0	0
PLGLB1	0	0	0	0	0	0	0	0

PLGLB2	0	0	0	0.432991	0	0	0	0
PLGRKT	31.622036	41.622778	29.577147	16.392972	2.075624	14.316312	22.685199	31.83406
PLIN1	0	0	0	0.141365	0	0.114879	0	0
PLIN2	0	0	0	0.267889	0	0.090907	0	0
PLIN3	43.084458	46.830037	53.639617	53.679003	43.504179	77.592881	88.173901	63.878194
PLIN4	0	0	0	0.575116	0	0	0	0
PLIN5	0	0.293073	0	0.204878	0	0	0	0
PLK1	41.464443	63.957266	86.505864	59.687147	209.351334	112.19679	50.619975	82.83496
PLK2	225.42568	331.997541	240.411288	265.860212	211.265613	368.341133	339.417245	326.878743
PLK3	23.001029	14.475806	13.567713	11.354583	1.713365	7.72406	23.969801	15.542749
PLK4	10.723065	84.21874	41.953235	47.757657	11.018629	65.105189	23.370211	93.859804
PLK5	0	0	0	0.03446	0	0	0	0
PLLP	7.19948	10.518048	19.978679	14.401449	0.631902	5.461569	0.578854	4.372253
PLLPP1	0	0	0	0	0	0	0	0
PLN	0	0	0	0	0	0	0	0
PLOD1	12.416313	19.316665	37.791105	32.051402	64.820555	34.913167	61.922609	25.715523
PLOD2	125.204721	189.380332	185.88955	108.303159	107.582442	45.037429	91.946714	108.88955
PLOD3	86.4646	25.006433	29.895806	23.840808	18.607599	26.985113	49.425318	19.661458
PLP1	0	0	0	0	0	0	0	0
PLP2	469.325527	275.200036	412.188392	373.593903	202.721685	179.06552	270.454403	194.605858
PLPBP	85.716294	88.815728	38.842747	103.190507	83.602298	56.890695	6.366069	38.495632
PLPP1	6.960982	11.102264	16.518867	13.688161	12.832226	8.098215	0	21.687643
PLPP2	33.421501	38.800247	51.760186	49.235301	36.153895	27.350994	40.500013	21.387043
PLPP3	24.600792	19.309924	7.316925	20.414378	2.154621	4.57331	0	4.839637
PLPP4	6.625875	2.885076	0	0.428345	0	0	0	0
PLPP5	32.048249	24.155501	5.7754	23.237819	20.182721	15.260855	0	18.191359
PLPP7	0	0.070957	0	0	0	0	0	0
PLPPR1	0	0	0	0	0	0	0	0
PLPPR2	11.153351	4.982237	19.405372	5.560419	0	4.045096	0	2.312098
PLPPR3	0	0	0	0	0	0	0	0
PLPPR4	0	0.281502	0.654947	0.017817	0	0	0	0
PLPPR5	3.318354	5.277355	0.744506	1.081786	0.219075	0.248747	0	3.104956
PLRG1	110.982304	68.470342	55.319418	65.323661	76.992927	43.833573	147.678406	65.497439

PLS1	23.305197	41.39382	27.020733	38.102558	40.265463	21.039332	26.421966	46.185767
PLS1P1	0	0	0	0	0	0	0	0
PLS3	457.514121	260.575747	212.82479	223.529026	220.569457	179.027729	284.715795	257.07223
PLSCR1	93.230614	96.423977	56.53156	83.265748	214.905682	161.940296	124.255259	178.53474
PLSCR2	0	0	0	0.181821	0	0	0	0
PLSCR3	39.623431	78.808766	55.681706	79.285655	35.386822	121.92991	26.770904	111.7732
PLSCR4	3.62124	1.75145	0	1.566814	0	1.367237	9.558809	0.238788
PLSCR4P1	0	0	0	0	0	0	0	0
PLSCR5	0	0	0	0	0	0	0	0
PLTP	31.419543	49.165242	71.724668	74.851399	22.004887	36.356068	24.147674	19.962962
PLVAP	0	0	0	0	0	0	0	0
PLXDC1	0	0.14067	7.235005	0.57015	0	0.823512	0	0
PLXDC2	0.782029	0.033745	0.2347	1.069697	0	0.177924	0	0
PLXNA1	0	6.304724	29.296146	0.093248	53.755239	0	46.113888	0.350901
PLXNA2	20.318222	65.424468	34.736672	103.931023	4.488144	45.25526	1.426765	26.26059
PLXNA3	22.315083	4.77795	7.954027	14.445807	14.846736	15.009006	0.647009	8.12963
PLXNA4	0	0.599178	0	0.337009	0	0	0	1.203496
PLXNB1	24.996205	2.278547	20.061338	4.440783	62.591654	5.84861	0.248722	4.041986
PLXNB2	34.93492	41.896718	70.315703	82.15494	83.545319	69.269814	51.5787	65.541233
PLXNB3	1.056032	13.23248	9.598732	4.805641	9.151901	4.978334	3.217692	1.387519
PLXNC1	3.938528	6.252865	8.764882	4.253014	0.341923	3.383342	0	4.301531
PLXND1	0	2.182248	6.658884	4.058444	19.372629	11.374924	0	2.493412
PM20D1	0	0	0	0	0	0	0	0
PM20D2	18.13303	7.749366	15.032811	4.191631	34.080478	6.352635	13.757006	16.933799
PMAIP1	0	15.130299	60.653451	11.085403	0	1.317359	0	6.612499
PMCH	0	0	0	0	0	0	0	0
PMCHL1	0	0	0	0	0	0	0	0
PMCHL2	0	0	0	0	0	0	0	0
PMEL	8.187272	2.574066	11.885128	2.886304	0	2.317196	0	0
PMEPA1	118.335274	186.900603	140.554359	263.266272	73.590227	174.404154	114.611578	138.066551
PMF1	21.3835	29.758755	44.092329	36.71736	28.162579	32.244074	0	35.007246
PMF1-BGLAF	0	0	0	0.535993	0	0.290309	0	3.065709
PMFBP1	0	1.032148	0	0.830754	0	1.208761	0	0

PMIS2	0	0	0	0	0	0	0	0
PML	18.198443	13.479039	16.067863	18.96964	88.394565	65.942878	39.070673	41.755705
PMM1	20.846099	22.572687	10.561383	27.05851	1.358544	24.335479	0	11.609482
PMM2	71.103447	85.458987	44.766567	108.921838	64.534322	66.379825	88.887936	68.229464
PMM2P1	0	0	0	0	0	0	0	0
PMM2P2	0	0	0	0	0	0	0	0
PMP2	0	0	0	0	0	0	0	0
PMP22	0	0	3.614948	0	4.80867	1.999966	0	1.638783
PMPCA	72.188217	68.352349	47.91088	66.798476	49.883086	52.734045	73.783962	52.143158
PMPCAP1	0	0	0	0	0	0	0	0
PMPCB	62.419338	40.459142	67.950704	35.274168	66.916776	20.19743	42.795279	29.326547
PMS1	16.841875	19.261397	9.784146	8.266244	14.946881	3.716227	16.746335	11.305563
PMS2	9.042055	50.113626	25.922745	56.90599	23.557076	32.89924	37.187352	37.623507
PMS2CL	11.780226	21.412628	20.96414	24.507353	4.013179	10.399414	0	4.70813
PMS2P10	0	0	0	0	0	0	0	0
PMS2P11	0	0.491181	0	0	0	0	0	0
PMS2P12	0	4.897426	0	3.893833	0	8.653105	0	0
PMS2P13	19.085175	3.717248	0	5.547502	0	7.963446	0	4.990178
PMS2P14	0	0	20.417244	0	0	0	0	0
PMS2P3	0	6.580542	0	7.887338	0	11.134357	0	7.923174
PMS2P5	0	7.276033	0	10.1295	17.422601	11.043689	0	8.382767
PMS2P6	0	13.502725	0	14.544211	0	12.177962	0	12.501094
PMS2P7	0	3.227251	0	1.759074	0	0.181665	0	1.649704
PMS2P8	0	0	0	0	12.259236	2.136269	0	0
PMS2P9	0	0	0	0.539588	0	0.667023	0	2.453751
PMVK	19.25664	48.062229	40.775671	56.366679	0	45.058009	0	42.419254
PNCK	0	0	0	0.536529	0	0.325816	0	0.100412
PNISR	44.817241	26.819828	21.008605	25.299859	20.251651	25.71697	66.35441	30.264172
PNKD	23.2066	21.350245	10.840491	24.500326	29.680867	37.568763	68.796142	20.895088
PNKDP1	0	0	0	0	0	0	0	0
PNKP	11.899926	23.60275	26.475969	28.147955	30.628095	30.964763	31.845216	31.876952
PNLDC1	0	0	0	0	0	0	0	0
PNLIP	0	0	0	0	0	0	0	0

PNLIPP1	0	0	0	0	0	0	0	0
PNLIPRP1	0	0	0	0	0	0	0	0
PNLIPRP2	0	0	0	0	0	0	0	1.19292
PNLIPRP3	0	0	0	0	0	0	0	0
PNMA2	22.878319	95.576823	31.75576	201.682994	6.043164	44.29398	0.168835	39.943687
PNMA3	0	0	0	0	0	0.052821	0	0.255646
PNMA5	0	0	0	0	0	0	0	0
PNMA6B	0	3.001828	0	4.766651	0	4.196554	0	3.132997
PNMA6E	0	0	0	0	0	0	0	0
PNMA8A	7.001195	7.04466	2.690172	7.075481	1.320193	10.185311	0.24315	6.395194
PNMA8B	0	0	0	0	0	0	0	0
PNMT	0	0	0	0.471596	0	0.472517	0	0
PNN	55.455463	68.164508	88.180838	83.638521	81.645098	85.596535	204.544653	62.636121
PNO1	33.687616	48.653913	20.531264	46.914251	26.577248	38.384098	17.444099	27.348924
PNOC	0	0	0	0	0	0	0	0
PNP	0	1.910609	0	0	0	8.323682	0	0
PNPLA1	0	0	0	0.161784	0	0	0	0
PNPLA10P	0	0	0	0	0	0	0	0
PNPLA2	18.354763	38.84243	35.361758	45.640638	52.344094	64.745953	8.561301	41.508161
PNPLA3	4.862592	0.220765	0	0	20.658782	3.451255	0	1.627782
PNPLA4	13.659373	42.344271	47.095513	27.640389	1.053818	34.014283	0	41.025867
PNPLA4P1	0	0	0	0	0	0	0	0
PNPLA5	4.274374	0	0	0	0	0.205698	0	0
PNPLA6	7.242315	20.580717	3.148866	18.362084	42.046885	45.014361	32.927738	19.219756
PNPLA7	0	0	0	0.209118	0	0.219014	0	0.140824
PNPLA8	5.749877	26.243047	16.1251	29.10703	33.641154	12.624983	54.959427	28.350337
PNPO	59.600572	68.318654	9.371304	47.097891	32.544398	36.206879	81.387699	43.025524
PNPP1	0	0	0	0	0	0	0	0
PNPT1	41.567351	36.853356	33.722332	13.789978	64.244327	6.344596	56.891594	23.233947
PNPT1P1	0	0	0	0	0	0	0	0
PNPT1P2	0	0	0	0	0	0	0	0
PNRC1	9.113152	14.037212	3.26875	6.207277	45.735414	1.018598	0	8.865173
PNRC2	68.260666	116.319752	49.606572	99.816282	229.663029	98.417418	70.360498	115.389576

PNRC2P1	0	0	0	0	0	0	0	0
POC1A	0	3.675824	13.95149	14.330328	4.214941	4.585248	0	18.356987
POC1B	11.904722	11.896039	6.960025	13.828829	32.120969	19.733716	25.559367	23.60563
POC1B-GALI	0	2.752155	0	1.409337	6.831889	3.822757	25.791255	1.562519
POC5	0	11.441203	6.276658	11.282407	4.940143	0.901507	0	2.564444
PODN	0	0	0	0	0	0	0	0
PODNL1	0	3.464901	1.102263	0.342192	20.787604	3.979328	0	4.360643
PODXL	4.345705	6.328496	9.765923	17.11528	78.18728	193.227914	159.745101	132.361637
PODXL2	0	2.214904	1.414389	4.539712	5.008705	8.157612	0	3.254383
POF1B	1.713289	0.809251	0.768669	2.408016	5.667165	3.1566	21.758166	3.420154
POFUT1	174.649622	154.592238	204.37812	201.304335	119.220985	67.026472	30.152614	62.575812
POFUT2	5.052455	0.049282	7.176773	10.026406	66.12971	16.932689	0.334137	18.423088
POGK	5.208236	67.240156	8.867192	74.990238	22.260436	80.810233	25.631836	59.287972
POGLUT1	6.469517	13.093022	12.769614	10.768339	12.849162	10.427468	35.634662	8.414771
POGLUT2	10.247974	8.098745	3.050417	11.947941	0.90062	5.156158	4.549374	7.028248
POGLUT2P1	0	0	0	0	0	0	0	0
POGLUT3	79.424489	46.422825	41.024268	38.948626	26.62706	41.755264	21.101988	16.303583
POGZ	3.939195	27.3047	25.486373	64.223103	31.178191	35.204909	27.118372	24.46317
POLA1	0	0	0	1.246472	0	0.481275	0	5.916081
POLA2	18.624621	6.102281	20.189987	22.043381	35.986654	24.139215	27.318215	35.848797
POLB	28.988005	65.882016	107.187329	45.88846	5.070996	37.831415	0	53.564505
POLD1	0	3.972047	0	2.058662	0	7.900292	0	4.058876
POLD2	0	16.314983	51.623485	26.137066	0	14.521428	0	21.963609
POLD2P1	0	0	0	0	0	0	0	0
POLD3	0	46.616316	0	85.086792	40.051201	52.150644	0	43.79356
POLD4	84.456789	129.48142	95.875759	185.96451	27.2018	65.122512	60.72951	52.016252
POLDIP2	77.921576	108.217677	55.606815	90.333739	115.895603	105.675168	52.991663	105.547759
POLDIP3	19.881988	17.116848	44.354961	30.151577	49.245172	26.36735	36.494449	20.6283
POLE	0	20.347627	18.463188	17.022737	11.553772	2.817403	0.305138	13.122234
POLE2	43.028653	13.251987	50.483997	10.564454	60.48719	4.424143	0	16.238645
POLE3	75.118517	128.007469	62.884215	93.825056	66.043419	104.980746	49.1726	119.185458
POLE4	134.495682	184.68232	19.891389	123.916969	48.778158	127.227056	138.908251	116.546302
POLE4P1	0	0	0	0	0	0	0	0

POLG	4.407128	19.812596	10.890591	22.379129	11.487802	38.176479	0	26.494717
POLG2	13.52374	16.546764	15.464705	19.89659	11.667917	11.654936	7.104429	13.816991
POLH	0	0	33.169744	0	0	4.744835	4.16642	8.810426
POLHP1	0	0	0	0	0	0	0	0
POLI	8.613373	14.488689	7.205567	5.296171	2.418239	3.602624	0	12.379358
POLK	12.692016	14.184656	13.879625	11.475178	38.695482	3.285124	0.184388	15.6086
POLL	27.624607	29.732835	36.761394	49.07745	27.58039	67.913542	5.050683	46.953005
POLM	1.435047	0.819567	13.707753	2.556691	13.452365	8.409377	15.312867	3.418669
POLN	0	0	0	0.025456	0	0.386798	0	0.083549
POLQ	6.985195	6.732531	3.638857	4.654353	18.749953	6.999269	2.063021	13.517031
POLR1A	38.983221	30.603651	28.465988	36.436673	36.990405	31.484642	22.323779	23.64135
POLR1B	29.085052	29.664086	0	42.478798	47.063113	26.34582	111.73156	47.372943
POLR1C	24.306381	27.690104	35.865295	31.729109	28.475847	33.165667	90.311313	47.617659
POLR1D	80.46197	157.919068	207.335325	152.399251	49.624702	42.232896	112.346844	151.85601
POLR1E	35.830295	46.199013	42.05743	48.620783	64.613339	58.53551	0	57.602765
POLR1F	21.242913	24.437461	14.487169	11.369314	48.737023	6.439477	7.856452	26.003165
POLR1G	22.253732	35.937966	11.107048	43.599236	35.481147	148.845441	0	80.10591
POLR1H	60.304775	45.28135	29.888646	48.88968	49.786107	50.697155	47.29207	55.738832
POLR1HASP	0	2.033547	0.908476	4.034453	3.832075	2.42634	5.957797	3.901442
POLR2A	0	20.306345	0	27.141239	0	42.581049	26.697873	47.560343
POLR2B	50.761489	35.616357	48.572857	34.964559	33.047327	31.755584	31.542799	43.770548
POLR2C	119.663403	135.840051	80.0133	125.214786	74.626548	144.613525	69.912189	160.003338
POLR2CP1	0	0	0	0	0	0	0	0
POLR2D	31.628992	101.837554	60.897267	89.129931	40.765125	116.703193	152.952696	157.157552
POLR2DP1	0	0	0	0	0	0	0	0
POLR2DP2	0	0	0	0	0	0	0	0
POLR2E	105.202162	245.887477	109.868813	271.987936	200.85652	465.939237	176.019511	374.380598
POLR2F	35.914841	161.03789	28.644798	140.43693	58.823719	189.734865	92.590847	153.109651
POLR2G	108.968446	49.859587	82.856916	54.46203	112.8039	97.439403	161.170389	90.814951
POLR2H	167.729046	166.988899	161.320034	191.172008	137.420959	129.314208	96.269956	183.253153
POLR2I	85.243928	120.688496	160.915102	145.851354	103.180957	206.92135	128.890671	196.202432
POLR2J2	0	0	0	0.86573	0	0.854079	0	0.317476
POLR2J3	7.25552	50.581748	1.281426	41.982903	28.984292	55.871726	19.600657	41.272406

POLR2J4	0	0	0.9444	18.841321	61.227326	35.593205	0	21.108586
POLR2K	82.575995	34.906917	43.645575	23.835586	33.59349	8.557315	92.715903	18.553235
POLR2KP1	0	0	0	1.900404	0	0	0	0
POLR2KP2	0	0	0	0	0	0	0	0
POLR2L	113.800337	142.029608	81.209678	101.653513	114.6032	229.934277	75.945417	200.216137
POLR2LP1	0	0	0	0	0	0	0	0
POLR2M	24.058152	70.201432	72.087846	80.741394	27.98571	68.015878	34.658778	54.373146
POLR2MP1	0	0	0	0.184422	0	0	0	0
POLR3A	16.675893	25.372554	19.404674	20.980784	22.032436	15.608019	15.665017	13.736252
POLR3B	8.669132	12.948601	7.190764	11.30479	35.647819	13.65879	14.818377	17.562246
POLR3C	19.881646	67.093898	11.304936	65.455006	32.08252	73.184867	24.873087	80.333029
POLR3D	37.480381	34.030733	32.692396	62.425667	83.736317	71.468926	1.141144	40.207352
POLR3DP1	0	0	0	0	0	0	0	0.903752
POLR3E	21.531971	112.026708	29.433728	116.733218	28.43681	127.266893	74.27979	88.9392
POLR3F	19.176246	17.420425	24.274147	9.790968	17.698837	6.836374	12.390036	10.022687
POLR3G	0	21.59589	0	19.085263	23.12505	20.424585	31.870777	30.61984
POLR3GL	41.205836	60.981046	22.458058	74.245495	29.428894	40.404089	13.702901	24.213253
POLR3GP1	0	0	0	0	0	0	0	0
POLR3GP2	0	0	0	0	0	0	0	0
POLR3H	43.071105	74.298228	8.504123	47.852296	30.515873	70.652312	49.876948	76.818607
POLR3K	0	0	0	0	0	0	28.068201	0
POLR3KP1	0	0	0	0	0	0	0	0
POLR3KP2	0	0	0	0	0	0	0	0
POLRMT	12.30059	18.431481	24.270609	16.618241	71.824658	44.98062	34.939767	24.188824
POLRMTP1	0	0	0	0.022391	0	0	0	0
POM121	2.326475	4.78437	3.017605	5.280928	1.200128	7.757542	0	4.95721
POM121B	0	0.082053	0	0.971295	0	0.214232	0	0.590775
POM121C	23.428365	42.991118	23.731984	67.070137	52.365118	75.439654	28.043355	69.885199
POM121L10F	0	0	0	0	0	0	0	0
POM121L13F	0	0	0	0	0	0	0	0
POM121L14F	0	0	0	0	0	0	0	0
POM121L15F	0	0	0	0	0	0	0	0
POM121L1P	0	0	0	0	0	0	0	0

POM121L2	0	0	0	0	0	0	0	0
POM121L3P	0	0	0	0	0	0	0	0
POM121L4P	0	0	0	0	0	0	0	0
POM121L6P	0	0	0	0	0	0	0	0
POM121L7P	0	0	0	0	0	0	0	0
POM121L8P	0	0	0	0	0	0	0	0
POM121L9P	0	0.195913	0	0	0	0	0	0
POMC	0	0	0	1.209702	8.772813	0.216332	0	0
POMGNT1	14.918058	28.515827	12.24361	27.322585	11.305562	64.386257	0	43.639652
POMGNT2	2.68547	28.544654	8.41064	24.017048	3.542489	25.601243	2.912515	27.171465
POMP	125.596703	60.433201	79.996829	27.682044	82.627189	23.929474	49.302564	58.409387
POMPP1	0	0	0	0	0	0	0	0
POMT1	0	0.137507	0	5.642487	0	0	0	4.362522
POMT2	0	32.986422	19.211442	23.581297	18.143802	3.754941	0	22.088505
POMZP3	15.148352	18.270156	12.80348	12.349841	2.31353	18.266906	4.073809	17.646661
PON1	0	0	0	0	0	0	0	0
PON2	37.565104	23.697029	47.769123	18.976726	23.788223	10.576986	32.296089	12.205415
PON3	0	0	0	0	0	0	0	0
POP1	7.61104	18.030242	5.59293	13.352682	13.131922	16.382234	8.761839	12.340639
POP4	35.457061	62.648101	35.13881	54.733286	85.143871	77.035761	212.509251	108.446118
POP5	0	11.68497	10.225089	3.728031	4.428321	3.574852	0	5.945727
POP7	47.295958	86.691298	24.912986	77.10278	38.43841	101.737012	21.396673	92.059088
POPDC2	0	0	0	0.301902	0	0.136952	0	0.168892
POPDC3	31.319515	29.592853	19.842202	9.783786	1.967865	3.314532	29.595204	12.614021
POR	27.998577	16.551407	9.342783	12.105716	45.561527	10.035899	14.299266	12.616871
PORCN	39.335314	23.589983	15.093319	21.763652	63.229157	40.668222	35.612581	35.594852
POSTN	0	0	0	0	0	0	0	0
POT1	44.283903	46.998096	18.582513	24.753886	22.216556	5.478562	18.263041	17.384543
POTEA	0	0	0	0	0	0	0	0
POTEB	0	0	0	0	0	0	0	0
POTEB2	0	0	0	0	0	0	0	0
POTEB3	0	0	0	0	0	0	0	0
POTEC	0	0	0	0	0	0	0	0

POTED	0	0	0	0	0	0	0	0
POTEE	0	0	0	0	0	0.039649	0	0
POTEF	0	0	0	0	0	0	0	0
POTEG	0	0	0	0	0	0	0	0
POTEH	0	0	0	0	0	0	0	0
POTEI	0	0	0	0	0	0	0	0
POTEKP	0	0.03982	0	0	0	0.09295	0	0
POTEM	0	0	0	0	0	0	0	0
POU1F1	0	0	0	0	0	0	0	0
POU2AF1	0	0	0	0	0	0	0	0.09423
POU2AF2	0	0	0	0	0	0	0	0
POU2AF3	0	1.024474	0	5.639065	0	1.755069	0	3.90449
POU2F1	10.083717	10.494194	10.432303	15.120293	12.383829	11.048924	38.792014	11.138851
POU2F2	0	3.290584	0	1.633941	2.851839	5.208576	6.529875	2.754567
POU2F3	0	1.065878	0	1.734029	0.97359	0.779337	0	1.429559
POU3F4	0	0	0	0	0	0	0	0
POU4F1	2.171056	5.431336	3.249278	4.27809	2.676046	0.325874	0	0.435714
POU4F2	0	0	0	0	0	0	0	0
POU4F3	0	0.224342	0	0	0	0.06551	0	0.053066
POU5F1	0	0.517813	0	0.492789	0	0	0	0.78526
POU5F1B	0	0.491333	0	0.578122	0	0.164082	0	0
POU5F1P2	0	0	0	0	0	0	0	0
POU5F1P3	0	0	0	0	0	0	0	0
POU5F1P4	0	0	0	0	0	0	0	0
POU5F1P5	0	0	0	0	0	0	0	0.274883
POU5F1P6	0	0	0	0	0	0	0	0
POU5F1P7	0	0	0	0	0	0	0	0
POU6F1	0	0.346482	12.218385	3.780649	0.831039	0.476113	0	4.881911
POU6F2	0	0.095565	0	0.164189	0	0	0	0
PP1P	0	0	0	0	0	0	0	0
PP2D1	0	0	0	0	0	0	0	0
PPA1	338.397472	253.32741	315.57442	165.491728	254.374165	137.656553	164.462313	251.523236
PPA2	126.824893	107.16784	130.28273	52.052181	150.32704	35.46151	98.524166	82.263512

PPAN	56.910972	37.531739	20.648952	54.253944	18.495422	56.754014	75.978548	39.081936
PPAN-P2RY1	0	0.983166	0	0.161963	0	0.909264	0	0.55406
PPARA	2.91698	4.639093	0	0.740467	17.885093	6.47029	0	4.647687
PPARD	12.428779	36.38578	17.522055	40.996908	10.783062	45.781193	23.44542	39.877637
PPARG	59.53677	39.989367	37.107388	43.878339	13.889816	9.932425	0	22.10993
PPARGC1A	0	0	0	0.157041	0	0	0	0
PPARGC1B	2.731475	4.689691	7.637677	5.482976	8.800606	9.29329	4.222193	8.217983
PPAT	41.444421	22.249985	27.479988	36.014808	30.45029	22.984964	25.641146	18.109522
PPATP1	0	0	0	0	0	0	0	0
PPATP2	0	0	0	0	0	0	0	0
PPBP	0	0	0	0	0	0	0	0
PPBPP1	0	0	0	0	0	0	0	0
PPBPP2	0	0	0	0	0	0	0	0
PPCDC	16.489308	21.799811	29.755294	29.416241	47.849112	25.682344	0	16.371273
PPCS	61.030302	51.360065	70.811221	39.900281	75.366196	33.767569	55.565453	66.725317
PPDPF	225.338313	359.458132	260.00051	315.76361	231.091077	337.139323	69.918187	358.725637
PPDPFL	0	0	0	0	0	0	0	0
PPDPFP1	0	0	0	0	0	0	0	0
PPDPFP2	0	0	0	0	0	0	0	0
PPEF1	0	0	0	0	0	0	0	0
PPEF2	0	0	0	0	0	0	0	0
PPFIA1	36.072709	55.947155	0	69.495035	102.630661	80.451062	89.790777	62.250486
PPFIA1P1	0	0	0	0	0	0	0	0
PPFIA2	0	0	0	0	0	0	0	0
PPFIA3	5.121523	3.20183	3.065222	6.037015	0	8.584214	2.399343	2.583643
PPFIA4	0	1.733577	0	1.218089	1.854805	1.433756	3.165965	1.861639
PPFIBP1	90.749865	175.8763	76.017159	170.33515	143.118692	135.649573	63.004066	158.56563
PPFIBP2	0	3.440614	14.383121	2.852543	0	6.174328	0	9.7322
PPHLN1	2.051988	26.84207	44.012923	22.978154	42.05218	13.219532	37.3562	21.054016
PPIA	1913.85746	1522.07725	1474.43691	1776.16854	3152.19954	2174.89139	3207.93077	3059.45644
PPIAL4F	0	0	0	0	0	0	0	0
PPIAP1	0	0	0	0	0	0	0	0
PPIAP10	0	0	0	0	0	0	0	0

PPIAP11	0	0.390406	0	0	0	0	0	0
PPIAP13	0	0	0	0.246695	0	0	0	0
PPIAP14	0	0	0	0	0	0	0	0
PPIAP15	0	0	0	0	0	0	0	0
PPIAP16	0	0	0	0	0	0	0	0
PPIAP17	0	0	0	0	0	0	0	0
PPIAP19	0	0	0	0	0	0	0	0
PPIAP2	0	0	0	0	0	0	0	0
PPIAP20	0	0	0	0	0	0	0	0
PPIAP21	0	0	0	0	0	0	0	0
PPIAP22	0	24.794752	0	0	132.368347	17.421387	184.759078	41.470801
PPIAP23	0	0	0	0	0	0	0	0
PPIAP24	0	0	0	0	0	0	0	0
PPIAP25	0	0	0	0	0	0	0	0
PPIAP26	0	0	0	0	0	0	0	0
PPIAP27	0	0	0	0	0	0	0	0
PPIAP28	0	0	0	0	0	0	0	0
PPIAP29	0	0	0	0	0	0	0	0
PPIAP3	0	1.875703	0	0.681771	0	0.890437	0	4.110525
PPIAP30	0	0	0	0	0	0	0	0
PPIAP31	0	0	0	0	0	0.464004	0	0
PPIAP32	0	0	0	0	0	0	0	0
PPIAP33	0	0	0	0	0	0	0	0
PPIAP34	0	0	0	0	0	0	0	0
PPIAP35	0	0	0	0	0	0	0	0
PPIAP36	0	0	0	0	0	0	0	0.909011
PPIAP37	0	0	0	0	0	0	0	0
PPIAP38	0	0	0	0	0	0	0	0
PPIAP39	0	0	0	0	0	0	0	0
PPIAP4	0	1.202435	0	1.137959	0	0.724997	0	0
PPIAP40	0	0	0	0	0	0	0	0
PPIAP41	0	0	0	0	0	0	0	0
PPIAP42	0	0	0	0	0	0	0	0

PPIAP43	0	0	0	0	0	0	0	0.412771
PPIAP44	0	0	0	0	0	0	0	0
PPIAP45	0	4.074291	0	0.993057	0	0	0	0
PPIAP46	0	0	0	0	0	0	0	0
PPIAP47	0	0	0	0.702278	0	0	0	0
PPIAP48	0	0	0	0	0	0	0	0
PPIAP49	0	0	0	0	0	0	0	0
PPIAP5	0	0	0	0	0	0	0	0
PPIAP50	0	0	0	0	0	0	0	0
PPIAP51	0	0	0	0	0	0	0	0
PPIAP52	0	0	0	0	0	0	0	0
PPIAP53	0	0	0	0	0	0	0	0
PPIAP54	0	0	0	0	0	0	0	0
PPIAP55	0	0	0	0.237663	0	0	0	0
PPIAP56	0	0	0	0	0	0	0	0
PPIAP57	0	0	0	0	0	0	0	0
PPIAP58	0	0	0	0	0	0	0	0
PPIAP59	0	0	0	0	0	0	0	0
PPIAP6	0	0	0	0	0	0	0	0
PPIAP60	0	0	0	0	0	0.461992	0	0
PPIAP62	0	0	0	0	0	0	0	0
PPIAP63	0	0	0	0	0	0	0	0
PPIAP64	0	0	0	0	0	0	0	0
PPIAP65	0	0	0	0	0	0	0	0
PPIAP66	0	0	0	0	0	0	0	0
PPIAP67	0	0	0	0	0	0	0	0
PPIAP68	0	0	0	0	0	0	0	0
PPIAP69	0	0	0	0	0	0	0	0
PPIAP7	0	0	0	0	0	0	0	0
PPIAP70	0	0	0	0	0	0	0	0
PPIAP71	0	0	0	0	0	0	0	0
PPIAP72	0	0	0	0	0	0	0	0
PPIAP73	0	0	0	0	0	0	0	0

PPIAP74	0	0	0	0	0	0	0	0
PPIAP75	0	0	0	0	0	0	0	0
PPIAP76	0	0	0	0	0	0	0	0
PPIAP77	0	0	0	0.693932	0	0	0	0
PPIAP78	0	0	0	0	0	0	0	0
PPIAP79	0	0	0	0	0	0	0	0
PPIAP8	0	0	0	0	0	0	0	0
PPIAP80	0	0	0	0	0	0	0	0
PPIAP81	0	0	0	0	0	0	0	0
PPIAP82	0	0	0	0	0	0	0	0
PPIAP83	0	0	0	0	0	0	0	0
PPIAP84	0	0	0	0	0	0	0	0
PPIAP85	0	0	0	0	0	0	0	0
PPIAP86	0	0	0	0	0	0	0	0
PPIAP87	0	0	0	0.237821	0	0	2.370397	0
PPIAP88	0	0	0	0	0	0	0	0
PPIAP89	0	0	0	0	0	0	0	0
PPIAP9	0	0	0	0	0	2.272328	0	0.420492
PPIAP90	0	0	0	0	0	0	0	0
PPIAP91	0	0	0	0	0	0	0	0
PPIAP93	0	0	0	0	0	0	0	0
PPIB	0	0	0	0	0	0	0	0
PPIC	0	0	0	0	0	0	0	0
PPID	30.631043	41.405943	32.443753	31.934842	32.795498	20.063469	66.484311	31.612962
PPIE	47.173806	13.435462	0	16.259606	15.631888	5.992753	25.780406	9.461167
PPIEL	0	0	0	0	0	0	0	0
PPIF	72.047825	102.672587	61.589475	98.150672	130.06408	135.212436	68.700411	132.164032
PPIG	5.633135	14.104695	7.325788	9.834879	71.325999	12.694571	55.390317	15.84819
PPIH	74.731402	48.212513	33.803797	58.116172	82.434541	80.83727	25.696465	82.629925
PPIHP1	0	0	0	0	0	0	0	0
PPIHP2	0	0	0	0	0	0	0	0
PPIL1	68.602556	43.294862	58.929193	33.262702	54.20153	28.940604	64.490137	47.97511
PPIL1P1	0	0	0	0	0	0	0	0

PPIL2	8.573437	21.842106	17.64899	14.450571	61.632583	27.043698	21.647099	19.325827
PPIL3	0	49.593074	35.554635	28.122067	39.588517	34.305809	21.288769	44.234665
PPIL4	0	0	0	0	0	0	0	0
PPIL6	8.187272	1.519961	13.03191	0.726879	0	0.245181	0	2.281339
PPIP5K1	20.11965	46.343261	19.924543	49.242334	44.398665	65.127832	168.497558	30.163561
PPIP5K2	41.008585	64.663797	26.397546	25.954451	79.223322	21.611269	78.658563	39.467254
PPL	16.104119	22.453055	21.950523	27.400694	43.787084	78.777452	50.388385	37.289447
PPM1A	46.208639	116.856499	47.242853	119.947927	53.191693	114.517815	82.646978	134.275255
PPM1AP1	0	0	0	0	0	0	0	0
PPM1B	21.848683	119.930314	16.855272	84.730817	104.780214	75.273287	29.510367	120.894252
PPM1D	12.328016	2.576473	8.379271	1.778006	1.037643	1.289799	0	2.636592
PPM1E	0.988627	0.531591	0	0.519916	0	0.049591	0	0.138723
PPM1F	5.078244	19.269942	8.723426	8.739761	15.427613	28.680642	96.498702	21.600375
PPM1G	171.097973	366.391438	154.309458	401.275397	147.857641	468.058479	111.580422	311.38142
PPM1H	7.808987	7.956728	3.163691	3.948887	2.125183	12.533627	2.696031	7.649226
PPM1J	6.608342	0.376639	2.136253	0.115533	1.222875	0.410392	0	0
PPM1K	2.058275	5.653265	6.336531	9.074004	3.158394	8.877375	51.029566	10.928874
PPM1L	3.706126	4.031757	0.529232	5.0469	2.305546	2.854987	0	2.926468
PPM1M	0	4.637677	6.597192	7.349244	4.558059	12.045642	30.604981	8.993081
PPM1N	9.155435	1.868467	7.851406	1.244549	0	1.294848	22.91266	6.696775
PPME1	73.892754	73.028226	80.803379	102.218526	99.644131	56.690016	166.796617	63.418886
PPOX	24.115553	10.001156	7.720923	9.467847	27.163155	6.044247	0	5.259228
PPP1CA	239.438821	344.750965	189.299906	299.741229	175.008337	262.448009	144.714695	292.833918
PPP1CB	119.119898	103.118184	102.523564	60.059019	133.722089	95.307091	151.478409	118.635823
PPP1CC	22.982807	354.419656	0	270.372287	65.457416	325.778968	384.530988	357.499493
PPP1R10	37.875127	180.776681	42.512789	274.842442	52.135323	305.949852	49.41657	254.026615
PPP1R10P1	0	0	0	0	0	0	0	0
PPP1R11	51.997338	44.084386	52.206694	49.529013	39.362651	40.607948	38.581928	39.42509
PPP1R11P1	0	0	0	0	0	0	0	0
PPP1R11P2	0	0	0	0	0	0	0	0
PPP1R12A	84.214699	55.229766	51.888455	40.72108	80.171119	32.875216	155.600355	42.964314
PPP1R12B	13.803454	3.997905	11.020645	12.123563	12.840628	5.936427	1.175464	2.811081
PPP1R12BP1	0	0	0	0	0	0	0	0

PPP1R12BP2	0	0	0	0	0	0	0	0
PPP1R12C	10.371902	11.797064	6.022624	14.025473	17.067324	40.534117	9.720849	18.639704
PPP1R13B	4.164647	6.045979	13.528027	5.064281	13.495179	5.505224	3.748579	9.891706
PPP1R13L	26.766746	48.874601	50.558846	42.888132	149.953228	86.701162	27.668383	57.013125
PPP1R14A	0	0	0	0	0	0	0	0
PPP1R14B	143.632208	190.605971	103.548604	212.485141	416.019036	460.118332	165.677595	462.186599
PPP1R14BP2	0	0	0	0	0	0	0	0
PPP1R14BP3	0	0	0	0	0	0.738188	0	0.596004
PPP1R14BP4	0	0	0	0	0	0	0	0
PPP1R14BP5	0	0	0	0	0	0	0	0
PPP1R14C	18.582701	32.78266	33.217973	34.448818	27.770859	19.730405	23.644439	29.774816
PPP1R14D	0	0	0	0	0	0	0	0
PPP1R15A	45.102484	127.311332	73.443242	220.09901	80.590914	346.012547	105.134286	156.339922
PPP1R15B	47.81876	54.914585	37.943484	59.97349	30.187266	47.282711	26.174769	68.595173
PPP1R16A	6.208511	58.206437	11.117492	53.23503	31.46429	61.539783	1.325644	46.301047
PPP1R16B	0.532206	0.022882	0	0.190325	0	0.026685	0	0.042682
PPP1R17	0	0	0	0	0	0	0	0
PPP1R18	32.622	45.314799	30.778637	58.050611	48.909192	94.117013	108.757726	61.471053
PPP1R1A	0	0	0	0	0	0	0	0
PPP1R1AP1	0	0	0	0	0	0	0	0
PPP1R1AP2	0	0	0	0	0	0	0	0
PPP1R1B	0	0.489345	0	1.21855	0	4.644736	0	1.394555
PPP1R1C	19.783004	1.517164	8.452286	3.748302	2.566647	5.221604	0	4.109147
PPP1R2	46.33791	121.01206	105.379775	103.706766	25.204616	90.921325	44.809797	89.584944
PPP1R21	14.951494	31.484777	27.18707	23.428098	9.048478	17.041432	0	21.601308
PPP1R26	66.412337	66.512782	39.147315	97.083885	25.048798	130.306463	16.349523	74.683637
PPP1R26P1	0	0	0	0	0	0	0	0
PPP1R26P2	0	0	0	0	0	0	0	0
PPP1R26P3	0	0	0	0	0	0	0	0
PPP1R26P4	0	0	0	0.023	0	0	0	0
PPP1R26P5	0	0	0	0	0	0	0	0
PPP1R27	0	0	0	0.121989	0	0	0	0
PPP1R2P1	0	0	0	0	0	0	0	0

PPP1R2P10	0	0	0	0	0	0	0	0
PPP1R2P2	0	0	0	0	0	0	0	0
PPP1R2P4	0	0	0	0	0	0	0	0
PPP1R2P5	0	0	0	0	0	0	0	0
PPP1R2P6	0	0	0	0	0	0	0	0
PPP1R35	12.132125	18.856487	7.321074	17.739108	16.360856	30.445712	20.816391	11.063924
PPP1R36	0	1.130346	0	0.261269	0	0.13239	0	0.109673
PPP1R37	15.27552	16.692322	12.26619	21.678827	26.35467	58.127892	0.496122	42.621192
PPP1R3A	0	0	0	0	0	0	0	0
PPP1R3B	10.059885	16.427345	6.381436	12.771783	2.970432	15.745833	4.469992	27.053117
PPP1R3C	1.337039	2.673371	3.589385	1.724898	6.455238	0.664412	8.435186	4.253057
PPP1R3D	4.555614	6.938134	2.451898	7.670245	0.721711	5.276972	2.659357	5.857003
PPP1R3E	5.923807	10.749442	5.27986	6.084903	6.165563	4.248893	29.394326	3.965911
PPP1R3F	0	0.803193	0	0.179529	0	0	0	0.58533
PPP1R42	0	0	0	0	0	0	0	0
PPP1R7	67.980561	105.662971	31.879656	92.965562	117.496673	168.348027	82.915886	149.426474
PPP1R8	31.658518	71.794513	12.957725	64.410889	61.636493	90.05848	39.106642	78.92074
PPP1R8P1	0	0	0	0	0	0	0	0
PPP1R9A	0	0.071474	0	0.562874	0.087647	0	0	0.09995
PPP1R9B	22.690454	80.503972	21.071158	76.423727	44.014252	195.781414	29.71005	136.239861
PPP2CA	124.390228	433.081495	130.250875	361.819287	131.806115	543.81807	111.434847	553.887552
PPP2CB	51.864566	125.161976	79.985964	62.329226	45.730368	45.364279	81.217634	107.341158
PPP2R1A	78.235054	55.543374	53.184082	67.045684	139.819298	97.921108	77.316426	108.222333
PPP2R1B	13.171621	13.474616	9.52098	12.152554	45.648887	21.552043	42.114875	30.723873
PPP2R2A	150.192649	179.028929	86.285146	141.031224	110.962092	82.489706	153.609957	135.434788
PPP2R2B	7.171108	45.709861	0.304328	35.362189	1.36643	5.650664	1.919064	4.684202
PPP2R2C	21.139157	67.973001	26.534199	68.033599	12.297133	36.790668	14.327696	30.738521
PPP2R2D	66.209838	67.241736	40.389491	58.018189	7.019016	42.156373	15.281212	52.867261
PPP2R2DP1	0	0	0	0	0	0	0	0
PPP2R3A	13.935155	24.702426	13.827125	19.133668	10.666072	18.011333	5.858111	28.158603
PPP2R3B	17.289604	3.177873	5.190519	1.854958	16.072001	7.187404	11.672555	4.873291
PPP2R3C	0	24.64663	6.695073	28.71612	28.192319	26.925886	20.570097	27.257041
PPP2R5A	31.548363	45.175308	36.749335	21.556734	11.958592	20.28527	26.707641	49.174963

PPP2R5B	19.102574	7.899786	4.434853	9.002427	13.808201	16.721788	3.006352	13.156603
PPP2R5C	124.251108	50.512757	52.846813	40.727912	62.519204	22.821095	191.989169	54.229066
PPP2R5CP	0	0	0	0	0	0	0	0
PPP2R5D	21.153256	24.473672	17.20998	30.169288	37.588195	66.266793	37.190981	52.084012
PPP2R5E	110.475412	117.322588	58.001861	62.343047	61.56496	58.59878	53.763363	117.469301
PPP3CA	86.59121	302.743755	45.542935	209.807463	50.175209	104.361196	89.495118	247.767199
PPP3CB	26.108289	23.289953	23.612568	17.407545	17.85335	9.990681	18.920648	16.346131
PPP3CC	28.93468	20.254375	31.943018	12.363611	5.313205	7.838828	0	8.164463
PPP3R1	117.238202	283.7628	91.47461	163.697354	162.070713	78.158674	139.964675	229.477171
PPP3R2	0	0	0	0	0	0	0	0
PPP4C	85.239363	274.535123	130.507783	271.36954	95.309014	313.793747	94.459391	258.368621
PPP4R1	52.603308	201.345544	175.285794	236.690555	92.343233	212.060575	145.092425	212.819571
PPP4R1L	0	0	0	0	0	0	0	0
PPP4R2	26.977096	149.18383	12.830453	92.453746	55.511256	71.354326	0	102.991518
PPP4R2P1	0	0	0	0.15326	0	0	0	0
PPP4R2P2	0	0	0	0	0	0	0	0
PPP4R2P3	0	0	2.784274	0	0	0	0	0
PPP4R2P4	0	0	0	0	0	0	0	0
PPP4R2P5	0	0	0	0	0	0	0	0
PPP4R2P6	0	0	0	0	0	0	0	0
PPP4R3A	19.051711	204.886242	15.424671	303.335061	136.44274	350.078859	71.684694	379.125109
PPP4R3B	61.259347	77.044996	40.553114	59.075477	83.240042	32.067335	24.730746	61.261743
PPP4R4	11.44599	40.901587	41.486212	53.017106	4.531555	15.777261	16.492444	20.039218
PPP5C	3.122415	7.664734	0	3.4684	36.95606	3.05025	21.85174	0.752071
PPP6C	0	24.623888	0	12.371209	5.606672	6.676997	19.812107	20.117164
PPP6R1	24.051736	57.049245	17.863467	62.998935	33.071673	156.466359	49.403067	119.795394
PPP6R2	24.88444	35.238196	12.5304	32.351208	15.306003	54.138968	11.370008	46.777715
PPP6R2P1	0	0	0	0	0	0	0	0
PPP6R3	144.453934	278.44686	137.63004	252.016214	274.554202	187.439403	281.209112	237.152324
PPRC1	36.761598	34.078678	34.683665	35.96688	30.970858	51.441539	26.074309	33.316709
PPT1	93.412755	43.87533	100.43749	64.425769	176.090032	63.725657	87.130033	52.368556
PPT2	12.04254	9.391528	11.684408	8.9039	32.168893	13.271046	92.7234	13.855145
PPT2-EGFL8	0	0.477529	0	0	0	0	0	0

PPTC7	15.07039	13.500482	16.481277	10.105964	24.059262	13.116649	19.790813	23.117403
PPWD1	29.46652	36.102941	38.023013	33.855479	38.225481	21.341734	46.636785	26.015723
PPY	0	0	0	0	0	0	0	0
PQBP1	0	3.040943	0	7.868765	4.901432	12.592216	59.617449	8.383537
PRAC1	154.810947	108.621984	141.980473	125.564395	138.481415	81.869586	3.501889	72.075809
PRAC2	58.521356	21.11155	7.198555	9.324785	2.543565	25.922384	0	24.944596
PRADC1	17.387071	18.131804	6.537708	15.551646	0	30.383031	0	36.668969
PRADC1P1	0	0	0	0	0	0	0	0
PRAF2	20.497504	16.351972	28.407772	24.649311	1.540562	7.837937	2.112676	7.434708
PRAM1	0	0	0	0	0	0	0	0
PRAME	0	0	0	0.383957	0	0	0	0
PRAMEF1	0	0	0	0	0	0	0	0
PRAMEF10	0	0	0	0	0	0	0	0
PRAMEF11	0	0	0	0	0	0	0	0
PRAMEF13	0	0	0	0	0	0	0	0
PRAMEF14	0	0	0	0	0	0	0	0
PRAMEF15	0	0	0	0	0	0	0	0
PRAMEF17	0	0	0	0	0	0	0	0
PRAMEF18	0	0	0	0	0	0	0	0
PRAMEF2	0	0	0	0	0	0	0	0
PRAMEF22	0	0	0	0	0	0	0	0
PRAMEF25	0	0	0	0	0	0	0	0
PRAMEF28P	0	0	0	0	0	0	0	0
PRAMEF29P	0	0	0	0	0	0	0	0
PRAMEF30P	0	0	0	0	0	0	0	0
PRAMEF31P	0	0	0	0	0	0	0	0
PRAMEF32P	0	0	0	0	0	0	0	0
PRAMEF33	0	0	0	0	0	0	0	0
PRAMEF34P	0	0	0	0	0	0	0	0
PRAMEF35P	0	0	0	0	0	0	0	0
PRAMEF36P	0	0	0	0	0	0	0	0
PRAMEF5	0	0	0	0	0	0	0	0
PRAMEF6	0	0	0	0.033154	0	0	0	0

PRAMEF7	0	0	0	0	0	0	0	0
PRAMEF8	0	0	0	0	0	0	0	0
PRAMEF9	0	0	0	0	0	0	0	0
PRAMENP	0	0	0	0	0	0	0	0
PRAP1	0	0	0	0	0	0	0	0.325039
PRB1	0	0	0	0	0	0	0	0
PRB2	0	0	0	0	0	0	0	0
PRB3	0	2.829635	0	2.641127	0.947045	0.953078	0	0.839387
PRB4	0	0	0	0	0	0	0	0
PRC1	52.5919	119.842209	67.580624	152.574343	154.510001	265.161791	217.47082	209.88939
PRCC	101.198082	111.955372	57.117182	132.613813	91.633556	167.856798	172.202276	112.240246
PRCD	0	0.376169	0	1.269005	0	0	0	0
PRCP	81.056071	28.455156	75.964314	28.897217	49.412735	12.828954	25.630874	18.646361
PRCPP1	0	0	0	0	0	0	0	0
PRDM1	2.807088	10.726029	7.415507	12.677707	2.663565	11.448868	6.930308	7.175957
PRDM10	0.923773	0	2.514491	3.925717	3.887548	13.21998	8.590687	9.56382
PRDM11	4.725994	2.455929	1.076097	1.508922	1.185012	8.726875	0	4.741894
PRDM13	2.067929	1.987294	0.926845	1.537942	0	0.773308	0	0.415679
PRDM14	0	0	0	0	0	0	0	0
PRDM15	18.41032	0	5.462231	0.923909	0	7.239734	0	7.888115
PRDM16	0.598841	0.851258	1.59784	1.818059	21.800475	12.910057	16.576234	6.381001
PRDM2	14.221806	28.746098	14.131159	28.336752	23.439872	52.033903	36.811084	37.376222
PRDM4	20.4958	47.144913	23.09815	46.373645	55.220945	102.978628	38.94994	90.114468
PRDM5	13.536598	10.151977	7.036463	12.600899	0.736027	12.182757	2.084404	9.592128
PRDM6	0	0.060621	0	0.582331	0	0.053002	0	0.042263
PRDM7	0	0	0	0	0	0	0	0
PRDM8	2.705061	5.938971	0	2.362786	0	0.099446	0	1.376046
PRDM9	0	0	0	0	0	0	0	0
PRDX1	1047.89782	824.152795	754.991555	768.737206	809.840312	562.239373	979.711905	735.74015
PRDX1P1	0	0	0	0	0	0	0	0
PRDX2	108.292563	134.361769	119.753972	141.459371	313.460103	362.849239	369.430304	285.855106
PRDX2P1	0	0	0	0	0	0	0	0
PRDX2P2	0	0	0	0	0	0	0	0

PRDX2P3	0	0	0	0	0	0	0	0
PRDX2P4	0	0	0	0	0	0	0	0
PRDX3	168.023293	78.2725	144.627586	47.097764	124.265017	36.100052	81.610095	61.524943
PRDX3P1	0	0	0	0	0	0	0	0
PRDX3P2	0	0	0	0	0	0	0	0
PRDX3P3	0	0	0	0	0	0	0	0
PRDX3P4	0	0	0	0	0	0	0	0
PRDX4	102.017281	117.549367	129.446164	82.147677	159.436595	68.876728	52.874197	114.740543
PRDX4P1	0	0	0	0	0	0	0	0
PRDX5	240.212756	382.053269	213.451756	420.122578	170.487367	493.997437	297.882457	333.417789
PRDX5P1	0	0	0	0	0	0	0	0
PRDX6	396.244132	250.988918	247.712375	220.393345	320.361356	202.820352	238.912856	151.34228
PREB	24.327999	58.875996	18.837036	54.916681	31.21712	74.540343	25.932308	70.514869
PRELID1	148.734253	154.096994	104.660961	161.592144	121.675546	173.251771	138.098328	171.355436
PRELID1P1	0	0	0	0	0	0.328145	1.844555	0
PRELID1P2	0	0	0	0	0	0	0	0
PRELID1P3	0	0	0	0	0	0	0	0
PRELID1P4	0	0	0	0.445411	0	0	0	0
PRELID1P5	0	0	0	0	0	0.311819	0	0
PRELID1P6	0	9.686124	13.279021	9.106389	0	4.532071	26.5255	10.83509
PRELID1P7	0	0	0	0	0	0	0	0
PRELID2	17.837565	30.612066	25.296587	32.358444	13.529401	24.50745	0	40.908523
PRELID2P1	0	0	0	0	0	0	0	0
PRELID3A	2.100729	6.739731	17.622406	6.49074	19.471259	2.795974	0	2.250394
PRELID3B	316.891796	144.165447	176.090218	123.720556	168.161655	69.090654	117.001114	113.155275
PRELID3BP1	0	0	0	0	0	0	0	0
PRELID3BP10	0	0	0	0	0	0	0	0
PRELID3BP11	0	0	0	0	0	0	0	0
PRELID3BP2	0	0	0	0	0	0	0	0
PRELID3BP3	0	0	0	0	0	0	0	0
PRELID3BP4	0	0	0	0	0	0	0	0
PRELID3BP5	0	0	9.606027	0	0	0	0	0
PRELID3BP6	0	0	0	0	0	0	0	0

PRELID3BP7	0	0	0	0	0	0	0	0
PRELID3BP8	0	0	0	0	0	0	0	0
PRELP	0	0	0	0	0	0	0	0
PREP	33.670844	46.481918	40.298638	38.498714	17.614079	27.435432	5.390082	51.09063
PREPL	29.9567	47.680428	31.221919	25.853659	27.096225	12.643738	20.921053	38.528537
PREX1	5.030425	4.273733	3.020551	4.143736	0.634016	2.431544	0.170629	1.904456
PREX2	0	0	0	0.007254	0	0	0	0
PRF1	0	0	0	0	0	0	0	0
PRG2	0	0	0	0	0	0	0	0.547889
PRG3	0	0	0	0	0	0	0	0
PRG4	0	0	0	0.016809	0	0	0	0
PRH1	8.973927	5.721441	21.104004	7.326142	3.922185	4.548674	0	7.429814
PRH2	0	0	0	0.540649	0	0	0	1.427186
PRICKLE1	4.049877	12.81592	0.903566	5.086228	0	0.351863	0	0.445637
PRICKLE1P1	0	0	0	0	0	0	0	0
PRICKLE2	0	0	0	0.029919	0.195311	1.18818	0	3.186282
PRICKLE3	43.227038	27.71124	31.492481	26.421893	19.82016	23.123508	16.136261	21.582415
PRICKLE4	0	0	0	0	0	0.243178	0	1.030172
PRIM1	112.134069	68.869196	85.755679	50.172162	31.840078	14.312768	186.999741	25.300552
PRIM2	29.743663	18.879908	22.604692	13.028213	28.242235	10.174665	14.782876	19.08461
PRIM2BP	0	0	0	0	0	0	0	0
PRIMA1	0	0.272381	0	0.488275	0	1.158895	0	0.767394
PRIMPOL	19.592972	38.281459	19.377996	35.06421	10.866764	26.805986	4.18521	30.122871
PRKAA1	49.199877	38.550751	32.825592	52.620958	29.343648	47.533016	23.425468	38.821268
PRKAA2	11.6848	6.58624	9.278342	4.32487	0	0.742534	8.637526	1.872387
PRKAB1	13.937026	15.536077	12.45674	19.083117	44.810982	21.59201	31.525919	20.973865
PRKAB2	10.996416	9.769215	13.723484	9.674615	7.746872	8.761962	7.739911	10.394377
PRKACA	0	2.71292	7.199923	4.193755	0	8.617664	0.399431	6.513654
PRKACB	29.161977	51.493321	14.411654	24.118706	14.766378	8.956095	29.47207	28.835916
PRKAG1	30.410513	5.60145	18.733439	6.90318	24.965958	7.90277	13.20522	1.610725
PRKAG2	16.244048	25.582312	19.713605	13.535295	27.370927	15.618088	10.612007	17.115978
PRKAG3	0	0	0	0	0	0	0	0
PRKAR1A	75.886223	169.590399	126.197841	147.588119	167.278569	117.491521	199.707795	127.8267

PRKAR1AP1	0	0	0	0	0	0	0	0
PRKAR1B	52.914627	65.343318	58.592006	75.745193	36.003283	75.793967	39.475646	59.574532
PRKAR2A	23.483161	79.559535	26.954468	52.859124	32.289217	47.811716	96.987936	65.129416
PRKAR2B	0	0	0.806629	0.241366	0	0.0449	0	0.180618
PRKCA	28.000017	35.450746	30.041078	32.487524	14.404752	12.156709	4.028967	15.369788
PRKCB	0	0	0	0.335357	0	0.481522	0	0.050272
PRKCD	20.106574	50.374706	12.0152	45.364934	29.301121	51.917152	79.803994	41.226806
PRKCE	3.964419	37.329747	18.986235	24.661261	1.346686	51.551998	3.450522	29.468832
PRKCG	0	0.181524	0	0.07774	0	0	0	0
PRKCH	0	12.343053	30.165713	29.778086	0	38.8386	15.810806	11.684118
PRKCI	81.408348	145.190263	110.818753	56.556082	79.264614	33.717073	245.711186	165.972122
PRKCIP1	0	0.177105	0	0	0	0	0	0
PRKCQ	3.246742	0.693011	0	0	0	0	0	0.267117
PRKCSH	63.516651	65.439044	24.339101	74.784419	46.789858	102.967912	121.43614	32.649725
PRKCZ	18.596265	38.00352	61.7199	42.548517	42.139759	57.920151	0	47.026164
PRKD1	8.196432	4.563808	3.673832	1.234526	0.311378	0.79316	0	1.168989
PRKD2	5.034345	52.80721	31.302958	58.260768	109.85183	104.978058	0	74.48367
PRKD3	24.992605	76.810042	10.214393	75.880567	0.91882	82.19004	86.923889	91.466101
PRKDC	0	45.667337	35.659008	32.516643	14.273461	17.052097	0	18.278233
PRKG1	0	0.861381	0	0.502992	0	0.083361	0	0.451306
PRKG2	12.721096	5.881744	7.710449	10.003258	0	0.213134	0	0.276092
PRKN	0	0.202872	0	0.154147	0	0	0	0
PRKRA	19.242019	22.541801	32.700874	13.431104	27.721637	13.153773	19.525613	17.877922
PRKRIP1	19.754651	16.202433	11.86762	24.926889	7.143222	39.126652	19.534733	25.797598
PRKX	11.199522	20.591258	9.512367	16.523168	43.949084	48.512853	64.268885	50.069928
PRKXP1	0	0.696031	0	0.808145	0.882856	0	0	0.273334
PRKY	0	3.234018	0	2.704118	19.222511	5.370154	0	6.359206
PRL	0	0	0	0	0	0	0	0
PRLH	0	0	0	0	0	0	0	0
PRLHR	0	0	0	0	0	0	0	0
PRLR	0	0	0	0	0	0	0	0
PRM1	0	0	0	0	0	0	0	0
PRM2	0	0	0	0	0	0	0	0

PRMT1	99.691967	160.072914	136.389606	184.298358	278.344085	302.868219	130.830716	271.001233
PRMT1P1	0	0	0	0	0	0	0	0
PRMT2	8.855508	137.070568	43.727507	136.722503	92.608168	246.677543	47.520987	242.507455
PRMT3	34.516153	25.185993	55.8113	18.468138	46.477843	18.57959	33.872163	44.0688
PRMT5	116.150589	94.603075	86.058837	117.903138	235.44795	145.253782	310.796665	121.066178
PRMT5P1	0	0	0	0	0	0	0	0
PRMT6	0	2.037351	0	0.586936	0	0	0	0
PRMT7	41.071608	43.126701	0	29.411724	42.28647	19.925385	67.531533	20.143273
PRMT8	0	0	0	0	0	0.055291	0	0.066348
PRMT9	3.447752	3.758022	7.696257	2.909082	6.817252	1.439708	0	5.269013
PRND	0	0	0	0	0	0	0	0
PRNP	130.981906	71.002596	140.914561	44.147123	121.8018	13.234175	96.66907	30.120413
PROC	0	0.428199	0	1.298301	1.386193	7.082882	0	0
PROCA1	0	3.848006	0	4.100064	0	0.699666	0	0.445059
PROCR	64.910203	40.466399	55.237108	51.735396	45.551962	21.050049	100.462598	12.941434
PRODH	0	0	0	0.207129	0	1.234782	0	2.028837
PRODH2	0	0	0	0	0	0	0	0
PRODHLP	0	0	0	0	0	0	0	0
PROK1	0	0	0	0.251906	0	0	0	0
PROK2	0	0	0	0	0	0	0	0
PROM1	0	0	0	0.330268	0	0	0	0
PROM2	7.122654	3.428789	2.367477	1.439446	144.760044	39.159895	27.143159	19.074162
PROP1	0	0	0	0	0	0	0	0
PRORP	1.662634	19.595069	2.733227	16.643986	43.896371	31.078736	133.253705	49.827828
PRORSD1P	0	3.93557	4.287982	4.393256	1.265482	1.981494	0	3.998822
PROS1	4.393863	5.691217	8.070515	4.536892	3.769158	3.219092	8.521128	0.95302
PROS2P	0	0	0	0	0	0	0	0
PROSER1	21.147934	26.267936	15.29705	40.663301	9.995137	16.372833	14.362247	23.122244
PROSER2	6.913444	9.019491	12.397668	17.768439	13.558239	19.585359	0	12.033578
PROSER3	15.138919	23.989624	6.64721	28.121061	19.237093	36.209758	34.523021	29.968035
PROX1	0	0.707526	0	1.258545	0	0.757341	1.953034	0.867741
PROX2	0	0	0	0	0	0	0	0
PROZ	0	0	0	0	0	0	0	0

PRP4K	8.129226	9.814243	8.170107	8.526728	17.743142	12.296766	39.897674	18.961254
PRPF18	0	27.381076	11.892773	24.484846	1.365762	17.053906	0	21.502442
PRPF19	65.866595	100.335294	61.51677	123.955209	126.011448	250.905357	100.840618	202.724496
PRPF19P1	0	0	0	0	0	0	0	0
PRPF3	21.197563	22.11708	22.583083	33.703011	43.242842	41.090121	49.520597	29.343385
PRPF31	43.952037	56.516423	23.862912	61.776946	78.851104	132.006042	48.377413	89.60453
PRPF38A	118.632362	245.819127	89.958994	237.528732	27.004269	95.99771	59.484479	114.255592
PRPF38AP1	0	0	0	0	0	0	0	0
PRPF38AP2	0	0	0	0	0	0	0	0
PRPF38B	30.498426	28.500734	25.675412	37.398951	26.386072	22.906014	32.420469	16.761836
PRPF39	18.053626	9.470256	2.112588	11.109435	0	9.062255	0	9.594751
PRPF4	0	0	0	0	0	0	0	0
PRPF40A	0	4.046752	7.429633	8.330487	0	11.591193	0	6.902882
PRPF40B	0	2.699408	3.807487	2.682993	32.959969	2.354148	0	0.626699
PRPF4BP1	0	0	0	0	0	0	0	0
PRPF6	48.433768	50.638056	46.358573	61.779553	44.162308	96.744664	74.352377	62.22365
PRPF8	93.27369	70.9069	96.674801	87.893362	284.208684	133.860493	150.580016	116.571324
PRPH	0	2.286291	0	3.091914	0	0.229773	0	0.568333
PRPH2	0	0	0	0	0	0	0	0
PRPS1	0	71.148367	0	81.606461	0	43.218616	0	48.315592
PRPS1P1	0	0	0	0	0	0	0	0
PRPS1P2	0	0.171581	0	0	0	0	0	0
PRPS2	0	27.028306	0	54.179754	0	104.861726	0	52.593919
PRPSAP1	43.467424	65.327218	33.021352	71.499771	0	68.589528	27.516906	88.755173
PRPSAP2	22.229043	80.694569	32.583147	41.77183	56.88165	85.58002	29.655233	100.798073
PRR11	43.574886	38.387041	20.984432	39.948965	60.891668	40.311788	93.586419	50.33027
PRR11P1	0	0	0	0	0	0	0	0
PRR12	6.665122	9.965253	7.055808	11.986698	13.244658	32.86864	13.710989	17.17443
PRR13	129.990539	119.454472	174.485516	134.784784	69.226227	80.646973	154.928063	61.272174
PRR13P1	0	0	0	0	0	0	0	0
PRR13P2	0	0	11.684775	0.318753	0	0	0	0
PRR13P3	0	0.576658	0	0	0	0	0	0
PRR13P4	0	0	0	0	0	0	0	0

PRR13P5	0	0	0	0.894666	0	0	0	0
PRR13P7	0	0	0	0	0	0	0	0
PRR14	0	17.485752	0	11.527953	10.989564	13.419614	17.918905	8.066555
PRR14L	16.136896	39.964106	17.413375	75.077166	30.495054	44.943933	88.240013	68.742393
PRR15	4.293018	45.719993	7.648202	41.320414	4.523718	5.29159	0.518487	6.095597
PRR15-DT	0	0	0	1.306874	0	0	0	1.071261
PRR15L	0	0	0	0	0	0	0	0.475901
PRR16	14.141829	27.604422	7.375301	24.152974	3.42268	10.397406	0	19.239917
PRR18	1.083363	0.277452	0	1.056366	0	0.107974	0	0.043565
PRR19	0	0	0	0	0	0	0	0
PRR20A	0	0	0	0	0	0	0	0
PRR20FP	0	0	0	0	0	0	0	0
PRR22	0	0	0	0.064019	0	0	0	0.21486
PRR23B	0	0	0	0	0	0	0	0
PRR23C	0	0	0	0	0	0	0	0
PRR23D2	0	0	0	0.054383	0	0	0	0
PRR23D3P	0	0	0	0	0	0	0	0
PRR23E	0	0	0	0	0	0	0	0
PRR23E2P	0	0	0	0	0	0	0	0
PRR27	0	0	0	0	0	0	0	0
PRR29	0	0	0	1.036815	0	0.49156	0	0.325937
PRR3	17.77265	11.345021	7.63227	12.343734	16.751673	12.415361	0	5.322892
PRR30	0	0	0	0	0	0	0	0
PRR32	0	0	0	0	0	0	0	0
PRR35	0	0.194654	0	0.111532	0	0	0	0
PRR36	0.73585	0.44174	2.642933	1.599681	1.166129	0.736238	0	0.177226
PRR3P1	0	0	0	0	0	0	0	0
PRR4	0	7.30877	0	5.515436	0	4.001748	0	3.122997
PRR5	0	7.1363	8.327687	8.336306	9.319952	15.499463	0	11.678766
PRR5-ARHG/	3.18948	0.463943	0	3.230022	0	1.249792	0.984845	2.186387
PRR5L	26.593565	44.767353	2.313516	41.739401	49.400753	179.937654	27.288151	173.381846
PRR7	2.467459	16.032571	3.434934	14.322317	11.293337	21.149841	11.01593	20.204561
PRR9	0	0	0	0	0	0	0	0

PRRC1	21.523516	21.949739	49.171487	28.063047	0	22.941199	10.742561	17.330424
PRRC2A	87.469833	183.190713	69.261424	222.249772	103.252411	461.987785	131.713245	266.518448
PRRC2C	97.311964	178.343137	112.506024	299.385225	161.580037	331.490505	258.126075	263.730154
PRRC2CP1	0	0	0	0	0	0	0	0
PRRG1	23.65826	15.633863	0	19.016262	24.215306	51.803749	0	42.971488
PRRG2	7.361119	3.181026	0	4.41579	4.762665	4.687652	0	4.085303
PRRG3	0	0	0	0	0	0	0	0
PRRG4	8.2336	8.894154	7.927843	7.303963	23.772671	19.951087	14.904071	23.212143
PRRT1	0	9.540399	2.389498	5.484489	0	4.039289	0	1.538485
PRRT2	0	0.876774	0	0.292382	5.896158	0.280952	0	0.335507
PRRT3	1.75527	2.116754	1.57482	4.297957	0	1.303368	12.360204	1.799903
PRRT4	2.960398	1.95548	4.382544	3.585093	0	1.377946	0	1.026762
PRRX1	0	0	0	0.171245	0	0	0	0
PRRX2	5.621421	1.876954	7.483067	2.776464	5.916249	2.473658	0	3.87733
PRSS1	0	0	0	0.124342	0	0	0	0
PRSS12	15.680191	33.455729	29.542393	22.563452	20.549772	31.330141	13.111026	18.085363
PRSS16	10.712365	6.497968	20.567923	11.533921	18.241384	16.513277	11.254788	10.038355
PRSS2	0	0	0	0	0	0	0	0
PRSS21	23.893659	20.293161	24.330577	22.496997	0	0.299424	0	0
PRSS22	10.352247	3.92231	5.733441	2.503506	13.582675	2.608147	0	0.888258
PRSS23	656.283533	2566.42575	573.782605	1929.46248	144.964181	448.334639	157.807871	500.577904
PRSS27	0	0.236244	0	0.478229	0	0.616537	0	0.574353
PRSS3	7.4202	0	0	0.400608	24.436676	1.069505	0	4.918869
PRSS30P	0	0	0	0.057492	0	0	0	0
PRSS33	0	0	0	0.204718	0	0	0	0
PRSS35	0	0	0	0	0	0.069248	0	0.112326
PRSS36	0	1.203979	7.829892	1.967063	0	0.423176	0	0.645947
PRSS37	0	0	0	0	0	0	0	0
PRSS3P1	0	0	0	0	0	0	0	0
PRSS3P2	0	0	0	0	0	0	0	0
PRSS3P3	0	0	0	0	0	0	0	0
PRSS3P4	0	0	0	0	0	0	0	0
PRSS40A	0	0	0	0	0	0	0	0

PRSS40B	0	0	0	0	0	0	0	0
PRSS42P	0	0	0	0.581873	0	0	0	0
PRSS46P	0	0	0	0	0	0	0	0
PRSS48	0	0	0	0	0	0	0	0
PRSS50	0	0	0	0	0	0	0	0
PRSS51	0	0	0	0	0	0	0	0
PRSS52P	0	0	0	0	0	0	0	0
PRSS53	0	0.339549	0.533884	0.426182	3.637497	0.289882	0	0.228009
PRSS54	0	0	0	0.223212	0	0	0	0
PRSS55	0	0	0	0	0	0	0	0
PRSS56	0	0	0	0	0	0	0	0
PRSS57	0	0	0	0	0	0	0	0
PRSS58	0	0	0	0	0	0	0	0
PRSS59P	0	0	0	0	0	0	0	0
PRSS8	20.969686	36.788109	37.409119	60.992762	12.560439	32.346452	114.785691	22.280952
PRTFDC1	0	2.500386	0	1.745707	0	1.333944	0	0.931194
PRTG	0.263575	2.466903	0.237307	3.790479	0	1.616478	0	0.292232
PRTN3	7.846469	0	0	1.223182	0	0.379039	0	0
PRUNE1	23.067884	22.114405	28.006469	27.215629	24.914045	31.165594	9.150677	35.890217
PRUNE1P1	0	0	0	0	0	0	0	0
PRUNE2	0	0.824098	0	0.051985	0	0.109106	0	1.085055
PRX	0	0	0	0.052578	0	0.607568	0	0.413467
PRXL2A	78.156584	154.264292	130.609681	151.421445	49.878107	88.464194	29.737523	91.071511
PRXL2AP1	0	0	0	0	0	0	0	0
PRXL2AP2	0	0	0	0	0	0	0	0
PRXL2B	6.785947	11.521737	10.607398	11.626982	32.188562	26.641787	25.475694	20.488268
PRXL2C	10.446872	18.360284	11.437345	9.454003	10.704799	5.247265	15.751703	11.131847
PRXL2CP1	0	0	0	0	0	0	0	0
PRY	0	0	0	0	0	0	0	0
PRY2	0	0	0	0	0	0	0	0
PRYP1	0	0	0	0	0	0	0	0
PRYP2	0	0	0	0	0	0	0	0
PRYP4	0	0	0	0	0	0	0	0.509788

PRYP5	0	0	0	0	0	0	0	0
PRYP6	0	0	0	0	0	0	0	0
PSAP	194.199832	233.00213	549.719478	431.092335	242.473393	262.260942	189.651329	143.475915
PSAPL1	0	0.272691	0	0	0	0.070694	0	0.028349
PSAT1	137.149102	133.85578	130.932656	125.873455	65.351001	45.657982	73.651486	54.744174
PSAT1P1	0	0	0	0	0	0	0	0
PSAT1P2	0	0	0	0	0	0	0	0
PSAT1P3	0	0	0	0.163509	0	0	0	0
PSAT1P4	0	0	0	0	0	0	0	0
PSCA	0	1.786352	0	0.094667	0	0.762621	0	0
PSD	0	1.768948	0	2.723314	0	1.486368	0	0.637406
PSD2	0	0	0	0.668204	0	0	0	0.029135
PSD3	16.98181	29.654124	15.866474	24.08279	25.801295	14.912919	5.215114	21.237921
PSD4	2.554765	20.433293	3.942211	13.875077	7.804442	70.86138	5.569848	75.926074
PSEN1	26.836827	42.672027	47.774872	80.191023	32.462268	70.226192	21.846428	55.047602
PSEN2	12.776607	14.059577	5.456608	16.356417	16.404765	19.501684	0	12.365297
PSENEN	53.627031	63.877911	39.237617	78.812476	16.738806	64.712053	24.25133	75.910318
PSG1	0	0	0	0	0	0.27508	0	1.040035
PSG10P	0	0	0	0	0	0	0	0
PSG11	0	0	0	0.135448	0	0	0	0
PSG2	0	0	0	0	0	0	0	0
PSG3	0	0	0	0	0	0	0	0.170876
PSG4	0	0.156386	3.884203	0.290398	15.65789	16.942196	0	12.77728
PSG5	0	0.394331	0	0	8.398037	2.419066	0	1.066599
PSG6	0	0	0	0	0	0.757029	0	0
PSG7	0	0	0	0	0	0.551156	0	0.422871
PSG8	0	0	0	0	0	0	0	0
PSG9	0	0	0	0	0	0.397493	0	0
PSIP1	91.620892	119.459323	95.020646	119.851458	102.394041	121.824585	108.085051	119.74024
PSIP1P1	0	0	0	0.286368	0	0	0	0
PSKH1	7.61016	34.562188	13.649887	28.987255	10.548383	27.214008	2.081932	16.749606
PSKH2	0	0	0	0	0	0	0	0
PSMA1	137.83274	61.482708	45.332578	44.817925	32.880741	58.71192	241.676605	94.556833

PSMA1P1	0	0	0	0	0	0	0	0
PSMA2	148.824379	61.592689	85.729845	36.944722	175.7079	44.841634	87.319246	136.44498
PSMA2P1	0	0	0	0	0	0	0	0
PSMA2P2	0	0	0	0	0	0	0	0
PSMA2P3	0	0	0	0	0	0	0	0
PSMA3	228.814946	235.067215	245.063238	136.645479	278.852374	90.532277	390.29867	304.953189
PSMA3P1	0	0	0	0	0	0	0	0
PSMA4	277.398443	264.615394	257.9085	175.177434	293.23597	172.887483	246.644898	444.962502
PSMA5	239.220805	271.68314	165.705352	231.799067	139.242475	154.451191	288.774288	116.534612
PSMA5P1	0	0	0	0	0	0	0	0
PSMA6	130.469949	2.841046	13.560287	41.124618	275.93102	56.316421	520.561734	23.596378
PSMA6P1	0	0	4.938614	0	0	0	0	0
PSMA6P2	0	0	0	0	0	0	0	0
PSMA6P3	0	0	0	0	0	0	0	0
PSMA6P4	0	0	0	0.139856	0	0	0	0
PSMA7	396.825219	1163.48877	349.193548	795.575451	450.3044	1236.63999	244.845978	1431.54604
PSMA8	0	0	0	0	0	0	0	0
PSMB1	218.325304	194.676278	231.593613	134.33573	379.823473	112.351297	296.436456	253.373739
PSMB10	39.818359	35.668096	38.529332	29.904943	70.924389	71.369765	50.712181	40.803532
PSMB2	105.310208	83.424065	112.41915	76.682545	119.87903	130.378336	205.14629	138.985208
PSMB3	365.852063	261.171091	214.396963	319.468752	394.028569	207.926037	210.644454	233.043277
PSMB3P1	0	0	0	0	0	0	0	0
PSMB3P2	0	0	0	0	0	0	0	0
PSMB4	266.093728	153.924641	309.72436	175.026744	231.969214	134.852703	246.871312	157.90373
PSMB5	67.108001	30.651564	39.477517	22.021964	18.56454	14.816391	0	44.522348
PSMB6	216.550429	104.960828	94.954221	84.162253	180.57165	138.303376	135.791187	112.511449
PSMB7	311.38811	195.135477	194.471624	208.995633	308.417655	218.539222	328.671164	224.199211
PSMB7P1	0	0	0	0	0	0	0	0
PSMB8	58.60717	55.898844	46.386591	69.236349	81.932052	100.517004	95.885896	83.291709
PSMB9	49.166129	42.809809	51.134093	47.994707	95.635428	83.749849	34.567264	60.50511
PSMC1	282.456658	455.605808	225.206371	374.623352	383.634689	567.404267	291.81311	445.75292
PSMC1P1	0	0	0	10.225385	0	34.100635	0	45.06818
PSMC1P10	0	0	0	0	0	0	0	0

PSMC1P11	0	0	0	0	0	0	0	0
PSMC1P12	0	0	0	0	0	0	0	0
PSMC1P13	0	0	0	0	0	0	0	0
PSMC1P2	0	0	0	0	0	0	0	0
PSMC1P3	0	0	0	0	0	0	0	0
PSMC1P4	0	0	0	0	0	0	0	0
PSMC1P5	0	0	0	0	0	0	0	0
PSMC1P6	0	0	0	0	0	0	0	0
PSMC1P7	0	0	0	0	0	0	0	0
PSMC1P8	0	0	0	0	0	0	0	0
PSMC1P9	0	0	0	0	0	0	0	0
PSMC2	58.770995	49.517849	22.322304	24.719017	71.381694	25.974454	86.858361	41.552378
PSMC2P1	0	0.118213	0	0.339397	0	0.414141	0	0.114137
PSMC2P2	0	0	0	0	0	0	0	0
PSMC3	84.800578	181.301264	80.375319	151.655341	73.821089	438.778254	183.297143	269.053236
PSMC3IP	18.426191	39.381227	0	25.036645	33.255392	34.204283	37.11802	22.382604
PSMC3P1	0	0	0	0	0	0	0	0
PSMC4	0	0	0	5.599899	0	4.301344	84.444855	8.679021
PSMC5	137.351016	318.202631	125.738134	298.764878	112.765916	306.547575	208.976008	294.436096
PSMC6	87.773232	123.481614	91.782702	116.449214	105.545002	88.505409	183.750686	130.26787
PSMD1	0	53.431586	87.268002	39.49042	70.55981	54.957018	80.788617	95.964637
PSMD10	34.302745	47.042504	45.755503	47.785722	121.618666	41.819027	47.857839	51.130695
PSMD10P1	0	0	0	0.149885	0	0	0	0
PSMD10P2	0	3.1844	0	7.547567	0	0.303423	0	1.333749
PSMD10P3	0	0	0	0	0	0	0	0
PSMD11	199.442633	172.776495	109.569329	143.410738	242.691623	121.553754	399.868963	179.65656
PSMD12	84.63313	110.418282	111.340167	61.142396	120.807518	67.960354	62.122374	126.374384
PSMD12P1	0	0	0	0	0	0	0	0
PSMD13	123.350163	113.240428	87.853828	96.204086	139.419375	120.811819	155.770361	158.426114
PSMD14	66.073482	115.969483	62.868707	74.265522	112.811693	112.808855	160.996651	237.470631
PSMD14P1	0	0	0	0	0	0	0	0
PSMD2	306.961441	435.240794	221.3745	334.525514	313.672756	523.907718	317.459489	447.114527
PSMD3	81.722109	141.24831	74.587593	126.424394	77.045476	186.72142	82.067914	155.398672

PSMD4	54.343711	65.115004	0	26.076756	0	30.602502	0	22.529222
PSMD4P1	0	0.282154	0	0.081928	0	0	0	0
PSMD5	53.03458	22.07654	35.355812	22.240431	64.375908	17.603341	107.683627	31.172871
PSMD6	0	26.407161	16.482361	22.76882	39.015011	33.594171	0	21.740519
PSMD7	201.272461	302.539838	162.913891	286.980784	264.669335	299.232624	360.036082	309.741624
PSMD7P1	0	0	0	0	0	0	0	0
PSMD8	101.728411	164.747169	68.342438	147.428157	129.503955	281.980924	161.499816	227.790445
PSMD8P1	0	0	0	0	0	0	0	0
PSMD9	43.629638	66.320498	43.044607	67.833695	37.968535	83.410115	25.455752	75.022477
PSME1	177.865083	233.853218	138.65458	291.53548	237.283799	559.503735	196.273664	338.117222
PSME2	322.681473	287.158045	232.236019	306.500095	478.169544	500.706105	295.672452	353.25118
PSME2P1	5.875132	0.711275	0	0.979685	0	3.937502	0	0.484498
PSME2P2	0	0.492243	0	0.144649	0	1.737153	0	0.50455
PSME2P3	0	0	0	0	0	0	0	0
PSME2P4	0	0	0	0	0	0	0	0
PSME2P5	0	0	0	0	0	0	0	0.211703
PSME2P6	0	0	0	0	0	0	0	0
PSME3	131.215455	136.755199	47.30204	107.381142	83.298117	125.119783	202.30053	99.500345
PSME3IP1	81.993374	178.240717	179.921029	188.94447	199.402665	173.764392	115.824362	131.65415
PSME4	43.992468	65.478343	40.518606	37.183455	58.465621	30.468604	65.465859	72.638375
PSMF1	168.932829	262.115823	89.910017	262.208793	115.985437	396.987184	173.230161	337.177791
PSMG1	92.275171	101.448806	97.615981	66.761577	190.848159	77.54897	198.424051	158.27487
PSMG2	41.490227	56.003726	65.372554	29.36066	33.654328	24.937971	0	50.014999
PSMG3	78.377401	172.252432	84.059445	151.037574	77.797396	62.153704	0	57.919409
PSMG3P1	0	0	0	0	0	0	0	0
PSMG3P2	0	0	0	0	0	0	0	0
PSMG4	30.057892	13.683301	2.738821	11.157465	9.417309	6.895644	18.29307	11.019018
PSORS1C1	4.171809	16.209109	27.64794	12.152609	0	11.085676	0	14.113178
PSORS1C2	0	0	0	0	0	0	0	0
PSPC1	38.365175	41.454491	33.647761	48.340445	0	39.286068	18.728522	43.947169
PSPC1P1	0	0	0	0	0	0	84.875996	0
PSPC1P2	0	0	0	0	0	0	0	0
PSPH	14.223536	73.961263	18.539402	83.442159	68.061732	90.155282	65.282482	158.576202

PSPHP1	0	0	0	0	0	0	0	0
PSPN	1.691226	1.774142	0.758818	1.238514	1.116502	0.380261	0	0.71322
PSRC1	46.998285	28.754073	18.777928	33.576746	2.883592	35.729158	12.496957	25.515432
PSTK	0	5.14863	0	2.639353	1.962618	1.939696	0	2.736122
PSTPIP1	0	0	0	0	0	1.536617	0	1.701022
PSTPIP2	5.595685	11.363657	11.029791	11.742154	13.58748	15.856763	6.200338	12.391228
PTAFR	13.043241	12.543183	8.16817	19.545808	8.525553	13.516402	3.825588	8.893881
PTAR1	30.956339	21.210809	8.160108	21.700672	24.184913	24.319125	9.364483	27.14491
PTBP1	155.842594	215.097171	221.264457	281.480963	287.701246	414.332201	392.841356	268.973141
PTBP1P	0	0	0	0.107706	0	0	0	0
PTBP2	0.315701	6.667949	11.956325	4.073897	0	1.771239	0	1.829338
PTBP3	46.666098	89.871662	31.782997	64.233632	20.802258	43.654131	81.986255	107.805722
PTCD1	0	4.827341	0	0	0	1.282802	58.953021	0.546533
PTCD2	27.154982	7.133505	9.169371	8.161981	13.328056	4.195882	3.997561	9.647437
PTCD2P1	0	0	0	0	0	0	0	0
PTCD2P2	0	0	0	0	0	0	0	0
PTCD3	30.276264	42.770819	18.757007	32.283086	7.434881	13.449795	203.490603	10.353514
PTCH1	26.158063	7.06104	2.387023	6.683485	0.947045	7.417118	0	5.049843
PTCH2	3.402341	0.245616	3.009388	0.018215	0.89422	0.495855	0	0.029938
PTCHD1	0	0	0	0.024319	0	0.024969	0	0.009938
PTCHD3	0	0	0	0	0	0	0	0
PTCHD3P1	0	0	0	0	0	0	0	0
PTCHD3P2	0	0	0	0	0	0	0	0
PTCHD3P3	0	0	0	0	0	0	0	0
PTCRA	0	0	0	0	0	0	0	0
PTDSS1	102.8412	90.038729	116.025297	79.042349	79.979698	54.562694	110.742509	69.022449
PTDSS2	6.998856	29.271117	16.318337	22.084162	45.708474	27.101549	0	21.539733
PTEN	6.401885	2.453663	6.291022	1.133889	11.346181	0.615147	87.477081	12.764854
PTENP1	0	0.705762	6.37838	0.821437	0.853225	0.462204	0	5.42914
PTER	7.928487	1.599064	1.865859	0.721369	3.313897	0.538924	0	0.833487
PTF1A	0	0	0	0	0	0	0	0
PTGDR	0	0	0	0	0	0	0	0
PTGDR2	1.168509	2.092464	2.093302	2.393285	0	0	0	0.376076

PTGDS	0	0	0	0	0	0	0	0
PTGER1	0	0	0	0.185901	0	0	0	0.207776
PTGER2	1.385832	1.352198	1.239782	0.741915	0	0.688286	0	0.948852
PTGER3	0	0	0	0	0	0	0	0
PTGER4	7.764829	25.91624	9.417453	19.945577	3.587461	10.552336	0	5.464387
PTGER4P2	0	2.239971	0	0	0	0	0	0
PTGER4P3	0	0	0	0	0	0	0	0
PTGES	26.142109	19.855225	37.645717	32.006588	38.418256	20.215863	29.166417	22.590939
PTGES2	16.666841	40.737985	47.982177	28.078579	44.517874	54.955351	0	42.522423
PTGES3	505.589144	570.404147	427.516281	373.302102	290.960217	278.537541	223.427741	468.77608
PTGES3L	0	0	4.439347	0.470666	0	0	0	0.404549
PTGES3L-AA	0	0	0	1.274664	0	0	0	0
PTGES3P1	0	0	9.03525	0	0	0	0	0
PTGES3P2	0	0	0	0	0	0	0	0
PTGES3P3	0	0	0	0.245431	0	0	0	0
PTGES3P4	0	0	0	0	0	0	0	0
PTGES3P5	0	0	0	0	0	0	0	0
PTGFR	0	0.227714	0	0.018534	0	0.186616	0	0.762857
PTGFRN	114.655255	195.511278	142.372056	179.77412	97.679257	139.693326	57.404492	110.333834
PTGIR	0	0	0	0	0	0	0	0
PTGIS	0	0	0	0.260753	0	0	0	0
PTGR1	168.716385	208.59053	176.800307	205.223795	102.930122	62.774309	111.858571	56.716324
PTGR2	15.108814	14.589588	11.954553	8.824355	16.484929	2.471896	2.29872	3.893018
PTGR3	36.717386	48.82359	11.274725	33.239847	39.596935	70.439621	28.678746	74.794053
PTGS1	16.701864	5.549125	10.737606	6.95323	53.201155	46.072736	69.797316	39.279281
PTGS2	129.969306	83.058802	210.664167	98.196117	60.523268	18.417312	51.973791	32.759148
PTH	0	0	0	0	0	0	0	0
PTH1R	0	0.462012	0	0	0	0	0	0
PTH2R	0	0	0	0	0	0	0	0
PTHLH	72.672011	149.566593	60.006459	121.904447	113.943879	259.428972	275.601909	265.623815
PTK2	198.913681	344.466012	215.812992	377.702844	76.681629	199.043594	197.912547	199.846668
PTK2B	13.773123	44.849034	47.608024	67.147175	31.718645	23.282778	26.505416	27.781424
PTK6	18.210081	15.150843	26.672336	19.055081	5.999406	2.392448	8.618752	1.409301

PTK7	37.809992	24.30218	33.892148	28.329302	33.836161	37.141752	20.473214	24.880653
PTMA	333.647538	2203.72338	30.330105	3147.32	153.188003	9705.47536	632.174885	2031.28863
PTMAP1	0	0	0	0	0	0	0	0
PTMAP10	0	0	0	0	0	0.866796	0	0
PTMAP11	0	0	0	0	0	0	0	0
PTMAP12	0	0	0	0.912126	0	1.727327	0	0
PTMAP15	0	0	0	0	0	0	0	0
PTMAP2	0	18.66386	0	2.919019	0	5.6682	0	0
PTMAP3	0	0	0	0	0	0.862282	0	0
PTMAP4	0	0	0	0	0	0	0	0
PTMAP5	0	0	0	0	0	0	0	0
PTMAP6	0	0	0	0	0	0	0	0
PTMAP8	0	0	0	0	0	0	0	0
PTMAP9	0	0	0	0	0	0	0	0
PTMS	54.833122	35.390157	12.030942	27.59813	0	59.588078	116.107324	26.180433
PTN	0	0	0	0	0	0	18.280422	0
PTOV1	40.530371	33.625757	42.158945	38.311789	54.670244	49.347288	138.680137	38.584768
PTOV1P1	0	0	0	0	0	0	0	0
PTP4A1	0	31.382119	0	45.803948	6.218052	69.32809	0	36.310152
PTP4A1P1	0	0	0	0	0	0	0	0
PTP4A1P2	0	0	0	0	0	0	0	0
PTP4A1P3	0	0	0	0	0	0	0	0
PTP4A1P4	0	0	0	0	0	0	0	0
PTP4A1P5	0	0	0	0	0	0	0	0
PTP4A1P6	0	0	0	0	0	0	0	0
PTP4A1P7	0	0	0	0.221435	0	0	0	0
PTP4A2	285.291354	300.829886	311.106989	233.141862	320.023417	147.509087	268.694444	370.375529
PTP4A2P1	10.740676	0.824076	0	2.503106	0	0	0	2.699474
PTP4A2P2	0	0	0	0	0	0	0	0
PTP4A3	0	9.104851	10.799167	4.377945	12.346888	0.726795	0	1.689794
PTPA	0	25.169158	0	20.040625	46.583437	46.350994	51.648611	29.551038
PTPDC1	6.037508	8.390717	3.410712	9.05345	5.618779	7.925246	3.514308	8.525122
PTPMT1	24.441161	95.254918	36.500572	56.752243	77.994131	85.679331	85.168853	106.016517

PTPN1	55.84152	72.489051	45.926838	61.620129	62.520446	60.451627	70.685837	65.431697
PTPN11	65.297062	108.520272	45.296682	103.007676	55.379674	99.554965	0	160.287392
PTPN11P1	0	0	0	0	0	0	0	0
PTPN11P2	0	0	0	0	0	0	0	0
PTPN11P3	0	0	0	0.575683	0	0.488304	0	0.080017
PTPN11P4	0	0	0	0	0	0	0	0
PTPN11P5	0	0	0	0	0	0	0	0
PTPN12	71.218217	117.870983	69.017231	87.214477	84.06194	55.662051	178.488996	95.179387
PTPN13	14.696293	27.665002	15.520123	21.479116	30.104308	38.583058	35.089385	59.505993
PTPN14	41.802478	53.311976	35.303958	85.057331	28.034317	108.692093	66.547224	72.761409
PTPN18	8.264609	45.490515	8.857544	39.394205	25.472772	61.546446	9.410943	49.799276
PTPN2	67.02915	61.143807	65.013498	54.849321	73.92936	33.563926	44.942448	47.872883
PTPN20	43.780267	17.252662	4.835103	11.132691	6.740144	8.312618	12.674009	14.012815
PTPN20CP	0	0	0	0.504463	0	0.46362	0	1.092146
PTPN21	4.803647	13.062691	17.917996	13.89842	4.361631	35.893841	8.568503	29.610561
PTPN22	0	0	0	0	0	0.76247	0	0
PTPN23	10.11626	26.413812	13.64634	24.18182	4.555158	29.229533	10.586252	14.866694
PTPN2P1	0	0	0	0	0	0	0	0
PTPN2P2	0	0	0	0	0	0	0	0
PTPN3	19.819265	34.906959	19.560376	33.220686	15.711509	29.016231	15.793386	34.507209
PTPN4	48.325827	53.874789	13.000931	52.582345	18.949907	50.436076	75.121985	71.328103
PTPN5	0	0	0	0	0	0	0	0
PTPN6	25.529498	36.170989	32.002937	37.118784	71.310605	37.699171	35.079339	14.539154
PTPN7	0	0	0	0.030637	0	0	0	0
PTPN9	0	0.489167	0	1.54793	0	1.14138	71.233502	0
PTPRA	48.428078	65.113504	31.125706	72.202108	28.778611	60.178297	33.340026	47.920147
PTPRB	9.474657	10.509029	9.306636	8.463	3.987507	12.398672	0.378559	10.774514
PTPRC	0	0	0	0	0	0	0	0
PTPRCAP	0	0	0	0	0	0	0	0
PTPRD	9.316393	0	0	0.063956	0	0	0	0
PTPRE	21.281383	7.676051	2.37265	7.614664	107.845424	29.03	30.566793	20.345498
PTPRF	86.065664	190.279468	124.756678	237.043374	136.626589	244.908915	90.509242	162.324611
PTPRG	0.687191	2.424397	0.927632	3.206192	3.066554	2.904053	0	2.244472

PTPRH	1.285246	5.156613	6.103807	22.112054	0.263371	0.943218	0	1.100852
PTPRJ	3.350824	4.356959	1.850457	3.619482	8.753178	5.754953	33.093288	4.292851
PTPRK	47.582587	72.997162	72.505027	124.553455	108.18625	177.747877	311.747946	245.703775
PTPRM	31.430589	30.549983	8.179652	43.690281	0.560459	11.578646	0.300651	19.330117
PTPRN	0	0	0	0.144292	0	0.704342	0	0
PTPRN2	0.699126	0.659839	6.905494	0.973592	0	0.139956	0	0.476991
PTPRO	0	0	0	0	0	0	0	0
PTPRQ	0	0	0	0	0	0	0	0.015824
PTPRR	8.158234	9.299653	0	8.018265	1.074793	8.734855	0	13.672203
PTPRS	21.037172	44.750443	37.230682	58.825042	0.372841	49.17179	48.607137	23.853097
PTPRT	0	0	0	0	0	0	0	0
PTPRU	14.683678	12.243451	20.057758	17.277698	29.281624	25.965356	56.000839	17.288325
PTPRVP	0	0	0	0	0	0	0	0
PTPRZ1	1.176799	0.517264	0	0.287339	0	0.765196	0	1.167357
PTRH1	0	8.406371	12.282091	4.512662	0	6.456323	0	3.456232
PTRH2	4.193439	23.795197	9.922567	25.508305	37.797994	11.694869	0	17.660593
PTRHD1	0	20.902126	40.790443	9.629659	0	5.350894	0	19.366089
PTS	22.892268	32.15095	36.207617	20.643212	54.189638	25.16279	26.839425	49.71151
PTTG1	177.912077	165.919191	100.025268	186.961563	158.211012	148.738915	241.207598	179.633509
PTTG1IP	97.360413	112.429438	107.200191	125.643477	84.102938	92.762945	104.467078	77.169304
PTTG1P1	0	0	0	0	0	0	0	0
PTTG1P2	0	0	0	0	0	0	0	0
PTTG2	0	0	0	0	0	0	0	0
PTTG3P	0	0	0	0	0	0	0	0
PTX3	22.243716	24.124946	23.154692	35.604889	5.862937	0.82429	0	2.095418
PTX4	0	0	0	0	0	0	0	0
PUDP	0	8.105339	0	10.235309	0	29.639168	34.30015	0.338644
PUDPP1	0	0	0	0	0	0	0	0
PUDPP2	0	0	0	0	0	0	0	0
PUDPP3	0	0	0	0	0	0	0	0
PUF60	16.706191	22.316679	15.342855	48.610362	40.291222	37.550914	19.861716	24.082146
PUM1	50.861218	63.878267	72.383727	105.73902	61.313509	123.95022	142.746146	79.330532
PUM2	81.862395	115.586533	33.417607	142.929027	51.319158	73.952615	64.988776	90.632952

PUM3	42.478918	44.485917	49.209756	34.495892	55.677861	33.919962	92.232004	49.592932
PURA	8.395402	12.226254	5.778424	15.275192	4.302969	17.571931	13.414289	18.047271
PURG	0	0	0	0	0	0	0	0.050554
PUS1	37.115987	93.415452	41.184152	73.029008	24.345995	85.710626	37.779864	57.304164
PUS10	3.020826	2.625961	8.032979	5.345537	0	3.785595	0	2.222781
PUS3	10.205381	31.919301	12.926805	28.642277	10.266182	35.728575	31.358929	35.928175
PUS7	18.167341	40.681167	18.761768	42.483228	15.831773	47.052931	24.181281	51.084197
PUS7L	13.995994	30.655646	0	14.675063	29.643478	10.497324	20.987159	21.18227
PUSL1	14.681994	26.452531	10.41623	26.735758	36.512438	61.866027	0	42.169609
PVALB	0	0	0	0	0	0	0	0
PVALEF	0	0	0	0	0	0	0	0
PVR	30.432981	60.683593	44.470302	71.521376	53.772542	103.2267	82.02029	70.930136
PVRIG2P	0	0	0	0	0	0	0	0
PWP1	63.604848	106.999813	79.594476	129.198011	115.054235	195.516442	177.850445	227.720755
PWP2	0	9.014972	0	0.653567	0	4.958171	19.109101	2.912235
PWWP2A	20.638856	12.007521	8.192203	10.200376	42.756504	14.733153	20.350973	20.894425
PWWP2AP1	0	0	0	0.036683	0	0	0	0
PWWP2B	3.889039	14.292547	11.60315	14.577652	7.524873	32.267926	7.236448	17.309795
PWWP3A	9.117199	25.220907	4.441355	25.435203	31.89226	37.087498	47.39124	39.041999
PWWP3B	3.341219	8.09108	2.248835	3.890542	0	0.118503	0	0.100641
PXDC1	0	36.604626	24.28331	49.889983	38.088275	42.143083	23.023944	63.394565
PXDN	25.658751	31.034416	55.53519	68.550542	48.454871	61.337082	108.812519	42.889541
PXDNL	0	0.146267	0	0.301909	0	0	0	0
PXK	5.281392	6.220871	12.074493	1.552113	4.979163	2.841525	0	1.233997
PXMP2	20.040222	28.263019	21.198882	20.657948	2.455144	30.19987	61.500048	19.474486
PXMP4	8.013794	8.096898	5.659052	11.049188	14.670844	6.773162	0	6.944602
PXN	287.222147	525.011229	230.641989	461.71355	389.50651	973.482898	358.076431	1219.05329
PXT1	0	0	0	0.040529	0	0	0	0
PXYLP1	25.462033	21.672441	13.071951	38.539308	16.528146	25.174928	0.958906	14.530836
PYCARD	27.482179	24.211224	28.827769	20.902986	14.11208	31.265374	0	23.506063
PYCR1	10.221632	77.881024	7.919839	70.162164	45.519483	16.876881	29.514627	17.188983
PYCR2	54.970926	82.374825	58.883919	91.292692	16.17516	69.275562	17.62574	50.241382
PYCR3	13.899658	10.567519	10.686213	9.205039	1.454546	8.835574	11.383396	5.961077

PYDC1	0	0	0	0	0	0	0	0
PYDC2	0	0	0	0	0	0	0	0
PYGB	52.099485	66.420307	83.010367	88.049198	105.357016	73.605395	43.892408	62.862329
PYGL	551.134972	685.518418	494.78973	730.932984	540.835234	510.380716	285.954289	488.480357
PYGM	0	0.055268	0	0.031614	0	0.064553	0	0.15682
PYGO1	7.828528	10.547909	9.925465	6.087111	0	0.707175	0.092596	1.23212
PYGO2	27.349613	68.85421	22.627999	63.274806	23.32432	61.724438	74.395034	49.017826
PYHIN1	0	0	0	0	0	0	0	0
PYHIN5P	0	0	0	0	0	0	0	0
PYM1	42.150617	147.493533	39.035423	151.958166	18.704354	80.014881	109.980221	73.734216
PYROXD1	7.570396	8.551969	12.824903	3.136953	26.385687	1.813793	6.50507	4.093
PYROXD2	1.713567	2.756551	1.530135	4.901635	0.451774	4.376093	7.468479	4.098959
PYURF	69.893803	73.409148	39.700631	37.588349	38.252017	34.587337	26.914609	89.487174
PYY	0	0	0	0	0	0	0	0
PYY2	0	0	0	0	0	0	0	0
PYY3	0	0	0	0	0	0	0	0
PZP	0	0	0	0	0	0	0	0
QARS1	54.832944	44.88027	82.755273	69.2324	126.293537	67.539276	51.657299	39.965328
QDPR	2.880054	19.254435	16.949878	16.803416	27.27645	22.560561	0	24.628334
QKI	106.107393	145.097944	111.443842	190.787444	118.144376	156.442041	58.854165	227.126695
QNG1	26.577553	25.472243	37.879491	22.693225	4.367923	15.775483	47.421659	18.223995
QPCT	20.519053	3.541668	16.856892	5.753777	20.201712	2.589538	0	2.811306
QPCTL	3.866455	5.780064	7.252409	5.432687	6.849753	5.345122	0	4.582804
QPRT	2.992149	4.177097	6.985043	3.229977	1.133721	1.427508	0	0.441097
QRFP	0	0	0	0	0	0	0	0
QRFPR	0	0	0	0	0	0	0	0
QRICH1	75.530149	56.293905	43.14056	63.761912	30.554284	67.773186	46.295601	60.299969
QRICH2	0	0.675928	0	0.536139	0	0.671488	0	0.293284
QRSL1	7.232166	15.721432	8.655196	8.436806	2.758803	7.949478	10.36534	15.921036
QRSL1P1	0	0	0	0	0	0	0	0
QRSL1P2	0	0	0	0	0	0	0	0
QRSL1P3	0	0	0	0	0	0	0	0
QSER1	11.699472	11.356439	4.35568	13.592146	22.53849	18.75028	64.565921	26.535274

QSOX1	35.634958	20.313955	52.479307	45.423162	88.421595	49.309458	91.648177	33.481001
QSOX2	0	5.88554	0	5.478037	9.588084	4.295366	0	0
QTRT1	16.510901	7.566234	30.513329	9.102252	7.450516	7.078643	0.662453	4.687852
QTRT1P1	0	0	0	0	0	0	0	0
QTRT2	51.600405	56.458025	108.228557	91.687738	25.311054	74.718787	128.586161	80.155814
R3HCC1	0	0.524711	0	0	1.0719	0.701469	0	2.171658
R3HCC1L	16.73003	12.851094	17.02701	7.259877	0	7.710479	6.099262	9.417005
R3HDM1	23.89935	39.38138	15.226406	31.010083	48.982504	37.951705	88.062045	65.620166
R3HDM2	49.950954	45.001505	10.418533	29.680463	11.31653	20.75508	62.223496	20.135225
R3HDM2P1	0	0	0	0	0	0	0	0
R3HDM2P2	0	0	0	0	0	0	0	0
R3HDM4	50.605341	36.102469	58.857683	43.595041	32.827396	52.116745	46.088269	45.669907
RAB10	168.880004	446.032059	134.798827	440.179636	102.888123	293.269876	193.788626	490.761064
RAB11A	191.603954	262.858542	234.188874	226.541062	222.766537	119.437832	100.473561	208.467942
RAB11AP1	0	0	0	0	0	0	0	0
RAB11AP2	0	0	0	0	0	0	0	0
RAB11B	33.617034	46.67333	39.905523	46.500788	18.298976	55.787062	66.022063	38.94404
RAB11FIP1	5.372466	13.358179	9.472204	15.335815	21.238729	21.894721	31.081199	11.50792
RAB11FIP1P1	0	0	0	0	0	0	0	0
RAB11FIP2	8.555754	25.563249	47.835703	8.284662	18.615069	20.165364	42.150355	30.520648
RAB11FIP3	0	5.038985	5.518122	7.400485	0	12.799049	36.567297	11.057586
RAB11FIP4	10.591447	14.252051	12.284807	23.663645	34.207792	26.321352	109.396927	18.057813
RAB11FIP5	16.348424	17.879395	25.189596	19.724219	13.464186	26.784708	8.82467	21.001581
RAB12	0	0	0	0	0	0	0	0
RAB13	0	0.353352	0	0.834999	0	2.322738	0	0.31887
RAB14	78.349494	77.440081	60.883494	54.317193	85.67204	43.956707	39.055557	98.083022
RAB15	3.930832	10.591459	7.930394	6.109289	0	16.60828	12.30557	14.370728
RAB17	17.109582	15.810953	10.422913	22.43618	88.260346	37.035509	27.880483	31.915732
RAB18	21.233264	35.083346	12.376306	20.987596	15.493012	11.179814	10.346619	25.868397
RAB19	0	5.21965	0	0	0	0	0	5.26563
RAB1A	188.480878	279.96654	156.215165	218.650746	125.866582	105.783572	119.960191	236.874206
RAB1AP1	0	0	0	0	0	0	0	0
RAB1AP2	0	0	0	0	0	0	0	0

RAB1B	242.981127	314.448834	186.148677	333.273699	117.069485	259.0703	97.980928	215.021578
RAB1C	0	0	0	0	0	0.351191	0	0
RAB20	4.763386	12.39438	6.356204	10.833638	1.881444	20.240499	0	22.754637
RAB21	114.538645	164.438491	85.053934	112.358179	181.437473	115.64411	119.011905	226.693179
RAB22A	22.334651	36.199079	31.484136	24.296867	18.387073	18.020306	16.165439	30.034369
RAB23	13.525107	14.111765	9.7788	8.696046	18.304269	9.051682	13.878072	21.923302
RAB24	8.977309	25.668516	21.10971	29.807034	7.877501	28.513921	0.720017	22.417483
RAB25	85.986698	120.94039	68.172419	150.416351	59.961396	135.310527	51.184264	101.988828
RAB26	4.145088	1.699117	5.937541	2.908486	0	0	0	1.647671
RAB27A	30.11282	57.431866	33.782645	40.79991	1.784952	43.975606	22.738158	53.534628
RAB27B	12.941655	26.46474	7.482	26.337521	37.637716	21.709506	13.744777	19.708513
RAB28	12.55417	18.993167	5.171629	13.724068	22.128656	5.93787	0	29.633833
RAB28P1	0	0	0	0	0	0	0	0
RAB28P2	0	0	0	0	0	0	0	0
RAB28P3	0	0	0	0	0	0	0	0
RAB28P4	0	0	0	0	0	0	0	0
RAB28P5	0	0	0	0	0	0	0	0
RAB29	27.237973	65.431212	32.75834	66.645987	16.245286	44.496946	22.655095	47.550431
RAB2A	208.220626	170.977912	171.982867	166.278668	105.471651	55.123844	54.189999	118.181736
RAB2B	27.165156	24.319808	20.681678	35.14275	61.796275	25.592054	12.337505	37.226648
RAB30	4.23499	17.493296	2.659629	23.313972	31.556809	33.119193	10.114863	36.672928
RAB31	32.064481	41.101108	31.045234	54.028686	50.816807	60.832137	20.531653	51.723009
RAB32	10.728141	50.695122	22.1197	30.732766	24.428913	84.270924	0.856933	117.637146
RAB33A	0	0.849364	0	2.677221	16.297046	1.79556	0	1.014708
RAB33B	5.335637	5.137353	2.393395	2.717545	4.931022	0.966863	0	3.73202
RAB34	102.297467	167.433393	98.915633	156.0034	82.731276	151.6768	145.906233	145.656599
RAB35	62.331234	142.668041	56.27324	124.843096	79.775254	255.929864	111.807358	199.646376
RAB36	0	0.811887	0	0.698817	0	3.62153	0	0
RAB37	0	1.311396	0	0.704101	0	0	0	0.194773
RAB38	68.168987	112.3147	87.524268	104.399762	53.836598	40.12894	10.992158	64.266385
RAB39A	0	0.469697	0	0.629435	0	0.36614	0	0
RAB39B	0.976014	3.462651	0	2.690198	0	0.097392	0	0.117679
RAB3A	0	1.646935	0	0.304625	1.550591	1.648533	1.417518	0

RAB3B	19.767817	15.809863	15.546006	11.394346	7.342625	4.430319	2.565785	5.360448
RAB3C	0	0	0	0	0	0	0	0
RAB3D	11.655429	18.222325	11.160722	22.945486	14.365076	23.393329	2.269682	24.191914
RAB3GAP1	7.154793	10.940325	0	9.663294	26.055953	6.522787	90.871638	15.801864
RAB3GAP2	19.609378	14.044445	27.31609	18.593995	9.657494	17.012859	37.298277	25.477181
RAB3IL1	9.346988	7.219737	1.450482	6.014425	8.929391	33.917595	0	28.882855
RAB3IP	24.792734	80.712642	36.283381	81.075204	65.109385	73.060951	83.563177	146.493481
RAB40A	0	0.448106	0	0.667144	0	0	0	0.071241
RAB40AL	0	0	0	0.128776	0	0	0	0
RAB40B	1.571724	15.242467	8.9448	5.37545	1.826522	12.403712	0	13.333765
RAB40C	14.534796	19.903337	18.920366	27.689578	2.949294	47.715797	0	39.341188
RAB41	0	0	0	0	0	0	0	0
RAB42	0	4.226269	1.592733	3.870198	3.028912	5.242657	0	3.866694
RAB42P1	0	0	0	0	0	0	0	0
RAB43	0	4.921012	0	4.799913	0	7.763159	0	11.750302
RAB43P1	0	0	10.266893	7.377003	0	19.227174	0	26.507461
RAB44	0	0	0	0.018887	0	0	0	0
RAB4A	0	16.64351	25.504899	22.521325	0	31.630963	6.730798	52.438875
RAB4B	6.462844	14.532621	6.738687	17.802192	24.408542	18.7837	14.626604	16.544896
RAB4B-EGLN	0	1.972842	6.541385	0	0	1.072685	0	1.002676
RAB5A	52.607504	127.74722	48.120892	128.806472	63.831991	75.400555	38.637925	137.300812
RAB5B	56.995112	87.419085	60.923424	90.770023	39.779004	57.945716	104.518669	55.992441
RAB5C	11.219741	10.996278	0	3.171515	35.47407	64.883925	0	26.576572
RAB5CP1	0	0	0	0	0	0	0	0
RAB5CP2	0	0.550861	0	0	0	0	0	0.864111
RAB5IF	223.532743	532.842287	167.652009	366.114022	157.139922	287.625517	229.494101	407.053564
RAB6A	59.663505	210.890223	27.405965	182.923854	29.185822	124.224734	0	149.794031
RAB6B	0	5.343443	3.711344	7.981198	1.186984	6.746764	0	10.548634
RAB6C-AS1	0	0	0	0	0	0	0	0
RAB7A	0	216.034799	82.57558	328.906823	42.454614	274.108717	0	35.083409
RAB7B	1.890329	4.329258	3.37258	1.967497	0	3.582758	0	1.670623
RAB8A	137.857195	103.634377	52.401468	102.164483	238.023048	138.800944	66.887923	97.82947
RAB8B	47.309131	23.880617	9.198855	16.121326	52.87615	11.548125	22.828005	26.689611

RAB9A	0	20.927274	28.457874	18.890864	36.945658	35.155132	0	38.441796
RAB9AP2	0	0	0	0	0	0	0	0
RAB9AP3	0	0	0	0	0	0	0	0
RAB9AP4	0	0	0	0	0	0	0	0
RAB9AP5	0	0	0	0	0	0	0	0
RAB9B	0.878369	0.639085	0	1.136183	0	0.350974	0	0.352844
RAB9BP1	0	0	0	0	0	0	0	0
RABAC1	44.514785	36.688855	34.971148	40.826952	56.945922	50.913566	4.999798	46.684267
RABEP1	29.490432	42.267333	53.674923	39.834187	59.66178	43.473233	17.045256	52.937099
RABEP2	4.748321	14.078027	4.243168	12.732365	21.218428	20.311791	1.141144	16.018023
RABEPK	30.144474	13.031887	17.973101	21.508076	19.41323	8.677862	14.777229	7.859392
RABEPKP1	0	0	0	0	0	0	0	0
RABGAP1	32.816292	52.222238	34.865202	68.919868	81.353959	49.377375	9.911829	53.121105
RABGAP1L	67.488856	61.058554	56.523534	67.706971	56.053976	20.819784	67.982806	34.743039
RABGEF1	27.244496	55.9066	29.634637	50.37134	23.76178	44.313303	6.193235	49.872357
RABGEF1P1	0	0	0	0	0	0	0	0
RABGEF1P2	0	0.162594	3.485484	0.479694	0	0	0	0.322662
RABGEF1P3	0	0	0	0.605872	0	4.142885	0	1.027904
RABGGTA	14.303415	12.865447	0	8.625029	1.832923	39.141285	50.142489	30.152833
RABGGTB	127.93239	64.145833	65.053457	48.40867	25.229708	10.779286	122.074966	32.428767
RABGGTBP1	0	0	0	0	0	0	0	0
RABL2A	5.422713	16.470371	0	12.496947	5.918124	13.334538	0	7.167517
RABL2B	4.648414	15.136418	9.65397	15.836291	6.813692	13.937382	33.046793	10.61155
RABL3	19.683649	9.551306	16.131968	15.0638	21.485439	5.492698	0	10.962795
RABL6	71.27862	86.187144	37.082443	82.895234	40.262424	201.556641	50.057022	161.654947
RAC1	679.564929	1348.29983	862.38698	1546.11286	410.708635	834.566774	584.494888	917.629475
RAC1P1	0	0	0	0	0	0	0	0
RAC1P2	0	0	0	0	0	2.468522	0	0.979744
RAC1P3	0	0	0	0	0	0	0	0
RAC1P4	0	0	0	0	0	0	0	0.331851
RAC1P5	0	0	0	0	0	0	0	0
RAC1P6	0	0	0	0	0	0	0	0
RAC1P7	0	0	0	0	0	0	0	0

RAC1P8	0	0	0	0	0	0	0	0
RAC1P9	0	0	0	0	0	0	0	0
RAC2	26.910319	44.11123	36.983464	63.553832	42.511111	41.5512	46.498807	31.892158
RAC3	59.05017	88.408638	41.16865	77.110553	14.035803	54.669905	42.332364	25.707024
RACGAP1	56.084299	123.344024	30.230706	125.68284	82.395713	149.069848	46.538982	140.964414
RACGAP1P1	0	0	0	0	0	0	0	0
RACK1	2477.09566	1659.46604	2995.24748	1941.56448	2009.35992	1905.30597	1572.34119	1968.42518
RACK1P1	0	0	0	0	0	0	0	0
RACK1P2	0	0	0	0	0	0	0	0
RACK1P3	0	0	0	0	0	0	0	0
RAD1	42.733048	73.600463	33.414735	40.253295	28.407624	25.877834	69.468921	59.591446
RAD17	24.496386	36.745814	24.569567	23.622857	14.092124	23.19196	92.213662	28.532597
RAD17P1	0	0	0	0	0	0	0	0
RAD17P2	0	0	0	0	0	0	0	0
RAD18	20.220825	18.759764	24.224646	9.826424	10.226494	7.703984	5.145265	15.323477
RAD1P1	0	0	0	0	0	0	0	0
RAD1P2	0	0	0	0	0	0	0	0
RAD21	0	0	41.390557	10.910336	0	17.063453	0	0
RAD21L1	0	0	0	0.330356	0	0	0	0
RAD21P1	0	0	0	0	0	0	0	0
RAD23A	63.49905	211.717889	88.262374	232.199835	162.023658	483.988231	100.769354	343.987122
RAD23B	137.131466	289.504276	133.16391	222.32307	109.24421	182.076863	130.937414	263.890558
RAD23BP1	0	0	0	0	0	0	0	0
RAD23BP2	0	0	0	0	0	0	0	0
RAD23BP3	0	0	0	0	0	0	0	0
RAD50	47.206409	36.268177	45.55067	26.771225	28.074498	11.087076	27.205386	19.426344
RAD51	34.807059	86.579922	16.55495	80.731065	18.001866	79.641606	42.324443	86.291137
RAD51AP1	9.443531	39.499846	21.457576	20.69032	44.575851	15.685857	19.718227	21.148628
RAD51AP1P1	0	0	0	0	0	0	0	0
RAD51B	51.694617	21.167264	6.278093	35.782794	27.52153	13.669926	21.938502	3.731801
RAD51C	13.000749	46.912085	45.910791	24.438334	39.736693	27.414666	116.83826	64.501839
RAD51D	8.248846	20.265907	9.481847	24.959338	6.297723	17.82931	26.790608	17.361292
RAD52	0	10.48844	7.587635	22.509691	2.105076	28.596565	0	10.553083

RAD52P1	0	0	0	0	0	0	0	0
RAD54B	11.995334	9.106692	25.112051	6.447512	14.002107	2.607632	40.130781	7.270942
RAD54L	9.800801	27.612907	6.830976	19.962488	26.242278	29.473582	0	41.591823
RAD54L2	6.571745	14.101512	8.576159	10.053388	11.89885	29.919233	17.492045	27.632697
RAD54L2P1	0	0	0	0	0	0	0	0
RAD9A	25.364377	40.88621	24.585363	38.088046	13.08278	28.899652	8.983514	22.667565
RAD9B	0	0.158364	0	0.550138	0.697458	1.057062	0	1.055239
RADIL	2.349734	5.030937	0	0.804672	0	0	0	0
RADX	3.682816	3.237633	10.830641	1.561037	1.964965	0.073917	0	0.545843
RAE1	45.817189	64.373623	68.308877	61.886594	115.120613	64.182257	122.197874	63.125826
RAET1E	1.645576	4.01425	2.939975	4.8517	7.810255	2.380235	0	1.02189
RAET1G	3.52407	2.080986	2.887106	4.722353	1.14777	4.479318	0	3.04228
RAET1K	0	0	0	0.231443	0	0	0	0
RAET1L	0	0	0	1.937654	0	1.60347	0	0
RAET1M	0	0	0	0	0	0	0	0
RAF1	0	0	0	0	0	0	0	0.879936
RAF1P1	0	0	0	0	0	0	0	0
RAG1	0.894573	0	0.813083	0.938157	0	1.017882	0	0.624599
RAG2	0	0	0	0	0	0	0	0
RAI1	15.563267	46.68094	7.080637	53.055094	23.534412	168.507462	7.999552	96.789826
RAI14	217.541143	274.540565	177.868983	345.468068	41.838746	50.816857	16.250662	68.613287
RAI2	0	0.57604	0	0.349585	4.978791	0.757523	5.353547	0.617439
RALA	31.478207	103.773967	34.703113	47.188444	164.554281	106.926332	164.739402	217.410868
RALB	49.368171	128.219327	45.437031	115.909643	80.13253	151.03311	107.666926	161.471002
RALBP1	27.11734	70.735733	48.338378	65.817133	53.4189	85.76151	63.809391	117.047892
RALBP1P1	0	0	0	0	0	0	0	0
RALBP1P2	0	0	0	0	0	0	0	0
RALGAPA1	24.627298	30.346003	19.076014	26.461082	12.824091	11.859686	125.065184	27.852817
RALGAPA1P1	0	0	0	0.145845	2.722338	0.036274	0	0.029167
RALGAPA2	0.367128	3.962551	4.848208	5.644387	27.874514	9.385184	49.96706	10.038398
RALGAPB	45.995887	59.324166	36.420532	45.540637	40.153176	31.431354	23.987364	56.048116
RALGDS	19.138482	32.694056	20.15429	54.484308	23.248007	47.046289	16.507453	26.664187
RALGPS1	6.048945	0.94832	5.020081	4.787218	2.814417	4.434285	0	5.579

RALGPS2	0	18.143108	0	23.986404	0	5.1687	65.705731	13.368972
RALY	191.477769	377.301534	221.851969	384.860955	208.955757	397.217419	131.841782	361.700387
RALYL	0	0	0	0	0	0	0	0
RAMAC	45.633892	45.932034	24.371055	43.151708	33.651929	36.355351	28.086914	34.987761
RAMP1	0	8.504678	0	3.695223	1.291793	7.537865	0	5.950961
RAMP2	8.08291	1.788513	0	4.941587	0	0.397426	0	0.337133
RAMP3	0	0	0	0	0	0	0	0
RAN	709.028649	1090.66224	730.385179	888.723502	942.889695	730.016392	853.449732	999.398175
RANBP1	114.211883	146.227349	29.216116	124.035467	111.185557	206.834276	52.105507	229.815314
RANBP10	26.814414	9.442027	14.54779	11.074272	27.507758	15.452634	14.121831	9.643604
RANBP17	26.782572	7.410489	20.473806	1.78029	42.192235	0.653182	6.97673	2.719433
RANBP1P1	0	0.322258	0	0	0	0	0	0
RANBP2	21.650071	19.194827	21.24302	12.694072	53.214346	13.040393	26.315783	33.118301
RANBP20P	0	0	0	0	0	0	0	0
RANBP3	50.790985	62.743004	35.307402	80.465846	142.515472	97.228696	120.90245	61.335519
RANBP3L	0	0	0	0	0	0	0	0
RANBP6	0	2.870561	0	1.134211	0	1.232874	0	0
RANBP9	39.3943	26.738901	23.842927	21.115166	20.800279	27.40874	48.415703	38.000361
RANGAP1	26.180872	35.169716	8.258404	48.874004	25.088048	76.600698	48.00563	47.983817
RANGRF	5.574189	17.680814	9.740962	23.594019	33.175535	32.606656	0	23.96312
RANP1	0	0.853857	0	0.836793	0	0.33223	36.530526	0.57894
RANP2	0	0	0	0	0	0	0	0
RANP3	0	0	0	0	0	0	0	0
RANP4	0	0	0	0	0	0	0	0
RANP5	0	0	0	0	0	0	0	0
RANP6	0	0	0	0	0	0	0	0
RANP7	0	0	0	0	0	0	0	0
RANP8	0	0	0	0	0	0	0	0
RANP9	0	0	0	0	0	0	0	0
RAP1A	100.003068	128.776971	109.157957	100.938891	64.153065	33.09203	42.103266	50.642502
RAP1AP	0	0	0	0	0	0	0	0
RAP1B	230.712347	311.359352	209.636949	160.481056	260.501484	171.893302	538.643123	571.431452
RAP1BL	0	0	0	0	0	0	0	0

RAP1BP1	0	0	0	0	0	0	0	0
RAP1BP2	0	0	0	0	0	0	0	0
RAP1BP3	0	0	0	0	0	0	0	0
RAP1GAP	4.127734	1.653549	6.116199	2.051849	0	10.344702	0.331692	10.815572
RAP1GAP2	7.840159	35.843166	17.183599	39.228833	13.85484	14.732258	43.227069	6.28164
RAP1GDS1	47.521944	44.539121	33.168334	33.117481	69.720101	17.596409	19.731177	36.214126
RAP2A	11.180429	15.839074	8.989904	15.948718	7.617729	13.905617	2.007437	22.566059
RAP2C	58.872743	41.868873	42.432071	38.962768	7.847042	26.699401	62.761877	51.479328
RAP2CP1	0	0	0	0	0	0	0	0
RAPGEF1	23.355189	32.253586	16.052879	32.112974	2.029126	57.800897	31.050326	36.71606
RAPGEF2	33.990063	27.371169	7.717915	19.007357	8.778341	15.958531	6.597609	18.616539
RAPGEF3	5.222165	4.291619	8.347487	5.784352	13.547059	4.633914	0	2.212733
RAPGEF4	11.269157	3.750705	0	4.950491	14.946225	5.175271	0	5.949826
RAPGEF5	7.34444	12.57884	11.44686	20.138631	7.485881	13.819881	0	15.441528
RAPGEF6	10.403054	14.015469	8.96487	13.542804	10.837568	8.805538	8.345773	8.159801
RAPGEFL1	4.856009	6.268532	12.193148	11.96955	0	18.223727	43.423707	7.412228
RAPH1	18.881036	37.959129	14.309673	38.247908	81.837401	45.473319	69.094429	53.561082
RAPSN	0	0	0	0	0	0	0	0
RARA	39.089308	40.498846	34.015299	69.548111	22.439901	76.635619	30.970746	66.263507
RARB	0	0	0	0.573364	0	0.852506	0	0.171942
RARG	60.513623	50.73518	39.578219	58.07482	54.258994	55.974174	54.552727	42.102266
RARRES1	0	0	0	0.803683	0	0	0	0
RARRES2	0	0	0	0	4.3196	0.383071	0	2.076942
RARRES2P1	0	0	0	0	0	0	0	0
RARRES2P10	0	0	0	0	0	0	0	0
RARRES2P11	0	0	0	0	0	0	0	0
RARRES2P2	0	0	0	0	0	0	0	0
RARRES2P3	0	0	0	0	0	0	0	0
RARRES2P5	0	0	0	0	0	0	0	0
RARRES2P6	0	0	0	0	0	0	0	0
RARRES2P7	0	0	0	0	0	0	0	0
RARRES2P8	0	0	0	0	0	0	0	0
RARRES2P9	0	0	0	0	0	0	0	0

RARS1	95.936955	91.812087	126.385424	55.463572	81.933576	37.488954	144.917325	91.15808
RARS1P1	0	0	0	0.06172	0	0	0	0
RARS2	30.625105	23.26188	19.162513	19.301056	10.096116	14.578313	41.197766	28.698879
RASA1	29.244764	21.927705	57.010579	7.757326	24.589359	2.022719	29.801436	36.817923
RASA2	0	7.051359	0	6.256699	29.327014	4.797062	0	7.013769
RASA3	7.841814	11.89989	13.374815	15.067894	6.627255	7.52805	4.580966	7.052774
RASA4	0	0.721016	0	1.612106	0	4.942283	14.425975	5.509578
RASA4B	0	0.403454	0	0	6.211913	0.587337	0	0
RASA4CP	0	0.136983	0	0	0	0	0	0.40864
RASA4DP	0	0	0	0	0	0	0	0
RASA4EP	0	0	2.892983	0.638237	0	1.92886	0	0.930437
RASAL1	0.86749	8.683212	6.819469	4.843244	23.345463	5.78366	0	3.598197
RASAL2	25.068957	7.238296	7.194138	11.938584	6.331125	9.68369	27.650593	9.223122
RASAL3	0	0	0	0.149482	0	0.30401	0	0
RASD1	6.044363	0.764631	0	2.931076	0	3.875557	12.44659	1.953838
RASD2	1.93169	2.725111	0.866117	3.157096	0	0.289164	4.931865	0.155263
RASEF	15.316046	24.47717	17.917129	24.061384	13.1826	32.258295	3.554943	32.376164
RASGEF1A	0	0	0	0	0	0.514321	0	1.020158
RASGEF1B	0	0	0	0.445132	0	0.197758	0	0.497532
RASGEF1C	0	0	0	0.257394	0	0	0	0
RASGRF1	3.771634	4.328565	0.706738	2.147911	10.286001	3.578714	0	3.264714
RASGRF2	3.798251	19.237716	0.683488	6.749723	3.886038	2.161311	1.889122	4.948611
RASGRP1	0	0.231528	4.536385	0.304885	0	0	0	0
RASGRP2	0	3.358518	0	3.431678	0.59434	3.163625	0	1.727817
RASGRP3	0	0.301914	0	1.857278	0	0.607562	0	2.775367
RASGRP4	0	0	0	0	0	0	0	0
RASIP1	2.09056	0	0	0.203891	0	0.219459	0	1.09359
RASL10A	0	0.423385	0	0.596773	0	1.263705	0	0.694549
RASL10B	0	0	0	0	0	0	0	0
RASL11A	0	0	0	0	0	0	0	0
RASL11B	6.599403	0.600319	0	0.690461	0	0.262081	0	0.164413
RASL12	0	0.114813	0	0.854086	0	0	0	0
RASSF1	4.278449	8.702028	10.715367	12.884394	4.936733	10.672276	0	5.237777

RASSF2	0	0.6761	0.56442	0.636343	0.163322	0.57627	0	0.582839
RASSF3	11.927396	18.996433	13.609036	13.683617	58.342638	29.194487	27.796489	47.550763
RASSF4	1.828589	5.81599	1.640259	6.741873	0.241406	0.730366	0	1.834143
RASSF5	2.03653	4.1194	9.585714	4.67069	7.099175	7.056326	11.989913	5.271072
RASSF6	15.705751	5.070293	11.202593	11.018135	3.653574	7.282488	0	5.727399
RASSF7	14.904288	23.57929	15.489328	23.326036	15.54357	43.561419	15.000968	27.104874
RASSF8	24.584579	35.087831	19.09197	36.65646	20.963822	37.04039	41.652692	36.966623
RASSF9	0.591203	0.304787	0	0.274605	0.312384	1.096073	0	0.592846
RAVER1	7.816501	23.190214	29.568133	29.757311	20.238881	36.270078	17.474192	27.522594
RAVER2	21.832937	10.664995	14.222581	8.644736	9.961596	2.904797	11.93668	8.023302
RAX	0	0.244287	0	0.418735	0	0	0	0
RAX2	0	0	0	0	0	0	0	0
RB1	0	0	19.739307	7.6041	0	4.03373	28.258276	0
RB1CC1	71.030667	43.241452	29.97044	41.786207	40.918719	36.814039	25.4548	39.883266
RBAK	52.012024	22.384898	23.654922	30.609828	14.510975	14.821739	67.534845	21.572752
RBAK-RBAKD	0	7.637784	4.578935	10.968961	0	3.261775	0	1.612673
RBBP4	280.143594	190.778724	140.538434	205.371371	170.294526	196.373253	247.94575	215.200108
RBBP4P1	0	0.630425	0	0.16833	0	0.567968	0	0.134486
RBBP4P2	0	0	0	0	0	0	0	0
RBBP4P3	0	0	0	0	0	0	0	0
RBBP4P4	0	0.119043	0	0	0	0	0	0
RBBP4P5	0	0	0	0.254367	0	0.284441	0	0.57474
RBBP4P6	0	0	0	0	0	0	0	0
RBBP4P7	0	0	0	0	0	0	0	0
RBBP5	25.919431	28.562197	22.323172	23.799798	23.706947	20.133007	16.068857	31.224282
RBBP6	66.76665	69.714858	23.340148	61.725615	76.663522	99.528261	89.666979	116.454949
RBBP7	74.539257	187.664632	58.685612	105.040434	80.872322	387.839466	264.258686	240.151476
RBBP8	84.559763	133.176198	46.615397	81.571854	240.513396	83.097373	178.486253	164.580945
RBBP8NL	2.410291	2.002958	0	1.702715	1.27212	3.41856	0	1.115327
RBBP9	21.533164	11.949197	28.094581	8.609355	20.015001	6.772855	41.097979	11.707977
RBCK1	98.472126	100.008645	77.535126	121.636627	59.349908	107.244055	28.353009	88.925711
RBFA	30.441665	35.662508	29.677944	36.893539	30.924269	48.623868	8.819218	30.055999
RBFOX1	0	0	0	0	0	0	0	0

RBFOX2	73.625265	109.321204	90.553708	102.454771	120.12722	96.198125	37.811338	87.041303
RBFOX3	0	0	0	0.079079	0	0.159803	0	0
RBIS	227.729602	116.678918	119.102812	46.419408	94.982774	18.26299	202.302983	47.18601
RBISP1	0	0	0	0	0	0	0	0
RBISP2	0	0	0	0	0	0	0	0
RBISP3	0	0	0	0	0	0	0	0
RBISP4	0	0	0	0	0	0	0	0
RBISP5	0	0	0	0	0	0	0	0
RBISP6	0	0	0	0	0	0	0	0
RBKS	2.964012	5.820059	10.246462	6.626361	3.89823	3.106477	0	0.258385
RBL1	19.364256	26.341148	32.20708	26.739641	0	16.348228	43.255319	24.57451
RBL2	45.216668	56.882316	77.575036	43.82431	34.528922	14.493589	24.338461	27.376425
RBM10	18.636591	62.173721	24.415139	64.640988	29.038918	100.122477	11.797963	84.402629
RBM11	0	1.325217	5.655092	6.461057	0	0.305757	0	1.327236
RBM11P1	0	0	0	0	0	0	0	0
RBM12	92.820533	160.593038	81.966003	188.951037	132.376617	186.686446	185.888099	190.025306
RBM12B	0	0	0	0	0	0	0	0.408442
RBM14	21.830262	11.416909	0	13.32299	0	20.660408	37.910871	18.817486
RBM14-RBM	0	3.769372	3.168685	4.39617	0	2.307989	0	13.025112
RBM15	32.066923	53.205942	28.322807	48.562991	39.11721	52.821444	14.11637	48.334072
RBM15B	23.980662	49.58225	21.562907	40.2156	18.751558	75.9905	17.062912	58.497056
RBM17	48.524951	221.680694	10.968329	183.778051	27.912861	192.029403	57.773828	197.224242
RBM17P1	0	0	0	0	0	0	0	0
RBM17P2	0	0	0	0	0	0	0	0
RBM17P3	0	0	0	0	0	0	0	0
RBM17P4	0	0	0	0	0	0	0	0
RBM18	21.760983	34.756248	34.863301	26.122027	22.16885	21.397575	41.201252	28.829519
RBM19	17.43614	27.354223	16.108311	27.835926	35.341786	59.338583	105.468745	40.483241
RBM20	0.443348	0.057253	0	0.162715	0	0.044504	0	0.337133
RBM22	47.679849	25.189609	37.301064	35.654233	23.579576	41.371369	31.559012	29.501483
RBM22P1	0	0	0	0	0	0	0	0
RBM22P12	0	0	0	0	0	0	0	0
RBM22P13	0	0	0	0	0	0	0	0

RBM22P2	0	0	0	0	0	0	0	0
RBM22P3	0	0	0	0	0	0	0	0
RBM22P4	0	0	0	0	0	0	0	0
RBM22P5	0	0	0	0	0	0	0	0
RBM22P6	0	0	0	0	0	0	0	0
RBM22P7	0	0	0	0	0	0	0	0
RBM23	31.28496	60.289663	33.707253	74.909172	35.441112	114.768114	59.783584	106.462409
RBM24	3.706406	2.778396	0	2.161416	0	1.454164	0	0.964008
RBM25	51.699039	34.996047	30.97366	31.514892	70.598914	44.542947	94.786373	40.41993
RBM26	18.729016	25.781273	1.854398	22.185796	3.649756	24.157386	20.153829	27.151742
RBM27	35.843438	20.859054	24.318035	14.600584	24.107318	9.546907	35.98847	18.575829
RBM28	39.58804	47.084585	61.046055	45.516052	63.294432	58.233058	39.581823	54.485343
RBM3	313.723244	190.078731	186.905992	228.729422	214.867359	142.017277	468.882765	162.734924
RBM33	37.235191	40.053103	14.481345	36.640788	34.689957	42.39476	25.854939	43.214246
RBM34	90.996849	141.771087	131.278	104.421214	170.586356	143.023167	145.084015	126.341128
RBM38	8.559617	42.984241	14.035568	29.75915	7.032287	63.861716	13.684391	57.801863
RBM39	138.330936	297.512456	275.297832	412.897693	240.185172	332.566624	174.233102	291.021028
RBM39P1	0	0	0	0	0	0	0	0
RBM4	105.794645	123.481582	37.180715	111.63083	109.343383	108.473545	250.065424	138.081725
RBM41	2.401643	14.020289	10.809181	5.127244	15.796689	3.47505	0	6.789741
RBM42	43.84582	57.838904	32.202403	55.448988	35.784292	117.667689	46.955529	79.527606
RBM43	1.578969	3.093278	3.543384	1.825403	2.502003	0.868371	11.529522	2.533966
RBM43P1	0	0	0	0	0	0	0	0.143025
RBM44	0	0	0	0	0	0	0	0.21992
RBM45	7.785437	5.010938	3.471446	3.158272	21.393864	0.819203	0	2.170869
RBM46	0	0	0	0	0	0	0	0
RBM47	11.507161	30.830933	7.466458	40.793368	26.693785	54.670817	33.225677	45.177949
RBM48	5.624194	2.322193	16.573607	2.43489	1.277522	1.336941	4.793542	2.482947
RBM48P1	0	0	0	0	0	0	0	0
RBM4B	6.703689	18.038983	19.285505	21.457232	1.699897	33.671301	23.960166	31.286514
RBM5	2.955135	23.513024	7.820583	18.119983	32.814642	33.368799	22.951472	12.196791
RBM6	22.96854	33.363226	24.222137	38.526489	21.160122	44.330889	79.694509	36.65023
RBM7	21.371556	27.644854	1.721514	25.614683	69.368043	25.241461	75.183029	81.023695

RBM7P1	0	0	0	0	0	0	0	0
RBM8A	222.403926	296.689749	188.280317	314.282373	301.922361	307.243331	248.741981	272.686736
RBMS1	39.513199	63.062884	0	44.117277	0.988171	74.641289	0	73.605002
RBMS1P1	0	0	0	0	0	0	0	0
RBMS2	65.445059	31.343291	24.24633	32.926635	40.967032	33.121246	43.784103	36.472794
RBMS2P1	0	0	0	0.34937	0	0.198346	0	0.58327
RBMS3	20.950731	6.440718	17.832616	1.748528	6.063607	7.11341	0	3.985653
RBMX	177.519293	225.126982	228.954921	244.30917	216.387551	274.994902	333.654269	283.651105
RBMX2	28.839218	55.592556	48.013779	62.44167	39.522039	39.905938	19.994482	46.816089
RBMX2P1	0	0	0	0	0	0	0	0
RBMX2P2	0	0	0	0	0	0	0	0
RBMX2P3	0	0	0	0	0	0	0	0
RBMX2P4	0	0	0	0	0	0	0	0
RBMX2P5	0	0	0	0	0	0	0	0
RBMXL1	14.576982	66.501915	14.859854	54.462851	15.668761	36.308002	46.01171	45.570676
RBMXP1	0	0	0	0	0	0	0	0
RBMXP2	0	0	0	0	0.84385	0	0	0
RBMXP3	0	0	0	0	0	0	0	0
RBMXP4	0	0	0	0	0	0.32276	0	0
RBMXP5	0	0.72032	0	0	0	0	0	0
RBMY1A1	0	0	0	0	0	0	0	0
RBMY1A3P	0	0	0	0	0	0	0	0
RBMY1B	0	0	0	0	0	0	0	0
RBMY1D	0	0	0	0	0	0	0	0
RBMY1E	0	0	0	0	0	0	0	0
RBMY1F	0	0	0	0	0	0	0	0
RBMY1HP	0	0	0	0	0	0	0	0
RBMY1J	0	0	0	0	0	0	0	0
RBMY1KP	0	0	0	0	0	0	0	0
RBMY2BP	0	0	0	0	0	0	0	0
RBMY2CP	0	0	0	0	0	0	0	0
RBMY2EP	0	0	0	0	0	0	0	0
RBMY2FP	0	0	0	0	0	0	0	0

RBMY2GP	0	0	0	0	0	0	0	0
RBMY2HP	0	0	0	0	0	0	0	0
RBMY2JP	0	0	0	0	0	0	0	0
RBMY2KP	0	0	0	0	0	0	0	0
RBMY2NP	0	0	0	0	0	0	0	0
RBMY2OP	0	0	0	0	0	0	0	0
RBMY2QP	0	0	0	0	0	0	0	0
RBMY2TP	0	0	0	0	0	0	0	0
RBMY2UP	0	0	0	0	0	0	0	0
RBMY2WP	0	0	0	0	0	0	0	0
RBMY2XP	0	0	0	0	0	0	0	0
RBMY2YP	0	0	0	0	0	0	0	0
RBMY3AP	0	0	0	0	0	0	0	0
RBP1	0	0	0	0.066125	5.814199	0	0	0
RBP2	0	0	0	0	0	0	0	0
RBP4	9.999331	5.316816	21.997411	9.35174	0	0.344913	0	0.410322
RBP5	0	0	1.238161	0.506852	0	0	0	0
RBP7	0	33.024064	68.009816	33.122791	0	8.666085	0	8.721438
RBPJ	11.867668	47.504281	0	45.605065	19.427491	31.306027	27.358463	74.137479
RBPJL	0	0	0	0	0	0	0	0
RBPJP2	0	0	0	0	0	0	0	0
RBPJP5	0	0	0	0	0	0	0	0
RBPJP6	0	0	0	0	0	0	0	0
RBPJP7	0	0	0	0	0	0	0	0
RBPMS	30.544783	19.536116	38.016374	20.126803	23.61039	13.467495	0	8.006822
RBPMS2	0	0.727729	1.798325	0.417548	0	0.397991	0	0
RBPMS2P1	0	0	0	0	0	0	0	0
RBPMSLP	0	0	0	0	0	0	0	0
RBSN	21.348794	42.746582	25.784617	30.660402	18.505025	19.819476	31.782627	32.23618
RBX1	0	0	0	0.517806	0	0	0	0.163181
RBX1P1	0	0	0	0	0	0	0	0
RBX1P2	0	4.658509	0	1.009349	0	0.950719	0	1.970702
RC3H1	2.215897	25.559808	7.810098	28.100093	26.399651	30.210937	68.010395	24.403494

RC3H2	42.424393	67.748645	42.550066	58.051629	45.046906	50.734735	35.199592	89.322704
RCAN1	11.127438	18.286038	15.643923	18.096091	12.307258	15.718157	16.196818	22.915931
RCAN2	0	0	0	0	0	0	0	0
RCAN3	5.344203	43.030476	7.638401	21.263556	23.826641	29.73568	16.595937	35.174973
RCBTB1	9.545018	26.437684	13.313918	20.161094	6.09407	11.368332	35.434126	16.175045
RCBTB2	0	4.581933	1.157008	0	2.387421	0.672747	0	1.932154
RCBTB2P1	0	0	0	0	0	0	0	0
RCC1	51.14987	76.600551	58.526334	77.008271	116.089226	148.21758	59.472143	125.969996
RCC1L	44.881025	78.764068	24.494244	73.950151	62.239437	117.187364	68.640491	107.310306
RCC2	102.983162	100.616957	97.562547	118.813172	221.022307	250.783863	187.599445	201.977341
RCC2P1	0	0	0	0	0	0	0	0
RCC2P2	0	0	0	0	0	0	0	0
RCC2P3	0	0	0	0	0	0	0	0
RCC2P4	0	0	0	0	0	0	0	0
RCC2P5	0	0	0	0	0	0	0	0
RCC2P6	0	0	0	0	0	0	0	0.092988
RCC2P7	0	0	0	0	0	0	0	0
RCC2P8	0	0	0	0	0	0	0	0
RCCD1	10.420304	26.547612	4.999542	27.887967	7.234734	30.90101	26.41907	23.04697
RCE1	12.865801	5.617007	23.923759	11.813981	2.775897	4.401788	16.748351	10.370248
RCHY1	15.046684	24.392844	18.709894	6.855859	3.527687	3.8855	0	16.963685
RCL1	23.504364	61.141248	18.16375	52.144694	17.098449	63.641094	144.346323	74.988282
RCN1	0	0	0	0.102648	0	0	44.69087	0.682019
RCN1P1	0	0	0	0	0	0	0	0
RCN1P2	0	0	0	0.412225	0	0.894887	0	0.202856
RCN2	94.65181	112.483411	60.798185	47.01953	90.039414	41.850555	50.447146	96.838983
RCN3	7.437562	4.785253	7.403593	1.897771	0	0.722178	0	0.297914
RCOR1	36.453248	40.705635	15.774062	24.495721	26.176166	16.839238	167.897682	27.502882
RCOR2	0	3.855892	2.067416	2.81187	0.913657	5.694019	0	2.878092
RCOR3	14.193607	15.999182	28.601634	20.946446	29.283641	12.154849	196.239786	16.443222
RCSD1	0	0	0	0	0	0	0	0
RCVRN	0	0	0	0	0	0	0	0
RD3	0	0	0	0	0	0	0	0

RD3L	0	0	0	0	0	0	0	0
RDH10	25.254873	82.324156	23.225709	80.871126	6.11531	23.47724	0	25.687526
RDH11	121.937805	75.856212	100.221407	76.156577	95.159131	65.662809	107.171137	43.132179
RDH12	0	0	0	0	0	0.28308	0	0
RDH13	12.185433	17.560664	18.099411	26.251255	3.645735	44.746336	7.078782	27.414491
RDH14	11.449916	101.78623	38.49335	71.476919	10.252718	58.164539	0	101.31966
RDH16	0	0	0	0	0	0	0	0
RDH5	0	0	0	1.28337	0	0.434012	0	0
RDH8	0	0	0	0	0	0	0	0
RDM1	0	7.157982	5.44918	7.901593	0	2.819762	0	5.43711
RDM1P1	0	0	0	0	0	0	0	0
RDM1P3	0	0	0	0	0	0	0	0
RDM1P4	0	3.902022	0	0	0	0	0	5.083815
RDM1P5	0	0	0	0	0	0	0	0
RDX	0	94.788974	57.449131	67.943324	45.829454	107.094648	61.53969	106.706671
RDXP1	0	0	0	0	0	0	0	0
RDXP2	0	0	0	0	0	0	0	0
RDXP3	0	0	0	0.168384	0	0	0	0
REC114	0	0	0	0	0	0	0	0
REC8	0	0	1.346699	1.124673	0	1.094668	0	0.312271
RECK	2.05552	2.229896	0.669291	1.785911	0.877281	1.996019	8.444942	1.664862
RECQL	84.811111	126.024784	56.801444	70.761875	38.091721	49.837157	82.984773	123.334613
RECQL4	25.142107	12.139259	16.834053	11.321978	49.766171	11.269042	75.015164	6.676651
RECQL5	32.35549	22.775267	15.366003	19.867878	18.312916	17.789355	33.617388	19.663151
REDIC1	0	0	0	0	0	0	0	0
REELD1	0	0	0	0	0	0.160682	0	0
REEP1	0	2.063384	0	0.318805	0	0.200766	0	0.413572
REEP2	0	0	0	4.463895	6.828711	3.667245	48.825859	2.023222
REEP3	41.702349	124.51619	56.251501	81.699079	33.550755	89.945983	25.859998	60.779889
REEP4	17.009895	47.313637	36.09201	62.698078	14.56378	72.607054	0	57.029332
REEP5	67.553946	151.345493	57.993396	124.064241	90.461011	138.781153	139.094151	164.857121
REEP6	2.679302	9.74523	2.379495	7.701191	15.998927	12.200538	0	7.630529
REG1A	0	0	0	0	0	0	0	0

REG1B	0	0	0	0	0	0	0	0
REG1CP	0	0	0	0	0	0	0	0
REG3A	0	0	0	0	0	0	0	0
REG3G	0	0	0	0	0	0	0	0
REG4	0	0.694596	0	0.219087	0	0	0	0
REL	10.283731	6.652697	16.238397	5.561797	9.162352	4.191624	18.776304	8.213115
RELA	82.398273	125.402314	94.276547	157.867175	59.498696	119.781981	57.430952	77.252231
RELB	9.116668	0	11.536309	0.343626	7.601022	0.612966	0	1.049097
RELCH	30.116602	15.087741	10.214536	11.711511	41.640584	8.673221	19.50268	18.965052
RELL1	23.868583	16.48269	13.998758	23.835172	12.604225	19.545954	26.117493	29.386573
RELL2	3.199285	9.067357	4.287982	11.917831	18.103332	9.134184	1.136101	3.869828
RELN	0	0	0.249318	0.061043	0.073208	0.069628	0	0.099814
RELT	11.413628	16.246206	10.25797	17.674605	13.518608	19.816776	6.156821	12.605293
REM1	0	0	0	0	0	0	0	0
REM2	0	0.503264	0	0.707309	0	0.185961	0	0.152019
REN	2.416385	0.206722	0	0.65588	3.817454	0	0	0
RENBP	0	0	0	0.178176	0	0	0	0
REPIN1	30.606288	37.262511	24.722451	46.097818	40.638674	91.991385	0	72.459096
REPS1	75.347595	57.02003	62.881492	44.113419	47.912155	57.276484	8.189733	82.207096
REPS2	5.661393	2.599116	4.110615	16.35289	7.349927	13.579847	2.95922	8.79494
RER1	116.52902	177.234728	128.634806	146.232057	177.094068	145.540415	478.821403	160.712332
RERE	36.415811	28.388227	46.597642	55.427198	126.084511	85.836526	45.893812	60.144622
REREP1Y	0	0	0	0	0	0	0	0
REREP2Y	0	0	0	0	0	0	0	0
RERG	0	0	0	0.253974	0.691671	0.366317	0	0
RERGL	0	0	0	0	0	2.564847	42.864777	2.002409
RESF1	8.856105	17.152193	4.683408	6.685735	5.369412	10.592508	9.524226	22.706464
RESP18	0	0	0	0	0	0	0	0
REST	22.117636	4.088369	0	0	18.558124	16.078563	0	0
RET	0	0	0	0	0	0	0	0
RETN	0	0	0	0	0	0	0	0
RETNLB	0	0	0	0	0	0	0	0
RETREG1	0	1.005857	0	0.363635	3.652294	0.469644	0	1.470479

RETREG2	18.02179	90.827896	23.304212	75.478604	50.98115	192.222527	67.730228	164.975944
RETREG3	33.59333	49.369685	43.621772	49.911023	24.043269	50.924579	26.667108	38.930431
RETSAT	23.447159	13.076459	21.014211	14.252511	3.3759	6.935129	23.090545	5.1297
REV1	34.258632	26.582246	15.939419	26.507923	44.962847	49.591798	35.320977	61.224552
REV3L	24.394554	11.469554	0	4.06537	56.856846	14.306139	89.164362	30.896424
REX1BD	0	32.97344	5.29686	31.995573	10.67912	53.405909	10.960328	37.193864
REXO1	8.546859	17.682792	19.013846	14.861443	6.772559	41.311356	6.760618	25.225112
REXO1L10P	0	0	0	0	0	0	0	0
REXO1L11P	0	0	0	0	0	0	0	0
REXO1L12P	0	0	0	0	0	0	0	0
REXO1L1P	0	0	0	0.085495	0	0	0	0
REXO1L2P	0	0	0	0	0	0	0	0
REXO1L3P	0	0	0	0	0	0	0	0
REXO1L4P	0	0	0	0	0	0	0	0
REXO1L5P	0	0	0	0	0	0	0	0
REXO1L6P	0	0	0	0	0	0	0	0
REXO1L8P	0	0	0	0	0	0	0	0
REXO1L9P	0	0	0	0	0	0	0	0
REXO2	101.362101	97.889118	84.153209	53.265379	150.294626	157.733091	231.656434	174.736665
REXO4	35.309217	54.167082	27.626166	76.711658	28.71597	85.386186	40.665982	65.000179
REXO5	8.229774	3.342391	6.952673	5.141646	17.149295	5.566008	0	4.74783
REXO6P	0	0	2.351928	0	0	0	0	0
RFC1	47.752578	79.671659	56.825352	91.767655	83.052325	51.882316	224.316704	56.698956
RFC2	37.787935	50.809918	61.727244	62.375098	81.552541	67.934607	68.765404	56.828179
RFC3	47.522594	22.642323	31.867778	13.088104	34.916699	12.119572	44.188494	22.509448
RFC3P1	0	0	0	0	0	0	0	0
RFC4	0	133.936502	7.119982	123.069948	40.982606	121.451988	162.355788	114.932993
RFC5	49.238733	68.36674	54.15497	65.020021	95.87403	62.980421	98.598895	86.75453
RFC5P1	0	0	0	0	0	0	0	0
RFESD	0	0.950916	0	1.853228	0	2.793465	2.573175	2.810365
RFESDP1	0	0	0	0	0	0	0	0
RFFL	0	17.32806	14.11161	22.777811	22.895549	33.818015	11.972306	7.559523
RFK	10.017959	23.916325	46.017554	10.584087	33.563576	14.179794	19.783515	25.632905

RFKP1	0	0	0	0.585548	0	0	0	0
RFKP2	0	0	0	0	0	0	0	0
RFKP3	0	0	0	0	0	0	0	0
RFKP4	0	0	0	0	0	0	0	0
RFKP5	0	0	0	0	0	0	0	0
RFKP6	0	0	0	0	0	0	0	0
RFLNA	0	1.069374	0	0	0	0	0	0
RFLNB	22.923854	25.574527	16.450062	17.541318	14.284334	18.344716	16.726808	23.732182
RFNG	67.477316	28.459357	6.689592	28.321169	112.054556	22.499227	147.252447	38.048587
RFPL1	0	0	0	0	0	0	0	0
RFPL2	0	0	0	0	0	0	0	0
RFPL3	0	0	0	0	0	0	0	0
RFPL4AP1	0	0	0	0	0	0	0	0
RFPL4AP3	0	0	0	0	0	0	0	0
RFPL4AP5	0	0	0	0	0	0	0	0
RFPL4AP6	0	0	0	0	0	0	0	0
RFPL4AP7	0	0	0	0	0	0	0	0
RFPL4B	0	0.310814	0	0.323464	0	0.36342	0	0.593756
RFT1	23.663195	15.383461	20.388738	11.505769	6.523237	8.9285	24.593332	10.965129
RFTN1	7.873095	12.139311	8.164499	12.48664	17.112471	15.014103	12.491738	19.743812
RFTN1P1	0	0	0	0	0	0.937538	0	0
RFTN2	0	0	0	0	0	0	0	0
RFWD3	76.49733	117.791442	48.224137	133.131719	103.917464	202.540685	118.884677	201.967012
RFX1	7.520014	7.159291	7.426915	8.188776	1.588919	17.853066	8.238333	11.054105
RFX2	14.470024	17.973036	4.60073	26.419169	50.219277	35.143535	8.305804	38.939488
RFX3	4.302201	2.703454	0	5.594315	8.686019	3.691776	0	2.25721
RFX4	0	0	0	0	0	0	0	0
RFX5	26.891165	27.441945	15.463758	20.867009	6.966263	13.161588	48.186358	33.58516
RFX6	0	0	0	0	0	0	0	0
RFX8	0	0	0	0.670301	0	0	0	0
RFXANK	38.625146	78.667956	22.084695	73.408	31.864763	173.626651	35.581729	130.308821
RFXAP	0	0	0	0	0	0	0	0
RGCC	8.140305	14.877991	0	13.849436	0	2.551961	0	3.656834

RGL1	0	2.09188	0	1.566835	0	1.778276	0	2.186382
RGL2	23.503198	23.933138	13.735397	21.08841	19.528362	39.700675	13.121958	21.623566
RGL3	11.338411	5.466921	2.20418	1.784186	1.463971	1.027753	0	0.567803
RGL4	0	0	0	0	0	0	0	0
RGL4P1	0	0	0	0	0	0	0	0
RGMA	0	4.353363	0	3.225116	0	7.090716	0	1.897346
RGMB	1.442623	18.645768	14.164036	10.878068	0	8.384655	0	0.259356
RGN	4.416328	12.219539	0	9.84661	0	4.043647	0	5.522598
RGP1	0	9.561548	0	4.596502	0	11.267043	11.835821	4.382215
RGPD1	0	0	0	0	0	0	0	0
RGPD2	0	2.242222	0	0.424339	0	0	0	0
RGPD3	0	0.037844	0	0.018697	0	0	0	0.123111
RGPD5	4.888744	5.891246	0	0.22103	0	4.310453	36.684335	0.736166
RGPD6	0	1.027384	0	1.688461	10.05115	1.335514	0	12.897785
RGPD8	0	0	0	0.298975	0	0	0	0
RGR	0	0	0	0	0	0	0	0
RGS1	0	0	0	0	0	0	0	0
RGS10	39.793181	61.045839	82.548246	61.499356	42.503373	33.44222	2.692965	53.448333
RGS11	0	1.285451	2.357781	0.899173	0	0.071362	0	0.1857
RGS12	7.113076	24.824083	6.602107	29.23923	2.813038	40.951405	76.700511	27.247429
RGS13	0	0	0	0	0	0	0	0
RGS14	19.202764	22.327035	12.609052	16.833365	17.768387	14.37099	17.112224	10.689828
RGS16	2.833965	4.21527	3.802277	6.31399	0.747572	3.728624	0	1.826515
RGS17	1.627144	1.243837	2.615158	1.357067	0.537535	0.142976	0	0.233009
RGS17P1	0	0	0	0.597762	0	0	0	0.712046
RGS18	0	0	0	0	0	0	0	0
RGS19	11.881531	29.333222	16.912054	30.093325	1.877207	35.182023	0	35.346368
RGS2	8.120744	13.122886	14.421867	9.282893	4.986503	3.577516	0	5.707971
RGS20	0	29.299728	4.481347	26.729604	4.353816	10.707763	0	6.445223
RGS21	0	0	0	0	0	0	0	0
RGS22	0	0	0	0.037869	0	0	0	0
RGS3	14.101032	35.475748	10.146465	27.597328	6.87416	26.144331	6.030947	15.365909
RGS4	0	0.38766	0	0	0	0	0	0.209528

RGS5	0	18.054585	6.852275	17.031902	0.151784	10.615937	0	13.16505
RGS6	0	0	0	0.029872	0	0	0	0.098047
RGS7	0	0	0	0	0	0.296217	0	0
RGS7BP	0	0	0	0	0	0	0	0
RGS8	0	0	0	0.014211	0	0	0	0
RGS9	1.234891	13.505167	3.317082	14.135095	0.651724	2.087269	0	6.187221
RGSL1	0	0	0	0	0	0	0	0
RHAG	0	0	0	0	0	0	0	0
RHBDD1	16.215786	35.875598	17.430432	61.722006	25.898768	41.42906	2.287024	46.547771
RHBDD2	31.796805	28.079084	21.926749	21.360482	30.757582	20.439402	0	24.040473
RHBDD3	6.902589	8.899992	10.824238	13.28779	10.083229	28.524985	0	18.623715
RHBDF1	11.22325	7.075925	1.099279	5.657038	12.506272	8.967309	61.161467	9.407749
RHBDF1P1	0	0	0	0.043542	0	0.088676	0	0.072387
RHBDF2	36.834307	48.065112	41.574155	88.594155	22.259547	103.100021	14.183352	80.822168
RHBDL1	0	3.212625	0	0.126764	0	0	0	0
RHBDL2	9.273669	11.371468	9.929218	23.520081	15.643667	17.63947	33.358132	17.164578
RHBDL3	0	0.867412	0	0.562813	0	0.110359	0	0.118655
RHBG	0	0	0	0.265588	0	0.410334	0	0
RHCE	0	0.409009	9.735358	0.820256	0	0.191332	0	0.078275
RHCG	0	0	0	0	0	0	0	0
RHD	0	0	0	0.098418	0	0	0	0
RHEB	78.176631	155.977956	72.404366	67.229398	109.837321	56.040368	55.382519	142.886681
RHEBL1	3.58418	20.839747	12.667998	27.350369	0	6.569962	0	3.472744
RHEBP1	27.415732	5.924718	0	0	22.951902	0	67.93018	12.184126
RHEBP2	0	10.861351	0	6.731607	0	2.253495	0	2.368569
RHEBP3	0	0	0	0	0	0	0	0
RHEX	0	0	0	0	0	0	0	0
RHNO1	65.886693	168.228494	67.806792	174.573147	124.644813	172.253724	55.112704	126.133596
RHOA	295.481685	358.960283	312.048967	370.252272	300.007902	252.950769	203.080511	387.797936
RHOBTB1	3.792582	8.479413	2.042995	15.441344	2.804663	5.557027	0	7.364469
RHOBTB2	14.520807	20.026367	8.293153	17.934007	10.286961	35.15767	8.038437	6.825338
RHOBTB3	38.287094	90.704828	40.529067	90.543305	87.136579	94.187578	26.607994	153.650951
RHOC	189.740571	171.725544	149.232264	155.21676	132.486329	218.208002	218.682393	156.287515

RHOD	143.689926	212.438683	136.288338	179.637807	111.601555	180.100544	113.341234	179.409849
RHOF	20.965552	59.034611	37.423604	94.069499	0	11.567158	0	11.111656
RHOG	31.196004	98.059484	35.230177	102.408333	25.681938	175.089001	22.759039	190.096794
RHOG2P	0	0	0	0	0	0	0	0
RHOH	0	0	0	0	0	0	0	0
RHOJ	0	0	0	0	0	1.621222	0	0.64983
RHOQ	4.800186	12.378991	6.957305	4.699316	17.747957	3.869895	32.055954	13.802387
RHOQP1	8.166154	5.477993	0	7.338999	0	6.348692	0	12.23032
RHOQP2	0	0	0	0	0	0	0	0.365941
RHOQP3	0	0	0	0	0	0	0	0
RHOT1	34.975494	13.044492	51.092243	8.174407	22.0015	3.083401	27.870194	17.467936
RHOT1P1	0	0	0	0	0	0	0	0
RHOT1P2	0	0	0	0.896009	0	1.697927	0	0
RHOT1P3	0	0	0	0	0	0	0	0
RHOT2	20.499067	23.737092	24.45427	39.32017	28.738923	47.430948	0	37.712133
RHOU	0	1.856171	0.748033	1.302307	4.622446	1.166296	0	0.87037
RHOV	10.747182	14.657561	11.48776	17.080817	2.264932	23.500354	0	10.255501
RHOXF1	0	0	0	0.591682	0	0	0	0
RHPN1	3.630847	2.688551	4.071277	2.928756	0.237014	1.479199	0	0.793357
RHPN2	9.50123	14.661655	20.450276	15.959849	28.637789	21.455189	40.57716	27.81653
RHPN2P1	0	0	0	0	0	0	0	0
RIBC1	0	5.143625	2.548026	7.179274	0	6.060941	0	3.662193
RIBC2	0	2.9027	0	3.918661	0	3.060831	0	3.760791
RIC1	19.019728	13.013887	16.35705	14.480604	26.672174	21.425636	10.903417	29.058458
RIC3	0	1.580273	0	0.847749	1.403753	8.783585	0	5.15791
RIC8A	55.939689	48.469316	9.970184	20.868368	39.50283	67.779385	65.715824	25.137809
RIC8B	9.382629	18.78522	1.746321	11.906936	2.952818	14.063752	24.397578	24.333915
RICTOR	7.435904	3.588336	27.281389	2.268256	10.2963	3.26238	74.682873	1.879316
RIDA	39.232344	23.085236	44.980316	14.834948	21.613056	7.770291	49.730487	12.036456
RIF1	8.845972	22.467393	43.16736	23.385841	49.624097	25.054865	76.209985	52.948172
RIGI	15.602254	4.410975	0	2.244466	27.005411	9.996581	12.261287	16.594396
RIIAD1	0	0	0	0	0	0	0	0
RILP	6.65385	11.803147	8.851602	11.941156	13.992327	10.052853	0	6.813386

RILPL1	23.803527	30.19612	23.659706	29.31773	6.812555	32.167633	6.261317	18.547582
RIMBP2	0	1.632658	0	2.783833	0	1.885869	0	0.935049
RIMBP3	0	0.469164	0	0.92574	0	0.950507	0	0.239139
RIMKLA	1.213055	2.917985	7.473879	3.65071	0	1.482145	3.848865	2.481942
RIMKLB	5.311437	27.701496	9.489238	30.458933	14.780358	39.773103	9.05595	31.524273
RIMKLBP1	0	0	0	0	0	0	0	0
RIMKLBP2	0	0	0	0	0	0	0	0
RIMOC1	37.979674	12.67355	37.712676	9.156376	46.735643	6.741214	31.702459	18.296426
RIMS1	0	0.905288	0	0.219776	0	0	0	0.484873
RIMS2	6.887179	12.227948	5.596845	7.909253	0	7.632667	0	8.749702
RIMS3	0.445479	0.115052	0	0.30519	0	1.09745	0	0.482094
RIMS4	0	0	6.443726	1.170062	2.38202	0	23.908192	0
RIN1	27.621601	10.262161	30.833999	14.589495	18.82178	12.34878	11.548049	5.258507
RIN2	70.855268	92.699118	61.275926	106.742669	28.715477	76.510547	19.743295	68.794539
RIN3	30.785157	42.218146	10.914536	34.659252	10.209873	52.767768	4.688934	39.118811
RING1	11.687676	54.270442	27.060483	61.136159	38.395534	87.774919	22.50287	61.743642
RINL	5.813947	4.263697	5.185002	6.027082	3.06437	2.196052	0	2.402914
RINT1	10.599101	14.588261	24.866272	12.928566	9.323921	13.473635	0.313094	27.275972
RIOK1	20.428209	42.917375	18.277795	36.953031	11.497192	43.236201	33.701999	46.27378
RIOK2	62.336502	62.680738	21.678293	32.601731	42.314527	10.152812	35.232858	24.545004
RIOK3	37.186623	41.555107	47.589162	47.621692	99.489191	32.583391	11.98328	49.1258
RIOK3P1	0	0	0	0	0	0	0	0
RIOX2	80.306333	87.618972	85.889397	66.756642	66.746961	48.337147	52.777498	66.836519
RIPK1	0	16.573825	0	38.863341	4.040254	31.723723	0	39.480477
RIPK2	28.021513	33.398349	15.719443	26.556535	7.812735	27.287694	18.051515	29.35834
RIPK3	0	0	0	0	0	0	0	0
RIPK4	18.147367	19.457831	20.159877	22.283862	19.396174	41.302061	44.992647	34.752852
RIPOR1	36.812862	70.961665	39.21189	89.649863	158.472755	106.051149	73.440383	87.053251
RIPOR2	0	0	0	0.015073	0	0.642577	0	0
RIPOR3	0	1.333268	0	2.433579	0	0	0	0.249158
RIPPLY1	0	0	0	0	0	0	0	0
RIPPLY2	0	3.593988	7.98288	6.078795	0	1.278458	0	2.345795
RIPPLY3	1.852554	2.231177	6.611779	3.598574	4.394627	1.14092	0	2.557249

RIT1	20.869972	11.98849	9.426635	25.834655	29.361228	20.514914	7.625039	20.00281
RIT2	0	0	0	0	0	0	0	0
RITA1	31.427203	68.702665	31.906987	62.128393	33.737403	87.002487	50.546169	90.22017
RLBP1	0	1.929417	0	1.679999	0	0	0	0
RLF	17.718066	8.156809	17.802816	4.116565	11.841715	4.25958	14.354565	11.952057
RLIG1	45.629262	51.862784	62.806963	37.723347	61.388136	60.465767	19.635644	100.161862
RLIG1P1	0	0	0	0	0	0	0	0
RLIG1P2	0	0	0	0	0	0	0	0
RLIG1P3	0	0	0	0	0	0	0	0
RLIM	16.891312	53.138132	17.241775	61.027597	23.679705	51.678464	59.232039	66.002764
RLIMP1	0	0	0	0	0	0	0	0
RLIMP2	0	0	0	0	0	0	0	0
RLN1	0	0	0	0.645583	0	0	0	1.11659
RLN2	5.736394	4.845628	0	0.272327	0	2.025124	0	0
RLN3	0	0	0	0	0	0	0	0
RMB8AP1	0	1.723518	0	0.466621	0	0	0	0
RMC1	19.699195	0	0	2.402776	8.664532	4.593454	18.1904	0
RMDN1	59.170403	48.200092	54.805482	29.725651	78.77601	12.48588	44.358765	24.617408
RMDN2	9.75185	2.602381	0	0.961159	0	0	0	0
RMDN3	22.924281	35.215619	25.923449	50.920048	54.675727	75.458736	54.078637	39.141601
RMI1	3.152673	5.466337	5.414518	1.469608	1.638056	0.932229	18.943938	8.58852
RMI2	72.643435	66.909066	81.111877	44.801968	16.682739	23.644835	1.181381	30.60823
RMND1	28.766055	13.070606	38.453442	15.148651	25.900295	8.036205	0	20.017412
RMND5A	42.037758	68.834653	34.320766	43.959272	47.772768	69.574508	26.941845	87.280756
RMND5B	16.492029	28.792546	16.256107	26.124555	31.342247	38.76611	13.731213	27.187445
RNA5-8SP2	0	0	0	0	0	0	0	0
RNA5-8SP4	0	0	0	0	0	0	0	0
RNA5-8SP5	0	0	0	0	0	0	0	0
RNA5-8SP6	703.332104	830.533767	2307.63389	50.80664	2513.21132	54.947031	993.183838	144.0406
RNA5-8SP7	0	0	0	0	0	0	0	0
RNA5SP100	0	0	0	0	0	0	0	0
RNA5SP101	0	0	0	0	0	0	0	0
RNA5SP102	0	0	0	0	0	0	0	0

RNA5SP103	0	0	0	0	0	0	0	0
RNA5SP104	0	0	0	0	0	0	0	0
RNA5SP105	0	0	0	0	0	0	0	0
RNA5SP106	0	0	0	0	0	0	0	0
RNA5SP107	0	0	0	0	0	0	0	0
RNA5SP108	0	0	0	0	0	0	0	0
RNA5SP109	0	0	0	0	0	0	0	0
RNA5SP110	0	0	0	0	0	0	0	0
RNA5SP111	0	0	0	0	0	0	0	0
RNA5SP112	0	0	0	0	0	0	0	0
RNA5SP113	0	0	0	0	0	0	0	0
RNA5SP114	0	0	0	0	0	0	0	0
RNA5SP115	0	0	0	0	0	0	0	0
RNA5SP116	0	0	0	0	0	0	0	0
RNA5SP117	0	0	0	0	0	0	0	0
RNA5SP118	0	0	0	0	0	0	0	0
RNA5SP119	0	0	0	0	0	0	0	0
RNA5SP120	0	0	0	0	0	0	0	0
RNA5SP121	0	0	0	0	0	0	0	0
RNA5SP122	0	0	0	0	0	0	0	0
RNA5SP123	0	0	0	0	0	0	0	0
RNA5SP124	0	0	0	0	0	0	0	0
RNA5SP125	0	0	0	0	0	0	0	0
RNA5SP126	0	0	0	0	0	0	0	0
RNA5SP127	0	0	0	0	0	0	0	0
RNA5SP128	0	0	0	0	0	0	0	0
RNA5SP129	0	0	0	0	0	0	0	0
RNA5SP130	0	0	0	0	0	0	0	0
RNA5SP131	0	0	0	0	0	0	0	0
RNA5SP132	0	0	0	0	0	0	0	0
RNA5SP133	0	0	0	0	0	0	0	0
RNA5SP134	0	0	0	0	0	0	0	0
RNA5SP135	0	0	0	0	0	0	0	0

RNA5SP136	0	0	0	0	0	0	0	0
RNA5SP137	0	0	0	0	0	0	0	0
RNA5SP138	0	0	0	0	0	0	0	0
RNA5SP139	0	0	0	0	0	0	0	0
RNA5SP140	0	0	0	0	0	0	0	0
RNA5SP141	0	5.31253	0	0	0	0	37.662194	0
RNA5SP142	0	0	0	0	0	0	0	0
RNA5SP143	0	0	0	0	0	0	0	0
RNA5SP144	0	0	0	0	0	0	0	0
RNA5SP145	0	0	0	14.335901	0	7.535327	0	0
RNA5SP146	0	0	0	0	0	0	0	0
RNA5SP147	0	0	0	0	0	0	0	0
RNA5SP148	0	0	0	0	0	0	0	0
RNA5SP149	0	0	0	19.956742	0	74.996857	0	0
RNA5SP151	0	0	0	0	0	0	0	0
RNA5SP152	0	0	0	0	0	0	0	0
RNA5SP153	0	0	0	0	0	0	0	0
RNA5SP154	0	0	0	0	0	0	0	0
RNA5SP155	0	0	0	0	0	0	0	0
RNA5SP156	0	0	0	0	0	0	0	0
RNA5SP157	0	0	0	0	0	0	0	0
RNA5SP158	0	0	0	0	0	0	0	0
RNA5SP159	0	0	0	0	0	0	0	0
RNA5SP160	0	0	0	0	0	0	0	0
RNA5SP161	0	0	0	0	0	0	0	0
RNA5SP162	0	0	0	0	0	0	0	0
RNA5SP163	0	0	0	0	0	0	0	0
RNA5SP164	0	0	0	0	0	0	0	0
RNA5SP165	0	0	0	0	0	0	0	0
RNA5SP166	0	0	0	0	0	0	0	0
RNA5SP167	0	0	0	0	0	0	0	0
RNA5SP168	0	0	0	0	0	0	0	0
RNA5SP169	0	0	0	0	0	0	0	0

RNA5SP170	0	0	0	0	0	0	0	0
RNA5SP171	0	0	0	0	0	0	0	0
RNA5SP173	0	0	0	0	0	0	0	0
RNA5SP174	0	0	0	27.298383	0	0	0	0
RNA5SP175	0	0	0	0	0	0	0	0
RNA5SP176	0	0	0	0	0	0	0	0
RNA5SP177	0	0	0	0	0	0	0	0
RNA5SP178	0	0	0	0	0	0	0	0
RNA5SP179	0	0	0	0	0	0	0	0
RNA5SP18	0	0	0	0	0	0	0	0
RNA5SP180	0	0	0	0	0	0	0	0
RNA5SP181	0	0	0	0	0	0	0	0
RNA5SP182	0	0	0	0	0	0	0	0
RNA5SP183	0	0	0	0	0	0	0	0
RNA5SP184	0	0	0	0	0	0	0	0
RNA5SP185	0	0	0	0	0	0	0	0
RNA5SP186	0	0	0	0	0	0	0	0
RNA5SP187	0	0	0	0	0	0	0	0
RNA5SP188	0	0	0	0	0	0	0	0
RNA5SP189	0	0	0	0	0	0	0	0
RNA5SP19	0	0	0	0	0	0	0	0
RNA5SP190	0	0	0	0	0	0	0	0
RNA5SP191	0	0	0	0	0	0	0	0
RNA5SP192	0	0	0	0	0	0	0	0
RNA5SP193	0	0	0	0	0	0	0	0
RNA5SP195	0	0	0	0	0	0	0	0
RNA5SP196	0	0	0	0	0	0	0	0
RNA5SP197	0	0	0	0	0	0	0	0
RNA5SP198	0	0	0	0	0	0	0	0
RNA5SP199	0	0	0	0	0	0	0	0
RNA5SP20	0	0	0	0	0	0	0	0
RNA5SP200	0	0	0	0	0	0	0	0
RNA5SP201	0	0	0	0	0	0	0	0

RNA5SP202	2542.68672	919.984265	9449.22565	498.21756	5897.00147	2794.38679	4314.35447	353.608654
RNA5SP203	0	0	0	0	0	0	0	0
RNA5SP204	0	0	0	0	0	0	0	0
RNA5SP205	0	0	0	0	0	0	0	0
RNA5SP206	0	0	0	0	0	0	0	0
RNA5SP207	0	0	0	0	0	0	0	0
RNA5SP208	0	0	0	0	0	0	0	0
RNA5SP209	0	0	0	0	0	0	0	0
RNA5SP21	0	0	0	0	0	0	0	0
RNA5SP210	0	0	0	0	0	0	0	0
RNA5SP211	0	0	0	0	0	0	0	0
RNA5SP212	0	0	0	0	0	0	0	0
RNA5SP213	0	0	0	0	0	0	0	0
RNA5SP214	0	0	0	0	0	0	0	0
RNA5SP215	0	0	0	0	0	0	0	0
RNA5SP216	0	0	0	0	0	0	0	0
RNA5SP217	0	0	0	0	0	0	0	0
RNA5SP218	0	0	0	0	0	0	0	0
RNA5SP219	0	0	0	0	0	0	0	0
RNA5SP22	0	0	0	0	0	0	0	0
RNA5SP220	0	0	0	0	0	0	0	0
RNA5SP221	0	0	0	0	0	0	0	0
RNA5SP222	0	0	0	0	0	0	0	0
RNA5SP223	0	0	0	0	0	0	0	0
RNA5SP224	0	0	0	0	0	0	0	0
RNA5SP225	0	0	0	0	0	0	0	0
RNA5SP226	9298.76489	0	1438.34769	0	2454.62222	0	11273.1218	305.647327
RNA5SP227	0	0	0	0	0	0	0	0
RNA5SP228	0	0	0	0	0	0	0	0
RNA5SP229	0	0	0	0	0	0	0	0
RNA5SP23	0	0	0	0	0	0	0	0
RNA5SP230	0	0	0	0	0	0	0	0
RNA5SP231	0	0	0	0	0	0	0	0

RNA5SP232	0	0	0	0	0	0	0	0
RNA5SP233	0	0	0	0	0	0	0	0
RNA5SP234	0	0	0	0	0	0	0	0
RNA5SP235	0	0	0	0	0	0	0	0
RNA5SP236	0	0	0	0	0	0	0	0
RNA5SP237	0	0	0	0	0	0	0	0
RNA5SP238	0	0	0	0	0	0	0	0
RNA5SP239	0	0	0	0	0	0	0	0
RNA5SP24	0	0	0	0	0	0	0	0
RNA5SP240	0	0	0	0	0	0	0	0
RNA5SP241	0	0	0	0	0	0	0	0
RNA5SP242	0	0	0	0	0	0	0	0
RNA5SP243	0	0	0	0	0	0	0	0
RNA5SP244	0	0	0	0	0	0	0	0
RNA5SP245	0	0	0	0	0	0	0	0
RNA5SP246	0	0	0	0	0	0	0	0
RNA5SP247	0	0	0	0	0	0	0	0
RNA5SP248	0	0	0	0	0	0	0	0
RNA5SP249	0	0	0	0	0	0	0	0
RNA5SP25	0	0	0	0	0	0	0	0
RNA5SP251	0	0	0	0	0	0	0	0
RNA5SP252	0	0	0	0	0	0	0	0
RNA5SP253	0	0	0	0	0	0	0	0
RNA5SP255	0	0	0	0	0	0	0	0
RNA5SP256	0	0	0	0	0	0	0	0
RNA5SP257	0	0	0	0	0	0	0	0
RNA5SP258	0	0	0	0	0	0	0	0
RNA5SP259	0	0	0	0	0	0	0	0
RNA5SP26	0	0	0	0	0	0	0	0
RNA5SP260	0	0	0	0	0	0	0	0
RNA5SP261	0	0	0	0	0	0	0	0
RNA5SP262	0	0	0	0	0	0	0	0
RNA5SP263	0	0	0	7.420843	0	0	0	0

RNA5SP264	0	0	0	0	0	0	0	0
RNA5SP265	0	0	0	0	0	0	0	0
RNA5SP266	0	0	0	0	0	0	0	0
RNA5SP267	0	0	0	0	0	0	0	0
RNA5SP268	0	0	0	0	0	0	0	0
RNA5SP269	0	0	0	0	0	0	0	0
RNA5SP27	0	0	0	0	0	0	0	0
RNA5SP270	0	0	0	0	0	0	0	0
RNA5SP271	0	0	0	0	0	0	0	0
RNA5SP272	0	0	0	0	0	0	0	0
RNA5SP273	0	0	0	0	0	0	0	0
RNA5SP274	0	0	0	0	0	0	0	0
RNA5SP275	0	0	0	0	0	0	0	0
RNA5SP276	0	0	0	0	0	0	0	0
RNA5SP277	0	0	0	0	0	0	0	0
RNA5SP278	0	0	0	0	0	0	0	0
RNA5SP279	0	0	0	0	0	0	0	0
RNA5SP28	0	0	0	0	0	0	0	0
RNA5SP280	0	0	0	0	0	0	0	0
RNA5SP281	0	0	0	0	0	0	0	0
RNA5SP282	0	0	0	0	0	0	0	0
RNA5SP283	0	0	0	40.090077	0	31.353368	0	26.669219
RNA5SP284	0	0	0	0	0	0	0	0
RNA5SP285	0	0	0	0	0	0	0	0
RNA5SP286	0	0	0	0	0	0	0	0
RNA5SP287	0	0	0	0	0	0	0	0
RNA5SP288	0	0	0	0	0	0	0	0
RNA5SP289	0	0	0	0	0	0	0	0
RNA5SP29	0	0	0	0	0	0	0	0
RNA5SP290	0	0	0	0	0	0	0	0
RNA5SP291	0	0	0	0	0	0	0	0
RNA5SP292	0	0	0	0	0	0	0	0
RNA5SP293	0	0	0	0	0	0	0	0

RNA5SP294	0	0	0	0	0	0	0	0
RNA5SP295	0	0	0	0	0	0	0	0
RNA5SP296	0	0	0	0	0	0	0	0
RNA5SP297	0	0	0	0	0	0	0	0
RNA5SP298	0	19.70945	0	0	0	0	0	0
RNA5SP299	0	0	0	0	0	0	0	0
RNA5SP30	0	0	0	0	0	0	0	0
RNA5SP300	0	0	0	0	0	0	0	0
RNA5SP301	0	0	0	0	0	0	0	0
RNA5SP302	0	0	0	0	0	0	0	0
RNA5SP303	0	0	0	0	0	0	0	0
RNA5SP304	0	0	0	0	0	0	0	0
RNA5SP305	0	0	0	0	0	0	0	0
RNA5SP306	0	0	0	0	0	0	0	0
RNA5SP307	0	0	0	0	0	0	0	0
RNA5SP308	0	0	0	0	0	0	0	0
RNA5SP309	0	0	0	0	0	0	0	0
RNA5SP31	0	0	0	0	0	0	0	0
RNA5SP310	0	0	0	0	0	0	0	0
RNA5SP311	0	0	0	0	0	0	0	0
RNA5SP315	0	0	0	0	0	0	0	0
RNA5SP318	0	0	0	0	0	0	0	0
RNA5SP319	0	0	0	0	0	0	0	0
RNA5SP320	0	0	0	0	0	0	0	0
RNA5SP321	0	0	0	0	0	0	0	0
RNA5SP322	0	0	0	0	0	0	0	0
RNA5SP323	0	0	0	0	0	0	0	0
RNA5SP324	0	0	0	0	0	0	0	0
RNA5SP325	0	0	0	0	0	0	0	0
RNA5SP326	0	0	0	0	0	0	0	0
RNA5SP327	0	0	0	0	0	0	0	0
RNA5SP328	0	0	0	0	0	0	0	0
RNA5SP329	0	0	0	0	0	0	0	0

RNA5SP33	0	0	0	0	0	0	0	0
RNA5SP330	0	0	0	0	0	0	0	0
RNA5SP331	0	0	0	0	0	0	0	0
RNA5SP332	0	0	0	0	0	0	0	0
RNA5SP334	0	0	0	0	0	0	0	0
RNA5SP335	0	0	0	0	0	0	0	0
RNA5SP336	0	0	0	0	0	0	0	0
RNA5SP337	0	0	0	0	0	0	0	0
RNA5SP338	0	0	0	0	0	0	0	0
RNA5SP339	0	0	0	0	0	0	0	0
RNA5SP34	0	0	0	0	0	0	0	0
RNA5SP340	0	0	0	0	0	0	0	0
RNA5SP341	0	0	0	0	0	0	0	0
RNA5SP344	0	0	0	0	0	0	0	0
RNA5SP345	0	0	0	0	0	0	0	0
RNA5SP346	0	0	0	0	0	0	0	0
RNA5SP347	0	0	0	0	0	0	0	0
RNA5SP349	0	0	0	0	0	0	0	0
RNA5SP35	0	0	0	0	0	0	0	0
RNA5SP350	0	0	0	0	0	0	0	0
RNA5SP351	0	0	0	0	0	0	0	0
RNA5SP352	0	0	0	0	0	0	0	0
RNA5SP353	0	0	0	0	0	0	0	0
RNA5SP354	0	0	0	0	0	0	0	0
RNA5SP355	0	0	0	0	0	0	0	0
RNA5SP356	0	0	0	0	0	0	0	0
RNA5SP357	0	0	0	0	0	0	0	0
RNA5SP358	0	0	0	0	0	0	0	0
RNA5SP359	0	0	0	0	0	0	0	0
RNA5SP36	0	0	0	0	0	0	0	0
RNA5SP360	0	0	0	0	0	0	0	0
RNA5SP361	0	0	0	0	0	0	0	0
RNA5SP362	0	0	0	0	0	0	0	0

RNA5SP363	0	0	0	0	0	0	0	0
RNA5SP364	0	0	0	0	0	0	0	0
RNA5SP365	0	0	0	0	0	0	0	0
RNA5SP366	0	0	0	0	0	0	0	0
RNA5SP367	0	0	0	0	0	0	0	0
RNA5SP368	0	0	0	0	0	0	0	0
RNA5SP369	0	0	0	0	0	0	0	0
RNA5SP37	0	0	0	0	0	0	0	0
RNA5SP370	0	0	586.605655	81.044685	0	0	0	0
RNA5SP371	0	0	0	0	0	0	0	0
RNA5SP372	0	0	0	0	0	0	0	0
RNA5SP373	0	0	0	0	0	0	0	0
RNA5SP374	0	0	0	0	0	0	0	0
RNA5SP375	0	0	0	0	0	0	0	0
RNA5SP377	0	0	0	0	0	0	0	0
RNA5SP378	0	0	0	0	0	0	0	0
RNA5SP379	0	0	0	0	0	0	0	0
RNA5SP38	0	0	0	0	0	0	0	0
RNA5SP380	0	0	0	0	0	0	0	0
RNA5SP382	0	0	0	0	0	0	0	0
RNA5SP383	0	0	0	0	0	0	0	0
RNA5SP384	0	0	0	0	0	0	0	0
RNA5SP385	0	0	0	0	0	0	0	0
RNA5SP386	0	0	0	0	0	0	0	0
RNA5SP387	0	0	0	0	0	0	0	0
RNA5SP388	0	0	0	0	0	0	0	0
RNA5SP389	0	1725.17824	14307.5201	937.919459	12117.1283	2769.42694	11273.1218	305.647327
RNA5SP39	0	0	0	0	0	0	0	0
RNA5SP390	0	0	0	0	0	0	0	0
RNA5SP391	0	0	0	0	0	0	0	0
RNA5SP392	0	0	0	0	0	0	0	0
RNA5SP393	0	0	0	0	0	0	0	0
RNA5SP394	0	0	0	0	0	0	0	0

RNA5SP395	0	0	0	0	0	0	0	0
RNA5SP396	0	0	0	0	0	0	0	0
RNA5SP397	0	0	0	0	0	0	0	0
RNA5SP399	0	0	0	0	0	0	0	0
RNA5SP40	0	0	0	0	0	0	0	0
RNA5SP400	0	0	0	0	0	0	0	0
RNA5SP401	0	0	0	0	0	0	0	0
RNA5SP402	0	0	0	0	0	9.737983	0	0
RNA5SP403	0	0	0	0	0	0	0	0
RNA5SP404	0	0	0	0	0	0	0	0
RNA5SP405	0	0	0	0	0	0	0	0
RNA5SP406	0	0	0	0	0	0	0	0
RNA5SP407	0	0	0	0	0	0	0	0
RNA5SP408	0	0	0	0	0	0	0	0
RNA5SP409	0	0	0	0	0	0	0	0
RNA5SP41	0	0	0	0	0	0	0	0
RNA5SP410	0	0	0	0	0	0	0	0
RNA5SP411	0	0	0	0	0	0	0	0
RNA5SP412	0	0	0	0	0	0	0	0
RNA5SP413	0	0	0	0	0	0	0	0
RNA5SP414	0	0	0	0	0	0	0	0
RNA5SP415	0	0	0	0	0	0	0	0
RNA5SP416	0	0	0	0	0	0	0	0
RNA5SP417	0	0	0	0	0	0	0	0
RNA5SP418	0	0	0	0	0	0	0	0
RNA5SP419	0	0	0	0	0	0	0	0
RNA5SP42	0	0	0	0	0	0	0	0
RNA5SP420	0	0	0	0	0	0	0	0
RNA5SP421	0	0	0	0	0	0	0	0
RNA5SP422	0	0	0	0	0	0	0	0
RNA5SP423	0	0	0	0	0	0	0	0
RNA5SP425	0	0	0	0	0	0	0	0
RNA5SP426	0	0	0	0	0	0	0	0

RNA5SP427	0	0	0	0	0	0	0	0
RNA5SP428	0	0	0	0	0	0	0	0
RNA5SP429	0	0	0	0	0	0	0	0
RNA5SP43	0	0	0	0	0	0	0	0
RNA5SP430	0	0	0	0	0	0	0	0
RNA5SP431	0	0	0	0	0	0	0	0
RNA5SP432	0	0	0	0	0	0	0	0
RNA5SP433	0	0	0	0	0	0	0	0
RNA5SP434	0	0	0	0	0	0	0	0
RNA5SP435	0	0	0	0	0	0	0	0
RNA5SP436	0	0	0	0	0	0	0	0
RNA5SP437	0	0	0	0	0	0	0	0
RNA5SP438	0	0	0	0	0	0	0	0
RNA5SP439	0	0	0	0	0	0	0	0
RNA5SP44	0	0	0	0	0	0	0	0
RNA5SP440	0	0	0	0	0	0	0	0
RNA5SP441	0	0	0	0	0	0	0	0
RNA5SP442	0	0	0	0	0	0	0	0
RNA5SP444	0	0	0	0	0	0	0	0
RNA5SP445	0	0	0	0	0	0	0	0
RNA5SP446	0	0	0	0	0	0	0	0
RNA5SP447	0	0	0	0	0	0	0	0
RNA5SP448	0	0	0	0	0	0	0	0
RNA5SP449	0	0	0	0	0	0	0	0
RNA5SP45	0	0	0	0	0	0	0	0
RNA5SP450	0	0	0	0	0	0	0	0
RNA5SP451	0	0	0	0	0	0	0	0
RNA5SP452	0	0	0	0	0	0	0	0
RNA5SP453	0	0	0	0	0	0	0	0
RNA5SP454	0	0	0	0	0	0	0	0
RNA5SP455	0	0	0	0	0	0	0	0
RNA5SP456	0	0	0	0	0	0	0	0
RNA5SP457	0	0	0	0	0	0	0	0

RNA5SP458	0	0	0	0	0	0	0	0
RNA5SP459	0	0	0	0	0	0	0	0
RNA5SP46	0	0	0	0	0	0	0	0
RNA5SP460	0	0	0	0	0	0	0	0
RNA5SP461	0	0	0	0	0	0	0	0
RNA5SP462	0	0	0	0	0	0	0	0
RNA5SP464	0	0	0	0	0	0	0	0
RNA5SP465	0	0	0	0	0	0	0	0
RNA5SP466	0	0	0	0	0	0	0	0
RNA5SP467	0	0	0	0	0	0	0	0
RNA5SP468	0	0	0	0	0	0	0	0
RNA5SP469	0	0	0	0	0	0	0	0
RNA5SP47	0	0	0	0	0	0	0	0
RNA5SP470	0	0	0	0	0	0	0	0
RNA5SP471	0	0	0	0	0	0	0	0
RNA5SP472	0	0	0	0	0	0	0	0
RNA5SP473	0	0	0	3.936474	0	0	0	0
RNA5SP474	0	0	0	0	0	0	0	0
RNA5SP475	0	0	0	0	0	0	0	0
RNA5SP476	0	0	0	0	0	0	0	0
RNA5SP477	0	0	0	0	0	0	0	0
RNA5SP478	0	0	0	0	0	0	0	0
RNA5SP479	0	0	0	0	0	0	0	0
RNA5SP48	0	0	0	0	0	0	0	0
RNA5SP480	0	0	0	0	0	0	0	0
RNA5SP481	0	0	0	0	0	0	0	0
RNA5SP482	0	0	0	0	0	0	0	0
RNA5SP483	0	0	0	0	0	0	0	0
RNA5SP484	0	0	0	0	0	0	0	0
RNA5SP485	0	0	0	0	0	0	0	0
RNA5SP486	0	0	0	0	0	0	0	0
RNA5SP487	0	0	0	0	0	0	0	0
RNA5SP488	0	0	0	0	0	0	0	0

RNA5SP489	0	0	0	0	0	0	0	0
RNA5SP49	0	0	0	0	0	0	0	0
RNA5SP490	0	0	0	3.188541	0	0	0	0
RNA5SP491	0	0	0	0	0	0	0	0
RNA5SP492	0	0	0	0	0	0	0	0
RNA5SP493	0	0	0	0	0	0	0	0
RNA5SP494	0	0	0	0	0	0	0	0
RNA5SP495	0	0	0	0	0	0	0	0
RNA5SP496	0	0	0	0	0	0	0	0
RNA5SP497	0	0	0	0	0	0	0	0
RNA5SP498	0	0	0	0	0	0	0	0
RNA5SP499	0	0	0	0	0	0	0	0
RNA5SP50	0	0	0	0	0	0	0	0
RNA5SP500	0	0	0	0	0	0	0	0
RNA5SP501	0	0	0	0	0	0	0	0
RNA5SP502	0	0	0	0	0	0	0	0
RNA5SP503	0	0	0	0	0	0	0	0
RNA5SP504	0	0	0	0	0	0	0	0
RNA5SP505	0	0	0	0	0	0	0	0
RNA5SP507	0	0	0	0	0	0	0	0
RNA5SP508	0	0	0	0	0	0	0	0
RNA5SP509	0	0	0	0	0	0	0	0
RNA5SP51	0	0	0	0	0	0	0	0
RNA5SP510	0	0	0	0	0	0	0	0
RNA5SP511	0	0	0	0	0	0	0	0
RNA5SP512	0	0	0	0	0	0	0	0
RNA5SP513	0	0	0	0	0	0	0	0
RNA5SP514	0	0	0	0	0	0	0	0
RNA5SP515	0	0	0	0	0	0	0	0
RNA5SP516	0	0	0	0	0	0	0	0
RNA5SP517	0	0	0	0	0	0	0	0
RNA5SP518	0	0	0	0	0	0	0	0
RNA5SP519	0	0	0	0	0	0	0	0

RNA5SP52	0	0	0	0	0	0	0	0
RNA5SP520	0	0	0	0	0	0	0	0
RNA5SP522	0	0	0	0	0	0	0	0
RNA5SP53	0	0	0	0	0	0	0	0
RNA5SP54	0	0	0	0	0	0	0	0
RNA5SP55	0	0	0	0	0	0	0	0
RNA5SP56	0	0	0	0	0	0	0	0
RNA5SP57	0	0	0	0	0	0	0	0
RNA5SP60	0	0	0	0	0	0	0	0
RNA5SP61	0	0	0	0	0	0	0	0
RNA5SP62	0	0	0	0	0	0	0	0
RNA5SP63	0	0	0	0	0	0	0	0
RNA5SP64	0	0	0	0	0	0	0	0
RNA5SP65	0	0	0	0	0	0	0	0
RNA5SP66	0	0	0	0	0	0	0	0
RNA5SP67	0	0	0	0	0	0	0	0
RNA5SP68	0	0	0	0	0	0	0	0
RNA5SP69	0	0	0	0	0	0	0	0
RNA5SP70	0	0	0	0	0	0	0	0
RNA5SP72	0	0	0	0	0	0	0	0
RNA5SP73	0	0	0	0	0	0	0	0
RNA5SP74	0	0	0	0	0	0	0	0
RNA5SP75	0	0	0	0	0	0	0	0
RNA5SP76	0	0	0	0	0	0	0	0
RNA5SP77	0	0	0	0	0	0	0	0
RNA5SP78	0	0	0	0	0	0	0	0
RNA5SP79	0	0	0	0	0	0	0	0
RNA5SP80	0	0	0	0	0	0	0	0
RNA5SP82	0	0	0	0	0	0	0	0
RNA5SP84	0	0	0	0	0	0	0	0
RNA5SP86	0	0	0	0	0	0	0	0
RNA5SP87	0	0	0	0	0	0	0	0
RNA5SP88	0	0	0	0	0	0	0	0

RNA5SP89	0	0	0	0	0	0	0	0
RNA5SP90	0	0	0	0	0	0	0	0
RNA5SP91	0	0	0	0	0	0	0	0
RNA5SP92	0	0	0	0	0	0	0	0
RNA5SP93	0	0	0	0	0	0	0	0
RNA5SP94	0	0	0	0	0	0	0	0
RNA5SP95	0	0	0	0	0	0	0	0
RNA5SP96	0	0	0	0	0	0	0	0
RNA5SP97	0	0	0	0	0	0	0	0
RNA5SP98	0	0	0	0	0	0	0	0
RNA5SP99	0	0	0	0	0	0	0	0
RNASE1	0	0	0	0.31567	0	0	0	0
RNASE11	0	0	0	0	0	0	0	0
RNASE12	0	0	0	0	0	0	0	0
RNASE13	0	0	0	0	0	0	0	0
RNASE2	0	0	0	0	0	0	0	0
RNASE4	8.615168	3.204015	7.253878	13.500417	2.124586	2.54557	0	5.150095
RNASE6	0	0	0	0	0	0	0	0
RNASE7	8.024265	10.546941	16.591256	22.63768	23.620248	34.055799	15.460298	28.200239
RNASE8	0	0	0	0	0	0	0	0.870056
RNASE9	0	0.617324	0	0	0	0	0	0
RNASEH1	41.193482	63.289863	33.304948	41.243442	36.275555	42.661085	18.801661	48.462932
RNASEH1P1	0	0	0	0	0	0	0	0
RNASEH1P2	0	0	0	0	0	0	0	0
RNASEH2A	66.306941	44.542146	13.459638	54.33547	34.599647	112.732005	39.513572	66.901005
RNASEH2B	11.793706	31.003929	18.694726	14.77078	7.680477	5.117538	69.586409	31.155849
RNASEH2C	28.606032	159.154629	81.0269	124.071649	71.737976	102.263207	41.342463	72.063646
RNASEH2CP1	0	0	0	0	0	2.244921	0	7.055592
RNASEK	39.157652	90.861495	83.910529	132.04706	115.736857	98.566083	102.399308	85.672762
RNASEK-C17	0	1.539612	0	0.147092	0	0	0.723822	0
RNASEL	0	2.044509	1.395775	2.108202	1.437215	3.537449	3.574738	3.995227
RNASET2	22.481867	54.89979	2.876252	64.246421	28.704165	26.905029	22.436159	40.039511
RND1	0	2.799275	0	1.507474	0	2.09337	0	0.780585

RND2	0	0	0	0.464294	0	0.198045	0	0.22268
RND3	80.196821	182.509706	38.316594	178.38141	116.450693	160.460606	73.182646	175.086681
RNF10	80.87939	237.42165	72.650592	180.33922	342.817134	409.148189	458.904383	398.77714
RNF103	7.644769	24.513627	11.140695	14.145041	4.288966	8.25066	6.506419	13.770203
RNF103-CHN	0	1.670167	0	8.06285	0	4.768665	0	1.337951
RNF10P1	0	0	0	0	0	0	0	0
RNF11	113.945627	181.862609	196.510256	128.114488	27.631963	52.157124	58.816005	89.334425
RNF111	37.593658	105.170054	10.73816	131.542493	64.07741	92.454995	32.364499	108.831398
RNF112	0	0	0	0.206141	0	0.105084	43.191203	0
RNF114	57.528194	55.812469	81.386218	69.182457	56.692244	52.472729	151.246574	49.560811
RNF115	6.338107	11.943912	8.351396	7.123788	9.120694	8.054633	8.538989	12.752704
RNF11P1	0	0	0	0	0	0	0	0
RNF11P2	0	0	0	0	0	0	0	0
RNF121	13.518597	45.996756	29.543395	36.579923	19.355764	24.155381	26.285244	22.038614
RNF122	0	2.602741	3.331075	4.75672	0	3.320111	0	1.582841
RNF123	9.28999	7.332248	38.848921	9.359932	8.791797	10.220018	27.962678	8.485448
RNF125	12.712125	4.779065	2.627953	2.626816	1.390617	1.254851	0	0.516073
RNF126	55.867878	79.474174	40.728473	75.346618	42.385319	148.128837	58.81218	127.628479
RNF126P1	0	0	0	0	0	0	0	0
RNF128	2.551762	6.329923	5.710649	6.854109	0	0	0	0.051357
RNF13	103.875743	58.837612	79.313767	39.971568	143.785413	31.852294	131.408373	55.731639
RNF130	22.406003	22.977674	29.427817	19.452507	19.051124	15.228876	20.706669	28.95855
RNF135	7.324119	29.341267	9.803633	24.090136	9.388572	25.247719	0	25.546272
RNF138	36.445746	43.415052	8.3951	41.447868	54.387474	60.044823	55.219216	60.560594
RNF138P1	0	0	0	0	0	0	0	0
RNF138P2	0	0	0	0	0	0	0	0
RNF139	0	0	0	0	0	0	0	9.23997
RNF13P1	0	0	0	0	0	0	0	0
RNF14	57.689772	68.515637	7.711783	62.273109	12.292251	39.95791	29.852145	91.200269
RNF141	56.24013	53.782864	44.289989	35.98408	56.222333	35.132877	40.684912	65.578006
RNF144A	0.569312	1.003088	6.734609	3.256175	0	2.133473	0	2.284264
RNF144B	0	0	0	11.414287	0	48.293817	19.6781	6.979669
RNF145	33.486921	0.872493	0	8.324835	22.674588	1.708686	64.399967	13.924132

RNF146	20.006332	30.037636	20.432887	22.748324	4.405529	14.250086	4.451423	19.138799
RNF148	0	0.782739	0	0	0	0	0	0
RNF149	7.200695	0.583472	9.250045	3.052509	24.253853	5.496276	29.898272	3.986504
RNF14P1	0	0	0	0	0	0	0	0
RNF14P2	0	0	0	0	0	0	0	0
RNF14P3	0	0	0	0	0	0	0	0
RNF14P4	0	0.124026	0	0	0	0	0	0
RNF150	0	0	0	0.210547	0	0.061839	0	0.812263
RNF151	0	0	0	0	0	0	0	0
RNF152	10.334645	23.630808	7.287894	15.643299	7.784398	34.215312	5.209677	24.192778
RNF152P1	0	0	0	0	0	0	0	0
RNF157	8.382389	2.586012	0	0.411558	0	0	0	0
RNF166	0	0.677919	0	0.711717	4.950151	0.726916	96.318914	0.336241
RNF167	95.947443	76.058961	41.477718	63.279707	84.070947	75.815106	161.518328	65.676583
RNF168	45.574439	85.136145	43.161613	88.070113	19.997268	80.398572	29.4672	74.520334
RNF169	19.755188	17.197696	27.027227	17.770963	18.255454	22.596801	18.471442	11.510905
RNF17	0	0	0	0	0	0	0	0
RNF170	8.717116	32.123086	11.250941	13.505114	6.874637	1.295504	0	8.274494
RNF175	0	0	0	0	0	0	0	0
RNF180	0	2.076336	0.594652	0.990325	0	0.100695	0	1.248326
RNF181	106.859931	12.849902	0	12.977691	0	11.265035	32.010998	2.247945
RNF182	1.019913	4.599617	4.303038	0.552839	0	0.931618	0	0.583572
RNF183	0	0.385839	0	0	0	0	0	0.194107
RNF185	23.230936	24.362769	13.190914	28.924602	16.01233	27.552959	23.559497	27.439098
RNF186	0	0	0	0	0	0	0	0
RNF19A	11.02517	62.929924	39.552797	72.457833	60.043118	69.898058	98.065283	60.942501
RNF19B	11.749267	15.805874	35.052732	15.766927	20.646254	10.878024	25.426597	20.162707
RNF19BPX	0	0	0	0	0	0	0	0
RNF19BPY	0	0	0	0	0	0	0	0
RNF2	15.645284	64.697108	45.661721	58.386013	21.377093	22.778821	27.626066	46.625969
RNF20	21.827829	36.58033	34.655808	41.673483	50.530614	46.080588	33.910878	29.893838
RNF207	2.99818	2.535674	1.683294	3.879096	1.759809	3.252577	0	3.982122
RNF208	0	0	0	0	0	0	0	0

RNF212	16.797835	10.586146	7.413125	6.851949	3.477912	3.976946	0	3.678742
RNF212B	0	0.146845	0	0.229866	0	0.17227	0	0.514373
RNF213	52.988024	43.135822	35.671766	50.135505	39.025004	49.131178	51.81848	43.96485
RNF214	15.383372	9.684291	10.299246	21.297887	12.915607	25.348194	10.663679	25.145489
RNF215	2.940176	10.009851	9.566688	5.237125	0	4.050878	3.63185	3.74802
RNF216	32.519146	36.380378	33.191912	38.217767	9.219572	21.113874	6.693444	16.607708
RNF216P1	20.164817	94.891363	38.466203	104.290299	32.462924	55.902619	61.198822	88.670096
RNF217	12.359348	9.710036	29.094562	11.08979	5.344959	22.911867	8.268891	23.508775
RNF220	77.085373	116.104439	64.695164	135.847094	42.123553	157.837501	41.916258	122.043147
RNF222	0	0	0	0.118011	0	0.06027	0	0
RNF223	1.836364	4.734517	9.831895	8.735895	4.840391	8.439872	0	6.671893
RNF227	3.884247	0	4.635582	3.552556	2.391334	2.901644	8.170324	0
RNF24	7.941174	23.266981	16.350532	21.923292	6.876313	15.104468	13.690436	17.474227
RNF25	25.659442	11.753019	30.665303	12.112434	18.652254	18.067098	23.507336	15.789208
RNF2P1	0	0.31432	0	0	0	0	0	0
RNF31	32.91993	10.041033	13.629431	13.255327	57.261621	22.950268	0	14.010472
RNF32	0	2.696029	0	2.093305	0	0.84129	0	3.799481
RNF34	27.005978	2.854515	34.647351	9.81347	20.470983	14.115436	10.208607	9.822074
RNF38	9.267684	7.966451	15.739245	12.147042	27.637047	14.13562	48.643861	13.223383
RNF39	0	2.935576	1.575078	3.121286	6.511876	6.451806	0	3.581051
RNF4	47.208822	134.081662	43.267313	135.136805	48.497436	154.751152	29.590696	140.06152
RNF40	38.786493	121.226811	77.328794	126.426545	46.595547	189.555378	129.462837	97.69364
RNF41	54.40795	89.525273	25.340512	105.886931	13.611003	73.10141	11.79709	60.496626
RNF43	1.867041	1.927085	0	0.836254	0	18.057446	5.555963	10.674523
RNF44	21.608355	58.030602	31.152473	70.941767	19.233286	66.098837	6.038227	51.083476
RNF5	27.014602	25.885173	38.834205	31.1958	51.478225	29.04408	35.643923	25.558577
RNF5P1	0	0	0	0	0	0	0	0
RNF6	36.448435	92.371251	28.271204	99.934126	40.709182	75.223557	36.845556	92.553238
RNF6P1	0	0	0	0	0	0	0	0
RNF7	115.035672	172.146226	133.534082	114.105209	90.193113	120.243656	66.297259	159.825559
RNF7P1	0	0	0	0	0	0	0	0
RNF8	30.540938	63.73158	14.633146	54.438035	57.120041	47.113314	48.121431	67.94741
RNFT1	6.5042	14.97903	6.917543	13.630368	24.802112	4.142633	47.69424	7.501695

RNFT1P2	0	0	0	0	0	0	0	0
RNFT1P3	0	0	0	0.087194	0	0	0	0
RNFT2	1.998183	2.22764	0	0.879816	0.745233	2.629092	10.144255	2.732588
RNGTT	15.54668	19.505758	14.639198	13.158603	20.726948	11.075396	0.165392	17.902025
RNGTTP1	0	0	0	0	0	0	0	0
RNH1	47.489706	91.522283	45.895658	84.23128	64.91897	176.170783	101.193771	147.79017
RNLS	8.498076	14.396433	6.675466	8.182442	4.85704	7.218592	0	1.914401
RNMT	32.665361	24.997092	22.358217	16.815923	18.984935	10.297152	44.875859	39.96893
RNPC3	25.12155	15.168504	9.086569	7.913037	29.224574	1.7903	10.907044	7.338537
RNPC3P1	0	0	0	0	0	0	0	0
RNPEP	48.37313	34.137409	34.358388	33.058119	48.864332	39.079759	59.545062	32.623484
RNPEPL1	10.712132	18.307966	15.472908	22.4974	9.599395	59.841033	34.904254	43.94432
RNPS1	122.199993	365.272643	141.410095	386.645317	157.059714	336.183703	128.744148	247.205617
RNPS1P1	0	0	0	0.109996	0	0	0	0
RO60	26.036262	47.668594	11.52251	33.05614	74.837645	24.57445	35.13457	21.608012
ROBO1	23.347784	17.958444	5.437602	16.096792	13.465178	1.709097	0	1.493798
ROBO2	0	0	2.245986	0.133112	0	0	0	0
ROBO2P1	0	0	0	0	0	0	0	0
ROBO3	4.280178	8.255814	14.34365	6.419922	2.835096	12.207681	0	7.259291
ROBO4	0	0	0	0	0.646606	0	0	0.131696
ROCK1	3.135519	6.242894	4.350616	7.588202	0.378757	10.936072	42.945822	10.570689
ROCK1P1	0	0	0	0	0	0	0	0
ROCK2	34.56481	51.626462	13.14174	34.889144	52.176528	15.361083	50.110892	54.891247
ROGDI	6.740504	18.005335	0	12.28477	18.826984	18.809197	0	7.823089
ROM1	4.645939	0.925125	0	1.427681	0	3.033421	0	1.990644
ROMO1	464.971702	412.155381	255.927447	405.225031	447.989767	414.03826	221.777295	354.740091
ROPN1	0	0	0	0	0	0	0	0.073742
ROPN1B	0	0	0	0.526461	0	0.281408	0	1.067815
ROPN1L	0	1.929425	0	5.092757	0	0.348313	0	1.148776
ROR1	4.927697	2.690144	0	2.743739	0.783769	6.983433	0	2.213784
ROR2	0.79306	5.703144	4.271232	8.001228	3.141647	8.418714	0	13.810593
RORA	17.954021	7.034101	5.572038	3.277199	0.156562	16.027956	12.04652	7.062947
RORB	0	0.332543	1.004125	0.786884	0	0	0	0

RORC	0	0	0	0	0	0	0	0
ROS1	0	0	0	0.05267	0	0	0	0
RP1	0	1.52137	0.815892	3.599195	0	0.517867	0	0.435125
RP1L1	0	0.106718	0	0.231505	0	0	0	0.047349
RP2	13.445306	10.508971	12.061781	5.731108	16.094047	5.551084	20.931738	10.478956
RP9	19.507027	60.399171	42.515659	65.919101	2.612598	66.168947	3.884876	77.759223
RP9P	3.130712	16.502915	8.741451	13.558862	12.89648	15.454472	30.281574	20.67178
RPA1	22.0916	25.17935	0	32.024215	88.514648	85.252433	22.647229	91.164416
RPA2	51.766967	68.396698	40.074327	60.030784	53.945538	105.751498	36.11876	99.775193
RPA2P1	0	0	0	0	0	0	0	0
RPA2P2	0	0	0	0	0	0	0	0
RPA2P3	0	0	0	0	0	0	0	0
RPA3	506.155476	518.353055	403.147188	294.747391	176.691567	78.579862	0.415587	121.695002
RPA3P1	0	0	0	0	0	0	0	0
RPA3P2	0	0	0	0	0	0	0	0
RPA4	0	0.389826	0	0	0	0	0	0.093904
RPAIN	8.075819	24.747382	34.961162	20.201406	69.734471	40.250644	0.978822	33.269497
RPAP1	17.591302	33.167069	25.682245	42.845947	16.493709	47.600781	29.403688	19.814374
RPAP2	25.399452	21.698884	11.424629	9.210253	16.373654	5.20786	31.213322	21.92765
RPAP2P1	0	0	0	0	0	0	0	0
RPAP3	62.456372	49.056291	50.879912	25.479621	26.078301	16.677668	42.305263	45.241012
RPE	109.986099	70.171578	74.44013	70.861413	94.128024	58.686196	90.120045	74.550114
RPE65	0	0	0	0	0	0	0	0
RPEL1	0	0	0	0	0	0	0	0
RPEP1	0	0	0	0	0	0	0	0
RPEP2	0	0	0	0	0	0	0	0
RPEP3	0	0	0	0	0	0	0	0
RPEP4	0	0	0	0	0	0	0	0
RPEP5	0	0	0	0	0	0	0	0
RPEP6	0	0	0	0	0	0	0	0
RPF1	37.514527	41.780609	54.213697	46.403774	25.429381	28.174965	39.346192	28.958566
RPF2	45.602354	46.294061	15.300643	31.805888	46.822572	36.689977	46.654121	60.582527
RPF2P1	0	0	0	0	0	0	0	0

RPF2P2	0	0	0	0	0	0	0	0
RPGR	5.307631	5.617552	3.562391	3.882298	8.661059	5.010907	0	13.10699
RPGRIP1	0	1.419114	0	0.117522	0	0.113283	0	0.209577
RPGRIP1L	25.040387	20.644515	32.896403	21.046389	12.172614	7.63732	5.191002	14.131619
RPH3A	0	0	0	0	0	0	0	0
RPH3AL	6.005062	43.436298	5.818202	17.2726	12.00075	9.4219	4.472356	7.572564
RPIA	25.147383	28.579266	39.679296	21.075311	20.391655	12.796138	13.566512	9.531845
RPIAP1	0	0	0	0	0	0	0	0
RPL10	2767.89545	1359.73961	3079.85946	1272.42793	2026.96441	1227.71574	2091.92135	1360.87153
RPL10A	1382.21072	701.757476	1532.99611	678.536105	1193.40418	538.096389	1001.11466	581.115922
RPL10AP1	0	0	0	0	0	0	0	0
RPL10AP12	0	0	0	0	0	0	0	0
RPL10AP2	0	0	0	0	0	0	0	0
RPL10AP3	0	0	0	0	0	0	0	0
RPL10AP5	0	0	0	0	0	0	0	0
RPL10AP6	0	0	0	0	1.798515	0	0	0.568967
RPL10P1	0	0	0	0	0	0	0	0
RPL10P10	0	0	0	0	0	0	0	0
RPL10P11	0	0	0	0	0	0	0	0
RPL10P12	0	0	0	0	0	0	0	0
RPL10P13	0	0	0	10.131911	0	1.001384	0	0
RPL10P14	0	0	0	0	0	0	0	0
RPL10P15	0	0	0	0	0	0	0	0
RPL10P16	0	0.32008	0	0.181596	0	1.186911	0	0.314214
RPL10P17	0	0	0	0	0	0	0	0
RPL10P18	0	0	0	0	0	0	0	0
RPL10P19	0	0	0	0	0	0	0	0
RPL10P2	0	0	0	0	0	0	0	0
RPL10P3	0	0.440092	0	0.269682	0	0	0	0
RPL10P4	0	0	0	0	0	0	0	0
RPL10P5	0	0	0	0	0	0	0	0
RPL10P6	0	0.761549	0	0	0	0	0	0
RPL10P7	0	0	0	0.16812	0	0	0	0

RPL10P8	0	0	0	0	0	0	0	0
RPL10P9	0	0.295267	0	0	1.85567	0	0	0.29596
RPL11	1773.55932	1073.12183	2032.82737	1346.93512	2025.1311	1194.20563	1889.67395	1452.17086
RPL11P1	0	0	0	0	0	0	0	0
RPL11P3	0	0	7.938681	0.215894	0	0	0	0
RPL11P4	0	0	0	0	0	0	0	0
RPL11P5	0	0	0	0	0	0	0	0
RPL12	3570.6844	822.357161	3983.56414	968.167504	2125.68288	550.791874	1563.61671	523.989116
RPL12P1	0	0	0	0	0	0	0	0
RPL12P10	0	0	8.52754	0.231999	0	0	0	0.420473
RPL12P11	0	0	0	0	0	0	0	0
RPL12P12	0	0	0	0	0	0.451116	0	0.416768
RPL12P13	0	1.534119	0	0.232692	0	0	0	0
RPL12P14	0	0	0	0.238193	0	0	0	0.431937
RPL12P15	0	0	0	0	0	0	0	0
RPL12P16	0	0	0	1.098071	0	0	0	0
RPL12P17	0	0	8.579969	0	0	0.456041	0	0
RPL12P18	0	0	0	0	0	0	0	0
RPL12P19	0	0	0	0	0	0	0	0
RPL12P2	0	0	0	0.268929	0	0	0	0
RPL12P20	0	0	0	0	0	0	0	0
RPL12P21	0	0	0	0.218935	0	0	0	0
RPL12P22	0	0	0	0	0	0	0	0
RPL12P23	0	0	0	0	0	0	0	0
RPL12P24	0	0	0	0	0	0	0	0
RPL12P25	0	0	0	0	0	0	0	1.278163
RPL12P26	0	0	0	0	0	0	0	0
RPL12P27	0	0	0	0	0	0	0	0
RPL12P28	0	1.176935	0	0	0	0	0	0
RPL12P29	0	0	0	0	0	0	0	0
RPL12P3	0	0	0	0	0	0	0	0
RPL12P30	0	0	0	0	0	0	0	0
RPL12P31	0	0	0	0	0	0	0	0

RPL12P32	0	0	0	0	0	0	0	0
RPL12P33	0	0	8.692399	0	0	0	0	0
RPL12P34	0	0	0	0	0	0	0	0
RPL12P35	0	0	0	0	0	0	0	0
RPL12P36	0	0	0	0	0	0	0	0
RPL12P37	0	0	0	0	0	0.916025	0	0
RPL12P38	0	0.402855	0	0	0	0	0	0
RPL12P39	0	0	0	0	0	0	0	0
RPL12P4	0	0	0	0.25869	0	0	0	0
RPL12P40	0	0	0	0	0	0	0	0
RPL12P41	0	0	0	0	0	0	0	0
RPL12P42	0	0	0	0.463651	0	0	0	0
RPL12P43	0	0	0	0	0	0	0	0
RPL12P44	0	0	0	0	0	0	0	0
RPL12P45	0	0	0	0	0	0	0	0
RPL12P46	0	0	0	0	0	0	0	0
RPL12P47	0	0	0	0	0	0	0	0
RPL12P48	0	0	0	0	0	0	0	0
RPL12P49	0	0	0	0	0	0	0	0
RPL12P5	0	0	0	0	0	0	0	0
RPL12P50	0	0	0	0	0	0	0	0
RPL12P6	0	0	0	0	0	0	0	0
RPL12P7	0	0	0	0.469597	0	0	0	0
RPL12P8	0	0.311472	0	0	0	0	0	0
RPL12P9	0	0	0	0	0	0	0	0
RPL13	3819.45353	2277.21536	4562.08892	2411.21965	2212.92713	2151.65897	1511.21353	2116.44452
RPL13A	1878.46791	735.771146	2213.78094	796.379107	1734.21118	1149.1065	1859.59342	809.861518
RPL13AP	0	0	0	0	0	0	0	0
RPL13AP10	0	0	0	0	0	0	0	0
RPL13AP11	0	0	0	0	0	0	0	0
RPL13AP12	0	0	0	0.182374	0	0	0	0.323377
RPL13AP13	0	0	0	0	0	0	0	0
RPL13AP14	0	0	0	0	0	0.350189	0	0

RPL13AP15	0	0	0	0	0	0	0	0
RPL13AP16	0	0	0	0	0	0	0	0
RPL13AP17	0	0	0	0	0	0	0	0
RPL13AP18	0	0	0	0	0	0	0	0
RPL13AP19	0	0	0	0	0	0	0	0
RPL13AP2	0	0	0	0	0	0	0	0
RPL13AP20	0	0	0	2.908829	10.031624	6.910792	0	5.6587
RPL13AP21	0	0	0	0	0	0	0	0
RPL13AP22	0	0	0	0	0	0	0	0
RPL13AP23	0	0	0	0	0	0	0	0.651753
RPL13AP24	0	1.170214	0	0.345998	0	0.342506	0	0
RPL13AP25	0	0	0	0	0	0	0	0
RPL13AP26	0	3.480586	0	1.154124	17.258722	4.797045	0	4.283984
RPL13AP27	0	0	0	0	0	0	0	0
RPL13AP3	0	0	0	0	0	0	0	0
RPL13AP5	0	0	0	1.631951	0	0	0	1.414097
RPL13AP6	0	0	0	0	0	0	0	0
RPL13AP7	0	0	0	0.803916	0	0.383773	1.876079	0
RPL13AP8	0	0	0	0	0	0	0	0.306173
RPL13AP9	0	0	0	0	0	0	0	1.089459
RPL13P	0	0	0	0	0	0	0	0
RPL13P12	123.857514	165.36425	220.425484	120.684066	191.171271	147.568752	34.787239	156.197915
RPL13P14	0	0	0	0	0	0	0	0
RPL13P2	0	0	0	0	0	0	0	0
RPL13P4	0	0	0	0	0	0	0	0
RPL13P5	7.437562	0	0	6.081098	0	0	0	5.751715
RPL13P6	0	0	0	0	0	0	0	0
RPL13P8	9.652173	1.113243	0	1.797072	0	2.641773	40.333329	1.623409
RPL14	783.478003	1009.855	910.685966	998.379338	478.589969	633.189856	444.577173	693.694471
RPL14P1	0	1.196195	0	5.709649	0	7.027117	0	1.991458
RPL14P2	0	0	0	0	0	0	0	0
RPL14P3	0	0	0	0	0	1.00894	0	0
RPL14P5	0	0	0	0	0	0	0	0

RPL14P6	0	0	0	0	0	0	0	0
RPL15	2397.74301	1246.72179	3317.11599	1246.28068	1731.88319	834.172464	1438.70698	1008.71804
RPL15P1	0	0	0	0	0	0	0	0
RPL15P11	0	0	0	0	0	0	0	0
RPL15P12	0	0	0	0	0	0	0	0
RPL15P13	0	0	0	0	0	0	0	0
RPL15P14	0	0	0	0	0	0	0	0
RPL15P15	0	0	0	0	0	0	0	0
RPL15P16	0	0	0	0	0	0	0	0
RPL15P17	0	0	0	0	0	0	0	0
RPL15P18	0	0	0	0	0	0	0	0
RPL15P19	0	0	0	0	0	0	0	0
RPL15P2	0	0	0	0	0	0	0	0
RPL15P20	0	0	0	0	0	0	0	0
RPL15P21	0	0	0	0	0	0	0	0
RPL15P22	0	0.31346	0	0.187344	0	0	0	0
RPL15P3	0	0	0	0	0	0.530324	0	0.870587
RPL15P4	0	0	0	0	0	0	0	0
RPL15P5	0	0	0	0	0	0	0	0
RPL15P6	0	0	0	0	0	0	0	0
RPL15P7	14.722416	0	0	0	0	0	0	0
RPL15P8	0	0	0	0	0	0	0	0
RPL15P9	0	0	0	0	0	0	0	0
RPL17	223.54786	424.310728	238.952187	505.313189	456.803404	882.343074	105.93402	661.983872
RPL17-C18or	18.586317	14.490424	0	1.41856	0	0.767682	11.46654	0
RPL17P1	0	0	0	0	0	0	0	0
RPL17P10	0	0	0	0	22.051273	0	0	0
RPL17P11	0	0	0	0	0	0	0	0
RPL17P12	0	0	0	0	0	0	0	0
RPL17P13	0	0	0	0	0	0	0	0
RPL17P14	0	0	0	0	0	0	0	0
RPL17P15	0	0	0	0	0	0	0	0
RPL17P16	0	0	0	0	0	0	0	0

RPL17P17	0	0	0	0	0	0	0	0
RPL17P18	0	0	0	0	0	0	0	0
RPL17P19	0	0	0	0	0	0	0	0
RPL17P2	0	0	0	0	0	0	0	0
RPL17P20	0	0	0	0	0	0	0	0
RPL17P21	0	0	0	0	0	0	0	0
RPL17P22	0	0	0	0	0	0	0	0
RPL17P23	0	0	0	0	0	0	0	0
RPL17P24	0	0	0	0	0	0	0	0
RPL17P25	0	0	0	0	0	0	0	0
RPL17P26	0	0	0	0	0	0	0	0
RPL17P27	0	0	0	0	0	0	0	0
RPL17P28	0	0	0	0	0	1.972347	0	2.506221
RPL17P29	0	0	0	0	0	0	0	0
RPL17P3	0	0	0	0	0	0	0	0
RPL17P30	0	0	0	0	0	0	0	0
RPL17P31	0	0	0	0	0	0	0	0
RPL17P33	0	0	0	0	0	0	0	0
RPL17P34	0	0.991167	0	0	0	0	0	0
RPL17P35	0	0	0	0	0	0	0	0
RPL17P36	0	0	0	0	0	0	0	0
RPL17P37	0	0	0	0	0	0	0	0
RPL17P38	0	0	0	0	0	0	0	0
RPL17P39	0	0.842258	0	0.239732	0	1.011276	0	0.388391
RPL17P4	0	0	0	0	0	0	0	0
RPL17P40	0	0	0	0	0	0	0	0
RPL17P41	0	0	0	0	0	0	0	0
RPL17P43	0	0	0	0	0	0	0	0
RPL17P44	0	0	0	0	0	0	0	0
RPL17P45	0	0	0	0	0	0	0	0
RPL17P46	0	0	0	0	0	0	0	0
RPL17P47	0	0	15.877363	3.459522	0	2.936277	0	1.54659
RPL17P48	0	0	0	0	0	0	0	0

RPL17P49	0	0	0	0	0	0	0	0
RPL17P5	0	0	0	0	0	0	0	0
RPL17P50	0	0	0	0.613859	0	0.804464	0	0
RPL17P51	0	0	0	0	0	0	0	0
RPL17P52	0	0	0	0	0	0	0	0
RPL17P6	0	0	0	0.282773	0	0	0	0.434509
RPL17P7	9.329101	0.837212	0	1.238767	0	3.887097	0	0.387608
RPL17P8	0	0	7.346398	0	0	0	0	0
RPL17P9	0	0	0	0	0	0	0	0.374888
RPL18	1055.87571	459.873631	1219.03017	574.983253	1068.77211	655.550938	1283.0622	585.741224
RPL18A	1347.21618	587.978796	1852.58941	801.811576	1504.35283	924.955892	1210.92476	1033.50703
RPL18AP13	0	0	0	0.633583	0	0.415042	0	0
RPL18AP14	0	0	0	0	0	0	0	0.77014
RPL18AP15	0	0	0	0	0	0	0	0
RPL18AP16	0	0	0	0	0	0	0	0
RPL18AP17	0	0	0	0	0	0	0	0
RPL18AP2	0	0	0	0	0	0	0	0
RPL18AP3	0	0	0	0.365759	0	0.723567	0	0
RPL18AP7	0	0	0	0	0	0	0	0
RPL18AP8	0	0	0	0	0	0	0	0
RPL18AP9	0	0	0	0	0	0	0	0
RPL18P1	0	0	0	0	0	0	0	0
RPL18P10	0	0	0	0	0	0	0	0
RPL18P11	0	0	0	0.21177	0	0	0	0
RPL18P13	0	0	0	0	0	0	0	0
RPL19	3119.10853	3051.76903	3273.39932	3556.71357	2174.58568	3062.0971	1989.5715	2250.03956
RPL19P1	0	0	0	0	0	0	0	0
RPL19P11	0	0	0	0	0	0	0	0
RPL19P13	0	0	0	0	0	0	0	0
RPL19P14	0	0	0	0	0	0	0	0
RPL19P16	0	0	0	0	0	0	0	0
RPL19P18	0	0	0	0	0	0	0	0
RPL19P2	0	0	0	0	0	0	0	0

RPL19P20	0	0	0	0	0	0	0	0
RPL19P21	0	0	0	0	0	0	0	0
RPL19P4	0	0	0	0	0	0	0	0
RPL19P5	0	0	0	0	0	0	2.314029	0
RPL19P6	0	0	0	0.18813	0	0	0	0
RPL19P7	0	0	0	0	0	0	0	0
RPL21	70.554064	84.516142	0	21.336679	0	24.887101	65.674481	17.051546
RPL21P1	0	0	0	0	0	0	0	0
RPL21P10	0	0	0	0	0	0	0	0
RPL21P100	0	0	0	0	0	0.437872	0	0
RPL21P103	0	0	0	0	0	0	0	0
RPL21P104	0	0	0	0	0	0	0	0
RPL21P106	0	0	0	0	0	0	0	0
RPL21P107	0	0	0	0	0	0	0	0
RPL21P108	0	0	0	0	0	0	0	0
RPL21P109	0	0	0	0	0	0	0	0
RPL21P11	0	0	0	0	0	0	0	0
RPL21P110	0	0	0	0	0	0	0	0
RPL21P111	0	0	0	0	0	0	0	0
RPL21P112	0	0	0	0	0	0	0	0
RPL21P113	0	0	0	0	0	0	0	0
RPL21P114	0	0	0	0	0	0	0	0
RPL21P115	0	0	0	0	0	0	0	0
RPL21P116	0	0	0	0	0	0	0	0
RPL21P117	0	0	0	0	0	0	0	0
RPL21P118	0	0	0	0	0	0.977787	0	0
RPL21P119	0	0	0	0	0	0	0	0
RPL21P12	0	0	0	0	0	0	0	0
RPL21P120	0	0	0	0	0	0	0	0
RPL21P121	0	0	0	0	0	0	0	0
RPL21P122	0	1.5874	0	0	0	0	0	0
RPL21P123	0	0	0	0	0	0	0	0
RPL21P125	0	0	0	0	0	0	0	0

RPL21P126	0	0	0	0	0	0	0	0
RPL21P127	0	0	0	0	0	0	0	0
RPL21P128	0	0	0	0	0	0	0	0
RPL21P129	0	0	0	0	0	0	0	0
RPL21P13	0	0	0	0	0	0	0	0
RPL21P130	0	0	0	0	0	0	0	0
RPL21P131	0	0	0	0	0	0	0	0
RPL21P132	0	0	0	0	0	0	0	0
RPL21P133	0	0	0	0	0	0	0	0
RPL21P134	0	0	0	0	0	0	0	0
RPL21P135	0	0	0	0	0	0	0	0
RPL21P136	0	0	0	0	0	0	0	0
RPL21P137	0	0	0	0	0	0	0	0
RPL21P14	0	0	0	0	0	0	0	0.906024
RPL21P15	0	0	0	0	0	0	0	0
RPL21P16	435.142218	135.428134	564.385431	0	0	0	306.058661	128.849249
RPL21P17	0	0	0	0	0	0	0	0
RPL21P18	0	0	0	0	0	0	0	0
RPL21P19	0	0	0	0.248362	0	0	0	0
RPL21P2	0	0	0	0	0	0	0	0
RPL21P20	0	0	0	0	0	0	0	0
RPL21P21	0	0	0	0	0	0	0	0
RPL21P22	0	0	0	0	0	0	0	0
RPL21P23	0	0	0	0	0	0	0	0
RPL21P24	0	0	0	0	0	0	0	0
RPL21P25	0	0	0	0	0	0	0	0
RPL21P26	0	0	0	0	0	0	0	0
RPL21P27	0	0	0	0	0	0	0	0
RPL21P28	0	0	0	0	0	0	0	0
RPL21P29	0	0	0	0	0	0	0	0
RPL21P3	0	0	0	0	0	0	0	0
RPL21P30	0	0	0	0	0	0	0	0
RPL21P31	0	0	0	0	0	0	0	0

RPL21P32	0	0	0	0	0	0	0	0
RPL21P33	0	0	0	0	0	0	0	0
RPL21P34	0	0	0	0	0	0	0	0
RPL21P35	0	0	0	0	0	0	0	0.47632
RPL21P36	0	0	0	0	0	0	0	0
RPL21P37	0	0	0	0	0	0	0	0
RPL21P38	0	0	0	0	0	0	0	0
RPL21P39	0	0	0	0	0	0	0	0.467722
RPL21P4	0	0	0	0.245916	0	0	0	0
RPL21P40	0	0	0	0	0	0	0	0
RPL21P41	0	0	0	0	0	0	0	0
RPL21P42	0	0	0	0	0	0	0	0
RPL21P43	0	0	0	0	0	0	0	0
RPL21P44	0	0	0	0	0	0	0	0
RPL21P45	0	0	0	0	0	0	0	0
RPL21P46	0	0	9.085653	0	0	0	0	0
RPL21P47	0	0	0	0	0	0	0	0
RPL21P48	0	0	0	0	0	0	0	0
RPL21P49	0	0	0	0	0	0	0	0
RPL21P5	0	0	0	0	0	0	0	0
RPL21P50	0	0	0	0	0	0	0	0
RPL21P51	0	0	0	0	0	0	0	0
RPL21P52	0	0	0	0	0	0	0	0
RPL21P53	0	0	0	0	0	0	0	0
RPL21P54	0	0	0	0	0	0	0	0
RPL21P55	0	0	0	0	0	0	0	0
RPL21P56	0	0	0	0	0	0	0	0
RPL21P57	0	0	0	0	0	0	0	0
RPL21P58	0	0	0	0	0	0	0	0
RPL21P59	0	0	0	0	0	0	0	0
RPL21P6	0	0	0	0	0	0	0	0
RPL21P60	10.718801	0	0	0	0	0	0	0
RPL21P61	0	0	0	0	0	0	0	0

RPL21P62	0	0	0	0	0	0	0	0
RPL21P63	0	0	0	0	0	0	0	0
RPL21P64	0	0	0	0	0	0	0	0
RPL21P65	0	0	0	0	0	0	0	0
RPL21P66	0	0	0	0	0	0	0	0
RPL21P67	0	0	0	0	0	0	0	0
RPL21P68	0	0	0	0	0	0	0	0
RPL21P69	0	0	0	0	0	0	0	0
RPL21P7	0	0	0	0	0	0	0	0
RPL21P70	0	0	0	0	0	0	0	0
RPL21P71	0	0	0	0	0	0	0	0
RPL21P72	0	0	0	0	0	0	0	0
RPL21P73	0	0	0	0	0	0	0	0
RPL21P74	0	0	0	0	0	0	0	0
RPL21P75	0	0	0	0	0	0	0	0
RPL21P76	0	0	0	0	0	0	0	0
RPL21P77	0	0	0	0	0	0	0	0
RPL21P78	0	0	0	0	0	0	0	0
RPL21P79	0	0	0	0	0	0	0	0
RPL21P8	0	0	0	0	0	0	0	0
RPL21P80	0	0	0	0	0	0	0	0
RPL21P81	0	0	0	0	0	0	0	0.906024
RPL21P82	0	0	0	0	0	0	0	0
RPL21P83	0	0	0	0	0	0	0	0
RPL21P84	0	0	0	0	0	0	0	0
RPL21P85	0	0	0	0	0	0	0	0
RPL21P86	0	0	0	0	0	0	0	0
RPL21P87	0	0	0	0	0	0	0	0
RPL21P88	0	0	0	0	0	0	0	0
RPL21P89	0	0	0	0	0	0	0	0
RPL21P9	0	0	0	0	0	0	0	0
RPL21P90	0	0	0	0	0	0	0	0
RPL21P91	0	0	0	0	0	0	0	0

RPL21P92	0	0	0	0	0	0	0	0
RPL21P93	0	0	0	0	0	0	0	0
RPL21P94	0	0	0	0	0	0	0	0
RPL21P95	0	0	0	0.249853	0	0	0	0
RPL21P96	0	0	0	0	0	0	0	0
RPL21P97	0	0	0	0.248047	0	0	0	0
RPL21P98	0	0	0	0	0	0	0	0.462989
RPL21P99	0	0	0	0	0	0	0	0
RPL22	604.284831	421.713937	609.595188	459.128349	791.668311	519.319862	965.6437	710.66
RPL22L1	489.23302	166.208093	472.23244	110.997624	66.778445	12.380051	54.247178	27.618384
RPL22P1	0	108.678512	188.562024	65.152876	0	172.911048	232.984054	0
RPL22P10	0	0	0	0	0	0	0	0
RPL22P11	0	0	0	0	0	0	0	0
RPL22P12	0	0	0	0	0	0	0	0
RPL22P13	0	0	0	0	0	0	0	0
RPL22P14	0	0	0	0	0	0	0	0
RPL22P16	0	0	0	0	0	0.703061	0	0
RPL22P17	0	0	0	0	0	0	0	0
RPL22P18	0	0	0	0	0	0	0	0
RPL22P19	0	0	0	0	0	0	0	0
RPL22P2	0	0	0	0	0	0	0	0
RPL22P20	0	0	0	0	0	0	0	0
RPL22P21	0	0	0	0	0	0	0	0
RPL22P22	0	0	0	0	0	0	0	0
RPL22P23	0	0	0	0	0	0	0	0
RPL22P24	0	0	0	0	0	0	0	0
RPL22P3	0	0	0	0	0	0	0	0
RPL22P4	0	0	0	0	0	0	0	0
RPL22P5	0	0	0	0	0	0	0	0
RPL22P6	0	0	0	0.343601	0	0	0	0
RPL22P7	0	0	0	0	0	0	0	0
RPL22P8	0	0	0	0	0	0	0	0
RPL23	3844.38583	2329.76652	5301.6457	2510.08182	2324.38399	1091.34916	2019.44844	1532.36716

RPL23A	1464.37176	1176.40282	1999.36193	1077.97189	1182.1347	827.57264	1143.52264	846.908168
RPL23AP1	0	0.827102	0	0.756821	2.820969	4.427132	0	4.149742
RPL23AP10	0	0	0	0	0	0	0	0
RPL23AP11	0	0	0	0	0	0	0	0
RPL23AP12	0	0	0	0	0	0	0	0
RPL23AP14	0	0	0	0	0	0	0	0
RPL23AP15	0	0	0	0	0	0	0	0
RPL23AP16	0	0	0	0	0	0	0	0
RPL23AP17	0	0	0	0	0	0	0	0
RPL23AP18	0	0	0	0	0	0	0	0
RPL23AP19	0	0	0	0	0	0	0	0
RPL23AP2	0	0	0	1.230156	0	0	0	0
RPL23AP20	0	0	0	0	0	0	0	0
RPL23AP22	0	0	0	0	0	0	0	0
RPL23AP23	0	0	0	0	0	0	0	0
RPL23AP24	0	0	0	0	0	0	0	0
RPL23AP25	0	0	0	2.437552	0	0	0	0
RPL23AP26	0	0	0	0	0	0	0	0
RPL23AP27	0	0	0	0	0	0	0	0
RPL23AP28	0	0	0	0	0	0	0	0
RPL23AP29	0	0	0	0	0	0	0	0
RPL23AP3	0	0	0	0	0	0	0	0
RPL23AP30	0	0	0	0	0	0	0	0
RPL23AP31	0	0	0	0	0	0	0	0
RPL23AP32	0	0	0	0	0	0	0	0
RPL23AP33	0	0	0	0	0	0	0	0
RPL23AP34	0	0	0	0	0	0	0	0
RPL23AP35	0	0	0	0	0	0	0	0
RPL23AP36	0	0	0	0	0	0	0	0
RPL23AP37	0	0	0	0	0	0	0	0
RPL23AP38	0	0	0	0	0	0	0	0
RPL23AP39	0	0	0	0	0	0	0	0
RPL23AP4	0	0	0	0	0	0	0	0

RPL23AP40	0	0	0	0	0	0	0	0
RPL23AP41	0	0	0	0	0	0	0	0
RPL23AP42	1728.33163	986.359168	1937.80343	1411.07338	1043.95112	1336.63368	1238.97679	903.628927
RPL23AP43	0	0	0	0	0	0	0	0
RPL23AP44	0	0	0	0	0	0	0	0
RPL23AP45	0	0	0	0	0	0	0	0
RPL23AP46	0	0	0	0	0	0	0	0
RPL23AP47	0	8.822056	0	5.877722	0	0	0	0
RPL23AP48	0	0	0	0	0	0	0	0
RPL23AP49	0	5.42512	0	0	0	2.32766	2.539838	0
RPL23AP50	0	0	0	0	0	0	0	0
RPL23AP52	0	0	0	0	0	0	0	0
RPL23AP53	0	1.607137	0	0	0	0	0	0
RPL23AP54	0	0	0	0	0	0	0	0
RPL23AP55	0	0	0	0	0	0	0	0
RPL23AP56	0	0	0	0	0	0	0	0
RPL23AP57	0	0	0	0	0	0	0	0
RPL23AP58	0	0	0	0	0	0	0	0
RPL23AP59	0	0	0	0	0	0	0	0
RPL23AP6	0	0	0	0	0	0	0	0
RPL23AP60	0	0	0	0	0	4.100419	0	9.075807
RPL23AP61	0	0	0	0	0	0	0	0
RPL23AP62	0	0	0	0	0	0	0	0
RPL23AP63	0	0	0	0	0	0	0	0
RPL23AP64	0	0	0	0	0	0	0	0
RPL23AP65	0	0	0	0	0	0	0	0.472934
RPL23AP66	0	0	0	0	0	0	0	0
RPL23AP67	0	1.639434	0	0	0	0	0	0
RPL23AP68	0	0	0	0	0	0	0	0
RPL23AP69	0	0	0	0	0	0	0	0
RPL23AP7	0	0	0	0	45.285383	25.926249	0	0
RPL23AP70	0	0	0	0	0	0	0	0
RPL23AP71	0	0	0	0	0	0	0	0

RPL23AP72	0	0	0	0	0	0	0	0
RPL23AP73	0	0	0	0	0	0	0	0
RPL23AP74	0	0	0	0.764221	0	0.994853	2.515424	0
RPL23AP75	0	0.43996	0	0	0	0	0	0
RPL23AP76	0	0	0	0	0	0	0	0
RPL23AP77	0	0	0	0	0	0	0	0
RPL23AP78	0	0	0	0	0	0	0	0
RPL23AP8	0	0	0	2.241506	0	0	0	0
RPL23AP80	0	0	0	0	0	0	0	0
RPL23AP81	0	0	0	0	0	0	0	0
RPL23AP82	0	0	47.879287	29.095711	0	29.523284	0	22.887236
RPL23AP83	0	0	0	0	0	0	0	0
RPL23AP84	0	0	0	0	0	0	0	0
RPL23AP85	0	0	0	0	0	0	0	0
RPL23AP86	0	0	0	0	0	0	0	0
RPL23AP87	0	0	0	0	0	0	0	0
RPL23AP88	0	0	0	0	0	0	0	0
RPL23AP89	0	0	0	0	0	0	0	0
RPL23AP90	0	0	0	0	0	0	0	0
RPL23AP91	0	0	0	0	0	0	0	0
RPL23AP92	0	0	0	0	0	0	0	0
RPL23AP93	0	0	0	0	0	0	0	0
RPL23AP94	0	0	0	0	0	0	0	0
RPL23AP95	0	0	0	0	0	0	0	1.371256
RPL23AP96	0	0	0	0	0	0	0	0
RPL23AP97	0	0	0	1.532657	0	0	0	0
RPL23P10	0	0	0	0	0	0	0	0
RPL23P11	0	0	0	0	0	0	0	0
RPL23P2	0	39.874692	0	14.128147	13.482364	32.552508	0	51.472234
RPL23P4	0	0	0	0	0	0	0	0
RPL23P5	0	0	0	0	0	0	0	0
RPL23P6	0	0.490901	0	0	0	0	0	0
RPL23P8	0	0	0	0	0	0	0	0

RPL24	2618.09357	2054.13432	2903.05995	2090.87511	2326.71189	1435.51382	1849.12479	1539.99432
RPL24P2	0	5.31275	18.302042	2.241976	0	2.916501	0	1.818574
RPL24P4	0	1.03343	0	0	0	0	0	0
RPL24P6	0	0	0	0.249853	0	0	0	0
RPL24P7	0	0	0	0	0	0	0	0
RPL24P8	0	0	0	0.24997	0	0	0	0
RPL24P9	0	0	0	0	0	0	0	0
RPL26	2564.02833	1429.45589	2603.60952	1576.55556	2257.05947	1070.39006	1589.66559	1173.8067
RPL26L1	122.920106	95.325816	61.279238	73.330422	64.309086	60.066678	96.077035	78.585097
RPL26P13	0	0	0	0	0	0	0	0
RPL26P15	0	0	0	0	0	0	0	0
RPL26P19	0	0	0	0	0	0	0	0
RPL26P20	0	0	0	0	0	0	0	0
RPL26P26	0	0	0	0	0	0	0	0
RPL26P27	0	0	0	0	0	0	0	0
RPL26P28	0	0	0	0	0	0	0	0
RPL26P29	0	0	0	0	0	0	0	0
RPL26P3	0	0	0	0	0	0	0	0
RPL26P30	0	0	0	0	0	0	0	0
RPL26P31	0	0	0	0	0	0	0	0
RPL26P34	0	0	0	0	0	0	0	0
RPL26P35	0	0	0	0	0	0	0	0
RPL26P36	0	0	0	0	0	0	0	0
RPL26P37	0	0	0	0	0	0	0	0
RPL26P6	0	0	0	0	0	0	0	0
RPL26P9	0	0	0	0	0	0	0	0
RPL27	21.828558	76.241963	36.138736	38.764528	90.106777	80.246744	72.019518	129.957581
RPL27A	1845.64026	865.637071	2231.06483	1059.8328	2907.96015	1162.97802	2505.61181	1223.25542
RPL27AP	0	0	0	0	0	0	0	0
RPL27AP3	0	0	0	0	0	0	0	0
RPL27AP4	0	0	0	0	0	0	0	0
RPL27AP5	0	0	0	0	0	0	0	0
RPL27AP7	0	0	0	0	0	0	0	0

RPL27AP8	0	0	0	0	0	0	0	0
RPL27AP9	0	0	0	0	0	0	0	0
RPL27P12	0	0	0	0	0	0	0	0
RPL27P5	0	0	0	0	0	0	0	0
RPL27P6	0	0	0	0	0	0	0	0
RPL27P7	0	0	0	0	0	0	0	0
RPL27P8	0	0	0	0	0	0	0	0
RPL27P9	0	0	0	0	0	0	0	0
RPL28	1160.9577	730.047959	1483.36334	888.75066	1484.60456	1219.24542	724.746544	1241.82091
RPL28P1	0	0	0	0	0	0	0	0
RPL28P2	0	0	0	0	0	0	0	0
RPL28P5	0	0	0	0	0	0	0	0
RPL28P6	0	0	0	0	0	0	0	0
RPL29	18.345927	22.563636	8.230058	14.71618	3.633001	15.585839	1.094242	28.744543
RPL29P1	0	0	0	0	0	0	0	0
RPL29P11	0	0	0	0.525181	0	0	0	0.472799
RPL29P12	0	0	0	0	0	0	0	0.469442
RPL29P13	0	0	0	0	0	0	0	0
RPL29P14	0	1.898628	0	0	29.541742	1.133784	0	1.086948
RPL29P15	0	0	0	0	0	0	0	0
RPL29P16	0	0	0	0	0	0	0	0
RPL29P17	0	0	0	0	0	0	0	0
RPL29P18	0	0	0	0	0	0	0	0
RPL29P19	11.317721	29.509959	19.212053	29.557457	0	23.93127	0	34.548805
RPL29P2	0	0	0	0	0	0	0	0
RPL29P20	0	2.648214	0	5.953506	0	5.785297	0	7.970811
RPL29P21	0	0	0	0	0	0	0	0
RPL29P22	0	0	0	0	0	0	0	0
RPL29P23	0	0	0	0	0	0	0	0
RPL29P24	0	0	0	0	0	0	0	0
RPL29P25	0	0	0	0	0	0	0	0
RPL29P26	0	0	0	0.271175	0	0	0	0
RPL29P27	0	0	0	0	0	0	0	0

RPL29P28	0	0	0	0	0	0	0	0
RPL29P29	0	0	0	0	0	0	0	0
RPL29P3	0	0	0	0.258966	0	0	0	0
RPL29P30	0	0	0	0	0	0	0	0
RPL29P31	0	0	0	0	0	0	0	0
RPL29P32	0	0	0	0	0	0	0	0
RPL29P33	0	0	0	0	0	0	0	0
RPL29P34	0	0	0	0	0	0	0	0
RPL29P35	0	0	0	0	0	0	0	0
RPL29P4	0	4.921732	0	0	0	0	0	0.732696
RPL29P5	0	0	0	0	0	0	0	0
RPL29P6	0	0	0	0	0	0	0	0
RPL29P7	0	0	0	0	0	0	0	0
RPL29P8	0	0	0	0	0	0	0	0
RPL29P9	0	0	0	0	0	0	0	0
RPL3	1795.21341	1652.59541	2032.88389	1800.25034	1149.41352	1571.15926	879.905749	1113.71043
RPL30	2601.89398	1739.03705	2521.85115	1405.79685	1229.13247	910.108847	983.462239	1728.32484
RPL30P1	0	0	0	0	0	0	0	0
RPL30P10	0	0	0	0	0	0	0	0
RPL30P11	0	0	0	0	0	0	0	0
RPL30P12	0	0	0	0	0	0	0	0
RPL30P13	0	0	0	0	0	0	0	0
RPL30P14	0	0	0	0	0	0	0	0
RPL30P15	0	0	0	0	0	0	0	0
RPL30P16	0	0	0	0	0	0	0	0
RPL30P2	0	0	0	0	0	0	0	0
RPL30P3	0	0	0	0	0	0	0	0
RPL30P4	0	0	0	0	0	0	0	0
RPL30P5	0	0	0	0	0	0	0	0
RPL30P6	0	0	0	0	0	0	0	0
RPL30P7	0	0	0	0	0	0	0	0
RPL30P9	0	0	0	0	0	0	0	0
RPL31	2232.41974	1428.17955	2703.9988	1447.49462	1996.42835	1698.77565	1609.11152	1413.11064

RPL31P1	0	0	0	0	0	0	0	0
RPL31P10	0	0	0	0	0	0	0	0
RPL31P11	0	0	0	0	0	0	0	0
RPL31P12	0	0	0	0	0	0	0	0
RPL31P13	0	0	0	0	0	0	0	0
RPL31P14	0	0	0	0	0	0	0	0
RPL31P15	0	0	0	0	0	0	0	0
RPL31P16	0	0	0	0	0	0	0	0
RPL31P17	0	0	0	0	0	0	0	0
RPL31P18	0	0	0	0	0	0	0	0
RPL31P19	0	0	0	0	0	0	0	0
RPL31P2	0	0	0	0	0	0	0	0
RPL31P20	0	0	0	0	0	0	0	0
RPL31P21	0	0	0	0	0	0	0	0
RPL31P22	0	0	0	0	0	0	0	0
RPL31P23	0	0	0	0	0	0	0	0
RPL31P24	0	0	0	0	0	0	0	0
RPL31P25	0	0	0	0	0	0	0	0
RPL31P26	0	0	0	0	0	0	0	0
RPL31P28	0	0	0	0	0	0	0	0
RPL31P29	0	0	0	0	0	0	0	0
RPL31P3	0	0	0	0	0	0	0	0
RPL31P30	0	0	0	1.553769	0	0	0	0
RPL31P31	0	0	0	0	0	0	0	0
RPL31P32	0	0	0	0	0	0	0	0
RPL31P33	0	0	0	0	0	0	0	0
RPL31P34	0	0	0	0	0	0	0	1.024242
RPL31P35	0	0	0	0	0	0	0	0
RPL31P36	0	0	0	0	0	0	0	0
RPL31P37	0	0	0	0	0	0	0	0
RPL31P38	0	0	0	0	0	0	0	0
RPL31P39	0	0	0	0	0	0	0	0
RPL31P4	0	0	0	0	0	0	0	0

RPL31P40	0	0	0	0	0	0	0	0
RPL31P41	0	0	0	0	0	0	0	0
RPL31P42	0	0	0	0	0	0	0	0
RPL31P43	0	0	0	0	0	0	0	0
RPL31P44	0	0	0	0	0	0	0	0
RPL31P45	0	0	0	0	0	0	0	0
RPL31P46	0	0	0	0	0	0	0	2.031732
RPL31P47	0	0	0	0	0	0	0	0
RPL31P48	0	0	0	0	0	0	0	0
RPL31P49	0	0	13.310488	0.727767	0	0	0	0
RPL31P5	0	0	0	0	0	0	0	0
RPL31P50	0	0	0	0	0	0	0	0
RPL31P51	0	0	0	0.382925	0	0	0	0
RPL31P52	0	0	0	0	0	0	0	0
RPL31P53	0	0	0	0	0	0	0	0
RPL31P54	0	0	0	0	0	0	0	0
RPL31P56	0	0	0	0	0	0	0	0
RPL31P57	0	0	0	0	0	0	0	0
RPL31P58	0	0	0	0	0	0	0	0
RPL31P59	0	0	0	0	0	0	0	0
RPL31P6	0	0	0	0	0	0	0	0
RPL31P60	0	0	0	0	0	0	0	0
RPL31P61	0	0	0	0	0	0	0	0
RPL31P62	0	0	0	0	0	0	0	0
RPL31P63	0	0	0	0	0	0	0	0.691792
RPL31P64	0	0	0	0	0	0	0	0
RPL31P7	0	0	0	0	0	0	0	0
RPL31P8	0	0	0	0	0	0	0	0
RPL31P9	0	0	0	0	0	0	0	0
RPL32	2805.9011	1232.94017	3480.055	1486.80332	1532.67687	820.555098	968.977192	701.239661
RPL32P1	0	0	0	0	0	0	0	0
RPL32P10	0	0	0	0	0	0	0	0
RPL32P11	0	0	0	0	0	0	0	0

RPL32P12	0	0	0	0	0	0	0	0
RPL32P13	0	0	0	0	0	0	0	0
RPL32P14	0	0	0	0	0	0	0	0
RPL32P15	0	0	0	0	0	0	0	0
RPL32P16	0	0	0	1.491511	0	0	0	0
RPL32P17	0	0	0	0	0	0	0	0
RPL32P18	0	0	0	0.428887	0	0	0	0
RPL32P19	0	0	0	0	0	0	0	0
RPL32P2	0	0	0	0	0	0	0	0
RPL32P20	0	0	0	1.264832	0	3.660405	0	5.325503
RPL32P21	0	0	0	0	0	0	0	0
RPL32P22	0	0	0	1.290604	0	0	0	0
RPL32P23	0	0	0	0	0	0	0	0
RPL32P24	0	37.625513	0	30.10178	0	13.233262	0	16.904524
RPL32P25	0	0	0	0	0	0	0	0
RPL32P26	0	0	0	0	0	0	0	0
RPL32P27	0	0	0	0.740758	0	0	0	1.873724
RPL32P28	0	0	0	0	0	0	0	0
RPL32P29	12.698722	3.829991	9.529568	6.188869	0	4.079272	0	0
RPL32P3	57.563617	0	0	0	0	0	0	4.112853
RPL32P30	0	0	0	0	0	0	0	0
RPL32P31	0	0	0	0	0	0	0	0
RPL32P32	17.207107	0	0	1.951549	0	5.214352	0	1.491773
RPL32P33	0	0	0	0	0	0	0	0
RPL32P34	0	0	0	0	0	0	0	0
RPL32P35	0	0	0	0	0	0	0	0
RPL32P36	0	0	0	0	0	0	0	0
RPL32P37	0	0	0	0	0	0	0	0
RPL32P4	0	0	0	0	0	0	0	0
RPL32P5	0	0	0	0	0	0	0	0
RPL32P6	0	1.592306	0	0.330721	0	0	0	0
RPL32P7	0	0	0	0	0	0	0	0
RPL32P8	0	0	0	0	0	0	0	0

RPL32P9	0	0	0	0	0	0	0	0
RPL34	40.80176	32.953685	14.47468	39.134416	15.954488	35.067764	112.188194	25.555936
RPL34P1	0	0.633451	0	0	0	0	0	0
RPL34P10	0	0	0	0	0	0	0	0
RPL34P11	0	0	0	0	0	0	0	0
RPL34P14	0	0	0	0	0	0	0	0
RPL34P16	0	0	0	0	0	0	0	0
RPL34P17	0	0	0	0	0	0	0	0
RPL34P18	0	0	0	0.40394	0	0	0	0
RPL34P19	0	0	0	0	0	0	0	0
RPL34P20	0	0	0	0	0	0	0	0
RPL34P21	0	0	0	0	0	0	0	0
RPL34P22	0	0	0	0	0	0	0	0
RPL34P23	0	0	0	0	0	0	0	0
RPL34P24	0	0	0	0	0	0	0	4.563686
RPL34P26	0	0	0	0	0	0	0	0
RPL34P27	0	0	0	0.824179	0	0	0	0
RPL34P29	0	0	0	0	0	0	0	0
RPL34P3	0	0	0	0	0	0	0	0
RPL34P31	0	0	0	0	0	0	0	0
RPL34P33	0	0	0	0	0	0	0	0
RPL34P34	0	0	0	0	0	0	0	0
RPL34P35	0	0	0	0	0	0	0	0
RPL34P36	0	0	0	0	0	0	0	0
RPL34P6	0	0	0	0	0	0	0	0.80911
RPL34P8	0	0	0	0	0	0	0	0
RPL34P9	0	0	0	0	0	0	0	0
RPL35	2839.98182	2234.16012	3315.73706	2585.06733	2162.87685	2323.77568	1681.75707	2266.70571
RPL35A	2871.89942	1184.56854	2939.64671	1135.12155	2441.53632	788.813351	2402.0474	1099.97571
RPL35AP	0	0	0	0	0	0	0	0
RPL35AP10	0	0	0	0	0	0	0	0
RPL35AP11	0	0	0	0	0	0	0	0
RPL35AP12	0	0	0	0	0	0	0	0

RPL35AP13	0	0	0	0	0	0	0	0
RPL35AP14	0	0	0	0	0	0	0	0
RPL35AP15	0	0	0	0	0	0	0	0
RPL35AP16	0	0	0	0	0	0	0	0
RPL35AP17	0	0	0	0	0	0	0	0
RPL35AP18	0	0	0	0	0	0	0	0
RPL35AP19	0	0	0	0	0	0	0	0
RPL35AP2	0	1.438032	0	0	0	0	0	0
RPL35AP20	0	0	0	1.358682	0	0	0	0
RPL35AP21	0	0	0	0	0	0	0	0
RPL35AP22	0	0	0	0	0	0	0	0
RPL35AP23	0	0	0	0	0	0	0	0
RPL35AP24	0	0	0	0	0	0	0	0
RPL35AP26	0	0	0	0	0	0	0	0
RPL35AP27	0	0	0	0	0	0	0	0
RPL35AP28	0	0	0	0	0	0	0	0
RPL35AP29	0	0	0	0	0	0	0	0
RPL35AP3	0	0	0	0	0	0	0	0
RPL35AP30	0	0	0	0	0	0	0	0
RPL35AP31	0	0	0	0	0	0	0	0
RPL35AP32	0	0	0	0	0	0	0	0
RPL35AP33	0	0	0	0	0	0	0	0
RPL35AP34	0	0	0	0	0	0	0	0
RPL35AP35	0	0	0	0	0	0	0	0
RPL35AP36	0	0	0	0	0	0	0	0
RPL35AP37	0	0	0	0	0	0	0	0
RPL35AP38	0	0	0	0	0	0	0	0
RPL35AP4	0	0	0	0	0	0	0	0
RPL35AP5	0	0	0	0	0	0	0	0
RPL35AP6	0	0	0	0	0	0	0	0
RPL35AP7	0	0	0	0	0	0	0	0
RPL35AP8	0	0	0	0	0	0	0	0
RPL35AP9	0	0	0	0	0	0	0	0

RPL35P1	0	1.83078	0	0.375402	0	0	0	1.430588
RPL35P10	0	0	0	0	0	0.738154	0	0
RPL35P2	0	0	0	0	0	0	0	0
RPL35P3	0	0	0	0	0	0	0	0
RPL35P4	0	0	0	0	0	0	0	0
RPL35P5	0	0	0	0	0	0	0	0
RPL35P6	0	0	0	0	0	0	0	2.103819
RPL35P8	0	0	0	0	0	0	0	0
RPL35P9	0	0	0	0	0	0	0	0
RPL36	883.457903	725.000883	1077.18211	994.740518	983.679806	797.38843	716.341307	735.294009
RPL36A	0	10.548848	7.998984	103.016932	35.735244	22.881507	562.547736	223.649047
RPL36A-HNR	0	40.370756	21.517262	49.907773	0	18.198923	0	0
RPL36AP1	0	0	0	0	0	0	0	0
RPL36AP10	0	0	17.300859	0	0	0	0	0
RPL36AP11	0	0	0	0	0	0	0	0
RPL36AP12	0	0	0	0	0	0	0	0
RPL36AP13	0	0	0	0	0	0	0	0
RPL36AP14	0	0	0	0	0	0	0	0
RPL36AP15	0	0	0	0	0	0	0	0
RPL36AP16	0	0	0	0	0	0	0	0
RPL36AP17	0	0	0	0	0	0	0	0
RPL36AP18	0	0	0	0	0	0	0	0
RPL36AP19	0	0	0	0	0	0	0	0
RPL36AP2	0	0	0	0	0	0	0	0
RPL36AP20	0	0	0	0	0	0	0	0
RPL36AP21	0	0	0	0	0	0	0	0
RPL36AP23	0	0	0	0	0	0	0	0
RPL36AP24	0	0	0	0	0	0	0	0
RPL36AP25	0	0	0	0	0	0	0	0
RPL36AP26	0	1.511922	0	0	0	0	0	0
RPL36AP27	0	0	0	0	0	0	0	0
RPL36AP28	0	0	0	0	0	0	0	0
RPL36AP29	0	0	0	0	0	0	0	0

RPL36AP30	0	0	0	0	0	0	0	0
RPL36AP31	0	0	0	0	0	0	0	0
RPL36AP32	0	0	0	0	0	0	0	0
RPL36AP33	0	0	0	0	0	0	0	0
RPL36AP34	0	0	0	0	0	0	0	0
RPL36AP35	0	0	0	0	0	0	0	0
RPL36AP36	0	0	0	0	0	0	0	0
RPL36AP37	2028.2849	1089.40684	2297.10478	1777.3268	1122.16365	880.922115	0	679.497561
RPL36AP38	0	0	0	0	0	0	0	0
RPL36AP39	0	0	0	0	0	0	0	0
RPL36AP4	0	0	0	0	0	0	0	0
RPL36AP40	0	0	0	0	0	0	0	0
RPL36AP41	0	0	0	0	0	0	0	0
RPL36AP43	0	0.797482	0	0	0	0	0	0
RPL36AP44	0	0	0	0	0	0	0	0
RPL36AP45	0	0	0	0	0	0	0	0
RPL36AP46	0	0	0	0	0	0	0	0
RPL36AP47	0	0	0	0	0	0	0	0
RPL36AP48	0	0	0	0	0	0	0	0
RPL36AP49	0	0	0	0	0	0	0	0
RPL36AP5	0	0	0	0	0	0	0	0
RPL36AP50	0	0	0	0	0	0	0	0
RPL36AP51	0	0	0	0	0	0	0	0
RPL36AP52	0	0	0	0	0	0	0	0
RPL36AP53	0	0	0	0	0	0	0	0
RPL36AP54	0	0	0	0	0	0	0	0
RPL36AP55	0	0	0	0	0	0	0	0
RPL36AP6	0	0	0	0	0	0	0	0
RPL36AP7	0	0	0	0	0	0	0	0
RPL36AP8	0	0	0	3.056868	0	0	0	3.009551
RPL36AP9	0	0	0	0	0	0	0	0
RPL36P1	0	0	0	0	0	0	0	0
RPL36P10	0	0	0	0	0	0	0	0

RPL36P11	0	0	0	0	0	0	0	0
RPL36P12	0	0	0	0	0	0	0	0
RPL36P13	0	0	0	0	0	0	0	0
RPL36P14	0	0	0	0	0	0	0	0
RPL36P15	0	0	0	0	0	0	0	0
RPL36P16	0	0	0	0	0	0	0	0
RPL36P17	0	0	0	0	0	0	0	0
RPL36P18	0	0	0	0	0	0	0	0
RPL36P19	0	0	0	0	0	0	0	0
RPL36P2	0	0	0	0	0	0	0	0
RPL36P20	0	0	0	0	0	0	0	0
RPL36P3	0	0	0	0	0	0	0	0
RPL36P4	0	0	0	0	0	0	0	0
RPL36P5	0	0	0	0	0	0	0	0
RPL36P6	0	0	0	0	0	0	0	0
RPL36P7	0	1.797119	0	0.595595	0	0	0	3.521006
RPL36P8	0	0	0	0	0	0	0	0
RPL36P9	0	0	0	0	0	0	0	0
RPL37	2984.97293	1133.17132	2869.18314	1356.41281	1943.58198	909.068144	1718.56247	930.541503
RPL37A	3549.45411	1531.53825	4165.55377	1786.43397	3065.1355	1411.10497	2159.46212	1511.04054
RPL37AP1	0	0	0	0	0	0	0	0
RPL37AP8	0	0	0	0	0	0	0	0
RPL37P1	0	0	0	0	0	0	0	0
RPL37P10	0	0	0	0	0	0	0	0
RPL37P11	0	0	0	0	0	0	0	0
RPL37P12	0	0	0	0	0	0	0	0
RPL37P13	0	0	0	0	0	0	0	0
RPL37P15	0	0	0	0	0	0	0	0
RPL37P18	0	0	0	0	0	0	0	0
RPL37P2	0	0	0	0	0	0	0	0
RPL37P21	0	0	0	0	0	0	0	0
RPL37P23	0	0	0	0	0	0	0	0
RPL37P24	0	0	0	0	0	0	0	0

RPL37P25	0	0	0	0	0	0	0	0
RPL37P3	0	0	0	0	0	0	0	0
RPL37P4	0	0	0	0	0	0	0	0
RPL37P6	0	0	0	0	0	0	0	0
RPL38	3311.8766	2874.80984	3353.59669	2729.5177	2050.1259	2487.99737	2097.3197	2825.59221
RPL38P1	0	0	0	0	0	0	0	0
RPL38P2	0	0	0	0	0	0	0	0
RPL38P3	0	0	0	0	0	0	0	0
RPL38P4	0	0	0	0	0	0	0	0
RPL38P5	0	0	0	0	0	0	0	0
RPL38P6	0	0	0	0	0	0	0	0
RPL39	6179.33031	1735.72528	6549.93835	1340.5962	4032.70475	578.883864	3864.93342	1470.83284
RPL39L	11.556916	49.715419	35.303228	30.168246	3.023499	53.089868	42.692075	68.088711
RPL39P	0	0	0	0	0	0	0	0
RPL39P14	0	0	0	0	0	0	0	0
RPL39P15	0	0	0	0	0	0	0	0
RPL39P16	0	0	0	11.904231	0	0	520.404281	16.259338
RPL39P17	0	0	0	0	0	0	0	0
RPL39P18	0	0	0	0	0	0	0	0
RPL39P25	0	0	0	0	0	0	0	0
RPL39P26	0	0	0	0	0	0	0	0
RPL39P28	0	0	0	0	0	0	0	0
RPL39P29	0	0	0	0	0	0	0	0
RPL39P3	0	0	0	0	0	0	0	0
RPL39P31	0	0	0	0	0	0	0	0
RPL39P33	0	0	0	0	0	0	0	0
RPL39P34	0	0	0	0	0	0	0	0
RPL39P36	0	0	0	0	0	0	0	19.60336
RPL39P38	0	0	0	0	0	0	0	0
RPL39P39	0	0	0	0	0	0	0	0
RPL39P40	0	0	0	0	0	0	0	0
RPL39P41	0	0	0	0	0	0	0	0
RPL39P5	0	0	0	0	0	0	0	0

RPL39P6	0	0	0	0	0	0	0	0
RPL3L	0	1.158097	1.435826	3.768286	0	0.159234	0	0
RPL3P1	0	0	0	0	0	0	0	0
RPL3P10	0	0	0	0	0	0	0	0
RPL3P11	0	0	0	0	0	0	0	0
RPL3P12	0	0	0	0	0	0	0	0
RPL3P13	0	0	0	0	0	0	0	0
RPL3P2	2.779048	16.33336	34.190317	13.884364	0.813764	14.211495	0	4.589299
RPL3P3	0	0	0	0	0	0	0	0
RPL3P4	0	0	0	0.594077	0	0	0	0
RPL3P5	0	0	0	0	0	0	0	0
RPL3P6	0	0	0	0	0	0	0	0
RPL3P7	0	0.255164	0	0	0	0	0	0
RPL3P8	0	0	0	0.075378	0	0	0	0
RPL3P9	0	0.393368	0	0	0	0	0	0.128205
RPL4	3831.2928	3878.28874	4560.38678	4295.07281	2227.01836	3086.01512	1690.44253	2337.7484
RPL41	5643.86995	2834.24948	7015.04934	3398.00193	2358.45357	1574.41921	2037.35696	1851.94328
RPL41P1	0	0	0	0	0	0	0	0
RPL41P2	28.956447	1.091794	0	0.985399	9.871195	0	0	0
RPL4P1	0	0	0	0.069485	0	0	0	0
RPL4P2	0	0	0	0	0	0	0	0
RPL4P3	0	0	0	0.067488	0	0.410149	0	0.113462
RPL4P4	0	1.392942	0	0.759354	0.783721	1.08881	0	0.121577
RPL4P5	0	0	0	0.28718	0	0.29025	0	0.240352
RPL4P6	0	0.743365	0	2.10485	0	1.375443	0	3.742836
RPL4P7	0	0	0	0	0	0	0	0
RPL5	6410.9413	7896.10756	7148.81511	8291.05216	1465.79582	2663.07242	1526.44661	2668.16086
RPL5P1	0	0	0	0	0	0	0	0
RPL5P10	0	0	0	0	0	0	0	0
RPL5P11	0	0	0	0	0	0	0	0
RPL5P12	0	0	0	0.112743	0	0	0	0
RPL5P13	0	0	0	0	0	0	0	0
RPL5P14	0	0	0	0	0	0	0	0

RPL5P15	0	0	0	0	0	0	0	0
RPL5P16	0	0	0	0	0	0	0	0
RPL5P17	0	0	0	0.325878	0	0	0	0.186119
RPL5P18	0	0	0	0.213062	0	0	0	0
RPL5P19	0	0	0	0	0	0	0	0
RPL5P2	0	0	0	0	0	0	0	0
RPL5P20	0	0	0	0	0	0	0	0
RPL5P21	0	0	0	0	0	0	0	0
RPL5P22	0	0	0	0	0	0	0	0
RPL5P23	0	0.37581	0	0.220043	0	0	0	0
RPL5P24	0	0	0	0	0	0	0	0
RPL5P25	0	0	0	0	0	0	0	0
RPL5P26	0	0	0	0	0	0	0	0
RPL5P27	0	0	0	0	0	0	0	0
RPL5P28	0	0	0	0.146479	0	0	0	0
RPL5P29	0	0	0	0.106093	0	0	0	0
RPL5P3	0	0	0	0	0	0	0	0
RPL5P30	4.455618	0.910554	7.835409	2.450217	0	0	0	0
RPL5P31	0	0	0	0	0	0	0	0
RPL5P32	0	0	0	0	0	0	0	0
RPL5P33	0	0	0	0	0	0	0	0
RPL5P34	0	0.184987	0	0.323377	0	0	0	0.920434
RPL5P35	0	0	0	0	0	0	0	0
RPL5P4	0	0	0	0.218499	0	0	0	0
RPL5P5	0	0	0	0	0	0	0	0
RPL5P6	0	0	0	0	0	0	0	0
RPL5P7	0	0	0	0	0	0	0	0
RPL5P8	0	0	0	0	0	0	0	0
RPL5P9	0	0	0	0	0	0	0	0.183661
RPL6	401.195413	359.013834	250.019519	489.594026	365.785273	1618.5067	396.916807	721.197045
RPL6P1	0	0	0	0	1.655641	0	0	0.261785
RPL6P10	9.955102	6.681285	8.810466	11.381343	0	18.394462	0	7.075731
RPL6P11	0	0	0	0	0	0	0	0

RPL6P12	0	0	0	0	0	0	0	0
RPL6P13	0	0	0	0	0	0	0	0
RPL6P14	0	0	0	0	0	0	0	0
RPL6P15	0	0	0	0	0	0	0	0
RPL6P16	0	0	0	0	0	0	0	0
RPL6P17	0	0	0	0	0	0.441065	0	0
RPL6P18	0	0	0	0	0	0	0	0
RPL6P19	0	0.618771	0	0.114491	1.246141	0.235097	0	0.195515
RPL6P2	0	0	0	0	0	0	0	0
RPL6P20	0	0.188937	0	0	0	0	0	0
RPL6P21	0	0	0	0	0	0	0	0
RPL6P22	0	0	0	0	0	0	0	0
RPL6P23	0	0	0	0	0	0	0	0
RPL6P24	0	0	0	0	0	0	0	0
RPL6P25	0	0	0	0	0	0	0	0
RPL6P26	0	0	0	0	0	0	0	0
RPL6P27	0	1.82347	0	0	0	1.190164	0	0.556734
RPL6P28	0	0	0	0.229501	0	0	0	0
RPL6P29	0	0	0	0	0	0	0	0
RPL6P3	0	0	0	0	0	0	0	0
RPL6P30	0	0	0	0	0	0	0	0
RPL6P31	0	0	0	0	0	0	0	0
RPL6P32	0	0	0	0	0	0	0	0
RPL6P4	0	0	0	0	0	0	0	0
RPL6P5	0	0	0	0	0	0	0	0
RPL6P6	0	0	0	0	0	0	0	0
RPL6P7	0	0	0	0	0	0	0	0
RPL6P8	0	0	0	0	0	0	0	0
RPL6P9	0	0	0	0	0	0	0	0
RPL7	2685.74875	1849.42615	3080.19286	1620.91334	1057.80066	804.452085	1022.50578	1023.10251
RPL7A	2934.16066	2375.00526	3439.44378	2857.88674	1991.55571	1980.09945	1755.33467	1431.14961
RPL7AP10	0	0	0	0	0	0	0	0
RPL7AP11	0	0	0	0.123119	0	0.247417	0	0.211453

RPL7AP12	0	0	0	0	0	0	0	0
RPL7AP13	0	0	0	0	0	0	0	0
RPL7AP14	0	0	0	0	0	0	0	0.209246
RPL7AP15	0	0	0	0.245527	0	0	0	0
RPL7AP16	0	0.844478	0	0.99314	0	0.99375	0	1.28532
RPL7AP17	0	0	0	0	0	0	0	0
RPL7AP18	0	0	0	0	0	0	0	0
RPL7AP19	0	0	0	0	0	0	0	0.30107
RPL7AP2	0	0	0	0	0	0	0	0
RPL7AP20	0	0.63441	0	0	0	0.247504	0	0
RPL7AP21	0	0	0	0	0	0	0	0
RPL7AP22	0	0	0	0.243593	0	0	0	0.419885
RPL7AP23	0	0	0	0	0	0	0	0
RPL7AP24	0	0	0	0	0	0	0	0
RPL7AP25	0	0	0	0	0	0	0	0
RPL7AP26	0	0	0	0	0	0	0	0
RPL7AP27	0	0.268958	0	0	0	0	0	0
RPL7AP28	0	0	0	0	0	0	0	0
RPL7AP29	0	0	0	0	0	0.249216	0	0
RPL7AP3	0	0	0	0	0	0	0	0
RPL7AP30	0	0	0	0.250435	0	0.764672	0	0
RPL7AP31	0	1.056127	0	0.24589	0	0	0	0
RPL7AP32	0	0	0	0	0	0.247026	0	0
RPL7AP33	0	0	0	0	0	0	0	0
RPL7AP34	0	0	0	0	0	0	0	0
RPL7AP35	0	0	0	0	0	0	0	0
RPL7AP36	0	0	0	0	0	0	0	0
RPL7AP37	0	0	0	0	0	0	0	0
RPL7AP38	0	0	0	0.121224	0	0	0	0.208917
RPL7AP39	0	1.388973	0	1.088723	0	0.557349	0	0.106071
RPL7AP4	0	0	0	0	0	0	0	0
RPL7AP40	0	0	0	0	0	0	0	0
RPL7AP41	0	0	0	0	0	0	0	0

RPL7AP42	0	0	0	0	0	0	0	0
RPL7AP43	0	0.295496	0	0.142578	0	0	0	0.106071
RPL7AP44	0	0	0	0	0	0	0	0
RPL7AP45	0	1.146757	0	15.21869	0	14.250704	0	7.46473
RPL7AP46	0	0	0	0	0	0	0	0
RPL7AP47	0	0	0	0	0	0	0	0
RPL7AP48	0	0	0	0	0	0	0	0
RPL7AP49	0	0	0	0	0	0	0	0
RPL7AP5	0	0	0	0	0	0	0	0
RPL7AP50	0	0	0	0	0	0	0	0
RPL7AP51	0	0	0	0	0	0	0	0
RPL7AP52	0	0	0	0	0	0	0	0
RPL7AP53	0	0	0	0	0	0	0	0
RPL7AP54	0	0	0	0	0	0	0	0
RPL7AP55	0	0	0	0	0	0	0	0
RPL7AP56	0	0	0	0	0	0	0	0
RPL7AP57	0	0	0	0	0	0	0	0
RPL7AP58	0	0	0	0	0	0	0	0
RPL7AP6	0	0	0	0.834135	0	0	0	0
RPL7AP60	0	0	0	0.491835	0	0	0	1.272354
RPL7AP61	0	0	0	0	0	0	0	0
RPL7AP62	0	0	0	0.493916	0	0	0	0
RPL7AP63	0	0	0	0	0	0	0	0
RPL7AP64	0	0	0	0	0	0	0	0
RPL7AP65	0	0	0	0	0	0	0	0
RPL7AP66	0	0	0	0	0	0	1.244453	0.433359
RPL7AP67	0	0	0	0	0	0	0	0
RPL7AP69	0	0	0	0	0	0	0	0
RPL7AP7	0	0	0	0	0	0	0	0
RPL7AP70	0	2.075654	0	0.852122	0	0.243663	0	0
RPL7AP71	0	0	0	0	0	0	0	0
RPL7AP72	0	0	0	0	0	0	0	0
RPL7AP73	0	0	0	0	0	0	0	0

RPL7AP74	0	0	0	0	0	0	0	0
RPL7AP75	0	0	0	0	0	0	0	0
RPL7AP76	0	4.822869	0	1.339487	0	0.516029	0	1.877765
RPL7AP77	0	0	0	0	0	0	0	0
RPL7AP78	0	0	0	0	0	0	0	0
RPL7AP79	0	0	0	0	0	0	0	0
RPL7AP8	0	0	0	0	0	0	0	0
RPL7AP80	0	0	0	0	0	0	0	0
RPL7AP81	0	0	0	0	0	0	0	0
RPL7AP82	0	0	0	0	0	0	0	0
RPL7AP83	0	0	0	0	0	0	0	0
RPL7AP9	0	0	0	0	0	0.243867	0	0.209942
RPL7L1	38.613803	36.870632	39.72601	75.102102	68.681395	53.429849	0	74.372152
RPL7L1P1	0	0	0	0	0	0	0	0
RPL7L1P10	0	0	0	0	0	0	0	0
RPL7L1P11	0	0	0	0	0	0	0	0
RPL7L1P12	0	0	0	0.137128	0	0	0	0
RPL7L1P13	0	0	0	0	0	0	0	0
RPL7L1P14	0	0	0	0	0	0	0	0
RPL7L1P15	0	0	0	0	0	0	0	0
RPL7L1P16	0	0	0	0	0	0	0	0
RPL7L1P17	0	0	0	0	0	0	0	0
RPL7L1P18	0	0	0	0	0	0	0	0
RPL7L1P19	0	0	0	0	0	0	0	0
RPL7L1P2	0	0	0	0	0	0	0	0
RPL7L1P20	0	0	0	0	0	0	0	0
RPL7L1P21	0	0	0	0	0	0	0	0
RPL7L1P22	0	0	0	0	0	0	0	0
RPL7L1P3	0	0	0	0	32.59632	0	0	0
RPL7L1P4	0	0	0	0	0	0	0	0
RPL7L1P5	0	0	0	0	0	0	0	0
RPL7L1P7	0	0	0	0	0	0	0	0
RPL7L1P8	0	0	0	0	0	0	0	0

RPL7L1P9	0	0	0	0	0	0	0	0
RPL7P1	8.308898	0.363371	0	1.174832	0	0	0	0
RPL7P10	0	0	0	0	0	0	0	0
RPL7P11	0	0	0	0	0	0	0	0
RPL7P12	0	0	0	0	0	0	0	0
RPL7P13	0	0	0	0	0	0	0	0
RPL7P14	0	0	0	0	0	0	0	0
RPL7P15	0	0	0	0	0	0	0	0
RPL7P16	0	0	0	0	0	0	0	0
RPL7P17	0	0	0	0	0	0	0	0
RPL7P18	0	0	0	0	0	0	0	0
RPL7P19	0	0	0	0.515011	0	0	0	0
RPL7P2	0	0	0	0	0	0	0	0
RPL7P20	0	0	0	0	0	0	0	0
RPL7P21	0	0	0	0	0	0	0	0
RPL7P22	0	0	0	0	0	0	0	0
RPL7P23	0	0.245087	0	0.28645	0	0.556959	0	0
RPL7P24	0	0	0	0	0	0.26324	0	0
RPL7P25	0	0	0	0	0	0	0	0
RPL7P26	0	0	0	0	0	0	0	0
RPL7P27	0	0	0	0	0	0	0	0
RPL7P28	0	0	0	0	0	0	0	0
RPL7P29	0	0	0	0	0	0	0	0
RPL7P3	0	0	0	0	0	0	0	0
RPL7P30	0	0	0	0	0	0	0	0
RPL7P31	0	0	0	0	0	0	0	0
RPL7P32	0	0.234141	0	0.140704	1.523628	0	0	0
RPL7P33	0	0	0	0.26808	0	0	0	0
RPL7P34	0	0	0	0	0	0	0	0
RPL7P35	0	0	0	0	0	0	0	0
RPL7P36	0	0	0	0	0	0	0	0
RPL7P37	0	0	0	0	0	0	0	0
RPL7P38	0	0	0	0.13474	0	0	0	0

RPL7P39	0	0	0	0	0	0	0	0
RPL7P4	0	0	0	0	0	0.546379	0	0
RPL7P40	0	0	0	0	0	0	0	0
RPL7P41	0	0	0	0	0	0	0	0
RPL7P42	0	0	0	0	0	0	0	0
RPL7P43	0	0	0	0.131532	0	0	0	0
RPL7P44	0	0	0	0	0	0	0	0
RPL7P45	0	0	0	0	0	0	0	0
RPL7P46	0	0	0	0	0	0	0	0
RPL7P47	0	0	0	0	0	0	0	0.353183
RPL7P48	0	0	0	0	0	0	0	0
RPL7P49	0	0	0	0.142432	0	0	0	0
RPL7P5	0	0	0	0	0	0	0	0
RPL7P50	0	0	0	0	0	0	0	0
RPL7P51	0	0	0	0	0	0	0	0
RPL7P52	0	0	0	0	0	0	0	0.23237
RPL7P53	0	0	0	0	0	0	0	0
RPL7P54	0	0	0	0	0	0	0	0
RPL7P55	0	0	0	0	0	0	0	0
RPL7P56	0	0	0	0	0	0	0	0
RPL7P57	0	0	0	0	0	0	0	0
RPL7P58	0	0	0	0	0	0	0	0
RPL7P59	0	0	0	0	0	0	0	0
RPL7P6	0	0.932172	0	0	0	0	0	0
RPL7P60	0	0	0	0	0	0	0	0
RPL7P61	0	0	0	0	0	0	0	0
RPL7P7	0	0	0	0	0	0	0	0
RPL7P8	0	0	0	0	0	0	0	0
RPL7P9	0	0	0	0.287501	0	0	0	2.195015
RPL8	2722.38417	1750.91021	3264.95277	2183.73976	1530.81874	1266.37828	1300.86742	1146.56995
RPL8P1	0	0	0	0	0	0	0	0
RPL8P2	0	0	0	0	0	0	0	0
RPL8P3	0	0	0	0	0	0	0	0

RPL8P4	0	0	0	0	0	0	0	0
RPL8P5	0	0	0	0	0	2.381937	0	0
RPL9	9.500884	13.092217	32.157773	12.434851	42.417531	35.028968	0	73.448074
RPL9P13	0	0	0	0	0	0	0	0
RPL9P14	0	0	0	0	0	0	0	0
RPL9P15	0	0	0	0	0	0	0	0
RPL9P16	0	0	0	0	0	0	0	0
RPL9P18	0	0	0	0	0	0	0	0
RPL9P2	0	0	0	0	0	0	0	0
RPL9P21	0	0	0	0	0	0	0	0
RPL9P25	0	0	0	2.428628	0	0	0	3.650359
RPL9P28	0	0	0	0	0	0	0	0
RPL9P29	0	0	0	0	0	0	0	0
RPL9P3	0	0	0	0	0	0	0	0
RPL9P30	0	0	0	0	0	0	0	0
RPL9P31	0	0	0	0	0	0	0	0
RPL9P32	0	0	0	0	0	0	0	0
RPL9P33	0	0	0	0	0	0	0	0
RPL9P5	0	0	0	0	0	0	0	0
RPL9P6	0	0	0	0	0	0	0	0
RPL9P7	0	0	0	0	0	0	0	0
RPLP0	2565.55431	881.813778	2961.4789	933.318678	2677.69566	936.96957	2402.07534	1199.2053
RPLP0P1	0	0	0	0	0	0	0	0
RPLP0P10	0	0	0	0	0	0	0	0
RPLP0P11	0	0	0	0	0	0	0	0
RPLP0P12	0	0	0	0	0	0	0	0
RPLP0P2	0	4.052807	3.573124	5.073637	0	0.800373	0	0.530674
RPLP0P3	0	0	0	0	0	0	0	0
RPLP0P4	0	0	0	0	0	0	0	0
RPLP0P5	0	0	0	0	0	0	0	0
RPLP0P6	0	0	0	0.253746	1.235755	0.25005	0	0
RPLP0P7	0	0	0	0	0	0	0	0
RPLP0P8	0	0	0	0	0	0	0	0

RPLP0P9	0	0	0	0	0	0	0	0
RPLP1	1063.95071	553.625878	1610.57701	485.38651	1182.42497	524.373773	874.557448	548.967358
RPLP1P10	0	0	0	0	0	0	0	0
RPLP1P11	0	0	0	0	0	0	0	0
RPLP1P13	0	0	0	0	0	0	0	0
RPLP1P5	0	0	0	0	0	0	0	0
RPLP1P6	0	0	0	0	0	0	0	0
RPLP2	1917.49684	1485.47325	2308.50667	1680.21028	1876.47459	1913.78212	1638.76705	2790.87904
RPLP2P1	0	0	0	0	0	0	0	0
RPN1	250.606221	241.962311	309.912782	285.832408	196.708474	144.985207	351.645724	134.471442
RPN2	248.442467	224.346324	372.044236	281.055989	223.421365	115.201426	136.988669	99.294469
RPP14	11.631603	8.546666	3.878879	17.640426	27.907028	31.712616	4.125714	10.693307
RPP21	32.128456	60.593346	63.158411	58.206386	0.629517	82.70687	45.841992	68.138953
RPP25L	32.049175	29.294478	12.069206	28.787363	14.40793	38.369906	24.003739	25.003583
RPP30	48.50189	40.030433	39.07982	25.678402	86.133819	26.038211	45.953868	43.567136
RPP38	9.69527	56.207694	12.571075	40.313967	20.541355	45.933834	38.340347	50.445584
RPP40	18.359317	7.39488	12.324657	3.794219	9.645781	3.870133	0	17.67164
RPP40P1	0	0	0	0	0	0	0	0
RPP40P2	0	0	0	0	0	0	0	0
RPRD1A	31.985032	47.025507	36.704347	38.756456	19.843695	37.667917	104.392347	53.263382
RPRD1B	0	56.607967	0	96.056849	0	94.422838	0	59.057445
RPRD2	7.885345	4.485895	6.572555	3.803779	7.477115	1.966544	0	5.507285
RPRM	0	0	0	0.18413	0	0	0	0
RPRML	0	0.285399	0	0.580257	0	0	0	0.701591
RPS10	1696.10301	596.712526	1929.04945	590.057992	1700.79606	681.452498	1259.93861	635.045829
RPS10-NUDT	8.604479	22.299725	18.177136	17.073441	11.346105	16.146314	0	15.548848
RPS10P10	0	0	0	0	0	0	0	0
RPS10P13	0	0	0	0	0	0	0	0
RPS10P14	0	0	0	0	0	0	0	0
RPS10P16	0	0	0	0	0	0	0	0
RPS10P18	0	0	0	0	0	0	0	0
RPS10P2	0	0	0	0	0	0	0	0
RPS10P20	0	0	0	0	0	0	0	0

RPS10P21	0	0	0	0	0	0	0	0
RPS10P27	0	0	0	0	0	0	0	0
RPS10P28	0	0	0	0	0	0	0	0
RPS10P29	0	0	0	0.265117	0	2.063043	0	0
RPS10P3	0	0	0	0	0	0	0	0
RPS10P30	0	0	0	0	0	0	0	0
RPS10P4	0	0	0	0	0	0	0	0.424645
RPS10P5	9.050906	1.065124	0	0.4261	0	0	0	0
RPS10P7	0	0	0	3.075602	0	0	2.341908	1.76641
RPS10P9	11.398436	0	0	0	0	0	0	0
RPS11	1600.24757	695.425751	1969.5619	859.028621	1928.44527	1101.47364	1292.55562	967.816492
RPS11P1	0	0	0	0	0	0	0	0
RPS11P5	0	0	0	0	0	0	0	0
RPS11P7	0	0	0	0	0	0	0	0
RPS12	2035.26688	1269.04856	2273.55898	976.477375	1399.51097	959.72803	1719.28283	1930.77898
RPS12P10	0	0	0	0	0	0	0	0
RPS12P14	0	0	0	0	0	0	0	0
RPS12P15	0	0	0	0	0	0	0	0
RPS12P16	0	0	0	0.317475	0	0	0	0
RPS12P17	0	0	0	0	0	0	0	0
RPS12P18	0	0	0	0	0	0	0	0
RPS12P2	0	0	0	0	0	0	0	0
RPS12P20	0	0	0	0	0	0	0	0
RPS12P21	0	0	0	0	0	0	0	0
RPS12P23	0	0	0	0	0	0	0	0
RPS12P24	0	0	0	0	0	0	0	0
RPS12P26	0	1.065584	0	0	0	0	0	0
RPS12P27	0	0	0	0	0	0	0	0
RPS12P28	0	0	0	0	0	0	0	0
RPS12P3	0	0	0	0	0	0	0	0
RPS12P31	0	0	0	0	0	0	0	0
RPS12P32	0	0	0	0.664562	0	0	0	0
RPS12P4	0	0	0	0	0	0	0	0

RPS12P5	0	0	0	0	0	0	0	0
RPS12P6	0	0	0	0	0	0	0	0
RPS12P7	0	0	0	0	0	0	0	0
RPS13	2188.85992	421.40018	2408.83045	362.97581	1692.34979	225.50777	1525.2587	525.848234
RPS13P2	0	0	0	0	0	0	0	0
RPS13P3	0	0	0	0	0	0	0	0
RPS13P4	0	0	0	0	0	0	0	0
RPS13P5	0	0	0	0	0	0	0	0
RPS14	2286.59459	749.694674	2607.22216	821.869664	1556.35796	631.6006	877.137544	621.662264
RPS14P4	0	1.965721	0	0	0	0.587698	0	0
RPS14P5	0	0	0	0	0	0	0	0
RPS14P7	0	0	0	0	0	0	0	0
RPS14P8	0	0	0	0	0	0	0	0
RPS15	25.364194	12.343227	0	10.120055	143.683544	36.113801	0	31.168067
RPS15A	2573.27053	1117.12734	3077.81463	1187.74727	2323.02814	703.280611	1138.75868	1028.40914
RPS15AP1	0	2.185691	0	0.339607	0	0	0	0
RPS15AP10	0	0	0	0.352709	0	0	0	0
RPS15AP11	0	0	0	0	0	0	0	0
RPS15AP12	0	0	0	0	0	0	0	0
RPS15AP13	0	0	0	0	0	0	0	0
RPS15AP14	0	0	0	0	0	0	0	1.266666
RPS15AP15	0	0	0	0	0	0	0	0
RPS15AP16	0	0	0	0	0	0	0	0
RPS15AP17	0	0	0	0	0	0	0	0
RPS15AP18	0	0	0	0	0	0	0	0
RPS15AP19	0	0	0	0	0	0	3.362419	0
RPS15AP20	0	0	0	0	0	0	0	0
RPS15AP24	0	0	0	0	0	0	0	0
RPS15AP25	0	0	0	0	0	0	0	0
RPS15AP27	0	0	0	0	0	0	0	0
RPS15AP28	0	0	0	0	0	0	0	0
RPS15AP29	0	0	0	0	0	0	0	0
RPS15AP3	0	0	0	0	0	0	0	0

RPS15AP30	0	0	0	0	0	0	0	0
RPS15AP32	0	0	0	0	0	0	0	0
RPS15AP34	0	0	0	0	0	0	0	0
RPS15AP36	0	0	0	0	0	0	0	0
RPS15AP37	0	0	0	0	0	0	0	0
RPS15AP38	0	0	0	0	0	0	0	0
RPS15AP39	0	0	0	0	0	0	0	0
RPS15AP40	0	0	0	0	0	0	0	0
RPS15AP5	0	0	0	0	0	0	0	0
RPS15AP6	0	0	0	0	0	0	0	0
RPS15AP7	0	0	0	0	0	0	0	0
RPS15AP9	0	0	0	0	0	0	0	0
RPS15P4	0	0	0	0.29041	0	0	0	0
RPS15P5	0	0.468238	0	0	0	0	0	0
RPS15P9	0	0	0	0	0	0	0	0
RPS16	1170.2313	579.841694	1172.92474	659.193987	1386.09924	696.422407	1188.32631	1037.37685
RPS16P2	0	0	0	3.923758	0	0	0	0
RPS16P3	0	0	0	0	0	0	0	0
RPS16P4	0	0	0	0	0	0	0	0
RPS16P5	0	0	0	0	0	0	0	0
RPS16P8	0	0	0	0	0	0	0	0
RPS16P9	0	0	0	0	0	0	0	0
RPS17	5543.41036	3333.90328	5487.72179	3324.60707	3003.18947	2213.32687	2752.08007	3097.76749
RPS17P1	0	0	0	0	0	0	0	0
RPS17P10	0	0	0	0	0	0	0	0
RPS17P11	0	0	0	0	0	0	0	0
RPS17P13	0	0	0	0	0	0	0	0
RPS17P14	0	0	0	0	0	0	0	0
RPS17P15	0	0	0	0	0	0	0	0
RPS17P16	0	1.034964	0	0	0	0	0	0
RPS17P17	0	0	0	0	0	0	0	0
RPS17P2	0	0	0	0	0	0	0	0
RPS17P5	0	0	0	0	0	0	0	0

RPS17P7	0	0	0	0	0	0	0	0
RPS17P8	0	0	0	0	0	0	0	0
RPS17P9	0	0	0	0	0	0	0	0
RPS18	4365.32186	1947.87372	4499.59805	2144.91724	3095.37443	1679.0085	2854.49903	2445.06567
RPS18P1	0	0	0	0	0	0	0	0
RPS18P12	0	0	0	0.262467	0	0	0	0
RPS18P13	0	0	0	0	0	0	0	0
RPS18P14	0	0	0	0	0	0	0	0.48521
RPS18P5	11.557145	1.84863	0	1.373531	0	0	0	1.490209
RPS18P6	0	0	0	0	0	0	0	0
RPS18P8	0	0	0	0	3.344942	0	0	0
RPS18P9	0	0	0	2.222784	0	4.72342	0	1.989592
RPS19	1360.16048	839.087168	2064.00824	869.260036	1851.13269	1389.14159	892.582722	1324.7655
RPS19BP1	39.620246	86.078853	43.423719	66.881278	37.636657	107.323368	30.416846	101.528991
RPS19P1	0	0	0	0	0	0	0	0
RPS19P3	0	3.180942	0	0	0	0	0	0
RPS19P6	0	0	0	0	0	0	0	0
RPS19P7	0	0	0	0	0	0	0	0
RPS2	4285.17323	1262.44829	4658.79807	1376.2134	2656.96043	897.461889	2039.07308	1070.07571
RPS20	59.257577	10.640647	0	8.222368	0	3.420698	0	9.239086
RPS20P1	0	0	0	0	0	0	0	0
RPS20P10	0	0	0	0	0	0	0	0
RPS20P12	0	0	0	0	0	0	0	0
RPS20P13	0	0	0	0	0	0	0	0
RPS20P14	0	0	0	0	0	0	0	0.751471
RPS20P15	0	0	0	0	0	0	0	0
RPS20P2	0	0	0	0	0	0	0	0
RPS20P20	0	0	0	0	0	0	0	0
RPS20P21	0	0	0	0	0	0	0	0
RPS20P22	0	0	0	0	0	0	0	0.548097
RPS20P23	0	0	0	0	0	0	0	0
RPS20P24	0	0	0	0.388442	0	0	0	0
RPS20P25	0	0	0	0	0	0	0	0

RPS20P31	0	0	0	0	0	0	0	0
RPS20P32	0	0	0	0	0	0	0	0
RPS20P33	0	0	0	1.205831	0	0.765872	0	0
RPS20P4	0	0	0	0	0	0	0	0
RPS20P5	0	0	0	0	0	0	0	0
RPS20P9	0	0	0	0	0	0	0	0
RPS21	2316.78311	1813.38379	2743.32091	1878.94225	1790.13164	1423.04759	1896.03904	2233.52906
RPS21P1	0	0	0	0	0	0	0	0
RPS21P2	0	0	0	0	0	0	0	0
RPS21P4	0	0	0	0	0	0	0	0
RPS21P7	0	0	0	0	0	0	0	0
RPS21P8	0	0	0	0	0	0	0	0
RPS23	3116.51247	1544.95749	3503.97365	1509.45918	1766.99798	885.848107	1960.34319	1552.9767
RPS23P1	0	0	0	0	0	0	0	0
RPS23P10	0	0	0	0	0	0	0	0
RPS23P2	0	0	0	0	0	0	0	0
RPS23P3	0	0	0	0	0	0	0	0
RPS23P4	0	0	0	0	0	0	0	0
RPS23P5	0	0	0	0	0	0	0	0
RPS23P6	0	0	0	0	0	0	0	0
RPS23P7	0	0	0	0	0	0	0	0
RPS23P8	0	3.34925	0	0	0	1.062569	0	0
RPS23P9	0	0	0	0	0	0	0	0
RPS24	3225.21563	3926.99365	3387.47105	3875.99018	2192.74828	3423.5039	2490.45127	2771.47026
RPS24P1	0	0	0	0	0	0	0	0
RPS24P10	0	0	0	0	0	0	0	0
RPS24P12	0	0	0	0	0	0	0	0
RPS24P13	0	0	0	0	0	0	0	1.243431
RPS24P14	0	0	0	0	0	0	0	0
RPS24P16	0	0	0	0	0	0	0	0
RPS24P17	0	0	0	0	0	0	0	0
RPS24P18	0	0	0	0	0	0	0	0
RPS24P19	0	0.558259	0	0.96179	0	1.205904	0	0

RPS24P21	0	0	0	0	0	0	0	0
RPS24P6	0	0	0	0	0	0	0	0
RPS24P7	0	0	0	0	0	0	0	0
RPS24P8	0	0	0	0	0	0	0	0
RPS24P9	0	0	0	0	0	0	0	0
RPS25	12996.1934	9070.87235	17467.6434	10041.4432	2386.45124	1955.08749	2119.05928	2269.44643
RPS25P10	0	0	0	0	0	0	0	0
RPS25P3	0	0	0	0.727767	0	0	0	0
RPS25P4	0	0	0	0	0	0	0	0
RPS25P5	0	0	0	0	0	0	0	0
RPS25P7	0	0	0	0	0	0	0	0
RPS25P9	0	0	0	0	0	0	0	0
RPS26	645.12077	337.477462	777.386209	393.989412	281.338323	179.103893	191.627862	271.317248
RPS26P11	0	0	0	0	0	0	0	0
RPS26P13	0	0	0	0	0	0	0	0
RPS26P15	0	0	0	0	0	0	0	0.860066
RPS26P18	0	0	0	0	0	0	0	0
RPS26P19	0	0	0	13.720178	0	15.207783	179.563731	34.45135
RPS26P2	0	0	0	0	0	0	0	0
RPS26P20	0	0	0	0.414168	0	0	0	0
RPS26P21	0	0	0	0	0	0	0	0
RPS26P22	0	0	0	0	0	0	0	0
RPS26P24	0	0	0	0	0	0	0	0
RPS26P28	0	0	0	0	0	0	0	0
RPS26P29	0	0	15.174347	0.416267	0	0	0	2.400366
RPS26P3	0	0	0	0	0	0	0	0
RPS26P30	0	0	0	0	0	0	0	0
RPS26P31	0	0	0	0	0	0	0	0.817245
RPS26P34	0	0	0	0	0	0	0	0
RPS26P35	0	0	0	0	0	0	0	0
RPS26P38	0	0	0	0	0	0	0	0
RPS26P39	0	0	0	0	0	0	0	0
RPS26P4	0	0	0	0	0	0	0	0

RPS26P40	0	0	0	0	0	0	0	0
RPS26P41	0	0	0	0	0	0	0	0
RPS26P42	0	0	0	0	0	0	0	0
RPS26P43	0	0	0	0	0	0	0	0
RPS26P45	0	0	0	0	0	0	0	0
RPS26P47	0	0	0	0	0	0	0	0
RPS26P48	0	0	0	0	0	0	0	0
RPS26P49	0	0	0	0	0	0	0	0
RPS26P5	0	0	0	0	0	0	0	0
RPS26P52	0	0	0	0	0	0	0	0
RPS26P54	0	0	0	0	0	0	0	0
RPS26P55	0	0	0	0	0	0	0	0
RPS26P56	0	0	0	0	0	0	0	0
RPS26P57	0	0	0	0	0	0	0	0
RPS26P58	0	0	0	0	0	0	0	0
RPS26P59	0	0	0	0	0	0	0	0
RPS26P6	0	0	0	1.56686	0	0	0	0
RPS26P8	0	0	0	0.482199	0	0	0	0
RPS27	4055.31865	1427.88678	4531.18073	1729.62863	2297.36089	1197.709	2513.8502	1398.32705
RPS27A	2022.45246	942.433804	2179.63731	766.519491	1535.32631	625.153301	1306.3211	782.431844
RPS27AP1	0	0	0	0	0	0	0	0
RPS27AP10	0	0	0	0	0	0	0	0
RPS27AP11	11.896126	0	0	0	0	0	0	0
RPS27AP12	0	0	0	0	0	0.501185	33.34156	0.471136
RPS27AP13	0	0	0	0	0	0	0	0
RPS27AP14	0	0	0	0	0	0	0	0
RPS27AP15	0	0	0	0	0	0	0	0
RPS27AP16	0	0	0	0	0	0	0	0
RPS27AP17	0	0	0	0	0	0	0	0
RPS27AP18	0	0	0	0	0	0	0	0
RPS27AP19	0	0	0	0	0	0	0	0
RPS27AP2	0	0	0	2.058082	0	0.501185	0	0
RPS27AP20	0	0	0	0	0	0	0	0

RPS27AP3	0	0	0	0	0	0	0	0
RPS27AP6	0	0	0	0	0	0	0	0
RPS27AP7	0	0	0	0	0	0	0	0
RPS27AP8	0	0	0	0	0	0	0	0
RPS27AP9	0	0	0	0	0	0	0	0
RPS27L	72.159296	31.13666	15.659784	31.988457	75.200688	26.50988	4.407717	37.505064
RPS27P1	0	0	0	0	0	0	0	0
RPS27P10	0	0	0	0	0	0	0	0
RPS27P11	0	0	0	0	0	0	0	0
RPS27P12	0	0	0	0	0	0	0	0
RPS27P13	0	0	0	0	0	0	0	0
RPS27P14	0	0	0	0	0	0	0	0
RPS27P15	0	0	0	0	0	0	0	0
RPS27P16	0	0	0	0	0	8.977823	0	0
RPS27P17	0	0	0	0	0	0	0	0
RPS27P18	0	0	0	0	0	0	0	0
RPS27P19	0	1.114051	0	0	0	0	0	0
RPS27P2	0	0	0	0	0	0	0	0
RPS27P20	0	0	0	0	0	0	0	0
RPS27P21	0	0	0	0	0	0	0	0
RPS27P22	0	0	0	0	0	0	0	0
RPS27P23	0	0	0	0	0	0	0	0
RPS27P24	0	0	0	0	0	0	0	0
RPS27P25	0	0	0	0	0	0	0	0
RPS27P26	0	0	0	0	0	0	0	0
RPS27P27	0	0	0	0	0	0	0	0
RPS27P28	0	0	0	0	0	0	0	0
RPS27P29	0	0	0	0	0	0	0	0
RPS27P3	0	0	0	0.846179	0	0	0	1.577376
RPS27P30	0	0	0	0	0	0	0	0
RPS27P4	0	0	0	0	0	0	0	0
RPS27P5	0	0	0	0	0	0	0	0
RPS27P6	0	0	0	0	0	0	0	0

RPS27P7	0	2.193371	0	0	0	0	0	0
RPS27P8	0	1.320291	0	0	0	1.674624	0	1.626547
RPS27P9	0	0	0	0.774087	0	0	0	0
RPS28	46.588939	144.629527	286.914233	142.027873	55.631783	116.30089	145.4625	117.420398
RPS28P1	0	0	0	0	0	0	0	0
RPS28P4	0	0	0	0	0	0	0	0
RPS28P5	0	0	0	0	0	0	0	0
RPS28P7	553.664357	471.901649	962.951996	543.990749	687.836404	428.33567	364.853278	415.506839
RPS28P8	0	0	0	0	0	0	0	0
RPS29	17.098658	7.748229	5.615316	7.472606	28.630796	9.717045	2.579612	12.977256
RPS29P1	0	0	0	0	0	0	0	0
RPS29P10	0	0	0	0	0	0	0	0
RPS29P11	0	0	0	0	0	0	0	0
RPS29P12	0	0	0	0	0	0	0	0
RPS29P13	0	0	0	0	0	0	0	0
RPS29P14	0	0	0	1.821854	0	0	0	0
RPS29P15	0	0	0	0	0	0	0	0
RPS29P16	0	0	0	0	0	0	0	0
RPS29P17	0	0	0	0	0	0	0	0
RPS29P19	0	0	0	0	0	0	0	0
RPS29P2	0	0	0	0	0	0	0	0
RPS29P20	0	0	0	0	0	0	0	0
RPS29P21	0	0	0	0	0	0	0	0
RPS29P22	0	0	0	0	0	0	0	0
RPS29P23	0	0	0	0	0	0	0	0
RPS29P24	0	0	0	0	0	0	0	0
RPS29P25	0	0	0	0	0	0	0	0
RPS29P26	0	0	0	0	0	0	0	0
RPS29P27	0	0	0	0	0	0	0	0
RPS29P28	0	0	0	0	0	0	0	0
RPS29P29	0	0	0	0	0	0	0	0
RPS29P3	0	0	0	0	0	0	0	0
RPS29P30	0	0	0	0	0	0	0	0

RPS29P31	0	0	0	0	0	0	0	0
RPS29P32	0	0	0	0	0	0	0	0
RPS29P33	0	0	0	0	0	0	0	0
RPS29P4	0	0	0	0	0	0	0	0
RPS29P5	0	0	0	0	0	0	0	0
RPS29P6	0	0	0	0	0	0	0	0
RPS29P7	0	0	0	0	0	0	0	0
RPS29P8	0	0	0	0	0	0	0	0
RPS29P9	0	0	0	0	0	0	0	0
RPS2P1	0	0	0	0	0	0	0	0
RPS2P15	0	0	0	0	0	0	0	0
RPS2P16	0	0	0	0	0	0	0	0
RPS2P17	0	0.210601	0	0.123345	0	0	0	0.856082
RPS2P18	0	0	0	0	0	0	0	0
RPS2P19	0	0	0	0	0	0	0	0
RPS2P2	0	0	6.522711	0	0	0	0	0
RPS2P24	0	0	0	0	0	0	0	0
RPS2P25	0	0	0	0	0	0	0	0
RPS2P28	0	0	0	0	0	0	0	0
RPS2P29	0	0	0	0.108494	0	0	0	0
RPS2P32	4.449329	2.909888	11.736937	3.617075	0	1.496004	0	0.546177
RPS2P35	0	0	0	0	0	0	0	0
RPS2P36	0	0	0	0	0	0	0	0
RPS2P39	0	0	0	0	0	0	0	0
RPS2P4	0	0	0	0	0	0.225089	0	0
RPS2P41	0	0	0	0	0	0	0	0
RPS2P44	0	0	0	0	0	0	0	0
RPS2P45	0	0	0	0	0	0.227472	0	0
RPS2P46	15.549446	6.164387	4.436165	2.576981	9.668869	1.798874	23.131454	2.670753
RPS2P48	0	0	0	0	0	0	0	0
RPS2P5	315.23671	119.973934	344.323202	126.060207	208.176823	89.024493	231.540445	73.070894
RPS2P52	0	0	0	0	0	0	0	0
RPS2P53	0	0	0	0	0	0	0	0

RPS2P54	0	0	0	0	0	0	0	0
RPS2P55	4.614456	0	0	0.112537	0	0	0	0
RPS2P6	0	0	0	0	0	0	0	0
RPS2P7	0	0.186416	0	0	0	0	0	0
RPS3	5673.04515	2852.14663	6715.52573	3634.65802	3202.65431	1609.85249	2804.73005	1607.24227
RPS3A	2639.53474	1096.64042	2780.83546	867.940642	1239.83006	450.963822	1512.10579	834.148674
RPS3AP1	0	0	0	0	0	0	0	0
RPS3AP10	0	0	0	0	0	0	0	0
RPS3AP11	0	0	0	0	0	0	0	0
RPS3AP12	0	0	0	0	0	0	0	0
RPS3AP13	0	0	0	0	0	0	0	0
RPS3AP14	0	0	0	0	0	0	0	0
RPS3AP15	0	0	0	0	0	0	0	0
RPS3AP16	0	0	0	0	0	0	0	0
RPS3AP17	0	0	0	0	0	0	0	0
RPS3AP18	0	0	0	0.379121	0	0	0	0
RPS3AP19	0	0	0	0	0	0	0	0
RPS3AP2	0	0.445669	0	0.131309	0	0	0	0.454688
RPS3AP20	0	1.103363	0	0.126711	0	0.253946	0	0
RPS3AP21	0	1.358365	0	0.871254	0	0	0	0
RPS3AP22	0	0	0	0	0	0	0	0
RPS3AP23	0	0	0	0	0	0	0	0
RPS3AP24	0	0	0	0	0	0	0	0
RPS3AP25	0	0	0	0	0	0	0	0
RPS3AP26	0	0.276408	0	0	1.743879	0	0	0
RPS3AP27	0	0	0	0	0	0	0	0
RPS3AP28	0	0	0	0	0	0	0	0
RPS3AP29	0	0	0	0	0	0	0	0.442718
RPS3AP3	0	0	0	0	0	0	0	0
RPS3AP30	0	0	0	0	0	0	0	0
RPS3AP31	0	0	0	0	0	0	0	0
RPS3AP32	0	0	0	0	0	0	0	0
RPS3AP33	0	0	0	0	0	0	0	0

RPS3AP34	0	0	0	0.251106	0	0	0	0.21683
RPS3AP35	0	0	0	0	0	0	0	0
RPS3AP36	0	0	0	0	0	0	0	0
RPS3AP37	0	0	0	0	0	0	0	0
RPS3AP38	0	0	0	0	0	0	0	0
RPS3AP39	0	0	0	0	0	0	0	0
RPS3AP4	0	0	0	0	0	0	0	0
RPS3AP40	0	0	0	0.125553	0	0	0	0
RPS3AP41	0	0	0	0	0	0	0	0
RPS3AP42	0	0	0	0	0	0	0	0
RPS3AP43	0	0	0	0	0	0	0	0
RPS3AP44	0	0	0	0	0	0	0	0
RPS3AP46	0	0	0	0	0	0	0	0
RPS3AP47	0	0.213597	0	0	0	0	1.230425	0
RPS3AP48	0	0	0	0	0	0	0	0
RPS3AP49	0	0	0	0	0	0	0	0
RPS3AP5	0	0	0	0	0	0.724252	0	1.065475
RPS3AP50	0	0	0	0	0	0	0	0
RPS3AP51	0	0	0	0	0	0	0	0
RPS3AP52	0	0	0	0	0	0	0	0
RPS3AP53	0	0	0	0	0	0	0	0
RPS3AP54	0	0	0	0.135924	0	0.271325	0	0
RPS3AP55	0	0	0	0	0	0	0	0
RPS3AP6	0	0	0	0	2.12021	0	0	0.31803
RPS3AP7	0	0.213454	0	0	0	0	0	0
RPS3AP8	0	0	0	0	0	0	0	0
RPS3AP9	0	0	0	0	0	0	0	0
RPS3P1	0	0	0	0	0	0	0	0
RPS3P2	0	0	0	0	0	0	0	0
RPS3P3	0	0	0	0	0	0.272256	0	0
RPS3P4	0	3.260919	0	0.547932	0	2.189394	0	0.475516
RPS3P6	0	0	0	0	0	0	0	0
RPS3P7	0	0	0	0	0	0	0	0

RPS4X	1663.41126	703.918784	1687.73021	786.943765	1130.5943	486.365245	925.956793	469.119289
RPS4XP1	0	0	0	0	0	0	0	0
RPS4XP10	0	0	0	0	0	0	0	0
RPS4XP11	0	0	0	0	0	0	0	0
RPS4XP12	0	0	0	0	0	0	0	0
RPS4XP13	0	0	0	0.123944	0	0	0	0
RPS4XP14	0	0	0	0	0	0	0	0
RPS4XP15	0	0	0	0	0	0	0	0
RPS4XP16	0	0	0	0.33406	0	0	0	0.878414
RPS4XP17	0	0	0	0.393927	0	0	0	0
RPS4XP18	0	0	0	0	0	0	0	0
RPS4XP19	0	0	0	0	0	0	0	0
RPS4XP2	0	0	0	0	0	0	0	0
RPS4XP20	0	0	0	0	0	0	0	0
RPS4XP21	0	0	0	0	0	0	1.318837	0
RPS4XP22	0	0.641293	0	0.754545	0	0	0	0
RPS4XP23	0	0	0	0	0	0	0	0
RPS4XP3	0	0	0	0	0	0	0	0
RPS4XP4	0	0	0	0	0	0	0	0
RPS4XP5	5.393037	0	0	0	0	0	0	0.886991
RPS4XP6	0	0	0	0	0	0	0	0
RPS4XP7	0	0	0	0	0	0	0	0
RPS4XP8	0	0	0	0	0	0	0	0
RPS4XP9	0	0	0	0	0	0	0	0
RPS4Y1	317.309074	240.846818	334.668531	269.301887	560.500585	310.878504	352.351683	335.436818
RPS5	741.443286	476.118736	835.811077	473.833637	805.245929	715.911583	652.51726	789.112054
RPS5P2	0	0	0	0	0	0	0	0
RPS5P3	0	0	0	0	0	0	0	0
RPS5P7	0	0	0	0	0	0	0	0
RPS5P8	0	0	0	0	0	0	0	0
RPS6	1625.02943	1827.47881	1717.01281	1693.17848	1683.03666	1663.42023	1549.47698	1745.78812
RPS6KA1	23.049671	52.839336	32.823028	51.137759	73.807536	139.145747	163.480729	108.048272
RPS6KA2	33.948776	36.980746	40.208266	103.297803	14.596096	54.530316	9.247941	41.787949

RPS6KA3	28.553726	48.8194	19.401316	27.629709	29.889621	23.646841	43.037066	81.485607
RPS6KA4	15.022512	42.507433	9.593831	34.977219	53.17032	119.045245	95.205015	87.553491
RPS6KA5	6.51765	9.41725	6.175423	14.68715	7.47288	19.851898	15.789004	20.309991
RPS6KA6	6.470281	12.742071	5.326635	6.040433	2.112445	2.240825	3.466317	4.208661
RPS6KB1	81.804397	95.218099	49.697822	131.788474	19.820215	85.09989	46.374052	91.116987
RPS6KB2	51.119073	138.26543	34.746288	110.21817	105.317355	188.979265	85.637812	150.420756
RPS6KC1	23.648907	26.823966	15.462173	15.570614	17.530279	14.430295	31.090373	19.387853
RPS6KL1	0	21.588251	2.480645	14.433117	0	2.330359	0	0.701083
RPS6P12	0	0	0	0.275723	0	0	0	0
RPS6P14	0	0	0	0	0	0	0	0
RPS6P15	0	0	0	0	0	0	0	0
RPS6P16	0	0	0	0	0	0.313595	0	0
RPS6P2	0	0	0	0	0	0	0	0
RPS6P20	0	0	0	0	0	0	0	0
RPS6P21	0	0	0	0	0	0	0	0
RPS6P22	0	0	0	0	0	0	0	0
RPS6P23	0	0	0	0	0	0	0	0
RPS6P25	0	0	0	0.265322	0	0	0	0
RPS6P26	0	0	0	0	0	0.259377	0	0.22451
RPS6P3	0	0	0	0	0	0	0	0
RPS6P4	0	0	0	0	0	0	0	0
RPS6P7	0	0	0	0	0	0	0	0
RPS6P8	0	0	0	0	0	0	0	0
RPS7	2329.60754	979.234245	2637.41963	874.662426	1847.7795	650.640631	1429.30148	941.673341
RPS7P1	9.700907	0.419998	0	0.235878	0	2.176581	0	0.848627
RPS7P10	0	0	0	0.179211	0	0	0	0
RPS7P11	0	0	0	0.201215	0	0.392433	0	0.35313
RPS7P12	0	0	0	0	0	0	0	0
RPS7P13	0	0	0	0	0	0	0	0
RPS7P14	0	0	0	0	0	0	0	0
RPS7P15	0	0	0	0	0	0	0	0
RPS7P2	0	0	0	0	0	0	0	0
RPS7P3	0	0	0	0	0	0	0	0

RPS7P4	0	0	6.819469	0	0	0	0	0
RPS7P5	0	0	0	0	0	0	0	0
RPS7P6	0	0	0	0	0	0	0	0
RPS7P7	0	0	0	0	0	0	0	0
RPS7P8	0	0	0	0	0	0	0	0
RPS7P9	0	0	0	0	0	0	0	0
RPS8	1770.97334	1796.74069	2221.54603	1901.94125	2146.50101	2220.33015	1676.24348	1902.24629
RPS8P10	0	0	0	0	0	0	0	0
RPS8P11	0	0	0	0	0	0	0	0
RPS8P3	0	0	0	0	0	0	0	0
RPS8P4	0	0	0	0	0	0	0	0
RPS8P5	0	0	0	0	0	0	0	0
RPS8P6	0	0	0	0	0	0	0	0
RPS9	579.610972	405.840953	724.147467	372.70288	722.673493	409.914653	540.348221	461.983714
RPS9P1	0	0	0	0	0	0	0	0
RPS9P2	0	0	0	0	0	0	0	0
RPSA	1076.28435	360.541662	1186.71879	322.113254	517.139597	212.989363	572.049514	321.048646
RPSA2	0	0	0	0	0	0	0	0
RPSAP1	0	0	0	0	0	0	0	0
RPSAP10	0	0	0	0	0	0	0	0
RPSAP11	0	0	0	0	0	0	0	0
RPSAP12	0	0	0	0.578868	0	0.94245	0	1.5621
RPSAP13	0	0	0	0.108932	0	0	0	1.679765
RPSAP14	0	0	0	1.193311	0	0.773754	0	3.84564
RPSAP15	0	0	0	0	0	0	0	0
RPSAP16	0	0	0	0.353697	0	0	0	0
RPSAP17	0	0	0	0	0	0	0	0
RPSAP18	0	0.746005	0	0	0	0.217447	0	0
RPSAP19	0	0	0	0	0	0.230173	0	0
RPSAP2	0	0	0	0	0	0	0	0
RPSAP20	0	0	0	0	0	0	0	0
RPSAP21	0	0	0	0	0	0	0	0
RPSAP22	0	0	0	0	0	0	0	0

RPSAP23	0	0	0	0	0	0	0	0
RPSAP24	0	0	0	0	0	0	0	0
RPSAP25	0	0	0	0	0	0	0	0
RPSAP26	0	1.37084	0	0	0	0	0	0
RPSAP27	0	0	0	0	0	0	0	0
RPSAP28	0	0	0	0	0	0	0	0
RPSAP29	0	0	0	0	0	0	0	0
RPSAP3	0	0	0	0	0	0	0	0
RPSAP31	0	0.181144	0	0	0	0.212856	0	0
RPSAP32	0	0	0	0	0	0	0	0
RPSAP33	0	0	0	0	0	0	0	0
RPSAP34	0	0	0	0	0	0	0	0
RPSAP35	0	0	0	0	0	0	0	0
RPSAP36	0	0	0	0	0	0	0	0.188313
RPSAP37	0	0	0	0	0	0	0	0
RPSAP38	0	0	0	0	0	0	0	0
RPSAP39	0	0	0	0	0	0	0	0
RPSAP4	0	0	0	0	0	0	0	0
RPSAP40	0	0	0	0	0	0	0	0
RPSAP41	0	8.738483	4.092429	5.898002	0	2.679825	0	3.626641
RPSAP42	0	0	0	0	0	0	0	0
RPSAP43	0	0	0	0	0	0	0	0
RPSAP44	0	0	0	0	0	0	0	0
RPSAP45	0	0	0	0	0	0	0	0
RPSAP46	0	0	0	0	0	0	0	0
RPSAP47	0	0	0	0	0	0	0	0
RPSAP48	0	0	0	0	0	0	0	0
RPSAP49	0	0	0	0	0	0	0	0
RPSAP5	0	0	0	0	0	0.216889	0	0
RPSAP50	0	0	0	0	0	0	0	0
RPSAP51	0	0	0	0	0	0	0	0
RPSAP52	6.563784	42.456176	5.809621	41.414646	0	8.461033	0	14.496649
RPSAP53	0	0	0	0	0	0	0	0

RPSAP54	0	0	0	0.110376	1.224252	0	0	0
RPSAP55	0	0	0	0	0	0	0	0
RPSAP56	0	0	0	0	0	0	0	0
RPSAP57	0	0	0	0	0	0	0	0
RPSAP59	0	0	0	0	0	0	0	0
RPSAP6	0	0	0	0	0	0.433253	0	0
RPSAP60	0	0	0	0	0	0	0	0
RPSAP61	0	0	0	0	0	0	0	0
RPSAP62	0	0.592148	0	0	0	0	0	0
RPSAP63	0	0	0	0	0	0	0	0
RPSAP64	0	0	0	0	0	0	0	0
RPSAP65	0	0	0	0	0	0	0	0
RPSAP66	0	0	0	0	0	0	0	0
RPSAP67	0	0	0	0	0	0	0	0
RPSAP68	0	0	0	0	0	0	0	0
RPSAP69	0	0.36815	0	0.538589	0	0.648995	0	0
RPSAP7	0	0	0	0	0	0	0	0
RPSAP70	0	1.133492	0	0.331938	0	0	0	0.379458
RPSAP71	0	0	0	0	0	0	0	0
RPSAP72	0	0	0	0	0	0	0	0
RPSAP73	0	0	0	0	0	0	0	0
RPSAP74	0	0	0	0	0	0	0	0
RPSAP75	0	0	0	0	0	0	0	0
RPSAP76	0	0	0	0	0	0	0	0
RPSAP8	0	0	0	0	0	0.215843	0	0
RPSAP9	0	1.14017	8.146249	1.642869	0	0	0	0.937986
RPTN	0	0	0	0	0	0	0	0
RPTOR	13.086991	30.047907	10.222694	35.203571	20.725045	40.214223	7.667699	42.903643
RPUSD1	30.017457	49.834897	15.098139	40.42992	22.365984	82.811658	12.926067	68.150393
RPUSD2	7.224572	22.814084	6.461023	23.473997	11.031129	24.823884	9.460621	24.563994
RPUSD3	0	5.715769	5.15504	3.506976	0	3.869714	29.863733	4.085049
RPUSD4	25.651041	17.334847	14.812761	19.106382	25.467911	31.003615	11.84128	38.630911
RRAD	0	1.242216	2.195666	2.508138	0	2.302584	0	1.10015

RRAGAP1	0	0	0	0	0	0	0	0
RRAGB	4.537114	6.724293	8.830152	1.871379	2.389927	6.918475	7.189216	9.970597
RRAGC	21.455485	33.805205	28.246757	15.060204	51.951363	23.814468	36.458312	59.637553
RRAGD	2.450709	3.379798	0.733175	1.265947	5.39326	0.633814	0	1.172734
RRAS	27.496866	33.941542	41.570901	48.986864	61.883513	38.122321	0	25.328226
RRAS2	97.627936	139.356201	101.74817	94.252216	78.178003	102.67128	153.163467	172.781313
RRAS2P1	0	0	0	0	0	0	0	0
RRAS2P2	0	0	0	0	0	0	0	0
RRBP1	68.152018	42.896485	62.97513	93.114866	86.407406	167.609642	53.359409	88.512428
RRBP1P1	0	0	0	0	0	0	0	0
RRBP1P2	0	0	0	0	0	0	0	0
RREB1	26.674519	36.464465	18.560083	58.851747	70.804089	117.498485	31.34859	87.89113
RRH	0	0	0	0	0	0	0	0
RRM1	247.617939	197.744911	155.643892	198.4467	162.26974	262.315026	246.933281	305.070639
RRM2	97.185083	210.907306	130.986006	148.851396	262.49281	245.979907	399.678384	300.556544
RRM2B	0	13.144786	0	6.287636	7.832307	1.990296	29.640346	6.620996
RRM2P2	0	0	0	0	0	0	0	0
RRM2P3	0	0.804117	0	0.697443	0	0.156653	0	0
RRM2P4	0	0	0	0	0	0	0	0
RRN3	51.53846	51.809691	38.297449	36.25738	39.076817	28.767986	41.784584	59.369705
RRN3P2	0	0	0	1.943345	18.715124	0	67.250479	0
RRN3P3	1.001652	7.451679	0	1.479476	0.263445	3.158122	0	6.285958
RRN3P4	0	0	0	0	0	0	0	0
RRP1	0	17.645016	6.155927	19.101992	73.737992	29.947948	75.262715	39.826458
RRP12	4.578454	9.332907	8.258403	1.757374	60.612172	1.317713	0	3.930924
RRP15	15.798365	53.968305	51.193236	45.340528	22.962145	51.23949	8.619464	31.692244
RRP1B	35.41939	75.944893	35.291667	61.690305	47.463798	125.655411	47.689727	109.842351
RRP36	47.990768	76.901934	56.037597	59.189412	58.879081	78.341098	71.9654	82.855789
RRP7A	47.450361	60.377047	42.467447	54.437226	13.505098	59.502534	27.600269	46.352118
RRP7BP	0	0	0	1.142958	0	1.039119	0	0
RRP8	27.071635	33.12527	17.971631	40.803746	32.133479	67.555325	23.402558	46.886412
RRP9	39.445566	20.173502	20.648583	18.645373	16.498577	11.862174	24.636809	9.953809
RRS1	41.03012	41.244364	29.254474	36.150936	22.161836	35.905899	21.986907	34.449557

RS1	0	0	0	0	0	0	0	0
RSAD1	12.423328	52.058284	22.228797	49.078948	34.958646	59.51475	12.055057	48.634666
RSAD2	0.97783	0.379136	0	0	15.86608	6.503908	6.367943	3.222587
RSBN1	15.357825	8.525994	13.610907	7.017093	12.052021	5.824325	2.997881	6.542346
RSBN1L	38.457871	11.167742	41.363917	10.238017	34.329985	10.24645	103.155238	10.322966
RSF1	74.765265	100.305718	140.226018	96.366822	69.055192	49.280669	71.076591	50.309588
RSKR	0	4.716453	9.259035	6.392662	3.897014	14.213311	0	5.205251
RSL1D1	287.530861	437.583403	300.318374	584.698205	292.831867	604.989836	695.801469	456.532683
RSL24D1	131.222797	80.830836	146.77063	55.155209	61.868116	23.307788	47.84817	36.7456
RSL24D1P1	0	0	0	0	0	0	0	0
RSL24D1P11	0	0	0	0	0	0	0	0
RSL24D1P2	0	0	0	0	0	0	0	0
RSL24D1P3	0	0	0	0	0	0	0	0
RSL24D1P4	0	0	0	0	0	0	0	0
RSL24D1P5	0	0	0	0	0	0	0	0
RSL24D1P6	0	0	0	0	0	0	0	0
RSL24D1P8	0	0	0	0	0	0	0	0
RSL24D1P9	0	0	0	0	0	0	0	0
RSPH1	0	3.776444	10.021399	8.471233	0.742826	2.070329	0	1.890252
RSPH10B	0	0	0	0	0	0	0	0
RSPH10B2	0	0	0	0.142406	0	0	0	0
RSPH14	0	0	0	0.449268	0	0	0	0
RSPH3	0	0	0	0	0	0	0	0
RSPH4A	0	0	0	0.062116	0	0	0	0.729018
RSPH6A	0	0	0	0	0	0	0	0
RSPH9	0	0	0	0	0	0	0	0.107489
RSPO1	0	0	0	0	0	0	0	0
RSPO2	1.368384	0	0	0	0	0	0	0
RSPO3	0	0	0	0	0	0	0	0
RSPO4	0	0	0	0	0	0	0	0
RSPRY1	71.745033	170.06792	24.481028	140.179704	46.020343	153.268488	33.481931	160.916552
RSRC1	29.698458	53.075246	27.147396	57.883794	41.838186	54.658676	56.524265	50.130344
RSRC2	48.185795	49.281396	36.152758	57.977581	43.271203	41.602927	58.089309	33.113455

RSRP1	0	11.473299	0	18.413255	11.064129	14.335793	0	18.982381
RSU1	13.786681	18.455115	7.973508	28.016977	33.119694	15.544031	4.881103	19.056288
RSU1P1	0	0	0	0	0	0	0	0
RSU1P2	0	0	0	0	0	0	0	0
RSU1P3	0	0	0	0	0	0	0	0
RTBDN	12.915462	3.721207	7.335003	12.478182	0	0.329866	0	0
RTCA	44.496048	45.407732	16.516524	24.11462	10.728144	18.466847	53.646014	23.861045
RTCB	46.36714	71.67969	39.869383	69.003683	37.125962	54.811447	109.720841	57.635307
RTEL1	12.880432	4.922483	21.381836	7.654511	11.708161	14.469396	0	18.14696
RTEL1-TNFRS	0	0.409296	0	0.526366	0	1.503017	0	0.319158
RTEL1P1	0	0	0	0.021967	0	0.044951	0	0
RTF1	0	51.912037	0	24.35156	128.147378	32.710478	60.39841	22.007267
RTF2	66.823422	219.62933	99.6174	213.841644	77.378037	204.311451	8.814721	158.606043
RTKN	41.967439	13.499006	0	12.388247	14.900612	29.679963	42.169158	16.089864
RTKN2	2.351352	2.157002	1.26871	1.261494	5.316422	1.986491	15.245635	5.780895
RTL1	0	0	0	0	0	0	0	0
RTL10	6.46358	11.486132	5.811657	9.038774	9.196568	21.380537	17.663267	23.837022
RTL5	0	0.487725	0	0	0.200333	1.820939	0	1.857518
RTL6	13.244961	30.834685	17.852932	24.268326	8.588857	37.000774	11.881978	36.155961
RTL8A	0	29.863964	86.388386	59.057494	13.638037	46.43738	0	34.291267
RTL8C	49.470851	0	0	0	0	0	35.588573	0
RTL9	0	0	0.561742	0.045844	0	0.125263	0	0.050165
RTN1	0	0.214858	0	0	0	0	0	0
RTN2	13.491402	9.244182	3.792246	7.12098	6.147439	11.440772	0	3.914476
RTN3	62.419372	50.566038	78.363671	41.743467	123.052003	62.886872	119.406999	66.928846
RTN3P1	0	0	0	0	0	0	0	0
RTN4	263.668428	138.335665	227.15735	125.492012	257.305481	116.049393	271.61219	136.192179
RTN4IP1	0	0	0	0	22.345004	0	0	0
RTN4R	5.934356	12.796732	1.763729	14.388029	2.182252	26.558362	10.043521	18.137798
RTN4RL1	0	0.471646	0	0.067259	0	1.192666	0	1.144433
RTN4RL2	0	3.381643	0	1.539815	0	1.761503	0	0.996269
RTP2	0	0.113316	0	0	0	0	0	0
RTP3	0	0	0	0	0	0	0	0

RTP4	0	1.305146	0	0.926153	10.709858	2.937899	0	2.615288
RTP5	0	0	0	0	0	0	0	0
RTRAF	328.505131	326.627316	372.596262	228.548836	253.490952	173.13724	150.080172	328.362494
RTRAFP1	0	0	0	0	0	0	0	0
RTRAFP2	0	0	0	0	0	0	0	0
RTTN	13.74522	24.971033	25.379943	21.008657	15.70414	9.333637	45.049297	15.511637
RUBCN	21.6189	43.402616	36.237883	43.172254	22.178478	62.552885	9.66623	51.067322
RUBCNL	0	0	0	0.484156	0	0.529987	0	0.288387
RUFY1	24.659834	31.797824	61.02198	30.688653	37.104121	25.943005	16.00977	19.453453
RUFY2	10.204187	6.063731	8.316188	8.549126	15.854228	7.934864	0	13.840453
RUFY3	12.066169	12.18556	5.327981	6.605889	13.309666	7.103565	0	6.768831
RUFY4	0	0	0	0.88841	0	0	0	0
RUNDC1	0	1.737435	0	0.805045	0	0.492533	0	0
RUNDC3A	5.60392	3.785929	6.867398	5.564079	0	1.113056	0	0.466116
RUNDC3B	10.860347	2.582622	4.748457	0.762299	0.270991	0	0	1.410626
RUNX1	30.192045	80.656458	25.600944	92.078955	37.326629	197.085793	98.31542	196.256509
RUNX1T1	0	0	0	0	0	0	0	0
RUNX2	4.547896	7.091348	7.915452	6.814357	14.806828	10.158917	5.457699	7.978336
RUNX3	0	0	0	2.838811	0	0	0	0
RUSC1	30.028522	56.901517	26.806063	50.967331	31.523268	98.188764	44.291611	75.712986
RUSC2	8.765126	22.125881	11.25041	34.83996	6.119644	45.5889	4.575812	28.569875
RUSF1	14.824186	5.577277	26.060144	20.942105	38.973927	8.538807	65.823478	12.354691
RUVBL1	0	13.102436	0.757151	7.879357	13.23526	7.776552	0	6.720297
RUVBL2	81.032502	85.74453	84.47507	116.752432	123.93188	227.743698	46.978846	180.221489
RUVBL2P1	0	0	0	0	0	0	0	0
RWDD1	40.55888	47.946141	40.459012	20.435483	32.799788	16.825333	43.379641	32.374925
RWDD2A	1.758207	4.778292	5.521047	3.647395	3.017687	1.975866	4.918864	1.906957
RWDD2B	28.884004	47.520711	34.990117	37.471667	2.768328	19.059765	14.738497	32.881579
RWDD3	55.54445	77.834441	61.031587	63.009035	9.260057	12.632964	0	16.26438
RWDD4	10.430832	32.33435	24.32462	19.606539	68.090783	22.294739	5.651167	51.26844
RWDD4P1	0	0	0	0	0	0	0	0
RWDD4P2	0	0	0	0	0	0	0	0
RXFP1	0	0	0	0	0	0	0	0

RXFP2	0	0	0	0	0	0	0	0
RXRA	34.73763	67.130482	26.984774	60.561857	27.376956	82.241524	15.781531	63.803845
RXRB	0	3.431616	0	11.633589	27.298812	20.61019	2.832432	12.20712
RXRG	0	0	0	0	0	0.247663	0	0
RXYLT1	19.310261	8.537794	0	18.435381	0	34.488153	56.991905	15.819459
RYK	0	11.521506	0	29.844149	0	25.679689	0	0
RYKP1	0	0	0	0	0	0	0	0
RYR1	2.074546	2.049009	1.307872	2.086083	2.13904	1.099785	1.469148	0.785263
RYR2	2.539014	0.234078	0	0.141295	0	0	0	0.392825
RYR3	0	0	0	0	0	0	0	0.150567
S100A1	0	0	0	0	0	0	0	0
S100A10	1363.93689	1622.58859	938.801475	1479.1248	1324.29947	1211.81286	1337.38044	1521.98299
S100A11	1733.51387	2617.27344	1314.5864	2637.15947	1272.12223	2074.10707	1349.07347	2449.47483
S100A11P1	0	0	0	0	0	0	0	0
S100A11P10	0	0	0	0	0	0	0	0
S100A11P2	0	0	0	0	0	0	0	0
S100A11P5	0	2.587298	0	0.568477	0	0	0	0
S100A11P6	0	0	0	0	0	0	0	0
S100A11P7	0	0	0	0	0	0	0	0
S100A11P8	0	0	0	0	0	0	0	0
S100A11P9	0	0	0	0	0	0	0	0
S100A12	0	0	0	0	0	0	0	0
S100A13	303.717068	422.095254	161.451669	327.800381	133.549857	329.11076	178.921489	261.208327
S100A14	10.548622	1.267911	0	1.953875	246.404443	283.490964	246.861381	222.670891
S100A15A	0	0	0	0	0	0	0	0
S100A16	180.836024	280.470676	137.645987	260.072619	104.336044	367.157679	141.675129	353.812277
S100A2	3391.09955	2412.20061	2427.80494	2282.58751	2041.09435	1753.38535	2053.51482	1773.8492
S100A3	5.644057	1.36372	0	1.608479	35.263915	1.873703	0	3.020804
S100A4	0	4.110893	0	4.663432	19.812562	5.762685	0	7.916975
S100A5	0	0	0	0	0	0	0	0
S100A6	1148.81477	1257.60494	1387.53321	1556.4847	1609.17856	1631.74626	1029.66108	1230.78168
S100A7	0	0	0	0	0	1.129784	0	1.082476
S100A7A	0	0	0	0	0	0.543795	0	0.061783

S100A7P1	0	0	0	0	0	0	0	0
S100A8	0	0	0	0	3.547022	10.371148	0	6.508948
S100A9	16.165821	1.581113	0	3.979743	6.316546	34.414165	0	18.205187
S100B	0	0	0	0	0	0	0	0
S100G	0	0	0	0	0	0	0	0
S100P	0	0	9.268315	6.054572	0	0.945358	0	1.383247
S100PBP	13.973567	52.146962	26.564451	47.070121	19.663535	75.061052	89.305933	52.732022
S100Z	0	0	0	0	0	0	0	0
S1PR1	13.163785	10.688439	8.75065	3.192845	0.347312	5.994974	6.38908	8.226203
S1PR2	0	3.39111	4.086497	4.71331	7.457679	5.140498	0	4.575784
S1PR3	5.239485	6.45181	0.68247	2.951866	1.003837	2.471162	0	3.770479
S1PR4	0	0	0	0	0	0	0	0
S1PR5	0	7.071921	0	5.411659	1.6563	3.37365	0	1.978462
SAA1	0	2.771147	47.379661	128.202782	217.969392	188.095469	111.387538	99.200935
SAA2	0	0.171443	22.028799	7.422932	5.209921	14.894215	18.899296	9.97364
SAA2-SAA4	0	0	0	0.841648	0	0.290787	0	0
SAA3P	0	0	0	0	0	0	0	0
SAA4	0	0	12.232369	1.181441	0	0	0	1.170827
SAAL1	31.567358	30.905684	26.917828	28.021465	80.593843	22.556253	59.14005	35.786683
SAC3D1	11.701988	3.589628	0	5.534877	9.355508	23.340897	0	13.069711
SACM1L	35.380203	20.321539	19.834169	10.788258	13.013883	12.83404	34.21673	22.372087
SACS	5.507747	10.933906	6.455649	6.583212	7.269207	9.049033	14.157744	19.637325
SAE1	47.811677	26.654767	0	22.9304	68.029091	22.532941	0	54.524402
SAE1P1	0	0	0	0	0	0	0	0
SAFB	51.767018	78.735626	53.762301	89.282871	77.34912	104.545627	106.358273	79.914128
SAFB2	18.274521	18.894887	14.290174	28.99898	12.180529	26.853775	135.745535	24.420234
SAG	0	0	0	0	0	0	0	0
SAGE2P	0	0	0	0	0	0	0	0
SAGE3P	0	0	0	0	0	0	0	0
SAGE4P	0	0	0	0	0	0	0	0
SALL1	0	0	0	0.501878	0	0	0	0
SALL1P1	0	0	0	0	0	0	0	0
SALL2	2.695463	3.602135	7.500589	4.231718	4.100743	5.397581	0	2.812264

SALL3	0	0	0	0	0	0	0	0
SALL4	9.354306	4.918442	2.254097	9.162191	0.165692	2.993414	0	2.311876
SALL4P1	0	0	0	0	0	0	0	0
SALL4P2	0	0	0	0	0	0	0	0
SALL4P5	0	0	0	0	0	0	0	0
SALL4P6	0	0	0	0	0	0	0	0
SALL4P7	0	0	0	0	0	0	0	0
SAMD1	0	32.255199	0	25.250453	0	77.064032	101.550475	48.127651
SAMD10	4.929296	2.680777	1.412281	2.47775	4.16766	4.561839	0	2.620422
SAMD11	0	0	1.815557	1.333081	0	0	0	1.187717
SAMD11P1	0	0	0	0	0	0	0	0
SAMD12	13.319216	39.801282	16.075691	66.95957	10.431015	60.542475	4.63616	61.928203
SAMD13	2.584822	1.081957	10.176636	1.499223	0	0	0	1.033352
SAMD14	0	0	0	1.500763	0	0.124359	0	0
SAMD15	0	0	0	0	0	0	0	0
SAMD3	0	0.209816	0	0.253799	0	0	0	0
SAMD4A	11.850468	13.723374	14.064386	18.650171	14.731981	28.489076	75.225476	28.947705
SAMD4B	60.015485	80.941061	30.775111	83.42383	43.392851	151.614065	96.517411	112.048019
SAMD5	8.210315	6.907351	9.22693	5.082882	0.271178	0.257348	0	0.678962
SAMD7	0	0	0	0	0	0	0	0
SAMD8	23.709378	30.686845	24.562349	20.727493	13.18365	13.550088	15.306363	20.494329
SAMD9	35.220397	31.871732	28.675879	13.64336	82.145133	15.327038	47.793408	46.329688
SAMD9L	19.821792	7.888187	5.591241	5.453221	23.649238	16.149256	11.916151	39.374856
SAMHD1	18.225784	16.685001	21.85507	12.865691	56.341496	18.669346	20.520956	23.212021
SAMM50	27.164637	39.69553	44.686667	27.560203	47.345371	26.465272	41.401649	49.989514
SAMM50P1	0	0	0	0	0	0	0	0
SAMSN1	0	0	0	0.063546	0	0	0	0
SANBR	8.751349	12.500737	12.896374	7.959094	3.239909	8.295095	8.15966	10.703838
SAP130	16.241246	1.623553	0	7.771851	25.919167	14.653638	27.711972	11.924449
SAP18	176.985857	194.931562	58.808331	168.379135	179.058208	114.127971	181.642238	98.554397
SAP18P1	0	0	0	0	0	0	0	0
SAP18P2	0	0	0	0	0	0	0	0
SAP18P3	0	0	0	0	0	0	0	0

SAP25	4.823691	0.392495	0	0	0	0	0	0
SAP30	17.253823	24.855703	6.102783	11.949531	18.137321	19.603282	16.549737	26.048656
SAP30BP	42.158972	157.824952	49.800102	188.793153	90.319255	146.060496	33.407256	116.300522
SAP30L	5.252007	47.757976	21.738028	49.766581	4.024378	45.134556	0.128023	48.199587
SAP30P1	0	0	0	0	0	0	0	0
SAPCD1	0	0	0	0	0	0.769851	0	1.113072
SAPCD2	13.891311	23.637569	7.789928	16.900584	22.008682	56.759212	34.218565	45.11502
SAPCD2P1	0	0	0	0	0	0	0	0
SAPCD2P2	0	0	0	0	0	0	0	0
SAPCD2P3	0	0	0	0	0	0	0	0
SAPCD2P4	0	0	0	0	0	0	0	0
SAR1A	22.550174	112.947324	27.270129	101.905522	85.517899	80.338777	35.79346	119.56959
SAR1AP1	0	0	0	0	0	0	0	0
SAR1AP2	0	0	0	0	0	0	0	0
SAR1AP3	0	0	0	0	0	0	0	0
SAR1AP4	0	0	0	0	0	0	0	0
SAR1B	88.302697	74.888334	46.607357	55.223349	70.946411	23.403502	35.675622	45.980227
SARAF	33.843904	78.25645	0	54.518196	98.348225	55.338159	99.189785	45.691641
SARDH	0	0	0	0	0	0.150137	0	0.502093
SARM1	4.868801	6.776786	5.058141	7.550927	20.236126	11.072256	50.401648	10.582616
SARNP	66.179785	71.441019	62.08422	80.847316	94.121056	61.180846	42.59522	52.33745
SARS1	289.560164	490.356553	327.489819	684.625451	114.808467	203.547664	54.585666	143.486908
SARS2	16.991374	5.853724	34.400154	3.608751	36.108856	0.509474	26.234399	2.487186
SART1	104.527856	88.795827	66.135622	138.761731	62.659935	142.839016	54.199358	95.926089
SART3	46.515746	54.679403	21.64393	59.816795	47.428333	93.138816	177.816027	68.804958
SASH1	3.032211	3.002442	7.583274	3.854116	5.050438	34.169193	7.817774	14.883432
SASH3	0	0	0	0	0	0	0	0
SASS6	17.203026	13.845224	10.805204	5.35614	14.536534	3.136854	9.835942	7.702382
SAT1	4.483156	14.92292	43.939634	20.965466	27.505713	17.372382	0	21.760485
SAT2	19.938805	17.935607	0	30.752735	3.665328	39.509282	0	32.74582
SATB1	15.92499	7.505723	3.968961	6.449096	0.233038	0.422981	0	0.433998
SATB2	3.867675	8.822584	4.243035	10.158411	4.089484	6.272554	7.736202	2.677791
SATL1	0	0.306229	0	0.440635	0	0.089346	0	0.167013

SAV1	15.303451	10.146721	1.528146	8.373076	157.986705	20.30498	0	25.40506
SAXO1	0	0	0	0.049508	0	0.018716	0	0
SAXO2	0	2.512817	0	1.973401	0	1.248501	0	0.488743
SAXO3	0	2.326912	8.655649	3.487342	0	0.692395	0	0.854943
SAXO4	0	0	0	0.18025	0	0	0	0
SAXO5	0	1.181859	6.706906	2.157017	0	0.206223	0	0
SAYSD1	0	7.425896	6.137063	4.107239	0.937875	5.959975	0	9.142245
SBDS	43.550111	58.697615	58.907106	28.412372	55.036979	19.221456	0	81.689579
SBF1	1.112463	3.819203	0.876782	4.155785	12.057668	7.226078	0.981257	7.552824
SBF1P1	0	0	0	0	0	0	0	0
SBF1P2	0	0	0	0	0	0	0	0
SBF2	0	2.608826	0	0	35.883748	0	11.085123	2.729204
SBK1	3.937955	10.592841	5.306345	13.889942	3.120772	9.168719	0	4.318906
SBK2	0	0	0	0	0	0	0	0
SBK3	0	0.483793	0	0.419928	0	0	0	0
SBNO1	24.783486	24.074433	28.787056	18.733892	14.584242	8.717044	14.630977	18.381558
SBNO2	32.50637	41.077682	26.156569	56.514164	51.983253	89.144083	93.379637	54.678125
SBSN	0	0	0	0	0	0	0	0.499318
SBSPON	0	0	0	0.021886	0	0	0	0
SC4MOP	0	0	0	0	0	0	0	0
SC5D	59.122827	14.717742	13.46382	7.161929	80.19782	5.676147	103.950862	19.261458
SCAF1	23.414759	23.108255	23.121134	28.701363	25.147706	70.489293	120.413268	44.445789
SCAF11	23.954813	61.190075	26.385655	70.752531	79.454482	90.800346	91.796626	98.482118
SCAF4	0	10.556689	33.71373	31.014544	45.635208	67.45538	28.688012	42.172585
SCAF8	21.349178	0	0	0.742867	24.49668	0.568783	25.006478	17.475784
SCAI	0	5.221107	7.011853	3.119669	3.790337	2.297136	53.914675	8.428465
SCAMP1	72.681183	40.599735	82.839189	41.922531	26.917853	20.129013	51.503469	29.659908
SCAMP2	83.839978	49.254748	52.184644	55.752518	47.496958	50.017482	31.66976	38.700557
SCAMP3	46.678073	67.97979	47.944841	89.251691	25.428843	82.652189	45.605624	64.894555
SCAMP4	19.041646	47.017107	11.794482	32.790213	65.471313	46.192651	32.146267	36.094543
SCAMP5	6.90479	10.329477	8.844454	9.781372	35.188697	15.736679	6.475159	11.710716
SCAND1	0	6.847872	6.479667	7.094015	0	8.306737	0	4.748992
SCAND2P	0	0	0	0	0	0	0	0

SCAND3	0	5.826673	5.525483	6.046544	0	2.058512	0	5.966446
SCAND3P1	0	0	0	0	0	0	0	0
SCAP	37.031198	28.802199	13.329736	42.47332	15.338291	48.801215	0	44.956715
SCAPER	4.180845	10.129297	8.761416	10.185382	2.640178	10.419032	26.450878	9.854487
SCARA3	2.624137	9.808905	1.569601	8.710241	11.779578	58.814349	0	34.847953
SCARA5	0	0.071279	0	0	0	0	0	0
SCARB1	6.012471	18.009362	16.669588	25.546289	21.222502	27.311447	128.751779	30.792433
SCARB2	59.25186	66.297403	55.909891	84.63497	114.143116	58.557088	52.958209	49.840504
SCARF1	0	0.110179	1.158423	0.194713	0	0.872904	0	0.601692
SCARF2	0	0	0	0	0	7.052684	0	12.317496
SCART1	0	0.089799	3.641792	0.157678	0	0	0	0.133969
SCCPDH	49.91826	33.242448	35.968337	20.175361	44.585157	22.975715	30.039926	45.118068
SCD	594.483328	633.862175	385.214487	391.642319	540.759163	406.628233	316.480141	397.631612
SCD5	11.03465	19.809769	16.161536	15.267734	5.242537	6.821623	0	5.347343
SCDP1	0	0	0	0	0	0	0	0
SCEL	1.08785	1.76329	0.97485	1.046668	1.148556	1.049469	0	0.817569
SCFD1	67.335068	65.545312	71.342845	49.353152	29.476079	11.496143	102.002279	43.53698
SCFD2	12.376306	3.828591	10.433468	4.227349	0.558691	0.859483	0	4.598894
SCG2	1.400612	0.238141	0	0.06816	0	0	0	0.112829
SCG3	0	0	0	0.025818	0	0	0	0
SCG5	0	0.527459	0	0.215025	0	1.111505	0	6.630624
SCGB1A1	0	0	0	0	0	0	0	0
SCGB1C2	0	0	0	0	0	0	0	0
SCGB1D1	0	0	0	0	0	0	0	0
SCGB1D2	0	0	0	0	0	0	0	0
SCGB1D5P	0	0	0	0	0	0	0	0
SCGB2A1	0	0	0	0	0	0	0	0
SCGB2A2	0	0	0	0	0	0	0	0
SCGB2B2	0	0.816415	0	0.161793	0	2.569561	0	0.852468
SCGB2B3P	0	0	0	0	0	0	0	0
SCGB3A1	0	0	0	1.233473	0	0	0	0
SCGB3A2	0	0	0	0	0	0	0	0
SCGN	0	0	0	0	0	0	0	0

SCHIP1	11.663438	38.110391	18.129714	42.144924	2.865477	64.24549	49.831942	67.54731
SCIMP	0	0	0	0	0	0	0	0
SCIN	2.64269	0.325155	0	0.888283	0	0	0	0.0532
SCLT1	12.842274	24.444062	8.080136	13.516767	21.275285	13.185444	18.650013	33.681426
SCLY	5.451917	13.329214	17.219169	17.802088	45.544155	39.290249	47.243475	41.92533
SCMH1	0	21.786262	32.392129	14.264277	9.537168	28.410043	0	21.771094
SCML1	6.252851	8.212665	6.784616	8.035008	7.998908	12.22428	35.020936	22.892293
SCML2	1.401857	2.162287	2.508008	0.947933	6.744783	3.213134	13.145783	3.435375
SCML2P1	0	0	0	0	0	0	0	0
SCML2P2	0	2.738648	0	1.030706	0	0	0	0.738934
SCML4	0	0	0	0	0	0	0	0
SCN10A	0	0	0	0	0	0	0	0
SCN11A	0	0	0	0	0	0	0	0
SCN1A	0	0	0	0	0	0	0	0
SCN1B	0	1.091126	0	0.360643	4.659976	0.405325	0	1.425113
SCN2A	0	0.327797	0	0.24255	0	0.019822	0	0.029039
SCN2B	0	0	0	0	0	0	0	0
SCN3A	0	0.286239	0	0.099283	0	0	0	0
SCN3B	0	0.27737	0	0.374267	0	0.060683	0	0.266462
SCN4A	0	0	0	0.020241	0	0	0	0
SCN4B	3.330627	0.226122	15.676746	4.07845	5.71699	0	0	2.561855
SCN5A	2.630116	0.110027	0	0.450674	1.311608	0.067925	0	0.150852
SCN7A	0	0	0	0	0	0	0	0
SCN8A	4.040827	2.02345	1.841081	0.715605	35.471632	0.571936	0	1.305717
SCN9A	0	0.476303	0	0.791411	0	0.077939	0	0.098993
SCNM1	44.148799	53.392008	34.145878	77.117093	10.85284	42.130361	42.733424	47.142447
SCNN1A	6.62079	6.454625	9.90202	13.279923	24.709815	9.611487	3.873517	6.627703
SCNN1B	0	0.652041	4.870481	0.18634	0	0	0	0
SCNN1D	0	0	0	0.23466	0	0.417664	0	0.153936
SCNN1G	0	0	2.574812	0.46694	0	0	0	0
SCO1	31.318713	44.412578	31.676233	32.920331	25.440449	45.977066	25.691203	42.712242
SCO2	5.848454	0.7677	0	0	0	0.523674	0	0.618452
SCOC	0	8.403307	0	0.319715	0	0	0	0

SCOCP1	0	0	0	0	0	0	0	0
SCP2	331.186746	304.957289	280.183245	248.488116	131.63535	60.808689	87.012022	84.833695
SCPEP1	9.565251	7.963395	0	13.557255	32.865935	4.81377	11.376874	0
SCRG1	0	0	0	0	0	0	0	0
SCRIB	12.161247	56.869633	39.306679	45.385071	52.462619	84.23476	32.559895	57.530513
SCRN1	62.163525	123.185544	59.763856	119.401166	110.180809	141.394172	101.16721	139.473128
SCRN2	16.811587	10.201402	16.599849	13.976909	18.978381	16.621912	1.005647	6.223113
SCRN3	16.045497	35.577497	28.083164	28.564426	20.220994	32.140395	39.033895	60.693061
SCRT1	0	0	0	0	0	0	0	0
SCRT2	0	0	0	0	0	0	0	0
SCT	0	0	0	0	0	0	0	0
SCTR	0	0	0	0	0	0	0	0
SCUBE1	0	0.014138	0	0.048175	0	0	0	0
SCUBE2	0	0	0	0	0	0	0	0.092944
SCUBE3	0.825612	0.355534	1.485394	0.727356	0	0.020726	0	0.016543
SCX	0	0.141516	0	0.164378	0	1.659752	0	0.417239
SCYGR1	0	0	0	0	0	0	0	0
SCYGR10	0	0	0	0	0	0	0	0
SCYGR2	0	0	0	0	0	0	0	0
SCYGR3	0	0	0	0	0	0	0	0
SCYGR5	0	0	0	0	0	0	0	0
SCYGR6	0	0	0	0	0	0	0	0
SCYGR7	0	0	0	0	0	0	0	0
SCYGR9	0	0	0	0	0	0	0	0
SCYL1	68.536778	86.207708	54.071224	89.792146	84.054869	78.414344	62.118942	58.565885
SCYL2	74.786202	32.814979	38.906857	31.914399	52.965454	50.994091	74.816711	69.206362
SCYL2P1	0	0	0	0.029741	0	0	0	0.44542
SCYL3	7.377024	5.098097	5.552535	4.495778	1.404279	2.781129	0	4.685501
SDAD1	30.184547	33.769785	27.044499	28.547529	18.720949	19.249267	20.682918	31.653807
SDAD1P1	0	0	0	0	0	0	0	0
SDAD1P2	0	0	0	0	0	0	0	0
SDAD1P3	0	0	0	0	0	0	0	0
SDAD1P4	0	0	0	0	0	0	0	0

SDC1	121.208408	183.090513	116.03272	232.578159	106.044606	280.226416	71.940994	152.746185
SDC2	2.252346	5.724387	1.809433	8.917729	0	6.482703	0	2.300724
SDC3	4.980941	16.847894	10.069564	13.139967	5.098943	9.895079	0	6.19453
SDC4	194.612049	158.490366	258.961691	217.855231	246.813148	180.938189	155.238443	153.043044
SDC4P	0	0	0	0	0	0	0	0
SDCBP	73.592021	96.17294	96.180455	74.52038	49.38348	31.90307	50.325232	82.468037
SDCBP2	0	0.487683	0	2.495875	0	2.152071	0	1.905748
SDCBP2P1	0	0	0	0	0	0	0	0
SDCBPP1	0	0	0	0	0	0	0	0
SDCBPP2	0	0	0	0	0	0	0	0
SDCBPP3	0	0	0	0	0	0	0	0
SDCCAG8	18.134819	62.781895	23.598898	41.939483	9.450099	27.109998	1.444915	21.058989
SDE2	20.717901	11.996088	13.387083	14.082521	23.197593	8.531431	24.199546	9.88455
SDF2	71.386156	88.187729	30.730589	95.687526	47.402117	55.348972	43.047569	66.488164
SDF2L1	14.741792	87.420016	25.857379	63.181439	78.556159	80.088118	1.769695	76.590116
SDF4	0	0	0	0.089726	0	3.8243	54.030286	4.435513
SDHA	71.100847	96.678752	116.219502	119.389465	70.679389	100.464081	110.288229	66.517866
SDHAF2	29.031001	18.785659	12.067545	11.221618	0	28.093609	22.187846	30.291766
SDHAF3	8.337	29.295309	0	12.414574	12.050295	8.149486	0	18.278881
SDHAF4	18.922931	6.552616	19.181797	12.687649	8.292828	2.30439	0	4.589345
SDHAP1	2.086607	6.036524	6.472762	8.5006	0	6.957924	0.423253	7.873667
SDHAP2	1.875019	8.856983	5.111871	13.460073	5.486705	14.541617	0	17.315696
SDHAP3	0	0	5.462459	0.901044	0	0	0	0
SDHAP4	0	0	0	0	0	0	0	0
SDHB	0	0	0	0.139078	149.485389	0	197.299897	0
SDHBP1	0	0	0	0	0	0	0	0
SDHC	130.095182	48.376699	80.301698	65.734366	124.154494	52.490922	91.136806	41.866882
SDHCP1	0	0	0	0	0	0	0	0
SDHCP2	0	0	0	0	0	0	0	0
SDHCP3	0	0	0	0	0	0	0	0
SDHCP4	0	0	0	0	0	0	0	0
SDHD	128.215999	39.090493	99.305298	28.737781	125.466974	25.860352	191.130903	53.841336
SDHDP1	0	0	0	0	0	0	0	0

SDHDP2	0	0	0	0	0	0	0	0
SDHDP3	0	0	0	0	0	0	0	0
SDHDP4	0	0	0	0	0	0	0	0
SDHDP5	0	0	0	0	0	0	0	0
SDHDP6	0	0	0	0	0	0	0	0
SDK1	8.787871	5.679937	0	7.082175	2.356727	0.245822	0	0.169787
SDK2	2.89291	4.572356	2.34379	5.517824	0.323412	0.322988	0	0.307417
SDR16C5	0	0	0	0.485946	0	0.379968	0	0.542206
SDR16C6P	0	0	0	0	0	0	0	0
SDR39U1	27.783173	14.787547	0	18.611695	30.126304	25.936606	41.65835	10.099076
SDR42E1	39.982186	80.841347	31.776413	72.468862	24.847858	84.280349	3.114311	93.237518
SDR42E1P1	0	0	0	0	0	0	0	0
SDR42E1P2	0	0	0	0	0	0	0	0
SDR42E1P3	0	0	0	0.917064	0	0	0	0
SDR42E1P4	0	0	0	0	0	0	0	0
SDR42E1P5	0	0	0	0	0	0	0	0
SDR9C7	0	0	0	0.095236	0	0	0	0
SDS	0	0	0	0	0	0	0	0
SDSL	0	1.523715	11.546706	2.237985	0	4.256509	0	4.741667
SEC11A	245.348021	150.209204	256.829763	136.646844	136.77181	58.0755	196.175671	122.708824
SEC11B	0	0	0	0	0	0	0	0
SEC11C	32.545588	45.52149	27.261824	27.581373	32.123608	11.24004	39.066375	23.832279
SEC13	72.377955	103.826309	107.529729	119.029087	31.75758	61.902971	13.561771	67.565117
SEC13P1	0	0	0	0	0	0	0	0
SEC14L1	39.250576	93.482638	56.713724	73.266152	90.691628	77.580461	108.940525	80.097691
SEC14L1P1	1.578019	1.739942	0	1.419232	9.155122	3.910353	0	5.153074
SEC14L2	54.979581	63.719994	59.2519	89.821417	50.32378	44.780034	47.862454	28.704664
SEC14L3	0	0	0	0	0	0	0	0
SEC14L4	0	0	0	0	0	0	0	0
SEC14L5	0.503392	0.368077	0.452604	0.14775	0	0.176739	2.086282	0.332479
SEC16A	46.005671	46.099595	49.033177	62.625483	48.988007	67.216672	28.807685	51.18867
SEC16B	0	0.678517	0	1.003319	0	0.101625	0	0.207866
SEC1P	0	0.223059	9.093789	0.031901	0	0.195404	0	0

SEC22A	43.434476	6.113366	28.339687	7.795917	21.464622	1.955018	103.263569	13.601482
SEC22B	1.540234	0.838037	0	0.341426	0	0.229103	0	0.690066
SEC22B2P	0	0	0	1.57433	0	0	0	0
SEC22B3P	0	3.883941	0	0	0	0	0	0
SEC22B4P	0	0	0	1.155312	0	0	0	0
SEC22C	53.82688	57.94775	36.735519	45.310902	48.264037	36.77356	25.17001	59.590545
SEC23A	112.251523	192.939636	180.672889	217.420839	82.915921	113.508188	79.625897	163.898468
SEC23B	33.886905	37.668755	41.942165	58.324519	52.452369	54.84513	88.651593	69.386526
SEC23IP	110.72679	86.839828	33.666853	86.826478	327.005779	97.435222	134.960929	105.678701
SEC24A	27.736629	27.303835	31.743797	30.327017	10.959133	12.650083	10.029972	19.852057
SEC24AP1	0	0	0	0	0	0	0	0
SEC24B	12.925919	13.072741	29.710041	10.397239	24.192217	5.331882	11.939914	11.634895
SEC24C	42.748764	34.899058	48.929339	42.147437	36.59904	36.433434	45.920233	25.635859
SEC24D	20.895616	33.959053	21.423498	57.200091	36.203662	25.769212	49.991851	22.773432
SEC31A	86.533084	100.378278	98.78269	163.301597	33.007657	78.734948	123.050481	65.160646
SEC31B	1.012053	0	0	0.497815	0	0	0	0
SEC61A1	95.379738	98.468283	98.935866	106.824628	62.086518	73.680568	56.596246	82.636074
SEC61A2	0	0.129339	0	0.710221	0	0.274838	0	0.612878
SEC61B	223.36141	157.011677	156.785437	98.981716	150.796088	62.481487	92.806661	123.931309
SEC61G	283.685562	159.710149	200.519244	78.383507	374.258265	75.639868	271.190442	243.063008
SEC61GP1	0	0	0	0	0	0	0	0
SEC62	122.487251	144.892042	123.61323	127.559871	124.25774	72.950678	133.646356	121.50606
SEC63	51.82742	89.758001	115.200019	24.774424	113.94912	11.674735	188.405321	31.206493
SEC63P1	0	0	0	0	0	0	0	0
SEC63P2	0	0	0	0	0	0	0	0
SECISBP2	0.637775	25.399569	0	11.568954	12.67021	13.953589	28.786815	15.928567
SECISBP2L	13.220364	24.618005	19.936018	23.641928	20.304907	16.204684	25.329444	39.008546
SECTM1	10.446728	1.552506	0	4.887081	10.944023	5.000736	39.192998	2.55798
SEH1L	57.877012	69.697822	46.636714	71.56291	32.743806	74.103252	31.548056	92.950203
SEL1L	36.264793	104.809468	53.505369	195.706513	32.844696	91.109435	88.11433	87.122594
SEL1L2	0	0	0	0	0	0	0	0
SEL1L3	20.538977	29.641474	25.321206	38.88816	26.225188	44.375983	71.783094	34.178979
SELE	0	0	0	0	0	0	0	0

SELENBP1	4.713523	2.638038	0	1.92795	0	2.535784	0	2.452076
SELENOF	209.602117	124.330922	241.651817	115.074673	102.086288	37.766437	171.771955	79.155086
SELENOH	39.509539	43.593675	85.930039	65.692863	78.476758	74.963435	0	68.427203
SELENOI	33.989003	11.504406	29.858311	11.139182	10.885116	2.130744	8.778772	6.02877
SELENOK	26.783946	31.324739	21.986151	25.914661	29.093893	27.17455	44.980555	10.469084
SELENOKP1	0	0	0	0	0	0	0	0
SELENOKP2	0	0	0	0	0	0	0	0
SELENOKP3	0	0	0	0	0	0	0	0
SELENOM	30.871427	31.487064	5.599447	46.092307	2.574414	23.56166	0	14.864099
SELENON	15.464661	25.514528	31.606738	24.967149	47.952245	44.394696	33.519031	30.121659
SELENOO	6.005403	4.760393	6.711237	4.414445	6.731655	9.567387	0	9.201039
SELENOP	0	0.558226	0	0.674876	0	0	0	0.446293
SELENOS	44.343113	99.60316	37.298869	83.194023	71.798848	71.582537	27.09635	97.984583
SELENOT	47.749478	52.409577	64.160304	22.893067	55.392999	11.097724	64.600228	71.403855
SELENOTP1	0	0	0	0	0	0	0	0
SELENOTP2	0	0	0	0	0	0	0	0
SELENOV	0	2.419118	0	0.633657	0	0	0	1.41442
SELENOW	53.436807	67.458613	13.811674	69.583232	63.800717	112.377099	89.308783	104.81305
SELENOWP1	0	0	0	0	0	0	0	0
SELL	0	0.9756	0	1.098448	0	0.67511	0	1.127416
SELP	0	0	0	0	0	0	0	0
SELPLG	0	0	0	0.267033	5.808301	0	0	0.445932
SEM1	72.810995	291.888778	108.054784	337.227325	98.615559	393.934492	289.15963	260.924512
SEM1P1	0	0	0	0	0	0	0	0
SEMA3A	6.360008	12.558612	33.484579	23.012503	2.941534	6.161043	7.756548	15.009343
SEMA3B	3.485104	1.355649	5.487904	2.032628	0	0.927854	0	0.601257
SEMA3C	61.362939	64.112495	33.810271	75.694335	20.631803	53.877985	33.459438	48.464351
SEMA3D	0	1.468389	0	0.30032	2.399611	0.951374	0	4.888229
SEMA3E	9.892196	12.283456	1.264043	15.527068	0.745566	5.317416	0	6.844715
SEMA3F	14.295151	24.446825	15.997946	37.150898	42.59434	103.571716	50.810704	73.037302
SEMA3G	0	0	0	0.149914	0	0	0	0.699375
SEMA4A	17.427247	7.777798	0	9.228126	0	11.848363	10.127814	5.178912
SEMA4B	71.846041	113.528487	31.064911	155.984233	0	95.301653	100.093866	57.464261

SEMA4C	6.36131	6.977673	6.807935	13.051907	10.030378	15.506399	0	6.860393
SEMA4D	0	24.787614	0	21.965818	6.256617	36.5678	17.832992	56.310906
SEMA4F	7.599912	4.864745	4.669385	6.55064	0	9.20795	14.176399	5.429904
SEMA4G	1.488174	0.151993	1.656995	1.049873	0	1.419302	35.134746	0
SEMA5A	7.692507	10.151311	15.67007	23.843615	1.656147	8.454555	2.860427	7.728008
SEMA5B	0	0.097457	4.129717	0.445995	0	0	0	0.081077
SEMA6A	0	2.231402	0	4.340578	0	0.20784	0	0
SEMA6B	2.570633	8.141248	12.315595	7.081768	1.131351	3.933212	0	1.72674
SEMA6C	0	0	0	0	0	1.497351	0	4.53176
SEMA6D	0	0.313542	0	0.029715	0	0	0	0.471692
SEMA7A	15.222274	13.20168	14.16435	11.537916	7.057238	10.141393	6.487008	9.775679
SEMG1	0	0	0	0	0	0	0	0
SENP1	37.742617	23.133124	0	17.963915	46.883572	4.467204	6.286617	17.454822
SENP2	116.656357	235.974116	78.754005	216.990611	198.906547	157.485207	71.697033	156.879939
SENP3	0	43.346985	30.7712	47.590292	25.07072	115.029662	32.091741	91.205128
SENP3-EIF4A	0	0	0	0.863901	0	0.795044	0	0
SENP5	64.783601	163.281678	40.470148	127.967057	78.647961	109.07437	122.104859	158.440825
SENP6	36.382	75.763908	38.42448	38.552382	41.998933	35.751165	48.447999	82.478286
SENP7	6.123438	3.410224	3.329208	2.04542	0.736875	0.754187	8.190092	2.227526
SENP8	0.789089	2.858815	7.760361	1.915279	0	0.938963	0	2.079567
SEPHS1	17.882511	56.024711	34.239611	52.654445	13.287221	54.573176	35.852453	59.997597
SEPHS1P1	0	0	0	0	0	0	0	0
SEPHS1P2	0	0	0	0	0	0	0	0
SEPHS1P4	0	0	0	0	0	0	0	0
SEPHS1P6	0	0	0	0	0	0	0	0
SEPHS1P7	0	0	0	0	0	0	0	0
SEPHS2P1	0	0	0	0	0	0	0	0
SEPSECS	4.894302	1.654626	0.532484	2.196884	2.427422	1.061742	9.862751	2.664246
SEPTIN1	0	1.079921	0	0.675628	0	0	0	1.401436
SEPTIN10	56.834033	65.424589	54.811306	52.694246	79.153566	63.054275	76.037434	77.462528
SEPTIN10P1	0	0	0	0	0	0	0	0
SEPTIN11	51.148507	132.492464	55.568765	79.423593	42.073605	120.639057	94.000565	45.732114
SEPTIN12	0	0	0	0	0	0	0	0

SEPTIN14	0	0	0	0.043506	0	0	0	0
SEPTIN14P1	0	0	0	0	0	0	0	0
SEPTIN14P12	0	0.652041	0	0	0	0	0	1.047551
SEPTIN14P17	0	0	0	0	0	0	0	0
SEPTIN14P19	0	7.745695	0	9.335611	0	9.785152	0	0
SEPTIN14P2	0	13.565426	0	1.801763	0	0	0	0
SEPTIN14P21	0	0	0	0	0	0	0	0
SEPTIN14P22	0	0	0	0	0	0	0	0
SEPTIN14P24	0	0	0	0	0	0	0	0
SEPTIN14P4	0	0	0	0	0	0	0	0
SEPTIN14P6	0	0.613294	0	0.408981	2.215224	1.313005	0	0
SEPTIN14P8	0	0	0	0	0	0	223.128119	0
SEPTIN2	156.675167	411.142624	114.625452	360.537305	317.618799	478.490996	387.312984	764.38528
SEPTIN2P1	0	0	0	0	0	0	0	0
SEPTIN3	0	0	0	0	0	1.018523	0	0
SEPTIN4	0	2.232528	2.375518	0.695651	0	0.552069	0	0.291356
SEPTIN5	1.760464	3.953099	18.087799	6.97316	18.585856	10.163741	0	3.791192
SEPTIN6	15.432823	18.741061	18.71771	15.211717	13.000696	15.417684	25.451452	16.103669
SEPTIN7	135.675887	155.501808	112.070164	149.969436	292.437216	249.15403	254.591735	227.394546
SEPTIN7P1	0	0	0	0	0	0	0	0
SEPTIN7P10	0	0	0	0	0	0	0	0
SEPTIN7P12	0	0	0	0	0	0	0	0
SEPTIN7P13	0	0	0	0	0	0	0	0
SEPTIN7P14	0	0	0	0	0	0	0	0
SEPTIN7P15	0	0	0	0	0	0	0	0
SEPTIN7P2	0	0	0	0	0	0	0	2.866378
SEPTIN7P3	0	0	0	0	0	0	0	0
SEPTIN7P4	0	0	0	0	0	0	0	0
SEPTIN7P5	0	0	0	0	0	0	0	0
SEPTIN7P6	0	0	0	0	0	0	0	0
SEPTIN7P7	0	0	0	0	0	0	0	0
SEPTIN7P8	0	0	0	0	0	0	0	0
SEPTIN7P9	0	0	0	0	0	0	0	0

SEPTIN8	35.184573	49.851153	36.295576	51.507711	41.893561	80.133112	20.090072	58.613319
SEPTIN9	264.42741	280.48873	377.107445	333.948668	187.136726	402.067476	299.351398	226.633185
SERAC1	11.317941	5.301954	4.550559	3.869691	7.172691	2.486296	0	1.921358
SERBP1	499.852885	819.810782	407.150809	959.556621	276.18395	652.110552	327.023373	476.817709
SERBP1P1	0	0.302296	0	0.318297	0	0.983269	0	0.134173
SERBP1P2	0	0	0	0	0	0	0	0
SERBP1P3	0	0	0	0.240191	1.320415	0	0	0
SERBP1P4	0	0	0	0	0	0	0	0
SERBP1P5	0	0	0	0	0	0	0	0.145885
SERBP1P6	4.296535	0.890213	0	0.751512	0	0	0	0
SERF1A	4.74014	0	2.224853	5.15879	22.249268	6.074226	0.111795	4.65828
SERF1AP1	0	0	0	0	0	0	0	0
SERF1B	51.854241	57.603502	11.332072	61.979612	15.980881	123.806211	92.650709	95.737369
SERF2	454.243695	540.261289	387.243546	648.627394	266.926633	724.512663	86.068358	509.479126
SERGEF	27.263427	41.720717	33.843132	28.159618	99.936229	43.37952	30.982104	35.897512
SERHL	0	0	0	0	0	0	0	0
SERHL2	0	2.276785	0	2.776728	0	0	0	0.792142
SERINC1	36.43363	15.416388	66.264541	17.215467	42.427832	7.690594	22.915644	16.071968
SERINC2	154.254551	124.498875	210.260873	136.479406	70.818572	82.887102	32.881368	66.188045
SERINC3	58.363696	45.111746	84.649638	53.11944	59.491075	21.420668	60.008807	31.275853
SERINC4	0	0	0	0	0	0	0	0
SERINC5	16.100836	20.660734	28.500411	14.344453	29.515125	10.097895	11.03975	18.342014
SERP1	71.14537	141.67177	84.757474	128.626716	55.395004	214.008635	76.684821	200.180697
SERP2	12.147711	4.325188	0	4.133938	0	7.422917	0	6.779337
SERPINA1	12.109776	6.67834	3.902872	7.509334	1.310492	7.536755	27.001743	1.05311
SERPINA10	0	0	0	0	0	0	0	0
SERPINA11	0	0	0	0	0	0	0	0
SERPINA12	0	0	0	0	0	0	0	0
SERPINA13P	0	0	0	0	0	0	0	0
SERPINA15P	0	0	0	0	0	0	0	0
SERPINA2	0	0	0	0	0	0	0	0
SERPINA3	0	0	0	0.189565	0	0.126587	0	0
SERPINA4	0	0	0	0	0	0	0	0

SERPINA5	0	0	0	0	0	0	0	0
SERPINA6	0	0	0	0	0	0	0	0
SERPINA7	0	0	0	0	0	0	0	0
SERPINA7P1	0	0	0	0	0	0	0	0
SERPINA9	0	0	0	0	0	0	0	0
SERPINB1	20.550258	35.611759	59.417724	45.365776	26.140084	29.776607	23.950164	39.486948
SERPINB10	0	0	0	0	0	0.554542	0	0
SERPINB11	0	0	0	0	0	0	0	0
SERPINB13	36.989743	29.924347	6.487485	28.911187	34.587155	66.341111	8.83711	46.20918
SERPINB2	71.368702	37.272129	77.864588	36.14693	10.201836	3.165455	19.926487	3.619702
SERPINB3	18.621113	10.313288	29.433453	19.368077	69.226918	37.282904	71.162497	30.012877
SERPINB4	5.998111	0	0	0.718466	13.634616	0	0	0
SERPINB5	160.063385	103.917656	142.080289	71.571744	246.811017	90.863896	365.839397	212.430356
SERPINB6	7.931552	32.341877	72.200787	41.508869	0.963779	43.846722	1.343384	46.177716
SERPINB7	62.988376	23.175944	22.285777	21.820544	21.266616	10.742009	19.855861	26.633412
SERPINB8	25.235112	17.72429	12.208233	29.59181	20.296141	28.484424	6.365995	40.100198
SERPINB8P1	0	0	0	0	0	0	0	0
SERPINB9	1.592129	1.466631	2.14367	1.555038	0	0.676557	0	0.223753
SERPINC1	0	0	0	0	0	0	0	0
SERPIND1	0	0	0	0	0	0	0	0
SERPINE2	0	83.616322	33.772145	106.793441	21.399291	96.872571	175.517614	103.926539
SERPINE3	0	0	0	0	0	0	0	0
SERPINE4P	0	0	0	0	0	0	0	0
SERPINF1	10.050804	14.259694	0	5.654978	0	7.27133	0	4.376995
SERPINF2	0	0.224957	0.676878	0.497112	0	0.363012	0	0.061031
SERPING1	0	1.069831	0	0.553103	0	1.315701	28.756228	0.156718
SERPINH1	0	8.152616	1.6818	6.35365	0.48528	10.629586	25.772817	4.804018
SERPINH1P1	0	0	0	0	0	0	0	0
SERPINI1	0	1.275353	0	0.274816	0	0.27117	0	0
SERPINI2	0	0	0	0	0	0	0	0
SERTAD1	11.609949	26.840362	11.851851	20.438447	13.994157	35.114946	6.427542	35.052016
SERTAD2	30.822783	55.440294	62.827317	57.741496	21.418065	47.697438	37.791244	57.448486
SERTAD3	21.361785	15.218644	11.857766	24.65786	30.920974	26.463372	55.664418	25.379962

SERTAD4	5.004667	18.86694	3.934528	13.91344	6.280056	21.444859	7.467914	26.949092
SERTAD4BP	0	0	0	0	0	0	0	0
SERTM1	0	0	0	0	0	0	0	0
SERTM2	0	0	0	0	0	0	0	0
SESN1	7.587715	5.957948	6.659867	1.844267	2.669505	3.751512	25.541154	10.450275
SESN2	9.614257	11.591057	11.208288	17.613367	6.853369	12.473706	6.078332	6.877435
SESN3	0	9.420156	0	2.466764	6.924888	3.143253	0	9.457932
SESTD1	10.289853	62.614056	35.668505	42.330473	53.208232	29.381285	78.673379	39.995219
SET	199.41251	244.551552	99.476634	243.053957	136.688984	358.467383	164.953369	92.287584
SETBP1	7.454154	8.930788	4.438052	11.582636	7.98605	16.64474	6.450931	14.474474
SETD1A	16.282362	21.55076	14.148434	21.391556	20.506595	42.478642	8.598658	20.536679
SETD1B	8.901427	11.048627	7.550272	5.779905	12.636977	15.068421	0	23.786129
SETD2	27.748911	3.366194	10.70147	6.110364	5.303697	6.255353	26.813061	3.430245
SETD3	44.818745	87.062801	50.090482	69.239633	49.441112	63.711773	47.835984	83.16638
SETD4	4.891654	4.719429	9.658228	11.389356	29.213157	8.448523	0	7.938537
SETD5	74.570916	170.511217	77.021664	179.019731	72.816895	149.099405	132.360887	128.95596
SETD6	8.847039	3.984977	15.287466	3.313746	35.110866	5.120493	10.286109	3.568808
SETD6P1	0	0	0	0	0	0	0	0
SETD7	1.855028	16.695514	0	24.02173	0	22.330736	0	10.496615
SETD9	8.630815	13.85929	22.76849	8.516261	35.135412	9.524084	0	11.694043
SETDB1	12.046394	19.258523	9.315234	30.394245	19.204003	28.105514	88.658943	22.612848
SETDB2	0	3.601737	0	2.264688	0.612827	2.450712	0	2.393729
SETMAR	0	4.458513	14.840032	6.901065	22.547713	5.453538	32.289931	5.756973
SETP1	0	0	0	0	0	0	0	0
SETP10	0	0	0	0	0	0	0	0
SETP11	0	0	0	0.294631	0	0	0	0
SETP12	0	0.205712	0	0	0	0	0	0.20821
SETP14	0	0.400595	0	0.111353	0	0.45394	0	0
SETP15	0	0	0	0	0	0	0	0
SETP16	0	0	0	0	0	0	0	0.246441
SETP17	0	0.808233	0	0	0	0	0	0
SETP2	0	0.174041	0	0.101631	0	0	0	0.34708
SETP20	0	0.768515	0	0.112586	0	0	0	0

SETP21	0	0	0	0	0	0	0	0
SETP22	0	0	0	0	0	0	0	0
SETP3	0	0	0	0	0	0.239161	0	0
SETP4	0	0	0	0	0	0	0	0
SETP5	0	0	0	0	0	0	0	0
SETP6	0	0	0	0	0	0	0	0
SETP7	0	0	0	0	0	0	0	0
SETP8	0	0	0	0.167062	0	0	0	0
SETP9	0	0	0	0	0	0	0	0
SETX	30.66468	38.684516	28.270068	25.548934	17.400409	14.642492	16.912772	34.492445
SEZ6	0	0.037531	0	0.148701	0	0	0	0
SEZ6L	0	0	0	0	0	0	0	0
SEZ6L2	0	8.222377	4.913144	26.19445	7.631019	11.204335	0	12.684411
SF1	115.893771	283.80026	168.351864	365.063523	236.738763	647.780043	388.38165	368.260165
SF3A1	50.736443	78.932524	43.285656	87.841271	181.922376	161.777046	119.815499	61.866909
SF3A2	104.338728	56.728186	71.997078	74.27229	83.424586	82.553789	163.541966	52.880892
SF3A2P1	0	0	0	0	0	0	0	0
SF3A3	48.671048	136.714173	59.850548	150.24938	140.317838	234.878192	176.923377	172.991776
SF3A3P1	0	0	0	0	0	0	0	0
SF3A3P2	0	0	0	0	0	0	0	0
SF3B1	0	39.270993	36.500225	106.441168	138.925975	133.154977	214.16956	95.687566
SF3B2	11.118738	8.694424	0.772666	14.646046	16.346749	19.270493	52.080125	4.06748
SF3B3	217.575382	388.829141	221.895419	468.159228	309.756593	299.890397	344.663927	292.34343
SF3B4	99.627396	73.321602	51.546558	75.836917	112.88032	67.08471	144.251661	65.452805
SF3B4P1	0	0	0	0	0	0	0	0
SF3B6	537.307839	443.630122	558.928078	382.804916	262.909465	137.832888	212.751403	187.198931
SFI1	7.092172	4.800858	13.498206	13.725574	10.371779	13.383101	2.02983	9.90642
SFMBT1	14.427331	26.026373	9.716728	21.089155	13.696953	19.771935	41.371253	26.6258
SFMBT2	0.814623	2.438247	1.465669	2.31258	1.614687	2.597323	2.185762	3.641096
SFN	608.296873	781.766496	447.804967	910.887233	1041.44635	1903.04625	874.323609	1192.81851
SFPQ	203.782874	241.546605	215.990138	237.661965	238.644603	317.924446	176.680098	257.414319
SFPQP1	0	0	0	0	0	0	0	0
SFR1	11.944568	0	10.123013	2.98587	1.410332	1.2164	0	4.611818

SFR1P1	0	0	0	0	0	0	0	0
SFR1P2	0	0	0	0	0	0	0	0
SFRP1	27.465998	32.108647	26.447776	26.442525	0.194491	0.256093	0	0.295585
SFRP2	0	0	0	0	0	0	0	0
SFRP4	0	0	0	0.226146	0	0	0	0
SFRP5	0	0.626982	1.65487	3.195852	0	0	0	0
SFSWAP	17.02282	6.642611	13.11519	7.652469	8.430028	8.001843	45.714133	7.910367
SFT2D1	29.686154	37.550174	53.972789	27.958848	65.571933	20.333219	25.767998	42.146032
SFT2D2	51.128276	38.816783	34.700518	28.996673	29.62402	31.426949	69.468984	44.684108
SFTA2	0	0	0	0	0	0	0	0
SFTA3	0	0	0	0	0	0	0	0
SFTPA1	0	0	0	0	0	0	0	0
SFTPA2	0	0	0	0	0	0	0	0
SFTPA3P	0	0	0	0	0	0	0	0
SFTPB	0	0	0	0	0	0	0	0
SFTPC	0	0	0	0	0	0	0	0
SFTPD	2.866955	0.791189	0	0.484218	0	0.311141	0	0.274286
SFXN1	224.486045	258.953787	106.169111	183.157591	109.605036	143.019919	65.599231	204.517809
SFXN2	14.980126	7.035203	14.719425	7.181317	6.683994	6.741181	22.194654	15.16973
SFXN3	0	0	8.306292	2.255637	11.937821	0	0	0
SFXN4	0	26.064455	28.296708	8.844334	17.157117	15.777349	24.50168	22.555802
SFXN4P1	0	0	0	0	0	0	0	0
SFXN5	2.431801	5.519095	3.63772	2.521944	24.640607	8.217941	0	2.713654
SGCA	0	0	0	0	0	0	0	0
SGCB	0	14.484567	6.552775	12.675169	26.063248	3.496524	35.906378	5.958457
SGCD	0	0	0	0	0	0	0	0
SGCE	24.155906	25.559145	27.992055	19.674919	77.967014	8.339237	54.448135	13.518158
SGCEP1	0	0	0	0	0	0	0	0
SGCZ	0	0	0	0	0	0	0	0
SGF29	28.16412	17.963688	14.304746	26.873382	38.283046	33.784284	66.870012	23.001314
SGIP1	0	0	0	0	0	0	0	0
SGK1	0.692556	13.236241	6.810453	37.979193	9.060256	82.454172	0	55.68546
SGK2	0	0	0	0.18848	0	0	0	0

SGK3	19.82906	18.333223	20.292432	13.316399	20.462024	8.506237	0	24.306896
SGMS1	15.357089	60.906374	10.403504	87.41983	19.256876	82.960988	3.777199	89.860798
SGMS2	22.127273	39.10095	18.300076	33.151679	6.142765	33.85205	50.05113	45.197279
SGO1	12.706703	26.242796	6.849792	13.675913	24.212424	12.499887	27.777131	24.758594
SGO1P1	0	0	0	0	0	0	0	0
SGO1P2	0	0	0	0	0	0	0	0
SGO2	19.379993	8.91178	13.236908	2.92477	27.832077	8.279711	30.557918	15.231025
SGPL1	0	49.463548	36.235604	56.252927	28.881932	60.921313	0	49.26549
SGPP1	6.998974	4.827846	4.482205	3.048151	7.390743	4.338865	0	4.26009
SGPP2	0.656736	0.591988	0.589961	0.962933	5.551502	1.183795	0	1.686484
SGSH	19.059312	12.220462	16.275816	8.531576	2.588389	25.147574	14.001222	7.769921
SGSM1	0	0	0	0.039816	0	3.349613	0	3.385362
SGSM2	14.556913	6.42571	1.86635	8.107274	39.141495	23.218264	8.891898	12.08346
SGSM3	0	14.50315	9.820956	12.082236	8.931091	17.107465	0	12.926884
SGTA	73.151748	60.616404	45.409343	66.659174	68.34927	116.31956	47.78758	91.315109
SGTB	7.783454	20.629813	9.684502	18.710658	3.163554	6.788934	4.960115	17.947041
SH2B1	3.227572	9.319803	12.454819	9.867339	9.179203	13.603782	46.549906	8.652459
SH2B2	1.633635	1.453812	1.45942	3.380773	0.430764	6.266313	0	2.863341
SH2B3	0	0.643292	0	0.803751	0	0	0	0
SH2D1A	0	0	0	0	0	0	0	0
SH2D1B	0	0.114237	0	0.130731	0	0.3336	0	0.108134
SH2D2A	0	14.033541	0	13.491218	0	2.394884	0	0.695246
SH2D3A	12.02226	18.050275	19.234458	26.508429	30.295224	36.783332	21.692405	23.260928
SH2D3C	4.189161	0	0	0.300886	0	0.109537	0	0
SH2D4A	36.991404	36.91876	10.051069	32.581296	30.472945	62.63842	37.836216	54.518696
SH2D4B	0	0.21317	0	0	0	0	0	0
SH2D5	7.662556	15.992742	4.904529	9.155404	5.489676	27.691556	5.053574	22.684978
SH2D6	0	0	0	0.369092	0	0.566532	0	0.141841
SH2D7	0	0	0	0	0	0	0	0.192703
SH3BGR	5.015577	8.058211	0	11.103709	0	11.849429	0	2.640985
SH3BGRL	43.876581	43.100156	33.783393	32.394544	12.612938	14.951055	50.143915	24.429494
SH3BGRL2	1.42641	3.273235	4.483379	3.083891	9.042322	2.141286	0	2.633537
SH3BGRL3	149.939745	261.512615	174.498094	300.652526	124.882503	348.503615	171.155933	353.348242

SH3BP1	14.077314	24.2727	2.307174	39.482568	13.702616	31.119632	141.619685	29.206582
SH3BP2	13.086478	6.649468	4.822315	7.592262	13.813938	21.596529	30.772331	12.426305
SH3BP4	37.444511	158.847653	43.334798	160.893436	39.902538	299.494314	195.843936	311.479911
SH3BP5	15.062107	27.112829	12.458254	19.024782	19.080547	15.339495	11.238172	12.512272
SH3BP5L	21.292109	27.741309	10.482444	39.478732	15.436408	41.126469	27.906847	26.44581
SH3D19	15.239206	21.095117	28.480658	21.75031	44.359887	35.861719	36.642763	22.9717
SH3D21	0	0	1.513835	0.294806	6.293689	1.46427	0	0.94001
SH3GL1	51.319485	92.484097	37.484826	96.56441	29.234757	135.174696	69.182939	115.902944
SH3GL1P1	0	0	0	0	0	0.485211	0	0.141898
SH3GL1P2	0	0.58051	0	0.084341	0	0	0	0
SH3GL1P3	0	0	0	0	0	0	0	0
SH3GL2	0	1.654017	2.318737	1.955257	1.367037	8.178281	11.946115	8.082249
SH3GL3	0	10.552843	9.670319	4.742643	1.702167	2.270793	9.364261	3.394198
SH3GLB1	115.641335	230.434657	110.162117	144.665481	32.404345	61.099645	50.300167	133.727115
SH3GLB2	26.537842	67.583296	38.197455	73.354624	20.17212	74.353867	40.022684	46.099521
SH3KBP1	11.314771	20.638405	16.037032	20.424722	22.838293	45.678674	4.314946	36.397965
SH3PXD2A	0	0	0	0.062305	0	0	0	0
SH3PXD2B	13.279365	33.895764	17.171784	44.165234	13.708865	64.746049	7.895081	37.571379
SH3RF1	28.844667	65.935217	18.930363	79.072389	19.443725	64.193949	12.754624	40.534644
SH3RF2	24.82633	33.146316	30.728371	41.727839	31.707745	34.073194	44.847891	22.548963
SH3TC1	10.553668	13.272843	18.376558	14.237023	0.661296	15.094337	3.483481	10.3003
SH3TC2	4.862888	9.309539	3.176217	16.248739	54.796511	55.207147	3.176382	50.427522
SH3YL1	51.506809	99.956644	50.546856	98.586602	17.559746	79.952253	81.183577	91.687683
SHANK1	0.684996	1.907799	0.451597	1.624541	0	0.125962	0	0.020137
SHANK2	0	8.687771	1.917332	16.220169	34.87863	19.532605	65.760275	7.13175
SHANK3	0	0	0	0	0	0	0	0
SHARPIN	8.999468	34.910968	42.778202	33.274399	15.671867	33.427256	24.116869	24.642353
SHB	3.770649	29.050324	13.073931	30.549274	10.247437	51.934252	17.330983	38.429128
SHBG	0	0	0	0.129985	0	0.906007	0	0
SHC1	45.046159	39.356992	30.147814	68.419075	82.927073	171.342299	73.970726	96.600699
SHC1P1	0	0	0	0	0	0	0	0
SHC1P2	0	0	0	0	0	0	0	0
SHC2	0	0	0	0	0	1.179244	0	0.443756

SHC3	0	0.141028	0	0.184213	0.951121	3.666056	1.117601	4.337243
SHC4	0	0.6984	0	1.121453	0	0.395669	0	0.290955
SHCBP1	23.37018	40.967743	11.342453	22.874476	85.665281	34.283628	142.970067	47.840933
SHCBP1L	0	0	0	0	0	0	0	0
SHD	0	0	0	0.196727	0	0	0	0
SHE	0.495713	0.170588	0.891437	0.097002	0	0.074596	0	0
SHF	6.436449	2.218829	5.994672	4.772537	1.953466	4.628393	5.617806	1.218301
SHFL	14.455101	18.530013	11.288307	23.561127	30.936217	53.053957	0.585896	23.005349
SHH	3.528501	8.727544	8.23901	15.301282	0	0.070631	0	0.848514
SHISA3	0	0	0	0	0.388778	0	0	0
SHISA4	0	3.104118	11.391939	1.784755	0	4.145101	0	2.898806
SHISA5	81.832014	97.565884	55.675057	106.20236	63.127037	119.41374	67.236341	93.950383
SHISA5P1	0	0	0	0	0	0	0	0
SHISA5P2	0	0	0	0	0	0	0	0
SHISA6	0	0	0	0	0	0	0	0
SHISA8	0	0	0	0	0	0	0	0
SHISA9	0	0.061966	0	0	0	0.040819	0	0
SHISAL1	3.780778	7.483409	0.84998	5.760123	1.248846	1.422722	0.115235	1.212474
SHISAL2A	0	0.617362	3.747246	0.203798	0	0	0	0
SHISAL2B	0	0.387482	0	0.731702	0	0.342646	0	0.195008
SHKBP1	27.758777	33.604197	81.791919	24.339515	47.433228	50.236274	36.518169	31.71861
SHLD1	0	2.014947	0	4.634925	2.61681	3.311302	14.180416	5.71793
SHLD2	3.376951	10.699085	12.437189	4.585078	0	4.35511	29.288305	7.485412
SHLD2P1	0	0.683441	0	0.33665	0	0	0	0.22742
SHLD2P2	0	0	0	0	0	0	0	0
SHLD2P3	0	0.413052	0	0.108846	0.628973	0.150846	0	0.075793
SHLD3	0	0	0	0	0	0	0	0
SHMT1	28.24557	28.791055	25.27466	27.55361	43.260435	39.973766	14.478657	36.373487
SHMT1P1	0	0	0	0	0	0	0	0
SHMT2	178.372377	93.660207	174.492442	150.985175	78.969821	55.499592	63.080052	37.661851
SHOC1	0	2.390667	1.572465	0.697222	0	0.035229	0	0.08476
SHOC2	46.869243	83.068857	0	69.709589	12.49014	72.061285	64.733817	62.204862
SHOX	0	0.037749	0	0	0	0	0	0

SHOX2	0	1.698608	0.989443	1.579975	0	0	0	0.14147
SHPRH	3.33788	5.758474	4.994574	5.083099	1.947703	5.362845	4.96564	7.834114
SHQ1	16.574189	33.003841	8.488877	34.618425	27.039038	30.530916	15.998849	44.598933
SHQ1P1	0	0	0	0	0	0	0	0
SHROOM1	4.562208	16.787297	3.108121	5.113492	9.585331	4.607878	0	6.300479
SHROOM2	7.915573	12.685352	2.555021	11.106048	5.941282	12.177178	8.48674	12.508255
SHROOM2P1	0	0.66039	0	0	0	0.142213	8.684804	0.173117
SHROOM3	18.699215	43.39647	11.765046	43.581631	16.08913	129.846673	37.639588	73.669604
SHROOM4	6.358853	5.606791	0	3.283612	0.190736	3.772113	0	3.249023
SHTN1	38.913719	24.964865	48.005299	22.886325	34.262574	20.317154	34.554458	25.990603
SI	0	0	0	0	0	0	0	0
SIAE	6.908608	12.262261	8.625686	7.614559	7.293272	4.081499	0	5.860107
SIAH1	26.178866	42.787786	26.415828	34.68324	27.611708	32.127556	9.116033	52.498458
SIAH1P1	0	0	0	0	0	0	0	0
SIAH2	28.150223	31.895702	25.169861	45.510025	20.71146	83.41259	66.076873	67.408838
SIAH3	0	0	0	0	0	0	0	0
SIDT1	0	0.471792	6.902107	0.161855	0	0.106211	0	1.38911
SIDT2	2.933666	0.233495	24.412382	3.032596	14.982473	2.190553	14.81581	4.791251
SIGIRR	10.176902	13.685245	7.003934	14.001062	25.328039	14.471001	18.362137	7.594879
SIGLEC1	0	0.228312	0	0	0	0	0	0
SIGLEC10	0	0	0	0.144006	0	0	0	0
SIGLEC11	0	0	0	0.074188	0	0	0	0
SIGLEC12	0	0	0	0	0	0	0	0
SIGLEC14	0	0	0	0	0	0	0	0
SIGLEC15	0	1.511203	0	0.68631	0	0.261503	0	0.370679
SIGLEC17P	0	0	0	0	0	0	0	0
SIGLEC18P	0	0	0	0	0	0	0	0
SIGLEC20P	0	0	0	0	0	0	0	0
SIGLEC21P	0	0	0	0	0	0	0	0
SIGLEC22P	0	0	0	0	0	0	0	0
SIGLEC27P	0	0	0	0	0	0	0	0
SIGLEC29P	0	0	0	0	0	0	0	0
SIGLEC30P	0	0	0	0	0	0	0	0

SIGLEC31P	0	0	0	0	0	0	0	0
SIGLEC6	0	0	0	0	0	0	0	0
SIGLEC7	0	0	0	0	0	0	0	0
SIGLEC8	0	0	0	0	0	0	0	0
SIGLEC9	0	0	0	0	0	0	0	0
SIGLECL1	0	0	0	0	0	0	0	0
SIGMAR1	77.745738	116.197091	94.412251	108.264171	78.570317	131.799882	122.046117	109.356158
SIK1	0	28.419669	34.18991	29.033613	31.164292	52.209639	0	106.238202
SIK2	9.016612	22.169602	8.114732	21.492164	47.235431	26.260179	50.671581	19.507591
SIK3	18.228786	9.037923	2.217345	16.873823	15.779277	10.007854	26.29728	6.906405
SIKE1	53.544941	177.364398	107.010698	161.80781	70.615284	109.31906	97.084484	107.313041
SIL1	17.292737	65.214687	36.28038	66.265723	29.259674	26.593438	30.259494	15.885093
SIM1	0	0	0	0.10084	0	0	0	0
SIM2	0.990739	8.356815	4.316101	16.461836	9.400511	19.331707	16.014004	14.464511
SIMC1	13.05988	12.794001	13.582821	16.899536	21.934569	7.041374	9.636626	5.941923
SIMC1P1	2.073527	1.536053	0	2.63372	0.7116	0.20955	0	0.419484
SIN3A	37.833062	60.792056	31.079273	81.131275	51.939161	75.943546	36.287885	76.977891
SIN3B	2.279844	1.989152	3.097428	2.91479	15.017472	9.265649	35.672685	14.400116
SINHCAF	198.143411	161.070275	78.37792	252.659508	103.59701	149.126039	114.258335	210.201615
SINHCAFP1	0	1.593175	0	0.470627	1.734693	0	0	3.023767
SINHCAFP2	0	0	0	1.334237	0	0.320397	0	0
SINHCAFP3	0	0	0	0	0	0.328492	0	0
SIPA1	15.211139	42.569028	17.052121	33.680764	6.275018	16.787363	92.858525	8.847418
SIPA1L1	37.746122	72.182274	26.825651	54.30518	1.013386	52.431297	6.710233	82.40858
SIPA1L2	0	3.146594	0	1.540523	0	0.381279	0	4.253132
SIPA1L3	37.881493	104.753245	50.317103	91.858611	39.638024	124.098842	84.016299	90.526887
SIRPA	11.980002	16.009323	9.860011	11.636526	2.033672	13.749243	9.993652	11.395249
SIRPAP1	0	0.502867	0	0.291134	0	0.392101	0	0
SIRPB1	0	0.795799	0	0.79599	0	0	0	1.104337
SIRPB2	4.500149	0.723932	0	1.294747	0	0.990862	0	0.503917
SIRPB3P	0	0	0	0	0	0	0	0
SIRPD	0	0	0	0	0	0	0	0
SIRPG	0	0	0	0	0	0	0	0

SIRT1	14.994716	14.028224	24.063828	7.572846	12.129744	4.738806	21.503984	11.279428
SIRT2	11.26764	6.476711	15.078199	15.949266	6.43448	40.872521	31.980457	29.370636
SIRT3	3.504229	19.986698	7.32386	21.344501	2.429674	27.045617	18.91485	19.655996
SIRT4	0	1.522908	0	1.249753	0	2.37983	0	2.478081
SIRT5	14.920818	3.134116	5.478477	8.843226	6.811424	13.431556	1.273287	8.469099
SIRT6	20.282168	7.032063	14.26503	7.01542	3.851518	6.066079	0	5.370225
SIRT7	8.179405	12.446669	11.365144	12.707051	8.006465	13.496594	1.483862	10.299818
SIT1	0	0	0	0	0	0	0	0
SIVA1	43.629438	120.379234	33.919007	74.941229	5.80501	88.781296	30.586347	111.570925
SIX1	9.490734	4.313049	11.661778	0	0	0	11.08578	0
SIX2	0	0.594994	0	0.568316	0	1.647643	0	0.816731
SIX3	0	0.47768	0	0.455222	0	0	0	0
SIX4	17.470194	44.363874	43.002384	50.818569	35.126143	46.841546	34.215781	39.122371
SIX5	0	2.103566	0	2.801568	2.163229	11.189384	13.705231	6.244288
SKA1	32.707369	26.737352	12.70959	11.866532	39.864576	17.67001	15.795465	29.553142
SKA2	85.454959	118.556259	114.211131	82.82446	122.103584	80.037716	267.67134	156.010124
SKA2P1	0	0	0	0	0	0	0	0
SKA3	15.747355	16.320102	8.814688	7.351971	4.461864	7.003131	89.604891	11.141439
SKAP1	0	0	0	0.353662	0	0	0	0
SKAP2	45.789243	45.269914	55.55579	51.454028	16.849184	31.484793	68.625044	43.543085
SKI	17.631286	16.895139	15.849631	17.456646	53.564498	67.749089	82.854774	45.025931
SKIC2	13.09162	7.617452	9.39685	6.70232	25.894119	16.981403	21.207297	11.180714
SKIC3	27.986658	18.930946	0	11.404546	47.201139	4.987295	0	8.563848
SKIC8	36.978915	70.253724	46.618245	51.495218	74.878753	35.383718	27.414081	50.498696
SKIDA1	0	0.108085	0	0	0	0.252471	0	2.951515
SKIL	97.208794	241.715246	100.367198	257.080346	49.41529	112.39173	64.252719	190.553808
SKINT1L	0	0	0	0	0	0	0	0
SKOR1	0	0.197486	0	0.180245	0	0.322685	0	0.368524
SKOR2	0	0	0	0	0	0	0	0
SKP1	597.016922	561.690503	459.042381	374.034926	336.84412	257.032788	416.012591	467.340466
SKP1P1	0	0	0	0	0	2.01756	0	0.441579
SKP1P2	0	0	0	0	0	0	0	0
SKP1P3	0	0	0	0	0	0	0	0

SKP1P4	0	0	0	0	0	0	0	0
SKP2	0	2.325236	0	10.767884	0	12.706148	0	12.61788
SKP2P1	0	0	0	0	0	0	0	0
SLA	0	0.16011	0	0	0	0	0	0
SLA2	0	0	0	0.030065	0	0	0	0
SLAIN1	0	2.286378	1.488464	2.039069	0	1.13285	0	1.18009
SLAIN2	76.538991	45.056774	108.113408	28.746784	85.104231	19.492577	153.756782	50.045671
SLAMF1	0	0	0	0	0	0	0	0
SLAMF6	0	0	0	0	0	0	0	0
SLAMF6P1	0	0	0	0	0	0	0	0
SLAMF7	0	0.06194	0	0	0	0.5292	0	0.57469
SLAMF8	0	0	0	0	0	0.178658	0	0
SLAMF9	0	0	0	0.302112	0	0	0	0
SLBP	89.147181	158.295234	105.551778	187.688056	63.909829	139.80197	94.127224	129.354503
SLC10A1	0	0	0	0	0	0	0	0
SLC10A3	24.303211	67.365141	22.095527	55.863403	17.178954	99.297197	0.96491	68.3422
SLC10A4	0	0.206953	0	0.118664	0	0.080626	0	0.196917
SLC10A5P1	0	0	0	0	0	0	0	0
SLC10A6	0	0	0	0.276709	0	0	0	0
SLC10A7	5.808369	5.376223	0	10.952124	1.038007	2.133429	0	3.011964
SLC11A1	0.920435	0	0	0.089834	0	0.459513	0	1.261418
SLC11A2	12.571019	17.270492	17.768867	19.551872	26.994316	4.133304	3.40946	9.17262
SLC12A1	0	0	0	0	0	0	0	0
SLC12A2	15.467963	11.3753	8.049536	9.361874	29.476797	10.944374	27.647533	22.085754
SLC12A3	0	0	0	0	0	0	0	0
SLC12A4	20.843343	20.049657	25.064884	12.453552	21.102067	12.327584	0	8.057357
SLC12A5	0	1.00781	11.921787	6.824198	0	2.506714	0	1.258122
SLC12A6	25.551542	25.852214	47.013071	25.183012	30.935068	13.734244	6.92796	21.25232
SLC12A7	9.259164	6.993178	12.184845	11.906356	20.506252	16.904539	0.300182	8.509626
SLC12A8	18.199682	2.552039	17.322344	7.758944	4.391713	4.07231	106.303573	7.907259
SLC12A9	14.404938	4.77054	5.12502	5.3173	1.793156	8.846776	12.571204	11.001532
SLC13A1	0	0	0	0	0	0	0	0
SLC13A2	0	0	0	0	0	0	0	0

SLC13A3	24.544895	18.8421	23.685097	37.293229	7.924045	3.45247	0	3.635201
SLC13A4	0	0.099063	0	0.167031	0	0	0	0
SLC13A5	0	0	0	0	0	1.130255	0	0.611083
SLC14A1	0	0	0	0.198744	0	1.514963	0	4.229579
SLC14A2	0	0	0	0	0	0	0	0
SLC15A1	0	0.092252	2.882041	0.470384	0	0	0	0
SLC15A2	15.990036	3.601322	3.766909	3.07328	2.214908	0.997723	0	3.48798
SLC15A3	0	0	0	0	0.511669	0.862551	0	0.313686
SLC15A4	16.926224	9.735074	10.99279	5.756208	29.819917	2.626472	13.008399	3.963571
SLC15A5	0	0	0	0	0	0	0	0
SLC16A1	0	83.818168	0	120.222294	0.797693	114.83539	0	178.288838
SLC16A10	0	0.557647	0	0.773494	0.168868	0	0	0.846756
SLC16A11	0	0	0	0	0	0	0	0
SLC16A12	2.819644	1.602642	1.266128	1.033279	1.48955	0.423316	0	0.367787
SLC16A13	0	2.176159	4.342115	0.315029	0	1.002156	0	0.985729
SLC16A14	3.051691	13.190551	6.849371	9.556692	0	9.34067	0	5.911584
SLC16A14P1	0	0	0	0	0	0	0	0
SLC16A1P1	0	0	0	0	0	0	0	0
SLC16A2	23.653786	11.858237	21.517851	9.117947	31.866099	14.22167	31.349842	14.617017
SLC16A3	0	3.143698	0	4.248942	0	4.03316	0	1.930034
SLC16A4	0	0.212765	0	0.086066	0.60223	0	0	0.216055
SLC16A5	1.900588	7.417557	9.334183	9.94493	1.968794	14.396497	0	9.447994
SLC16A6	0	0	0	0.04007	0	0	0	0.655124
SLC16A6P1	0	0	0	0	0	0.174539	0	0
SLC16A7	4.349979	9.015541	2.937196	10.191583	20.101954	25.139739	2.256484	19.637947
SLC16A8	0	0	0	0	0	0	0	0
SLC16A9	0	0.233669	0	0.177707	0	0.181808	0	0.146298
SLC17A1	0	0	0	0	0	0	0	0
SLC17A2	0	0	0	0	0	0	0	0
SLC17A3	0	0	0	0	0	0	0	0
SLC17A4	0	0	0	0	0	0	0	0
SLC17A5	5.070033	3.595644	9.090573	3.412514	4.818319	2.937804	13.310709	3.913093
SLC17A6	0	0	0	0	0	0	0	0

SLC17A7	0	0	1.372055	0.111101	0	0	0	0
SLC17A8	0	0	0	0	0	0	0	0
SLC17A9	0	0.460328	0	0	0	0	0	0
SLC18A1	0	0	0	0	0	0	0	0
SLC18A2	0	0	0	0.021092	0	0	0	0
SLC18A3	0	0	0	0	0	0	0	0
SLC18B1	3.021411	5.198277	4.97214	6.916687	11.363185	4.201828	10.253947	6.905673
SLC19A1	5.547939	0.985019	2.238908	5.683549	4.329902	7.334499	2.395538	4.894049
SLC19A2	7.910994	4.797828	6.20657	3.359516	9.659293	2.635269	0	4.304545
SLC19A3	0	0.161469	0	0.35033	0	1.45697	0	0.229457
SLC19A4P	0	0	0	0	0	0	0	0
SLC1A1	0.896864	0	0	0.109432	0	0	0	0
SLC1A2	0	0	0	0	0	0	0	0
SLC1A3	0	26.753789	0	37.351019	13.025385	19.549854	0	8.41603
SLC1A4	15.833826	13.026658	21.328189	16.437849	8.364401	11.300113	7.535618	4.105843
SLC1A5	98.164376	173.049188	157.873476	305.890646	157.525414	308.6236	57.757717	232.165344
SLC1A6	2.052842	2.346388	2.521358	1.322305	0	0.279919	0	0.760266
SLC1A7	0	0	0	0	0	0	0	0
SLC20A1	48.804995	88.263338	36.304232	46.974515	47.304193	41.801061	28.301616	83.770495
SLC20A1P1	0	0	0	0	0	0	0	0
SLC20A1P2	0	0	0	0	0	0	0	0
SLC20A1P3	0	0	0	0	0	0	0	0
SLC20A2	57.651943	53.540926	23.39679	58.060274	25.09568	53.062172	79.175562	71.328124
SLC22A1	0	2.845522	0	0.293154	0	0	0	0
SLC22A10	0	0	0	0	0	0	0	0
SLC22A11	0	0	0	0	0	0	0	0
SLC22A12	0	0	0	0	0	0	0	0
SLC22A13	0	0	0	0	0	0	0	0
SLC22A14	0	0	0	0.075924	0	0	0	0
SLC22A15	4.87981	3.591339	3.756547	2.429108	0	1.770104	0	1.035161
SLC22A16	0	0	0	0	0	0	0	0
SLC22A17	0	0.521927	0	0.728653	65.663839	0.628702	0	0
SLC22A18	0	9.643307	0	2.057591	0	3.748211	1.641774	0.515285

SLC22A2	0	0	0	0.280396	0	0.812589	0	0
SLC22A20P	0	0	0	0	0	0	0	0
SLC22A23	3.41679	5.196209	2.591968	1.408512	1.004478	6.711287	6.069398	1.463742
SLC22A24	0	0	0	0	0	0	0	0
SLC22A25	0	0	0	0	0	0	0	0
SLC22A3	10.332867	4.104418	4.631203	4.182491	0	1.957778	0	2.533972
SLC22A31	0	0.635374	0	2.557165	0	0.503163	0	0.227754
SLC22A4	0	1.174063	0	1.328825	2.89736	1.962583	0	1.744103
SLC22A5	0.369827	3.204435	0	4.777882	21.484694	4.446929	0	1.984979
SLC22A6	0	0	0	0	0	0	0	0
SLC22A7	0	0	0	0	0	0	0	0
SLC22A8	0	0	0	0	0	0	0	0
SLC22A9	0	0	0	0	0	0	0	0
SLC23A1	0	0	0	0	0	0	0	0
SLC23A2	19.555866	36.801253	21.773893	40.243131	18.210123	38.234536	34.213901	49.927174
SLC23A3	0	0	0	0.075223	0	0	0	0
SLC23A4P	0	0	0	0	0	0	0	0
SLC24A1	2.383139	19.848285	11.5091	24.527201	1.950841	15.37818	0	6.752533
SLC24A4	0.787315	0.725162	0	0.22973	0	0	0	0
SLC24A5	0	0	0	0	0	0	0	0
SLC25A1	78.544207	81.560493	43.173822	58.588596	83.826237	126.843621	123.764332	114.742891
SLC25A10	10.948019	25.031255	14.399156	22.298702	11.8129	33.731237	18.544773	31.85887
SLC25A11	33.433399	36.080162	31.653056	27.44858	40.712953	46.715217	54.990414	44.903786
SLC25A12	9.239773	16.27026	14.905849	6.630991	3.040552	6.779868	19.067161	13.749089
SLC25A13	21.209181	18.082918	0	5.272345	0	7.842053	28.055714	13.343907
SLC25A14	16.565465	26.087676	20.671269	21.878796	15.803056	8.859294	0	15.653705
SLC25A14P1	0	0	0	0	0	0	0	0
SLC25A15	5.021813	14.264518	4.465763	9.522742	11.079214	8.63708	48.060263	13.952362
SLC25A15P1	0	0	0	0	0	0	0	0
SLC25A15P2	0	0	0	0	0	0	0	0
SLC25A15P3	0	0	0	0	0	0	0	0
SLC25A15P4	0	0	0	0	0	0	0	0
SLC25A15P5	0	0	0	0	0	0	0	0

SLC25A16	4.969608	10.664754	14.767789	7.312655	4.947202	2.399456	6.152515	8.917215
SLC25A17	23.148765	19.582768	33.304151	17.403025	11.309489	24.450511	136.031081	28.269564
SLC25A18	0	0.356266	0	0.301256	0	0.45625	0	0.331041
SLC25A18P1	0	0	0	0	0	0	0	0
SLC25A19	27.046264	53.702558	22.083757	70.532211	21.791003	91.196742	7.587805	76.772419
SLC25A1P1	0	0	0	0	0	0	0	0
SLC25A1P2	0	0	0	0	0	0	0	0
SLC25A1P3	0	0	0	0	0	0	0	0
SLC25A1P4	0	0	0	0	0	0	0	0
SLC25A1P5	0	0.212765	0	0.125147	0	0	0	0
SLC25A20	3.953756	5.587901	2.334541	2.82861	6.91671	2.926325	9.495987	5.442036
SLC25A20P1	0	0	0	0	0	0	0	0
SLC25A21	0	9.224804	0	6.499466	0.98626	0.273606	0	2.800113
SLC25A22	53.153503	56.846817	31.837153	65.998238	42.346459	104.731457	49.257999	98.265795
SLC25A23	24.471501	35.226909	25.477316	40.466918	31.200629	37.045406	17.660279	23.167773
SLC25A24	122.274527	140.522084	121.866584	84.364383	24.212181	41.5666	39.528522	72.632744
SLC25A24P1	0	0	0	0	0	0	0	0
SLC25A24P2	0	0	0	0	0	0	0	0
SLC25A25	0	18.174582	0	16.16437	0	24.846619	18.867655	21.277464
SLC25A26	22.529858	25.064009	6.629495	28.471106	8.273466	21.61727	22.472916	24.198012
SLC25A27	0	1.054918	5.864308	1.278385	0	1.089958	10.774238	1.202548
SLC25A28	21.131849	30.758162	4.810138	42.800689	21.221504	37.901227	15.609334	25.632499
SLC25A29	11.618744	27.508274	12.566509	31.97533	13.536249	60.56162	49.253114	34.952858
SLC25A3	0.667268	5.330441	7.982276	16.022034	38.846292	261.814315	49.744587	63.821258
SLC25A30	5.522611	12.878965	5.68838	12.817125	5.521668	7.693654	9.178522	19.998718
SLC25A31	0	0	0	0	0	0	0	0
SLC25A32	68.536278	48.93771	20.388139	19.657043	20.27826	11.627393	27.854037	38.366315
SLC25A33	2.138423	9.599242	5.377775	5.433623	5.869837	11.848418	9.1658	19.075654
SLC25A34	0	0	0	0	0	0.053335	0	0.086059
SLC25A35	0	6.054357	4.805128	7.469352	0.946104	2.597616	0	2.654149
SLC25A36	45.318342	47.150116	51.487324	36.173962	36.436987	58.801466	30.001627	52.851648
SLC25A36P1	0	0	0	0	0	0	0	0
SLC25A37	19.878716	23.189439	38.681461	26.217491	14.121596	29.82805	21.756347	27.401961

SLC25A38	21.279282	49.113529	17.621622	34.166348	22.014443	48.523914	41.279766	47.430871
SLC25A38P1	0	0	0	0	0	0	0	0
SLC25A39	71.699726	168.240242	98.874132	135.30354	84.464834	195.742269	58.650677	182.19555
SLC25A39P1	0	0	0	0	0	0	0	0
SLC25A39P2	0	0	0	0	0	0	0	0
SLC25A3P1	0	0	0	0	0	0	0	0
SLC25A3P2	0	0	0	0	0	0	0	0
SLC25A3P3	0	0	0	0	0	0	0	0
SLC25A4	10.887318	12.548581	3.788419	10.390199	9.525224	15.307645	25.168762	17.40307
SLC25A40	22.270191	25.791213	24.733654	25.392367	16.776947	22.050339	5.992814	22.017837
SLC25A41	0	0	0	0.062367	0	0.126492	0	0
SLC25A42	4.497264	0.589389	0	3.492076	9.73584	1.020338	0	0.248513
SLC25A43	50.464392	21.57536	35.20124	14.395634	21.47394	14.33968	11.849099	17.545753
SLC25A44	13.911457	18.660711	1.421739	15.832765	52.164597	17.403067	5.729578	10.896707
SLC25A45	0	2.252953	1.254004	1.232607	7.441636	2.482903	0	1.824159
SLC25A46	34.953061	61.897631	40.320258	32.31991	20.333511	24.738549	52.268778	55.184206
SLC25A47	0	0	0	0	0	0	0	0
SLC25A47P1	0	0	0	0	0	0	0	0
SLC25A48	0	0	0	0.249401	0	0.362148	0	0.199395
SLC25A5	679.127267	403.593089	674.785927	355.261011	451.053269	285.050055	540.854058	350.258802
SLC25A51	24.05793	21.923533	12.854053	16.888087	24.951369	19.253682	71.291318	49.724329
SLC25A51P1	0	0	0	0	0	0	0	0
SLC25A51P2	0	0	0	0	0	0	0	0
SLC25A51P3	0	0	0	0	0	0	0	0
SLC25A51P4	0	0	0	0	0	0	0	0
SLC25A53	5.757348	6.482754	5.025483	12.255657	0	16.388817	0	15.736579
SLC25A53P1	0	0	0	0	0	0	0	0.175227
SLC25A5P1	0	0	0	0	0	0	0	0
SLC25A5P2	0	0	0	0	0	0	0	0
SLC25A5P3	0	0	0	0	0	0	0	0
SLC25A5P4	0	0	0	0	0	0	0	0
SLC25A5P5	0	0.180664	0	0	0	0	0	0.180754
SLC25A5P6	0	0	0	0	0	0	0	0

SLC25A5P7	0	0	0	0	0	0	0	0
SLC25A5P8	0	0	0	0	0	0	0	0
SLC25A5P9	0	0	0	0	0	0	0	0
SLC25A6	268.588188	168.539069	451.638722	243.656408	396.767081	314.458618	263.811619	269.495989
SLC25A6P1	0	0	0	0	0	0	0	0
SLC25A6P2	0	0	0	0	0	0	0	0
SLC25A6P3	0	0	0	0.108321	0	0	0	0
SLC25A6P4	0	0	0	0	0	0	0	0
SLC25A6P5	0	0	0	0	0	0	0	0
SLC25A6P6	0	0	0	0	0	0	0	0
SLC26A1	0	0	0	0	0	0.349846	0	0
SLC26A10P	0	0	0	0	0	0	0	0
SLC26A11	3.75588	3.531496	0	2.336354	0	3.671145	0	0.779147
SLC26A2	0	0	0	0.230015	0	0	31.056327	0
SLC26A3	0	0	0	0	0	0	0	0
SLC26A4	0	0	0	0.179414	0	0	0	0
SLC26A5	0	0	0	0	0	0	0	0
SLC26A6	5.153592	1.879107	1.109579	1.092968	5.113773	2.045271	0	1.284636
SLC26A7	0	0.136533	0	0.236881	0	0	0	0
SLC26A8	0	0	0	0	0	0	0	0
SLC26A9	0	0	0	0	0	0	0	0
SLC27A1	1.892829	1.335486	4.565534	2.288037	0	4.236842	0	2.955479
SLC27A2	23.69554	24.331071	28.18502	17.570184	10.927037	5.618555	115.897923	10.733268
SLC27A3	2.849337	6.228785	38.634404	2.663063	31.662046	3.644536	0	2.241245
SLC27A4	19.66468	23.517058	15.771791	16.681064	15.573048	24.563541	8.050051	22.128315
SLC27A5	13.715099	12.022447	41.649414	18.211268	0	17.629305	0	10.416828
SLC27A6	0	0.063408	0	0.858056	0	0	0	0.094654
SLC28A1	0	0	0	0	0	0	0	0
SLC28A2	0	0	0	0	0	0	0	0
SLC28A3	0	0	0	0	0	0	0	0
SLC29A1	23.230045	8.878574	18.334744	22.906535	48.033459	30.042413	0	29.65421
SLC29A2	10.564811	15.739555	16.253716	24.629448	4.776715	10.560374	0	7.238492
SLC29A3	12.408372	10.492355	8.157504	9.333518	9.531357	4.507634	10.69129	3.047691

SLC29A4	3.560974	1.270672	6.137579	1.41587	0	0.491858	0	0.298417
SLC29A4P1	0	0	0	0	0	0	0	0
SLC29A4P2	0	0	0	0	0	0	0	0
SLC2A1	14.369593	55.041408	54.70802	126.869999	85.957149	236.668532	46.973384	187.501199
SLC2A10	3.897634	8.431901	10.496668	7.811281	5.352729	4.456696	1.897456	2.158224
SLC2A11	12.463666	2.428652	3.220835	4.827099	1.370549	4.120645	30.189235	3.511748
SLC2A13	0.895768	4.495996	1.20847	1.783812	1.538674	1.248816	0	2.133165
SLC2A13P1	0	0	0	0	0	0	0	0
SLC2A14	0	0	0	0	0	0	0	0
SLC2A2	0	0	0	0	0	0	0	0
SLC2A3	0	0.222193	3.287905	0.063346	0	0.550862	0	1.053002
SLC2A3P1	0	3.93276	15.784468	7.563445	0	0	0	0
SLC2A3P2	0	0	0	0	0	0	0	0
SLC2A3P4	0	0	0	0	0	0	0	0
SLC2A4	0	0.671314	0	0.136619	0	0	0	0.160743
SLC2A4RG	43.185949	66.923614	90.363199	49.225567	73.915551	79.352842	28.473836	49.080964
SLC2A5	0	0	0	1.430662	0	2.707865	0	0.572997
SLC2A6	8.981346	5.259108	10.647465	5.539248	4.31885	4.931775	0	4.312415
SLC2A8	0	7.127174	9.065259	2.548833	1.354666	5.126382	0	2.717992
SLC2A9	3.75141	9.406612	3.546373	14.667228	0.494373	2.063049	0	1.680378
SLC2AXP1	0	0	0	0	0	0	0	0
SLC30A1	7.72886	6.051058	7.443375	4.107969	6.126191	2.020234	4.171069	4.715689
SLC30A10	0	0	0	0.126091	0	0	0	0.222963
SLC30A2	0	0	0	0	0	0	0	0
SLC30A3	0	1.327927	0	1.637054	0	0.385435	0	0
SLC30A4	1.820247	2.663527	1.227786	2.235136	3.487388	4.088245	0	4.358692
SLC30A5	31.991721	30.930858	25.253669	33.53313	55.326414	35.753746	12.578331	33.313356
SLC30A6	19.276344	12.984816	15.094646	9.599959	27.743336	6.512531	10.278793	16.925111
SLC30A6P1	0	0	0	0	0	0	0	0
SLC30A7	27.281935	11.40799	32.665227	8.830343	8.101369	2.072222	44.159648	5.718002
SLC30A8	0	0	0	0	0	0	0	0
SLC30A9	37.880147	33.843955	51.238603	28.970633	48.936024	19.293271	5.781225	37.251888
SLC31A1	23.406852	19.486505	23.496426	19.309288	20.366962	10.89079	25.651894	16.002335

SLC31A1P1	0	0	0	0	0	0	0	0
SLC31A2	8.190032	17.963601	1.824883	14.138788	14.566046	10.296973	39.587783	18.699768
SLC32A1	0	0	0	0	0	0	0	0
SLC33A1	56.377994	23.984538	32.776122	32.030579	31.708632	14.838492	5.508608	17.746266
SLC34A1	0	0	1.033332	0.112434	0	0	0	0.046404
SLC34A2	0	0	0	0	0	0	0	0
SLC34A3	0	0.13769	0	0.078948	0	0.080463	0	0
SLC35A1	4.290096	3.267699	11.783055	2.197678	7.959656	1.584374	0	6.242023
SLC35A2	20.957746	32.262753	30.543866	34.867225	20.949901	20.457895	42.651181	23.256987
SLC35A3	25.631356	14.318	42.342745	8.754904	34.89963	1.85971	6.792832	6.63375
SLC35A4	0	17.42938	0	21.666989	0	0	0	21.389263
SLC35A5	25.098756	32.418115	10.825933	25.395933	10.404082	6.865476	22.848693	25.418011
SLC35B1	49.992683	79.448618	68.613969	56.766823	43.30957	27.955918	86.414314	37.163248
SLC35B2	48.21579	71.791314	39.804953	80.415657	39.043007	84.441357	38.48989	70.231612
SLC35B3	9.123349	5.78844	1.628322	4.021419	2.141381	2.944763	21.66659	4.462387
SLC35B4	14.935929	12.362007	5.238534	11.214927	15.639815	9.760367	5.563296	7.083312
SLC35C1	0	0	0	1.158928	0	4.900392	0	0
SLC35C2	29.477923	52.602362	23.101064	61.052478	5.777084	36.033997	0	33.88743
SLC35C2P1	0	0	0	0	0	0	0	0
SLC35D1	6.81852	6.541646	11.316637	3.104193	6.375007	1.709564	5.114141	5.364038
SLC35D2	53.430596	20.756566	29.251457	17.451193	8.739811	4.61096	20.395792	8.910379
SLC35D3	0	0.371977	0	0.071008	0	0	0	0.117619
SLC35E1	25.438454	28.943766	30.22339	21.41967	57.713132	45.399313	81.253098	37.551077
SLC35E1P1	0	0	0	0.110646	0	1.554503	0	0
SLC35E2A	0	3.911752	0	12.265468	0	16.391521	0	7.218046
SLC35E2B	2.4536	6.286781	5.293559	5.525474	7.593865	11.456382	7.150444	9.835996
SLC35E3	0.741521	1.021263	1.780712	3.142274	0.725508	0.99264	3.511484	1.200842
SLC35E4	2.625083	4.526218	0	5.567729	0	6.001589	0	6.164682
SLC35F1	0	0	0	0.071663	0	0	0	0
SLC35F2	163.363335	173.301993	231.078533	156.423872	112.466575	51.792896	58.315119	83.635275
SLC35F3	0	2.55207	1.094622	2.61485	1.935501	2.663904	0	2.355368
SLC35F4	0	0	0	0	0	0	0	0
SLC35F5	13.881116	22.291483	18.762672	8.489343	29.62422	17.565089	52.923235	23.906801

SLC35F6	28.81101	23.96602	20.537441	19.320951	41.940721	17.56638	28.45583	23.074047
SLC35G1	10.601316	6.949626	9.093104	4.334069	5.465466	5.574593	9.742002	11.350182
SLC35G2	40.830531	70.852103	33.466155	40.785926	14.590106	10.353395	0.432133	14.384704
SLC35G4	0	0	0	0	0	0	0	0
SLC36A1	10.217592	6.902582	7.097086	8.554998	14.334535	6.064948	3.711275	10.761661
SLC36A2	0	0	0	0	0	0	0	0
SLC36A3	0	0	0	0	0	0	0	0
SLC36A4	75.647919	53.174762	45.715581	29.577664	35.881494	6.136787	2.756263	13.457106
SLC37A1	3.248974	8.812125	3.882088	11.842703	12.863653	21.872864	0	18.261034
SLC37A2	2.509104	12.874721	5.615863	11.086797	7.260284	23.199031	0.318004	20.656835
SLC37A3	12.286166	28.323459	30.603103	26.042096	29.643559	27.10111	64.966746	29.196462
SLC37A4	60.948405	66.854719	78.401631	109.770257	2.205966	17.358667	23.797079	23.277495
SLC38A1	29.705119	100.526923	15.641993	96.219818	49.299944	101.357806	75.205811	112.017735
SLC38A10	32.582097	31.635274	17.302589	43.877275	27.267174	49.84562	23.340128	25.766347
SLC38A11	0	0	0	0.064942	0	0	0	0
SLC38A2	177.221613	274.631383	154.093759	436.562496	70.089615	225.182407	50.603319	276.913421
SLC38A3	0	0	0	0.426287	0	0.547521	0	1.38979
SLC38A4	3.49497	0.897541	1.489012	0.668336	2.768597	1.796112	0	1.976848
SLC38A5	52.249284	22.891576	32.968371	19.672155	0	0	0	1.054978
SLC38A6	31.923659	4.225163	10.583497	0.636975	38.85447	3.286714	44.326297	2.961588
SLC38A7	15.304757	21.195997	5.841286	33.987321	45.760814	40.77225	2.024534	50.223253
SLC38A8	0	0	0	0	0	0	0	0
SLC38A9	19.772642	18.454647	42.694355	26.073117	29.287196	15.236917	11.247346	30.39038
SLC39A1	86.685016	74.243283	95.626279	94.372705	117.114642	98.144285	31.177573	74.465794
SLC39A10	72.017801	37.870947	46.090191	46.379437	34.073977	17.524938	51.048508	24.770772
SLC39A11	0	25.29141	22.392497	22.695557	13.390155	4.419447	17.601338	7.530945
SLC39A12	0	0	0	0	0	0	0	0
SLC39A13	8.23482	11.817396	5.353819	15.160371	21.046667	25.289712	9.782559	13.818469
SLC39A14	31.10792	33.215983	51.186982	33.264276	91.23199	31.408153	25.188478	36.525729
SLC39A1P1	0	0	0	0	0	0	0	0
SLC39A2	0	0	0	0.194262	0	0	0	0
SLC39A3	14.269126	58.296486	17.77881	60.597725	35.981981	70.25947	20.418262	73.865116
SLC39A4	20.764259	56.163294	17.824553	42.169369	0	3.928251	0	6.043772

SLC39A5	0	0	0	0.219098	0	0	0	0
SLC39A6	35.410848	55.352152	46.279612	62.979212	101.734707	44.756745	73.583261	70.061293
SLC39A7	84.12125	152.386526	83.506461	208.481759	74.262646	138.004484	76.895275	115.800027
SLC39A8	18.316577	38.668411	12.37175	17.087349	12.683122	7.353823	0	8.410262
SLC39A9	45.930782	119.058695	48.786101	123.673149	50.858999	90.58184	42.076539	130.099636
SLC3A1	0	0.609456	0	0.06172	0	0	0	0
SLC3A2	75.067865	199.59178	88.008169	386.633952	112.643854	254.981986	88.036131	144.290418
SLC40A1	0	2.038482	0	0.626827	0	0.31993	0	0
SLC41A1	25.432538	87.424284	36.673534	83.346219	26.701162	117.328621	69.136436	85.038364
SLC41A2	30.477496	14.954952	6.691144	10.210723	34.912942	6.488198	57.006489	16.287603
SLC41A3	26.891688	13.037274	19.00256	7.824422	15.117156	16.937182	45.141316	22.831259
SLC43A1	4.348698	13.759313	18.148626	9.527013	3.441223	4.775654	7.381756	2.346468
SLC43A2	2.137774	3.235302	3.783104	2.116672	1.494332	3.624975	0	3.206906
SLC43A3	11.50072	33.194589	14.038407	34.252822	44.554372	139.27187	99.6066	119.220009
SLC44A1	28.031987	56.823508	38.606088	39.794937	32.496272	22.011578	31.591133	52.724278
SLC44A2	21.734778	26.236054	19.488122	38.624119	34.090498	53.018876	54.187927	33.773349
SLC44A3	32.7149	23.288973	24.056083	28.211609	14.356163	6.498023	0	4.680918
SLC44A3-AS1	28.530047	27.062781	18.126711	22.013221	20.839236	35.43409	78.035345	23.841405
SLC44A3P1	0	0	0	0	0	0	0	0
SLC44A4	0	0	0	0	0	0	0	0
SLC44A5	0	0	0	0	0	0	0	0
SLC45A1	0	0.181214	0	0.321593	0	0.198871	0	0
SLC45A2	0	0	0	0	0	0	0	0
SLC45A3	11.41559	8.736238	3.73564	10.331013	1.297185	4.898422	0	4.169991
SLC45A4	19.158442	23.745682	13.247619	18.936471	1.027032	13.726757	0	14.546363
SLC46A1	4.496776	17.266324	14.653656	20.203456	6.270056	15.567343	0.769057	13.574976
SLC46A2	0	0	0	0	0	0	0	0
SLC46A3	0	1.10319	1.444584	0.466477	0	0	0	0.709795
SLC47A1	7.789326	2.217819	4.5628	3.110915	0.731741	2.88781	0	0.59629
SLC47A1P1	0	0	0	0	0	0	0	0
SLC47A1P2	0	0	0	0	0	0	0	0
SLC47A2	0	0.367385	0	0.831584	3.12303	1.205371	0	0.129753
SLC48A1	15.79127	45.327185	17.39994	63.326597	40.126309	57.213641	23.29142	42.200712

SLC49A3	0	0	0	0	0	0	0	0
SLC49A4	23.244598	5.96482	12.605841	5.177379	79.680361	6.538969	6.592096	9.828913
SLC4A1	0	0	0	0	0	0	0	0
SLC4A10	1.951423	1.379025	5.375578	0.363691	0	0	0	0
SLC4A11	3.404555	3.148347	5.168539	3.952905	47.859776	13.085117	10.090651	12.088435
SLC4A1AP	11.934859	14.473728	6.651738	17.483597	11.978291	14.737503	30.457972	15.280605
SLC4A1APP1	0	0	0	0	0	0	0	0
SLC4A1APP2	0	0	0	0	0	0	0	0
SLC4A2	47.462397	39.536518	19.410153	51.061383	20.970491	85.878742	3.464535	43.074279
SLC4A3	0.839979	4.250719	1.456206	2.122393	13.765494	3.476206	0	1.89246
SLC4A4	0	0.216221	0	0.899665	0	0	0	0
SLC4A5	0	0	2.883015	0.187674	0	0	0	0
SLC4A7	27.512957	43.990409	63.745386	64.527779	41.212275	22.353545	18.670082	36.536791
SLC4A8	0	2.193599	0	0.600435	0	0.844801	0	0.475834
SLC4A9	0	0.151845	0	0.40919	0	0.059105	0	0.095551
SLC50A1	77.66752	75.914969	75.293238	103.501724	52.083039	37.427795	79.157385	35.786462
SLC51A	0	0	0	0.478624	0	0	0	0
SLC51B	0	0	0	0	0	0	0	0
SLC52A1	1.438321	1.772129	1.286356	3.228553	0	1.142303	0	0.985056
SLC52A2	29.418123	13.929943	0	5.336624	0	3.792169	0	4.521225
SLC52A3	0	5.5337	5.811065	9.381091	0.982336	7.589608	2.836285	1.939659
SLC5A1	0	0	0	0	0	0	0	0
SLC5A10	0	0	0	0	0	0.333702	0	0
SLC5A11	0	0	0	0.375349	0	0	0	0.643468
SLC5A12	0	0	0	0	0	0	0	0
SLC5A2	0	1.912299	0	0.313651	0	0.522662	0	0.076286
SLC5A3	9.968888	8.499511	15.954978	9.188304	20.6428	10.99183	21.065753	28.987517
SLC5A4P1	0	0	0	0	0	0	0	0
SLC5A5	0	0.078835	0.82657	0.269817	0	0	0	0.037026
SLC5A6	35.249317	19.508864	10.258858	14.948029	27.830367	26.12973	0.359884	25.776223
SLC5A7	0	0	0	0	0	0	0	0
SLC5A8	0	0	0	0	0	0	0	0
SLC5A9	0	0	0	0	0	0	0	0

SLC66A1	7.775834	10.610558	10.401632	13.270572	4.375103	21.363231	11.448097	17.046475
SLC66A1LP	0	0	0	0	0	0	0	0
SLC66A2	12.379955	32.607322	5.279232	25.643784	12.815943	74.50304	188.971607	67.994243
SLC66A2P1	0	0	0	0	0	0	0	0
SLC66A3	6.166559	9.098246	14.048801	7.816249	2.868414	1.925399	86.916531	5.161094
SLC6A1	0	0.035144	0	0.101154	0	0	0	0
SLC6A10P	0	0	0	0	0	0	0	0
SLC6A11	5.427159	7.008095	6.649357	6.97612	0.819047	4.566784	0.5864	5.658781
SLC6A12	0	0.66958	0	4.084733	0	0.086223	0	1.00078
SLC6A13	0	0	0	0	0	0	0	0
SLC6A14	0	0	0	0	0	0	0	0
SLC6A14P1	0	0	0	0	0	0	0	0
SLC6A14P2	0	0	0	0	0	0	0	0
SLC6A14P3	0	0	0	0	0	0	0	0
SLC6A15	37.713622	37.311699	4.474806	42.386129	1.888201	7.171758	0.749887	3.141332
SLC6A16	0	0	0	0	0	1.225489	0	0.56959
SLC6A17	3.538438	7.283326	4.090369	7.096758	0.934981	2.231074	0	1.52002
SLC6A18	0	0	0	0	0	0	0	0
SLC6A19	0	0	0	0	0	0	0	0
SLC6A2	7.488123	7.361807	7.593045	6.54045	0.279533	1.413498	0	0.693491
SLC6A20	0	0	0	0	0.16445	0.58801	0	0
SLC6A21P	0	0	0	0	0	0	0	0
SLC6A3	0	0	0	0.04101	0	0	0	0
SLC6A4	0	0	0	0	0	0	0	0
SLC6A5	0	0	0	0	0	0	0	0
SLC6A6	10.646048	16.936796	31.316029	26.111584	6.65902	41.917445	13.912815	23.831176
SLC6A6P1	0	0	0	0	0	0	0	0
SLC6A7	0	0	0	0.056473	0	0.091453	0	0.110396
SLC6A8	91.170898	273.71058	99.332988	306.676826	79.75289	358.499581	92.818176	413.694163
SLC6A9	17.390946	2.273886	0	8.527705	0.489439	14.257176	0	3.970306
SLC7A1	35.220159	45.035843	54.644712	47.983888	41.30223	44.280066	28.345093	39.492384
SLC7A10	0	0.151273	0	0.104786	0	0	0	0
SLC7A11	49.637383	36.258003	94.940716	44.112162	8.453253	3.532333	6.422222	7.702928

SLC7A13	0	0	0	0	0	0	0	0
SLC7A14	0.316574	0.0546	0	0.289414	9.411306	1.407399	1.236152	4.589517
SLC7A15P	0	0	0	0	0	0	0	0
SLC7A2	0	2.645245	10.208039	1.3873	5.309604	2.511099	10.127506	3.041119
SLC7A3	0	0	0	0	0	0	0	0
SLC7A4	0	0.063027	0	0	5.089713	2.135637	6.477919	1.136472
SLC7A5	274.67729	668.624804	479.440372	697.767246	197.660511	640.87867	133.700805	423.459432
SLC7A5P2	0	0	0	0	0	0	0	0
SLC7A6	25.567987	48.204165	49.954501	51.699179	57.350614	60.103203	8.635107	48.657328
SLC7A6OS	10.876285	21.977926	8.495649	19.606393	25.310875	21.622451	0	15.863873
SLC7A7	0	0	0	0.369319	0	0	0	0.290588
SLC7A8	70.224723	212.988351	34.91153	189.255668	82.213447	368.072657	46.830645	229.484888
SLC7A9	0	0	0	0	0	0	0	0
SLC8A1	10.257622	11.073544	5.931621	7.696322	13.026038	1.765156	1.163943	3.052599
SLC8A2	0	0.157715	0	0.629806	0	0.033047	0	0
SLC8A3	0	0	0	0.057879	0	0.159054	0	0
SLC8B1	10.036593	55.712709	11.756937	76.238682	19.033792	105.323434	0.363278	73.033673
SLC9A1	14.536549	22.918115	49.342933	33.506318	31.997877	38.77932	61.852675	24.356188
SLC9A2	0	0	0	0	0	0	0	0
SLC9A3	8.207518	21.931354	6.80683	19.683155	4.336425	16.786141	5.075587	18.129423
SLC9A3P1	0	0	0	0	0	0	0	0
SLC9A3P2	0	0	0	0	0	0	0	0
SLC9A3P3	0	0	0	0	0	0	0	0
SLC9A4	0	0	0	0	0	0	0	0
SLC9A5	1.656126	1.683786	1.719137	0.945301	0	1.607779	0	0.837974
SLC9A6	19.282115	1.560521	0	0	42.853527	5.801533	3.042859	3.828281
SLC9A7	27.051414	21.269462	67.963355	38.52446	9.023095	17.21913	4.715468	10.892952
SLC9A7P1	0	0	0	0	0	0.556749	0	1.292355
SLC9A8	0	0	0	0.965866	1.963386	0.547496	4.942223	0
SLC9A9	0	0	0	1.734756	0	0	0	3.235893
SLC9B1	0	0	0	0.580736	0	0	0	0
SLC9B1P1	0	0	0	0	0	0	0	0
SLC9B1P2	0	0	0	0	0	0	0	0

SLC9B1P3	0	0	0	0	0	0	0	0
SLC9B1P4	0	0	0	0	0	0	0	0
SLC9B2	3.707657	17.030245	0	13.935516	5.845906	3.666523	0	10.426041
SLC9C1	0	0	0	0	0	0	0	0
SLC9C2	0	0	0	0	0	0	0	0
SLCO1A2	0	7.41436	0	0	2.165395	0.497245	0	1.873135
SLCO1B3	0	10.982379	0	4.793098	0	5.824501	23.935773	14.890652
SLCO1B3-SL(	0	0.200631	0	0.076896	0	0	3.081755	0
SLCO1B7	0	0	0	0	0	0	0	0.946551
SLCO1C1	0	0	0	0	0	0	0	0
SLCO2A1	0	0	0	0	0	2.16399	0	0.29981
SLCO2B1	0	0	0	0	0	0	0	0
SLCO3A1	19.69233	19.224967	20.480235	19.286574	29.440154	22.218725	24.611446	16.326735
SLCO4A1	8.891577	11.099731	2.274206	15.084978	42.242932	28.008494	33.82038	31.436597
SLCO4C1	0	0	0	0	0	0	0	0
SLCO5A1	0	0.166905	0	0.388959	0	0	0	0.162567
SLCO6A1	0	0	0	0	0	0	0	0
SLF1	14.801257	8.810411	12.789426	12.884853	23.169632	7.761093	28.021033	6.856408
SLF2	20.203539	26.633301	19.077133	25.151033	31.563118	22.419209	23.731018	30.839818
SLFN11	62.381078	131.81976	81.375113	162.461853	63.274279	93.778024	56.328916	106.423585
SLFN12	0	0	0	0.114668	0	1.309779	0	5.171486
SLFN12L	0	0	0	0	0	0	0	0
SLFN13	52.205966	54.182001	16.093251	54.815826	9.424468	28.028529	5.595185	27.624783
SLFN14	0	0	0	0	0	0	0	0
SLFN5	45.089099	77.527361	59.363866	100.796782	12.165848	16.084052	22.55593	23.431288
SLFNL1	0	0.054322	0	0	0	0	0	0
SLIRP	139.787421	17.108108	0	16.529861	0	0	16.025899	20.335001
SLIRPP1	0	0	0	0	0	0	0	0
SLIRPP2	0	0	0	0	0	0	0	0
SLIT1	0	0	0	0.105466	0	0.036003	0	0
SLIT2	0	0.293265	0	0.636962	0.635131	7.832313	8.632701	8.665442
SLIT3	4.014016	3.798076	1.802815	3.053292	0	0	0	0
SLITRK1	0	0	0	0	0	0	0	0

SLITRK2	0	0	0	0	0	0	0	0
SLITRK3	0	2.075016	0	1.091439	0	0	0	0
SLITRK4	1.13089	0.727149	3.562169	1.230503	0	0	0	0
SLITRK5	1.054695	2.363477	1.900385	2.363408	0	0.091045	0.036806	0.03618
SLITRK6	11.667792	12.866868	4.872054	3.501594	0.307927	0.581532	0	6.549473
SLK	45.841212	50.380402	43.40242	47.787619	106.603525	44.452835	36.234802	54.138887
SLMAP	71.860978	196.021881	31.609848	203.820759	30.684195	239.282429	49.920949	272.714362
SLN	0	0	0	0	0	0	0	0
SLPI	22.90517	30.324969	46.188188	69.971621	83.605726	29.809632	0	12.390256
SLTM	172.122025	64.571126	110.043091	82.375817	194.267205	120.156912	103.247967	71.455103
SLU7	27.7291	64.259083	27.439798	84.706392	101.577906	53.76436	78.471091	48.492175
SLURP1	0	0	0	0	0	0	0	0
SLURP2	0	0	0	0	0	0	0	0
SLX1A	7.181778	1.025536	0	0.308986	0.478746	0.346503	0	0.346641
SLX1B	7.181778	11.59036	0	5.021022	0.478746	8.926347	0	10.772603
SLX4	6.629708	25.752926	4.770324	26.362784	18.006516	34.170672	9.593157	28.721863
SLX4IP	13.418448	16.176034	8.202387	14.323956	10.352645	9.580278	10.85866	16.237998
SLX9	17.797315	27.525242	35.21081	18.867652	19.306838	43.818251	43.585846	27.315963
SMAD1	10.065086	41.24909	30.005539	46.718469	27.726999	34.082173	61.101446	27.64231
SMAD2	110.273803	83.70702	76.852185	79.775398	124.738864	85.636665	181.011454	106.338921
SMAD3	126.587823	382.075646	128.618947	583.753386	143.930906	554.713867	71.682362	277.133161
SMAD4	17.159749	52.520267	23.679001	39.896165	27.18171	40.675354	5.122264	43.001091
SMAD5	22.402511	176.665866	94.501087	165.40758	29.90186	126.121869	40.077899	179.005441
SMAD6	1.729743	10.477621	2.116786	9.627796	6.704511	15.739875	0	11.522688
SMAD7	8.063688	9.937937	11.134651	17.113552	11.405623	30.690455	0	13.50961
SMAGP	18.85197	45.228051	3.170533	40.403358	23.069795	24.509021	0	35.819737
SMAP1	9.941225	18.132075	6.351289	15.4424	11.988028	16.340245	0.688884	18.034941
SMAP2	11.582043	35.534349	13.487256	29.476017	20.702436	49.767339	29.725278	33.011666
SMARCA1	10.843683	11.251741	15.037696	9.720825	27.486385	5.574375	0	13.353825
SMARCA2	13.808824	21.06646	29.537771	14.853988	65.363694	48.875629	181.837289	32.824866
SMARCA4	43.784914	38.258694	34.349852	52.357796	89.929253	130.597271	113.715323	71.085058
SMARCA5	39.922446	45.836599	33.265455	31.474391	25.432494	24.32514	37.309265	52.793874
SMARCAD1	0	14.469351	0	13.707876	0	5.965022	43.935909	15.069967

SMARCAL1	18.60117	26.926792	14.776298	21.250069	30.232527	24.631276	9.865005	18.457155
SMARCB1	56.153398	109.155729	30.779171	81.724514	149.587945	154.30128	105.334159	115.118843
SMARCC1	69.596121	74.975978	51.896279	83.729911	160.177475	83.559755	86.316221	75.823976
SMARCC2	68.467506	79.146737	53.256381	86.366561	51.948911	69.36273	88.020157	48.014815
SMARCD1	29.526478	37.044223	14.752256	26.474575	116.924434	50.254077	35.336863	46.197704
SMARCD2	0	0.557628	0	4.888711	24.686832	18.450343	0	8.142555
SMARCD3	0	2.314973	5.671289	2.995518	3.354111	2.17387	0	0
SMARCE1	92.506351	137.83088	108.071989	139.985794	90.937433	126.872187	193.490228	121.972535
SMARCE1P1	0	0.392028	0	0.075748	0	0	0	0.512144
SMARCE1P2	0	0	0	0	0	0	0	0
SMARCE1P3	0	0	0	0	0	0	0	0
SMARCE1P4	0	0	0	0	0	0	0	0
SMARCE1P5	0	0	0	0	0	0	0	0.246555
SMARCE1P6	0	0	0	0	0	0	0	0
SMARCE1P7	0	0	0	0	0	0	0	0
SMBD1P	0	0	11.239243	0.306423	0	0	0	0
SMC1A	90.637362	63.722963	65.543824	63.431855	51.992783	109.815228	28.273056	50.210944
SMC1B	0	0.039056	0	0.044555	0	0	0	0
SMC2	30.993027	37.54451	39.832953	24.92401	63.528736	19.910618	39.852542	40.583809
SMC3	0	0	0	0.187863	0	0	0	0
SMC3P1	0	0	0	0	0	0	0	0
SMC4	178.588374	142.631802	109.765173	89.394944	220.718681	97.796522	264.464274	144.674083
SMC4P1	0	0	0	0	0	0	0	0
SMC5	28.34598	20.624562	16.216863	47.652736	19.789152	37.647416	42.476171	29.297902
SMC6	33.178298	45.512842	30.50285	30.213057	41.821653	33.831031	38.772338	45.322927
SMCHD1	18.402604	13.793574	29.169308	12.292572	14.059462	7.663246	28.00993	12.51476
SMCO1	0	0	0	0	0	0	0	0
SMCO2	0	0	0	0	0	0	0	4.556573
SMCO3	0	0	0	0	0	0	0	0
SMCO4	22.341256	85.674015	114.335635	91.19794	2.343947	48.252141	19.177817	33.433448
SMCO4P1	0	0	0	0	0	0	0	0
SMCP	0	0.217163	0	0	0	0	0	0
SMCR8	8.935172	31.140414	9.087497	31.335227	13.655645	70.931001	12.699295	80.396444

SMDT1	0	39.943679	13.350932	31.220096	40.844841	20.965489	56.145628	24.884656
SMG1	27.762239	95.681951	29.301352	168.041051	61.104653	135.979976	108.138181	148.453566
SMG1P1	0	0	0	2.413703	4.933527	1.073418	0	3.0168
SMG1P2	0	1.061256	5.031769	0.234659	0	0.649758	0	1.046624
SMG1P3	0	3.763778	12.080574	1.514647	0	0	0	5.329591
SMG1P4	0	0	0	0	0	0	0	0.218486
SMG1P5	0	0	0	0	0	0	0	0
SMG1P6	1.553714	0	0	0	0	0	0	0.093347
SMG1P7	0	0.158422	0	0	0	0	0	0.239725
SMG5	33.137357	63.093321	38.166934	68.298097	25.49666	81.139031	33.502641	61.977609
SMG6	9.798099	15.888657	5.358014	18.8607	12.512631	27.707743	42.455162	21.148962
SMG7	41.909722	92.10878	52.232439	80.848545	81.564909	91.518355	62.345211	125.054771
SMG8	14.355703	0	0	0.201207	11.197071	0	0	2.412957
SMG9	10.356273	14.400131	21.461798	21.584838	28.260681	48.032564	7.632305	24.22402
SMIM1	0	4.710089	0	4.109381	0	6.773172	0	1.936619
SMIM10	21.831146	21.671265	12.943998	11.559026	7.664154	5.359526	23.400263	8.545241
SMIM10L1	0	0	0	8.234897	0	26.146056	0	0
SMIM10L2A	0	1.287433	2.805938	1.206029	0	0.281564	0	0.10023
SMIM10L2B	0	1.479358	0	1.77863	0	1.191405	0	0.866862
SMIM11	0	0.713469	1.059805	0.361991	0	2.841913	0	3.68878
SMIM11P1	0	0	0	0	0	0	0	0
SMIM11P2	0	0	0	0	0	0	0	0
SMIM12	34.34122	78.402812	22.856556	69.283882	32.283868	109.962815	34.216722	108.988805
SMIM12P1	0	0	0	0	0	0	0	0
SMIM13	10.963664	26.539859	9.631307	24.705723	19.473232	31.430012	6.69204	30.426381
SMIM14	26.762845	17.57697	11.206936	13.70861	1.784133	2.207439	0.126614	1.52754
SMIM15	123.429123	274.87124	101.061237	238.475826	77.928661	207.597487	128.467187	268.734673
SMIM15P1	0	0	0	0	0	0	0	0
SMIM15P2	0	0	0	0	0	0	0	0
SMIM17	0	0	0	0	0	0	0	0
SMIM19	32.10706	106.080217	42.179644	37.384332	32.708473	8.334546	0	21.440297
SMIM20	0	16.144944	0	2.390707	59.834736	0	0	0
SMIM21	0	0	0	0	0	0	0	0

SMIM22	0	0	0	0	0	0	0	0
SMIM23	0	0	0	0	0	0	0	0
SMIM24	0	2.44506	0	2.817827	0	8.090255	0	1.505959
SMIM26	19.827764	54.961311	60.409317	40.971731	90.994231	44.485085	0	47.392377
SMIM27	0	0.60917	0	1.334139	65.261766	1.115834	0	1.258292
SMIM29	15.541336	21.339275	8.874527	18.823139	8.141226	20.560971	0	14.99925
SMIM3	4.482271	11.401447	0	5.885048	0	7.545477	0	12.3213
SMIM30	45.713227	60.673162	36.979784	41.889449	48.169152	25.886448	99.680378	47.120634
SMIM31	0	0	0	0.704375	0	0	0	0
SMIM35	0	0	0	0	0	0	0	0
SMIM36	0	0	0	0	0	0	0	0
SMIM38	0	0	0	0.193063	0	0	0	0
SMIM43	0	0	0	0.497031	0	0	0	0.412749
SMIM47	0	1.134676	0	0.711685	0	0	0	0.881271
SMIM5	0	0	0	0	0	0.113673	0	0
SMIM6	0	0	0	0	0	0.221453	0	0
SMIM7	51.081401	48.543439	94.240231	60.712382	32.087909	54.532011	15.255149	85.477272
SMIM7P1	0	0	0	0	0	0	0	0
SMIM8	3.27066	3.425634	8.35106	1.908358	1.122868	4.959718	46.286753	2.908953
SMIM9	0	0	0	0	0	0	0	0
SMKR1	12.945644	12.358307	3.537545	6.469785	0	2.130418	0	7.414637
SMLR1	0	0	0	0	0	0	0	0
SMN1	45.89079	47.014143	62.356594	0.595318	24.184652	1.131054	0	12.11336
SMN2	163.364037	82.79424	84.482278	51.295758	92.789055	42.363401	0	78.517813
SMNDC1	19.215744	75.88215	34.139431	78.799586	71.917835	87.854005	74.545182	101.259266
SMO	10.801709	18.755087	15.767607	15.86253	10.971062	26.947057	9.705216	19.277863
SMOC1	0	0	0	0	14.60376	0	0	0.143438
SMOC2	0	0	0	0	0	0	0	0
SMOX	8.336689	53.407746	12.799574	46.995807	16.367738	35.598186	30.791049	34.285921
SMPD1	6.290033	9.708038	5.065184	9.898741	23.09111	34.067399	41.424957	25.001438
SMPD2	4.201458	12.918469	1.871754	7.888751	25.361122	14.699377	0	17.840597
SMPD3	0	0.677748	0	0.21201	0	0.867627	0	0.62137
SMPD4	29.514257	38.071712	40.077365	35.163934	29.114239	55.361013	43.84778	54.956954

SMPD4BP	0	0.641136	0	1.300369	0	0	0	0
SMPD4P1	0	0	0	0	0	0	0	0
SMPD4P2	0	0	0	0	0	0	0	0
SMPD5	0	0	0	0	0	0	0	0
SMPDL3A	0	0.845904	0	0.822675	6.870992	0.886052	0	2.257606
SMPDL3B	3.74695	2.453506	3.252917	6.786165	0.987575	16.538991	37.272956	10.680334
SMPX	0	0	0	0	0	0	0	0
SMR3B	0	0	0	0	0	0	0	0
SMS	159.73338	221.488048	138.64668	199.200944	329.027434	221.37122	262.681255	367.17531
SMSP1	0	0	0	0	0	0	0	0
SMTN	54.842265	1.748728	1.051941	5.520299	14.683405	3.859484	77.668532	5.782454
SMTNL2	0	0	0	0.044088	0	0	0	0.073313
SMU1	18.58776	17.583409	14.678477	16.638493	30.909799	12.604461	23.990726	22.046899
SMU1P1	0	0	0	0	0	0	0	0
SMUG1	50.485155	53.986125	37.895743	56.066236	0	19.543345	2.564735	23.400972
SMUG1P1	0	0	0	0	0	0	0	0
SMURF1	0	1.473083	0	8.857832	0	5.329087	0	3.367246
SMURF2	77.33271	100.2414	46.707392	61.254574	39.109661	43.519991	55.714413	75.48751
SMURF2P1	0	0	0	0	32.76997	0	0	0
SMYD1	0	0	0	0	0	0	0	0
SMYD2	32.298542	58.410592	19.794096	35.94793	27.659334	46.745142	41.338934	80.755614
SMYD3	40.655843	34.112057	45.347422	30.173569	26.210054	17.95326	8.943743	22.522415
SMYD4	1.789026	2.955511	0	3.986044	0	5.027426	57.562137	14.964814
SMYD5	34.30446	50.880961	61.22521	70.803685	19.996745	58.186209	35.170404	43.412955
SNAI1	0	1.134845	1.846221	2.360129	0	0.510589	0	0.418677
SNAI1P1	0	0	0	0	0	0	0	0
SNAI2	42.627862	94.175064	32.450213	88.464052	16.23764	80.878017	106.738498	158.129509
SNAI3	0	1.280764	5.415534	1.71848	0.533613	0.099873	0	0.081833
SNAP23	110.794369	155.78955	83.496879	183.427192	127.109891	124.024754	10.062665	79.437217
SNAP23P1	0	0	28.736105	4.029423	0	0	0	1.597558
SNAP25	0	0	0	0	0	0	0	0
SNAP29	25.950208	32.397952	20.829222	56.977169	17.56636	68.796329	0.376524	65.594985
SNAP47	0	9.335586	21.727233	13.195685	2.882773	11.143931	31.901413	6.837741

SNAP91	2.018659	2.756313	0	0	0	0	0	0.244704
SNAPC1	23.547298	29.676993	36.295765	18.097619	38.657389	19.898583	27.303267	37.693603
SNAPC2	7.69139	29.902703	10.346506	29.457459	14.935559	25.018801	0	18.737504
SNAPC3	15.136544	9.420891	0	10.157832	19.789781	10.654369	68.28252	6.716293
SNAPC4	5.87756	6.419124	4.001604	6.433754	0.444128	8.504198	3.3406	4.8943
SNAPC5	37.235962	91.240024	31.818795	92.890715	50.513253	94.872448	26.98319	89.577412
SNAPC5P1	0	0	0	0	0	0	0	0
SNAPIN	0	7.962352	0	13.140152	49.7289	0.213545	23.435721	2.985159
SNCA	100.313689	186.069661	2.743982	125.5502	25.542549	69.710842	13.334214	26.134645
SNCAIP	0	1.687627	0	0.181301	0	0	0	0
SNCB	0	4.127798	3.537545	6.084343	0	0.780844	0	1.455714
SNCG	33.026841	75.191541	4.812764	80.058431	0	25.38247	0	15.418881
SND1	66.622879	176.763989	139.594835	230.507166	130.141878	163.689209	112.788493	147.234758
SNED1	0.723852	0	0	0.532533	0	0.289732	0	0
SNF8	51.955183	233.660389	87.469387	196.75557	107.088619	220.986781	171.83747	253.936726
SNF8P1	0	0	0	0	0	0	0	0
SNHG32	56.488627	17.41164	58.710163	25.974183	65.552268	12.110337	1.837036	18.501143
SNHG33	0	0.340932	0	0.668792	0	0	0	0
SNIP1	7.337481	10.259892	8.419245	23.15269	8.529583	20.016481	8.604711	15.123977
SNN	16.371577	46.419819	22.014537	42.043786	9.453954	36.890658	6.466779	37.524633
SNORC	0	2.099308	1.086254	1.687105	0	0.181086	0	0.195247
SNPH	0	0.843606	0.588495	0.709714	1.038314	3.28099	0	1.736275
SNRK	10.707171	20.092989	9.054089	22.654956	9.705175	26.386319	15.530524	21.977937
SNRNP200	84.071297	84.4411	97.240995	136.033897	179.024787	115.298399	143.071888	99.842821
SNRNP25	0	10.690533	0	0.422687	0	0	0	0
SNRNP27	49.744743	43.288441	33.949778	37.676554	59.504037	27.644186	10.269544	20.187288
SNRNP35	0	25.70948	0	33.21069	19.633027	26.081007	0	15.16543
SNRNP40	52.84784	41.126853	50.982378	36.626706	89.484831	41.185616	136.212697	47.832994
SNRNP40P1	0	0	0	0	0	0	0	0
SNRNP48	14.322689	10.102712	4.254183	5.467319	11.681858	7.696291	5.959892	4.059564
SNRNP70	99.483749	216.440179	91.75306	246.87158	101.046834	338.694614	147.444862	243.386632
SNRPA	77.832564	158.618704	127.026702	177.33607	145.720726	221.256616	145.503226	195.689503
SNRPA1	66.668512	212.72184	112.575386	172.29951	147.793875	241.432933	175.248523	361.210821

SNRPA1P1	0	0	0	0	1.42025	0	0	0
SNRPA1P2	0	0	0	0.136653	0	0	0	0
SNRPB	196.725845	336.013051	146.533434	314.845228	145.946638	315.365222	280.826333	271.973331
SNRPB2	44.886543	77.437865	102.732032	65.666093	105.493646	81.269277	118.083528	127.439217
SNRPB2P1	0	0	0	0	0	0	0	0
SNRPBP1	0	0	0	0	0	0	0	0
SNRPC	116.470283	122.367664	72.527076	131.877373	98.6555	119.932423	40.919752	126.431064
SNRPCP1	0	0	0	0	0	0	0	0
SNRPCP10	0	0	0	0	0	0	0	0
SNRPCP11	0	0	0	0	0	0	0	0
SNRPCP13	0	0	0	0	0	0	0	0
SNRPCP14	0	0	0	0	0	0	0	0
SNRPCP15	0	0	0	0	0	0	0	0
SNRPCP16	0	0	0	2.266264	0	0	0	0
SNRPCP17	0	0	0	0	0	0	0	0
SNRPCP18	0	0	0	0	0	0	0	0
SNRPCP19	0	0	0	0.26157	0	0	0	0.959689
SNRPCP2	0	0	0	0	0	0	0	0
SNRPCP20	0	0	0	0	0	0	0	0
SNRPCP3	0	0	0	0	0	0.484443	0	0
SNRPCP4	0	0	0	0	0	0	0	0
SNRPCP5	0	0	0	0	0	0	0	0
SNRPCP6	0	0	0	0	0	0	0	0
SNRPCP8	0	0	0	0	0	0	0	0
SNRPCP9	0	0	0	0	0	0	0	0
SNRPD1	212.100495	531.785058	245.271351	435.672049	381.629839	351.404585	313.610707	348.49083
SNRPD2	197.383444	281.968608	205.29181	328.997702	465.159482	525.899294	342.827451	366.602799
SNRPD2P1	0	0	0	0	0	0	0	0
SNRPD2P2	0	0	0	0	0	0	0	0
SNRPD3	25.216934	43.065815	104.312252	67.030888	189.823447	102.597332	21.565688	61.099121
SNRPE	260.525664	379.048719	162.702244	64.535141	281.853068	58.383096	241.607137	207.935392
SNRPEP10	0	0	0	0	0	0	0	0
SNRPEP11	0	0	0	0	0	0	0	0

SNRPEP2	0	0	0	0	0	0	0	0
SNRPEP3	0	0	0	0	0	0	0	0
SNRPEP4	0	32.825051	0	12.509525	0	0	0	10.811303
SNRPEP5	0	0	0	0	0	0	0	0
SNRPEP6	0	0	0	0	0	0	0	0
SNRPEP7	0	0	0	0	0	0	0	0
SNRPEP8	0	0	0	0	0	0	0	0
SNRPEP9	0	0	0	0	0	0	0	0
SNRPF	0	0	0	0.245665	0	39.232361	0	0.809171
SNRPFP1	0	0	0	0	0	0	0	0
SNRPFP2	0	0	0	0	0	0	0	0
SNRPFP3	0	0	0	0	0	0	0	0
SNRPFP4	0	0	0	0	0	0	0	0
SNRPG	403.710267	420.546693	188.109085	339.512297	287.75485	170.826709	287.761278	223.937195
SNRPGP1	0	0	0	0	0	0	0	0
SNRPGP10	0	0	0	0	0	0	0	0
SNRPGP11	0	0	0	0	0	0	0	0
SNRPGP12	0	0	0	0	0	0	0	0
SNRPGP13	0	0	0	0	0	0	0	0
SNRPGP14	0	0	0	0	0	0	0	0
SNRPGP15	0	0	0	0	0	0	0	0
SNRPGP16	0	0	0	0	0	0	0	0
SNRPGP17	0	0	0	0	0	0	0	0
SNRPGP18	0	0	0	0	0	0	0	0
SNRPGP19	0	0	0	0	0	0	0	0
SNRPGP2	0	0	0	0	0	0	0	0
SNRPGP20	0	0	0	0	0	0	0	0
SNRPGP3	0	0	0	0	0	0	0	0
SNRPGP4	0	0	0	0	0	0	0	0
SNRPGP5	0	0	0	0	0	0	0	0
SNRPGP6	0	0	0	0	0	0	0	0
SNRPGP7	0	0	0	0	0	0	0	0
SNRPGP8	0	0	0	0	0	0	0	0

SNRPGP9	0	0	0	0	0	0	0	0
SNRPN	31.066258	42.762677	24.965467	9.607183	0	33.996956	90.240324	34.842402
SNRPNP2	0	0	0	0	0	0	0	0
SNTB1	0	0	0	0.217377	0	0	0	0.053169
SNTB2	0	12.150899	0	6.694539	0	13.556517	0	6.293398
SNTG1	0	0	0	0	0	0	0	0
SNTG2	0	0.520422	0	0	0	0	0	0
SNTN	0	0	0	0	0	0	0	0
SNU13	186.85958	135.641329	140.585515	145.692832	172.712507	69.680669	26.249916	85.125585
SNUPN	23.514109	2.79482	16.868008	8.534348	37.298086	11.759856	0	11.918293
SNURF	0	2.307389	0	63.670547	82.850619	197.812648	138.70701	95.980882
SNURFL	0	0	0	0	0	0	0	0
SNW1	0	13.557224	0	51.31471	65.367794	74.472434	85.144516	39.890379
SNX1	39.646252	126.134224	38.769785	107.503417	51.856771	124.014056	230.77943	95.264084
SNX10	0	1.082694	0	0.534623	0	0.582475	3.794616	0.551632
SNX11	0	23.394599	0	20.326931	47.611747	38.322889	68.688491	22.002556
SNX12	79.264415	51.999221	67.464676	59.984654	34.796628	36.792063	49.065596	48.695819
SNX13	33.391236	58.724656	18.645558	47.550497	74.208106	36.227195	3.328284	61.61771
SNX14	41.889521	29.338622	48.543301	10.415511	16.817799	9.829365	0	29.884469
SNX15	20.106287	19.760721	11.417879	11.646444	66.712474	25.184686	10.172958	13.960279
SNX16	4.821836	8.959936	18.538171	4.793168	3.69715	5.005083	0	5.987672
SNX17	27.554425	72.392637	72.236481	67.767151	45.13147	54.97879	4.710753	64.935009
SNX18	11.883726	18.908016	20.230717	23.710853	20.304208	32.725143	3.504654	35.680648
SNX18P10	0	0	0	0	0	0	0	0
SNX18P12	0	0	0	0	0	0.260226	0	0
SNX18P13	0	1.408056	0	1.061745	0	4.589367	0	1.908082
SNX18P14	0	0	0	0	0	0	0	0
SNX18P1Y	0	0	0	0	0	0	0	0
SNX18P2	0	0	0	0	0	0	0	0
SNX18P23	0	0	0	0	0	0.901668	0	0
SNX18P24	0	0	0	0	0	0	0	0
SNX18P25	0	0	0	0	0	0	0	0
SNX18P26	0	0	0	0	0	0	0	0

SNX18P27	0	0	0	0	0	0	0	0
SNX18P3	0	1.098278	0	1.631862	0	1.791625	0	4.605337
SNX18P4	0	0	0	0	0	0	0	0
SNX18P5	0	0	0	0	0	0	0	0
SNX18P7	0	4.424719	0	0.70497	0	7.835249	0	6.36581
SNX18P8	0	0	0	0	0	0.438027	0	0
SNX18P9	0	0	0	0.115436	0	0	0	0
SNX19	0	28.531831	19.50671	22.339596	38.030227	121.174114	29.825727	77.181054
SNX19P1	0	0	0	0	0	0	0	0
SNX19P2	0	0.578831	0	0.220461	0	0	0	0.1821
SNX19P3	0	0.04891	0	0	0	0	0	0
SNX19P4	0	0	0	0	0	0	0	0
SNX2	80.496265	70.354481	67.484898	55.343029	159.89455	47.061075	109.880355	69.308272
SNX20	0	0	0	0	0	0	0	0
SNX21	9.659629	12.022469	2.899684	15.988132	6.691394	18.988778	17.823411	14.482769
SNX22	0.993895	1.94922	2.663282	1.767471	0	2.361526	0	0.778786
SNX24	39.575902	23.568197	20.284921	20.401721	76.404917	10.593434	83.195982	20.676545
SNX25	7.309689	8.991008	9.9096	9.718403	1.653903	5.689438	13.486888	9.873268
SNX25P1	9.691878	0	0	0.707252	0	1.571803	0	0.412065
SNX27	53.617913	38.421534	41.831487	38.208809	44.019377	21.574232	20.262118	24.400091
SNX29	9.862882	15.525913	7.453564	17.440264	26.888347	14.973842	2.694677	19.033256
SNX29P1	0	0.977485	0	0.14356	0	0.740425	0	0
SNX29P2	0	0	0	1.969615	0	0	0	0
SNX2P1	0	0	0	0	0	0	0	0
SNX2P2	0	0	0	0	0	0	0	0
SNX3	111.409016	103.390251	97.544424	51.289583	64.492042	57.751771	175.918606	125.030823
SNX30	7.548151	18.373024	10.561986	21.937293	9.077682	33.284257	12.9896	17.31664
SNX31	0	0.118245	0	0.476782	0	0.069068	0	0
SNX32	0	1.14012	0	0.549023	0.7449	0.841239	0	0.113745
SNX33	23.645655	33.099683	16.973832	38.069906	23.739793	60.934689	0	40.424145
SNX33P1	0	0	0	0	0	0	0	0
SNX3P1X	0	0	0	0	0	0	0	0
SNX3P1Y	0	0	0	0	0	0	0	0

SNX4	63.980963	55.139535	38.784886	40.00013	62.612792	28.56314	15.478725	52.487516
SNX5	174.522866	182.722217	163.652649	90.546655	196.046374	128.798076	361.739323	403.658848
SNX5P1	0	0.152839	0	0	0	0	0	0
SNX5P2	0	0	0	0	0	0	0	0
SNX6	179.519243	111.474044	74.576516	62.679863	72.591785	61.883052	50.01027	110.650122
SNX6P1	0	0	0	0	0	0	0	0
SNX7	75.741128	85.247887	56.103057	43.581088	61.727462	12.827557	24.364671	45.45419
SNX8	29.83758	104.506388	47.628886	110.512265	52.067188	67.383588	74.466309	50.038764
SNX9	0	0	0	0	0	0	0	0
SOAT1	50.256067	37.822966	37.433429	29.776551	25.095235	17.386432	22.514594	38.676676
SOAT2	0	0	0	0	0	0	0	0
SOBP	0	0.596054	2.075413	0.520718	0.61421	0.966913	2.925101	2.721289
SOCS1	0	0	0	0.089534	0	0	0	0
SOCS2	13.352829	15.853502	8.94484	11.217179	7.591147	12.870988	4.178121	13.464285
SOCS2P1	0	0	0	0	0	0	0	0
SOCS2P2	0	0	0	0	0	0	0	0
SOCS3	27.188917	55.48846	30.983539	72.959936	12.066324	47.648776	13.202196	35.078711
SOCS4	28.597979	44.457593	24.065751	32.96238	8.461067	21.369668	7.230443	35.878146
SOCS5	4.814851	13.457799	2.471158	6.838755	1.635204	6.506493	7.873142	12.602738
SOCS5P1	0	0	0	0	0	0	0	0
SOCS5P2	0	0	0	0	0	0	0	0
SOCS5P3	0	0	0	0	0	0	0	0
SOCS5P4	0	0.191629	0	0.110397	0	0.112129	0	0
SOCS5P5	0	0	0	0	0	0	0	0
SOCS6	6.281722	25.445362	2.053006	15.9357	7.433123	32.315537	2.087512	37.162191
SOCS6P1	0	0	0	0.056039	0	0	0	0
SOCS7	6.065778	18.079604	22.159788	24.103343	13.786734	20.354318	51.317853	18.128385
SOD1	0	3.079835	0	2.271732	12.488756	0.436624	0	1.698183
SOD1P1	0	0	0	0	0	0	0	0
SOD1P2	0	0	0	0	0	0	0	0
SOD1P3	0	0	0	0	0	0	0	0
SOD2	204.677308	151.891718	298.719649	242.387481	340.740417	255.372862	241.123327	186.639481
SOD2P1	0	0	0	0	0	0	0	0

SOD3	0	2.463116	4.545857	2.781826	14.13799	0.626971	0	1.347213
SOHLH1	0	0	0	0	0	0	0	0
SOHLH2	0	0	0	0.21637	0	0	0	0
SON	41.021904	86.871979	15.388874	126.481588	254.471649	191.988457	218.413575	172.710121
SORBS1	7.134313	20.107245	3.946373	20.867203	0	7.316707	0	4.65679
SORBS2	0.989806	6.39341	4.737472	13.273469	0	49.892523	51.670937	65.645398
SORBS3	50.163961	86.164901	96.774056	75.899187	82.832703	109.543322	138.44755	83.27085
SORCS1	0	0	0	0	0	0	0	0
SORCS2	0	2.250096	1.899024	5.806771	19.970331	0.219741	0	0
SORCS3	0	0	0	0	0	0	0	0
SORD	87.915174	78.757731	59.987379	89.397529	149.897056	107.955888	70.741772	93.661361
SORD2P	0	0	0	0	0	2.811185	0	0
SORL1	42.563152	49.298864	106.637635	113.596545	77.098624	51.094081	53.079919	23.252572
SORT1	23.154237	26.420172	35.813757	13.453588	62.784653	9.620145	71.905143	9.28042
SOS1	0	30.410928	10.075226	24.579258	9.835402	26.212991	38.384263	35.043355
SOS2	22.750197	27.959913	16.56869	18.915132	14.72716	10.256687	19.184886	30.171052
SOST	0	0	0	0	0	0.222059	0	0.120256
SOSTDC1	0	0	0	0	0	0	0	0
SOWAHC	35.467649	52.274099	28.029641	37.667218	39.346763	59.315264	45.428751	76.220764
SOWAHCP2	0	0	0	0	0	0	0	0
SOWAHCP5	0	0	0	0.677794	0	0	0	0
SOWAHD	2.201993	0	0	0.533329	0	0.433533	0	0.623422
SOX10	0	0	0	0	0	0	0	0
SOX11	0	0	0	0	0	0	0	0
SOX13	26.171082	41.394287	19.746185	48.516416	24.990458	82.019357	24.443294	40.530199
SOX15	0	0.381404	0	0	0	10.412663	34.399915	3.889286
SOX17	0	0.185735	1.303465	0.248188	0	0.36168	0	0.176182
SOX18	0	0.713964	0	1.139227	0	0.371054	0	0.151654
SOX2	6.76846	4.43336	0	3.162953	8.214045	7.532702	0	9.212419
SOX30	0	0.447646	0	0.515205	0	0.209483	0	0.171925
SOX5	0	0	0	0.185874	0	0	0	0
SOX5P1	0	0	0	0	0	0	0	0
SOX6	0.365468	0.15748	0.657706	0.7101	0.772757	2.403826	0	1.505064

SOX7	26.467855	36.857618	24.232306	38.648201	9.606476	18.514842	3.538068	24.906402
SOX8	0	1.158404	0	2.762979	0	0.108195	0	0.087311
SOX9	24.391903	18.87232	19.670829	17.39391	15.103512	14.922036	27.22007	17.667362
SP1	42.466511	36.709586	59.863851	37.399804	25.59766	19.837538	28.390317	16.089536
SP100	53.966083	140.018673	50.483184	126.440564	83.653526	223.569762	533.354255	257.505824
SP110	26.532303	0.290582	12.341401	7.241436	0.327269	11.060181	98.58066	4.192694
SP140	0	10.584005	6.931077	6.980101	1.950841	15.410113	2.541149	13.618012
SP140L	0	0.539058	11.35972	5.525098	23.303983	4.698167	23.995158	3.927804
SP2	9.9801	14.286745	11.923128	11.993839	53.76191	22.692629	6.557491	20.345225
SP3	0	29.283627	0	24.27661	32.350269	85.485382	44.451201	34.764293
SP3P	0	0	0	0	0	0	0	0
SP4	8.02238	21.224673	4.674984	10.699294	18.935508	18.000513	10.563468	11.640692
SP5	0	0	0	0	0	0	0	0
SP6	0	3.730553	2.345981	4.036353	1.611002	5.752405	8.905655	3.234365
SP7	0	0	0	0	0	0	0	0
SP8	0	0	0	0.149771	0	0	0	0
SP9	1.329707	0.056574	0	0.485522	0	0.066082	0	0
SPA17	4.662565	4.134668	1.782463	0.827941	0.977416	0.262769	2.577678	0.598607
SPA17P1	0	0	0	0	0	0	0	0
SPAAR	0	0	0	0	0	0	0	0
SPACA1	0	0	0	0	0	0	0	0
SPACA3	0	0	0	0	0	0	0	0
SPACA4	0	0	0	0	0	0.38547	0	0
SPACA5	0	0	0	0	0	0	0	0
SPACA6	0	0.623046	0	1.490966	3.687841	0	0	1.156427
SPACA7	0	0	0	0	0	0	0	0
SPACA7BP	0	0	0	0	0	0	0	0
SPACA9	4.593766	14.490729	11.379452	13.892379	0	9.334836	0	7.442203
SPACDR	0	0.463436	0	0.63714	0	1.135762	0	2.023522
SPADH	0	0	0	0	0	0	0	0
SPAG1	9.727113	14.849109	7.933445	12.488927	1.167476	13.869985	0	12.614477
SPAG11A	0	0	0	0	0	0	0	0
SPAG11B	0	0	0	0	0	0	0	0

SPAG16	5.370291	18.466515	37.156807	3.434883	17.881688	3.430504	1.868069	20.422313
SPAG17	0	1.509226	0	0	0	0	0	1.535336
SPAG4	12.520195	16.236734	0	24.040579	0	0.234171	0	0
SPAG5	66.130481	98.846252	28.260631	91.093779	37.143609	152.930242	54.066282	130.900929
SPAG6	0	0	0	0	0	0	0	0
SPAG7	54.008196	64.313041	57.559082	67.649687	76.774387	65.432092	44.991609	42.935691
SPAG8	0	0.719799	0	0.598392	0	0.565832	0	0.539938
SPAG9	42.807069	56.642253	73.774779	49.830856	152.551295	89.00196	191.355994	67.321003
SPAM1	0	0	0	0	0	0	0	0
SPANXA2	0	0	0	1.33485	0	0	0	0
SPANXB1	0	0	0	0	0	0	0	0
SPANXD	0	0	0	0	0	0	0	0
SPANXN3	0	0	0	0	0	0	0	0
SPANXN4	0	0	0	0	0	0	0	0
SPARC	18.196389	14.273222	19.332924	14.58809	216.79251	173.804228	82.928855	107.79531
SPARCL1	0	0	0	0	0	0	0	0
SPART	19.194189	67.116439	22.313154	55.844662	101.088688	52.504681	48.029121	71.012753
SPAST	0	17.086899	15.319387	11.062021	5.529008	10.603526	0	13.640896
SPATA1	0	0.323706	0	0.739686	0	0	0	0
SPATA12	0	0.466388	0	0.53462	0	0.077862	0	0
SPATA13	7.241109	8.550884	9.258259	15.038893	42.836887	20.717769	30.695171	12.321504
SPATA16	0	0	0	0	0	0	0	0
SPATA17	10.894672	0.337795	0	4.174258	0	1.262579	0	0.786586
SPATA18	1.197363	1.538336	0	1.244704	0	1.357353	0	0.949544
SPATA19	0	0	0	0	0	0	0	0
SPATA2	15.517136	30.567894	11.727767	29.109701	7.979959	34.383902	7.155008	26.958621
SPATA20	18.537898	22.344457	18.811435	22.67749	43.754519	54.394891	33.327858	48.46677
SPATA20P1	0	0	0	0.282279	0	0	0	4.697568
SPATA21	0	0.117742	0	0.333263	0	0.137417	0.223618	0
SPATA22	0	0	0	0	0	0	0	0
SPATA24	0	6.922088	0	7.329901	2.70493	7.709757	0	6.91684
SPATA2L	1.467792	9.101891	17.895567	10.569802	14.814391	15.804896	0.355895	8.771937
SPATA2P1	0	0	0	0	0	0	0	0

SPATA3	0	0	0	0.852956	0	0	0	0
SPATA31A1	0	0	0	0	0	0	0	0
SPATA31A6	0	0	0	0	0	0	0	0
SPATA31A7	0	0	0	0	0	0	0	0
SPATA31B1P	0	0	0	0	0	0	0	0
SPATA31D1	0	0	0	0	0	0	0	0
SPATA31D2P	0	0	0	0	0	0	0	0
SPATA31D5P	0	0	0	0	0	0	0	0
SPATA31E2P	0	0	0	0	0	0	0	0
SPATA31E3P	0	0	0	0	0	0	0	0
SPATA31F2P	0	0	0	0	0	0	0	0
SPATA31F3	0	0	0	0	0	0	0	0
SPATA31G1	0	0	0	0	0	0	0	0
SPATA32	0	0	0	0	0	0	0	0.23642
SPATA33	6.026632	31.93882	0	26.12918	7.036575	33.899329	14.734869	45.473766
SPATA4	0	0.436024	11.826011	0	0	0.621837	0	0
SPATA45	0	0	8.330051	0	0	0	0	0
SPATA46	0	0	0	0	0	0	0	0
SPATA6	0	5.42926	18.506667	11.748411	0.801381	0.465785	0	1.262841
SPATA6L	19.073449	1.979886	0	3.570669	0	1.429992	0	0.181273
SPATA7	14.768348	10.552961	22.806447	9.146559	0.973164	5.337172	0	5.113214
SPATA8	0	0	0	0	0	0	0	0
SPATA9	0	0.974011	0	0.175576	0	0.286995	0	2.50847
SPATC1	0	0	0	0	0	0	0	0
SPATC1L	0	0	0	1.064637	0	0	0	0.513275
SPATS1	0	0	0	0	0	0	0	0
SPATS2	49.686342	130.869613	52.983772	79.3645	13.986011	31.841557	38.665332	46.094164
SPATS2L	113.426955	299.589032	88.37673	249.414557	123.435967	521.612084	158.723902	445.318398
SPC24	12.132082	12.728523	13.467	8.5605	15.575047	14.734727	0	3.814303
SPC25	8.162805	21.17443	33.815875	6.435674	18.616748	9.580137	0	36.759736
SPCS1	144.307058	115.287341	131.036957	80.420952	87.635567	40.093685	145.598448	88.133934
SPCS2	307.16931	220.583697	436.3578	296.272552	251.541045	127.752161	643.759619	95.946635
SPCS2P1	0	0	0	0	0	0	0	0

SPCS2P2	0	0	0	0	0	0	0	0
SPCS2P3	0	0	0	0	0	0	0	0
SPCS2P4	0	55.218877	45.01161	26.905313	0	0	0	14.440366
SPCS3	70.435997	96.740827	94.589394	81.851449	71.596428	60.569246	41.826946	66.82206
SPDEF	0	0	0	0.136386	0	0.277642	0	0
SPDL1	29.565364	63.400211	31.988095	40.059986	61.661959	69.184727	86.485177	86.516181
SPDYA	0	0	0	0	0	0	0	0
SPDYE1	0	0	0	0	0	0.127144	0	0
SPDYE10	0	0	0	0	0	0	0	0
SPDYE16	0.967921	0	0	0.031783	0	0	0	0
SPDYE19P	0	0	0	0	0	0	0	0
SPDYE20P	0	0	0	0	0	0	0	0
SPDYE22P	0	0	0	0	0	0	0	0
SPDYE2B	0	0.12786	0	0	0	0	0	0
SPDYE3	0	0	0	0	0	0	0	0
SPDYE4	0	0	0	0	0	0	0	0
SPDYE7P	0	0	0	0	4.845316	0	0	0
SPDYE9	0	0	0	0	0	0	0	0
SPECC1	35.753028	71.503554	55.882782	83.87063	22.257109	87.169218	22.552015	45.757716
SPECC1L	32.524578	34.741727	20.57731	52.518894	59.840146	59.791243	46.194332	67.361348
SPECC1L-AD	0	0.244808	0	0.236056	1.017313	1.591935	5.670078	1.029431
SPECC1P1	0	0	0	0	0	0	0	0
SPECC1P2	0	0	0	0	0	0	0	0
SPEF1	0	0	0	1.311655	0	0.888259	0	0.091319
SPEF2	3.3267	9.252589	1.877446	3.576139	0	1.503459	33.224795	1.170438
SPEG	5.756839	7.506284	6.995994	7.191084	19.326399	14.134948	0	7.852139
SPEGNB	0	0	0	0.838393	0	3.660405	0	1.775168
SPEM1	0	0	0	0	0	0	0	0
SPEM2	0	0	0	0.052076	0	0.139597	0	0
SPEM3	0	0	0	0	0	0	0	0
SPEN	36.205558	56.853398	49.009727	57.648094	32.697497	86.649967	38.708815	56.794476
SPESP1	0	0	0	0	0	0	0	0
SPG11	12.775655	18.826204	16.904993	10.226427	14.599677	15.034221	88.364757	8.873478

SPG21	104.535748	124.86866	88.738646	85.446383	60.910008	58.714142	63.223977	129.467358
SPG7	59.060658	39.826702	52.714971	55.449083	117.962732	52.067168	54.22853	44.136822
SPHK1	2.487459	10.223316	6.783216	11.818653	11.974168	16.64247	20.06214	15.28556
SPHK2	4.096261	5.11848	8.415719	4.239631	13.819314	10.344113	11.920528	9.40589
SPHKAP	0	0	0	0	0	0	0	0
SPI1	0	0	0	0	0	0	0	0
SPIB	0	0	0	0.098316	0	0	0	0
SPICE1	29.838105	54.638813	50.991446	45.971126	22.166434	16.767203	10.712527	33.312807
SPICP1	0	0	0	0	0	0	0	0
SPICP2	0	0.227287	4.9296	0.26808	0	0	0	0
SPICP3	0	0	0	0	0	0	0	0
SPICP4	5.897116	0.473492	0	0	0	0	0	0
SPICP5	0	0	0	0	0	0	0	0
SPIDR	59.06106	97.534355	43.759196	104.339271	76.162559	51.610994	82.137547	45.499312
SPIN1	88.03777	148.493084	36.408043	113.12098	136.480678	192.574902	70.768097	252.472837
SPIN2A	0	0	0	0	0	0	0	0
SPIN2B	0	8.649603	0	4.881054	2.475279	10.45145	0	4.837637
SPIN2P1	0	0	0	0	0	0	0	0
SPIN3	6.713544	15.136818	2.765071	11.779751	22.040898	11.736806	0	15.107807
SPINDOC	19.58387	36.08212	33.372591	33.788814	13.141065	67.68979	50.849152	40.246211
SPINK1	0	0	0	0	0	0	0	0
SPINK13	0	0	0	0	0	0	0	0
SPINK14	0	0	0	0	0	0	0	0
SPINK2	0	2.302244	10.40006	0.566591	0	0	0	0
SPINK4	0	0	0	0	0	0	0	0
SPINK5	1.265635	2.739381	0	0.83878	0.373786	3.307825	3.75339	0.639666
SPINK6	0	0.39685	0	0	32.357234	2.038707	0	2.634799
SPINK7	0	0	0	0.397487	0	0	0	0
SPINK9	0	0	0	0	0	0.348654	0	0
SPINT1	108.326942	31.817532	20.580673	39.291957	2.051334	31.916052	64.361409	19.968292
SPINT2	200.341075	431.465534	222.925716	522.363316	288.365173	502.46096	271.130902	409.558383
SPINT5P	0	0	0	0	0	0	0	0
SPIRE1	12.9627	41.150473	29.538307	63.563105	59.181642	48.717579	0	45.992488

SPIRE2	3.103215	4.519818	6.334303	5.770499	2.748404	3.597105	0	3.158665
SPMAP1	0	0	0	0	0	0	0	0
SPMAP2	6.443028	9.084209	2.852335	10.669853	0	0.431194	0	0.261972
SPMAP2L	0	0	0	0	0	0	0	0
SPMIP1	0	0.18239	0	0.124784	0	0.21284	0	0.239547
SPMIP10	0	0	0	0	0	0	0	0
SPMIP11	0	0	0	0	0	0	0	0
SPMIP2	0	0	0	0	0	0	0	0
SPMIP3	0	0	0	0	0	0	0	0
SPMIP3P1	0	0	0	0	0	0	0	0
SPMIP4	2.636576	7.377869	3.562169	8.266261	1.311058	3.15592	8.693063	1.51261
SPMIP5	0	0	0	0	0	0	0	0
SPMIP6	0	0	0	0.179414	0	0	0	0
SPMIP8	0	0.777644	0	0.211375	0	0.215634	0	0.17504
SPMIP9	0	0	0	0	0	0	0	0
SPN	0	0	0	0	0	0	0	0
SPNS1	10.342448	28.661532	20.948067	31.256888	40.579045	31.125434	12.761368	27.919678
SPNS2	0	1.884499	0	1.157604	7.823232	1.660235	1.431496	2.152297
SPNS3	0	0	0	0	0	0	0	0
SPO11	0	0	0	0	0	0	0	0
SPOCD1	12.015586	18.781687	35.402587	30.094316	1.205746	8.291569	0	4.372214
SPOCK1	28.516752	111.056257	14.595707	180.80252	0	63.933264	10.403464	27.721634
SPOCK2	0	0	0	0.030569	2.312788	1.002126	0	1.283665
SPOCK3	0	0.207508	3.54727	2.692896	0	0	0	0
SPON1	0	0	0	0.015822	0	0	0	0
SPON2	0	0.284844	0	0.666376	0	0.11111	0	0.173431
SPOP	33.09865	99.282874	44.466992	102.755101	65.93133	58.158805	61.257991	98.189557
SPOPL	12.823464	27.492213	13.819608	20.186409	13.069299	24.047496	49.073197	23.589571
SPOPLP1	0	0	0	0	0	0	0	0
SPOPLP2	0	0	0	0	0	0	0	0
SPOPLP3	0	0	0	0	0	0	0	0
SPOUT1	37.692018	27.2395	15.135406	37.319255	12.2556	48.122903	3.576983	34.237187
SPP1	0	3.08943	0	0.087369	0	0	0	0

SPP2	0	0	0	0	0	0	0	0
SPPL2A	27.546617	28.055545	43.930528	15.414497	80.3339	6.436743	11.389459	27.888595
SPPL2B	4.730254	6.42086	4.866516	7.200502	8.994133	11.749387	8.400771	5.066962
SPPL2C	0	0	0	0	0	0	0	0
SPPL3	21.954699	39.912553	17.183924	25.507891	45.149684	19.915255	24.3851	39.291153
SPR	42.921214	41.049735	20.197908	28.138312	32.567818	23.280342	24.342733	21.582386
SPRED1	17.346701	54.206928	28.735006	41.552681	25.265652	44.500657	5.423552	52.472164
SPRED2	27.926289	85.785424	27.419323	89.336395	15.135805	101.221844	122.063817	77.088882
SPRED3	0	1.501253	3.08091	2.814141	1.630939	1.797818	0	5.344934
SPRING1	15.986235	18.241466	11.354006	15.551858	41.792327	63.93864	12.40768	55.502378
SPRING1P1	0	0	0	0	0	0	0	0
SPRING1P2	0	0	0	0	0	0	0	0
SPRING1P3	0	0	0	0	0	0	0	0
SPRN	0	0.1371	0	0	0	0	0	0
SPRR1B	7.23367	4.858569	0	1.192376	3.774852	7.763767	0	12.614922
SPRR2A	0	0	0	0	0	0	0	0
SPRR2C	0	0	0	0	0	0	0	0
SPRR2D	0	0	0	0	0	0	0	0.527922
SPRR2E	0	0	0	0.150468	0	0	0	0.525723
SPRR2F	0	0	0	0.151648	0	0	0	0
SPRR2G	0	0	0	0	0	0	0	0
SPRR3	0	1.704095	0	0	0	1.480089	0	1.079876
SPRR4	0	0	0	0.263963	0	0	0	0
SPRTN	17.496984	20.309071	10.225304	27.229611	48.474771	20.041022	1.708129	17.397806
SPRY1	7.609674	15.053042	3.906811	30.669608	3.072959	18.564459	0	30.97507
SPRY2	10.49446	62.523023	5.361393	39.125856	4.744995	20.514942	0.726898	29.402304
SPRY3	0.35674	0.230606	0.321016	0.576375	0	0.573513	0	0.814648
SPRY4	5.303616	6.571122	11.630122	8.505631	4.189995	4.022477	3.391	6.425445
SPRYD3	26.668098	23.206373	25.965467	27.830496	11.322213	16.454963	1.850233	8.769101
SPRYD4	2.97763	2.594116	1.072139	1.990636	0	1.37717	1.816909	1.180642
SPRYD7	4.387746	5.735788	2.948824	5.1935	0.506525	6.391716	0	4.008359
SPRYD7P1	0	0	0	0	0	0	0	0
SPSB1	18.696975	79.604724	17.453503	77.830795	12.975541	85.802245	0.524176	56.251258

SPSB2	6.171783	3.030153	2.136253	0.58941	0.811523	0.536877	0	0.604511
SPSB3	14.749453	8.609081	14.90348	9.781064	5.24609	12.892493	0	9.798877
SPSB4	2.009649	1.741414	0	1.208982	0	0.056481	0	0.324801
SPTA1	0	0	0	0	0	0	0	0
SPTAN1	38.79996	43.856796	53.725511	55.575848	75.566427	91.298529	179.448703	74.305674
SPTB	0	0.055111	0	0.181039	0.084331	0.705546	0	0.436652
SPTBN1	48.699135	75.227442	62.102484	122.39569	62.789708	226.949445	103.518098	177.367887
SPTBN2	0	8.434173	0	0.104703	0	18.666709	0	0
SPTBN4	1.852554	1.069156	0	2.530352	1.687736	0.753615	0	1.257937
SPTBN5	0.273715	0.143663	0	0.100556	0.072356	0.165168	1.403136	0.010961
SPTLC1	25.46383	10.113289	21.701375	20.11846	59.566711	6.036143	7.941182	14.923462
SPTLC1P1	0	0	0	0	0	0	0	0
SPTLC1P2	0	0	0	0	0	0	0	0
SPTLC1P3	0	0	0	0	0	0	0	0
SPTLC1P4	0	0	0	0	0	0	0	0
SPTLC1P5	0	0	0	0	0	0	0	0
SPTLC2	0	34.269789	0	37.188534	0	50.724777	30.600325	38.873269
SPTLC3	1.049352	16.447077	0.471683	8.306085	1.247699	16.446679	1.27896	14.010653
SPTSSA	25.435255	19.223078	21.630923	14.586759	21.14082	14.798908	9.569833	13.565504
SPTSSB	0	0	0	0.457466	0	0	0	0
SPTY2D1	22.690893	51.213276	18.823791	62.354894	60.694672	62.809165	50.727416	52.768849
SPX	0	3.016304	1.872988	1.974345	0	0	0	1.389097
SPZ1	0	0	0	0	0	0	0	0
SQLE	124.938597	431.639426	92.48639	356.404839	93.605605	204.965629	113.891312	277.005361
SQOR	104.739315	109.685605	134.327182	128.542133	87.016696	67.6223	0	73.636657
SQSTM1	277.443993	254.037701	348.119304	417.929933	172.590512	275.745149	182.219209	189.473823
SQSTM1P1	0	0	0	0	0	0	0	0
SRA1	27.704693	39.059848	28.411962	28.966372	63.398752	43.2671	39.097271	36.513922
SRARP	0	0	0	0	0	0	0	0
SRBD1	17.192062	13.936653	14.610553	17.607463	24.369388	12.731464	13.865921	10.978571
SRC	65.891686	81.656889	42.065652	82.567528	37.933887	60.158834	70.795234	43.712975
SRCAP	0	4.640098	6.794199	3.365247	7.816473	6.495802	0	6.173412
SRCIN1	2.832313	3.567475	4.414506	5.729621	1.122175	3.467405	0	2.107572

SRD5A1	14.356817	4.668769	8.147224	2.985038	0.60785	1.055262	8.174113	1.640715
SRD5A1P1	0	0	0	0	0	0	0	0
SRD5A2	0	0	0	0	0	0.072748	0	0
SRD5A2P1	0	0	0	0	0	0	0	0
SRD5A3	21.728294	10.026542	9.433871	3.947334	3.649473	2.783476	0.197832	4.929159
SRD5A3P1	0	0	0	0	0	0	0	0
SREBF1	39.490998	39.907632	24.140887	45.72936	285.083088	157.328505	110.89677	44.252762
SREBF2	75.560956	91.615921	97.48953	93.289608	62.960433	70.894793	12.764271	50.053163
SREK1	31.786997	28.464772	40.91808	31.837446	34.154018	24.415121	55.691474	25.9149
SREK1IP1	14.12497	36.03299	7.621332	23.433097	52.831915	29.633954	36.305373	44.276777
SREK1IP1P1	0	0.905873	0	0	0	0	0	0
SREK1IP1P2	0	0	0	0	0	0	0	0
SRF	17.132641	20.725094	16.079055	18.025037	19.333077	34.102687	23.505307	25.263662
SRFBP1	0	0	0	0	0	0	0	1.005792
SRGAP1	0.829349	28.749147	1.344594	20.715527	0.162522	55.367826	275.989356	22.984924
SRGAP2	24.501234	43.975508	4.536461	49.061715	32.260219	104.39625	10.326902	41.979795
SRGAP2B	4.378039	21.059967	9.822642	69.716764	1.676308	12.077548	2.176138	4.734067
SRGAP2C	0	24.567442	0	53.309552	0	40.141348	0	30.46494
SRGAP2D	0	10.371949	0	14.581725	0	9.735509	0	5.079697
SRGAP3	0	2.874319	0	2.19752	3.262365	9.800588	0	6.903015
SRGN	0	0.126909	0	0	16.022237	0	0	0
SRGNP1	0	0	0	0	0	0	0	0
SRI	178.734615	230.171573	115.779755	212.385888	107.517773	110.677224	44.424324	116.538758
SRIP1	0	0	0	0	0	0	0	0
SRIP2	0	0	0	0	0	0	0	0
SRIP3	0	0	0	0	0	0	0	0
SRL	0	0	0	0.038194	0	0.293783	0	0.094198
SRM	64.420726	49.544036	41.550706	40.466505	77.016057	93.749621	96.478866	111.417729
SRMP1	0	0	0	0	0	0	0	0
SRMP2	0	0	0	0	0	0	0	0
SRMP3	0	0	0	0	0	0	0	0
SRP14	44.207683	250.492521	144.036518	212.189158	233.895904	278.688105	302.077467	176.803278
SRP14P1	0	0	0	0	0	0	0	0

SRP14P2	0	0	0	2.060447	0	0.784203	0	0
SRP14P3	0	0	0	0	0	0	0	0
SRP14P4	0	0	0	0	0	0	0	0
SRP14P5	0	0	0	0	0	0	0	0
SRP19	145.974899	176.121383	73.738714	128.733368	127.342051	73.552843	74.392334	138.558395
SRP54	80.237955	84.722062	71.705644	81.053077	56.616121	41.809891	74.343815	103.153831
SRP54-AS1	0	0	0	0.389905	0	1.143705	0	0
SRP68	73.860393	151.053026	63.039582	162.833985	65.647144	98.941433	50.886851	105.519428
SRP68P1	0	0	0	0	0	0	0	0
SRP68P2	0	0	0	0	0	0	0	0
SRP68P3	0	0	0	0	0	0	0	0
SRP72	121.356462	87.820378	89.740407	125.72294	156.181999	107.621166	101.819881	79.780381
SRP72P1	0	0	0	0	0	0	0	0
SRP72P2	0	0	0	0	0	0	0	0
SRP9	86.96147	144.49183	84.88412	132.797325	127.56453	141.600814	37.217003	122.778793
SRP9P1	0	177.910673	0	107.115157	0	83.441547	0	127.342691
SRPK1	57.521413	127.697363	60.478419	116.263603	88.462171	121.18484	132.349453	100.437567
SRPK2	29.501206	32.551458	51.480837	36.972729	52.731459	35.973552	116.743399	35.595492
SRPK2P	0	0	0	0	0	0	0	0
SRPK3	0	0	0	0.172323	0	0.088812	0	0
SRPRA	41.265375	39.344801	56.43039	45.508817	119.334728	80.691428	106.836614	82.943201
SRPRB	0	47.436108	52.441357	42.361384	0	10.337745	17.129345	20.221238
SRPX	4.089703	5.124741	6.781372	6.001631	4.162328	3.384325	0	1.838921
SRPX2	26.548217	66.899451	48.599841	141.520382	32.706084	17.914767	9.870182	10.396947
SRR	36.431098	23.74447	3.680809	17.815769	20.402641	8.260477	6.986577	18.484352
SRRD	18.074927	41.987701	44.436021	36.909105	74.735156	58.67903	88.869188	80.974326
SRRM1	59.735605	77.316562	60.203369	75.653938	165.070933	159.13502	76.88794	138.804367
SRRM1P2	0	0	0	0	0	0	0	0
SRRM1P3	0	0	0	0	0	0	0	0
SRRM2	114.281595	132.75447	164.203874	183.342043	338.023515	308.699298	439.526132	185.402582
SRRM3	8.014482	6.510374	5.728833	10.109033	0	4.146664	0	3.102212
SRRM4	0	0	0	0	0	0	0	0
SRRM5	0	0.360534	0	0	0	0.286309	0	0

SRRT	43.168789	95.636631	40.413083	88.272172	151.113647	153.854614	30.516148	101.195341
SRSF1	203.149192	256.598808	171.460581	218.317251	355.3386	251.910748	347.203538	249.377235
SRSF10	81.531396	41.560331	89.791926	42.713279	55.190243	45.862834	147.733444	60.49716
SRSF10P1	0	0	0	0	0	0	0	0
SRSF10P2	0	0	0	0	0	0	0	0
SRSF11	67.159042	53.087619	39.340914	62.277539	95.50751	108.770772	73.722686	68.243722
SRSF12	0	0.277122	0	0.519415	0	0.521305	0	1.172071
SRSF1P1	0	0	0	0	0	0	0	0
SRSF2	8.269199	74.811712	21.65023	62.28326	105.216461	148.628997	75.969974	90.947807
SRSF2P1	0	0	0	0	0	0	0	0
SRSF3	208.645281	261.93213	200.1967	208.310586	268.56097	246.846926	352.860181	267.615812
SRSF3P1	0	0	0	0	0	0	0	0
SRSF3P2	0	0	0	0	0	0	0	0
SRSF3P4	0	0	0	0	0	0	0	0
SRSF3P5	0	0	0	0	0	0	0	0
SRSF3P6	0	0	0	0	0	0	0	0
SRSF4	35.971957	105.498958	51.930712	128.94795	80.977473	159.08809	214.009321	118.826083
SRSF5	36.324481	262.428113	232.229508	326.001439	152.504335	310.286395	536.72759	213.268043
SRSF6	102.429995	164.075937	96.765431	168.083029	150.530558	198.418521	173.077984	249.643509
SRSF6P1	0	0	0	0	0	0	0	0
SRSF6P2	0	0.163334	0	0.187122	0	0	0	0
SRSF7	108.532538	166.411235	114.735348	153.379197	188.924862	173.871305	147.415476	217.975857
SRSF8	39.741654	45.683808	39.089873	34.66478	17.77235	7.180331	21.004197	16.100336
SRSF8BP	0	0	0	0	0	0	0	0
SRSF8CP	0	0	0	0	0	0	0	0
SRSF9	293.867914	727.621996	235.031936	558.072681	304.212333	869.946164	338.320693	1065.33797
SRSF9P1	0	0	0	0.21177	0	0	0	0
SS18	96.24805	54.401431	44.914298	57.069649	34.536656	51.656865	16.447148	50.275051
SS18L1	19.937751	10.523067	9.077222	14.906288	3.139065	11.855295	10.5484	10.470068
SS18L2	0	0	0	0	0	0	0	1.288932
SS18L2P1	0	0	0	0	0	0	0	0
SS18L2P2	0	0	0	0	0	0	0	0
SSB	136.665221	150.60321	112.96297	101.232198	181.034188	119.891328	197.290382	216.112485

SSBL2P	0	0	0	0	0	0	0	0
SSBL3P	0	0	0	0	0	0	0	0
SSBL4P	2.980831	0.496554	0	0.503123	0	1.018312	0	0.484193
SSBL5P	0	0	0	0	0	0	0	0
SSBL6P	0	0	0	0	0	0	0	0
SSBP1	242.331056	333.675737	207.579574	263.021277	249.586724	235.955338	197.188583	310.96224
SSBP2	5.293412	10.810133	0	7.008133	0	6.346219	0	4.016039
SSBP3	0	0	0	8.36726	15.678475	12.62652	1.439517	0
SSBP3P1	0	0	0	0	0	0	0	0
SSBP3P2	0	0	0	0	0	0	0	0
SSBP3P3	0	0	0	0	0	0	0	0
SSBP3P4	0	0	0	0	0	0	0	0
SSBP3P5	0	0	0	0	0	0	0	0
SSBP3P6	0	0	0	0	0	0	0	0
SSBP4	34.022507	81.391155	59.511458	58.749929	33.606389	82.55412	38.892067	85.191515
SSC4D	2.409369	2.037194	2.685249	1.180764	4.780711	0.591087	0	0.861979
SSC5D	0	0	0.590697	0	0.293611	0.055417	0	0
SSH1	44.019031	71.340202	45.39378	59.696022	84.375803	210.731133	117.620722	161.375152
SSH2	32.06998	51.296605	41.538372	63.563737	46.641933	45.407637	38.018686	32.099406
SSH3	64.676257	53.420591	35.696161	55.26896	38.834879	67.698531	42.514515	45.221967
SSMEM1	0	0	0	0	0	0	0	0
SSNA1	51.030117	83.93989	50.736715	75.451186	8.401477	113.949093	2.765423	87.264376
SSPN	0	12.496167	3.397664	4.847354	0	1.438811	0	1.215402
SSR1	110.863249	104.650823	99.082045	102.047016	111.716266	73.265106	68.868721	84.970926
SSR1P1	0	0	0	0	0	0	0	0
SSR1P2	0	0	0	0	0	0	0	0
SSR2	206.272563	101.160098	235.230437	169.519873	161.30566	82.988865	183.193187	77.900112
SSR3	162.320738	101.099236	153.357937	70.611499	47.242164	34.196872	99.139183	57.429241
SSR4	0	7.713225	20.283543	5.870937	0	1.62082	0	3.54122
SSR4P1	0	4.670958	0	0	0	0	0	0
SSRP1	159.537501	333.474513	113.026209	340.647981	251.983883	871.779081	220.027805	513.681166
SST	0	0	0	0	0	0	0	0
SSTR1	0	0	0	0.018302	0.197908	0.337321	0	0.270724

SSTR2	0.42018	0.162832	0	0.483262	0	0.021094	0	0.033678
SSTR3	0	0	0	0.113406	0	0	0	0.093223
SSU72	115.303132	163.947089	89.633298	137.870192	73.191888	217.611765	151.604486	248.395432
SSU72P1	0	0	0	0	0	0	0	0
SSU72P6	0	0	0	0	0	0	0	0
SSUH2	0	0	0	0.528771	0	0.306659	0	0.127928
SSX11P	0	0	0	0	0	0	0	0
SSX13P	0	0	0	0	0	0	0	0
SSX14P	0	0	0	0	0	0	0	0
SSX18P	0	0	0	0	0	0	0	0
SSX19P	0	0	0	0	0	0	0	0
SSX2	0	0	0	0	0	0	0	0
SSX20P	0	0	0	0	0	0	0	0
SSX21P	0	0	0	0	0	0	0	0
SSX2B	0	0	0	0	0	0	0	0
SSX2IP	29.183116	82.122692	33.489073	38.844502	15.132039	23.63511	9.09113	48.924694
SSX2IPP1	0	0	0	0	0	0	0	0
SSX3	0	0	0	0	0	0	0	0
SSX4	0	0	0	0	0	0	0	0
SSX5	0	0	0	0	0	0	0	0
SSX6P	0	0	0	0	0	0	0	0
SSX9P	0	0	0	0	0	0	0	0
SSXP10	0	0	0	0	0	0	0	0
ST13	146.752623	226.265067	144.25745	255.056158	188.601053	175.589182	36.039329	156.60135
ST13P10	0	0	0	0.084483	0	0	0	0
ST13P11	0	0	0	0.139352	0	0	0	0.484368
ST13P12	0	0	0	0	0	0	0	0
ST13P13	0	0	0	0	0	0	0	0
ST13P14	0	0	0	0	0	0	0	0
ST13P15	0	0	0	0	0	0	0	0
ST13P16	0	0	0	0	0	0	0	0
ST13P18	0	0.159007	0	0	0	0.362979	0	0
ST13P19	0	0	0	0	0	0	0	0

ST13P2	0	0	0	0	0	0	0	0
ST13P20	0	0	0	0	0	0.346794	0	0
ST13P21	0	0	0	0	0	0	0	0
ST13P3	0	0	0	0	0	0	0	0
ST13P4	0	0	3.146243	0.259506	0	0	0	0
ST13P5	0	0	0	0	0	0	0	0
ST13P6	0	0	0	0	0	0	0	0
ST13P7	0	0.149788	0	0	0	0	0	0.143225
ST14	37.361985	85.89768	35.306427	128.107363	62.910908	382.364273	41.730555	214.570497
ST18	0	0	0	0.210382	0	0	0	0
ST20	0	0.494056	10.1696	1.308839	11.831065	6.463899	0	10.717777
ST20-MTHFS	0	1.989313	0	14.114706	0	0.68393	0	0.812759
ST3GAL1	7.615191	72.641969	9.214747	61.518749	11.483977	65.140199	4.706525	45.423628
ST3GAL1P1	0	0	0	0	0	0	0	0
ST3GAL2	11.08098	44.059233	3.298607	31.757162	22.735237	54.574588	1.788812	49.633422
ST3GAL3	0	2.835236	0	7.583531	0	4.796624	0	5.990546
ST3GAL4	42.51769	35.367198	24.89467	18.878855	22.581618	39.237452	103.210698	22.40314
ST3GAL5	1.847125	6.483047	9.124683	5.49115	13.744311	1.394903	0	1.649016
ST3GAL6	0	7.894678	0	2.82831	6.385316	1.829241	0	5.495797
ST6GAL1	7.363617	6.760447	4.809628	20.491024	9.095866	18.243166	0	13.69993
ST6GAL2	0	0.180122	0	0	0	0	0	0
ST6GALNAC1	0	0	8.147624	0.664643	0	1.758655	0	0.34027
ST6GALNAC2	0	27.690287	0	28.172624	28.800414	14.829246	0	4.217271
ST6GALNAC2	0	0	0	0	0	0	0	0
ST6GALNAC3	0	0.040774	0	0.478808	0	0	0	0
ST6GALNAC4	16.271004	24.247014	7.380089	26.346216	26.593777	48.368323	27.016684	27.189971
ST6GALNAC4	0	0	0	0	0	0	0	0
ST6GALNAC5	0	0	0	0	0	0	0	0
ST6GALNAC6	0	0.750398	0	6.393426	11.838759	3.070407	20.019153	0.179188
ST7	24.778564	15.526275	26.182601	8.10083	23.986796	12.457195	57.609131	10.290504
ST7L	20.267246	11.244996	18.525997	10.845073	0.992867	8.535869	41.702708	14.296987
ST8SIA1	0	0.294388	0	0.163735	0	0	0	0
ST8SIA2	0	0	0	0	0	0	0	0

ST8SIA3	0	0	0	0	0	0	0	0
ST8SIA4	0	0.861357	2.770328	0.4773	0.1357	0.051511	0	0
ST8SIA5	0	0	0	0.040088	0	0.245111	0	0
ST8SIA6	0	0.683435	0	0.150614	0	0.046447	0	0.055655
STAB1	0	0	0	0	0	0	0	0
STAB2	0	0	0	0	0	0	0	0
STAC	0	3.92542	18.296659	6.459267	6.314099	1.920588	0	4.430729
STAC2	0	0	0	0.435866	0	0	0	0.03902
STAC3	2.257185	0.666326	0	2.031603	0	0	0	0
STAG1	38.692573	47.317419	26.014252	50.029842	38.238046	39.209258	71.521324	77.06274
STAG2	83.8122	220.899504	97.298757	232.99765	51.110394	254.12574	81.199964	285.836111
STAG3	0	0.047334	0	0.186726	0	0	0	0
STAG3L1	0	0	0	0	0	0	13.755554	0
STAG3L2	0	0	0	0	5.959869	0	0	0
STAG3L3	0	0	0	0.647345	0	0	0	0
STAG3L4	0	0	0	0	0	0	0	0
STAG3L5P	0	0	0	0	0	0	0	0
STAG3L5P-P'	12.367313	5.789852	7.346398	9.013425	11.607842	16.582481	41.858894	11.473229
STAM	20.887128	12.158559	3.080509	11.28021	27.183879	6.196753	12.946944	15.732003
STAM2	23.764938	19.472406	5.89154	11.431739	36.354637	8.40349	14.729897	23.798367
STAMBP	9.142372	8.435766	15.922911	6.241318	5.851016	3.64433	0	4.917128
STAMBPL1	0	6.97133	3.06357	5.66153	7.688494	9.524828	21.183503	13.197921
STAP1	0	0	0	0	0	0	0	0
STAP2	5.802419	28.299924	89.765633	35.8123	5.288529	28.4349	73.805681	28.310591
STAR	0	0	0	0	0	0	0	0
STARD10	26.080381	54.258762	44.702182	76.947106	55.561363	67.367967	134.177066	38.104705
STARD13	10.099931	12.802424	10.875176	9.450683	10.934811	9.523897	41.997597	11.999802
STARD3	22.221471	71.878357	88.356223	78.869353	39.799423	66.549289	35.434928	62.530422
STARD3NL	39.520056	37.965419	11.747468	27.526074	41.100594	28.604316	52.455161	26.882964
STARD4	75.547493	46.728888	43.385697	26.306681	53.718624	14.699267	127.258672	42.332322
STARD5	7.941211	3.695783	1.80587	5.815419	12.891757	14.379389	0	7.006326
STARD6	0	1.476824	0	0.582603	0	0	0	0
STARD7	0	113.021899	0	44.218149	0	180.634554	42.196549	8.381837

STARD8	0	0.973123	0	2.318294	0	0.872003	0	0.609038
STARD9	0.407928	0.748654	0.551097	1.501839	0	2.733332	0	2.052929
STARP1	0	0	0	0	0	0	0	0
STAT1	9.892289	54.379865	13.186808	49.389407	86.164878	163.782454	0	207.176357
STAT2	17.845433	12.939639	26.377201	14.070381	35.761655	21.100445	56.084448	12.53237
STAT3	38.532042	44.171438	31.520322	60.04019	37.00623	104.154487	162.302978	83.242486
STAT4	1.326349	1.429662	2.373935	2.29238	14.465082	1.884292	0	2.908114
STAT5A	3.374685	4.667561	1.702479	6.866838	2.868683	10.073548	3.694358	6.811237
STAT5B	2.995472	20.215852	0	1.328592	0	6.274211	18.142655	0
STAT6	41.744551	43.826735	40.460091	62.083052	25.049674	45.617203	43.476289	25.393423
STATH	0	0	0	0	0	0	0	0
STAU1	132.593937	241.931859	129.561619	257.041609	147.413094	221.464897	23.968233	254.501597
STAU2	40.529655	87.320018	60.001583	92.603258	46.432621	37.197842	29.038581	65.621739
STAU2P1	0	0	0	0	0	0	0	0
STBD1P1	0	0	0	0	0	0	0	0
STC1	0	0	0	0.041763	0	0.198747	0	0
STC2	0	41.71366	40.227644	162.357527	35.311784	134.765089	77.237848	106.294383
STEAP1	15.220832	33.736055	21.583959	17.163503	25.482616	5.938464	0	8.997788
STEAP1B	0	0	0	0.567091	0.773138	1.121704	0	0.477317
STEAP2	9.667691	21.427299	20.434003	15.256105	0.890824	3.721485	1.673509	5.077396
STEAP3	6.187248	9.419415	5.554459	7.955408	13.152203	12.576805	0.941311	8.790512
STEAP4	0	0	0	0.063306	2.333754	0.0728	0	0.380129
STEEP1	12.192163	14.080129	10.152114	12.378963	13.297548	6.730329	17.236858	6.507828
STH	0	0	0	0	0	0	0	0
STIL	6.501311	12.194517	0	1.655191	0	3.64535	29.475001	17.489384
STIM1	9.813009	32.143087	24.384525	61.683197	33.145289	69.101212	19.292965	34.675298
STIM2	7.095741	3.784291	0	2.291509	0	0.902628	26.520938	2.461753
STIMATE	17.776054	0	0	9.245077	0	17.224226	0	3.110915
STIMATE-MU	0	1.835657	4.850497	0.063783	0	0.118515	12.011817	4.563107
STING1	11.107304	12.86296	19.018867	17.206817	10.278606	36.96329	0	29.319198
STIP1	0	35.131219	0	47.662919	0	47.607819	582.924467	53.554218
STIP1P3	0	0	0	0	0	0	0	0
STK10	21.188582	25.38564	11.415167	32.178186	17.798242	51.396911	8.5867	43.378759

STK11	0	23.077082	0	20.890897	11.887239	92.010782	28.458573	73.981794
STK11IP	27.741463	4.652067	4.939307	2.88231	2.064118	3.207322	1.785743	3.67113
STK16	13.875256	22.691718	9.605088	31.076794	34.721172	32.490484	5.418249	23.217746
STK16P1	0	0	0	0	0	0	0	0
STK17A	35.251517	64.370076	56.813989	29.398683	37.935376	41.498479	70.242024	92.578806
STK17B	9.909033	21.833694	16.919594	9.567651	9.726565	4.125984	7.242456	19.596105
STK19	18.850865	6.731064	10.290363	8.654007	64.198524	9.292069	0	6.258075
STK19B	0	0	0	0	0	0	0	0
STK24	202.854877	354.422236	302.530115	357.244939	99.326284	146.886566	104.060004	171.73841
STK24P1	0	0	0	0	0	0	0	0.346506
STK25	28.47749	53.851938	24.808948	57.946227	108.187183	127.981826	121.442428	106.33139
STK25P1	0	1.959658	0	1.608895	0	0.921757	0	0.987255
STK26	68.444262	89.834067	40.14931	75.376791	60.638992	67.533534	37.23912	131.813876
STK3	63.027489	33.551818	31.565573	19.93869	61.093754	14.912637	22.593938	23.576194
STK31	0	0	0	0	0	0	0	0
STK32A	0.655916	2.57719	0.589227	2.587447	0	0	0	0
STK32B	0	1.247421	0	0.321232	0	1.305835	3.381223	1.167588
STK32C	11.356819	13.080245	11.056956	20.176799	0.493787	24.009295	0	9.670272
STK33	11.276645	18.264942	10.457431	12.500915	1.941794	11.953403	16.596833	27.191119
STK33P1	0	0	0	0	0	0	0	0
STK35	0	0	0	0	0	8.373168	0	3.303405
STK36	3.39839	9.337194	4.292236	8.229194	13.135773	5.773208	5.997681	11.482175
STK38	17.606523	48.83467	29.921851	32.345783	36.210478	43.616776	47.778915	72.550945
STK38L	28.936037	62.454844	46.681082	71.174547	31.099049	46.598545	105.221814	70.662407
STK39	16.329169	27.60882	19.213112	23.130716	22.361413	22.393252	20.590781	23.318667
STK4	45.884217	53.167574	42.148587	48.076712	39.87497	32.262708	0	45.431941
STK40	0	13.833861	27.456911	5.275707	2.727086	8.938645	0	11.658942
STKLD1	0	0.848415	0	0.124743	0	0	0	0.231517
STMN1	399.994413	409.660592	277.249685	403.849806	550.332301	706.857582	497.66208	576.757283
STMN1P1	0	0	0	0	0	0	0	2.589203
STMN1P2	0	0	0	0	0	0	0	0
STMN2	0	0	0	0	0	0	0	0
STMN3	13.741243	17.080558	19.107301	18.013358	13.586764	7.874024	8.141795	5.613901

STMN3P1	0	0	0	0	0	0	0	0
STMN4	0	0	0	0	0	0	0	0
STMND1	0	0	0	0	0	0.406663	0	0
STMP1	19.376091	19.707113	11.143451	11.670277	8.032409	3.350265	0.335738	11.525689
STN1	0.577569	2.636429	1.663049	4.249551	0.818472	2.401166	4.969042	1.729793
STOM	93.416255	45.858861	58.417617	64.777263	0	2.354501	0	1.033485
STOML1	3.821916	6.343764	6.904471	6.05307	2.707673	7.464125	12.479373	6.346387
STOML2	122.726295	170.707526	123.217952	159.990461	149.559545	236.220984	123.733314	225.335378
STOML3	0	0	0	0	0	0	0	0
STON1	0	1.09935	0	0.148852	0	0.227677	0	0.308439
STON1-GTF2	0	0.519749	0	0	2.235449	0.610423	0	0
STON2	55.931429	72.233501	37.456811	58.620062	31.33651	72.962238	49.84319	117.1179
STOX1	1.017608	0.434728	1.824534	0.496679	0	0.913456	7.42095	0.909568
STOX2	8.909637	14.429974	4.019962	19.158322	0.726357	2.980823	2.184069	1.863153
STPG1	9.34738	22.84157	14.430161	23.538652	4.551459	18.311939	28.639283	31.562865
STPG2	1.844963	0	0	0.089555	0	0	0	0
STPG3	0	0	0	0	0	0	0	0
STPG4	10.658909	20.549107	0	21.266572	0	0	0	0.688901
STRA6	4.417776	2.259929	15.334884	5.434159	0	0.091325	0	0
STRA8	0	0	0	0	0	0	0	0
STRADA	24.002899	12.621341	9.01836	17.732194	100.387027	12.556204	0	11.091491
STRADB	23.19419	13.942508	17.533098	11.35262	12.842701	5.212929	0	20.910417
STRADBP1	0	0	0	0.069299	0	0	0	0
STRAP	199.513131	210.117801	148.059294	136.435951	224.440477	121.166073	212.470819	188.77449
STRBP	30.993369	37.869727	18.408762	29.221987	13.932225	29.463267	128.735854	22.07025
STRC	0	0	0	0.073857	0	0	0	0
STRCP1	0	0	0	0	0	0	0	0
STRIP1	26.152356	18.011454	22.064386	20.889163	20.567597	19.036958	30.309656	9.710717
STRIP2	8.506324	9.275299	6.31522	7.294041	7.442488	9.123708	0	7.971708
STRIT1	0	0	0	0	0	0	0	0
STRN	28.99408	11.675827	6.450861	18.079106	10.548736	13.003581	3.104956	16.492128
STRN3	116.735004	46.827116	34.119993	70.412599	157.576817	76.135115	136.511051	82.997289
STRN4	16.471632	60.912749	64.060275	65.17787	144.740054	169.857736	123.901513	75.268248

STS	0	0.354769	0	0.176986	0	0	0	0
STSP1	0	0	0	0	0	0	0	0
STT3A	55.717472	44.327253	62.696771	46.342055	462.870414	52.677	163.955793	75.518834
STT3B	5.316278	20.008041	101.790466	6.776131	0	6.754656	21.061296	21.802685
STUB1	40.884614	112.681778	30.854876	97.115896	80.419149	173.557275	28.218659	171.274814
STUB1P1	0	0	0	0	0	0	0	0
STUM	0.416186	0.268821	0.374381	0.367492	0	0	0	0
STX10	14.904153	30.282126	24.965467	43.399595	51.674724	90.381333	64.691676	61.868072
STX12	13.269453	22.35536	11.889875	16.577137	13.425738	8.880038	54.105065	8.715007
STX16	57.001251	147.069509	61.493247	184.270446	90.829083	105.489568	42.463096	113.217213
STX16-NPEPI	0	1.327546	0	0	0	10.806758	0	0.403094
STX17	21.587183	28.995247	11.843054	23.847034	18.512242	16.613565	53.336259	24.398915
STX18	11.174812	41.204827	17.117492	40.046991	15.691399	28.487386	33.585835	32.445785
STX19	0	2.303081	2.890042	3.065263	0	0.476534	0	0.663898
STX1A	4.23418	2.568218	4.524635	9.342797	0	4.172139	0	1.590762
STX1B	0	1.94365	0	1.954746	0	0.140513	0	0.760268
STX2	0	6.750727	0	3.233689	0	4.283822	0	2.722953
STX3	29.067141	43.205427	22.384939	27.088643	71.21198	59.312409	25.601036	66.607865
STX4	10.280273	74.4249	43.141123	89.565762	40.797598	92.890885	86.395561	66.675755
STX5	37.080458	0	0	2.231977	13.753381	1.632345	51.397205	2.363787
STX6	53.822236	83.545659	41.61206	75.864364	26.808982	41.693333	51.105399	50.546473
STX7	24.30991	39.580641	36.82372	36.271609	8.672044	19.544284	15.020138	30.791753
STX8	44.274492	81.723	33.873963	87.509559	47.033188	38.529943	93.073099	44.399753
STX8P1	0	0	0	0	0	0	0	0
STXBP1	0	2.032226	0	5.854803	14.831698	2.239725	0	2.575811
STXBP2	59.994494	63.784432	32.972435	57.673257	59.877344	52.565398	96.540072	43.072814
STXBP3	27.31382	126.90246	28.134514	66.710881	35.788834	53.489895	26.934652	37.863481
STXBP4	46.729012	6.212333	5.42274	4.328674	5.68013	3.130054	7.863513	11.592778
STXBP5	7.241748	4.586869	5.51265	9.095664	5.937067	11.99979	26.616713	17.459199
STXBP5L	0	0	0	0.047865	0	0	0	0
STXBP6	0	1.262559	0	0.495276	0	1.183273	0	0.378548
STYK1	4.872748	14.542421	4.363362	7.143426	2.250247	7.799942	5.324195	5.318462
STYX	28.95765	12.456015	18.147103	7.244298	20.528373	7.21964	34.280081	15.890824

STYXL1	35.951642	64.543763	34.606016	67.41498	105.24375	55.71463	49.416897	49.057478
STYXL2	0	0	0	0	0	0	0	0
SUB1	344.710149	342.665824	179.743003	195.8446	201.669511	97.948967	88.663073	248.383276
SUB1P1	0	0	0	0	0	0	0	0
SUB1P2	0	0	0	0	0	0	0	0
SUB1P3	0	0	0	0	0	0	0	0
SUB1P4	0	0	0	0	0	0	0	0
SUCLA2	20.310878	52.577923	32.631461	24.864082	56.094128	19.276509	51.326383	56.550492
SUCLA2P1	0	0	0	0.1287	0	0	0	0
SUCLA2P2	0	0	0	0	0	0	0	0
SUCLA2P3	0	0	0	0	0	0	0	0
SUCLG1	107.346676	102.80598	82.348958	64.576437	43.504035	41.72703	25.481296	51.814537
SUCLG2	0	17.822364	0	24.779469	0	17.460867	0	25.129255
SUCLG2P2	0	0	0	0	0	0	0	0
SUCLG2P4	0	0	0	0	0	0	0	0
SUCNR1	0	0	0	0	0	0	0	0
SUCO	36.90379	59.884336	28.276075	39.614041	28.216064	14.803929	8.833858	41.707684
SUDS3	31.933133	48.020241	28.056669	35.850038	55.285039	51.946367	26.880084	32.886929
SUDS3P1	0	0	0	0.111447	0	0	0	0
SUFU	0	0	2.645661	0.056808	3.968175	0	0	1.601771
SUGCT	0	0	0	0	20.595313	4.459398	0	3.904305
SUGP1	29.644102	11.441003	40.29283	14.073811	24.176543	33.071463	81.378874	15.53226
SUGP2	9.743469	23.553422	19.932693	25.10932	12.131926	49.958232	135.701086	40.823118
SUGT1	25.21596	71.327749	31.027189	29.482705	24.569377	25.69874	49.046145	46.882899
SUGT1P1	0	4.88915	0	0	0	0.484443	0	0.906024
SUGT1P2	0	0	0	0	0	0	0	0
SUGT1P3	0	0	0	0	0	0	0	0
SUGT1P4	0	0	0	0	0	0	0	0
SULF1	0.985782	2.830236	8.739475	3.187064	0	0.279605	0	0.056054
SULF2	10.720142	21.382299	7.600357	16.652047	46.439053	52.968154	29.881815	33.141288
SULT1A1	0	4.089823	8.270641	2.95204	0	3.127595	0	3.126949
SULT1A2	0	1.542938	0	0.175231	19.045842	0.989166	0	1.586712
SULT1A3	0	5.735676	11.319386	1.085216	0	0.969159	0	0

SULT1A4	0	0	0	0	12.25368	0	4.025388	0
SULT1B1	0.461188	0.198474	0	0.011283	0	0	0	0.036973
SULT1C2	0	0	0	0.601799	0	0	0	0
SULT1C2P2	0	0	0	0	0	0	0	0
SULT1C4	0	0	0	0	0	0	0	0
SULT1C5P	0	0	0	0	0	0	0	0
SULT1D1P	0	0	0	0	0	0	0	0
SULT1D1P2	0	0	0	0.28434	0	0	0	0
SULT1E1	3.966194	1.254857	0	0.336642	0	0	0	0.109539
SULT2A1	0	0	0	0	0	0	0	0
SULT2B1	0	0	0	0	1.548208	0	0	0
SULT4A1	0	1.109865	0	1.118544	0	0	0	0
SULT6B1	0	0	0	0	0	0	0	0
SULT6B2P	0	0	0	0	0	0	0	0
SUMF1	24.080184	21.463832	30.121912	23.996048	25.303984	8.528379	0.777895	8.803142
SUMF2	107.297153	92.28288	133.336443	60.212337	139.728672	97.719211	181.359323	84.033633
SUMO1	11.513561	36.734059	0	51.618892	108.341305	100.623684	49.804383	54.134659
SUMO1P1	0	0	0	0	0	0	0	0
SUMO1P2	0	0	0	0	0	0	0	0
SUMO1P3	0	0	0	0	0	0	0	0
SUMO1P4	0	0	0	0	0	0	0	0
SUMO2	548.959148	613.110284	489.98102	558.437142	474.496079	395.724637	690.82812	439.394122
SUMO2P1	0	0	0	0	86.269146	0	0	0
SUMO2P10	0	0	0	0	0	0	0	0
SUMO2P12	0	0	0	0	0	0	0	0
SUMO2P13	0	0	0	0	0	0	0	0
SUMO2P14	0	0	0	0	0	0	0	0
SUMO2P15	0	0	0	0	0	0	0	0
SUMO2P16	0	0	0	0	0	0	0	0
SUMO2P17	0	0	0	0	0	0	0	0
SUMO2P18	0	0	0	0	0	0	0	0
SUMO2P19	0	0	0	0	0	0	0	0
SUMO2P2	0	0	0	0	0	0	0	0

SUMO2P20	0	0	0	0	0	0	0	0
SUMO2P21	0	0	0	0.764876	0	0	0	0
SUMO2P3	0	0	0	0	0	0	0	0
SUMO2P4	0	0	0	0	0	0	0	0
SUMO2P5	0	0	0	0	0	0	0	0
SUMO2P6	0	0	0	0	0	0	0	0
SUMO2P7	0	0	0	0	0	0	0	0
SUMO2P8	0	0	0	0	0	0	0	0
SUMO3	0	3.868778	5.975319	0.145359	0	3.700787	133.747601	2.405874
SUN1	123.659377	146.103507	89.469052	309.423717	115.541164	256.024648	53.258075	194.713578
SUN2	40.371742	38.751423	13.883755	41.766819	52.831455	84.733407	69.01939	52.072051
SUN3	5.383827	15.241821	18.130517	15.422048	0	0.329854	0	0
SUN5	0	0	0	0	0	0	0	0
SUOX	16.335892	13.012321	5.311296	17.26341	10.96761	10.170584	35.174047	16.562953
SUPT16H	159.001398	170.925948	140.118008	146.700008	208.675086	195.070247	176.971755	247.24352
SUPT16HP1	0	0	0	0.02641	0	0.054103	0.262981	0
SUPT20H	19.728975	60.460648	18.210241	45.981071	17.767048	43.121314	53.111086	47.898245
SUPT3H	2.858381	11.051849	13.930194	8.973152	15.380787	10.359496	12.724034	10.552299
SUPT4H1	120.943541	100.805226	114.218932	90.772605	79.810086	103.125441	19.962908	101.775016
SUPT4H1P1	0	0	0	0	0	0	0	0
SUPT4H1P2	0	0	0	0	0	0	0	0
SUPT5H	30.501697	62.001894	29.499007	65.623496	31.189301	158.781274	94.715991	50.67212
SUPT6H	36.483131	51.757581	49.620999	54.72127	115.068666	66.231167	148.173486	54.983991
SUPT7L	26.227194	62.341038	12.403995	69.099946	11.740641	61.318888	23.326738	70.68984
SUPV3L1	50.181703	35.093066	29.510169	27.530501	37.740958	18.203928	72.367316	28.339811
SURF1	80.411822	85.687828	49.085768	79.450345	44.077932	67.988345	77.490029	57.587973
SURF2	69.927229	78.065168	29.805381	66.229746	90.318343	77.582044	32.335106	78.654266
SURF4	88.36993	134.921997	129.775752	123.912057	293.081264	86.061737	127.036802	90.735234
SURF6	25.040531	27.732769	18.856859	31.264128	40.604725	36.672772	6.628041	22.51893
SURF6P1	0	0	0	0	0	0	0	0
SUSD1	0	9.188755	11.680118	14.4522	19.073377	7.345386	20.510231	4.795603
SUSD2	0	0	0	0.064801	0	0.066384	0	0.212035
SUSD2P1	0	0	0	0	0	0	0	0

SUSD2P2	0	0	0	0	0	0	0	0
SUSD3	3.531968	6.240046	0	4.129338	1.469129	7.195717	0	3.668956
SUSD4	0	11.472045	0	2.070029	0.349057	5.399749	12.017174	3.893998
SUSD5	0	0.183587	0.640627	0.331114	0	0.555019	0	0.085896
SUSD6	16.951119	20.005145	16.320029	40.40646	20.15217	35.124999	14.160515	24.746367
SUV39H1	0	0	0	0.342947	0	0	0	0
SUV39H2	6.843607	27.071234	21.730497	2.549957	11.179384	3.621206	31.161123	15.643498
SUZ12	38.681935	14.622708	42.012798	7.158633	14.915218	5.625742	34.887913	18.162914
SUZ12P1	0	10.175881	0	3.226681	34.682635	1.626934	0	3.512641
SV2A	9.769027	15.267842	9.445706	18.028018	3.374041	15.812305	6.193543	11.20728
SV2B	0	0	0	0.138362	0	0	0	0
SV2C	0	0	0	0	0	0.014892	0	0
SVBP	0	21.278997	18.786215	17.356206	18.422351	29.003953	25.455558	44.735837
SVEP1	0.786582	1.968541	1.652455	3.807953	0.069312	0.171423	0	0.020998
SVIL	3.852403	4.671508	1.732077	6.337398	4.219258	16.107306	0.352236	12.24226
SVIL2P	0	0.566553	0	0.093952	0	0	0	0
SVIP	5.870352	23.597001	44.089633	18.56483	8.915376	27.741833	5.895775	38.328754
SVOP	0	0	0	0	0	0	0	0
SVOPL	0	0.429379	0	0.061967	0	0	0	0
SWAP70	24.407057	25.220557	31.32165	20.11716	85.097229	59.092833	98.934455	48.169466
SWI5	24.457638	4.560014	0	1.321374	0	0.641738	0	1.843436
SWT1	1.72501	4.070719	2.321706	1.368392	0	0.560132	4.196905	2.641388
SYAP1	17.95255	44.070585	13.765623	17.922013	35.96036	34.481924	18.4052	52.382187
SYBU	0	2.653502	3.451355	3.169998	17.2207	21.742289	19.506221	9.076832
SYCE1	0	0	0	0	0	0	0	0
SYCE1L	0	2.346825	0	7.447295	0	2.790774	0	0
SYCE2	0	1.176935	0	1.685746	0	0	0	0
SYCE3	0	0	0	1.281354	0	0	0	0.47632
SYCP1	0	0	0	0	0	0	0	0
SYCP2	0	3.979407	8.78796	0.224486	0	1.637626	0	0.837226
SYCP2L	0	0	0	0.319529	0	0.11517	0	0
SYCP3	0	0	0	0	0	0.262606	0	0.036113
SYDE1	8.77244	5.177618	5.728779	4.526913	1.157728	19.620106	49.043255	11.178182

SYDE2	5.96687	8.262906	11.260217	4.851324	5.832655	2.391724	0	5.097037
SYF2	70.23037	35.503451	51.790938	37.190935	100.417128	28.763075	53.719925	39.989601
SYF2P1	0	0	0	0	0	0	0	0
SYF2P2	0	0	0	0	0	0	0	0
SYK	11.25226	30.860051	15.490114	24.173299	12.516928	31.031531	53.416597	29.243725
SYMPK	0	26.629771	19.229832	24.406995	55.050041	37.970514	20.454959	18.850127
SYN1	1.054724	1.252573	0	0.308583	0	0.804464	0	0.258027
SYN2	1.004954	1.07398	0	0.346774	0	0.150371	0	0.326834
SYN3	0	0	0	0	0	0	0	0
SYNC	0	3.047229	0	2.673022	15.558787	7.322814	6.760698	5.632773
SYNCRIPP1	0	0	0	0	0	0	0	0
SYNDIG1	1.651611	3.848599	0	1.444659	0	0	0	0.266441
SYNDIG1L	0	0.239604	0	0.068584	0	0	0	0
SYNE1	3.127689	3.574493	1.019185	4.324661	1.116165	3.909866	0	2.522524
SYNE2	23.597028	19.640299	12.712166	44.144312	45.624614	86.978111	92.27381	84.497717
SYNE3	0.947639	0.34768	2.802623	1.613837	2.442614	4.909263	6.083138	3.226224
SYNE4	0	0.528267	0	0.446656	0	0.37151	0	0
SYNGAP1	14.054818	4.067729	3.514069	5.302647	27.35459	7.404851	0	4.634678
SYNGR1	10.015936	18.959667	6.423174	17.299852	2.15423	6.94059	0	6.548872
SYNGR2	46.647992	102.793692	112.05584	166.891627	58.357287	92.038136	72.619776	90.532582
SYNGR2P1	0	0	0	0	0	0	0	0
SYNGR3	5.544771	1.810633	6.110672	2.087493	0.902082	1.354596	0	1.266316
SYNGR4	0	0	0	0	0	0.728242	0	0
SYNJ1	0	2.725897	1.92246	0.719049	11.098823	3.038923	7.172058	7.142802
SYNJ2	24.773718	46.002825	18.817145	47.798375	14.239925	79.667768	28.257683	75.700279
SYNJ2BP	53.650627	30.373366	20.305765	20.224891	41.37152	11.225248	129.346015	25.752348
SYNJ2BP-CO	0	27.493933	30.461021	31.514101	0	33.095652	42.133762	16.494008
SYNM	0	1.410193	2.969573	0.537738	0	0	0.203098	0
SYNPO	20.586381	37.897246	29.026223	35.571353	8.684484	19.442708	44.057435	12.943488
SYNPO2	0.925842	1.290853	0.754187	1.733174	0.24445	0.853066	0	0.345644
SYNPO2L	0	0	0	0.195461	0	0	0	0
SYNPR	0	0	0	0	0	0	0	0
SYNRG	22.604032	20.131863	23.77111	18.605707	65.755961	12.874685	33.582492	15.311168

SYP	0	0.442575	0	0.276167	0	0.90157	0	0
SYPL1	26.593751	31.454845	0	22.386772	0	0.239923	48.24626	20.210081
SYPL1P1	0	0	0	0	0	0	0	0
SYPL1P2	5.799722	0	0	0.550465	0	0	0	0
SYPL2	0	0	0	0	0	0	0	0
SYS1	30.70622	33.35209	24.250337	33.587026	22.08604	28.967683	24.170748	30.959577
SYS1-DBNDI	0	1.228788	0	4.942799	0	1.213229	42.373055	4.769052
SYT1	2.788463	10.22695	9.993753	9.240536	0	0.061865	0	0
SYT10	0	0.238245	0	0.449044	0	0.110589	0	0
SYT11	0.63059	0.054154	0	0.262008	4.164641	1.200163	0	1.948008
SYT12	15.143271	41.743654	7.1923	36.040728	4.707697	26.216835	56.368222	20.469014
SYT13	0	0	0	0	0	0.348593	0	0.228469
SYT14	2.826135	4.049566	5.337513	1.713148	15.825892	2.765088	11.61176	10.54494
SYT14P1	0	0	0	0	0	0	0	0
SYT15	0	3.820176	8.528739	1.966179	0.666882	1.1215	0	0.838535
SYT16	9.905006	17.508188	4.115259	10.921646	1.449776	12.610641	3.849613	14.544245
SYT17	0	12.148262	9.863289	14.652004	58.854285	8.308296	15.359993	11.939848
SYT2	0	0.091322	0.381569	0.145323	0	0.063885	0	0.170004
SYT3	0	0.228952	0	0.884305	0	0.601743	0	0
SYT4	0	0	0	0	0	0	0	0
SYT5	0	0	0	0.063202	0	0	0	0
SYT6	0	0	0	0	0	0	0	0
SYT7	12.147761	16.521383	19.041075	25.924946	11.46316	26.062901	1.643694	16.147532
SYT8	0	0	0	0.245702	0	0.713608	0	1.039519
SYT9	0	0	0	0.040088	0	0	0	0
SYTL1	3.786262	11.431983	3.675727	4.979338	10.64886	16.517241	20.802292	11.076103
SYTL2	0.519573	6.398802	1.377176	1.17943	0.81029	9.009221	0	8.036441
SYTL3	0	0	1.124951	0	0	0.377298	0	0
SYTL4	9.934513	8.962419	1.828722	8.110553	5.804577	7.250091	9.236218	2.50433
SYTL5	0	0.119655	0	0.034071	0	0.663042	0	0.553891
SYVN1	2.372196	3.3007	1.021761	6.447286	25.084311	13.454125	35.452103	4.902483
SZRD1	94.552769	114.349474	58.687672	135.701238	73.5638	143.660562	92.91917	133.59142
SZRD1P1	0	0	0	0	0	0	0	0

SZT2	5.178115	4.43816	7.677762	2.07701	50.142672	5.224383	10.652341	8.667233
TAAR2	0	0	0	0	0	0	0	0
TAAR3P	0	0	0	0	0	0	0	0
TAAR4P	0	0	0	0	0	0	0	0
TAAR6	0	0	0	0	0	0	0	0
TAAR7P	0	0	0	0	0	0	0	0
TAB1	36.351504	7.680376	6.560657	10.65361	12.144687	13.559378	2.795495	9.063618
TAB2	54.299564	42.699233	0	55.232019	11.451116	34.80689	39.81342	46.700939
TAB3	21.284964	23.638064	14.468494	18.290717	27.401635	27.218482	38.121289	36.086421
TAB3P1	0	0	0	0	0	0	0	0
TAC1	0	0	0	0	0	0	0	0
TAC3	0	0	0	0.136645	0	0	0	0
TAC4	0	0	0	0	0	0	0	0
TACC1	47.177603	132.006468	34.905204	110.764989	47.114815	138.107663	111.839347	107.112163
TACC1P1	0	0	0	0	0	0	0	0
TACC2	69.17842	96.2459	23.075028	110.749452	35.052177	119.903122	57.812764	99.117406
TACC3	26.726339	95.38805	37.773471	69.116904	107.658815	192.259875	56.929962	143.219108
TACO1	19.974221	71.152477	19.977063	58.921459	59.815428	82.317227	33.707154	73.095328
TACR1	0	3.464663	0	0.931339	0	0.141713	0	0
TACR2	0	0	0	0	0	0	0	0
TACR3	0	0.078475	0	0.029774	0	0	0	0
TADA1	11.537099	8.589252	17.667265	8.049303	8.257052	5.223666	11.976719	6.779925
TADA2A	29.275643	39.479541	16.915261	36.962227	9.430042	29.016633	0	30.168523
TADA2B	5.271556	23.256888	14.179948	20.467598	57.079655	56.471486	10.550322	44.28642
TADA3	3.620894	13.409421	0	19.960662	21.741525	27.926089	18.584681	1.722504
TAF1	0	1.218953	0	4.685596	39.493301	3.730819	0	0
TAF10	1.469049	12.473477	0.597242	5.404807	2.557883	11.745744	2.207833	11.09402
TAF11	14.75854	0	0	1.140626	0	0.238528	0	0.561919
TAF11L11	0	0	0	0	0	0	0	0
TAF11L12	0	0	0	0	0	0	0	0
TAF11L13	0	0	0	0	0	0	0	0
TAF11L14	0	0	0	0	0	0	0	0
TAF11L2	0	0	0	0	0	0	0	0

TAF11L3	0	0	0	0	0	0	0	0
TAF11L4	0	0	0	0	0	0	0	0
TAF11L5	0	0	0	0	0	0	0	0
TAF11L6	0	0	0	0	0	0	0	0
TAF11L7	0	0	0	0	0	0	0	0
TAF11L8	0	0	0	0	0	0	0	0
TAF11L9	0	0	0	0	0	0	0	0
TAF12	0	12.960942	9.02702	16.979841	2.205329	17.451362	0	16.947038
TAF13P2	0	0	0	0	0	0	0	0
TAF15	140.049167	99.314587	73.473374	106.589926	162.218205	83.526712	161.744428	79.710388
TAF1A	28.667173	30.371771	25.813472	21.914649	21.174772	8.298274	10.581195	19.059211
TAF1B	18.15457	34.679923	34.27488	26.138433	10.77333	36.82682	17.970026	32.65901
TAF1C	13.037469	13.562347	3.407358	14.865964	3.603696	34.53933	194.232503	32.170485
TAF1D	593.559963	275.915755	773.547062	258.086591	104.497021	52.515366	187.065226	67.66654
TAF1L	0	0	0	0	0	0	0	0
TAF2	22.397722	33.214286	17.538642	16.082537	27.004355	9.838366	14.583829	42.452655
TAF3	3.359274	6.372116	3.017453	5.978939	4.96925	6.827803	2.127273	5.472886
TAF4	11.040832	32.312543	55.163909	32.508044	30.856575	64.477411	0	9.846259
TAF4B	11.873387	18.426268	8.662028	18.755354	10.548673	10.836485	12.935337	19.936552
TAF5	7.17421	5.910245	6.431285	3.473906	7.575436	3.57745	2.491243	6.633372
TAF5L	6.233987	32.00131	1.975278	14.575767	25.287497	14.044443	10.563917	19.358474
TAF5LP1	0	0	0	0	0	0	0	0
TAF6	22.509811	35.078854	33.273672	30.252627	6.656369	36.550849	22.975576	28.678228
TAF6L	5.140701	6.886139	7.829892	7.104065	3.889242	18.414407	2.124443	16.224437
TAF7	0	0	0	0.222218	0	0	0	0.397943
TAF7L	0	0	0	0	0	0.081161	0	0.058865
TAF8	14.549637	18.985659	20.112729	23.621982	12.777162	26.270383	21.804462	28.48536
TAF9	76.151671	131.520557	96.882173	109.427487	99.338525	105.130462	108.229384	182.257438
TAF9B	43.327344	9.45775	27.378107	10.108164	29.253343	5.639862	21.961994	10.349973
TAF9BP1	0	0	0	0	0	0	0	0
TAF9BP2	0	0	0	0	0	0	0	0
TAF9P1	0	0	0	0	0	0	0	0
TAF9P3	0	0	0	0	0	0	0	0

TAFA1	0	0	0	0	0	0	0	0
TAFA2	0	0.372706	0	0.60329	0	0.175299	0	0.643717
TAFA3	2.869565	0.286067	0	1.148376	0	1.600806	0	3.260953
TAFA4	0	0.392292	0	0.112396	0	0	0	0
TAFA5	0	0	0	0	0	0	0	0
TAFAZZIN	17.325489	29.250849	12.879105	28.812785	27.780937	28.34472	87.075822	24.494284
TAGAP	0	0.281457	0	0.133968	0	0	0	0.382902
TAGLN	0	0	0	2.296417	4.888648	5.935453	27.277811	3.364711
TAGLN2	0	275.396048	0	300.106999	181.807121	301.812175	232.062541	200.801786
TAGLN2P1	0	0	6.756293	0.187135	0	1.136743	0	0
TAGLN3	6.783942	2.981793	11.776764	3.246624	0	2.248584	0	0.406912
TAL1	0	0.060652	0	0.086357	0	0.176892	0	0.226993
TALDO1	194.643132	262.875657	202.745756	272.712361	292.950902	276.26066	187.688443	267.036222
TAMALIN	0	0	0	0.129821	0	0.309326	0	0
TAMM41	0	5.92237	9.777321	8.050908	0	4.085984	0	8.617479
TANC1	10.337902	21.690597	12.785754	22.469312	19.49316	41.211263	11.345951	34.754795
TANC2	12.868162	50.019656	12.54358	45.684087	89.150034	47.283141	10.412097	44.452408
TANGO2	8.375006	13.583368	7.700427	14.75269	13.361993	23.791582	0	13.837505
TANGO6	8.031441	20.049264	6.011927	20.588488	33.977302	11.526375	1.849987	16.636042
TANK	0	80.738754	33.071264	36.563592	22.157994	42.894398	27.457159	83.214619
TAOK1	10.86982	67.909121	0	113.205024	80.268731	101.936702	6.440285	126.769052
TAOK2	14.849255	33.630597	18.750367	40.582632	17.574528	43.136472	29.879357	37.66384
TAOK3	41.323654	64.305067	49.953349	67.716575	56.531341	66.456595	74.055188	64.393038
TAP1	9.243396	29.500738	45.596163	24.779345	48.470407	72.535773	38.623282	54.563668
TAP2	36.313162	21.258519	16.474832	21.346865	57.687229	43.951241	27.405768	22.597391
TAPBP	25.567918	48.993158	74.075793	38.945946	107.280042	83.865568	56.324914	47.563691
TAPBPL	0	8.672683	0	7.264567	1.878966	4.683525	0	1.67393
TAPT1	7.178655	4.57911	8.859731	4.786371	5.792954	2.489044	0	7.6334
TARBP1	10.401084	3.473545	5.543485	2.887689	10.144421	3.483396	29.605672	7.008777
TARBP2	13.366179	10.597196	11.86369	17.006826	3.893041	3.622143	0	14.613615
TARDBP	115.029059	227.968194	94.957032	201.97763	248.590065	227.581766	241.439883	264.896599
TARDBPP1	0	0	0	0	0	0	0	0
TARDBPP2	0	0	0	0	0	0	0	0

TARDBPP4	0	0	0	0	0	0	0	0
TARM1	0	0	0	0	0	0	0	0
TARS1	285.69904	440.115124	274.056607	468.249224	341.615902	428.747655	491.638212	270.383193
TARS2	26.024486	29.150057	34.775526	37.042811	10.746219	27.01722	79.892486	19.125225
TARS2P1	0	0	0	0	0	0	0	0
TARS3	1.819404	4.749564	3.892542	3.788346	13.012112	10.480634	11.258661	14.085441
TAS1R1	0	0	0	0.089258	0	0	0	0
TAS1R3	1.915257	0.696027	1.717578	1.004527	0.252828	0.716825	0	0.115452
TAS2R1	0	0.668504	0	0	0	0	0	0
TAS2R13	0	0	0	0	0	0	0	0
TAS2R14	0	0	0	0.063487	0	0	0	0
TAS2R15P	0	0.173545	0	0.101365	0	0	0	0
TAS2R16	0	0	0	0	0	0	0	0
TAS2R19	0	0.475864	0	0	0	0	0	0
TAS2R20	0	0.330349	0	0.063598	0	0.064477	0	0
TAS2R2P	0	0	0	0	0	0	0	0
TAS2R3	0	0	0	0.082628	0	0	0	0.139851
TAS2R30	0	0	0	0	0	0	0	0
TAS2R31	0	0.310388	0	0.180705	0	0	0	0
TAS2R38	0	0	0	0	0	0	0	0
TAS2R39	0	0	0	0	0	0	0	0
TAS2R40	0	0	0	0	0	0	0	0
TAS2R41	0	0	0	0	0	0	0	0
TAS2R42	0	0	0	0	0	0	0	0
TAS2R43	0	0	3.717929	0.303298	0	0	0	0.457082
TAS2R46	0	0	0	0	0	0	0	0
TAS2R5	0	0	0	0.078437	0	0	0	0
TAS2R50	0	0	0	0	0	0	0	0
TAS2R60	0	0	0	0	0	0	0	0
TAS2R62P	0	0	0	0	0	0	0	0
TAS2R63P	0	0	0	0	0	0	0	0
TAS2R64P	0	0	0	0	0	0	0	0
TAS2R6P	0	0	0	0	0	0	0	0

TAS2R7	0	0	0	0	0	0	0	0
TAS2R9	0	0	0	0	0	0	0	0
TASOR	47.735164	41.543159	30.26646	19.882777	50.673712	18.522926	55.750794	38.963745
TASOR2	23.520828	71.567468	25.718735	68.992584	21.967434	68.642645	50.029988	111.418035
TASP1	17.364678	41.022278	17.586684	34.643949	31.682381	29.789279	44.714691	29.303918
TAT	0	0	0	0.061369	0	0	0	0
TATDN1	87.507365	41.84267	89.361995	24.628833	37.427512	10.284174	122.295631	40.263199
TATDN1P1	0	0	0	0	0	0	0	0
TATDN2	34.281865	40.590819	23.230301	33.515343	15.599452	32.411169	88.334773	30.853133
TATDN2P1	0	0	0	0	0	0	0	0
TATDN2P2	0	0	0	0	0	0	0	0
TATDN2P3	0	0	0	0	0	0	0	0
TATDN3	30.714109	31.560308	18.68001	19.010578	20.704266	12.864773	0	28.021986
TAX1BP1	68.083379	85.708784	85.925236	52.871264	123.667077	58.256172	116.165365	87.447768
TAX1BP3	51.746398	83.670796	43.355317	65.181126	68.067395	141.529784	81.60704	141.541141
TBATA	0	0	0	0	0	0	0	0
TBC1D1	41.400147	33.448555	15.736429	42.003662	19.744303	38.019545	48.43617	35.871083
TBC1D10A	0	0	19.167294	3.193203	26.344143	0.274721	0	6.56849
TBC1D10B	17.632442	29.332724	17.479973	28.823898	13.434824	53.023773	9.9309	36.18673
TBC1D10C	0	0	0	0	0	0	0	0
TBC1D12	0	0.159249	0	0	0	0	0	0
TBC1D13	12.019158	14.506108	32.429657	23.006041	8.614637	20.482369	26.02006	20.125199
TBC1D14	28.965268	78.810651	42.484581	68.733268	29.362907	97.998409	37.178498	77.214687
TBC1D15	0	35.807368	47.721671	38.778515	60.163732	31.387054	0.379701	56.872305
TBC1D16	17.455406	44.637489	14.145028	40.799557	13.706627	65.302781	5.244138	39.005518
TBC1D17	11.281265	11.959118	13.591678	10.948979	37.940049	45.346817	43.411011	36.014449
TBC1D19	12.685688	5.520169	6.999239	3.202786	16.155395	4.682248	16.468688	5.338181
TBC1D2	40.258064	42.993847	43.097892	54.134407	71.512059	78.244495	58.057797	61.514459
TBC1D20	17.007929	20.238381	21.158668	22.791957	15.234257	21.493607	19.611737	20.677418
TBC1D21	0	0	0	0	0	0	0	0
TBC1D22A	15.128808	12.478318	14.307181	16.857916	2.871338	16.048835	11.368792	20.371543
TBC1D22B	10.575683	7.069723	4.31088	8.138267	2.538285	9.307304	25.482238	7.418357
TBC1D23	40.894234	33.12644	27.402134	24.548176	11.607067	29.320562	7.727568	41.395285

TBC1D24	6.398088	7.487207	0	8.578782	10.263992	18.118031	7.413821	11.512762
TBC1D25	7.876783	24.780181	11.778516	33.338541	8.319607	53.129889	3.619609	41.00891
TBC1D26	0	0	0	0	0	0	0	0
TBC1D27P	0	0	0	0	0	0	0	0
TBC1D28	0	0	0	0	0	0	0	0
TBC1D29P	0	0	0	0	0	0	0	0
TBC1D2B	0	27.681369	0	35.966611	17.984764	40.022714	4.555208	28.56368
TBC1D30	15.270114	5.986957	0	2.17283	11.943675	7.890554	0	14.141739
TBC1D31	26.449882	13.502888	28.972979	6.633856	21.616325	7.810816	20.527056	12.817747
TBC1D32	0	1.642728	2.879279	0.266308	0	1.894148	0	3.58128
TBC1D3B	0	0.957522	0	1.410255	0	1.151661	0	0
TBC1D3F	0	0	0	0	0	0	0	0
TBC1D3G	3.909065	0	0	0	2.403736	0	0	0
TBC1D3H	3.909065	0	0	0	0	0	0	0
TBC1D3I	0	0	0	0	5.459284	0	0	1.191643
TBC1D3JP	0	0	0	0	0	0	0	0
TBC1D3K	0	0	0	0	0	3.715462	0	0
TBC1D3L	3.909065	0.580254	0	4.011402	0	0.356265	0	0
TBC1D3P1	0	0	0	0	0	0	0	0
TBC1D3P2	0	0	0	0	0	0	0	0
TBC1D3P3	0	0	0	0	0	0	0	0
TBC1D3P4	0	0	0	0	0	0	0	0
TBC1D3P5	0	0	0	0	0	0	0	0
TBC1D3P6	0	0	0	0	0	0	0	0
TBC1D3P7	0	0	0	0	0	0	0	0
TBC1D4	6.305561	16.502964	4.251498	15.778175	14.668956	16.554151	1.665135	13.297181
TBC1D5	41.242453	123.140203	47.447918	104.346325	36.922656	36.770975	7.711231	56.175659
TBC1D7	10.50176	24.754988	18.854305	20.869344	22.208537	24.853525	0	37.893577
TBC1D8	9.153564	20.132184	10.674006	16.335456	6.042155	14.97139	49.917086	19.980287
TBC1D8B	13.431001	8.867873	14.500367	6.555432	4.811351	2.010513	0	4.585722
TBC1D9	6.456006	13.112278	14.226851	10.746879	3.566358	11.116935	0	11.710924
TBC1D9B	1.875461	12.484175	0	8.689178	33.300267	15.646022	98.587041	21.762849
TBCA	0	8.348646	1.358341	3.129507	0	7.609372	418.080677	12.814001

TBCAP1	0	0	0	0	0	0	0	0
TBCAP2	0	0	0	0	0	0	0	0
TBCAP3	0	0	0	0	0	1.901438	0	6.897456
TBCB	49.443969	141.60777	36.054507	144.007784	60.776994	234.909291	31.507461	203.358243
TBCCD1	17.288632	42.338239	5.895609	33.65521	8.923849	30.646579	28.474557	56.548399
TBCD	10.99135	33.455585	12.714111	32.568219	27.006108	30.72491	72.606237	33.44531
TBCE	26.383269	12.169944	16.516807	19.846038	38.576912	10.55072	100.946225	19.389055
TBCEL	11.430039	2.602514	3.426668	4.586142	42.318507	7.364457	8.117385	18.275106
TBCEL-TECTA	0	2.669827	0	0	0	0	0	0
TBCK	9.663106	6.202152	0	4.225958	8.388788	4.12027	0	1.897797
TBK1	30.152463	33.573038	27.418013	22.896771	33.429494	20.137046	37.304899	56.310805
TBKBP1	24.187066	10.444431	2.155593	9.241701	20.62167	19.359407	0	7.795395
TBL1X	0	14.823841	17.205948	19.455511	1.483256	8.222416	0	0
TBL1XR1	77.553285	355.944252	103.797561	269.368969	152.51223	127.212212	117.044482	245.60529
TBL1Y	1.480895	1.006107	1.324112	0.744156	0	2.768052	0	0
TBL1YP1	0	0	0	0	0	0	0	0
TBL2	0	14.798237	33.921191	24.706419	9.849733	19.234483	0	13.937355
TBL3	38.518484	25.037803	6.589821	12.267277	29.73894	21.458192	10.416906	24.579218
TBP	0	16.312237	0	29.894828	0	31.89147	44.458951	42.826163
TBPL1	24.441466	34.55953	25.398374	17.627617	27.401606	12.839872	59.430964	38.138208
TBPL1P1	0	0	0	0	0	0	0	0
TBPL2	0	0	0	0	0	0	0	0
TBR1	0	0	0	0	0	0	0	0
TBRG1	13.66201	26.055034	13.658671	34.583031	18.55734	71.822579	6.806432	74.473009
TBRG4	23.357512	42.709927	16.552559	39.176385	31.397242	96.824465	116.607013	94.239807
TBX1	2.459757	3.573803	0	0.497047	0	0.715787	0	0.813517
TBX15	1.263605	3.069166	1.708801	4.680036	0	0	0	0
TBX18	3.532207	18.799064	5.444248	13.951736	31.005251	19.77805	0	23.517137
TBX19	0	0.244213	0	0.904882	0	0.21399	0	0.271525
TBX2	0	1.171667	0	0.567873	0.263288	0.547219	0	0
TBX20	0	0	0	0	0	0	0	0
TBX21	0	0.167705	0	0	0	0	0	0.370212
TBX22	0	0	0	0	0	0	0	0

TBX3	3.464126	7.601954	1.244506	6.828056	4.667639	14.959562	0	22.446882
TBX4	0	0	0	0.095789	0	0	0	1.310248
TBX5	0	0	0	0	0	0	0	0
TBX6	0.977405	0.989929	0	1.742579	0	0.337675	0	0.789619
TBXA2R	0	0	1.72414	0	0	0.286309	0	0.608281
TBXAS1	0	4.257985	0	1.321557	0	2.589895	0	1.046739
TBXT	0	0	0	0.238351	0	0	0	0
TC2N	2.786614	2.927085	12.691965	3.718867	24.784675	2.443386	12.008744	9.358854
TCAF1	31.962604	23.262122	42.351385	28.033453	12.115641	15.313328	12.977519	12.597983
TCAF1P1	0	3.328551	3.668111	3.357436	0	3.610402	4.432208	1.537139
TCAF2	2.364368	1.0579	0.53119	0.848559	0.164115	0.580752	0	1.648834
TCAF2C	0	0	0	0	0	1.496727	0	0
TCAF2P1	0	0	0	0	0	0	0	0
TCAIM	12.723728	21.070922	41.163606	18.820884	11.816036	7.040271	25.234606	24.46548
TCAM1P	0	0	0	0	0	0	0	0
TCAP	0	1.23629	0	0	0	0	0	0
TCEA1	117.810202	51.757127	124.024197	17.687385	38.096158	8.564093	38.788205	26.567939
TCEA1P1	0	0	0	0	0	0	0	0
TCEA1P2	0	0.580748	24.281378	0	0	0	35.259551	0
TCEA1P3	0	0	0	0	0	0	0	0
TCEA1P4	0	0.584594	0	0.348758	1.251114	0.91381	0	1.174052
TCEA2	28.88263	35.255261	72.622165	50.173759	35.199016	63.2724	14.909592	47.682039
TCEA3	24.125427	0.180792	0	0.226165	0	0.231943	0	0
TCEAL1	41.123065	58.509794	19.926352	32.509429	22.82199	50.19893	0	51.231598
TCEAL2	3.516207	0.727183	0	2.782594	0	3.801555	0	1.384684
TCEAL3	43.355265	117.211137	44.630217	129.515045	61.460365	163.272151	67.04256	97.132722
TCEAL4	0	187.84051	138.722805	188.071589	98.118404	201.076605	22.784124	145.149088
TCEAL4P1	0	0	0	0	0	0	0	0
TCEAL5	0	0	0	0	0	0	0	0
TCEAL7	0	0	0	0	0	0	0	0.579643
TCEAL8	54.102181	91.903747	66.194094	78.853199	58.043737	54.395274	20.756834	68.880153
TCEAL8P1	0	0	0	0	0	0	0	0
TCEAL9	224.152981	389.748762	143.873402	356.590155	163.736929	288.965552	58.984471	224.883359

TCEAL9P1	0	0	0	0	0	0	0	0
TCEANC	0	0.284876	0	0.378482	0	0.357505	0	0
TCEANC2	19.685902	33.545819	5.815196	21.10324	2.764895	4.416636	1.000425	19.536843
TCERG1	32.160278	21.550204	21.802171	14.888853	138.36704	29.745332	71.862952	16.897229
TCERG1L	0	1.014115	0	0.693518	0	0	0	0
TCERG1P1	0	0	0	0	0	0	0	0
TCERG1P2	0	0	0	0	0	0	0	0
TCF12	97.968322	147.339715	47.008495	143.069501	64.610335	130.695805	83.415045	117.991914
TCF19	21.648061	98.192929	29.112542	75.63906	31.73375	127.046292	48.179233	106.038767
TCF20	26.767372	45.412463	22.133322	61.520911	50.390383	111.492959	31.581052	67.029157
TCF21	0	0	0	0	0	0	0	0
TCF23	0	0	0	0	0	0	0	0
TCF24	0	0	0	0	0	0	0	0
TCF25	76.27394	94.435529	124.865129	84.301205	42.359859	118.544069	105.058968	78.849374
TCF3	32.670308	37.489938	30.710199	25.956434	68.676363	88.909373	9.616309	68.971432
TCF3P1	0	0	0	0	0	0	0	0
TCF4	17.298244	4.219386	5.636932	7.776197	0.934537	3.357949	23.520814	3.122911
TCF7	11.978176	13.152269	15.935885	11.432506	25.901873	9.005494	0	4.34857
TCF7L1	7.875906	4.07067	5.040215	3.537264	2.969583	13.452263	9.566772	8.195375
TCF7L2	16.998501	30.049524	28.797732	49.237795	33.218331	30.002792	106.96997	28.893021
TCFL5	5.275086	10.603939	6.439795	12.699443	13.773068	14.965409	3.706036	13.843947
TCHHL1	0	0	0	0	0	0.045978	0	0
TCHP	22.330482	29.880169	9.698596	13.4551	20.767487	43.554247	67.612452	34.497121
TCIRG1	16.626665	5.819627	8.366913	7.581069	26.403617	6.19336	26.680932	4.221714
TCL1A	0	0	0	0	0	0	0	0
TCL1B	0	0	0	0	0	0	0	0
TCN1	0	0.226157	0	0	0	0	0	0
TCN2	0	2.281737	1.667492	1.567634	0	2.18789	0	0
TCOF1	72.103671	80.248766	52.808741	79.802625	3.154143	147.259435	174.184335	64.416128
TCP1	263.683007	410.564052	288.63853	326.304149	231.333636	405.543123	279.67344	754.651969
TCP10L	0	0	0	0.023587	0	0.048922	0	0
TCP10L3	0	0	0	0	0	0	0	0
TCP11	2.265296	0	0	0.709933	0	0	0	0

TCP11L1	7.670812	12.375288	9.214803	17.682279	30.695908	52.350916	50.386045	71.222278
TCP11L2	26.430362	12.738159	16.15274	24.118025	18.267128	29.236872	10.584947	33.726632
TCP11X1	0	0	0	0	0	0	0	0
TCP11X2	0	0	0	0	0	0	0	0
TCP11X3P	0	0	0	0	0	0	0	0
TCP1P1	0	0	0	0	0	0	0	0
TCP1P3	0	0	0	0	0	0	0	0
TCTA	0	0	6.738687	0.761227	29.283479	0.346845	0	0
TCTE1	1.085976	0	0	0	0	0	0	0
TCTN1	7.323676	13.272961	17.547537	10.889692	14.590617	8.058455	19.340938	6.368723
TCTN2	6.941574	21.490193	22.119243	21.501004	3.053333	9.50918	0.3283	11.045305
TCTN3	32.501172	21.421666	29.959434	24.194067	20.515441	11.015843	0	12.287998
TDG	13.661129	24.608375	24.382763	26.141381	40.400171	70.853128	77.669371	82.733293
TDGP1	0	0	0	0	0	0	0	0
TDH	0	0.32002	0	0.123144	0	0.242011	0	0
TDO2	0	1.92061	0	2.059356	0	0	0	0
TDP1	34.866867	104.183258	7.867306	105.753602	39.408017	129.42061	0	82.617862
TDP2	28.794654	19.405577	20.881063	16.017594	32.259081	24.434189	17.857661	24.551282
TDRD1	0	0	0	0	0	0	0	0
TDRD10	0	0	0	0	0	0	0	0
TDRD12	0	0	0	0	0	0	0	0.123281
TDRD15	0	0	0	0	0	0	0	0
TDRD3	5.449877	5.04299	10.511523	5.002404	4.337619	4.6444	0	5.901302
TDRD5	0	0.161351	0	0.161094	0	0.047081	0	0
TDRD6	0	0	0	0	0	0	0	0
TDRD7	7.195385	12.353263	8.87544	11.313512	10.686882	10.419779	5.688326	12.321706
TDRD9	0	0	0	0	0	0.099681	0	0
TDRKH	34.44442	45.837193	18.292567	45.057548	27.988875	12.919276	0	23.343329
TDRP	0	0	0	0	0	0	0	0
TEAD1	12.826051	25.751886	41.255245	12.772055	34.76347	16.619939	83.186144	18.381212
TEAD2	37.410632	10.383142	15.643923	11.829017	0	20.366313	23.08606	17.452008
TEAD4	0	9.029638	0	0	38.65759	0.74393	0	2.319837
TEC	2.71922	0.7368	3.738917	1.560724	2.572668	0.622692	0	0.280565

TECPR1	11.096156	12.199231	0	5.188741	50.120869	18.588225	0	5.040824
TECPR2	5.757292	6.637297	1.997151	9.297236	25.416402	14.540063	17.874082	6.883456
TECR	101.733425	97.08171	59.440512	111.683896	144.161899	199.557569	77.044611	150.573478
TECRL	0	0	0	0	0	0	0	0
TECRP1	0	0	0	0	0	0	0	0
TECRP2	0	0	0	0	0	0	0	0
TECTA	0	0	0	0.073605	0	0	0	0.080434
TECTB	0	0	0	0	0	0	0	0
TEDC1	8.067382	8.918886	7.110491	5.66347	22.236683	12.607974	21.701081	12.437388
TEDC2	4.357095	10.081721	19.957396	10.784219	10.58406	15.953597	33.41042	9.921568
TEDDM2P	0	0	0	0	0	0	0	0
TEDDM3P	0	0	0	0	0	0	0	0
TEF	1.501853	5.440601	3.371066	4.695195	1.189988	7.113839	25.979397	4.249322
TEFM	3.274058	27.796201	14.164474	18.603559	4.61316	16.325645	58.28556	32.609314
TEK	0	0	0	0	0	0.04311	0	0
TEKT1	0	0	0	0	0	0	0	0
TEKT2	0	0.708504	2.145931	0.917322	0	1.421752	0	1.17226
TEKT3	0	0	0	0	0	0	0	0.690771
TEKT4	0	0	0	0.11333	0	0	0	0
TEKT4P2	0	0.398398	0	0	0	0.756093	0	0.171031
TEKT4P3	0	0	0	0	0	0	0	0
TEKT5	0	0	0	0	0	0	0	0
TEKTL1	0	0	0	0	0	0	0	0
TELO2	4.027521	8.087871	7.221641	10.114783	11.9613	21.970176	11.259517	18.999765
TEN1	10.040804	19.368565	17.578325	19.47175	11.516279	17.952495	0	19.175718
TEN1-CDK3	2.250876	0.236805	0	0.529079	0	1.058124	0	0.315534
TENM1	0	0	0	0	0	0	0	0
TENM2	27.5472	35.852917	43.940366	49.82872	0.09013	0.205611	0.097939	0
TENM3	12.443923	20.795484	3.170372	19.926049	0	1.888511	0	2.614419
TENM4	14.067137	7.02327	0	8.533257	0	0.797089	0	2.290452
TENT2	13.911587	45.987182	14.762007	65.019341	12.12794	47.04525	38.04856	54.347025
TENT4A	22.922411	36.975471	17.527825	44.329715	23.365348	51.137808	42.230891	40.727081
TENT4B	1.031938	5.145333	0	3.7486	0.18003	1.587322	0	6.565558

TENT5A	16.348234	22.729572	20.106302	14.024883	14.310682	10.733095	7.316891	21.145454
TENT5B	0	1.644758	0	1.220786	0	2.490957	0	1.559483
TENT5C	0.576181	0.64372	1.035668	0.690258	0.456683	0.40426	0	0.855052
TENT5D	0	0	0	0	0	0	0	0
TEP1	27.181763	8.496003	8.572262	10.774951	18.570671	13.77812	5.87442	5.061682
TEPSIN	4.495487	4.176987	14.12736	2.752987	1.185686	5.143902	0	3.423336
TERB1	0	0	0	0	0	0	0	0
TERB2	0	0	0	0	0	0	0	0
TERF1	9.625436	11.700813	12.577946	7.954355	7.013847	4.117385	10.681267	7.45265
TERF1P1	0	0	0	0	0	0	0	0
TERF1P2	0	0	0	0	0	0	0	0
TERF1P3	0	0	0	0	0	0	0	0
TERF1P4	0	0	0	0	0	0	0	0
TERF1P5	0	0	0	0	0	0	0	0
TERF1P6	0	0	0	0	0	0	0	0
TERF1P7	0	0	0	0	0	0	0	0
TERF2	46.772377	60.138094	43.249368	60.591754	124.206613	60.128005	47.375676	59.3682
TERF2IP	27.515099	42.111074	27.475022	34.534607	24.754079	30.903615	59.20922	37.546146
TERF2IPP1	0	0	0	0	0	0	0	0
TERT	0	0	0	0.045271	0.371454	0	0	0.090281
TES	90.118729	230.633807	100.496732	266.472759	137.591193	233.200961	163.471121	289.300836
TESC	0	1.493964	0	0.500085	0	0	0	0
TESK1	0	8.031123	7.212169	4.646808	0	15.275647	0	12.177812
TESK2	2.608787	1.910158	4.980158	4.683227	0.344158	4.356646	0	5.922158
TESMIN	33.53041	24.591119	38.401652	26.976891	4.717307	20.293192	62.731831	15.940233
TESPA1	0	0	0	0	0	0	0	0
TET1	0.334303	0.331455	0.300864	0.376499	0	0.419982	0	0.455315
TET1P1	0	0	0	0	0	0	0	0
TET2	12.509473	3.777667	3.317932	6.660912	4.52721	1.88613	2.535358	6.749032
TET3	0	0	0	0.956049	0	0.45298	0	1.01004
TEX10	14.88809	28.969791	13.007672	14.095331	26.453231	11.54186	26.803332	27.788679
TEX101	0	0	0	0	0.657312	0.122496	0	0
TEX101P1	0	0	0	0	0	0	0	0

TEX11	0	0	0	0	0	0	0	0
TEX12	0	0	0	0	0	0	0	0
TEX13B	0	0	0	0	0	0	0	0
TEX13D	0	0	0	0	0	0	0	0
TEX14	0	0	0	0.160423	0	0	0	0
TEX14BP	0	0	0	0	0	0	0	0
TEX15	0	0	0	0	0	0	0	0
TEX19	0	0.837103	0	0.480584	0	0	0	0.217911
TEX2	1.576466	9.704097	0	5.178869	34.360689	1.9906	12.320552	4.487193
TEX21P	0	0	0	0.279702	0	0	0	0
TEX22	0	0	0	0	0	0.951134	0	0
TEX26	0	0	0	0	0	0	0	0
TEX261	32.694396	34.914567	30.20273	18.495824	42.841034	35.440716	34.444432	41.851351
TEX264	12.081924	27.190786	12.245267	21.016064	27.902882	34.032472	47.594157	14.620359
TEX28	0	0	0	0	0	0	0	0
TEX28P2	0	0	0	0	0	0	0	0
TEX28P3	0	0	0	0	0	0	0	0
TEX29	0	0	0	0	0	0	0	0
TEX30	34.006185	35.290646	26.062036	19.354124	59.534879	10.346822	14.135332	28.49589
TEX35	0	1.77622	0	0.139168	0	0	0	0
TEX36	0	0	0	0	0	0	0	0
TEX38	0	0	0	0.092294	0	0	0	0
TEX46	0	0	0	0	0	0	0	0
TEX47	0	0	0	0	0	0	0	0
TEX48	0	0	0	0	0	0	0	0
TEX50	0	0	0	0	0	0	0	0
TEX51	0	0	0	0	0	0	0	0
TEX53	0	0	0	0	0	0	0	0
TEX55	0	0	0	0.13703	0	0	0	0
TEX56P	0	0	0	0	0	0	0	0
TEX9	19.473035	3.854457	7.923219	2.956897	4.649229	1.950467	15.81029	5.524223
TF	0	1.244727	0	0.087295	0	0.008857	0	0.023297
TFAM	174.495556	209.011441	92.850996	70.163399	104.967539	49.556342	168.57968	157.168145

TFAMP1	0	0	0	0	0	0	0	0
TFAMP2	0	0	0	0	0	0	0	0
TFAP2A	18.185358	26.765421	21.25487	31.51884	31.284707	49.384095	23.73447	21.520872
TFAP2B	0	0	0	0	0	0	0	0
TFAP2C	11.767985	23.631635	9.486235	18.382101	16.387031	35.957043	79.995274	28.364131
TFAP2D	0	0	0	0	0	0	0	0
TFAP2E	0	0	0	0.254811	0	0.22746	0	0
TFAP4	20.7008	30.361466	17.074642	62.319206	8.818215	48.241824	2.129834	32.760809
TFB1M	10.157277	13.580278	2.158484	13.489938	28.126453	14.483033	123.967784	15.013405
TFB2M	19.694665	23.100918	24.585403	21.159828	39.44365	12.342675	28.571648	30.312372
TFB2MP1	0	0.391278	0	0.075599	0	0.152887	0	0
TFCP2	42.992194	59.267564	14.151549	52.40863	3.202552	31.574051	0	27.157931
TFCP2L1	2.077426	2.541294	1.557919	1.742236	5.673951	2.679094	0	3.38584
TFDP1	210.371629	354.494729	182.494023	300.470756	197.782872	467.656003	283.254669	423.21205
TFDP1P1	0	0	0	0	0	0	0	0
TFDP1P2	3.012166	0.12566	0	0.290961	0	3.534497	0	3.925488
TFDP1P3	0	0	0	0	0	0	0	0.296828
TFDP2	69.213426	242.450887	89.739456	236.23856	127.897417	118.528207	9.506069	171.728245
TFE3	40.730605	43.363581	24.578115	41.559391	39.897788	48.712807	35.148393	39.028525
TFEB	0	0.313476	0	0	0	4.074914	0	1.031137
TFEC	0	0.526576	0	0.278306	0	0.073868	0	0.120585
TFF1	0	0	0	0	0	0	0	0
TFF2	0	0	0	0	0	0	0	0
TFF3	0	0	0	0	0	0	0	0
TFG	188.759405	210.793911	118.738959	158.900129	154.231765	105.859399	132.940312	177.957966
TFGP1	0	0	0	0	0	0	0	0
TFIP11	33.219204	25.207648	11.718726	31.579	32.508526	62.169143	46.399603	42.501123
TFPI	14.361419	21.835455	18.669768	24.633737	0	3.234806	44.445737	6.803771
TFPI2	161.173291	196.055749	146.652958	143.19617	30.980273	5.481502	12.62042	8.328736
TFPT	5.338236	44.791039	5.46867	60.917903	62.585754	104.092732	88.653346	80.503895
TFR2	0	0.561177	0	1.861456	0	0.54344	0	1.206056
TFRC	117.502499	124.793055	163.027773	159.453028	227.890173	141.332786	199.876238	165.778214
TG	0	0.082084	0	0.055963	0	1.433868	0	0.580203

TGDS	17.582377	11.231506	19.163172	6.158402	9.781745	2.371332	9.920383	7.819929
TGFA	55.291547	150.199199	69.048809	171.081127	44.352492	100.222937	39.650093	138.431279
TGFB1	0	4.936519	0	3.763807	0	2.611438	23.469753	3.110438
TGFB1I1	24.507155	7.77071	10.628543	20.119718	82.953359	17.155362	84.360162	13.313725
TGFB2	10.535299	24.194936	20.841419	29.935417	12.159711	16.898816	0	35.508585
TGFB3	0	0.599429	0	0.083012	0	3.198205	0	0.288447
TGFBI	310.773231	207.541959	447.769011	286.254619	60.197466	42.429277	60.526354	41.830368
TGFBR1	23.835427	66.279402	32.881715	41.932911	13.687741	25.648045	23.447315	56.362756
TGFBR1P1	0	0	0	0	0	0	0	0
TGFBR2	30.653951	19.321909	29.448933	15.854721	43.119921	13.876458	18.921192	22.577586
TGFBR3	7.978189	3.953179	0.92075	3.551322	24.839928	10.316251	3.869722	5.685855
TGFBR3L	10.613064	16.950746	0	21.030926	0	15.463348	0	14.636594
TGFBRAP1	8.736943	20.112387	4.953927	12.233864	8.665072	41.754543	8.878685	54.126573
TGIF1	46.849574	94.930319	21.482316	148.798536	22.405144	172.736064	130.090471	144.807881
TGIF1P1	0	0	0	0	0	0	0	0
TGIF2	30.916996	48.677126	0	35.495288	21.852918	31.556493	7.27385	51.517861
TGIF2-RAB5II	0	5.476535	0	8.051422	0	1.49042	0	0
TGIF2LX	0	0	0	0	0	0	0	0
TGIF2LY	0	0	0	0	0	0	0	0
TGIF2P1	0	0	0	0	0	0	0	0
TGM1	4.411986	0	0	0.054614	17.892641	44.537567	0	29.646625
TGM2	1.87794	1.611151	1.774672	2.66952	0.49496	2.747643	0	1.451089
TGM3	0	0	0	0	0	0	0	0
TGM4	0	0	0	0	0	0	0	0
TGM5	0	0	0	0	0	0	0	0.386238
TGM6	0	0	0	0	0	0	0	0
TGM7	0	0	0	0	0	0	0	0
TGOLN2	39.221942	51.984845	48.02314	80.148576	29.92156	57.70734	0	37.286855
TGS1	0	7.771096	0	1.165279	45.465925	2.981916	0	1.809703
TH	0	0.881515	0	0.395969	0	0	0	0
THA1P	0	0	0	0	0	0	0	0
THADA	3.45846	5.26994	19.483858	1.612807	28.874622	4.554318	25.127546	7.010857
THAP1	7.165931	31.393537	7.121565	31.728661	6.305077	18.783843	0	28.26394

THAP10	8.898788	7.07915	1.510616	1.31485	1.337921	2.428052	0	9.67386
THAP12	155.821448	192.323268	151.342097	90.991495	65.261698	65.020488	64.675792	139.814267
THAP12P1	0	0	0	0.075981	0	0.156254	0	0
THAP12P2	0	0	0	0	0	0	0	0
THAP12P3	0	0	0	0	0	0	0	0
THAP12P4	0	0	0	0	0	0	0	0
THAP12P5	0	0	0	0	0	0	0	0
THAP12P6	0	0	0	0	0	0	0	0
THAP12P7	1.602152	0	0	0	0	0.272749	0	0
THAP12P8	0	0	0	0	0	0	0	0
THAP12P9	0	0	0	0	0	0	0	0
THAP2	1.483824	7.063836	2.664615	3.856872	10.422947	3.741308	0	8.349605
THAP3	0	2.874093	1.505814	6.087164	13.58224	6.374392	0	6.161636
THAP3P1	0	0	0	0	0	0	0	0
THAP4	26.64921	64.62683	35.704446	64.978278	28.545428	149.782553	25.83029	108.71324
THAP5	24.463746	16.362288	18.050379	9.361229	12.251473	8.030831	2.873377	16.832564
THAP5P1	0	0	0	0	0	0	0	0
THAP5P2	0	0	0	0	0	0	0	0
THAP6	4.865003	6.93707	5.040038	9.237953	1.717053	12.447419	16.087517	9.201976
THAP7	38.842963	31.500051	15.540604	22.809089	29.836551	52.229946	40.174267	30.170689
THAP8	0	1.065201	0	0.220476	4.404716	1.911763	0	2.108804
THAP9	1.992124	6.10347	1.786122	2.759575	4.729966	2.690017	23.143703	4.390508
THBS1	0	31.002021	0	42.986503	34.987115	380.940333	205.100875	93.349044
THBS2	0	3.322811	4.068309	5.962	0	0	0	2.200293
THBS3	5.224668	6.586962	18.92061	8.58426	19.349491	7.330077	5.789459	6.519739
THBS4	0	3.383938	4.134046	5.352437	0	0	0	0.217749
THEM4	18.732502	30.153939	9.586766	22.954736	10.649405	16.536343	2.32899	16.651939
THEM5	0	0	0	0	0	0	0	0
THEM6	9.200941	9.890679	16.448777	12.080278	13.346269	13.458665	0	7.169822
THEM7P	0	0	0	0	0	0	0	0
THEMIS	0	0	0	0	0	0	0	0
THEMIS2	0	0.849209	4.843342	2.670633	35.1585	1.921556	0	3.855128
THEMIS3P	0	0	0	0	0	0	0	0

THG1L	12.834571	13.833659	32.846325	23.6749	9.545747	12.135372	11.902304	13.584525
THNSL1	2.661061	4.365309	2.387364	2.725148	0	0.442996	0	1.924209
THNSL2	0	0	0	0.221752	0	0	0	0
THOC1	26.715988	21.25144	5.403935	16.883191	25.591422	19.059282	40.058663	28.884116
THOC2	40.785634	29.783879	42.870047	32.684396	55.075207	26.420081	102.157126	30.945978
THOC3	0	0	9.834396	41.624629	3.745732	22.558081	0	9.921647
THOC5	46.863823	42.681531	17.32957	44.065857	48.060218	61.438001	127.609516	52.21542
THOC6	14.116494	48.649655	30.064197	54.059177	21.756009	59.716466	6.420167	51.219184
THOC7	122.228903	186.400772	55.273209	108.473054	61.85373	83.000525	60.4789	172.346545
THOP1	80.19511	116.972564	76.091434	101.772445	124.903924	164.907509	83.642085	124.81858
THPO	0	0	0	0	0	0	0	0
THRA	5.112948	15.837205	13.640824	10.888637	0	15.190468	2.938099	26.240885
THRAP3	103.001458	82.691059	53.631703	91.116321	158.952132	146.068397	174.689143	124.341764
THRAP3P1	0	0	0	0	0	0	0	0
THRAP3P2	0	0	0	0	0	0	0	0
THRAP3P3	0	0	0	0	0	0	0	0
THRB	6.052306	22.105874	6.330919	23.589112	29.686194	28.229922	6.516746	19.603632
THRSP	0	0	0	0	0	0	0	0
THSD1	19.733102	41.545305	15.299465	53.33426	7.751536	31.477686	2.694182	26.274668
THSD1P1	0	0	0	5.197221	4.213626	2.081851	0	0
THSD4	14.682301	15.043986	14.470875	28.402105	17.017138	35.725644	51.353258	17.391503
THSD7A	0	0	0	0	0	0	0	0
THSD7B	4.253343	3.268868	2.389748	7.429319	0	0	0	0
THSD8	8.034042	37.805956	17.207189	36.216978	75.736349	40.627059	38.115781	41.288107
THTPA	0	3.44209	0	14.858362	0.588207	13.710136	0.539164	13.845801
THUMPD1	38.97734	24.660466	33.892128	29.432808	20.859866	24.068548	29.592312	33.47489
THUMPD1P1	0	0	0	0	0	0	0	0
THUMPD2	21.504755	21.265792	14.516487	14.818992	13.0852	7.809561	0	15.186317
THUMPD3	0	0	0	0	0	0	0	0
THUMPD3P1	0	0	0	0	0	0	0	0.098451
THY1	0	0.119435	0	0.258057	0	0	0	0
THYN1	32.535217	59.566038	52.851381	70.028006	94.361763	103.853773	50.989867	110.517023
TIA1	5.667171	51.813328	14.331103	37.061074	31.280427	45.432785	2.949883	54.326606

TIAL1	13.985875	34.479583	16.423855	19.092224	142.929115	40.894387	81.129362	33.373078
TIAL1P1	0	0	0	0	0	0	0	0
TIAM1	12.578291	28.858031	4.040258	24.389073	9.660785	26.23209	0	24.626315
TIAM2	3.119487	7.085132	4.872496	3.762377	17.226438	3.602645	0	1.368573
TICAM2	0	0	0	0	0	0	0	0
TICRR	0	1.369233	4.173707	3.85325	12.439033	12.046359	16.860051	3.830993
TIE1	0	0	0	0	0	0	0	0.043655
TIFA	15.117388	46.441342	23.506872	27.283054	2.928525	18.866382	12.916504	39.451952
TIFAB	0	0	0	0	0	0	0	0
TIGAR	2.481895	28.849322	1.559388	14.759904	47.860235	24.555246	48.707428	34.553597
TIGD3	0	1.229925	1.526028	0.747147	0	4.059463	0	2.895567
TIGD6	4.211191	14.692979	3.766999	17.270867	2.263095	9.61878	0	12.614034
TIGD7	4.383371	26.103964	5.238208	24.232298	21.625397	17.765148	0	37.777998
TIGIT	0	0	0	0	0	0	0	0
TIMD4	0	0	0	0	0	0	0	0
TIMELESS	46.810524	75.332142	26.569702	54.270031	10.722647	66.754563	57.812813	44.801021
TIMM10	20.748345	72.522873	33.930554	74.667651	76.436702	117.213013	85.905505	133.526599
TIMM10B	12.102354	39.187221	35.699277	23.904707	24.565235	27.250024	89.836982	26.42944
TIMM13	42.347154	47.413307	30.515254	49.244622	32.263182	72.66538	7.200674	59.957622
TIMM17A	107.659643	89.959807	42.121785	64.925237	54.358358	49.334417	29.074372	65.494734
TIMM17B	78.244202	61.440164	57.859083	53.642201	49.654835	44.111287	33.882483	25.292743
TIMM17BP1	0	0	0	0	0	0	0	0
TIMM21	84.339738	134.556124	33.952301	124.686184	43.071599	123.030689	41.272441	159.160408
TIMM22	9.460859	18.583772	15.07533	16.591198	14.429252	26.841349	15.584525	23.034339
TIMM23	106.484109	167.77718	85.590861	150.969947	145.698624	119.339096	64.702394	158.966064
TIMM23B	3.25478	7.458311	7.358993	9.092332	6.418141	5.935541	0	5.912881
TIMM29	13.546206	11.196186	16.839754	9.244319	26.379773	11.791381	9.133027	17.032617
TIMM44	69.478108	52.590996	16.943322	65.564762	71.607042	84.629197	84.924857	50.841362
TIMM50	82.60168	44.688755	30.98911	51.089882	57.863234	105.91314	172.635444	81.126518
TIMM8A	24.053209	39.828959	24.4912	19.617883	33.918852	19.426596	29.517765	35.002046
TIMM8AP1	0	0	0	0	0	0	0	0
TIMM8B	7.814655	57.765944	10.241266	59.383806	7.976951	68.565649	0	73.339514
TIMM8BP1	0	0	0	0	0	0	0	0

TIMM8BP2	0	0	0	0	0	0	0	0
TIMM9	140.297878	216.850324	86.021708	150.754315	73.046556	103.583744	63.664709	227.707584
TIMM9P1	0	0	0	0	0	0	0	0
TIMM9P2	0	0	0	0	0	0	0	0
TIMM9P3	0	0	0	0	0	0	0	0
TIMMDC1	82.535192	144.49375	88.58938	137.356755	161.52454	137.227238	150.09926	152.710888
TIMP1	223.554205	145.928774	246.981811	202.706919	155.781744	75.374935	84.090794	49.931018
TIMP2	37.361829	107.095287	64.618955	162.883706	30.233333	156.197569	40.671538	100.652008
TIMP4	18.716922	12.190158	22.108654	11.950199	0.820307	1.216082	20.234521	3.422795
TINAG	0	0	0	0.194178	0	0	0	0
TINAGL1	298.806907	474.014537	439.077525	601.615726	137.841334	260.448164	116.702761	155.572741
TINCR	0	1.054932	0	0	17.718234	0	0	1.248852
TINF2	16.858802	42.631012	15.612673	53.053402	9.726027	80.635865	0	72.894931
TIPARP	161.845982	341.120687	143.279076	373.887556	105.883132	173.203761	199.39583	434.35593
TIPIN	73.621256	43.959651	30.756535	40.986364	75.955279	24.032339	10.052247	31.721435
TIPINP1	0	0	0	0	0	0	0	0
TIPINP2	0	0	0	0	0	0	0	0
TIPRL	30.238172	73.165431	16.054661	36.628608	53.705642	37.059045	9.522861	59.007236
TIRAP	0	12.053926	3.928533	10.226532	14.590419	26.305577	18.112382	9.537337
TJAP1	16.512928	60.362759	9.448389	69.545589	10.593464	96.763661	22.41754	64.078212
TJAP1P1	0	0	0	0	0	0	0	0
TJP1	95.658467	125.512804	85.223535	93.345692	148.365294	95.09432	62.54742	112.179508
TJP2	72.582611	150.059493	50.986171	175.249669	73.799622	279.324612	58.611803	222.220148
TJP3	19.301826	4.272677	14.267797	15.287737	0	1.767927	0	0.458968
TK1	121.286463	63.335077	62.887594	60.094345	112.080417	91.58289	30.452465	77.372664
TK2	0	11.521435	20.991993	8.273804	9.258642	7.624955	72.470884	19.315921
TKFC	2.376306	36.971131	0	42.362369	64.212163	62.133387	23.656002	50.039253
TKT	350.784364	467.572153	485.25428	548.489458	320.452619	351.65546	159.285958	230.394371
TKTL1	0	0	0	0	0	0	0	0.05437
TLCD1	22.03118	68.72053	49.338979	62.760878	24.114454	52.701079	0	35.113329
TLCD2	12.717461	12.479192	11.431137	13.907902	4.96712	8.347353	8.355268	4.922725
TLCD3A	18.537164	39.322646	19.330083	30.886041	25.258619	64.820243	12.097687	69.118918
TLCD3B	0	0.820429	0	2.295973	0	0.119279	0	0.269326

TLCD4	9.044441	4.343676	10.273504	2.356656	3.647885	0.130937	4.294521	0.903628
TLCD4-RWDI	0	7.098673	0	4.397546	0	0.928162	0	2.086715
TLCD5	22.578993	30.712378	42.203537	19.534495	1.853426	6.766384	0	6.757909
TLDC2	0	0	0	0.076271	0	0	0	0
TLE1	7.976764	78.527592	20.924592	61.472215	54.335067	100.820217	54.202751	89.726549
TLE1P1	0	1.808253	0	0.847971	0	3.026491	5.459077	0.684117
TLE2	22.536355	1.226589	4.114178	1.386546	0	0.608592	0	0.633333
TLE3	35.120403	92.126989	22.549439	76.389917	31.153472	112.630679	17.254451	113.325551
TLE4	19.401762	3.69158	3.624021	14.4407	3.55249	17.106659	0	7.827686
TLE5	0	56.750555	71.263163	39.067387	94.041092	50.934041	62.380492	14.889623
TLE6	0	3.225272	0	4.604295	0	1.481457	0	0.861269
TLK1	49.043852	95.111915	63.199945	107.351528	37.66503	114.480004	63.427455	133.386694
TLK1P1	0	0	0	0	0	0	0	0
TLK2	16.566346	62.146178	42.400391	44.255091	39.916657	29.810188	20.773786	43.034567
TLK2P1	0	0	0	0	0	0	0	0
TLK2P2	0	0.196114	1.370071	0	0	0	0	0
TLL1	4.108188	14.320284	4.304875	23.746578	37.250867	19.668876	14.34071	29.887045
TLL2	0.478625	3.415078	0.430396	3.397793	6.420986	4.100471	1.400411	2.467843
TLN1	36.227482	63.624784	28.227906	78.305326	74.680072	160.894695	150.487483	85.670756
TLN2	0.634014	1.015371	1.70887	1.695399	2.697116	5.305062	0.154453	6.740986
TLR1	0	0	0	0.931193	0	0	0	0
TLR10	0	0	0	0	0	0	0	0
TLR12P	0	0	0	0	0	0	0	0
TLR2	0	0.487597	0	0.131811	0	0	0	0.207791
TLR3	0	1.57977	4.194962	3.079664	0	2.82159	0	4.29824
TLR4	1.513731	3.663658	3.056653	1.923599	0	0.89692	0	3.054453
TLR5	0	0.410577	0	0.273475	0	0	0	0.041802
TLR7	0	0	0	0	0	0	0	0
TLR8	0	0	0	0	0	0	0	0
TLX1	0	1.609034	0	1.072914	0	0.647968	0	0.461626
TLX2	0	0.403504	0	0.231293	0	0	0	0
TLX3	0	0	0	0.112978	0	0	0	0
TM2D1	69.776455	50.525553	147.461721	34.807498	34.766723	9.137578	0	26.735878

TM2D2	50.990025	112.967517	23.842122	77.604885	54.381173	44.358703	58.412591	64.286354
TM2D3	13.759085	12.771191	19.484633	7.411852	17.917347	10.821951	21.198511	18.074106
TM4SF1	70.641561	53.779133	63.780801	50.710132	24.816913	15.757477	6.101568	9.188757
TM4SF18	0.889524	0.038065	6.16929	0	0	0	0	0
TM4SF19	0	0.928175	8.477149	2.062103	0	0.30207	0	0.793062
TM4SF19-DY	0	1.741976	3.559699	0	2.120471	0	0	0
TM4SF20	0	0	0	0	0	0	0	0
TM4SF4	0	0	0	0	0	0	0	0
TM4SF5	0	0	0	0.28175	0	0	0	0
TM6SF1	0	0	0	0.948047	0	0.224581	0	0
TM6SF2	0	0	0	0.540845	0	0.348173	0	0.191227
TM7SF2	8.727906	20.01744	8.583723	6.356094	42.682503	10.96522	0	12.492876
TM7SF3	91.009172	110.087764	38.356477	70.257814	55.744472	29.576328	59.969386	56.185726
TM9SF1	24.874955	103.527742	50.434227	121.882737	97.740961	122.149661	65.498694	105.859594
TM9SF2	0	31.398745	3.951326	32.198722	0	9.942309	0	12.87708
TM9SF3	169.009246	180.673164	196.931604	49.450629	59.120631	22.048437	293.207722	89.450317
TM9SF4	60.607904	0	0	10.893459	0	3.390147	0	5.550894
TM9SF5P	0	0	0	0	0	0	0	0
TMA16	46.682311	74.439763	42.528019	71.389271	61.000512	40.13978	10.221141	48.974809
TMA16P1	0	0	0	0	0	0	0	0
TMA16P2	0	0	0	0	0	0	0	0
TMA7	308.510284	308.190471	114.887512	261.381043	191.048556	448.721672	62.339344	276.15151
TMBIM1	96.329299	83.888158	154.637039	135.895179	177.646129	132.821434	173.504871	110.805609
TMBIM4	17.079766	29.105463	19.466611	8.743805	15.878915	10.254534	122.131852	43.716503
TMBIM6	247.080325	310.621215	289.051253	287.465706	235.677424	160.282502	551.376367	136.944606
TMBIM7P	0	0	0	0	0	0	0	0
TMC1	0	1.696804	0	1.127275	0	0	0	0.747088
TMC2	0	0	0	0.092644	0	0	0	0
TMC3	0	0	0	0	0	0	0	0
TMC4	12.300124	8.655103	0	6.371401	7.714537	8.045775	0	0
TMC5	0	1.411827	0.807087	1.047004	0.237554	3.992701	0	1.877442
TMC6	23.60357	37.064102	17.490431	32.564223	7.425908	31.455696	82.334203	18.050684
TMC7	3.736827	4.537899	4.697206	2.774609	4.160403	0.876988	0	2.55001

TMC8	0.841999	3.316962	1.511181	4.143689	21.03712	1.689351	0.20488	1.694189
TMCC1	31.142272	62.342589	31.76543	40.70623	5.434637	30.101467	7.288448	59.665788
TMCC1P1	0	0	0	0	0	0	0	0
TMCC2	3.796904	1.926051	2.686781	2.632385	0.333955	2.871829	0	1.672456
TMCC3	4.985143	14.580242	4.064563	15.482268	35.015901	18.115836	4.224755	19.23925
TMCO1	69.437779	84.423787	59.145547	82.27557	82.077723	52.85013	54.61997	76.980273
TMCO2	0	0	0	0	0	0	0	0
TMCO3	48.882025	43.972829	47.569501	40.536255	16.010196	33.975086	130.870444	61.301886
TMCO4	5.6807	5.966614	15.710947	8.337665	0	12.769032	10.178074	6.029296
TMCO5A	0	0	0	0	0	0	0	0
TMCO5B	0	0	0	0	0	0	0	0
TMCO6	18.501943	0.872867	3.406944	2.011614	7.148008	0.559961	0	0.532935
TMED1	17.906076	24.447846	17.941554	24.974589	4.009933	30.424151	8.40321	10.755169
TMED10	235.019713	178.170712	332.339307	235.999858	253.962074	136.002219	169.706793	128.123228
TMED10P1	0	0	0	0.157835	0	0	0	0
TMED10P2	0	3.480586	0	3.287063	0	1.370584	0	2.443467
TMED11P	0	0	0	0	0	0	0	0
TMED2	192.288964	337.932464	360.873707	275.900305	128.832884	161.904903	55.407588	187.012789
TMED2P1	0	0	0	0	0	0	0	0
TMED3	132.64492	172.978538	134.605612	222.147582	27.699164	120.123581	38.790438	136.47117
TMED4	26.041366	31.182472	40.059312	33.910128	53.727802	47.245379	65.174882	38.768057
TMED5	129.618549	105.374085	107.967871	60.679289	42.329823	18.232704	60.641014	29.728107
TMED6	0	0	0	0	0	0.623547	0	0
TMED7	79.736866	80.532402	51.47739	67.735077	53.083883	43.223548	41.484992	64.434614
TMED7-TICA	7.898093	8.12565	21.9996	10.539834	1.213645	8.676533	0	6.734664
TMED8	39.932729	26.724704	39.289352	20.799642	21.656454	12.843963	23.944292	16.171518
TMED9	0	0	0	5.52344	0	17.468242	0	25.202761
TMEFF1	0	0	0	0.282139	0	0	0	1.472314
TMEFF2	0	0	0	0	0	0	0	0
TMEM100	0	0	0	0	0	0	0	0
TMEM101	16.448135	5.919248	27.178844	8.738112	8.043996	9.533888	0	6.511112
TMEM102	1.772343	7.150758	9.492563	9.715578	3.169488	11.335569	0.429006	11.57817
TMEM104	3.705023	2.38749	3.610356	1.711829	0	0	0	1.417818

TMEM106A	2.045119	3.146647	4.583403	5.912182	3.239257	4.028857	0.606175	4.539015
TMEM106B	7.871321	5.319756	14.457617	3.561168	5.359851	2.769176	34.915222	5.05901
TMEM106C	157.712467	151.465376	160.596408	143.839922	44.752766	71.139369	99.760868	85.707914
TMEM107	14.415071	28.231127	35.973057	34.545365	3.279324	11.57996	17.710659	14.042773
TMEM108	0	3.319814	0	2.272978	0	0.658418	0	1.41617
TMEM109	16.201992	25.882701	17.370321	21.931485	41.441496	40.210292	55.209389	39.050693
TMEM11	27.86413	60.476801	26.305703	58.920017	8.584135	85.906667	27.107374	100.300734
TMEM114	0	0	0	0	0	0	0	0
TMEM115	14.74858	25.276336	7.319651	32.930663	7.777869	42.041632	0.396962	26.76503
TMEM116	0	5.99528	0	6.17139	12.299734	10.581796	0	17.778978
TMEM117	2.563175	2.539471	8.518803	2.538927	3.209688	2.846168	0	7.230379
TMEM119	0	0	0	0	0	0	0	0
TMEM120A	5.395295	8.040493	14.949547	5.436653	17.862813	5.015165	0	7.117273
TMEM120B	7.774802	7.377834	5.075998	12.980686	11.667784	12.70042	17.188727	7.342395
TMEM121	0	10.828443	0	2.408628	0	0	0	2.33715
TMEM121B	0	0	0.748822	0	0	0.083394	0	0.067023
TMEM123	2305.06274	2573.94602	2889.27603	2806.08867	453.32486	203.024027	325.126659	320.192652
TMEM125	1.953597	30.817655	10.452639	39.25486	0	11.408097	0	9.287515
TMEM126A	82.160302	90.690846	52.67289	55.809392	41.18118	24.336124	18.183643	66.376196
TMEM126B	22.377488	37.106256	46.240664	36.87384	7.757416	9.566916	66.496491	13.674458
TMEM128	11.900813	8.846935	10.55158	3.549423	0.782574	1.930289	10.014739	3.721921
TMEM129	0	14.264175	4.524878	14.745607	0.922475	26.903744	2.453906	18.800891
TMEM130	0	0	0	0.073787	0	0	0	0
TMEM131	10.156004	9.877253	10.436989	7.62132	33.753984	7.140879	49.630647	8.805598
TMEM131L	6.127111	5.08912	2.062765	2.138696	4.143793	3.339797	2.796605	2.779872
TMEM132A	20.390286	14.628377	53.173818	37.46591	11.235145	46.04629	66.546914	30.048715
TMEM132B	0	0	0	0	0	0.083375	0	0.135878
TMEM132D	0	0	0	0	0	0	0	0
TMEM132E	0	0	0	0.028292	0	0	0	0
TMEM134	25.08926	52.027138	58.166345	40.074239	13.385961	33.7983	0	20.352698
TMEM135	84.636893	37.273561	40.634324	37.979172	22.777314	5.836488	6.332754	19.29693
TMEM138	61.392958	43.944876	19.287348	33.350966	28.935813	70.137508	0	65.666077
TMEM139	6.411677	3.803794	5.577486	7.38218	0	2.793201	0	1.550392

TMEM140	3.476271	1.6928	4.655575	1.520661	3.248939	5.58936	18.93629	3.087935
TMEM141	62.516634	90.599503	77.516769	104.087763	46.95258	95.72852	1.489599	73.523425
TMEM143	0	4.246051	0	4.667184	1.990298	5.271349	0.858968	5.545301
TMEM144	0	2.611042	12.29469	1.782242	0.310682	1.938351	7.433702	2.800901
TMEM145	0	0.247333	0	0.562091	0	0.773097	0	0.37164
TMEM147	44.496198	77.266641	48.551722	56.896594	30.19045	58.319164	111.496131	75.589975
TMEM14A	47.020287	43.052278	27.646537	31.388183	13.375071	21.958067	42.172648	44.771584
TMEM14B	0	35.669024	79.433816	11.783546	115.661681	15.096112	73.740247	49.27075
TMEM14C	75.731068	100.78354	62.819602	60.641799	75.581733	54.198954	0	92.802596
TMEM14DP	0	0	0	0	0	0	0	0
TMEM14EP	0	0	0	0	0	0	0	0
TMEM150A	0	5.166276	7.584492	3.2615	11.662195	0.714069	0	1.391953
TMEM150B	0	0	0	0	0	0	0	0
TMEM150C	0	1.612602	0.943459	0.205312	0	0	0	0
TMEM151A	1.338743	2.562815	0	5.441929	0	0.532197	0	0.053905
TMEM151B	0	0	0	0	0	0	0	0
TMEM154	13.622644	24.581528	9.678491	17.18842	16.404975	12.683688	4.162122	10.753031
TMEM156	25.972934	34.512076	42.855749	29.746115	17.389193	4.498838	0	8.761301
TMEM160	0	8.239271	5.804863	5.524223	19.201249	16.306922	11.023084	13.557764
TMEM161A	2.143589	7.341748	19.392473	7.788071	23.27042	5.031098	0	8.477961
TMEM161B	15.314605	9.313004	0	3.851633	2.332394	5.490232	67.298776	7.288331
TMEM161BP:	0	0	0	0	0	0	0	0
TMEM163	0	0	0	0	0	0.091168	0	0
TMEM164	9.209657	16.021182	14.77356	19.271367	12.684169	20.578654	12.81871	20.660013
TMEM165	18.037855	38.103318	42.008559	34.675921	15.422757	23.526865	5.394872	33.650501
TMEM167A	108.56102	58.708993	50.102908	39.840841	89.421738	25.546569	69.16095	58.231186
TMEM167AP.	0	0	0	0	0	0	0	0
TMEM167AP:	0	0	0	0	0	0	0	0
TMEM167B	67.105148	69.425878	57.757943	54.905479	38.666889	12.955053	55.063895	26.694182
TMEM168	0	9.283495	7.611846	8.373607	3.635114	5.730355	32.854277	7.604814
TMEM169	0	0	0	0.10317	0	0.364638	0	0
TMEM17	10.717946	2.035579	5.728443	3.181116	0.851409	1.427228	11.391376	1.73344
TMEM170A	60.113348	34.351849	15.323408	22.481106	28.683397	19.566505	33.269641	40.639528

TMEM170B	0	0.077993	0.325725	0.035444	0	0.054554	0	0.188533
TMEM171	0	3.703943	10.541796	2.719258	0.779646	8.39137	10.720126	12.539785
TMEM174	0	0	0	0	0	0	0	0
TMEM175	3.534748	3.094238	11.045233	7.094845	0	6.489617	0	3.29502
TMEM176A	0	0	0	0	0	0	0	0
TMEM176B	0	0	0	0	0	0	0	0
TMEM177	21.692576	24.052657	9.630868	26.221166	6.422194	25.685036	27.233213	36.43257
TMEM178A	0	0.957521	0	0.652438	0	0.10488	0	0
TMEM178B	0	2.227059	0	3.118389	0	0	0	0
TMEM179	0	0	0	0	0	0	0	0
TMEM179B	11.002772	20.894086	27.508851	25.763463	4.037753	24.279252	39.529033	35.582497
TMEM18	17.35922	27.3404	34.054914	23.534024	24.420942	12.53806	30.127646	15.480896
TMEM182	23.421732	8.235864	7.930853	11.745751	7.438873	7.625568	0	10.375045
TMEM183A	58.647409	21.269637	35.09258	28.187128	39.920821	29.153315	12.83864	29.855214
TMEM183AP	0	0	0	0	0	0	0	0
TMEM183AP	0	0	0	0	0	0	0	0
TMEM183AP	0	0	0	0	0	0	0	0
TMEM183AP	0	0	0	0	0	0	0	0
TMEM183AP	0	0	0	0	0	0	0	0
TMEM183AP	0	0	0	0	0	0	0	0
TMEM183BP	0	0	0	3.948457	33.442864	4.194641	65.878975	4.278687
TMEM184A	17.19025	39.992491	32.743269	59.334724	10.212728	25.23715	5.241556	19.916218
TMEM184B	23.853496	28.865286	26.581654	39.437975	53.765038	54.03204	18.019239	44.89119
TMEM184C	0	28.870086	45.133441	37.834261	0	30.038321	5.558163	36.961104
TMEM184CP	0	0	0	0	0	0	0	0
TMEM185A	17.525286	36.628731	17.077045	42.340714	59.623722	36.341329	0	39.537107
TMEM185AP	0	0	0	0	0	0	16.869297	0
TMEM185B	9.337616	35.463106	10.861951	34.858389	14.802782	42.456894	16.332477	48.313245
TMEM186	25.106994	42.70857	33.478131	44.251747	7.931526	21.676181	13.314897	21.867313
TMEM187	7.454901	17.228013	8.837283	23.109579	1.308435	13.48364	13.180326	7.318294
TMEM19	0	15.831182	62.752233	22.14761	46.71237	23.971246	0	68.440442
TMEM190	0	0	0	0.175751	0	0	0	0
TMEM191A	0	0.589919	0	0	0	0	0	0

TMEM191B	0	0	0	0	0	0.298333	0	0
TMEM191C	0	0	0	0.033228	0	0	0	0.217887
TMEM192	14.719182	24.535961	17.906529	12.918187	16.721878	6.138648	1.495978	13.723629
TMEM196	0	0	0	0	0	0	0	0
TMEM198	0	1.139743	0	0	0	0.156169	0	0
TMEM198B	0	0	0	0.174883	0	0	0	0
TMEM199	4.3499	21.519868	7.796123	14.831724	43.875867	15.175795	50.08228	16.161605
TMEM200A	3.482291	0	0	1.05805	0	0	0	0.560352
TMEM200B	1.379766	8.99895	8.640778	6.802451	2.547955	5.4871	0	3.58863
TMEM200C	0	0.219145	0	0.044775	0	0	0	0
TMEM201	16.463171	8.07772	13.75953	11.68155	8.477745	18.367644	13.15164	14.521756
TMEM202	0	0	0	0	0	0	0	0
TMEM203	29.619147	93.92806	24.336274	70.761063	21.002177	83.20202	27.496889	93.649623
TMEM204	0	0	0	0	0	0	0	0
TMEM205	49.613745	64.029406	63.826346	77.271495	27.206131	28.839404	0	24.039152
TMEM208	15.98763	46.898594	27.931146	61.566418	62.405587	34.128563	0	33.521896
TMEM209	39.773272	17.319302	33.062762	19.092836	24.492986	10.679557	12.865712	10.548044
TMEM210	0	0	0	0	0	0	0	0
TMEM212	0	0	0	0.138603	0	0	0	0.14994
TMEM213	0	0	0	0	0	0.039978	0	0
TMEM214	41.726941	16.983732	38.400974	30.631382	28.457187	25.279327	29.86782	19.463481
TMEM215	0	0	0	0	0	0	0	0
TMEM216	0	0	0	1.788575	0	0	18.686925	6.749899
TMEM217	0	1.279209	0	0.604522	0	0.7919	0	3.05591
TMEM217B	0	0	0	0	0	0	0	0
TMEM218	14.193477	37.880682	3.419241	33.754394	36.483148	29.98294	0	39.084424
TMEM219	50.117058	24.679709	53.041362	33.701851	24.795839	26.632709	0	20.675007
TMEM220	0	0.216563	0	0.196145	0	0	0	0
TMEM221	0	0.216701	0	0.704504	0.449824	0.253312	0	0.481804
TMEM222	7.123548	2.091589	19.01192	15.780462	18.132726	14.240808	37.24976	6.614059
TMEM223	0	1.350163	0	0.609523	4.3196	0.598404	0	1.018295
TMEM225	0	0	0	0	0	0	0	0
TMEM225B	0	0	0	0	0	0	0	0

TMEM229B	0	0	0	0	0	0	0	0
TMEM229BP:	0	0	0	0	0	0	0	0
TMEM230	7.314656	22.156794	24.9281	64.645726	38.520369	49.189725	0	34.422614
TMEM230P1	0	0	0	0	0	0	0	0
TMEM230P2	0	0	0	0	0	0	0	0
TMEM231	5.42983	14.572331	36.781818	16.067576	27.660213	11.109818	0	12.655847
TMEM231P1	0	0	0	0	0	0	0	0
TMEM232	0	0.452589	0	2.68318	0	0	0	1.070406
TMEM233	0	0	0	0	0	0	0	0
TMEM234	5.864206	4.148531	0	3.091068	10.809656	10.744237	19.427677	5.083739
TMEM235	0	0	0	0	0	0	0	0
TMEM236	0	0	0	0	0	0	0	0
TMEM237	5.293412	14.05196	20.963323	21.644943	14.963082	41.81764	23.975254	34.335039
TMEM238L	0	0	0	0	0	0	0	0
TMEM239	0	0	0	0	0	0	0	0
TMEM240	0	1.715085	0	0.362006	0	0.210336	0	0
TMEM241	21.09004	32.210787	12.515468	29.519888	4.988961	9.010274	46.786784	17.621151
TMEM242	0	0	0	1.347802	0	0	0	2.498716
TMEM243	17.941301	22.039208	15.852663	18.013401	43.994775	12.296126	0	19.847223
TMEM245	21.13928	13.017642	18.54802	11.559139	33.723792	9.796033	4.724728	10.992386
TMEM247	0	0	0	0	0	0	0	0
TMEM248	78.442643	122.551702	41.83701	148.481785	79.862801	155.080864	54.048715	174.677302
TMEM248P1	0	0	0	0	0	0	0	0
TMEM249	0	0	0	0	0	0	0	0
TMEM25	95.757654	87.042581	67.910546	145.211862	2.798611	7.982884	19.217201	7.22428
TMEM250	28.617119	55.933558	23.493527	51.332845	11.023167	72.376105	17.958713	59.284303
TMEM253	0	1.942294	0	0.737638	0	1.598381	0	1.42555
TMEM254	29.383383	27.677836	21.125601	25.802927	7.471324	2.17914	26.461435	17.572603
TMEM255A	7.383069	0	0	2.282804	3.394724	4.313573	0	2.805003
TMEM255B	0	18.62685	0	6.54564	0	2.577701	0	7.371558
TMEM256	70.535404	36.660997	94.608091	40.623267	135.220336	19.67685	57.760885	19.728524
TMEM256-PI	0	0	0	3.26052	0	0.424556	0	1.094108
TMEM256P1	0	0	0	0	0	0	0	0

TMEM256P2	0	0	0	0	0	0	0	0
TMEM258	2.475109	3.611249	0	2.742067	0	1.848854	19.889992	3.70494
TMEM258P1	0	0	0	0	0	0	0	0
TMEM259	77.27803	97.703245	81.471017	118.605181	55.847155	191.052087	122.68418	120.915964
TMEM26	0	0	0	0	0	0	0	0
TMEM260	3.093624	3.857389	1.536923	3.143607	9.498194	0.889513	0	5.365629
TMEM262	0	0.034227	0	0.039013	0	0.279562	0	0
TMEM263	87.259041	200.650384	53.143777	230.619085	26.510191	183.250188	50.320443	183.175843
TMEM266	0	0.076228	0	0.087527	0	0.435367	0	0
TMEM267	3.861926	24.114484	9.962882	8.585563	29.333316	5.266817	33.304589	25.39969
TMEM268	5.123438	19.191873	10.515505	10.724892	4.059681	11.637475	33.048207	14.545825
TMEM269	0	0.364175	0	0.937793	0	0	0	0
TMEM270	0	0.769503	0	1.679023	0	1.130889	0	0.387022
TMEM272	0	0	0	0	0	0	0	0
TMEM273	0	0	0	0	0	0	0	0
TMEM274P	0	0	0	0	0	0	0	0
TMEM275	0	0	0	0	0	0	0	0
TMEM276	16.10757	9.419797	8.557006	12.711147	4.12827	7.447275	0	3.400528
TMEM276-ZI	0	10.277205	10.611943	9.283295	0	10.915431	27.158209	10.526303
TMEM277P	4.189161	0	3.689008	0	0	0	0	0
TMEM30A	29.774598	40.882597	85.785643	12.952956	67.517625	11.967589	15.586092	45.434809
TMEM30B	0	0	0	10.641331	0	47.184543	0	0
TMEM30BP1	0	0.741405	0	0.689773	0	0.173966	0	0
TMEM30CP	0	0	0	0	0	0	0	0
TMEM31	0	0.231135	0	0.136404	0	0.272254	0	0.236732
TMEM33	91.46944	34.717552	85.619247	21.121203	47.359873	21.447027	78.827467	35.390429
TMEM35A	0	0.719291	1.517067	0.536439	0	0	0	0
TMEM37	0	0.176625	0	0.203238	0	0	0	0.593242
TMEM38A	10.302972	8.796315	9.222278	7.229509	0.679618	5.625545	0	7.327969
TMEM38B	6.563803	10.578695	3.364002	12.346233	5.694152	6.306662	24.987885	14.984853
TMEM38BP1	0	0	0	0	0	0	0	0
TMEM39A	26.032119	66.506799	40.955439	105.203177	43.706161	67.483418	54.923122	81.927796
TMEM39B	12.446059	14.155954	19.519954	17.230378	8.967501	18.87282	23.515461	11.682891

TMEM40	40.990139	45.93541	52.936288	83.909612	54.666674	60.383177	67.686743	32.1851
TMEM41A	54.89396	55.665767	29.670946	39.684041	25.943095	28.386609	113.969372	44.815445
TMEM41B	27.949608	19.482756	29.732117	20.375601	24.490524	27.076953	18.245429	26.641354
TMEM42	11.918048	20.139932	14.09308	22.701058	1.043006	7.737362	0	10.464586
TMEM44	21.701466	12.785788	34.919851	28.725773	20.776254	2.22618	0	12.096079
TMEM45A	24.13449	21.605929	34.324594	18.625472	27.002799	16.111161	72.608525	28.962537
TMEM45B	2.165682	4.397506	0	2.979338	0	1.223327	0	8.741916
TMEM47	2.45198	2.030514	5.134871	2.693882	0	0.122552	0	0.098466
TMEM50A	74.584739	102.657497	88.896815	74.859277	80.041317	85.38896	132.445327	121.236908
TMEM50B	5.831801	15.556917	6.559441	7.517195	10.156919	3.841014	6.758891	15.361338
TMEM51	3.801175	34.880675	6.296828	41.996191	12.086739	61.844725	0	46.931756
TMEM52	0	0	0	3.875854	0	2.333628	0	1.028499
TMEM52B	15.091615	91.957418	21.049131	72.353093	5.020704	8.622008	3.480806	13.174332
TMEM53	2.446393	3.871634	1.729738	5.862381	3.59054	2.955526	0	3.344618
TMEM54	14.895687	48.58962	25.128378	45.654044	19.563196	66.20565	57.956338	59.715429
TMEM59	236.004382	204.311411	311.839844	220.037746	123.119033	57.869025	167.446332	93.021145
TMEM59L	0	1.845048	0	2.708766	0	0.323825	0	0.088684
TMEM60	13.63645	24.867234	7.989589	16.076622	33.378135	6.107146	37.919322	12.281907
TMEM61	0	9.062606	7.819337	11.908909	0	0.574014	0	0.954634
TMEM62	6.245417	11.749494	14.538044	14.345954	7.25116	13.616333	78.038778	15.241077
TMEM63A	9.435986	5.782739	4.324306	7.076046	8.694202	9.550865	26.607898	8.771065
TMEM63B	10.362912	19.724882	15.49362	26.002249	49.132101	42.005443	44.279104	24.610822
TMEM63C	1.825659	3.175892	1.093654	5.079092	0.321541	1.243897	0	1.464929
TMEM64	5.629217	9.911755	8.244053	2.580641	3.717234	4.325571	7.664135	7.963439
TMEM67	17.039033	1.648511	6.876085	6.111759	0	0	0	0
TMEM68	42.810131	48.016627	34.753479	29.699521	27.54986	10.23519	9.311185	25.497937
TMEM69	73.792701	36.438095	52.493219	34.919364	62.170595	22.576233	32.028008	54.519719
TMEM69P1	0	0	0	0	0	0	0	0
TMEM69P2	0	0	0	0	0	0	0	0
TMEM70	18.757459	16.935733	18.273084	9.732095	37.157705	7.641901	0	18.46213
TMEM71	0	0.66568	0	1.339868	0.495254	2.152343	0	0.63576
TMEM72	0	0	0	0	0	0	0	0
TMEM74	0	0.111114	0.464589	0.101108	0	0.025915	0	0.12432

TMEM74B	0	0.687375	0	1.38357	0	0	0	0
TMEM79	3.197662	3.929293	7.016082	9.694803	0.471181	6.07391	0	4.823712
TMEM80	0	0.235392	1.510616	0.139744	0	1.336543	0	3.977682
TMEM81	0	2.178328	0	2.982103	0	1.611495	0	2.115076
TMEM82	0	0	0	0	0	0	0	0
TMEM86A	0	0.472598	0	1.235583	0.243096	0.827363	0	1.072798
TMEM86B	0	0.857119	2.117156	0.896543	0	1.320947	0	0.6406
TMEM87A	0	33.253899	0	3.332978	25.156235	1.915115	0	10.861403
TMEM87B	0	8.110606	0	3.201613	20.505243	9.71651	0	6.147249
TMEM88	0	0	0	0	0	0.439081	0	0
TMEM8B	0.226296	1.366116	0	1.499471	1.015083	2.063334	0	0.828169
TMEM9	37.280315	35.911753	50.029119	52.502265	24.677489	26.535871	48.047688	27.355452
TMEM91	23.463174	20.015978	8.138296	18.471878	33.694478	5.274173	0	1.709975
TMEM92	1.256052	7.167873	5.622529	6.74653	0.331433	1.821227	0	0.960528
TMEM94	60.396887	14.636818	9.913693	22.539764	46.386548	37.512002	28.64386	19.770519
TMEM95	0	0.398514	0	0	0	0	0	0
TMEM97	42.866557	59.129469	25.978749	29.634698	28.453616	17.776573	33.880021	27.124202
TMEM97P1	0	0	0	0	0	0	0	0
TMEM97P2	0	0	0	0	0	0	0	0
TMEM98	19.600649	12.841385	0	10.93405	12.916505	9.870471	0.507893	10.290441
TMEM9B	30.162581	31.216563	20.319873	16.833792	23.637285	22.301869	0	46.951449
TMF1	17.037962	24.878447	33.034574	25.064995	15.543834	14.635268	16.683607	23.980499
TMF1P1	0	0	0	0	0	0	0	0
TMIE	0	0.823039	0	3.005257	0	0	0	0.263642
TMIGD1	0	0	0	0	0	0	0	0
TMIGD2	0	0	0	0	0	0	0	0
TMIGD3	0	0	0	0	0	0	0	0
TMLHE	5.018478	18.335364	5.254211	16.488374	3.092245	10.694148	36.323388	24.938102
TMLHEP1	0	8.280144	0	10.803142	0	7.013078	114.228476	16.31523
TMOD1	0	2.801894	11.109516	4.990784	0	1.005324	0	1.053324
TMOD2	17.179216	7.237033	6.13257	4.067235	0.743302	2.371468	1.801232	2.967054
TMOD3	64.776838	90.149997	42.984939	72.92404	84.444605	84.895568	68.307199	163.828892
TMOD4	0	0	0	0.509495	0	0	2.78225	0

TMPO	70.00132	191.173579	80.706266	176.135585	194.744089	326.529288	210.252295	450.905278
TMPOP1	0	0	0	0.070179	0	0	0	0.118098
TMPOP2	0	7.600714	0	3.433925	0	3.321993	0	7.049501
TMPPE	1.929325	1.413361	0.865063	1.307897	3.310859	1.451621	0	1.64196
TMPRSS11A	0	0	0	0	0	0	0	0
TMPRSS11B	0	0	0	0	0	0	0	0
TMPRSS11BN	0	0	0	0	0	0	0	0
TMPRSS11CF	0	0	0	0	0	0	0	0
TMPRSS11D	0	0	0	0	0	0	0	0
TMPRSS11E	4.87815	3.76744	7.211922	2.201729	5.959866	4.114037	0	3.405792
TMPRSS11GF	0	0	0	0	0	0	0	0
TMPRSS12	0	0	0	0.059856	0	0	0	0
TMPRSS13	20.383899	26.601637	24.374558	22.848776	2.762386	3.956064	4.668127	4.363768
TMPRSS15	0	0	0	0.289477	0	0	0	0
TMPRSS2	11.5635	0.051192	0.924433	1.247846	0	1.124115	0	0.421336
TMPRSS3	0	0	0	1.597752	4.758075	1.731164	0	0.993107
TMPRSS4	22.666774	0.69286	49.401562	14.067715	27.239683	19.28451	12.484332	5.343505
TMPRSS5	0	0.245259	0	0.777187	0.510416	0	0	0.502379
TMPRSS6	0	0.137238	0	0	0	0	0	0
TMPRSS7	0	1.129269	0	1.006131	0	0.146606	0	0.238127
TMPRSS9	0	0	0	0.096183	0	0.195689	0	0
TMSB10	5104.68165	6315.4903	4806.40448	7973.76744	2403.52911	3420.76364	2014.01864	2675.27773
TMSB10P1	0	0	0	3.437355	0	0	0	0
TMSB10P2	0	0	0	0	0	0	0	0
TMSB15A	0	1.09739	0	1.154267	1.825671	0	0	0
TMSB15B	33.630629	10.572688	1.547633	7.26821	1.01712	4.4622	0	4.455957
TMSB15C	1.499209	0.827373	2.680697	1.272114	0	0.520562	5.451733	0.688371
TMSB4X	1365.01971	1529.8753	1042.53146	1134.31153	2868.33918	3178.1661	2887.29459	2897.78855
TMSB4XP1	0	0	0	0	0	0	0	0
TMSB4XP2	0	0	0	0	0	0	0	0
TMSB4XP3	0	0	0	0	0	0	0	0
TMSB4XP4	0	0.577202	0	0	0	1.695594	0	0.603876
TMSB4XP6	0	0	0	13.301615	0	0	0	0

TMSB4XP8	0	0	0	3.322707	31.180575	0	0	20.776086
TMSB4Y	0	1.793622	0	0.489309	5.03382	1.644415	0	3.647876
TMT1A	0	0.229345	1.925918	0.288139	2.836221	3.261483	0	0.910481
TMT1AP1	0	0	0	0	0	0	0	0
TMT1B	2.783408	1.626952	0	2.754805	0	1.769704	0	1.580815
TMTC1	0.73119	0.394649	3.418182	0.456638	0	0.09298	0	0.101779
TMTC2	2.902739	8.002253	4.239503	8.476109	1.372064	5.827663	6.125012	6.358055
TMTC3	72.377096	19.679997	30.457036	9.705156	66.319432	16.947505	27.549191	28.467994
TMTC4	7.642452	1.560068	0	8.779292	2.220377	4.505584	0	2.471136
TMUB1	23.062995	30.437171	22.647971	47.016052	6.017949	50.040585	0	34.200589
TMUB2	21.10553	48.806255	33.23616	58.971	7.814468	38.557244	0	38.343279
TMX1	53.727958	64.313091	57.459244	56.036755	63.610422	43.999557	62.864114	58.8168
TMX1P1	0	0	0	0	0	0	0	0
TMX1P2	0	0	0	0	0	0	0	0
TMX2	47.509808	39.01718	50.011248	39.705815	121.107997	77.216309	107.932032	64.235147
TMX2P1	0	0	0	0	0	0.287779	0	0
TMX3	12.459899	23.140054	9.947336	12.753283	117.522455	4.205114	158.260784	14.244121
TMX4	10.649524	20.940728	11.009502	12.677699	9.989142	10.745785	0	17.255033
TNC	7.638289	15.333423	8.88397	15.302995	10.28045	21.778264	72.282897	13.636874
TNF	0	0	0	0	0	0.104056	0	0
TNFAIP1	40.022839	36.583764	40.071585	51.631918	49.849305	111.808938	9.280977	48.292166
TNFAIP2	27.036585	28.596713	31.470932	98.343047	46.111016	116.584422	10.670757	58.657993
TNFAIP3	48.992108	58.117495	55.635657	68.3811	44.88604	70.61878	63.093627	136.813359
TNFAIP6	0	0	0	0	0	0	0	0
TNFAIP8	77.932648	71.624741	25.127767	83.39033	21.738026	55.976235	15.800684	80.760048
TNFAIP8L1	3.470916	4.728695	2.335698	4.744129	4.582622	5.591569	0	4.426592
TNFAIP8L2	0	0	0	0	0	0	0	0
TNFAIP8L3	0	0.079514	1.679315	0.411091	0	0	0	0.608078
TNFRSF10A	10.104709	2.173622	0	5.446263	38.364505	8.406183	0	0
TNFRSF10B	36.358352	78.006342	68.968067	138.255693	41.091102	93.60283	52.841735	68.360252
TNFRSF10C	4.436811	0	0	0	2.190883	0	0	0
TNFRSF10D	8.464356	7.360044	5.060979	6.539366	0.248309	2.018495	0	2.418663
TNFRSF11A	0.395754	0.99222	0	0.870033	1.568902	2.850311	0	1.570682

TNFRSF11B	0	0	0	0.113073	8.627546	1.109169	0	1.602884
TNFRSF12A	243.732842	192.942468	164.19901	189.67127	66.793018	58.041715	172.376087	84.436975
TNFRSF13B	0	0	0	0	0	0	0	0
TNFRSF14	0	5.649721	1.921098	8.160333	25.561191	15.631885	0	8.553487
TNFRSF17	0	0	0	0	0	0	0	0
TNFRSF18	9.445019	1.739679	5.267457	3.222356	0	1.190409	0	1.354577
TNFRSF19	4.766757	4.531152	5.842274	2.543771	0	0.119154	0	0.255529
TNFRSF1A	0	75.758719	46.224579	113.7155	63.099234	54.453708	0	37.140178
TNFRSF1B	0	0.079316	0	0.904916	0	0.092572	0	0.037255
TNFRSF21	82.230902	150.636903	102.763624	202.841066	74.648523	121.473471	56.628189	101.413751
TNFRSF25	0	2.244688	5.267457	2.611072	2.727596	2.250774	0	2.174998
TNFRSF4	0	0	0	0	0	0	0	0
TNFRSF6B	0	0	0	0	0	0	0	0
TNFRSF8	1.44624	2.333034	0	1.969576	0	1.343519	0	0.31863
TNFRSF9	0	0	0	0.184546	0	0	0	0
TNFSF10	6.587571	19.579389	45.045309	32.942918	34.102917	36.089872	0.53026	31.277875
TNFSF11	0	0.72898	2.098398	0.645673	0	0	0	0
TNFSF12	26.519175	2.761819	3.894849	4.546454	1.14417	2.768489	5.725815	4.607697
TNFSF12-TN	0	0	0	0	0	0	0	0
TNFSF13	11.620807	6.260789	8.402703	5.759757	29.01889	3.78479	28.123143	6.027713
TNFSF13B	0	0	0	0.031575	2.654466	0.751659	0	0.72988
TNFSF14	0	0.334706	0.681488	0.129769	0	0	0	0
TNFSF15	2.955221	1.271264	0.442871	0.975867	4.034615	2.174213	2.882002	3.633307
TNFSF4	0.971202	0.083031	0	0	0	0.048458	0	0
TNFSF8	0	0	0	0	0	0	0	0
TNFSF9	2.173148	1.828976	1.935489	2.737434	0.572454	1.497962	0	1.230328
TNIK	12.787514	19.927493	9.60537	20.972766	9.275731	11.149561	11.993255	7.863553
TNIP1	90.815487	213.882198	145.674659	278.95498	120.624409	332.022119	106.307804	215.160043
TNIP2	12.352161	26.577272	4.403114	13.888247	67.205996	25.072117	0	21.979383
TNIP2P1	0	0	0	0	0	0	0	0
TNIP3	5.853338	0	0	1.14619	0	0.141391	0	0.114729
TNK1	5.550358	3.754945	4.973186	5.112832	2.343938	8.590097	5.933425	7.069039
TNK2	7.024428	1.781588	0	1.584829	0	1.315472	5.509406	1.730783

TNKS	8.337458	4.614076	3.240749	2.988997	8.279892	3.340915	3.259667	2.952797
TNKS1BP1	49.344052	41.354692	41.466934	39.534654	80.796621	151.141914	50.353131	64.062421
TNKS2	29.654788	35.203133	31.337518	20.39729	25.706385	14.818842	22.447581	35.803517
TNMD	0	0	0	0	0	0	0	0
TNN	0	0	0	0	0	0	0	0
TNNC1	0	1.100481	0	0	0	0	0	0
TNNC2	0	0.427528	0	0.449656	0	0.894902	0	2.617707
TNNI1	0	0	0	0	0	0.026667	0	0.021327
TNNI2	0	0	0	0	0	0	0	0
TNNI3	9.208572	19.340233	5.643883	24.956672	0	0	0	0
TNNI3K	0	0.994724	0	0.195452	0	0	0	0
TNNT1	35.596329	162.387668	99.491896	190.344783	3.067964	55.943666	0	21.991622
TNNT2	0	0	0	0	0	0	0	0.881146
TNNT3	0	0.489626	0	0.181129	0	0	0	0
TNP1	0	0	0	0	0	0	0	0
TNP2	0	0	0	0	0	0	0	0
TNPO1	94.138705	122.933482	127.749539	83.737364	89.178754	93.631076	383.278455	143.814631
TNPO1P1	0	0	0	0	0	0	0	0
TNPO1P2	0	0.270696	0	0	0	0.063168	0	0
TNPO1P3	0	0	0	0	0	0	0	0
TNPO2	17.796509	48.306562	25.407689	41.905354	44.853299	69.065501	147.546133	72.076739
TNPO3	4.091645	23.294475	2.401797	39.501445	20.842322	44.94891	0	38.059271
TNPO3P1	0	0	0	0	0	0	0	0
TNPO3P2	0	0	0	0	0	0	0	0
TNR	0	0	0	0	0	0	0	0
TNRC18	102.987798	278.885965	77.791201	359.069094	124.507536	382.261389	141.827989	208.644597
TNRC18P1	0	0.116702	0	0	0	0	0	0
TNRC18P2	0	0	0	0	0	0	0	0
TNRC18P3	0	0	0	0	0	0	0	0
TNRC6A	27.765111	45.272644	27.136954	36.708487	31.26872	27.421821	16.51951	30.143261
TNRC6B	6.925626	12.140357	13.472742	19.49895	36.75739	23.061934	20.480582	21.862414
TNRC6C	0	2.039522	0.820533	7.875981	7.588316	8.399653	0	9.843244
TNS1	0.767194	7.421436	0	1.322091	9.465691	3.510621	10.251068	7.409143

TNS2	0	1.937222	6.319937	1.807537	17.197129	6.461695	0	3.230915
TNS3	20.015293	19.662766	14.939361	21.987673	28.585988	78.68846	11.83905	64.769546
TNS4	21.802107	24.419751	12.320937	19.569192	71.027188	75.031516	97.073619	83.598231
TNXA	0	0	0	0	0	0	0	0
TNXB	0	0.697742	1.103119	0.893482	0	6.107866	2.617363	0.225729
TOB1	42.904843	122.677473	39.770392	120.188447	55.635539	159.67011	64.582893	195.454253
TOB2	24.284211	55.666505	27.934314	57.670777	32.269139	76.261644	17.735219	70.33502
TOB2P1	0	0.962917	0	3.740877	0	0.565189	0	0
TOE1	4.753095	9.167755	8.255121	8.625567	4.877205	10.578859	41.390756	14.815587
TOGARAM1	27.783138	13.766184	44.560546	14.964449	13.508857	7.047139	34.030455	18.191538
TOGARAM2	0	0	0	0.043764	0	0.144572	0	0
TOLLIP	37.073072	37.809683	48.250102	29.554678	29.485718	57.641586	6.1398	46.72154
TOM1	3.109338	7.080661	2.778991	12.309655	5.330024	6.245596	0	10.025544
TOM1L1	50.383373	87.195031	66.298436	76.916239	59.50176	33.522064	91.587353	50.553459
TOM1L2	0	5.682182	0	8.756236	56.960811	19.839949	32.421536	15.311023
TOMM20	190.713165	93.7278	143.806184	123.813394	73.001509	73.399761	498.457788	90.857137
TOMM20L	7.437562	0	0	0.349913	0	0	0	0.618326
TOMM20P1	0	0	0	0	0	0	0	0
TOMM20P2	0	0	0	0	0	0	0	0
TOMM20P3	0	0	0	0	0	0	0	0
TOMM20P4	0	0	0	0	0	0	0	0
TOMM22	52.123396	46.575965	31.99504	39.129767	42.733279	26.366781	75.603883	33.533284
TOMM22P1	0	0	0	0	0	0	0	0
TOMM22P2	0	0	0	0	0	0	0	0
TOMM22P3	0	0	0	0	0	0	0	0
TOMM22P4	0	0	0	0	0	0	0	0
TOMM22P5	0	0	0	0	0	0	0	0
TOMM22P6	0	0	0	0	0	0	0	0
TOMM34	96.879637	99.560901	40.884093	84.260466	81.726774	106.286011	53.725725	93.086969
TOMM40	63.604418	92.551987	60.767383	73.34085	127.012097	171.587262	96.670819	146.124135
TOMM40L	8.996721	18.809332	11.504516	17.546262	6.782355	28.624517	11.40522	18.176327
TOMM40P1	0	0	0	0	0	0	0	0
TOMM40P2	0	0	0	0	0	0	0	0

TOMM40P3	0	0	0	0	0	0	0	0
TOMM40P4	0	0	0	0.093195	0	0	0	0.157794
TOMM5	223.20436	242.689386	188.346904	121.749983	233.46027	147.333483	154.029267	271.142732
TOMM5P1	0	0	0	0	0	0	0	0
TOMM6	9.277508	24.896728	30.268731	24.336735	22.940603	15.271661	156.942226	28.406945
TOMM7	96.744901	119.561936	85.52163	91.779036	40.783304	146.752453	0	1.535314
TOMM70	0	0.430413	0	1.978295	0	1.913304	28.900579	5.756448
TONSL	5.075119	5.037029	3.906279	3.020204	1.914974	7.251876	3.530632	3.834232
TOP1	107.420161	93.673276	68.495307	81.340437	116.503949	119.039427	145.989881	113.939579
TOP1MT	0	31.996752	35.058466	21.071148	25.153352	4.431058	47.246477	13.221179
TOP1P1	0	0	0	0.163959	0	0	0	0.579891
TOP2A	130.96385	79.214259	100.742837	53.787481	202.46905	83.794005	208.100409	123.446929
TOP2B	56.312746	56.046243	38.631463	46.402362	46.907737	47.965393	69.051575	72.668464
TOP3A	16.303066	40.626841	8.397534	25.252268	19.929294	36.606016	75.826637	46.512003
TOP3B	0	7.269096	19.144493	4.51853	11.384346	9.78419	16.55394	15.98849
TOP3BP1	0	0	0	0	0	0	0	0
TOP6BL	5.73648	11.250553	0	15.028103	28.399255	15.035096	1.837036	8.367982
TOPAZ1	0	0	0	0	0	0	0	0
TOPBP1	6.408584	27.948431	53.84957	40.899361	14.425067	28.702624	131.231476	39.600611
TOPORS	23.215922	12.36602	12.337547	10.081792	18.642266	12.336648	7.579237	22.372582
TOPORSLP	0	0	0	0	0	0	0	0
TOR1A	30.423649	54.793777	22.268653	44.380685	34.246741	35.326941	75.304718	47.467673
TOR1AIP1	60.707359	84.976993	88.079387	124.614375	40.619013	62.76499	116.32389	84.52533
TOR1AIP2	54.890259	258.877029	96.718132	196.406621	83.548889	124.118041	56.838702	396.761746
TOR1B	17.274944	28.731152	23.201521	37.277399	22.1413	35.707538	47.627915	33.114037
TOR1BP1	0	0	0	0	0	0	0	0
TOR2A	4.868394	9.236942	8.541416	10.615274	5.005832	9.684322	28.957421	8.753679
TOR3A	33.320021	101.425947	28.947893	76.441674	26.539615	70.012838	13.633487	77.051596
TOR4A	7.114272	11.958911	9.2243	12.218232	5.636556	10.907897	0.384797	8.193323
TOX	7.39361	11.100331	5.087598	7.379195	4.276976	3.197629	4.138531	3.544745
TOX2	4.1326	8.700327	12.860002	10.659155	6.100244	2.035773	7.719809	2.129481
TOX3	0	0	0	0	0	0	0	0
TOX4	45.235804	68.015035	32.370501	112.380142	135.483152	107.589612	49.70646	90.697571

TOX4P1	0	0	0	0	0	0	0	0
TP53	54.287574	5.021295	0	5.966131	0	6.65265	35.633045	3.35703
TP53AIP1	0	0	0	0.168973	0	0.311084	0	0.09423
TP53BP1	35.01385	47.826459	32.306195	53.027623	33.58435	45.574308	75.893813	36.442264
TP53BP2	16.29678	69.39374	16.11334	73.787154	30.162536	76.174659	4.658567	101.168766
TP53I11	6.549695	7.925278	8.838953	9.836415	4.944693	21.293979	0	5.815299
TP53I13	2.444495	19.216293	17.390671	16.228267	1.287221	25.908919	12.379114	10.952447
TP53I3	19.544853	19.324024	7.736686	18.768269	68.646067	29.362006	15.14894	30.046093
TP53INP1	2.325554	1.939253	1.567496	1.556839	1.075214	1.948724	1.133399	4.426155
TP53INP2	9.864193	5.410653	2.950968	5.558296	6.078201	5.194476	7.001402	4.108147
TP53RK	23.521341	16.703986	3.771333	12.26397	14.86808	14.428693	16.278116	19.722558
TP53TG3	0	0	0	0	0	0	0	0
TP53TG3B	0	0	0	0	0	0	0	0
TP53TG3C	0	0	0	0	0	0	0	0
TP53TG3D	0	0	0	0	0	0	0	0
TP53TG3E	0	0	0	0	0	0	0	0
TP53TG3GP	0	0	0	0	0	0	0	0
TP53TG3HP	0	0	0	0	0	0	0	0
TP53TG5	0	0	0	0	0	0	0	0
TP63	6.623373	99.325746	70.420551	164.152056	119.333011	205.994557	51.178747	95.399433
TP73	5.367731	7.375893	4.287249	7.746534	7.219433	12.11746	10.080907	5.277794
TPBG	33.886773	58.374148	41.623495	72.040304	25.838492	52.87153	21.853037	59.478958
TPCN1	9.475907	5.470785	18.162422	20.848964	38.373724	28.025034	40.841958	20.132536
TPCN2	0	3.844087	5.301766	4.194836	2.745015	3.655853	6.301773	3.235761
TPD52	30.193698	173.957214	120.146545	225.049083	58.925395	209.369696	18.68306	228.488069
TPD52L1	42.520474	257.447981	47.348905	204.761951	112.913576	190.378713	35.238523	241.170993
TPD52L2	37.491195	50.506083	8.247388	55.72781	4.549547	75.147965	34.33058	60.50809
TPD52L3	0	0	0	0	0	0	0	0
TPGS1	6.775427	3.018795	8.990087	2.281886	0.8904	4.417432	18.690997	1.922534
TPGS2	94.182317	77.657683	78.942448	69.109802	129.679662	77.311359	119.111482	128.814885
TPH1	0	0	0	0	0	0	0	0.134336
TPH2	0	0	0	0	0	0	0	0.433594
TPI1	934.185565	965.109111	726.461111	1265.29677	1017.95305	1308.66683	761.967023	1132.02745

TPI1P1	0	1.082223	0	0	0	0.289904	2.842144	0.785878
TPI1P2	0	0	0	0	0	0	1.314339	0
TPI1P3	0	0	0	0	0	0	0	0
TPI1P4	0	0	0	0	0	0	0	0
TPK1	0	5.8299	0	1.520784	0	0.957028	0	1.723301
TPM1	124.07452	267.140081	125.873127	241.560787	182.397489	371.407005	163.440114	328.72374
TPM2	2.672487	0.524713	3.334191	2.493893	0.992129	1.540986	0	0.462109
TPM2P1	0	0	0	0	0	0	0	0
TPM3	411.366156	541.631326	351.951993	571.599553	352.450499	592.440211	513.446824	456.526174
TPM3P1	0	0	0	0	0	0	0	0
TPM3P2	0	0	0	0	0	0	0	0
TPM3P3	0	0	0	0	0	0	0	0
TPM3P4	0	0	0	0	0	0	0	0
TPM3P5	0	0.23309	0	0.135109	0	0.275064	0	0.936176
TPM3P6	0	0.258644	4.9296	0.666442	0	2.139726	0	1.99694
TPM3P7	0	0	10.033224	0.409211	18.042853	0	0	0.710195
TPM3P8	0	0	0	0	0	0	0	0
TPM3P9	5.759277	34.446973	4.127325	40.324709	9.423912	54.60375	0	55.091404
TPM4	238.230796	417.70702	289.641012	438.811166	477.465315	784.979451	514.656964	823.368015
TPM4P1	0	0	0	0	0	0	0	0
TPM4P2	5.955217	29.339699	10.816949	22.215645	0	8.12805	0	8.550192
TPM4P3	0	0.251385	0	0	0	0	0	0
TPMT	10.505058	23.069823	11.299006	15.009246	17.469964	18.800466	4.595665	32.56681
TPMTP1	0	0	0	0.405642	0	0	0	0
TPMTP2	0	0	0	0	0	0	0	0
TPMTP3	0	0	0	0	0	0	0	0
TPMTP4	0	0	0	0	0	0	0	0
TPO	0	0	0	0	0	0	0	0
TPP1	34.597507	22.239544	46.137615	30.660391	50.436673	28.786068	75.971953	12.921985
TPP2	28.462033	23.049562	28.512942	18.37437	14.086982	8.813247	36.831833	16.019222
TPPP	1.087738	4.185027	3.911073	4.628193	1.293295	4.471668	0	2.181031
TPPP2	0	0	0	0	0	0	0	0
TPPP3	0	3.103883	1.809776	4.336926	12.851796	1.457099	0	0.920842

TPR	49.828875	51.352622	46.846744	56.454058	66.636111	39.933345	115.988372	56.464181
TPRA1	24.369661	6.37658	20.742068	27.019072	42.290811	30.831745	65.952951	20.425833
TPRG1	0	0.180933	7.560517	0.25399	3.703707	5.673271	3.333913	3.260266
TPRG1L	8.528735	17.45696	22.885099	13.556612	31.12192	24.567545	68.95002	24.278281
TPRG1LP1	0	0	0	0.129985	0	0	0	0.224911
TPRKB	72.676132	57.041383	20.258078	38.171822	36.690002	25.525961	54.510678	74.480227
TPRKBP1	0	0	0	0	0	0	0	0
TPRKBP2	0	0	0	0	0	0	0	0
TPRN	3.274058	10.768506	3.419518	11.05151	4.74822	40.576248	11.10349	8.804355
TPRX1P1	0	0	0	0	0	0	0	0
TPRXL	0	1.147096	0	1.049011	0	0	0	0.467641
TPSAB1	0	0	0	0	0	0	0	0
TPSB2	0	0	0	0.309354	0	0	0	0
TPSD1	0	0	0	0	0	0	0	0
TPSG1	0	0	0	0	0	0	0	0
TPST1	25.445869	38.349397	19.402603	56.310691	125.383855	37.443933	8.13752	38.476743
TPST2	3.532767	16.051318	3.160577	13.38999	0.931307	15.700449	0	12.895439
TPST2P1	0	0	0	0	0	0	0	0
TPT1	3112.7743	862.750757	3323.86942	608.888483	2259.23732	539.081963	1863.28796	1136.0414
TPT1P1	0	0	0	0	0	0	0	0
TPT1P10	0	0	0	0	0	0	0	0.84649
TPT1P11	0	0	0	0	0	0	0	0
TPT1P12	0	0	0	0	0	0	0	0
TPT1P13	0	0	0	0	0	0	0	0
TPT1P14	0	0	0	0	0	0	0	0
TPT1P15	0	0	0	0	0	0	0	0
TPT1P2	0	0	0	0	0	0	0	0
TPT1P3	0	0	0	0	0	0	0	0
TPT1P4	0	0	0	0.218319	0	0	0	0
TPT1P5	0	0	0	0	0	0	0	0.781932
TPT1P6	0	0	0	0	0	0	0	0
TPT1P7	0	0	0	0	0	0	0	0
TPT1P9	0	0.367376	0	0	0	0	0	0

TPTE	0	0	0	0	0	0	0	0
TPTE2	0	0	0	0	0	0	0	0
TPTE2P1	0	0	0	0	0	0	0	0
TPTE2P2	0	0	0	0	0	0	0	0
TPTE2P3	0	0	0	0	0	0	0	0
TPTE2P4	0	0	0	0	0	0	0	0
TPTE2P5	0	0	0	0.137372	0	0	0	0
TPTE2P6	0	0	0	0	0	0	0	0
TPTE2P7	0	0	0	0	0	0	0	0
TPTEP1	0	0	0	0	0	0	0	0.422988
TPTEP2	0	0	0	0	0	0	0	0
TPTEP2-CSN	0	6.095596	0	3.241241	0	0	0	0
TPX2	194.516759	388.657411	151.238302	340.215103	176.749136	346.196105	155.879971	425.785474
TRA2A	106.236705	71.245116	81.937415	79.071094	60.87452	67.678638	186.688603	57.707942
TRA2B	177.623437	263.603985	165.699161	214.668426	200.748317	285.588736	146.650178	267.249042
TRABD	9.510971	12.75287	13.137075	12.84583	0	25.982303	0	22.120115
TRABD2A	25.422841	51.111669	22.874516	42.813786	6.828202	13.528696	0.73378	13.875956
TRABD2B	0	0.569939	0	0.021014	0	1.327463	0	0.292558
TRAC	0	0	0	0.190265	0	0	0	0
TRADD	7.277049	18.972285	19.415997	20.573234	1.277359	20.481934	0	14.634953
TRAF1	0.892546	1.574223	2.048996	2.055798	0	1.683657	0	0.583647
TRAF2	18.163288	21.393003	21.649778	26.572994	19.161758	48.599597	25.4458	34.15837
TRAF3	5.521974	10.16921	35.691374	5.380848	11.841659	1.552629	44.772074	10.644095
TRAF3IP1	8.252444	36.13247	11.109388	38.900327	10.898947	57.410981	2.409864	26.343992
TRAF3IP2	8.178913	17.725019	0.690703	30.082723	16.638403	47.864578	60.39711	20.762986
TRAF3IP3	0	0	0	0	0	0.064816	0	0
TRAF4	23.258097	69.28576	27.083265	76.070177	32.84721	134.800483	9.320961	110.668021
TRAF5	5.889853	2.601017	6.398657	4.118662	10.708607	2.539903	0	6.701628
TRAF6	3.683416	14.227738	5.890778	16.615759	1.514276	26.790763	67.703171	41.865067
TRAF6P1	0	0	0	0	0	0	0	0
TRAF7	45.03541	70.838661	87.175638	86.486035	36.149403	112.745758	113.681506	84.060514
TRAFD1	34.744139	39.912447	20.733306	29.251507	63.492505	61.709381	76.057087	63.438685
TRAIP	17.13567	20.823599	10.146062	17.286795	9.775359	24.597656	117.256908	19.348598

TRAJ1	0	0	0	0	0	0	0	0
TRAJ10	0	0	0	0	0	0	0	0
TRAJ11	0	0	0	0	0	0	0	0
TRAJ12	0	0	0	0	0	0	0	0
TRAJ13	0	0	0	0	0	0	0	0
TRAJ14	0	0	0	0	0	0	0	0
TRAJ16	0	0	0	0	0	0	0	0
TRAJ17	0	0	0	0	0	0	0	0
TRAJ18	0	0	0	0	0	0	0	0
TRAJ19	0	0	0	0	0	0	0	0
TRAJ2	0	0	0	0	0	0	0	0
TRAJ20	0	0	0	0	0	0	0	0
TRAJ21	0	0	0	0	0	0	0	0
TRAJ22	0	0	0	0	0	0	0	0
TRAJ23	0	0	0	0	0	0	0	0
TRAJ24	0	0	0	0	0	0	0	0
TRAJ25	0	0	0	0	0	0	0	0
TRAJ26	0	0	0	0	0	0	0	0
TRAJ27	0	0	0	0	0	0	0	0
TRAJ28	0	0	0	0	0	0	0	0
TRAJ29	0	0	0	0	0	0	0	0
TRAJ3	0	0	0	0	0	0	0	0
TRAJ30	0	0	0	0	0	0	0	0
TRAJ31	0	0	0	0	0	0	0	0
TRAJ32	0	0	0	0	0	0	0	0
TRAJ33	0	0	0	0	0	0	0	0
TRAJ34	0	0	0	0	0	0	0	0
TRAJ35	0	0	0	0	0	0	0	0
TRAJ36	0	0	0	0	0	0	0	0
TRAJ37	0	0	0	0	0	0	0	0
TRAJ38	0	0	0	0	0	0	0	0
TRAJ39	0	0	0	0	0	0	0	0
TRAJ4	0	0	0	0	0	0	0	0

TRAJ40	0	0	0	0	0	0	0	0
TRAJ41	0	0	0	0	0	0	0	0
TRAJ42	0	0	0	0	0	0	0	0
TRAJ43	0	0	0	0	0	0	0	0
TRAJ44	0	0	0	0	0	0	0	0
TRAJ45	0	0	0	0	0	0	0	0
TRAJ46	0	0	0	0	0	0	0	0
TRAJ47	0	0	0	0	0	0	0	0
TRAJ48	0	0	0	0	0	0	0	0
TRAJ49	0	0	0	0	0	0	0	0
TRAJ5	0	0	0	0	0	0	0	0
TRAJ50	0	0	0	0	0	0	0	0
TRAJ51	0	0	0	0	0	0	0	0
TRAJ52	0	0	0	0	0	0	0	0
TRAJ53	0	0	0	0	0	0	0	0
TRAJ54	0	0	0	0	0	0	0	0
TRAJ55	0	0	0	0	0	0	0	0
TRAJ56	0	0	0	0	0	0	0	0
TRAJ57	0	0	0	0	0	0	0	0
TRAJ58	0	0	0	0	0	0	0	0
TRAJ59	0	0	0	0	0	0	0	0
TRAJ6	0	0	0	0	0	0	0	0
TRAJ60	0	0	0	0	0	0	0	0
TRAJ61	0	0	0	0	0	0	0	0
TRAJ7	0	0	0	0	0	0	0	0
TRAJ8	0	0	0	0	0	0	0	0
TRAJ9	0	0	0	0	0	0	0	0
TRAK1	31.941122	72.374762	22.957907	81.098577	16.469757	59.345415	11.519956	57.211128
TRAK2	5.07935	16.17633	5.936791	8.891786	20.822268	20.463802	9.039357	35.308784
TRAM1	189.23647	206.947017	306.485404	49.459422	87.014561	44.96269	51.249853	101.382513
TRAM1L1	3.427134	1.74098	1.530135	0.832398	0	0.593584	0	0.691311
TRAM2	6.888583	6.446621	7.847169	7.05713	14.684276	20.348952	21.053413	20.285787
TRANK1	0.918273	1.935607	3.103309	2.656051	4.935511	6.387255	0	5.482919

TRAP1	117.239994	166.702561	110.511303	216.115647	233.934362	215.884513	188.390836	209.890812
TRAPPC1	21.444252	0	25.140431	16.081854	55.539904	26.549464	71.890569	8.583812
TRAPPC10	34.752965	23.731902	22.907697	26.712111	19.33524	47.499671	37.604745	34.240891
TRAPPC11	18.174381	20.820237	24.132944	25.880366	58.991925	13.881774	22.73691	23.33859
TRAPPC12	69.468175	28.599933	22.094422	30.033978	27.154086	36.009438	69.417492	29.492299
TRAPPC13	27.452941	14.157409	25.453039	7.248235	18.309992	3.072158	34.606645	10.787019
TRAPPC13P1	0	0	0	0	0	0	0	0
TRAPPC14	5.316584	3.614149	12.360872	3.818913	1.753363	3.903161	11.510654	4.17411
TRAPPC2	9.677993	1.119727	7.800553	1.281264	7.882308	0.506074	0	1.263427
TRAPPC2B	0	0.464631	0	0.300033	0	1.089131	0	0.787378
TRAPPC2L	70.366755	97.680966	92.702169	70.617855	183.084331	84.16296	99.202166	116.440334
TRAPPC2LP1	0	0	0	0	0	0	0	0
TRAPPC2P10	0	0	0	0	0	0	0	0
TRAPPC2P2	0	0	0	0	0	0	0	0
TRAPPC2P3	0	0	0	0	0	0	0	0
TRAPPC2P4	0	0	0	0	0	0	0	0
TRAPPC2P5	0	0	0	0	0	0	0	0
TRAPPC2P7	0	0	0	0	0	0	0	0
TRAPPC2P8	0	0	0	0	0	0	0	0
TRAPPC2P9	0	0	0	0	0	0	0	0
TRAPPC3	70.16377	72.475303	30.548928	64.116221	40.628352	97.408314	98.763017	124.173998
TRAPPC3L	0	0	0	0	0	0	0	0
TRAPPC4	254.621734	213.608782	298.705596	238.579818	50.281145	37.843116	50.877396	47.733444
TRAPPC5	0	75.17444	23.702691	96.142423	0	91.104109	3.85749	97.742751
TRAPPC6A	6.16222	6.482938	28.779125	13.609826	0	5.566247	0	2.447906
TRAPPC6B	22.606322	62.241365	34.849548	58.346759	25.371436	44.884922	56.801193	52.618545
TRAPPC8	21.31712	22.367313	9.581323	12.21292	26.555082	13.809211	44.811502	39.678302
TRAPPC9	0	8.715792	9.23401	4.09451	5.051504	0.484363	0	0.938171
TRARG1	0	0	0	0	0	0	0	0
TRAT1	0	0	0	0	0	0	0	0
TRAV1-1	0	0	0	0	0	0	0	0
TRAV1-2	0	0	0	0	0	0	0	0
TRAV10	0	0	0	0	0	0	0	0

TRAV11	0	0	0	0	0	0	0	0
TRAV12-1	0	0	0	0	0	0	0	0
TRAV12-2	0	0	0	0	0	0	0	0
TRAV12-3	0	0	0	0	0	0	0	0
TRAV13-1	0	0	0	0	0	0	0	0
TRAV13-2	0	0	0	0	0	0	0	0
TRAV14DV4	0	0	0	0	0	0	0	0
TRAV15	0	0	0	0	0	0	0	0
TRAV16	0	0	0	0	0	0	0	0
TRAV17	0	0	0	0	0	0	0	0
TRAV18	0	0	0	0	0	0	0	0
TRAV19	0	0	0	0	0	0	0	0
TRAV2	0	0	0	0	0	0	0	0
TRAV20	0	0	0	0	0	0	0	0
TRAV21	0	0	0	0	0	0	0	0
TRAV22	0	0	0	0	0	0	0	0
TRAV23DV6	0	0	0	0	0	0	0	0
TRAV24	0	0	0	0	0	0	0	0
TRAV25	0	0	0	0	0	0	0	0
TRAV26-1	0	0	0	0	0	0	0	0
TRAV26-2	0	0	0	0	0	0	0	0
TRAV27	0	0	0	0	0	0	0	0
TRAV28	0	0	0	0	0	0	0	0
TRAV29DV5	0	0	0	0	0	0	0	0
TRAV3	0	0	0	0	0	0	0	0
TRAV30	0	0	0	0	0	0	0	0
TRAV31	0	0	0	0	0	0	0	0
TRAV32	0	0	0	0	0	0	0	0
TRAV33	0	0	0	0	0	0	0	0
TRAV34	0	0	0	0	0	0	0	0
TRAV35	0	0	0	0	0	0	0	0
TRAV36DV7	0	0	0	0	0	0	0	0
TRAV37	0	0	0	0	0	0	0	0

TRAV38-1	0	0	0	0	0	0	0	0
TRAV38-2DV	0	0	0	0	0	0	0	0
TRAV39	0	0	0	0	0	0	0	0
TRAV4	0	0	0	0	0	0	0	0
TRAV40	0	0	0	0	0	0	0	0
TRAV41	0	0	0	0	0	0	0	0
TRAV5	0	0	0	0	0	0	0	0
TRAV6	0	0	0	0	0	0	0	0
TRAV7	0	0	0	0	0	0	0	0
TRAV8-1	0	0	0	0	0	0	0	0
TRAV8-2	0	0	0	0	0	0	0	0
TRAV8-3	0	0	0	0	0	0	0	0
TRAV8-4	0	0	0	0	0	0	0	0
TRAV8-5	0	0	0	0	0	0	0	0
TRAV8-6	0	0	0	0	0	0	0	0
TRAV8-7	0	0	0	0	0	0	0	0
TRAV9-1	0	0	0	0	0	0	0	0
TRAV9-2	0	0	0	0	0	0	0	0
TRBC1	0	0	0	0	0	0	0	0.228482
TRBC2	11.031324	56.730269	4.820926	28.295423	0	15.976494	0	16.562624
TRBD1	0	0	0	0	0	0	0	0
TRBJ1-1	0	0	0	0	0	0	0	0
TRBJ1-2	0	0	0	0	0	0	0	0
TRBJ1-3	0	0	0	0	0	0	0	0
TRBJ1-4	0	0	0	0	0	0	0	0
TRBJ1-5	0	0	0	0	0	0	0	0
TRBJ1-6	0	0	0	0	0	0	0	0
TRBJ2-1	0	0	0	0	0	0	0	0
TRBJ2-2	0	0	0	0	0	0	0	0
TRBJ2-2P	0	0	0	0	0	0	0	0
TRBJ2-3	0	0	0	0	0	0	0	0
TRBJ2-4	0	0	0	0	0	0	0	0
TRBJ2-5	0	0	0	0	0	0	0	0

TRBJ2-6	0	0	0	0	0	0	0	0
TRBJ2-7	0	0	0	0	0	0	0	0
TRBV1	0	0	0	0	0	0	0	0
TRBV10-1	0	0	0	0	0	0	0	0
TRBV10-2	0	0	0	0	0	0	0	0
TRBV10-3	0	0	0	0	0	0	0	0
TRBV11-1	0	0	0	0	0	0	0	0
TRBV11-3	0	0	0	0	0	0	0	0
TRBV12-1	0	0	0	0	0	0	0	0
TRBV12-2	0	0	0	0	0	0	0	0
TRBV12-3	0	0	0	0	0	0	0	0
TRBV12-4	0	0	0	0.637506	0	0	0	1.193948
TRBV12-5	0	0	0	0	0	0	0	0
TRBV13	0	0	0	0	0	0	0	0
TRBV14	0	0	0	0	0	0	0	0
TRBV15	0	0	0	0	0	0	0	0
TRBV16	0	0	0	0	0	0	0	0
TRBV17	0	0	0	0	0	0	0	0
TRBV18	0	0	0	0	0	0	0	0
TRBV19	0	0	0	0	0	0	0	0
TRBV2	0	0	0	0	0	0	0	0
TRBV20-1	0	0	0	0	0	0	0	0
TRBV21-1	0	0	0	0	0	0	0	0
TRBV21OR9-	0	0	0	0	0	0	0	0
TRBV22-1	0	0	0	0	0	0	0	0
TRBV22OR9-	0	0	0	0	0	0	0	0
TRBV23-1	0	0	0	0	0	0	0	0
TRBV23OR9-	0	0	0	0	0	0	0	0
TRBV24-1	0	0	0	0	0	0	0	0
TRBV24OR9-	0	0	0	0	0	0	0	0
TRBV25-1	0	0	0	0	0	0	0	0
TRBV25OR9-	0	0	0	0	0	0	0	0
TRBV26	0	0	0	0	0	0	0	0

TRBV26OR9-	0	0.862433	0	0.568477	0	0	0	3.352091
TRBV27	0	0	0	0	0	0	0	0
TRBV28	0	0	0	0	0	0	0	0
TRBV29-1	0	0	0	0	0	0	0	0
TRBV29OR9-	0	0	0	0	0	0	0	0
TRBV3-1	0	0	0	0	0	0	0	0
TRBV30	0	0	0	1.093792	0	0	0	0
TRBV4-1	0	0	0	0	0	0	0	0
TRBV4-2	0	0	0	0	0	0	0	0
TRBV5-1	0	0	0	0	0	0	0	0
TRBV5-2	0	0	0	0	0	0	0	0
TRBV5-3	0	0	0	0	0	0	0	0
TRBV5-4	0	0	0	0	0	0	0	0
TRBV5-5	0	0	0	0	0	0	0	0
TRBV5-6	0	0	0	0	0	0	0	0
TRBV5-7	0	0	0	0	0	0	0	0
TRBV6-1	0	0	0	0	0	0	0	0
TRBV6-2	0	0	0	0	0	0	0	0
TRBV6-4	0	0	0	0	0	0	0	0
TRBV6-5	0	0	0	0	0	0	0	0
TRBV6-6	0	0	0	0	0	0	0	0
TRBV6-7	0	0	0	0	0	0	0	0
TRBV6-8	0	0	0	0	0	0	0	0
TRBV7-1	0	0	0	0	0	0	0	0
TRBV7-2	0	0	0	0	0	0	0	0
TRBV7-3	0	0	0	0.661443	0	0	0	0
TRBV7-4	0	0	0	0	0	0	0	0
TRBV7-5	0	0	0	0	0	0	0	0
TRBV7-6	0	0	0	0	0	0	0	0
TRBV7-7	0	0	0	0	0	0	0	0
TRBV7-9	0	0	0	0	0	0	0	0
TRBV8-1	0	0	0	0	0	0	0	0
TRBV8-2	0	0	0	0	0	0	0	2.63916

TRBV9	0	0	0	0	0	0	0	0
TRBVA	0	0	0	0	0	0	0	0
TRBVB	0	0	0	0	0	0	0	0
TRDC	0	0	0	0	0	0	0	0
TRDD1	0	0	0	0	0	0	0	0
TRDD2	0	0	0	0	0	0	0	0
TRDD3	0	0	0	0	0	0	0	0
TRDJ1	0	0	0	0	0	0	0	0
TRDJ2	0	0	0	0	0	0	0	0
TRDJ3	0	0	0	0	0	0	0	0
TRDJ4	0	0	0	0	0	0	0	0
TRDMT1	6.282712	3.079743	16.793948	0.850871	0	0.615872	0	0.754332
TRDN	0	0	0	0	0	0	0	0
TRDV1	0	0	0	0	0	0	0	0
TRDV2	0	0	0	0	0	0	0	0
TRDV3	0	0	0	0	0	0	0	0
TREH	0	0	0	0.208918	0	0	0	0
TREHP1	0	0	0	0	0	0	0	0
TREM1	0	0	0	0	0	0.153665	0	0
TREM2	0	0	0	0	0	0	0	0
TREML1	0	0	0	0	0	0	0	0
TREML2	0	0	0	0	0	0	0	0
TREML3P	0	0	0	0	0	0	0	0
TREML4	0	0	0	0	0	0	0	0
TREML5P	0	0	0	0	0	0	0	0
TRERF1	7.218564	20.280813	7.220468	25.034842	16.018849	39.248842	10.957456	30.531781
TREX1	0	0	0	0	6.623468	19.208538	0	11.288363
TREX2	0	0	0	0	0	0	0	0
TRGC1	0	0	0	0	0	0	0	0
TRGC2	0	0	0	0	0	0	0	0
TRGJ1	0	0	0	0	0	0	0	0
TRGJP	0	0	0	0	0	0	0	0
TRGJP1	0	0	0	0	0	0	0	0

TRGJP2	0	0	0	0	0	0	0	0
TRGV1	0	0	0	0	0	0	0	0
TRGV2	0	0	0	0	0	0	0	0
TRGV3	0	0	0	0	0	0	0	0
TRGV4	0	0	0	0	0	0	0	0
TRGV5	0	0	0	0	0	0	0	0
TRGV5P	0	0	0	0	0	0	0	0
TRGV6	0	0	0	0	0	0	0	0
TRGV7	0	0	0	0	0	0	0	0
TRGV8	0	0	0	0	0	0	0	0
TRGV9	0	0	0	0	0	0	0	0
TRGVA	0	0	0	0	0	0	0	0
TRH	0	0	0	0	0	0	0	0
TRHDE	10.876719	3.152685	0	4.034286	8.096927	1.116334	25.800865	1.816106
TRHR	0	0	0	0	0	0	0	0
TRIAP1	52.130314	65.976635	23.073407	40.392814	67.659532	47.239375	39.88884	115.114853
TRIAP1P1	0	0	0	0	0	0	0	0
TRIB1	37.87055	82.523931	44.786673	112.497372	27.749495	67.590352	26.508644	70.933508
TRIB2	1.2149	8.062874	6.588667	4.387184	14.892343	10.120604	6.489911	9.780809
TRIB3	0	0	0	0.094617	0	0	0	0
TRIM10	0	0	0	0	0	0	0	0
TRIM11	17.06024	31.219868	14.425975	36.716212	22.580188	49.446008	29.849683	29.125019
TRIM13	11.919887	27.321037	17.105418	16.989386	7.997688	10.79396	0	32.606384
TRIM14	34.202867	15.607118	15.151074	16.810482	18.214143	32.553942	26.078291	27.742727
TRIM15	0	0	0	0.565734	0	0	0	0
TRIM16	10.35798	34.930365	31.62823	49.339112	103.087926	91.33399	33.088836	81.609072
TRIM16L	22.057405	0	0	8.092221	0	0	0	0
TRIM17	0	2.900739	0	1.235005	8.588316	2.594812	0	0.202832
TRIM2	2.877871	1.483419	2.718149	0.804679	0	0.387146	0	1.15888
TRIM21	14.397327	13.545854	16.062356	16.034018	33.207877	29.020988	81.174716	20.979771
TRIM22	3.496455	14.805644	8.178701	10.505337	17.224842	45.160106	0	28.556741
TRIM23	9.534081	10.916953	15.408034	8.909472	3.166924	5.512262	4.376953	7.51015
TRIM24	19.919421	11.808296	7.136599	4.144512	7.766884	5.686529	24.422902	8.090256

TRIM25	61.540308	70.557314	48.39185	82.307955	56.451714	133.512873	194.460111	71.529461
TRIM26	24.134287	72.84221	35.419021	70.662339	57.167764	143.648599	25.259547	129.687818
TRIM26BP	0	0	0	0	0	0	0	0
TRIM27	43.377192	87.426383	25.145932	91.588841	137.041767	132.160582	35.972777	103.260245
TRIM28	195.796214	350.83835	190.466094	385.189685	442.370425	849.596267	586.020977	800.642067
TRIM29	222.959544	207.07043	400.784401	360.220581	200.370748	259.356128	406.046719	129.233308
TRIM3	0	2.732917	7.290285	4.021362	2.022334	10.826777	11.965445	7.18188
TRIM31	5.68474	5.832016	48.756499	26.208722	0	0.445367	0	0.218162
TRIM32	17.988462	19.889053	17.75088	9.392627	0	9.092555	16.846197	14.254847
TRIM33	46.858615	34.746958	55.389132	39.525203	52.911502	23.455773	135.091113	32.859466
TRIM34	0	10.359296	19.845181	5.309342	4.331562	8.019122	28.112436	8.018679
TRIM35	11.033441	19.374272	9.196747	14.033937	10.823064	17.706236	80.123022	7.1269
TRIM36	3.260851	16.75191	2.194994	18.489421	6.02947	10.662446	24.108094	9.360157
TRIM37	45.183533	84.429168	31.060278	77.448476	255.167604	62.7932	76.89169	79.817831
TRIM38	4.437238	8.091856	6.450616	9.467831	7.307651	11.73162	5.247173	11.718154
TRIM39	5.708535	22.414107	6.314104	25.144109	11.333789	42.653329	38.456592	40.510699
TRIM39-RPP2	6.417151	0	0	0	0	0	0	0
TRIM4	6.075658	16.385342	32.149971	22.885513	6.0505	13.500921	37.822663	20.529279
TRIM40	0	0	0	0	0	0	0	0
TRIM41	8.195367	35.964979	20.15901	34.880331	7.939279	36.687214	15.631974	23.576471
TRIM42	0	0	0	0	0	0	0	0
TRIM43CP	0	0	0	0.08962	0	0	0	0
TRIM44	18.45341	81.12096	58.995521	80.765516	60.575174	170.210349	41.348313	110.146909
TRIM45	0.951845	16.128932	0	11.298343	3.227059	8.568885	0	7.37344
TRIM46	1.127544	1.485929	1.848622	0.780663	0	0.84242	0	0.866341
TRIM47	22.843426	32.190649	14.68856	24.188348	11.244413	47.161111	24.751578	34.169306
TRIM49	0	0	0	0	0	0	0	0
TRIM49B	0	0	0	0	0	0	0	0
TRIM49C	0	0	0	0	0	0	0	0
TRIM49D1	0	0	0	0	0	0	0	0
TRIM49D2	0	0	0	0	0	0	0	0
TRIM5	0	16.251377	26.213961	21.665901	2.038055	33.331492	25.045691	8.189108
TRIM50	0	0	0	0	0	0	0	0

TRIM51	0	0	0	0	0	0	0	0
TRIM51BP	0	0	0	0	0	0	0	0
TRIM51CP	0	0	0	0	0	0	0	0
TRIM51DP	0	0	0	0	0	0	0	0
TRIM51EP	0	0	0	0	0	0	0	0
TRIM51FP	0	0	0	0	0	0	0	0
TRIM51HP	0	0	0	0	0	0	0	0
TRIM51JP	0	0	0	0	0	0	0	0
TRIM52	1.650047	5.186039	3.444524	7.548704	0	10.505368	11.488089	7.554206
TRIM53AP	0	0	0	0	0	0	0	0
TRIM53BP	0	0	0	0	0	0	0	0
TRIM53CP	0	0	0	0	0	0	0	0
TRIM54	0	0	0	0	0	0	0	0
TRIM55	0	0.79087	0	0.943399	0	0	0	0.644448
TRIM56	18.51248	26.272363	19.638575	41.356176	22.860815	48.483653	45.71656	34.060539
TRIM58	0	0	0	0	0	0	0	0
TRIM59	44.815162	23.390304	26.862022	15.286971	55.88985	22.646261	21.486128	57.973483
TRIM59-IFT8	0	11.233691	0	0	0	1.135401	0	6.863597
TRIM6	12.31514	29.941006	7.09897	19.406918	11.015487	4.764785	0	7.424084
TRIM6-TRIM3	3.289162	1.527973	0	3.6102	0	3.925886	0	1.541717
TRIM60	0	0	0	0	0	0	0	0
TRIM60P10Y	0	0	0	0	0	0	0	0
TRIM60P11Y	0	0	0	0	0	0	0	0
TRIM60P13	0	0	0	0	0	0	0	0
TRIM60P14	0	0	0	0	0	0	0	0
TRIM60P15	0	0	0	0	0	0	0	0
TRIM60P16	0	0	0	0	0	0	0	0
TRIM60P17	0	0	0	0	0	0	0	0
TRIM60P18	0	0	0	0	0	0	0	0
TRIM60P19	0	0	0	0	0	0	0	0
TRIM60P3Y	0	0	0	0	0	0	0	0
TRIM60P5Y	0	0	0	0	0	0	0	0
TRIM60P8Y	0	0	0	0	0	0	0	0

TRIM60P9Y	0	0	0	0	0	0	0	0
TRIM61	0	0	0	0	0	0	0	0
TRIM62	3.46996	3.007771	1.556706	3.747665	4.352294	6.702952	0	6.446403
TRIM63	0	0	0	0	0	0	0	0
TRIM64C	0	0	0	0	0	0	0	0
TRIM64DP	0	0	0	0	0	0	0	0
TRIM64EP	0	0	0	0	0	0	0	0
TRIM64FP	0	0	0	0	0	0	0	0
TRIM65	8.448416	12.720641	14.924321	14.721086	9.620129	19.77353	16.776678	17.050431
TRIM66	1.994457	8.929428	4.265756	4.246679	10.892544	4.409012	0	4.626562
TRIM67	0	0	0	0.016892	0	0	0	0
TRIM67-AS1	0	0	0	0	0	0	0	0
TRIM68	8.029392	21.074952	10.347138	21.680564	9.008702	40.801366	6.099927	31.120805
TRIM69	35.313598	30.420939	15.313367	26.189385	31.369193	33.249479	37.113592	37.060284
TRIM7	30.940209	50.42152	27.106648	42.588717	15.002657	48.831996	7.748362	46.24938
TRIM71	0	0.047818	0	0	0	0	0	0
TRIM72	0	0	0	0	0	0	0	0
TRIM73	0	0	0	0.126387	0	0.228317	0	0
TRIM74	0	0	0	0.584556	0	0	0	0.657467
TRIM77	0	0	0	0	0	0	0	0
TRIM77BP	0	0	0	0	0	0	0	0
TRIM8	15.974035	70.694922	44.589149	101.701407	43.235815	98.306877	87.829292	91.878738
TRIM80P	0	0.100856	0	0	0	0	0	0
TRIM9	2.305078	2.910369	3.083982	3.353745	0	0.444358	0	0.498086
TRIML1	0	0	0	0	0	0	0	0
TRIML2	12.994091	17.170512	5.786762	17.060473	9.465896	12.737264	0	15.475594
TRIO	96.615349	93.610948	105.651353	169.987422	41.535945	124.315663	136.833765	110.377348
TRIOBP	28.451397	73.010599	29.378134	71.515423	9.964682	73.320645	8.99711	45.236247
TRIP10	45.380309	94.009448	57.208975	93.299088	77.072285	117.106144	238.652821	75.187573
TRIP11	23.072462	56.358498	36.783388	42.15937	26.695704	42.14913	49.345597	66.750227
TRIP12	86.99804	209.322749	122.413142	327.771793	139.869491	453.531187	199.85106	437.405034
TRIP13	37.866994	90.011359	20.072924	56.037979	44.766442	67.750875	46.637979	120.822461
TRIP4	42.860493	10.485135	0	12.315665	0	4.543218	101.901864	7.142658

TRIP6	25.058437	28.212133	22.328705	32.098026	64.983659	80.792271	94.858704	53.496083
TRIQK	13.013312	49.317772	40.241653	33.351182	43.896306	8.451291	0	13.184725
TRIR	149.572376	136.337254	143.409457	189.893973	262.697553	296.45455	140.44828	206.767252
TRIT1	0.710085	9.41159	42.71633	8.461314	0	20.128691	150.30987	7.25058
TRMO	0	0	15.761365	14.561939	18.248391	15.456534	0	21.480542
TRMT1	7.563929	13.029812	30.267788	14.662994	48.229016	61.176449	110.741922	38.824608
TRMT10A	10.608434	5.392316	9.131068	8.093424	0	4.259157	0	3.304976
TRMT10B	8.656413	12.381641	8.015628	11.12776	14.604384	12.782151	2.539216	13.181641
TRMT10BP1	0	0	0	0	0	0	0	0
TRMT10C	65.770078	48.736375	53.48079	32.907991	57.606425	19.051393	63.622261	45.476407
TRMT11	5.698327	33.201345	13.554658	20.176421	60.319982	18.558819	64.728036	31.972019
TRMT112	193.789398	106.92232	101.427317	110.598444	170.61166	229.370405	151.576311	180.593954
TRMT112P1	0	0	0	0	0	0	0	0
TRMT112P2	0	0	0	0	0	0	0	0
TRMT112P3	0	0	0	0	0	0	0	0
TRMT112P4	0	0	0	0	0	0	0	0
TRMT112P5	0	0	0	0	0	0	0	0
TRMT112P6	0	0	0	0	0	0	0	0
TRMT112P7	0	0	0	0	0	0	0	0
TRMT112P8	0	0	0	0	0	0	0	0
TRMT12	14.019681	53.589592	11.137746	41.392434	51.959546	69.747734	22.273379	66.347279
TRMT13	0	1.175928	0	17.151903	0	2.574066	0	0.495587
TRMT1L	11.317825	33.88456	14.225971	20.880148	31.23326	5.01917	12.12321	30.152077
TRMT1P1	0	0	0	0	0	0	0	0
TRMT2A	26.867898	30.287597	36.841915	24.138894	19.271564	88.593257	0	63.574028
TRMT2B	10.785442	25.017372	17.445195	23.486805	13.095625	22.162069	26.287023	18.09529
TRMT44	5.262596	1.948943	0	4.582987	1.395448	2.448625	8.732019	3.361516
TRMT5	0	40.957643	48.627997	27.690669	0	14.275995	1.551667	30.680481
TRMT6	29.850066	45.955104	19.540945	35.460097	36.96633	54.729375	8.920514	72.692298
TRMT61A	2.38476	6.212847	5.339438	7.956661	3.017191	12.537018	3.474773	8.689112
TRMT61B	24.677877	39.096727	30.489324	30.491254	3.001841	13.832335	76.675836	23.542976
TRMT9B	0	1.750892	0	0.131877	0.143107	0	0	0.303601
TRMU	13.178679	48.666649	2.665105	45.260401	29.480895	48.82054	0	55.467965

TRNAU1AP	9.755894	42.942466	28.025559	37.252292	48.459032	43.304222	20.735352	48.285393
TRNP1	33.693464	28.901099	39.574376	20.498889	1.25713	8.21085	10.731119	13.438553
TRNT1	51.866686	46.232347	37.67103	51.348834	14.422446	14.944477	86.0786	21.354307
TRNT1P1	0	0	0	0	0	0	0	0
TRNT1P2	0	0	0	0	0	0	0	0
TRO	2.970162	4.241094	6.552775	9.63609	1.175101	3.418513	34.698692	4.950154
TROAP	25.151441	51.171866	31.466952	41.289064	16.618532	83.582174	5.324195	49.322527
TRPA1	0	0.243258	0	0.415757	0	0	0.291206	0
TRPC1	0	6.320412	5.467637	3.773247	0	5.648729	0	1.3558
TRPC3	0	0	0	0.03488	0	0	0	0
TRPC4	0	0	0	0.089395	0	0	0	0
TRPC4AP	52.91327	31.982167	67.343291	36.459645	38.510696	22.635468	39.926978	23.096936
TRPC5OS	0	0	0	0	0	0	0	0
TRPC6	1.391952	0.666719	1.884084	0.307518	0	0.063597	0	0
TRPC6P1	0	0	0	0	0	0	0	0
TRPC6P10	0	0	0	0.357426	0	0	0	0
TRPC6P2	0	0	0	0	1.193783	0	0	0
TRPC6P3	0	0	0	0	0	0	0	0
TRPC6P4	0	0	0	0	0	0	0	0
TRPC6P5	0	0	0	0	0	0	0	0
TRPC6P6	0	0	0	0	0	0	0	0
TRPC6P7	0	0	0	0	0	0	0	0
TRPC6P8	0	0	0	0	0	0	0	0
TRPC6P9	0	0	0	0	0	0	0	0
TRPC7	0	0	0	0	0	0	0	0
TRPM1	0	0	0	0	0	0	0	0
TRPM2	0.626329	0	0	0	0	0	0	0
TRPM3	0	0	0	0	0	0	0	0
TRPM4	0	3.283859	5.176232	4.691418	3.3116	2.630514	34.171652	3.659358
TRPM5	0	0.120656	0	0.022945	0	0	0	0
TRPM6	0	0.033688	0	0.704677	0.103349	0	0	0.015669
TRPM7	35.566173	35.127695	38.0353	20.360535	39.395115	11.96676	26.010363	32.246464
TRPM8	0	0.311182	0	0	0	0.553992	0	0

TRPS1	4.424606	4.045235	0.608036	5.186389	9.651141	21.419008	5.648132	17.267303
TRPT1	12.054791	18.073107	38.134137	18.806104	28.413174	40.517628	0	27.945823
TRPV1	0	0	0	0	2.129747	0.158653	0	0.563913
TRPV2	0	0.310484	0	1.222725	0	1.472232	0	0.981019
TRPV3	0	1.002499	1.163737	0.53818	0	0.625272	0	0.391079
TRPV4	0	0.994314	2.669367	0.950736	0.393735	0	0	3.678353
TRPV5	0	0	0	0	0	0	0	0
TRPV6	0	0	0	0.072	0	0	0	0
TRRAP	12.919004	17.099908	11.156587	12.607202	1.405323	21.164269	66.89071	12.604166
TRUB1	11.737604	37.681073	12.279064	21.501744	12.653348	19.618176	7.610315	20.010747
TRUB2	8.417357	27.977117	20.953053	34.213833	34.907748	38.240605	14.503092	28.190871
TSACC	0	0	0	2.873592	0	2.190084	0	0.521952
TSBP1	0	0	0	0	0	0	0	0
TSC1	35.858464	21.663151	15.108657	26.69003	12.469311	18.774579	13.112572	9.717588
TSC2	25.565242	17.230914	21.043877	15.921492	21.944239	19.716501	92.677922	24.075591
TSC22D1	20.846567	47.769667	18.227141	79.774528	6.206496	91.96603	45.847052	36.80535
TSC22D2	25.905676	107.01099	48.770268	90.372345	61.17549	38.15248	29.831129	92.713665
TSC22D3	13.549214	18.493397	24.287338	22.821715	0.901594	9.155509	16.875752	12.77965
TSC22D4	16.244048	54.902046	11.883791	60.400759	6.232066	94.841162	25.063155	66.166223
TSEN15	31.225061	2.449696	25.134558	11.711455	31.978944	3.392009	38.801445	7.087876
TSEN15P1	0	0	0	0	0	0	0	0
TSEN15P2	0	0	0	0	0	0	0	0
TSEN15P3	0	0	0	0	0	0	0	0
TSEN2	0	4.029929	10.258577	4.473785	0	0.060189	0	5.241961
TSEN2P1	0	0	0	0	0	0	0	0
TSEN34	37.962007	33.302976	38.659512	34.592421	70.194158	57.478767	40.422923	40.533118
TSEN54	17.976131	21.518141	21.433157	15.048297	3.587354	26.882099	0	21.78511
TSFM	88.263007	62.791535	35.682246	65.130446	96.019809	86.756413	57.158149	94.88797
TSG101	67.688129	100.392485	77.486412	76.106068	127.117856	103.918252	91.344157	121.789369
TSGA10	0	1.298555	3.008679	6.033161	0.360219	7.386112	0	5.230557
TSGA10IP	0	0	0	0.16292	0	0	0	0
TSGA13	0	0	0	0	0	0	0	0
TSHR	0	0	0	0	0	0	0	0

TSHZ1	0	0.937963	0	3.810405	9.132044	12.210295	0	11.554674
TSHZ2	6.461844	0.894074	5.632703	2.738378	0	0	0	0
TSHZ3	0	0	0	0	0	0	0	0
TSKS	0	0	0	0	0	0	0	0
TSKU	30.473125	59.940709	24.668755	64.130705	56.2144	86.360737	19.111782	63.654799
TSLP	4.245223	4.811083	6.328661	4.097075	0	0.491807	0	1.083022
TSN	78.188311	51.223584	40.191534	38.142915	50.618685	39.644642	131.807915	75.602874
TSNARE1	8.570597	7.413719	1.461671	18.499404	10.121158	15.354494	8.323335	4.551729
TSNAX	33.789993	34.497636	26.434807	22.845506	81.720126	11.210156	111.315098	31.939252
TSNAX-DISC	0	0	0	0.234994	0	0	0	0
TSNAXIP1	0	2.742942	0	1.533376	0	1.016459	4.17046	1.159622
TSPAN1	80.571689	64.931334	81.493113	71.105773	52.749154	58.42282	51.965492	45.124133
TSPAN10	1.840653	0.622336	1.642437	2.72185	7.762655	0.90959	0	0.371535
TSPAN11	0	0	0	0	0	0	0	0
TSPAN11-AS	0	1.31033	0	2.257733	0	0	0	0.610514
TSPAN12	3.205135	1.915978	0	1.044739	9.70325	1.447836	0	1.555604
TSPAN13	61.568509	154.343133	59.924229	86.795671	48.191335	47.468504	40.6233	83.617393
TSPAN14	38.416716	76.588213	31.968207	97.982019	42.636537	102.966051	16.534278	95.450006
TSPAN15	98.899614	87.729666	72.096273	97.245774	54.763716	26.21646	10.025706	31.043328
TSPAN16	0	0	0	0	0	0	0	0
TSPAN17	24.759909	45.289192	17.404478	33.931399	18.214735	59.886929	26.842159	49.448426
TSPAN18	0	0	0	0	0	0.116876	0	0
TSPAN19	0	0.622901	0	0.258966	0	0	0	0
TSPAN2	6.464156	0.792679	0.931706	2.962872	11.38609	2.720843	41.767772	1.170076
TSPAN3	132.699515	172.29984	155.782217	186.957051	101.978493	122.255894	109.140252	158.423298
TSPAN31	53.025765	57.672677	102.720516	50.625296	34.479463	19.778014	19.883827	32.567932
TSPAN32	0	0	0	0	0	0	0	0
TSPAN33	4.55581	2.652018	1.868064	4.001028	9.389974	3.624647	0	2.473925
TSPAN4	3.719527	19.02044	10.923891	15.199806	37.484533	43.90165	43.454752	29.988426
TSPAN5	57.71804	98.866791	28.901894	64.576093	14.730181	23.334402	39.837375	43.646962
TSPAN6	0	3.455706	0	1.783799	20.013589	0.257915	0	2.921728
TSPAN7	7.807387	1.028089	3.745976	0	0	0	0	0
TSPAN8	7.103445	0.41382	5.887853	0.800614	0	0.780677	0	0.270692

TSPAN9	16.516176	10.714891	4.830649	14.496743	1.624131	14.562791	0	11.014316
TSPEAR	0	0	0	0	0	0	0	0.033538
TSPO	53.141924	35.215444	73.290126	36.593078	61.320722	59.691977	27.850875	44.884706
TSPO2	0	0	0	0	0	0	0	0
TSPOAP1	0.431808	0.631773	0	0.909276	0.606171	4.443529	0	1.204442
TSPY1	0	0	0	0	0	0	0	0
TSPY10	0	0	0	0	0	0	0	0
TSPY11P	0	0	0	0	0	0	0	0
TSPY12P	0	0	0	0	0	0	0	0
TSPY13P	0	0	0	0	0	0	0	0
TSPY14P	0	0	0	0	0	0	0	0
TSPY15P	0	0	0	0	0	0	0	0
TSPY16P	0	0	0	0	0	0	0	0
TSPY17P	0	0	0	0	0	0	0	0
TSPY18P	0	0	0	0	0	0	0	0
TSPY2	0	0	0	0	0	0	0	0
TSPY20P	0	0	0	0	0	0	0	0
TSPY21P	0	0	0	0	0	0	0	0
TSPY22P	0	0	0	0	0	0	0	0
TSPY23P	0	0	0	0	0	0	0	0
TSPY24P	0	0	0	0	0	0	0	0
TSPY25P	0	0	0	0	0	0	0	0
TSPY3	0	0	0	0	0	0	0	0
TSPY4	0	0	0	0	0	0	0	0
TSPY5P	0	0	0	0	0	0	0	0
TSPY6P	0	0	0	0	0	0	0	0
TSPY7P	0	0	0	0	0	0	0	0
TSPY8	0	0	0	0	0	0	0	0
TSPYL1	0	0	0	0.042123	0.191005	0	0	0.137933
TSPYL2	14.373811	17.282804	26.845279	15.687135	15.172827	14.400588	28.584085	15.28863
TSPYL6	0	0	0	0	0	0	0	0
TSR1	0	17.085551	0	29.866973	0	78.410714	36.235276	29.840055
TSR2	21.053953	29.760626	22.530792	35.94866	28.441892	24.915862	38.626291	20.713437

TSR3	46.062888	74.770366	32.654933	71.856482	41.994769	109.347684	25.828044	82.023626
TSSC4	27.412059	30.939234	18.72124	34.724522	49.709429	71.627024	18.245157	65.632617
TSSK1A	0	0	0	0.25652	0	0	0	0
TSSK2	0	0.23262	0	0	0	0.408743	0	0
TSSK3	5.930403	0.951936	0	0	0	4.199102	0	0
TSSK4	0	0	0	0	0	0	0	0
TSSK5P	0	0	0	0	0	0	0	0
TSSK6	0	0.259611	3.989187	1.632267	0	0	0	0
TST	6.841607	22.23506	21.17784	18.762579	3.596168	17.647293	0	14.619063
TSTD1	68.635736	64.215591	106.174184	105.655713	52.554612	39.701436	0	29.645491
TSTD2	10.419897	13.60441	7.194405	10.625368	29.839567	5.920528	0	9.163549
TSTD3	1.660311	0.584362	0	0.568476	0	0.566009	0	1.306659
TTBK1	0	0.078117	0	0.033305	0	0	0	0.018188
TTBK2	15.153011	31.240444	17.75914	45.839009	74.23874	33.338956	34.420381	42.757571
TTC1	71.966087	91.628034	40.11076	78.37658	52.793126	64.687085	136.900688	80.434519
TTC12	9.370657	27.437651	16.885556	25.60942	9.805303	39.74035	8.544705	14.753093
TTC13	6.458006	4.444989	9.293725	2.971072	22.100809	1.759053	0	2.663388
TTC14	5.358925	10.381773	4.010833	3.265235	2.872537	5.696325	0	12.197851
TTC16	0	0.326842	0	0.142722	0	0.116649	0	0
TTC17	28.447908	18.542743	35.836446	30.654003	60.662672	27.347115	35.598767	21.377639
TTC19	23.942963	18.733633	17.858865	12.764762	49.99751	8.109419	19.382241	14.680352
TTC21A	0	2.776214	1.505577	1.074418	0	0.900465	0	2.258989
TTC21B	5.422536	4.606743	3.870987	6.309453	5.411929	7.010679	5.510035	7.34541
TTC22	17.604302	13.420401	12.29804	14.436631	3.103586	8.354782	0.23819	6.417881
TTC23	20.902377	69.356642	67.892391	58.038729	42.954794	58.77421	7.46749	73.078312
TTC23L	0	0	0	1.728159	0	0	0	0
TTC24	0	0	0	0	0	0	0	0
TTC27	32.387849	57.913814	20.604431	37.456067	29.220714	21.076649	53.681389	45.508405
TTC28	0.568409	2.479345	8.931849	2.03433	0.075128	0	0	0.401096
TTC29	0	0	0	0	0	0	0	0
TTC3	5.094446	76.138598	3.365676	78.555691	9.384144	60.92376	12.517449	52.022127
TTC31	7.211571	5.567841	0	12.229307	16.21858	11.752094	0	2.790336
TTC32	11.280131	21.876318	11.96298	16.807581	0	9.584444	0	7.902037

TTC33	1.779617	6.277917	19.463476	12.580524	21.535184	8.989655	1.589994	7.768164
TTC34	0.88702	0.151837	0	0	0	0.154889	0	0
TTC36	0	1.939517	0	0	0	0	0	0
TTC38	1.323009	6.149212	4.736003	6.4006	12.851052	6.673145	8.026278	8.710496
TTC39A	3.948802	4.444194	7.794441	6.477324	3.209154	6.131248	0	7.293864
TTC39B	4.355791	2.295246	2.735939	2.858162	0	1.931758	0	3.713158
TTC39C	11.637362	21.356546	13.166601	15.925837	30.504051	7.343637	0	15.686829
TTC39CP1	0	0	0	0	0	0	0	0
TTC39DP	0	0	0	0	0	0	0	0
TTC3P1	1.085444	2.827122	1.976516	1.128376	0	0.709165	6.254687	2.679201
TTC4	52.232416	65.249474	28.54531	64.338876	14.925658	21.96253	6.77055	22.972842
TTC41P	0	0	0	0.111106	0	0	0	0
TTC4P1	0	0	0	0	0	0	0	0
TTC5	8.306599	17.635314	13.010545	21.957888	16.575151	19.670961	7.080207	18.253884
TTC6	0	0.597646	0	0.398953	0	0.279014	0	0.336892
TTC7A	10.909917	30.960878	26.194678	24.363865	32.156256	58.473185	8.853406	35.713028
TTC7B	11.109427	36.056526	53.899678	23.012836	25.890545	35.65899	104.468848	34.155218
TTC8	21.03761	31.390237	0	33.603931	16.964754	22.213198	0.502208	26.886674
TTC9	0.641891	2.14953	1.730021	5.067039	0.508702	6.461183	0	4.609908
TTC9B	0	0.250258	0	0.1075	0	0	0	0
TTC9C	23.367469	34.101169	24.061772	40.016066	25.933764	48.236937	0	58.000606
TTF1	18.106015	20.782642	19.090996	18.748391	57.441821	16.993528	18.589941	19.532517
TTF2	21.454745	113.005045	56.190287	112.298947	84.735259	59.342337	84.608953	56.570898
TTI1	34.075954	73.486268	43.021484	81.862693	28.839554	52.763376	30.822041	77.724243
TTI2	9.187532	14.600526	17.29339	17.893947	14.467217	6.288115	64.985931	9.980601
TTK	21.523437	21.988287	13.193469	5.770722	90.264395	7.748778	54.152406	15.485383
TTL	55.153473	76.042431	17.189641	40.019533	67.537169	71.444802	41.798116	104.622317
TTLL1	0	5.192827	5.575961	9.964821	0	2.302857	0	2.051145
TTLL10	0	0	0	0	0	0.27894	0	0
TTLL11	3.759168	11.373923	20.326053	19.474999	14.989697	10.260123	0	3.402768
TTLL12	20.669091	45.599407	22.945597	44.78102	21.826678	60.759153	21.536945	45.638064
TTLL13	3.892719	0.317513	0	1.110933	0	1.14305	0	0
TTLL2	0	0	0	0	0	0	0	0

TTLL3	1.73785	3.660205	0	2.170479	0	2.357846	0	0.27085
TTLL4	13.435594	26.421448	30.391151	34.794722	63.460983	75.91452	51.484698	60.025186
TTLL5	45.93892	60.285069	26.365037	74.549723	36.042817	87.291824	46.124533	75.683945
TTLL6	0	0.152797	0	0.153452	0	1.275451	0	0.37573
TTLL7	21.123183	26.249954	20.234791	20.731187	0	1.868831	0	6.179868
TTLL8	0	0	0	0	0	0	0	0
TTLL9	0	0	0	1.338253	0	0	0	0
TTN	0	0	0	0	0	0	0	0
TTPA	0	0	0	0	0	0	0	0
TTPAL	32.874016	65.245396	31.899738	55.916798	30.20513	67.295684	58.885102	66.360729
TTR	0	0	0	0	0	0	0	0
TTTY11	0	0	0	0	0	0	0	0
TTTY14	0	0	0	0.252274	0	0.983807	0	1.383247
TTTY16	0	1.078595	0	0.525945	0	2.370539	0	4.966113
TTTY23B	0	0	0	0	0	0	0	0
TTTY25P	0	0	0	0	0	0	0	0
TTTY9A	0	0	0	0	0	0	0	0
TTYH1	0	3.586504	0	2.194454	0	0.570404	0	0
TTYH2	0.970006	0.331722	7.58063	0.87547	0	0.72599	0	1.383181
TTYH3	38.87578	37.18808	40.431342	34.303403	52.649814	83.345928	52.489322	56.68992
TUB	7.581152	8.033019	4.544127	8.58349	8.547032	29.593652	10.473109	20.460692
TUBA1A	9.589154	23.995829	24.789414	10.382855	6.706648	10.78194	0	9.229638
TUBA1B	806.265538	436.130224	547.002751	356.772199	1013.45598	514.177208	876.42398	586.532647
TUBA1C	453.095629	251.695712	287.164743	219.066349	379.297197	280.3656	434.542597	326.115016
TUBA3C	0	0	0	0	0	0	0	0
TUBA3D	0	0	0	0	0	0	0	0
TUBA3E	0	0	0	0	0	0	0	0
TUBA3GP	0	0	0	0	0	0	0	0
TUBA4A	175.101372	105.88935	101.54205	99.143263	350.308351	184.530436	216.86804	180.122769
TUBA4B	0	0	0	0	0	0	0	0
TUBA5P	0	0.694526	0	0.389759	0	0	0	0
TUBA8	0	0	0	0	0	0	0	0
TUBAL3	0	0	0	0.057712	0	0	0	0

TUBAP1	0	0	0	0	0	0	0	0
TUBAP10	0	0	0	0	0	0	0	0
TUBAP11	0	0	0	0	0	0.143116	0	0
TUBAP12	0	0	0	0	0	0	0	0
TUBAP13	0	0	0	0	0	0	0	0
TUBAP14	0	0	0	0	0	0	0	0
TUBAP15	0	0	0	0	0	0	0	0
TUBAP2	0	0	0	0.053853	0	0	0	0
TUBAP4	0	0	0	0	0	0	0	0
TUBAP6	0	0	0	0	0	0	0	0
TUBAP7	0	0	0	0	0	0	0	0
TUBAP8	0	0	0	0	0	0	0	0
TUBAP9	0	0	0	0	0	0	0	0
TUBB	481.797015	412.319785	356.862267	330.693963	774.180028	756.451711	833.166115	886.654709
TUBB1P1	0	0	0	0	0	0	0	0
TUBB1P2	0	0	0	0	0	0	0	0
TUBB2A	15.403192	14.71136	2.593336	9.453578	21.595229	24.368047	13.827915	25.251515
TUBB2B	0	23.953812	20.59569	32.309492	1.823851	10.914718	0	4.208529
TUBB2BP1	0	0	0	0	0	0	0	0
TUBB3	45.703148	44.425189	31.500691	26.369263	48.086621	60.247306	48.175215	67.092048
TUBB3P1	0	0	0	0	0	0	0	0
TUBB3P2	0	0	0	0	0	0	0	0
TUBB4A	7.52827	3.726716	2.692123	4.469276	0	0.449819	0	0.97085
TUBB4AP1	0	0	0	0	0	0	0	0
TUBB4B	426.191735	617.47415	366.824407	612.611285	550.819373	849.603802	628.479381	688.032558
TUBB4BP1	0	0	0	0	0	0	0	0
TUBB4BP2	0	0	0	0	0	0	0	0
TUBB4BP3	0	0	0	0	0	0	0	0
TUBB4BP4	0	0	0	0	0	0	0	0
TUBB4BP5	0	0	0	0	0	0	0	0
TUBB4BP6	0	0	0	0	0	0	0	0
TUBB4BP7	0	0	0	0	0	0	0	0
TUBB4BP8	0	0	0	0	0	0	0	0

TUBB6	16.384917	64.437085	43.431922	92.794799	68.795556	127.032889	0	48.727615
TUBB7P	0	0.250563	0	0.42249	0	0.775358	0	1.140039
TUBB8	5.146611	0.182665	0	0.26377	0	0.598863	0	0.913405
TUBB8B	0	0.650017	0	0	0	0.31084	0	0
TUBB8P1	0	0	0	0	0	0	0	0
TUBB8P10	0	0	0	0	0	0	0	0
TUBB8P11	0	0.152792	0	0.094353	0	0	0	0
TUBB8P2	0	0	0	0	0	0	0	0
TUBB8P3	0	0	0	0	0	0	0	0
TUBB8P4	0	0	0	0	0	0	0	0
TUBB8P5	0	0	0	0	0	0	0	0
TUBB8P6	0	0	0	0	0	0	0	0
TUBB8P7	0	0	0	0	0	0	0	0
TUBB8P8	0	1.086758	0	0.106244	0	0	0	0.338755
TUBB8P9	0	0	0	0.13922	0	0	0	0
TUBBP1	0	0	0	0.069628	0	0	0	0
TUBBP10	2.820772	0	0	0.277788	11.874635	0	0	0
TUBBP11	0	0	0	0	0	0	0	0
TUBBP2	0	0	0	0	0	0	0	0.584571
TUBBP3	0	0	0	0	0	0	0	0
TUBBP5	7.691623	3.303953	2.432445	0	0	0	15.830839	0
TUBBP6	0	0	0	0	0	0	0	0
TUBBP8	0	0	0	0	0	0	0	0
TUBBP9	0	0	0	0	0	0	0	0
TUBD1	9.84168	16.357057	4.888751	19.196679	0.853917	5.376692	4.769396	16.109287
TUBE1	21.069529	25.057327	35.981693	17.099723	2.134036	6.259615	8.335042	11.255729
TUBG1	117.864698	109.969849	54.068124	99.606252	101.5516	127.90229	89.604658	108.588798
TUBG1P	0	0	0	0	0	0	0	0
TUBG2	17.225594	16.964183	13.291665	18.411101	13.423513	25.739144	30.178769	20.04358
TUBGCP2	0	8.484605	0	6.039888	7.652594	4.498922	42.305312	8.743386
TUBGCP3	49.667934	71.870159	40.36036	74.173586	25.650225	47.400435	29.001422	73.412247
TUBGCP4	11.537326	30.413744	11.75152	21.945405	17.72838	20.195308	33.562179	26.0266
TUBGCP5	20.361359	37.847963	18.26732	35.124245	56.106238	37.291571	20.028278	59.887722

TUBGCP6	26.791947	6.907613	2.408378	4.864179	9.924002	7.33426	9.872009	3.827268
TUFM	134.535918	188.865755	173.79355	224.873888	186.439866	225.090266	146.582436	177.101158
TUFMP1	0	0	0	0	0	0	0	0
TUFT1	41.521767	30.205858	25.259482	57.292097	34.081993	56.533298	44.076721	57.761114
TULP1	0	0	0	0	0	0	0	0
TULP2	0	0	0	0.562541	0	0	0	0
TULP3	49.793395	38.530079	27.175783	53.354631	37.269673	30.188345	56.893135	35.707259
TULP3P1	0	0	0	0	0	0	0	0
TULP4	44.869845	111.973333	51.421574	65.207864	57.710522	104.316642	51.547971	158.637834
TUNAR	0	0	0	0	0	0	0	0
TUSC2	33.951082	52.62939	24.575585	47.088733	25.695452	43.614793	36.546529	53.294367
TUSC2P1	0	0	0	0	0	0	0	0
TUSC2P2	0	0	0	0	0	0	0	0
TUSC3	44.749161	60.488161	67.365075	35.472864	38.975634	4.119061	23.907806	27.979377
TUT1	4.771325	7.873667	5.942471	8.862811	13.278507	21.440436	81.732733	14.606669
TUT4	89.28791	136.350354	90.31748	125.925264	65.293512	27.542012	77.013907	43.943675
TUT7	12.795267	8.724333	10.976506	11.933936	17.517076	15.6512	20.018853	9.718067
TVP23A	0	0	0	0	0	0.203912	0	0
TVP23B	32.655072	41.608573	43.055122	40.989594	76.18256	18.54869	33.138491	45.372709
TVP23BP1	0	0	0	0	0	0	0	0
TVP23BP2	0	0	0	0	0	0	0	0
TVP23BP3	0	0	0	0	0	0	0	0
TVP23C	5.136014	36.127878	18.83625	20.709492	31.530676	14.25661	105.770889	20.550052
TVP23C-CDR	0	20.29141	6.574927	29.216047	10.584423	19.787366	10.399701	14.298971
TVP23CP1	0	0	0	0	0	0	0	0.860192
TVP23CP2	0	0	0	0	0	0	0	0
TVP23CP3	0	0	0	0	0	0	0	0
TWF1	57.948221	73.085045	74.433078	32.681003	130.305525	12.8755	108.846186	54.95784
TWF1P1	0	0	0	0	0	0	0	0
TWF1P2	0	0	0	0	0	0	0	0
TWF2	13.221196	29.013478	15.697716	30.486839	31.369897	45.226626	27.668722	33.421239
TWIST1	8.71663	9.719622	5.822326	7.284566	2.379462	7.820442	0	2.291284
TWIST2	16.325611	27.437409	4.831808	19.243682	20.048805	22.261781	0	21.85175

TWNK	27.160955	94.547988	29.560219	122.981778	46.33949	107.081223	14.588147	89.131296
TWSG1	35.769609	45.944984	20.234027	28.146182	22.379339	28.631845	66.792933	40.581258
TXK	0	3.927068	2.289407	1.905107	0	0.649873	0	0.70988
TXLNA	39.724102	50.028539	33.426558	50.602045	66.210933	97.16309	51.163202	67.878894
TXLNB	0	0	0	0.034776	0	0	0	0
TXLNG	18.858528	10.325275	15.577112	9.902813	30.880946	14.035249	22.403718	19.653565
TXLNGY	0	0	0	0.292463	0	0.489591	0	1.315166
TXN	1235.78498	894.330512	959.735949	588.388551	901.532891	298.796987	515.256077	556.878196
TXN2	43.761627	46.743685	22.952815	44.80568	45.627789	32.053729	45.435877	26.79923
TXN2P1	0	3.102946	0	0	0	0	0	0
TXNDC11	0	13.91875	0	11.973133	0	14.436541	83.378211	13.424181
TXNDC12	0	0	0	0.420427	0	1.334076	0	2.508561
TXNDC15	21.822854	26.173369	15.621395	37.073286	53.78742	31.980522	122.437681	23.104568
TXNDC16	4.324241	7.701762	7.766302	6.002389	1.903605	2.560292	0.175486	6.929616
TXNDC17	310.679055	325.225847	150.166628	203.704569	305.292829	168.424419	185.730597	272.302608
TXNDC2	0	3.843324	0	0.679134	0	0	0	1.292465
TXNDC5	0	0.249768	0	4.28338	0	37.522684	44.628646	0
TXNDC8	0	0	0	0	0	0	0	0
TXNDC9	53.857988	44.785405	40.964407	22.04616	89.261886	43.303235	77.871117	85.851278
TXNIP	0	0	0	8.258486	0	19.313453	0	0
TXNL1	58.488516	46.015335	35.730115	21.51461	61.524017	28.01056	95.893855	79.951492
TXNL1P1	0	0	0	0	0	0	0	0
TXNL4A	134.058575	235.373919	143.478832	158.442266	94.923635	184.366141	57.777331	218.251953
TXNL4AP1	0	0	0	0	0	0	0	0
TXNL4B	25.621115	27.162229	18.099316	25.938271	20.63412	17.476985	40.493805	30.965079
TXNP1	0	0	0	0	0	0	0	0
TXNP2	0	0	0	0	0	0	0	0
TXNP3	0	0	0	0	0	0	0	0
TXNP4	0	0	0	1.532329	0	0	0	0
TXNP5	0	0	0	0	0	0	0	0
TXNP6	0	0	0	0	0	0	0	0
TXNP7	0	0	0	0	0	0	0	0
TXNRD1	219.727468	435.533876	305.195735	743.699024	264.098184	470.238033	282.968415	502.483066

TXNRD2	3.492828	9.977454	9.993435	6.789144	21.110534	9.019067	4.504563	5.577856
TXNRD3	0	0	0	0	10.758901	0	0	0
TYK2	5.684548	0	0	2.353059	0	1.747868	0	1.196101
TYMP	0	8.79396	0	5.976734	1.183932	8.702355	0	4.448226
TYMS	89.372562	58.814294	47.108468	48.591862	105.130247	121.345742	110.498803	124.702626
TYMSP1	0	0	0	0	0	0	0	0
TYMSP2	0	0	0	0	0	0	0	0
TYR	0	0	0	0	0	0	0	0
TYRL	0	0	0	0	0	0	0	0
TYRO3	9.779353	17.098009	9.671678	13.514398	35.510722	17.411455	57.098106	14.49131
TYRO3P	0	0	0	0.091331	0	0.06218	3.338243	0.352184
TYROBP	0	0	0	0	0	0	0	0
TYRP1	0	0	0	0.247887	0	0	0	0
TYSND1	7.762116	8.665181	9.506991	8.248996	8.266514	15.116267	9.009778	10.218662
TYW1	8.014081	12.862462	5.388845	13.644099	16.774799	13.178288	2.494469	12.339114
TYW1B	0	1.015812	0	0.605677	0	0.248396	0	1.4452
TYW3	56.456529	45.750762	23.456648	21.936259	10.486794	12.953151	22.445213	43.121912
TYW5	4.466853	3.325164	4.156705	3.879164	23.00619	3.715813	4.928566	4.552477
U2AF1	57.516076	0.719383	37.583905	0.914348	19.441908	9.186618	4.47405	2.202891
U2AF1L4	3.982722	2.740975	1.779186	2.107367	0	11.552572	0	7.188199
U2AF2	43.833986	126.424581	90.979392	144.632469	53.78567	249.657254	104.860722	181.077884
U2SURP	155.331449	125.576621	145.505156	90.196478	311.737157	100.380051	231.142388	162.678886
U2SURPP1	0	0	0	0	0	0	0	0
UACA	22.031936	30.200626	22.907162	22.73436	54.443363	37.295311	73.207504	53.113772
UAP1	0	22.581541	0	8.7085	0	23.018452	0	60.1673
UAP1L1	1.044587	3.139606	4.681552	3.592713	10.514922	7.927291	17.18774	7.354524
UBA1	169.75647	168.937753	155.506875	187.758025	138.277058	223.011518	76.014077	181.297328
UBA2	175.675946	260.718435	181.899807	266.9014	246.368105	264.346942	306.995186	353.931452
UBA3	29.435944	33.199541	26.296562	32.987305	32.725569	25.101036	68.064403	31.237243
UBA5	63.984471	85.300094	56.675509	62.570673	33.063917	76.185759	107.070043	70.190317
UBA52	874.889819	380.615816	865.065162	483.966575	963.434731	585.576534	729.065287	587.760224
UBA52P3	0	0	0	0	0	0	0	0
UBA52P4	0	0	0	0	0	0	0	0

UBA52P5	0	0	0	0	0	0	0	0
UBA52P6	15.481095	0	0	2.116254	0	5.412865	0	2.669474
UBA52P7	0	0	0	0	0	0	0	0
UBA52P8	0	0	0	0	0	0	0	0
UBA52P9	0	0	0	0	0	0	0	0
UBA5P1	0	0	0	0	0	0	0	0
UBA6	52.840019	67.542629	29.439759	43.03791	174.290311	20.990072	125.843974	68.670944
UBA7	6.697542	1.035759	4.527907	2.09668	22.151176	9.945557	16.377232	6.229491
UBAC1	0	0	0	0.284386	15.330797	0.786563	0	1.430085
UBAC2	127.591659	80.8813	43.701159	63.364253	21.513252	21.981147	22.80898	32.901253
UBALD1	15.886752	35.925769	15.370841	40.075222	11.537361	57.809766	18.756965	38.922979
UBALD2	32.458109	86.730824	17.75739	84.241325	21.033997	135.083414	13.242096	72.342033
UBAP1	35.002076	70.169889	35.984747	71.646677	49.183682	84.310627	56.804329	83.435972
UBAP1L	0	0.150901	0	0.096492	0	0.131959	0	0
UBAP2	10.51538	6.827824	0	4.703454	4.919524	14.535483	165.752915	1.178166
UBAP2L	189.385565	133.650799	136.791596	181.307705	104.626263	171.000008	324.397265	133.526936
UBASH3A	0	0	0	0	0	0	0	0
UBASH3B	106.143397	280.682373	100.966483	333.590854	13.587997	72.503335	76.45503	56.916011
UBB	518.383664	982.376773	682.119871	1300.61154	1099.08884	1480.17188	923.053397	907.036835
UBBP1	0	0	0	0	0	0	0	0
UBBP2	0	0	0	0	0	0	0	0
UBBP3	0	0	0	0	0	0	0	0
UBBP4	0	0.178282	0	0.207425	0	0.210113	0	0.72329
UBBP5	0	0	0	0	0	0	0	0
UBC	962.167906	654.211607	1144.18915	1022.10755	1002.21245	831.928599	719.114947	626.171891
UBD	0	0	0	0	0	0	0	0.510492
UBDP1	0	0	0	0	0	0	0	0
UBE2A	99.997778	81.707205	76.048779	72.056155	95.977168	89.138193	85.448994	88.4007
UBE2B	18.178696	18.136366	56.506236	10.940739	0	20.0086	0	26.494971
UBE2C	241.116841	176.218326	181.256913	180.545935	121.128955	175.969407	291.199783	228.590893
UBE2CP1	0	0	0	0	0	0	0	0
UBE2CP2	0	1.480311	0	2.015886	0	0	0	2.022851
UBE2CP3	0	0	0	0	0	0	0	0

UBE2CP4	0	0	0	0	0	0	0	0
UBE2CP5	0	2.73181	0	0	0	0	0	0
UBE2D1	27.854104	18.536167	16.193588	12.521492	25.600292	10.150451	22.745602	12.639801
UBE2D2	133.493748	188.787484	162.029083	132.344512	130.904154	245.047032	83.903565	205.825358
UBE2D3	210.598026	432.283538	241.877038	383.213717	220.501551	499.009437	220.4775	507.53811
UBE2D3P1	0	0	0	0	0	0	0	0
UBE2D3P2	0	0	0	0	0	0	0	0
UBE2D3P3	0	0	0	0	0	0	0	0
UBE2D3P4	0	0	0	0	0	0	0	0
UBE2D3P5	0	0	0	0	0	0	0	0
UBE2D4	9.957683	19.505489	4.468207	13.345736	64.885523	28.473628	31.578084	21.783804
UBE2E1	0	56.223665	15.649578	75.83037	24.063303	90.058341	0	53.211911
UBE2E2	24.095276	45.6742	20.620104	34.388803	14.545018	16.333123	19.936943	25.055993
UBE2E3	73.149514	77.881896	58.585319	40.165601	41.159757	66.235572	238.819928	72.175721
UBE2E4P	0	0	0	0	0	0	0	0
UBE2F	34.656011	50.276207	32.949896	29.662807	0	73.098509	17.691178	75.205547
UBE2F-SCLY	0	0	0	0.162727	0	0	0	0
UBE2FP1	0	8.912937	10.039952	1.914134	33.682375	4.249332	57.218502	4.535306
UBE2FP2	0	0	0	0	0	0	0	0
UBE2FP3	0	0	0	0	0	0	0	0
UBE2G1	39.499927	77.754104	16.120318	49.277995	71.298639	73.283173	206.931233	118.656501
UBE2G2	25.743038	85.352159	26.145607	74.421974	74.218625	125.539234	52.958328	127.985417
UBE2H	67.754204	115.605939	0	201.317052	29.36504	147.629936	81.592951	87.411967
UBE2HP1	0	1.976178	0	0.791564	0	0	0	0
UBE2I	23.560344	19.00614	14.644299	23.193015	29.331476	17.146258	0	19.032319
UBE2J1	22.96408	19.953638	27.010543	15.178677	11.083779	13.262438	28.332107	23.847458
UBE2J2	18.766645	76.732983	29.876875	42.322846	79.878472	109.791385	4.066929	109.693159
UBE2K	139.284386	220.489544	34.429928	194.492587	45.241473	113.169235	67.337187	158.807333
UBE2L2	0	0	0	0	0	0	0	0
UBE2L3	0	0.909045	0	0.200645	0	0.178854	0	1.336947
UBE2L4	0	0	0	0	0	0.509533	0	0
UBE2L5	0	0	0	0	0	0	0	0
UBE2L6	58.760533	50.66936	37.771928	56.629464	283.65659	188.493274	226.076288	124.469424

UBE2M	0	73.078603	0	94.17111	0	155.982419	66.206587	84.754847
UBE2MP1	0	1.885069	0	0	0	0	0	0
UBE2N	211.981114	140.625956	178.227215	104.716373	239.441177	168.056146	381.974784	165.403076
UBE2NL	0	0	0	0	0	0	0	0
UBE2NP1	0	0	0	0	0	0	0	0
UBE2O	16.99355	20.025289	23.105634	22.744833	29.036379	32.779244	12.125613	22.374602
UBE2Q1	44.358619	173.494536	37.946347	149.867991	47.516942	176.737835	151.327786	104.878589
UBE2Q2	0	46.859201	23.149953	28.935332	31.60491	41.0644	9.83254	35.623945
UBE2Q2P1	9.710741	0.679992	5.032147	0.258819	2.702537	1.065721	0	0
UBE2Q2P10	0	0	0	0	0	0	0	0
UBE2Q2P11	0	2.074719	0	0	0	0	0	0
UBE2Q2P12	0	0	0	0	0	0	0	0
UBE2Q2P13	0	0.564514	0	0	0	0	0	0
UBE2Q2P16	0	0.653765	0	0	0	0	0	2.026127
UBE2Q2P2	0	0.524729	0	0.800375	0	0.914039	0	0
UBE2Q2P5Y	0	0	0	0	0	0	0	0
UBE2Q2P6	0	0	0	0	0	0	0	0
UBE2Q2P8	0	0	0	0	0	0	0	0
UBE2Q2P9	0	0	0	0	0	0	0	0
UBE2QL1	0	0	0	0.026989	0	0.05533	0	0
UBE2R2	28.631299	56.443725	30.982435	53.182423	46.148839	67.880949	50.047962	83.859095
UBE2S	139.60431	444.929577	149.250044	371.924477	322.420199	909.977035	250.77766	788.686445
UBE2SP1	0	0	0	0	0	0	0	0
UBE2SP2	0	0	0	0	0	0	0	0
UBE2T	99.857758	81.377788	75.296878	63.586785	93.324201	35.989005	55.018843	40.529511
UBE2U	0	0	0	0	0	0	0	0
UBE2V1	57.369372	40.572489	12.550818	67.727249	95.169455	63.394814	113.513434	24.853202
UBE2V1P1	0	2.256922	0	0.275367	0	0	0	0
UBE2V1P10	0	0	0	0	0	0	0	0
UBE2V1P11	0	0	0	0	0	0	0	0
UBE2V1P12	0	0	0	0	0	0	0	0
UBE2V1P13	0	0	0	0	0	0	0	0
UBE2V1P14	0	0	0	0	0	0	0	0

UBE2V1P15	0	0	0	0	0	0	0	0
UBE2V1P16	0	0	0	0	0	0	0	0
UBE2V1P2	0	0	0	0	0	0	0	0
UBE2V1P3	0	0	0	0	0	0	0	0
UBE2V1P4	0	0	0	0	0	0	0	0
UBE2V1P5	0	0	0	0	0	0	0	0
UBE2V1P6	0	0	0	0	0	0	0	0
UBE2V1P7	0	0	0	0	0	0	0	0
UBE2V1P8	0	0	0	0	0	0	0	0
UBE2V1P9	0	0	0	0	0	0	0	0
UBE2V2	90.170523	120.345036	97.619409	102.354916	144.253607	67.460325	156.638582	111.76864
UBE2V2P1	0	0	0	0	0	0	0	0
UBE2V2P2	0	0	0	0	0	0	0	0
UBE2V2P3	0	0	0	0	0	0	0	0
UBE2V2P4	0	0	0	0	0	0	0	0
UBE2W	11.681212	2.718493	5.032461	2.824147	37.983942	0.30685	3.480366	0.46628
UBE2WP1	0	0	0	0	0	0	0	0
UBE2Z	74.984034	56.726888	77.907798	61.960533	87.426725	47.363679	56.773673	47.369484
UBE3A	43.072899	160.135449	4.050898	238.382447	56.143931	161.702415	148.700659	231.844058
UBE3AP2	0	0	0	0	0	0	0	0
UBE3B	49.134714	107.147086	25.695804	107.026883	72.74649	174.612865	44.181679	244.527457
UBE3C	58.27178	104.974382	62.754341	74.421986	73.080102	84.483529	129.33259	149.251174
UBE3D	8.092942	3.290662	5.261141	4.14518	2.341128	4.061579	0	3.480017
UBE4A	7.955218	9.671561	0	18.759486	0	5.329854	0	0
UBE4B	18.398609	32.119116	31.596726	36.233105	24.488718	45.185658	70.337063	61.324975
UBFD1	29.830827	41.861031	56.960446	53.820506	39.631565	39.803037	34.719812	38.022287
UBFD1P1	0	0	0	0	0	0	0	0
UBIAD1	3.900909	9.525954	0	7.797132	0.685979	18.024653	38.09083	16.522038
UBL4A	51.468584	120.827772	30.243231	114.236737	43.638966	199.664937	7.777299	133.240988
UBL5	8.089938	72.256126	158.207791	85.507146	0	73.219139	137.342221	75.208494
UBL5P1	0	0	0	0	0	0	0	0
UBL5P2	0	0	0	0	0	0	0	0
UBL5P3	0	0	0	0	0	0	0	0

UBL5P4	0	0	0	0	0	0	0	0
UBL7	18.463554	30.686896	18.285796	35.542575	36.283168	36.249762	15.747825	29.275974
UBLCP1	37.910422	17.081564	15.527375	7.832725	40.822663	6.575863	56.649263	16.812175
UBN1	36.41669	31.561203	0	36.23132	16.631792	56.055335	57.873419	43.807913
UBN2	3.697412	7.091755	3.326039	3.933659	13.742527	3.884093	0.703772	4.939708
UBOX5	1.531406	5.841956	4.124533	4.564233	1.415599	5.267968	0	5.607024
UBP1	52.853254	95.975812	42.381031	95.874022	71.630904	61.7823	96.017173	92.139522
UBQLN1	51.32893	80.243815	48.360761	82.795309	81.197202	84.248995	108.41589	132.898838
UBQLN1P1	0	0	0	0	0	0	0	0
UBQLN3	0	0	0	0	0	0	0	0
UBQLN4	28.75179	35.335616	38.375685	31.727918	32.079412	51.174436	33.384382	38.439678
UBQLN4P1	0	0.842778	0	0.197131	0	0.274238	0	0.582137
UBQLN4P2	0	0	0	0	0	0	0	0
UBR1	12.164147	23.330906	49.055608	18.615688	23.913244	9.579607	14.527661	16.733925
UBR2	0	0	0	0	6.085527	0	9.534995	0
UBR3	0	0	0	0.077324	33.722314	0.374349	20.201316	2.662497
UBR4	32.886129	21.984382	39.758476	31.733297	45.145283	47.962787	57.919488	31.653653
UBR5	42.593086	51.240452	52.410469	52.518468	31.089022	24.324574	86.656308	29.569922
UBR7	42.457523	67.600144	66.964702	51.69756	35.695842	56.577662	144.821344	78.746895
UBTD1	17.230742	19.67302	15.348031	13.686385	5.106502	33.411836	0	22.90498
UBTD2	35.965625	59.455212	29.202173	44.350213	29.664129	28.775426	19.932343	45.65755
UBTD2P1	0	0	0	0	0	0	0	0
UBTF	57.296991	76.892825	24.415399	83.453472	51.059857	146.299157	62.074185	103.369043
UBTFL1	0	0	0	0	0	0	0	0
UBTFL10	0	3.244738	0	0	0	0	0	0
UBTFL11	0	0	0	0	0	0	0	0
UBTFL2	0	0	0	0	0	0	0	0
UBTFL3	0	0	0	0	0	0	0	0
UBTFL5	0	0	0	0	0	0	0	0
UBTFL6	0	0	0	0	0	0	0	0
UBTFL7	0	0	0	0	0	0	0	0
UBTFL8	0	0	0	0	0	0	0	0
UBTFL9	0	0	0	0	0	0	0	0

UBXN1	0	71.220406	6.957829	36.615103	0	195.046765	81.042043	103.145512
UBXN10	0	0	0	0	0	0	0	0
UBXN11	1.531397	7.86808	5.242387	7.220541	0.388234	16.832283	10.003023	12.626884
UBXN2A	35.939251	68.280797	17.791218	30.529089	17.574585	30.443378	35.221783	61.427764
UBXN2AP1	0	0	0	0	0	0	0	0
UBXN2B	12.509263	19.97223	17.186385	19.800193	1.7122	3.116802	2.726243	9.42134
UBXN4	45.687907	80.919466	48.873578	67.41449	45.704118	98.729198	32.702774	82.982676
UBXN6	38.229542	48.350772	30.867378	63.416497	56.323272	109.90797	66.46423	80.277624
UBXN7	54.583286	48.278221	56.243814	79.820415	88.715288	49.833275	113.394699	41.612305
UBXN8	9.947711	4.377638	18.316211	4.806998	21.355458	3.482446	0	3.030547
UCHL1	5.240675	5.657303	0	5.275589	6.08005	11.509962	0	6.791362
UCHL3	0	6.485964	0	5.370676	17.130659	6.116071	0	11.130823
UCHL5	75.91997	91.098955	107.748157	50.239866	99.826081	34.877662	38.306046	86.032017
UCK1	39.46235	49.857507	24.118585	37.148397	31.792789	54.095556	37.605292	46.152601
UCK2	82.792966	124.613227	126.765895	119.203577	99.319733	101.711625	86.85875	92.683723
UCKL1	5.715548	18.173326	20.927775	18.488079	10.544414	42.922192	24.884121	34.837864
UCMA	0	0	0	0	0	0	0	0
UCN	0	0	0	1.227635	0	0	0	0
UCN2	0	0	0	0.522134	0	0.471109	0	0.097086
UCN3	0	0	0	0	0	0	0	0
UCP1	0	0	0	0	0	0	0	0
UCP2	0	2.034039	0	0	0	0	0	0
UCP3	0	1.06568	0	0.183069	0	0.878967	0	0.121356
UEVLD	16.548096	26.10758	24.86602	13.8075	59.972876	11.801359	23.27636	22.962678
UFC1	69.841902	81.024187	82.613391	99.146565	54.776479	106.342964	51.211649	121.89756
UFC1P1	0	0	0	0	0	0	0	0
UFD1	42.528748	89.642587	139.893202	69.736667	148.918726	123.35622	248.923658	126.982889
UFL1	11.695795	7.605179	14.698955	5.940909	12.355504	3.703808	11.387542	8.963607
UFM1	30.627997	17.209054	49.623856	12.340983	48.560705	7.034226	46.925257	16.863107
UFM1P1	0	0	0	0	0	0	0	0
UFM1P2	0	0	0	0	0	0	0	0
UFM1P3	0	0	0	0	0	0	0	0
UFSP1	0	1.759144	0	1.025022	10.20051	2.628207	0	1.584911

UFSP2	8.470663	6.969088	7.546659	9.067047	24.563507	3.904195	73.50929	19.662005
UGCG	80.146766	87.60467	92.902837	64.19919	30.979543	29.422523	74.494506	40.71811
UGDH	153.893979	385.465587	155.387457	330.371345	76.788008	83.086359	55.198338	129.818069
UGGT1	38.618573	19.270862	61.365315	37.367882	74.0664	29.71741	41.122913	30.707988
UGGT2	37.933418	4.294155	14.532659	2.486282	17.302818	1.967363	0	1.837422
UGP2	21.828418	70.842474	8.700218	72.262158	45.44367	99.057906	0	67.334177
UGT1A1	6.153079	3.813146	5.497449	4.620274	0	1.83178	6.803571	2.440754
UGT1A10	0	4.72906	0	1.848259	2.75395	4.767869	0	2.850814
UGT1A11P	0	0	0	0	0	0	0	0
UGT1A12P	0	0	0	0	0	0	0	0
UGT1A13P	0	0	0	0	0	0	0	0
UGT1A2P	0	0	0	0.441322	0	0.221453	0	0.378321
UGT1A4	0	0	0	0.586445	0	0	0	0.226489
UGT1A6	124.910438	150.86688	99.914842	181.673248	89.2448	92.515392	94.621415	119.423964
UGT1A7	1.518712	0.064843	2.715271	0.186436	0	0.076082	0	0.245687
UGT1A9	0	0	0	0	0	0	0	0
UGT2A1	0	0	0	0	0	0	0	0
UGT2A2	0	0	0	0	0	0	0	0
UGT2A3	0	0	0	0	0	0	0	0
UGT2A3P7	0	0	0	0	0	0	0	0
UGT2B10	0	0	0	0	0	0	0	0
UGT2B11	0	0	0	0	0	0	0	0
UGT2B15	0	0	0	0	0	0	0	0
UGT2B24P	0	0	0	0	0	0	0	0
UGT2B25P	0	0	0	0	0	0	0	0
UGT2B26P	0	0	0	0	0	0	0	0
UGT2B27P	0	0	0	0	0	0	0	0
UGT2B28	0	0	0	0	0	0	0	0
UGT2B29P	0	0	0	0	0	0	0	0
UGT2B4	0	0	0	0	0	0	0	0
UGT2B7	0	0	0	0	0	0	0	0.094609
UGT3A1	0	0	0	0.224103	0	0.107828	0	0
UGT3A2	0	0	0	0.382112	0	0.072468	0	0.292401

UGT8	8.8802	12.339798	8.763483	13.632839	0	0.682111	3.888394	1.789917
UHMK1	65.37884	38.860876	45.223559	20.779128	46.976485	13.221996	46.467287	42.94459
UHRF1	55.157226	61.149692	24.040014	53.649059	99.261895	102.807597	91.380564	73.206802
UHRF2	8.362057	17.883095	18.550358	21.83527	9.224543	16.917715	114.285441	30.849092
UHRF2P1	0	0	0	0	0	0	0	0
UIMC1	39.425055	72.092009	22.421289	93.354463	27.365995	85.952369	89.01819	58.563058
ULBP1	4.164547	2.934639	4.66618	4.100481	0	0.363351	0	0.669725
ULBP2	8.212431	8.762785	14.582192	17.49369	5.042519	9.764441	0	5.380416
ULBP3	5.715595	6.135907	1.929841	8.368344	10.371225	6.357678	0.261643	8.391212
ULK1	4.90727	5.980359	16.032949	8.511461	7.723244	8.296732	5.828575	7.023533
ULK2	6.054946	17.695287	2.551423	16.962779	47.668314	10.371455	56.629137	12.467439
ULK3	9.206959	12.74022	9.952854	15.794031	15.14173	21.925417	6.702977	16.123915
ULK4	17.534004	18.550152	8.261054	20.071755	21.165761	4.683024	37.470523	16.623309
ULK4P1	0	0	0	0	0	0	0	0
ULK4P2	0	0	0	0	0	0	0	0
ULK4P3	0	0	0	0	0	0	0	0
UMAD1	56.489093	31.238608	19.88314	31.668392	7.761776	8.670095	12.280511	13.078599
UMOD	0	0	0	0	0	0	0	0
UMODL1	0	0	0	0.060681	0	0	0	0
UMPS	103.918077	76.702699	26.773844	78.059399	59.426449	157.576876	72.784666	120.54728
UNC119	13.103321	1.985314	5.487659	0.651845	4.39868	1.839555	7.361462	1.300725
UNC119B	21.776868	25.798142	18.877967	16.913244	19.039807	18.581763	11.882876	24.602417
UNC13A	0	0	0	0	0	1.697689	0	0.634483
UNC13B	4.63566	10.459907	5.09383	14.773924	11.296159	29.582476	5.900797	21.673537
UNC13C	0	0	0	0	0	0	0	0.512162
UNC13D	0	0	0	12.859139	0	0	0	9.556843
UNC45A	33.252508	33.205121	29.46684	51.25575	50.892802	79.842606	79.458817	53.079676
UNC45B	0	0	0	0	0	0	0	0
UNC50	0	2.868895	15.356998	0.43063	0.511158	1.317525	0	2.242217
UNC5A	0	0.532021	0	0.281733	0	0.532189	0	0.762061
UNC5B	4.261042	7.259574	8.089329	9.978319	3.986623	2.850771	0	1.19948
UNC5C	0	0	0	0	0	0	0	0
UNC5CL	0	0.398808	0	0.258892	0	0	0	0

UNC5D	1.115284	1.118942	2.883452	1.968211	0	0	0	0.056338
UNC79	0	0.247891	0	0.21585	0.608327	0.1734	0	0.16908
UNC80	0	0.03469	0	0	0	0	0	0
UNC93A	0	0	0	0	0	0	0	0
UNC93B1	29.670545	8.854508	23.139663	10.92026	3.545293	12.985605	1.154825	11.917388
UNC93B2	0	0	0	0.273772	0	0	0	0
UNC93B3	0	0	0	0	0	0	0	0
UNC93B4	0	0	0	0	0	0	0	0
UNC93B5	0	0	0	0	0	0	0	0
UNC93B6	0	0	0	0	0	0.22983	0	0
UNC93B7	0	0	0	0	0	0	0	0
UNC93B8	0	0	0	0	0	0	0	0
UNCX	0	0.700104	0	0.080301	0	0	0	0
UNG	25.974986	45.128587	42.200369	58.008774	9.524924	121.978857	179.522534	36.776148
UNGP1	0	0	0	0	0	0	0	0
UNGP3	0	1.279115	0	0	0	0.301696	0	0
UNK	35.771826	23.090937	18.920253	24.326096	23.16274	27.160055	0	35.6956
UNKL	16.633023	15.255217	36.935991	6.223564	12.182047	7.570162	0	10.669809
UPB1	0	0	0	0	0	0	0	0
UPF1	31.364055	2.120051	16.607781	11.792003	30.93985	8.548949	0	16.802418
UPF2	11.382495	5.196591	10.226574	14.141047	25.10802	14.901797	6.470169	8.50326
UPF3A	23.448064	50.157746	51.183453	46.300883	76.293148	52.005328	41.019332	54.471676
UPF3AP1	0	0	0	0	0	0	0	0
UPF3AP2	0	0	0	0.098572	0	0.195137	0	0.329856
UPF3AP3	0	0.652526	0	0.768286	0	0	0	0
UPF3B	49.812135	10.843081	5.689985	10.617977	1.569813	6.894155	0	7.205083
UPK1A	0	0	0	0	0	0	0	0
UPK1B	1.797613	1.138123	1.604422	1.050803	0	0	0	0
UPK2	21.00163	13.991715	10.929133	11.430168	0	0.785219	0	1.373198
UPK3A	0	0	0	0	0	0	0	0
UPK3B	0	0	2.104614	0.234859	0.62293	3.20334	0	1.925132
UPK3BL1	0	6.459508	16.911327	4.001769	18.101689	0	0	3.580704
UPK3BL3P	5.171945	0.705216	0	2.632725	0	7.821634	39.310845	3.433738

UPK3BP1	0	2.534157	0	0.774597	0	0.880591	0	1.623409
UPP1	24.236057	200.157996	66.886881	244.47928	80.239669	292.245979	26.477478	314.123112
UPP2	0	0	0	0	0	0	0	0
UPRT	20.305639	18.411352	13.827213	22.925086	4.719552	15.150386	33.022133	22.871362
UQCC1	45.386425	65.935865	60.541389	60.250799	56.707574	24.561749	62.700628	20.559257
UQCC2	101.268581	181.174171	198.942614	197.689216	117.193089	139.74293	126.408402	100.393438
UQCC3	1.807924	12.689254	8.067654	7.813077	13.3439	43.333693	7.875559	31.76863
UQCC4	21.488431	32.405314	22.692917	26.78565	5.637936	43.639873	3.077177	35.677261
UQCC5	86.739435	84.659725	46.585024	54.744182	116.771303	111.079314	82.068058	83.409258
UQCC6	8.925609	23.269254	10.94031	21.329385	15.313642	39.068056	0	25.952893
UQCR10	55.14775	84.319219	71.956661	85.728794	159.858855	112.807528	3.682843	124.064129
UQCR10P1	0	0	0	0	0	0	0	0
UQCR11	36.889068	62.997032	68.498285	40.965707	91.551493	61.744937	116.34791	60.388385
UQCRB	301.100118	391.058274	269.017335	312.417824	152.03881	97.623707	149.68228	191.679436
UQCRBP1	0	0	0	0	0	0	0	0
UQCRBP2	0	0	0	0	0	0	0	0
UQCRBP3	0	0	0	0	0	0	0	0
UQCRC1	112.867923	166.415406	113.332915	162.215595	108.268435	203.377531	0	197.956764
UQCRC2	113.934012	118.970184	135.670502	145.931124	116.438845	83.988482	153.467505	108.637724
UQCRC2P1	0	0	0	0	0	0	0	0
UQCRFS1	28.235383	31.288864	19.463611	20.673393	35.544017	33.803925	39.582412	58.454731
UQCRFS1P1	0	0	0	0.243232	0	4.81306	0	2.028873
UQCRFS1P2	0	0	0	0	0	0	0	0
UQCRFS1P3	0	0	0	0	0	0	0	0
UQCRH	305.295088	280.579396	269.518576	230.044982	314.739355	312.613803	217.689554	340.488634
UQCRHL	0	0.069701	0	0.202384	0	0.08224	0	0.130849
UQCRHP1	0	0	0	0.647692	0	0	0	0
UQCRHP2	0	0	0	0	0	0	0	0
UQCRHP3	0	0	0	0	0	0	0	0
UQCRHP4	0	0	0	0	0	0	0	0
UQCRQ	519.819657	729.806832	444.677046	470.936898	639.509357	524.45232	187.625415	577.571939
URAHP	0	2.50459	0	2.670584	0	0	0	0.61888
URB1	8.002557	8.897118	12.806489	11.411863	11.360375	17.588036	18.302543	16.437573

URB2	9.309084	17.624179	13.072077	11.777671	13.219509	17.289739	14.886808	26.55294
URGCP	13.631323	43.236858	6.452855	69.20689	0	159.785682	46.267983	126.151342
URGCP-MRP	96.070178	0	0	0	0	0	0	2.663471
URI1	73.73195	202.497877	50.244508	149.955731	67.249143	183.546713	69.309987	268.954319
URM1	36.734446	7.057232	0	12.805017	20.79452	8.063454	0	10.783286
UROC1	0	0	0	0	0	0	0	0
UROD	40.729063	29.193505	16.59763	30.250062	57.306764	45.174651	48.560997	47.86076
UROS	39.162117	44.710689	36.155702	32.61996	64.538105	34.247997	57.122889	52.04722
USB1	1.130853	57.706072	6.074663	33.672627	54.585634	20.593136	69.301626	26.228245
USE1	8.390739	16.220752	14.312968	13.418241	15.014404	29.394527	17.228157	28.755122
USF1	20.555196	84.459005	20.777916	78.49798	26.095107	112.249506	7.481957	91.073014
USF2	25.885665	43.68817	38.282403	45.190527	90.807954	98.815745	76.482715	69.550769
USF3	15.153114	15.155934	19.365877	14.96943	11.776654	8.380465	21.973565	10.295223
USH1C	0	0	0	0	0	0	0	0
USH1G	0	0.399481	0	1.093901	0.24662	4.382761	4.090102	2.327033
USH2A	0	0	0	0.016475	0	0	0	0
USHBP1	0	0	0	0	0	0.416668	0	0
USO1	33.024483	21.721505	37.508409	16.324365	0	8.492776	0	21.382159
USP1	141.31354	208.285857	96.764938	89.448113	75.469726	38.866868	86.660623	133.028233
USP10	26.416056	156.434623	30.578243	208.21634	56.487788	284.942391	107.460572	192.367579
USP10P3	0	0	0	0	0	0	0	0
USP11	42.966437	43.696447	58.231937	59.775251	80.28575	53.833254	102.144274	36.566382
USP12	12.67216	9.971508	14.724406	5.398037	14.175309	5.892525	21.595982	9.90464
USP12P1	0	0	0	0	0	0	0	0
USP12P2	0	0	0	0	0	0	0	0
USP12PX	0	0	0	0	0	0	0	0
USP12PY	0	0	0	0	0	0	0	0
USP13	19.496814	23.94152	19.6022	25.20705	8.543912	18.32156	49.453819	28.798331
USP14	69.648216	89.88625	38.651406	43.382383	100.781074	44.980689	123.233414	118.280677
USP15	34.808884	56.677512	89.02624	35.388489	118.954228	44.341039	191.235179	100.319821
USP16	21.853309	34.396576	24.728041	13.658972	27.517751	27.360386	41.918842	49.25364
USP17L1	0	0	0	0	0	0	0	0
USP17L10	0	0	0	0	0	0	0	0

USP17L11	0	0	0	0	0	0	0	0
USP17L12	0	0	0	0	0	0	0	0
USP17L13	0	0	0	0	0	0	0	0
USP17L14P	2.236365	0	0	0.216619	0	0	0	0.122866
USP17L16P	0	0	0	0	0	0	0	0
USP17L17	0	0	0	0	0	0	0	0
USP17L18	0	0	0	0	0	0	0	0
USP17L19	0	0	0	0	0	0	0	0
USP17L20	0	0	0	0	0	0	0	0
USP17L21	0	0	0	0	0	0	0	0
USP17L22	0	0	0	0	0	0	0	0
USP17L23	0	0	0	0	0	0	0	0
USP17L26	0	0	0	0	0	0	0	0
USP17L3	0	0	0	0	0	0	0	0
USP17L4	0	0	0	0	0	0	0	0
USP17L5	0	0	0	0	0	0	0	0
USP17L6P	3.110252	0	0	0	0	0	0	0
USP17L8	0	0	0	0	0	0	0	0
USP17L9P	0	0	0	0.216619	0	0	0	0
USP19	0	7.712083	0	6.258031	0	15.980268	0.794597	6.387322
USP2	18.765965	33.870831	9.508547	31.256181	13.491679	6.331918	10.520148	4.219387
USP20	8.088682	5.360263	0	0.924635	0	3.529504	0	1.695739
USP21	30.861409	20.115111	18.248138	24.361352	36.649319	16.054243	9.18691	15.710523
USP21P1	0	0.23634	0	1.131227	0	0	0	0
USP21P2	0	0	0	0	0	0	0	0
USP22	96.637817	122.174126	75.624903	129.776015	79.595497	211.755089	250.243276	207.392469
USP24	43.776762	48.941076	45.07689	33.786073	18.146689	13.468242	17.608877	33.160997
USP24P1	0	0	0	0	0	0	0	0
USP25	16.987098	41.550082	14.182769	25.935887	43.847397	26.646512	90.499358	49.917577
USP26	0	0.116968	0	0.022239	0	0	0	0
USP28	40.038792	59.070613	64.761486	60.248797	62.835316	92.210417	116.346682	101.692985
USP29	0	0	0	0	0	0	0	0
USP3	46.276126	41.44048	56.632038	27.57378	21.987503	15.926569	33.334694	32.234641

USP30	6.415229	9.39593	8.406975	14.63869	35.348824	5.721531	33.877007	10.424074
USP31	2.464464	0.492612	4.525484	15.43266	2.667225	21.656145	0	8.994085
USP32	51.515986	92.436926	45.004486	92.339664	82.733628	102.232576	87.405763	105.669699
USP32P1	0	1.311251	11.195755	1.819586	0	0	0	0.522857
USP32P2	0	0	0	0	0	0	0	0
USP32P3	0	0	0	0	0	0	0	0
USP32P4	0	0	0	0	0	0.337484	0	0
USP33	41.505859	48.633606	29.921108	37.062269	64.145723	23.863046	75.259366	41.383655
USP34	19.131947	13.480326	38.68853	7.328561	36.333802	8.893403	118.512404	5.685251
USP35	2.636576	4.73488	9.300746	9.698396	5.137314	4.01939	0	2.774719
USP36	11.2753	41.125269	11.67949	57.725429	50.860016	50.510464	20.86448	47.309843
USP37	13.44128	37.471931	15.114262	25.429282	45.784401	28.978425	19.458262	49.636619
USP37P1	0	0	0	0	0	0	0	0
USP38	0	14.959341	0	9.247098	4.768001	7.172974	9.707179	15.817781
USP39	23.646611	43.290757	48.008421	44.458591	57.999049	20.382544	99.304394	28.258986
USP4	7.256128	16.498707	13.948158	12.028988	27.972574	35.211862	97.050131	17.786481
USP40	18.647363	27.876898	12.481101	21.364607	5.649488	125.303306	30.229923	138.62105
USP41P	0	0	0	0	0	0	0	0
USP42	15.558559	3.323329	0	13.775803	16.089395	13.895853	15.7104	7.479953
USP43	9.232035	7.232836	3.53545	6.247383	8.122306	11.415315	2.80191	8.501492
USP44	3.296662	10.808164	4.437973	5.597791	0.508237	3.683144	9.794297	4.543079
USP45	7.774688	9.649711	9.077019	4.959798	39.940769	3.777062	0	11.071492
USP46	9.942129	15.77693	10.995164	13.736136	2.438492	14.575567	6.917068	14.141962
USP47	3.01269	9.522049	2.928019	5.613941	9.343767	6.232091	65.408637	6.395208
USP48	7.363637	40.000773	47.990331	35.198448	80.990752	18.64853	55.918276	45.271089
USP49	1.068406	1.822459	10.599017	2.518568	17.363504	1.376258	13.563137	1.289389
USP5	7.614575	29.61189	25.328713	45.235455	17.249046	59.895062	72.265585	33.43856
USP50	0	0	0	0	0	0	0	0
USP51	0	3.13665	0	1.596484	0	0	0	0
USP53	32.312059	42.800418	0.698746	49.273849	4.505898	31.011945	12.629459	45.013776
USP54	11.368487	44.238075	31.355867	45.533302	59.647484	35.174718	12.045848	56.235545
USP6	0	0	0	0	0	0	0	0
USP6NL	5.097397	6.201872	1.796052	8.262208	3.654401	6.599938	0	10.075296

USP7	111.705636	165.882707	102.383064	130.868023	140.193137	184.88892	306.179136	206.609294
USP8	83.527176	107.301632	68.626077	91.277705	128.092192	90.123466	42.216659	98.682036
USP8P1	0	0	0	0	0	0.210238	0	0
USP8P2	0	0	0	0	0	0	0	0
USP9X	29.07271	40.861056	30.9985	30.886234	44.900152	49.198962	91.011006	89.703922
USP9Y	15.999553	42.170223	12.378778	44.837369	32.868648	67.332173	25.491667	92.670789
USP9YP1	0	0	0	0	0	0	0	0
USP9YP10	0	0	0	0	0	0	0	0
USP9YP11	0	0	0	0	0	0	0	0
USP9YP12	0	0	0	0	0	0	0	0
USP9YP13	0	0	0	0	0	0	0	0
USP9YP14	0	0	0	0	0	0	0	0
USP9YP15	0	0	0	0	0	0	0	0
USP9YP16	0	0	0	0	0	0	0	0
USP9YP17	0	0	0	0	0	0	0	0
USP9YP18	0	0	0	0	0	0	0	0
USP9YP19	0	0	0	0	0	0	0	0
USP9YP2	0	0	0	0	0	0	0	0
USP9YP20	0	0	0	0	0	0	0	0
USP9YP21	0	0	0	0	0	0	0	0
USP9YP22	0	0	0	0	0	0	0	0
USP9YP23	0	0	0	0	0	0	0	0
USP9YP24	0	0	0	0	0	0	0	0
USP9YP25	0	0	0	0	0	0	0	0
USP9YP26	0	0	0	0	0	0	0	0
USP9YP27	0	0	0	0	0	0	0	0
USP9YP28	0	0	0	0	0	0	0	0
USP9YP29	0	0	0	0	0	0	0	0
USP9YP3	0	0	0	0	0	0	0	0
USP9YP30	0	0	0	0	0	0	0	0
USP9YP31	0	0	0	0	0	0	0	0
USP9YP33	0	0	0	0	0	0	0	0
USP9YP34	0	0	0	0	0	0	0	0

USP9YP35	0	0	0	0	0	0	0	0
USP9YP36	0	0	0	0	0	0	0	0
USP9YP4	0	0	0	0	0	0	0	0
USP9YP5	0	0	0	0	0	0	0	0
USP9YP6	0	0	0	0	0	0	0	0
USP9YP7	0	0	0	0	0	0	0	0
USP9YP8	0	0	0	0	0	0	0	0
USP9YP9	0	0	0	0	0	0	0	0
USPL1	4.684013	14.205805	5.40959	14.633232	2.375908	6.837578	5.867239	9.608031
UST	0.730901	8.952107	1.968925	6.038474	2.316594	2.01115	3.915086	2.581773
UTF1	0	0	0	0.14745	0	0	0	0
UTP11	27.417072	38.810202	41.313658	50.244254	80.020345	40.873937	58.513447	64.323405
UTP14A	27.058287	54.87843	41.270419	61.077416	49.074958	46.969442	101.932216	30.670251
UTP14C	0	6.869618	0	0	4.551719	0	0	3.465846
UTP15	28.429651	21.594969	15.150468	13.399088	26.065475	8.626693	38.654844	22.475032
UTP18	33.523294	65.345255	31.571204	45.723737	90.039501	70.20502	39.859082	83.785634
UTP20	20.662855	28.296828	16.349637	20.654182	33.709847	22.766437	48.437085	47.228765
UTP23	24.395807	62.270497	12.158099	40.038755	36.514561	16.271114	42.725833	25.127276
UTP25	14.814963	10.888396	12.646048	12.812955	5.421533	6.715953	10.101441	8.327988
UTP3	13.730931	40.259997	26.054385	33.32569	29.413141	31.797373	21.610514	31.021795
UTP4	114.432033	77.608652	96.317599	86.263438	86.604111	56.958753	187.345618	96.104562
UTP6	45.091822	59.503267	52.311655	60.494227	73.448942	50.285012	247.95331	85.864426
UTRN	10.767961	13.357568	0	18.777915	0	11.261244	0	10.786496
UTS2	0	0	0	0	0	0	0	0
UTS2B	0	0.975737	0	0	0	0	0	0
UTY	4.564851	6.254146	4.09474	5.4988	9.35236	12.885191	30.489802	10.962475
UVRAG	23.920347	39.527978	21.923699	33.165569	44.920198	16.494651	17.204554	34.186093
UVSSA	0	0.222466	0	4.765028	3.674491	6.551186	0	2.440436
UXS1	13.520075	80.011065	25.063538	75.916761	33.963258	75.064384	12.831714	58.674668
UXT	0	5.60175	0	10.489727	0	9.216677	0	15.581592
VAC14	59.904166	58.482225	38.282777	59.150823	24.567022	77.78659	11.429327	67.329798
VAMP1	7.127833	4.977273	0	3.975415	0	2.097027	0	2.189788
VAMP2	9.736209	4.304862	17.492614	6.14853	18.827151	6.839044	8.255398	4.021643

VAMP3	64.79611	59.795644	62.984844	44.94916	79.137838	63.270837	52.84786	77.449211
VAMP4	12.096158	10.33627	5.316271	9.55585	18.137964	5.768802	0	15.0532
VAMP5	0	3.194675	0	2.031142	0	2.794802	0	0
VAMP7	40.536856	24.255681	23.40698	13.732928	53.469317	11.301423	29.886049	23.632014
VAMP8	108.125526	179.384719	99.612812	169.413358	83.111691	152.123901	63.050554	112.888082
VAMP9P	0	0	0	0	0	0	0	0
VANGL1	37.455095	63.103579	24.965449	47.745056	19.036857	41.588029	23.96659	40.698325
VANGL2	4.876898	3.115831	6.573306	3.695537	9.018811	5.039059	2.822049	3.741549
VAPA	272.658244	216.957897	196.497214	143.654191	195.430291	102.172006	167.870425	239.335745
VAPB	105.079043	187.204798	62.63325	171.384246	75.209159	136.814147	180.003552	142.230109
VARS1	16.051019	48.283323	49.543075	42.405421	56.764538	104.278284	101.794159	59.401226
VARS2	7.697279	9.773023	17.915388	9.800241	7.710269	14.602664	11.051455	9.69145
VASH1	0.503071	2.207062	2.261577	5.020584	0	2.447543	0.594484	3.780231
VASH2	0.930214	4.622553	0	3.695549	0	1.336743	0	1.852067
VASN	0	1.126905	1.076381	1.083335	6.026477	3.050656	7.588556	1.741041
VASP	45.070146	77.747431	34.218774	75.740521	77.133765	178.511573	122.652237	134.234494
VAT1	102.750588	85.185546	124.524515	112.171401	117.579913	71.670184	52.017422	47.659561
VAT1L	0	0	0	0	0	0	0	0
VAV1	0	0.219648	1.154658	1.208287	0	0	0	0
VAV2	45.453264	67.438364	41.292792	81.666074	68.695284	68.236434	75.654504	67.680582
VAV3	27.907199	11.523006	4.094034	9.534124	2.693959	1.675494	0	1.649278
VAX1	0	0.2195	0	0.263686	0	0	0	0
VAX2	0	2.789507	5.710402	1.009441	0	1.03127	0	0.262244
VBP1	0	0	0	0	39.520572	0	0	16.801312
VCAM1	0	0	0	0.100079	0	0	0	0
VCAN	0	3.114326	0	0.288242	0	0.019118	0	0
VCF1	45.813039	58.366872	22.672692	29.611222	51.382954	40.24893	13.466711	30.069808
VCF2	16.417963	23.361213	16.516064	15.53048	0	10.175126	0	10.795446
VCL	0	19.172229	14.381304	21.899792	0.583128	32.52238	0	16.992929
VCP	122.356466	247.434501	98.429671	225.307914	190.648655	399.71309	142.145699	458.554501
VCPIP1	12.256328	12.79349	10.450531	10.86664	5.882534	6.33814	2.754904	9.709166
VCPKMT	1.984345	3.916808	1.769218	6.041295	5.322429	4.954001	5.75702	7.191717
VCX	0	0	0	0	0	0	0	0

VCX2	0	0	0	0	0	0	0	0
VCX3A	0	0.479766	0	0.465919	0	0	0	0.317802
VCX3B	0	0	0	0	0	0	0	0
VDAC1	473.374076	308.962476	457.057922	392.35085	372.570897	421.953942	353.54926	313.621122
VDAC1P1	0	0	0	0.11718	0	0	0	0
VDAC1P10	0	0	0	0.213062	0	0	0	0
VDAC1P11	0	0	0	0	0	0	0	0
VDAC1P12	0	0	0	0	0	0	0	0
VDAC1P13	0	0	0	0	0	0	0	0
VDAC1P2	0	0.194107	0	0.11316	0	0	0	0
VDAC1P3	0	0	0	0	0	0	0	0
VDAC1P4	0	0.196813	0	0	0	0	0	0
VDAC1P5	0	0	0	0	0	0	0	0
VDAC1P6	0	0	0	0	0	0	0	0
VDAC1P7	0	0	0	0	0	0	0	0
VDAC1P8	0	0	0	0.112415	0	0	0	0
VDAC1P9	0	0	0	0	0	0	0	0
VDAC2	286.049997	220.511073	219.635049	151.255623	256.383595	74.424306	141.396144	236.640903
VDAC2P1	0	0	0	0	0	0	0	0
VDAC2P2	0	0	0	0	0	0	0	0
VDAC2P3	0	0	0	0	0	0	0	0
VDAC2P4	0	0	0	0	0	0	0	0
VDAC2P5	0	0	0	0.206668	0	1.724253	0	1.336753
VDAC3	186.399082	184.874075	149.773728	143.227113	164.677228	60.003463	168.605301	112.765532
VDAC3P1	0	0	0	0	0	0	0	0
VDR	0	0.21435	0	3.897621	0	2.934445	0	2.103463
VEGFA	10.387942	80.591448	46.865454	137.836855	34.76232	190.646651	150.730551	185.383542
VEGFB	41.415978	25.366044	42.308423	27.444213	94.461503	22.896781	0	17.82895
VEGFC	30.37425	50.404307	33.940892	52.10817	104.529431	71.568802	101.973898	106.707768
VEGFD	0	0	0	0.040614	0	0	0	0
VENTX	0	0	0	0.134835	0	0	0	0
VENTXP1	0	0	0	0	0	0	0	0
VENTXP2	0	0	0	0	0	0	0	0

VENTXP3	0	0	0	0	0	0	0	0
VENTXP4	0	0	0	0	0	0	0	0
VENTXP5	0	0	0	0	0	0	0	0
VENTXP6	0	0	0	0	0	0	0	0
VENTXP7	0	0	0	0	0	0	0	0
VEPH1	0	8.409857	0.745287	9.390954	0	2.546514	0	3.600869
VEZF1	43.018068	47.050138	35.280351	43.566012	19.608622	36.932738	15.203995	44.240584
VEZF1P1	0	0	0	0	0	0	0	0
VEZT	69.814401	178.786106	109.891067	224.850683	193.903666	198.542583	265.830238	299.734091
VEZTP1	0	0	0	0	0	0	0	0
VGF	0	2.209122	0	1.814895	0	0.614403	0	0.37525
VGLL1	10.374173	1.22536	0	0.710982	81.770599	22.917294	0	24.737526
VGLL2	0	0	0	0.050085	0	0	0	0
VGLL3	41.023736	14.053377	20.984532	24.204284	3.981206	8.66779	1.6504	5.808941
VGLL4	23.401262	37.917888	40.121617	75.086186	35.22279	51.466358	10.608865	44.373129
VIL1	0	0	0	0	0	0	0	0
VILL	0	0	5.963227	0.01928	23.506615	0.080595	0	2.112913
VIM	43.038764	29.708744	23.372311	11.987849	6.411639	4.067352	14.03357	14.567596
VINAC1P	0	0	0	0	0	0	0	0
VIP	0	0	0	0	0	0	0	0
VIPAS39	20.149612	18.208173	15.626366	21.651268	30.832757	20.331452	0	26.372612
VIPR1	20.348113	2.4554	4.631041	3.843638	3.034164	4.036601	0	1.727152
VIPR2	0	0	0	0	0	0	0	0.073016
VIRMA	32.912809	33.510573	81.654634	30.45253	23.401169	25.08414	109.409551	20.153698
VIT	0	2.540106	0	0.973153	4.623723	1.92501	0	3.166264
VKORC1	89.732064	110.174296	117.617884	122.504802	16.129041	86.846148	0	112.356564
VKORC1L1	15.452308	28.634565	22.81838	20.086372	18.114729	13.440213	27.776507	17.312038
VKORC1P1	0	0	0	0	0	0	0	0
VLDLR	0	0.561708	0	0.297764	0.195227	0.190034	0	0.391982
VMA21	25.793065	35.308619	0	20.440121	0	11.915914	116.846397	15.389825
VMO1	0	0	0	0.580417	0	0.632174	0	0.232798
VMP1	115.13287	50.049057	88.428181	48.082597	119.686363	20.246019	129.884859	39.872742
VN1R104P	0	0	0	0	0	0	0	0

VN1R105P	0	0	0	0	0	0	0	0
VN1R107P	0	0	0	0	0	0	0	0
VN1R108P	0	0	0	0.232607	0	0	0	0.399911
VN1R10P	0	0	0	0	0	0	0	0
VN1R110P	0	0	0	0.191918	0	0	0	0
VN1R11P	0	0	0	0	0	0	0	0
VN1R12P	0	0	0	0	0	0	0	0
VN1R13P	0	0	0	0	0	0	0	0
VN1R17P	0	0	0	0	0	0	0	0
VN1R18P	0	0	0	0	0	0	0	0
VN1R2	0	0	0	0	0	0	0	0
VN1R20P	0	0.496639	0	0.096557	0	0	0	0.328974
VN1R21P	0	0	0	0	0	0	0	0
VN1R25P	0	0	0	0	0	0	0	0
VN1R28P	0	0	0	0	0	0	0	0
VN1R3	0	0	0	0	0	0	0	0
VN1R31P	0	0	0	0	0	0	0	0
VN1R32P	0	0	0	0	0	0	0	0
VN1R33P	0	0	0	0	0	0	0	0
VN1R34P	0	0	0	0	0	0	0	0
VN1R35P	0	0	0	0	0	0	0	0
VN1R36P	0	0	0	0	0	0	0	0
VN1R37P	0	0	0	0	0	0	0	0
VN1R38P	0	0	0	0	0	0	0	0
VN1R4	0	0	0	0	0	0	0	0
VN1R40P	0	0	0	0	0	0	0	0
VN1R42P	0	0	0	0	0	0	0	0
VN1R46P	0	0	0	0	0	0	0	0
VN1R48P	0	0	0	0	0	0	0	0
VN1R5	0	0	0	0	0	0	0	0
VN1R51P	0	0	0	0	0	0	0	0.169118
VN1R53P	0	0	0	0	0	0	0	0
VN1R54P	0	0	0	0	0	0	0	0

VN1R64P	0	0	0	0	0	0	0	0
VN1R65P	0	0	0	0	0	0	0	0
VN1R66P	0	0	0	0	0	0	0	0
VN1R67P	0	0	0	0	0	0	0	0
VN1R68P	0	0	0	0	0	0	0	0
VN1R69P	0	0	0	0	0	0	0	0
VN1R6P	0	0	0	0	0	0	0	0
VN1R70P	0	0	0	0	0	0	0	0
VN1R71P	0	0	0	0	0	0	0	0
VN1R74P	0	0	0	0	0	0	0	0
VN1R76P	0	0	0	0	0	0	0	0
VN1R78P	0	0	0	0	0	0	0	0
VN1R79P	0	0	0	0	0	0	0	0
VN1R7P	0	0	0	0	0	0	0	0
VN1R80P	0	0	0	0	0	0	0	0
VN1R81P	16.227102	0	0	0.368889	20.687588	2.824489	113.401347	7.714003
VN1R82P	0	0	0	0	3.993013	0	0	0
VN1R83P	0	0	0	0.275367	0	0	0	0
VN1R84P	0	0	0	0	0	0	0	0
VN1R85P	0	0	0	0	0	0	0	0
VN1R87P	0	0	0	0	0	0	0	0
VN1R88P	0	0	0	0	0	0	0	0
VN1R8P	0	0	0	0	0	0	0	0
VN1R90P	0	0	0	0	0	0	0	0
VN1R91P	0	0	0	0	0	0	0	0
VN1R92P	0	0	0	0	0	0	0	0
VN1R93P	0	0	0	0	0	0	0	0
VN1R95P	0	1.787607	0	0.863577	0	0.423884	0	1.165772
VN1R96P	0	0	0	0	0	0	0	0
VN1R9P	0	0	0	0	0	0	0	0
VN2R10P	0	0	0	0.112415	0	0	0	0
VN2R17P	0	0	0	0	0	0	0	0
VN2R19P	0	0	0	0	0	0	0	0

VN2R1P	0	0	0	0	0	0	0	0
VN2R3P	0	0	0	0	0	0	0	0
VN2R9P	0	0	0	0	0	0	0	0
VNN1	0.858979	0	0.770754	0.503197	0.226824	0.300388	4.806806	0.310518
VNN2	0	0.083249	0	1.634176	0	0.486825	0	0.318844
VOPP1	62.915204	115.112433	55.337173	95.801797	60.681704	191.200826	40.412054	186.265049
VPREB3	0	0.754408	0	0.576999	0	0	0	0
VPS11	9.314623	1.996606	1.633684	6.625123	0	0	61.063343	0.143864
VPS13A	21.912857	5.473734	9.401979	3.058113	14.783527	2.528912	38.725886	7.19648
VPS13B	0	7.715846	0	1.75348	23.495701	0.89687	0	7.884229
VPS13C	14.816082	15.222522	30.126272	6.986274	13.022549	5.953945	49.608665	17.224358
VPS13D	11.970837	15.444294	15.095601	27.828146	27.196501	33.752628	69.972799	23.411287
VPS16	11.237297	4.626568	11.988978	7.827192	7.400579	11.235124	6.086766	9.148479
VPS18	17.293345	21.826172	10.726556	21.953442	40.533377	34.925942	19.736443	33.483573
VPS25	62.731876	50.726344	49.298627	72.455702	76.929939	45.618617	44.041073	36.25125
VPS25P1	0	0	0	0.450568	0	0	0	0
VPS26A	88.062113	121.788831	124.437128	57.528516	59.6924	50.478517	72.320198	92.221272
VPS26AP1	0	0	0	0	0	0	0	0
VPS26B	14.502505	28.633556	8.943998	27.471547	26.325982	67.527147	17.842323	67.143722
VPS26BP1	0	0	0	0	0	0	0	0
VPS26C	19.427361	80.839035	45.871988	76.006315	50.383325	83.525195	29.328394	103.954932
VPS26CP1	0	0	0	0	0	0	0	0
VPS28	56.272096	66.32744	36.324832	78.576333	22.299306	88.640276	112.174428	67.21035
VPS29	146.763744	147.012844	142.193043	104.123615	221.060742	119.758932	101.168211	194.862052
VPS33A	0	4.184651	7.563299	10.601906	0	5.98744	0	15.552737
VPS33B	9.3135	2.912824	0	10.140979	40.861999	9.713938	9.147662	14.944285
VPS35	172.685897	164.307725	122.998807	104.750504	137.768366	62.976754	162.621396	104.534454
VPS35L	0	3.976397	22.491055	1.971618	1.265492	8.130757	84.457157	0.726276
VPS35P1	0	0	0	0	0	0	0	0
VPS36	5.201955	17.430385	22.231154	19.19547	11.580324	13.880913	42.852943	20.442058
VPS37A	38.508423	43.214251	18.470477	29.552837	48.965049	30.72603	0	39.071233
VPS37B	23.741992	41.364898	20.137523	47.509152	21.762085	74.43919	78.782201	48.212123
VPS37C	7.596859	17.22853	11.334752	17.834138	40.773408	35.621179	18.133645	32.008371

VPS37D	0	0.587558	0	0.479005	0	1.402532	0	0.44436
VPS39	26.936829	23.337333	10.913284	23.551396	28.199208	23.767879	28.412176	15.7131
VPS41	124.346768	46.882358	58.42393	50.245999	60.661255	60.077246	31.972453	64.936411
VPS45	8.819071	15.684106	15.77178	12.112837	7.110253	6.884332	0	14.566303
VPS4A	19.06702	64.080189	23.419225	51.578949	20.777712	99.812172	34.988224	88.986442
VPS4B	57.369497	95.277038	29.667665	80.106336	165.226607	118.215667	154.129493	136.334658
VPS50	9.239005	26.028942	12.983001	6.473711	20.787307	6.028506	44.761953	13.073567
VPS51	10.178209	31.681028	91.074276	32.279944	69.86691	81.695333	87.797885	43.300677
VPS51P1	0	0	0	0	0	0	0	0
VPS51P10	0	0	0	0	0	0	0	0
VPS51P11	0	0	0	0	0	0	0	0
VPS51P12	0	0	0	0	0	0	0	0
VPS51P13	0	0	0	0	0	0	0	0
VPS51P14	0	0	0	0	0	0	0	0
VPS51P15	0	0	0	0	0	0	0	0
VPS51P16	0	0	0	0	0	0	0	0
VPS51P17	0	0	0	0	0	0	0	0
VPS51P18	0	0	0	0	0	0	0	0
VPS51P2	0	0	0	0	0	0	0	0
VPS51P3	0	0	0	0	0	0	0	0
VPS51P4	0	0	0	0	0	0	0	0
VPS51P5	0	0	0	0	0	0	0	0
VPS51P6	0	0	0	0	0	0	0	0
VPS51P7	0	0	0	0	0	0	0	0
VPS51P8	0	0	0	0	0	0	0	0
VPS51P9	0	0	0	0	0	0	0	0
VPS52	19.563625	21.243254	21.835739	19.396957	22.958049	22.744195	75.27304	19.613908
VPS53	5.998292	8.342004	3.889532	5.147269	93.385766	11.609528	2.438874	11.687417
VPS54	32.181256	32.107593	19.323688	11.987621	21.744815	9.252133	14.594607	25.288753
VPS72	0	26.252198	22.744643	17.660678	27.305649	35.210131	56.26135	10.906444
VPS8	70.244191	30.644487	15.250349	17.549446	19.434328	15.080113	86.114182	24.784721
VPS9D1	0	4.052206	1.139382	3.595169	0.335839	9.325624	0	9.617799
VRK1	53.699706	58.524797	39.859168	29.556088	31.140061	16.533605	23.124522	46.160578

VRK2	22.428377	4.193163	26.608154	3.06886	9.458914	2.299555	16.838156	3.679613
VRK3	45.021779	54.295634	11.271161	59.568263	15.666525	67.972635	47.893524	54.364846
VRTN	0	0	0	0	0	0	0	0
VSIG1	0	1.562167	0	5.720386	0	2.448138	0	3.330658
VSIG10	26.012946	45.656088	24.216345	40.607651	11.879358	36.968759	14.478921	43.350406
VSIG10L	1.491405	3.329429	1.333431	3.640999	0	2.136449	0.361573	2.103483
VSIG10L2	0	0	0	0	0	0	0	0
VSIG2	0	0	0	0.232481	0	0.156653	0	0.13085
VSIG4	0	0	0	0	0	0	0	0
VSIG8	0	0	0	0.21006	0	0	0	0
VSIR	5.565847	25.621812	7.498228	31.176829	8.632481	16.809472	1.863717	12.115695
VSNL1	8.021462	8.672564	0	10.743172	0	11.536097	0	15.49153
VSTM1	0	1.089629	0	0	0	0	0	0.449476
VSTM2A	0	0	0	0	0	0	0	0
VSTM2L	0	0	0	0.130994	0	0.533544	0	0.711655
VSTM4	0	0	0	0	0	0.12938	0	0.06085
VSTM5	0	0	0	0.178988	0	3.220677	0	0
VSX1	0	0	0	0	0	0	0	0
VSX2	0	0	0	0.108414	0	0.110796	0	0
VTA1	60.941537	69.247008	52.115838	46.197107	43.909798	38.223821	67.497648	68.964618
VTA1P1	0	0	0	0	0	0	0	0
VTA1P2	0	0	0	0	0	0	0	0
VTCN1	0	0.22379	1.338455	0.192038	0	0.148524	0	0.120659
VTCN1P1	0	0	0	0	0	0	0	0
VTI1A	6.278945	20.139268	2.628138	17.776022	9.65351	24.908966	0	11.920878
VTI1B	110.695099	159.042766	104.406736	132.717432	61.430586	131.732847	102.159282	173.794048
VTI1BP1	0	0	0	0	0	0	0	0
VTI1BP2	0	0	0	0	0	0	0	0
VTI1BP3	0	0	0	0	0	0	0	0
VTI1BP4	0	0	0	0	0	0	0	0
VTN	0	0	0	0	0	0	0	0
VWA1	3.6579	14.66452	4.385731	14.872107	39.255261	43.056194	67.378485	22.14372
VWA2	2.783219	1.581264	1.167464	5.416878	2.647281	1.227719	0	1.302523

VWA3A	0	0	0	0	0	0	0	0.059002
VWA3B	0	0	0	0	0	0.089374	0	0
VWA5A	0	0	0	0.339924	2.758128	1.36557	0	2.151563
VWA5B1	0	0	0	0.559043	0	0	0	0
VWA5B2	0	0	0	0.078204	0	0.100532	0	0
VWA7	0	0.448385	0	0.623788	0	0.61084	0	0.57818
VWA8	9.01791	10.490744	13.441631	7.324227	5.957735	3.235901	85.011052	5.517801
VWA8P1	0	0	0	0	0	0	0	0
VWC2	0	0.024414	0	0	0	0	0	0
VWC2L	0	0	0	0	0	0	0	0
VWCE	0	0.316068	0	0.482609	0	0	0	0
VWDE	9.868772	2.431882	2.462636	2.349901	2.105515	0.93618	0	0.196059
VWF	0	0.603603	0	0.249056	0	0	0	0
VWFP1	0	0.137856	0	0	0	0	0	0
VXN	0	1.041682	0	0	0	0	0	0.928964
WAC	25.355545	30.278534	2.575813	28.824811	35.788295	33.208805	18.737198	22.784356
WAPL	36.528422	69.134526	18.632266	49.002105	3.017704	36.767276	41.972831	55.85798
WARS1	214.532216	340.974552	192.5044	456.852193	136.932372	315.35168	224.398826	264.648996
WARS1P1	0	0	0	0	0	0	0	0.490686
WARS2	0	8.995655	0	6.409443	0	6.760304	33.671016	4.462968
WARS2P1	0	0	0	0	0	0	0	0
WAS	0	2.260623	0	0	0	0.405111	0	0
WASF1	9.654043	14.320139	6.795431	10.490595	14.856726	32.983044	19.961822	33.558163
WASF1P1	0	0	0	0	0	0	0	0
WASF2	49.308183	68.061473	61.835851	90.461259	95.09571	123.377479	161.885172	117.996002
WASF3	3.31385	3.298851	2.564325	4.303983	0	2.790906	7.894685	8.139604
WASF4P	0	0.108026	0	0	0.67158	0	0	0
WASF5P	0	0	0	0	0	0	0	0
WASH2P	14.74454	2.335125	12.201085	1.793245	0	1.950047	1.920529	1.045741
WASH3P	0	0	0	0	0	0	0	0
WASH4P	0	0	0	0.383234	0	0	0	2.087012
WASH5P	0	0	0	0	0	0	0	0
WASH6P	8.973927	8.172652	12.564545	14.094254	16.587468	19.958574	0	15.127101

WASH8P	0	4.750876	17.442304	9.391628	7.462461	13.544085	0	6.913806
WASH9P	0	3.822212	0	4.272404	11.665373	5.923068	8.271555	5.598963
WASHC1	5.447092	2.685975	11.644578	3.627826	7.882995	3.464063	0	2.961371
WASHC2A	21.939468	24.396059	24.144502	29.936294	37.637474	37.105377	20.000272	32.39591
WASHC2C	22.696138	8.668405	0	22.503798	0	14.949543	14.546381	13.765801
WASHC3	48.623849	21.275896	31.186672	3.546276	16.174929	13.664657	21.397437	21.949155
WASHC3P1	0	0	0	0	0	0	0	0
WASHC4	12.831265	13.947085	8.658295	11.574292	0	13.540525	124.345706	24.467522
WASHC5	40.640358	73.704744	35.048525	44.859145	102.172262	52.953168	101.710757	69.969812
WASL	21.870658	37.159808	45.365994	38.707648	14.142086	16.521453	3.488352	27.217736
WBP1	6.765987	16.276292	24.358594	27.174328	14.03035	15.306396	0	10.98129
WBP11	71.406579	178.97413	57.132869	146.636847	78.09661	180.204412	162.000667	214.172472
WBP11P1	0	0	0	0	0	0	0	0
WBP11P2	0	0	0	0	0	0.118784	0	0
WBP11P3	0	0	0	0	0	0	0	0
WBP1L	39.767384	69.892475	22.229503	58.298862	18.670225	54.695159	9.385096	44.653361
WBP1LP1	0	0	0	0	0	0	0	0
WBP1LP10	0	0	0	0	0	0	0	0
WBP1LP11	0	0	0	0	0	0	0	0
WBP1LP12	0	0	0	0	0	0	0	0
WBP1LP2	0	0.478086	0	0.278544	0	0.748266	0	1.243988
WBP1LP3	0	0	0	0	0	0	0	0
WBP1LP4	0	0	0	0	0	0	0	0
WBP1LP5	0	0	0	0	0	0	0	0
WBP1LP6	0	0	0	0	0	0	0	0
WBP1LP7	0	0	0	0	0	0	0	0
WBP1LP8	0	0	0	0	0	0	0	0
WBP1P1	0	0	0	0	0	0	0	0
WBP1P2	0	0	0	0	0	0	0	0
WBP2	37.533872	52.14142	80.419467	78.843174	118.384239	64.224049	128.161266	35.201965
WBP2NL	0	2.104338	0	0.654665	0	1.197221	0	2.386186
WBP2P1	0	0	0	0	0	0	0	0
WBP4	4.289516	14.338007	15.345977	14.402693	20.744257	12.350443	0	11.347252

WDCP	13.762421	17.649244	14.664206	20.465782	6.586867	13.020924	17.661154	12.319304
WDFY1	26.364729	18.28363	21.756801	22.93791	35.760199	23.987764	101.337835	23.590652
WDFY2	1.715734	2.264574	1.852846	2.171578	2.720966	1.491467	0	1.911949
WDFY3	37.408084	16.991101	19.450091	19.449142	11.249883	30.948891	0	20.550313
WDFY4	0	0	0	0	0	0	0	0
WDHD1	55.211669	115.34302	56.931799	81.339282	77.599642	48.787303	58.232228	104.898375
WDPCP	8.021462	10.155374	4.038185	9.248275	20.819033	5.03174	0	17.438013
WDR1	109.948697	191.905898	103.152439	236.014186	88.834334	273.130925	88.313099	256.539955
WDR11	47.771645	45.902706	34.226642	55.744515	41.051285	24.381089	83.559333	42.962053
WDR12	89.759465	87.023168	82.714171	55.238755	76.598626	61.798489	13.68019	109.294592
WDR13	7.655102	61.276593	45.499584	62.340417	12.410776	59.784443	0	62.784926
WDR17	3.300833	2.720193	3.179222	1.337266	0.184137	0.252772	0	1.858313
WDR18	22.377328	8.013462	60.56755	4.342884	23.918111	0.416578	145.610599	20.050132
WDR19	6.202467	4.41458	3.359907	6.903716	3.928548	1.307213	0	2.143615
WDR20	25.851505	27.045342	17.263805	16.333764	29.311761	19.305026	10.94346	20.181239
WDR24	3.090741	15.489919	13.852977	16.309037	9.075906	36.193381	3.263696	27.208155
WDR25	8.043726	5.037558	0	9.898999	4.239053	5.974073	11.666567	4.786214
WDR26	8.867644	59.794182	52.858922	54.835427	22.191153	32.026978	16.801548	55.397863
WDR27	8.160428	23.099635	23.87813	23.787753	4.247919	9.584749	0	16.758781
WDR3	41.99224	109.243509	40.946059	130.904539	72.428926	103.985265	31.209498	102.33664
WDR31	4.716051	14.041293	6.644039	17.767829	5.217477	10.158017	1.743008	6.27163
WDR33	20.901999	46.609878	26.069919	46.026608	27.633084	43.91139	56.297737	70.270045
WDR35	0	4.875453	0	6.069097	4.569052	2.605205	0	3.741095
WDR36	0	0	0	2.916111	14.180412	5.314632	0	0
WDR37	0	0.168852	0	0	0	0	0	1.171831
WDR38	0	0	0	0	0	0	0	0
WDR4	0	6.381722	5.707561	8.827124	42.387407	10.469809	20.335959	20.729723
WDR41	57.0223	41.771536	81.615018	37.475361	20.692934	23.816087	59.931489	50.021401
WDR43	67.106593	227.281006	67.214732	189.252732	174.085024	133.012094	70.133554	203.880488
WDR44	15.474774	30.834287	16.555513	25.951679	4.77626	15.451347	16.975859	20.533741
WDR45	45.726914	47.400642	32.800058	69.817196	35.543514	89.892845	60.009347	50.399323
WDR45B	85.872686	152.101257	110.359346	116.04086	130.809727	118.799277	85.845404	164.893319
WDR45BP1	0	0	0	0	0	0	0	0

WDR45P1	0	0	0	0	0	0	0	0
WDR46	52.715524	43.781032	24.630278	49.936199	64.305207	73.583438	69.892643	69.004552
WDR47	0	17.950612	9.068087	7.051911	8.892683	8.47432	3.122028	6.068011
WDR48	18.921224	11.237242	12.716566	9.075079	18.022472	8.810434	3.448121	12.869129
WDR49	0	1.592158	0	0.082015	0	0	0	0
WDR4P1	0	0	0	0	0	0	0	0
WDR4P2	0	0	0	0	0	0	0	0
WDR5	0	0	0	0.168545	0	9.606411	39.722956	0
WDR53	24.823059	69.646522	33.770835	86.06964	58.387936	45.897404	21.0587	51.492392
WDR53P1	0	0	0	0	0	0	0	0
WDR54	45.803412	49.588649	49.230829	61.036219	50.744966	33.42157	23.43359	31.300474
WDR55	3.012545	5.550162	5.521469	8.58297	1.577118	11.495003	0	4.735766
WDR59	34.394878	76.265266	39.210656	94.200188	106.03884	114.700359	112.000468	75.048748
WDR6	28.385615	51.845811	21.761489	53.871189	81.949599	52.841238	76.362041	38.08436
WDR62	3.142064	3.095794	1.521023	2.047453	6.413643	9.685773	0	8.98454
WDR64	0	0	0	0	0.596413	0	0	0
WDR7	47.741781	15.570498	5.242647	7.633701	45.307263	8.159633	52.002931	10.372996
WDR70	0	46.05557	0	36.160604	0	26.775458	131.977346	37.273244
WDR70P1	0	0	0	0	0	0	0	0
WDR72	0	0	0	0.022498	0	0	0	0
WDR73	22.660991	29.844085	13.059977	31.019344	22.667758	31.160304	6.440555	55.713939
WDR74	1261.44562	89.990843	1184.53386	29.337923	1748.99035	56.073779	2140.91045	83.133062
WDR75	38.385201	25.008078	26.343088	15.719342	40.512847	8.751949	20.187082	32.638809
WDR76	45.00899	47.535947	42.725042	48.484683	123.898743	25.917217	14.96471	50.607568
WDR77	56.792704	79.035183	53.357156	40.131306	47.563546	22.182004	49.46124	31.678626
WDR77P1	0	0	0	0	0	0	0	0
WDR81	1.022703	2.419032	0	3.435556	10.197348	6.75569	32.020655	3.443581
WDR82	49.940528	106.529107	55.180621	78.698846	44.033114	62.793615	30.145416	106.218359
WDR82P1	0	0	0	0	0	0	0	0
WDR82P2	0	0	0	0	0	0	0	0
WDR83	15.270599	11.344235	9.402661	13.785498	8.688935	29.87101	20.985778	18.289623
WDR83OS	57.86936	37.169022	87.699722	45.501788	209.504284	89.61563	107.076938	83.146112
WDR86	0	0	0	0	0	0	0	0

WDR87	0	0	0	0	0	0	0	0
WDR87BP	0	0	0	0	0	0	0	0
WDR88	0	0.267868	0	0.046306	0	0	0	0
WDR89	0	17.550203	25.459325	16.040079	17.945806	7.063577	23.776233	27.109654
WDR90	9.332648	2.557942	20.115226	4.172742	30.482795	5.87675	68.352693	4.531494
WDR91	0	1.910149	5.802864	3.032393	2.503541	3.35468	0	4.337407
WDR93	0	0.234714	0	0.539202	0	0	0	0.31252
WDR95P	0	0	0	0	0	0	0	0
WDR97	0	0.077776	0	0.225057	0	0.036608	0	0.091975
WDSUB1	2.225313	5.810345	12.45248	0	4.913679	5.559063	29.827868	1.68251
WDTC1	3.851907	12.852479	7.511439	19.099811	15.365546	34.575449	0	25.869702
WEE1	17.444006	28.184427	19.930415	27.129603	62.368665	26.121605	32.995915	51.372834
WEE1P1	0	0	0	0	0	0	0	0
WEE1P2	0	0	0	0	0	0	0	0
WEE2	1.095412	0	0	0.106804	0	0	0	0
WEE2P1	0	0	0	0	0	0	0	0
WFDC1	0	0	0	0	0	0	0	0
WFDC10A	0	0	0	0	0	0	0	0
WFDC10B	0	0	0	0	0	0.485918	0	1.363801
WFDC11	0	0	0	0	0	0	0	0
WFDC12	0	0	0	0	0	0	0	0
WFDC13	0	0	0	0	0	0	0	0
WFDC2	82.084981	86.902599	162.104018	172.671395	8.550896	14.698472	0	6.166657
WFDC21P	0	0	0	1.353019	0	17.868511	0	0
WFDC3	0	4.70239	0	1.937433	0	1.802436	0	2.413951
WFDC5	0	0	0	0	0	0	0	0
WFDC8	0	0	0	0	0	0	0	0
WFIKKN1	0	0.05763	0.603106	0.131251	0	0.336132	0	0.107771
WFIKKN2	0	0	0	0	0	0	0	0
WFS1	7.183553	7.831688	5.726036	8.146271	1.357408	5.835488	4.240597	5.982404
WHAMM	6.409768	12.673259	2.303423	9.320949	26.606108	12.073947	0	7.173692
WHAMMP1	0	0	0	1.246679	0	0	0	0
WHAMMP2	0	0	0	0.318303	5.760895	0.181191	0	0

WHAMMP3	0	0.618477	0	1.333141	0	1.46928	0	0.905581
WHAMMP4	0	0.305452	0	0	0	0.528625	0	2.854191
WHRN	10.650835	12.159352	5.823333	10.124215	11.142767	25.680013	8.622135	18.358802
WHSC1L2P	1.625212	0.483083	1.451964	0.276842	0	0	0	0.065665
WIF1	0	1.463515	0	0.341413	0	0	0	0
WIPF1	1.10812	4.685144	1.928402	2.616753	0	0.894698	0	0.594698
WIPF2	22.426371	50.45478	32.384465	58.238954	69.813807	62.759859	19.038332	43.063126
WIPF3	0	0	0	0	0	0	0	0
WIPI1	32.78849	17.317123	26.098009	16.980185	0	10.14264	0	4.9036
WIPI2	102.083641	106.331128	60.974206	146.126551	64.217774	127.812563	62.710164	115.372438
WIZ	0	1.077995	3.385828	3.669844	2.720185	16.945421	0	11.123248
WIZP1	0	0	0	0	0	0	0	0
WLS	60.553635	219.405599	103.00102	287.394119	120.258548	192.27652	11.090685	160.670059
WNK1	66.701593	146.196652	135.402496	120.873122	97.583089	125.635653	82.403234	129.407688
WNK2	3.882058	2.813782	0	4.571457	11.528169	6.593103	0.368313	6.092436
WNK3	5.587292	3.268942	0	0.563675	0	0	0	1.467712
WNK4	3.924813	1.11026	5.637086	2.588868	0.207308	1.018903	9.051794	0.339812
WNT10A	10.105596	10.575439	6.016881	15.569949	50.176089	40.474222	30.183694	27.140541
WNT10B	3.056571	9.759872	10.928954	10.664741	2.418382	4.319685	0	2.895077
WNT11	1.836364	0.77615	0	2.139403	4.840391	0.272254	0	0.444793
WNT16	0	0.501573	0.952311	0.440382	0	0	0	0
WNT2	0	0	0	0	0	0.435477	0	0
WNT2B	0	1.140931	1.856979	2.137412	0.315622	1.573585	0	0.949115
WNT3	1.01564	10.196621	5.463081	7.980284	2.413034	9.572953	6.419126	7.267277
WNT3A	0	0.812827	1.004125	0.60091	3.253792	0.614087	0	0.180305
WNT4	0	0.756571	1.494886	2.097864	5.278614	13.598301	0	5.589163
WNT5A	0.538021	0	0.483643	1.786044	3.163544	0.708109	0	2.19964
WNT5B	0	3.775503	0	0.989087	0	3.985474	0	5.967658
WNT6	2.052842	0.173042	0	0.746446	1.62265	0	0	0
WNT7A	0	0	0	0	0	0	51.810895	0
WNT7B	19.257939	70.005302	26.298836	67.071828	32.060802	154.252948	42.785881	120.895223
WNT8A	0	0	0	0	0	0	0	0
WNT9A	0	0.211956	0	0.382595	0.217816	3.256493	0	2.119929

WNT9B	0	0	0	0	0	0.032958	0	0
WRAP53	7.946143	5.779174	6.879866	1.830275	13.359651	2.194187	10.305761	5.485215
WRAP73	17.156445	14.937508	5.585833	16.413037	5.505276	30.852401	7.990959	31.004139
WRN	8.404976	9.545714	6.560741	3.125566	3.747322	1.825101	33.166521	4.528697
WRNIP1	29.380946	49.629458	19.439642	51.18608	27.294323	71.061198	38.224326	68.461191
WSB1	52.725289	89.956006	27.830292	81.215153	35.068821	52.054816	57.442218	96.530612
WSB2	0	26.880758	21.740423	36.542757	87.418479	71.262209	122.59015	35.40601
WSCD1	0	0	0	1.269185	0	0.669467	0	0
WSCD2	0	0	0	0	0	0.034851	0	0.787579
WT1	9.861247	2.333471	0	0	0	0	0	1.669537
WTAP	78.775612	182.671052	85.050208	142.571923	101.826089	159.239031	78.785223	231.851043
WTAPP1	0	2.261149	0	4.660501	0	1.53449	0	0.309659
WTAPP2	0	0	0	0	0	0	22.23392	0
WTIP	0	0.383626	0	1.664613	13.584347	2.515877	0	3.984399
WWC1	42.248594	103.171666	47.329862	129.569314	84.296237	197.414949	122.155297	142.727086
WWC2	13.364609	35.03706	21.151627	36.83513	14.218827	37.518672	7.73287	42.494147
WWC3	0	2.101288	0	0	0	0	0	0
WWOX	10.723997	38.731116	12.324694	48.984117	39.664655	54.381629	34.714919	47.144447
WWP1	63.744651	101.764983	92.637277	85.82175	16.362144	53.275855	32.804064	86.72565
WWP1P1	0	0	0	0	0	0	0	0
WWP2	50.320169	18.169788	2.311505	28.451281	43.597628	50.792851	26.073603	27.362847
WWTR1	45.171786	25.416432	17.316509	34.733652	28.891295	32.386992	15.404306	31.353991
XAB2	11.51556	7.789741	15.476824	13.239412	14.85471	12.600195	4.969128	11.692611
XAF1	10.376724	1.833381	3.496435	1.813219	31.324753	21.115732	37.130476	21.168698
XAGE1A	0	0	0	0	0	0	0	0
XAGE1B	0	0	0	0	0	0	0	0
XAGE2	0	0	0	0	0	0	0	0
XAGE3	0	0	0	0	0	0	0	0
XAGE5	0	0	0	0	0	0	0	0
XBP1	0	18.479781	77.435247	26.791643	9.136978	13.088261	32.846322	20.71183
XBP1P1	0	0	0	0	0	0	0	0
XCL1	0	0	0	0	0	0	0	0
XCL2	0	0	0	0	0	0	0	0

XDH	18.732189	8.75264	6.657841	5.926369	10.990191	9.044065	1.249799	12.900623
XG	0	0	0	0	5.619348	4.595517	0	8.210548
XGY1	0	0	0	0	0	0	0	0
XIAP	0	96.543311	18.557334	66.496407	0	41.781517	0	99.244485
XIAPP1	0	0	0	0	0	0	0	0
XIAPP2	0	0	0	0	0	0	0	0
XIAPP3	0	0	0	0	0	0	0	0
XIRP1	0	0	0	0	0	0	0	0
XIRP2	0	0	0	0	0	0	0	0
XK	0.631601	0.62376	1.134925	0.463106	0.834262	1.07554	0	2.432591
XKR3	0	0	0	0	0	0	0	0
XKR4	0	0	0	0	0	0	0	0
XKR5	0	0	0	0.032764	0	0	0	0
XKR6	0	0.930845	0	0.545608	0	0.274131	0	0.331871
XKR7	0	0	0	0	0	0.084266	0	0
XKR9	1.951179	8.472952	0	5.161569	0.514194	0.481517	0	1.641589
XKRX	1.179441	2.452036	0	0.524666	0	0.058712	0	0.496336
XKRY	0	0	0	0	0	0	0	0
XKRYP1	0	0	0	0	0	0	0	0
XKRYP3	0	0	0	0	0	0	0	0
XKRYP5	0	0	0	0	0	0	0	0
XKRYP6	0	0	0	0	0	0	0	0
XKRYP7	0	0	0	0	0	0	0	0
XNDC1N	14.899777	6.956136	23.91145	18.912472	5.783401	9.510231	26.770348	9.976162
XPA	0	0.562548	0	1.585844	0	0	45.02302	0.57329
XPC	0	19.30757	0	39.459777	18.286961	17.539044	22.845278	10.328622
XPNPEP1	31.849573	42.747856	56.946205	46.150131	99.887811	50.203568	43.238636	39.317325
XPNPEP2	0	0	0	0	0	0	0	0
XPNPEP3	91.880925	26.454608	24.225262	18.61807	23.132698	12.905162	23.318978	9.850364
XPO1	77.159853	66.488518	37.484608	121.380753	2.176564	115.071142	127.086727	118.440042
XPO4	0	3.329083	0	0.769061	0	1.57415	0	3.783578
XPO5	33.730916	44.313842	26.451269	30.568709	41.153213	37.757628	126.143557	43.265897
XPO6	27.041541	109.182601	45.492343	49.63194	13.694925	78.703543	45.052364	110.366061

XPO7	76.178564	66.081238	49.674592	68.633895	97.426011	35.67403	23.261066	58.087457
XPOT	133.166603	427.973008	185.013034	348.406827	207.217787	226.700805	125.119862	444.217832
XPOTP1	0	0	0	0.10411	0	0	0	0.23111
XPR1	44.186028	37.96867	52.076954	42.136159	18.020563	12.56755	0	15.652351
XRCC1	15.669142	30.202123	35.965584	36.602845	37.883255	57.360434	39.620712	18.685901
XRCC2	8.93212	14.298208	4.936949	6.765396	9.526746	5.571368	13.343804	12.000228
XRCC3	35.760995	52.771561	4.78988	47.62875	34.847507	60.269043	0.324704	47.708727
XRCC4	17.851219	8.902281	0	8.812825	12.347875	7.645165	28.899217	13.456598
XRCC5	199.252509	169.18634	147.659395	139.611747	392.109824	153.521167	247.01532	272.135791
XRCC6	31.961878	150.420533	0	75.281592	89.358319	289.178964	0	52.681522
XRCC6P1	0	0	0	0	0	0	0	0
XRCC6P2	0	0	0	0	0	0.096203	0.465269	0
XRCC6P3	0	0	0	0	0	0	0	0
XRCC6P4	0	0	0	0	0	0	0	0
XRCC6P5	0	0	0	0	0	0	0	0
XRN1	6.912648	14.540477	23.527961	8.199946	45.603774	6.027808	4.86529	6.761501
XRN2	84.06732	84.430398	82.394286	63.051436	93.421962	52.148366	105.290314	90.808389
XRRA1	39.309098	73.432591	9.306202	96.767912	12.126235	88.733754	74.523992	59.48869
XXYLT1	7.473768	8.432221	7.164652	6.076761	22.931463	23.783855	0	24.059436
XYLB	6.901322	0.159459	3.42584	1.786793	3.558068	1.692014	5.689072	1.45846
XYLT1	0	0	0	0	0	0	0	2.274309
XYLT2	10.22228	3.017475	9.163851	0.509047	16.907707	8.934923	0	0.442363
YAE1	18.340455	35.313784	28.201399	30.684294	23.76019	27.772507	39.951515	37.569722
YAF2	0	7.344794	34.535122	8.676898	9.917055	7.181412	0	4.199872
YAP1	388.435762	702.629208	335.277164	639.559597	123.498025	245.228688	59.903318	211.871391
YAP1P1	0	0	0	0	0	0	0	0
YAP1P2	0	0	0	0	0	0	0	0
YAP1P3	0	0	0	0	0	0	0	0
YARS1	0	19.117342	18.308871	50.697097	0	61.077458	13.023521	24.690989
YARS2	0	1.967628	39.565085	4.181599	71.181851	3.34137	127.494285	2.738104
YBEY	34.232327	44.795619	26.724639	34.990659	51.153966	19.761223	18.076752	39.318824
YBX1	891.874655	1557.29107	768.900545	1244.35536	1446.74793	2773.94262	1317.29029	2226.50776
YBX1P1	0	0	0	0.757895	1.221304	1.865517	0	0

YBX1P10	0	0.171624	0	0.408266	0	0.602483	0	1.061928
YBX1P2	0	0.162197	0	0	0	0	0	0.319259
YBX1P3	0	0	0	0	0	0	0	0
YBX1P4	0	0.204787	0	0	0	0	0	0
YBX1P5	0	0	0	0	0	0	0	0.194707
YBX1P6	0	0	0	0.092404	0	0.186201	0	0
YBX1P7	0	0	0	0	0	0	0	0
YBX1P8	0	0	0	0	0	0	0	0
YBX1P9	0	0	0	0	0	0	0	0
YBX2	0	0.270718	0	0.247096	0	0	0.51779	0
YBX2P1	0	0	0	0	0	0	0	0
YBX2P2	0	0	0	0	0	0	0	0
YBX3	210.422878	378.592569	301.429371	449.968196	432.629906	695.965947	316.373157	511.395234
YBX3P1	0	0	0	0	0	0	0	0
YDJC	10.172444	23.138402	0	21.032761	26.10867	57.521069	39.637025	54.3866
YEATS2	59.254973	74.950106	62.110716	83.38797	38.476016	78.892255	47.388937	68.638059
YEATS4	48.92211	79.628439	32.02	66.82753	54.272785	124.38222	22.176856	127.352231
YES1	55.604841	35.279589	46.093983	56.330106	37.847616	43.232139	66.379203	67.472959
YES1P1	0	0	0	0	0	0	0	0
YIF1A	94.341359	154.635621	97.644527	136.151	46.250169	90.761685	138.154132	74.111598
YIF1B	9.186182	18.825476	27.921737	26.23668	37.234834	27.172312	0.995257	19.539729
YIPF1	53.217858	33.930213	56.037738	41.257045	28.958812	14.183141	10.948888	19.236857
YIPF2	10.975048	20.1667	23.670096	29.302191	7.783075	31.091978	29.034335	18.279657
YIPF3	33.343702	39.051318	28.789322	59.490365	31.275737	65.999727	58.476957	54.100678
YIPF4	22.494231	46.834911	35.147717	18.976096	19.515075	18.593282	2.67997	53.980278
YIPF5	58.95155	57.813568	60.156556	50.126437	68.557031	19.396938	44.610203	41.788832
YIPF6	71.088506	40.955045	47.536611	20.379781	16.866399	14.242347	37.528577	26.020692
YIPF7	0	0	0	0	0	0	0	0
YJEFN3	0	0	0	0.029514	0	0.120586	0	0.09751
YJU2	17.687609	11.431388	2.245986	10.184755	21.70968	16.110657	0	13.92222
YJU2B	29.048056	6.636641	5.377021	10.330876	7.051654	18.386979	0	13.552436
YKT6	35.970573	97.740488	43.700713	74.002661	132.029786	176.301435	123.195182	216.034068
YLPM1	59.758578	58.004411	52.774963	68.073833	53.467272	57.927006	76.413748	49.247312

YME1L1	3.17509	8.244407	0	7.99832	0.266628	4.442873	64.128181	8.532627
YME1L1P1	0	0	0	0	0	0	0	0
YOD1	0	0	0	0	0	0.380989	14.090491	0
YPEL1	0	1.445121	2.751685	2.096319	0	2.787723	0	1.107887
YPEL2	4.519507	0.959166	1.672336	2.601672	2.458499	2.402931	0	2.702353
YPEL3	37.511226	34.45057	30.233349	65.850965	10.539319	54.779511	22.964942	36.379977
YPEL4	0	0	0	1.166319	0	0	0	0.453338
YPEL5	38.748019	50.393799	58.622674	96.935376	80.738712	35.31563	40.851444	34.544224
YPEL5P1	0	0	0	0	0	0	0	0
YPEL5P2	0	0	0	0	0	0	0	0
YPEL5P3	0	0	0	0	0	0	0	0
YPEL5P4	0	0	0	0	0	0	0	0
YRDC	9.468681	13.676618	15.203671	9.649124	18.467023	13.842056	0	19.906373
YRDCP1	0	0	0	0	0	0	0	0
YRDCP2	0	0	0	0	0	0	0	0
YRDCP3	0	0	0	0	0	0	0	0
YTHDC1	27.419308	76.136301	32.045263	79.393771	75.16584	77.209315	69.620293	77.994968
YTHDC2	12.357721	8.305909	7.869718	8.583621	11.700633	12.31898	9.037516	16.828356
YTHDF1	46.007584	173.509688	63.732098	171.867541	41.126328	185.168727	47.269284	205.142862
YTHDF1P1	0	0	0	0	0	0	0	0
YTHDF2	49.726859	71.480013	42.608932	80.786308	59.44683	93.461481	117.103308	126.527733
YTHDF2P1	0	0	0	0	0	0	0	0
YTHDF3	38.505439	106.784323	43.699441	100.410394	39.279671	88.217714	19.272695	100.762945
YTHDF3P1	0	0	0	0	0	0	0	0
YWHAB	348.268927	994.245561	248.449244	693.64254	219.720419	786.51069	217.812257	933.004289
YWHABP1	0	0	0	0	0	0	0	0
YWHABP2	0	0	0	0	0	0	0	0.981927
YWHAE	524.269985	1067.61454	455.672871	1026.47699	628.931608	1592.70454	746.601118	1394.968
YWHAEP1	0	0.219608	0	0.129333	0	0.258535	0	0
YWHAEP2	0	0	0	0	0	0	0	0
YWHAEP3	0	0	0	0	0	0	0	0
YWHAEP4	0	0	0	0	0	0	0	0
YWHAEP5	0	0	0	0	0	0	0	0.891693

YWHAEP7	0	0	0	0	0	0	0	0
YWHAG	162.009665	187.774229	155.778664	164.693951	174.18381	176.523819	167.435068	227.56475
YWHAH	117.573752	426.646885	82.18919	350.959325	105.289056	391.182809	176.280154	527.764715
YWHAQ	372.76918	801.404418	322.127743	697.819715	391.682306	689.596649	509.825346	848.014472
YWHAQP1	0	0	0	0	0	0	0	0
YWHAQP10	0	0	0	0	0	0	0	0
YWHAQP4	0	0	0	0	0	0	0	0
YWHAQP5	0	0	0	0	0	0	0	0
YWHAQP6	0	0	0	0	0	0	0	0
YWHAQP7	0	0.792719	0	0.31375	0	0	0	0
YWHAQP8	0	0	0	0	0	0	0	0
YWHAQP9	0	0	0	0	0	0	0	0
YWHAZ	1102.13867	1265.77013	948.78458	1162.52857	1012.64599	774.637233	997.678015	1049.78417
YWHAZP1	0	0	0	0	0	0	0	0
YWHAZP10	0	0	0	0	0	0.282642	0	0
YWHAZP2	0	0	0	0	0	0	0	0
YWHAZP3	0	0.531136	0	0	0	0	0	0
YWHAZP4	0	0	0	0.142432	0	0	0	0.24797
YWHAZP5	0	0.95536	0	0	0	0.550912	0	0.239071
YWHAZP6	0	0	0	0.135016	0	0	0	0
YWHAZP7	0	0	0	0	0	0	0	0
YWHAZP8	0	0	0	0	0	0	0	0
YY1	68.426767	224.946592	60.412867	164.880077	146.785217	217.712736	74.839006	215.362779
YY1AP1	0	62.466821	60.202725	63.513174	19.40305	80.47668	25.691691	58.697157
YY1P1	0	0	0	0	0	0	0	0
YY1P2	0	0	0	0	0	0	0	0
ZACN	2.846244	1.035031	0	0.311051	0	1.273683	0	0
ZACNP1	0	0	0	0	0	0	0	0
ZAN	0	0	0	0	0	0	0	0
ZAP70	0	0	0	0	0	0	0	0
ZAR1	0	0.310554	0	0.179153	0	0.121189	0	0
ZAR1L	0	0	0	0	0	0	0	0
ZBBX	0	1.897335	0	0.115241	0	0	0	0

ZBED1	8.036188	25.967148	10.251197	21.303287	23.786297	90.722788	63.080357	69.777889
ZBED1P1	0	0	0	0	0	0	0	0
ZBED2	34.15218	114.367147	12.487146	77.522036	50.35593	246.69276	31.602877	284.968647
ZBED3	2.648511	3.553584	2.857127	4.003173	2.939158	3.744992	30.525021	4.226814
ZBED4	13.623612	37.617595	18.16592	31.921152	10.551963	38.353229	15.464226	41.768678
ZBED5	21.404061	46.733639	12.398998	47.231332	6.644505	74.114283	62.314448	68.338359
ZBP1	0	0	0	0	0	0.412975	0	0.067286
ZBTB1	60.769456	51.86399	24.025602	32.579191	23.783218	22.767296	50.737409	69.388627
ZBTB10	2.938821	0.996908	2.926239	1.889358	1.890672	0.654312	0	0.886696
ZBTB11	1.631943	0	0	0.113589	0.72078	1.358091	2.746909	0.767898
ZBTB12	1.863506	1.41722	2.493922	0	3.683766	0	0	0
ZBTB12BP	0	0	0	0	0	0	0	0
ZBTB14	5.98962	26.36492	27.257172	24.247649	5.270757	27.53548	0	23.232621
ZBTB16	0	0	0	0	0	0	0	0
ZBTB17	11.772293	11.097673	16.090369	2.722626	1.786427	17.548423	40.850317	5.958392
ZBTB18	6.203905	11.549683	8.746321	8.440491	1.404117	8.206377	6.482255	7.725915
ZBTB2	15.115135	23.348797	19.350967	20.84485	8.549384	31.369425	36.729766	36.69402
ZBTB20	0.233985	1.375019	15.530888	2.677199	0	1.570419	0	1.780005
ZBTB21	5.810669	24.973706	12.99346	36.216395	9.700795	29.356259	42.915015	42.222838
ZBTB22	7.683278	14.145723	12.578592	12.267616	4.73033	26.522242	26.730609	15.020418
ZBTB25	0	43.522286	8.524862	39.389871	22.736076	25.746493	12.701768	46.496689
ZBTB26	7.379171	14.967774	9.938726	6.893751	1.1694	3.19175	8.982975	9.826781
ZBTB2P1	0	0	0	0	0	0	0	0
ZBTB3	0	0	0	0	0	3.252778	0	0.863536
ZBTB32	0	0.08576	0	0	0	0	0	0.082206
ZBTB33	31.347609	23.572852	45.250691	14.225569	22.52511	8.68051	20.161507	18.822868
ZBTB37	0	2.069956	0	1.216119	5.517538	0	0	0
ZBTB38	13.336119	58.041016	13.862569	54.837618	44.988592	161.623326	127.897923	340.843462
ZBTB39	7.770544	9.42323	6.520239	8.716687	3.14825	7.663708	1.894232	8.620067
ZBTB4	17.653942	20.804145	18.466725	25.683013	22.217792	43.923084	16.374977	30.735586
ZBTB40	5.006503	14.498405	9.669368	14.634456	5.868438	18.618413	1.972961	19.247467
ZBTB41	9.915644	18.850132	0	11.990102	5.042469	6.519555	0	15.470709
ZBTB42	0	6.921457	1.649302	4.820201	12.843375	17.242846	0	8.311237

ZBTB43	52.775391	28.368314	10.497706	38.009904	9.404259	26.739978	4.744062	36.881971
ZBTB44	30.496629	43.012943	56.129467	37.130064	82.083283	65.810295	35.241154	91.216727
ZBTB45	2.907154	6.331155	4.34258	5.666577	1.708928	13.496278	0	9.024302
ZBTB45P1	0	1.765161	0	1.677274	1.30434	2.644217	0	0
ZBTB45P2	0	0	0	0	0	1.867922	0	1.041477
ZBTB46	0	0	0	0.896778	0	0	0	1.025895
ZBTB47	0.593541	2.22271	1.240864	2.07491	0.156809	6.363157	2.169394	3.68158
ZBTB48	14.459653	7.591661	3.40204	5.506229	2.749535	10.90511	17.281141	7.897561
ZBTB49	1.435047	5.811771	0	6.268716	4.921	4.511559	2.15371	5.574529
ZBTB5	5.595489	6.271223	3.769001	5.570852	10.345974	10.326326	11.922932	10.610757
ZBTB6	19.320255	5.310987	16.618647	3.557626	10.204535	1.649868	13.322595	4.072996
ZBTB7A	21.673556	30.143015	26.284496	33.185638	22.641392	58.37964	38.092503	37.30949
ZBTB7B	7.350597	14.947151	14.921651	20.133996	10.736916	28.974893	12.664619	17.163774
ZBTB7C	0	0.22902	0	0.066472	0	0.20427	0	0.300221
ZBTB8A	4.115162	5.402443	15.394366	3.47719	7.09056	3.818525	1.573125	9.57867
ZBTB8B	0.249217	0.642201	0.252613	0.649413	0.065883	0.143355	0	0.479215
ZBTB8OS	48.325077	11.957804	34.656573	23.103057	0	14.988206	0	23.432391
ZBTB8OSP1	0	0	0	0	0	0	0	0
ZBTB8OSP2	0	0	0	0	0	0	0	0
ZBTB9	6.225679	8.108407	3.344405	7.763329	16.321986	12.45635	33.282052	6.955972
ZC2HC1A	3.417666	3.82026	4.519267	2.257636	5.256465	0.44652	0	0.928121
ZC2HC1B	0	0	0	0	0	0	0	0
ZC2HC1C	0	4.450725	0.756395	7.221244	0.44517	1.368304	0	5.022226
ZC3H10	2.726041	8.612018	0.81723	13.172214	0	7.173483	0	2.351727
ZC3H11A	79.021125	271.852052	78.707849	267.09825	98.692194	238.895504	49.423691	284.40418
ZC3H12A	14.034693	25.693917	25.126854	55.330788	29.288784	57.837029	21.059382	54.490245
ZC3H12B	2.189755	5.253065	0	5.53967	0	1.392114	0	1.282848
ZC3H12C	18.297299	20.51287	16.920222	30.066642	13.088811	36.091857	16.512718	42.131093
ZC3H12D	0	0.114361	0	0.27473	0	0	0	0
ZC3H13	14.023238	21.870117	25.833466	24.28921	20.13217	15.478227	32.569905	18.706755
ZC3H14	74.649646	121.723354	53.024487	125.994303	142.472403	122.251055	66.53797	125.21579
ZC3H15	100.608188	107.791156	91.365422	70.038236	103.436445	96.748834	195.4624	113.898604
ZC3H18	15.964322	45.612412	0.669745	46.260595	1.411249	63.027836	23.49034	28.247882

ZC3H3	14.811587	11.464764	12.816994	13.415491	9.166022	10.503661	3.239518	4.803254
ZC3H4	37.730485	37.924841	32.306503	68.314321	22.639294	153.623817	149.889742	89.050345
ZC3H6	0.555144	0.730663	19.944793	5.10255	0.256814	1.46847	0	7.671409
ZC3H7A	35.240586	59.009272	60.998826	41.644906	121.372469	23.74803	170.831946	56.931235
ZC3H7B	18.176641	46.364821	23.269756	40.236537	34.927362	68.90936	29.399854	49.608835
ZC3H8	0.683571	2.448813	0.589849	21.274181	15.618905	19.494355	12.007404	18.469273
ZC3HAV1	24.316706	24.513414	23.896948	22.230408	43.391027	33.198864	23.62574	50.046594
ZC3HAV1L	1.991869	4.284899	10.655108	3.187929	6.298527	5.404722	5.297055	7.886486
ZC3HC1	20.238435	2.034214	6.428576	0	11.128135	7.243792	13.776673	2.92786
ZC4H2	10.22775	16.619697	8.887041	19.960213	6.485215	7.310294	43.741382	4.74173
ZCCHC10	43.043248	34.235453	42.032247	23.941384	40.801886	15.377469	36.79526	27.49059
ZCCHC10P1	0	0	0	0	0	0	0	0
ZCCHC10P2	0	0	0	0	0	0	0	0
ZCCHC12	0	0.0664	0	0.076111	0	0	0	0
ZCCHC14	16.726421	25.522421	16.853436	24.348718	21.91604	37.855179	0	36.333841
ZCCHC17	13.339669	35.236682	0	9.805316	41.254347	26.313541	62.285658	30.191703
ZCCHC18	0	0.148056	0	3.532509	0	0.290946	0	0
ZCCHC2	22.378382	15.268302	22.281825	18.269857	49.563854	20.50764	41.507048	28.833793
ZCCHC24	0	4.001585	0	0	0	0	0	0.894939
ZCCHC4	9.165798	6.292666	11.880685	3.745121	14.45779	4.297975	0	3.688238
ZCCHC7	9.663331	8.164871	0	18.862701	11.383421	32.259789	96.043058	6.895973
ZCCHC8	5.284648	1.450059	0	0.70416	0	0.14217	0	0
ZCCHC9	31.826976	88.181714	37.69323	72.75252	42.417308	45.405049	0	70.424259
ZCRB1	86.290642	192.980395	58.834919	142.737338	57.29837	99.106918	39.884978	128.761686
ZCRB1P1	0	0	0	0	0	0	0	0
ZCWPW1	0	2.15868	0	1.802227	0	2.417287	0	2.279748
ZCWPW2	0.993237	0	0	0.532843	0	0.049546	0	0.039922
ZDBF2	3.977714	11.824423	8.46012	14.359838	3.440729	6.254983	11.452861	13.887652
ZDHHC1	7.437562	1.903903	0	0.848816	17.458827	0.920985	0	0
ZDHHC11	0	0.400612	3.499227	4.069997	0	6.071585	0	2.779659
ZDHHC11B	0	0	0	0	0	0.264461	0	0
ZDHHC12	59.616626	40.187505	57.977699	42.29803	31.855981	29.793246	14.498299	21.902608
ZDHHC13	18.01621	28.999527	21.565468	11.994083	25.883256	8.549766	29.947982	35.951582

ZDHHC14	1.068309	2.959168	1.607144	2.467413	0	3.606365	0	3.524815
ZDHHC15	0.584298	0.360374	0	0.158267	0	0	0	0
ZDHHC16	43.529184	40.293513	34.113831	42.728243	45.367395	26.220949	22.720779	34.137921
ZDHHC17	0	13.607139	23.303062	8.552144	0	5.23103	57.139333	9.38619
ZDHHC18	11.022127	16.122285	19.638451	12.863074	24.89884	27.934622	12.808536	21.200772
ZDHHC19	0	0	0	0	0	0	0	0
ZDHHC1P1	0	0	0	0	0	0	0	0
ZDHHC2	3.294481	16.514569	1.974214	6.941394	3.046571	1.156088	4.28244	13.071342
ZDHHC20	36.775846	30.641541	44.089273	16.270949	45.261023	6.489744	14.666074	24.273984
ZDHHC20P1	0	0	0	0	0	0	0	0
ZDHHC20P2	0	0	0	0	0	0	0	0
ZDHHC20P3	0	0	0	0	0	0	0	0
ZDHHC20P4	0	0	0	0	0	0	0	0
ZDHHC21	0	1.641999	0	1.231833	0	3.160901	0	7.660254
ZDHHC22	0	0.960999	0.876535	1.049126	0	0.341394	0	0
ZDHHC23	7.546045	16.27459	3.720493	15.478084	1.109183	5.985287	0	7.011759
ZDHHC24	12.591905	6.316103	6.741463	7.860037	8.929382	3.33515	0.166177	4.526291
ZDHHC3	24.115762	38.244421	40.247658	42.196315	27.560955	47.52272	89.035742	40.360723
ZDHHC4	108.455909	134.869082	83.286805	125.260488	35.039026	52.046785	77.291579	66.612539
ZDHHC4P1	0	0	0	0	0	0	0	0
ZDHHC5	34.039816	116.01003	46.510839	120.874764	53.748299	226.87178	225.801348	224.939728
ZDHHC6	31.735155	65.278914	33.517822	43.143475	46.446795	50.586682	105.518515	69.187628
ZDHHC7	0	89.486573	61.605356	93.437046	59.361533	128.452298	41.272347	72.271548
ZDHHC8	37.233615	39.010863	32.750076	39.427413	43.144796	81.225996	0.265154	56.322663
ZDHHC8BP	0	0.451377	0	0	0.680381	0.189063	0	0
ZDHHC9	30.364785	42.933026	51.793611	56.439589	27.35826	26.801658	15.786566	24.28191
ZEB1	0	0.185576	0	0.68382	0	0	0	0
ZEB2	0	0.110702	0	1.17493	0	0.211153	0	0.173344
ZEB2P1	0	0	0	0	0	0	0	0
ZER1	17.641162	35.285047	18.591875	45.552277	15.800648	50.107483	54.079646	44.326615
ZFAND1	130.308772	190.434602	106.126563	183.724382	77.458958	26.15661	86.689335	50.11002
ZFAND2A	0	1.43857	0	2.011758	0	0.395158	0	0.162905
ZFAND2AP1	0	0	0	0	0	0	0	0

ZFAND2AP2	0	0	0	0	0	0	0	0
ZFAND2B	4.145088	11.084831	16.199024	10.607782	13.054573	19.100603	0	19.037913
ZFAND3	45.086654	118.20514	120.270251	126.859614	81.495192	98.25042	38.133887	78.740234
ZFAND4	18.179592	8.68282	10.47835	7.653729	17.927976	0.182442	0	5.278109
ZFAND5	136.268727	393.915657	54.422738	397.142077	96.655008	423.543885	117.512599	614.768115
ZFAND6	38.503752	109.429532	74.502431	101.615266	86.840442	66.255585	121.722829	120.892465
ZFAND6P1	0	0	0	0	0	0	0	0
ZFAT	5.111941	5.33359	17.823692	9.444907	32.432245	19.126363	19.344222	7.818688
ZFC3H1	30.477367	8.446482	0	9.024811	52.95168	11.273389	133.117861	2.216121
ZFHX2	0	0.954146	0.311277	1.705833	0	5.485919	0	1.025407
ZFHX3	12.09695	11.936185	12.166285	12.937475	25.012433	50.403078	13.342975	35.221617
ZFHX4	0	10.251543	5.228974	11.487827	0	27.383384	8.855142	23.797177
ZFP1	21.450303	24.240747	31.261191	27.859931	10.694181	19.609668	1.953034	26.67026
ZFP14	1.294006	0.1857	0.775952	0.253307	12.569667	0.473407	0	0.259308
ZFP2	0	0	0	0.068889	0	0	0	0
ZFP28	0	0	0	0	0	0	0	0
ZFP3	0	0	0	0.016036	0.866701	0.427128	0	1.184818
ZFP30	0	15.301275	2.659628	14.072169	3.520547	24.496968	0	26.875116
ZFP36	0	1.191805	0	0.390009	13.967702	4.81618	33.673737	0
ZFP36L1	0	2.848875	0	1.416932	0	55.933019	0	9.427785
ZFP36L2	62.869915	106.680308	62.040016	83.22263	44.109615	130.940271	47.626818	121.834859
ZFP37	2.858553	8.976216	7.450737	4.559352	0.752786	2.659815	0	2.45692
ZFP41	1.187948	22.952702	0	25.973894	43.921388	29.42562	0	12.734372
ZFP42	0	0.124275	0	0.17793	0	0	0	0.117894
ZFP57	0	0	0	0.318265	0	0	0	0
ZFP62	23.888307	61.304819	49.61026	48.172673	16.750648	42.470086	0	45.83337
ZFP64	44.162592	60.1702	49.852593	88.689408	28.618998	52.01683	37.25414	46.662001
ZFP64P1	0	0	0	0	0	0	0	0
ZFP69	3.282582	10.347414	2.932392	5.743951	0.432777	3.002135	0	3.544042
ZFP69B	1.7585	18.26701	7.312118	10.575555	2.781492	4.26602	0	6.606564
ZFP82	1.389687	0.337837	1.789211	0.445459	0	0.656318	0	1.096356
ZFP90	82.398652	84.465505	98.127237	87.774639	46.533085	50.862241	42.231097	66.267888
ZFP91	58.05196	47.748768	34.446984	35.918775	68.051926	51.775088	65.519113	72.369394

ZFP91-CNTF	0	18.655207	0	44.144036	0	81.813927	0	40.730542
ZFP91P1	0	0	0	0	0	0.157429	0	0
ZFPL1	26.301165	24.5316	48.960476	30.703287	35.163867	52.457904	1.877162	43.839002
ZFPM1	3.603315	17.534658	5.396625	18.021411	1.745253	8.967555	2.271274	8.229579
ZFPM2	0	1.427598	0	2.60817	0	0	0	0
ZFR	53.450306	24.960904	35.593105	22.018023	28.649498	16.441291	61.791992	23.497137
ZFR2	0	3.03191	0	0.109704	2.469378	0	0	0
ZFRP1	0	0	0	0	0	0	0	0
ZFTA	1.139447	2.59521	1.024099	2.228727	3.612561	5.83515	0	6.447554
ZFTRAF1	2.405518	3.689747	0.512161	11.31615	10.744636	19.989375	5.018464	12.830145
ZFX	9.141115	22.802765	5.004194	10.847325	46.762032	19.733176	34.311393	49.127846
ZFY	26.138936	20.185186	9.770759	20.191599	3.509784	36.291329	1.401879	61.702018
ZFYVE1	3.771706	16.926239	21.635004	22.756145	13.321866	26.888985	15.77131	25.808592
ZFYVE16	10.188834	18.387156	14.246977	8.39982	18.583332	5.659071	166.85863	17.719183
ZFYVE19	19.618487	28.196273	15.450813	48.55701	18.190751	56.571536	5.253095	39.319961
ZFYVE21	36.044007	0.806249	0	4.27293	0	5.28304	0	6.923244
ZFYVE26	12.288397	21.710876	10.162668	20.752885	11.110389	22.925348	109.176223	22.862788
ZFYVE27	5.072724	9.508188	10.217576	12.783923	5.688637	7.643827	22.215383	7.141218
ZFYVE28	0	1.947312	0	0.721666	0.271609	0.128393	0	0.11739
ZFYVE9	0	33.528062	0	11.772261	6.148395	7.617423	30.394833	10.480357
ZFYVE9P1	0	0	0	0	0	0	0	0
ZFYVE9P2	0	0	0	0	0	0	0	0
ZG16B	0	0	0	0	0	0	0	1.484529
ZGLP1	0	0.189431	0	0.167031	0	0	0	0.608707
ZGPAT	7.840591	12.965857	15.653311	7.208503	0	16.058749	0	10.125509
ZGRF1	0	9.325942	19.499406	4.586308	1.322857	4.893237	49.060346	7.895025
ZHX1	36.76171	72.108432	43.243579	50.665842	43.980559	65.92017	76.068962	149.834123
ZHX1-C8orf7	0	12.191745	0	8.374016	0	20.147453	0	5.40699
ZHX2	9.808782	32.212557	18.297546	37.314874	10.193083	42.151369	3.490015	34.997591
ZHX3	6.402219	38.14912	44.717203	33.690584	34.404163	73.68989	5.705563	55.006901
ZIC1	0	0.053329	0.557883	2.869718	0	0	0	0
ZIC2	44.291427	15.479235	12.191648	16.318548	2.993039	8.415598	0	5.610581
ZIC3	0	0	0	0	0	0	0	0

ZIC4	0	3.253794	1.679516	2.431509	0	0.140204	0	0.312369
ZIK1	12.954457	24.981739	7.858148	19.37325	26.581117	15.583427	0	24.732344
ZIK1P1	0	0	0	0	0	0	0	0
ZIM2	0	0	0	0	0	0	0	0
[truncated: 58,359 more chars]
